# Intrarenal activation of adaptive immune effectors is associated with tubular damage and impaired renal function in lupus nephritis

**DOI:** 10.1136/annrheumdis-2018-213485

**Published:** 2018-07-31

**Authors:** Cristina Pamfil, Zuzanna Makowska, Aurélie De Groof, Gaëlle Tilman, Sepideh Babaei, Christine Galant, Pauline Montigny, Nathalie Demoulin, Michel Jadoul, Selda Aydin, Ralf Lesche, Fiona McDonald, Frédéric A Houssiau, Bernard R Lauwerys

**Affiliations:** 1 Department of Rheumatology, Cliniques Universitaires Saint-Luc, Brussels, Belgium; 2 Pôle de pathologies rhumatismales systémiques et inflammatoires, Institut de Recherche Expérimentale et Clinique, Université catholique de Louvain, Brussels, Belgium; 3 Pharmaceuticals, Research and Development, Bayer AG, Berlin, Germany; 4 Department of Pathology, Cliniques Universitaires Saint-Luc, Brussels, Belgium; 5 Department of Nephrology, Cliniques Universitaires Saint-Luc, Brussels, Belgium; 6 Pôle de Néphrologie, Institut de Recherche Expérimentale et Clinique, Université catholique de Louvain, Brussels, Belgium

**Keywords:** lupus nephritis, systemic lupus erythematosus, T cells

## Abstract

**Objectives:**

Chronic renal impairment remains a feared complication of lupus nephritis (LN). The present work aimed at identifying mechanisms and markers of disease severity in renal tissue samples from patients with LN.

**Methods:**

We performed high-throughput transcriptomic studies (Illumina HumanHT-12 v4 Expression BeadChip) on archived kidney biopsies from 32 patients with LN and eight controls (pretransplant donors). Histological staging (glomerular and tubular scores) and immunohistochemistry experiments were performed on the same and on a replication set of 37 LN kidney biopsy samples.

**Results:**

A group of LN samples was identified by unsupervised clustering studies based on their gene expression features, that is, the overexpression of transcripts involved in antigen presentation, T and B cell activation. These samples were characterised by a significantly lower estimated glomerular filtration rate (eGFR) at the time of biopsy (T0) compared with the other systemic lupus erythematosus samples. Yet, apparent disease duration at T0, double-stranded DNA antibody titres at T0 and other relevant characteristics (serum C3, proteinuria, histological scores, numbers of previous flares) were not different between groups.

Immunohistochemistry studies confirmed the association between interstitial infiltration by adaptive immune effectors and decreased renal function in the same and in a replication group of LN kidney biopsies. This was associated with transcriptomic, histological and immunohistochemical evidence of renal tubular cell involvement.

**Conclusion:**

Interstitial infiltration of LN kidney biopsies by adaptive immune effectors is associated with impaired renal tubular cell function and decreased eGFR. These results open new perspectives in evaluating and treating patients with LN, focusing on intrarenal mechanisms of immune cell activation.

## Introduction

Lupus nephritis (LN) is a severe complication of systemic lupus erythematosus (SLE), initiated by the deposition of anti-double stranded DNA (anti-dsDNA) autoantibodies in glomerular basement membranes.^1–3^ Treatment of LN requires the use of immunosuppressants, in order to avoid evolution to end-stage renal disease.^4–6^ Yet, despite major improvements in treatment strategies over the last decades, a significant proportion of patients display renal damage accrual, and 10% develop renal failure after 10 years.^7 8^ Because the disease mainly affects young women, these numbers represent a heavy toll in terms of quality of life and health expenditures.

In recent years, it appeared that the lupus kidney is not just a passive target of systemic autoimmunity features, but also hosts mechanisms that contribute to the pathogenesis of the disease, independently of the systemic activation of immune effectors.^9 10^ The identification of anti-dsDNA-producing long-lived plasma cells in kidneys from SLE mice is a good illustration of how second wave immune effectors take advantage of the local inflammatory environment to perpetuate the production of pathogenic autoantibodies.^11 12^ In human SLE kidneys, the presence of T and B cell aggregates and germinal centres is another indication of the development of an organised immune response in the target organ.^10^

In this study, we intended to establish a comprehensive map of intrarenal molecular profiles associated with disease severity, by performing high-throughput transcriptomic studies on kidney biopsies from patients with LN, followed for a long period of time in a single LN reference centre, and controls (cadaveric donors). An independent set of LN kidney biopsies was used for histological and immunohistochemistry confirmation of our findings. In all patients, longitudinal clinical and biological data were collected systematically, which enabled us to correlate transcriptomic and/or immunohistochemistry patterns with longitudinal markers of renal and systemic disease activity. Although we used a ‘bottom-up’ approach to our analyses (ie, predefined clinical variables were not used to categorise the samples; instead, the samples were categorised based on their transcriptomic characteristics), our hypothesis was that intrarenal molecular patterns in our samples were associated with impaired renal function.

During the course of our study, our observations made us focus on tubulointerstitial involvement as a variable associated with renal outcomes in LN. Evaluation of tubulointerstitial structures is not part of the present International Society of Nephrology/Renal Pathology Society (ISN/RPS) classification of LN. Yet, several groups developed semiquantitative scores of interstitial fibrosis, inflammation and tubular cell damage in order to address the issue in the context of the disease. In this study, we also took advantage of the recent identification of Syndecan-1 (SDC1, CD138) as a marker of renal tubular cell stress. SDC1 is a lectin expressed at the surface of plasma cells, but also epithelial cells. In epithelial cells, cell surface SDC1 is cleaved by inflammation-induced proteases (eg, ADAMTS, MMP), and loss of cell surface SDC1 is associated with higher susceptibility to cell damage, in gastroenterological, but also renal disorders.^13–19^

## Patients and methods

### Patients and kidney biopsies

All patients with LN included in this study were recruited in a single centre (Université catholique de Louvain, Brussels, Belgium). They met the 1982 American College of Rheumatology revised classification criteria for the diagnosis of SLE,^20^ and had biopsy-proven nephritis. Patients’ demographics are displayed in table 1. See online supplementary file 1 for additional specifications and ethical approval.

10.1136/annrheumdis-2018-213485.supp1 Supplementary file 1

**Table 1:** Characteristics of the patients with SLE included in this study

	Discovery set (transcriptomic and immunohistochemistry studies)	Confirmation set (immunohistochemistry studies)
Number of patients (female/male)	32 (23/9)	37 (34/3)
Age (years, median, IQR)	30 (35.7–35.7)	27 (22–40)
Apparent renal disease duration (months; median, range)	4 (0–144)	0 (0–156)
Duration of follow-up after the biopsy (months; median, range)	82 (5–258)	109 (37–256)
Ethnicity		
Caucasian	26	31
African	3	3
Asiatic	3	1
Other	0	1
SLEDAI (median; range)	16 (4–25)	16 (6–24)
Serum dsDNA antibody titres (U/mL; median, IQR)	87.3 (32.2–291.3)	300.5 (66.7–686.7)
Serum C3 (mg/dL, median, IQR)	64 (48.7–80.2)	58.0 (33–68.5)
eGFR (mL/min/1.73 m^2^, median, IQR)	73.0 (48.7–93.0)	82.0 (60.0–104.0)
Proteinuria (g/24 hours, median, IQR)	2.6 (1.9–4.4)	2.0 (1.2–5.2)
Immunosuppressive therapy at biopsy		
None (patients, n)	16	19
Methylprednisolone (patients, n; dose range)	7 (4–16)	16 (4–6)
Azathioprine (patients, n)	5	5
Mycophenolate mofetil (patients, n)	4	0
Glomerular histology		
Class III (+V)	4	6
Class IV (+V)	27	31
Class V	1	0
Activity index (median, IQR)	8 (5–11)	10 (5–12)
Chronicity index (median, IQR)	1 (1–2)	1 (0–2)

There are no significant differences between the groups using Mann-Whitney and Χ^2^ tests.

eGFR, estimated glomerular filtration rate; SLE, systemic lupus erythematosus; SLEDAI, SLE Disease Activity Index.

### High-throughput transcriptomic studies

RNA was extracted from 60 SLE and 27 control biopsies using NucleoSpin technology (Macherey-Nagel). The samples were tested for RNA integrity by Bioanalyzer (Agilent) measurements. Samples with an RNA integrity number >6 and RNA quantity >50 ng were used for target synthesis (ie, from 32 SLE and eight control biopsies). There was no significant bias in clinical, biological or histological characteristics between the samples with good quality RNA and the samples dropped from the study. See online supplementary file 1 for the description of HumanHT-12 v4 Expression BeadChip (Illumina) hybridisations.

### Histological and immunohistochemistry experiments

ISN/RPS classification of the SLE renal biopsies and glomerular activity/chronicity indices (Morel-Maroger semiquantitative scores) were retrieved from the medical files of the patients. In addition, a semiquantitative score for renal tubular cell atrophy and interstitial fibrosis (0–3) was generated using H&E stained slides.

See online supplementary file 1 for the description of CD3, CD20, CD8 and SDC1 immunostaining experiments.

### Statistical analyses

See online supplementary file 1.^21 22^

## Results

**Figure 1:**
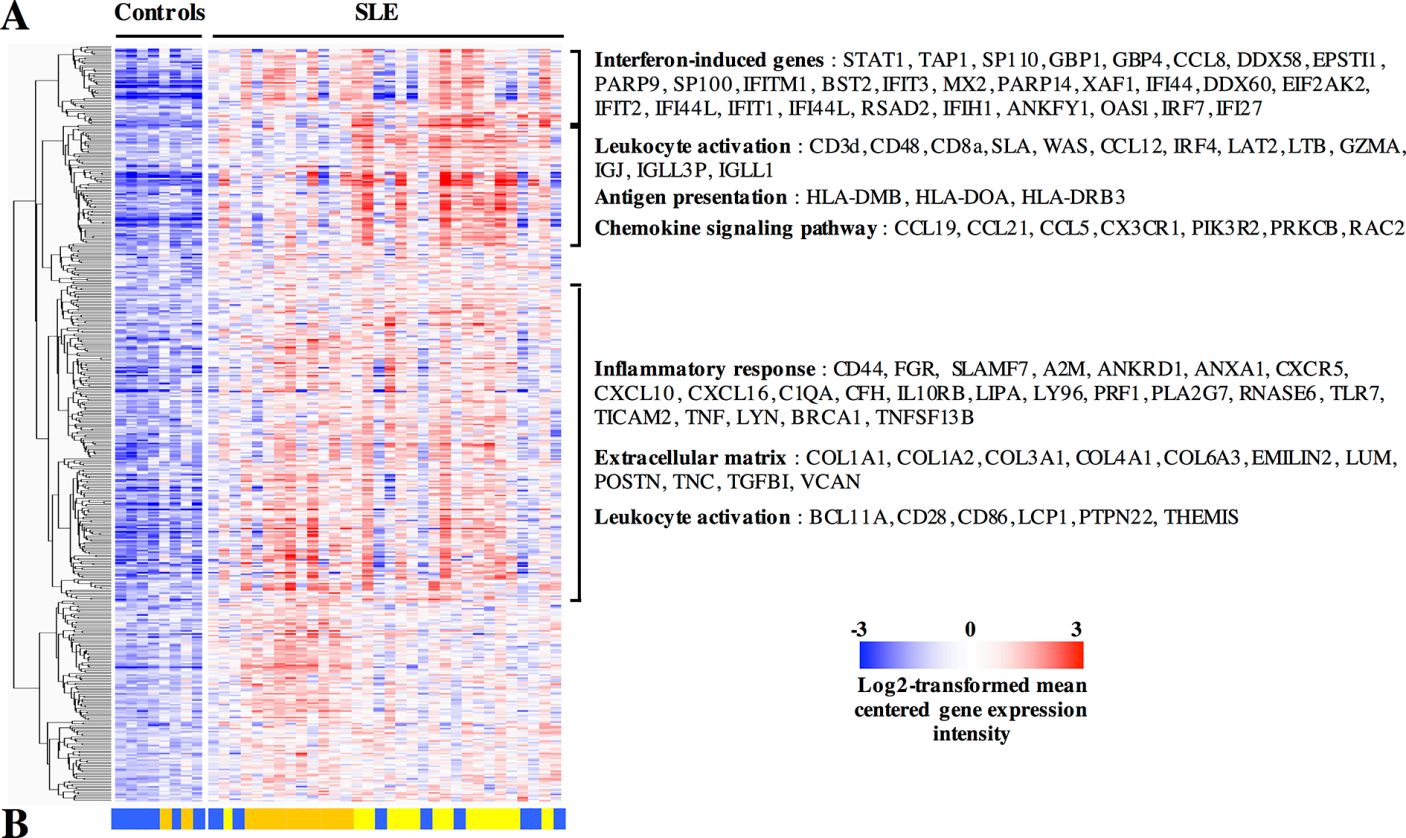
Transcripts overexpressed in systemic lupus erythematosus (SLE) compared with control kidney biopsies. (A) Gene expression was determined in kidney biopsies by microarray analyses. Moderated t-tests with Benjamini-Hochberg corrections for multiple comparisons identified 478 transcripts upregulated in SLE compared with control biopsies with a p value <0.05. The figure displays log2-transformed mean-centred cyclic loess-normalised intensities across all samples. Pathway analyses indicated that a significant percentage of transcripts clustered into the indicated gene ontology terms. The complete lists of transcripts and pathways are available in online supplementary table 1. (B) An unsupervised hierarchical clustering algorithm (Pearson-centred algorithm, Ward’s linkage rule) using the same transcripts distributed the samples in three clusters, identified by a colour (same colours as in figure 2).

High-density transcriptomic studies (Illumina BeadChip arrays) were performed using renal biopsies from 32 patients with LN and eight controls. All patients had proliferative (class III or class IV) nephritis with or without membranous (class V) involvement, except for one who had an isolated class V (table 1). After normalisation of the transcriptomic data, we found that LN samples were characterised by the overexpression of 478 transcripts compared with controls. Further data mining indicated that these transcripts were significantly enriched in the following pathways: inflammatory response, antigen presentation, leucocyte activation, interferon signature and chemokine signalling (figure 1A, online supplementary table 1). Conversely, 329 transcripts were downregulated in LN compared with control renal biopsies (online supplementary table 2). In the latter, pathway analyses showed a significant enrichment in the following gene ontologies: mitochondrion, oxidation reduction, organic acid transport, hydrogen ion transmembrane transporter activity and glutathione transferase activity, many of which point at renal proximal tubular cell functions.

10.1136/annrheumdis-2018-213485.supp2 Supplementary file 2

10.1136/annrheumdis-2018-213485.supp3 Supplementary file 3

**Figure 2:**
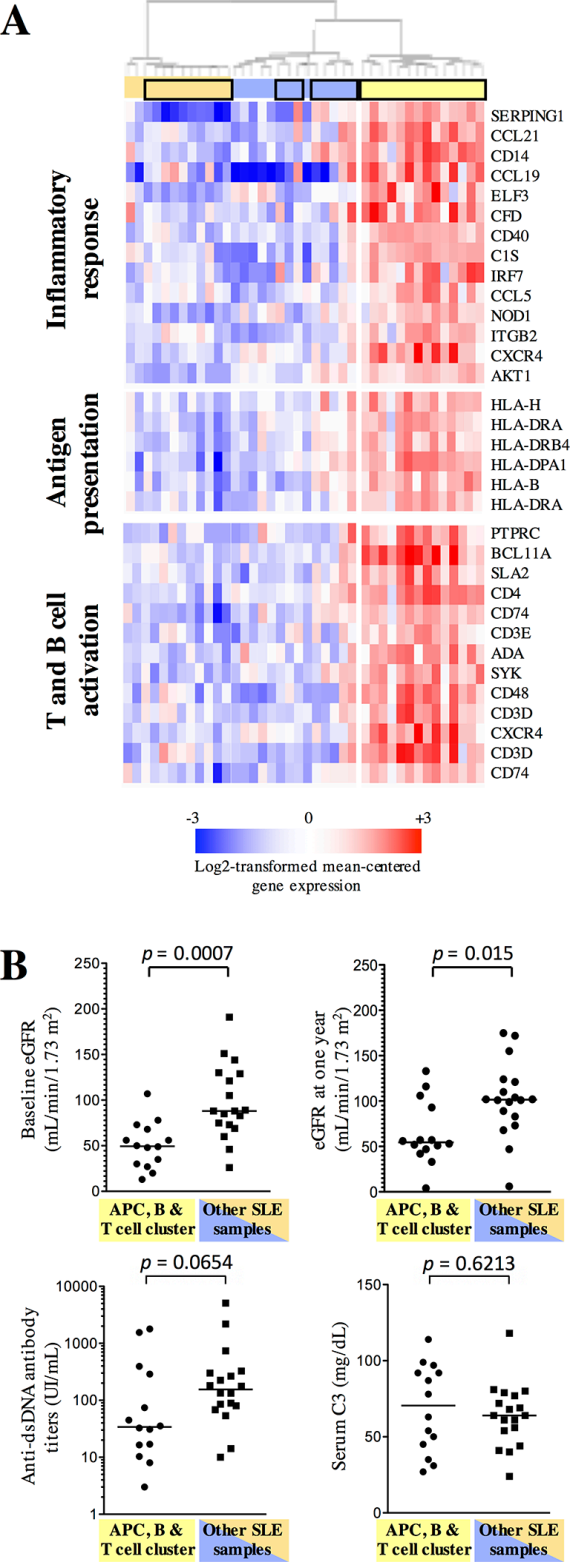
Transcriptomic profiling singles out a subgroup of systemic lupus erythematosus (SLE) kidney biopsy samples with decreased estimated glomerular filtration rate (eGFR). (A) A cluster of SLE kidney biopsy samples, identified by hierarchical clustering algorithm (Pearson-centred algorithm, Ward’s linkage rule), is characterised by the overexpression of 271 transcripts (by moderated t-test, with Bonferroni correction for multiple comparisons) compared with the other lupus samples, significantly enriched in the indicated pathways. Samples designated by a black box are SLE samples. The complete lists of transcripts and pathways are available in online supplementary table 3. (B) Distribution of biological data in sera of patients with SLE at the time of the biopsy and at 1 year, according to the transcriptomic clusters determined in (A). Horizontal lines represent the median values and IQRs. P values were calculated by Mann-Whitney test. APC, antigen-presenting cells.

LN samples were not homogeneous in the differential expression of these transcripts, and unsupervised hierarchical clustering studies distributed the biopsies in three groups (figures 1B and 2). Out of these, one group of samples overexpressed transcripts belonging to pathways pointing at local activation of adaptive immune effectors: antigen presentation, T and B cell activation. From a clinical and biological point of view, these patients had a significantly lower estimated glomerular filtration rate (eGFR) at baseline. Yet, there were no clinical or biological factors explaining this difference: renal disease duration, number of renal flares prior to the renal biopsy, number of renal flares after biopsy, glomerular activity and chronicity indices; even anti-dsDNA antibody titres were not different in these compared with the other LN samples (figure 2, table 2). Intriguingly, eGFR was still significantly lower in these patients at 1 year, and at last follow-up visit (figure 2, table 2). Univariate analyses indicated a link between eGFR at follow-up and eGFR at baseline, renal molecular cluster, glomerular chronicity index in the biopsy and induction therapy with cyclophosphamide, but only eGFR at baseline remained as an independent variable predictive of eGFR at follow-up using multivariate analyses.

10.1136/annrheumdis-2018-213485.supp6 Supplementary file 6

**Table 2:** Clinical, biological and histological characteristics of patients with LN categorised according to the transcriptomic clusters determined in figure 2A

	APC, T and B cell cluster	Other LN samples	P values
Number of patients	14	18	
Sex (male/female)	5/9	14/4	0.3997*
Ethnicity (Caucasians/non-Caucasians)	3/11	15/3	0.7321*
Age (years, median, IQR)	28.5 (25.5–36.2)	31.0 (24.7–38.5)	0.9545†
**Baseline**			
eGFR (mL/min/1.72 m^2^, median, IQR)	49.5 (29.2–69.2)	88 (72.0–129.3)	**0.0007†**
Proteinuria (g/24 hours, median, IQR)	3.39 (2.04–5.88)	2.45 (1.14–3.89)	0.0839†
Serum C3 (mg/dL, median, IQR)	70.5 (42.5–93.2)	64 (51.5–77.5)	0.6213†
Serum dsDNA antibody titres (U/mL; median; IQR)	34.1 (14.9–315.1)	155.5 (77.1–306.8)	0.0654†
SLEDAI-2K (median; IQR)	18 (13–20)	16 (16–20)	0.9229†
Glomerular histology			0.5127*
Class III (+V)	3	2	
Class IV (+V)	11	15	
Class V	0	1	
Activity index (median, IQR)	10 (4–13)	7 (5–10)	0.1759†
Chronicity index (median, IQR)	2 (1–2)	1 (1–2)	0.1544†
Apparent renal disease duration (median, months; IQR)	4.5 (1–39)	3.5 (2.7–16.2)	0.9847†
Number of renal flares at biopsy (median; months; IQR)	1 (1–2)	1 (1–1)	0.1832†
Immunosuppressive therapy at biopsy			0.3086*
None (patients, n)	9	7	
Methylprednisolone (patients, n; mg/day; dose range)	2 (4–16)	5 (8–32)	
Azathioprine (patients, n)	1	5	
Mycophenolate mofetil (patients, n)	2	2	
Intravenous cyclophosphamide (Euro-Lupus) (patients, n)	1	0	
**At year 1**			
eGFR (mL/min/1.72 m^2^, median, IQR)	54.5 (45.7–96.2)	101.5 (80.5–121.8)	**0.0150†**
Proteinuria (g/24 hours, median, IQR)	0.97 (0.34–2.51)	0.87 (0.13–2.0)	0.5590†
Serum C3 (mg/dL, median, IQR)	87.55 (76.7–107.3)	86.5 (70.5–103.0)	0.8196†
Serum dsDNA antibody titres (U/mL; median; IQR)	13.3 (8.5–46.2)	33.5 (17.7–72.1)	0.0984†
Immunosuppressive therapy after renal biopsy			0.1038*
Intravenous steroids alone (patients, n)	2	0	
Mycophenolate mofetil (patients, n)	7	5	
Intravenous cyclophosphamide (Euro-Lupus) (patients, n)	3	11	
Intravenous cyclophosphamide (NIH) (patients, n)	1	1	
Rituximab	1	0	
**At follow-up**			
Duration of follow-up (months; median; IQR)	91 (20–129)	86 (41–136)	0.8197†
Total number of renal flares at follow-up (n; median; IQR)	1.5 (1.0–3.0)	1.5 (1.0–2.0)	0.4835†
eGFR (mL/min/1.72 m^2^, median, IQR)	51.0 (11.7–72.0)	88.5 (67.7–114.5)	**0.0215†**
ESRD (patients, n)	5	3	0.1476‡

Significant P values are in bold.

*P values calculated by Χ^2^ tests.

†P values calculated by Mann-Whitney tests.

‡P values calculated by Gehan-Breslow-Wilcoxon test.

APC, antigen-presenting cells, eGFR, estimated glomerular filtration rate; ESRD, end- stage renal disease; LN, lupus nephritis; NIH, National Institutes of Health; SLEDAI, SLE Disease Activity Index.

**Figure 3:**
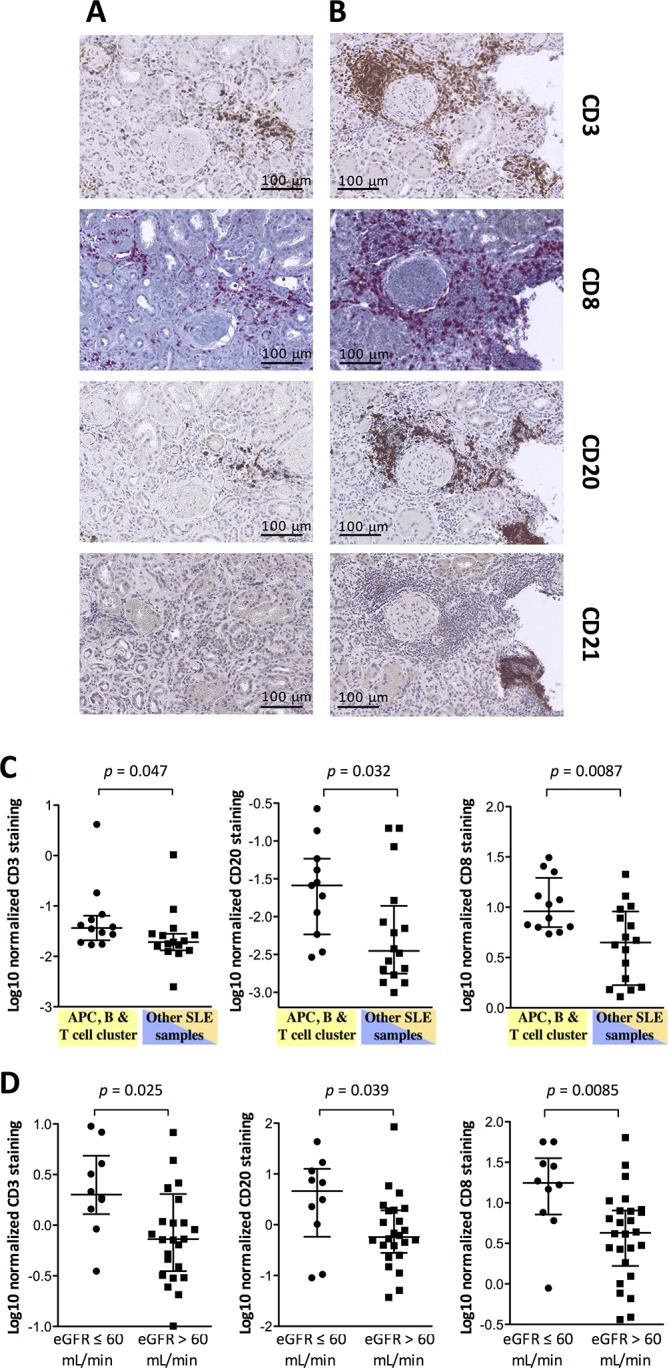
The presence of adaptive immune effectors in the systemic lupus erythematosus (SLE) kidney interstitium is associated with decreased estimated glomerular filtration rate (eGFR). (A) CD21-negative and (B) CD21-positive T and B cell aggregates in the interstitium are shown in serial sections from two SLE kidney biopsies. (C) Digital quantification of the CD3, CD8 and CD20 stains (Log2 scale) confirms higher numbers of T and B cells in SLE kidney biopsy fragments from the cluster of samples overexpressing T and B cell-associated transcripts in the microarray data. (D) Digital quantification of the CD3, CD8 and CD20 stains in an independent group of SLE kidney biopsies indicates higher numbers of T and B cells in patients with decreased baseline eGFR. Horizontal lines represent the median values and IQRs. P values were calculated by Mann-Whitney test. APC, antigen-presenting cells.

Thus, our transcriptomic data indicated a link between the presence of transcripts associated with the activation of an adaptive immune response in the lupus kidney, and decreased renal function at baseline. We therefore performed immunohistochemistry experiments aiming at the detection of CD3+ T, CD8+ T and CD20+ B cells in renal biopsies from patients with LN. In view of previous reports indicating the presence of tertiary lymphoid structures in LN biopsies, we also tried to detect CD21+ follicular dendritic cells. At first, we stained the samples used in the high-throughput transcriptomic studies. Out of the 30 stained biopsy specimens available, we found very little CD21 positive samples (n=3). In these few samples, CD21+ cells colocalised with CD3+ and CD20+ cells, indicating the presence of tertiary lymphoid structures. Otherwise, CD3+ and CD20+ cell aggregates were found in the majority of the samples with either a periglomerular or an intertubular distribution (figure 3A,B). After digital quantification of the stains, we confirmed that the group of samples enriched in antigen presentation, T and B cell activation transcripts were significantly more positive for CD3, CD8 and CD20 than the other LN samples (figure 3C). Accordingly, digital quantification of the CD3 and CD8 stains was significantly higher in samples from patients with a baseline eGFR≤60 mL/min/1.73 m^2^ compared with >60 mL/min/1.73 m^2^. Although CD20 correlated significantly with the CD3 stain (Spearman r=0.4985, p=0.0112), and negatively with eGFR (Spearman r=−0.4947, r=0.0119), there was no significant difference in CD20 quantification between eGFR≤60 and eGFR>60 mL/min/1.73 m^2^ (online supplementary figure 1).

10.1136/annrheumdis-2018-213485.supp4 Supplementary file 4

These immunohistochemistry experiments were repeated in an independent set of 37 LN biopsies. Again, CD21 positive cells and tertiary lymphoid structures were found in three samples only. Aggregates of CD3+, CD8+ and CD20+ cells were found in a majority of samples in the kidney interstitium. After digital quantification, we also found that CD3 and CD20 correlated significantly with each other (Spearman r=0.4950, p=0.0034). CD3+, CD8+ and CD20+ cells were significantly higher in the biopsy samples from patients with an eGFR≤60 mL/min compared with >60 mL/min in this second cohort of samples (figure 3D).

**Figure 4:**
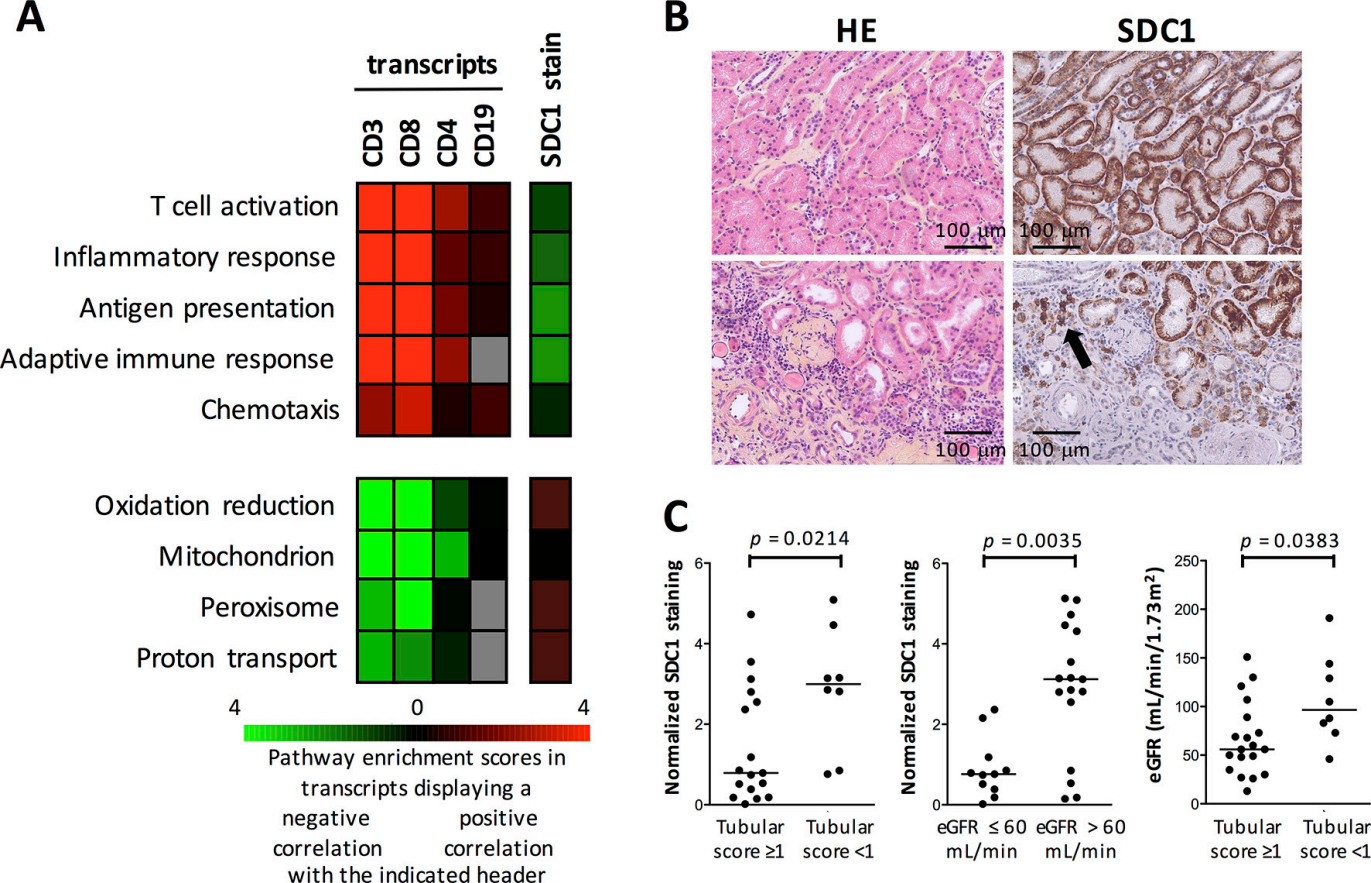
The presence of adaptive immune effectors in the systemic lupus erythematosus (SLE) kidney interstitium is associated with impaired renal tubular cell phenotype and functions. (A) The microarray data set was analysed to identify genes that show >0.4 or <−0.4 value of Pearson correlation to CD3, CD8, CD4, CD19 transcripts from the same microarray data set, or to the digital quantification of SDC1 in the histological specimens from the same biopsies. The resulting gene sets were then annotated using DAVID software and the selected top pathway enrichment scores (−log10 p value) are shown as different hues of green (for the sets of genes showing positive correlation with the respective variable) and red (negative correlation). Grey squares indicate that the pathway is not enriched in the list of transcripts. Transcripts and pathways are listed in online supplementary tables 4–8. (B) H&E and SDC1 stains on consecutive slides from patients without (upper line) or with (lower line) tubular infiltration by mononuclear cells. The arrow indicates CD138-positive infiltrating plasmablasts. (C) Quantification of the SDC1 stain in SLE kidney biopsies used in the microarray experiments indicates lower SDC1 in patients with higher renal tubular cell damage and lower estimated glomerular filtration rate (eGFR). P values were calculated by Mann-Whitney tests.

In order to understand the link between the presence of adaptive immune effectors in lupus kidney biopsies and decreased kidney function, we went back to the high-throughput transcriptomic data and identified transcripts and pathways that correlated (positively or negatively) with *CD3*, *CD4*, *CD8* and *CD19* transcripts (there was no transcript corresponding to *CD20* on the Illumina platform) in lupus kidney biopsy samples. Not surprisingly, a positive correlation was found between the expression of these transcripts and transcripts involved in inflammatory responses, adaptive immune responses, antigen presentation, T cell activation and chemotaxis. By contrast, a negative correlation was found with transcripts associated with renal tubular cell functions: oxidation reduction, mitochondrion, peroxisome and proton transport (figure 4A). Accordingly, digital quantification of the CD3+ T and CD20+ B cell stains in the kidney interstitium of these samples was significantly associated with increased histological scores of renal tubular cell damage (CD3: Spearman r=0.6229, p=0.0015; CD20: Spearman r=0.4771, p=0.0213). Renal tubular cell damage itself displayed a significant negative correlation with eGFR (Spearman r=−0.4732, p=0.0226).

10.1136/annrheumdis-2018-213485.supp7 Supplementary file 7

Finally, we evaluated the loss of SDC1 expression at the surface of renal tubular cells by immunohistochemistry. SDC1 is cleaved at the surface of renal tubular cells by MMP7 (online supplementary file 1 and supplementary figure 2). In both cohorts of lupus biopsy samples, we found that SDC1 was expressed by a few infiltrating plasma cells, but the vast majority of stained cells were renal tubular cells (figure 4B). Quantitative evaluation of the SDC1 stain correlated positively with the expression of transcripts associated with renal tubular cell functions (figure 4A). Hence, it was significantly higher in biopsies where no tubular damage was evidenced at histological evaluation. Accordingly, it was significantly higher in patients with an eGFR higher than 60 mL/min, due to the significant association between tubular damage and eGFR (figure 4C). Of note, quantitative evaluation of the SDC1 stain correlated negatively with transcripts associated with T, B and antigen-presenting cell activation in the same set of samples (figure 4A), providing further support to the link between the presence of adaptive immune effectors in the lupus kidney, and renal tubular cell damage-mediated impairment of renal function.

10.1136/annrheumdis-2018-213485.supp5 Supplementary file 5

## Discussion

The development of new therapeutic strategies using immunosuppressive drugs (intravenous cyclophosphamide, mycophenolate mofetil in association with corticosteroids) significantly improved renal outcomes in LN.^23^ Yet, up to 10% of the patients develop end-stage renal disease at 10 years, which is a major concern in a population of mainly young female patients. In this perspective, several studies were performed in order to identify early markers of disease severity in patients with LN. Thus, evidence indicates that decreased eGFR and high glomerular chronicity indices at histological evaluation of baseline kidney biopsies are associated with poor renal outcomes.^24–29^ More recently, it appeared that reaching a proteinuria <0.7–0.8 g/24 hours at 1 year after initiation of therapy was predictive of a good renal outcome at 10 years, an observation that potentially translates into a treat-to-target approach of LN.^30 31^ Yet, our current approach to the care of patients with LN is still hampered by an incomplete understanding of the pathogenic events characteristic of the disease.

Our present results bring important additional information on markers and mechanisms of renal inflammation in LN, by pointing at the role of activated T and B cells in the kidney interstitium as potential mediators of renal tubular cell damage. In two independent groups of kidney biopsies, we did not find an association between decreased renal function and markers of systemic disease activity or histological indices of glomerular involvement (activity and chronicity indices). By contrast, a decreased eGFR was observed in patients with histological evidence of tubular damage, and transcriptomic evidence of T and B cell activation in the kidney, which was further confirmed by immunohistochemistry.

In a recent study, Parikh *et al* evaluated the expression of 511 immune response genes in longitudinal (before and after administration of induction therapy) kidney biopsies from four patients with LN who did not respond to therapy, compared with five patients who displayed a complete response, and four controls. In line with our own results, they reported that transcripts associated with leucocyte infiltration, T cell activation, complement pathway and interferon signature increased between baseline and follow-up in non-responders.^32^ In the present study, we instead used an unbiased analytical approach, grouping the samples based on their molecular characteristics rather than predefined clinical categories, resulting in the identification of a link between T and B cell infiltration and activation, tubular damage and decreased renal function in LN.

Tubulointerstitial lesions were previously already identified as a prognostic marker in LN.^33–37^ Interstitial infiltration by inflammatory cells and tubular atrophy potentially result from or are amplified by several kinds of insults in the context of LN: proteinuria, deposition of immune complexes in the interstitium, rupture of the Bowman’s capsule and presentation of cryptic antigens by juxtaglomerular antigen-presenting cells, induction of proinflammatory molecules at the surface of renal tubular cells (eg, ICAM-1, CD40).^38–43^ Several lines of evidence are in favour of an antigen-dependent local expansion of adaptive immune effectors. Thus, Chang *et al* demonstrated the presence of cells from the adaptive immune system in the interstitium of lupus kidney biopsies, organised either in T and B cell aggregates, or in germinal centres in the presence of CD21+ follicular dendritic cells. By sequencing the Ig repertoire of one germinal centre and four T:B cell aggregates, they found evidence of clonal restriction in all samples tested, and extensive somatic hypermutation in all but one T and B cell aggregate, in line with an antigen-driven clonal selection.^10^ In a later publication, authors from the same group found that vimentin is overexpressed by mononuclear cells infiltrating the renal interstitium in lupus biopsies, and is a recurrent target of locally produced immunoglobulins.^44^ Similarly, evaluation of the local T cell receptor repertoire is also indicative of a clonal expansion of a restricted population of T cells.^45 46^ Local antigens, driving the proliferation of these T cells in the lupus kidney, were not identified yet.

Our high-density transcriptomic data provide further support to the hypothesis that infiltrating adaptive immune effectors display a toxic effect on renal tubular cells, thereby contributing to the development of renal failure. Thus, transcripts associated with CD3, CD4, CD8 and B cells displayed strong correlations with each other, which is not a surprise in the context of an organised immune response. Little CD21+ follicular dendritic cells were found, but this could be biased by the small size of the renal biopsy samples. Negative correlations were found with transcripts associated with renal tubular cell functions. The link between the presence of T and B cells in the renal interstitium and tubular cell damage was confirmed by immunohistochemistry and histological scoring of biopsy specimens. The strongest and most consistent correlations between cell infiltration and renal tubular cell involvement were observed for CD3 and CD8 positive cells. These data are in line with observations by Couzi *et al*, who reported that CD8 T cells are the most prevalent T cells infiltrating lupus kidneys, and also found a significant correlation between CD8 T cells in the kidney interstitium and decreased renal function in 25 patients with LN.^47^ The possibility that in particular CD8 T cells play an amplificatory role in LN-related renal lesions is also emphasised by the observation that the presence of a T effector-memory signature in CD8 T cells isolated from the peripheral blood of patients with LN is associated with a higher probability of renal relapses and a higher number of disease flares per month.^48^ Based on data from the same group, that is, presence of a similar CD8 effector-memory T cell signature in the blood of patients with ANCA-associated vasculitis (AAV) and a higher rate of renal relapses, it is tempting to speculate that secondary activation of immune effectors against autoantigens locally modified by an initial renal injury is not restricted to LN. Thus, evidence indicates that interstitial infiltration by CD3 T cells and tubular damage is associated with poor response to rituximab therapy and lower eGFR at 12 months in AAV.^49^ Similarly, previous studies had identified the presence of interstitial inflammation as a significant prognostic marker in patients with AAV treated with cyclophosphamide and corticosteroids.^50^

That renal tubular cells in the lupus kidney are particularly targeted by infiltrating adaptive immune effectors is also illustrated by our SDC1 data. Although SDC1 is expressed by a few plasma cells infiltrating the lupus kidney, this lectin is mainly expressed by renal tubular cells.^13–15^ SDC1 is cleaved from the surface of epithelial cells by inflammation-induced proteases.^16 17^ In the lupus kidney, MMP7 is a good candidate in view of the strong correlation between the loss of SDC1 and MMP7 expression, and our in vitro data demonstrating the loss of SDC1 expression at the surface of a renal tubular cell line after exposure to MMP7. In inflammatory bowel diseases, loss of SDC1 at the surface of intestinal epithelial cells is associated with a higher susceptibility to cell damage,^18 19^ and the same holds true in renal tubular cells in view of our transcriptomic and histological data linking SDC1 expression and renal tubular cell viability and function. Using the SDC1 stain as a marker of renal tubular cell integrity, we confirmed the negative correlation with the expression of genes associated with the activation of adaptive immune effectors.

Elevated SDC1 concentrations are found in the sera of patients with SLE as compared with controls.^15^ It will be of interest to evaluate whether serum SDC1 in these patients is associated with renal disease and poorer renal outcomes. Overall, our data are a strong trigger to evaluate the link between markers of renal tubular cell damage and poorer renal outcomes in LN individuals, together with an assessment of the distribution and activation patterns of immune cells in urine specimens of these patients. In addition, mechanistic data are needed, in order to provide definite evidence about the pathogenic amplificatory role of CD8 T cells in LN. Also, while our observations were made in a mainly Caucasian population of patients with LN, it will be of importance to extend our studies to ethnically diverse populations, in particular due to the known influence of ethnicity on renal outcomes in LN. Would such observations confirm our data, they would open new perspectives in evaluating and treating patients with LN, focusing on intrarenal mechanisms of immune cell activation.

10.1136/annrheumdis-2018-213485.supp8 Supplementary file 8

10.1136/annrheumdis-2018-213485.supp9 Supplementary file 9

10.1136/annrheumdis-2018-213485.supp10 Supplementary file 10

10.1136/annrheumdis-2018-213485.supp11 Supplementary file 11
