# Supplementary material for: Intrarenal activation of adaptive immune effectors is associated with tubular damage and impaired renal function in lupus nephritis
Source: Ann Rheum Dis. 2018 Jul 31;77(12):1782–9. doi: 10.1136/annrheumdis-2018-213485 (PMC6241616; doi:10.1136/annrheumdis-2018-213485)
Supplement: Supplementary file 1 [file annrheumdis-2018-213485supp001.docx]

**PATIENTS AND METHODS**

**Patients and kidney biopsies**

All LN patients included in this study were recruited in a single center (Université catholique de Louvain, Brussels, Belgium). They met the 1982 ACR revised classification criteria for the diagnosis of SLE,[20] and had biopsy proven nephritis. Patients included in the discovery (transcriptomic experiments) group were selected based on the availability of residual frozen (-80° C) kidney biopsy material (samples were collected between October 1995 and October 2015). Independent patients in the confirmation (immunohistochemistry experiments) group were part of the LOULUNIC (LOUvain LUpus Nephritis InCeption) cohort, i.e. patients biopsied and followed-up from the diagnosis of LN and first renal biopsy, and were included in the present study based on the availability of paraffin-embedded renal biopsy material (samples were collected between January 1996 and April 2012). Patients’ demographics are displayed in Table 1. All patients were prescribed hydroxychloroquine, in addition to the indicated therapies. Control biopsies were obtained from cadaveric donors, prior to transplantation of the organ. The controls were 5 females and 3 males, aged 46 ± 14 years. All the biopsies used in this study were residual corporal material, initially taken for diagnostic purposes. Clinical data, biological parameters and SLEDAI 2000 scores were retrieved from the medical files of the patients. eGFR values were calculated using the CKD-EPI formula. The Ethical Committee of the Université catholique de Louvain (UCL) approved the study and initially ruled that patients consent was not required for the use of residual corporal material, in agreement with Belgian regulations on human studies. However, in order to ensure formal compliance with German regulations on the use of residual corporal material, we did ask all SLE patients included in the study to sign a consent form, after re-approval by the UCL Ethical Committee.

**High throughput transcriptomic studies**

RNA was extracted from 60 SLE and 27 control biopsies using Nucleospin technology (Macherey-Nagel). The samples were tested for RNA integrity by Bioanalyzer (Agilent) measurements. Samples with a RNA Integrity Number (RIN) >6 and RNA quantity >50 ng were used for target synthesis (i.e. from 32 SLE and 8 control biopsies). There was no significant bias in clinical, biological or histological characteristics between the samples with good quality RNA and the samples dropped from the study. Target synthesis and labeling was performed at Bayer’s research laboratory in Berlin with NuGEN Ovation kit, with input of 5ng total RNA. Samples were randomized with respect to SLE/control and the date of RNA extraction. For each sample, 750ng of labeled cRNAs was hybridized to HumanHT-12 v4 Expression Beadchips (Illumina), washed according to the manufacturer’s protocols and stained with Streptavidin-Cy3 to detect the biotinylated transcripts. Fluorescent signals were acquired using BeadArray Reader scanner (Illumina).

**Histological and immunohistochemistry experiments**

ISN/RPS classification of the SLE renal biopsies and glomerular activity/chronicity indices (Morel-Maroger semi-quantitative scores) were retrieved from the medical files of the patients. In addition, a semi-quantitative score for renal tubular cell atrophy and interstitial fibrosis (0-3) was generated using hematoxylin-eosin stained slides.

For the immunostaining experiments, deparaffination and rehydration of the 5 μm paraffin-embedded tissue sections was performed through a series of washes with xylene and graded alcohols. After incubation with 0.05% pronase for antigen retrieval and quenching of endogenous peroxidase activity by 10-minute incubation with a peroxidase blocking reagent, non-specific reactions were inhibited by immersing the slides in 1x Dako buffer solution supplemented with 5% bovine serum albumin and 2% milk powder. The slides were incubated at 37°C for 1 hour with the following primary antibodies: mouse anti-human CD20 (BioCare Medical; 1:200), CD21 (Thermo Scientific; 1:400), SDC1 (Dako; 1:50), CD8 (Dako; 1:24) and rabbit anti-human CD3 (Dako; 1:400). Specifically, bound antibodies were labeled with Envision + system horseradish peroxidase-labeled polymer (brown color, Dako) or Ultraview Universal Alkaline Phosphatase (red color, Dako). Slides used for the CD8 stain were CD3-stained slides, unstained using an acidic isopropyl alcohol solution, hence the different labeling color. Slides were digitalized on a SCN400 slide scanner (Leica), and quantification of the stains was performed using FRIDA software v1.1.0 (average of the quantification of 6 pictures/slide at a 40x magnification, normalized for the surface of the nuclei).

**Effect of MMP7 on Syndecan-1 expression by renal tubular cells**

LE-9715 (PTEC) renal tubular cells were cultured in Dulbecco’s modified Eagle medium (DMEM, Gibco Life Technologies), supplemented with 10% fetal bovine serum, 100 μg/mL streptomycin (Gibco), 100 U/mL penicillin (Gibco), and incubated for 2 hours in the presence of APMA (Sigma)-activated MMP7 (R&D Systems, 320, 480 and 640 ng/mL). Cells were detached by a 5-minute incubation in a 2 mM EDTA solution, next washed and resuspended in sodium phosphate (1 mM, pH = 7.4) buffer containing 137 nM NaCl, 5 mM KCl, 0.4 mM MgSO4, 0.3 mM MgCl2, 5 mM Glucose, 4 mM NaHCO3, 1 mM EDTA and 3% FCS. Cells were stained with an APC-labeled anti-SDC1 antibody (BioLegend), next analyzed on a FACSCanto II flow cytometer (Beckton-Dickinson).

**Statistical analyses**

The bead-level data from the microarray experiments were summarized to probe-level data using GenomeStudio 2.0 software (Illumina). The probe-level data for genes and negative controls were then exported from GenomeStudio and read into R software. The arrays were background-corrected using a method based on the intensity values of the negative probes, implemented in MBCB package (R statistical software). Low-quality probes (more than two mismatches to transcriptomic targets) were eliminated, and the data were normalized by cyclic loess (limma package). Further statistical analyses of the microarray data were performed on GeneSpring 12.6.1 software. Differences in gene expression between lupus and control biopsies were analyzed using a moderated t test with Benjamini-Hochberg correction for multiple comparisons (*p* value threshold set to 0.05). Unsupervised hierarchical clustering of the samples was performed using a Pearson-centered algorithm with a Ward’s linkage rule. Pathway analyses were performed using DAVID 6.7 software. Enrichment scores are –log10 *p* values, calculated by modified Fisher Exact test by comparing proportions of transcripts belonging to a given pathway in the tested gene list compared to the whole transcriptome.[21, 22]

Multivariate analyses were performed using linear stepwise regression on SPSS in order to identify independent variables predictive of eGFR at last follow-up visit. The following variables were considered: age, sex, SLEDAI at baseline, eGFR at baseline, proteinuria at baseline, anti-dsDNA antibody titers at baseline, glomerular activity index, glomerular chronicity index, renal molecular cluster, proteinuria at last follow-up visit, use of cyclophosphamide during induction therapy, use of mycophenolate mofetil during induction therapy, duration of follow-up, numbers of flares during follow-up.

Correlation studies between expression of CD3, CD8, CD19 or quantitative evaluation of the CD138 stain and transcriptomic data were performed on Excel. Pathway analyses were performed using DAVID 6.7 software on transcripts displaying a *r* < -0.4 or > 0.4 with these parameters across all SLE samples. All other t tests and correlation analyses were performed on Prism 5.0, using non-parametric tests.
