# Supplementary material for: Intrarenal activation of adaptive immune effectors is associated with tubular damage and impaired renal function in lupus nephritis
Source: Ann Rheum Dis. 2018 Jul 31;77(12):1782–9. doi: 10.1136/annrheumdis-2018-213485 (PMC6241616; doi:10.1136/annrheumdis-2018-213485)
Supplement: Supplementary file 2 [file annrheumdis-2018-213485supp002.pdf]

Supplementary Table 1 : Transcripts and pathways up-regulated in SLE compared to control renal biopsies

Differences in (Log2-transformed, mean-centered) gene expression between lupus and control biopsies were analyzed using a moderated t test with Benjamini-Hochberg correction for multiple comparisons (p value threshold set to 0.05). Pathway analyses were performed using DAVID software. Enrichment scores are –log10 p values, calculated by modified Fisher Exact test by comparing proportions of transcripts belonging to a given pathway in the tested gene list compared to the whole transcriptome.[14, 15]

Transcripts

Identifier	[Control]	[SLE]	Gene Symbol
ILMN_2054019	-1.8367431	0.8733817	ISG15
ILMN_3240375	-1.9034421	0.8672207	abParts
ILMN_1739508	-1.7163734	0.8066571	abParts
ILMN_2103107	-1.8484101	0.6729792	ADAMDEC1
ILMN_1778977	-0.7100609	0.62325823	TYROBP
ILMN_1704286	-0.7749682	0.619152	FXYD5
ILMN_2170814	-2.5021515	0.6106421	LAMP3
ILMN_1680274	-1.6995718	0.5754184	abParts
ILMN_1652199	-1.6970621	0.57067966	abParts
ILMN_2083066	-2.5462198	0.5612969	IGLL3P
ILMN_1674228	-0.9006702	0.55473113	abParts
ILMN_1659913	-0.8059585	0.5446736	ISG20
ILMN_1672606	-0.7296701	0.5338008	OAS1
ILMN_1695851	-0.8407382	0.532421	PARVG
ILMN_2146766	-0.7411417	0.5317069	FABP5
ILMN_1696302	-0.8049086	0.5251267	FABP5
ILMN_1886655	-1.267512	0.5189515	TCRVB
ILMN_1752899	-0.6961275	0.518324	BCL11A
ILMN_2325837	-0.9341917	0.5164521	CD3D
ILMN_1779852	-0.8254615	0.5040414	FABP5L3
ILMN_1754507	-2.2113416	0.4878948	IRF4
ILMN_1682993	-0.8189868	0.48173356	NKG7
ILMN_1714602	-1.0103395	0.46908656	CD86
ILMN_1710734	-0.9052631	0.45434737	GZMK
ILMN_1791759	-1.2359245	0.45214474	CXCL10
ILMN_1801246	-1.8158823	0.44969594	IFITM1
ILMN_1677505	-0.8335129	0.4461307	CCL21
ILMN_2231928	-2.2008712	0.4434064	MX2
ILMN_2375825	-0.4494968	0.44047216	CD37
ILMN_1719759	-1.0855303	0.43799433	TNC
ILMN_1790761	-1.575065	0.43785053	POSTN
ILMN_2232121	-1.0042269	0.43288076	GPR65
ILMN_2135272	-0.969066	0.43147218	GIMAP2
ILMN_1728106	-1.2595581	0.4270727	TNF
ILMN_3249667	-0.7323164	0.42657584	HLA-DQA1
ILMN_2254635	-2.3059683	0.41904867	ITGAX
ILMN_3266606	-1.1168858	0.4060247	FABP5
ILMN_2208903	-0.7734711	0.38791603	CD52
ILMN_1769229	-1.2805104	0.38425326	BCL2A1
ILMN_1787509	-0.9047725	0.3832354	PRIC285
ILMN_2179083	-0.9967498	0.3825167	LOXL4
ILMN_1749006	-0.8976072	0.37745827	RCSD1
ILMN_2061043	-1.1870687	0.37040937	CD48
ILMN_1758067	-1.4429107	0.367198	RGS4
ILMN_1806908	-1.0466263	0.36677915	PRKCB
ILMN_1662843	-1.2426662	0.36529452	CD53
ILMN_1682312	-0.6436449	0.3637218	CYBB
ILMN_1798181	-1.2807904	0.35389298	IRF7
ILMN_1780368	-1.1570473	0.35253793	GPR18
ILMN_1693452	-1.0383923	0.35075316	GAL3ST4

Pathways

**Annotation Cluster 1**	**Enrichment Score: 8.52**		**Count**	**P_Value**	**Benjamini**
	GOTERM_BP_FAT	defense response	42	2.20E-10	1.60E-07
	GOTERM_BP_FAT	response to wounding	38	5.10E-10	2.70E-07
	GOTERM_BP_FAT	inflammatory response	25	2.40E-07	6.40E-05
**Annotation Cluster 2**	**Enrichment Score: 6.1**		**Count**	**P_Value**	**Benjamini**
	SP_PIR_KEYWORDS	signal	115	1.90E-10	6.90E-08
	UP_SEQ_FEATURE	signal peptide	115	2.80E-10	3.70E-07
	SP_PIR_KEYWORDS	disulfide bond	102	7.20E-09	8.90E-07
	UP_SEQ_FEATURE	disulfide bond	98	2.70E-08	1.80E-05
	SP_PIR_KEYWORDS	glycoprotein	131	1.20E-07	1.10E-05
	UP_SEQ_FEATURE	glycosylation site:N-linked (GlcNAc...)	119	1.20E-05	4.10E-03
	GOTERM_CC_FAT	extracellular region part	41	6.90E-05	7.20E-03
	GOTERM_CC_FAT	extracellular space	31	2.60E-04	1.20E-02
	GOTERM_CC_FAT	extracellular region	67	4.30E-04	1.70E-02
	SP_PIR_KEYWORDS	Secreted	53	1.00E-03	1.50E-02
**Annotation Cluster 3**	**Enrichment Score: 4.85**		**Count**	**P_Value**	**Benjamini**
	GOTERM_BP_FAT	cell activation	27	1.10E-09	4.50E-07
	GOTERM_BP_FAT	leukocyte activation	22	9.60E-08	3.40E-05
	GOTERM_BP_FAT	T cell activation	16	1.20E-07	3.50E-05
	GOTERM_BP_FAT	lymphocyte activation	19	4.40E-07	1.00E-04
	GOTERM_BP_FAT	immune system development	20	1.20E-05	2.20E-03
	GOTERM_BP_FAT	hemopoietic or lymphoid organ development	19	1.90E-05	3.10E-03
	GOTERM_BP_FAT	hemopoiesis	18	2.00E-05	2.80E-03
	GOTERM_BP_FAT	T cell differentiation	8	5.60E-04	4.40E-02
	GOTERM_BP_FAT	leukocyte differentiation	11	6.50E-04	4.80E-02
	GOTERM_BP_FAT	lymphocyte differentiation	8	7.80E-03	2.40E-01
	GOTERM_BP_FAT	T cell selection	3	6.50E-02	5.70E-01
**Annotation Cluster 4**	**Enrichment Score: 4.59**		**Count**	**P_Value**	**Benjamini**
	SP_PIR_KEYWORDS	lipoprotein	34	6.60E-07	4.10E-05
	UP_SEQ_FEATURE	lipid moiety-binding region:S-palmitoyl cysteine	14	7.40E-05	1.20E-02
	SP_PIR_KEYWORDS	palmitate	14	3.50E-04	6.20E-03
**Annotation Cluster 5**	**Enrichment Score: 4.12**		**Count**	**P_Value**	**Benjamini**
	GOTERM_CC_FAT	plasma membrane part	81	1.90E-06	2.90E-04
	GOTERM_CC_FAT	intrinsic to plasma membrane	48	9.50E-05	7.40E-03
	GOTERM_CC_FAT	integral to plasma membrane	46	2.20E-04	1.40E-02
	UP_SEQ_FEATURE	topological domain:Extracellular	78	8.40E-04	9.70E-02
**Annotation Cluster 6**	**Enrichment Score: 3.95**		**Count**	**P_Value**	**Benjamini**
	UP_SEQ_FEATURE	domain:SH3	15	1.30E-05	3.60E-03
	SP_PIR_KEYWORDS	sh3 domain	15	8.30E-05	1.80E-03
	INTERPRO	Src homology-3 domain	15	1.10E-04	1.80E-02
	SMART	SH3	15	1.40E-03	2.10E-01
**Annotation Cluster 7**	**Enrichment Score: 3.64**		**Count**	**P_Value**	**Benjamini**
	GOTERM_BP_FAT	positive regulation of response to stimulus	20	1.20E-06	2.60E-04
	GOTERM_BP_FAT	positive regulation of immune response	14	2.00E-05	3.00E-03
	GOTERM_BP_FAT	positive regulation of immune system process	18	2.20E-05	2.90E-03
	GOTERM_BP_FAT	immune effector process	13	4.20E-05	5.10E-03
	GOTERM_BP_FAT	activation of immune response	10	2.30E-04	2.10E-02
	GOTERM_BP_FAT	leukocyte mediated immunity	9	6.20E-04	4.70E-02
	GOTERM_BP_FAT	immune response-activating signal transduction	7	9.60E-04	6.70E-02
	GOTERM_BP_FAT	immune response-regulating signal transduction	7	1.40E-03	8.50E-02
	GOTERM_BP_FAT	immune response-activating cell surface receptor signaling pathway	6	1.60E-03	9.10E-02

ILMN_2369018	-1.3167466	0.34742686	EVI2A
ILMN_1772964	-1.1127529	0.33681542	CCL8
ILMN_2217574	-0.6778461	0.33079284	FABP5L3
ILMN_1760509	-1.4685779	0.33047506	EOMES
ILMN_1673357	-0.7304227	0.32993287	SLA2
ILMN_1687301	-1.5809488	0.32957417	VCAN
ILMN_1728923	-0.9579498	0.3291558	F2RL2
ILMN_1656310	-0.4940499	0.32283896	IDO1
ILMN_1801504	-0.9557369	0.32217026	RUNX1
ILMN_1749362	-1.1738353	0.31501293	CD28
ILMN_1677827	-1.610075	0.31439185	TLR7
ILMN_1723480	-1.4555889	0.31439024	BST2
ILMN_1674402	-0.6813584	0.30988973	TMEM71
ILMN_1675640	-0.99256	0.308559	OAS1
ILMN_2145670	-1.3313366	0.30529922	TNC
ILMN_2085862	-1.3194237	0.30307707	SLC15A3
ILMN_1802708	-0.8654579	0.30057782	BTN3A1
ILMN_2058782	-1.6705484	0.29868892	IFI27
ILMN_2412192	-0.5491448	0.29791167	CFH
ILMN_1740633	-1.1358054	0.29771525	PRF1
ILMN_2202894	-1.643364	0.29721236	F2RL2
ILMN_3302919	-0.7347454	0.29306167	MYOF
ILMN_1657871	-2.23516	0.29136372	RSAD2
ILMN_1662358	-2.3834696	0.2906471	MX1
ILMN_1780533	-1.423368	0.29047525	RNASE6
ILMN_3237946	-0.7705273	0.28753045	PXDN
ILMN_1691364	-1.4878128	0.28612438	STAT1
ILMN_2042651	-1.6108624	0.2831158	EVI2B
ILMN_2066849	-1.3265369	0.28190893	FAM26F
ILMN_1724533	-0.9776902	0.28160933	LY96
ILMN_2105441	-1.7997382	0.27848127	IGJ
ILMN_2413808	-1.3141	0.27765673	CD53
ILMN_3234828	-0.3738681	0.27627262	TMEM229B
ILMN_1684040	-1.7639973	0.27530622	THEMIS
ILMN_1778723	-1.2069848	0.2744472	AMICA1
ILMN_1685275	-0.6273471	0.2738763	MCAM
ILMN_3306672	-0.7661769	0.27119064	PATL2
ILMN_2326512	-1.8256159	0.26943186	CASP1
ILMN_2353732	-1.9275862	0.26927188	CD8A
ILMN_1734740	-1.1187886	0.26920912	GPR65
ILMN_2307861	-1.0338793	0.26916945	COL6A3
ILMN_1768595	-0.8213046	0.26846257	DLG4
ILMN_2098126	-0.7770375	0.2672991	CCL5
ILMN_3220934	-0.5118439	0.26631477	NCRNA00152
ILMN_2146761	-0.4733385	0.26463622	FABP5
ILMN_1723486	-0.8193373	0.26113445	HK2
ILMN_1731644	-0.4773678	0.2600093	SETDB2
ILMN_2184184	-0.9364551	0.2593097	ANXA1
ILMN_1765446	-0.651159	0.2588973	EMP3
ILMN_1736729	-0.4527157	0.25189105	OAS2
ILMN_1695590	-1.5135725	0.25181645	ADRB2
ILMN_1731418	-1.4074057	0.25135908	SP110
ILMN_1737918	-0.7864966	0.25117314	C1QA
ILMN_1706483	-1.0443532	0.250543	C1orf116
ILMN_1808657	-0.6577458	0.25034893	FMO3
ILMN_1677997	-0.9073625	0.2456177	MAZ

	GOTERM_BP_FAT	immune response-regulating cell surface receptor signaling pathway	6	2.20E-03	1.20E-01
	GOTERM_BP_FAT	antigen receptor-mediated signaling pathway	5	5.80E-03	2.10E-01
**Annotation Cluster 8**	**Enrichment Score: 3.39**		**Count**	**P_Value**	**Benjamini**
	GOTERM_BP_FAT	cell adhesion	32	1.80E-04	1.90E-02
	GOTERM_BP_FAT	biological adhesion	32	1.80E-04	1.80E-02
	SP_PIR_KEYWORDS	cell adhesion	19	2.10E-03	2.60E-02
**Annotation Cluster 9**	**Enrichment Score: 3.39**		**Count**	**P_Value**	**Benjamini**
	SP_PIR_KEYWORDS	glycoprotein	131	1.20E-07	1.10E-05
	GOTERM_CC_FAT	plasma membrane	122	1.00E-06	3.20E-04
	UP_SEQ_FEATURE	glycosylation site:N-linked (GlcNAc...)	119	1.20E-05	4.10E-03
	SP_PIR_KEYWORDS	membrane	158	2.30E-04	4.30E-03
	UP_SEQ_FEATURE	topological domain:Cytoplasmic	94	5.30E-04	6.90E-02
	UP_SEQ_FEATURE	topological domain:Extracellular	78	8.40E-04	9.70E-02
	SP_PIR_KEYWORDS	transmembrane	121	8.00E-03	7.60E-02
	UP_SEQ_FEATURE	transmembrane region	119	1.20E-02	5.10E-01
	GOTERM_CC_FAT	intrinsic to membrane	135	6.50E-02	6.00E-01
	GOTERM_CC_FAT	integral to membrane	127	1.50E-01	8.40E-01
**Annotation Cluster 10**	**Enrichment Score: 3.32**		**Count**	**P_Value**	**Benjamini**
	SP_PIR_KEYWORDS	chemotaxis	11	1.50E-06	6.30E-05
	KEGG_PATHWAY	Chemokine signaling pathway	20	2.00E-06	2.40E-04
	GOTERM_BP_FAT	taxis	16	2.60E-06	5.00E-04
	GOTERM_BP_FAT	chemotaxis	16	2.60E-06	5.00E-04
	GOTERM_BP_FAT	locomotory behavior	18	1.30E-04	1.40E-02
	GOTERM_MF_FAT	chemokine activity	7	4.80E-04	1.20E-01
	GOTERM_MF_FAT	chemokine receptor binding	7	6.70E-04	1.10E-01
	SP_PIR_KEYWORDS	inflammatory response	8	8.40E-04	1.40E-02
	SP_PIR_KEYWORDS	cytokine	12	1.00E-03	1.50E-02
	INTERPRO	Small chemokine, interleukin-8-like	6	1.30E-03	9.50E-02
	GOTERM_BP_FAT	behavior	21	3.90E-03	1.80E-01
	SMART	SCY	6	4.00E-03	2.10E-01
	KEGG_PATHWAY	Cytokine-cytokine receptor interaction	17	4.50E-03	3.50E-02
	GOTERM_MF_FAT	cytokine activity	11	1.10E-02	5.60E-01
	INTERPRO	Small chemokine, C-C group, conserved site	4	1.30E-02	3.40E-01
	PIR_SUPERFAMILY	PIRSF001950:small inducible chemokine, C/CC types	4	1.80E-02	6.90E-01
	BBID	109.Chemokine_families	6	2.20E-02	6.60E-01
**Annotation Cluster 11**	**Enrichment Score: 2.85**		**Count**	**P_Value**	**Benjamini**
	UP_SEQ_FEATURE	domain:Ig-like V-type 2	5	1.00E-03	1.10E-01
	INTERPRO	Immunoglobulin V-set	14	1.60E-03	1.00E-01
	UP_SEQ_FEATURE	domain:Ig-like V-type 1	5	1.70E-03	1.60E-01
**Annotation Cluster 12**	**Enrichment Score: 2.54**		**Count**	**P_Value**	**Benjamini**
	KEGG_PATHWAY	Allograft rejection	8	7.20E-05	2.80E-03
	KEGG_PATHWAY	Graft-versus-host disease	8	1.20E-04	3.60E-03
	UP_SEQ_FEATURE	domain:Ig-like C1-type	7	1.30E-04	1.90E-02
	KEGG_PATHWAY	Type I diabetes mellitus	8	2.00E-04	4.70E-03
	KEGG_PATHWAY	Viral myocarditis	10	2.20E-04	4.30E-03
	INTERPRO	Immunoglobulin C1-set	8	4.20E-04	4.00E-02
	KEGG_PATHWAY	Intestinal immune network for IgA production	8	5.30E-04	9.00E-03
	KEGG_PATHWAY	Systemic lupus erythematosus	11	6.40E-04	9.40E-03
	KEGG_PATHWAY	Asthma	6	1.40E-03	1.90E-02
	INTERPRO	Immunoglobulin/major histocompatibility complex, conserved site	8	1.80E-03	9.90E-02
	SMART	IGc1	8	1.90E-03	1.50E-01
	GOTERM_BP_FAT	antigen processing and presentation	8	2.40E-03	1.20E-01
	KEGG_PATHWAY	Autoimmune thyroid disease	7	3.70E-03	3.30E-02
	KEGG_PATHWAY	Cell adhesion molecules (CAMs)	11	5.50E-03	4.00E-02
	GOTERM_BP_FAT	antigen processing and presentation of peptide or polysaccharide antigen via MHC class II	5	5.80E-03	2.10E-01
	GOTERM_CC_FAT	MHC protein complex	6	8.30E-03	1.80E-01

ILMN_1753064	-0.6260933	0.24519011	TTC13
ILMN_1655077	-0.7821897	0.24446805	PRDM1
ILMN_2148785	-0.5040276	0.24378406	GBP1
ILMN_1682799	-1.0445333	0.24377693	STAMBPL1
ILMN_1750246	-0.6299647	0.2434005	ZBTB1
ILMN_2203271	-2.2243528	0.24243772	FPR3
ILMN_2131467	-0.397852	0.24238987	GNAI1
ILMN_1701195	-0.8428546	0.24176498	PLA2G7
ILMN_2150196	-0.8684133	0.24121656	LRRC25
ILMN_1686623	-1.1544287	0.24103889	CSF1R
ILMN_1773079	-1.0618627	0.24067923	COL3A1
ILMN_1738675	-0.5899928	0.24046308	PTPN6
ILMN_2145033	-1.5628619	0.23933251	CCR5
ILMN_2055156	-0.7168464	0.23903461	PAG1
ILMN_1668411	-0.741487	0.23653066	FHL2
ILMN_2390586	-0.9880923	0.23087709	SP100
ILMN_1795158	-0.4236481	0.23035878	FGR
ILMN_1781155	-0.792679	0.22834751	LYN
ILMN_2148944	-0.5236613	0.22767676	ADCY4
ILMN_2355953	-0.4987555	0.22675383	LILRB4
ILMN_1769129	-2.6877906	0.22659782	CCL19
ILMN_1654586	-0.5864834	0.22578712	RASA3
ILMN_2393765	-3.0009851	0.22573021	IGLL1
ILMN_1800787	-0.8008962	0.2256744	RFTN1
ILMN_1716246	-1.1581569	0.22510645	FRZB
ILMN_1803825	-1.177887	0.22443794	CXCL12
ILMN_1748538	-0.6969305	0.22319187	ALDH1A2
ILMN_1712505	-0.6336315	0.22281049	KDELC1
ILMN_2262044	-0.9458075	0.22100846	PARP10
ILMN_1808405	-1.3138167	0.22031488	HLA-DQA1
ILMN_1672661	-0.6772578	0.21959968	SP110
ILMN_1751079	-0.8478336	0.21942043	TAP1
ILMN_1669703	-1.0861342	0.21830858	TNK2
ILMN_2331087	-0.6249117	0.21772788	MS4A7
ILMN_1842448	-0.6529515	0.21661535	AI866653
ILMN_1661594	-0.6992096	0.21564007	C2orf42
ILMN_1733579	-0.8560787	0.21515703	EVI2A
ILMN_2105919	-0.5137864	0.21502116	FGF2
ILMN_2344283	-1.2531308	0.21422459	FMO3
ILMN_1705247	-0.4828205	0.21087095	ACSL5
ILMN_1763452	-1.3882258	0.2101784	EVI2B
ILMN_2049766	-0.819701	0.21005131	NFE2L3
ILMN_1791447	-0.6749299	0.20970264	CXCL12
ILMN_1797001	-1.04925	0.20879143	DDX58
ILMN_2066858	-1.0778518	0.20609286	TNFSF13B
ILMN_3238525	-0.840078	0.20518312	CARD17
ILMN_1671404	-0.3086096	0.20476213	SVIL
ILMN_1771385	-0.9513072	0.2039975	GBP4
ILMN_2107068	-0.4971687	0.20379758	HOXA2
ILMN_1710923	-0.852091	0.20088993	SLAMF7
ILMN_1701877	-0.7365986	0.19854017	AXL
ILMN_1662026	-2.3509529	0.19770975	BTK
ILMN_2081682	-0.3063551	0.19752866	SMAP2
ILMN_1776723	-0.6018739	0.19542946	PHF11
ILMN_1811301	-0.5809573	0.19494492	INPP5E
ILMN_2162860	-0.6841633	0.19357748	SLFN11
ILMN_1795762	-1.6215433	0.19356228	PLEK

	SP_PIR_KEYWORDS	mhc ii	4	2.30E-02	1.60E-01
	PIR_SUPERFAMILY	PIRSF001991:class II histocompatibility antigen	4	2.40E-02	7.10E-01
	GOTERM_CC_FAT	MHC class II protein complex	4	2.50E-02	4.00E-01
	KEGG_PATHWAY	Antigen processing and presentation	7	3.60E-02	1.80E-01
	GOTERM_MF_FAT	MHC class II receptor activity	3	6.40E-02	5.90E-01
	INTERPRO	MHC class II, alpha/beta chain, N-terminal	3	7.10E-02	7.00E-01
	UP_SEQ_FEATURE	region of interest:Connecting peptide	3	1.80E-01	9.90E-01
**Annotation Cluster 13**	**Enrichment Score: 2.29**		**Count**	**P_Value**	**Benjamini**
	INTERPRO	Immunoglobulin-like fold	31	8.10E-07	5.60E-04
	INTERPRO	Immunoglobulin-like	29	1.10E-06	3.70E-04
	SP_PIR_KEYWORDS	Immunoglobulin domain	26	8.70E-06	2.90E-04
	INTERPRO	Immunoglobulin subtype	18	3.80E-04	4.30E-02
	INTERPRO	Immunoglobulin V-set	14	1.60E-03	1.00E-01
	UP_SEQ_FEATURE	domain:Ig-like C2-type 1	11	5.00E-03	3.60E-01
	UP_SEQ_FEATURE	domain:Ig-like C2-type 2	11	5.20E-03	3.40E-01
	SMART	IG	18	5.80E-03	2.20E-01
	UP_SEQ_FEATURE	domain:Ig-like C2-type 5	4	9.70E-02	9.50E-01
	UP_SEQ_FEATURE	domain:Ig-like C2-type 3	6	9.80E-02	9.50E-01
	INTERPRO	Immunoglobulin	8	1.10E-01	8.20E-01
	UP_SEQ_FEATURE	domain:Ig-like C2-type 4	4	1.90E-01	9.90E-01
	INTERPRO	Immunoglobulin subtype 2	5	5.90E-01	1.00E+00
	INTERPRO	Immunoglobulin I-set	3	7.70E-01	1.00E+00
	SMART	IGc2	5	7.90E-01	1.00E+00
**Annotation Cluster 14**	**Enrichment Score: 2.29**		**Count**	**P_Value**	**Benjamini**
	INTERPRO	Guanylate-binding protein, C-terminal	4	2.60E-04	3.60E-02
	PIR_SUPERFAMILY	PIRSF005552:guanine nucleotide-binding protein 1	4	4.60E-04	8.90E-02
	INTERPRO	Guanylate-binding protein, N-terminal	4	1.20E-03	9.60E-02
	GOTERM_MF_FAT	GTPase activity	10	4.40E-02	4.90E-01
	SP_PIR_KEYWORDS	prenylation	4	6.00E-01	9.40E-01
**Annotation Cluster 15**	**Enrichment Score: 2.26**		**Count**	**P_Value**	**Benjamini**
	GOTERM_BP_FAT	mast cell activation	4	5.50E-04	4.50E-02
	GOTERM_BP_FAT	cell activation during immune response	6	1.10E-03	7.20E-02
	GOTERM_BP_FAT	leukocyte activation during immune response	6	1.10E-03	7.20E-02
	GOTERM_BP_FAT	myeloid leukocyte activation	6	3.30E-03	1.60E-01
	GOTERM_BP_FAT	mast cell degranulation	3	4.70E-03	1.90E-01
	GOTERM_BP_FAT	mast cell mediated immunity	3	4.70E-03	1.90E-01
	GOTERM_BP_FAT	myeloid cell activation during immune response	4	6.80E-03	2.20E-01
	GOTERM_BP_FAT	leukocyte degranulation	3	2.80E-02	4.30E-01
	GOTERM_BP_FAT	myeloid leukocyte mediated immunity	3	4.20E-02	4.70E-01
	GOTERM_BP_FAT	regulated secretory pathway	3	5.90E-02	5.50E-01
**Annotation Cluster 16**	**Enrichment Score: 2.26**		**Count**	**P_Value**	**Benjamini**
	UP_SEQ_FEATURE	glycosylation site:O-linked (Gal...)	5	1.80E-05	4.10E-03
	SP_PIR_KEYWORDS	triple helix	7	2.90E-05	8.80E-04
	SP_PIR_KEYWORDS	hydroxylysine	7	2.90E-05	8.80E-04
	SP_PIR_KEYWORDS	pyroglutamic acid	8	3.20E-05	9.10E-04
	GOTERM_MF_FAT	platelet-derived growth factor binding	5	6.60E-05	3.40E-02
	SP_PIR_KEYWORDS	hydroxyproline	7	8.10E-05	1.90E-03
	SP_PIR_KEYWORDS	trimer	6	1.40E-04	2.90E-03
	GOTERM_CC_FAT	collagen	6	9.50E-04	3.30E-02
	GOTERM_CC_FAT	fibrillar collagen	4	2.00E-03	5.10E-02
	SP_PIR_KEYWORDS	hydroxylation	7	2.90E-03	3.20E-02
	SP_PIR_KEYWORDS	collagen	8	2.90E-03	3.10E-02
	KEGG_PATHWAY	ECM-receptor interaction	9	3.20E-03	3.10E-02
	SP_PIR_KEYWORDS	skin	3	3.80E-03	3.80E-02
	PIR_SUPERFAMILY	PIRSF002255:collagen alpha 1(I) chain	3	5.60E-03	4.30E-01
	UP_SEQ_FEATURE	propeptide:N-terminal propeptide	3	5.70E-03	3.40E-01
	KEGG_PATHWAY	Focal adhesion	14	6.40E-03	4.30E-02

ILMN_1769118	-0.6864126	0.19338933	sept-09
ILMN_1775486	-0.9369748	0.19133283	SSPN
ILMN_2376205	-1.2679341	0.19055454	LTB
ILMN_1802157	-0.4378756	0.1905373	THOC1
ILMN_1761733	-1.1943467	0.19034739	HLA-DMB
ILMN_1766411	-0.4739769	0.19005528	AP1S2
ILMN_1702301	-0.8188756	0.18990526	DOCK10
ILMN_2355831	-0.7947089	0.18943101	FHL2
ILMN_1693009	-0.6449313	0.18806794	FGL2
ILMN_2335072	-0.6986206	0.18791698	TAF1C
ILMN_1682428	-0.4835178	0.18789467	HENMT1
ILMN_1653438	-0.5087999	0.18765485	PHF14
ILMN_1742307	-0.6138526	0.18763377	MEST
ILMN_1803560	-1.0579265	0.18705413	LAT2
ILMN_2196328	-0.9337432	0.18687055	POSTN
ILMN_1674706	-0.5913538	0.18563332	MTHFD2
ILMN_1699217	-0.7707384	0.18500535	C1orf96
ILMN_1653856	-1.3466251	0.18485016	UBASH3B
ILMN_1701789	-2.004902	0.18479297	IFIT3
ILMN_1758315	-1.0181562	0.18420744	SLC9A9
ILMN_1750961	-0.8616837	0.18414715	TM6SF1
ILMN_1728639	-0.5478027	0.18408255	FCGR3B
ILMN_1706779	-0.5501093	0.18396571	LIG1
ILMN_1685680	-0.7034726	0.18370552	SPATA1
ILMN_2175912	-0.9459248	0.18345703	ITGB2
ILMN_1738027	-0.4626849	0.18197875	BRCA1
ILMN_3236858	-0.5093583	0.18181755	NYNRIN
ILMN_2114568	-0.8746334	0.18175074	GBP5
ILMN_2373831	-0.9518257	0.18167904	BTN3A3
ILMN_1674250	-1.2010549	0.1805769	NCKAP1L
ILMN_1771057	-0.7285394	0.18004651	XAB2
ILMN_2399523	-0.495345	0.17881615	JAG2
ILMN_1768482	-2.152835	0.17718779	CD8A
ILMN_2120273	-0.5052606	0.17538813	AP1S2
ILMN_2112580	-0.7201615	0.17467757	FCGR3A
ILMN_1797342	-0.6990012	0.17328602	FNBP1
ILMN_1682761	-0.5960339	0.17276324	C17orf87
ILMN_1684352	-0.5583657	0.17271927	TRIM45
ILMN_3307841	-1.1468112	0.17166331	AGR2
ILMN_2415144	-1.261158	0.17139485	SP110
ILMN_1717261	-0.5074661	0.17112969	HLA-DRB1
ILMN_2123743	-0.851247	0.17013052	FCER1G
ILMN_2320513	-0.6221451	0.1697949	APBB3
ILMN_1672611	-1.1591105	0.16951554	CDH11
ILMN_1750400	-0.8579902	0.16903764	C19orf66
ILMN_1677608	-0.9510541	0.16882162	NEUROD4
ILMN_2109708	-1.0622737	0.16779476	TYMP
ILMN_2053527	-1.2896768	0.16774197	PARP9
ILMN_1854031	-1.0422922	0.16767006	CD722920
ILMN_1716552	-0.3899185	0.16749883	ENAH
ILMN_1751572	-0.9353575	0.16673219	TLE1
ILMN_1790529	-1.4788342	0.16615602	LUM
ILMN_2163873	-0.9824466	0.16482532	FNDC1
ILMN_1693766	-0.5237026	0.16480169	CEP135
ILMN_1781373	-1.2364535	0.16454612	IFIH1
ILMN_1794594	-0.4929295	0.16383012	RASGRP2
ILMN_1697377	-0.733766	0.16293058	ANKRD36B

	INTERPRO	Collagen triple helix repeat	7	6.80E-03	2.40E-01
	GOTERM_BP_FAT	extracellular matrix organization	8	8.20E-03	2.50E-01
	SP_PIR_KEYWORDS	extracellular matrix	12	9.00E-03	8.10E-02
	UP_SEQ_FEATURE	propeptide:C-terminal propeptide	3	1.00E-02	4.80E-01
	UP_SEQ_FEATURE	region of interest:Triple-helical region	4	1.00E-02	4.70E-01
	SP_PIR_KEYWORDS	Ehlers-Danlos syndrome	3	1.90E-02	1.40E-01
	UP_SEQ_FEATURE	domain:Fibrillar collagen NC1	3	1.90E-02	5.60E-01
	INTERPRO	Fibrillar collagen, C-terminal	3	2.00E-02	4.20E-01
	GOTERM_BP_FAT	collagen fibril organization	4	2.60E-02	4.30E-01
	GOTERM_BP_FAT	skin development	4	2.60E-02	4.30E-01
	GOTERM_MF_FAT	growth factor binding	7	2.80E-02	4.60E-01
	GOTERM_BP_FAT	extracellular structure organization	9	2.80E-02	4.30E-01
	SMART	COLFI	3	3.20E-02	4.30E-01
	GOTERM_MF_FAT	extracellular matrix structural constituent	6	4.10E-02	4.90E-01
	GOTERM_CC_FAT	extracellular matrix	14	4.20E-02	5.30E-01
	GOTERM_CC_FAT	extracellular matrix part	7	4.50E-02	5.30E-01
	GOTERM_CC_FAT	proteinaceous extracellular matrix	13	5.20E-02	5.50E-01
	GOTERM_BP_FAT	odontogenesis	4	1.20E-01	7.10E-01
	GOTERM_BP_FAT	epidermis development	7	2.20E-01	8.60E-01
	GOTERM_BP_FAT	ectoderm development	7	2.80E-01	9.00E-01
	GOTERM_MF_FAT	structural molecule activity	13	7.40E-01	1.00E+00
**Annotation Cluster 17**	**Enrichment Score: 2.14**		**Count**	**P_Value**	**Benjamini**
	SP_PIR_KEYWORDS	trimer	6	1.40E-04	2.90E-03
	UP_SEQ_FEATURE	domain:VWFC	4	5.20E-03	3.50E-01
	INTERPRO	von Willebrand factor, type C	4	4.60E-02	5.70E-01
	SMART	VWC	4	8.60E-02	6.70E-01
**Annotation Cluster 18**	**Enrichment Score: 2**		**Count**	**P_Value**	**Benjamini**
	GOTERM_MF_FAT	identical protein binding	27	1.90E-03	1.80E-01
	GOTERM_MF_FAT	protein dimerization activity	21	1.60E-02	5.10E-01
	GOTERM_MF_FAT	protein homodimerization activity	14	3.30E-02	4.70E-01
**Annotation Cluster 19**	**Enrichment Score: 1.99**		**Count**	**P_Value**	**Benjamini**
	INTERPRO	Pleckstrin homology-type	19	4.30E-05	9.70E-03
	INTERPRO	Pleckstrin homology	14	4.00E-03	1.60E-01
	GOTERM_MF_FAT	GTPase regulator activity	17	1.70E-02	4.90E-01
	GOTERM_MF_FAT	nucleoside-triphosphatase regulator activity	17	2.00E-02	4.60E-01
	UP_SEQ_FEATURE	domain:PH	11	2.10E-02	5.80E-01
	GOTERM_MF_FAT	GTPase activator activity	11	2.40E-02	4.50E-01
	SMART	PH	14	3.00E-02	4.40E-01
	GOTERM_MF_FAT	enzyme activator activity	14	3.40E-02	4.60E-01
	SP_PIR_KEYWORDS	GTPase activation	8	4.00E-02	2.40E-01
**Annotation Cluster 20**	**Enrichment Score: 1.94**		**Count**	**P_Value**	**Benjamini**
	GOTERM_BP_FAT	circulatory system process	12	2.90E-03	1.40E-01
	GOTERM_BP_FAT	blood circulation	12	2.90E-03	1.40E-01
	GOTERM_BP_FAT	regulation of blood pressure	5	1.80E-01	8.10E-01
**Annotation Cluster 21**	**Enrichment Score: 1.93**		**Count**	**P_Value**	**Benjamini**
	SP_PIR_KEYWORDS	SH2 domain	9	1.70E-03	2.40E-02
	INTERPRO	SH2 motif	9	1.80E-03	1.00E-01
	SMART	SH2	9	8.40E-03	2.50E-01
	UP_SEQ_FEATURE	domain:SH2	7	1.30E-02	5.10E-01
	KEGG_PATHWAY	Jak-STAT signaling pathway	5	6.80E-01	9.30E-01
**Annotation Cluster 22**	**Enrichment Score: 1.87**		**Count**	**P_Value**	**Benjamini**
	SP_PIR_KEYWORDS	myristylation	5	7.30E-03	7.10E-02
	SP_PIR_KEYWORDS	blocked amino end	7	1.00E-02	8.60E-02
	SP_PIR_KEYWORDS	thiolester bond	4	3.40E-02	2.10E-01
**Annotation Cluster 23**	**Enrichment Score: 1.82**		**Count**	**P_Value**	**Benjamini**
	GOTERM_BP_FAT	response to organic substance	30	1.30E-03	8.20E-02
	GOTERM_BP_FAT	response to hormone stimulus	16	1.70E-02	3.70E-01

ILMN_2053345	-1.0464411	0.16142471	KCNT2
ILMN_2348788	-0.8400175	0.16100022	CD44
ILMN_2129927	-0.4411718	0.16062467	EXT1
ILMN_1754894	-0.6684889	0.16053428	C1orf162
ILMN_1671565	-0.8811511	0.15950754	RNASET2
ILMN_1665738	-0.5216289	0.15925722	FLI1
ILMN_1777325	-0.8521843	0.15826768	STAT1
ILMN_1858599	-0.3757296	0.15656587	RBM43
ILMN_1803429	-1.1652212	0.15593497	CD44
ILMN_1690105	-1.3125963	0.15538809	STAT1
ILMN_1698551	-0.4980893	0.1544606	RCN1
ILMN_1760027	-0.6957563	0.15445444	WAS
ILMN_1689518	-0.6729684	0.15435107	PECAM1
ILMN_1752046	-0.8322395	0.15407388	SH2B3
ILMN_1808590	-1.0341556	0.1538482	GUCY1A3
ILMN_1667947	-0.5844314	0.15268582	NPY1R
ILMN_1797425	-0.5200506	0.15247124	DDX55
ILMN_3252608	-0.7166695	0.15206487	HCRP1
ILMN_1699496	-0.3706721	0.15120575	PHF21A
ILMN_2309848	-1.0085205	0.15017693	FXYD5
ILMN_1726565	-0.4903091	0.15010406	PIK3R2
ILMN_2401770	-0.6743532	0.14875394	PHF14
ILMN_1714523	-0.6782292	0.14785935	HEPH
ILMN_2376723	-1.4681809	0.14697123	CDKN2B
ILMN_1811313	-0.6984525	0.1466987	SLIT3
ILMN_3259146	-0.9624002	0.14500356	BST2
ILMN_1713751	-0.9840141	0.14473715	ADAM19
ILMN_1699931	-1.003425	0.14460169	HCST
ILMN_1725678	-1.1343282	0.14421207	GFRA1
ILMN_1704665	-0.68296	0.14387353	GPM6B
ILMN_1742544	-0.6991372	0.14363457	MEF2C
ILMN_1659075	-0.8373866	0.14355	HLA-DOA
ILMN_1697409	-0.5747517	0.14324132	TNFRSF14
ILMN_1810836	-0.5323594	0.1430238	PDE5A
ILMN_1897405	-0.4541245	0.1429059	CA420307
ILMN_2103761	-0.32567	0.14212725	TLE4
ILMN_1805842	-0.3127734	0.14195496	FHL1
ILMN_1694325	-0.6372663	0.14186426	NFIX
ILMN_1735764	-0.6439887	0.14098056	HTR2B
ILMN_1790149	-0.9141578	0.14082532	HGF
ILMN_1779324	-1.1017444	0.14040168	GZMA
ILMN_2115005	-1.0511112	0.13994657	FGD2
ILMN_1764571	-0.7244104	0.13976014	ARHGAP23
ILMN_1754249	-1.7247378	0.13942018	TARP
ILMN_1796409	-1.4578474	0.1390564	C1QB
ILMN_1768551	-0.7304808	0.13891879	PATL2
ILMN_2061565	-0.8066601	0.13852021	PLCH2
ILMN_1653028	-0.7519424	0.1373429	COL4A1
ILMN_2255133	-2.1671016	0.13648851	BCL11A
ILMN_1796629	-0.806825	0.13480131	EDNRA
ILMN_3252556	-0.9037436	0.13330415	CARD16
ILMN_1748473	-0.7021659	0.13273199	GIMAP4
ILMN_2388547	-1.5174031	0.13248418	EPSTI1
ILMN_1818617	-0.9279863	0.13082033	AL133627
ILMN_1668374	-0.4292723	0.12997523	ITGB5
ILMN_1804571	-0.5105165	0.1295662	ZKSCAN4

	GOTERM_BP_FAT	response to endogenous stimulus	16	3.60E-02	4.40E-01
	GOTERM_BP_FAT	response to steroid hormone stimulus	9	6.30E-02	5.70E-01
**Annotation Cluster 24**	**Enrichment Score: 1.82**		**Count**	**P_Value**	**Benjamini**
	GOTERM_BP_FAT	immune effector process	13	4.20E-05	5.10E-03
	GOTERM_BP_FAT	leukocyte mediated immunity	9	6.20E-04	4.70E-02
	GOTERM_BP_FAT	lymphocyte mediated immunity	6	1.90E-02	4.00E-01
	GOTERM_BP_FAT	adaptive immune response	5	9.10E-02	6.50E-01
	GOTERM_BP_FAT	adaptive immune response based on somatic recombination of immune receptors built from immunoglobulin superfamily domains	5	9.10E-02	6.50E-01
	GOTERM_BP_FAT	B cell mediated immunity	4	1.30E-01	7.30E-01
	GOTERM_BP_FAT	immunoglobulin mediated immune response	3	3.40E-01	9.30E-01
**Annotation Cluster 25**	**Enrichment Score: 1.8**		**Count**	**P_Value**	**Benjamini**
	GOTERM_BP_FAT	response to defenses of other organism during symbiotic interaction	3	1.60E-02	3.60E-01
	GOTERM_BP_FAT	response to host defenses	3	1.60E-02	3.60E-01
	GOTERM_BP_FAT	response to host	3	1.60E-02	3.60E-01
**Annotation Cluster 26**	**Enrichment Score: 1.78**		**Count**	**P_Value**	**Benjamini**
	GOTERM_BP_FAT	exocytosis	9	4.00E-03	1.80E-01
	GOTERM_BP_FAT	secretion by cell	12	6.40E-03	2.20E-01
	GOTERM_BP_FAT	secretion	13	3.50E-02	4.50E-01
	GOTERM_BP_FAT	vesicle-mediated transport	19	8.90E-02	6.50E-01
**Annotation Cluster 27**	**Enrichment Score: 1.76**		**Count**	**P_Value**	**Benjamini**
	GOTERM_BP_FAT	regulation of cytokine production	12	2.30E-03	1.20E-01
	GOTERM_BP_FAT	positive regulation of multicellular organismal process	11	4.50E-02	4.80E-01
	GOTERM_BP_FAT	positive regulation of cytokine production	6	4.90E-02	5.10E-01
**Annotation Cluster 28**	**Enrichment Score: 1.65**		**Count**	**P_Value**	**Benjamini**
	GOTERM_BP_FAT	antigen processing and presentation	8	2.40E-03	1.20E-01
	GOTERM_BP_FAT	antigen processing and presentation of exogenous peptide antigen	3	2.30E-02	4.20E-01
	GOTERM_BP_FAT	antigen processing and presentation of exogenous antigen	3	3.70E-02	4.50E-01
	GOTERM_BP_FAT	antigen processing and presentation of peptide antigen	3	1.30E-01	7.30E-01
**Annotation Cluster 29**	**Enrichment Score: 1.63**		**Count**	**P_Value**	**Benjamini**
	GOTERM_BP_FAT	positive regulation of cytokine biosynthetic process	6	4.00E-03	1.80E-01
	GOTERM_BP_FAT	regulation of cytokine biosynthetic process	7	5.80E-03	2.10E-01
	GOTERM_BP_FAT	positive regulation of interleukin-2 biosynthetic process	3	3.20E-02	4.40E-01
	GOTERM_BP_FAT	regulation of interleukin-2 biosynthetic process	3	6.50E-02	5.70E-01
	GOTERM_BP_FAT	regulation of interleukin-2 production	3	1.40E-01	7.60E-01
**Annotation Cluster 30**	**Enrichment Score: 1.63**		**Count**	**P_Value**	**Benjamini**
	SP_PIR_KEYWORDS	gtp-binding	14	1.50E-02	1.20E-01
	UP_SEQ_FEATURE	nucleotide phosphate-binding region:GTP	13	1.60E-02	5.40E-01
	GOTERM_MF_FAT	guanyl ribonucleotide binding	16	2.20E-02	4.50E-01
	GOTERM_MF_FAT	guanyl nucleotide binding	16	2.20E-02	4.50E-01
	GOTERM_MF_FAT	GTP binding	15	3.60E-02	4.60E-01
	GOTERM_MF_FAT	GTPase activity	10	4.40E-02	4.90E-01
**Annotation Cluster 31**	**Enrichment Score: 1.62**		**Count**	**P_Value**	**Benjamini**
	GOTERM_BP_FAT	cell motion	22	2.00E-03	1.10E-01
	GOTERM_BP_FAT	localization of cell	14	1.90E-02	3.90E-01
	GOTERM_BP_FAT	cell motility	14	1.90E-02	3.90E-01
	GOTERM_BP_FAT	cell migration	13	2.00E-02	4.00E-01
	GOTERM_BP_FAT	leukocyte migration	5	3.70E-02	4.50E-01
	GOTERM_BP_FAT	neuron development	10	3.40E-01	9.30E-01
**Annotation Cluster 32**	**Enrichment Score: 1.62**		**Count**	**P_Value**	**Benjamini**
	GOTERM_BP_FAT	skeletal system development	15	1.10E-02	3.10E-01
	GOTERM_BP_FAT	osteoblast differentiation	5	1.40E-02	3.30E-01
	GOTERM_BP_FAT	ossification	8	1.40E-02	3.40E-01
	GOTERM_BP_FAT	bone development	8	1.90E-02	3.90E-01
	GOTERM_BP_FAT	biomineral formation	3	2.00E-01	8.30E-01
**Annotation Cluster 33**	**Enrichment Score: 1.61**		**Count**	**P_Value**	**Benjamini**
	UP_SEQ_FEATURE	repeat:HAT 5	3	1.90E-02	5.60E-01

ILMN_1848095	-0.8107949	0.12893517	NA
ILMN_1695640	-1.2297163	0.12878244	PTPN22
ILMN_1682165	-0.5414724	0.12867746	NT5C2
ILMN_2222688	-0.4617698	0.12545523	TMSB4X
ILMN_3222425	-0.4439984	0.12462959	LOC729852
ILMN_1768394	-0.2999259	0.12444508	ARPC5
ILMN_1814022	-0.4016976	0.12436828	NR1H3
ILMN_3238116	-0.571442	0.12429227	NBPF11
ILMN_1711383	-0.4462715	0.12396342	STK4
ILMN_1812926	-0.5183303	0.12353233	ANTXR2
ILMN_1805448	-0.4803053	0.12333091	EPB41L2
ILMN_1656111	-0.3010579	0.12267488	MYLIP
ILMN_1752520	-0.6901116	0.12224111	SLFN11
ILMN_1791873	-0.6042855	0.12214205	COG1
ILMN_1690921	-0.475829	0.12163639	STAT2
ILMN_1737833	-0.5696403	0.12110119	ATN1
ILMN_2326509	-2.0144005	0.120608	CASP1
ILMN_2208413	-1.40638	0.1202912	ARHGAP15
ILMN_1727402	-0.9682615	0.11974773	HCLS1
ILMN_1711617	-0.8764327	0.11954744	GMFG
ILMN_1777998	-0.769028	0.11922258	ARHGAP25
ILMN_1662932	-1.3524692	0.11833018	LCP1
ILMN_1815154	-0.3131096	0.11772004	MYH10
ILMN_1739541	-0.5683731	0.11725833	NMI
ILMN_1815673	-0.8984177	0.11664414	DKK3
ILMN_1813753	-0.8199365	0.11578794	PTN
ILMN_2364521	-0.8218051	0.11534408	AXL
ILMN_1663080	-1.0312574	0.11457998	LFNG
ILMN_1803423	-0.8187965	0.11438009	ARHGEF6
ILMN_1738832	-0.9569222	0.11290038	SACS
ILMN_1747968	-0.5250922	0.11259931	RBM33
ILMN_1718063	-0.3632585	0.11231238	LIPA
ILMN_1698419	-0.3359468	0.1099484	NCOR2
ILMN_3300663	-0.4206458	0.10986987	SLC35E2B
ILMN_2347805	-0.4750857	0.10956416	EXOC1
ILMN_2229877	-0.8076528	0.10956222	PCDH18
ILMN_1810910	-0.8282412	0.10938713	CFH
ILMN_2077406	-0.7479565	0.10932532	CCDC18
ILMN_3245625	-0.3613911	0.10917917	RFX7
ILMN_1728478	-0.4733229	0.10900313	CXCL16
ILMN_1806790	-0.5863853	0.10892174	ROBO1
ILMN_2253732	-0.8138144	0.10890111	ST8SIA4
ILMN_3240586	-0.4137484	0.10812488	PLD6
ILMN_3242725	-0.5643714	0.10779354	KIF26A
ILMN_2246328	-1.1970055	0.10777731	PTPN22
ILMN_1705442	-0.6908666	0.10772623	CMTM3
ILMN_1733538	-0.8262795	0.10733758	RGS10
ILMN_3243185	-0.5303395	0.10715491	RERGL
ILMN_1795930	-0.8828546	0.10697748	PTGER4
ILMN_1802109	-0.6104525	0.10639039	KLHL29
ILMN_1665686	-0.5991011	0.10587843	FAM38B
ILMN_1761941	-0.5115597	0.1056436	FAM198B
ILMN_1683494	-0.6096175	0.10531268	TMEM154
ILMN_1655595	-0.3709378	0.10528863	SERPINE2
ILMN_2317581	-0.6959561	0.10484695	SHANK3
ILMN_1776213	-0.681404	0.10361286	RGMB

	UP_SEQ_FEATURE	repeat:HAT 4	3	2.30E-02	5.90E-01
	UP_SEQ_FEATURE	repeat:HAT 3	3	2.70E-02	6.40E-01
	UP_SEQ_FEATURE	repeat:HAT 1	3	2.70E-02	6.40E-01
	UP_SEQ_FEATURE	repeat:HAT 2	3	2.70E-02	6.40E-01
**Annotation Cluster 34**	**Enrichment Score: 1.6**		**Count**	**P_Value**	**Benjamini**
	SP_PIR_KEYWORDS	LIM domain	6	1.30E-02	1.10E-01
	INTERPRO	Zinc finger, LIM-type	6	1.60E-02	3.60E-01
	UP_SEQ_FEATURE	domain:LIM zinc-binding 2	5	1.80E-02	5.30E-01
	UP_SEQ_FEATURE	domain:LIM zinc-binding 1	5	1.80E-02	5.30E-01
	SMART	LIM	6	4.20E-02	4.90E-01
	UP_SEQ_FEATURE	domain:LIM zinc-binding 3	3	8.80E-02	9.50E-01
**Annotation Cluster 35**	**Enrichment Score: 1.58**		**Count**	**P_Value**	**Benjamini**
	SP_PIR_KEYWORDS	heparin-binding	6	9.40E-03	8.20E-02
	GOTERM_MF_FAT	glycosaminoglycan binding	9	1.20E-02	5.00E-01
	GOTERM_MF_FAT	polysaccharide binding	9	2.00E-02	4.50E-01
	GOTERM_MF_FAT	pattern binding	9	2.00E-02	4.50E-01
	GOTERM_MF_FAT	heparin binding	7	2.60E-02	4.60E-01
	GOTERM_MF_FAT	carbohydrate binding	11	2.60E-01	9.10E-01
**Annotation Cluster 36**	**Enrichment Score: 1.56**		**Count**	**P_Value**	**Benjamini**
	GOTERM_BP_FAT	regulation of programmed cell death	28	2.20E-02	4.10E-01
	GOTERM_BP_FAT	regulation of cell death	28	2.30E-02	4.20E-01
	GOTERM_BP_FAT	induction of apoptosis	14	2.60E-02	4.30E-01
	GOTERM_BP_FAT	induction of programmed cell death	14	2.60E-02	4.20E-01
	GOTERM_BP_FAT	positive regulation of apoptosis	17	3.00E-02	4.40E-01
	GOTERM_BP_FAT	positive regulation of programmed cell death	17	3.10E-02	4.40E-01
	GOTERM_BP_FAT	positive regulation of cell death	17	3.30E-02	4.40E-01
	GOTERM_BP_FAT	regulation of apoptosis	27	3.30E-02	4.40E-01
**Annotation Cluster 37**	**Enrichment Score: 1.55**		**Count**	**P_Value**	**Benjamini**
	INTERPRO	Zinc finger, PHD-type	8	2.20E-03	1.00E-01
	INTERPRO	Zinc finger, PHD-type, conserved site	8	2.70E-03	1.10E-01
	INTERPRO	Zinc finger, PHD-finger	7	7.20E-03	2.40E-01
	SMART	PHD	8	9.00E-03	2.30E-01
	UP_SEQ_FEATURE	zinc finger region:PHD-type	4	8.60E-02	9.50E-01
	INTERPRO	Zinc finger, RING-type	7	5.50E-01	1.00E+00
	SMART	RING	7	7.90E-01	1.00E+00
**Annotation Cluster 38**	**Enrichment Score: 1.53**		**Count**	**P_Value**	**Benjamini**
	SP_PIR_KEYWORDS	cytoskeleton	23	8.60E-03	7.90E-02
	GOTERM_MF_FAT	cytoskeletal protein binding	20	1.60E-02	5.20E-01
	SP_PIR_KEYWORDS	actin-binding	11	2.60E-02	1.80E-01
	GOTERM_MF_FAT	actin binding	14	2.80E-02	4.70E-01
	GOTERM_MF_FAT	actin filament binding	5	2.90E-02	4.50E-01
	GOTERM_CC_FAT	actin cytoskeleton	9	2.50E-01	9.10E-01
**Annotation Cluster 39**	**Enrichment Score: 1.52**		**Count**	**P_Value**	**Benjamini**
	INTERPRO	Bromodomain	5	8.20E-03	2.50E-01
	SMART	BROMO	5	2.00E-02	3.90E-01
	INTERPRO	Bromodomain, conserved site	4	3.50E-02	5.60E-01
	SP_PIR_KEYWORDS	bromodomain	4	4.20E-02	2.40E-01
	UP_SEQ_FEATURE	domain:Bromo	3	1.10E-01	9.50E-01
**Annotation Cluster 40**	**Enrichment Score: 1.52**		**Count**	**P_Value**	**Benjamini**
	GOTERM_BP_FAT	integrin-mediated signaling pathway	7	4.40E-03	1.80E-01
	INTERPRO	von Willebrand factor, type A	6	2.10E-02	4.30E-01
	GOTERM_CC_FAT	integrin complex	4	2.50E-02	4.00E-01
	SP_PIR_KEYWORDS	integrin	4	4.80E-02	2.60E-01
	SMART	VWA	6	5.50E-02	5.60E-01
	UP_SEQ_FEATURE	domain:VWFA	4	1.30E-01	9.70E-01
**Annotation Cluster 41**	**Enrichment Score: 1.5**		**Count**	**P_Value**	**Benjamini**

ILMN_1791702	-0.2409067	0.10345653	SMARCA2
ILMN_2042595	-0.5229749	0.10331385	PCM1
ILMN_1740430	-0.310527	0.10329175	SLC2A4RG
ILMN_1739428	-1.0048952	0.10326946	IFIT2
ILMN_2408851	-0.7082942	0.102796	ARHGAP30
ILMN_1677239	-0.4022552	0.10207376	CCDC14
ILMN_1670926	-0.9411953	0.10141872	CHST15
ILMN_1652000	-0.5560677	0.10119058	FAM156B
ILMN_2230892	-0.6070417	0.10088368	IL10RB
ILMN_2157951	-0.4193257	0.10070315	STX6
ILMN_1691444	-0.5584114	0.10005394	UHRF2
ILMN_1697268	-0.836865	0.09821321	EMILIN2
ILMN_1749915	-0.5981271	0.09710187	C1orf63
ILMN_1703477	-0.5192355	0.09669668	ARHGEF2
ILMN_2171289	-1.8790541	0.09620509	SAMSN1
ILMN_1684497	-0.630926	0.09565401	PYROXD2
ILMN_1734543	-0.692723	0.09520887	PTPRE
ILMN_3307926	-0.5134761	0.09490384	ADRBK1
ILMN_1709795	-1.5240674	0.09409073	RAC2
ILMN_1716264	-1.9172883	0.09344087	ANKRD1
ILMN_1898682	-0.3377023	0.09318444	PHIP
ILMN_2069128	-0.5487559	0.09207791	EPB41L2
ILMN_1691539	-0.5962633	0.0919627	LAT
ILMN_2167805	-0.7360011	0.09138069	LUM
ILMN_3230435	-0.5040896	0.09065887	VOPP1
ILMN_1893633	-0.8568681	0.08968221	LOC439949
ILMN_1694840	-0.8034787	0.0894372	MATN2
ILMN_1741942	-0.398401	0.08924177	STX16
ILMN_1776157	-0.7247066	0.08898276	sept-04
ILMN_1666775	-0.7897341	0.08870249	CACNA1C
ILMN_1707695	-2.0889034	0.08849268	IFIT1
ILMN_1887174	-0.5456803	0.08846726	KIAA0146
ILMN_1742789	-0.891553	0.0884307	LPXN
ILMN_1767665	-0.9345106	0.08790112	GPX8
ILMN_2082273	-0.4205569	0.08783904	RGS5
ILMN_1799487	-0.5319377	0.08777045	N4BP2L1
ILMN_2398159	-0.8470689	0.0872646	DKK3
ILMN_2098947	-0.4422655	0.08719556	AK095700
ILMN_1794782	-0.7457796	0.0850738	ABCG1
ILMN_1745607	-0.7011312	0.08489525	A2M
ILMN_1666785	-0.3553518	0.08374754	SYNE1
ILMN_2148459	-0.6083047	0.08253118	B2M
ILMN_1791912	-0.6120568	0.08252943	SIDT2
ILMN_2086470	-0.5840968	0.08087385	PDGFRA
ILMN_1678841	-0.740746	0.08058992	UBD
ILMN_2057981	-0.6686943	0.07909441	FAM164A
ILMN_1898518	-0.7783019	0.07836554	GFRA1
ILMN_1911042	-0.320335	0.07807413	PIP4K2A
ILMN_1806752	-0.5997548	0.07768083	PLEKHH2
ILMN_1795181	-1.0102658	0.07754815	DDX60
ILMN_1757552	-0.418643	0.0775457	PTRF
ILMN_1687501	-1.0911491	0.07745464	MOXD1
ILMN_1706643	-0.6055509	0.07680008	COL6A3
ILMN_1665909	-0.297439	0.07678253	LASP1

	GOTERM_BP_FAT	positive regulation of adaptive immune response based on somatic recombination of immune receptors built from immunoglobulin superfamily domains	4	2.80E-02	4.30E-01
	GOTERM_BP_FAT	positive regulation of adaptive immune response	4	3.00E-02	4.40E-01
	GOTERM_BP_FAT	regulation of adaptive immune response based on somatic recombination of immune receptors built from immunoglobulin superfamily domains	5	3.30E-02	4.40E-01
	GOTERM_BP_FAT	regulation of adaptive immune response	5	3.50E-02	4.40E-01
**Annotation Cluster 42**	**Enrichment Score: 1.5**		**Count**	**P_Value**	**Benjamini**
	GOTERM_BP_FAT	negative regulation of transcription, DNA-dependent	17	5.80E-03	2.10E-01
	GOTERM_BP_FAT	negative regulation of transcription from RNA polymerase II promoter	14	6.30E-03	2.20E-01
	GOTERM_BP_FAT	negative regulation of RNA metabolic process	17	6.80E-03	2.30E-01
	GOTERM_BP_FAT	negative regulation of biosynthetic process	23	8.50E-03	2.60E-01
	GOTERM_BP_FAT	negative regulation of cellular biosynthetic process	22	1.30E-02	3.30E-01
	GOTERM_BP_FAT	regulation of transcription from RNA polymerase II promoter	26	1.90E-02	3.90E-01
	GOTERM_BP_FAT	negative regulation of nitrogen compound metabolic process	20	2.20E-02	4.10E-01
	GOTERM_BP_FAT	negative regulation of transcription	18	2.70E-02	4.20E-01
	GOTERM_BP_FAT	negative regulation of macromolecule biosynthetic process	20	3.50E-02	4.50E-01
	GOTERM_BP_FAT	negative regulation of nucleobase, nucleoside, nucleotide and nucleic acid metabolic process	19	3.60E-02	4.40E-01
	GOTERM_BP_FAT	negative regulation of gene expression	18	5.50E-02	5.50E-01
	GOTERM_BP_FAT	negative regulation of macromolecule metabolic process	21	1.90E-01	8.20E-01
	GOTERM_BP_FAT	regulation of transcription, DNA-dependent	41	4.70E-01	9.70E-01
	GOTERM_BP_FAT	regulation of RNA metabolic process	41	5.30E-01	9.80E-01
**Annotation Cluster 43**	**Enrichment Score: 1.49**		**Count**	**P_Value**	**Benjamini**
	INTERPRO	Interferon regulatory factor-3	3	7.90E-03	2.50E-01
	UP_SEQ_FEATURE	DNA-binding region:Tryptophan pentad repeat	3	1.30E-02	5.00E-01
	INTERPRO	Interferon regulatory factor	3	1.30E-02	3.50E-01
	INTERPRO	Interferon regulatory factor, conserved site	3	1.30E-02	3.50E-01
	SMART	IRF	3	2.20E-02	3.80E-01
	INTERPRO	SMAD domain-like	3	3.50E-02	5.50E-01
	GOTERM_MF_FAT	RNA polymerase II transcription factor activity	8	2.90E-01	9.30E-01
	INTERPRO	Winged helix repressor DNA-binding	6	3.20E-01	9.80E-01
**Annotation Cluster 44**	**Enrichment Score: 1.47**		**Count**	**P_Value**	**Benjamini**
	GOTERM_BP_FAT	actin cytoskeleton organization	11	2.90E-02	4.30E-01
	GOTERM_BP_FAT	cytoskeleton organization	17	3.30E-02	4.40E-01
	GOTERM_BP_FAT	actin filament-based process	11	4.20E-02	4.70E-01
**Annotation Cluster 45**	**Enrichment Score: 1.46**		**Count**	**P_Value**	**Benjamini**
	GOTERM_BP_FAT	regulation of cell activation	11	5.60E-03	2.10E-01
	GOTERM_BP_FAT	regulation of lymphocyte activation	10	5.60E-03	2.10E-01
	GOTERM_BP_FAT	negative regulation of immune system process	7	1.00E-02	2.90E-01
	GOTERM_BP_FAT	regulation of leukocyte activation	10	1.20E-02	3.10E-01
	GOTERM_BP_FAT	negative regulation of lymphocyte activation	4	1.20E-01	7.10E-01
	GOTERM_BP_FAT	negative regulation of leukocyte activation	4	1.30E-01	7.40E-01
	GOTERM_BP_FAT	negative regulation of cell activation	4	1.50E-01	7.70E-01
	GOTERM_BP_FAT	negative regulation of T cell activation	3	2.40E-01	8.70E-01
**Annotation Cluster 46**	**Enrichment Score: 1.43**		**Count**	**P_Value**	**Benjamini**
	INTERPRO	Tumour necrosis factor-like	6	2.00E-03	1.00E-01
	INTERPRO	Tumor Necrosis Factor	3	4.50E-02	5.80E-01
	SMART	TNF	3	7.10E-02	6.30E-01
	GOTERM_MF_FAT	tumor necrosis factor receptor binding	3	7.70E-02	6.30E-01
	GOTERM_MF_FAT	tumor necrosis factor receptor superfamily binding	3	1.50E-01	7.80E-01
**Annotation Cluster 47**	**Enrichment Score: 1.42**		**Count**	**P_Value**	**Benjamini**
	GOTERM_BP_FAT	response to nutrient	9	1.30E-02	3.20E-01
	GOTERM_BP_FAT	response to vitamin A	5	1.40E-02	3.30E-01
	GOTERM_BP_FAT	response to extracellular stimulus	11	2.50E-02	4.20E-01
	GOTERM_BP_FAT	response to vitamin	5	5.80E-02	5.50E-01
	GOTERM_BP_FAT	response to nutrient levels	9	7.10E-02	6.00E-01
	GOTERM_BP_FAT	response to retinoic acid	3	1.60E-01	8.00E-01

ILMN_1703487	-0.4389223	0.07673918	LMO4
ILMN_1799672	-0.4509294	0.07661649	CROCC
ILMN_1731086	-0.4449785	0.07578874	ISL2
ILMN_3241046	-1.3381329	0.07547525	MYBL1
ILMN_1713449	-0.8047564	0.07521348	TBX3
ILMN_2323944	-0.4712394	0.07488582	FAM110A
ILMN_1657683	-0.5632711	0.07470582	C1orf198
ILMN_1732799	-0.6397462	0.07385269	CD34
ILMN_1651826	-0.8626967	0.07272068	BASP1
ILMN_3245066	-0.3758309	0.07259768	DENND4B
ILMN_1742618	-3.0690904	0.071611	XAF1
ILMN_1715392	-0.4913739	0.06900385	PRPF3
ILMN_1673933	-0.4612814	0.06829594	NPIP
ILMN_1794470	-0.4384409	0.06795633	ANKFY1
ILMN_1678842	-0.9679654	0.06760374	THBS2
ILMN_1652512	-0.5816504	0.06735905	C2CD2
ILMN_2122103	-0.4310925	0.06677511	ETS1
ILMN_1663195	-0.5292518	0.06537721	MCM7
ILMN_2407389	-0.6593715	0.06515683	GPNMB
ILMN_2224143	-0.3779939	0.06345493	MCM3
ILMN_1808634	-0.3223416	0.06081992	DRAM2
ILMN_1829768	-1.0059533	0.05623433	BC047110
ILMN_1693538	-0.3450073	0.0555692	STK36
ILMN_1764709	-0.5225451	0.05520299	MAFB
ILMN_1778691	-0.5633244	0.05478557	TIA1
ILMN_1731224	-1.1919034	0.05411996	PARP9
ILMN_3244110	-0.426818	0.05406091	FAM156B
ILMN_1678961	-0.4991477	0.05171973	FRMD4A
ILMN_1864685	-0.7294178	0.04989669	BC062365
ILMN_1708006	-0.8261792	0.0494976	MICA
ILMN_3207230	-0.5748084	0.04940914	LOC283588
ILMN_2104356	-1.2947727	0.04938726	COL1A2
ILMN_2131523	-0.8579557	0.04890701	SACS
ILMN_1677038	-1.0649506	0.04827893	C7orf58
ILMN_1660844	-0.5447026	0.04755662	INTS4
ILMN_2277334	-0.5340706	0.04749291	KCNK2
ILMN_3236156	-0.3006278	0.04617408	OSTC
ILMN_1774077	-0.7700593	0.0439755	GBP2
ILMN_1658926	-0.5617282	0.04273477	NOTCH3
ILMN_1750075	-0.4129226	0.04257144	DMTF1
ILMN_1651346	-0.824809	0.04253814	TICAM2
ILMN_1668526	-1.3196023	0.04111992	GVIN1
ILMN_1701308	-1.2155994	0.03902149	COL1A1
ILMN_1773059	-0.6809862	0.03829685	GPR124
ILMN_1662773	-0.4721382	0.03742247	SNURF-SNRPN
ILMN_2396991	-1.237827	0.03555138	HCST
ILMN_1809094	-0.8183383	0.03473254	AX747733
ILMN_2131177	-0.6153819	0.03458107	GUCY1A3
ILMN_1782412	-1.0307853	0.03383414	IRX2
ILMN_2223941	-0.5435536	0.03249535	FBLN5
ILMN_1666594	-1.4824603	0.03224498	IRF8
ILMN_2129234	-0.3020076	0.03192231	TMEM47
ILMN_1760062	-2.460666	0.03003611	IFI44
ILMN_3300358	-0.7018808	0.02966945	ZNF84
ILMN_1721704	-0.3054528	0.02947792	FNTA
ILMN_2383693	-0.404843	0.02874064	UPF2
ILMN_2233366	-0.453265	0.0280124	ASAP1

			Count	P_Value	Benjamini
**Annotation Cluster 48**	**Enrichment Score: 1.42**		**Count**	**P_Value**	**Benjamini**
	INTERPRO	Tumour necrosis factor-like	6	2.00E-03	1.00E-01
	SP_PIR_KEYWORDS	collagen	8	2.90E-03	3.10E-02
	UP_SEQ_FEATURE	domain:C1q	3	1.30E-01	9.70E-01
	INTERPRO	Complement C1q protein	3	1.30E-01	8.50E-01
	UP_SEQ_FEATURE	domain:Collagen-like	3	1.60E-01	9.80E-01
	SMART	C1Q	3	2.00E-01	8.30E-01
**Annotation Cluster 49**	**Enrichment Score: 1.41**		**Count**	**P_Value**	**Benjamini**
	GOTERM_BP_FAT	regulation of T cell activation	9	4.40E-03	1.90E-01
	GOTERM_BP_FAT	regulation of cell activation	11	5.60E-03	2.10E-01
	GOTERM_BP_FAT	regulation of lymphocyte activation	10	5.60E-03	2.10E-01
	GOTERM_BP_FAT	regulation of leukocyte activation	10	1.20E-02	3.10E-01
	GOTERM_BP_FAT	regulation of T cell proliferation	4	1.60E-01	7.80E-01
	GOTERM_BP_FAT	regulation of lymphocyte proliferation	4	2.80E-01	9.00E-01
	GOTERM_BP_FAT	regulation of leukocyte proliferation	4	2.80E-01	9.10E-01
	GOTERM_BP_FAT	regulation of mononuclear cell proliferation	4	2.80E-01	9.10E-01
**Annotation Cluster 50**	**Enrichment Score: 1.39**		**Count**	**P_Value**	**Benjamini**
	GOTERM_BP_FAT	positive regulation of defense response	6	2.30E-02	4.10E-01
	GOTERM_BP_FAT	regulation of inflammatory response	6	2.60E-02	4.30E-01
	GOTERM_BP_FAT	positive regulation of inflammatory response	4	2.80E-02	4.30E-01
	GOTERM_BP_FAT	positive regulation of response to external stimulus	5	5.30E-02	5.30E-01
	GOTERM_BP_FAT	regulation of response to external stimulus	8	6.30E-02	5.70E-01
	GOTERM_BP_FAT	regulation of acute inflammatory response	3	7.80E-02	6.20E-01
**Annotation Cluster 51**	**Enrichment Score: 1.39**		**Count**	**P_Value**	**Benjamini**
	UP_SEQ_FEATURE	propeptide:Removed in mature form	11	1.70E-02	5.40E-01
	UP_SEQ_FEATURE	lipid moiety-binding region:GPI-anchor amidated serine	5	2.30E-02	5.90E-01
	SP_PIR_KEYWORDS	gpi-anchor	7	4.00E-02	2.30E-01
	GOTERM_CC_FAT	anchored to membrane	10	5.60E-02	5.60E-01
	SP_PIR_KEYWORDS	phosphatidylinositol linkage	3	1.30E-01	4.90E-01
**Annotation Cluster 52**	**Enrichment Score: 1.32**		**Count**	**P_Value**	**Benjamini**
	GOTERM_BP_FAT	apoptosis	22	2.60E-02	4.30E-01
	GOTERM_BP_FAT	cell death	25	2.90E-02	4.30E-01
	GOTERM_BP_FAT	programmed cell death	22	3.00E-02	4.40E-01
	GOTERM_BP_FAT	death	25	3.00E-02	4.40E-01
	SP_PIR_KEYWORDS	Apoptosis	10	3.50E-01	7.70E-01
**Annotation Cluster 53**	**Enrichment Score: 1.29**		**Count**	**P_Value**	**Benjamini**
	SP_PIR_KEYWORDS	GTPase activation	8	4.00E-02	2.40E-01
	UP_SEQ_FEATURE	domain:Rho-GAP	5	4.10E-02	7.80E-01
	INTERPRO	RhoGAP	5	4.50E-02	5.80E-01
	SMART	RhoGAP	5	9.70E-02	6.70E-01
**Annotation Cluster 54**	**Enrichment Score: 1.26**		**Count**	**P_Value**	**Benjamini**
	GOTERM_BP_FAT	regulation of immune effector process	8	7.00E-03	2.30E-01
	GOTERM_BP_FAT	regulation of defense response to virus	3	6.50E-02	5.70E-01
	GOTERM_BP_FAT	regulation of response to biotic stimulus	3	1.30E-01	7.40E-01
	GOTERM_BP_FAT	regulation of multi-organism process	3	1.50E-01	7.70E-01
**Annotation Cluster 55**	**Enrichment Score: 1.26**		**Count**	**P_Value**	**Benjamini**
	UP_SEQ_FEATURE	domain:EGF-like 9	5	1.00E-03	1.10E-01
	UP_SEQ_FEATURE	domain:EGF-like 8	4	1.30E-02	4.90E-01
	UP_SEQ_FEATURE	domain:EGF-like 7	4	1.60E-02	5.40E-01
	UP_SEQ_FEATURE	domain:EGF-like 3	6	1.70E-02	5.50E-01
	INTERPRO	EGF-like region, conserved site	12	3.50E-02	5.70E-01
	SP_PIR_KEYWORDS	egf-like domain	10	4.10E-02	2.40E-01
	INTERPRO	EGF-like, type 3	9	4.20E-02	5.90E-01
	INTERPRO	EGF	7	4.30E-02	5.90E-01
	INTERPRO	EGF-like calcium-binding	6	4.40E-02	5.90E-01
	UP_SEQ_FEATURE	domain:EGF-like 5	4	4.80E-02	8.20E-01
	UP_SEQ_FEATURE	domain:EGF-like 6; calcium-binding	3	8.80E-02	9.50E-01

ILMN_1666208	-0.7825285	0.02779676	MIS18BP1
ILMN_2328094	-0.7981768	0.02583879	DACT1
ILMN_1792506	-0.616087	0.02408154	PLA1A
ILMN_1790136	-0.4774218	0.02381867	C20orf20
ILMN_2402172	-0.7193549	0.02330241	sept-04
ILMN_1807042	-0.8996551	0.02306721	MARCKS
ILMN_1812473	-0.5861107	0.02185519	MLLT3
ILMN_1717934	-0.7884955	0.02136703	SYT11
ILMN_1767448	-0.5449091	0.01176193	LHFP
ILMN_1701441	-0.8963832	0.01134446	LPAR1
ILMN_1755727	-0.4310157	0.01116782	KDM5B
ILMN_1836958	-0.7591832	0.01066616	BM703456
ILMN_2395373	-0.711017	0.00998119	GABBR1
ILMN_1678143	-0.7815368	0.00972039	ARHGDIB
ILMN_1789007	-1.7765667	0.0091216	APOC1
ILMN_1778238	-0.4870963	0.00862525	UTP6
ILMN_1742230	-0.6534504	0.0061385	BAZ1A
ILMN_3231881	-0.358492	0.0047442	PTMA
ILMN_1711894	-1.7565196	0.00328988	MYB
ILMN_1691731	-1.3722534	-5.04E-04	PARP14
ILMN_1753101	-1.2060335	-0.0021272	VTCN1
ILMN_2344650	-0.5655251	-0.003253	N4BP2L1
ILMN_1714809	-0.4781135	-0.0040298	RPIA
ILMN_3220952	-0.9335808	-0.0156769	ANKRD36
ILMN_2325028	-0.8411629	-0.0159033	ODF2L
ILMN_1700448	-0.9654986	-0.0248813	SIM2
ILMN_1830984	-0.8059158	-0.0326325	BF509118
ILMN_1736670	-0.9749128	-0.0442319	PPP1R3C
ILMN_2088437	-1.4015719	-0.0448213	CX3CR1
ILMN_1710937	-1.2329658	-0.0533357	IFI16
ILMN_1706502	-0.7579128	-0.0572491	EIF2AK2
ILMN_2351466	-2.0834424	-0.0743181	NTM
ILMN_1663866	-1.4258738	-0.081615	TGFBI
ILMN_1723912	-3.1892138	-0.1006719	IFI44L
ILMN_1835092	-2.9079618	-0.1650913	IFI44L

	UP_SEQ_FEATURE	domain:EGF-like 1	6	9.30E-02	9.50E-01
	UP_SEQ_FEATURE	domain:EGF-like 2	5	1.00E-01	9.50E-01
	SMART	EGF_CA	6	1.10E-01	6.80E-01
	UP_SEQ_FEATURE	domain:EGF-like 4	4	1.10E-01	9.50E-01
	INTERPRO	EGF-like	8	1.10E-01	8.20E-01
	INTERPRO	EGF-like calcium-binding, conserved site	5	1.30E-01	8.50E-01
	INTERPRO	EGF-type aspartate/asparagine hydroxylation conserved site	5	1.30E-01	8.50E-01
	INTERPRO	EGF calcium-binding	4	1.80E-01	8.90E-01
	UP_SEQ_FEATURE	domain:EGF-like 6	3	2.10E-01	9.90E-01
	SMART	EGF	8	2.70E-01	9.00E-01
**Annotation Cluster 56**	**Enrichment Score: 1.26**		**Count**	**P_Value**	**Benjamini**
	BIOCARTA	Neutrophil and Its Surface Molecules	3	4.20E-02	8.40E-01
	BIOCARTA	Adhesion Molecules on Lymphocyte	3	5.30E-02	8.20E-01
	BIOCARTA	Monocyte and its Surface Molecules	3	7.60E-02	8.70E-01
**Annotation Cluster 57**	**Enrichment Score: 1.22**		**Count**	**P_Value**	**Benjamini**
	GOTERM_CC_FAT	endosome membrane	5	3.70E-02	5.00E-01
	GOTERM_CC_FAT	endosomal part	5	3.70E-02	5.00E-01
	GOTERM_CC_FAT	endosome	11	1.60E-01	8.60E-01
**Annotation Cluster 58**	**Enrichment Score: 1.21**		**Count**	**P_Value**	**Benjamini**
	GOTERM_BP_FAT	positive regulation of protein amino acid phosphorylation	7	1.40E-02	3.30E-01
	GOTERM_BP_FAT	positive regulation of phosphorylation	7	2.10E-02	4.00E-01
	GOTERM_BP_FAT	positive regulation of phosphate metabolic process	7	2.30E-02	4.20E-01
	GOTERM_BP_FAT	positive regulation of phosphorus metabolic process	7	2.30E-02	4.20E-01
	GOTERM_BP_FAT	positive regulation of cellular protein metabolic process	11	3.40E-02	4.50E-01
	GOTERM_BP_FAT	positive regulation of protein metabolic process	11	4.40E-02	4.80E-01
	GOTERM_BP_FAT	positive regulation of protein modification process	9	5.60E-02	5.40E-01
	GOTERM_BP_FAT	regulation of peptidyl-tyrosine phosphorylation	5	6.30E-02	5.70E-01
	GOTERM_BP_FAT	positive regulation of peptidyl-tyrosine phosphorylation	4	8.50E-02	6.40E-01
	GOTERM_BP_FAT	regulation of protein amino acid phosphorylation	8	8.90E-02	6.50E-01
	GOTERM_BP_FAT	regulation of phosphorus metabolic process	16	1.20E-01	7.20E-01
	GOTERM_BP_FAT	regulation of phosphate metabolic process	16	1.20E-01	7.20E-01
	GOTERM_BP_FAT	regulation of phosphorylation	15	1.50E-01	7.70E-01
	GOTERM_BP_FAT	regulation of protein modification process	10	2.10E-01	8.40E-01
	GOTERM_BP_FAT	regulation of cellular protein metabolic process	14	2.60E-01	8.80E-01
**Annotation Cluster 59**	**Enrichment Score: 1.2**		**Count**	**P_Value**	**Benjamini**
	KEGG_PATHWAY	Fc epsilon RI signaling pathway	8	8.00E-03	5.10E-02
	KEGG_PATHWAY	B cell receptor signaling pathway	7	2.30E-02	1.20E-01
	BIOCARTA	Fc Epsilon Receptor I Signaling in Mast Cells	4	1.30E-01	8.60E-01
	KEGG_PATHWAY	VEGF signaling pathway	3	6.60E-01	9.20E-01
**Annotation Cluster 60**	**Enrichment Score: 1.19**		**Count**	**P_Value**	**Benjamini**
	GOTERM_MF_FAT	phosphoinositide phospholipase C activity	4	1.70E-02	4.50E-01
	GOTERM_MF_FAT	phospholipase C activity	4	3.00E-02	4.60E-01
	GOTERM_MF_FAT	lipase activity	6	6.90E-02	6.10E-01
	GOTERM_MF_FAT	peptide receptor activity	6	1.10E-01	7.00E-01
	GOTERM_MF_FAT	peptide receptor activity, G-protein coupled	6	1.10E-01	7.00E-01
	GOTERM_MF_FAT	phospholipase activity	5	1.10E-01	7.00E-01
	GOTERM_MF_FAT	phosphoric diester hydrolase activity	5	1.10E-01	7.10E-01
**Annotation Cluster 61**	**Enrichment Score: 1.09**		**Count**	**P_Value**	**Benjamini**
	INTERPRO	FERM/acyl-CoA-binding protein, 3-helical bundle	4	5.50E-02	6.30E-01
	INTERPRO	FERM domain	4	6.80E-02	6.90E-01
	INTERPRO	FERM central domain	4	6.80E-02	6.90E-01
	UP_SEQ_FEATURE	domain:FERM	4	7.10E-02	9.10E-01
	INTERPRO	Band 4.1 domain	4	7.90E-02	7.10E-01
	INTERPRO	FERM conserved site	4	7.90E-02	7.10E-01
	INTERPRO	Band 4.1 subgroup	3	8.90E-02	7.50E-01
	INTERPRO	FERM, N-terminal	3	1.20E-01	8.40E-01
	SMART	B41	4	1.40E-01	7.30E-01
**Annotation Cluster 62**	**Enrichment Score: 1.08**		**Count**	**P_Value**	**Benjamini**

	GOTERM_BP_FAT	positive regulation of cell motion	7	2.10E-02	4.10E-01
	GOTERM_BP_FAT	regulation of cell motion	10	2.80E-02	4.30E-01
	INTERPRO	Small chemokine, C-X-C, conserved site	3	4.50E-02	5.80E-01
	GOTERM_BP_FAT	positive regulation of cell migration	5	1.30E-01	7.40E-01
	GOTERM_BP_FAT	positive regulation of locomotion	5	1.70E-01	8.10E-01
	GOTERM_BP_FAT	regulation of cell migration	7	1.70E-01	8.10E-01
	GOTERM_BP_FAT	regulation of locomotion	7	2.50E-01	8.80E-01
**Annotation Cluster 63**	**Enrichment Score: 1.06**		**Count**	**P_Value**	**Benjamini**
	GOTERM_BP_FAT	myeloid cell differentiation	8	4.50E-03	1.80E-01
	GOTERM_BP_FAT	homeostasis of number of cells	5	1.80E-01	8.10E-01
	GOTERM_BP_FAT	erythrocyte differentiation	3	2.50E-01	8.80E-01
	GOTERM_BP_FAT	erythrocyte homeostasis	3	3.00E-01	9.10E-01
**Annotation Cluster 64**	**Enrichment Score: 1.02**		**Count**	**P_Value**	**Benjamini**
	UP_SEQ_FEATURE	repeat:IV	3	9.50E-02	9.50E-01
	UP_SEQ_FEATURE	repeat:II	3	9.50E-02	9.50E-01
	UP_SEQ_FEATURE	repeat:III	3	9.50E-02	9.50E-01
	UP_SEQ_FEATURE	repeat:I	3	9.50E-02	9.50E-01
**Annotation Cluster 65**	**Enrichment Score: 1.02**		**Count**	**P_Value**	**Benjamini**
	GOTERM_BP_FAT	regulation of transcription from RNA polymerase II promoter	26	1.90E-02	3.90E-01
	GOTERM_BP_FAT	positive regulation of macromolecule metabolic process	29	2.50E-02	4.20E-01
	GOTERM_BP_FAT	positive regulation of biosynthetic process	24	3.50E-02	4.40E-01
	GOTERM_BP_FAT	positive regulation of specific transcription from RNA polymerase II promoter	5	3.70E-02	4.50E-01
	GOTERM_BP_FAT	positive regulation of gene-specific transcription	6	4.30E-02	4.80E-01
	GOTERM_BP_FAT	positive regulation of macromolecule biosynthetic process	22	5.60E-02	5.40E-01
	GOTERM_BP_FAT	regulation of specific transcription from RNA polymerase II promoter	6	5.70E-02	5.50E-01
	GOTERM_BP_FAT	regulation of gene-specific transcription	7	7.70E-02	6.20E-01
	GOTERM_BP_FAT	positive regulation of cellular biosynthetic process	22	8.20E-02	6.30E-01
	GOTERM_BP_FAT	positive regulation of gene expression	19	9.30E-02	6.60E-01
	GOTERM_BP_FAT	positive regulation of nucleobase, nucleoside, nucleotide and nucleic acid metabolic process	19	1.50E-01	7.70E-01
	GOTERM_BP_FAT	positive regulation of transcription, DNA-dependent	15	1.70E-01	8.00E-01
	GOTERM_BP_FAT	positive regulation of RNA metabolic process	15	1.80E-01	8.20E-01
	GOTERM_BP_FAT	positive regulation of nitrogen compound metabolic process	19	1.80E-01	8.20E-01
	GOTERM_BP_FAT	positive regulation of transcription	17	1.90E-01	8.10E-01
	GOTERM_BP_FAT	negative regulation of specific transcription from RNA polymerase II promoter	3	2.30E-01	8.60E-01
	GOTERM_BP_FAT	negative regulation of gene-specific transcription	3	2.90E-01	9.10E-01
	GOTERM_MF_FAT	transcription activator activity	12	2.90E-01	9.30E-01
	GOTERM_BP_FAT	positive regulation of transcription from RNA polymerase II promoter	11	3.00E-01	9.20E-01
**Annotation Cluster 66**	**Enrichment Score: 1.01**		**Count**	**P_Value**	**Benjamini**
	UP_SEQ_FEATURE	domain:PARP catalytic	3	4.40E-02	8.00E-01
	INTERPRO	Poly(ADP-ribose) polymerase, catalytic region	3	4.50E-02	5.80E-01
	GOTERM_MF_FAT	transferase activity, transferring pentosyl groups	4	8.00E-02	6.30E-01
	GOTERM_MF_FAT	NAD+ ADP-ribosyltransferase activity	3	1.00E-01	7.00E-01
	SP_PIR_KEYWORDS	nad	5	5.20E-01	9.00E-01
**Annotation Cluster 67**	**Enrichment Score: 1.01**		**Count**	**P_Value**	**Benjamini**
	GOTERM_MF_FAT	protein binding, bridging	6	5.60E-02	5.50E-01
	GOTERM_MF_FAT	SH3/SH2 adaptor activity	4	9.20E-02	6.70E-01
	GOTERM_MF_FAT	molecular adaptor activity	4	1.80E-01	8.30E-01
