# Supplementary material for: Intrarenal activation of adaptive immune effectors is associated with tubular damage and impaired renal function in lupus nephritis
Source: Ann Rheum Dis. 2018 Jul 31;77(12):1782–9. doi: 10.1136/annrheumdis-2018-213485 (PMC6241616; doi:10.1136/annrheumdis-2018-213485)
Supplement: Supplementary file 3 [file annrheumdis-2018-213485supp003.pdf]

Supplementary Table 2 : Transcripts and pathways down-regulated in SLE compared to control renal biopsies

Differences in (Log2-transformed, mean-centered) gene expression between lupus and control biopsies were analyzed using a moderated t test with Benjamini-Hochberg correction for multiple comparisons (*p* value threshold set to 0.05). Pathway analyses were performed using DAVID software. Enrichment scores are –log10 *p* values, calculated by modified Fisher Exact test by comparing proportions of transcripts belonging to a given pathway in the tested gene list compared to the whole transcriptome.[14, 15]

Transcripts

Identifier	[Control]	[SLE]	Gene Symbol
ILMN_1751607	2.8195102	0.25910223	FOSB
ILMN_1682763	2.1716797	-0.4070203	ALB
ILMN_1781285	1.7535293	-0.0462692	DUSP1
ILMN_1723522	1.718832	-0.2997489	APOLD1
ILMN_1765232	1.632873	0.14076953	RNLS
ILMN_1662880	1.6177534	-0.1763568	FIS
ILMN_1813361	1.6158535	0.08449265	ANGPTL7
ILMN_2047618	1.6081884	-0.0522185	KCNE1
ILMN_1711015	1.577714	-0.2619377	CRYAA
ILMN_3241522	1.5764322	0.24194288	ASS1
ILMN_1655613	1.530049	-0.5030355	GSTA2
ILMN_1881598	1.51809	-0.475416	AW451118
ILMN_3247082	1.5137397	-0.0834251	FAM150B
ILMN_2089752	1.5075352	-0.1929424	FAM150B
ILMN_1696450	1.4678926	0.06374492	KLK1
ILMN_3275388	1.4390585	0.21589966	ASS1
ILMN_1782939	1.3710135	-0.3634806	ALB
ILMN_2133205	1.3688912	-0.1144226	GPX2
ILMN_1772894	1.3178729	-0.2878681	TMEM27
ILMN_2339955	1.3147991	0.04464598	NR4A2
ILMN_1737096	1.3111312	-0.2938448	CLDN10
ILMN_1705627	1.2944648	-5.71E-04	USP2
ILMN_1739168	1.2869183	-0.1076895	OR2T10
ILMN_2237474	1.2793396	-0.1041819	TBC1D8B
ILMN_2188264	1.274447	-0.1758597	CYR61
ILMN_1767113	1.260937	-0.3245975	AOX1
ILMN_1810233	1.2288419	-0.0995989	UGT2B11
ILMN_2304404	1.2267187	-0.0876096	PBLD
ILMN_3235647	1.2152774	0.0331636	SIK1
ILMN_1714880	1.2068622	-0.0267972	LIMS3L
ILMN_1718152	1.2048471	0.02960346	DEPDC7
ILMN_1781952	1.2007838	-0.2865848	MGST1
ILMN_1811330	1.194678	-0.115554	FAM134B
ILMN_3247998	1.1940734	-0.0809081	STAP1
ILMN_1808114	1.182895	-0.0124336	LYVE1
ILMN_2171396	1.1632164	-0.1204032	MT1H
ILMN_2386973	1.1416459	-0.2422134	PKP2
ILMN_3305273	1.1362882	-0.3057672	PSAT1
ILMN_1769478	1.135193	0.02884816	SPATA24
ILMN_2269256	1.1302254	-0.2344332	DNAJC12
ILMN_1680590	1.1216688	-0.2095973	LOC644662
ILMN_1738268	1.1201845	-0.2776719	IP6K3
ILMN_1687757	1.0949409	-0.1497835	AKR1C4
ILMN_1740233	1.0700529	-0.1506468	UGT1A1
ILMN_1668312	1.0680447	-0.2967684	SLC2A9
ILMN_2189859	1.0647331	-0.1103592	TMEM192
ILMN_1662049	1.0636001	-0.150321	AGPAT5
ILMN_2312194	1.0485913	-0.0375959	CYB5A
ILMN_1811820	1.0399048	0.00878903	SRRM4

Pathways

Annotation Cluster 1	Enrichment Score: 7.43	Database	Pathway	Count	P_Value	Benjamini
		SP_PIR_KEYWORDS	mitochondrion	52	6.80E-20	2.30E-17
		GOTERM_CC_FAT	mitochondrion	60	2.70E-19	7.50E-17
		GOTERM_CC_FAT	mitochondrial part	42	7.40E-17	1.50E-14
		GOTERM_CC_FAT	mitochondrial envelope	34	3.00E-15	2.80E-13
		GOTERM_CC_FAT	mitochondrial inner membrane	28	8.40E-14	5.80E-12
		GOTERM_CC_FAT	mitochondrial membrane	31	1.50E-13	8.60E-12
		UP_SEQ_FEATURE	transit peptide:Mitochondrion	32	3.90E-13	2.80E-10
		GOTERM_CC_FAT	organelle inner membrane	28	4.80E-13	2.20E-11
		SP_PIR_KEYWORDS	transit peptide	32	5.50E-13	9.10E-11
		GOTERM_CC_FAT	organelle envelope	37	1.60E-12	6.30E-11
		GOTERM_CC_FAT	envelope	37	1.70E-12	6.00E-11
		SP_PIR_KEYWORDS	mitochondrion inner membrane	21	2.20E-12	2.40E-10
		GOTERM_BP_FAT	generation of precursor metabolites and energy	26	5.80E-12	8.30E-09
		SP_PIR_KEYWORDS	electron transport	13	1.20E-08	9.50E-07
		GOTERM_BP_FAT	electron transport chain	14	1.30E-08	6.10E-06
		GOTERM_CC_FAT	respiratory chain	12	1.30E-08	4.10E-07
		KEGG_PATHWAY	Parkinson's disease	15	8.30E-08	1.00E-05
		GOTERM_CC_FAT	organelle membrane	41	8.50E-08	2.40E-06
		KEGG_PATHWAY	Oxidative phosphorylation	15	1.00E-07	6.40E-06
		SP_PIR_KEYWORDS	respiratory chain	10	5.00E-07	3.30E-05
		GOTERM_CC_FAT	mitochondrial membrane part	12	2.60E-06	6.60E-05
		KEGG_PATHWAY	Huntington's disease	15	5.50E-06	2.30E-04
		KEGG_PATHWAY	Alzheimer's disease	14	9.40E-06	2.90E-04
		GOTERM_BP_FAT	energy derivation by oxidation of organic compounds	12	9.40E-06	3.40E-03
		GOTERM_BP_FAT	cellular respiration	10	1.30E-05	3.80E-03
		GOTERM_BP_FAT	oxidative phosphorylation	10	1.40E-05	3.40E-03
		GOTERM_BP_FAT	mitochondrial electron transport, NADH to ubiquinone	7	3.30E-05	6.70E-03
		GOTERM_CC_FAT	mitochondrial respiratory chain complex I	7	3.50E-05	8.00E-04
		GOTERM_CC_FAT	NADH dehydrogenase complex	7	3.50E-05	8.00E-04
		GOTERM_CC_FAT	respiratory chain complex I	7	3.50E-05	8.00E-04
		GOTERM_CC_FAT	mitochondrial respiratory chain	8	4.50E-05	9.60E-04
		GOTERM_MF_FAT	NADH dehydrogenase (quinone) activity	7	4.80E-05	1.20E-02
		GOTERM_MF_FAT	NADH dehydrogenase activity	7	4.80E-05	1.20E-02
		GOTERM_MF_FAT	NADH dehydrogenase (ubiquinone) activity	7	4.80E-05	1.20E-02
		GOTERM_MF_FAT	oxidoreductase activity, acting on NADH or NADPH, quinone or similar compound as acceptor	7	1.00E-04	1.70E-02
		GOTERM_BP_FAT	mitochondrial ATP synthesis coupled electron transport	7	1.70E-04	3.00E-02
		GOTERM_BP_FAT	ATP synthesis coupled electron transport	7	1.70E-04	3.00E-02
		GOTERM_MF_FAT	oxidoreductase activity, acting on NADH or NADPH	8	2.90E-04	3.70E-02
		GOTERM_BP_FAT	respiratory electron transport chain	7	3.60E-04	3.90E-02
		SP_PIR_KEYWORDS	ubiquinone	5	7.00E-04	2.30E-02
		GOTERM_BP_FAT	phosphate metabolic process	22	5.20E-02	7.60E-01
		GOTERM_BP_FAT	phosphorus metabolic process	22	5.20E-02	7.60E-01
		GOTERM_BP_FAT	phosphorylation	16	2.10E-01	9.60E-01
	**Annotation Cluster 2**	**Enrichment Score: 6.54**				
		GOTERM_BP_FAT	oxidation reduction	34	2.80E-10	2.00E-07
		GOTERM_MF_FAT	electron carrier activity	15	8.70E-06	4.40E-03
		SP_PIR_KEYWORDS	oxidoreductase	23	9.80E-06	4.60E-04
	**Annotation Cluster 3**	**Enrichment Score: 2.6**				
		GOTERM_BP_FAT	carboxylic acid transport	10	3.30E-04	4.70E-02

ILMN_1782043	1.0332422	-0.1227269	GRIA3
ILMN_3250143	1.0300611	-0.0564306	REPS2
ILMN_1706390	1.0134594	-0.2236356	UGT1A1
ILMN_1689378	1.0084586	-0.2354401	CCRN4L
ILMN_1748983	1.0038464	-0.1547011	RTN4
ILMN_1810053	0.99213433	-0.312822	SLC16A7
ILMN_1876151	0.98127204	-0.3414059	CB305890
ILMN_1811278	0.9807387	-0.2580181	RNF186
ILMN_1755720	0.9684951	-0.184279	SLC2A2
ILMN_1683670	0.96819836	-0.1571321	SLC10A2
ILMN_1701604	0.96207404	-0.0850419	TBC1D8B
ILMN_2053281	0.95962596	-0.0292761	C14orf149
ILMN_2388669	0.95724815	-0.0951897	GRIA3
ILMN_1777499	0.95073074	-0.1377198	AK4
ILMN_1740426	0.9498979	-0.10806	RASD1
ILMN_1724668	0.9484998	0.0501605	REPS2
ILMN_1799569	0.9418677	-0.1631222	LIMS3L
ILMN_1673676	0.9341879	-0.0402413	SNX5
ILMN_3242551	0.92472047	-0.1664283	SDHC
ILMN_1756417	0.91983074	-0.1803922	ANKRD37
ILMN_1798971	0.91112417	-0.2227707	FZD5
ILMN_1772302	0.9103715	-6.72E-04	MTHFS
ILMN_1736704	0.90578675	-0.1651932	DIXDC1
ILMN_1765912	0.90520287	-0.3181076	ACSM2B
ILMN_1754247	0.903439	-0.3227119	SLC3A1
ILMN_1810275	0.9014448	-0.1991627	SLC7A7
ILMN_2358647	0.89975286	-0.1244767	FBXO21
ILMN_1751785	0.8951009	-0.2306716	DMRT2
ILMN_1800898	0.8931896	-0.1257509	ARG2
ILMN_2140799	0.8904562	-0.2353809	FAM24B
ILMN_1663640	0.8857964	-0.1596044	MAOA
ILMN_1713422	0.88561606	-0.0579409	ST18
ILMN_1844919	0.87539464	-0.23078	AK093878
ILMN_1704531	0.8697802	-0.155027	PTGR1
ILMN_1712563	0.8646523	-0.1038424	PSAT1
ILMN_3251346	0.8639838	-0.2412076	NOX4
ILMN_1668134	0.8630255	-0.0979174	GSTM1
ILMN_1701025	0.85867244	-0.1537947	EPHX1
ILMN_1654109	0.85772145	-0.1375875	EGFLAM
ILMN_1708580	0.85520774	-0.3925881	PDZK1IP1
ILMN_1800130	0.8539483	-0.0647397	ETNK2
ILMN_1741971	0.85209924	-0.1104246	KIAA1161
ILMN_2293067	0.84815323	-0.1981659	PM20D1
ILMN_1667374	0.8431695	-0.1874143	C14orf149
ILMN_3263423	0.8425559	-0.0288956	LOC100129027
ILMN_1766918	0.8394562	-0.1835204	KCNK5
ILMN_1866952	0.83362013	0.00594585	BG698340
ILMN_1694075	0.8289164	-0.0277374	GADD45A
ILMN_1791728	0.82780117	-0.0263146	SLC25A25
ILMN_1692861	0.8232512	-0.3941874	UNQ2559
ILMN_2383163	0.81794274	-0.2088923	DEPDC7
ILMN_2381769	0.816575	-0.1165364	ATE1
ILMN_1725773	0.8114041	-0.2485765	DNAJC12
ILMN_2211483	0.8108752	-0.0144548	MAEL
ILMN_2287061	0.8020937	-0.109242	HIF3A
ILMN_1709681	0.8003777	-0.2887038	GABRB3
ILMN_1796560	0.79985785	-0.1001414	PFKFB2

	GOTERM_BP_FAT	organic acid transport	10	3.50E-04	4.10E-02
	GOTERM_BP_FAT	organic anion transport	5	4.00E-03	2.60E-01
	GOTERM_MF_FAT	monocarboxylic acid transmembrane transporter activity	4	5.80E-03	2.40E-01
	GOTERM_BP_FAT	monocarboxylic acid transport	4	3.80E-02	7.00E-01
**Annotation Cluster 4**	**Enrichment Score: 2.04**		**Count**	**P_Value**	**Benjamini**
	GOTERM_BP_FAT	carboxylic acid catabolic process	9	2.40E-04	3.70E-02
	GOTERM_BP_FAT	organic acid catabolic process	9	2.40E-04	3.70E-02
	GOTERM_BP_FAT	amine catabolic process	7	1.00E-03	1.00E-01
	GOTERM_BP_FAT	cellular amino acid catabolic process	6	3.30E-03	2.30E-01
	GOTERM_MF_FAT	cofactor binding	11	5.40E-03	2.40E-01
	GOTERM_BP_FAT	amine biosynthetic process	6	6.90E-03	3.80E-01
	GOTERM_MF_FAT	pyridoxal phosphate binding	5	9.60E-03	3.00E-01
	GOTERM_MF_FAT	vitamin B6 binding	5	9.60E-03	3.00E-01
	SP_PIR_KEYWORDS	pyridoxal phosphate	5	1.00E-02	2.40E-01
	KEGG_PATHWAY	Glycine, serine and threonine metabolism	4	2.00E-02	2.20E-01
	INTERPRO	Pyridoxal phosphate-dependent transferase, major region, subdomain 1	4	2.30E-02	9.60E-01
	GOTERM_BP_FAT	cellular amino acid biosynthetic process	4	3.90E-02	7.10E-01
	GOTERM_MF_FAT	vitamin binding	6	5.10E-02	6.30E-01
	GOTERM_BP_FAT	serine family amino acid metabolic process	3	5.60E-02	7.80E-01
	GOTERM_BP_FAT	carboxylic acid biosynthetic process	6	8.00E-02	8.40E-01
	GOTERM_BP_FAT	organic acid biosynthetic process	6	8.00E-02	8.40E-01
**Annotation Cluster 5**	**Enrichment Score: 1.86**		**Count**	**P_Value**	**Benjamini**
	GOTERM_BP_FAT	carboxylic acid transport	10	3.30E-04	4.70E-02
	GOTERM_BP_FAT	organic acid transport	10	3.50E-04	4.10E-02
	GOTERM_BP_FAT	amino acid transport	6	1.10E-02	4.40E-01
	GOTERM_BP_FAT	amine transport	6	3.10E-02	6.70E-01
	GOTERM_BP_FAT	L-amino acid transport	3	4.50E-02	7.30E-01
	GOTERM_MF_FAT	amino acid transmembrane transporter activity	4	6.30E-02	6.70E-01
	GOTERM_MF_FAT	amine transmembrane transporter activity	4	1.10E-01	7.90E-01
	GOTERM_MF_FAT	L-amino acid transmembrane transporter activity	3	1.20E-01	8.10E-01
**Annotation Cluster 6**	**Enrichment Score: 1.82**		**Count**	**P_Value**	**Benjamini**
	GOTERM_MF_FAT	hydrogen ion transmembrane transporter activity	8	4.80E-04	4.80E-02
	GOTERM_MF_FAT	monovalent inorganic cation transmembrane transporter activity	8	1.10E-03	8.00E-02
	GOTERM_MF_FAT	cytochrome-c oxidase activity	3	6.90E-02	6.80E-01
	GOTERM_MF_FAT	oxidoreductase activity, acting on heme group of donors, oxygen as acceptor	3	6.90E-02	6.80E-01
	GOTERM_MF_FAT	oxidoreductase activity, acting on heme group of donors	3	6.90E-02	6.80E-01
	GOTERM_MF_FAT	heme-copper terminal oxidase activity	3	6.90E-02	6.80E-01
**Annotation Cluster 7**	**Enrichment Score: 1.79**		**Count**	**P_Value**	**Benjamini**
	GOTERM_BP_FAT	coenzyme metabolic process	9	2.00E-03	1.60E-01
	GOTERM_BP_FAT	cofactor metabolic process	10	2.50E-03	1.90E-01
	GOTERM_BP_FAT	coenzyme biosynthetic process	4	8.20E-02	8.40E-01
	GOTERM_BP_FAT	cofactor biosynthetic process	4	1.70E-01	9.50E-01
**Annotation Cluster 8**	**Enrichment Score: 1.77**		**Count**	**P_Value**	**Benjamini**
	GOTERM_BP_FAT	protein complex biogenesis	18	1.40E-03	1.20E-01
	GOTERM_BP_FAT	protein complex assembly	18	1.40E-03	1.20E-01
	GOTERM_BP_FAT	macromolecular complex assembly	18	2.00E-02	5.70E-01
	GOTERM_BP_FAT	macromolecular complex subunit organization	18	3.40E-02	6.90E-01
	GOTERM_BP_FAT	protein oligomerization	7	4.50E-02	7.40E-01
	GOTERM_BP_FAT	cellular macromolecular complex subunit organization	7	4.30E-01	9.90E-01
**Annotation Cluster 9**	**Enrichment Score: 1.76**		**Count**	**P_Value**	**Benjamini**
	GOTERM_CC_FAT	mitochondrial intermembrane space	5	2.00E-03	3.60E-02
	GOTERM_CC_FAT	organelle envelope lumen	5	3.50E-03	5.90E-02
	SP_PIR_KEYWORDS	Apoptosis	5	7.70E-01	1.00E+00
**Annotation Cluster 10**	**Enrichment Score: 1.74**		**Count**	**P_Value**	**Benjamini**
	INTERPRO	UDP-glucuronosyl/UDP-glucosyltransferase	4	8.30E-04	3.70E-01
	PIR_SUPERFAMILY	PIRSF005678:glucuronosyltransferase	4	1.00E-03	1.40E-01
	KEGG_PATHWAY	Pentose and glucuronate interconversions	4	4.30E-03	7.50E-02

ILMN_2311020	0.7967522	-0.231525	DNAJC12
ILMN_1760727	0.796078	-0.1365215	ANG
ILMN_1672191	0.7951682	-0.1318213	ATP5F1
ILMN_1666280	0.793285	-0.1056904	COX11
ILMN_1778357	0.78927904	-0.1400197	DNMT3L
ILMN_2266334	0.7832703	-0.1595441	BCAP29
ILMN_1738491	0.7827796	-0.1136861	SNX30
ILMN_1654385	0.78150266	-0.1364169	ASB13
ILMN_1756723	0.78099924	-0.15574	DPP7
ILMN_1729514	0.7792935	-0.302168	CMTM4
ILMN_1672443	0.77397776	-0.0800685	QDPR
ILMN_2338038	0.7729864	-0.4243262	AK4
ILMN_2231911	0.7704615	0.01107736	AUH
ILMN_1777521	0.7697696	-0.0761192	GLUD2
ILMN_1665510	0.7655499	-0.0504299	ERRFI1
ILMN_1682935	0.7580247	-0.0786566	LYPLAL1
ILMN_1803073	0.7517055	-0.1846666	DNAJC12
ILMN_1806754	0.74729544	-0.0430816	GLDC
ILMN_1693210	0.7441589	-0.0216614	NSMCE2
ILMN_1781386	0.74344814	-0.0522447	WIPI1
ILMN_1762255	0.73785657	-0.0403223	GSTM1
ILMN_1674049	0.73529196	-0.27112	TEAD1
ILMN_2274923	0.73335314	-0.2149434	MOCS2
ILMN_1753502	0.73177016	-0.3400245	IGSF11
ILMN_1713124	0.73093295	-0.2341701	AKR1C3
ILMN_1784661	0.7306732	-0.099162	TMEM2
ILMN_1692535	0.7300153	-0.2228338	DPP4
ILMN_2133038	0.7248892	-0.1838501	UGT3A1
ILMN_1685678	0.7192287	-0.2980072	EEF1B2
ILMN_2184556	0.71878004	-0.4122432	SLC4A4
ILMN_1704139	0.7152482	-0.065184	DHRSX
ILMN_1733956	0.7119044	-0.0826878	IARS
ILMN_1734897	0.71177924	-0.3864933	SLC4A4
ILMN_2408572	0.7102041	-0.0850099	ANG
ILMN_1678678	0.7098415	-0.0032822	SLC37A4
ILMN_1662331	0.70453346	-0.0670416	PDSS2
ILMN_2098437	0.70245284	-0.1573015	ST13
ILMN_3248403	0.70005864	-0.0514489	SDHC
ILMN_1750158	0.6932785	-0.1909055	ACOX1
ILMN_2367239	0.6911485	-0.0624428	RCAN1
ILMN_1689869	0.68983126	-0.1554704	GPR89A
ILMN_1658917	0.6896757	-0.249306	SLC1A1
ILMN_1847793	0.6842585	-0.0584133	CA449405
ILMN_1701269	0.68261766	-0.010596	ATP5C1
ILMN_1652155	0.67902696	-0.0658027	DHX9
ILMN_1703894	0.6754489	-0.0718316	SLX1A
ILMN_2373791	0.67069197	-0.0848189	ENPP2
ILMN_1662795	0.66952646	-0.1549185	CA2
ILMN_2369924	0.6683334	-0.0939546	NDUFB6
ILMN_2113957	0.6657509	-0.1576956	PEX13
ILMN_3211707	0.6648283	-0.1255081	LOC100306975
ILMN_1749405	0.6613723	-0.0259727	KIAA1191
ILMN_3300198	0.65759516	-0.2543633	MMP24
ILMN_3183789	0.65351874	-0.0820105	UQCRFS1
ILMN_2171295	0.65084594	-0.1587324	CDK14
ILMN_1764090	0.6498643	-0.1883149	AK4

	GOTERM_MF_FAT	glucuronosyltransferase activity	4	4.50E-03	2.50E-01
	KEGG_PATHWAY	Starch and sucrose metabolism	5	7.70E-03	1.10E-01
	KEGG_PATHWAY	Steroid hormone biosynthesis	5	1.10E-02	1.40E-01
	KEGG_PATHWAY	Porphyrin and chlorophyll metabolism	4	2.40E-02	2.20E-01
	KEGG_PATHWAY	Androgen and estrogen metabolism	4	3.20E-02	2.40E-01
	KEGG_PATHWAY	Ascorbate and aldarate metabolism	3	4.00E-02	2.70E-01
	KEGG_PATHWAY	Drug metabolism	3	1.90E-01	7.20E-01
	KEGG_PATHWAY	Retinol metabolism	3	2.70E-01	8.20E-01
	SP_PIR_KEYWORDS	glycosyltransferase	4	5.60E-01	9.90E-01
**Annotation Cluster 11**	**Enrichment Score: 1.66**		**Count**	**P_Value**	**Benjamini**
	GOTERM_BP_FAT	glucose transport	4	8.80E-03	4.20E-01
	GOTERM_BP_FAT	hexose transport	4	9.60E-03	4.30E-01
	GOTERM_BP_FAT	monosaccharide transport	4	1.10E-02	4.40E-01
	SP_PIR_KEYWORDS	sugar transport	4	1.40E-02	3.00E-01
	GOTERM_MF_FAT	hexose transmembrane transporter activity	3	3.10E-02	5.00E-01
	GOTERM_MF_FAT	monosaccharide transmembrane transporter activity	3	3.40E-02	5.20E-01
	GOTERM_MF_FAT	sugar transmembrane transporter activity	3	6.10E-02	6.70E-01
	GOTERM_BP_FAT	carbohydrate transport	4	6.10E-02	7.80E-01
**Annotation Cluster 12**	**Enrichment Score: 1.64**		**Count**	**P_Value**	**Benjamini**
	UP_SEQ_FEATURE	nucleotide phosphate-binding region:NADP	5	1.70E-02	7.90E-01
	SP_PIR_KEYWORDS	nadp	7	2.00E-02	3.40E-01
	GOTERM_MF_FAT	oxidoreductase activity, acting on the CH-CH group of donors, NAD or NADP as acceptor	3	3.40E-02	5.20E-01
**Annotation Cluster 13**	**Enrichment Score: 1.59**		**Count**	**P_Value**	**Benjamini**
	GOTERM_MF_FAT	transferase activity, transferring alkyl or aryl (other than methyl) groups	5	6.90E-03	2.50E-01
	GOTERM_MF_FAT	glutathione transferase activity	3	3.80E-02	5.40E-01
	KEGG_PATHWAY	Glutathione metabolism	4	6.80E-02	4.00E-01
**Annotation Cluster 14**	**Enrichment Score: 1.5**		**Count**	**P_Value**	**Benjamini**
	GOTERM_MF_FAT	amino acid binding	5	1.40E-02	3.80E-01
	GOTERM_MF_FAT	carboxylic acid binding	7	2.50E-02	4.50E-01
	GOTERM_MF_FAT	amine binding	5	9.10E-02	7.60E-01
**Annotation Cluster 15**	**Enrichment Score: 1.48**		**Count**	**P_Value**	**Benjamini**
	GOTERM_BP_FAT	fatty acid metabolic process	8	2.80E-02	6.50E-01
	GOTERM_BP_FAT	icosanoid metabolic process	4	3.20E-02	6.70E-01
	GOTERM_BP_FAT	unsaturated fatty acid metabolic process	4	3.90E-02	7.10E-01
**Annotation Cluster 16**	**Enrichment Score: 1.47**		**Count**	**P_Value**	**Benjamini**
	GOTERM_MF_FAT	symporter activity	8	5.40E-03	2.70E-01
	GOTERM_MF_FAT	solute:cation symporter activity	5	6.00E-02	6.80E-01
	GOTERM_BP_FAT	anion transport	6	6.10E-02	7.80E-01
	SP_PIR_KEYWORDS	Symport	5	6.50E-02	5.50E-01
**Annotation Cluster 17**	**Enrichment Score: 1.43**		**Count**	**P_Value**	**Benjamini**
	GOTERM_BP_FAT	mitochondrion organization	8	4.40E-03	2.70E-01
	GOTERM_BP_FAT	NADH dehydrogenase complex assembly	3	9.10E-03	4.20E-01
	GOTERM_BP_FAT	mitochondrial respiratory chain complex I assembly	3	9.10E-03	4.20E-01
	GOTERM_BP_FAT	mitochondrial respiratory chain complex assembly	3	1.30E-02	4.70E-01
	GOTERM_BP_FAT	cellular protein complex assembly	6	9.20E-02	8.60E-01
	GOTERM_BP_FAT	cellular macromolecular complex subunit organization	7	4.30E-01	9.90E-01
	GOTERM_BP_FAT	cellular macromolecular complex assembly	6	5.10E-01	1.00E+00
**Annotation Cluster 18**	**Enrichment Score: 1.38**		**Count**	**P_Value**	**Benjamini**
	KEGG_PATHWAY	Metabolism of xenobiotics by cytochrome P450	9	1.40E-05	3.40E-04
	SP_PIR_KEYWORDS	microsome	5	5.70E-02	5.30E-01
	SP_PIR_KEYWORDS	endoplasmic reticulum	14	1.80E-01	8.40E-01
	GOTERM_CC_FAT	microsome	6	2.80E-01	8.50E-01
	GOTERM_CC_FAT	vesicular fraction	6	3.00E-01	8.60E-01
	GOTERM_CC_FAT	endoplasmic reticulum	16	4.70E-01	9.40E-01
**Annotation Cluster 19**	**Enrichment Score: 1.37**		**Count**	**P_Value**	**Benjamini**
	GOTERM_CC_FAT	mitochondrial matrix	13	1.60E-04	3.10E-03

ILMN_1667670	0.6483701	-0.1738529	SLC25A15
ILMN_1659524	0.6456087	-0.0406404	NDUFAF4
ILMN_1717357	0.6431675	-0.0651603	AIFM1
ILMN_1764629	0.6428138	-0.089202	SLC39A14
ILMN_1754421	0.637601	-0.2653099	NDUFAF1
ILMN_1692651	0.6365469	-0.1482794	PHB
ILMN_1686985	0.6347615	-0.0879968	MTM1
ILMN_1761961	0.6305251	-0.0809065	CCDC90A
ILMN_1729216	0.630064	-0.2543142	CRYAB
ILMN_1680314	0.6282971	-0.1115901	TXN
ILMN_1797974	0.62468326	-0.058843	AIG1
ILMN_1733374	0.62257844	-0.1551499	FAM82B
ILMN_1682601	0.6207918	-0.0724094	HBXIP
ILMN_3285959	0.6203924	-0.1615806	ST13
ILMN_1689162	0.6197751	-0.1552239	ACTR8
ILMN_1687359	0.6122366	-0.129858	MRPS23
ILMN_1678323	0.6101367	-0.0616936	AASS
ILMN_2389151	0.60771495	-0.0218537	UGP2
ILMN_3236481	0.606375	-0.1919171	HIGD1A
ILMN_1669633	0.60514337	-0.1672284	ACP1
ILMN_1753026	0.6002035	-0.3630205	LPPR1
ILMN_1758938	0.59658355	-0.1210987	SLC31A2
ILMN_2314417	0.59574836	-0.1271723	HYAL1
ILMN_1663220	0.59540725	-0.0697363	MRPL22
ILMN_2151281	0.59334505	-0.0981981	GABARAPL1
ILMN_3237721	0.5926957	-0.1983706	TMCO7
ILMN_1743361	0.58877814	-0.0571174	PTGER3
ILMN_2390416	0.58876026	-0.0907461	BAG6
ILMN_1746241	0.588495	-0.1362032	SDHC
ILMN_2213297	0.5849724	-0.3041122	C11orf54
ILMN_3254984	0.5827307	-0.1347452	MKKS
ILMN_1708341	0.58206224	-0.097523	PDZK1
ILMN_1912737	0.5772733	-0.1418957	HIPK2
ILMN_1779616	0.5770341	-0.1302274	SUCLG1
ILMN_1764795	0.57696605	-0.2079817	FMN2
ILMN_1764087	0.5764295	-0.1342754	SYPL1
ILMN_3251155	0.5761449	-0.1355765	PCBP2
ILMN_1659885	0.57567596	-0.1864566	ACSS3
ILMN_1813389	0.57451254	-0.0281603	MRPS7
ILMN_1681670	0.5726577	-0.1242306	SLC25A4
ILMN_1748661	0.5707083	-0.2201065	AKT1
ILMN_2050790	0.5694572	-0.1080938	HSPB2-C11orf52
ILMN_2038776	0.5688461	-0.0949912	TXN
ILMN_2108590	0.56566083	-0.1029146	THSD7B
ILMN_1917290	0.5637789	-0.2370254	LAMP2
ILMN_1659753	0.5637259	-0.0775361	LAMP2
ILMN_1708672	0.5602941	-0.1115319	ACAT2
ILMN_3239568	0.55898	-0.1504812	MYLK4
ILMN_2395974	0.5571463	-0.1129833	PRDX3
ILMN_1668408	0.5559756	-0.1807759	AIFM1
ILMN_1776104	0.5537595	-0.1644785	NDUFS5
ILMN_1672004	0.5492055	-0.3571072	TOB1
ILMN_1729320	0.54754436	-0.1312527	ALOX15
ILMN_1760575	0.5447637	-0.1687263	PTP4A1
ILMN_1781374	0.54243946	0.00726491	TUFT1
ILMN_1716053	0.5413481	-0.0155876	AK2
ILMN_2211263	0.54009885	-0.1265631	RFK

	GOTERM_CC_FAT	mitochondrial lumen	13	1.60E-04	3.10E-03
	GOTERM_CC_FAT	membrane-enclosed lumen	29	5.10E-01	9.60E-01
	GOTERM_CC_FAT	organelle lumen	26	7.10E-01	9.80E-01
	GOTERM_CC_FAT	intracellular organelle lumen	25	7.40E-01	9.90E-01
	GOTERM_CC_FAT	nuclear lumen	11	1.00E+00	1.00E+00
**Annotation Cluster 20**	**Enrichment Score: 1.33**		**Count**	**P_Value**	**Benjamini**
	UP_SEQ_FEATURE	repeat:Solcar 3	4	3.30E-02	9.30E-01
	UP_SEQ_FEATURE	repeat:Solcar 2	4	3.80E-02	9.40E-01
	UP_SEQ_FEATURE	repeat:Solcar 1	4	3.80E-02	9.40E-01
	INTERPRO	Mitochondrial substrate carrier	4	4.20E-02	9.80E-01
	INTERPRO	Mitochondrial substrate/solute carrier	4	5.10E-02	9.80E-01
	INTERPRO	Adenine nucleotide translocator 1	3	5.70E-02	9.80E-01
	PIR_SUPERFAMILY	PIRSF002458:ADP,ATP carrier protein	3	8.30E-02	1.00E+00
**Annotation Cluster 21**	**Enrichment Score: 1.28**		**Count**	**P_Value**	**Benjamini**
	INTERPRO	Thioredoxin fold	7	4.00E-03	6.70E-01
	INTERPRO	Thioredoxin-like	4	1.60E-02	9.50E-01
	INTERPRO	Thioredoxin, conserved site	3	5.70E-02	9.80E-01
	GOTERM_BP_FAT	cell redox homeostasis	4	6.60E-02	8.00E-01
	UP_SEQ_FEATURE	domain:Thioredoxin	3	7.50E-02	9.90E-01
	SP_PIR_KEYWORDS	Redox-active center	3	1.10E-01	7.30E-01
	GOTERM_BP_FAT	cellular homeostasis	8	5.40E-01	1.00E+00
**Annotation Cluster 22**	**Enrichment Score: 1.24**		**Count**	**P_Value**	**Benjamini**
	GOTERM_BP_FAT	glucose metabolic process	8	7.70E-03	4.00E-01
	GOTERM_BP_FAT	carbohydrate catabolic process	6	2.30E-02	6.00E-01
	GOTERM_BP_FAT	pyruvate metabolic process	4	2.40E-02	6.10E-01
	GOTERM_BP_FAT	hexose metabolic process	8	2.40E-02	6.00E-01
	GOTERM_BP_FAT	alcohol catabolic process	5	3.20E-02	6.80E-01
	GOTERM_BP_FAT	monosaccharide metabolic process	8	4.70E-02	7.40E-01
	GOTERM_BP_FAT	carbohydrate biosynthetic process	5	7.40E-02	8.20E-01
	GOTERM_BP_FAT	cellular carbohydrate catabolic process	4	1.30E-01	9.20E-01
	GOTERM_BP_FAT	glucose catabolic process	3	2.10E-01	9.60E-01
	GOTERM_BP_FAT	hexose catabolic process	3	2.70E-01	9.80E-01
	GOTERM_BP_FAT	monosaccharide catabolic process	3	2.80E-01	9.80E-01
**Annotation Cluster 23**	**Enrichment Score: 1.2**		**Count**	**P_Value**	**Benjamini**
	GOTERM_CC_FAT	mitochondrial outer membrane	5	4.60E-02	4.20E-01
	GOTERM_CC_FAT	organelle outer membrane	5	7.00E-02	5.30E-01
	GOTERM_CC_FAT	outer membrane	5	7.80E-02	5.30E-01
**Annotation Cluster 24**	**Enrichment Score: 1.19**		**Count**	**P_Value**	**Benjamini**
	GOTERM_CC_FAT	organellar ribosome	5	5.60E-03	8.80E-02
	GOTERM_CC_FAT	mitochondrial ribosome	5	5.60E-03	8.80E-02
	SP_PIR_KEYWORDS	ribosomal protein	8	1.50E-02	3.00E-01
	SP_PIR_KEYWORDS	ribonucleoprotein	9	3.80E-02	4.40E-01
	GOTERM_CC_FAT	ribosome	8	4.30E-02	4.40E-01
	GOTERM_CC_FAT	ribosomal subunit	6	4.30E-02	4.30E-01
	GOTERM_BP_FAT	translation	9	1.20E-01	9.00E-01
	GOTERM_MF_FAT	structural constituent of ribosome	6	1.20E-01	8.00E-01
	GOTERM_CC_FAT	ribonucleoprotein complex	11	2.40E-01	8.30E-01
	GOTERM_CC_FAT	small ribosomal subunit	3	2.40E-01	8.20E-01
	GOTERM_CC_FAT	large ribosomal subunit	3	2.60E-01	8.40E-01
	GOTERM_MF_FAT	structural molecule activity	10	6.50E-01	1.00E+00
**Annotation Cluster 25**	**Enrichment Score: 1.18**		**Count**	**P_Value**	**Benjamini**
	GOTERM_CC_FAT	vacuole	10	1.40E-02	1.90E-01
	GOTERM_CC_FAT	lytic vacuole	7	9.70E-02	6.00E-01
	GOTERM_CC_FAT	lysosome	7	9.70E-02	6.00E-01
	SP_PIR_KEYWORDS	lysosome	5	1.50E-01	8.10E-01
**Annotation Cluster 26**	**Enrichment Score: 1.13**		**Count**	**P_Value**	**Benjamini**
	GOTERM_BP_FAT	lipid oxidation	4	2.00E-02	5.80E-01

ILMN_3229324	0.53662777	-0.1225109	SGK1
ILMN_3275696	0.53566384	-0.1055134	SNX5
ILMN_1673026	0.53407526	-0.1165511	CHCHD3
ILMN_1804656	0.5337118	-0.1619804	C12orf62
ILMN_1730416	0.5325369	-0.138679	CYCS
ILMN_1673535	0.5323554	-0.1959483	MYLK4
ILMN_1789535	0.53226197	-0.1648442	DHDDS
ILMN_1662316	0.5316055	-0.2208822	VPS33A
ILMN_1662306	0.5277954	-0.0726951	RABL3
ILMN_1681890	0.52750564	-0.0092852	DYNLT3
ILMN_1812312	0.5266838	-0.0266107	NDUFS4
ILMN_1786388	0.524405	-0.0741832	RNF113A
ILMN_1800659	0.5241793	-0.0158091	PGM1
ILMN_2233401	0.51928926	-0.1372421	FTHL3P
ILMN_1780799	0.5191908	-0.282548	ENPP2
ILMN_1669851	0.5184017	-0.1055392	STAG3L4
ILMN_1779751	0.51746875	-0.0576643	C7orf55
ILMN_2395969	0.51736766	-0.0928268	PRDX3
ILMN_1838313	0.5166446	-0.0301759	LRRC8B
ILMN_2187718	0.51658463	-0.2029427	COX17
ILMN_2313821	0.51613426	-0.1150754	AIFM1
ILMN_1674344	0.51510006	-0.1477752	PARD6G
ILMN_1729775	0.5132178	-0.0708344	OPA1
ILMN_1673282	0.51235974	-0.2129595	LAMP2
ILMN_2405642	0.51151264	-0.1576971	DHDDS
ILMN_1810604	0.50765324	-0.2420962	ELMOD1
ILMN_1699728	0.5057842	-0.1541611	BTD
ILMN_1804673	0.50361586	-0.1682482	SLC16A4
ILMN_1749641	0.5027475	-0.1186625	FBXO3
ILMN_1690386	0.5009279	-0.1851168	CSRP2BP
ILMN_1742887	0.4983462	-0.1662009	HBXIP
ILMN_1778523	0.4969263	-0.188863	KLF9
ILMN_3303673	0.49638113	-0.0317633	LOC729852
ILMN_1701749	0.4942459	-0.214608	UQCRFS1
ILMN_2391231	0.49253243	-0.1014882	SORD
ILMN_2366246	0.4909737	-0.0665305	SEC23B
ILMN_1663840	0.4890622	-0.1559719	OR2T29
ILMN_3244070	0.4888593	-0.0898611	URG7
ILMN_1728132	0.4878155	-0.1961908	LDHB
ILMN_1723846	0.4874211	-0.1976832	METTL21B
ILMN_1714401	0.4864093	-0.035512	SNX27
ILMN_2195665	0.48557532	-0.0631011	PANK3
ILMN_1771805	0.48275137	-0.0504277	ELK4
ILMN_2363273	0.47807437	-0.0603694	ZNF226
ILMN_1666471	0.4752401	-0.2127193	UQCRQ
ILMN_1793474	0.47494924	-0.1218603	INSIG1
ILMN_2189993	0.47404963	-0.0843789	MRPS35
ILMN_1788738	0.4736591	-0.1682116	ZNRF3
ILMN_1789283	0.47337997	-0.0368182	PPP2R5C
ILMN_1722698	0.46818358	-0.2791058	RCHY1
ILMN_2081335	0.46583247	-0.0924524	C7orf44
ILMN_1782754	0.45977533	-0.1490223	PAGE4
ILMN_1793966	0.4575815	-0.1328245	BOLA1
ILMN_1744059	0.45672715	-0.0715276	DCTN6
ILMN_2274180	0.4565025	-0.1387646	MKKS
ILMN_1696911	0.45303917	-0.1904686	FTH1
ILMN_1692707	0.44214332	-0.1433443	C2orf79

	GOTERM_BP_FAT	fatty acid oxidation	4	2.00E-02	5.80E-01
	GOTERM_BP_FAT	fatty acid metabolic process	8	2.80E-02	6.50E-01
	GOTERM_BP_FAT	lipid modification	4	8.20E-02	8.40E-01
	GOTERM_BP_FAT	fatty acid catabolic process	3	9.90E-02	8.70E-01
	GOTERM_BP_FAT	cellular lipid catabolic process	3	3.10E-01	9.90E-01
	GOTERM_BP_FAT	lipid catabolic process	4	4.70E-01	1.00E+00
**Annotation Cluster 27**	**Enrichment Score: 1.07**		**Count**	**P_Value**	**Benjamini**
	GOTERM_BP_FAT	response to endogenous stimulus	13	1.70E-02	5.40E-01
	GOTERM_BP_FAT	response to organic substance	18	3.90E-02	7.10E-01
	GOTERM_BP_FAT	response to hormone stimulus	10	9.50E-02	8.70E-01
	GOTERM_BP_FAT	response to steroid hormone stimulus	6	1.60E-01	9.40E-01
	GOTERM_BP_FAT	response to abiotic stimulus	9	1.80E-01	9.50E-01
	GOTERM_BP_FAT	response to estrogen stimulus	4	2.00E-01	9.60E-01
**Annotation Cluster 28**	**Enrichment Score: 1.03**		**Count**	**P_Value**	**Benjamini**
	GOTERM_BP_FAT	carbohydrate biosynthetic process	5	7.40E-02	8.20E-01
	GOTERM_BP_FAT	cellular carbohydrate biosynthetic process	4	8.20E-02	8.40E-01
	GOTERM_BP_FAT	alcohol biosynthetic process	3	1.40E-01	9.20E-01
**Annotation Cluster 29**	**Enrichment Score: 1.02**		**Count**	**P_Value**	**Benjamini**
	GOTERM_MF_FAT	phosphoprotein phosphatase activity	8	1.40E-02	3.70E-01
	GOTERM_MF_FAT	phosphatase activity	10	1.60E-02	3.80E-01
	GOTERM_MF_FAT	protein tyrosine phosphatase activity	6	2.30E-02	4.40E-01
	SP_PIR_KEYWORDS	protein phosphatase	6	3.30E-02	4.20E-01
	GOTERM_BP_FAT	protein amino acid dephosphorylation	5	1.30E-01	9.20E-01
	GOTERM_BP_FAT	dephosphorylation	5	1.90E-01	9.50E-01
	UP_SEQ_FEATURE	active site:Phosphocysteine intermediate	3	3.20E-01	1.00E+00
	INTERPRO	Protein-tyrosine phosphatase	3	3.40E-01	1.00E+00
	INTERPRO	Dual-specific/protein-tyrosine phosphatase, conserved region	3	3.50E-01	1.00E+00
	INTERPRO	Protein-tyrosine phosphatase, active site	3	3.80E-01	1.00E+00
**Annotation Cluster 30**	**Enrichment Score: 1**		**Count**	**P_Value**	**Benjamini**
	GOTERM_CC_FAT	cell fraction	24	5.40E-02	4.50E-01
	GOTERM_CC_FAT	insoluble fraction	18	1.20E-01	6.70E-01
	GOTERM_CC_FAT	membrane fraction	17	1.50E-01	6.90E-01

ILMN_2173835	0.4409039	-0.1652497	FTHL3P
ILMN_1722662	0.43740088	-0.2066791	RAD23B
ILMN_2380754	0.43717372	-0.0433467	CCNG1
ILMN_1661945	0.4368875	-0.0505693	C14orf156
ILMN_1811933	0.4361068	-0.3302737	SHMT1
ILMN_2356031	0.43607378	-0.1596443	RNF121
ILMN_1765644	0.4335481	-0.1304037	COMMD8
ILMN_1761850	0.43353033	-0.1006181	ATRN
ILMN_2083567	0.4328398	-0.1518936	PHLPP2
ILMN_1665887	0.42973006	-0.1064787	WDR61
ILMN_1778631	0.42883274	-0.0766349	DEFB136
ILMN_1771620	0.42253804	-0.0876486	SNRPB2
ILMN_1662263	0.42182165	-0.1539048	MDP1
ILMN_2341952	0.41888434	-0.0459762	MRPL35
ILMN_1742705	0.41700476	-0.1381625	SLC39A11
ILMN_1726769	0.41590095	-0.1434764	CNDP2
ILMN_1720838	0.4097488	-0.063075	DECR1
ILMN_1815319	0.40853834	-0.1554309	CMTM4
ILMN_1701331	0.40701497	-0.0424514	UBE2M
ILMN_1744432	0.40570152	-0.0886855	COX8C
ILMN_1737862	0.4046042	-0.2743336	PSMB4
ILMN_1730391	0.40252173	-0.2537092	MRPS18A
ILMN_1766762	0.40246844	-0.128025	DYNLRB1
ILMN_3297880	0.3999284	-0.0981417	SEC13
ILMN_2294976	0.39817518	-0.0649271	ANG
ILMN_1799367	0.3968433	-0.0441484	TMX2
ILMN_1777644	0.3948608	-0.1542635	INPP5J
ILMN_2097421	0.39421952	-0.0463418	MRPL51
ILMN_2225537	0.39069605	-0.1320155	PTGR1
ILMN_2332713	0.3895538	-0.1557675	SLC25A3
ILMN_2081863	0.3872285	-0.114128	NDUFB4
ILMN_1756355	0.3868723	-0.131216	NDUFS3
ILMN_1805536	0.3865556	-0.1555171	LOC649395
ILMN_1741572	0.38331878	-0.055803	AKAP8
ILMN_1669818	0.38285017	-0.131959	ESD
ILMN_1759729	0.38075674	-0.1003085	NDUFA8
ILMN_1891857	0.37954158	-0.1100024	RAB2A
ILMN_1696974	0.37809336	-0.1079748	ANG
ILMN_2157510	0.37748522	-0.1191362	RDH5
ILMN_2096012	0.37461853	-0.213255	UHMK1
ILMN_2133713	0.36997807	-0.0800922	FAM76A
ILMN_1666004	0.36997592	-0.0599306	WASL
ILMN_1742250	0.36988783	-0.1800445	CCNH
ILMN_1721989	0.36958754	-0.066355	ATP5F1
ILMN_1737738	0.363845	-0.1494603	NDUFA12
ILMN_1770412	0.35878038	-0.1046594	AHCYL1
ILMN_2096322	0.35643977	-0.0885951	ADIPOR1
ILMN_1811327	0.32078636	-0.1003164	MRPL27
ILMN_1796235	0.32004058	-0.2009104	CIRH1A
ILMN_2044927	0.31497407	-0.1327809	RNF5
ILMN_1790973	0.30834568	-0.0419093	CDS2
ILMN_1764494	0.26841974	-0.1058139	ATP5A1
ILMN_1656145	0.24360895	-0.1872449	GOT1
