# Supplementary material for: Intrarenal activation of adaptive immune effectors is associated with tubular damage and impaired renal function in lupus nephritis
Source: Ann Rheum Dis. 2018 Jul 31;77(12):1782–9. doi: 10.1136/annrheumdis-2018-213485 (PMC6241616; doi:10.1136/annrheumdis-2018-213485)
Supplement: Supplementary file 6 [file annrheumdis-2018-213485supp006.pdf]

Supplementary Table 3 : Transcriptomic profiling singles out a subgroup of SLE kidney biopsy samples with decreased eGFR

A cluster of SLE kidney biopsy samples, identified by hierarchical clustering algorithm (Pearson-centered algorithm, Ward's linkage rule) is characterized by the overexpression of 271 transcripts (by moderated t test, with Bonferroni correction for multiple comparisons) compared to the other lupus samples, significantly enriched in the indicated pathways.

Transcripts

Identifier	[Control]	[SLE]	Gene Symbol
ILMN_1654319	-0.3232504	1.9405183	HAPLN3
ILMN_1738116	-0.2354067	1.9201676	TMEM119
ILMN_1804601	-0.2589666	1.8242207	DKFZp686O16217
ILMN_1659800	-0.2759814	1.7896557	BCL11A
ILMN_1718936	-0.2632236	1.6821613	LST1
ILMN_1704286	-0.3620017	1.6446545	FXYD5
ILMN_2178226	-0.2631313	1.5874728	KRT86
ILMN_2316236	-0.3898594	1.5715406	HOPX
ILMN_1685403	-0.2286288	1.5471464	MMP7
ILMN_1701424	-0.4379956	1.504757	LAMC2
ILMN_1778977	-0.2593867	1.5005594	TYROBP
ILMN_3307693	-0.9245362	1.479397	WFDC2
ILMN_2325837	-0.4301998	1.4455804	CD3D
ILMN_1770673	-0.2988024	1.427377	AKNA
ILMN_1886655	-0.5120298	1.4127944	TCRVB
ILMN_1715760	-0.2148992	1.4113554	HUAT
ILMN_1727284	-0.1995767	1.3429725	CD4
ILMN_2280548	-0.3113598	1.32024	FYB
ILMN_1769129	-1.2588445	1.3199118	CCL19
ILMN_2254635	-0.901885	1.3150588	ITGAX
ILMN_1659688	-0.3078076	1.3150438	LGALS3BP
ILMN_1731233	-0.3580598	1.314427	GZMH
ILMN_1772359	-0.3015788	1.306691	LAPTM5
ILMN_1777190	-0.212863	1.2945279	CFD
ILMN_2320888	-0.2540839	1.2926085	CXCR4
ILMN_1740015	-0.2876653	1.27568	CD14
ILMN_2232177	-0.5919564	1.2677429	ACTN1
ILMN_1791726	-0.1165403	1.2661247	TUBB3
ILMN_1677505	-0.3786097	1.2465667	CCL21
ILMN_2393765	-1.3157812	1.2446996	IGLL1
ILMN_2192072	-0.4492103	1.2325146	MMP7
ILMN_2088124	-0.6159105	1.2310873	TMEM154
ILMN_2400947	-0.2970516	1.2233969	CELF2
ILMN_1682993	-0.3171521	1.2221096	NKG7
ILMN_1773964	-0.3415281	1.2133846	H1FX
ILMN_1656868	-0.5244166	1.2129158	NPIPL3
ILMN_1659913	-0.2276194	1.2071422	ISG20
ILMN_1700935	-0.5817646	1.2067223	HAS2
ILMN_2109489	-0.3629279	1.1954819	GZMB
ILMN_1719759	-0.4349685	1.1886253	TNC
ILMN_1715508	-0.4731118	1.1747693	NNMT
ILMN_1668039	-0.3205495	1.1702818	GYPC
ILMN_2061043	-0.5361143	1.1639661	CD48
ILMN_1801504	-0.523013	1.1615633	RUNX1
ILMN_3246766	-0.2767946	1.1546525	NPIPL3
ILMN_2366041	-0.6716672	1.1506507	ITM2C
ILMN_1718558	-0.4340115	1.1472871	PARP12
ILMN_2384544	-0.2476885	1.1429716	ADAM15

Pathways

			Count	P_Value	Benjamini
Annotation Cluster 1	Enrichment Score: 7.58		Count	P_Value	Benjamini
	SP_PIR_KEYWORDS	signal	84	3.80E-13	1.20E-10
	UP_SEQ_FEATURE	signal peptide	84	5.40E-13	5.30E-10
	SP_PIR_KEYWORDS	disulfide bond	68	1.90E-08	3.00E-06
	UP_SEQ_FEATURE	disulfide bond	66	3.40E-08	1.70E-05
	SP_PIR_KEYWORDS	glycoprotein	84	5.70E-07	3.10E-05
	SP_PIR_KEYWORDS	Secreted	43	2.20E-06	8.90E-05
	GOTERM_CC_FAT	extracellular region	51	2.50E-05	2.00E-03
	UP_SEQ_FEATURE	glycosylation site:N-linked (GlcNAc...)	75	5.40E-05	1.70E-02
Annotation Cluster 2	Enrichment Score: 4.94		Count	P_Value	Benjamini
	GOTERM_BP_FAT	cell adhesion	27	3.20E-06	1.90E-03
	GOTERM_BP_FAT	biological adhesion	27	3.30E-06	1.40E-03
	SP_PIR_KEYWORDS	cell adhesion	16	1.40E-04	4.10E-03
Annotation Cluster 3	Enrichment Score: 4.58		Count	P_Value	Benjamini
	GOTERM_BP_FAT	defense response	27	2.80E-07	2.50E-04
	GOTERM_BP_FAT	response to wounding	20	1.20E-04	1.60E-02
	GOTERM_BP_FAT	inflammatory response	14	5.30E-04	3.10E-02
Annotation Cluster 4	Enrichment Score: 3.74		Count	P_Value	Benjamini
	SP_PIR_KEYWORDS	extracellular matrix	16	1.70E-07	1.80E-05
	SP_PIR_KEYWORDS	Secreted	43	2.20E-06	8.90E-05
	GOTERM_CC_FAT	extracellular region part	33	3.90E-06	9.50E-04
	GOTERM_CC_FAT	proteinaceous extracellular matrix	17	1.20E-05	1.40E-03
	GOTERM_CC_FAT	extracellular region	51	2.50E-05	2.00E-03
	GOTERM_CC_FAT	extracellular matrix	17	3.00E-05	1.80E-03
	GOTERM_CC_FAT	extracellular space	22	6.50E-04	1.40E-02
	KEGG_PATHWAY	ECM-receptor interaction	6	1.80E-02	1.10E-01
	GOTERM_CC_FAT	basement membrane	5	2.40E-02	2.80E-01
	GOTERM_CC_FAT	extracellular matrix part	6	2.50E-02	2.80E-01
	SP_PIR_KEYWORDS	basement membrane	3	7.60E-02	4.10E-01
Annotation Cluster 5	Enrichment Score: 3.62		Count	P_Value	Benjamini
	GOTERM_BP_FAT	positive regulation of signal transduction	15	5.50E-05	9.50E-03
	GOTERM_BP_FAT	regulation of protein kinase cascade	13	1.60E-04	1.80E-02
	GOTERM_BP_FAT	positive regulation of cell communication	15	1.70E-04	1.70E-02
	GOTERM_BP_FAT	positive regulation of I-kappaB kinase/NF-kappaB cascade	8	3.60E-04	2.70E-02
	GOTERM_BP_FAT	positive regulation of protein kinase cascade	10	4.80E-04	3.20E-02
	GOTERM_BP_FAT	regulation of I-kappaB kinase/NF-kappaB cascade	8	6.60E-04	3.50E-02
Annotation Cluster 6	Enrichment Score: 3.27		Count	P_Value	Benjamini
	GOTERM_BP_FAT	positive regulation of response to stimulus	14	2.20E-05	4.80E-03
	GOTERM_BP_FAT	positive regulation of immune system process	13	1.10E-04	1.50E-02
	GOTERM_BP_FAT	positive regulation of immune response	10	1.70E-04	1.70E-02
	GOTERM_BP_FAT	activation of immune response	8	3.00E-04	2.50E-02
	GOTERM_BP_FAT	lymphocyte mediated immunity	7	3.80E-04	2.70E-02
	GOTERM_BP_FAT	immune effector process	9	5.10E-04	3.20E-02
	GOTERM_BP_FAT	adaptive immune response	7	6.40E-04	3.70E-02
	GOTERM_BP_FAT	adaptive immune response based on somatic recombination of immune receptors built from immunoglobulin superfamily domains	7	6.40E-04	3.70E-02
	GOTERM_BP_FAT	negative regulation of immune system process	7	9.50E-04	4.60E-02
	GOTERM_BP_FAT	B cell mediated immunity	6	9.80E-04	4.50E-02
	GOTERM_BP_FAT	leukocyte mediated immunity	7	1.10E-03	5.10E-02
	GOTERM_BP_FAT	immune response-activating signal transduction	5	5.50E-03	1.50E-01

ILMN_1805737	-0.4868437	1.1419221	PFKP
ILMN_2114720	-0.2910049	1.1358421	SLPI
ILMN_1695851	-0.2123577	1.1309191	PARVG
ILMN_1762594	-0.2364039	1.1272446	NOD2
ILMN_1758895	-0.5701722	1.1245126	CTSK
ILMN_1687301	-0.6841173	1.1204166	VCAN
ILMN_1678827	-0.1769902	1.0991265	SPDYE7P
ILMN_1695404	-0.2068565	1.0963432	LY6E
ILMN_1680453	-0.6901841	1.0810174	ITM2C
ILMN_1747593	-0.5384941	1.079707	ISLR
ILMN_2387496	-0.6742429	1.0743917	COAS3
ILMN_1749372	-0.2665476	1.0736879	GGT5
ILMN_1767556	-0.3998735	1.0715685	C10orf10
ILMN_1736178	-0.4081671	1.068581	AEBP1
ILMN_1772218	-0.4511487	1.0665296	HLA-DPA1
ILMN_2408987	-1.0307062	1.0658907	SMARCD3
ILMN_2376205	-0.7288805	1.0646546	LTB
ILMN_2376204	-0.3408106	1.0642538	LTB
ILMN_1750805	-0.318508	1.0515127	ARHGAP30
ILMN_1752755	-0.7749436	1.049824	VWF
ILMN_2400935	-0.4563044	1.0462416	TAGLN
ILMN_1799062	-0.4098382	1.0438011	NFKB2
ILMN_1769201	-0.4936057	1.0263821	ELF3
ILMN_1708858	-0.7217799	1.0184444	CSNK1E
ILMN_1798181	-0.4995381	1.0047315	IRF7
ILMN_2353202	-0.5386101	1.0010649	PTK7
ILMN_1803686	-0.3430935	1.0008456	ADA
ILMN_1653652	-0.3883707	1.0007647	PTPRC
ILMN_2413779	-0.4982294	0.9999508	SEZ6L2
ILMN_1778668	-0.3758617	0.99820423	TAGLN
ILMN_1744487	-0.5831665	0.9951158	C1QTNF5
ILMN_2261416	-0.3355521	0.99143887	CD3D
ILMN_1765649	-0.2809076	0.9906851	IRF3
ILMN_1745148	-0.4152842	0.9902657	ZNFX1
ILMN_2117508	-0.4875161	0.9897423	CTHRC1
ILMN_2125747	-0.2444001	0.9888768	CORO1A
ILMN_1785005	-0.3391531	0.98682004	NCF4
ILMN_1717793	-0.4363792	0.9829914	C19orf33
ILMN_1676099	-0.1844222	0.98020583	SPON2
ILMN_1768311	-0.2497203	0.97880334	NPIPL3
ILMN_1775762	-0.2994535	0.975834	GNAI2
ILMN_1689968	-0.412196	0.97220707	PLEKHO2
ILMN_1687358	-0.2986337	0.9660672	TMPRSS4
ILMN_1704294	-0.2695893	0.96394974	CDH3
ILMN_1778319	-0.3999426	0.95954514	DMKN
ILMN_1662799	-0.2291535	0.95948136	GPSM3
ILMN_1723978	-0.5471423	0.9585848	LGALS1
ILMN_1673357	-0.334264	0.95752394	SLA2
ILMN_1730670	-0.4762125	0.95550716	FSTL3
ILMN_1691662	-0.2668986	0.95518255	LOC100101266
ILMN_1778941	-0.400535	0.95327836	HNF1B
ILMN_2309848	-0.6369321	0.949838	FXYD5
ILMN_2354334	-0.4306184	0.9450449	ATXN2L
ILMN_1768595	-0.4287379	0.9405392	DLG4
ILMN_2358652	-0.3726217	0.9391262	NXF1

	GOTERM_BP_FAT	immunoglobulin mediated immune response	5	6.30E-03	1.50E-01
**Annotation Cluster 7**	**Enrichment Score: 2.61**			**P_Value**	**Benjamini**
	SP_PIR_KEYWORDS	immune response	15	4.20E-07	3.30E-05
	KEGG_PATHWAY	Cell adhesion molecules (CAMs)	12	2.40E-05	2.40E-03
	BIOCARTA	B Lymphocyte Cell Surface Molecules	5	8.60E-05	2.80E-03
	UP_SEQ_FEATURE	domain:Ig-like C1-type	6	1.00E-04	2.40E-02
	SP_PIR_KEYWORDS	heterodimer	8	2.10E-04	5.50E-03
	KEGG_PATHWAY	Allograft rejection	6	4.40E-04	1.10E-02
	BIOCARTA	Bystander B Cell Activation	4	6.80E-04	1.30E-02
	INTERPRO	Immunoglobulin/major histocompatibility complex, conserved site	7	8.10E-04	1.90E-01
	GOTERM_BP_FAT	antigen processing and presentation	7	9.50E-04	4.60E-02
	UP_SEQ_FEATURE	region of interest:Connecting peptide	5	1.00E-03	1.50E-01
	UP_SEQ_FEATURE	region of interest:Alpha-2	4	1.60E-03	2.00E-01
	UP_SEQ_FEATURE	region of interest:Alpha-1	4	1.60E-03	2.00E-01
	INTERPRO	Immunoglobulin C1-set	6	1.70E-03	2.50E-01
	GOTERM_MF_FAT	MHC class II receptor activity	4	1.90E-03	2.10E-01
	KEGG_PATHWAY	Autoimmune thyroid disease	6	2.20E-03	4.40E-02
	BIOCARTA	Antigen Dependent B Cell Activation	4	2.50E-03	4.10E-02
	SMART	IGc1	6	4.80E-03	4.90E-01
	KEGG_PATHWAY	Graft-versus-host disease	5	5.30E-03	7.40E-02
	SP_PIR_KEYWORDS	mhc ii	4	5.60E-03	8.10E-02
	GOTERM_BP_FAT	antigen processing and presentation of peptide antigen	4	6.40E-03	1.50E-01
	KEGG_PATHWAY	Type I diabetes mellitus	5	6.90E-03	8.50E-02
	GOTERM_CC_FAT	MHC protein complex	5	8.40E-03	1.30E-01
	KEGG_PATHWAY	Systemic lupus erythematosus	7	9.10E-03	9.90E-02
	KEGG_PATHWAY	Viral myocarditis	6	9.20E-03	9.00E-02
	GOTERM_BP_FAT	antigen processing and presentation of peptide or polysaccharide antigen via MHC class II	4	1.00E-02	2.10E-01
	KEGG_PATHWAY	Intestinal immune network for IgA production	5	1.20E-02	8.90E-02
	KEGG_PATHWAY	Asthma	4	1.50E-02	1.10E-01
	KEGG_PATHWAY	Antigen processing and presentation	6	1.70E-02	1.10E-01
	BIOCARTA	Antigen Processing and Presentation	3	2.30E-02	1.90E-01
	INTERPRO	MHC class II, alpha/beta chain, N-terminal	3	3.10E-02	6.40E-01
	GOTERM_CC_FAT	MHC class II protein complex	3	6.20E-02	5.10E-01
	PIR_SUPERFAMILY	PIRSF001991:class II histocompatibility antigen	3	6.30E-02	8.20E-01
	BIOCARTA	Th1/Th2 Differentiation	3	8.30E-02	4.80E-01
**Annotation Cluster 8**	**Enrichment Score: 2.53**		**Count**	**P_Value**	**Benjamini**
	SP_PIR_KEYWORDS	transmembrane protein	25	5.70E-07	3.60E-05
	GOTERM_CC_FAT	integral to plasma membrane	35	4.70E-05	2.30E-03
	GOTERM_CC_FAT	plasma membrane part	53	6.70E-05	2.70E-03
	GOTERM_CC_FAT	intrinsic to plasma membrane	35	7.40E-05	2.60E-03
	GOTERM_CC_FAT	plasma membrane	75	5.70E-04	1.40E-02
	UP_SEQ_FEATURE	topological domain:Extracellular	49	2.30E-03	2.20E-01
	SP_PIR_KEYWORDS	membrane	93	4.90E-03	7.50E-02
	UP_SEQ_FEATURE	topological domain:Cytoplasmic	53	2.20E-02	6.80E-01
	SP_PIR_KEYWORDS	transmembrane	70	5.90E-02	3.60E-01
	UP_SEQ_FEATURE	transmembrane region	69	7.00E-02	9.10E-01
	SP_PIR_KEYWORDS	receptor	26	7.70E-02	4.00E-01
	GOTERM_CC_FAT	integral to membrane	77	4.40E-01	9.30E-01
	GOTERM_CC_FAT	intrinsic to membrane	78	5.40E-01	9.50E-01
**Annotation Cluster 9**	**Enrichment Score: 2.17**		**Count**	**P_Value**	**Benjamini**
	GOTERM_BP_FAT	protein complex assembly	17	1.60E-03	6.30E-02
	GOTERM_BP_FAT	protein complex biogenesis	17	1.60E-03	6.30E-02
	GOTERM_BP_FAT	macromolecular complex assembly	18	9.70E-03	2.00E-01
	GOTERM_BP_FAT	macromolecular complex subunit organization	18	1.80E-02	2.70E-01
	GOTERM_BP_FAT	protein oligomerization	7	3.20E-02	4.00E-01

ILMN_2206746	-0.3115692	0.9350952	BGN
ILMN_1751161	-0.1818679	0.9343799	COL7A1
ILMN_2375319	-0.2491265	0.9336667	RASGRP2
ILMN_2176063	-0.3155814	0.93324673	FCGR1C
ILMN_2367384	-0.5173004	0.93168837	EPHB2
ILMN_1727271	-0.3755743	0.9295296	WARS
ILMN_1770454	-0.4362049	0.9271239	AGRN
ILMN_2208903	-0.2595646	0.9267303	CD52
ILMN_1685625	-0.5453749	0.92595446	UCP2
ILMN_1711994	-0.4643634	0.9215085	TCIRG1
ILMN_1791057	-0.2764992	0.920075	IFNAR2
ILMN_2085862	-0.5261843	0.9159903	SLC15A3
ILMN_2070052	-0.3671393	0.9148916	NPIPL3
ILMN_1679880	-0.3090046	0.91471004	THOC6
ILMN_3243664	-0.4874971	0.91295207	NPIPL3
ILMN_2163723	-0.4142269	0.9102357	KRT7
ILMN_3243238	-0.2350038	0.90686095	TPSAB1
ILMN_1781626	-0.6013486	0.90442723	C1S
ILMN_1700541	-0.3621139	0.903176	FBLN1
ILMN_2206098	-0.3154438	0.9030856	ATG10
ILMN_2405009	-0.6482333	0.9002332	C1orf151
ILMN_1675979	-0.2072399	0.8990485	RBMS3
ILMN_2143685	-0.5237997	0.89562464	CLDN7
ILMN_3237946	-0.36541	0.8955296	PXDN
ILMN_2059549	-0.3507316	0.89245695	SYK
ILMN_1780663	-0.20952	0.890468	KLHL30
ILMN_1786823	-0.4492833	0.88830346	ICAM2
ILMN_1688780	-0.2806633	0.8874346	S100A4
ILMN_1666819	-0.2121657	0.88143635	PHLDB1
ILMN_1711566	-0.6160435	0.87931806	TIMP1
ILMN_1761788	-0.318937	0.8782567	MOXD1
ILMN_1815895	-0.3069491	0.8761135	HLA-DRB1
ILMN_1782057	-0.3002019	0.8751354	ATP8B2
ILMN_1691290	-0.3353199	0.87388796	CELSR3
ILMN_2049536	-0.3740616	0.8712451	TRPV2
ILMN_1807972	-0.2756432	0.8699051	MICAL1
ILMN_1751034	-0.3884175	0.8682534	ITPRIPL2
ILMN_1659905	-0.4851455	0.86781186	FKBP10
ILMN_1782538	-0.4386903	0.8672385	VIM
ILMN_1705783	-0.1545213	0.8622637	NXF1
ILMN_3203186	-0.2641656	0.8597576	AL543610
ILMN_1765446	-0.342277	0.8553318	EMP3
ILMN_1800354	-0.76649	0.854432	CST3
ILMN_1703692	-0.318653	0.8499179	TUBB
ILMN_1724994	-0.3724864	0.8418111	COL4A2
ILMN_2366330	-0.2997862	0.83980304	FERMT3
ILMN_2169801	-0.2454689	0.83863986	TPSAB1
ILMN_2390299	-0.5079346	0.8307578	PSMB8
ILMN_1799120	-0.2293101	0.83003724	WFDC2
ILMN_1670305	-1.0948417	0.8281291	SERPING1
ILMN_2226955	-0.2938926	0.8268525	VOPP1
ILMN_1778401	-0.4915565	0.82583493	HLA-B
ILMN_3236021	-0.3892692	0.824837	ANKRD36
ILMN_1716563	-0.2985041	0.8247995	PRKCB

Annotation Cluster 10	Enrichment Score: 2.15		Count	P_Value	Benjamini
	BIOCARTA	Lck and Fyn tyrosine kinases in initiation of TCR Activation	6	2.70E-06	2.60E-04
	GOTERM_BP_FAT	T cell selection	6	4.50E-06	1.60E-03
	GOTERM_BP_FAT	T cell activation	11	8.80E-06	2.50E-03
	GOTERM_BP_FAT	cell activation	16	9.40E-06	2.30E-03
	BIOCARTA	Activation of Csk by cAMP-dependent Protein Kinase Inhibits Signaling through the T Cell Receptor	6	3.20E-05	1.60E-03
	KEGG_PATHWAY	Primary immunodeficiency	7	3.40E-05	1.70E-03
	GOTERM_BP_FAT	regulation of peptidyl-tyrosine phosphorylation	8	3.80E-05	7.40E-03
	GOTERM_BP_FAT	lymphocyte activation	12	9.20E-05	1.40E-02
	GOTERM_BP_FAT	positive regulation of immune system process	13	1.10E-04	1.50E-02
	GOTERM_BP_FAT	leukocyte activation	13	1.20E-04	1.50E-02
	BIOCARTA	T Helper Cell Surface Molecules	5	1.30E-04	3.10E-03
	GOTERM_BP_FAT	regulation of calcium-mediated signaling	5	1.40E-04	1.60E-02
	GOTERM_BP_FAT	positive regulation of immune response	10	1.70E-04	1.70E-02
	GOTERM_BP_FAT	hemopoietic or lymphoid organ development	13	2.40E-04	2.20E-02
	GOTERM_BP_FAT	T cell differentiation	7	2.60E-04	2.20E-02
	GOTERM_BP_FAT	activation of immune response	8	3.00E-04	2.50E-02
	GOTERM_CC_FAT	cell surface	15	4.10E-04	1.20E-02
	GOTERM_BP_FAT	immune system development	13	4.20E-04	2.90E-02
	GOTERM_BP_FAT	lymphocyte differentiation	8	5.20E-04	3.20E-02
	GOTERM_CC_FAT	T cell receptor complex	4	5.50E-04	1.50E-02
	GOTERM_BP_FAT	thymic T cell selection	4	6.50E-04	3.60E-02
	GOTERM_CC_FAT	external side of plasma membrane	10	6.70E-04	1.40E-02
	BIOCARTA	Bystander B Cell Activation	4	6.80E-04	1.30E-02
	OMIM_DISEASE	Severe combined immunodeficiency, T cell-negative, B-cell/natural killer-cell positive	3	7.30E-04	1.10E-01
	GOTERM_BP_FAT	regulation of cellular protein metabolic process	17	8.00E-04	4.00E-02
	GOTERM_BP_FAT	negative regulation of immune system process	7	9.50E-04	4.60E-02
	GOTERM_BP_FAT	regulation of lymphocyte activation	9	9.70E-04	4.60E-02
	GOTERM_BP_FAT	immune response-regulating signal transduction	6	9.80E-04	4.50E-02
	GOTERM_CC_FAT	receptor complex	8	1.30E-03	2.30E-02
	GOTERM_BP_FAT	positive regulation of calcium-mediated signaling	4	1.50E-03	6.40E-02
	GOTERM_BP_FAT	hemopoiesis	11	1.50E-03	6.50E-02
	GOTERM_BP_FAT	regulation of leukocyte activation	9	2.00E-03	7.70E-02
	GOTERM_BP_FAT	positive regulation of lymphocyte activation	7	2.10E-03	7.90E-02
	GOTERM_BP_FAT	leukocyte differentiation	8	2.10E-03	7.80E-02
	GOTERM_BP_FAT	regulation of protein modification process	12	2.50E-03	8.80E-02
	BIOCARTA	Antigen Dependent B Cell Activation	4	2.50E-03	4.10E-02
	BIOCARTA	T Cytotoxic Cell Surface Molecules	4	2.50E-03	4.10E-02
	GOTERM_BP_FAT	immune response-regulating cell surface receptor signaling pathway	5	2.50E-03	8.80E-02
	GOTERM_BP_FAT	regulation of protein amino acid phosphorylation	9	2.60E-03	8.90E-02
	GOTERM_BP_FAT	regulation of cell activation	9	2.80E-03	9.30E-02
	GOTERM_BP_FAT	positive regulation of leukocyte activation	7	3.30E-03	1.10E-01
	GOTERM_BP_FAT	positive thymic T cell selection	3	3.70E-03	1.20E-01
	GOTERM_BP_FAT	positive regulation of peptidyl-tyrosine phosphorylation	5	3.80E-03	1.20E-01
	GOTERM_BP_FAT	positive regulation of T cell activation	6	3.80E-03	1.20E-01
	GOTERM_BP_FAT	positive regulation of cell activation	7	4.20E-03	1.20E-01
	KEGG_PATHWAY	Hematopoietic cell lineage	7	4.60E-03	7.60E-02
	BIOCARTA	CTL mediated immune response against target cells	4	4.90E-03	6.80E-02
	GOTERM_BP_FAT	negative thymic T cell selection	3	4.90E-03	1.40E-01
	GOTERM_BP_FAT	regulation of B cell activation	5	5.10E-03	1.40E-01
	GOTERM_BP_FAT	positive regulation of alpha-beta T cell activation	4	5.20E-03	1.40E-01
	GOTERM_BP_FAT	immune response-activating signal transduction	5	5.50E-03	1.50E-01
	GOTERM_BP_FAT	regulation of lymphocyte proliferation	6	5.60E-03	1.50E-01
	GOTERM_BP_FAT	T cell differentiation in the thymus	4	5.80E-03	1.50E-01

ILMN_1665943	-0.2437771	0.8244704	MAP4K1
ILMN_1779257	-0.3663034	0.8213204	CD40
ILMN_1684306	-0.4423175	0.8178873	S100A4
ILMN_1713901	-0.2925592	0.81592023	KDELR3
ILMN_1785061	-0.2745637	0.8151372	EPHB2
ILMN_2111739	-0.4774336	0.8130309	MAN2C1
ILMN_2408400	-0.2415248	0.81145334	NSUN5
ILMN_1666078	-0.2263312	0.80954427	HLA-H
ILMN_2058251	-0.3064233	0.80710375	VIM
ILMN_2062468	-0.5679551	0.8070511	IGFBP7
ILMN_1794707	-0.354936	0.80371445	ATHL1
ILMN_1805750	-0.39717	0.79880387	IFITM3
ILMN_1735148	-0.3728589	0.79594487	SEC24B
ILMN_1808777	-0.3808807	0.79474026	EHD2
ILMN_1689004	-0.4236876	0.7932473	TNFRSF12A
ILMN_1785272	-0.4044341	0.79318005	COL1A2
ILMN_1789599	-0.6776331	0.7889098	C1orf151
ILMN_2098126	-0.33206	0.7836308	CCL5
ILMN_1713636	-0.3276164	0.7820336	S100A6
ILMN_1720287	-0.2498446	0.7791793	E4F1
ILMN_1690125	-0.4682108	0.77619785	PDLIM7
ILMN_1747195	-0.5296944	0.77611643	PSMB8
ILMN_1712545	-0.3020494	0.7758006	S100A3
ILMN_1730777	-0.3567672	0.77183926	KRT19
ILMN_1671295	-0.2948444	0.77169406	MST151
ILMN_1653220	-0.2644714	0.7711403	PITPNM1
ILMN_1705750	-0.4122929	0.7699518	TGM2
ILMN_3248247	-0.3075986	0.76758534	LOC349114
ILMN_1654246	-0.6002158	0.7673532	SIRT6
ILMN_1815500	-0.3069432	0.7641663	ITPR3
ILMN_1787109	-0.5007137	0.76132065	CLK2
ILMN_1719449	-0.3440301	0.754508	DCLK2
ILMN_1815057	-0.3384102	0.75351226	PDGFRB
ILMN_2155719	-0.5270782	0.7532077	NBPF1
ILMN_1777342	-0.2784544	0.75301546	PREX1
ILMN_1811702	-0.4603844	0.7490145	GRN
ILMN_1705602	-0.4110136	0.7484236	KLHL17
ILMN_1651767	-0.3164083	0.7468634	MKL1
ILMN_1895548	-0.2717167	0.7460645	AK021726
ILMN_1780806	-0.3624143	0.7455973	ANKRD36B
ILMN_3228688	-0.582792	0.74362123	HLA-DRB1
ILMN_1738675	-0.2855184	0.7427395	PTPN6
ILMN_2090105	-0.9173182	0.7417683	TAGLN2
ILMN_1752526	-0.308724	0.73536175	RNF144B
ILMN_2101885	-0.3070515	0.73364246	TUBB
ILMN_1675709	-0.2769983	0.7305087	ARFGAP1
ILMN_1803988	-0.3516586	0.72845715	MCL1
ILMN_1708025	-0.3238424	0.7281853	RBPMS
ILMN_3246005	-0.3095196	0.72722805	KDM5B
ILMN_1691717	-0.3052667	0.72658294	RHBDF2
ILMN_1807493	-0.4520136	0.7222337	ACVRL1
ILMN_1727043	-0.2914142	0.721914	GLT25D1
ILMN_1814917	-0.3462726	0.72037286	TLE2
ILMN_1652631	-0.5696183	0.71945685	GLIPR2

GOTERM_BP_FAT	regulation of leukocyte proliferation	6	5.90E-03	1.50E-01
GOTERM_BP_FAT	regulation of mononuclear cell proliferation	6	5.90E-03	1.50E-01
GOTERM_BP_FAT	positive T cell selection	3	6.30E-03	1.50E-01
GOTERM_BP_FAT	negative T cell selection	3	6.30E-03	1.50E-01
GOTERM_BP_FAT	positive regulation of alpha-beta T cell proliferation	3	6.30E-03	1.50E-01
GOTERM_BP_FAT	positive regulation of lymphocyte proliferation	5	6.70E-03	1.60E-01
GOTERM_BP_FAT	positive regulation of mononuclear cell proliferation	5	7.10E-03	1.60E-01
GOTERM_BP_FAT	positive regulation of leukocyte proliferation	5	7.10E-03	1.60E-01
GOTERM_BP_FAT	positive regulation of T cell differentiation	4	9.30E-03	2.00E-01
BIOCARTA	The Co-Stimulatory Signal During T-cell Activation	4	9.80E-03	1.10E-01
GOTERM_BP_FAT	positive regulation of B cell activation	4	1.00E-02	2.10E-01
GOTERM_BP_FAT	antigen receptor-mediated signaling pathway	4	1.00E-02	2.10E-01
GOTERM_BP_FAT	regulation of humoral immune response	3	1.10E-02	2.20E-01
GOTERM_BP_FAT	positive regulation of cell proliferation	13	1.20E-02	2.20E-01
GOTERM_BP_FAT	regulation of phosphorylation	14	1.20E-02	2.20E-01
GOTERM_BP_FAT	positive regulation of lymphocyte differentiation	4	1.20E-02	2.20E-01
GOTERM_BP_FAT	regulation of cytokine production	8	1.20E-02	2.20E-01
GOTERM_BP_FAT	regulation of alpha-beta T cell activation	4	1.30E-02	2.30E-01
GOTERM_BP_FAT	regulation of alpha-beta T cell proliferation	3	1.30E-02	2.30E-01
GOTERM_MF_FAT	protein kinase binding	7	1.40E-02	3.50E-01
GOTERM_BP_FAT	positive regulation of protein modification process	8	1.40E-02	2.50E-01
GOTERM_BP_FAT	positive regulation of cellular protein metabolic process	9	1.50E-02	2.50E-01
GOTERM_BP_FAT	regulation of phosphorus metabolic process	14	1.60E-02	2.60E-01
GOTERM_BP_FAT	regulation of phosphate metabolic process	14	1.60E-02	2.60E-01
GOTERM_BP_FAT	immune response-activating cell surface receptor signaling pathway	4	1.60E-02	2.60E-01
SP_PIR_KEYWORDS	scid	3	1.70E-02	1.60E-01
GOTERM_BP_FAT	regulation of MAPKKK cascade	6	1.70E-02	2.70E-01
KEGG_PATHWAY	Antigen processing and presentation	6	1.70E-02	1.10E-01
BIOCARTA	HIV Induced T Cell Apoptosis	3	1.90E-02	1.70E-01
GOTERM_BP_FAT	positive regulation of protein metabolic process	9	1.90E-02	2.80E-01
GOTERM_BP_FAT	protein amino acid phosphorylation	17	2.10E-02	3.10E-01
GOTERM_BP_FAT	regulation of T cell activation	6	2.20E-02	3.20E-01
BIOCARTA	IL 5 Signaling Pathway	3	2.30E-02	1.90E-01
GOTERM_BP_FAT	activation of MAPK activity	5	2.60E-02	3.50E-01
GOTERM_BP_FAT	positive regulation of cytokine biosynthetic process	4	2.80E-02	3.60E-01
GOTERM_BP_FAT	positive regulation of B cell proliferation	3	3.00E-02	3.80E-01
GOTERM_BP_FAT	protein kinase cascade	11	3.10E-02	3.90E-01
GOTERM_MF_FAT	kinase binding	7	3.20E-02	4.80E-01
GOTERM_BP_FAT	regulation of T cell differentiation	4	3.20E-02	4.00E-01
GOTERM_BP_FAT	positive regulation of protein amino acid phosphorylation	5	3.40E-02	4.10E-01
GOTERM_BP_FAT	positive regulation of protein kinase activity	8	3.40E-02	4.00E-01
GOTERM_BP_FAT	cell proliferation	12	3.70E-02	4.30E-01
GOTERM_BP_FAT	regulation of adaptive immune response based on somatic recombination of immune receptors built from immunoglobulin superfamily domains	4	3.90E-02	4.40E-01
GOTERM_BP_FAT	positive regulation of kinase activity	8	4.00E-02	4.40E-01
GOTERM_BP_FAT	MAPKKK cascade	7	4.10E-02	4.50E-01
GOTERM_BP_FAT	regulation of adaptive immune response	4	4.10E-02	4.40E-01
GOTERM_BP_FAT	regulation of B cell mediated immunity	3	4.20E-02	4.50E-01
GOTERM_BP_FAT	regulation of immunoglobulin mediated immune response	3	4.20E-02	4.50E-01
GOTERM_BP_FAT	positive regulation of phosphorylation	5	4.40E-02	4.60E-01
GOTERM_BP_FAT	positive regulation of transferase activity	8	4.70E-02	4.70E-01
KEGG_PATHWAY	T cell receptor signaling pathway	6	4.70E-02	2.10E-01
GOTERM_BP_FAT	positive regulation of phosphate metabolic process	5	4.80E-02	4.80E-01
GOTERM_BP_FAT	positive regulation of phosphorus metabolic process	5	4.80E-02	4.80E-01
BIOCARTA	IL 17 Signaling Pathway	3	5.00E-02	3.40E-01

ILMN_1689655	-0.352833	0.7193123	HLA-DRA
ILMN_2157441	-0.3784043	0.7175189	HLA-DRA
ILMN_3229210	-0.3558461	0.71513367	FLJ45340
ILMN_1779010	-0.3212784	0.71320313	MAP3K3
ILMN_1765021	-0.4371012	0.7113927	TOP3B
ILMN_2380237	-0.3958364	0.71032566	C1QTNF1
ILMN_2278729	-0.3459377	0.7091275	EIF3B
ILMN_2375992	-0.344336	0.7083419	SPINT1
ILMN_3230608	-0.4429316	0.7077526	NBPF14
ILMN_2371911	-0.9583868	0.7015006	MUC1
ILMN_1736567	-0.5390354	0.7010446	CD74
ILMN_1668748	-0.3158949	0.70089024	MED15
ILMN_1811117	-0.3808893	0.69952255	DKFZp667P0924
ILMN_1703593	-0.394547	0.6987069	BAIAP2L1
ILMN_1778111	-0.3320264	0.69664574	FLJ45340
ILMN_2366634	-0.3590136	0.69484407	PKM2
ILMN_2407434	-0.3631673	0.6850699	DCLK2
ILMN_2382431	-0.8878012	0.6826305	ANKRD36
ILMN_1699931	-0.4965842	0.6793603	HCST
ILMN_2383489	-0.3749546	0.6792119	CDK5RAP3
ILMN_1752592	-0.3833149	0.6780105	HLA-DRB4
ILMN_2379644	-0.5249819	0.67535543	CD74
ILMN_1701643	-0.3674708	0.6733213	GDPD5
ILMN_1656920	-0.499193	0.67319584	CRIP1
ILMN_1737157	-0.6897214	0.6702828	GRAMD1A
ILMN_1739794	-0.4857385	0.666808	CD3E
ILMN_1758315	-0.4451488	0.6659471	SLC9A9
ILMN_2175912	-0.4234652	0.6652375	ITGB2
ILMN_1683026	-0.388655	0.66396236	PSMB10
ILMN_1753584	-0.5324534	0.66179246	KRT8
ILMN_1727402	-0.5068444	0.6616992	HCLS1
ILMN_2147133	-0.4381964	0.65923274	NBPF1
ILMN_1676256	-0.3549909	0.658206	TPSAB1
ILMN_1737205	-0.4199736	0.65487623	MCM4
ILMN_1790637	-0.375038	0.65421516	C11orf80
ILMN_2161746	-0.4203926	0.6510027	TRIP10
ILMN_2410783	-0.6115214	0.6435011	GAA
ILMN_2064606	-0.4619445	0.64152145	TBC1D2B
ILMN_1674874	-0.3659361	0.64023876	MFSD10
ILMN_1761138	-0.4229533	0.6345543	C9orf142
ILMN_1742330	-0.5733025	0.631179	PLXNB1
ILMN_1754842	-1.1469843	0.6292545	DLGAP4
ILMN_2395375	-0.43235	0.62881	GABBR1
ILMN_3200362	-0.5219584	0.6270053	NBPF1
ILMN_1761912	-0.5853727	0.61685646	MGAT1
ILMN_2398847	-0.4906956	0.6160503	ARHGAP17
ILMN_1773885	-0.9395435	0.61211187	DMAP1
ILMN_2066066	-0.4463386	0.6036648	HLA-DRB6
ILMN_1683576	-0.4752797	0.6011738	MAGED2
ILMN_2349658	-0.976337	0.5986835	TSPO
ILMN_1778360	-0.5825829	0.594741	PYGB
ILMN_1653828	-0.452502	0.5902511	CHFR
ILMN_2410909	-0.4180519	0.585066	AKT1
ILMN_2114422	-0.4646005	0.5784965	NOD1

	GOTERM_BP_FAT	regulation of immune effector process	5	5.00E-02	4.80E-01
	GOTERM_BP_FAT	regulation of leukocyte mediated immunity	4	5.10E-02	4.90E-01
	GOTERM_BP_FAT	positive regulation of MAP kinase activity	5	5.10E-02	4.90E-01
	GOTERM_BP_FAT	negative regulation of cell communication	8	5.40E-02	5.00E-01
	GOTERM_BP_FAT	regulation of lymphocyte differentiation	4	5.50E-02	5.00E-01
	GOTERM_BP_FAT	regulation of B cell proliferation	3	5.60E-02	5.00E-01
	GOTERM_BP_FAT	positive regulation of adaptive immune response based on somatic recombination of immune receptors built from immunoglobulin superfamily domains	3	6.30E-02	5.40E-01
	GOTERM_BP_FAT	positive regulation of adaptive immune response	3	6.70E-02	5.50E-01
	GOTERM_BP_FAT	regulation of cytokine biosynthetic process	4	8.10E-02	6.10E-01
	GOTERM_BP_FAT	negative regulation of signal transduction	7	8.30E-02	6.10E-01
	GOTERM_BP_FAT	B cell activation	4	8.60E-02	6.20E-01
	GOTERM_MF_FAT	enzyme binding	12	8.80E-02	7.10E-01
	GOTERM_BP_FAT	positive regulation of T cell proliferation	3	9.90E-02	6.50E-01
	GOTERM_BP_FAT	positive regulation of catalytic activity	12	1.00E-01	6.50E-01
	GOTERM_BP_FAT	regulation of protein kinase activity	9	1.00E-01	6.50E-01
	GOTERM_BP_FAT	positive regulation of molecular function	13	1.10E-01	6.70E-01
	GOTERM_BP_FAT	regulation of production of molecular mediator of immune response	3	1.10E-01	6.70E-01
	GOTERM_BP_FAT	regulation of kinase activity	9	1.20E-01	6.80E-01
	GOTERM_BP_FAT	positive regulation of immune effector process	3	1.20E-01	6.90E-01
	GOTERM_BP_FAT	regulation of MAP kinase activity	5	1.30E-01	7.00E-01
	BIOCARTA	Cytokines and Inflammatory Response	3	1.30E-01	6.30E-01
	GOTERM_BP_FAT	regulation of transferase activity	9	1.40E-01	7.20E-01
	GOTERM_BP_FAT	negative regulation of response to stimulus	4	1.60E-01	7.50E-01
	GOTERM_BP_FAT	regulation of lymphocyte mediated immunity	3	1.70E-01	7.70E-01
	SP_PIR_KEYWORDS	alternative initiation	3	1.70E-01	6.20E-01
	GOTERM_BP_FAT	regulation of T cell proliferation	3	2.10E-01	8.20E-01
	GOTERM_BP_FAT	regulation of secretion	4	5.20E-01	9.80E-01
	GOTERM_BP_FAT	regulation of cellular localization	3	8.60E-01	1.00E+00
**Annotation Cluster 11**	**Enrichment Score: 1.9**		**Count**	**P_Value**	**Benjamini**
	GOTERM_BP_FAT	response to virus	8	7.40E-04	3.80E-02
	KEGG_PATHWAY	Toll-like receptor signaling pathway	7	1.00E-02	8.90E-02
	KEGG_PATHWAY	Cytosolic DNA-sensing pathway	3	2.70E-01	6.80E-01
**Annotation Cluster 12**	**Enrichment Score: 1.87**		**Count**	**P_Value**	**Benjamini**
	GOTERM_BP_FAT	programmed cell death	19	1.80E-03	7.10E-02
	GOTERM_BP_FAT	apoptosis	17	8.50E-03	1.90E-01
	GOTERM_BP_FAT	cell death	19	9.70E-03	2.00E-01
	GOTERM_BP_FAT	death	19	1.00E-02	2.10E-01
	SP_PIR_KEYWORDS	Apoptosis	7	2.90E-01	7.40E-01
**Annotation Cluster 13**	**Enrichment Score: 1.71**		**Count**	**P_Value**	**Benjamini**
	SP_PIR_KEYWORDS	collagen	7	8.80E-04	1.70E-02
	INTERPRO	Collagen triple helix repeat	6	4.20E-03	3.10E-01
	SP_PIR_KEYWORDS	hydroxyproline	4	9.20E-03	1.20E-01
	SP_PIR_KEYWORDS	hydroxylation	5	9.60E-03	1.20E-01
	GOTERM_BP_FAT	extracellular structure organization	7	2.50E-02	3.40E-01
	GOTERM_CC_FAT	extracellular matrix part	6	2.50E-02	2.80E-01
	SP_PIR_KEYWORDS	hydroxylysine	3	5.10E-02	3.30E-01
	SP_PIR_KEYWORDS	triple helix	3	5.10E-02	3.30E-01
	GOTERM_BP_FAT	extracellular matrix organization	5	5.40E-02	4.90E-01
	UP_SEQ_FEATURE	domain:Collagen-like	3	6.70E-02	9.10E-01
	GOTERM_CC_FAT	collagen	3	8.70E-02	6.00E-01
**Annotation Cluster 14**	**Enrichment Score: 1.68**		**Count**	**P_Value**	**Benjamini**
	GOTERM_BP_FAT	regulation of protein kinase cascade	13	1.60E-04	1.80E-02
	GOTERM_BP_FAT	regulation of JNK cascade	5	1.20E-02	2.20E-01
	GOTERM_BP_FAT	regulation of stress-activated protein kinase signaling pathway	5	1.50E-02	2.50E-01

ILMN_1786197	-0.4536233	0.56482023	NR2F1
ILMN_1752340	-0.6061316	0.56157315	ARF5
ILMN_1761068	-0.5335256	0.56072944	DKFZp434K1323
ILMN_1728049	-0.517863	0.51490104	S100A16
ILMN_1772888	-0.6104952	0.44741943	AX746718
ILMN_3281563	-0.7814937	0.377732	RPL12

	GOTERM_BP_FAT	regulation of MAPKKK cascade		6	1.70E-02	2.70E-01
	GOTERM_BP_FAT	regulation of cellular response to stress		5	5.30E-02	4.90E-01
	GOTERM_BP_FAT	positive regulation of molecular function		13	1.10E-01	6.70E-01
	GOTERM_BP_FAT	positive regulation of transport		4	5.90E-01	9.90E-01
**Annotation Cluster 15**	**Enrichment Score: 1.56**		**Count**		**P_Value**	**Benjamini**
	SP_PIR_KEYWORDS	protease inhibitor		6	8.50E-03	1.10E-01
	GOTERM_MF_FAT	endopeptidase inhibitor activity		7	1.30E-02	3.60E-01
	SP_PIR_KEYWORDS	Serine protease inhibitor		5	1.40E-02	1.50E-01
	GOTERM_MF_FAT	peptidase inhibitor activity		7	1.60E-02	3.60E-01
	GOTERM_MF_FAT	serine-type endopeptidase inhibitor activity		5	3.40E-02	4.80E-01
	UP_SEQ_FEATURE	site:Reactive bond		3	9.80E-02	9.30E-01
	GOTERM_MF_FAT	enzyme inhibitor activity		7	1.50E-01	7.50E-01
**Annotation Cluster 16**	**Enrichment Score: 1.54**		**Count**		**P_Value**	**Benjamini**
	SP_PIR_KEYWORDS	zymogen		10	7.40E-04	1.60E-02
	UP_SEQ_FEATURE	propeptide:Activation peptide		6	1.70E-03	1.90E-01
	UP_SEQ_FEATURE	domain:Peptidase S1		6	8.40E-03	5.30E-01
	INTERPRO	Peptidase S1A, chymotrypsin		6	1.00E-02	4.50E-01
	INTERPRO	Peptidase S1/S6, chymotrypsin/Hap, active site		6	1.10E-02	4.30E-01
	INTERPRO	Peptidase S1 and S6, chymotrypsin/Hap		6	1.60E-02	4.50E-01
	PIR_SUPERFAMILY	PIRSF001135:trypsin		4	1.90E-02	7.10E-01
	SP_PIR_KEYWORDS	Serine protease		6	2.20E-02	2.00E-01
	SP_PIR_KEYWORDS	serine proteinase		4	2.50E-02	2.20E-01
	GOTERM_MF_FAT	endopeptidase activity		11	2.80E-02	4.50E-01
	SP_PIR_KEYWORDS	Protease		12	2.80E-02	2.30E-01
	SMART	Tryp_SPc		6	4.00E-02	8.50E-01
	GOTERM_MF_FAT	serine-type endopeptidase activity		6	5.40E-02	6.00E-01
	GOTERM_MF_FAT	peptidase activity		13	7.90E-02	7.20E-01
	UP_SEQ_FEATURE	active site:Charge relay system		6	8.30E-02	9.30E-01
	GOTERM_MF_FAT	serine-type peptidase activity		6	8.80E-02	7.00E-01
	GOTERM_MF_FAT	serine hydrolase activity		6	9.10E-02	6.90E-01
	GOTERM_MF_FAT	peptidase activity, acting on L-amino acid peptides		12	1.10E-01	7.20E-01
	GOTERM_BP_FAT	proteolysis		18	2.70E-01	8.80E-01
	SP_PIR_KEYWORDS	hydrolase		22	2.80E-01	7.40E-01
**Annotation Cluster 17**	**Enrichment Score: 1.51**		**Count**		**P_Value**	**Benjamini**
	UP_SEQ_FEATURE	region of interest:Coil 2		5	6.80E-03	4.90E-01
	UP_SEQ_FEATURE	region of interest:Linker 12		5	6.80E-03	4.90E-01
	UP_SEQ_FEATURE	region of interest:Coil 1B		5	9.40E-03	5.30E-01
	UP_SEQ_FEATURE	region of interest:Linker 1		5	9.40E-03	5.30E-01
	UP_SEQ_FEATURE	region of interest:Coil 1A		5	9.40E-03	5.30E-01
	UP_SEQ_FEATURE	region of interest:Rod		5	9.80E-03	5.20E-01
	UP_SEQ_FEATURE	region of interest:Head		5	1.10E-02	5.30E-01
	INTERPRO	Filament		5	1.30E-02	4.30E-01
	INTERPRO	Intermediate filament protein, conserved site		5	1.30E-02	4.30E-01
	SP_PIR_KEYWORDS	Intermediate filament		5	1.30E-02	1.40E-01
	INTERPRO	Intermediate filament protein		5	1.40E-02	4.20E-01
	PIR_SUPERFAMILY	PIRSF002282:cytoskeletal keratin		5	1.60E-02	8.70E-01
	INTERPRO	Type II keratin		3	4.80E-02	7.10E-01
	UP_SEQ_FEATURE	site:Stutter		3	5.70E-02	8.90E-01
	UP_SEQ_FEATURE	region of interest:Tail		4	5.90E-02	8.90E-01
	GOTERM_BP_FAT	cytoskeleton organization		11	7.70E-02	6.00E-01
	GOTERM_CC_FAT	intermediate filament		6	1.20E-01	6.90E-01
	GOTERM_CC_FAT	intermediate filament cytoskeleton		6	1.20E-01	7.00E-01
	SP_PIR_KEYWORDS	keratin		4	2.40E-01	7.00E-01
	GOTERM_CC_FAT	keratin filament		3	3.50E-01	9.20E-01
	GOTERM_CC_FAT	cytoskeletal part		14	5.90E-01	9.60E-01
**Annotation Cluster 18**	**Enrichment Score: 1.46**		**Count**		**P_Value**	**Benjamini**
	INTERPRO	Immunoglobulin-like fold		20	7.70E-05	3.90E-02
	INTERPRO	Immunoglobulin/major histocompatibility complex, conserved site		7	8.10E-04	1.90E-01
	INTERPRO	Immunoglobulin-like		16	1.70E-03	2.00E-01

	SP_PIR_KEYWORDS	Immunoglobulin domain	13	9.60E-03	1.10E-01
	INTERPRO	Immunoglobulin subtype	9	5.80E-02	7.30E-01
	UP_SEQ_FEATURE	domain:Ig-like C2-type 1	6	7.40E-02	9.20E-01
	UP_SEQ_FEATURE	domain:Ig-like C2-type 2	6	7.60E-02	9.20E-01
	SMART	IG	9	1.70E-01	9.60E-01
	UP_SEQ_FEATURE	domain:Ig-like C2-type 3	4	1.70E-01	9.80E-01
	UP_SEQ_FEATURE	domain:Ig-like V-type	4	1.80E-01	9.80E-01
	INTERPRO	Immunoglobulin subtype 2	5	2.60E-01	9.60E-01
	SMART	IGc2	5	4.30E-01	9.90E-01
	INTERPRO	Immunoglobulin	4	4.70E-01	1.00E+00
**Annotation Cluster 19**	**Enrichment Score: 1.46**		**Count**	**P_Value**	**Benjamini**
	GOTERM_BP_FAT	in utero embryonic development	8	1.10E-02	2.10E-01
	GOTERM_BP_FAT	blastocyst development	4	2.10E-02	3.00E-01
	GOTERM_BP_FAT	blastocyst formation	3	3.00E-02	3.80E-01
	GOTERM_BP_FAT	chordate embryonic development	9	8.60E-02	6.20E-01
	GOTERM_BP_FAT	embryonic development ending in birth or egg hatching	9	8.80E-02	6.20E-01
**Annotation Cluster 20**	**Enrichment Score: 1.44**		**Count**	**P_Value**	**Benjamini**
	GOTERM_CC_FAT	lysosome	8	3.00E-02	3.00E-01
	GOTERM_CC_FAT	lytic vacuole	8	3.00E-02	3.00E-01
	SP_PIR_KEYWORDS	lysosome	6	3.10E-02	2.40E-01
	GOTERM_CC_FAT	vacuole	8	6.60E-02	5.20E-01
**Annotation Cluster 21**	**Enrichment Score: 1.33**		**Count**	**P_Value**	**Benjamini**
	SP_PIR_KEYWORDS	calcium binding	7	1.30E-03	2.30E-02
	INTERPRO	S100/CaBP-9k-type, calcium binding, subdomain	4	4.60E-03	2.90E-01
	INTERPRO	S100/CaBP-9k-type, calcium binding	4	5.10E-03	2.90E-01
	UP_SEQ_FEATURE	calcium-binding region:1; low affinity	3	1.90E-02	6.60E-01
	INTERPRO	EF-HAND 1	8	2.00E-02	5.10E-01
	UP_SEQ_FEATURE	domain:EF-hand 1	7	2.10E-02	6.80E-01
	UP_SEQ_FEATURE	domain:EF-hand 2	7	2.10E-02	6.80E-01
	UP_SEQ_FEATURE	calcium-binding region:2; high affinity	3	2.50E-02	7.10E-01
	SP_PIR_KEYWORDS	EF hand	4	3.30E-02	2.40E-01
	PIR_SUPERFAMILY	PIRSF002353:S-100 protein	3	3.60E-02	7.90E-01
	INTERPRO	EF-HAND 2	7	5.50E-02	7.30E-01
	INTERPRO	EF-Hand type	7	7.90E-02	7.60E-01
	UP_SEQ_FEATURE	calcium-binding region:2	3	3.90E-01	1.00E+00
	UP_SEQ_FEATURE	calcium-binding region:1	3	4.40E-01	1.00E+00
	INTERPRO	EF hand	3	4.90E-01	1.00E+00
	INTERPRO	Calcium-binding EF-hand	3	5.40E-01	1.00E+00
	SMART	EFh	3	6.80E-01	1.00E+00
**Annotation Cluster 22**	**Enrichment Score: 1.31**		**Count**	**P_Value**	**Benjamini**
	GOTERM_BP_FAT	cell migration	11	4.70E-03	1.40E-01
	GOTERM_BP_FAT	cell motion	15	5.70E-03	1.50E-01
	GOTERM_BP_FAT	cell motility	11	9.70E-03	2.10E-01
	GOTERM_BP_FAT	localization of cell	11	9.70E-03	2.10E-01
	SP_PIR_KEYWORDS	inflammatory response	5	1.30E-02	1.40E-01
	GOTERM_BP_FAT	cell chemotaxis	4	1.60E-02	2.60E-01
	KEGG_PATHWAY	Cytokine-cytokine receptor interaction	11	2.10E-02	1.10E-01
	GOTERM_BP_FAT	taxis	7	2.30E-02	3.20E-01
	GOTERM_BP_FAT	chemotaxis	7	2.30E-02	3.20E-01
	INTERPRO	Small chemokine, C-C group, conserved site	3	3.90E-02	6.70E-01
	PIR_SUPERFAMILY	PIRSF001950:small inducible chemokine, C/CC types	3	5.10E-02	8.10E-01
	GOTERM_BP_FAT	locomotory behavior	8	8.20E-02	6.10E-01
	GOTERM_BP_FAT	leukocyte chemotaxis	3	9.10E-02	6.30E-01
	INTERPRO	Small chemokine, interleukin-8-like	3	9.40E-02	8.00E-01
	GOTERM_BP_FAT	behavior	11	1.10E-01	6.70E-01
	GOTERM_MF_FAT	chemokine activity	3	1.20E-01	7.30E-01
	GOTERM_MF_FAT	chemokine receptor binding	3	1.40E-01	7.60E-01
	SMART	SCY	3	1.40E-01	9.50E-01
	SP_PIR_KEYWORDS	cytokine	5	1.60E-01	6.10E-01
	GOTERM_BP_FAT	leukocyte migration	3	1.80E-01	7.90E-01

	BBID	109.Chemokine_families	3	2.00E-01	9.60E-01
	SP_PIR_KEYWORDS	chemotaxis	3	2.10E-01	6.70E-01
	GOTERM_MF_FAT	cytokine activity	5	2.60E-01	8.70E-01
**Annotation Cluster 23**	**Enrichment Score: 1.31**		**Count**	**P_Value**	**Benjamini**
	GOTERM_BP_FAT	virus-host interaction	3	3.00E-02	3.80E-01
	GOTERM_BP_FAT	viral reproductive process	4	5.30E-02	4.90E-01
	GOTERM_BP_FAT	viral reproduction	4	7.30E-02	5.80E-01
**Annotation Cluster 24**	**Enrichment Score: 1.31**		**Count**	**P_Value**	**Benjamini**
	GOTERM_BP_FAT	negative regulation of immune system process	7	9.50E-04	4.60E-02
	GOTERM_BP_FAT	negative regulation of lymphocyte activation	3	1.70E-01	7.70E-01
	GOTERM_BP_FAT	negative regulation of leukocyte activation	3	1.80E-01	7.90E-01
	GOTERM_BP_FAT	negative regulation of cell activation	3	2.00E-01	8.10E-01
**Annotation Cluster 25**	**Enrichment Score: 1.29**		**Count**	**P_Value**	**Benjamini**
	SP_PIR_KEYWORDS	sushi	4	2.80E-02	2.30E-01
	INTERPRO	Sushi/SCR/CCP	4	3.40E-02	6.50E-01
	INTERPRO	Complement control module	4	3.50E-02	6.50E-01
	SMART	CCP	4	6.20E-02	8.90E-01
	UP_SEQ_FEATURE	domain:Sushi 2	3	9.50E-02	9.30E-01
	UP_SEQ_FEATURE	domain:Sushi 1	3	9.50E-02	9.30E-01
**Annotation Cluster 26**	**Enrichment Score: 1.26**		**Count**	**P_Value**	**Benjamini**
	GOTERM_BP_FAT	cellular calcium ion homeostasis	8	1.30E-02	2.30E-01
	GOTERM_BP_FAT	calcium ion homeostasis	8	1.50E-02	2.50E-01
	GOTERM_BP_FAT	cellular metal ion homeostasis	8	1.80E-02	2.80E-01
	GOTERM_BP_FAT	metal ion homeostasis	8	2.30E-02	3.20E-01
	GOTERM_BP_FAT	cellular di-, tri-valent inorganic cation homeostasis	8	3.70E-02	4.20E-01
	GOTERM_BP_FAT	cation homeostasis	9	4.30E-02	4.50E-01
	GOTERM_BP_FAT	di-, tri-valent inorganic cation homeostasis	8	4.60E-02	4.70E-01
	GOTERM_BP_FAT	homeostatic process	17	5.20E-02	4.90E-01
	GOTERM_BP_FAT	cellular cation homeostasis	8	6.00E-02	5.20E-01
	GOTERM_BP_FAT	elevation of cytosolic calcium ion concentration	5	6.40E-02	5.40E-01
	GOTERM_BP_FAT	cytosolic calcium ion homeostasis	5	7.90E-02	6.00E-01
	GOTERM_BP_FAT	cellular homeostasis	11	1.10E-01	6.70E-01
	GOTERM_BP_FAT	ion homeostasis	10	1.10E-01	6.70E-01
	GOTERM_BP_FAT	cellular ion homeostasis	9	1.40E-01	7.20E-01
	GOTERM_BP_FAT	cellular chemical homeostasis	9	1.50E-01	7.40E-01
	GOTERM_BP_FAT	chemical homeostasis	10	2.70E-01	8.80E-01
**Annotation Cluster 27**	**Enrichment Score: 1.2**		**Count**	**P_Value**	**Benjamini**
	UP_SEQ_FEATURE	domain:SH3	7	1.90E-02	6.80E-01
	SP_PIR_KEYWORDS	sh3 domain	7	3.70E-02	2.60E-01
	INTERPRO	Src homology-3 domain	7	5.30E-02	7.30E-01
	SMART	SH3	7	1.30E-01	9.80E-01
	INTERPRO	Variant SH3	3	2.00E-01	9.20E-01
**Annotation Cluster 28**	**Enrichment Score: 1.16**		**Count**	**P_Value**	**Benjamini**
	INTERPRO	Calponin-like actin-binding	5	1.20E-02	4.30E-01
	SMART	CH	5	2.70E-02	8.50E-01
	UP_SEQ_FEATURE	domain:CH	3	8.70E-02	9.30E-01
	SP_PIR_KEYWORDS	cytoskeleton	6	8.70E-01	1.00E+00
**Annotation Cluster 29**	**Enrichment Score: 1.14**		**Count**	**P_Value**	**Benjamini**
	KEGG_PATHWAY	Toll-like receptor signaling pathway	7	1.00E-02	8.90E-02
	GOTERM_BP_FAT	response to lipopolysaccharide	4	8.90E-02	6.20E-01
	GOTERM_BP_FAT	response to molecule of bacterial origin	4	1.10E-01	6.80E-01
	GOTERM_BP_FAT	response to bacterium	5	2.70E-01	8.80E-01
**Annotation Cluster 30**	**Enrichment Score: 1.1**		**Count**	**P_Value**	**Benjamini**
	SP_PIR_KEYWORDS	phosphotransferase	7	3.10E-02	2.40E-01
	SP_PIR_KEYWORDS	autophosphorylation	3	1.10E-01	5.20E-01
	SP_PIR_KEYWORDS	ATP	6	1.40E-01	5.90E-01
**Annotation Cluster 31**	**Enrichment Score: 1.09**		**Count**	**P_Value**	**Benjamini**
	GOTERM_BP_FAT	muscle organ development	8	2.60E-02	3.40E-01
	GOTERM_BP_FAT	muscle tissue development	5	9.20E-02	6.30E-01
	GOTERM_BP_FAT	striated muscle tissue development	4	2.20E-01	8.40E-01

**Annotation Cluster 32**	**Enrichment Score: 1.09**		**Count**	**P_Value**	**Benjamini**
	GOTERM_CC_FAT	platelet alpha granule	5	7.90E-03	1.30E-01
	GOTERM_CC_FAT	platelet alpha granule lumen	4	2.00E-02	2.60E-01
	GOTERM_CC_FAT	cytoplasmic membrane-bounded vesicle lumen	4	2.40E-02	2.90E-01
	GOTERM_CC_FAT	vesicle lumen	4	2.70E-02	2.80E-01
	GOTERM_CC_FAT	secretory granule	6	1.10E-01	6.80E-01
	GOTERM_CC_FAT	cytoplasmic vesicle part	6	1.20E-01	7.00E-01
	GOTERM_CC_FAT	cytoplasmic membrane-bounded vesicle	11	2.50E-01	8.70E-01
	GOTERM_CC_FAT	membrane-bounded vesicle	11	2.80E-01	8.90E-01
	GOTERM_CC_FAT	vesicle	12	3.40E-01	9.20E-01
	GOTERM_CC_FAT	cytoplasmic vesicle	11	4.20E-01	9.40E-01
**Annotation Cluster 33**	**Enrichment Score: 1.06**		**Count**	**P_Value**	**Benjamini**
	SP_PIR_KEYWORDS	laminin egf-like domain	3	4.80E-02	3.20E-01
	INTERPRO	EGF-like, laminin	3	9.40E-02	8.00E-01
	SMART	EGF_Lam	3	1.40E-01	9.50E-01
**Annotation Cluster 34**	**Enrichment Score: 1.06**		**Count**	**P_Value**	**Benjamini**
	GOTERM_MF_FAT	glycosaminoglycan binding	8	2.60E-03	2.20E-01
	GOTERM_MF_FAT	polysaccharide binding	8	4.40E-03	2.90E-01
	GOTERM_MF_FAT	pattern binding	8	4.40E-03	2.90E-01
	GOTERM_MF_FAT	peptidoglycan binding	3	5.90E-03	2.80E-01
	GOTERM_MF_FAT	carbohydrate binding	12	7.50E-03	3.00E-01
	GOTERM_BP_FAT	positive regulation of tumor necrosis factor production	3	1.70E-02	2.70E-01
	GOTERM_BP_FAT	regulation of tumor necrosis factor production	3	6.70E-02	5.50E-01
	GOTERM_BP_FAT	positive regulation of cytokine production	4	1.30E-01	7.00E-01
	GOTERM_BP_FAT	positive regulation of MAPKKK cascade	3	1.40E-01	7.20E-01
	UP_SEQ_FEATURE	repeat:LRR 4	6	1.80E-01	9.80E-01
	UP_SEQ_FEATURE	repeat:LRR 6	5	2.30E-01	9.90E-01
	GOTERM_BP_FAT	response to bacterium	5	2.70E-01	8.80E-01
	UP_SEQ_FEATURE	repeat:LRR 3	6	2.80E-01	1.00E+00
	INTERPRO	Leucine-rich repeat	5	2.80E-01	9.70E-01
	UP_SEQ_FEATURE	repeat:LRR 5	5	2.90E-01	1.00E+00
	UP_SEQ_FEATURE	repeat:LRR 1	6	3.20E-01	1.00E+00
	SP_PIR_KEYWORDS	leucine-rich repeat	6	3.30E-01	7.80E-01
	UP_SEQ_FEATURE	repeat:LRR 2	6	3.30E-01	1.00E+00
	UP_SEQ_FEATURE	repeat:LRR 9	3	4.70E-01	1.00E+00
	UP_SEQ_FEATURE	repeat:LRR 8	3	5.30E-01	1.00E+00
	UP_SEQ_FEATURE	repeat:LRR 7	3	6.20E-01	1.00E+00
**Annotation Cluster 35**	**Enrichment Score: 1.06**		**Count**	**P_Value**	**Benjamini**
	INTERPRO	EGF-like region, conserved site	11	4.10E-03	3.50E-01
	SP_PIR_KEYWORDS	egf-like domain	7	5.40E-02	3.40E-01
	INTERPRO	EGF	5	7.70E-02	7.80E-01
	INTERPRO	EGF-like, type 3	6	9.90E-02	8.10E-01
	INTERPRO	EGF-like	6	1.10E-01	8.30E-01
	INTERPRO	EGF-type aspartate/asparagine hydroxylation conserved site	4	1.20E-01	8.50E-01
	UP_SEQ_FEATURE	domain:EGF-like 2; calcium-binding	3	1.40E-01	9.80E-01
	SMART	EGF	6	2.30E-01	9.50E-01
	UP_SEQ_FEATURE	domain:EGF-like 1	3	4.10E-01	1.00E+00
**Annotation Cluster 36**	**Enrichment Score: 1.04**		**Count**	**P_Value**	**Benjamini**
	GOTERM_MF_FAT	identical protein binding	15	4.50E-02	5.50E-01
	GOTERM_MF_FAT	protein dimerization activity	12	1.10E-01	7.20E-01
	GOTERM_MF_FAT	protein homodimerization activity	8	1.60E-01	7.60E-01
**Annotation Cluster 37**	**Enrichment Score: 1.04**		**Count**	**P_Value**	**Benjamini**
	GOTERM_BP_FAT	activation of immune response	8	3.00E-04	2.50E-02
	GOTERM_BP_FAT	innate immune response	6	4.10E-02	4.50E-01
	SP_PIR_KEYWORDS	innate immunity	4	6.30E-02	3.70E-01
	GOTERM_BP_FAT	humoral immune response	4	9.40E-02	6.40E-01
	GOTERM_BP_FAT	complement activation	3	1.10E-01	6.80E-01
	GOTERM_BP_FAT	activation of plasma proteins involved in acute inflammatory response	3	1.20E-01	6.80E-01
	KEGG_PATHWAY	Complement and coagulation cascades	4	1.30E-01	4.40E-01

	GOTERM_BP_FAT	protein maturation by peptide bond cleavage		3	3.30E-01	9.20E-01
	GOTERM_BP_FAT	acute inflammatory response		3	3.90E-01	9.50E-01
	GOTERM_BP_FAT	protein processing		3	4.50E-01	9.70E-01
	GOTERM_BP_FAT	protein maturation		3	5.00E-01	9.80E-01
**Annotation Cluster 38**	**Enrichment Score: 1.01**		**Count**		**P_Value**	**Benjamini**
	INTERPRO	von Willebrand factor, type A		4	7.80E-02	7.70E-01
	UP_SEQ_FEATURE	short sequence motif:Cell attachment site		4	8.60E-02	9.30E-01
	SMART	VWA		4	1.40E-01	9.70E-01
**Annotation Cluster 39**	**Enrichment Score: 0.98**		**Count**		**P_Value**	**Benjamini**
	UP_SEQ_FEATURE	domain:CUB 1		3	6.70E-02	9.10E-01
	UP_SEQ_FEATURE	domain:CUB 2		3	6.70E-02	9.10E-01
	INTERPRO	CUB		3	1.40E-01	8.60E-01
	SMART	CUB		3	2.00E-01	9.40E-01
