# Supplementary figures and images for: Intrarenal activation of adaptive immune effectors is associated with tubular damage and impaired renal function in lupus nephritis
Source: Ann Rheum Dis. 2018 Jul 31;77(12):1782–9. doi: 10.1136/annrheumdis-2018-213485 (PMC6241616; doi:10.1136/annrheumdis-2018-213485)

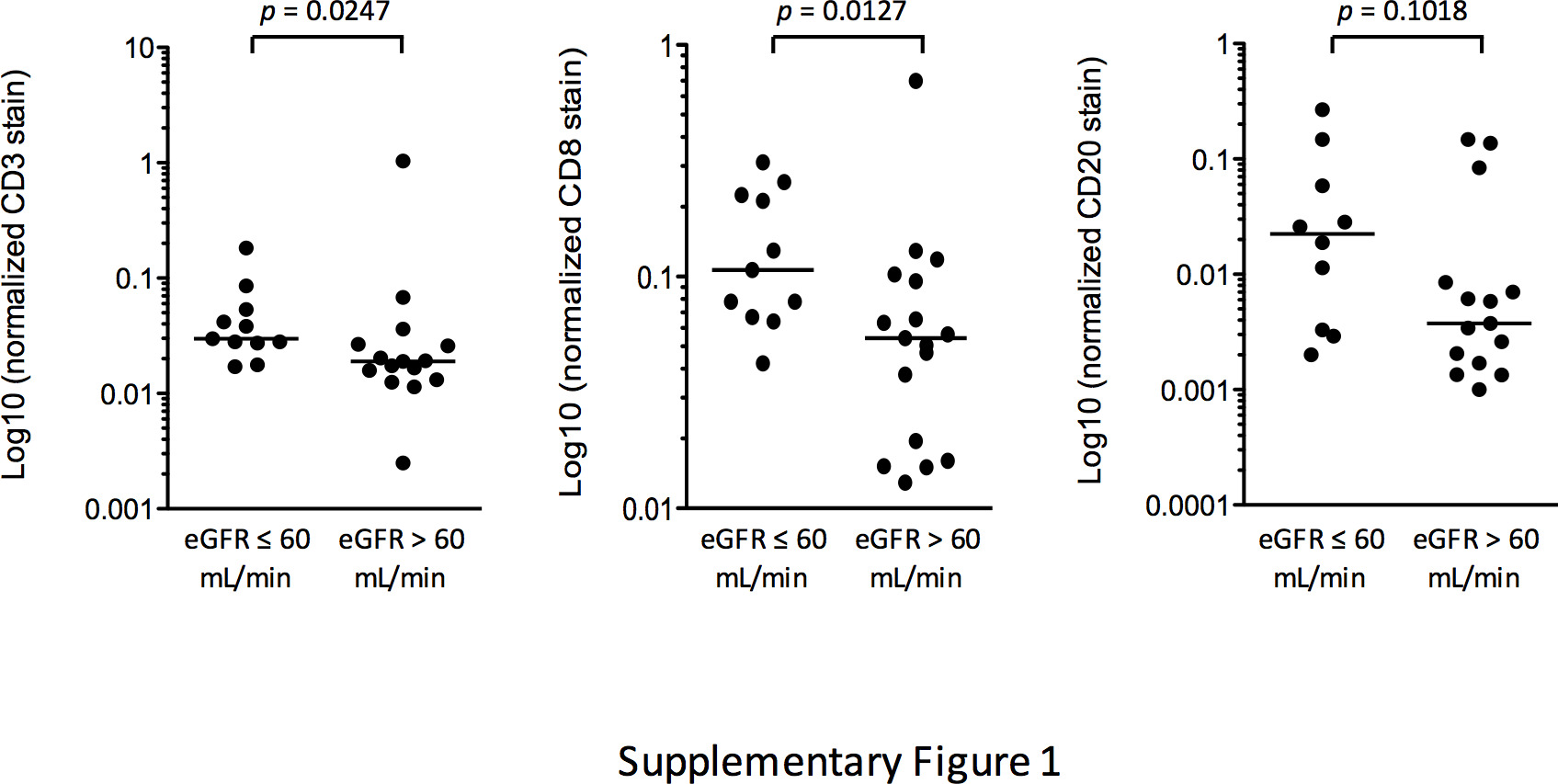

Supplement: Supplementary file 4 [file annrheumdis-2018-213485supp004.jpg]

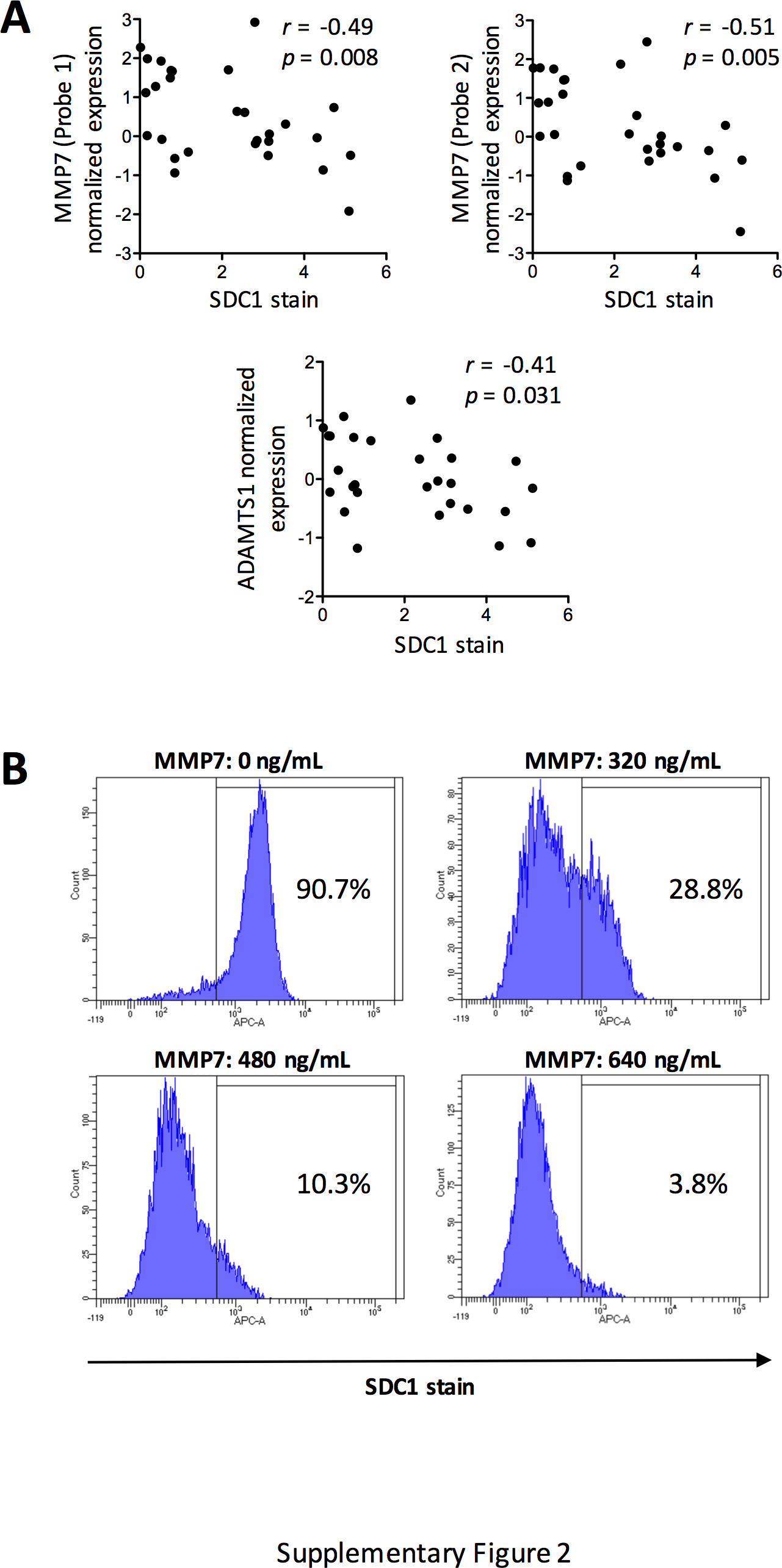

Supplement: Supplementary file 5 [file annrheumdis-2018-213485supp005.jpg]
