# Supplementary material for: Intrarenal activation of adaptive immune effectors is associated with tubular damage and impaired renal function in lupus nephritis
Source: Ann Rheum Dis. 2018 Jul 31;77(12):1782–9. doi: 10.1136/annrheumdis-2018-213485 (PMC6241616; doi:10.1136/annrheumdis-2018-213485)
Supplement: Supplementary file 7 [file annrheumdis-2018-213485supp007.pdf]

Supplementary Table 4 : Transcripts and DAVID pathways correlating with the expression of CD8a in lupus kidney biopsies

Positive correlation

Transcripts

Identifier	Gene Symbol	Correlation coefficient with CD8a
ILMN_2353732	CD8A	1
ILMN_1768482	CD8A	0.979686707
ILMN_1779324	GZMA	0.873333729
ILMN_2393765	IGLL1	0.851582892
ILMN_2122374	FAM49B	0.847212542
ILMN_1763452	EVI2B	0.843460941
ILMN_2083066	IGLL3P	0.838996022
ILMN_1716651	RUNX2	0.831882364
ILMN_1769129	CCL19	0.818224657
ILMN_1754507	IRF4	0.810884807
ILMN_2105441	IGJ	0.80713872
ILMN_1659075	HLA-DOA	0.803521768
ILMN_2208413	ARHGAP15	0.802049622
ILMN_2325837	CD3D	0.788912749
ILMN_1811049	POU2AF1	0.781591378
ILMN_3240375	abParts	0.779415662
ILMN_1691341	IL7R	0.774655206
ILMN_2061043	CD48	0.7745978
ILMN_1667081	CCND2	0.758231418
ILMN_2254635	ITGAX	0.756063553
ILMN_1886655	TCRVB	0.754105378
ILMN_1710734	GZMK	0.752051991
ILMN_1797875	ALOX5AP	0.749564057
ILMN_2208903	CD52	0.749548341
ILMN_1680274	abParts	0.742033438
ILMN_2261416	CD3D	0.73928993
ILMN_1739508	abParts	0.73870528
ILMN_1652199	abParts	0.738079032
ILMN_2098126	CCL5	0.736481022
ILMN_1739794	CD3E	0.732300996
ILMN_1673357	SLA2	0.73088311
ILMN_1684887	SAMSN1	0.729726225
ILMN_2347693	NCOA3	0.729002273
ILMN_1790529	LUM	0.727037355
ILMN_2246328	PTPN22	0.725329263
ILMN_3232921	CCR2	0.721539608
ILMN_2157441	HLA-DRA	0.721217789
ILMN_1682993	NKG7	0.719291182
ILMN_1715647	VANGL2	0.714677389
ILMN_1697554	SASH3	0.714618446
ILMN_1674228	abParts	0.712983949
ILMN_1676924	CD247	0.712958975
ILMN_1686116	THBS1	0.711645157
ILMN_1662843	CD53	0.711509893
ILMN_1713751	ADAM19	0.710863577
ILMN_1796962	PPP3R1	0.710367708
ILMN_1706539	KDM3B	0.707968869
ILMN_2093343	PLAC8	0.705045803
ILMN_1690907	CCR6	0.703261207
ILMN_1747775	STX2	0.701625717
ILMN_1806908	PRKCB	0.701297604
ILMN_1785272	COL1A2	0.700259921
ILMN_1773352	CCL5	0.700226821
ILMN_1797362	LIMK1	0.699833127
ILMN_1750805	ARHGAP30	0.699046258
ILMN_1807042	MARCKS	0.697983994
ILMN_1801504	RUNX1	0.697412576
ILMN_1743646	VASP	0.697313722
ILMN_1677505	CCL21	0.695091586
ILMN_2114568	GBP5	0.693020938
ILMN_1748283	PIM2	0.692842392
ILMN_1652631	GLIPR2	0.692781989
ILMN_1672611	CDH11	0.691349791
ILMN_1682312	CYBB	0.690837533
ILMN_1665943	MAP4K1	0.690771136
ILMN_1688959	CD27	0.690540291
ILMN_1794386	IL2RG	0.689321964
ILMN_1815734	FCHSD2	0.689045951
ILMN_2307861	COL6A3	0.688508441
ILMN_1699574	NRP1	0.686774604
ILMN_3240321	AEN	0.686142625
ILMN_1678143	ARHGDIB	0.685896814
ILMN_2309848	FXYD5	0.683177069
ILMN_2352303	RASSF2	0.682002806
ILMN_1699931	HCST	0.679420315
ILMN_1752520	SLFN11	0.679115884
ILMN_1746090	STT3A	0.678374943
ILMN_1761788	MOXD1	0.678117121
ILMN_1810559	RHOQ	0.677993303
ILMN_1800638	NAPOR-1	0.676109361
ILMN_1695311	HLA-DMA	0.675203098
ILMN_2340217	PTPRC	0.673832701
ILMN_1768284	P2RY8	0.673231913
ILMN_1704286	FXYD5	0.670757359
ILMN_1761733	HLA-DMB	0.668004467
ILMN_3299520	PRKCB	0.667521032
ILMN_1812392	TMSB10	0.666932606
ILMN_1766408	CBFB	0.666699522
ILMN_1752526	RNF144B	0.666590948

Pathways

**Annotation Cluster 1**	**Enrichment Score: 13.42**		**Count**	**P_Value**	**Benjamini**
	SP_PIR_KEYWORDS	signal	319	2.30E-19	4.40E-17
	UP_SEQ_FEATURE	signal peptide	319	5.30E-19	1.70E-15
	SP_PIR_KEYWORDS	disulfide bond	278	1.50E-14	2.10E-12
	UP_SEQ_FEATURE	disulfide bond	268	1.10E-13	1.70E-10
	SP_PIR_KEYWORDS	glycoprotein	357	2.60E-10	2.20E-08
	UP_SEQ_FEATURE	glycosylation site:N-linked (GlcNAc...)	332	6.40E-08	3.40E-05
**Annotation Cluster 2**	**Enrichment Score: 10.81**		**Count**	**P_Value**	**Benjamini**
	GOTERM_BP_FAT	defense response	97	1.60E-14	1.10E-11
	GOTERM_BP_FAT	inflammatory response	57	1.40E-10	5.30E-08
	GOTERM_BP_FAT	response to wounding	76	1.60E-09	3.70E-07
**Annotation Cluster 3**	**Enrichment Score: 9.07**		**Count**	**P_Value**	**Benjamini**
	SP_PIR_KEYWORDS	signal	319	2.30E-19	4.40E-17
	UP_SEQ_FEATURE	signal peptide	319	5.30E-19	1.70E-15
	SP_PIR_KEYWORDS	Secreted	149	6.00E-06	2.00E-04
	GOTERM_CC_FAT	extracellular region part	103	6.50E-06	8.60E-04
	GOTERM_CC_FAT	extracellular region	180	1.30E-04	5.70E-03
	GOTERM_CC_FAT	extracellular space	71	5.90E-04	1.80E-02
**Annotation Cluster 4**	**Enrichment Score: 9.01**		**Count**	**P_Value**	**Benjamini**
	GOTERM_BP_FAT	cell activation	61	4.80E-15	5.30E-12
	GOTERM_BP_FAT	leukocyte activation	55	6.80E-15	5.60E-12
	GOTERM_BP_FAT	lymphocyte activation	47	1.80E-13	1.00E-10
	GOTERM_BP_FAT	T cell activation	35	2.60E-12	1.20E-09
	GOTERM_BP_FAT	leukocyte differentiation	29	6.10E-08	8.80E-06
	GOTERM_BP_FAT	hemopoiesis	40	2.90E-07	3.40E-05
	GOTERM_BP_FAT	lymphocyte differentiation	24	4.00E-07	4.60E-05
	GOTERM_BP_FAT	hemopoietic or lymphoid organ development	42	5.20E-07	5.70E-05
	GOTERM_BP_FAT	immune system development	42	2.50E-06	2.10E-04
	GOTERM_BP_FAT	T cell differentiation	17	6.10E-06	4.60E-04
**Annotation Cluster 5**	**Enrichment Score: 7.57**		**Count**	**P_Value**	**Benjamini**
	GOTERM_BP_FAT	positive regulation of immune system process	57	1.30E-16	1.80E-13
	GOTERM_BP_FAT	positive regulation of immune response	37	8.60E-12	3.60E-09
	GOTERM_BP_FAT	positive regulation of response to stimulus	46	3.40E-10	1.10E-07
	GOTERM_BP_FAT	activation of immune response	27	5.50E-10	1.60E-07
	GOTERM_BP_FAT	immune response-regulating signal transduction	20	2.70E-09	5.60E-07
	GOTERM_BP_FAT	immune effector process	31	6.60E-09	1.30E-06
	GOTERM_BP_FAT	immune response-regulating cell surface receptor signaling pathway	16	5.80E-08	8.80E-06
	GOTERM_BP_FAT	immune response-activating signal transduction	17	2.30E-07	2.80E-05
	GOTERM_BP_FAT	immune response-activating cell surface receptor signaling pathway	13	7.50E-06	5.40E-04
	GOTERM_BP_FAT	antigen receptor-mediated signaling pathway	11	5.00E-05	2.80E-03
	GOTERM_BP_FAT	B cell receptor signaling pathway	5	2.10E-03	6.60E-02
	GOTERM_BP_FAT	T cell receptor signaling pathway	7	3.90E-03	1.00E-01
**Annotation Cluster 6**	**Enrichment Score: 7.14**		**Count**	**P_Value**	**Benjamini**
	INTERPRO	Immunoglobulin-like fold	79	6.80E-11	9.80E-08
	SP_PIR_KEYWORDS	Immunoglobulin domain	68	1.10E-10	1.00E-08
	INTERPRO	Immunoglobulin-like	72	4.10E-10	3.00E-07
	INTERPRO	Immunoglobulin subtype	46	2.00E-06	5.70E-04
	SMART	IG	46	1.40E-04	1.30E-02
	INTERPRO	Immunoglobulin V-set	33	1.80E-04	2.90E-02
**Annotation Cluster 7**	**Enrichment Score: 5.52**		**Count**	**P_Value**	**Benjamini**
	UP_SEQ_FEATURE	domain:Ig-like C1-type	20	3.70E-13	4.00E-10
	KEGG_PATHWAY	Viral myocarditis	26	9.60E-11	1.40E-08
	KEGG_PATHWAY	Cell adhesion molecules (CAMs)	36	1.50E-10	1.10E-08
	KEGG_PATHWAY	Allograft rejection	18	4.80E-10	2.40E-08
	GOTERM_BP_FAT	antigen processing and presentation	25	9.30E-10	2.60E-07
	KEGG_PATHWAY	Graft-versus-host disease	18	2.30E-09	8.40E-08
	INTERPRO	Immunoglobulin C1-set	21	6.60E-09	3.20E-06
	GOTERM_BP_FAT	antigen processing and presentation of peptide antigen	14	1.10E-08	1.80E-06
	SP_PIR_KEYWORDS	heterodimer	25	1.20E-08	8.40E-07
	INTERPRO	Immunoglobulin/major histocompatibility complex, conserved site	23	2.50E-08	9.10E-06
	UP_SEQ_FEATURE	region of interest:Connecting peptide	15	2.70E-08	1.80E-05
	KEGG_PATHWAY	Autoimmune thyroid disease	19	4.20E-08	1.20E-06
	KEGG_PATHWAY	Type I diabetes mellitus	17	6.90E-08	1.50E-06
	UP_SEQ_FEATURE	region of interest:Alpha-2	11	8.40E-08	3.40E-05
	UP_SEQ_FEATURE	region of interest:Alpha-1	11	8.40E-08	3.40E-05
	SMART	IGc1	21	1.20E-07	3.40E-05
	GOTERM_CC_FAT	MHC protein complex	18	1.70E-07	4.40E-05
	KEGG_PATHWAY	Systemic lupus erythematosus	26	2.00E-07	3.30E-06
	KEGG_PATHWAY	Antigen processing and presentation	23	4.40E-07	6.50E-06
	GOTERM_BP_FAT	antigen processing and presentation of peptide or polysaccharide antigen via MHC class II	13	1.00E-06	9.50E-05
	KEGG_PATHWAY	Asthma	13	1.10E-06	1.50E-05
	GOTERM_MF_FAT	MHC class II receptor activity	10	1.20E-06	1.10E-03
	SP_PIR_KEYWORDS	mhc ii	12	1.30E-06	4.80E-05
	GOTERM_MF_FAT	MHC protein binding	11	2.00E-06	9.50E-04
	GOTERM_MF_FAT	MHC class I protein binding	9	2.90E-06	9.00E-04
	KEGG_PATHWAY	Intestinal immune network for IgA production	16	4.50E-06	5.50E-05
	SP_PIR_KEYWORDS	mhc i	7	9.20E-06	2.80E-04
	INTERPRO	MHC class II, alpha/beta chain, N-terminal	9	4.50E-05	9.10E-03
	PIR_SUPERFAMILY	PIRSF001991:class II histocompatibility antigen	10	6.50E-05	3.50E-02
	GOTERM_BP_FAT	antigen processing and presentation of peptide antigen via MHC class I	8	7.70E-05	3.90E-03
	UP_SEQ_FEATURE	region of interest:Alpha-3	6	9.30E-05	2.50E-02
	GOTERM_CC_FAT	MHC class II protein complex	10	9.90E-05	4.80E-03
	GOTERM_CC_FAT	MHC class I peptide loading complex	6	1.60E-04	6.40E-03
	PIR_SUPERFAMILY	PIRSF001990:class I histocompatibility antigen	6	2.20E-04	5.80E-02
	GOTERM_MF_FAT	MHC class I receptor activity	7	5.50E-04	3.60E-02
	GOTERM_CC_FAT	TAP complex	5	6.70E-04	2.00E-02
	GOTERM_CC_FAT	MHC class I protein complex	8	2.40E-03	6.00E-02
	UP_SEQ_FEATURE	region of interest:Beta-2	5	4.90E-03	4.20E-01
	UP_SEQ_FEATURE	region of interest:Beta-1	5	4.90E-03	4.20E-01
	INTERPRO	MHC class II, alpha chain, N-terminal	5	5.80E-03	3.60E-01

Negative correlation

Transcripts

Identifier	Gene Symbol	Correlation coefficient with CD8a
ILMN_1700771	PRUNE2	-0.400058295
ILMN_1721549	PLA2G1B	-0.400328462
ILMN_1808272	KCNJ10	-0.400468542
ILMN_1796136	RAB37	-0.40052267
ILMN_1690939	TYMP	-0.400576232
ILMN_1805534	PPP4R1L	-0.400582979
ILMN_1665437	CYP2E1	-0.400598437
ILMN_1731948	PCK1	-0.400774781
ILMN_1803824	ZDHHC9	-0.400972943
ILMN_1658110	C18orf19	-0.400993489
ILMN_1778611	GBAS	-0.401005236
ILMN_1712308	FSTL4	-0.401051404
ILMN_2156971	PRY	-0.401104574
ILMN_1776666	GRIK3	-0.401108313
ILMN_1670255	MAGEA9B	-0.401142424
ILMN_1674349	HRASLS5	-0.401239572
ILMN_1743594	UBXN7	-0.401307884
ILMN_1736077	LIAS	-0.401321098
ILMN_2150284	RNPC3	-0.401378913
ILMN_1659365	TEKT4P2	-0.40138806
ILMN_1877996	AW195018	-0.401439898
ILMN_1747962	UTS2R	-0.401679027
ILMN_1737025	PLCL2	-0.401835395
ILMN_1668270	ZDHHC18	-0.401872968
ILMN_2044085	RQCD1	-0.401906623
ILMN_1756358	FBXO36	-0.401923292
ILMN_2211780	SLC25A4	-0.40219111
ILMN_2350607	C20orf7	-0.402299971
ILMN_1743635	ALG14	-0.402300471
ILMN_1744748	HM031116	-0.402390957
ILMN_1725145	POLA1	-0.402468501
ILMN_2138745	C14orf37	-0.402678025
ILMN_1663640	MAOA	-0.402779011
ILMN_1784283	USH1C	-0.402780174
ILMN_1762972	CHD9	-0.402794069
ILMN_3178050	SPECC1	-0.40282738
ILMN_1686259	PRODH2	-0.402830211
ILMN_1812600	NA	-0.402957355
ILMN_1797391	SLC22A7	-0.40302308
ILMN_1710064	SDR16C5	-0.403059961
ILMN_2380999	RECQL	-0.403101358
ILMN_1685636	KCNN2	-0.403178531
ILMN_1674021	LOC401321	-0.403218171
ILMN_3243863	DB462629	-0.40324045
ILMN_1780625	HR	-0.403289759
ILMN_1756623	NT5C1B	-0.403342922
ILMN_3261022	NA	-0.403345903
ILMN_1753286	MYO19	-0.403359388
ILMN_1685796	CSDE1	-0.403379863
ILMN_1813341	PTGFR	-0.403664001
ILMN_1827779	AK023464	-0.403680903
ILMN_3236330	DQ581928	-0.403826146
ILMN_3274344	NA	-0.403833144
ILMN_1669572	RNF126	-0.403855056
ILMN_1796969	POU4F3	-0.403864332
ILMN_3242710	LOC157627	-0.403901277
ILMN_2187830	CCNH	-0.404182473
ILMN_1786498	STARD13	-0.40418898
ILMN_3212469	LOC401561	-0.404269201
ILMN_3234570	CECR4	-0.404302393
ILMN_2207562	AP1AR	-0.404325109
ILMN_2150019	SUCLA2	-0.404391617
ILMN_1744295	CDKN2A	-0.404421822
ILMN_3244220	DEFB107B	-0.404479775
ILMN_1759448	CPZ	-0.404526686
ILMN_2163306	FAM120A	-0.404681788
ILMN_1781381	AK123658	-0.4047257
ILMN_3238358	SCARNA22	-0.404878746
ILMN_1755354	YBX2	-0.404926479
ILMN_1676055	AK055272	-0.404971677
ILMN_2145781	RBMY2FP	-0.405033419
ILMN_1778104	ACADM	-0.40540081
ILMN_3232128	FLJ37786	-0.405402756
ILMN_1727778	NTNG1	-0.405431845
ILMN_1718135	PRAMEF11	-0.405529656
ILMN_2404629	EFTUD1	-0.405552054
ILMN_3237473	CBWD1	-0.405741086
ILMN_1655537	ING1	-0.405766784
ILMN_3251375	WBP11P1	-0.40590988
ILMN_1661363	AF064105	-0.405970404
ILMN_1724166	OR56B1	-0.406209309
ILMN_3246534	TSG1	-0.406222474
ILMN_1798712	USP4	-0.406239293
ILMN_1673752	C1orf91	-0.406371095
ILMN_3248440	SNORA81	-0.406389633
ILMN_3309059	MIR93	-0.406433778
ILMN_1802192	C10orf99	-0.406439389
ILMN_1652379	SUCLG2	-0.4064482
ILMN_2184556	SLC4A4	-0.406451037

Pathways

**Annotation Cluster 1**	**Enrichment Score: 18.91**		**Count**	**P_Value**	**Benjamini**
	GOTERM_BP_FAT	oxidation reduction	105	9.60E-24	2.90E-20
	SP_PIR_KEYWORDS	oxidoreductase	97	1.60E-23	5.00E-21
	GOTERM_MF_FAT	electron carrier activity	43	1.20E-11	6.40E-09
**Annotation Cluster 2**	**Enrichment Score: 10.74**		**Count**	**P_Value**	**Benjamini**
	SP_PIR_KEYWORDS	mitochondrion	130	3.10E-27	1.90E-24
	GOTERM_CC_FAT	mitochondrion	158	7.70E-26	3.80E-23
	SP_PIR_KEYWORDS	transit peptide	85	1.30E-21	2.50E-19
	UP_SEQ_FEATURE	transit peptide:Mitochondrion	83	7.00E-21	1.90E-17
	GOTERM_CC_FAT	mitochondrial part	100	1.20E-20	3.00E-18
	GOTERM_CC_FAT	mitochondrial envelope	68	2.90E-13	4.70E-11
	GOTERM_CC_FAT	mitochondrial inner membrane	55	1.20E-12	1.50E-10
	GOTERM_CC_FAT	mitochondrial membrane	63	4.80E-12	4.70E-10
	GOTERM_CC_FAT	organelle inner membrane	56	7.10E-12	5.80E-10
	SP_PIR_KEYWORDS	mitochondrion inner membrane	34	7.20E-09	8.70E-07
	GOTERM_CC_FAT	organelle envelope	75	2.00E-08	1.20E-06
	GOTERM_CC_FAT	envelope	75	2.30E-08	1.30E-06
	GOTERM_CC_FAT	mitochondrial membrane part	26	1.20E-07	6.20E-06
	KEGG_PATHWAY	Oxidative phosphorylation	26	2.70E-06	8.30E-05
	GOTERM_CC_FAT	organelle membrane	101	2.90E-05	1.30E-03
	GOTERM_CC_FAT	mitochondrial respiratory chain	14	1.10E-04	3.90E-03
	GOTERM_CC_FAT	respiratory chain	15	1.60E-04	5.00E-03
	KEGG_PATHWAY	Alzheimer's disease	23	2.20E-03	1.90E-02
	KEGG_PATHWAY	Parkinson's disease	19	3.40E-03	2.60E-02
	KEGG_PATHWAY	Huntington's disease	22	1.40E-02	9.30E-02
**Annotation Cluster 3**	**Enrichment Score: 9.05**		**Count**	**P_Value**	**Benjamini**
	GOTERM_BP_FAT	carboxylic acid catabolic process	29	1.10E-11	1.10E-08
	GOTERM_BP_FAT	organic acid catabolic process	29	1.10E-11	1.10E-08
	GOTERM_BP_FAT	amine catabolic process	20	4.10E-08	2.50E-05
	GOTERM_BP_FAT	cellular amino acid catabolic process	18	1.40E-07	5.30E-05
**Annotation Cluster 4**	**Enrichment Score: 6.14**		**Count**	**P_Value**	**Benjamini**
	KEGG_PATHWAY	Propanoate metabolism	15	8.40E-09	1.50E-06
	KEGG_PATHWAY	Valine, leucine and isoleucine degradation	16	1.40E-07	8.30E-06
	KEGG_PATHWAY	Fatty acid metabolism	11	3.30E-04	3.80E-03
**Annotation Cluster 5**	**Enrichment Score: 5.96**		**Count**	**P_Value**	**Benjamini**
	GOTERM_MF_FAT	carboxylic acid binding	28	7.60E-08	2.10E-05
	GOTERM_MF_FAT	monocarboxylic acid binding	16	3.30E-07	7.10E-05
	GOTERM_MF_FAT	fatty acid binding	11	5.10E-05	7.90E-03
**Annotation Cluster 6**	**Enrichment Score: 4.52**		**Count**	**P_Value**	**Benjamini**
	UP_SEQ_FEATURE	short sequence motif:Microbody targeting signal	12	1.00E-05	9.20E-03
	SP_PIR_KEYWORDS	peroxisome	17	3.20E-05	2.20E-03
	GOTERM_CC_FAT	microbody	19	4.90E-05	2.00E-03
	GOTERM_CC_FAT	peroxisome	19	4.90E-05	2.00E-03
**Annotation Cluster 7**	**Enrichment Score: 4.16**		**Count**	**P_Value**	**Benjamini**
	GOTERM_BP_FAT	coenzyme metabolic process	27	3.80E-07	1.30E-04
	GOTERM_BP_FAT	cofactor metabolic process	30	1.50E-06	4.40E-04
	GOTERM_BP_FAT	coenzyme biosynthetic process	11	5.00E-03	2.20E-01
	GOTERM_BP_FAT	cofactor biosynthetic process	13	8.10E-03	2.80E-01
**Annotation Cluster 8**	**Enrichment Score: 4.12**		**Count**	**P_Value**	**Benjamini**
	GOTERM_BP_FAT	lipid catabolic process	26	1.30E-05	2.60E-03
	GOTERM_BP_FAT	cellular lipid catabolic process	16	1.80E-05	3.30E-03
	GOTERM_BP_FAT	fatty acid catabolic process	11	2.00E-05	3.50E-03
	GOTERM_BP_FAT	lipid oxidation	11	4.30E-05	6.40E-03
	GOTERM_BP_FAT	fatty acid oxidation	11	4.30E-05	6.40E-03
	GOTERM_BP_FAT	lipid modification	13	4.20E-04	3.60E-02
	GOTERM_BP_FAT	fatty acid beta-oxidation	7	4.00E-03	1.90E-01
**Annotation Cluster 9**	**Enrichment Score: 4.11**		**Count**	**P_Value**	**Benjamini**
	GOTERM_BP_FAT	cellular respiration	22	7.40E-08	3.20E-05
	GOTERM_BP_FAT	aerobic respiration	12	2.00E-06	5.50E-04
	KEGG_PATHWAY	Citrate cycle (TCA cycle)	11	3.10E-05	6.20E-04
	GOTERM_BP_FAT	cofactor catabolic process	10	3.50E-05	5.60E-03
	GOTERM_BP_FAT	coenzyme catabolic process	9	6.10E-05	8.30E-03
	GOTERM_BP_FAT	tricarboxylic acid cycle	8	1.90E-04	2.30E-02
	GOTERM_BP_FAT	acetyl-CoA catabolic process	8	1.90E-04	2.30E-02
	GOTERM_BP_FAT	acetyl-CoA metabolic process	9	2.40E-04	2.60E-02
	SP_PIR_KEYWORDS	tricarboxylic acid cycle	6	5.00E-03	1.20E-01
	GOTERM_MF_FAT	succinate dehydrogenase activity	3	1.90E-02	4.30E-01
**Annotation Cluster 10**	**Enrichment Score: 3.6**		**Count**	**P_Value**	**Benjamini**
	KEGG_PATHWAY	Metabolism of xenobiotics by cytochrome P450	17	2.20E-06	8.10E-05
	KEGG_PATHWAY	Drug metabolism	17	3.60E-06	9.30E-05
	KEGG_PATHWAY	Ascorbate and aldarate metabolism	9	7.70E-06	1.80E-04
	INTERPRO	UDP-glucuronosyl/UDP-glucosyltransferase	7	4.30E-05	3.30E-02
	KEGG_PATHWAY	Retinol metabolism	14	6.60E-05	1.10E-03
	SP_PIR_KEYWORDS	microsome	18	7.20E-05	4.00E-03
	PIR_SUPERFAMILY	PIRSF005678:glucuronosyltransferase	7	8.50E-05	4.20E-02
	KEGG_PATHWAY	Starch and sucrose metabolism	12	1.10E-04	1.50E-03
	KEGG_PATHWAY	Pentose and glucuronate interconversions	8	1.30E-04	1.70E-03
	KEGG_PATHWAY	Drug metabolism	12	1.30E-04	1.60E-03
	KEGG_PATHWAY	Steroid hormone biosynthesis	11	1.10E-03	1.10E-02
	GOTERM_MF_FAT	glucuronosyltransferase activity	7	1.40E-03	1.10E-01
	KEGG_PATHWAY	Porphyrin and chlorophyll metabolism	9	1.60E-03	1.60E-02
	KEGG_PATHWAY	Androgen and estrogen metabolism	8	1.30E-02	8.90E-02
	GOTERM_BP_FAT	response to xenobiotic stimulus	5	3.70E-02	5.70E-01
	GOTERM_BP_FAT	xenobiotic metabolic process	4	9.90E-02	8.30E-01
**Annotation Cluster 11**	**Enrichment Score: 3.6**		**Count**	**P_Value**	**Benjamini**
	INTERPRO	NAD(P)-binding domain	24	1.50E-05	2.40E-02
	PIR_SUPERFAMILY	PIRSF000092:short-chain dehydrogenase	7	8.50E-05	4.20E-02
	UP_SEQ_FEATURE	nucleotide phosphate-binding region:NADP	13	5.90E-04	2.30E-01
	INTERPRO	Glucose/ribitol dehydrogenase	11	7.60E-04	3.30E-01
	INTERPRO	Short-chain dehydrogenase/reductase SDR	11	1.80E-03	3.80E-01

ILMN_1770673	AKNA	0.663599803
ILMN_1684040	THEMIS	0.662849327
ILMN_1695640	PTPN22	0.662651533
ILMN_1666594	IRF8	0.662418387
ILMN_2400947	CELF2	0.661837182
ILMN_2376204	LTB	0.660402766
ILMN_3214389	HLA-DQB1	0.657928071
ILMN_1709795	RAC2	0.657747761
ILMN_2369018	EVI2A	0.654152271
ILMN_1737015	RPL39	0.653659552
ILMN_1686623	CSF1R	0.6532817
ILMN_2175912	ITGB2	0.652830865
ILMN_1796409	C1QB	0.65225815
ILMN_2087692	CYBRD1	0.649647065
ILMN_1786176	CD37	0.649410553
ILMN_1725992	RAPGEF6	0.648411039
ILMN_2413808	CD53	0.647552799
ILMN_2376205	LTB	0.646931041
ILMN_1791447	CXCL12	0.646513676
ILMN_1794677	TMC6	0.646193931
ILMN_1659800	BCL11A	0.645472255
ILMN_1734740	GPR65	0.64483016
ILMN_2399264	sept-06	0.644739871
ILMN_1807825	LY86	0.644540555
ILMN_1804601	DKFZp686O16217	0.64435421
ILMN_1790533	PHACTR2	0.643785252
ILMN_1653652	PTPRC	0.643222451
ILMN_1731233	GZMH	0.641744314
ILMN_1675979	RBMS3	0.640827652
ILMN_1763487	CTLA4	0.640579082
ILMN_1747622	CD33	0.640432382
ILMN_1708167	CDCP1	0.63736429
ILMN_1733579	EVI2A	0.636921311
ILMN_1687301	VCAN	0.636772457
ILMN_1663080	LFNG	0.636170995
ILMN_1711566	TIMP1	0.63525613
ILMN_1794473	PHF17	0.635227681
ILMN_3236904	LOC648740	0.635152195
ILMN_1683271	TMSB4X	0.634919967
ILMN_2327346	SSBP4	0.634757175
ILMN_1689655	HLA-DRA	0.634529709
ILMN_1768016	TNFRSF17	0.63423453
ILMN_2258816	SAMD3	0.634095811
ILMN_1786789	FAM102B	0.634003574
ILMN_1765258	HLA-E	0.632673626
ILMN_1760347	SRGN	0.632136812
ILMN_1782057	ATP8B2	0.631904545
ILMN_1662799	GPSM3	0.631806419
ILMN_1685387	PIGR	0.63065527
ILMN_1774077	GBP2	0.630248406
ILMN_1665964	GAB2	0.629921037
ILMN_1754894	C1orf162	0.629749985
ILMN_3210917	SET	0.629579599
ILMN_1802106	APOBEC3G	0.629537454
ILMN_2176755	KIAA0895L	0.629524451
ILMN_2104356	COL1A2	0.629491707
ILMN_1655935	ADCY7	0.629328278
ILMN_3249400	abParts	0.627964064
ILMN_1733998	DHRS9	0.627644804
ILMN_2129563	LDLRAD3	0.627249465
ILMN_1797822	SEL1L3	0.627098346
ILMN_1676256	TPSAB1	0.626763633
ILMN_1772218	HLA-DPA1	0.626042984
ILMN_2042651	EVI2B	0.625729231
ILMN_2101885	TUBB	0.625562301
ILMN_1685625	UCP2	0.625369503
ILMN_1740633	PRF1	0.625116491
ILMN_1757827	VOPP1	0.624264732
ILMN_2280548	FYB	0.623460974
ILMN_2079655	KLRB1	0.622892049
ILMN_1666269	CTSZ	0.620914344
ILMN_1788108	TXNDC5	0.620581685
ILMN_1738675	PTPN6	0.620434331
ILMN_2155228	CRLF3	0.620193102
ILMN_1653026	PLAC8	0.619917357
ILMN_2117508	CTHRC1	0.619857221
ILMN_1723684	DARC	0.618907069
ILMN_3228688	HLA-DRB1	0.615133022
ILMN_2123743	FCER1G	0.614721347
ILMN_1760509	EOMES	0.614124506
ILMN_2396991	HCST	0.613943988
ILMN_3240316	TMSB4X	0.613190592
ILMN_1696591	RB1	0.612819262
ILMN_1696494	CMTM6	0.611892445
ILMN_2359800	MS4A6A	0.611627525
ILMN_1722781	EGR3	0.611115745
ILMN_1761464	CD74	0.610382231
ILMN_2348788	CD44	0.610337518
ILMN_1719759	TNC	0.608756563
ILMN_1784661	TMEM2	0.608713078
ILMN_2412192	CFH	0.608181813
ILMN_1771333	CD47	0.60678531
ILMN_2384591	HN1	0.606579249

	INTERPRO	MHC class I, alpha chain, alpha1 and alpha2	7	6.90E-03	3.80E-01
	INTERPRO	MHC class I-like antigen recognition	7	8.30E-03	3.90E-01
	SP_PIR_KEYWORDS	transplantation antigen	3	2.10E-02	1.40E-01
	INTERPRO	MHC class II, beta chain, N-terminal	5	2.20E-02	5.60E-01
	INTERPRO	MHC class I, alpha chain, C-terminal	3	2.30E-02	5.70E-01
	BBID	70.Signal_peptides_(MHC)_class_I_molecules	3	9.10E-02	9.80E-01
**Annotation Cluster 8**	**Enrichment Score: 5.46**		**Count**	**P_Value**	**Benjamini**
	GOTERM_BP_FAT	antigen processing and presentation of peptide antigen	14	1.10E-08	1.80E-06
	GOTERM_BP_FAT	antigen processing and presentation of peptide or polysaccharide antigen via MHC class II	13	1.00E-06	9.50E-05
	GOTERM_BP_FAT	antigen processing and presentation of exogenous peptide antigen	8	1.90E-06	1.70E-04
	GOTERM_BP_FAT	antigen processing and presentation of exogenous antigen	8	1.60E-05	1.00E-03
	GOTERM_BP_FAT	antigen processing and presentation of peptide antigen via MHC class II	6	7.30E-05	3.70E-03
	GOTERM_BP_FAT	antigen processing and presentation of exogenous peptide antigen via MHC class II	6	7.30E-05	3.70E-03
**Annotation Cluster 9**	**Enrichment Score: 5.06**		**Count**	**P_Value**	**Benjamini**
	SP_PIR_KEYWORDS	glycoprotein	357	2.60E-10	2.20E-08
	UP_SEQ_FEATURE	glycosylation site:N-linked (GlcNAc...)	332	6.40E-08	3.40E-05
	UP_SEQ_FEATURE	topological domain:Cytoplasmic	280	7.10E-08	3.30E-05
	UP_SEQ_FEATURE	topological domain:Extracellular	231	2.50E-07	8.90E-05
	SP_PIR_KEYWORDS	membrane	462	1.20E-06	4.80E-05
	GOTERM_CC_FAT	plasma membrane	318	1.90E-05	1.10E-03
	UP_SEQ_FEATURE	transmembrane region	360	1.30E-04	3.10E-02
	SP_PIR_KEYWORDS	transmembrane	361	1.80E-04	3.00E-03
	GOTERM_CC_FAT	intrinsic to membrane	399	1.00E-01	6.20E-01
	GOTERM_CC_FAT	integral to membrane	382	1.70E-01	7.50E-01
**Annotation Cluster 10**	**Enrichment Score: 4.99**		**Count**	**P_Value**	**Benjamini**
	GOTERM_CC_FAT	intrinsic to plasma membrane	124	7.30E-06	7.70E-04
	GOTERM_CC_FAT	integral to plasma membrane	121	1.10E-05	8.20E-04
	GOTERM_CC_FAT	plasma membrane part	201	1.40E-05	9.00E-04
**Annotation Cluster 11**	**Enrichment Score: 4.71**		**Count**	**P_Value**	**Benjamini**
	GOTERM_BP_FAT	positive regulation of immune system process	57	1.30E-16	1.80E-13
	GOTERM_BP_FAT	regulation of lymphocyte activation	34	1.30E-09	3.30E-07
	GOTERM_BP_FAT	regulation of leukocyte activation	36	2.00E-09	4.50E-07
	GOTERM_BP_FAT	regulation of cell activation	36	8.70E-09	1.60E-06
	GOTERM_BP_FAT	positive regulation of leukocyte activation	26	4.00E-08	6.60E-06
	GOTERM_BP_FAT	regulation of T cell activation	27	7.90E-08	1.10E-05
	GOTERM_BP_FAT	positive regulation of cell activation	26	1.10E-07	1.40E-05
	GOTERM_BP_FAT	negative regulation of immune system process	22	1.30E-07	1.70E-05
	GOTERM_BP_FAT	positive regulation of lymphocyte activation	23	5.40E-07	5.70E-05
	GOTERM_BP_FAT	regulation of lymphocyte proliferation	21	6.30E-07	6.50E-05
	GOTERM_BP_FAT	regulation of mononuclear cell proliferation	21	7.70E-07	7.70E-05
	GOTERM_BP_FAT	regulation of leukocyte proliferation	21	7.70E-07	7.70E-05
	GOTERM_BP_FAT	regulation of B cell proliferation	12	1.20E-06	1.10E-04
	GOTERM_BP_FAT	regulation of B cell activation	15	5.90E-06	4.50E-04
	GOTERM_BP_FAT	positive regulation of T cell activation	18	1.30E-05	8.50E-04
	GOTERM_BP_FAT	regulation of lymphocyte differentiation	16	1.80E-05	1.10E-03
	GOTERM_BP_FAT	positive regulation of B cell activation	11	5.00E-05	2.80E-03
	GOTERM_BP_FAT	positive regulation of lymphocyte proliferation	14	7.10E-05	3.70E-03
	GOTERM_BP_FAT	positive regulation of leukocyte proliferation	14	8.70E-05	4.20E-03
	GOTERM_BP_FAT	positive regulation of mononuclear cell proliferation	14	8.70E-05	4.20E-03
	GOTERM_BP_FAT	positive regulation of lymphocyte differentiation	11	8.80E-05	4.20E-03
	GOTERM_BP_FAT	regulation of T cell differentiation	13	1.40E-04	6.40E-03
	GOTERM_BP_FAT	positive regulation of B cell proliferation	8	2.60E-04	1.10E-02
	GOTERM_BP_FAT	regulation of T cell proliferation	13	9.50E-04	3.40E-02
	GOTERM_BP_FAT	negative regulation of lymphocyte activation	12	9.80E-04	3.50E-02
	GOTERM_BP_FAT	positive regulation of T cell differentiation	9	1.20E-03	4.10E-02
	GOTERM_BP_FAT	negative regulation of leukocyte proliferation	9	1.20E-03	4.10E-02
	GOTERM_BP_FAT	negative regulation of lymphocyte proliferation	9	1.20E-03	4.10E-02
	GOTERM_BP_FAT	negative regulation of mononuclear cell proliferation	9	1.20E-03	4.10E-02
	GOTERM_BP_FAT	negative regulation of leukocyte activation	12	1.60E-03	5.10E-02
	GOTERM_BP_FAT	negative regulation of cell activation	12	2.80E-03	7.90E-02
	GOTERM_BP_FAT	negative regulation of T cell activation	9	7.20E-03	1.60E-01
	GOTERM_BP_FAT	negative regulation of T cell proliferation	7	9.00E-03	1.90E-01
	GOTERM_BP_FAT	negative regulation of B cell proliferation	4	1.40E-02	2.50E-01
	GOTERM_BP_FAT	positive regulation of T cell proliferation	8	1.60E-02	2.70E-01
	GOTERM_BP_FAT	regulation of B cell differentiation	5	2.10E-02	3.20E-01
	GOTERM_BP_FAT	positive regulation of cell proliferation	40	2.90E-02	3.90E-01
	GOTERM_BP_FAT	negative regulation of B cell activation	5	3.20E-02	4.10E-01
**Annotation Cluster 12**	**Enrichment Score: 4.71**		**Count**	**P_Value**	**Benjamini**
	GOTERM_BP_FAT	cell adhesion	79	1.50E-05	1.00E-03
	GOTERM_BP_FAT	biological adhesion	79	1.60E-05	1.00E-03
	SP_PIR_KEYWORDS	cell adhesion	49	3.10E-05	6.80E-04
**Annotation Cluster 13**	**Enrichment Score: 4.56**		**Count**	**P_Value**	**Benjamini**
	GOTERM_BP_FAT	immune effector process	31	6.60E-09	1.30E-06
	GOTERM_BP_FAT	leukocyte mediated immunity	23	5.50E-08	8.60E-06
	GOTERM_BP_FAT	lymphocyte mediated immunity	19	8.10E-07	7.90E-05
	GOTERM_BP_FAT	B cell mediated immunity	16	3.80E-06	3.10E-04
	GOTERM_BP_FAT	immunoglobulin mediated immune response	15	1.20E-05	8.40E-04
	GOTERM_BP_FAT	adaptive immune response based on somatic recombination of immune receptors built from immunoglobulin superfamily domains	18	1.50E-05	9.80E-04
	GOTERM_BP_FAT	adaptive immune response	18	1.50E-05	9.80E-04
	SP_PIR_KEYWORDS	complement pathway	6	3.00E-02	1.80E-01
	GOTERM_BP_FAT	complement activation, classical pathway	6	4.60E-02	4.90E-01
	GOTERM_BP_FAT	humoral immune response mediated by circulating immunoglobulin	6	5.90E-02	5.60E-01
**Annotation Cluster 14**	**Enrichment Score: 4.32**		**Count**	**P_Value**	**Benjamini**
	SP_PIR_KEYWORDS	Apoptosis	46	2.20E-05	5.10E-04
	GOTERM_BP_FAT	programmed cell death	70	3.00E-05	1.80E-03
	GOTERM_BP_FAT	apoptosis	68	6.30E-05	3.40E-03
	GOTERM_BP_FAT	cell death	78	6.70E-05	3.50E-03
	GOTERM_BP_FAT	death	78	8.50E-05	4.20E-03
**Annotation Cluster 15**	**Enrichment Score: 3.81**		**Count**	**P_Value**	**Benjamini**
	GOTERM_BP_FAT	positive regulation of signal transduction	40	6.50E-05	3.50E-03
	GOTERM_BP_FAT	positive regulation of I-kappaB kinase/NF-kappaB cascade	19	1.00E-04	4.70E-03
	GOTERM_BP_FAT	regulation of I-kappaB kinase/NF-kappaB cascade	20	1.20E-04	5.50E-03
	GOTERM_BP_FAT	positive regulation of cell communication	42	1.60E-04	7.20E-03

ILMN_1723846	METTL21B	-0.406466647
ILMN_1753026	LPPR1	-0.406482258
ILMN_1679555	TIMM23	-0.406643891
ILMN_1656638	BHMT	-0.406668635
ILMN_3307659	SFT2D2	-0.406739219
ILMN_1655892	BE266556	-0.406773994
ILMN_1728799	FBP1	-0.406801455
ILMN_2323801	MOCS1	-0.406811456
ILMN_2059886	TTC38	-0.406832136
ILMN_1708798	EAF2	-0.406847717
ILMN_1685781	C14orf142	-0.406876586
ILMN_1777759	FIGNL2	-0.406882004
ILMN_1710312	TMEM31	-0.406962311
ILMN_2085659	OTOGL	-0.406965149
ILMN_3297876	VSTM2B	-0.406973066
ILMN_1730572	HNRPDL	-0.406992159
ILMN_1656327	GLT1D1	-0.407003264
ILMN_2138754	C9orf68	-0.407033822
ILMN_1680580	LOC148709	-0.407063238
ILMN_2246510	TSC1	-0.407092219
ILMN_3199958	PCDH11Y	-0.407191873
ILMN_1692056	HS3ST3A1	-0.40722485
ILMN_1852793	BC067908	-0.407245476
ILMN_1757747	ASPDH	-0.407277074
ILMN_1656672	MYLK	-0.407625829
ILMN_2366795	KIF27	-0.407660624
ILMN_1784269	AASDH	-0.407677044
ILMN_1681221	C9orf100	-0.40768282
ILMN_1788394	TTLL6	-0.407751892
ILMN_2166706	FBXO22OS	-0.407774573
ILMN_1751636	ANKS3	-0.40782564
ILMN_3219380	NANOGNB	-0.407838224
ILMN_2393341	LIAS	-0.407850136
ILMN_1693664	POMGNT1	-0.40785411
ILMN_1681651	FOLH1	-0.407942371
ILMN_1697301	ST8SIA3	-0.408004155
ILMN_1775034	MEIG1	-0.408088461
ILMN_1699674	ZNF703	-0.408210705
ILMN_3243135	SNORA37	-0.408411016
ILMN_1692535	DPP4	-0.408540441
ILMN_1811933	SHMT1	-0.408653126
ILMN_1855682	BU634332	-0.408764337
ILMN_1712298	ANKRD46	-0.408816791
ILMN_1769547	DIO1	-0.408840991
ILMN_1730441	OR52K2	-0.408990927
ILMN_1708660	RWDD4	-0.40905975
ILMN_1754234	ZMYND11	-0.409086919
ILMN_1680925	SLC9A3R1	-0.40920705
ILMN_1779751	C7orf55	-0.409208618
ILMN_1795920	OR2M4	-0.409249036
ILMN_1755843	SLC26A8	-0.40928643
ILMN_1696661	FAM186A	-0.409315146
ILMN_1751941	TMEM106A	-0.409348261
ILMN_1735108	ANKS6	-0.409451602
ILMN_2396697	GRIK2	-0.409452906
ILMN_1652006	SMC1A	-0.409468178
ILMN_2133038	UGT3A1	-0.409489243
ILMN_1811719	MYO7A	-0.409495838
ILMN_1677384	PTGER3	-0.409702323
ILMN_1765287	DPY19L3	-0.409740692
ILMN_1685696	C3orf55	-0.409902991
ILMN_2372403	ALDH5A1	-0.410057044
ILMN_1683146	FTH1	-0.410131967
ILMN_3240173	MTSS1L	-0.410150972
ILMN_1800874	MRO	-0.410152152
ILMN_3232806	HIGD1A	-0.410190531
ILMN_2134855	PIN4	-0.410209718
ILMN_1888492	AA083069	-0.41052115
ILMN_1794643	ZGPAT	-0.410663413
ILMN_1728074	PHAX	-0.41067411
ILMN_1800130	ETNK2	-0.410874206
ILMN_1684106	NR1I3	-0.410911299
ILMN_2236625	UTS2	-0.410914628
ILMN_1910948	AK091312	-0.410984154
ILMN_3247625	SNORD98	-0.411083046
ILMN_1746883	SAT2	-0.411267825
ILMN_1700042	TLN2	-0.411298878
ILMN_1740052	SPEG	-0.411341197
ILMN_2048683	FSCB	-0.411346898
ILMN_1651310	MLLT4	-0.41136575
ILMN_2385416	GPX5	-0.411485421
ILMN_1770853	CHST5	-0.411503989
ILMN_1769934	GRHL1	-0.411534414
ILMN_1843198	AK4	-0.411547521
ILMN_3225014	ZNF285A	-0.411621058
ILMN_2225537	PTGR1	-0.411746413
ILMN_1680144	HLA-DQA2	-0.411747287
ILMN_1659914	AK8	-0.411750847
ILMN_3238299	NHEDC1	-0.4118144
ILMN_2061116	MATN1	-0.41187255
ILMN_1771223	PADI2	-0.411898246
ILMN_1867777	BU535897	-0.411952579
ILMN_1735658	RTTN	-0.411958167

**Annotation Cluster 12**	**Enrichment Score: 3.52**		**Count**	**P_Value**	**Benjamini**
	GOTERM_BP_FAT	generation of precursor metabolites and energy	52	4.90E-12	7.30E-09
	GOTERM_BP_FAT	energy derivation by oxidation of organic compounds	30	1.50E-09	1.10E-06
	SP_PIR_KEYWORDS	mitochondrion inner membrane	34	7.20E-09	8.70E-07
	GOTERM_BP_FAT	cellular respiration	22	7.40E-08	3.20E-05
	GOTERM_CC_FAT	mitochondrial membrane part	26	1.20E-07	6.20E-06
	KEGG_PATHWAY	Oxidative phosphorylation	26	2.70E-06	8.30E-05
	GOTERM_BP_FAT	oxidative phosphorylation	18	3.00E-05	4.90E-03
	GOTERM_CC_FAT	mitochondrial respiratory chain	14	1.10E-04	3.90E-03
	GOTERM_CC_FAT	respiratory chain	15	1.60E-04	5.00E-03
	GOTERM_BP_FAT	electron transport chain	18	2.10E-04	2.40E-02
	SP_PIR_KEYWORDS	respiratory chain	13	5.00E-04	1.90E-02
	GOTERM_BP_FAT	respiratory electron transport chain	12	8.00E-04	6.00E-02
	OMIM_DISEASE	Mitochondrial complex I deficiency	5	9.00E-04	3.10E-01
	SP_PIR_KEYWORDS	electron transport	15	1.20E-03	3.90E-02
	KEGG_PATHWAY	Alzheimer's disease	23	2.20E-03	1.90E-02
	GOTERM_MF_FAT	oxidoreductase activity, acting on NADH or NADPH	13	3.40E-03	2.00E-01
	KEGG_PATHWAY	Parkinson's disease	19	3.40E-03	2.60E-02
	GOTERM_MF_FAT	NADH dehydrogenase (ubiquinone) activity	8	1.20E-02	3.50E-01
	GOTERM_MF_FAT	NADH dehydrogenase (quinone) activity	8	1.20E-02	3.50E-01
	GOTERM_MF_FAT	NADH dehydrogenase activity	8	1.20E-02	3.50E-01
	KEGG_PATHWAY	Huntington's disease	22	1.40E-02	9.30E-02
	GOTERM_MF_FAT	oxidoreductase activity, acting on NADH or NADPH, quinone or similar compound as acceptor	8	2.40E-02	4.90E-01
	GOTERM_BP_FAT	mitochondrial electron transport, NADH to ubiquinone	7	2.90E-02	5.20E-01
	GOTERM_BP_FAT	ATP synthesis coupled electron transport	8	3.70E-02	5.60E-01
	GOTERM_BP_FAT	mitochondrial ATP synthesis coupled electron transport	8	3.70E-02	5.60E-01
	GOTERM_CC_FAT	NADH dehydrogenase complex	7	4.20E-02	4.90E-01
	GOTERM_CC_FAT	mitochondrial respiratory chain complex I	7	4.20E-02	4.90E-01
	GOTERM_CC_FAT	respiratory chain complex I	7	4.20E-02	4.90E-01
	SP_PIR_KEYWORDS	ubiquinone	4	2.30E-01	8.30E-01
**Annotation Cluster 13**	**Enrichment Score: 3.03**		**Count**	**P_Value**	**Benjamini**
	GOTERM_BP_FAT	water-soluble vitamin metabolic process	10	3.00E-04	3.10E-02
	GOTERM_BP_FAT	betaine metabolic process	4	1.60E-03	9.90E-02
	GOTERM_BP_FAT	carnitine metabolic process	4	1.60E-03	9.90E-02
**Annotation Cluster 14**	**Enrichment Score: 2.81**		**Count**	**P_Value**	**Benjamini**
	GOTERM_CC_FAT	vesicular fraction	32	9.10E-05	3.40E-03
	GOTERM_CC_FAT	microsome	31	1.30E-04	4.10E-03
	GOTERM_CC_FAT	membrane fraction	69	4.70E-03	1.20E-01
	GOTERM_CC_FAT	insoluble fraction	71	4.90E-03	1.20E-01
	GOTERM_CC_FAT	cell fraction	82	3.40E-02	4.30E-01
**Annotation Cluster 15**	**Enrichment Score: 2.8**		**Count**	**P_Value**	**Benjamini**
	GOTERM_BP_FAT	glucose metabolic process	27	3.80E-07	1.30E-04
	GOTERM_BP_FAT	monosaccharide metabolic process	32	2.50E-06	6.30E-04
	GOTERM_BP_FAT	hexose metabolic process	29	3.30E-06	7.00E-04
	KEGG_PATHWAY	Glycolysis / Gluconeogenesis	15	5.00E-05	9.20E-04
	GOTERM_BP_FAT	pyruvate metabolic process	11	8.50E-05	1.10E-02
	GOTERM_BP_FAT	alcohol biosynthetic process	11	1.30E-04	1.60E-02
	GOTERM_BP_FAT	hexose biosynthetic process	9	2.40E-04	2.60E-02
	GOTERM_BP_FAT	cellular carbohydrate catabolic process	15	2.60E-04	2.70E-02
	GOTERM_BP_FAT	gluconeogenesis	8	3.40E-04	3.30E-02
	GOTERM_BP_FAT	carbohydrate catabolic process	17	3.80E-04	3.40E-02
	GOTERM_BP_FAT	alcohol catabolic process	14	5.50E-04	4.50E-02
	GOTERM_BP_FAT	monosaccharide biosynthetic process	9	8.70E-04	6.30E-02
	GOTERM_BP_FAT	cellular carbohydrate biosynthetic process	12	1.50E-03	9.50E-02
	GOTERM_BP_FAT	carbohydrate biosynthetic process	15	2.60E-03	1.40E-01
	GOTERM_MF_FAT	oxidoreductase activity, acting on the aldehyde or oxo group of donors, disulfide as acceptor	4	9.20E-03	3.10E-01
	GOTERM_MF_FAT	pyruvate dehydrogenase activity	3	1.00E-02	3.10E-01
	GOTERM_MF_FAT	pyruvate dehydrogenase (acetyl-transferring) activity	3	1.00E-02	3.10E-01
	GOTERM_BP_FAT	hexose catabolic process	10	1.50E-02	3.80E-01
	GOTERM_BP_FAT	glycolysis	8	1.50E-02	3.90E-01
	GOTERM_BP_FAT	glucose catabolic process	9	1.50E-02	3.90E-01
	GOTERM_BP_FAT	monosaccharide catabolic process	10	1.80E-02	4.10E-01
	SP_PIR_KEYWORDS	thiamine pyrophosphate	4	2.60E-02	3.10E-01
	SP_PIR_KEYWORDS	thiamin pyrophosphate	3	2.80E-02	3.20E-01
	SP_PIR_KEYWORDS	glycolysis	7	3.40E-02	3.60E-01
	SP_PIR_KEYWORDS	pyruvate	4	6.50E-02	5.00E-01
	KEGG_PATHWAY	Valine, leucine and isoleucine biosynthesis	3	1.80E-01	6.10E-01
	SP_PIR_KEYWORDS	heterotetramer	4	7.40E-01	9.90E-01
**Annotation Cluster 16**	**Enrichment Score: 2.7**		**Count**	**P_Value**	**Benjamini**
	SP_PIR_KEYWORDS	Flavoprotein	23	8.10E-08	8.20E-06
	SP_PIR_KEYWORDS	FAD	18	1.80E-04	9.10E-03
	UP_SEQ_FEATURE	binding site:Substrate; via amide nitrogen	8	1.80E-04	1.20E-01
	GOTERM_MF_FAT	FAD binding	14	2.70E-04	3.60E-02
	UP_SEQ_FEATURE	binding site:Substrate; via carbonyl oxygen	8	4.20E-04	2.00E-01
	UP_SEQ_FEATURE	nucleotide phosphate-binding region:FAD	11	1.90E-03	4.30E-01
	INTERPRO	Acyl-CoA dehydrogenase/oxidase, N-terminal	5	6.80E-03	6.60E-01
	INTERPRO	Acyl-CoA oxidase/dehydrogenase, type1/2, C-terminal	5	8.90E-03	6.30E-01
	INTERPRO	Acyl-CoA oxidase/dehydrogenase, central region	5	8.90E-03	6.30E-01
	GOTERM_MF_FAT	acyl-CoA dehydrogenase activity	5	9.80E-03	3.20E-01
	INTERPRO	Acyl-CoA dehydrogenase, N-terminal	4	2.20E-02	8.10E-01
	INTERPRO	Acyl-CoA oxidase/dehydrogenase, type 1	4	3.50E-02	9.00E-01
	PIR_SUPERFAMILY	PIRSF000182:acyl-CoA dehydrogenase	3	5.30E-02	1.00E+00
	INTERPRO	Acyl-CoA dehydrogenase, conserved site	3	1.10E-01	9.90E-01
**Annotation Cluster 17**	**Enrichment Score: 2.43**		**Count**	**P_Value**	**Benjamini**
	GOTERM_BP_FAT	alditol metabolic process	8	3.40E-04	3.30E-02
	GOTERM_BP_FAT	glycerol metabolic process	7	1.40E-03	9.20E-02
	GOTERM_MF_FAT	NAD or NADH binding	9	5.80E-03	2.40E-01
	GOTERM_BP_FAT	polyol metabolic process	8	6.40E-03	2.50E-01
	GOTERM_BP_FAT	alditol catabolic process	3	9.10E-03	3.00E-01
	GOTERM_BP_FAT	polyol catabolic process	3	1.80E-02	4.10E-01
**Annotation Cluster 18**	**Enrichment Score: 2.34**		**Count**	**P_Value**	**Benjamini**
	GOTERM_CC_FAT	mitochondrial lumen	41	1.10E-09	8.10E-08
	GOTERM_CC_FAT	mitochondrial matrix	41	1.10E-09	8.10E-08

ILMN_2181968	CBL	0.606045894
ILMN_2196550	C13orf18	0.605897828
ILMN_2169152	SRGN	0.605538276
ILMN_2120965	NPAT	0.605378814
ILMN_1711272	SERPING1	0.605088424
ILMN_3247064	SNRNP40	0.604743386
ILMN_1662932	LCP1	0.604545163
ILMN_1716080	CBL	0.604504512
ILMN_1760027	WAS	0.604401443
ILMN_1723486	HK2	0.604279551
ILMN_1727284	CD4	0.60420109
ILMN_1700428	HLA-DOB	0.604033998
ILMN_1777998	ARHGAP25	0.603919721
ILMN_1664691	DAPP1	0.602275452
ILMN_1724293	KDELR2	0.602021259
ILMN_1803560	LAT2	0.601951917
ILMN_2113333	LGALS9B	0.600722957
ILMN_1809433	XBP1	0.600395804
ILMN_1713058	PSTPIP2	0.599392687
ILMN_1687888	BTN3A1	0.598555999
ILMN_1786720	PROM1	0.597899298
ILMN_1798379	NTM	0.596966135
ILMN_1685403	MMP7	0.596823483
ILMN_1701877	AXL	0.59680269
ILMN_1742427	POLR1D	0.596482413
ILMN_1756937	ST8SIA4	0.596448333
ILMN_1771261	SYNC	0.596409017
ILMN_2115005	FGD2	0.595959263
ILMN_1673682	GATAD2A	0.595487326
ILMN_1765446	EMP3	0.595412119
ILMN_1734653	FNDC1	0.594373707
ILMN_2315979	LBH	0.594161531
ILMN_1712305	CYBRD1	0.593714732
ILMN_2193233	MZB1	0.592415886
ILMN_1809400	FAM49B	0.592225946
ILMN_1787345	FKBP11	0.59145019
ILMN_2379644	CD74	0.590067684
ILMN_2115434	RAB32	0.589890504
ILMN_1737146	TRAM1	0.589781649
ILMN_1668417	WIPF1	0.58952472
ILMN_2117987	TFDP1	0.589297139
ILMN_1651433	DCK	0.588675286
ILMN_2109416	NAPSB	0.587491356
ILMN_2226955	VOPP1	0.587088493
ILMN_1710937	IFI16	0.586048375
ILMN_1780368	GPR18	0.586023222
ILMN_1767448	LHFP	0.585177803
ILMN_1814194	TCF4	0.584858958
ILMN_1781155	LYN	0.584815345
ILMN_1727402	HCLS1	0.58468761
ILMN_2313926	CDC42SE2	0.584512047
ILMN_2388701	ST3GAL5	0.5844603
ILMN_2176063	FCGR1C	0.58418888
ILMN_2249920	FYN	0.584182797
ILMN_1724422	SELL	0.583985345
ILMN_2058251	VIM	0.583855607
ILMN_2381037	LIMS1	0.583853497
ILMN_2179837	BANF1	0.583803587
ILMN_1797988	KLRD1	0.583424679
ILMN_2400759	CPVL	0.583355378
ILMN_3243238	TPSAB1	0.582984476
ILMN_1710482	APLP2	0.582271186
ILMN_1778977	TYROBP	0.582244767
ILMN_1785902	C1QC	0.58180647
ILMN_1800091	RARRES1	0.58157835
ILMN_1780663	KLHL30	0.581317966
ILMN_1664099	GLIS3	0.580989642
ILMN_2415162	sept-01	0.580545891
ILMN_1781626	C1S	0.580525801
ILMN_2143314	SPIB	0.579798736
ILMN_1652198	CCM2	0.579431755
ILMN_1717197	CD3G	0.579237813
ILMN_1872759	BX099468	0.579211026
ILMN_1711383	STK4	0.579190057
ILMN_1793672	SIX5	0.577840885
ILMN_1808634	DRAM2	0.577489944
ILMN_2066066	HLA-DRB6	0.576693669
ILMN_1745356	CXCL9	0.5766773
ILMN_1695025	CD2	0.575943185
ILMN_2192072	MMP7	0.575640117
ILMN_1716563	PRKCB	0.575596169
ILMN_1744109	OR2A42	0.575443115
ILMN_1800058	NKX2-5	0.575439851
ILMN_1656129	SLC39A10	0.575194973
ILMN_2200636	KIAA1267	0.575073029
ILMN_1805466	SOX9	0.57439515
ILMN_1790918	ZNF236	0.573816387
ILMN_2316236	HOPX	0.573373582
ILMN_1729269	LOC285074	0.572955663
ILMN_1800317	WNT5A	0.572942095
ILMN_2360710	TPM1	0.572741597
ILMN_1733538	RGS10	0.57231079
ILMN_1672503	DPYSL2	0.572224734

	GOTERM_BP_FAT	regulation of protein kinase cascade	34	2.30E-04	9.60E-03
	GOTERM_BP_FAT	positive regulation of protein kinase cascade	25	4.90E-04	1.90E-02
**Annotation Cluster 16**	**Enrichment Score: 3.67**		**Count**	**P_Value**	**Benjamini**
	GOTERM_MF_FAT	glycosaminoglycan binding	26	4.70E-06	1.10E-03
	GOTERM_MF_FAT	pattern binding	26	2.60E-05	3.10E-03
	GOTERM_MF_FAT	polysaccharide binding	26	2.60E-05	3.10E-03
	GOTERM_MF_FAT	carbohydrate binding	41	6.40E-04	3.90E-02
	SP_PIR_KEYWORDS	heparin-binding	11	6.20E-03	5.50E-02
	GOTERM_MF_FAT	heparin binding	15	7.80E-03	2.60E-01
**Annotation Cluster 17**	**Enrichment Score: 3.51**		**Count**	**P_Value**	**Benjamini**
	SP_PIR_KEYWORDS	extracellular matrix	34	1.40E-05	3.80E-04
	GOTERM_CC_FAT	extracellular matrix	43	2.40E-04	9.20E-03
	GOTERM_CC_FAT	proteinaceous extracellular matrix	40	3.90E-04	1.40E-02
	GOTERM_CC_FAT	extracellular matrix part	17	6.70E-03	1.30E-01
**Annotation Cluster 18**	**Enrichment Score: 3.41**		**Count**	**P_Value**	**Benjamini**
	SP_PIR_KEYWORDS	proteoglycan	14	1.80E-05	4.50E-04
	UP_SEQ_FEATURE	glycosylation site:O-linked (Xyl...) (glycosaminoglycan)	6	1.10E-03	1.70E-01
	SP_PIR_KEYWORDS	chondroitin sulfate proteoglycan	6	2.90E-03	3.10E-02
**Annotation Cluster 19**	**Enrichment Score: 3.31**		**Count**	**P_Value**	**Benjamini**
	GOTERM_BP_FAT	actin cytoskeleton organization	37	2.00E-06	1.70E-04
	GOTERM_BP_FAT	actin filament-based process	37	9.00E-06	6.40E-04
	SP_PIR_KEYWORDS	actin-binding	32	1.30E-04	2.30E-03
	GOTERM_MF_FAT	actin binding	39	4.80E-04	3.70E-02
	GOTERM_BP_FAT	cytoskeleton organization	44	1.10E-02	2.10E-01
	GOTERM_MF_FAT	cytoskeletal protein binding	47	1.70E-02	3.80E-01
	GOTERM_CC_FAT	actin cytoskeleton	28	3.30E-02	3.90E-01
**Annotation Cluster 20**	**Enrichment Score: 3.23**		**Count**	**P_Value**	**Benjamini**
	KEGG_PATHWAY	Chemokine signaling pathway	39	7.90E-08	1.50E-06
	GOTERM_MF_FAT	chemokine activity	14	5.70E-06	1.10E-03
	GOTERM_MF_FAT	chemokine receptor binding	14	1.20E-05	1.90E-03
	SP_PIR_KEYWORDS	chemotaxis	16	2.70E-05	6.20E-04
	GOTERM_BP_FAT	chemotaxis	27	3.60E-05	2.10E-03
	GOTERM_BP_FAT	taxis	27	3.60E-05	2.10E-03
	INTERPRO	Small chemokine, interleukin-8-like	12	4.40E-05	1.10E-02
	SMART	SCY	12	2.10E-04	1.50E-02
	KEGG_PATHWAY	Cytokine-cytokine receptor interaction	39	2.90E-04	2.20E-03
	INTERPRO	Small chemokine, C-X-C, conserved site	7	5.00E-04	6.40E-02
	PIR_SUPERFAMILY	PIRSF002522:CXC chemokine	6	2.60E-03	3.70E-01
	SP_PIR_KEYWORDS	cytokine	22	3.90E-03	3.90E-02
	INTERPRO	Small chemokine, C-X-C	5	8.00E-03	3.90E-01
	BBID	109.Chemokine_families	11	8.50E-03	5.10E-01
	GOTERM_MF_FAT	cytokine activity	23	1.00E-02	2.90E-01
	INTERPRO	Small chemokine, C-X-C/Interleukin 8	5	1.10E-02	4.60E-01
	GOTERM_BP_FAT	locomotory behavior	30	1.40E-02	2.50E-01
	INTERPRO	Small chemokine, C-C group, conserved site	6	2.10E-02	5.70E-01
	PIR_SUPERFAMILY	PIRSF001950:small inducible chemokine, C/CC types	6	2.50E-02	8.50E-01
	GOTERM_BP_FAT	behavior	38	2.20E-01	8.70E-01
**Annotation Cluster 21**	**Enrichment Score: 3.14**		**Count**	**P_Value**	**Benjamini**
	SP_PIR_KEYWORDS	sh3-binding	14	7.10E-05	1.40E-03
	GOTERM_MF_FAT	SH3 domain binding	18	1.70E-04	1.60E-02
	GOTERM_MF_FAT	protein domain specific binding	38	1.20E-03	6.50E-02
	UP_SEQ_FEATURE	short sequence motif:SH3-binding	9	1.90E-02	7.50E-01
**Annotation Cluster 22**	**Enrichment Score: 3.13**		**Count**	**P_Value**	**Benjamini**
	GOTERM_BP_FAT	T cell selection	8	1.80E-04	7.60E-03
	BIOCARTA	T Helper Cell Surface Molecules	8	1.80E-04	1.00E-02
	OMIM_DISEASE	Severe combined immunodeficiency, T cell-negative, B-cell/natural killer-cell positive	3	1.30E-02	6.90E-01
**Annotation Cluster 23**	**Enrichment Score: 3.12**		**Count**	**P_Value**	**Benjamini**
	GOTERM_BP_FAT	cell motion	60	7.20E-06	5.30E-04
	GOTERM_BP_FAT	cell migration	33	2.70E-03	7.70E-02
	GOTERM_BP_FAT	cell motility	35	4.20E-03	1.00E-01
	GOTERM_BP_FAT	localization of cell	35	4.20E-03	1.00E-01
**Annotation Cluster 24**	**Enrichment Score: 2.97**		**Count**	**P_Value**	**Benjamini**
	BIOCARTA	Lck and Fyn tyrosine kinases in initiation of TCR Activation	9	5.40E-06	1.30E-03
	BIOCARTA	CTL mediated immune response against target cells	10	1.40E-05	1.60E-03
	BIOCARTA	Activation of Csk by cAMP-dependent Protein Kinase Inhibits Signaling through the T Cell Receptor	10	5.40E-05	4.20E-03
	INTERPRO	Phosphorylated immunoreceptor signaling ITAM	6	5.50E-05	9.90E-03
	GOTERM_CC_FAT	T cell receptor complex	7	7.00E-05	3.70E-03
	UP_SEQ_FEATURE	domain:ITAM	6	9.30E-05	2.50E-02
	SMART	ITAM	6	1.30E-04	1.90E-02
	BIOCARTA	T Cytotoxic Cell Surface Molecules	8	1.80E-04	1.00E-02
	BIOCARTA	T Helper Cell Surface Molecules	8	1.80E-04	1.00E-02
	BIOCARTA	HIV Induced T Cell Apoptosis	7	2.00E-04	9.10E-03
	GOTERM_CC_FAT	alpha-beta T cell receptor complex	4	1.30E-03	3.30E-02
	BIOCARTA	IL 17 Signaling Pathway	7	6.20E-03	2.10E-01
	BIOCARTA	T Cell Receptor and CD3 Complex	4	6.70E-03	2.00E-01
	PIR_SUPERFAMILY	PIRSF001993:T-cell surface glycoprotein CD3 delta chain	3	1.60E-02	7.60E-01
	BIOCARTA	The Co-Stimulatory Signal During T-cell Activation	7	2.20E-02	4.00E-01
	BIOCARTA	Role of Tob in T-cell activation	5	8.30E-02	6.90E-01
	BIOCARTA	IL12 and Stat4 Dependent Signaling Pathway in Th1 Development	6	8.90E-02	6.80E-01
	BIOCARTA	T Cell Receptor Signaling Pathway	8	1.10E-01	7.40E-01
	BIOCARTA	NO2-dependent IL 12 Pathway in NK cells	4	2.10E-01	8.80E-01
**Annotation Cluster 25**	**Enrichment Score: 2.81**		**Count**	**P_Value**	**Benjamini**
	INTERPRO	Pleckstrin homology	34	5.80E-04	6.70E-02
	UP_SEQ_FEATURE	domain:PH	29	7.90E-04	1.40E-01
	INTERPRO	Pleckstrin homology-type	35	1.40E-03	1.30E-01
	SMART	PH	34	8.90E-03	3.50E-01
**Annotation Cluster 26**	**Enrichment Score: 2.78**		**Count**	**P_Value**	**Benjamini**
	GOTERM_MF_FAT	immunoglobulin binding	7	2.50E-04	2.10E-02
	SP_PIR_KEYWORDS	immunoglobulin receptor	6	3.00E-04	4.50E-03
	GOTERM_MF_FAT	IgG binding	5	1.10E-03	6.20E-02
	SP_PIR_KEYWORDS	igg-binding protein	4	6.80E-03	5.80E-02
	GOTERM_MF_FAT	immunoglobulin receptor activity	3	2.40E-02	4.10E-01
**Annotation Cluster 27**	**Enrichment Score: 2.76**		**Count**	**P_Value**	**Benjamini**

ILMN_1805271	ABCA11P	-0.412035845
ILMN_1764500	C3orf10	-0.412105742
ILMN_1764861	ISOC1	-0.412285975
ILMN_1760651	LOC253724	-0.412489243
ILMN_1714223	LETM2	-0.412532637
ILMN_1785107	NXT2	-0.412565658
ILMN_1715113	HARS2	-0.412694722
ILMN_1696935	RBM39	-0.412710201
ILMN_2112599	C16orf80	-0.412898746
ILMN_1690327	CLCN5	-0.412971979
ILMN_1724040	ANKRD57	-0.413002746
ILMN_3259884	SLC6A19	-0.413041194
ILMN_1681601	SUCNR1	-0.413116118
ILMN_1757882	PPP1R16A	-0.413160256
ILMN_2136971	FABP3	-0.413178929
ILMN_2228180	MSRA	-0.41318787
ILMN_1819991	AI792396	-0.413202571
ILMN_1793290	WDR60	-0.413209822
ILMN_1808305	RTCD1	-0.413232137
ILMN_1730401	LOC442421	-0.413299184
ILMN_1766038	C11orf88	-0.413299674
ILMN_1781073	PTPN5	-0.41330858
ILMN_2323979	WARS2	-0.41338098
ILMN_1750062	PPARGC1A	-0.413391875
ILMN_1687785	PPA2	-0.413537409
ILMN_1793508	C7orf36	-0.413640501
ILMN_1732269	GP6	-0.41365255
ILMN_1724671	MAP6	-0.413722238
ILMN_1836309	MCF2L-AS1	-0.413784593
ILMN_2131123	AMY2A	-0.4138108
ILMN_1911500	DA455367	-0.413987951
ILMN_1709570	IMMP1L	-0.414083268
ILMN_2333708	STAG2	-0.414111614
ILMN_1855966	DB338170	-0.414125184
ILMN_1733675	MPP1	-0.414186394
ILMN_1803094	PDGFD	-0.414236957
ILMN_1669338	CYP4X1	-0.414326018
ILMN_1812678	AGMO	-0.414406423
ILMN_3249439	ZNF878	-0.414880756
ILMN_1777439	TCL6	-0.415000777
ILMN_1679782	BLOC1S2	-0.415119368
ILMN_1697166	C9orf78	-0.415156462
ILMN_1702739	TMEM146	-0.415290665
ILMN_1749162	DIO2	-0.415310806
ILMN_1797281	RGPD6	-0.415328119
ILMN_1671542	NLGN4X	-0.41550828
ILMN_2323366	SDHC	-0.41556923
ILMN_1753449	CST1	-0.415611505
ILMN_2184525	LOC220115	-0.415612675
ILMN_1795419	CNPPD1	-0.415632858
ILMN_1762634	OR10K1	-0.415638956
ILMN_1675445	KIAA2018	-0.415645516
ILMN_1695092	WRB	-0.415929829
ILMN_2178994	TUBB8	-0.416205867
ILMN_1718018	PFN3	-0.416247448
ILMN_2141259	SPAG5	-0.41625448
ILMN_1720617	OR2T6	-0.416380076
ILMN_2103003	OR4N3P	-0.416425847
ILMN_2357062	IL1RAP	-0.416430998
ILMN_1839750	NA	-0.416452851
ILMN_2246661	FAHD1	-0.41651917
ILMN_1749403	TSPAN33	-0.4165722
ILMN_1689274	NIPA1	-0.416658367
ILMN_1737988	PRNP	-0.416707677
ILMN_1764850	HPCAL1	-0.416799331
ILMN_1695378	SREBF1	-0.416966257
ILMN_1681670	SLC25A4	-0.416985603
ILMN_1707551	AFMID	-0.416991933
ILMN_1799367	TMX2	-0.417294402
ILMN_1804248	FDPS	-0.417433376
ILMN_1807745	GYS2	-0.417468628
ILMN_3238123	ZNF844	-0.417660785
ILMN_2352917	LTBP4	-0.418059293
ILMN_1794989	TMEM191B	-0.418073062
ILMN_1675756	KCNJ15	-0.418222913
ILMN_1764457	HEATR7B1	-0.418243206
ILMN_1666718	NA	-0.418336767
ILMN_2351241	RNF14	-0.418373154
ILMN_1785095	ATP6V0E2	-0.418384892
ILMN_1784286	NDUFA1	-0.418425003
ILMN_1915913	BG722090	-0.418456263
ILMN_1766851	TMEM126B	-0.418466226
ILMN_1672057	AQP7	-0.418495413
ILMN_1795673	C9orf173	-0.418632659
ILMN_2334204	ERCC6L	-0.418679616
ILMN_1681374	ALDOA	-0.418708223
ILMN_1803318	PPFIA2	-0.418717525
ILMN_1680251	SORCS1	-0.418775027
ILMN_1903568	CR625988	-0.418788897
ILMN_1688971	NOL11	-0.418891842
ILMN_3300358	ZNF84	-0.418982521
ILMN_1675329	TBC1D3	-0.419033984
ILMN_2258977	XAGE1A	-0.419243926

	GOTERM_CC_FAT	membrane-enclosed lumen	118	3.70E-01	9.70E-01
	GOTERM_CC_FAT	organelle lumen	111	5.80E-01	9.90E-01
	GOTERM_CC_FAT	intracellular organelle lumen	107	6.50E-01	9.90E-01
	GOTERM_CC_FAT	nucleoplasm	39	9.90E-01	1.00E+00
	GOTERM_CC_FAT	nucleoplasm part	21	1.00E+00	1.00E+00
	GOTERM_CC_FAT	nuclear lumen	61	1.00E+00	1.00E+00
**Annotation Cluster 19**	**Enrichment Score: 2.21**		**Count**	**P_Value**	**Benjamini**
	GOTERM_MF_FAT	iron ion binding	39	1.30E-05	2.40E-03
	SP_PIR_KEYWORDS	iron	34	5.30E-05	3.20E-03
	KEGG_PATHWAY	Linoleic acid metabolism	9	5.00E-04	5.30E-03
	SP_PIR_KEYWORDS	metalloprotein	17	1.40E-03	4.10E-02
	COG_ONTOLOGY	Secondary metabolites biosynthesis, transport, and catabolism	12	2.50E-03	2.80E-02
	UP_SEQ_FEATURE	metal ion-binding site:Iron (heme axial ligand)	14	3.10E-03	5.60E-01
	GOTERM_MF_FAT	tetrapyrrole binding	17	3.90E-03	2.00E-01
	INTERPRO	Cytochrome P450, C-terminal region	10	4.30E-03	6.20E-01
	GOTERM_MF_FAT	heme binding	16	5.10E-03	2.30E-01
	INTERPRO	Cytochrome P450, E-class, group I	9	6.40E-03	6.70E-01
	GOTERM_MF_FAT	oxidoreductase activity, acting on paired donors, with incorporation or reduction of molecular oxygen, reduced flavin or flavoprotein as one donor, and incorporation of one atom of oxygen	7	7.40E-03	2.70E-01
	INTERPRO	Cytochrome P450	10	7.70E-03	6.70E-01
	INTERPRO	Cytochrome P450, conserved site	10	8.60E-03	6.50E-01
	SP_PIR_KEYWORDS	Monooxygenase	11	1.10E-02	1.90E-01
	GOTERM_MF_FAT	oxygen binding	8	1.20E-02	3.50E-01
	GOTERM_MF_FAT	aromatase activity	6	1.40E-02	3.60E-01
	SP_PIR_KEYWORDS	heme	14	1.70E-02	2.60E-01
	INTERPRO	Cytochrome P450, E-class, CYP3A	3	1.80E-02	7.80E-01
	SP_PIR_KEYWORDS	electron transfer	8	2.00E-02	2.70E-01
	INTERPRO	Cytochrome P450, E-class, group II	3	2.90E-02	8.80E-01
	PIR_SUPERFAMILY	PIRSF000051:cytochrome P450 CYP3A5	3	3.70E-02	1.00E+00
	SP_PIR_KEYWORDS	chromoprotein	8	5.50E-02	4.60E-01
	BIOCARTA	Nuclear Receptors in Lipid Metabolism and Toxicity	6	5.90E-02	9.50E-01
	PIR_SUPERFAMILY	PIRSF000045:cytochrome P450 CYP2D6	4	2.20E-01	1.00E+00
**Annotation Cluster 20**	**Enrichment Score: 2.15**		**Count**	**P_Value**	**Benjamini**
	GOTERM_BP_FAT	ion transport	65	9.60E-04	6.80E-02
	SP_PIR_KEYWORDS	ion transport	49	3.60E-03	8.70E-02
	GOTERM_BP_FAT	cation transport	47	5.00E-03	2.20E-01
	GOTERM_BP_FAT	monovalent inorganic cation transport	30	7.00E-03	2.70E-01
	GOTERM_BP_FAT	transmembrane transport	47	8.00E-03	2.90E-01
	SP_PIR_KEYWORDS	transport	115	1.20E-02	2.10E-01
	GOTERM_BP_FAT	metal ion transport	35	7.00E-02	7.50E-01
**Annotation Cluster 21**	**Enrichment Score: 2.08**		**Count**	**P_Value**	**Benjamini**
	GOTERM_BP_FAT	protein homotetramerization	7	2.70E-03	1.40E-01
	GOTERM_BP_FAT	protein tetramerization	8	5.50E-03	2.30E-01
	GOTERM_BP_FAT	protein homooligomerization	12	1.70E-02	4.10E-01
	GOTERM_BP_FAT	protein oligomerization	18	1.90E-02	4.20E-01
**Annotation Cluster 22**	**Enrichment Score: 2.03**		**Count**	**P_Value**	**Benjamini**
	GOTERM_BP_FAT	response to nutrient levels	23	1.60E-03	9.80E-02
	GOTERM_BP_FAT	response to extracellular stimulus	24	3.10E-03	1.50E-01
	GOTERM_BP_FAT	response to nutrient	12	1.60E-01	9.10E-01
**Annotation Cluster 23**	**Enrichment Score: 2.02**		**Count**	**P_Value**	**Benjamini**
	GOTERM_BP_FAT	protein complex biogenesis	45	2.50E-03	1.40E-01
	GOTERM_BP_FAT	protein complex assembly	45	2.50E-03	1.40E-01
	GOTERM_BP_FAT	protein oligomerization	18	1.90E-02	4.20E-01
	GOTERM_BP_FAT	macromolecular complex assembly	51	2.20E-02	4.40E-01
	GOTERM_BP_FAT	macromolecular complex subunit organization	53	3.00E-02	5.40E-01
**Annotation Cluster 24**	**Enrichment Score: 2.02**		**Count**	**P_Value**	**Benjamini**
	KEGG_PATHWAY	beta-Alanine metabolism	9	7.40E-05	1.10E-03
	KEGG_PATHWAY	Tryptophan metabolism	11	3.30E-04	3.80E-03
	KEGG_PATHWAY	Fatty acid metabolism	11	3.30E-04	3.80E-03
	GOTERM_BP_FAT	cellular aldehyde metabolic process	8	7.30E-04	5.60E-02
	KEGG_PATHWAY	Limonene and pinene degradation	6	2.00E-03	1.80E-02
	UP_SEQ_FEATURE	nucleotide phosphate-binding region:NAD	12	3.20E-03	5.40E-01
	KEGG_PATHWAY	Lysine degradation	9	1.10E-02	7.50E-02
	INTERPRO	Aldehyde dehydrogenase	5	1.70E-02	7.80E-01
	INTERPRO	Aldehyde dehydrogenase, N-terminal	5	2.10E-02	8.10E-01
	GOTERM_MF_FAT	oxidoreductase activity, acting on the aldehyde or oxo group of donors, NAD or NADP as acceptor	5	4.40E-02	6.10E-01
	INTERPRO	Aldehyde dehydrogenase, conserved site	5	7.40E-02	9.80E-01
	GOTERM_MF_FAT	aldehyde dehydrogenase (NAD) activity	3	7.70E-02	7.20E-01
	KEGG_PATHWAY	Histidine metabolism	5	1.40E-01	5.50E-01
	UP_SEQ_FEATURE	site:Transition state stabilizer	4	1.60E-01	1.00E+00
	KEGG_PATHWAY	Glycerolipid metabolism	4	6.20E-01	9.20E-01
**Annotation Cluster 25**	**Enrichment Score: 1.85**		**Count**	**P_Value**	**Benjamini**
	GOTERM_MF_FAT	oxidoreductase activity, acting on the CH-NH2 group of donors	6	3.20E-03	2.00E-01
	KEGG_PATHWAY	Glycine, serine and threonine metabolism	7	1.90E-02	1.20E-01
	GOTERM_MF_FAT	oxidoreductase activity, acting on the CH-NH2 group of donors, oxygen as acceptor	4	4.60E-02	6.10E-01
**Annotation Cluster 26**	**Enrichment Score: 1.83**		**Count**	**P_Value**	**Benjamini**
	GOTERM_MF_FAT	adenylate kinase activity	5	7.00E-04	7.30E-02
	INTERPRO	Adenylate kinase, subfamily	3	9.50E-03	6.30E-01
	INTERPRO	Adenylate kinase, zinc-finger lid region	3	9.50E-03	6.30E-01
	GOTERM_MF_FAT	nucleotide kinase activity	6	9.90E-03	3.10E-01
	INTERPRO	Adenylate kinase	4	1.20E-02	7.00E-01
	UP_SEQ_FEATURE	nucleotide phosphate-binding region:AMP	4	3.30E-02	9.80E-01
	UP_SEQ_FEATURE	binding site:AMP	4	3.30E-02	9.80E-01
	PIR_SUPERFAMILY	PIRSF000734:adenylate kinase	3	3.70E-02	1.00E+00
	GOTERM_MF_FAT	nucleobase, nucleoside, nucleotide kinase activity	7	4.00E-02	6.10E-01
	GOTERM_MF_FAT	phosphotransferase activity, phosphate group as acceptor	6	4.30E-02	6.20E-01
**Annotation Cluster 27**	**Enrichment Score: 1.83**		**Count**	**P_Value**	**Benjamini**
	INTERPRO	Crotonase, core	6	1.10E-03	3.60E-01
	INTERPRO	Enoyl-CoA hydratase/isomerase, conserved site	4	2.20E-02	8.10E-01
	GOTERM_MF_FAT	hydro-lyase activity	7	4.40E-02	6.10E-01
	GOTERM_MF_FAT	enoyl-CoA hydratase activity	3	4.50E-02	6.10E-01
**Annotation Cluster 28**	**Enrichment Score: 1.78**		**Count**	**P_Value**	**Benjamini**
	GOTERM_CC_FAT	proton-transporting two-sector ATPase complex	11	3.20E-04	9.20E-03
	GOTERM_CC_FAT	mitochondrial proton-transporting ATP synthase complex	4	1.10E-01	7.60E-01

ILMN_1745420	PHF19	0.571474481
ILMN_1670302	HK3	0.571359655
ILMN_1771599	PLOD2	0.571249153
ILMN_1799134	KLRD1	0.570544886
ILMN_1671565	RNASET2	0.570444106
ILMN_2415776	WWOX	0.570364587
ILMN_1792473	AIF1	0.570050113
ILMN_2222688	TMSB4X	0.569906652
ILMN_1658830	C10orf26	0.569689786
ILMN_1734895	SFT2D1	0.569510507
ILMN_1768595	DLG4	0.567313376
ILMN_3249667	HLA-DQA1	0.566883679
ILMN_1716300	CDH7	0.566453175
ILMN_1805027	KLC3	0.56619684
ILMN_1721035	MS4A6A	0.565646093
ILMN_1724533	LY96	0.565610408
ILMN_1660368	TRRAP	0.565596634
ILMN_1654319	HAPLN3	0.564580509
ILMN_1752592	HLA-DRB4	0.564545772
ILMN_1705876	NAP1L1	0.564249354
ILMN_1706643	COL6A3	0.563595273
ILMN_1747593	ISLR	0.563025501
ILMN_1669409	VSIG4	0.562592684
ILMN_1769245	GLIPR1	0.561555944
ILMN_2214232	AIM2	0.56120833
ILMN_1662026	BTK	0.560600139
ILMN_3192156	LOC100130932	0.560425184
ILMN_1709860	UNC45A	0.560357435
ILMN_1754121	CSK	0.5601164
ILMN_1762835	HELZ	0.560089485
ILMN_1815895	HLA-DRB1	0.559778002
ILMN_1717261	HLA-DRB1	0.559384372
ILMN_1762594	NOD2	0.558954219
ILMN_3303101	AK027616	0.558421261
ILMN_1668876	HECTD2	0.558139934
ILMN_1670256	PDK1	0.558113707
ILMN_1679929	KLF13	0.558057416
ILMN_1785060	TSPAN14	0.556692289
ILMN_1801766	CCDC109B	0.556631343
ILMN_2168217	GPR183	0.556573899
ILMN_2203950	HLA-A	0.556011697
ILMN_2400030	PTPN2	0.555329444
ILMN_1674250	NCKAP1L	0.555059935
ILMN_1687501	MOXD1	0.554897839
ILMN_2388090	MAPK14	0.554487579
ILMN_1783593	CCL13	0.554428477
ILMN_2167805	LUM	0.553872206
ILMN_1677589	hCG_1818547	0.553810404
ILMN_2391458	ACSL4	0.553352478
ILMN_1724686	CLDN1	0.553200079
ILMN_1792951	ZHX2	0.553154741
ILMN_1660663	DYRK1A	0.552581458
ILMN_1797341	ARID1A	0.552265806
ILMN_1758250	TRAFD1	0.551326923
ILMN_1811507	LBH	0.550441215
ILMN_1745242	PLSCR1	0.550350226
ILMN_1672661	SP110	0.548912626
ILMN_1693552	CD300A	0.548010154
ILMN_2323491	NUP62	0.547993647
ILMN_2188333	CD69	0.547825656
ILMN_1799814	SNRNP40	0.547342602
ILMN_2348808	SNX16	0.546927292
ILMN_2163819	KIF21B	0.546785886
ILMN_1656904	SLC1A4	0.546160788
ILMN_1712389	CMTM1	0.545967899
ILMN_1659913	ISG20	0.545914993
ILMN_1687998	LPGAT1	0.545400128
ILMN_1680996	ALOX5	0.545290206
ILMN_1688780	S100A4	0.544819144
ILMN_1710923	SLAMF7	0.544763641
ILMN_1727761	GMEB1	0.544616845
ILMN_1735910	VMO1	0.544347822
ILMN_1769550	SLFN5	0.544299426
ILMN_1695851	PARVG	0.543985179
ILMN_1701596	RPS20	0.543871166
ILMN_2067890	CXCL11	0.543748383
ILMN_1693452	GAL3ST4	0.543376471
ILMN_1791211	DOK2	0.543113702
ILMN_1727043	GLT25D1	0.543033038
ILMN_1679185	LEF1	0.542942573
ILMN_1703538	AIF1	0.542906857
ILMN_1701308	COL1A1	0.542835996
ILMN_2067656	CCND2	0.542686167
ILMN_1692177	TSC22D1	0.542510464
ILMN_2154115	PSD4	0.542472245
ILMN_1744212	INPP5D	0.542365844
ILMN_1712687	PAK2	0.542360455
ILMN_1690268	HNRNPUL1	0.542316058
ILMN_1736567	CD74	0.542245205

	SP_PIR_KEYWORDS	SH2 domain	18	4.40E-04	6.30E-03
	UP_SEQ_FEATURE	domain:SH2	16	8.10E-04	1.30E-01
	INTERPRO	SH2 motif	17	2.20E-03	1.90E-01
	SMART	SH2	17	1.20E-02	3.50E-01
**Annotation Cluster 28**	**Enrichment Score: 2.64**		**Count**	**P_Value**	**Benjamini**
	GOTERM_BP_FAT	regulation of cytokine production	29	4.60E-05	2.60E-03
	GOTERM_BP_FAT	positive regulation of cytokine production	14	8.60E-03	1.80E-01
	GOTERM_BP_FAT	positive regulation of multicellular organismal process	26	3.10E-02	4.00E-01
**Annotation Cluster 29**	**Enrichment Score: 2.57**		**Count**	**P_Value**	**Benjamini**
	GOTERM_BP_FAT	vasculature development	34	2.60E-04	1.10E-02
	GOTERM_BP_FAT	blood vessel development	33	3.60E-04	1.50E-02
	GOTERM_BP_FAT	blood vessel morphogenesis	25	1.10E-02	2.10E-01
	GOTERM_BP_FAT	angiogenesis	17	4.90E-02	5.10E-01
**Annotation Cluster 30**	**Enrichment Score: 2.52**		**Count**	**P_Value**	**Benjamini**
	GOTERM_BP_FAT	cell activation during immune response	10	5.90E-04	2.30E-02
	GOTERM_BP_FAT	leukocyte activation during immune response	10	5.90E-04	2.30E-02
	GOTERM_BP_FAT	myeloid leukocyte activation	11	9.70E-04	3.50E-02
	GOTERM_BP_FAT	myeloid cell activation during immune response	6	6.20E-03	1.40E-01
	GOTERM_BP_FAT	lymphocyte activation during immune response	4	1.20E-01	7.50E-01
**Annotation Cluster 31**	**Enrichment Score: 2.48**		**Count**	**P_Value**	**Benjamini**
	GOTERM_BP_FAT	T cell selection	8	1.80E-04	7.60E-03
	GOTERM_BP_FAT	thymic T cell selection	6	1.30E-03	4.20E-02
	GOTERM_BP_FAT	positive T cell selection	5	2.10E-03	6.60E-02
	GOTERM_BP_FAT	negative T cell selection	5	2.10E-03	6.60E-02
	GOTERM_BP_FAT	positive thymic T cell selection	4	9.30E-03	1.90E-01
	GOTERM_BP_FAT	negative thymic T cell selection	4	1.40E-02	2.50E-01
	GOTERM_BP_FAT	T cell differentiation in the thymus	6	3.50E-02	4.30E-01
**Annotation Cluster 32**	**Enrichment Score: 2.43**		**Count**	**P_Value**	**Benjamini**
	GOTERM_BP_FAT	activation of immune response	27	5.50E-10	1.60E-07
	GOTERM_BP_FAT	innate immune response	27	2.30E-06	2.00E-04
	GOTERM_BP_FAT	humoral immune response	19	5.30E-06	4.20E-04
	SP_PIR_KEYWORDS	innate immunity	17	1.80E-05	4.70E-04
	GOTERM_BP_FAT	complement activation	10	1.90E-03	6.10E-02
	GOTERM_BP_FAT	activation of plasma proteins involved in acute inflammatory response	10	2.30E-03	6.90E-02
	GOTERM_BP_FAT	protein maturation by peptide bond cleavage	14	5.90E-03	1.40E-01
	SP_PIR_KEYWORDS	complement alternate pathway	5	6.60E-03	5.70E-02
	GOTERM_BP_FAT	complement activation, alternative pathway	5	1.70E-02	2.80E-01
	SP_PIR_KEYWORDS	complement pathway	6	3.00E-02	1.80E-01
	GOTERM_BP_FAT	acute inflammatory response	13	3.70E-02	4.40E-01
	GOTERM_BP_FAT	protein maturation	15	4.20E-02	4.70E-01
	GOTERM_BP_FAT	protein processing	14	4.50E-02	4.80E-01
	GOTERM_BP_FAT	complement activation, classical pathway	6	4.60E-02	4.90E-01
	KEGG_PATHWAY	Complement and coagulation cascades	11	5.20E-02	2.20E-01
	GOTERM_BP_FAT	humoral immune response mediated by circulating immunoglobulin	6	5.90E-02	5.60E-01
	SP_PIR_KEYWORDS	plasma	10	1.20E-01	4.70E-01
	BIOCARTA	Complement Pathway	5	1.50E-01	8.10E-01
	BIOCARTA	Alternative Complement Pathway	3	3.50E-01	9.50E-01
	BIOCARTA	Classical Complement Pathway	3	4.50E-01	9.70E-01
**Annotation Cluster 33**	**Enrichment Score: 2.34**		**Count**	**P_Value**	**Benjamini**
	GOTERM_BP_FAT	regulation of apoptosis	80	8.00E-04	2.90E-02
	GOTERM_BP_FAT	regulation of programmed cell death	80	1.10E-03	3.90E-02
	GOTERM_BP_FAT	regulation of cell death	80	1.20E-03	4.10E-02
	GOTERM_BP_FAT	induction of apoptosis	37	2.50E-03	7.50E-02
	GOTERM_BP_FAT	induction of programmed cell death	37	2.70E-03	7.80E-02
	GOTERM_BP_FAT	positive regulation of apoptosis	46	3.30E-03	9.10E-02
	GOTERM_BP_FAT	positive regulation of programmed cell death	46	3.80E-03	9.90E-02
	GOTERM_BP_FAT	anti-apoptosis	26	4.20E-03	1.00E-01
	GOTERM_BP_FAT	positive regulation of cell death	46	4.20E-03	1.00E-01
	GOTERM_BP_FAT	negative regulation of apoptosis	36	2.00E-02	3.10E-01
	GOTERM_BP_FAT	induction of apoptosis by extracellular signals	15	2.20E-02	3.30E-01
	GOTERM_BP_FAT	negative regulation of programmed cell death	36	2.40E-02	3.50E-01
	GOTERM_BP_FAT	negative regulation of cell death	36	2.50E-02	3.60E-01
**Annotation Cluster 34**	**Enrichment Score: 2.33**		**Count**	**P_Value**	**Benjamini**
	SP_PIR_KEYWORDS	sh3 domain	26	1.20E-03	1.60E-02
	UP_SEQ_FEATURE	domain:SH3	23	1.40E-03	2.00E-01
	INTERPRO	Src homology-3 domain	25	6.20E-03	3.60E-01
	SMART	SH3	25	4.20E-02	5.90E-01
**Annotation Cluster 35**	**Enrichment Score: 2.33**		**Count**	**P_Value**	**Benjamini**
	UP_SEQ_FEATURE	glycosylation site:O-linked (Gal...)	7	4.00E-06	1.20E-03
	SP_PIR_KEYWORDS	hydroxylation	17	5.00E-06	1.80E-04
	SP_PIR_KEYWORDS	hydroxylysine	11	1.00E-05	2.90E-04
	SP_PIR_KEYWORDS	triple helix	11	1.00E-05	2.90E-04
	SP_PIR_KEYWORDS	hydroxyproline	11	5.80E-05	1.10E-03
	SP_PIR_KEYWORDS	trimer	9	1.20E-04	2.10E-03
	SP_PIR_KEYWORDS	collagen	17	2.10E-04	3.30E-03
	GOTERM_CC_FAT	collagen	10	4.80E-04	1.60E-02
	INTERPRO	Collagen triple helix repeat	15	1.00E-03	1.10E-01
	SP_PIR_KEYWORDS	pyroglutamic acid	10	1.70E-03	2.10E-02
	GOTERM_CC_FAT	fibrillar collagen	5	7.20E-03	1.30E-01
	UP_SEQ_FEATURE	propeptide:C-terminal propeptide	4	1.10E-02	6.30E-01
	GOTERM_MF_FAT	extracellular matrix structural constituent	13	1.10E-02	3.00E-01
	UP_SEQ_FEATURE	region of interest:Triple-helical region	6	1.20E-02	6.50E-01
	GOTERM_BP_FAT	collagen fibril organization	7	1.30E-02	2.40E-01
	SP_PIR_KEYWORDS	Ehlers-Danlos syndrome	4	2.70E-02	1.70E-01
	UP_SEQ_FEATURE	domain:Fibrillar collagen NC1	4	2.70E-02	8.00E-01
	INTERPRO	Fibrillar collagen, C-terminal	4	3.10E-02	6.20E-01
	GOTERM_MF_FAT	platelet-derived growth factor binding	4	3.20E-02	4.90E-01
	SP_PIR_KEYWORDS	skin	3	3.40E-02	2.00E-01
	GOTERM_BP_FAT	collagen metabolic process	6	4.00E-02	4.60E-01

ILMN_1709256	PLEC	-0.419293542
ILMN_2228938	ECM2	-0.419315448
ILMN_1751040	RAB23	-0.419380993
ILMN_1813400	CBR4	-0.419415233
ILMN_2207170	WWC2	-0.419421284
ILMN_1862424	AW292223	-0.419565674
ILMN_1724052	ZNF814	-0.419676286
ILMN_1786444	LPL	-0.419689072
ILMN_3297880	SEC13	-0.419722524
ILMN_2284400	VSX1	-0.419730064
ILMN_1675671	ICOSLG	-0.419740496
ILMN_1677723	ANGPT1	-0.419748572
ILMN_1773650	LRRN3	-0.419821629
ILMN_1664592	C1orf95	-0.419979299
ILMN_1813427	FLJ41327	-0.419980234
ILMN_1876151	CB305890	-0.420086655
ILMN_1806052	UNC119	-0.420156215
ILMN_1738246	OR10A7	-0.42018301
ILMN_1817816	BC032017	-0.420256514
ILMN_3184048	FAM115C	-0.420269728
ILMN_1786273	C1orf122	-0.420325475
ILMN_2415157	ARID5A	-0.420530292
ILMN_1755638	TAF7L	-0.420573186
ILMN_1701127	CC2D1A	-0.42057836
ILMN_2353030	GRAP	-0.420753714
ILMN_1797965	PDP2	-0.420937165
ILMN_1754149	LETMD1	-0.421024472
ILMN_1783337	DECR2	-0.42108193
ILMN_1806778	UBE2E1	-0.421118323
ILMN_2178509	OR52A5	-0.421187724
ILMN_1688218	RASGRP4	-0.421274314
ILMN_1784967	EPB41L4B	-0.421279242
ILMN_1736103	ITPR2	-0.42138505
ILMN_1720595	MDGA1	-0.421568182
ILMN_1681456	FOXL2	-0.421666354
ILMN_1791884	SNHG11	-0.421680721
ILMN_1801476	CDS1	-0.421707342
ILMN_1888359	BMPR1B	-0.421752432
ILMN_1713397	NCCRP1	-0.421780587
ILMN_1768867	AP3B1	-0.421791176
ILMN_1874678	BQ050540	-0.421826256
ILMN_1788625	EXOC5	-0.42188295
ILMN_2102951	FAHD2A	-0.421898738
ILMN_1833027	AI936635	-0.422008295
ILMN_1729225	SORCS2	-0.422052526
ILMN_1806710	ESPN	-0.422078283
ILMN_1730319	ZSCAN18	-0.422100433
ILMN_1690389	OR10AD1	-0.422187969
ILMN_1763202	LOC441179	-0.422209888
ILMN_1806186	P2RX3	-0.422252626
ILMN_1853093	BG325888	-0.422347507
ILMN_1718924	ETFA	-0.422362239
ILMN_1697176	GFAP	-0.422540883
ILMN_1814206	HTR1B	-0.422660353
ILMN_1745811	TDRD3	-0.422777181
ILMN_1678186	ATP6V0A4	-0.422812939
ILMN_1683575	TMLHE	-0.422844174
ILMN_1752810	LARP6	-0.422952918
ILMN_2347888	LARP4	-0.423034642
ILMN_1659312	PMP22	-0.423064273
ILMN_1675687	NA	-0.423146226
ILMN_1714197	ACSS2	-0.423428779
ILMN_1666364	COQ10A	-0.423537928
ILMN_1801403	DCUN1D4	-0.423574436
ILMN_1802223	ATP2B2	-0.423612645
ILMN_1699334	AATK	-0.423725586
ILMN_1737162	LGI3	-0.423837297
ILMN_1764186	ATXN1L	-0.423990837
ILMN_1795704	KIAA0232	-0.423999175
ILMN_1764609	PWWP2B	-0.424093555
ILMN_1710075	FAM89A	-0.424186202
ILMN_2376263	SMARCA1	-0.424502813
ILMN_1738268	IP6K3	-0.42460081
ILMN_2173740	ASB8	-0.424648513
ILMN_3241149	TMEM236	-0.424716247
ILMN_1704943	GPN3	-0.424737165
ILMN_1730646	WDR87	-0.424945422
ILMN_1659010	AK124016	-0.425020863
ILMN_1781472	CDC42BPA	-0.425100232
ILMN_2062754	SRA1	-0.425105335
ILMN_1729095	PDZD2	-0.425240963
ILMN_1742534	COL4A5	-0.425589755
ILMN_2168903	FAM47B	-0.425614338
ILMN_1798659	CCDC28A	-0.425625902
ILMN_3225624	TATDN1	-0.425630391
ILMN_2201533	C17orf61	-0.425646709
ILMN_1700202	TMEM135	-0.425717608
ILMN_1804834	C6orf130	-0.425747222
ILMN_1719471	MSH3	-0.425760321

	GOTERM_CC_FAT	proton-transporting ATP synthase complex	4	1.30E-01	8.10E-01
**Annotation Cluster 29**	**Enrichment Score: 1.7**		**Count**	**P_Value**	**Benjamini**
	GOTERM_MF_FAT	ligase activity, forming carbon-sulfur bonds	7	4.30E-03	2.10E-01
	GOTERM_MF_FAT	acid-thiol ligase activity	6	5.30E-03	2.30E-01
	GOTERM_MF_FAT	CoA-ligase activity	3	7.70E-02	7.20E-01
	INTERPRO	AMP-dependent synthetase and ligase	5	9.10E-02	9.90E-01
**Annotation Cluster 30**	**Enrichment Score: 1.64**		**Count**	**P_Value**	**Benjamini**
	GOTERM_BP_FAT	oxidative phosphorylation	18	3.00E-05	4.90E-03
	GOTERM_CC_FAT	proton-transporting two-sector ATPase complex	11	3.20E-04	9.20E-03
	GOTERM_BP_FAT	nitrogen compound biosynthetic process	35	3.60E-04	3.30E-02
	GOTERM_BP_FAT	hydrogen transport	12	7.00E-04	5.50E-02
	GOTERM_MF_FAT	hydrogen ion transmembrane transporter activity	15	7.80E-04	7.40E-02
	SP_PIR_KEYWORDS	Hydrogen ion transport	10	1.10E-03	3.80E-02
	GOTERM_BP_FAT	energy coupled proton transport, down electrochemical gradient	9	1.50E-03	9.50E-02
	GOTERM_BP_FAT	ATP synthesis coupled proton transport	9	1.50E-03	9.50E-02
	GOTERM_BP_FAT	proton transport	11	2.00E-03	1.10E-01
	GOTERM_MF_FAT	monovalent inorganic cation transmembrane transporter activity	15	3.20E-03	2.10E-01
	GOTERM_MF_FAT	inorganic cation transmembrane transporter activity	19	3.50E-03	1.90E-01
	GOTERM_BP_FAT	purine nucleoside triphosphate biosynthetic process	14	3.60E-03	1.70E-01
	GOTERM_BP_FAT	ATP biosynthetic process	13	4.00E-03	1.90E-01
	GOTERM_BP_FAT	nucleoside triphosphate biosynthetic process	14	4.60E-03	2.10E-01
	GOTERM_BP_FAT	ion transmembrane transport	9	5.60E-03	2.30E-01
	GOTERM_CC_FAT	proton-transporting V-type ATPase complex	6	6.10E-03	1.40E-01
	GOTERM_BP_FAT	purine ribonucleoside triphosphate biosynthetic process	13	8.80E-03	3.00E-01
	GOTERM_BP_FAT	ribonucleoside triphosphate biosynthetic process	13	9.50E-03	3.10E-01
	GOTERM_BP_FAT	ribonucleotide biosynthetic process	15	9.80E-03	3.10E-01
	GOTERM_BP_FAT	nucleobase, nucleoside, nucleotide and nucleic acid biosynthetic process	20	1.30E-02	3.50E-01
	GOTERM_BP_FAT	nucleobase, nucleoside and nucleotide biosynthetic process	20	1.30E-02	3.50E-01
	GOTERM_BP_FAT	purine ribonucleotide biosynthetic process	14	1.40E-02	3.80E-01
	GOTERM_BP_FAT	ATP metabolic process	13	1.50E-02	3.90E-01
	GOTERM_BP_FAT	nucleotide biosynthetic process	19	1.70E-02	4.20E-01
	GOTERM_BP_FAT	purine nucleotide biosynthetic process	16	1.90E-02	4.30E-01
	GOTERM_BP_FAT	purine nucleoside triphosphate metabolic process	14	2.00E-02	4.20E-01
	GOTERM_CC_FAT	vacuolar proton-transporting V-type ATPase complex	4	2.00E-02	3.30E-01
	GOTERM_CC_FAT	proton-transporting two-sector ATPase complex, catalytic domain	5	2.20E-02	3.40E-01
	GOTERM_BP_FAT	purine ribonucleoside triphosphate metabolic process	13	3.20E-02	5.50E-01
	GOTERM_BP_FAT	nucleoside triphosphate metabolic process	14	3.30E-02	5.60E-01
	GOTERM_BP_FAT	purine nucleotide metabolic process	18	3.30E-02	5.50E-01
	GOTERM_BP_FAT	ribonucleoside triphosphate metabolic process	13	3.40E-02	5.50E-01
	GOTERM_BP_FAT	ribonucleotide metabolic process	15	3.70E-02	5.70E-01
	GOTERM_MF_FAT	proton-transporting ATPase activity, rotational mechanism	5	4.40E-02	6.10E-01
	GOTERM_BP_FAT	purine ribonucleotide metabolic process	14	4.70E-02	6.30E-01
	GOTERM_MF_FAT	cation-transporting ATPase activity	6	5.90E-02	6.90E-01
	GOTERM_BP_FAT	energy coupled proton transport, against electrochemical gradient	3	8.70E-02	7.90E-01
	GOTERM_BP_FAT	ATP hydrolysis coupled proton transport	3	8.70E-02	7.90E-01
	KEGG_PATHWAY	Vibrio cholerae infection	8	9.60E-02	4.40E-01
	GOTERM_BP_FAT	regulation of pH	5	1.20E-01	8.60E-01
	GOTERM_MF_FAT	ATPase activity, coupled to transmembrane movement of ions	8	1.50E-01	8.50E-01
	GOTERM_CC_FAT	proton-transporting two-sector ATPase complex, proton-transporting domain	4	1.80E-01	8.60E-01
	GOTERM_MF_FAT	hydrolase activity, acting on acid anhydrides, catalyzing transmembrane movement of substances	10	2.10E-01	9.10E-01
	GOTERM_MF_FAT	hydrogen ion transporting ATP synthase activity, rotational mechanism	3	2.40E-01	9.30E-01
	GOTERM_BP_FAT	monovalent inorganic cation homeostasis	5	2.50E-01	9.60E-01
	GOTERM_CC_FAT	vacuolar membrane	6	2.60E-01	9.20E-01
	GOTERM_MF_FAT	P-P-bond-hydrolysis-driven transmembrane transporter activity	10	3.00E-01	9.50E-01
	GOTERM_MF_FAT	primary active transmembrane transporter activity	10	3.00E-01	9.50E-01
	KEGG_PATHWAY	Epithelial cell signaling in Helicobacter pylori infection	7	3.40E-01	7.80E-01
	GOTERM_CC_FAT	vacuolar part	6	3.60E-01	9.60E-01
	GOTERM_MF_FAT	ATPase activity, coupled to transmembrane movement of substances	8	4.70E-01	9.90E-01
	GOTERM_MF_FAT	ATPase activity, coupled to movement of substances	8	4.80E-01	9.90E-01
	GOTERM_MF_FAT	ATPase activity	21	5.10E-01	9.90E-01
	GOTERM_MF_FAT	ATPase activity, coupled	17	5.50E-01	9.90E-01
	GOTERM_MF_FAT	ATPase activity, coupled to transmembrane movement of ions, phosphorylative mechanism	3	8.30E-01	1.00E+00
**Annotation Cluster 31**	**Enrichment Score: 1.61**		**Count**	**P_Value**	**Benjamini**
	GOTERM_BP_FAT	response to insulin stimulus	16	4.50E-04	3.80E-02
	GOTERM_BP_FAT	response to peptide hormone stimulus	19	2.60E-03	1.40E-01
	GOTERM_BP_FAT	cellular response to insulin stimulus	9	3.60E-02	5.60E-01
	GOTERM_BP_FAT	response to hormone stimulus	30	4.00E-02	5.80E-01
	GOTERM_BP_FAT	response to endogenous stimulus	32	5.00E-02	6.40E-01
	GOTERM_BP_FAT	response to organic substance	47	2.00E-01	9.40E-01
	GOTERM_BP_FAT	cellular response to hormone stimulus	10	3.30E-01	9.80E-01
**Annotation Cluster 32**	**Enrichment Score: 1.57**		**Count**	**P_Value**	**Benjamini**
	GOTERM_BP_FAT	glutamate metabolic process	5	1.10E-02	3.20E-01
	GOTERM_BP_FAT	dicarboxylic acid metabolic process	7	1.10E-02	3.30E-01
	KEGG_PATHWAY	Alanine, aspartate and glutamate metabolism	5	1.70E-01	6.10E-01
**Annotation Cluster 33**	**Enrichment Score: 1.57**		**Count**	**P_Value**	**Benjamini**
	GOTERM_BP_FAT	regulation of hormone levels	17	1.10E-02	3.30E-01
	GOTERM_BP_FAT	hormone metabolic process	13	1.60E-02	3.90E-01
	GOTERM_BP_FAT	cellular hormone metabolic process	7	1.10E-01	8.50E-01
**Annotation Cluster 34**	**Enrichment Score: 1.52**		**Count**	**P_Value**	**Benjamini**
	GOTERM_BP_FAT	glutamate metabolic process	5	1.10E-02	3.20E-01
	GOTERM_BP_FAT	glutamine family amino acid metabolic process	8	2.60E-02	4.90E-01
	GOTERM_BP_FAT	glutamine family amino acid catabolic process	4	9.90E-02	8.30E-01

ILMN_2364521	AXL	0.542158719
ILMN_1653220	PITPNM1	0.542033299
ILMN_1711894	MYB	0.541942618
ILMN_2395711	ARRB2	0.541905357
ILMN_1737683	FGG	0.541293955
ILMN_1733937	MMD	0.541175792
ILMN_2060413	CD24	0.540923355
ILMN_2088124	TMEM154	0.54081551
ILMN_3209399	KRT18	0.540552229
ILMN_3192001	CCL15	0.540354123
ILMN_1766275	PIK3CD	0.540185995
ILMN_2413622	RFX4	0.539983118
ILMN_1688698	ZEB2	0.539498138
ILMN_1710434	TBC1D10C	0.539326381
ILMN_1798006	ANKRD35	0.539324171
ILMN_1717101	MINK1	0.539255605
ILMN_1661839	OR52A5	0.539195389
ILMN_2125747	CORO1A	0.538811741
ILMN_1693338	CYP1B1	0.538794159
ILMN_1769911	SLC38A1	0.538354576
ILMN_1775542	FAIM3	0.538105777
ILMN_1742230	BAZ1A	0.537948112
ILMN_2365465	XBP1	0.537815715
ILMN_1763091	C14orf43	0.537813281
ILMN_1680403	SSR4	0.537692812
ILMN_1803392	P2RX5-TAX1BP3	0.53767956
ILMN_2313079	NLRP1	0.537576315
ILMN_2282641	TBXAS1	0.537496085
ILMN_1713749	CORO1A	0.537065883
ILMN_1737964	HIATL1	0.537062767
ILMN_1681798	sept-06	0.535288615
ILMN_1657153	ACTR3	0.535131758
ILMN_1669447	PYHIN1	0.535131608
ILMN_1770247	HOXA10-HOXA9	0.534469473
ILMN_1765557	OLFML2B	0.534281024
ILMN_1898605	BX119059	0.533822264
ILMN_3253579	HAUS8	0.533708741
ILMN_1718936	LST1	0.533685528
ILMN_1701441	LPAR1	0.533584285
ILMN_1696704	TLE4	0.533575509
ILMN_1658327	BAZ1A	0.533441732
ILMN_1761463	EFHD2	0.533391753
ILMN_1673708	HDAC9	0.533243883
ILMN_2059505	ARPP19	0.533095609
ILMN_1803825	CXCL12	0.532957472
ILMN_3236825	RAPGEF5	0.532957273
ILMN_1691476	MYLK	0.532906275
ILMN_1732705	HCFC1	0.532655329
ILMN_1740609	CCL15	0.532083263
ILMN_2388605	ACTR2	0.531872163
ILMN_2112580	FCGR3A	0.531607296
ILMN_2301624	MACF1	0.531190638
ILMN_2361427	PMS2P3	0.530463908
ILMN_1812973	RRP1B	0.529984363
ILMN_2375825	CD37	0.529971865
ILMN_2201678	FSTL1	0.529042966
ILMN_2094360	NR2F2	0.528995672
ILMN_2355953	LILRB4	0.528635646
ILMN_1764788	TNFRSF1B	0.528559586
ILMN_2109489	GZMB	0.528278857
ILMN_1797813	SUZ12	0.528141197
ILMN_1739103	MPZL1	0.5281348
ILMN_1728934	PRC1	0.527854316
ILMN_2228035	HS3ST1	0.527755219
ILMN_1784774	P2RY10	0.527700179
ILMN_1717334	VAV1	0.527665251
ILMN_3248562	SNORD49A	0.52766457
ILMN_1779257	CD40	0.527627596
ILMN_2401978	STAT3	0.526758962
ILMN_1747386	GLIS3	0.526051034
ILMN_1676528	BTN3A2	0.525696218
ILMN_1813314	HIST1H2BK	0.525667946
ILMN_1653828	CHFR	0.525570339
ILMN_1861376	AL080095	0.525553332
ILMN_1746604	IL6ST	0.525318987
ILMN_1709486	SRPX	0.524927756
ILMN_1683026	PSMB10	0.524529619
ILMN_1652366	NLRP7	0.523767182
ILMN_1800302	LRTM1	0.523125588
ILMN_1780533	RNASE6	0.523036373
ILMN_1738424	CDC42	0.522741055
ILMN_1734205	RASSF1	0.522728233
ILMN_1736178	AEBP1	0.522717341
ILMN_1719835	OSCP1	0.522704637
ILMN_3183573	NA	0.522653131
ILMN_1677997	MAZ	0.522238876
ILMN_1730842	PTPRC	0.521576258
ILMN_1742332	KCTD12	0.521545558
ILMN_1749345	STX5	0.521353791
ILMN_3229033	TDG	0.520737908
ILMN_1809488	SPCS2	0.520711651
ILMN_2116714	SLC39A1	0.520560078
ILMN_2391912	SEC14L1	0.520405383
ILMN_2080342	LGALS9C	0.520398144
ILMN_1669881	TSPAN13	0.520298317
ILMN_1760858	RAB8A	0.52012829
ILMN_1782538	VIM	0.51992954
ILMN_2082244	FOXK1	0.519539139

	GOTERM_BP_FAT	collagen biosynthetic process	3	4.10E-02	4.70E-01
	GOTERM_BP_FAT	skin development	6	4.60E-02	4.90E-01
	PIR_SUPERFAMILY	PIRSF002255:collagen alpha 1(I) chain	3	4.80E-02	9.50E-01
	SMART	COLFI	4	4.80E-02	6.20E-01
	UP_SEQ_FEATURE	propeptide:N-terminal propeptide	3	4.90E-02	9.20E-01
	GOTERM_BP_FAT	multicellular organismal macromolecule metabolic process	6	5.90E-02	5.60E-01
	UP_SEQ_FEATURE	region of interest:Nonhelical region	3	8.40E-02	9.80E-01
	UP_SEQ_FEATURE	domain:VWFA 2	4	8.40E-02	9.80E-01
	GOTERM_BP_FAT	multicellular organismal metabolic process	6	1.10E-01	7.20E-01
	UP_SEQ_FEATURE	domain:VWFA 1	3	2.60E-01	1.00E+00
**Annotation Cluster 36**	**Enrichment Score: 2.24**		**Count**	**P_Value**	**Benjamini**
	KEGG_PATHWAY	Fc epsilon RI signaling pathway	17	4.50E-04	3.20E-03
	KEGG_PATHWAY	B cell receptor signaling pathway	15	2.60E-03	1.50E-02
	KEGG_PATHWAY	VEGF signaling pathway	10	1.60E-01	4.70E-01
**Annotation Cluster 37**	**Enrichment Score: 2.19**		**Count**	**P_Value**	**Benjamini**
	UP_SEQ_FEATURE	domain:Fibrinogen C-terminal	8	3.00E-03	3.10E-01
	INTERPRO	Fibrinogen, alpha/beta/gamma chain, C-terminal globular	8	3.90E-03	2.80E-01
	SMART	FBG	8	1.00E-02	3.40E-01
	INTERPRO	Fibrinogen, alpha/beta/gamma chain, C-terminal globular, subdomain 1	6	1.50E-02	4.90E-01
**Annotation Cluster 38**	**Enrichment Score: 2.03**		**Count**	**P_Value**	**Benjamini**
	GOTERM_BP_FAT	regulation of inflammatory response	14	1.90E-03	6.10E-02
	GOTERM_BP_FAT	positive regulation of defense response	13	4.00E-03	1.00E-01
	GOTERM_BP_FAT	regulation of response to external stimulus	21	6.60E-03	1.50E-01
	GOTERM_BP_FAT	positive regulation of acute inflammatory response	5	7.10E-03	1.60E-01
	GOTERM_BP_FAT	positive regulation of response to external stimulus	11	1.20E-02	2.20E-01
	GOTERM_BP_FAT	regulation of acute inflammatory response	6	1.20E-02	2.30E-01
	GOTERM_BP_FAT	positive regulation of inflammatory response	7	1.50E-02	2.60E-01
	SP_PIR_KEYWORDS	inflammation	5	6.50E-02	3.10E-01
**Annotation Cluster 39**	**Enrichment Score: 1.96**		**Count**	**P_Value**	**Benjamini**
	SP_PIR_KEYWORDS	tyrosine-specific protein kinase	10	2.40E-03	2.70E-02
	SP_PIR_KEYWORDS	transforming protein	8	4.10E-03	3.90E-02
	SP_PIR_KEYWORDS	myristylation	8	9.00E-03	7.50E-02
	GOTERM_MF_FAT	non-membrane spanning protein tyrosine kinase activity	8	1.70E-02	3.80E-01
	SP_PIR_KEYWORDS	thiolester bond	7	2.20E-02	1.40E-01
	PIR_SUPERFAMILY	PIRSF000601:tyrosine-protein kinase, proto-oncogene SRC type	4	5.50E-02	9.40E-01
**Annotation Cluster 40**	**Enrichment Score: 1.94**		**Count**	**P_Value**	**Benjamini**
	GOTERM_CC_FAT	cell cortex	21	2.60E-03	6.00E-02
	GOTERM_CC_FAT	cell cortex part	13	1.20E-02	1.90E-01
	GOTERM_CC_FAT	cortical cytoskeleton	8	5.00E-02	5.00E-01
**Annotation Cluster 41**	**Enrichment Score: 1.93**		**Count**	**P_Value**	**Benjamini**
	GOTERM_BP_FAT	negative regulation of macromolecule metabolic process	73	1.40E-03	4.70E-02
	SP_PIR_KEYWORDS	repressor	43	2.70E-03	3.00E-02
	GOTERM_BP_FAT	negative regulation of RNA metabolic process	40	3.70E-03	9.70E-02
	GOTERM_BP_FAT	negative regulation of nitrogen compound metabolic process	52	6.50E-03	1.50E-01
	GOTERM_BP_FAT	negative regulation of transcription, DNA-dependent	38	8.10E-03	1.70E-01
	GOTERM_BP_FAT	negative regulation of transcription	46	1.00E-02	2.10E-01
	GOTERM_BP_FAT	negative regulation of nucleobase, nucleoside, nucleotide and nucleic acid metabolic process	50	1.20E-02	2.30E-01
	GOTERM_BP_FAT	negative regulation of transcription from RNA polymerase II promoter	29	1.70E-02	2.80E-01
	GOTERM_MF_FAT	transcription repressor activity	32	1.80E-02	3.90E-01
	GOTERM_BP_FAT	negative regulation of gene expression	48	2.10E-02	3.20E-01
	GOTERM_BP_FAT	negative regulation of macromolecule biosynthetic process	51	2.40E-02	3.50E-01
	GOTERM_BP_FAT	negative regulation of biosynthetic process	53	2.50E-02	3.60E-01
	GOTERM_BP_FAT	negative regulation of cellular biosynthetic process	52	2.50E-02	3.60E-01
	GOTERM_BP_FAT	regulation of transcription from RNA polymerase II promoter	60	1.00E-01	7.10E-01
**Annotation Cluster 42**	**Enrichment Score: 1.88**		**Count**	**P_Value**	**Benjamini**
	GOTERM_CC_FAT	histone methyltransferase complex	6	1.30E-02	2.00E-01
	GOTERM_CC_FAT	methyltransferase complex	6	1.30E-02	2.00E-01
	GOTERM_CC_FAT	MLL5-L complex	4	1.40E-02	2.10E-01
**Annotation Cluster 43**	**Enrichment Score: 1.85**		**Count**	**P_Value**	**Benjamini**
	INTERPRO	Guanylate-binding protein, C-terminal	4	7.90E-03	4.10E-01
	PIR_SUPERFAMILY	PIRSF005552:guanine nucleotide-binding protein 1	4	1.10E-02	7.10E-01
	INTERPRO	Guanylate-binding protein, N-terminal	4	3.10E-02	6.20E-01
**Annotation Cluster 44**	**Enrichment Score: 1.81**		**Count**	**P_Value**	**Benjamini**
	GOTERM_BP_FAT	response to molecule of bacterial origin	15	2.20E-03	6.60E-02
	GOTERM_BP_FAT	response to lipopolysaccharide	11	3.80E-02	4.50E-01
	GOTERM_BP_FAT	response to bacterium	21	4.50E-02	4.80E-01
**Annotation Cluster 45**	**Enrichment Score: 1.81**		**Count**	**P_Value**	**Benjamini**
	GOTERM_MF_FAT	protein binding, bridging	16	1.20E-03	6.30E-02
	GOTERM_MF_FAT	SH3/SH2 adaptor activity	8	4.10E-02	5.20E-01
	GOTERM_MF_FAT	molecular adaptor activity	9	7.40E-02	7.00E-01
**Annotation Cluster 46**	**Enrichment Score: 1.75**		**Count**	**P_Value**	**Benjamini**
	GOTERM_BP_FAT	positive regulation of T cell differentiation	9	1.20E-03	4.10E-02
	SP_PIR_KEYWORDS	scid	5	1.80E-02	1.30E-01
	GOTERM_BP_FAT	positive regulation of T cell differentiation in the thymus	3	2.60E-02	3.70E-01
	GOTERM_BP_FAT	regulation of T cell differentiation in the thymus	3	1.70E-01	8.30E-01
**Annotation Cluster 47**	**Enrichment Score: 1.75**		**Count**	**P_Value**	**Benjamini**
	UP_SEQ_FEATURE	short sequence motif:SH3-binding 3	4	9.10E-04	1.40E-01
	UP_SEQ_FEATURE	short sequence motif:SH3-binding 2	4	2.20E-03	2.60E-01
	UP_SEQ_FEATURE	short sequence motif:SH3-binding 1	4	2.20E-03	2.60E-01
	GOTERM_BP_FAT	phospholipid scrambling	3	2.60E-02	3.70E-01
	INTERPRO	Scramblase	3	3.70E-02	6.60E-01
	GOTERM_MF_FAT	phospholipid transporter activity	6	4.00E-02	5.30E-01
	PIR_SUPERFAMILY	PIRSF037928:phospholipid scramblase	3	4.80E-02	9.50E-01
	GOTERM_BP_FAT	plasma membrane organization	4	9.40E-02	6.80E-01
	GOTERM_MF_FAT	lipid transporter activity	7	2.40E-01	9.20E-01
**Annotation Cluster 48**	**Enrichment Score: 1.73**		**Count**	**P_Value**	**Benjamini**
	GOTERM_BP_FAT	extracellular matrix organization	16	5.10E-03	1.20E-01
	GOTERM_BP_FAT	collagen fibril organization	7	1.30E-02	2.40E-01
	GOTERM_BP_FAT	extracellular structure organization	17	9.80E-02	6.90E-01
**Annotation Cluster 49**	**Enrichment Score: 1.73**		**Count**	**P_Value**	**Benjamini**
	UP_SEQ_FEATURE	DNA-binding region:Tryptophan pentad repeat	4	1.50E-02	7.00E-01
	INTERPRO	Interferon regulatory factor, conserved site	4	1.70E-02	5.20E-01
	INTERPRO	Interferon regulatory factor	4	1.70E-02	5.20E-01
	SMART	IRF	4	2.80E-02	5.60E-01
**Annotation Cluster 50**	**Enrichment Score: 1.71**		**Count**	**P_Value**	**Benjamini**
	GOTERM_BP_FAT	regulation of inflammatory response	14	1.90E-03	6.10E-02
	GOTERM_BP_FAT	negative regulation of response to stimulus	15	8.60E-03	1.80E-01
	GOTERM_BP_FAT	negative regulation of defense response	8	1.00E-02	2.10E-01

ILMN_3250798	C11orf58	-0.425834421
ILMN_3236192	NA	-0.425843395
ILMN_1892506	CACNA1C	-0.425955008
ILMN_1895897	BF514393	-0.426075959
ILMN_3251737	EEF1A1	-0.426166297
ILMN_3239073	AY343892	-0.426402786
ILMN_2404085	CLIP1	-0.426519039
ILMN_3234892	MARCH11	-0.426626181
ILMN_2156699	ACOT6	-0.426634642
ILMN_1897796	BQ773461	-0.426662393
ILMN_1678919	YOD1	-0.426716693
ILMN_1762714	VGLL2	-0.42674999
ILMN_1679979	PLK3	-0.426809302
ILMN_1659285	PSMG1	-0.426812772
ILMN_1696708	C14orf166	-0.426884542
ILMN_1744611	DCAF13	-0.426927838
ILMN_1796165	GLRX5	-0.426984348
ILMN_3308330	MIR545	-0.427153939
ILMN_1788778	sept-11	-0.427244676
ILMN_3286312	NEK2	-0.427250374
ILMN_2057826	PHF3	-0.427296659
ILMN_1861380	BX088900	-0.427318405
ILMN_3200465	LOC100132724	-0.427342226
ILMN_1738033	OR2T33	-0.427420816
ILMN_3296096	SLC9A7P1	-0.427436752
ILMN_1760110	AK023936	-0.427439814
ILMN_1667263	BMS1P5	-0.427510006
ILMN_1689200	DHDH	-0.427850568
ILMN_1787595	B3GALNT1	-0.428685747
ILMN_1915734	DA722162	-0.428805689
ILMN_1775814	GHR	-0.428936957
ILMN_1682857	NDUFAF2	-0.428941234
ILMN_1707649	MPDZ	-0.428947541
ILMN_1707065	SALL3	-0.42901524
ILMN_3292122	NA	-0.4290776
ILMN_3301236	LOC728558	-0.429136649
ILMN_1660186	SYF2	-0.429338098
ILMN_1686405	KDR	-0.429366063
ILMN_2290118	MEGF9	-0.429391008
ILMN_3264466	FAM54B	-0.429677162
ILMN_1788878	MORN4	-0.429679257
ILMN_1803850	TSPAN15	-0.429970546
ILMN_1679919	ASCC2	-0.430017785
ILMN_1695717	RBM41	-0.430091428
ILMN_1658413	HYDIN	-0.430108716
ILMN_1681884	LOC100506049	-0.430111242
ILMN_1746676	CLDN8	-0.430235272
ILMN_1654759	WWC2	-0.430287957
ILMN_1745152	UQCC	-0.430290972
ILMN_1781560	ST3GAL6	-0.430316846
ILMN_1675172	RGS9BP	-0.430410284
ILMN_3187741	IGLON5	-0.430432892
ILMN_1787830	BASE	-0.430563832
ILMN_1652465	SLC26A4	-0.43065092
ILMN_1666967	BRP44L	-0.43067833
ILMN_1766920	EP400NL	-0.430685188
ILMN_1743040	LOC283104	-0.430760354
ILMN_1899387	BC039440	-0.430900843
ILMN_2414786	DIO1	-0.430909461
ILMN_1744614	PLEKHG6	-0.430962256
ILMN_1763371	RGS3	-0.43107328
ILMN_1774757	ZNF496	-0.431181714
ILMN_1663407	SURF1	-0.431244963
ILMN_3225432	LAMTOR3	-0.431272597
ILMN_1691333	ZNF57	-0.431336769
ILMN_2167011	ECHDC1	-0.431397707
ILMN_2158587	EEF1E1	-0.431403484
ILMN_2117526	DMGDH	-0.431572475
ILMN_2293594	LAMA3	-0.431574449
ILMN_1716803	RALYL	-0.431616374
ILMN_1659631	LOC440905	-0.431683987
ILMN_1728660	PMPCB	-0.431686903
ILMN_3241953	A2LD1	-0.431744235
ILMN_1758731	CYP2J2	-0.432164336
ILMN_2391522	MRPL47	-0.432299921
ILMN_1727466	KCNMB4	-0.43236285
ILMN_1753249	DDX10	-0.432368902
ILMN_1823732	NA	-0.4324789
ILMN_1770692	WDR12	-0.432579939
ILMN_1752668	DAAM2	-0.432766136
ILMN_2380754	CCNG1	-0.432862358
ILMN_2098743	ACOT13	-0.432903272
ILMN_1731518	PLD6	-0.43291548
ILMN_1712405	LOC100506085	-0.433039721
ILMN_3237062	STBD1	-0.433063567
ILMN_1728883	AB074415	-0.433109869
ILMN_3237035	SNORA54	-0.433165217
ILMN_1806754	GLDC	-0.433304654
ILMN_3244402	RIMBP3	-0.433329278
ILMN_3239568	MYLK4	-0.433555817
ILMN_1766079	SULT1C2	-0.433625106
ILMN_1671183	AMY2A	-0.433722286
ILMN_2159311	RFPL2	-0.433817856
ILMN_2211189	IKBKAP	-0.433820219
ILMN_1703092	RECQL4	-0.433868961
ILMN_1810942	CYP3A5	-0.434023537
ILMN_1872769	BX103063	-0.434102037
ILMN_1772302	MTHFS	-0.434163191

ILMN_1687533	SEMA4D	0.519439179
ILMN_2367010	GPR126	0.519409791
ILMN_1693233	KIAA0513	0.518984111
ILMN_1738832	SACS	0.518789672
ILMN_1651958	MGP	0.518749732
ILMN_1713561	C20orf103	0.518618413
ILMN_1748767	H3F3A	0.518611689
ILMN_1706505	COL5A1	0.518501999
ILMN_1659905	FKBP10	0.51778822
ILMN_1749789	HIST1H1D	0.517385181
ILMN_1755353	LOC646484	0.517359773
ILMN_1731418	SP110	0.516672367
ILMN_1653797	C6orf62	0.516409073
ILMN_1730906	FILIP1L	0.516342457
ILMN_1752755	VWF	0.51584319
ILMN_2246882	SP140	0.515398091
ILMN_1703692	TUBB	0.515373368
ILMN_1774733	SOCS1	0.515160688
ILMN_1686401	PDCD2	0.51515617
ILMN_1765547	IRF2	0.515132444
ILMN_1664464	PTGDS	0.514995203
ILMN_2414165	PRDM1	0.514869207
ILMN_2170813	LAMP3	0.514854618
ILMN_1768940	COL15A1	0.514819308
ILMN_1653498	IGSF6	0.51464226
ILMN_3219558	TUBA1C	0.51463854
ILMN_1772359	LAPTM5	0.514583115
ILMN_2379599	CD163	0.51407854
ILMN_1737586	EIF4H	0.514071307
ILMN_2388155	CASP3	0.514056975
ILMN_3248122	MED1	0.514045603
ILMN_1736180	FRAT1	0.513911587
ILMN_1758315	SLC9A9	0.513768304
ILMN_1787844	C19orf10	0.513285785
ILMN_2113049	QPCT	0.513038583
ILMN_1705442	CMTM3	0.512361037
ILMN_1805673	ALKBH4	0.512010829
ILMN_2399304	NAV2	0.511715806
ILMN_3243061	SIGLEC14	0.511462976
ILMN_1775762	GNAI2	0.511146532
ILMN_1672417	PTPRCAP	0.510922759
ILMN_1772036	STEAP4	0.510841905
ILMN_1679880	THOC6	0.510806899
ILMN_1769615	FLRT2	0.510746445
ILMN_1751572	TLE1	0.510349743
ILMN_1654939	TMED2	0.510307414
ILMN_3248324	MGC16384	0.510283635
ILMN_1773079	COL3A1	0.510071055
ILMN_1812795	RUNX1T1	0.509851865
ILMN_1669842	CHAF1A	0.509556509
ILMN_1707491	FAM30A	0.509426057
ILMN_1779234	CXCL6	0.509210037
ILMN_1696004	LRRK1	0.509125796
ILMN_2366330	FERMT3	0.5090588
ILMN_1691693	FCRH3	0.508919776
ILMN_2351466	NTM	0.508739255
ILMN_2320888	CXCR4	0.508379809
ILMN_1700147	VPREB3	0.508164615
ILMN_1724598	RABL2A	0.507915242
ILMN_1691662	LOC100101266	0.507463597
ILMN_2328952	CLCC1	0.507288586
ILMN_2077550	RACGAP1	0.507273648
ILMN_1726392	NIN	0.507098161
ILMN_1783909	COL6A2	0.506953109
ILMN_1778401	HLA-B	0.506796739
ILMN_1681101	MARCH1	0.506702524
ILMN_1658586	GPR161	0.506516035
ILMN_2169439	ITGAV	0.506240584
ILMN_2255133	BCL11A	0.505996909
ILMN_2396444	CD14	0.505971823
ILMN_1724907	NUDT3	0.505861202
ILMN_3234841	RPL23AP7	0.505769005
ILMN_1724139	TMEM123	0.505763396
ILMN_3232447	AI880175	0.505755041
ILMN_1673788	CDV3	0.505518792
ILMN_1722622	CD163	0.505402829
ILMN_1758418	TNFSF13B	0.505239005
ILMN_1773389	PLTP	0.505123684
ILMN_3304898	TUBB	0.504961219
ILMN_1693009	FGL2	0.504780088
ILMN_1697377	ANKRD36B	0.504188354
ILMN_2394750	EXOC4	0.504149868
ILMN_1759312	AMPD1	0.503849565
ILMN_1701424	LAMC2	0.503589319
ILMN_2329834	CPNE1	0.503494471
ILMN_2317923	TMEM132A	0.503283566
ILMN_2352190	CLIP2	0.503184367
ILMN_1664010	ELF1	0.50292913
ILMN_1654586	RASA3	0.502902143
ILMN_1702708	PPP2R3A	0.502783714
ILMN_1796762	CCDC102A	0.502770407
ILMN_1753608	TMEM131	0.50276988
ILMN_1656920	CRIP1	0.502717157
ILMN_1708416	ARL6IP1	0.502712897
ILMN_1813491	SPTLC1	0.502177661

	GOTERM_BP_FAT	negative regulation of inflammatory response	6	5.90E-02	5.60E-01
	GOTERM_BP_FAT	negative regulation of response to external stimulus	6	2.60E-01	9.10E-01
**Annotation Cluster 51**	**Enrichment Score: 1.71**		**Count**	**P_Value**	**Benjamini**
	INTERPRO	Immunoglobulin subtype	46	2.00E-06	5.70E-04
	SMART	IG	46	1.40E-04	1.30E-02
	UP_SEQ_FEATURE	domain:Ig-like C2-type 1	23	3.50E-03	3.40E-01
	UP_SEQ_FEATURE	domain:Ig-like C2-type 2	23	3.80E-03	3.50E-01
	INTERPRO	Immunoglobulin	22	2.30E-02	5.80E-01
	UP_SEQ_FEATURE	domain:Ig-like C2-type 3	13	7.60E-02	9.70E-01
	UP_SEQ_FEATURE	domain:Ig-like C2-type 6	6	7.60E-02	9.70E-01
	UP_SEQ_FEATURE	domain:Ig-like C2-type 4	9	9.80E-02	9.90E-01
	UP_SEQ_FEATURE	domain:Ig-like C2-type 5	7	1.20E-01	9.90E-01
	UP_SEQ_FEATURE	domain:Ig-like C2-type 7	4	1.50E-01	1.00E+00
	INTERPRO	Immunoglobulin subtype 2	18	1.80E-01	9.50E-01
	SMART	IGc2	18	4.30E-01	9.70E-01
	INTERPRO	Immunoglobulin I-set	8	8.00E-01	1.00E+00
**Annotation Cluster 52**	**Enrichment Score: 1.71**		**Count**	**P_Value**	**Benjamini**
	GOTERM_BP_FAT	positive regulation of adaptive immune response	10	1.70E-04	7.50E-03
	GOTERM_BP_FAT	regulation of immune effector process	18	5.10E-04	2.00E-02
	GOTERM_BP_FAT	positive regulation of immune effector process	11	6.70E-04	2.60E-02
	GOTERM_BP_FAT	positive regulation of adaptive immune response based on somatic recombination of immune receptors built from immunoglobulin superfamily domains	9	7.50E-04	2.80E-02
	GOTERM_BP_FAT	regulation of adaptive immune response	12	1.30E-03	4.50E-02
	GOTERM_BP_FAT	regulation of leukocyte mediated immunity	12	2.80E-03	7.90E-02
	GOTERM_BP_FAT	regulation of lymphocyte mediated immunity	11	3.50E-03	9.30E-02
	GOTERM_BP_FAT	regulation of adaptive immune response based on somatic recombination of immune receptors built from immunoglobulin superfamily domains	11	4.00E-03	1.00E-01
	GOTERM_BP_FAT	positive regulation of leukocyte mediated immunity	8	7.60E-03	1.60E-01
	GOTERM_BP_FAT	positive regulation of lymphocyte mediated immunity	8	7.60E-03	1.60E-01
	GOTERM_BP_FAT	regulation of inflammatory response to antigenic stimulus	4	1.40E-02	2.50E-01
	GOTERM_BP_FAT	positive regulation of inflammatory response to antigenic stimulus	3	2.60E-02	3.70E-01
	GOTERM_BP_FAT	positive regulation of B cell mediated immunity	4	3.60E-02	4.30E-01
	GOTERM_BP_FAT	positive regulation of immunoglobulin mediated immune response	4	3.60E-02	4.30E-01
	GOTERM_BP_FAT	regulation of T cell mediated immunity	5	5.30E-02	5.30E-01
	GOTERM_BP_FAT	regulation of production of molecular mediator of immune response	7	6.10E-02	5.60E-01
	GOTERM_BP_FAT	regulation of B cell mediated immunity	5	8.00E-02	6.40E-01
	GOTERM_BP_FAT	regulation of immunoglobulin mediated immune response	5	8.00E-02	6.40E-01
	GOTERM_BP_FAT	positive regulation of T cell mediated immunity	4	8.00E-02	6.40E-01
	GOTERM_BP_FAT	regulation of leukocyte mediated cytotoxicity	5	9.00E-02	6.70E-01
	GOTERM_BP_FAT	regulation of cell killing	5	1.20E-01	7.50E-01
	GOTERM_BP_FAT	regulation of T cell mediated cytotoxicity	3	1.20E-01	7.50E-01
	GOTERM_BP_FAT	immunoglobulin production	4	3.40E-01	9.50E-01
	GOTERM_BP_FAT	production of molecular mediator of immune response	4	3.60E-01	9.50E-01
	GOTERM_BP_FAT	positive regulation of leukocyte mediated cytotoxicity	3	4.30E-01	9.70E-01
	GOTERM_BP_FAT	positive regulation of cell killing	3	5.00E-01	9.80E-01
**Annotation Cluster 53**	**Enrichment Score: 1.7**		**Count**	**P_Value**	**Benjamini**
	GOTERM_BP_FAT	regulation of calcium-mediated signaling	6	1.00E-02	2.00E-01
	GOTERM_BP_FAT	positive regulation of calcium-mediated signaling	5	2.60E-02	3.70E-01
	GOTERM_BP_FAT	positive regulation of alpha-beta T cell activation	6	3.00E-02	4.00E-01
**Annotation Cluster 54**	**Enrichment Score: 1.68**		**Count**	**P_Value**	**Benjamini**
	UP_SEQ_FEATURE	domain:DAPIN	6	9.90E-03	6.30E-01
	INTERPRO	Pyrin	6	1.20E-02	4.70E-01
	INTERPRO	HIN-200/IF120x	3	2.30E-02	5.70E-01
	INTERPRO	DEATH-like	9	6.90E-02	8.00E-01
**Annotation Cluster 55**	**Enrichment Score: 1.66**		**Count**	**P_Value**	**Benjamini**
	GOTERM_BP_FAT	positive regulation of cytokine biosynthetic process	10	5.00E-03	1.20E-01
	GOTERM_BP_FAT	regulation of interleukin-2 production	7	1.50E-02	2.60E-01
	GOTERM_BP_FAT	regulation of cytokine biosynthetic process	11	3.00E-02	4.00E-01
	GOTERM_BP_FAT	regulation of interleukin-2 biosynthetic process	5	3.80E-02	4.50E-01
	GOTERM_BP_FAT	positive regulation of interleukin-2 biosynthetic process	4	5.60E-02	5.40E-01
**Annotation Cluster 56**	**Enrichment Score: 1.61**		**Count**	**P_Value**	**Benjamini**
	GOTERM_MF_FAT	kinase binding	21	1.50E-02	3.70E-01
	GOTERM_MF_FAT	enzyme binding	47	3.00E-02	4.70E-01
	GOTERM_MF_FAT	protein kinase binding	17	3.40E-02	4.90E-01
**Annotation Cluster 57**	**Enrichment Score: 1.58**		**Count**	**P_Value**	**Benjamini**
	SP_PIR_KEYWORDS	GTPase activation	19	9.30E-03	7.60E-02
	UP_SEQ_FEATURE	domain:Rho-GAP	10	1.80E-02	7.30E-01
	INTERPRO	RhoGAP	10	2.90E-02	6.10E-01
	GOTERM_MF_FAT	GTPase activator activity	23	3.50E-02	4.90E-01
	SMART	RhoGAP	10	7.40E-02	6.50E-01
**Annotation Cluster 58**	**Enrichment Score: 1.56**		**Count**	**P_Value**	**Benjamini**
	SP_PIR_KEYWORDS	tyrosine-specific protein kinase	10	2.40E-03	2.70E-02
	SP_PIR_KEYWORDS	autophosphorylation	10	2.70E-03	3.00E-02
	SP_PIR_KEYWORDS	phosphotransferase	24	3.10E-03	3.20E-02
	GOTERM_BP_FAT	peptidyl-tyrosine modification	9	1.60E-02	2.70E-01
	GOTERM_MF_FAT	non-membrane spanning protein tyrosine kinase activity	8	1.70E-02	3.80E-01
	SP_PIR_KEYWORDS	tyrosine-protein kinase	14	1.90E-02	1.30E-01
	INTERPRO	Tyrosine protein kinase	15	2.10E-02	5.70E-01
	INTERPRO	Tyrosine protein kinase, active site	13	2.40E-02	5.60E-01
	SP_PIR_KEYWORDS	ATP	23	3.50E-02	2.00E-01
	GOTERM_BP_FAT	peptidyl-tyrosine phosphorylation	8	3.70E-02	4.40E-01
	GOTERM_MF_FAT	protein tyrosine kinase activity	18	4.80E-02	5.70E-01
	SMART	TyrKc	15	7.30E-02	6.70E-01
	GOTERM_MF_FAT	transmembrane receptor protein tyrosine kinase activity	9	7.40E-02	7.00E-01
	INTERPRO	Protein kinase, ATP binding site	38	9.50E-02	8.60E-01
	UP_SEQ_FEATURE	binding site:ATP	42	1.10E-01	9.90E-01
	INTERPRO	Protein kinase, core	39	1.10E-01	8.90E-01
	UP_SEQ_FEATURE	domain:Protein kinase	36	1.50E-01	1.00E+00
**Annotation Cluster 59**	**Enrichment Score: 1.54**		**Count**	**P_Value**	**Benjamini**
	UP_SEQ_FEATURE	domain:VWFC	7	5.50E-04	1.10E-01
	INTERPRO	von Willebrand factor, type C	8	1.40E-02	4.80E-01
	SMART	VWC	8	3.30E-02	5.90E-01
	UP_SEQ_FEATURE	region of interest:Heparin-binding	4	1.20E-01	9.90E-01
	GOTERM_BP_FAT	regulation of cell-substrate adhesion	4	6.20E-01	9.90E-01
**Annotation Cluster 60**	**Enrichment Score: 1.54**		**Count**	**P_Value**	**Benjamini**
	GOTERM_BP_FAT	positive regulation of immune effector process	11	6.70E-04	2.60E-02
	GOTERM_BP_FAT	positive regulation of immunoglobulin production	3	5.90E-02	5.60E-01

ILMN_3187680	ACCS	-0.434273601
ILMN_1658548	LOC401180	-0.434301354
ILMN_1775569	CRISP2	-0.434324694
ILMN_2122952	CISD1	-0.434332614
ILMN_1813374	C19orf28	-0.434339413
ILMN_1761049	EIF2C3	-0.434447469
ILMN_1665621	HSF1	-0.434502935
ILMN_2388425	EXTL2	-0.43452297
ILMN_1668507	DDAH1	-0.434530764
ILMN_3209098	FAM71E2	-0.434668528
ILMN_1800049	MAPT	-0.434680407
ILMN_3309234	MIR181D	-0.434711774
ILMN_1683755	SOX13	-0.434846792
ILMN_2310814	MAPT	-0.435180042
ILMN_1680420	NUDT13	-0.435267065
ILMN_2388975	CERK	-0.435436604
ILMN_1711139	VPS37A	-0.435443746
ILMN_1774565	KAAG1	-0.435444092
ILMN_3244019	KANK1	-0.435533662
ILMN_1660698	GTPBP8	-0.435637776
ILMN_1685343	NUPL1	-0.435662766
ILMN_1686968	ZNF362	-0.435793827
ILMN_1674560	GBA2	-0.43580444
ILMN_3244449	SNORD9	-0.435921778
ILMN_1765438	C6orf184	-0.435968921
ILMN_1761264	BMP8A	-0.436050729
ILMN_1808457	TBX18	-0.436066954
ILMN_2134888	TUBE1	-0.43624955
ILMN_1693771	ASPH	-0.436271075
ILMN_1672717	C10orf57	-0.436437074
ILMN_1877912	BX102609	-0.436444502
ILMN_2406815	LRRC17	-0.436447501
ILMN_1769155	CYP2C8	-0.436725424
ILMN_1804569	GAS2	-0.436731209
ILMN_1795767	GRHL1	-0.436765448
ILMN_1708382	C3orf75	-0.436894336
ILMN_1689156	MMAB	-0.436907549
ILMN_2115862	ESPNL	-0.43702817
ILMN_3274572	ST13	-0.437182937
ILMN_2398926	C17orf58	-0.437200672
ILMN_1697469	SRSF6	-0.437233997
ILMN_1733781	FSD1	-0.437254435
ILMN_1730575	GCLC	-0.437334441
ILMN_3249963	PCBP2	-0.437366287
ILMN_2388517	MTERFD3	-0.437369439
ILMN_1904578	ODZ1	-0.437460093
ILMN_1800873	SEZ6L2	-0.437492697
ILMN_2404033	ZNF205	-0.437605023
ILMN_1754738	AP1M1	-0.437668917
ILMN_1729402	CHRM2	-0.437798767
ILMN_2336595	ACSS2	-0.43791479
ILMN_1660815	CAB39L	-0.43813087
ILMN_1713846	PPM1H	-0.438137504
ILMN_1775175	RPL3L	-0.438137984
ILMN_1755047	LRRC2	-0.438185494
ILMN_1729515	PIN4	-0.438269246
ILMN_2156267	EIF2AK1	-0.438490247
ILMN_2200503	NIT2	-0.438607227
ILMN_1782938	SLC16A10	-0.438674111
ILMN_1685396	CLIC5	-0.438680591
ILMN_1887357	AK095715	-0.438709312
ILMN_3247723	NOP16	-0.438848999
ILMN_3245038	AQP7	-0.438870012
ILMN_1868037	CA441597	-0.439174032
ILMN_3237520	C6orf127	-0.439220853
ILMN_1686951	TM6SF2	-0.439237695
ILMN_1683302	SMC4	-0.439237826
ILMN_2110094	CFC1	-0.439253234
ILMN_1669747	UNC13B	-0.439352705
ILMN_2363744	PACRG	-0.439445678
ILMN_1729816	VDAC3	-0.439461192
ILMN_1792182	RDH12	-0.43957916
ILMN_3181457	DB201663	-0.439792078
ILMN_1688220	SYNPO2	-0.439877604
ILMN_1909247	CD299155	-0.439888654
ILMN_2358278	RSRC2	-0.439925626
ILMN_3187300	NA	-0.4399299
ILMN_2167808	TM4SF5	-0.440280501
ILMN_3300516	NA	-0.440321973
ILMN_1769601	TBCK	-0.440345527
ILMN_1804329	TUSC2	-0.440509992
ILMN_2212590	TMEM170A	-0.440563978
ILMN_2230566	RAB40B	-0.440587885
ILMN_1830259	AI630821	-0.440951859
ILMN_3251171	DNASE1L1	-0.440953862
ILMN_2189371	NTNG1	-0.441028597
ILMN_2179397	TATDN1	-0.44103387
ILMN_1875763	AI290919	-0.441036237
ILMN_1760199	CALB1	-0.44109685
ILMN_1767277	PLGLA	-0.441098595
ILMN_1743357	XPNPEP2	-0.44110727
ILMN_1753450	ATXN3L	-0.441239002
ILMN_1789342	NDUFS2	-0.441429806
ILMN_1656257	DKFZp434O0320	-0.441735565
ILMN_2287543	SHISA3	-0.441801239

ILMN_1668526	GVIN1	0.502134327
ILMN_3266471	SNRPC	0.502069028
ILMN_1792323	HDC	0.501908382
ILMN_3250321	PTP4A3	0.501884874
ILMN_1683658	FKBP1A	0.500912724
ILMN_1783182	FBN1	0.500902908
ILMN_1708006	MICA	0.50072348
ILMN_1814282	AEN	0.500720751
ILMN_1768391	ARL4C	0.500429722
ILMN_2065783	EXOC2	0.500390809
ILMN_1727194	CALU	0.500361911
ILMN_2161577	CXCL6	0.500240901
ILMN_2350634	EFEMP1	0.500206558
ILMN_1806415	TTLL1	0.500133453
ILMN_2145670	TNC	0.499902436
ILMN_1659158	SAMD3	0.499796669
ILMN_2103107	ADAMDEC1	0.499783162
ILMN_1667966	FAM129A	0.499772816
ILMN_2289849	FCGR2A	0.499770278
ILMN_2046730	S100A10	0.499655898
ILMN_1728478	CXCL16	0.499648824
ILMN_1720124	RCC2	0.4995933
ILMN_1773059	GPR124	0.499553381
ILMN_1770892	YY1	0.499319867
ILMN_2145501	ANGPTL1	0.499287642
ILMN_1739885	SLC41A3	0.498930906
ILMN_3241870	FRMD8	0.498891755
ILMN_1702787	SEMA4A	0.498525311
ILMN_3246005	KDM5B	0.498336371
ILMN_2328972	DNMT3B	0.497852497
ILMN_1776678	GIMAP7	0.497755073
ILMN_1777342	PREX1	0.497650991
ILMN_2384544	ADAM15	0.49700085
ILMN_1652237	CBR3	0.496843407
ILMN_2131523	SACS	0.496414693
ILMN_1713496	ST3GAL5	0.496247538
ILMN_1713676	FLJ40330	0.496128814
ILMN_2148459	B2M	0.496016298
ILMN_1738578	FILIP1L	0.495603084
ILMN_1769705	H3F3C	0.495596519
ILMN_2384496	ST6GAL1	0.494986289
ILMN_1801767	ABHD3	0.494887245
ILMN_1673352	IFITM2	0.494874549
ILMN_1759915	ARPC1A	0.494657516
ILMN_1666050	TMUB1	0.494520754
ILMN_1773388	C13orf18	0.494502622
ILMN_2150258	ZFP36L2	0.494331692
ILMN_1790778	PNMA2	0.493965071
ILMN_1701613	RARRES3	0.493865386
ILMN_1672589	SEMA4B	0.493656469
ILMN_1768488	TERF2	0.493630659
ILMN_1750181	TESC	0.493587988
ILMN_1677765	LRP8	0.49346876
ILMN_1751368	HNRNPD	0.493326882
ILMN_1804117	FAM89B	0.492879984
ILMN_1734878	CD79A	0.492627639
ILMN_1751444	NCAPG	0.492236342
ILMN_3194911	ATF6B	0.492071889
ILMN_1742450	TAPBP	0.491946553
ILMN_1750271	MOBP	0.491887629
ILMN_2365881	ATG16L1	0.491852087
ILMN_1840742	AA431235	0.491683004
ILMN_2356991	CD47	0.491672352
ILMN_1674036	BIRC7	0.49156155
ILMN_1779828	EDEM1	0.491418122
ILMN_1786697	TRIM9	0.491286863
ILMN_2347145	DCN	0.49086298
ILMN_1793371	KIAA0430	0.490393749
ILMN_1689968	PLEKHO2	0.490307169
ILMN_1789502	GPC4	0.490240829
ILMN_1814998	POTEKP	0.490143791
ILMN_1716382	C12orf75	0.49000653
ILMN_2074258	BARD1	0.489771583
ILMN_1687538	ETS1	0.48955585
ILMN_1691165	CLSPN	0.489179941
ILMN_1676361	ARHGAP22	0.48915052
ILMN_1810910	CFH	0.489085283
ILMN_1716687	TPM1	0.489048001
ILMN_2327244	CALCA	0.488929487
ILMN_1742544	MEF2C	0.488487575
ILMN_1676128	DNMT3A	0.488230002
ILMN_1678842	THBS2	0.487925561
ILMN_1674386	PITX1	0.48785589
ILMN_1781824	FASLG	0.487431803
ILMN_1666932	FCGR2A	0.487320898
ILMN_1795025	NFX1	0.48723245
ILMN_1716988	OPN3	0.487129838
ILMN_1674533	TRPV6	0.486954576
ILMN_1782741	CD300LB	0.486873453
ILMN_3311070	MIR33B	0.486681487
ILMN_1765146	IFNAR2	0.48652426
ILMN_2145033	CCR5	0.486445397

	GOTERM_BP_FAT	regulation of production of molecular mediator of immune response	7	6.10E-02	5.60E-01
	GOTERM_BP_FAT	positive regulation of production of molecular mediator of immune response	4	8.00E-02	6.40E-01
	GOTERM_BP_FAT	regulation of immunoglobulin production	5	1.00E-01	7.00E-01
**Annotation Cluster 61**	**Enrichment Score: 1.51**		**Count**	**P_Value**	**Benjamini**
	SP_PIR_KEYWORDS	inflammatory response	20	1.50E-07	7.60E-06
	UP_SEQ_FEATURE	domain:TIR	8	4.70E-04	1.00E-01
	INTERPRO	Toll-Interleukin receptor	8	4.80E-04	6.60E-02
	SMART	TIR	8	1.40E-03	7.60E-02
	INTERPRO	Toll-like receptor	4	2.50E-03	2.00E-01
	GOTERM_BP_FAT	positive regulation of tumor necrosis factor production	6	2.60E-03	7.60E-02
	GOTERM_BP_FAT	I-kappaB kinase/NF-kappaB cascade	12	3.60E-03	9.50E-02
	PIR_SUPERFAMILY	PIRSF037595:Toll-like_receptor	4	3.70E-03	3.90E-01
	GOTERM_BP_FAT	response to host immune response	4	5.60E-03	1.30E-01
	GOTERM_BP_FAT	response to immune response of other organism during symbiotic interaction	4	5.60E-03	1.30E-01
	GOTERM_BP_FAT	myeloid cell activation during immune response	6	6.20E-03	1.40E-01
	GOTERM_BP_FAT	regulation of tumor necrosis factor production	7	1.80E-02	2.90E-01
	GOTERM_BP_FAT	response to host defenses	4	2.00E-02	3.10E-01
	GOTERM_BP_FAT	response to defenses of other organism during symbiotic interaction	4	2.00E-02	3.10E-01
	GOTERM_BP_FAT	response to host	4	2.00E-02	3.10E-01
	GOTERM_BP_FAT	pattern recognition receptor signaling pathway	5	2.60E-02	3.70E-01
	GOTERM_BP_FAT	regulation of innate immune response	9	3.10E-02	4.00E-01
	GOTERM_BP_FAT	innate immune response-activating signal transduction	5	3.80E-02	4.50E-01
	GOTERM_BP_FAT	activation of innate immune response	5	3.80E-02	4.50E-01
	GOTERM_BP_FAT	positive regulation by symbiont of host immune response	3	4.10E-02	4.70E-01
	GOTERM_BP_FAT	modulation by symbiont of host immune response	3	4.10E-02	4.70E-01
	GOTERM_BP_FAT	positive regulation by organism of immune response of other organism during symbiotic interaction	3	4.10E-02	4.70E-01
	GOTERM_BP_FAT	modulation by symbiont of host innate immunity	3	4.10E-02	4.70E-01
	GOTERM_BP_FAT	positive regulation by symbiont of host innate immunity	3	4.10E-02	4.70E-01
	GOTERM_BP_FAT	modulation by organism of immune response of other organism during symbiotic interaction	3	4.10E-02	4.70E-01
	GOTERM_BP_FAT	modulation by organism of innate immunity in other organism during symbiotic interaction	3	4.10E-02	4.70E-01
	GOTERM_BP_FAT	positive regulation by organism of innate immunity in other organism during symbiotic interaction	3	4.10E-02	4.70E-01
	GOTERM_BP_FAT	microglial cell activation during immune response	3	4.10E-02	4.70E-01
	INTERPRO	Interleukin-1 receptor, type I/Toll precursor	4	4.80E-02	7.30E-01
	GOTERM_BP_FAT	modulation by symbiont of host defense response	3	5.90E-02	5.60E-01
	GOTERM_BP_FAT	positive regulation by organism of defense response of other organism during symbiotic interaction	3	5.90E-02	5.60E-01
	GOTERM_BP_FAT	positive regulation by symbiont of host defense response	3	5.90E-02	5.60E-01
	GOTERM_BP_FAT	modulation by organism of defense response of other organism during symbiotic interaction	3	5.90E-02	5.60E-01
	GOTERM_BP_FAT	toll-like receptor signaling pathway	4	6.70E-02	5.90E-01
	GOTERM_BP_FAT	microglial cell activation	3	7.90E-02	6.40E-01
	GOTERM_BP_FAT	macrophage activation	4	8.00E-02	6.40E-01
	GOTERM_BP_FAT	regulation of interleukin-6 production	6	9.90E-02	7.00E-01
	GOTERM_BP_FAT	macrophage activation during immune response	3	1.00E-01	7.00E-01
	GOTERM_MF_FAT	peptidoglycan binding	3	1.20E-01	8.00E-01
	GOTERM_BP_FAT	modification by symbiont of host morphology or physiology	3	1.20E-01	7.50E-01
	BIOCARTA	Toll-Like Receptor Pathway	8	1.60E-01	8.20E-01
	UP_SEQ_FEATURE	repeat:LRR 13	8	1.90E-01	1.00E+00
	GOTERM_BP_FAT	positive regulation of innate immune response	6	2.10E-01	8.70E-01
	GOTERM_BP_FAT	positive regulation of binding	8	2.90E-01	9.30E-01
	GOTERM_BP_FAT	positive regulation of NF-kappaB transcription factor activity	5	3.10E-01	9.40E-01
	GOTERM_BP_FAT	positive regulation of DNA binding	7	3.50E-01	9.50E-01
	GOTERM_BP_FAT	positive regulation of interleukin-6 production	3	3.80E-01	9.60E-01
	GOTERM_BP_FAT	positive regulation of transcription factor activity	6	4.00E-01	9.70E-01
	GOTERM_BP_FAT	regulation of DNA binding	9	6.00E-01	9.90E-01
	GOTERM_BP_FAT	regulation of transcription factor activity	7	7.20E-01	1.00E+00
	GOTERM_BP_FAT	regulation of binding	10	7.40E-01	1.00E+00
**Annotation Cluster 62**	**Enrichment Score: 1.51**		**Count**	**P_Value**	**Benjamini**
	GOTERM_MF_FAT	eukaryotic cell surface binding	6	4.00E-03	1.70E-01
	GOTERM_MF_FAT	cell surface binding	6	4.00E-02	5.30E-01
	GOTERM_CC_FAT	anchored to plasma membrane	4	1.90E-01	7.90E-01
**Annotation Cluster 63**	**Enrichment Score: 1.5**		**Count**	**P_Value**	**Benjamini**
	GOTERM_MF_FAT	transcription corepressor activity	18	1.50E-02	3.70E-01
	GOTERM_MF_FAT	transcription repressor activity	32	1.80E-02	3.90E-01
	GOTERM_MF_FAT	transcription activator activity	39	2.20E-02	4.20E-01
	GOTERM_MF_FAT	transcription factor binding	47	2.30E-02	4.10E-01
	GOTERM_MF_FAT	transcription cofactor activity	34	4.10E-02	5.30E-01
	GOTERM_MF_FAT	transcription coactivator activity	19	1.80E-01	8.80E-01
**Annotation Cluster 64**	**Enrichment Score: 1.46**		**Count**	**P_Value**	**Benjamini**
	GOTERM_BP_FAT	maintenance of protein location	10	5.00E-03	1.20E-01
	GOTERM_BP_FAT	protein retention in ER lumen	4	9.30E-03	1.90E-01
	GOTERM_BP_FAT	maintenance of location	10	3.10E-02	4.00E-01
	GOTERM_BP_FAT	maintenance of protein location in cell	6	1.50E-01	7.90E-01
	GOTERM_BP_FAT	maintenance of location in cell	6	2.40E-01	8.90E-01
**Annotation Cluster 65**	**Enrichment Score: 1.44**		**Count**	**P_Value**	**Benjamini**
	UP_SEQ_FEATURE	region of interest:Beta-galactoside binding 1	3	1.10E-02	6.40E-01
	UP_SEQ_FEATURE	region of interest:Beta-galactoside binding 2	3	1.10E-02	6.40E-01
	UP_SEQ_FEATURE	domain:Galectin 2	3	4.90E-02	9.20E-01
	UP_SEQ_FEATURE	domain:Galectin 1	3	4.90E-02	9.20E-01
	INTERPRO	Galectin, carbohydrate recognition domain	4	7.00E-02	8.00E-01
	SMART	GLECT	4	1.10E-01	7.40E-01
**Annotation Cluster 66**	**Enrichment Score: 1.44**		**Count**	**P_Value**	**Benjamini**
	GOTERM_CC_FAT	platelet alpha granule	11	4.60E-03	9.80E-02
	GOTERM_CC_FAT	platelet alpha granule lumen	9	6.30E-03	1.30E-01
	GOTERM_CC_FAT	cytoplasmic membrane-bounded vesicle lumen	9	9.80E-03	1.70E-01
	GOTERM_CC_FAT	vesicle lumen	9	1.30E-02	2.00E-01
	GOTERM_CC_FAT	secretory granule	18	1.20E-01	6.50E-01
	GOTERM_CC_FAT	cytoplasmic vesicle part	15	4.20E-01	9.20E-01
	GOTERM_BP_FAT	negative regulation of proteolysis	3	4.50E-01	9.80E-01
**Annotation Cluster 67**	**Enrichment Score: 1.42**		**Count**	**P_Value**	**Benjamini**
	GOTERM_CC_FAT	extracellular matrix part	17	6.70E-03	1.30E-01
	GOTERM_CC_FAT	basement membrane	10	8.90E-02	6.10E-01
	SP_PIR_KEYWORDS	basement membrane	6	9.00E-02	4.00E-01
**Annotation Cluster 68**	**Enrichment Score: 1.42**		**Count**	**P_Value**	**Benjamini**

ILMN_1753498	COASY	-0.44184581
ILMN_1687392	NRK	-0.441893837
ILMN_2151281	GABARAPL1	-0.442144704
ILMN_2145396	AKR7A3	-0.442315078
ILMN_1717357	AIFM1	-0.442410762
ILMN_1681674	MAD1L1	-0.442559235
ILMN_1767649	PCDHGC3	-0.442560517
ILMN_1720482	CEND1	-0.442682561
ILMN_1766103	KCNK18	-0.442852728
ILMN_1652037	UPP2	-0.442859322
ILMN_1724021	SLC6A19	-0.442965601
ILMN_1654526	LOC100093698	-0.443004156
ILMN_1907451	BC038752	-0.443040868
ILMN_1792449	ALS2CR11	-0.443116937
ILMN_1779264	PSMG1	-0.443342452
ILMN_1798557	FAM153A	-0.443440132
ILMN_1740949	LRRC19	-0.44360544
ILMN_1801698	ACSM5	-0.443932605
ILMN_1781104	MAP2K7	-0.444048565
ILMN_2082130	C1orf123	-0.444076459
ILMN_1742040	GDPD4	-0.444142814
ILMN_3246345	UCRP	-0.444175648
ILMN_1694514	ZDHHC11	-0.444185751
ILMN_1787511	THUMPD2	-0.444230326
ILMN_1681641	XTP6	-0.444251371
ILMN_1840311	DA494348	-0.444326762
ILMN_1792138	UQCRH	-0.444437518
ILMN_1776577	DSCC1	-0.444443752
ILMN_1705144	ULK1	-0.444481213
ILMN_1703826	UNC5D	-0.444529337
ILMN_2259949	BCKDHB	-0.444587731
ILMN_1814316	RBFOX1	-0.44459197
ILMN_1762993	PIGV	-0.444738905
ILMN_3239946	LOC729375	-0.445003494
ILMN_1807397	NDUFB5	-0.445175155
ILMN_2330697	NR1I3	-0.445294152
ILMN_1811478	MFI2	-0.445303766
ILMN_1713219	RGS12	-0.445360649
ILMN_1782881	DLEU7	-0.445374219
ILMN_1783497	PANK1	-0.445450884
ILMN_1667641	ACACA	-0.44576525
ILMN_3234735	ERI2	-0.445920568
ILMN_2188959	ACOT2	-0.446033483
ILMN_1787591	XPA	-0.446132235
ILMN_2183885	C7orf11	-0.446269591
ILMN_1796560	PFKFB2	-0.446312466
ILMN_3184595	NA	-0.446605606
ILMN_1686291	DIO1	-0.446691529
ILMN_3310281	MIRLET7E	-0.446768059
ILMN_1698673	EFCAB7	-0.446801684
ILMN_1684730	POM121L2	-0.446863322
ILMN_2352633	ARHGAP24	-0.446939323
ILMN_1656940	ABLIM3	-0.44695517
ILMN_1711247	TRIM24	-0.446960905
ILMN_1798249	AK4	-0.44712351
ILMN_1769556	NPR3	-0.447158805
ILMN_1704321	IL2	-0.447336104
ILMN_1668012	SLC25A13	-0.447385447
ILMN_3187862	DNAH14	-0.447428737
ILMN_1739813	HYAL1	-0.447554496
ILMN_1797332	NARS2	-0.447579401
ILMN_2140799	FAM24B	-0.447620495
ILMN_1660729	ATP6V1C2	-0.447713247
ILMN_1786278	FAM149A	-0.448006985
ILMN_1675365	LARP4	-0.448013103
ILMN_1752646	NA	-0.448090623
ILMN_1685043	CYP3A7	-0.448097164
ILMN_1771061	BRD4	-0.448110561
ILMN_1786433	BCCIP	-0.448134229
ILMN_1721680	LOC644075	-0.44874355
ILMN_2207988	SERPINI1	-0.449190859
ILMN_3238353	RNASEH2C	-0.449212818
ILMN_3282032	USP17L2	-0.449420511
ILMN_1803073	DNAJC12	-0.449441226
ILMN_1748694	NHSL2	-0.449518655
ILMN_1701831	GSTA1	-0.449522403
ILMN_2314140	PAX6	-0.449573609
ILMN_1657962	KRTAP1-1	-0.449660612
ILMN_1679209	HSPA9	-0.449724642
ILMN_1705116	SLC22A23	-0.449847612
ILMN_1662935	C1QTNF7	-0.449890302
ILMN_1696699	FAM184A	-0.449907874
ILMN_1724609	SLC2A8	-0.449933926
ILMN_2072568	CLDN8	-0.449936193
ILMN_1701512	KIAA0391	-0.450028702
ILMN_1730432	IGSF8	-0.450083821
ILMN_1693882	TAPT1	-0.45031564
ILMN_1723743	ROM1	-0.450358677
ILMN_1777556	SOAT2	-0.450359631
ILMN_1777366	TTC35	-0.450541986
ILMN_3200885	TNFRSF14	-0.450653609
ILMN_1781951	SCN8A	-0.45068709

ILMN_1789007	APOC1	0.486258812
ILMN_1674855	TRAM1L1	0.486172213
ILMN_1669727	WAC	0.486104749
ILMN_2383435	PSMD10	0.486057524
ILMN_3203765	FLJ00317	0.485846015
ILMN_1711361	ZNF319	0.485740187
ILMN_1790909	NFE2L2	0.485575825
ILMN_1703803	ADAMTS3	0.485564248
ILMN_2374362	FAM108B1	0.485564134
ILMN_1758100	GALR3	0.485491993
ILMN_1680102	C11orf45	0.485368157
ILMN_1791057	IFNAR2	0.485339991
ILMN_1749011	NECAP2	0.485335054
ILMN_2414325	TNFAIP8	0.485306836
ILMN_1754660	ZCCHC24	0.485046115
ILMN_2231051	TCP11L2	0.484915625
ILMN_1699711	DEDD	0.484649898
ILMN_1757877	HCFC1R1	0.484377354
ILMN_1681679	TSPO	0.484360858
ILMN_1743130	PTGFRN	0.483998806
ILMN_2205050	PRKX	0.483930978
ILMN_3200140	LOC645638	0.483852466
ILMN_1712950	PFN1	0.483803209
ILMN_1716547	NAGK	0.483708967
ILMN_2162860	SLFN11	0.483690165
ILMN_1764036	P2RY14	0.483557643
ILMN_1702229	CECR6	0.483535628
ILMN_1808501	SH3KBP1	0.483483571
ILMN_1676241	BCOR	0.483288988
ILMN_1808114	LYVE1	0.483131065
ILMN_1726809	BHLHE41	0.483042254
ILMN_1855286	C6orf204	0.483003621
ILMN_2382500	MS4A14	0.482912847
ILMN_1683980	PLEKHM2	0.482798316
ILMN_2062714	PTGDR	0.482783702
ILMN_1657983	TERF2IP	0.482639283
ILMN_1700935	HAS2	0.482634042
ILMN_2088990	CXorf21	0.482466462
ILMN_1682799	STAMBPL1	0.482163417
ILMN_1869109	NUCKS1	0.48210558
ILMN_1790689	CRISPLD2	0.481915025
ILMN_1663313	AMY1A	0.481831892
ILMN_1709549	PLEKHM1	0.481831437
ILMN_1763907	CENPW	0.481747969
ILMN_2206722	FER1L4	0.481574905
ILMN_1693394	BCKDK	0.481319103
ILMN_2367020	SEC61G	0.481262606
ILMN_1661266	HLA-DQB1	0.48122947
ILMN_3220265	CP	0.481117153
ILMN_1740842	SALL2	0.481065402
ILMN_2056032	CD99	0.480994092
ILMN_1701643	GDPD5	0.480725037
ILMN_1807088	TTC33	0.480501584
ILMN_1776105	PSPH	0.480294887
ILMN_2308849	MYADM	0.480151488
ILMN_1660817	DDB2	0.48010761
ILMN_1704380	INCA1	0.479892945
ILMN_1809583	CREBBP	0.479490676
ILMN_1681301	AIM2	0.479172652
ILMN_1810584	IL1R1	0.478315438
ILMN_2121437	NCL	0.478175501
ILMN_3240793	OR2A4	0.477860348
ILMN_1815745	SOX4	0.477196671
ILMN_1695356	RNF166	0.477104279
ILMN_1670926	CHST15	0.476885777
ILMN_1751432	OR9K2	0.476769892
ILMN_1675848	MYL12A	0.476734193
ILMN_1738116	TMEM119	0.476661404
ILMN_2353202	PTK7	0.476593363
ILMN_3246957	DKFZp666P158	0.476128154
ILMN_1691290	CELSR3	0.476020423
ILMN_1721344	MOBKL2A	0.475910241
ILMN_1737918	C1QA	0.475572118
ILMN_1698307	DBNL	0.475506082
ILMN_1691892	TAGLN2	0.475317368
ILMN_1651346	TICAM2	0.475312666
ILMN_1741727	QPCT	0.475237321
ILMN_1673804	ZNF426	0.475000205
ILMN_1811313	SLIT3	0.474998531
ILMN_1773276	WHAMML2	0.47453312
ILMN_1746695	FLJ40330	0.474396902
ILMN_2329165	MYO1C	0.474317428
ILMN_1757074	GNG10	0.474209124
ILMN_1778561	WEE1	0.474073424
ILMN_2232121	GPR65	0.474050626
ILMN_1786168	LOC400464	0.474003733
ILMN_1898771	TCRA	0.473887623
ILMN_1657129	SKAP2	0.473805901
ILMN_1701558	MAP1A	0.473642983
ILMN_1755758	RIF1	0.473496302
ILMN_2160476	CCL22	0.473323533
ILMN_1763386	BID	0.473261071
ILMN_1730777	KRT19	0.473257177
ILMN_1808405	HLA-DQA1	0.473094491
ILMN_1708537	RBPJ	0.473003542
ILMN_2267383	CU687675	0.472629627
ILMN_1654560	TLR1	0.472266774
ILMN_1661717	TFDP1	0.472065305
ILMN_2404063	APP	0.471859854
ILMN_1792587	VPS4B	0.471590366
ILMN_1673757	CASP8	0.471529578

	GOTERM_MF_FAT	identical protein binding	57	1.90E-02	4.00E-01
	GOTERM_MF_FAT	protein dimerization activity	49	2.40E-02	4.10E-01
	GOTERM_MF_FAT	protein homodimerization activity	29	1.20E-01	8.00E-01
**Annotation Cluster 69**	**Enrichment Score: 1.4**		**Count**	**P_Value**	**Benjamini**
	GOTERM_BP_FAT	cell separation during cytokinesis	3	2.60E-02	3.70E-01
	GOTERM_BP_FAT	cytokinetic process	3	4.10E-02	4.70E-01
	GOTERM_BP_FAT	cytokinesis	7	6.10E-02	5.60E-01
**Annotation Cluster 70**	**Enrichment Score: 1.38**		**Count**	**P_Value**	**Benjamini**
	UP_SEQ_FEATURE	glycosylation site:O-linked (Gal...)	7	4.00E-06	1.20E-03
	SP_PIR_KEYWORDS	complement pathway	6	3.00E-02	1.80E-01
	GOTERM_BP_FAT	complement activation, classical pathway	6	4.60E-02	4.90E-01
	GOTERM_BP_FAT	humoral immune response mediated by circulating immunoglobulin	6	5.90E-02	5.60E-01
	PIR_SUPERFAMILY	PIRSF002477:complement subcomponent C1q chain A	3	1.40E-01	1.00E+00
	UP_SEQ_FEATURE	domain:Collagen-like	5	1.80E-01	1.00E+00
	UP_SEQ_FEATURE	domain:C1q	4	3.00E-01	1.00E+00
	INTERPRO	Complement C1q protein	4	3.50E-01	9.90E-01
	SMART	C1Q	4	4.60E-01	9.70E-01
**Annotation Cluster 71**	**Enrichment Score: 1.37**		**Count**	**P_Value**	**Benjamini**
	GOTERM_BP_FAT	myeloid cell differentiation	15	4.50E-03	1.10E-01
	GOTERM_BP_FAT	erythrocyte differentiation	7	7.30E-02	6.20E-01
	GOTERM_BP_FAT	homeostasis of number of cells	12	8.40E-02	6.50E-01
	GOTERM_BP_FAT	erythrocyte homeostasis	7	1.20E-01	7.40E-01
**Annotation Cluster 72**	**Enrichment Score: 1.35**		**Count**	**P_Value**	**Benjamini**
	INTERPRO	von Willebrand factor, type A	12	1.30E-02	4.70E-01
	SMART	VWA	12	4.10E-02	6.10E-01
	UP_SEQ_FEATURE	domain:VWFA 2	4	8.40E-02	9.80E-01
	UP_SEQ_FEATURE	domain:VWFA	8	8.80E-02	9.80E-01
**Annotation Cluster 73**	**Enrichment Score: 1.34**		**Count**	**P_Value**	**Benjamini**
	GOTERM_MF_FAT	tumor necrosis factor receptor superfamily binding	8	3.50E-03	1.60E-01
	INTERPRO	Tumour necrosis factor-like	8	2.50E-02	5.80E-01
	INTERPRO	Tumor Necrosis Factor	4	9.50E-02	8.60E-01
	SMART	TNF	4	1.40E-01	8.10E-01
	GOTERM_MF_FAT	tumor necrosis factor receptor binding	4	1.60E-01	8.60E-01
**Annotation Cluster 74**	**Enrichment Score: 1.34**		**Count**	**P_Value**	**Benjamini**
	INTERPRO	Hyalin	3	1.20E-02	4.60E-01
	UP_SEQ_FEATURE	domain:Sushi 1	8	1.80E-02	7.40E-01
	UP_SEQ_FEATURE	domain:Sushi 2	8	1.80E-02	7.40E-01
	SP_PIR_KEYWORDS	sushi	9	2.10E-02	1.40E-01
	INTERPRO	Sushi/SCR/CCP	9	2.80E-02	6.00E-01
	INTERPRO	Complement control module	9	3.10E-02	6.20E-01
	UP_SEQ_FEATURE	domain:Sushi 3	6	4.50E-02	9.10E-01
	SMART	CCP	9	6.60E-02	6.50E-01
	UP_SEQ_FEATURE	domain:Sushi 5	3	4.00E-01	1.00E+00
	UP_SEQ_FEATURE	domain:Sushi 4	3	5.10E-01	1.00E+00
**Annotation Cluster 75**	**Enrichment Score: 1.33**		**Count**	**P_Value**	**Benjamini**
	GOTERM_BP_FAT	regulation of protein modification process	32	1.20E-02	2.30E-01
	GOTERM_BP_FAT	regulation of protein amino acid phosphorylation	21	1.60E-02	2.70E-01
	GOTERM_BP_FAT	regulation of peptidyl-tyrosine phosphorylation	11	1.80E-02	2.90E-01
	GOTERM_BP_FAT	positive regulation of phosphorylation	13	3.50E-02	4.30E-01
	GOTERM_BP_FAT	negative regulation of protein modification process	15	3.50E-02	4.30E-01
	GOTERM_BP_FAT	negative regulation of cellular protein metabolic process	20	4.20E-02	4.70E-01
	GOTERM_BP_FAT	positive regulation of phosphorus metabolic process	13	4.20E-02	4.70E-01
	GOTERM_BP_FAT	positive regulation of phosphate metabolic process	13	4.20E-02	4.70E-01
	GOTERM_BP_FAT	negative regulation of protein metabolic process	20	5.80E-02	5.50E-01
	GOTERM_BP_FAT	positive regulation of protein modification process	20	5.80E-02	5.50E-01
	GOTERM_BP_FAT	regulation of cellular protein metabolic process	42	7.80E-02	6.30E-01
	GOTERM_BP_FAT	positive regulation of protein amino acid phosphorylation	11	8.60E-02	6.60E-01
	GOTERM_BP_FAT	positive regulation of peptidyl-tyrosine phosphorylation	7	1.00E-01	7.00E-01
	GOTERM_BP_FAT	positive regulation of protein metabolic process	23	1.20E-01	7.40E-01
	GOTERM_BP_FAT	positive regulation of cellular protein metabolic process	22	1.30E-01	7.60E-01
**Annotation Cluster 76**	**Enrichment Score: 1.32**		**Count**	**P_Value**	**Benjamini**
	UP_SEQ_FEATURE	domain:FCH	5	3.20E-02	8.40E-01
	INTERPRO	Fps/Fes/Fer/CIP4 homology	5	4.40E-02	7.10E-01
	SMART	FCH	5	7.50E-02	6.50E-01
**Annotation Cluster 77**	**Enrichment Score: 1.32**		**Count**	**P_Value**	**Benjamini**
	UP_SEQ_FEATURE	nucleotide phosphate-binding region:GTP	32	2.90E-03	3.20E-01
	SP_PIR_KEYWORDS	gtp-binding	34	4.10E-03	4.00E-02
	GOTERM_MF_FAT	GTPase activity	25	6.60E-03	2.40E-01
	GOTERM_MF_FAT	GTP binding	37	1.40E-02	3.60E-01
	SP_PIR_KEYWORDS	prenylation	18	1.60E-02	1.20E-01
	GOTERM_MF_FAT	guanyl ribonucleotide binding	37	2.00E-02	4.10E-01
	GOTERM_MF_FAT	guanyl nucleotide binding	37	2.00E-02	4.10E-01
	UP_SEQ_FEATURE	short sequence motif:Effector region	11	7.00E-02	9.60E-01
	GOTERM_BP_FAT	small GTPase mediated signal transduction	29	7.40E-02	6.20E-01
	UP_SEQ_FEATURE	lipid moiety-binding region:S-geranylgeranyl cysteine	11	1.00E-01	9.90E-01
	INTERPRO	Ras	12	1.90E-01	9.60E-01
	INTERPRO	Ras GTPase	12	2.70E-01	9.80E-01
	INTERPRO	Ras small GTPase, Rab type	6	3.70E-01	9.90E-01
	INTERPRO	Small GTP-binding protein	13	4.10E-01	1.00E+00
	SMART	RAB	6	5.20E-01	9.80E-01
**Annotation Cluster 78**	**Enrichment Score: 1.28**		**Count**	**P_Value**	**Benjamini**
	INTERPRO	Calponin-like actin-binding	12	5.40E-03	3.50E-01
	UP_SEQ_FEATURE	domain:CH	8	1.40E-02	6.80E-01
	SMART	CH	12	1.80E-02	4.50E-01
	INTERPRO	SM22/calponin	4	3.10E-02	6.20E-01
	INTERPRO	Calponin repeat	3	5.30E-02	7.50E-01
	COG_ONTOLOGY	Cytoskeleton	5	6.10E-02	7.80E-01
	UP_SEQ_FEATURE	domain:CH 1	4	2.30E-01	1.00E+00
	UP_SEQ_FEATURE	domain:CH 2	4	2.30E-01	1.00E+00
	INTERPRO	Actinin-type, actin-binding, conserved site	3	4.50E-01	1.00E+00
**Annotation Cluster 79**	**Enrichment Score: 1.21**		**Count**	**P_Value**	**Benjamini**
	UP_SEQ_FEATURE	propeptide:Removed in mature form	27	2.10E-03	2.70E-01
	UP_SEQ_FEATURE	lipid moiety-binding region:GPI-anchor amidated serine	7	1.20E-01	9.90E-01
	SP_PIR_KEYWORDS	phosphatidylinositol linkage	5	1.20E-01	4.80E-01
	SP_PIR_KEYWORDS	gpi-anchor	12	1.50E-01	5.50E-01
	GOTERM_CC_FAT	anchored to membrane	20	1.90E-01	7.90E-01
**Annotation Cluster 80**	**Enrichment Score: 1.2**		**Count**	**P_Value**	**Benjamini**
	GOTERM_BP_FAT	positive regulation of myeloid cell differentiation	9	1.50E-03	4.80E-02
	GOTERM_BP_FAT	regulation of myeloid cell differentiation	11	2.10E-02	3.20E-01
	GOTERM_BP_FAT	positive regulation of erythrocyte differentiation	4	3.60E-02	4.30E-01
	GOTERM_BP_FAT	negative regulation of granulocyte differentiation	3	4.10E-02	4.70E-01

ILMN_1861414	BF593077	-0.450741044
ILMN_1827736	PDK3	-0.450796268
ILMN_3247458	HSPA7	-0.450913092
ILMN_1901774	BF513180	-0.450919279
ILMN_1657010	FAM90A12	-0.451001404
ILMN_1797794	AK126307	-0.45100599
ILMN_1860789	BM974067	-0.451014585
ILMN_1738938	TIMM8B	-0.45102886
ILMN_1789600	C6orf127	-0.451056687
ILMN_1751051	C7orf25	-0.451195743
ILMN_1740048	MYOD1	-0.451229485
ILMN_2358628	ADK	-0.451402977
ILMN_2195914	GGH	-0.451413565
ILMN_2137208	GOLT1A	-0.451453281
ILMN_1676660	DNAH6	-0.451564442
ILMN_2227368	SELT	-0.451574652
ILMN_2339006	KIAA0564	-0.451605352
ILMN_1694491	CCNG1	-0.451633938
ILMN_1720496	GUCY1A2	-0.451653857
ILMN_1690621	GPR98	-0.451660189
ILMN_1683716	DKFZp434F1622	-0.451740506
ILMN_1682062	RYR1	-0.451880225
ILMN_1673542	AKAP14	-0.452012233
ILMN_2104106	XPR1	-0.452013986
ILMN_1714446	PLG	-0.452282359
ILMN_2092536	MOBKL3	-0.452291934
ILMN_1672354	AX721218	-0.452365062
ILMN_3310543	MIR1258	-0.452415033
ILMN_2103014	CCDC104	-0.452426867
ILMN_2156936	SCCPDH	-0.452663957
ILMN_1773395	BLOC1S1-RDH5	-0.452721889
ILMN_3261449	LOC100130815	-0.452804163
ILMN_1912130	AK097103	-0.453125598
ILMN_3238785	SNHG9	-0.453156423
ILMN_2070792	ACSM5	-0.453181171
ILMN_1777066	NIF3L1	-0.453192213
ILMN_1746952	SLC25A48	-0.4532047
ILMN_1737398	PTPLAD1	-0.453243615
ILMN_1813544	OXCT1	-0.453478994
ILMN_1755423	PTCRA	-0.453532188
ILMN_1759396	NNT	-0.453602816
ILMN_1708779	GNLY	-0.45360598
ILMN_1782274	NYX	-0.453815961
ILMN_1665033	NPR3	-0.453887202
ILMN_1735052	ULK1	-0.454000204
ILMN_3309914	MIR660	-0.454000991
ILMN_1749804	ADSSL1	-0.454054316
ILMN_2151739	CAT	-0.454057758
ILMN_3277332	OR7E14P	-0.454085042
ILMN_1773742	DNAJB9	-0.454178639
ILMN_2390609	ANK3	-0.454202199
ILMN_1882041	H16080	-0.454240368
ILMN_1762207	SGSM1	-0.454240938
ILMN_2068951	PDHA2	-0.454520644
ILMN_1665775	MOSC2	-0.454531378
ILMN_1832155	DCUN1D5	-0.454627604
ILMN_2381064	TPD52	-0.45476704
ILMN_1805765	CMYA5	-0.454804169
ILMN_2167252	FAM151A	-0.454942672
ILMN_1776685	PCDH15	-0.45504211
ILMN_1757867	MTM	-0.455355537
ILMN_3207902	GUSBP10	-0.455521624
ILMN_1856998	BC053535	-0.455544595
ILMN_1698769	FAM75D1	-0.455641982
ILMN_1785404	FGFBP1	-0.455719865
ILMN_1735719	UNG	-0.455879648
ILMN_1697611	AMBN	-0.455902367
ILMN_1676545	AK093382	-0.456116206
ILMN_1693230	HYAL1	-0.456171447
ILMN_3183789	UQCRFS1	-0.456197826
ILMN_1699562	EFCAB1	-0.456291339
ILMN_1770306	OR51B2	-0.456344261
ILMN_1735402	DCAF5	-0.456612547
ILMN_1771800	PRKCA	-0.456646736
ILMN_2219681	RBP2	-0.456658887
ILMN_1798043	LOC339674	-0.456764597
ILMN_1667564	ALDH3A2	-0.456765708
ILMN_1699648	ZNF516	-0.456892702
ILMN_1810233	UGT2B11	-0.457026629
ILMN_1776653	SCML1	-0.457033543
ILMN_1778242	CALM1	-0.457269728
ILMN_2344204	PRR13	-0.457275904
ILMN_1813344	C20orf7	-0.457424388
ILMN_2389064	C15orf48	-0.457448137
ILMN_3301551	LPA	-0.457547843
ILMN_1662578	C1GALT1	-0.457602281
ILMN_1741970	JUP	-0.457630953
ILMN_3306028	BM932296	-0.457676213
ILMN_1903651	BX417275	-0.457696919
ILMN_1654427	AF086464	-0.457988599
ILMN_1653228	C3orf22	-0.458255238
ILMN_1744941	TCOF1	-0.458279197
ILMN_3241522	ASS1	-0.458393265
ILMN_1853631	BX115738	-0.458503464
ILMN_1808487	PLA2G12B	-0.458559618
ILMN_1764959	FAM154A	-0.45859741
ILMN_1658861	FAM86B1	-0.458619468
ILMN_2407346	LDHD	-0.458776235
ILMN_3271630	FGGY	-0.458851336
ILMN_2045729	WDR12	-0.458861916
ILMN_1734640	SDHA	-0.45897001

ILMN_3309021	MIR142	0.471396045
ILMN_1791483	PDE4D	0.471165152
ILMN_2136455	C3orf64	0.470851841
ILMN_1769702	GPAA1	0.470819957
ILMN_2136133	PABPC1	0.470772137
ILMN_2321578	P2RY10	0.470628219
ILMN_1860540	AA709087	0.470532362
ILMN_3220952	ANKRD36	0.470491107
ILMN_1661646	BANK1	0.470368566
ILMN_2331087	MS4A7	0.470165988
ILMN_3176090	RCC2	0.470072646
ILMN_1796712	S100A10	0.470064857
ILMN_1657836	PLEKHG2	0.469990723
ILMN_3253901	HAVCR1	0.469685103
ILMN_3247494	SNORA36B	0.469639955
ILMN_1684836	AKAP12	0.469105751
ILMN_2348905	CTLA4	0.469092687
ILMN_1785646	PMP22	0.469023995
ILMN_2351795	C18orf1	0.468997297
ILMN_1707070	PCOLCE	0.468969493
ILMN_2360448	TAF1A	0.468931035
ILMN_1709747	EXOG	0.468906314
ILMN_1652787	PIK3AP1	0.468880644
ILMN_1780898	PRKCH	0.468726858
ILMN_1898518	GFRA1	0.468572614
ILMN_1809889	CCDC117	0.468515555
ILMN_1754279	FBXW7	0.468486392
ILMN_1712112	RCAN1	0.468451597
ILMN_1680453	ITM2C	0.468073991
ILMN_1803686	ADA	0.467907507
ILMN_3247893	F8A1	0.467696547
ILMN_1796738	SPAST	0.467505404
ILMN_1815656	SERINC3	0.467409139
ILMN_1706935	CCDC136	0.467375843
ILMN_3242485	TLK2	0.467235007
ILMN_3242067	SNORA58	0.466849041
ILMN_1696243	FLJ23152	0.466774251
ILMN_1805693	GMIP	0.466745036
ILMN_1802456	DCTD	0.46643135
ILMN_1751034	ITPRIPL2	0.466403331
ILMN_1663618	STAT3	0.466012799
ILMN_1864900	MIAT	0.465927198
ILMN_1692398	CNTNAP1	0.465412821
ILMN_1748488	TXNL4B	0.464957582
ILMN_1714600	NAV3	0.464936139
ILMN_3245764	SNORA39	0.464882387
ILMN_1746565	CD6	0.464790684
ILMN_1789846	ITGB6	0.464763999
ILMN_1916931	BX092585	0.464589473
ILMN_2390299	PSMB8	0.464119051
ILMN_1806782	ZNF235	0.464077255
ILMN_1679809	GSTP1	0.463934385
ILMN_1754249	TARP	0.463845415
ILMN_1713384	TSHZ2	0.463518258
ILMN_1763842	PTRH1	0.463432626
ILMN_1657421	YWHAE	0.463282516
ILMN_1786357	ARAP1	0.463051253
ILMN_3215206	SMN1	0.462993453
ILMN_1677827	TLR7	0.462871734
ILMN_1786015	CTCF	0.462699708
ILMN_2313901	PAM	0.462475655
ILMN_1784320	ELMO1	0.462336228
ILMN_1732885	SLX4	0.462271316
ILMN_1732921	ITGB8	0.462230401
ILMN_1779147	ENC1	0.46219442
ILMN_2140059	LAMA4	0.461901018
ILMN_1705750	TGM2	0.461865708
ILMN_3299558	SFRS18	0.461553932
ILMN_1767349	ABCB4	0.461531825
ILMN_1775073	ZNF273	0.461271332
ILMN_1777190	CFD	0.461258106
ILMN_1809484	TMOD3	0.461247894
ILMN_2188264	CYR61	0.461218262
ILMN_1740466	FAM46A	0.461145433
ILMN_1653514	RBBP4	0.461074783
ILMN_1832656	AW578902	0.460798661
ILMN_2196337	C12orf11	0.460637976
ILMN_2386849	DSE	0.460379309
ILMN_1814789	UBAP2L	0.460294121
ILMN_2194577	PLVAP	0.460281384
ILMN_1741003	ANXA5	0.460209295
ILMN_1808707	FSCN1	0.4601333
ILMN_1730940	KLHDC3	0.460024764
ILMN_1656951	APCDD1	0.459955462
ILMN_2170814	LAMP3	0.459909732
ILMN_3294114	NA	0.459892135
ILMN_2280568	ANKRD36	0.459614123
ILMN_1685202	KRBA2	0.459523259
ILMN_1814985	PDLIM7	0.459433306
ILMN_1757646	UFM1	0.459334629
ILMN_1910180	AK024680	0.459131464
ILMN_1883937	CR936671	0.458971842
ILMN_1684306	S100A4	0.458842014
ILMN_1740015	CD14	0.458804442
ILMN_1655625	GPATCH1	0.458604763
ILMN_1743987	TRPM2	0.458595151
ILMN_1800276	RCN1	0.458526824
ILMN_2163873	FNDC1	0.458298103
ILMN_2370336	MS4A4A	0.458186683
ILMN_1805750	IFITM3	0.457809342
ILMN_2154950	ZNF423	0.457682544

	GOTERM_BP_FAT	regulation of granulocyte differentiation	3	7.90E-02	6.40E-01
	GOTERM_BP_FAT	negative regulation of myeloid leukocyte differentiation	4	1.10E-01	7.20E-01
	GOTERM_BP_FAT	positive regulation of myeloid leukocyte differentiation	4	1.20E-01	7.50E-01
	GOTERM_BP_FAT	regulation of erythrocyte differentiation	4	1.20E-01	7.50E-01
	GOTERM_BP_FAT	negative regulation of myeloid cell differentiation	5	1.50E-01	7.90E-01
	GOTERM_BP_FAT	regulation of myeloid leukocyte differentiation	6	1.50E-01	7.90E-01
	GOTERM_BP_FAT	regulation of osteoclast differentiation	3	4.50E-01	9.80E-01
**Annotation Cluster 81**	**Enrichment Score: 1.14**		**Count**	**P_Value**	**Benjamini**
	UP_SEQ_FEATURE	repeat:LRR 11	14	1.00E-02	6.40E-01
	INTERPRO	Cysteine-rich flanking region, C-terminal	13	1.20E-02	4.60E-01
	UP_SEQ_FEATURE	repeat:LRR 10	15	1.40E-02	6.80E-01
	UP_SEQ_FEATURE	repeat:LRR 5	24	1.70E-02	7.30E-01
	UP_SEQ_FEATURE	repeat:LRR 9	16	2.00E-02	7.50E-01
	UP_SEQ_FEATURE	repeat:LRR 6	22	2.10E-02	7.40E-01
	UP_SEQ_FEATURE	repeat:LRR 8	17	2.30E-02	7.60E-01
	UP_SEQ_FEATURE	repeat:LRR 7	19	2.70E-02	8.00E-01
	INTERPRO	Leucine-rich repeat	22	3.80E-02	6.60E-01
	SMART	LRRCT	13	3.90E-02	6.20E-01
	UP_SEQ_FEATURE	repeat:LRR 4	24	4.10E-02	8.90E-01
	INTERPRO	Leucine-rich repeat, typical subtype	15	4.60E-02	7.20E-01
	UP_SEQ_FEATURE	repeat:LRR 12	11	5.50E-02	9.40E-01
	INTERPRO	Leucine-rich repeat, cysteine-rich flanking region, N-terminal	12	5.70E-02	7.70E-01
	SMART	LRR_TYP	15	1.40E-01	8.10E-01
	SMART	LRRNT	12	1.50E-01	8.10E-01
	UP_SEQ_FEATURE	repeat:LRR 14	7	1.70E-01	1.00E+00
	UP_SEQ_FEATURE	repeat:LRR 3	24	1.70E-01	1.00E+00
	UP_SEQ_FEATURE	repeat:LRR 1	25	1.80E-01	1.00E+00
	UP_SEQ_FEATURE	repeat:LRR 2	25	1.90E-01	1.00E+00
	UP_SEQ_FEATURE	repeat:LRR 13	8	1.90E-01	1.00E+00
	SP_PIR_KEYWORDS	leucine-rich repeat	25	1.90E-01	6.20E-01
	UP_SEQ_FEATURE	repeat:LRR 15	5	3.90E-01	1.00E+00
	UP_SEQ_FEATURE	repeat:LRR 18	3	5.10E-01	1.00E+00
	UP_SEQ_FEATURE	repeat:LRR 16	4	5.30E-01	1.00E+00
	UP_SEQ_FEATURE	repeat:LRR 17	3	6.60E-01	1.00E+00
**Annotation Cluster 82**	**Enrichment Score: 1.13**		**Count**	**P_Value**	**Benjamini**
	GOTERM_BP_FAT	regulation of organelle organization	25	1.50E-02	2.60E-01
	GOTERM_BP_FAT	negative regulation of cellular component organization	18	1.80E-02	2.90E-01
	GOTERM_BP_FAT	regulation of cellular component biogenesis	17	3.60E-02	4.30E-01
	INTERPRO	Thymosin beta-4, chordata	3	3.70E-02	6.60E-01
	INTERPRO	Thymosin beta-4	3	3.70E-02	6.60E-01
	GOTERM_BP_FAT	regulation of actin cytoskeleton organization	12	4.20E-02	4.70E-01
	GOTERM_BP_FAT	regulation of protein complex assembly	12	4.50E-02	4.80E-01
	PIR_SUPERFAMILY	PIRSF001828:Thymosin_beta	3	4.80E-02	9.50E-01
	SMART	THY	3	5.20E-02	6.20E-01
	GOTERM_BP_FAT	regulation of actin filament-based process	12	5.20E-02	5.20E-01
	GOTERM_BP_FAT	negative regulation of organelle organization	11	5.50E-02	5.40E-01
	GOTERM_BP_FAT	sequestering of actin monomers	3	5.90E-02	5.60E-01
	GOTERM_BP_FAT	regulation of cytoskeleton organization	15	8.60E-02	6.60E-01
	GOTERM_BP_FAT	regulation of actin polymerization or depolymerization	8	1.30E-01	7.50E-01
	GOTERM_BP_FAT	regulation of actin filament length	8	1.40E-01	7.80E-01
	GOTERM_BP_FAT	regulation of actin filament polymerization	7	1.70E-01	8.20E-01
	GOTERM_BP_FAT	negative regulation of cytoskeleton organization	7	1.80E-01	8.30E-01
	GOTERM_BP_FAT	regulation of protein polymerization	8	1.90E-01	8.40E-01
	GOTERM_BP_FAT	negative regulation of protein complex assembly	5	2.10E-01	8.60E-01
	GOTERM_BP_FAT	negative regulation of actin filament polymerization	4	3.00E-01	9.30E-01
	GOTERM_BP_FAT	negative regulation of protein polymerization	4	3.20E-01	9.40E-01
**Annotation Cluster 83**	**Enrichment Score: 1.11**		**Count**	**P_Value**	**Benjamini**
	GOTERM_BP_FAT	myeloid cell activation during immune response	6	6.20E-03	1.40E-01
	GOTERM_BP_FAT	myeloid leukocyte mediated immunity	4	8.00E-02	6.40E-01
	GOTERM_BP_FAT	leukocyte degranulation	3	2.00E-01	8.60E-01
	GOTERM_BP_FAT	regulated secretory pathway	3	3.60E-01	9.50E-01
**Annotation Cluster 84**	**Enrichment Score: 1.11**		**Count**	**P_Value**	**Benjamini**
	GOTERM_MF_FAT	cytokine receptor activity	10	9.50E-03	2.80E-01
	UP_SEQ_FEATURE	short sequence motif:Box 1 motif	4	1.80E-01	1.00E+00
	UP_SEQ_FEATURE	short sequence motif:WSXWS motif	4	2.70E-01	1.00E+00
**Annotation Cluster 85**	**Enrichment Score: 1.09**		**Count**	**P_Value**	**Benjamini**
	GOTERM_BP_FAT	JAK-STAT cascade	8	1.60E-02	2.70E-01
	INTERPRO	STAT transcription factor, all-alpha	3	7.20E-02	8.00E-01
	INTERPRO	STAT transcription factor, DNA-binding	3	7.20E-02	8.00E-01
	INTERPRO	STAT transcription factor, protein interaction	3	7.20E-02	8.00E-01
	INTERPRO	STAT transcription factor, core	3	7.20E-02	8.00E-01
	INTERPRO	STAT transcription factor, DNA-binding, subdomain	3	7.20E-02	8.00E-01
	PIR_SUPERFAMILY	PIRSF015568:signal transducer and transcription activator STAT5A	3	9.10E-02	9.70E-01
	BBID	48.mice_minus_JAKs_and_STATs	3	6.40E-01	1.00E+00
**Annotation Cluster 86**	**Enrichment Score: 1.09**		**Count**	**P_Value**	**Benjamini**
	GOTERM_BP_FAT	leukocyte migration	9	4.10E-02	4.60E-01
	GOTERM_BP_FAT	lymphocyte chemotaxis	3	7.90E-02	6.40E-01
	GOTERM_BP_FAT	leukocyte chemotaxis	6	1.10E-01	7.20E-01
	GOTERM_BP_FAT	cell chemotaxis	6	1.30E-01	7.60E-01
**Annotation Cluster 87**	**Enrichment Score: 1.06**		**Count**	**P_Value**	**Benjamini**
	BIOCARTA	IL 17 Signaling Pathway	7	6.20E-03	2.10E-01
	BBID	81.Structure_of_Caps_and_SMACs	5	1.70E-01	9.50E-01
	BBID	7.IL17_hematopoesis	3	6.40E-01	1.00E+00
**Annotation Cluster 88**	**Enrichment Score: 1.06**		**Count**	**P_Value**	**Benjamini**
	INTERPRO	Link	4	5.90E-02	7.70E-01
	SMART	LINK	4	9.00E-02	7.00E-01
	INTERPRO	C-type lectin-like	11	9.00E-02	8.50E-01
	GOTERM_MF_FAT	hyaluronic acid binding	4	1.30E-01	8.20E-01
**Annotation Cluster 89**	**Enrichment Score: 1.05**		**Count**	**P_Value**	**Benjamini**
	GOTERM_BP_FAT	integrin-mediated signaling pathway	13	2.80E-03	7.90E-02
	GOTERM_CC_FAT	integrin complex	7	1.30E-02	1.90E-01
	INTERPRO	Plexin/semaphorin/integrin	8	2.50E-02	5.80E-01
	SP_PIR_KEYWORDS	integrin	7	3.80E-02	2.20E-01
	SMART	PSI	8	5.70E-02	6.40E-01
	INTERPRO	Integrin beta subunit, subgroup	3	7.20E-02	8.00E-01
	UP_SEQ_FEATURE	domain:VWFA	8	8.80E-02	9.80E-01
	PIR_SUPERFAMILY	PIRSF002512:Integrin_B	3	9.10E-02	9.70E-01
	PIR_SUPERFAMILY	PIRSF002512:integrin, beta subunit	3	9.10E-02	9.70E-01
	INTERPRO	Integrin beta subunit, N-terminal	3	9.10E-02	8.50E-01
	UP_SEQ_FEATURE	region of interest:Cysteine-rich tandem repeats	3	1.00E-01	9.90E-01
	INTERPRO	Integrin beta subunit, C-terminal	3	1.10E-01	8.90E-01

ILMN_3237241	FAM32A	-0.458991045
ILMN_1664993	SLX1B-SULT1A4	-0.459082102
ILMN_1706441	UPK3B	-0.459136745
ILMN_1764175	GPD1	-0.459353293
ILMN_1803218	GPR139	-0.459376636
ILMN_3238143	D2HGDH	-0.45940668
ILMN_3235584	TRIM66	-0.459694136
ILMN_1729650	PEX7	-0.459720821
ILMN_1673503	CACNB4	-0.459794998
ILMN_1783835	ZNF845	-0.459836979
ILMN_2134039	ACN9	-0.46002319
ILMN_1712999	MGAM	-0.460039898
ILMN_1804090	SLC25A10	-0.460119825
ILMN_1702218	POLI	-0.460172067
ILMN_1905072	CX758427	-0.460260193
ILMN_3226405	PRAMEF11	-0.46040765
ILMN_1701507	EHHADH	-0.460466999
ILMN_2381697	P4HA2	-0.460477882
ILMN_2205350	NDUFAF4	-0.460488426
ILMN_1733390	LARP1B	-0.460662677
ILMN_2393968	SYNJ1	-0.460704607
ILMN_2230579	RABGEF1	-0.460812725
ILMN_1685917	EMCN	-0.460818784
ILMN_1796749	KIF1C	-0.460834566
ILMN_2195721	KRTAP21-1	-0.461018972
ILMN_2224333	C3orf65	-0.461713105
ILMN_2377240	AKTIP	-0.461812646
ILMN_1671619	HMX2	-0.461888676
ILMN_2207504	LEP	-0.461921592
ILMN_2322747	ARHGAP5	-0.462043186
ILMN_1776263	TMEM174	-0.462100938
ILMN_3262348	IP6K2	-0.462248323
ILMN_2365595	GMPR2	-0.46226139
ILMN_1804945	ZNF667	-0.462459486
ILMN_1698731	SEC31B	-0.462895527
ILMN_1718520	RNLS	-0.463077405
ILMN_1728450	MCART3P	-0.463132298
ILMN_1719032	LSM3	-0.463370716
ILMN_1687291	CDNF	-0.463411445
ILMN_2324574	RALGAPA1	-0.463483866
ILMN_1769083	GSTA3	-0.463786411
ILMN_1732300	POLR2C	-0.464124933
ILMN_3292690	NA	-0.464153254
ILMN_1779597	CIDEC	-0.464278563
ILMN_1802246	KLK10	-0.464399602
ILMN_2212156	PPIG	-0.465143359
ILMN_3269076	NA	-0.46521511
ILMN_1849399	LOC100630918	-0.46532251
ILMN_1898826	BG564551	-0.465341168
ILMN_1704029	AK000451	-0.465501387
ILMN_1662081	KRTAP3-2	-0.465645261
ILMN_1703346	CHRNE	-0.465776575
ILMN_2293631	MGAT5B	-0.465980196
ILMN_1752574	LEAP2	-0.466098109
ILMN_2313821	AIFM1	-0.466356791
ILMN_1808938	PIGF	-0.466546479
ILMN_1808262	OR8K3	-0.466745479
ILMN_1785069	KIAA1845	-0.466974694
ILMN_1730611	RTN4	-0.467081098
ILMN_1660261	TDRD6	-0.467095652
ILMN_1795839	SCCPDH	-0.467104292
ILMN_1703222	FRS2	-0.467331963
ILMN_2166543	DNAJC6	-0.46760525
ILMN_1775703	TRAPPC6A	-0.467777382
ILMN_1693932	LOC285733	-0.467838956
ILMN_2215194	GCLC	-0.467843617
ILMN_1701293	COX7A2	-0.467935095
ILMN_1686637	RBMXL3	-0.468046283
ILMN_1765770	SYCP2	-0.46805026
ILMN_1850372	AI798827	-0.468120985
ILMN_2162135	C5orf44	-0.468394178
ILMN_1788131	CYP26A1	-0.468438034
ILMN_1750018	SEL1L2	-0.468491133
ILMN_2341363	ATP5A1	-0.468509855
ILMN_1710873	ZNF330	-0.468569181
ILMN_1798546	HDAC6	-0.468631981
ILMN_1791833	PCDHA@	-0.468811156
ILMN_1746426	TOMM70A	-0.468855961
ILMN_2169692	SCN9A	-0.468937429
ILMN_2225735	CRBN	-0.468966624
ILMN_1677866	FOLH1B	-0.469036755
ILMN_1744210	SDHA	-0.469205518
ILMN_1692674	NKD1	-0.469974202
ILMN_1699852	CES4A	-0.470226411
ILMN_1713139	DLGAP2	-0.470278187
ILMN_1749244	LYRM1	-0.470308019
ILMN_1737588	C21orf33	-0.470418676
ILMN_1773200	CP110	-0.470473269
ILMN_1662675	ADAM2	-0.470552435
ILMN_2314244	LRRC23	-0.470745468
ILMN_1725105	EEF1E1	-0.470840988
ILMN_1707741	TBC1D26	-0.47128691
ILMN_3244200	FLJ00326	-0.471331773
ILMN_1892573	BC031234	-0.471396052
ILMN_1837152	CA421506	-0.471439442
ILMN_1679194	UGT2B7	-0.471453417
ILMN_1669366	IFT88	-0.471478787
ILMN_1774028	MTFR1	-0.47151348
ILMN_3263423	LOC100129027	-0.471605747
ILMN_2379560	CDC14B	-0.471802478
ILMN_2302983	TRPM3	-0.471817431

ILMN_1788886	TOX	0.457599277
ILMN_1717207	MMP25	0.457535427
ILMN_2137084	LIN9	0.457451571
ILMN_1690921	STAT2	0.457411543
ILMN_1745963	FOLR2	0.457300409
ILMN_1723042	CLDN3	0.457295007
ILMN_3236021	ANKRD36	0.457293548
ILMN_1682928	CPVL	0.457155899
ILMN_1770085	BTG2	0.457115098
ILMN_1780268	C1orf56	0.456978832
ILMN_2366041	ITM2C	0.456972153
ILMN_1775004	KCNQ3	0.456849586
ILMN_1689004	TNFRSF12A	0.456810235
ILMN_1750153	OR52A1	0.456761581
ILMN_2062524	RBBP4	0.456639033
ILMN_1778723	AMICA1	0.456523593
ILMN_2226304	ANKRD50	0.45628001
ILMN_1651826	BASP1	0.456137492
ILMN_1682761	C17orf87	0.455801889
ILMN_1745583	EXOC1	0.455768392
ILMN_1803988	MCL1	0.455720645
ILMN_2143685	CLDN7	0.455662819
ILMN_1748812	TMEM130	0.455261602
ILMN_1672834	SSH2	0.455165574
ILMN_1715625	C20orf94	0.45494532
ILMN_1673543	PGM2	0.454649266
ILMN_1725090	CTHRC1	0.454574789
ILMN_2101278	RGS18	0.454540448
ILMN_2141482	SERPINF1	0.454461009
ILMN_1781916	AV723037	0.454308945
ILMN_1719905	TLR10	0.454067984
ILMN_2137312	KCNJ5	0.454001416
ILMN_1706455	TTC5	0.453654986
ILMN_1670130	ARID3A	0.453646396
ILMN_1755942	O3FAR1	0.453635652
ILMN_1673023	EP400	0.453546111
ILMN_1740903	C7orf49	0.453471469
ILMN_1806956	ATAD5	0.453336428
ILMN_1815652	O1/O11 and JK2	0.453086729
ILMN_1727917	FAM26E	0.452860364
ILMN_1757019	NTN4	0.452738855
ILMN_1755589	DIP2B	0.452680461
ILMN_1903565	GNA11	0.452672677
ILMN_1699348	MS4A4E	0.452436004
ILMN_2261600	FCGR1B	0.452373964
ILMN_2257015	AGER	0.452369798
ILMN_1715947	HNRNPA1	0.452302743
ILMN_2059744	IL7	0.452256998
ILMN_1911042	PIP4K2A	0.452203346
ILMN_1735467	NPIPL2	0.452095592
ILMN_1793517	RASAL1	0.452059448
ILMN_1769282	FRMD6	0.451980801
ILMN_1806320	PPFIBP1	0.451812455
ILMN_1771057	XAB2	0.451737153
ILMN_1790162	ZC3H11A	0.451485196
ILMN_1671114	ZNF865	0.451440317
ILMN_1708375	IRF1	0.451382231
ILMN_3238803	RASAL3	0.451260258
ILMN_1801383	SMG1	0.451236636
ILMN_1698144	CFH	0.451194786
ILMN_1742001	CD160	0.451060476
ILMN_1654571	FCHO1	0.450815006
ILMN_1786197	NR2F1	0.450661682
ILMN_1744822	BECN1	0.450345375
ILMN_1715639	FLJ00310	0.45030731
ILMN_2094396	LRRC8C	0.450234879
ILMN_1801130	STOML1	0.4500943
ILMN_1715760	HUAT	0.450065684
ILMN_2167416	MR1	0.450000896
ILMN_1799208	CSGALNACT2	0.44978829
ILMN_3287952	CDC27	0.449704602
ILMN_1678841	UBD	0.44958398
ILMN_1807277	IFI30	0.449577291
ILMN_2373831	BTN3A3	0.449406325
ILMN_1665219	LTBP4	0.449341699
ILMN_3231881	PTMA	0.449308018
ILMN_1666444	RBMS1	0.449302623
ILMN_3246292	RPL23A	0.44926722
ILMN_1791478	MTPN	0.449039022
ILMN_1686388	TPM3	0.44899138
ILMN_2244009	LBH	0.448751199
ILMN_2337923	TPD52L1	0.448593361
ILMN_1769229	BCL2A1	0.447954958
ILMN_2092850	HPSE	0.447871525
ILMN_1677581	RPGRIP1L	0.447862061
ILMN_1702301	DOCK10	0.447692744
ILMN_1725427	B2M	0.447571703
ILMN_1748290	FAM175A	0.447540675
ILMN_2391150	FILIP1L	0.447500883
ILMN_1803036	TARBP1	0.447318586
ILMN_1815261	PDIA4	0.447277622
ILMN_1772189	ABCD1	0.447061328
ILMN_1800261	TUBA1B	0.446657078
ILMN_3248176	C2CD4D	0.446652625
ILMN_1736700	ALDOA	0.44662908
ILMN_1783606	MLL5	0.446478136
ILMN_1652825	IL10RA	0.446368695
ILMN_2089340	FAM127B	0.446333669
ILMN_1657683	C1orf198	0.446192535
ILMN_2347234	PRMT1	0.446014955
ILMN_1657627	CBFA2T3	0.445938061
ILMN_2371685	UBE2E1	0.445932641

	INTERPRO	Integrin beta subunit		3	1.10E-01	8.90E-01
	INTERPRO	EGF, extracellular		8	1.10E-01	8.90E-01
	SMART	INB		3	1.20E-01	7.90E-01
	UP_SEQ_FEATURE	repeat:III		3	4.90E-01	1.00E+00
	UP_SEQ_FEATURE	repeat:IV		3	4.90E-01	1.00E+00
	UP_SEQ_FEATURE	repeat:I		3	4.90E-01	1.00E+00
	UP_SEQ_FEATURE	repeat:II		3	4.90E-01	1.00E+00
**Annotation Cluster 90**	**Enrichment Score: 1.04**		**Count**		**P_Value**	**Benjamini**
	GOTERM_CC_FAT	vesicle		58	6.00E-02	5.30E-01
	GOTERM_CC_FAT	cytoplasmic vesicle		55	7.60E-02	5.90E-01
	GOTERM_CC_FAT	membrane-bounded vesicle		48	1.10E-01	6.40E-01
	GOTERM_CC_FAT	cytoplasmic membrane-bounded vesicle		46	1.40E-01	6.80E-01
**Annotation Cluster 91**	**Enrichment Score: 1.03**		**Count**		**P_Value**	**Benjamini**
	GOTERM_BP_FAT	regulation of alpha-beta T cell activation		8	1.00E-02	2.10E-01
	GOTERM_BP_FAT	positive regulation of alpha-beta T cell activation		6	3.00E-02	4.00E-01
	GOTERM_BP_FAT	regulation of alpha-beta T cell proliferation		4	5.60E-02	5.40E-01
	GOTERM_BP_FAT	positive regulation of alpha-beta T cell proliferation		3	1.20E-01	7.50E-01
	GOTERM_BP_FAT	regulation of alpha-beta T cell differentiation		4	2.50E-01	9.00E-01
	GOTERM_BP_FAT	regulation of CD4-positive, alpha beta T cell differentiation		3	2.80E-01	9.20E-01
	GOTERM_BP_FAT	positive regulation of alpha-beta T cell differentiation		3	4.10E-01	9.70E-01
**Annotation Cluster 92**	**Enrichment Score: 1.03**		**Count**		**P_Value**	**Benjamini**
	GOTERM_BP_FAT	lymphocyte proliferation		8	2.40E-02	3.50E-01
	GOTERM_BP_FAT	mononuclear cell proliferation		8	3.00E-02	4.00E-01
	GOTERM_BP_FAT	leukocyte proliferation		8	3.00E-02	4.00E-01
	GOTERM_BP_FAT	B cell proliferation		4	5.60E-02	5.40E-01
	GOTERM_BP_FAT	T cell proliferation		5	1.10E-01	7.30E-01
	GOTERM_BP_FAT	regulation of DNA recombination		3	5.40E-01	9.90E-01
	GOTERM_BP_FAT	regulation of DNA metabolic process		6	9.00E-01	1.00E+00

ILMN_1685886	MAPK4	-0.47186298
ILMN_3278603	LOC644189	-0.471904248
ILMN_1801240	OR5D14	-0.471916863
ILMN_1667018	ACE2	-0.472008069
ILMN_1814333	SERPINI1	-0.472026311
ILMN_1795304	CRLF2	-0.472077862
ILMN_1753745	HDDC2	-0.472131
ILMN_1775742	RNF128	-0.472167675
ILMN_1718932	MTRR	-0.472272619
ILMN_1694075	GADD45A	-0.472306322
ILMN_2181064	GBA3	-0.472319443
ILMN_2142117	LYPLAL1	-0.472380253
ILMN_3305942	LOC729970	-0.472545771
ILMN_1717935	SPTA1	-0.472733677
ILMN_1772369	PDHA1	-0.47281696
ILMN_2141118	C15orf59	-0.472931317
ILMN_1735192	COLEC11	-0.47296937
ILMN_1724658	BNIP3	-0.473087529
ILMN_1674104	S100A7L2	-0.473171083
ILMN_2178116	MMP20	-0.47324118
ILMN_1750262	BC132949	-0.473259535
ILMN_1775962	MCOLN3	-0.473339078
ILMN_2223922	AGTPBP1	-0.473395369
ILMN_1727606	C12orf59	-0.473496797
ILMN_1905546	AK055145	-0.473681673
ILMN_3269377	FLJ00278	-0.473695962
ILMN_1673605	PRSS57	-0.473703467
ILMN_2097052	CDY2B	-0.473706518
ILMN_2316918	PANK1	-0.473831202
ILMN_1674135	RALGPS1	-0.473943886
ILMN_1710000	PEX11G	-0.474000149
ILMN_1656145	GOT1	-0.474033215
ILMN_2367428	FAM96A	-0.474267352
ILMN_1687824	SEPHS2	-0.474321877
ILMN_1910334	FLJ36644	-0.474349212
ILMN_1744832	LOC399715	-0.474378244
ILMN_1763878	CDADC1	-0.474449127
ILMN_1794951	SLC39A5	-0.474554027
ILMN_2402463	SHMT1	-0.474597784
ILMN_2179018	NDUFAB1	-0.474927016
ILMN_2183216	TDP2	-0.474952153
ILMN_1724893	KCTD4	-0.475222561
ILMN_2253805	GFRA1	-0.475392081
ILMN_1712751	HADHA	-0.475397895
ILMN_2053679	ACADM	-0.475436395
ILMN_1726752	APTX	-0.475563281
ILMN_1871361	AX747287	-0.475587001
ILMN_1677962	GPHN	-0.475862778
ILMN_1812073	ATP6V1B1	-0.476129953
ILMN_1737561	N4BP2L2	-0.476132557
ILMN_1803774	STK16	-0.476246623
ILMN_1778617	TAF9	-0.476257559
ILMN_1755352	TUB	-0.476976412
ILMN_1813800	NUFIP1	-0.4772747
ILMN_2058512	PSMA2	-0.477321034
ILMN_1755897	UGT2B7	-0.477355872
ILMN_1800935	ARV1	-0.477361633
ILMN_2343563	ANAPC11	-0.477465636
ILMN_1801378	COQ3	-0.477848903
ILMN_1903159	PPM1A	-0.477930726
ILMN_1785218	MTCH2	-0.477934431
ILMN_3266764	LOC728448	-0.478126722
ILMN_3262976	FLJ46446	-0.478495543
ILMN_1729965	CXorf66	-0.478520472
ILMN_1916599	LOC100133985	-0.47853459
ILMN_1809077	RFPL4B	-0.478579739
ILMN_2375386	RNPS1	-0.478680556
ILMN_2191634	RPL37	-0.478785313
ILMN_2231911	AUH	-0.479014955
ILMN_1763695	C9orf24	-0.479129727
ILMN_2130468	ENPP7	-0.479308219
ILMN_1891927	DCBLD1	-0.479448549
ILMN_1765198	HEATR4	-0.479625113
ILMN_1778173	AK3	-0.479643671
ILMN_2354269	FAM164C	-0.480058282
ILMN_1762033	NAPEPLD	-0.480060098
ILMN_1659214	CTXN3	-0.480202569
ILMN_1810604	ELMOD1	-0.48048766
ILMN_3307772	PACSIN2	-0.480672047
ILMN_3307025	ZDHHC4	-0.48067658
ILMN_1761131	ECI2	-0.480689873
ILMN_1657682	GFM1	-0.480853377
ILMN_1745043	LDHC	-0.481232256
ILMN_1709247	PPP2R1B	-0.48172228
ILMN_3302625	DA626616	-0.481857914
ILMN_2276461	MAP2	-0.48214932
ILMN_1783333	C16orf61	-0.482200079
ILMN_1785821	TDP2	-0.482372179
ILMN_1674098	SORBS2	-0.482563353
ILMN_1859334	BI517762	-0.482595372
ILMN_1716999	ZSCAN1	-0.482691557
ILMN_1746945	CSMD1	-0.482892119
ILMN_1811022	HMX1	-0.483262139
ILMN_1732187	TMEM143	-0.484053669
ILMN_1807095	MRPS36	-0.484229864
ILMN_1679647	CHIA	-0.484358067
ILMN_2108493	RHOF	-0.48438826
ILMN_3251497	GSTA1	-0.48458883
ILMN_1805251	MGC26484	-0.48485798
ILMN_2397841	SNCB	-0.484909555
ILMN_1795976	SFXN2	-0.484983866
ILMN_1695731	TUBG1	-0.485010679

ILMN_1706268	PTGDR	0.445743173
ILMN_3236373	MSL2	0.445629891
ILMN_2179083	LOXL4	0.445628691
ILMN_1778360	PYGB	0.445612914
ILMN_1756204	RPS6KA4	0.445574738
ILMN_1704294	CDH3	0.445524382
ILMN_1656291	TSKS	0.445412864
ILMN_1768870	CAPZA2	0.445310708
ILMN_1755415	SAMD3	0.445110312
ILMN_1746408	MIDN	0.445104553
ILMN_2173004	RAB8B	0.444991827
ILMN_2278636	CUX1	0.444756064
ILMN_2345353	LST1	0.444655906
ILMN_1763260	HIF1A	0.444563587
ILMN_1782977	UBA52	0.44439838
ILMN_2313158	MBNL1	0.444269661
ILMN_1760360	LTN1	0.444234099
ILMN_2098616	C5orf39	0.444213151
ILMN_1767320	MAPK1	0.44417774
ILMN_1863308	CV575492	0.443827134
ILMN_1652412	PHKB	0.443748828
ILMN_3289508	CR604709	0.443649544
ILMN_1686097	TOP2A	0.443526833
ILMN_1733511	GOLGA3	0.443261372
ILMN_2386008	MPZL1	0.443253764
ILMN_1694913	LMO3	0.442734263
ILMN_1778064	FICD	0.442714305
ILMN_2232177	ACTN1	0.442623692
ILMN_1811851	PRRX1	0.442561044
ILMN_1740298	SIGLEC5	0.442313042
ILMN_1778144	SLC48A1	0.442252615
ILMN_2383964	CDCA4	0.4420507
ILMN_2402341	MAPK3	0.442012153
ILMN_1755173	PLEKHA4	0.441991209
ILMN_2342579	IL7R	0.441898579
ILMN_1724059	GAS2L1	0.441891191
ILMN_2077758	CTDSPL2	0.441825246
ILMN_1711792	GPBP1	0.441538966
ILMN_1829845	NA	0.441363733
ILMN_1715508	NNMT	0.441034435
ILMN_1759154	PABPN1	0.44088579
ILMN_2114720	SLPI	0.440881117
ILMN_1700081	FST	0.440838243
ILMN_1728677	CREB5	0.440556315
ILMN_1847308	FAM168A	0.440511603
ILMN_2066849	FAM26F	0.440508234
ILMN_1697024	STYX	0.440441227
ILMN_3235514	GPR183	0.44039574
ILMN_1676213	SRPX2	0.440255745
ILMN_1697370	CST11	0.440232176
ILMN_1652313	RNF215	0.440217218
ILMN_2106331	H3F3A	0.440056216
ILMN_1656670	HLA-G	0.440031333
ILMN_2058782	IFI27	0.43993911
ILMN_3241123	FLJ00326	0.439881822
ILMN_1681160	CCT6P1	0.439874046
ILMN_2151277	LPGAT1	0.439852291
ILMN_1785005	NCF4	0.439789606
ILMN_1683891	TFAP2C	0.439469024
ILMN_2301193	ATP8B2	0.439422945
ILMN_1783771	UBE2Z	0.439303883
ILMN_1766916	RPAP3	0.439070101
ILMN_1767556	C10orf10	0.438985167
ILMN_1655137	ZCCHC11	0.438910311
ILMN_1670134	FADS1	0.438907852
ILMN_1730670	FSTL3	0.438850911
ILMN_1705892	SH2D1A	0.438714921
ILMN_2203271	FPR3	0.438566056
ILMN_2413779	SEZ6L2	0.438322508
ILMN_1723978	LGALS1	0.438298858
ILMN_3252556	CARD16	0.438207912
ILMN_2364174	CSNK1A1	0.438207647
ILMN_3289171	RPS27	0.437948746
ILMN_1739770	MED7	0.437937352
ILMN_1713182	C3	0.43781048
ILMN_1731714	CREB5	0.437618078
ILMN_1776464	PARP4	0.437488843
ILMN_1772074	C19orf51	0.437417167
ILMN_1811702	GRN	0.437406644
ILMN_1661337	SRM	0.437380935
ILMN_1704196	DSG2	0.437234182
ILMN_1758895	CTSK	0.437080467
ILMN_1683597	RPL23A	0.437047873
ILMN_1745522	PF4V1	0.436897394
ILMN_2219246	ITPRIPL2	0.436817642
ILMN_1708858	CSNK1E	0.436752795
ILMN_1711899	ANXA2	0.436657658
ILMN_2376108	PSMB9	0.436599725
ILMN_1771385	GBP4	0.436191759
ILMN_1656501	DUSP5	0.435948531
ILMN_1724407	TACC3	0.435703097
ILMN_2230892	IL10RB	0.435688228
ILMN_1668039	GYPC	0.435618056
ILMN_1790951	C19orf50	0.435581155
ILMN_1730529	CAB39L	0.435556408
ILMN_2325763	VCAM1	0.435519505
ILMN_2383611	PTPRE	0.435468843
ILMN_2331525	PRKD2	0.435250616
ILMN_1761159	ESYT1	0.435216858
ILMN_2410986	STAT3	0.434897608
ILMN_1690105	STAT1	0.434806097
ILMN_1715500	SS18L1	0.434759963

ILMN_2413816	GRB14	-0.485224817
ILMN_1728746	GAB2	-0.48523319
ILMN_2319952	VDR	-0.48536093
ILMN_3248460	SNORD79	-0.485510061
ILMN_1735996	NOX4	-0.485684372
ILMN_1676383	ENPP6	-0.485736049
ILMN_1698487	SDHD	-0.48582935
ILMN_1750880	LOC196394	-0.485908044
ILMN_1680590	LOC644662	-0.486197245
ILMN_1805024	ERBB2IP	-0.486467637
ILMN_1662438	SOD1	-0.486520722
ILMN_1786382	LOC389834	-0.486581677
ILMN_1653793	PDPK1	-0.486733178
ILMN_1745991	GNRH1	-0.4868385
ILMN_1763975	NCRNA00083	-0.487142637
ILMN_1671051	CD276	-0.487210861
ILMN_2069196	CAPZA3	-0.487285448
ILMN_1679797	ADARB1	-0.487561666
ILMN_1685854	C5orf53	-0.487599639
ILMN_1726198	CTSL3	-0.4876215
ILMN_1774742	MTTP	-0.487946196
ILMN_1694653	CNDP1	-0.488016894
ILMN_2064898	CCDC56	-0.488160238
ILMN_1668498	CWF19L2	-0.488169807
ILMN_1754241	C16orf73	-0.4882889
ILMN_1761946	PROM2	-0.488445286
ILMN_2103774	PIP5KL1	-0.488456324
ILMN_2128931	FAT2	-0.488482639
ILMN_1735064	PPP1R3E	-0.488588783
ILMN_1667791	PPFIA4	-0.488939578
ILMN_1757693	C17orf51	-0.48899806
ILMN_1655377	MRPS22	-0.489004562
ILMN_1690979	SLC17A3	-0.489168167
ILMN_2381397	HSPD1	-0.489194309
ILMN_1789096	OSTalpha	-0.489209868
ILMN_2079285	ATP5L	-0.489303322
ILMN_2383229	A1CF	-0.489400688
ILMN_1814526	ADD3	-0.489445534
ILMN_1765232	RNLS	-0.489566723
ILMN_1793578	ZFP37	-0.48993458
ILMN_1670547	GRM1	-0.490038194
ILMN_3294965	RRAS2	-0.490159822
ILMN_2355033	KIAA1147	-0.490174939
ILMN_1677919	GMPR2	-0.490202698
ILMN_1751943	MME	-0.490354886
ILMN_1723414	HACL1	-0.490527151
ILMN_1793832	GRB14	-0.490726285
ILMN_1658940	DDX25	-0.49076593
ILMN_2172174	PNP	-0.490777104
ILMN_1745345	MT4	-0.490799854
ILMN_1707464	MST1	-0.491048327
ILMN_1720838	DECR1	-0.491263442
ILMN_1712161	BCOR	-0.491554807
ILMN_1738849	SLC9A2	-0.491600438
ILMN_2131811	CLPS	-0.491694578
ILMN_1829989	LOC220930	-0.491832265
ILMN_1706690	KHK	-0.492025908
ILMN_3236423	RAB3GAP2	-0.492151537
ILMN_1692651	PHB	-0.492655191
ILMN_1707548	RAD18	-0.492708727
ILMN_1705049	TMEM67	-0.492782656
ILMN_1782070	NPL	-0.492968704
ILMN_1658318	C11orf93	-0.493067422
ILMN_2117736	MGAT4C	-0.493155881
ILMN_1742444	X63359	-0.493321817
ILMN_1806330	C17orf78	-0.493325239
ILMN_1790036	C20orf202	-0.493329884
ILMN_2176450	CA3	-0.493330774
ILMN_1654402	IL18BP	-0.493343558
ILMN_1660635	LACTB2	-0.493890512
ILMN_1812312	NDUFS4	-0.493934591
ILMN_1728798	TEKT4P2	-0.494220077
ILMN_1803676	ENOSF1	-0.49442633
ILMN_1788267	PPP1R14D	-0.494441997
ILMN_1785284	ALDH6A1	-0.494556966
ILMN_1674152	NFKBIB	-0.494661163
ILMN_3234089	N4BP2L2	-0.494674317
ILMN_1677043	AKR7A2	-0.494805449
ILMN_2344079	ZGPAT	-0.494930929
ILMN_2268068	MAPKAP1	-0.49530015
ILMN_2052331	MYOC	-0.496146517
ILMN_2058347	NT5DC1	-0.496224396
ILMN_1726743	MRPS30	-0.496401913
ILMN_1767474	HAO2	-0.496416487
ILMN_1717757	CALML4	-0.496676861
ILMN_1882485	CF552129	-0.496938344
ILMN_1699411	GNG4	-0.496973117
ILMN_1673793	SUN3	-0.497207621
ILMN_1686573	DEFB1	-0.497495864
ILMN_2292646	GAD1	-0.497584363
ILMN_1810200	ARPC2	-0.497791751
ILMN_3236472	WEE2	-0.4979425
ILMN_2194467	SGCB	-0.498057682
ILMN_1717853	DMGDH	-0.498148378
ILMN_1686906	TP53INP2	-0.498317383
ILMN_1783443	ALOX15	-0.498497757
ILMN_1688543	APOA2	-0.498559545
ILMN_1809931	NDRG1	-0.498566011
ILMN_2393461	DDO	-0.498751158
ILMN_3308168	FAM41AY1	-0.49879344
ILMN_1813270	ELF5	-0.499135583
ILMN_2120210	RCAN2	-0.499184588

ILMN_1772387	TLR2	0.434622301
ILMN_2099528	BTLA	0.434543999
ILMN_1721580	TBX15	0.434356048
ILMN_1819608	ARRB1	0.434208762
ILMN_2130525	TSPAN13	0.434170357
ILMN_2377669	CD247	0.434110187
ILMN_1737041	HABP2	0.433447469
ILMN_1776005	OSTC	0.433355297
ILMN_1719039	UBE2G1	0.433217588
ILMN_1712419	DCDC2	0.433121328
ILMN_1759954	PTMA	0.43311473
ILMN_1672004	TOB1	0.433087403
ILMN_1713249	PHF19	0.433038592
ILMN_1785988	CSNK1A1	0.432985393
ILMN_1772612	ANGPTL2	0.432925711
ILMN_1652525	FAM125B	0.432860995
ILMN_1666208	MIS18BP1	0.432634486
ILMN_1803977	GALNT1	0.432550814
ILMN_1662768	NHEDC2	0.432548771
ILMN_1737599	TRIM5	0.432435355
ILMN_1777344	RPS13	0.432353639
ILMN_3246560	EMB	0.432337279
ILMN_1666715	WHSC1L1	0.432327769
ILMN_3236381	MXRA8	0.432209453
ILMN_1656386	SEC24D	0.431914267
ILMN_2241953	PILRA	0.43189174
ILMN_1712887	SLC10A3	0.431889181
ILMN_1779677	ZCCHC6	0.431738839
ILMN_1658955	EIF4E	0.431614251
ILMN_1690806	CR613620	0.431600478
ILMN_1789599	C1orf151	0.431558138
ILMN_3272603	FAM60A	0.431376363
ILMN_1768505	IL13RA1	0.431185885
ILMN_1767662	LASS6	0.431175425
ILMN_2333367	FKBP1A	0.431137489
ILMN_2272074	TROVE2	0.431035481
ILMN_1669692	IKZF3	0.43083824
ILMN_2064606	TBC1D2B	0.430761934
ILMN_1786024	POLR3H	0.430744053
ILMN_1652161	PNKD	0.430647752
ILMN_1744113	SCNM1	0.430643576
ILMN_3270833	NA	0.43058365
ILMN_1745807	TMEM62	0.430379823
ILMN_1731287	ARFGAP3	0.430171581
ILMN_1657991	LOC391722	0.430143759
ILMN_1897254	BX383912	0.430106436
ILMN_1680772	TCRGV	0.430076028
ILMN_2386179	ZMYND8	0.429998814
ILMN_1655283	INPP4A	0.429952182
ILMN_1655572	C6orf59	0.429948667
ILMN_1718629	NRIP1	0.429800467
ILMN_3237946	PXDN	0.429797978
ILMN_1749634	PLRG1	0.429751827
ILMN_1782167	RPL32	0.429539191
ILMN_1910908	BG205162	0.429518547
ILMN_1714499	TLK2	0.429477679
ILMN_1722872	MYH9	0.429400549
ILMN_2053345	KCNT2	0.429331195
ILMN_1701114	GBP1	0.429207189
ILMN_1761442	WDR24	0.429168867
ILMN_1810037	RUSC2	0.429130555
ILMN_1651950	TPST1	0.429022445
ILMN_2090105	TAGLN2	0.429008572
ILMN_2407619	CDC25C	0.428959816
ILMN_2350574	MYADM	0.428918454
ILMN_2109994	MIA-RAB4B	0.428629613
ILMN_3306628	C18orf25	0.428603322
ILMN_1785831	ZDHHC13	0.428577225
ILMN_1727135	FIBCD1	0.428505665
ILMN_3247223	TPBG	0.428466645
ILMN_1745130	RBFOX2	0.428420918
ILMN_1804444	PDIA2	0.428411127
ILMN_2213136	LEF1	0.428344554
ILMN_3302905	RPL23A	0.428314223
ILMN_3307693	WFDC2	0.428222924
ILMN_1669210	RPL4	0.428202285
ILMN_2363621	RBBP8	0.427981058
ILMN_1719695	NFKBIZ	0.427924066
ILMN_2053178	ACTG1	0.427897591
ILMN_2063114	TAF1D	0.427814401
ILMN_1808059	BCAS4	0.427514046
ILMN_1780108	PTPN22	0.427466872
ILMN_1678669	RRM2	0.427464083
ILMN_2092693	LSM12	0.427399574
ILMN_1652512	C2CD2	0.427371123
ILMN_2402392	COL8A1	0.42730106
ILMN_3251100	MEF2A	0.427118071
ILMN_1685122	COL9A2	0.426987988
ILMN_1686360	ZNF124	0.426935689
ILMN_1793474	INSIG1	0.426864599
ILMN_1705763	SCT	0.426754931
ILMN_1740197	ZNF234	0.426634136
ILMN_1675479	ZNF646	0.426615173
ILMN_1744929	PTGER1	0.426555259
ILMN_2324421	TXNRD1	0.426542949
ILMN_3230521	FLJ40330	0.426500061
ILMN_3264073	RPS27	0.426413875
ILMN_1703263	SP140	0.426326277
ILMN_1702691	TNFAIP3	0.426321744
ILMN_1652123	HMGN1	0.426303968
ILMN_1748438	POLR2G	0.426270248
ILMN_2318568	HCFC1R1	0.426262679

ILMN_1662738	ACSM3	-0.499208254
ILMN_3185020	AI926420	-0.499249207
ILMN_2370910	RAD51L3	-0.499280092
ILMN_2393456	DDO	-0.49933447
ILMN_1745887	FBXO21	-0.499625694
ILMN_1744534	LYRM5	-0.499749965
ILMN_1738058	CPNE6	-0.499771275
ILMN_1806473	BEX5	-0.49993877
ILMN_1685974	SLC25A33	-0.500226991
ILMN_1754423	C3orf23	-0.500300243
ILMN_2357730	CDK20	-0.50036511
ILMN_1818856	BE549596	-0.500533366
ILMN_1653358	FUT3	-0.500859047
ILMN_3235803	AQP7P3	-0.50088916
ILMN_1758750	EARS2	-0.500960635
ILMN_1748323	CXCL14	-0.500974665
ILMN_1794875	AGPAT9	-0.500989569
ILMN_3230880	BEND7	-0.501360509
ILMN_1741695	COL12A1	-0.5014294
ILMN_2331232	TNFRSF6B	-0.501469367
ILMN_3306482	GCSH	-0.501487404
ILMN_1671791	PCK2	-0.502102533
ILMN_2296510	SLC5A12	-0.50235117
ILMN_1655613	GSTA2	-0.50235825
ILMN_2142353	GRTP1	-0.502423665
ILMN_1672479	TRIM55	-0.502816781
ILMN_3187758	SELK	-0.503248184
ILMN_1795300	ARHGEF37	-0.503315741
ILMN_1739274	PDHB	-0.50380106
ILMN_1724504	SETD3	-0.503992383
ILMN_1769433	IQGAP2	-0.504012224
ILMN_1860963	BM715829	-0.504042576
ILMN_1819515	AF009300	-0.5041679
ILMN_2226628	ZNF184	-0.504221439
ILMN_1733366	MAST1	-0.504424597
ILMN_1875967	BG222050	-0.504471984
ILMN_1701454	OR2L13	-0.504559082
ILMN_1698680	ARL17B	-0.504668948
ILMN_3247438	C19orf69	-0.504763896
ILMN_1745817	NELL1	-0.505003682
ILMN_1707585	SLC46A2	-0.505712375
ILMN_1803743	AX747098	-0.506197539
ILMN_2406634	DNMT3L	-0.506243129
ILMN_3243788	TMEM146	-0.506261249
ILMN_3180456	NA	-0.506333915
ILMN_3225886	NOX4	-0.50637689
ILMN_1658800	BRPF3	-0.506403267
ILMN_1811880	SLC34A3	-0.506562339
ILMN_1672605	C7orf41	-0.506646681
ILMN_1651259	UGP2	-0.506794525
ILMN_1789653	PBLD	-0.507117024
ILMN_1676625	SS18L1	-0.507425975
ILMN_1712943	CCK	-0.507561701
ILMN_1778301	C6orf132	-0.507835251
ILMN_2148497	C20orf107	-0.507935691
ILMN_1758272	MYPN	-0.507953216
ILMN_3298870	FAM182B	-0.508221693
ILMN_1710903	MAPT	-0.508311776
ILMN_1807448	FAM8A1	-0.508574179
ILMN_1762531	FGF9	-0.50868513
ILMN_1693270	SUSD2	-0.509007976
ILMN_2244699	MAPK7	-0.509085121
ILMN_1678170	MME	-0.509121703
ILMN_1669123	AK125437	-0.509206616
ILMN_1708672	ACAT2	-0.509242898
ILMN_1803745	SUOX	-0.509517874
ILMN_1761804	ALDH9A1	-0.509758034
ILMN_2372698	RGN	-0.509770611
ILMN_3248032	NCCRP1	-0.510109252
ILMN_1739582	HOXA10	-0.510313253
ILMN_2375879	VEGFA	-0.510415338
ILMN_1661994	ESRRG	-0.510487705
ILMN_1776601	OR4F5	-0.510591919
ILMN_1660963	CCIN	-0.510856919
ILMN_1669617	GRB10	-0.511137899
ILMN_2166865	ENY2	-0.511151344
ILMN_1792671	C12orf29	-0.511357017
ILMN_3273383	AY461701	-0.511387038
ILMN_1741431	AOC2	-0.511745421
ILMN_1662158	RPL26L1	-0.511950448
ILMN_1680390	GCNT2	-0.512892865
ILMN_1748650	MRPL45	-0.513306447
ILMN_1682763	ALB	-0.513400458
ILMN_1699684	PBLD	-0.513455501
ILMN_1809259	HRASLS2	-0.513478157
ILMN_1758034	ETFDH	-0.513764006
ILMN_1887508	AI206543	-0.513965491
ILMN_1813938	CHCHD4	-0.514008053
ILMN_1738346	EFHA1	-0.514176572
ILMN_1815203	HMGCS2	-0.514209731
ILMN_2113470	GSTA1	-0.514312674
ILMN_1715437	CASC5	-0.514444059
ILMN_1691432	PRDM4	-0.514517828
ILMN_1780861	METRNL	-0.514898195
ILMN_1778951	C6orf203	-0.515038647
ILMN_1707592	RGN	-0.515175991
ILMN_3251567	FBXW2	-0.515813789
ILMN_1777845	NOTCH2	-0.516231103
ILMN_1658917	SLC1A1	-0.516353033
ILMN_1815949	SLC24A4	-0.516558381
ILMN_1733135	TFAP2A	-0.516837352
ILMN_3272441	PTPRQ	-0.516966688

ILMN_2188451	HIST1H2AH	0.426236258
ILMN_1705594	NAT10	0.426160324
ILMN_1679482	TUBA3C	0.426147868
ILMN_1808874	AK127262	0.426142462
ILMN_1728435	C12orf45	0.425811025
ILMN_2110271	OLFM2	0.425709697
ILMN_1702636	TUBB6	0.425372373
ILMN_3294339	LOC100131289	0.425260739
ILMN_1812559	SLC7A6	0.425207353
ILMN_2393497	ATXN3	0.425072642
ILMN_1766914	MFAP4	0.425072282
ILMN_2225135	GCNT1	0.425036132
ILMN_1651209	SLC35E2	0.424909503
ILMN_1655876	TMEM159	0.424879729
ILMN_1708841	GOLPH3	0.424851811
ILMN_1735594	CDC42SE2	0.424633756
ILMN_2227195	CCDC65	0.424598883
ILMN_1693986	WHAMML2	0.424432196
ILMN_1760374	CD8A	0.424407406
ILMN_1690139	KIAA0748	0.424156178
ILMN_1715203	DNAH7	0.42402283
ILMN_1720819	SPCS2	0.423789899
ILMN_2233783	CD38	0.423743979
ILMN_1748529	RLN2	0.423662949
ILMN_1688279	PVRIG	0.423584213
ILMN_2049536	TRPV2	0.423572112
ILMN_2278850	RAB24	0.423526811
ILMN_1738523	MYD88	0.423488776
ILMN_1774287	CFB	0.423446323
ILMN_1698766	PYCARD	0.423321112
ILMN_2090607	IRF2	0.423291527
ILMN_3199780	CAP1	0.423239935
ILMN_2206141	BAGE	0.423183964
ILMN_2351298	WIPF1	0.423121778
ILMN_1805519	DQ893812	0.423097713
ILMN_1804652	PLEKHH3	0.423061393
ILMN_1773066	CDKN2AIP	0.423047728
ILMN_1789839	GTF3C1	0.423022548
ILMN_2070072	RPS7	0.422819015
ILMN_2163187	FLJ35776	0.42280689
ILMN_1660900	SNORA7B	0.422800664
ILMN_1824052	AA909514	0.422769328
ILMN_1783329	ZACN	0.422718765
ILMN_1770281	ERC1	0.422331227
ILMN_1654031	TPTE2	0.422210266
ILMN_2414027	CMTM1	0.422055341
ILMN_1716983	LILRA2	0.422022128
ILMN_1672124	FAM198B	0.421870139
ILMN_3288018	LOC645323	0.421855067
ILMN_1681591	PTPN1	0.421554524
ILMN_1752923	IFNAR1	0.421493221
ILMN_1661359	CXorf65	0.421419793
ILMN_1654735	SLCO3A1	0.421412848
ILMN_2363586	SDCBP	0.421295159
ILMN_1772486	ELF2	0.421264542
ILMN_1766955	VCAM1	0.421260966
ILMN_1651767	MKL1	0.421258711
ILMN_1773964	H1FX	0.421193858
ILMN_1670378	FAM115A	0.421140803
ILMN_1718552	CXCL13	0.421030719
ILMN_1740426	RASD1	0.420924785
ILMN_2379734	CTBP1	0.420879215
ILMN_2396287	RFX2	0.420713436
ILMN_1656073	MYL12A	0.420643232
ILMN_1736015	PHF17	0.420607716
ILMN_2410864	RAB28	0.420492511
ILMN_1683082	RPUSD1	0.420466857
ILMN_1660832	RPL18A	0.420411273
ILMN_2233366	ASAP1	0.420292278
ILMN_1743347	AKT2	0.42028485
ILMN_1722923	SRGAP3	0.420100958
ILMN_1692539	SH3BP1	0.420043968
ILMN_2155719	NBPF1	0.419816012
ILMN_1872122	LCOR	0.419631791
ILMN_1666078	HLA-H	0.419592123
ILMN_1683036	BRWD1-IT2	0.419449967
ILMN_1664063	FAM129C	0.419346988
ILMN_2343618	SAMD3	0.419337104
ILMN_1755024	IKBKE	0.419244023
ILMN_1699440	ZBTB47	0.419170676
ILMN_1698067	NBPF1	0.419120689
ILMN_1763000	ADAP2	0.419113398
ILMN_1771593	RRM1	0.419007709
ILMN_2153787	ARRDC5	0.418987921
ILMN_1726114	SLC45A3	0.418960459
ILMN_1718558	PARP12	0.418882561
ILMN_2128293	APIP	0.41864951
ILMN_3251733	C19orf43	0.418647711
ILMN_2085862	SLC15A3	0.418638297
ILMN_1760563	PRRC2A	0.418610647
ILMN_1770290	CNN2	0.418497857
ILMN_1744887	RLN2	0.418274316
ILMN_3309995	MIR383	0.418267267
ILMN_1720344	NIPA2	0.418175382
ILMN_3221499	ZNF735	0.418155045
ILMN_1762260	C3	0.418085603
ILMN_3275345	RPS27	0.41807729
ILMN_3270813	NA	0.418056
ILMN_2071809	MGP	0.418040872
ILMN_1692731	TTYH3	0.417974627
ILMN_1690561	GZMM	0.417806132
ILMN_1659029	FAM116B	0.41773041

ILMN_1736441	PDXP	-0.517129473
ILMN_1662795	CA2	-0.517877699
ILMN_1827211	U43604	-0.517895776
ILMN_1738854	CACHD1	-0.51802405
ILMN_1897333	BX098521	-0.518589793
ILMN_1826853	NA	-0.519285599
ILMN_1662417	LRPPRC	-0.519916292
ILMN_1770725	AIF1L	-0.520044771
ILMN_1831214	AW592010	-0.520427228
ILMN_3237589	PHAX	-0.520627824
ILMN_1730229	CGNL1	-0.520771213
ILMN_1686804	CDK20	-0.521166852
ILMN_1740233	UGT1A1	-0.521174601
ILMN_1750800	ACO1	-0.521249998
ILMN_1731941	APOM	-0.521444993
ILMN_1763558	RGN	-0.521548568
ILMN_2163796	RABGGTB	-0.521763335
ILMN_2369104	TRAPPC6B	-0.521775481
ILMN_1797310	ATP6V1D	-0.521875795
ILMN_1804662	NRG4	-0.52201763
ILMN_3307836	SLC5A12	-0.522149637
ILMN_1741204	KLHDC2	-0.522396099
ILMN_1661491	SH3GL2	-0.522598779
ILMN_2297887	SEZ6L2	-0.522852419
ILMN_1711245	SCG3	-0.52325608
ILMN_1813350	HSD11B2	-0.5232935
ILMN_1784834	OR52E6	-0.523324668
ILMN_1704166	LNP1	-0.523430022
ILMN_1795778	P4HA2	-0.523543519
ILMN_3261111	IP6K3	-0.523734841
ILMN_2371825	AGL	-0.523913857
ILMN_1803882	VEGFA	-0.523969402
ILMN_1772057	COL28A1	-0.524026093
ILMN_1782730	ZNF473	-0.524066579
ILMN_1790603	ATPAF1	-0.524461662
ILMN_1652722	C14orf2	-0.524581174
ILMN_1805147	ACSM5	-0.524782966
ILMN_2128967	C11orf1	-0.524827605
ILMN_1696450	KLK1	-0.52493008
ILMN_1853824	MGAT3	-0.52519718
ILMN_1669114	WNK4	-0.525564689
ILMN_1814296	TRPM6	-0.525686444
ILMN_1712786	AHCYL2	-0.525743185
ILMN_1773073	PHYH	-0.525843939
ILMN_2224230	TTTY21	-0.52598447
ILMN_3237085	NTF4	-0.526155155
ILMN_1683905	C19orf21	-0.526486266
ILMN_1699320	LOC339524	-0.526605798
ILMN_1806787	CSDC2	-0.526617015
ILMN_2183687	LIME1	-0.526839511
ILMN_1691572	TST	-0.526977744
ILMN_2143220	ANGPTL3	-0.527059729
ILMN_1745688	HPD	-0.527122213
ILMN_1722532	KDM3A	-0.527263705
ILMN_1882652	BG697942	-0.527716317
ILMN_1699937	HPS4	-0.52790616
ILMN_1749405	KIAA1191	-0.528195899
ILMN_1664071	TNNT2	-0.529047238
ILMN_1703174	FREM2	-0.529269078
ILMN_3305339	UBA5	-0.529389579
ILMN_1782937	GSTA1	-0.529390084
ILMN_1797154	AZGP1	-0.52939045
ILMN_1778734	FAN1	-0.529822636
ILMN_1788122	GSTA5	-0.529974168
ILMN_1765103	CST8	-0.530674355
ILMN_1764494	ATP5A1	-0.530768756
ILMN_2043306	EPB41L5	-0.530984116
ILMN_1758941	REEP5	-0.53107345
ILMN_2311020	DNAJC12	-0.531187763
ILMN_2052208	GADD45A	-0.531297129
ILMN_1800659	PGM1	-0.531468513
ILMN_1807101	F11	-0.531748271
ILMN_2096985	ALDH6A1	-0.532371901
ILMN_1786388	RNF113A	-0.532502805
ILMN_1737152	DAO	-0.532909751
ILMN_2290776	AGBL5	-0.533283314
ILMN_1781242	SOST	-0.533354231
ILMN_1756162	EXOSC8	-0.533946485
ILMN_1727813	BRP44	-0.534297944
ILMN_2373515	HSP90AA1	-0.535175503
ILMN_1759973	NDUFA5	-0.535502407
ILMN_1778844	FAM5B	-0.535940023
ILMN_1808238	RBPMS2	-0.536294466
ILMN_1714384	PCCA	-0.53646934
ILMN_2168228	ATOH7	-0.536831235
ILMN_1764266	CKMT2	-0.537083454
ILMN_1718853	UQCRC2	-0.537136305
ILMN_1740265	ACOT7	-0.537144734
ILMN_2175020	RPL32P3	-0.537381679
ILMN_1694359	UPF0639	-0.53741043
ILMN_2057220	HRSP12	-0.537452901
ILMN_1736954	ZBTB7B	-0.537575523
ILMN_1695193	ANGPTL3	-0.537664271
ILMN_1690783	TREML1	-0.537693568
ILMN_2216815	MAP7	-0.537730832
ILMN_3236866	ULK4	-0.537747664
ILMN_1811278	RNF186	-0.538336189
ILMN_2289576	VWA1	-0.538371939
ILMN_1763461	ALDH7A1	-0.538402776
ILMN_2380561	PKLR	-0.539004819
ILMN_1726667	LOC728758	-0.539050257
ILMN_2199676	CEP152	-0.539166845

ILMN_2186061	PFKFB3	0.417660192
ILMN_1761833	SLC40A1	0.417650029
ILMN_1693650	FES	0.417542393
ILMN_3209220	NA	0.417501011
ILMN_1862018	ATXN7L3	0.417422219
ILMN_2325194	PHF6	0.417394928
ILMN_1717809	RNF24	0.417357668
ILMN_1656732	LOC440300	0.4173066
ILMN_1670101	CLEC2D	0.417290281
ILMN_1749448	GLMN	0.417254655
ILMN_1741404	MSC	0.417164353
ILMN_1674640	CXCR6	0.417146933
ILMN_1703908	NA	0.41711947
ILMN_1808395	ACAP1	0.417114521
ILMN_1749662	GPX1	0.417111988
ILMN_2368318	FGR	0.417014523
ILMN_1807535	YWHAE	0.416927101
ILMN_2367384	EPHB2	0.416884406
ILMN_2149226	CAV1	0.416453675
ILMN_1713901	KDELR3	0.416388066
ILMN_1676091	HNRNPA1	0.41622215
ILMN_2096784	TFAP2C	0.416209958
ILMN_1726578	ZNF93	0.41604881
ILMN_1809477	CARHSP1	0.415892852
ILMN_1710221	GPR35	0.415832341
ILMN_2089073	ATP9A	0.415818712
ILMN_1691717	RHBDF2	0.415769801
ILMN_1668863	LYPD1	0.415618137
ILMN_1681239	MYO1F	0.415571114
ILMN_1726289	C12orf35	0.415514639
ILMN_1710216	AVEN	0.415414154
ILMN_2402272	TCEAL8	0.415399772
ILMN_1720181	CLSTN1	0.415206827
ILMN_1752802	CLPTM1L	0.415168609
ILMN_1806721	MS4A2	0.415164431
ILMN_1661309	XKR4	0.415157201
ILMN_3238481	SNORD60	0.415114925
ILMN_1720476	PHF2	0.415015791
ILMN_2150161	ZRANB3	0.414935612
ILMN_1800220	KCTD3	0.414858947
ILMN_3248928	UBN2	0.414582768
ILMN_1680145	SART1	0.414490626
ILMN_1731928	LY9	0.414426397
ILMN_1803561	PLA2G4A	0.414316426
ILMN_1767665	GPX8	0.414133019
ILMN_1801307	TNFSF10	0.414066282
ILMN_1664466	KLHL9	0.413908363
ILMN_1736806	PAG1	0.413867796
ILMN_1887174	KIAA0146	0.413809879
ILMN_1694223	DGCR8	0.413708447
ILMN_1675156	CDC42	0.413692461
ILMN_3241446	ODF3B	0.413633306
ILMN_2158902	PXDNL	0.413515069
ILMN_1763640	NCKAP5L	0.413345086
ILMN_1703433	PLSCR3	0.413294512
ILMN_1749006	RCSD1	0.413284499
ILMN_1751904	EDNRB	0.413251946
ILMN_3242753	NBPF1	0.413087545
ILMN_1769671	RYK	0.413087165
ILMN_2112638	SVEP1	0.41298778
ILMN_1765829	NUFIP2	0.412742373
ILMN_2116127	NPEPPS	0.412697844
ILMN_1731180	EXOC2	0.412675758
ILMN_1784141	JAKMIP1	0.412598732
ILMN_2252309	DPP7	0.412521148
ILMN_1708959	MFN1	0.412455127
ILMN_2049021	PTTG3	0.412410943
ILMN_1733581	C16orf35	0.412370078
ILMN_1758983	OTOP1	0.412196852
ILMN_1666819	PHLDB1	0.412185351
ILMN_1695423	CD9	0.412144435
ILMN_1663925	OR2L5	0.411991812
ILMN_1856861	INO80D	0.411831354
ILMN_3238560	IFI27L2	0.411724428
ILMN_1671295	MST151	0.411640204
ILMN_3236169	MACC1	0.411585575
ILMN_1756360	RPL35A	0.411556316
ILMN_1802523	HS3ST5	0.411544817
ILMN_2385178	MIB2	0.411417507
ILMN_1701393	TBX3	0.411322903
ILMN_1694475	GTPBP2	0.411230194
ILMN_1759883	SRP9	0.411170562
ILMN_1757338	PLSCR4	0.411055498
ILMN_1703228	AGFG2	0.410957506
ILMN_3246625	KIAA1045	0.410797888
ILMN_3244607	FAM117B	0.410675233
ILMN_1719986	PIK3IP1	0.410664945
ILMN_1763382	NPPB	0.41017508
ILMN_1726589	CD248	0.410127271
ILMN_2168520	C11orf70	0.410103331
ILMN_1796244	CD2BP2	0.409984858
ILMN_2143795	NCRNA00152	0.409976983
ILMN_1712636	NVL	0.40990025
ILMN_3249866	NA	0.409848964
ILMN_2177413	TBL3	0.409719664
ILMN_2242463	CTSC	0.409622204
ILMN_2303166	MBNL3	0.409593283
ILMN_1702604	KCNA3	0.40957505
ILMN_1742947	SEC14L4	0.409360464
ILMN_1711994	TCIRG1	0.409327091
ILMN_2184184	ANXA1	0.409291635
ILMN_1692896	JMJD4	0.409240971

ILMN_1731181	TEX2	-0.539457065
ILMN_1712530	AKAP1	-0.53985608
ILMN_1687315	RXRA	-0.540682616
ILMN_1772958	MAP1LC3A	-0.541136757
ILMN_3269995	LOC100129042	-0.541942019
ILMN_1809304	PRAMEF12	-0.541993691
ILMN_1876924	WNK1	-0.542461742
ILMN_1761260	COBLL1	-0.542512683
ILMN_2233552	POLD3	-0.542991933
ILMN_3246214	B3GNT1	-0.543174959
ILMN_1703678	DNM1P33	-0.54344193
ILMN_2285375	SORD	-0.543592997
ILMN_1794968	LRRC28	-0.543767953
ILMN_2198239	HGD	-0.543885443
ILMN_1688864	CABP4	-0.543889776
ILMN_2317739	ITSN2	-0.544145111
ILMN_3203059	LOC100131176	-0.544307725
ILMN_3238269	NDUFA6	-0.54476094
ILMN_1663605	RNF123	-0.545641659
ILMN_3297082	LOC729080	-0.545746866
ILMN_1739429	HS6ST2	-0.54579897
ILMN_1794890	SLC38A4	-0.545811231
ILMN_1688455	KIAA0564	-0.546287297
ILMN_1674703	UBA5	-0.546474451
ILMN_2244631	STEAP1	-0.546551755
ILMN_1739659	ZDHHC6	-0.54779174
ILMN_1752952	MMP28	-0.548653537
ILMN_1696318	LOC100130691	-0.548705992
ILMN_1739925	SLC28A1	-0.548965444
ILMN_1676042	NA	-0.549144451
ILMN_1881598	AW451118	-0.549282264
ILMN_2215355	AK126539	-0.549533144
ILMN_1692517	SORD	-0.550630857
ILMN_1781859	UGT2B28	-0.550645189
ILMN_2168747	GSTA2	-0.550689713
ILMN_3305768	GSTA7P	-0.550784054
ILMN_1807339	HRG	-0.551635717
ILMN_1760174	MCCC1	-0.55194636
ILMN_2115421	SLC25A16	-0.552246724
ILMN_2183772	CCDC121	-0.552384116
ILMN_1777031	PKLR	-0.552412016
ILMN_2166457	HPGD	-0.552644609
ILMN_1798485	ATP6V1E1	-0.552733705
ILMN_2336585	CTAG2	-0.553322431
ILMN_2339779	ATP6V1E1	-0.553986787
ILMN_2184049	COX7B	-0.554055405
ILMN_1776724	LYPD6	-0.554080454
ILMN_1891725	BX111675	-0.55418394
ILMN_1782015	FCRLB	-0.554372767
ILMN_1806310	A1CF	-0.55450949
ILMN_2162819	UGT2B11	-0.554905065
ILMN_1754491	IYD	-0.555083178
ILMN_1766896	ZDHHC19	-0.555800091
ILMN_3191541	PTPRQ	-0.556309619
ILMN_1736972	GKAP1	-0.55634514
ILMN_1670385	TUBAL3	-0.556866498
ILMN_3251540	GSTA2	-0.556889644
ILMN_2133125	LRRC43	-0.556913817
ILMN_1718001	PHKB	-0.557140237
ILMN_2363634	ADHFE1	-0.557389477
ILMN_1699011	GTDC1	-0.557551345
ILMN_1800898	ARG2	-0.557582729
ILMN_1720235	ADSSL1	-0.557833379
ILMN_1690733	EGF	-0.558111822
ILMN_1728803	HMP19	-0.558583456
ILMN_2296843	GCDH	-0.558638287
ILMN_3245331	DHRS4L1	-0.558930928
ILMN_1733333	CALML3	-0.559153649
ILMN_1850574	BG943031	-0.562963253
ILMN_2374191	FNTA	-0.563012507
ILMN_1702858	ADHFE1	-0.563546607
ILMN_1806040	TYMS	-0.563573249
ILMN_3305614	ZNF812	-0.564366479
ILMN_1688886	GPC5	-0.565094193
ILMN_2069224	PVALB	-0.565136982
ILMN_2228780	SLC5A11	-0.56539883
ILMN_2211546	HAO1	-0.566257024
ILMN_2352609	OGG1	-0.566755205
ILMN_1758189	OR4F17	-0.567216459
ILMN_1776493	MTUS1	-0.567659808
ILMN_1861652	BQ575680	-0.567675662
ILMN_1712231	SH3YL1	-0.56794066
ILMN_1683133	KLF15	-0.567974457
ILMN_1764607	UGT1A1	-0.568577494
ILMN_3307954	L3MBTL4	-0.568724889
ILMN_1725352	RUVBL1	-0.569359348
ILMN_1808677	BC141517	-0.569539104
ILMN_2262901	RUFY3	-0.569657107
ILMN_1687508	ALDH7A1	-0.57015289
ILMN_1810324	SGCB	-0.570744689
ILMN_1761511	APOH	-0.571018985
ILMN_1702489	TRIM63	-0.571234802
ILMN_1809352	PRL	-0.571379016
ILMN_2371001	ATP6V1G3	-0.571542954
ILMN_2218104	PAH	-0.572114062
ILMN_2380181	MAG	-0.573094942
ILMN_1720998	CA12	-0.574295403
ILMN_1754315	MMAA	-0.574780064
ILMN_1746788	TRIM10	-0.576504793
ILMN_1749081	AUTS2	-0.577415214
ILMN_3226181	NUDT7	-0.578374817
ILMN_1772206	CYP3A4	-0.579003069

ILMN_1804339	CAMK1G	0.409232738
ILMN_1765923	C17orf101	0.409167815
ILMN_2225144	EIF4E3	0.409055855
ILMN_1683441	NCAPD3	0.409030907
ILMN_1739496	PRRX1	0.409006761
ILMN_1692261	EPHB1	0.408946755
ILMN_2134224	ATP13A1	0.408868275
ILMN_1772991	MIR671	0.408853244
ILMN_1808590	GUCY1A3	0.408811581
ILMN_1760683	SRSF9	0.408767555
ILMN_2151817	PFN1	0.408751462
ILMN_2206746	BGN	0.408680759
ILMN_3233388	RELL1	0.408515326
ILMN_1765409	STAM	0.408447493
ILMN_1754746	CCNB3	0.40837607
ILMN_2354191	CD8B	0.408373244
ILMN_3248247	LOC349114	0.408338865
ILMN_2082035	GALNT1	0.408324549
ILMN_1721333	AURKC	0.408291814
ILMN_1680367	C10orf90	0.408250468
ILMN_2402930	H3F3A	0.408070068
ILMN_1801118	SNRNP25	0.408023057
ILMN_3246801	DNAJC30	0.407932599
ILMN_1731048	TLR1	0.407930551
ILMN_1711617	GMFG	0.407807931
ILMN_1803429	CD44	0.407755639
ILMN_1777296	ACTB	0.407726547
ILMN_2291954	SLA	0.407701178
ILMN_2412922	C14orf43	0.407638555
ILMN_1741869	WDR47	0.407482394
ILMN_3247797	abParts	0.407442946
ILMN_1720511	LRRN1	0.407430682
ILMN_1752953	BCL2L12	0.407427893
ILMN_1784294	CPA4	0.407351504
ILMN_2365307	CD276	0.407303643
ILMN_1744239	FEM1B	0.407229805
ILMN_2094952	NUAK2	0.407222553
ILMN_2367753	ATP2B4	0.407218499
ILMN_1741422	FUT8	0.407201682
ILMN_1771308	BAAT	0.407199747
ILMN_1708041	PLEKHF1	0.407175881
ILMN_1751161	COL7A1	0.407096496
ILMN_2400935	TAGLN	0.407066568
ILMN_1796082	DHX57	0.406950719
ILMN_1673363	CD97	0.406934185
ILMN_1759983	DR1	0.406876211
ILMN_1720996	SLC12A2	0.406797011
ILMN_1675162	AK097193	0.406746567
ILMN_1754130	TRIM52	0.406742522
ILMN_2325506	BCAS4	0.406577416
ILMN_2219351	CENPO	0.406461651
ILMN_1789913	SMPDL3B	0.406272747
ILMN_1700378	ZWILCH	0.406259845
ILMN_1761540	SEMA3F	0.406238147
ILMN_2409167	ANXA2	0.406224506
ILMN_3236752	COAS3	0.406143591
ILMN_2368530	IL32	0.406121209
ILMN_2148785	GBP1	0.406030882
ILMN_1750761	NCR1	0.405994254
ILMN_2297864	MTMR14	0.40598887
ILMN_1730622	EVL	0.405957415
ILMN_1725644	UBE2D2	0.405716669
ILMN_1764754	RAMP1	0.405714535
ILMN_2199768	SLITRK4	0.405610391
ILMN_2129015	AFF1	0.40550332
ILMN_2390427	FLT4	0.405493508
ILMN_2360291	UGCGL1	0.405471767
ILMN_2066858	TNFSF13B	0.405419117
ILMN_3211199	ZNF316	0.405297968
ILMN_1777564	MAD2L1	0.405280667
ILMN_1700067	BTN3A2	0.405223794
ILMN_1768197	ROD1	0.405205631
ILMN_2382657	ARHGAP9	0.405204775
ILMN_2148819	TUBA1A	0.405149089
ILMN_1687592	WWC3	0.405141879
ILMN_3220934	NCRNA00152	0.405108576
ILMN_1767377	SMA4	0.405050431
ILMN_2400352	LRDD	0.405006518
ILMN_1669584	ILF3	0.405004046
ILMN_1798679	ANO9	0.40484745
ILMN_2396982	BCL2L12	0.404433884
ILMN_1681542	HIST1H4E	0.404382048
ILMN_1762899	EGR1	0.404362604
ILMN_3194118	C3orf16	0.404309058
ILMN_1766551	CPA3	0.404148165
ILMN_1747499	EMID1	0.404049613
ILMN_2343775	RPL38	0.404012236
ILMN_1733157	THSD1	0.403989191
ILMN_1742432	COBRA1	0.403981554
ILMN_1738694	WNT7B	0.403914993
ILMN_1781752	CLEC16A	0.403776549
ILMN_1775327	PKM2	0.403756842
ILMN_1791097	RSBN1	0.403753679
ILMN_3211187	TUBB2A	0.403753581
ILMN_1685830	SLC7A2	0.403556149
ILMN_1747451	PLCXD1	0.403531645
ILMN_3182487	BG722090	0.403487618
ILMN_1808777	EHD2	0.403307383
ILMN_3245923	AK301885	0.402930098
ILMN_1655046	NUTF2	0.402886751
ILMN_1654392	KHNYN	0.402880539
ILMN_1786612	PSME2	0.402718929

ILMN_1651604	MARK4	-0.579185945
ILMN_3246902	ABCC6P1	-0.579440398
ILMN_2043809	PFKM	-0.579868421
ILMN_1759910	SERPINA5	-0.580196376
ILMN_1663538	CLYBL	-0.580771636
ILMN_3182171	FGGY	-0.581109618
ILMN_1701497	CD5L	-0.581939676
ILMN_2212878	ESM1	-0.582094199
ILMN_2313434	TCP1	-0.582316387
ILMN_1808379	PLCD4	-0.583094515
ILMN_2147503	ALG13	-0.583274376
ILMN_1771084	ACSM3	-0.584096354
ILMN_1752214	BC141517	-0.584419315
ILMN_1738736	SNX4	-0.585396306
ILMN_1719468	EPM2A	-0.586245529
ILMN_1712430	ATP5G1	-0.586448069
ILMN_1748819	MRPL22	-0.586779542
ILMN_1681437	DCXR	-0.587731567
ILMN_1709635	SLC38A11	-0.587937657
ILMN_1802027	MGST2	-0.588165283
ILMN_1826447	BX107250	-0.589681093
ILMN_1712088	CLYBL	-0.589726246
ILMN_1732808	TOX3	-0.590099598
ILMN_2067682	LOC401431	-0.59189122
ILMN_1782579	IMMT	-0.59292823
ILMN_1705066	BTBD11	-0.592983431
ILMN_3288525	NA	-0.593028202
ILMN_1773262	ESM1	-0.593092449
ILMN_1784578	LRRC37A3	-0.594761115
ILMN_1685978	ATPIF1	-0.594923735
ILMN_1696675	CES2	-0.594988276
ILMN_1678720	SULT1C2P1	-0.595613107
ILMN_1859665	AW629225	-0.595837395
ILMN_1729281	SPHK2	-0.596105312
ILMN_2078074	MUT	-0.596139871
ILMN_1836218	LOC25845	-0.596157116
ILMN_1792744	SLC13A2	-0.597663072
ILMN_1763196	WDR72	-0.597813654
ILMN_2227817	GSTA3	-0.597817469
ILMN_1896474	AW269407	-0.597824984
ILMN_2226324	BRP44L	-0.598397894
ILMN_1751258	NDUFA4	-0.598635717
ILMN_1908274	AW236020	-0.59908023
ILMN_1753729	KNG1	-0.600371458
ILMN_1746819	C5	-0.600883539
ILMN_1685496	RGS7	-0.602245302
ILMN_1727178	C11orf92	-0.602617445
ILMN_1685703	ACOX2	-0.602707363
ILMN_1666939	MEI1	-0.603189574
ILMN_1809695	CAMK2G	-0.603207024
ILMN_1726273	ATP6V1G3	-0.604313858
ILMN_2376133	KIAA1191	-0.605124466
ILMN_1740920	ACADSB	-0.607603298
ILMN_1729482	KLK15	-0.60819346
ILMN_2196588	C18orf32	-0.608610031
ILMN_1761058	NPHP3-ACAD11	-0.60936502
ILMN_3306997	METTL1	-0.60940641
ILMN_3239925	LOC25845	-0.610644013
ILMN_1824490	BG618056	-0.610680966
ILMN_2054607	CYP4V2	-0.611190169
ILMN_1728467	GHITM	-0.613134427
ILMN_2161832	VPS37A	-0.613523403
ILMN_1801443	TSKU	-0.615195496
ILMN_1704247	LAMA3	-0.616805915
ILMN_2343097	NCALD	-0.617285357
ILMN_1685441	ASAP3	-0.617627034
ILMN_1745988	NUDT12	-0.621088638
ILMN_1788895	SRD5A2	-0.621494099
ILMN_1736969	SLITRK3	-0.623097006
ILMN_1777797	AFM	-0.623559015
ILMN_1670542	AK2	-0.62558549
ILMN_2232036	EFCAB7	-0.625854312
ILMN_1763092	MCCD1	-0.626401725
ILMN_1798853	CTSL1P2	-0.626894997
ILMN_2341548	MYO5B	-0.62916488
ILMN_1826572	AK094562	-0.630831038
ILMN_1878728	DB222718	-0.6308546
ILMN_1684585	ACSL1	-0.631605474
ILMN_1785252	SLC26A6	-0.631966776
ILMN_1768719	RDH11	-0.632212543
ILMN_2124361	LEAP2	-0.634775452
ILMN_1791588	S71824	-0.642483736
ILMN_1727309	FAM82A2	-0.642564793
ILMN_2199439	CA2	-0.643799017
ILMN_1700810	HSP90AA1	-0.645977781
ILMN_1694106	GPD1L	-0.656480919
ILMN_1779241	CRYM	-0.658657608
ILMN_1748352	CTSL2	-0.659673905
ILMN_1770245	EPB41L5	-0.660070465
ILMN_1753877	MYADML2	-0.660219722
ILMN_1778357	DNMT3L	-0.660374324
ILMN_3246634	SDHA	-0.662612025
ILMN_3245250	CTSL1P8	-0.665082308
ILMN_2193936	HRG	-0.668100275
ILMN_1654151	COX6C	-0.675159256
ILMN_1706579	SHBG	-0.676413554
ILMN_1803673	LOC113230	-0.677095151
ILMN_2050911	SLC22A4	-0.686403787
ILMN_3270542	UQCRFS1	-0.689758015
ILMN_1678965	FABP1	-0.69801149
ILMN_1740418	CYP27B1	-0.716855413
ILMN_1794844	THRSP	-0.723994052

ILMN_2230998	TMEM186	0.402696872
ILMN_1652611	C1D	0.402689208
ILMN_2350266	FBXO46	0.402651601
ILMN_1685371	SUMF2	0.402597428
ILMN_1654065	ATOH8	0.402590865
ILMN_3246391	SNORD52	0.402522447
ILMN_2070375	GPR82	0.402338398
ILMN_1796085	PPM1B	0.402315968
ILMN_1706434	CSDAP1	0.402257386
ILMN_1704753	EPAS1	0.402216039
ILMN_3262298	LOC100130357	0.402075502
ILMN_1663866	TGFBI	0.402040888
ILMN_1674519	ZP2	0.401815794
ILMN_1814799	S100A13	0.401656514
ILMN_2364828	OGT	0.401545481
ILMN_1762787	RNF26	0.401529643
ILMN_1698100	ANX2P2	0.40146465
ILMN_3289346	AX747493	0.401407661
ILMN_2222234	PRDX4	0.401386729
ILMN_2243912	RAB37	0.401302072
ILMN_1784717	RPS19	0.401267792
ILMN_1779185	SPECC1L	0.401240483
ILMN_1697220	NT5E	0.401130225
ILMN_1719105	KRTAP20-1	0.401072137
ILMN_1733947	FKBP8	0.401029305
ILMN_3283015	NSA2	0.400953922
ILMN_1697417	CEP170	0.400772249
ILMN_1802251	PTTG1IP	0.400745049
ILMN_1717327	KCNJ14	0.400744433
ILMN_1680435	NPEPPS	0.40068875
ILMN_1784364	STARD5	0.400666852
ILMN_1842797	AW293882	0.400634792
ILMN_1830984	BF509118	0.400628925
ILMN_3178471	ESPNL	0.400611827
ILMN_2412214	HUAT	0.400468568
ILMN_1743137	PRRC2C	0.400336982
ILMN_1873838	IGKC	0.400154093
ILMN_1787951	RABL5	0.400100335
ILMN_1831763	BQ709011	0.400019768
