# Supplementary material for: Intrarenal activation of adaptive immune effectors is associated with tubular damage and impaired renal function in lupus nephritis
Source: Ann Rheum Dis. 2018 Jul 31;77(12):1782–9. doi: 10.1136/annrheumdis-2018-213485 (PMC6241616; doi:10.1136/annrheumdis-2018-213485)
Supplement: Supplementary file 8 [file annrheumdis-2018-213485supp008.pdf]

Supplementary Table 5 : Transcripts and DAVID pathways correlating with the expression of CD4 in lupus kidney biopsies

Positive correlation

Transcripts

Identifier	Gene Symbol	Correlation coefficient with CD4
ILMN_1727284	CD4	1
ILMN_1772218	HLA-DPA1	0.934229063
ILMN_1778977	TYROBP	0.933302249
ILMN_1689655	HLA-DRA	0.915891173
ILMN_3228688	HLA-DRB1	0.906190291
ILMN_2157441	HLA-DRA	0.902996561
ILMN_2066066	HLA-DRB6	0.901531942
ILMN_1736567	CD74	0.900464276
ILMN_2366330	FERMT3	0.896532157
ILMN_1815895	HLA-DRB1	0.895853418
ILMN_2379644	CD74	0.890964861
ILMN_2049536	TRPV2	0.889679914
ILMN_1718936	LST1	0.886044814
ILMN_1718558	PARP12	0.883825964
ILMN_3240316	TMSB4X	0.881816413
ILMN_1683026	PSMB10	0.881679636
ILMN_2179837	BANF1	0.881663753
ILMN_1786612	PSME2	0.881166328
ILMN_1653828	CHFR	0.879799743
ILMN_1754842	DLGAP4	0.875737515
ILMN_1668039	GYPC	0.874017008
ILMN_1752340	ARF5	0.871364059
ILMN_1654319	HAPLN3	0.86967548
ILMN_2390299	PSMB8	0.868740369
ILMN_1747195	PSMB8	0.866391356
ILMN_2176063	FCGR1C	0.866366101
ILMN_2111739	MAN2C1	0.866279082
ILMN_1807277	IFI30	0.86456805
ILMN_1794473	PHF17	0.863368562
ILMN_1758250	TRAFD1	0.862805609
ILMN_1772359	LAPTM5	0.862504464
ILMN_1679809	GSTP1	0.861629683
ILMN_1651788	MAP3K11	0.861566346
ILMN_1778360	PYGB	0.861254289
ILMN_2349658	TSPO	0.860302388
ILMN_1740015	CD14	0.858368684
ILMN_1760683	SRSF9	0.857616844
ILMN_2062468	IGFBP7	0.857204295
ILMN_1715024	LSS	0.857094666
ILMN_1723706	GRAMD4	0.856571802
ILMN_1782057	ATP8B2	0.855792595
ILMN_2366041	ITM2C	0.855750662
ILMN_1761464	CD74	0.854043623
ILMN_1689968	PLEKHO2	0.853935274
ILMN_1680453	ITM2C	0.853816711
ILMN_1691892	TAGLN2	0.853601352
ILMN_1683271	TMSB4X	0.853486495
ILMN_1785005	NCF4	0.852288271
ILMN_1685005	TNFRSF1A	0.852016046
ILMN_1741371	TMEM8A	0.851368336
ILMN_1752592	HLA-DRB4	0.851148745
ILMN_2400947	CELF2	0.850982825
ILMN_2370772	EIF4G1	0.850763294
ILMN_1712950	PFN1	0.850098377
ILMN_1793950	POTEM	0.849590981
ILMN_1669550	MAD2L2	0.849558148
ILMN_1717261	HLA-DRB1	0.84901389
ILMN_1659688	LGALS3BP	0.8488395
ILMN_1660832	RPL18A	0.848663905
ILMN_1745148	ZNFX1	0.848322315
ILMN_3194911	ATF6B	0.847634385
ILMN_1775762	GNAI2	0.847561057
ILMN_1720476	PHF2	0.847273916
ILMN_1723978	LGALS1	0.846002437
ILMN_1738116	TMEM119	0.845930944
ILMN_1782538	VIM	0.845340049
ILMN_1733947	FKBP8	0.844948911
ILMN_2376108	PSMB9	0.84365789
ILMN_1687533	SEMA4D	0.843172109
ILMN_2278729	EIF3B	0.842759453
ILMN_3229210	FLJ45340	0.842202302
ILMN_1770673	AKNA	0.842092
ILMN_1653220	PITPNM1	0.841307988
ILMN_1749011	NECAP2	0.840455038
ILMN_2379734	CTBP1	0.83935087
ILMN_1695311	HLA-DMA	0.838589567
ILMN_1755733	RPLP2	0.838180672
ILMN_2387496	COAS3	0.837846899
ILMN_1786024	POLR3H	0.837564447
ILMN_1772486	ELF2	0.835726208
ILMN_2120965	NPAT	0.835607122
ILMN_1716547	NAGK	0.834757851
ILMN_1749662	GPX1	0.834646595
ILMN_1778255	FARSA	0.834189514

Pathways

**Annotation Cluster 1**	**Enrichment Score: 26.47**		**Count**	**P_Value**	**Benjamini**
	GOTERM_BP_FAT	translational elongation	74	2.50E-42	1.00E-38
	SP_PIR_KEYWORDS	ribosome	60	2.00E-41	4.60E-39
	KEGG_PATHWAY	Ribosome	65	3.80E-35	6.60E-33
	SP_PIR_KEYWORDS	protein biosynthesis	91	4.10E-34	7.20E-32
	SP_PIR_KEYWORDS	ribonucleoprotein	114	4.80E-34	6.70E-32
	GOTERM_CC_FAT	cytosolic ribosome	59	3.20E-33	2.30E-30
	SP_PIR_KEYWORDS	ribosomal protein	85	3.10E-29	3.60E-27
	GOTERM_CC_FAT	ribosomal subunit	70	6.50E-28	2.30E-25
	GOTERM_BP_FAT	translation	122	6.60E-28	1.40E-24
	GOTERM_CC_FAT	cytosolic part	76	5.60E-27	1.30E-24
	GOTERM_MF_FAT	structural constituent of ribosome	79	6.80E-27	8.90E-24
	GOTERM_CC_FAT	ribonucleoprotein complex	160	2.50E-26	4.30E-24
	GOTERM_CC_FAT	ribosome	90	7.50E-25	8.70E-23
	GOTERM_CC_FAT	cytosolic small ribosomal subunit	30	1.80E-17	1.80E-15
	GOTERM_CC_FAT	cytosolic large ribosomal subunit	29	3.10E-17	2.70E-15
	GOTERM_CC_FAT	large ribosomal subunit	39	5.60E-17	8.70E-15
	GOTERM_MF_FAT	structural molecule activity	154	1.30E-15	5.80E-13
	GOTERM_CC_FAT	small ribosomal subunit	32	7.30E-12	3.40E-10
**Annotation Cluster 2**	**Enrichment Score: 10.95**		**Count**	**P_Value**	**Benjamini**
	GOTERM_CC_FAT	intracellular non-membrane-bounded organelle	477	3.80E-16	2.30E-14
	GOTERM_CC_FAT	non-membrane-bounded organelle	477	3.80E-16	2.30E-14
	GOTERM_CC_FAT	cytoskeleton	214	9.90E-03	1.30E-01
**Annotation Cluster 3**	**Enrichment Score: 10.64**		**Count**	**P_Value**	**Benjamini**
	GOTERM_CC_FAT	intracellular organelle lumen	343	3.90E-14	2.50E-12
	GOTERM_CC_FAT	membrane-enclosed lumen	354	6.40E-14	3.70E-12
	GOTERM_CC_FAT	organelle lumen	348	7.50E-14	4.10E-12
	GOTERM_CC_FAT	nuclear lumen	284	1.90E-12	9.40E-11
	GOTERM_CC_FAT	nucleoplasm	170	3.50E-07	1.50E-05
	GOTERM_CC_FAT	nucleolus	138	1.30E-06	4.90E-05
**Annotation Cluster 4**	**Enrichment Score: 6.84**		**Count**	**P_Value**	**Benjamini**
	SP_PIR_KEYWORDS	ubl conjugation	117	4.10E-08	2.20E-06
	UP_SEQ_FEATURE	cross-link:Glycyl lysine isopeptide (Lys-Gly) (interchain with G-Cter in ubiquitin)	53	4.50E-08	8.10E-05
	SP_PIR_KEYWORDS	isopeptide bond	69	1.70E-06	5.80E-05
**Annotation Cluster 5**	**Enrichment Score: 5.35**		**Count**	**P_Value**	**Benjamini**
	GOTERM_BP_FAT	macromolecular complex assembly	136	1.00E-07	7.20E-05
	GOTERM_BP_FAT	macromolecular complex subunit organization	138	1.70E-06	3.10E-04
	GOTERM_BP_FAT	cellular macromolecular complex assembly	73	2.10E-06	3.20E-04
	GOTERM_BP_FAT	cellular macromolecular complex subunit organization	77	1.20E-05	1.20E-03
	GOTERM_BP_FAT	protein complex assembly	99	4.10E-05	3.20E-03
	GOTERM_BP_FAT	protein complex biogenesis	99	4.10E-05	3.20E-03
**Annotation Cluster 6**	**Enrichment Score: 4.78**		**Count**	**P_Value**	**Benjamini**
	SP_PIR_KEYWORDS	mrna splicing	54	8.30E-08	3.90E-06
	SP_PIR_KEYWORDS	mrna processing	61	4.30E-07	1.70E-05
	GOTERM_BP_FAT	RNA splicing	69	4.80E-07	2.20E-04
	GOTERM_BP_FAT	RNA processing	114	4.80E-07	2.00E-04
	GOTERM_BP_FAT	mRNA metabolic process	83	1.00E-06	2.40E-04
	GOTERM_BP_FAT	mRNA processing	74	1.50E-06	2.90E-04
	SP_PIR_KEYWORDS	Spliceosome	30	1.70E-04	3.30E-03
	KEGG_PATHWAY	Spliceosome	34	6.70E-04	3.80E-02
	GOTERM_BP_FAT	nuclear mRNA splicing, via spliceosome	36	6.80E-04	3.10E-02
	GOTERM_BP_FAT	RNA splicing, via transesterification reactions with bulged adenosine as nucleophile	36	6.80E-04	3.10E-02
	GOTERM_BP_FAT	RNA splicing, via transesterification reactions	36	6.80E-04	3.10E-02
	GOTERM_CC_FAT	spliceosome	32	1.00E-03	2.10E-02
**Annotation Cluster 7**	**Enrichment Score: 4.43**		**Count**	**P_Value**	**Benjamini**
	GOTERM_BP_FAT	regulation of cellular protein metabolic process	112	4.70E-10	6.50E-07
	GOTERM_BP_FAT	regulation of protein modification process	71	4.70E-07	2.40E-04
	GOTERM_BP_FAT	positive regulation of ubiquitin-protein ligase activity during mitotic cell cycle	26	7.50E-07	2.60E-04
	GOTERM_BP_FAT	positive regulation of protein metabolic process	61	7.50E-07	2.40E-04
	GOTERM_BP_FAT	negative regulation of protein modification process	37	8.50E-07	2.30E-04
	GOTERM_BP_FAT	positive regulation of cellular protein metabolic process	59	8.60E-07	2.20E-04
	GOTERM_BP_FAT	negative regulation of cellular protein metabolic process	49	9.10E-07	2.20E-04
	GOTERM_BP_FAT	negative regulation of ubiquitin-protein ligase activity during mitotic cell cycle	25	1.10E-06	2.50E-04
	GOTERM_BP_FAT	anaphase-promoting complex-dependent proteasomal ubiquitin-dependent protein catabolic process	25	1.10E-06	2.50E-04
	GOTERM_BP_FAT	positive regulation of ubiquitin-protein ligase activity	26	1.40E-06	2.90E-04
	GOTERM_BP_FAT	positive regulation of protein ubiquitination	29	1.60E-06	3.10E-04
	GOTERM_BP_FAT	negative regulation of molecular function	76	1.80E-06	3.10E-04
	GOTERM_BP_FAT	regulation of ubiquitin-protein ligase activity during mitotic cell cycle	26	1.90E-06	3.00E-04
	GOTERM_BP_FAT	negative regulation of ubiquitin-protein ligase activity	25	2.10E-06	3.20E-04
	GOTERM_BP_FAT	negative regulation of ligase activity	25	2.10E-06	3.20E-04
	GOTERM_BP_FAT	negative regulation of protein metabolic process	49	3.00E-06	4.30E-04
	GOTERM_BP_FAT	positive regulation of ligase activity	26	3.40E-06	4.40E-04
	GOTERM_BP_FAT	regulation of ubiquitin-protein ligase activity	27	3.80E-06	4.80E-04
	GOTERM_BP_FAT	negative regulation of protein ubiquitination	26	4.50E-06	5.50E-04
	GOTERM_BP_FAT	regulation of protein ubiquitination	31	8.20E-06	9.20E-04
	GOTERM_BP_FAT	regulation of ligase activity	27	8.40E-06	9.20E-04
	GOTERM_BP_FAT	negative regulation of catalytic activity	63	1.50E-05	1.50E-03
	GOTERM_BP_FAT	positive regulation of protein modification process	47	1.60E-05	1.50E-03
	GOTERM_BP_FAT	proteasomal protein catabolic process	30	3.50E-05	2.90E-03
	GOTERM_BP_FAT	proteasomal ubiquitin-dependent protein catabolic process	30	3.50E-05	2.90E-03
	INTERPRO	Proteasome, alpha and beta subunits	9	9.40E-05	4.70E-02
	SP_PIR_KEYWORDS	proteasome	18	2.10E-04	4.00E-03
	INTERPRO	Proteasome, beta-type subunit, conserved site	8	2.20E-04	9.00E-02
	GOTERM_CC_FAT	proteasome complex	20	2.50E-04	6.60E-03
	SP_PIR_KEYWORDS	proteinase	9	3.90E-04	6.80E-03

Negative correlation

Transcripts

Identifier	Gene Symbol	Correlation coefficient with CD4
ILMN_1681389	C2H2 zinc finger pr[illegible]	-0.40001984
ILMN_1768954	RIBC1	-0.400186083
ILMN_1699190	SORCS1	-0.400223681
ILMN_3249088	LOC93432	-0.400285215
ILMN_1680436	CSH2	-0.400299744
ILMN_1722755	KRTAP6-2	-0.400334007
ILMN_3310171	MIR210	-0.400359894
ILMN_1799903	GALNTL1	-0.400392176
ILMN_1754727	ARMCX5	-0.40044397
ILMN_1660020	LOC644662	-0.400562184
ILMN_2369826	C16orf13	-0.400562661
ILMN_2206554	SLC35F5	-0.400563498
ILMN_1853093	BG325888	-0.400578134
ILMN_2331232	TNFRSF6B	-0.400582848
ILMN_1700169	C15orf44	-0.400627977
ILMN_1771689	EXD2	-0.400641297
ILMN_1679134	NSMCE4A	-0.400646612
ILMN_1838375	BM714348	-0.400665624
ILMN_1653266	DNAJB14	-0.400749707
ILMN_1719316	TMED3	-0.400813303
ILMN_3257233	AK126616	-0.400986267
ILMN_1692276	GGPS1	-0.400992588
ILMN_1790595	TFAP2E	-0.401012982
ILMN_1734559	SORD	-0.401202426
ILMN_1698731	SEC31B	-0.401253836
ILMN_1652333	FN3KRP	-0.401355089
ILMN_2052863	RNF5	-0.401425107
ILMN_3311095	MIR643	-0.401427458
ILMN_1663263	EPHA10	-0.401468095
ILMN_3255211	KIAA2015	-0.401494305
ILMN_1676548	BZW2	-0.401528449
ILMN_1709020	MYBL2	-0.401560655
ILMN_1651341	LOC401010	-0.40162232
ILMN_3285238	BX537874	-0.40162554
ILMN_1701239	SPRR2E	-0.401647677
ILMN_2288290	CDY1B	-0.4017477
ILMN_1674297	HCFC2	-0.401754371
ILMN_1769071	XKRX	-0.40180268
ILMN_1773968	SERBP1	-0.401804507
ILMN_2222163	HIST1H3B	-0.401887856
ILMN_1781672	GAB1	-0.401901556
ILMN_1660852	ZC3H6	-0.401907906
ILMN_1736045	KCNJ16	-0.401981532
ILMN_2389528	MTL5	-0.401986801
ILMN_1805842	FHL1	-0.401998765
ILMN_1791702	SMARCA2	-0.402008017
ILMN_1806022	RNF212	-0.402016792
ILMN_1760849	NETO2	-0.402029764
ILMN_3277402	LOC441242	-0.402055879
ILMN_1732584	SHISA6	-0.40209057
ILMN_1740920	ACADSB	-0.40236063
ILMN_1727252	WNT8A	-0.402361321
ILMN_1791679	DNER	-0.402409976
ILMN_1714813	DPY19L2P2	-0.402417622
ILMN_1751036	SMAD2	-0.402457469
ILMN_1823229	BX094072	-0.4024611
ILMN_1769704	FLJ39632	-0.402484676
ILMN_2101025	ZNF75D	-0.402489395
ILMN_1724149	OCLN	-0.40249034
ILMN_2377862	RAB6A	-0.402512772
ILMN_3231804	LL0XNC01-19D8.1	-0.402512855
ILMN_3310336	MIR450A1	-0.402559411
ILMN_2203147	TMPRSS12	-0.402569462
ILMN_1726815	HIST1H3G	-0.402576019
ILMN_1745110	LAPTM4A	-0.402745881
ILMN_1781764	RPL36A	-0.402746902
ILMN_1792002	PAPPA2	-0.402751808
ILMN_1688831	SYT7	-0.402796218
ILMN_1723574	TSG1	-0.402856432
ILMN_1821279	BM678326	-0.402875566
ILMN_1877778	DA086964	-0.402971114
ILMN_1678306	NA	-0.403011806
ILMN_1769918	APBB1	-0.403049265
ILMN_3243908	SNHG7	-0.403056774
ILMN_1669486	tMDC	-0.403074616
ILMN_1908258	BF510793	-0.403134314
ILMN_2099594	SRP9	-0.403137412
ILMN_2156267	EIF2AK1	-0.403168571
ILMN_1776552	FUBP1	-0.403195196
ILMN_2174127	DCBLD2	-0.403197796
ILMN_3206565	XAGE5	-0.403233714
ILMN_1747854	GOLGA8E	-0.403251728
ILMN_1838254	NA	-0.403256259
ILMN_1662158	RPL26L1	-0.403302701

Pathways

**Annotation Cluster 1**	**Enrichment Score: 3.16**		**Count**	**P_Value**	**Benjamini**
	INTERPRO	Ubiquitin-conjugating enzyme/RWD-like	17	2.00E-05	4.20E-02
	SMART	UBCc	14	1.00E-04	3.50E-02
	SP_PIR_KEYWORDS	ubl conjugation pathway	81	1.30E-04	2.30E-02
	GOTERM_MF_FAT	small conjugating protein ligase activity	35	1.40E-04	4.40E-02
	SP_PIR_KEYWORDS	ligase	54	1.50E-04	2.00E-02
	GOTERM_MF_FAT	acid-amino acid ligase activity	40	1.60E-04	4.00E-02
	INTERPRO	Ubiquitin-conjugating enzyme, E2	14	2.10E-04	2.00E-01
	GOTERM_MF_FAT	ubiquitin-protein ligase activity	31	3.60E-04	7.40E-02
	GOTERM_MF_FAT	ligase activity, forming carbon-nitrogen bonds	43	3.90E-04	6.10E-02
	GOTERM_BP_FAT	protein ubiquitination	25	1.40E-03	5.70E-01
	GOTERM_BP_FAT	protein modification by small protein conjugation	26	2.80E-03	6.00E-01
	UP_SEQ_FEATURE	active site:Glycyl thioester intermediate	16	4.70E-03	9.90E-01
	GOTERM_BP_FAT	protein modification by small protein conjugation or removal	28	9.90E-03	8.80E-01
	KEGG_PATHWAY	Ubiquitin mediated proteolysis	25	1.60E-02	7.80E-01
	GOTERM_CC_FAT	ubiquitin ligase complex	16	5.60E-02	9.00E-01
**Annotation Cluster 2**	**Enrichment Score: 2.83**		**Count**	**P_Value**	**Benjamini**
	GOTERM_BP_FAT	dephosphorylation	41	5.20E-08	1.80E-04
	GOTERM_BP_FAT	protein amino acid dephosphorylation	36	2.50E-07	4.40E-04
	GOTERM_MF_FAT	phosphoprotein phosphatase activity	41	4.70E-07	6.10E-04
	GOTERM_MF_FAT	phosphatase activity	54	6.40E-07	4.10E-04
	GOTERM_MF_FAT	protein tyrosine phosphatase activity	28	8.70E-06	3.70E-03
	SP_PIR_KEYWORDS	protein phosphatase	30	6.30E-05	1.40E-02
	UP_SEQ_FEATURE	active site:Phosphocysteine intermediate	21	3.00E-04	7.30E-01
	INTERPRO	Protein-tyrosine phosphatase, active site	22	3.20E-04	2.10E-01
	INTERPRO	Dual-specific/protein-tyrosine phosphatase, conserved region	20	7.80E-04	2.90E-01
	SMART	PTPc	12	1.30E-03	2.10E-01
	INTERPRO	Protein-tyrosine phosphatase	19	1.80E-03	4.80E-01
	INTERPRO	Protein-tyrosine phosphatase, receptor/non-receptor type	12	2.30E-03	5.20E-01
	UP_SEQ_FEATURE	domain:Tyrosine-protein phosphatase	13	2.60E-02	1.00E+00
	GOTERM_MF_FAT	protein tyrosine/serine/threonine phosphatase activity	10	4.00E-02	8.30E-01
	SP_PIR_KEYWORDS	tyrosine-specific phosphatase	7	9.90E-02	8.40E-01
	SP_PIR_KEYWORDS	phosphoric monoester hydrolase	12	1.00E-01	8.30E-01
	INTERPRO	Dual specificity phosphatase, catalytic domain	8	1.10E-01	1.00E+00
	INTERPRO	Protein-tyrosine phosphatase, dual specificity	7	1.90E-01	1.00E+00
	GOTERM_MF_FAT	transmembrane receptor protein phosphatase activity	4	3.00E-01	9.90E-01
	UP_SEQ_FEATURE	domain:Tyrosine-protein phosphatase 2	3	3.60E-01	1.00E+00
	UP_SEQ_FEATURE	domain:Tyrosine-protein phosphatase 1	3	3.60E-01	1.00E+00
	SMART	DSPc	3	8.00E-01	1.00E+00
**Annotation Cluster 3**	**Enrichment Score: 2.62**		**Count**	**P_Value**	**Benjamini**
	SP_PIR_KEYWORDS	ubl conjugation pathway	81	1.30E-04	2.30E-02
	GOTERM_BP_FAT	modification-dependent protein catabolic process	88	3.50E-04	2.70E-01
	GOTERM_BP_FAT	modification-dependent macromolecule catabolic process	88	3.50E-04	2.70E-01
	GOTERM_BP_FAT	proteolysis involved in cellular protein catabolic process	88	1.40E-03	5.20E-01
	GOTERM_BP_FAT	cellular protein catabolic process	88	1.70E-03	5.20E-01
	GOTERM_BP_FAT	cellular macromolecule catabolic process	102	2.30E-03	6.00E-01
	GOTERM_BP_FAT	protein catabolic process	88	4.00E-03	6.70E-01
	GOTERM_BP_FAT	macromolecule catabolic process	106	5.70E-03	7.40E-01
	GOTERM_BP_FAT	proteolysis	136	1.00E-02	8.40E-01
	GOTERM_BP_FAT	ubiquitin-dependent protein catabolic process	30	2.70E-01	9.90E-01
**Annotation Cluster 4**	**Enrichment Score: 2.24**		**Count**	**P_Value**	**Benjamini**
	GOTERM_CC_FAT	mitochondrion	158	4.70E-05	2.70E-02
	SP_PIR_KEYWORDS	mitochondrion	120	2.40E-04	2.30E-02
	SP_PIR_KEYWORDS	transit peptide	71	2.10E-03	1.20E-01
	UP_SEQ_FEATURE	transit peptide:Mitochondrion	70	2.30E-03	9.60E-01
	GOTERM_CC_FAT	mitochondrial part	80	2.70E-02	8.70E-01
	GOTERM_CC_FAT	mitochondrial envelope	55	9.00E-02	9.20E-01
	GOTERM_CC_FAT	mitochondrial matrix	32	9.70E-02	9.20E-01
	GOTERM_CC_FAT	mitochondrial lumen	32	9.70E-02	9.20E-01
**Annotation Cluster 5**	**Enrichment Score: 2.15**		**Count**	**P_Value**	**Benjamini**
	GOTERM_BP_FAT	cofactor metabolic process	36	1.30E-03	5.90E-01
	GOTERM_BP_FAT	coenzyme metabolic process	29	2.80E-03	6.30E-01
	GOTERM_BP_FAT	cofactor biosynthetic process	19	1.30E-02	8.70E-01
	GOTERM_BP_FAT	coenzyme biosynthetic process	13	5.70E-02	9.80E-01
**Annotation Cluster 6**	**Enrichment Score: 2.04**		**Count**	**P_Value**	**Benjamini**
	GOTERM_BP_FAT	water-soluble vitamin metabolic process	11	7.50E-03	8.10E-01
	GOTERM_BP_FAT	betaine metabolic process	4	1.00E-02	8.70E-01
	GOTERM_BP_FAT	carnitine metabolic process	4	1.00E-02	8.70E-01
**Annotation Cluster 7**	**Enrichment Score: 1.94**		**Count**	**P_Value**	**Benjamini**
	UP_SEQ_FEATURE	domain:Myotubularin phosphatase	6	1.10E-02	1.00E+00
	INTERPRO	Myotubularin phosphatase	6	1.20E-02	9.40E-01
	INTERPRO	Myotubularin-related	6	1.20E-02	9.40E-01
**Annotation Cluster 8**	**Enrichment Score: 1.43**		**Count**	**P_Value**	**Benjamini**
	GOTERM_BP_FAT	carboxylic acid catabolic process	20	2.40E-02	9.60E-01
	GOTERM_BP_FAT	organic acid catabolic process	20	2.40E-02	9.60E-01
	GOTERM_BP_FAT	cellular amino acid catabolic process	13	5.20E-02	9.80E-01
	GOTERM_BP_FAT	amine catabolic process	14	6.60E-02	9.80E-01
**Annotation Cluster 9**	**Enrichment Score: 1.38**		**Count**	**P_Value**	**Benjamini**
	GOTERM_MF_FAT	enzyme binding	71	2.80E-02	8.40E-01
	GOTERM_MF_FAT	protein kinase binding	24	4.00E-02	8.30E-01
	GOTERM_MF_FAT	kinase binding	27	6.50E-02	8.90E-01
**Annotation Cluster 10**	**Enrichment Score: 1.33**		**Count**	**P_Value**	**Benjamini**
	SP_PIR_KEYWORDS	nucleotide-binding	213	2.10E-03	1.30E-01
	GOTERM_MF_FAT	nucleotide binding	276	5.40E-03	4.10E-01
	SP_PIR_KEYWORDS	atp-binding	162	2.30E-02	6.10E-01

ILMN_1728224	OGFR	0.834143823
ILMN_2400935	TAGLN	0.834136449
ILMN_1697567	TPM3	0.833912646
ILMN_2310968	RUFY1	0.833741884
ILMN_1690610	RALY	0.833051016
ILMN_1799815	XYLT2	0.832847321
ILMN_1708858	CSNK1E	0.832300175
ILMN_3237826	CNTNAP3B	0.831914342
ILMN_1679880	THOC6	0.831798261
ILMN_2391891	MAP2K5	0.831765836
ILMN_3234828	TMEM229B	0.83164614
ILMN_1742450	TAPBP	0.831521403
ILMN_1695341	KCNK10	0.831345574
ILMN_1815083	WHSC2	0.831045368
ILMN_1774733	SOCS1	0.830948173
ILMN_1681998	AP2B1	0.830807069
ILMN_2396571	GPR108	0.829917167
ILMN_1704286	FXYD5	0.829720373
ILMN_2222688	TMSB4X	0.829404615
ILMN_1727043	GLT25D1	0.82934787
ILMN_1654246	SIRT6	0.829048964
ILMN_2070052	NPIPL3	0.828694714
ILMN_1701643	GDPD5	0.828367608
ILMN_1684306	S100A4	0.828034127
ILMN_2090105	TAGLN2	0.827793534
ILMN_1745807	TMEM62	0.827749835
ILMN_3241979	TMEM179B	0.827418143
ILMN_2064606	TBC1D2B	0.827258435
ILMN_1679039	CASKIN1	0.826970028
ILMN_2410783	GAA	0.826601383
ILMN_2408987	SMARCD3	0.826251666
ILMN_1800354	CST3	0.825786839
ILMN_1787109	CLK2	0.82575294
ILMN_2382431	ANKRD36	0.824841327
ILMN_1803810	RRBP1	0.824545261
ILMN_1812327	RNF19A	0.82428379
ILMN_2155719	NBPF1	0.824018549
ILMN_1805750	IFITM3	0.823656128
ILMN_2115633	CD320	0.823505643
ILMN_1718769	ITSN1	0.823342308
ILMN_1685088	RPL36	0.823301945
ILMN_1693552	CD300A	0.823185387
ILMN_1736256	CALR	0.823016189
ILMN_1765021	TOP3B	0.822718182
ILMN_1652825	IL10RA	0.822561758
ILMN_1786823	ICAM2	0.821328086
ILMN_2200562	LOC595101	0.821150358
ILMN_1680403	SSR4	0.820979014
ILMN_1689953	CD81	0.820970821
ILMN_1761912	MGAT1	0.820751551
ILMN_1683576	MAGED2	0.81984923
ILMN_2367707	PKN1	0.818771419
ILMN_1727271	WARS	0.8183549
ILMN_1720124	RCC2	0.817782509
ILMN_2368068	TCF20	0.817716324
ILMN_1760890	SEPN1	0.817576064
ILMN_1712944	AES	0.816642821
ILMN_1754121	CSK	0.816591048
ILMN_2092756	TMEM109	0.816343494
ILMN_2159453	STXBP2	0.816323572
ILMN_3203186	AL543610	0.816262196
ILMN_1702787	SEMA4A	0.816184641
ILMN_2397776	ASB6	0.816125049
ILMN_1747556	CDK9	0.816085417
ILMN_3247325	PP13759	0.815788721
ILMN_1704753	EPAS1	0.815724159
ILMN_1756360	RPL35A	0.815498871
ILMN_2312906	MIA3	0.815209851
ILMN_1765801	GAA	0.815204248
ILMN_1765258	HLA-E	0.814863854
ILMN_1727642	MOGS	0.814624244
ILMN_3243664	NPIPL3	0.814276952
ILMN_1737074	RPS2	0.813976656
ILMN_1808860	STX5	0.813882479
ILMN_1770787	DDAH2	0.813281485
ILMN_3244065	C9orf69	0.812726623
ILMN_1760563	PRRC2A	0.812521977
ILMN_1667813	RPL7	0.812505284
ILMN_1704315	RPL27A	0.81238402
ILMN_3225505	RPS8	0.812380428
ILMN_1673352	IFITM2	0.81218353
ILMN_1685286	PPP1R12C	0.811843659
ILMN_1744912	CTTN	0.811662697
ILMN_1666976	PLD3	0.811528784
ILMN_1733511	GOLGA3	0.811321895
ILMN_1729408	C7orf28B	0.811262169
ILMN_1718044	ELAC2	0.811198184
ILMN_3251733	C19orf43	0.811058332
ILMN_1811702	GRN	0.810939309
ILMN_1759154	PABPN1	0.810934215
ILMN_1675709	ARFGAP1	0.810665935
ILMN_2403852	CYTH1	0.810197199
ILMN_1784467	NUP210	0.810119504
ILMN_1653708	CORO1B	0.809969137
ILMN_2254635	ITGAX	0.809557107
ILMN_1666635	RPS14	0.809246354

	INTERPRO	Proteasome, subunit alpha/beta	9	1.30E-03	2.40E-01
	SP_PIR_KEYWORDS	threonine protease	9	1.40E-03	2.10E-02
	GOTERM_MF_FAT	threonine-type peptidase activity	9	2.20E-03	1.20E-01
	GOTERM_MF_FAT	threonine-type endopeptidase activity	9	2.20E-03	1.20E-01
	GOTERM_CC_FAT	proteasome core complex	9	2.80E-03	4.80E-02
	GOTERM_BP_FAT	mitotic cell cycle	67	6.00E-03	1.40E-01
	KEGG_PATHWAY	Proteasome	15	7.20E-03	1.50E-01
	PIR_SUPERFAMILY	PIRSF001212:multicatalytic endopeptidase complex chain C9	4	2.00E-02	9.90E-01
	UP_SEQ_FEATURE	site:Cleavage; by autocatalysis	3	4.00E-02	9.90E-01
	INTERPRO	Peptidase T1A, proteasome beta-subunit	4	4.70E-02	9.20E-01
	GOTERM_BP_FAT	ubiquitin-dependent protein catabolic process	42	5.30E-02	5.10E-01
	PIR_SUPERFAMILY	PIRSF001214:multicatalytic endopeptidase complex chain C5	3	9.10E-02	1.00E+00
**Annotation Cluster 8**	**Enrichment Score: 4.36**		**Count**	**P_Value**	**Benjamini**
	SP_PIR_KEYWORDS	sh3 domain	50	3.40E-06	1.00E-04
	UP_SEQ_FEATURE	domain:SH3	41	6.30E-05	6.50E-02
	INTERPRO	Src homology-3 domain	48	8.00E-05	4.90E-02
	SMART	SH3	48	2.10E-04	4.30E-02
**Annotation Cluster 9**	**Enrichment Score: 4.32**		**Count**	**P_Value**	**Benjamini**
	GOTERM_BP_FAT	regulation of protein kinase cascade	64	1.60E-07	9.30E-05
	GOTERM_BP_FAT	positive regulation of I-kappaB kinase/NF-kappaB cascade	29	3.50E-05	2.90E-03
	GOTERM_BP_FAT	positive regulation of protein kinase cascade	42	4.70E-05	3.50E-03
	GOTERM_BP_FAT	regulation of I-kappaB kinase/NF-kappaB cascade	30	9.20E-05	6.10E-03
	GOTERM_BP_FAT	positive regulation of cell communication	66	4.70E-04	2.40E-02
	GOTERM_BP_FAT	positive regulation of signal transduction	59	1.00E-03	4.30E-02
**Annotation Cluster 10**	**Enrichment Score: 4.32**		**Count**	**P_Value**	**Benjamini**
	GOTERM_BP_FAT	vesicle-mediated transport	125	1.10E-08	1.20E-05
	GOTERM_BP_FAT	membrane organization	78	6.90E-05	4.90E-03
	GOTERM_BP_FAT	endocytosis	50	1.30E-04	8.50E-03
	GOTERM_BP_FAT	membrane invagination	50	1.30E-04	8.50E-03
	SP_PIR_KEYWORDS	Endocytosis	20	1.90E-02	1.60E-01
**Annotation Cluster 11**	**Enrichment Score: 4.23**		**Count**	**P_Value**	**Benjamini**
	GOTERM_MF_FAT	transcription factor binding	111	2.40E-08	7.80E-06
	GOTERM_MF_FAT	transcription activator activity	78	3.40E-04	3.70E-02
	GOTERM_MF_FAT	transcription cofactor activity	70	4.80E-04	4.40E-02
	GOTERM_MF_FAT	transcription coactivator activity	43	3.10E-03	1.40E-01
**Annotation Cluster 12**	**Enrichment Score: 4.18**		**Count**	**P_Value**	**Benjamini**
	GOTERM_BP_FAT	positive regulation of cell death	91	6.60E-06	7.90E-04
	GOTERM_BP_FAT	positive regulation of apoptosis	90	7.30E-06	8.40E-04
	GOTERM_BP_FAT	regulation of apoptosis	149	9.80E-06	1.00E-03
	GOTERM_BP_FAT	positive regulation of programmed cell death	90	9.80E-06	1.00E-03
	GOTERM_BP_FAT	regulation of cell death	150	1.30E-05	1.30E-03
	GOTERM_BP_FAT	regulation of programmed cell death	149	1.70E-05	1.50E-03
	GOTERM_BP_FAT	induction of apoptosis	69	3.60E-05	2.90E-03
	GOTERM_BP_FAT	induction of programmed cell death	69	4.00E-05	3.10E-03
	GOTERM_BP_FAT	induction of apoptosis by extracellular signals	31	8.80E-05	6.00E-03
	GOTERM_BP_FAT	negative regulation of apoptosis	71	2.80E-04	1.60E-02
	GOTERM_BP_FAT	negative regulation of programmed cell death	71	4.30E-04	2.30E-02
	GOTERM_BP_FAT	negative regulation of cell death	71	4.70E-04	2.40E-02
	GOTERM_BP_FAT	anti-apoptosis	36	6.80E-02	5.80E-01
**Annotation Cluster 13**	**Enrichment Score: 4.17**		**Count**	**P_Value**	**Benjamini**
	GOTERM_BP_FAT	protein localization	167	6.60E-07	2.50E-04
	GOTERM_BP_FAT	intracellular transport	131	8.00E-07	2.40E-04
	SP_PIR_KEYWORDS	protein transport	95	1.80E-06	5.90E-05
	GOTERM_BP_FAT	protein transport	145	3.00E-06	4.20E-04
	GOTERM_BP_FAT	establishment of protein localization	146	3.10E-06	4.20E-04
	GOTERM_BP_FAT	intracellular protein transport	71	1.40E-03	4.90E-02
	GOTERM_BP_FAT	cellular protein localization	75	3.00E-03	8.40E-02
	GOTERM_BP_FAT	cellular macromolecule localization	75	3.60E-03	9.70E-02
	SP_PIR_KEYWORDS	transport	212	2.30E-01	6.70E-01
**Annotation Cluster 14**	**Enrichment Score: 4.13**		**Count**	**P_Value**	**Benjamini**
	SP_PIR_KEYWORDS	endoplasmic reticulum	133	2.30E-07	9.60E-06
	GOTERM_CC_FAT	endoplasmic reticulum	168	1.30E-04	3.80E-03
	GOTERM_CC_FAT	endoplasmic reticulum part	62	1.40E-02	1.60E-01
**Annotation Cluster 15**	**Enrichment Score: 4.12**		**Count**	**P_Value**	**Benjamini**
	GOTERM_BP_FAT	programmed cell death	117	2.30E-05	2.00E-03
	GOTERM_BP_FAT	apoptosis	115	3.00E-05	2.60E-03
	GOTERM_BP_FAT	cell death	132	5.30E-05	3.80E-03
	GOTERM_BP_FAT	death	132	7.30E-05	5.10E-03
	SP_PIR_KEYWORDS	Apoptosis	68	9.10E-04	1.50E-02
**Annotation Cluster 16**	**Enrichment Score: 4.12**		**Count**	**P_Value**	**Benjamini**
	UP_SEQ_FEATURE	short sequence motif:Prevents secretion from ER	21	1.10E-05	1.40E-02
	INTERPRO	Endoplasmic reticulum, targeting sequence	17	6.80E-05	5.60E-02
	GOTERM_CC_FAT	endoplasmic reticulum lumen	23	5.90E-04	1.40E-02
**Annotation Cluster 17**	**Enrichment Score: 4.11**		**Count**	**P_Value**	**Benjamini**
	SP_PIR_KEYWORDS	rna-binding	118	1.20E-10	7.30E-09
	INTERPRO	Nucleotide-binding, alpha-beta plait	52	3.70E-06	9.30E-03
	INTERPRO	RNA recognition motif, RNP-1	51	6.20E-06	7.90E-03
	SMART	RRM	51	1.90E-05	7.80E-03
	UP_SEQ_FEATURE	domain:RRM	28	8.60E-04	3.40E-01
	UP_SEQ_FEATURE	domain:RRM 2	22	8.80E-03	9.00E-01
	UP_SEQ_FEATURE	domain:RRM 1	22	8.80E-03	9.00E-01
	UP_SEQ_FEATURE	domain:RRM 3	8	4.00E-01	1.00E+00
**Annotation Cluster 18**	**Enrichment Score: 4.07**		**Count**	**P_Value**	**Benjamini**
	SP_PIR_KEYWORDS	lysosome	42	2.30E-07	1.00E-05
	GOTERM_CC_FAT	lysosome	50	5.90E-05	1.90E-03
	GOTERM_CC_FAT	lytic vacuole	50	5.90E-05	1.90E-03
	GOTERM_CC_FAT	vacuole	53	7.90E-04	1.70E-02
	KEGG_PATHWAY	Lysosome	29	6.70E-03	1.50E-01
**Annotation Cluster 19**	**Enrichment Score: 4.03**		**Count**	**P_Value**	**Benjamini**
	SP_PIR_KEYWORDS	actin-binding	55	8.70E-06	2.50E-04
	GOTERM_BP_FAT	actin cytoskeleton organization	54	1.60E-05	1.50E-03
	GOTERM_CC_FAT	actin cytoskeleton	61	3.30E-05	1.20E-03
	GOTERM_BP_FAT	actin filament-based process	55	5.10E-05	3.70E-03
	GOTERM_MF_FAT	actin binding	68	5.40E-05	1.20E-02
	GOTERM_MF_FAT	cytoskeletal protein binding	93	2.70E-04	3.10E-02
	GOTERM_BP_FAT	cytoskeleton organization	74	1.80E-02	2.80E-01

ILMN_2415267	RREB1	-0.403322364
ILMN_3301599	ZNF285A	-0.403344101
ILMN_2266595	LILRA5	-0.403391731
ILMN_1653310	ZNF580	-0.403431767
ILMN_1698148	CHD5	-0.403504114
ILMN_3242443	SNORD91B	-0.403676051
ILMN_1665970	KIAA0786	-0.403690352
ILMN_3247170	SNORD32B	-0.403796598
ILMN_3309724	MIR509-2	-0.403853321
ILMN_1666121	FKBP9	-0.40387984
ILMN_1753249	DDX10	-0.403932092
ILMN_1715416	NUP188	-0.403964757
ILMN_1690866	KBTBD10	-0.403969664
ILMN_1688971	NOL11	-0.4039802
ILMN_3239861	C10orf140	-0.404014265
ILMN_1654474	LOC647946	-0.404030321
ILMN_3187429	LOC100130071	-0.404042737
ILMN_3267319	NA	-0.404072718
ILMN_3308768	MIR302B	-0.404077099
ILMN_1694007	ADRB3	-0.404129686
ILMN_1786303	LILRA3	-0.404172836
ILMN_1872266	BM690100	-0.404185474
ILMN_1834317	AW294430	-0.404193601
ILMN_3249608	ANKLE2	-0.404204387
ILMN_1688242	C6	-0.40424357
ILMN_2328378	OSBPL3	-0.404285779
ILMN_1793894	ANAPC13	-0.404287303
ILMN_1779051	CYP2A7	-0.404336237
ILMN_1776283	LGALS12	-0.404347986
ILMN_1808907	HIST1H2BN	-0.404363208
ILMN_3235171	CCDC64B	-0.404377561
ILMN_1695687	SALL4	-0.404436407
ILMN_1796627	PKIG	-0.40463093
ILMN_1766830	RELT	-0.404642882
ILMN_1769912	OR4K14	-0.404665497
ILMN_1784367	HSPD1	-0.404668926
ILMN_1699767	PDE8A	-0.404714577
ILMN_1674587	TBC1D3C	-0.404749011
ILMN_1762531	FGF9	-0.404889035
ILMN_1900657	BM678241	-0.404905405
ILMN_3236330	DQ581928	-0.40500449
ILMN_1674719	NID1	-0.405014639
ILMN_2150187	KCTD18	-0.405032765
ILMN_1808674	CPN1	-0.405036699
ILMN_1786936	BM919382	-0.405215564
ILMN_3308861	MIR525	-0.405220568
ILMN_1728496	SYT9	-0.405235374
ILMN_2336647	NNT	-0.405246086
ILMN_1712754	NFKBIB	-0.405253738
ILMN_1797794	AK126307	-0.405264157
ILMN_1706558	TRMT61B	-0.405281547
ILMN_1657295	CPA6	-0.405305236
ILMN_1705637	ACR	-0.405319831
ILMN_1715607	CHMP4A	-0.405424093
ILMN_1674341	POTEC	-0.405475228
ILMN_1749821	MED28	-0.405529566
ILMN_3245250	CTSL1P8	-0.405576831
ILMN_1722533	KATNAL1	-0.405617181
ILMN_1728798	TEKT4P2	-0.405717756
ILMN_1693107	MLH3	-0.405770143
ILMN_1715703	DALRD3	-0.405800747
ILMN_1889942	AW450078	-0.405810082
ILMN_1658232	KDM2B	-0.405816326
ILMN_3269310	AK127969	-0.405818196
ILMN_1777024	PCDHA@	-0.40588058
ILMN_1743537	U60319	-0.405904161
ILMN_1655715	BI769462	-0.405921308
ILMN_1719471	MSH3	-0.405956289
ILMN_1812070	ABCB1	-0.406024563
ILMN_1800796	LOC100506548	-0.406049239
ILMN_1847363	LOC100288144	-0.406091118
ILMN_2120022	ARL5B	-0.406097912
ILMN_1895120	ADORA1	-0.406169421
ILMN_1685636	KCNN2	-0.406214143
ILMN_1862900	BQ447249	-0.406223415
ILMN_2216637	STK32B	-0.406277959
ILMN_3244516	C12orf51	-0.406280973
ILMN_1706901	HOXD13	-0.406310479
ILMN_1841334	CREB1	-0.406316435
ILMN_1757845	SPIRE1	-0.406331527
ILMN_1849117	AL109791	-0.406452302
ILMN_1881780	BE042871	-0.406473248
ILMN_1703229	HIATL1	-0.406506573
ILMN_1667715	NA	-0.40655125
ILMN_1741843	RD3	-0.406604417
ILMN_1670158	FAM83A	-0.40660538
ILMN_3291203	AB593110	-0.406677058
ILMN_1763670	TM2D1	-0.406693236
ILMN_1689515	CPLX3	-0.406756066
ILMN_1708363	LOXL1	-0.406841347
ILMN_1803408	KRT18	-0.406937003
ILMN_2182980	ALDH1L2	-0.406947349
ILMN_2285091	ZNF3	-0.406950351
ILMN_1768958	RASGRP1	-0.406950458
ILMN_1743357	XPNPEP2	-0.406969184
ILMN_1696590	CHST4	-0.406973918

	GOTERM_MF_FAT	purine nucleotide binding	231	2.60E-02	8.30E-01
	GOTERM_MF_FAT	purine ribonucleotide binding	219	4.50E-02	8.40E-01
	GOTERM_MF_FAT	ribonucleotide binding	219	4.50E-02	8.40E-01
	GOTERM_MF_FAT	purine nucleoside binding	192	4.80E-02	8.50E-01
	GOTERM_MF_FAT	adenyl nucleotide binding	189	5.20E-02	8.60E-01
	GOTERM_MF_FAT	nucleoside binding	192	6.10E-02	8.80E-01
	GOTERM_MF_FAT	adenyl ribonucleotide binding	177	8.40E-02	9.20E-01
	GOTERM_MF_FAT	ATP binding	175	8.50E-02	9.20E-01
	INTERPRO	Protein kinase, core	61	8.90E-02	1.00E+00
	SP_PIR_KEYWORDS	kinase	83	1.10E-01	8.50E-01
	UP_SEQ_FEATURE	binding site:ATP	63	2.60E-01	1.00E+00
	UP_SEQ_FEATURE	nucleotide phosphate-binding region:ATP	108	2.80E-01	1.00E+00
**Annotation Cluster 11**	**Enrichment Score: 1.28**		**Count**	**P_Value**	**Benjamini**
	GOTERM_BP_FAT	negative regulation of phosphate metabolic process	11	2.70E-02	9.60E-01
	GOTERM_BP_FAT	negative regulation of phosphorus metabolic process	11	2.70E-02	9.60E-01
	GOTERM_BP_FAT	negative regulation of phosphorylation	10	4.40E-02	9.80E-01
	GOTERM_BP_FAT	negative regulation of protein amino acid phosphorylation	6	2.50E-01	9.90E-01
**Annotation Cluster 12**	**Enrichment Score: 1.2**		**Count**	**P_Value**	**Benjamini**
	GOTERM_BP_FAT	branched chain family amino acid metabolic process	6	2.10E-02	9.50E-01
	GOTERM_BP_FAT	branched chain family amino acid catabolic process	4	1.00E-01	9.80E-01
	KEGG_PATHWAY	Valine, leucine and isoleucine degradation	9	1.10E-01	9.40E-01
**Annotation Cluster 13**	**Enrichment Score: 1.19**		**Count**	**P_Value**	**Benjamini**
	GOTERM_CC_FAT	autophagic vacuole	6	1.70E-02	9.20E-01
	PIR_SUPERFAMILY	PIRSF005455:microtubule-associated proteins 1 light chain 3A/3B	3	9.30E-02	1.00E+00
	INTERPRO	Light chain 3 (LC3)	3	1.60E-01	1.00E+00
**Annotation Cluster 14**	**Enrichment Score: 1.19**		**Count**	**P_Value**	**Benjamini**
	INTERPRO	Glutathione S-transferase, alpha class	5	5.80E-04	2.70E-01
	GOTERM_MF_FAT	transferase activity, transferring alkyl or aryl (other than methyl) groups	12	1.30E-02	6.40E-01
	INTERPRO	Glutathione S-transferase, C-terminal	7	1.90E-02	9.80E-01
	PIR_SUPERFAMILY	PIRSF000503:glutathione transferase	5	4.30E-02	1.00E+00
	GOTERM_MF_FAT	glutathione transferase activity	6	5.50E-02	8.70E-01
	UP_SEQ_FEATURE	domain:GST N-terminal	6	6.20E-02	1.00E+00
	INTERPRO	Glutathione S-transferase, N-terminal	6	7.60E-02	1.00E+00
	INTERPRO	Glutathione S-transferase, C-terminal-like	7	8.00E-02	1.00E+00
	INTERPRO	Glutathione S-transferase/chloride channel, C-terminal	7	9.10E-02	1.00E+00
	UP_SEQ_FEATURE	domain:GST C-terminal	7	1.30E-01	1.00E+00
	KEGG_PATHWAY	Glutathione metabolism	9	1.90E-01	9.40E-01
	KEGG_PATHWAY	Metabolism of xenobiotics by cytochrome P450	9	3.50E-01	9.50E-01
	KEGG_PATHWAY	Drug metabolism	9	3.90E-01	9.60E-01
	INTERPRO	Thioredoxin fold	12	5.10E-01	1.00E+00
**Annotation Cluster 15**	**Enrichment Score: 1.19**		**Count**	**P_Value**	**Benjamini**
	GOTERM_BP_FAT	carboxylic acid biosynthetic process	24	6.10E-02	9.80E-01
	GOTERM_BP_FAT	organic acid biosynthetic process	24	6.10E-02	9.80E-01
	GOTERM_BP_FAT	fatty acid biosynthetic process	14	7.10E-02	9.80E-01
**Annotation Cluster 16**	**Enrichment Score: 1.17**		**Count**	**P_Value**	**Benjamini**
	SP_PIR_KEYWORDS	tyrosine-protein kinase	22	5.20E-03	2.40E-01
	INTERPRO	Tyrosine protein kinase, active site	18	2.60E-02	9.90E-01
	GOTERM_MF_FAT	protein tyrosine kinase activity	27	3.00E-02	8.40E-01
	SMART	TyrKc	19	4.20E-02	9.80E-01
	GOTERM_MF_FAT	non-membrane spanning protein tyrosine kinase activity	9	6.60E-02	8.80E-01
	INTERPRO	Tyrosine protein kinase	19	7.20E-02	1.00E+00
	INTERPRO	Protein kinase, core	61	8.90E-02	1.00E+00
	SP_PIR_KEYWORDS	tyrosine-specific protein kinase	9	1.20E-01	8.40E-01
	INTERPRO	Protein kinase, ATP binding site	56	1.70E-01	1.00E+00
	UP_SEQ_FEATURE	domain:Protein kinase	57	1.70E-01	1.00E+00
	GOTERM_MF_FAT	transmembrane receptor protein tyrosine kinase activity	8	5.80E-01	1.00E+00
**Annotation Cluster 17**	**Enrichment Score: 1.16**		**Count**	**P_Value**	**Benjamini**
	GOTERM_MF_FAT	lipoic acid binding	4	3.10E-02	8.20E-01
	SP_PIR_KEYWORDS	lipoyl	3	8.80E-02	8.60E-01
	INTERPRO	2-oxo acid dehydrogenase, lipoyl-binding site	3	1.30E-01	1.00E+00
**Annotation Cluster 18**	**Enrichment Score: 1.14**		**Count**	**P_Value**	**Benjamini**
	GOTERM_CC_FAT	organelle envelope	83	2.60E-02	8.90E-01
	GOTERM_CC_FAT	mitochondrial part	80	2.70E-02	8.70E-01
	GOTERM_CC_FAT	envelope	83	2.80E-02	8.50E-01
	GOTERM_CC_FAT	organelle inner membrane	46	5.30E-02	9.00E-01
	GOTERM_CC_FAT	organelle membrane	134	6.70E-02	9.00E-01
	GOTERM_CC_FAT	mitochondrial envelope	55	9.00E-02	9.20E-01
	GOTERM_CC_FAT	nuclear envelope	29	1.10E-01	9.20E-01
	GOTERM_CC_FAT	mitochondrial membrane	50	1.50E-01	9.30E-01
	GOTERM_CC_FAT	mitochondrial inner membrane	39	1.90E-01	9.40E-01
	SP_PIR_KEYWORDS	mitochondrion inner membrane	25	2.10E-01	9.00E-01
**Annotation Cluster 19**	**Enrichment Score: 1.13**		**Count**	**P_Value**	**Benjamini**
	GOTERM_BP_FAT	embryonic morphogenesis	44	3.50E-02	9.70E-01
	GOTERM_BP_FAT	embryonic hindlimb morphogenesis	6	3.50E-02	9.70E-01
	GOTERM_BP_FAT	embryonic appendage morphogenesis	16	3.90E-02	9.70E-01
	GOTERM_BP_FAT	embryonic limb morphogenesis	16	3.90E-02	9.70E-01
	GOTERM_BP_FAT	limb morphogenesis	16	9.90E-02	9.80E-01
	GOTERM_BP_FAT	appendage morphogenesis	16	9.90E-02	9.80E-01
	GOTERM_BP_FAT	embryonic digit morphogenesis	6	1.20E-01	9.80E-01
	GOTERM_BP_FAT	limb development	16	1.30E-01	9.80E-01
	GOTERM_BP_FAT	appendage development	16	1.30E-01	9.80E-01
	GOTERM_BP_FAT	hindlimb morphogenesis	6	1.30E-01	9.80E-01
**Annotation Cluster 20**	**Enrichment Score: 1.11**		**Count**	**P_Value**	**Benjamini**
	GOTERM_BP_FAT	ion homeostasis	58	1.90E-02	9.40E-01
	GOTERM_BP_FAT	chemical homeostasis	68	4.00E-02	9.70E-01
	GOTERM_BP_FAT	cation homeostasis	41	4.10E-02	9.70E-01
	GOTERM_BP_FAT	di-, tri-valent inorganic cation homeostasis	35	4.60E-02	9.80E-01
	GOTERM_BP_FAT	cellular ion homeostasis	51	5.00E-02	9.80E-01
	GOTERM_BP_FAT	cellular chemical homeostasis	51	6.30E-02	9.80E-01
	GOTERM_BP_FAT	cellular di-, tri-valent inorganic cation homeostasis	32	8.40E-02	9.80E-01
	GOTERM_BP_FAT	calcium ion homeostasis	27	9.50E-02	9.80E-01
	GOTERM_BP_FAT	cellular homeostasis	59	1.10E-01	9.70E-01
	GOTERM_BP_FAT	cellular cation homeostasis	34	1.30E-01	9.80E-01
	GOTERM_BP_FAT	metal ion homeostasis	28	1.40E-01	9.80E-01
	GOTERM_BP_FAT	homeostatic process	90	1.40E-01	9.80E-01
	GOTERM_BP_FAT	cellular calcium ion homeostasis	25	1.60E-01	9.80E-01

ILMN_2350266	FBXO46	0.809012563
ILMN_1662799	GPSM3	0.808974076
ILMN_1673357	SLA2	0.808936701
ILMN_1735180	NCSTN	0.808844231
ILMN_1814789	UBAP2L	0.808685874
ILMN_1759700	NLGN3	0.808282349
ILMN_3236904	LOC648740	0.807906427
ILMN_2109994	MIA-RAB4B	0.807572263
ILMN_1801043	GSN	0.807523537
ILMN_2397024	SPOP	0.807300201
ILMN_1715760	HUAT	0.807291519
ILMN_2103362	ARHGAP27	0.806776335
ILMN_3238633	SDHAF2	0.806667048
ILMN_1705750	TGM2	0.806331049
ILMN_2333107	AES	0.806240321
ILMN_1741148	ALDOA	0.806239454
ILMN_2376204	LTB	0.805355045
ILMN_3275345	RPS27	0.805077537
ILMN_2408039	EEF1D	0.804486489
ILMN_2375992	SPINT1	0.804384209
ILMN_1666361	SLC23A2	0.804202607
ILMN_1770454	AGRN	0.803888579
ILMN_1699598	AP2M1	0.803789511
ILMN_1709549	PLEKHM1	0.803771165
ILMN_2125747	CORO1A	0.803651999
ILMN_2111932	SERINC2	0.803473224
ILMN_1773885	DMAP1	0.803425398
ILMN_1792528	RPS25	0.803400366
ILMN_2103841	AIP	0.803367166
ILMN_2396444	CD14	0.803001296
ILMN_2116366	RPL12	0.802712608
ILMN_1724581	EDC3	0.802294496
ILMN_3246360	RPS18	0.801990001
ILMN_2372974	SIRPA	0.801909314
ILMN_2272074	TROVE2	0.801859722
ILMN_2388507	AKT1	0.80165812
ILMN_2403555	DHX30	0.801562828
ILMN_3200830	CCT6P3	0.801458582
ILMN_2402798	AP2M1	0.801281577
ILMN_3225761	RPS14	0.800977472
ILMN_1715926	RPL32	0.800936561
ILMN_1732725	PPP6R3	0.800704411
ILMN_1791447	CXCL12	0.800532308
ILMN_1803988	MCL1	0.800442675
ILMN_1671291	EIF3I	0.800439881
ILMN_1777342	PREX1	0.800420607
ILMN_1768595	DLG4	0.80029691
ILMN_1809484	TMOD3	0.800285794
ILMN_2053178	ACTG1	0.799847542
ILMN_1768505	IL13RA1	0.799728713
ILMN_1744347	DQ895289	0.799551618
ILMN_1666385	CALM3	0.799447958
ILMN_1695404	LY6E	0.799441874
ILMN_3281563	RPL12	0.799004073
ILMN_1769702	GPAA1	0.798983762
ILMN_1701655	SLC24A6	0.798848798
ILMN_2411963	RBM39	0.79857177
ILMN_1669727	WAC	0.797953256
ILMN_2218277	RPS2	0.797791526
ILMN_1719303	P4HB	0.797743734
ILMN_1662166	PTK7	0.797561568
ILMN_2247594	RPLP1	0.797244996
ILMN_1761969	DERL2	0.797224682
ILMN_2386530	RPLP1	0.796900533
ILMN_1781276	TMCO4	0.796834571
ILMN_1724994	COL4A2	0.796800999
ILMN_2080342	LGALS9C	0.796661258
ILMN_1754195	RPL31	0.796429946
ILMN_3204210	RPL10	0.796319812
ILMN_3176090	RCC2	0.796147666
ILMN_2353202	PTK7	0.796076585
ILMN_1713086	RPL27A	0.795807754
ILMN_2160819	RPS27	0.795556984
ILMN_2398847	ARHGAP17	0.795019615
ILMN_1811921	CSRP1	0.794999755
ILMN_1707336	ARPC4	0.794946569
ILMN_3198090	FAM201B	0.794946164
ILMN_1783771	UBE2Z	0.794821422
ILMN_2412214	HUAT	0.794776221
ILMN_1732885	SLX4	0.794412295
ILMN_1696839	RPS27	0.794200605
ILMN_1670305	SERPING1	0.794188549
ILMN_1802106	APOBEC3G	0.794033059
ILMN_3266186	HDAC7	0.79362989
ILMN_3290380	XRCC6	0.793356046
ILMN_1717334	VAV1	0.793019919
ILMN_1772177	ASCC3	0.793011011
ILMN_1770742	TMEM55B	0.792839673
ILMN_1746516	RPS25	0.792595871
ILMN_2061043	CD48	0.792338043
ILMN_1656868	NPIPL3	0.792295858
ILMN_1789338	SORBS3	0.792119514
ILMN_1705464	MRPL41	0.792005986
ILMN_1654289	ELK1	0.791938088

**Annotation Cluster 20**	**Enrichment Score: 3.67**		**Count**	**P_Value**	**Benjamini**
	GOTERM_CC_FAT	Golgi apparatus part	65	4.10E-05	1.40E-03
	SP_PIR_KEYWORDS	golgi apparatus	102	1.40E-04	2.90E-03
	GOTERM_CC_FAT	Golgi apparatus	147	1.80E-03	3.40E-02
**Annotation Cluster 21**	**Enrichment Score: 3.17**		**Count**	**P_Value**	**Benjamini**
	SP_PIR_KEYWORDS	chromatin regulator	48	2.50E-05	6.30E-04
	GOTERM_BP_FAT	chromatin organization	75	2.70E-04	1.50E-02
	GOTERM_BP_FAT	chromosome organization	90	6.40E-04	2.90E-02
	GOTERM_BP_FAT	chromatin modification	55	1.40E-03	5.00E-02
	GOTERM_BP_FAT	covalent chromatin modification	26	2.30E-02	3.20E-01
**Annotation Cluster 22**	**Enrichment Score: 3.02**		**Count**	**P_Value**	**Benjamini**
	GOTERM_BP_FAT	regulation of cellular response to stress	33	1.80E-06	3.00E-04
	GOTERM_BP_FAT	regulation of stress-activated protein kinase signaling pathway	23	4.60E-05	3.50E-03
	GOTERM_BP_FAT	regulation of MAPKKK cascade	30	1.30E-04	8.40E-03
	GOTERM_BP_FAT	regulation of JNK cascade	21	1.70E-04	1.10E-02
	GOTERM_BP_FAT	activation of JUN kinase activity	12	2.30E-04	1.40E-02
	GOTERM_BP_FAT	stress-activated protein kinase signaling pathway	20	3.30E-04	1.90E-02
	GOTERM_BP_FAT	JNK cascade	19	4.10E-04	2.10E-02
	GOTERM_BP_FAT	positive regulation of kinase activity	49	8.10E-04	3.50E-02
	GOTERM_BP_FAT	MAPKKK cascade	41	8.60E-04	3.60E-02
	GOTERM_BP_FAT	positive regulation of transferase activity	50	1.10E-03	4.30E-02
	GOTERM_BP_FAT	positive regulation of JUN kinase activity	12	1.80E-03	5.90E-02
	GOTERM_BP_FAT	regulation of JUN kinase activity	13	2.00E-03	6.30E-02
	GOTERM_BP_FAT	positive regulation of protein kinase activity	46	2.10E-03	6.40E-02
	GOTERM_BP_FAT	regulation of protein amino acid phosphorylation	36	6.00E-03	1.40E-01
	GOTERM_BP_FAT	activation of MAPK activity	20	9.20E-03	1.90E-01
	GOTERM_BP_FAT	positive regulation of MAP kinase activity	23	1.30E-02	2.30E-01
	GOTERM_BP_FAT	regulation of MAP kinase activity	29	1.70E-02	2.70E-01
	GOTERM_BP_FAT	activation of protein kinase activity	21	1.10E-01	7.10E-01
**Annotation Cluster 23**	**Enrichment Score: 2.96**		**Count**	**P_Value**	**Benjamini**
	UP_SEQ_FEATURE	domain:PH	49	2.20E-04	1.80E-01
	INTERPRO	Pleckstrin homology	55	6.90E-04	2.00E-01
	SMART	PH	55	1.80E-03	1.70E-01
	INTERPRO	Pleckstrin homology-type	55	5.30E-03	5.10E-01
**Annotation Cluster 24**	**Enrichment Score: 2.77**		**Count**	**P_Value**	**Benjamini**
	GOTERM_BP_FAT	negative regulation of macromolecule metabolic process	151	1.20E-08	1.00E-05
	GOTERM_BP_FAT	negative regulation of RNA metabolic process	71	5.50E-04	2.70E-02
	GOTERM_BP_FAT	negative regulation of gene expression	93	6.30E-04	3.00E-02
	GOTERM_BP_FAT	negative regulation of transcription, DNA-dependent	68	1.50E-03	5.10E-02
	GOTERM_BP_FAT	regulation of transcription from RNA polymerase II promoter	121	4.80E-03	1.20E-01
	GOTERM_BP_FAT	negative regulation of nucleobase, nucleoside, nucleotide and nucleic acid metabolic process	89	5.00E-03	1.20E-01
	GOTERM_BP_FAT	negative regulation of nitrogen compound metabolic process	90	5.10E-03	1.30E-01
	GOTERM_BP_FAT	negative regulation of transcription	80	7.30E-03	1.60E-01
	GOTERM_BP_FAT	negative regulation of macromolecule biosynthetic process	91	1.40E-02	2.50E-01
	GOTERM_BP_FAT	negative regulation of biosynthetic process	94	1.80E-02	2.80E-01
	GOTERM_BP_FAT	negative regulation of cellular biosynthetic process	92	2.00E-02	3.00E-01
	GOTERM_BP_FAT	negative regulation of transcription from RNA polymerase II promoter	48	2.10E-02	3.10E-01
**Annotation Cluster 25**	**Enrichment Score: 2.74**		**Count**	**P_Value**	**Benjamini**
	GOTERM_BP_FAT	positive regulation of molecular function	113	2.40E-05	2.00E-03
	GOTERM_BP_FAT	positive regulation of catalytic activity	98	2.10E-04	1.30E-02
	GOTERM_BP_FAT	protein kinase cascade	72	6.30E-04	3.00E-02
	GOTERM_BP_FAT	positive regulation of kinase activity	49	8.10E-04	3.50E-02
	GOTERM_BP_FAT	MAPKKK cascade	41	8.60E-04	3.60E-02
	GOTERM_BP_FAT	positive regulation of transferase activity	50	1.10E-03	4.30E-02
	GOTERM_BP_FAT	regulation of phosphate metabolic process	88	1.50E-03	5.10E-02
	GOTERM_BP_FAT	regulation of phosphorus metabolic process	88	1.50E-03	5.10E-02
	GOTERM_BP_FAT	regulation of phosphorylation	85	1.60E-03	5.30E-02
	GOTERM_BP_FAT	regulation of kinase activity	68	1.60E-03	5.40E-02
	GOTERM_BP_FAT	regulation of transferase activity	70	1.90E-03	6.00E-02
	GOTERM_BP_FAT	positive regulation of protein kinase activity	46	2.10E-03	6.40E-02
	GOTERM_BP_FAT	regulation of protein kinase activity	65	2.70E-03	7.70E-02
	GOTERM_BP_FAT	activation of MAPK activity	20	9.20E-03	1.90E-01
	GOTERM_BP_FAT	positive regulation of MAP kinase activity	23	1.30E-02	2.30E-01
	GOTERM_BP_FAT	regulation of MAP kinase activity	29	1.70E-02	2.70E-01
	GOTERM_BP_FAT	activation of protein kinase activity	21	1.10E-01	7.10E-01
**Annotation Cluster 26**	**Enrichment Score: 2.5**		**Count**	**P_Value**	**Benjamini**
	GOTERM_BP_FAT	leukocyte differentiation	33	3.40E-04	1.80E-02
	GOTERM_BP_FAT	immune system development	57	5.80E-04	2.80E-02
	GOTERM_BP_FAT	leukocyte activation	51	7.30E-04	3.20E-02
	GOTERM_BP_FAT	cell activation	58	9.00E-04	3.70E-02
	GOTERM_BP_FAT	lymphocyte activation	43	1.20E-03	4.60E-02
	GOTERM_BP_FAT	hemopoietic or lymphoid organ development	53	1.30E-03	4.70E-02
	GOTERM_BP_FAT	hemopoiesis	49	1.30E-03	4.70E-02
	GOTERM_BP_FAT	T cell activation	30	1.70E-03	5.60E-02
	GOTERM_BP_FAT	lymphocyte differentiation	24	7.10E-03	1.60E-01
	GOTERM_BP_FAT	T cell selection	8	8.10E-03	1.70E-01
	GOTERM_BP_FAT	B cell activation	17	3.70E-02	4.20E-01
	GOTERM_BP_FAT	T cell differentiation	15	4.20E-02	4.50E-01
	GOTERM_BP_FAT	B cell differentiation	11	9.30E-02	6.60E-01
**Annotation Cluster 27**	**Enrichment Score: 2.46**		**Count**	**P_Value**	**Benjamini**
	GOTERM_BP_FAT	regulation of translation	32	1.60E-03	5.30E-02
	GOTERM_BP_FAT	posttranscriptional regulation of gene expression	44	2.20E-03	6.50E-02
	SP_PIR_KEYWORDS	translation regulation	16	1.20E-02	1.10E-01
**Annotation Cluster 28**	**Enrichment Score: 2.41**		**Count**	**P_Value**	**Benjamini**
	SP_PIR_KEYWORDS	P-loop	27	1.20E-03	1.80E-02
	SP_PIR_KEYWORDS	nucleotide binding	25	1.80E-03	2.50E-02
	SP_PIR_KEYWORDS	GTP binding	15	2.70E-02	2.00E-01
**Annotation Cluster 29**	**Enrichment Score: 2.23**		**Count**	**P_Value**	**Benjamini**
	SP_PIR_KEYWORDS	diamond-blackfan anemia	7	1.90E-05	5.10E-04
	GOTERM_BP_FAT	ribonucleoprotein complex biogenesis	40	1.10E-03	4.30E-02
	GOTERM_BP_FAT	ribosomal small subunit biogenesis	7	1.30E-03	4.80E-02
	GOTERM_BP_FAT	ribosome biogenesis	29	2.10E-03	6.50E-02
	GOTERM_BP_FAT	ncRNA metabolic process	44	1.10E-02	2.10E-01
	GOTERM_BP_FAT	rRNA processing	21	1.60E-02	2.60E-01

ILMN_1732468	HSPA4L	-0.407005857
ILMN_1725833	IFLTD1	-0.407019599
ILMN_2388585	GOPC	-0.407049292
ILMN_3271312	NA	-0.407053001
ILMN_3181507	FLJ39609	-0.407059047
ILMN_1652963	C1orf194	-0.407111688
ILMN_3229447	NA	-0.407150295
ILMN_1760088	CCKBR	-0.407153725
ILMN_1678502	BC171856	-0.407198651
ILMN_1814333	SERPINI1	-0.40725014
ILMN_1711878	ENOPH1	-0.407324313
ILMN_1690984	FGF13	-0.407327647
ILMN_2269136	AGAP3	-0.407347742
ILMN_1805766	POU6F1	-0.407359125
ILMN_2389590	PRKAR1A	-0.407405283
ILMN_1785786	AK095242	-0.407418691
ILMN_1652959	ABCD2	-0.407424713
ILMN_1657959	AY227114	-0.407469618
ILMN_2179915	ARHGEF38	-0.407487438
ILMN_1725031	LHFPL1	-0.407496853
ILMN_1912334	HYDIN	-0.407511711
ILMN_1888195	BM975708	-0.407521396
ILMN_1844068	AA813722	-0.407569769
ILMN_1788349	TMEM202	-0.407577832
ILMN_2352609	OGG1	-0.407604957
ILMN_1861380	BX088900	-0.407622862
ILMN_2285952	PCDH11X	-0.407739278
ILMN_3257004	AK124173	-0.40776638
ILMN_2228180	MSRA	-0.407778609
ILMN_1808404	RHBDF1	-0.40778088
ILMN_3252969	LOC100128300	-0.407784387
ILMN_1895825	AA779998	-0.407822822
ILMN_1910511	AF086162	-0.407835417
ILMN_1815238	MAP2K3	-0.407841949
ILMN_1805826	BIVM	-0.407850446
ILMN_1676625	SS18L1	-0.407899207
ILMN_1653182	PMS2L13	-0.407937469
ILMN_1745282	RAGE	-0.407961706
ILMN_1789692	MARK2	-0.40798923
ILMN_1835451	BX097896	-0.408059017
ILMN_2358733	TAZ	-0.408073665
ILMN_3249677	DEFB105A	-0.408078649
ILMN_1803018	KIFC2	-0.408087412
ILMN_2367743	TUBG1	-0.408120759
ILMN_2234605	AGRN	-0.408132158
ILMN_1788400	KIAA2022	-0.408179235
ILMN_2283245	C9orf84	-0.408231909
ILMN_1665365	MATR3	-0.408239832
ILMN_1672582	XAGE1A	-0.408251276
ILMN_2072568	CLDN8	-0.408302394
ILMN_3305354	SP140L	-0.408316213
ILMN_2404516	PCDHGC3	-0.408330171
ILMN_1821915	BM683706	-0.408346941
ILMN_1668927	CXorf42	-0.408438586
ILMN_1656949	SLFN14	-0.408548805
ILMN_1730351	TEX19	-0.40857797
ILMN_3246388	MED14	-0.408578597
ILMN_1851451	CD515724	-0.408628143
ILMN_1710619	C17orf73	-0.408628168
ILMN_2085659	OTOGL	-0.408641693
ILMN_1759579	ZNF91	-0.408650877
ILMN_2047240	ARSA	-0.408707673
ILMN_1710204	TNFRSF4	-0.408721908
ILMN_1829919	NA	-0.408774729
ILMN_3185563	BRD7	-0.408785939
ILMN_1662738	ACSM3	-0.408798282
ILMN_2083137	LOC284379	-0.408802917
ILMN_1722127	RAD54B	-0.408840813
ILMN_1811479	SPINK6	-0.408902295
ILMN_1885165	AB072895	-0.408911619
ILMN_1807515	CSTF2T	-0.408968392
ILMN_1797893	N4BP2L2	-0.409023885
ILMN_1729645	BBC3	-0.409061858
ILMN_1714604	LOC283710	-0.409078951
ILMN_1681402	FLJ38379	-0.409124133
ILMN_2371147	BCL7C	-0.40913884
ILMN_3182885	LOC100130542	-0.409153135
ILMN_1777031	PKLR	-0.409188448
ILMN_1703470	TBX20	-0.409194416
ILMN_1700810	HSP90AA1	-0.409223405
ILMN_1730369	GOLGA6L1	-0.409241863
ILMN_1705201	FOXC2	-0.409330505
ILMN_2131467	GNAI1	-0.409332907
ILMN_2223561	CRHBP	-0.409357068
ILMN_1653927	SNORD83A	-0.409397019
ILMN_1669083	TXNRD2	-0.409400808
ILMN_2195665	PANK3	-0.409486077
ILMN_1707649	MPDZ	-0.409503458
ILMN_2359835	SFTPB	-0.409594714
ILMN_3310075	MIR1297	-0.409635001
ILMN_1808873	C9orf110	-0.409667062
ILMN_1802834	NCRNA00087	-0.409711806
ILMN_1768202	ANKRD24	-0.409739022
ILMN_3245360	FAM201B	-0.409760466

	GOTERM_BP_FAT	cellular metal ion homeostasis	26	1.90E-01	9.90E-01
**Annotation Cluster 21**	**Enrichment Score: 1.1**		**Count**	**P_Value**	**Benjamini**
	SMART	SH3	30	4.80E-02	9.70E-01
	SP_PIR_KEYWORDS	sh3 domain	30	7.40E-02	8.50E-01
	INTERPRO	Src homology-3 domain	30	9.50E-02	1.00E+00
	UP_SEQ_FEATURE	domain:SH3	25	1.20E-01	1.00E+00
**Annotation Cluster 22**	**Enrichment Score: 1.1**		**Count**	**P_Value**	**Benjamini**
	UP_SEQ_FEATURE	repeat:WD 7	25	2.40E-02	1.00E+00
	UP_SEQ_FEATURE	repeat:WD 6	29	4.70E-02	1.00E+00
	UP_SEQ_FEATURE	repeat:WD 5	34	4.70E-02	1.00E+00
	SMART	WD40	36	5.00E-02	9.50E-01
	UP_SEQ_FEATURE	repeat:WD 4	36	5.10E-02	1.00E+00
	INTERPRO	WD40 repeat, subgroup	34	7.80E-02	1.00E+00
	UP_SEQ_FEATURE	repeat:WD 3	36	9.30E-02	1.00E+00
	INTERPRO	WD40 repeat, region	33	9.50E-02	1.00E+00
	INTERPRO	WD40/YVTN repeat-like	40	1.00E-01	1.00E+00
	INTERPRO	WD40 repeat	36	1.10E-01	1.00E+00
	INTERPRO	WD40 repeat, conserved site	37	1.20E-01	1.00E+00
	INTERPRO	WD40 repeat 2	31	1.20E-01	1.00E+00
	UP_SEQ_FEATURE	repeat:WD 1	36	1.30E-01	1.00E+00
	UP_SEQ_FEATURE	repeat:WD 2	36	1.30E-01	1.00E+00
	SP_PIR_KEYWORDS	wd repeat	36	1.30E-01	8.50E-01
**Annotation Cluster 23**	**Enrichment Score: 1.08**		**Count**	**P_Value**	**Benjamini**
	GOTERM_BP_FAT	sensory perception of sound	17	4.80E-02	9.80E-01
	GOTERM_BP_FAT	sensory perception of mechanical stimulus	17	7.60E-02	9.80E-01
	SP_PIR_KEYWORDS	deafness	15	1.60E-01	8.70E-01
**Annotation Cluster 24**	**Enrichment Score: 1.07**		**Count**	**P_Value**	**Benjamini**
	INTERPRO	Zinc finger, C3HC4 RING-type	35	2.60E-02	9.90E-01
	SMART	RING	40	4.00E-02	9.90E-01
	INTERPRO	Zinc finger, RING-type	40	9.00E-02	1.00E+00
	INTERPRO	Zinc finger, RING-type, conserved site	37	1.30E-01	1.00E+00
	UP_SEQ_FEATURE	zinc finger region:RING-type	25	3.60E-01	1.00E+00
**Annotation Cluster 25**	**Enrichment Score: 1.07**		**Count**	**P_Value**	**Benjamini**
	GOTERM_BP_FAT	striated muscle cell differentiation	18	1.00E-02	8.30E-01
	GOTERM_BP_FAT	muscle cell differentiation	22	1.60E-02	9.10E-01
	GOTERM_BP_FAT	cardiac cell differentiation	8	2.40E-02	9.50E-01
	GOTERM_BP_FAT	cardiac muscle cell differentiation	7	2.90E-02	9.60E-01
	GOTERM_BP_FAT	striated muscle tissue development	20	4.50E-02	9.80E-01
	GOTERM_BP_FAT	muscle tissue development	20	6.80E-02	9.80E-01
	GOTERM_BP_FAT	muscle organ development	29	1.30E-01	9.80E-01
	GOTERM_BP_FAT	cardiac muscle tissue development	10	1.40E-01	9.80E-01
	GOTERM_BP_FAT	skeletal muscle organ development	11	1.60E-01	9.80E-01
	GOTERM_BP_FAT	skeletal muscle tissue development	11	1.60E-01	9.80E-01
	GOTERM_BP_FAT	ventricular cardiac muscle cell differentiation	3	2.90E-01	9.90E-01
	GOTERM_BP_FAT	muscle cell development	8	3.80E-01	1.00E+00
	GOTERM_BP_FAT	heart development	22	6.90E-01	1.00E+00
**Annotation Cluster 26**	**Enrichment Score: 1.06**		**Count**	**P_Value**	**Benjamini**
	GOTERM_MF_FAT	protein serine/threonine phosphatase activity	13	2.10E-03	2.20E-01
	GOTERM_CC_FAT	protein serine/threonine phosphatase complex	11	8.30E-03	8.10E-01
	INTERPRO	Protein phosphatase 2C, manganese/magnesium aspartate binding site	5	6.60E-02	1.00E+00
	INTERPRO	Protein phosphatase 2C	5	9.80E-02	1.00E+00
	INTERPRO	Protein phosphatase 2C, N-terminal	5	1.20E-01	1.00E+00
	SMART	PP2Cc	5	1.30E-01	9.70E-01
	SMART	PP2C_SIG	5	1.30E-01	9.70E-01
	UP_SEQ_FEATURE	metal ion-binding site:Manganese 1; via carbonyl oxygen	4	1.50E-01	1.00E+00
	INTERPRO	Protein phosphatase 2C-related	5	1.60E-01	1.00E+00
	UP_SEQ_FEATURE	metal ion-binding site:Manganese 1	5	3.10E-01	1.00E+00
	UP_SEQ_FEATURE	metal ion-binding site:Manganese 2	5	3.10E-01	1.00E+00
	UP_SEQ_FEATURE	domain:PP2C-like	3	4.00E-01	1.00E+00
**Annotation Cluster 27**	**Enrichment Score: 1.05**		**Count**	**P_Value**	**Benjamini**
	SP_PIR_KEYWORDS	iron-sulfur	10	3.60E-02	7.20E-01
	GOTERM_MF_FAT	iron-sulfur cluster binding	11	3.60E-02	8.40E-01
	GOTERM_MF_FAT	metal cluster binding	11	3.60E-02	8.40E-01
	UP_SEQ_FEATURE	metal ion-binding site:Iron-sulfur (4Fe-4S)	4	2.30E-01	1.00E+00
	GOTERM_MF_FAT	4 iron, 4 sulfur cluster binding	4	5.10E-01	1.00E+00
**Annotation Cluster 28**	**Enrichment Score: 1.04**		**Count**	**P_Value**	**Benjamini**
	GOTERM_CC_FAT	nuclear inner membrane	7	3.10E-02	8.50E-01
	GOTERM_CC_FAT	intrinsic to nuclear inner membrane	3	1.30E-01	9.30E-01
	GOTERM_CC_FAT	integral to nuclear inner membrane	3	1.30E-01	9.30E-01
	GOTERM_CC_FAT	nuclear membrane part	3	1.30E-01	9.30E-01
**Annotation Cluster 29**	**Enrichment Score: 1.03**		**Count**	**P_Value**	**Benjamini**
	SP_PIR_KEYWORDS	oxidoreductase	74	3.00E-02	6.70E-01
	GOTERM_BP_FAT	oxidation reduction	79	1.00E-01	9.80E-01
	GOTERM_MF_FAT	electron carrier activity	28	2.60E-01	9.90E-01
**Annotation Cluster 30**	**Enrichment Score: 1.01**		**Count**	**P_Value**	**Benjamini**
	SMART	BAR	5	5.40E-02	9.10E-01
	INTERPRO	BAR	5	6.60E-02	1.00E+00
	UP_SEQ_FEATURE	domain:BAR	4	2.60E-01	1.00E+00
**Annotation Cluster 31**	**Enrichment Score: 0.99**		**Count**	**P_Value**	**Benjamini**
	GOTERM_MF_FAT	calcium-activated potassium channel activity	5	6.80E-02	8.80E-01
	GOTERM_MF_FAT	ion gated channel activity	5	1.00E-01	9.30E-01
	GOTERM_MF_FAT	calcium activated cation channel activity	5	1.60E-01	9.70E-01
**Annotation Cluster 32**	**Enrichment Score: 0.99**		**Count**	**P_Value**	**Benjamini**
	UP_SEQ_FEATURE	domain:RWD	4	7.90E-02	1.00E+00
	SMART	RWD	4	1.10E-01	9.70E-01
	INTERPRO	RWD	4	1.30E-01	1.00E+00
**Annotation Cluster 33**	**Enrichment Score: 0.98**		**Count**	**P_Value**	**Benjamini**
	GOTERM_BP_FAT	visual perception	31	7.40E-02	9.80E-01
	GOTERM_BP_FAT	sensory perception of light stimulus	31	7.40E-02	9.80E-01
	SP_PIR_KEYWORDS	vision	19	2.00E-01	8.90E-01
**Annotation Cluster 34**	**Enrichment Score: 0.98**		**Count**	**P_Value**	**Benjamini**
	GOTERM_BP_FAT	phosphorus metabolic process	138	2.50E-04	2.60E-01
	GOTERM_BP_FAT	phosphate metabolic process	138	2.50E-04	2.60E-01
	INTERPRO	Protein kinase, core	61	8.90E-02	1.00E+00

ILMN_2112417	PGAM1	0.791899701
ILMN_2338785	RPS14	0.791880494
ILMN_1655935	ADCY7	0.791767264
ILMN_2064655	CXorf40A	0.791749416
ILMN_1768480	VGLL4	0.79171757
ILMN_2160005	NUMA1	0.791697747
ILMN_2389013	ADRM1	0.791673118
ILMN_1789599	C1orf151	0.791277954
ILMN_1688780	S100A4	0.791254338
ILMN_3215206	SMN1	0.79122124
ILMN_1843949	BX648950	0.79108187
ILMN_1749447	RPS9	0.791023843
ILMN_1758315	SLC9A9	0.790913175
ILMN_1736481	SECISBP2	0.790882054
ILMN_1664099	GLIS3	0.790834381
ILMN_1773964	H1FX	0.790453314
ILMN_1691165	CLSPN	0.790365162
ILMN_1764721	RPL8	0.790283302
ILMN_1767256	MBTPS1	0.790115347
ILMN_2106331	H3F3A	0.789967611
ILMN_1749372	GGT5	0.789946841
ILMN_2210581	B3GAT3	0.789941542
ILMN_1776464	PARP4	0.789653482
ILMN_2133360	RPS2	0.789516596
ILMN_1661917	RPL10	0.789486302
ILMN_1669424	YBX1	0.789406382
ILMN_1788108	TXNDC5	0.789346279
ILMN_1653469	RPL12	0.789199249
ILMN_2366634	PKM2	0.789174743
ILMN_1744604	CYBA	0.788946337
ILMN_2322972	ZFYVE27	0.788643489
ILMN_1779010	MAP3K3	0.788318762
ILMN_2316278	MAGED4B	0.788125674
ILMN_3224204	PSMG4	0.787986273
ILMN_1668748	MED15	0.787745868
ILMN_2388701	ST3GAL5	0.787653518
ILMN_1746257	DAZAP1	0.787453647
ILMN_2232177	ACTN1	0.787426614
ILMN_1712357	HNRNPK	0.78732257
ILMN_2393254	CAPNS1	0.787318113
ILMN_1781626	C1S	0.787303856
ILMN_2393144	ARL6IP4	0.787242066
ILMN_3199798	RPL10	0.78720251
ILMN_3252771	RPL18A	0.787101171
ILMN_1784717	RPS19	0.787086843
ILMN_1805448	EPB41L2	0.787047333
ILMN_1756146	WDR45	0.786862934
ILMN_1725366	SLC27A5	0.786836089
ILMN_1768470	EIF4G1	0.786670408
ILMN_1658160	FAM156B	0.786436154
ILMN_2166506	XRCC6	0.786393211
ILMN_3241970	POLR2J2	0.786362849
ILMN_1756204	RPS6KA4	0.78621872
ILMN_1802089	SYMPK	0.78581435
ILMN_1695261	RPL10	0.785808679
ILMN_1701854	GNG5	0.785772334
ILMN_1734205	RASSF1	0.785591359
ILMN_2124769	YBX1	0.785561291
ILMN_2141452	RPL18A	0.785517593
ILMN_3248247	LOC349114	0.785437791
ILMN_1770247	HOXA10-HOXA9	0.78541072
ILMN_2207363	RABAC1	0.785279134
ILMN_1779185	SPECC1L	0.785021591
ILMN_2337241	RPS15A	0.784956044
ILMN_2141444	RPL18A	0.784202608
ILMN_1804476	GMPPA	0.784199465
ILMN_1682938	ARF3	0.783963454
ILMN_2410909	AKT1	0.783637457
ILMN_1778401	HLA-B	0.783588956
ILMN_2402930	H3F3A	0.78355458
ILMN_3273641	ATF6B	0.783470309
ILMN_1782543	EEF1D	0.783369687
ILMN_3264073	RPS27	0.783236259
ILMN_3307887	PRR14L	0.783152904
ILMN_1806757	MYBBP1A	0.782903961
ILMN_1658472	APH1A	0.782749522
ILMN_1802456	DCTD	0.782597135
ILMN_1715804	PITPNA	0.782564606
ILMN_2275803	LRRC45	0.782401421
ILMN_1815682	C3orf37	0.782242486
ILMN_3217172	RPS2	0.782242413
ILMN_1691487	TRAF2	0.78212567
ILMN_1802627	PSMG3	0.782060602
ILMN_3220792	RPL13AP5	0.781908832
ILMN_1660498	RPS27	0.781890402
ILMN_1775734	SH2D3C	0.781885883
ILMN_1671494	USP5	0.781625672
ILMN_1742330	PLXNB1	0.781413669
ILMN_2402341	MAPK3	0.781178848
ILMN_1754303	RPL30	0.780884178
ILMN_1782621	RPS12	0.780762002
ILMN_1758895	CTSK	0.780723974
ILMN_1751753	IDH2	0.780715539
ILMN_1709044	TGIF2	0.780626798
ILMN_1786195	PRKACA	0.780591569

	GOTERM_BP_FAT	rRNA metabolic process	21	2.40E-02	3.30E-01
	GOTERM_BP_FAT	ncRNA processing	34	4.80E-02	4.90E-01
	SP_PIR_KEYWORDS	rrna processing	7	7.60E-01	9.80E-01
**Annotation Cluster 30**	**Enrichment Score: 2.2**		**Count**	**P_Value**	**Benjamini**
	SP_PIR_KEYWORDS	phosphotransferase	50	8.90E-07	3.30E-05
	GOTERM_MF_FAT	nucleotide binding	346	7.60E-05	1.20E-02
	SP_PIR_KEYWORDS	nucleotide-binding	253	1.10E-04	2.40E-03
	SP_PIR_KEYWORDS	atp-binding	203	1.90E-04	3.60E-03
	SP_PIR_KEYWORDS	kinase	114	3.30E-04	5.90E-03
	GOTERM_MF_FAT	purine ribonucleotide binding	279	1.10E-03	9.30E-02
	GOTERM_MF_FAT	ribonucleotide binding	279	1.10E-03	9.30E-02
	GOTERM_MF_FAT	ATP binding	229	1.20E-03	9.10E-02
	GOTERM_MF_FAT	adenyl ribonucleotide binding	230	1.90E-03	1.20E-01
	GOTERM_MF_FAT	purine nucleotide binding	286	2.80E-03	1.30E-01
	GOTERM_MF_FAT	adenyl nucleotide binding	237	4.80E-03	1.80E-01
	GOTERM_MF_FAT	purine nucleoside binding	240	5.20E-03	1.80E-01
	GOTERM_MF_FAT	nucleoside binding	241	5.90E-03	1.90E-01
	GOTERM_MF_FAT	protein kinase activity	100	6.40E-03	2.00E-01
	UP_SEQ_FEATURE	binding site:ATP	86	7.10E-03	8.50E-01
	GOTERM_BP_FAT	phosphorylation	130	7.70E-03	1.70E-01
	GOTERM_BP_FAT	protein amino acid phosphorylation	109	1.30E-02	2.20E-01
	UP_SEQ_FEATURE	domain:Protein kinase	74	1.40E-02	9.40E-01
	GOTERM_MF_FAT	protein serine/threonine kinase activity	72	1.50E-02	3.50E-01
	SP_PIR_KEYWORDS	serine/threonine-protein kinase	61	1.80E-02	1.50E-01
	UP_SEQ_FEATURE	nucleotide phosphate-binding region:ATP	138	2.10E-02	9.70E-01
	SP_PIR_KEYWORDS	transferase	191	3.40E-02	2.20E-01
	INTERPRO	Protein kinase, core	74	4.40E-02	9.20E-01
	GOTERM_BP_FAT	phosphate metabolic process	146	5.90E-02	5.40E-01
	GOTERM_BP_FAT	phosphorus metabolic process	146	5.90E-02	5.40E-01
	INTERPRO	Serine/threonine protein kinase, active site	55	8.00E-02	9.60E-01
	INTERPRO	Protein kinase, ATP binding site	68	9.90E-02	9.60E-01
	INTERPRO	Serine/threonine protein kinase-related	54	1.30E-01	9.80E-01
	INTERPRO	Serine/threonine protein kinase	40	1.40E-01	9.80E-01
	UP_SEQ_FEATURE	active site:Proton acceptor	89	1.70E-01	1.00E+00
	SMART	S_TKc	40	2.10E-01	9.00E-01
**Annotation Cluster 31**	**Enrichment Score: 2.15**		**Count**	**P_Value**	**Benjamini**
	GOTERM_BP_FAT	DNA packaging	30	4.80E-04	2.40E-02
	SP_PIR_KEYWORDS	citrullination	10	6.00E-04	1.00E-02
	KEGG_PATHWAY	Systemic lupus erythematosus	28	1.10E-03	4.50E-02
	GOTERM_BP_FAT	chromatin assembly or disassembly	30	1.90E-03	6.00E-02
	GOTERM_BP_FAT	nucleosome assembly	22	2.50E-03	7.20E-02
	GOTERM_BP_FAT	chromatin assembly	22	3.90E-03	1.00E-01
	GOTERM_CC_FAT	chromatin	41	5.30E-03	7.70E-02
	INTERPRO	Histone H2A	8	6.40E-03	5.20E-01
	GOTERM_BP_FAT	protein-DNA complex assembly	22	6.70E-03	1.50E-01
	SP_PIR_KEYWORDS	chromosomal protein	29	7.60E-03	7.90E-02
	SMART	H2A	8	8.10E-03	4.30E-01
	INTERPRO	Histone core	13	8.30E-03	5.60E-01
	GOTERM_BP_FAT	nucleosome organization	22	8.70E-03	1.80E-01
	SP_PIR_KEYWORDS	nucleosome core	13	9.90E-03	9.60E-02
	GOTERM_CC_FAT	nucleosome	15	3.60E-02	2.80E-01
	PIR_SUPERFAMILY	PIRSF002048:histone H2A	6	4.20E-02	1.00E+00
	GOTERM_CC_FAT	protein-DNA complex	17	1.00E-01	5.00E-01
	INTERPRO	Histone-fold	10	2.40E-01	1.00E+00
**Annotation Cluster 32**	**Enrichment Score: 2.11**		**Count**	**P_Value**	**Benjamini**
	SP_PIR_KEYWORDS	GTPase activation	36	2.70E-04	5.00E-03
	UP_SEQ_FEATURE	domain:Rho-GAP	16	9.60E-03	9.10E-01
	INTERPRO	RhoGAP	16	1.80E-02	7.90E-01
	GOTERM_MF_FAT	GTPase activator activity	40	2.20E-02	4.10E-01
	SMART	RhoGAP	16	2.60E-02	5.90E-01
**Annotation Cluster 33**	**Enrichment Score: 2.1**		**Count**	**P_Value**	**Benjamini**
	GOTERM_CC_FAT	NuRD complex	8	6.10E-04	1.40E-02
	GOTERM_CC_FAT	chromatin remodeling complex	19	4.70E-03	7.10E-02
	GOTERM_CC_FAT	transcriptional repressor complex	12	1.60E-02	1.70E-01
	GOTERM_CC_FAT	histone deacetylase complex	10	8.80E-02	4.70E-01
**Annotation Cluster 34**	**Enrichment Score: 2.08**		**Count**	**P_Value**	**Benjamini**
	INTERPRO	Zinc finger, PHD-type	23	1.50E-03	2.60E-01
	INTERPRO	Zinc finger, PHD-finger	22	1.70E-03	2.60E-01
	SMART	PHD	23	2.60E-03	1.90E-01
	INTERPRO	Zinc finger, PHD-type, conserved site	21	1.10E-02	6.40E-01
	UP_SEQ_FEATURE	zinc finger region:PHD-type	12	4.40E-02	1.00E+00
	UP_SEQ_FEATURE	zinc finger region:PHD-type 1	8	9.60E-02	1.00E+00
**Annotation Cluster 35**	**Enrichment Score: 2.07**		**Count**	**P_Value**	**Benjamini**
	GOTERM_MF_FAT	translation factor activity, nucleic acid binding	25	1.20E-03	9.30E-02
	SP_PIR_KEYWORDS	Initiation factor	15	2.70E-03	3.50E-02
	GOTERM_BP_FAT	translational initiation	14	4.30E-03	1.10E-01
	GOTERM_MF_FAT	translation initiation factor activity	16	9.00E-03	2.50E-01
	GOTERM_CC_FAT	eukaryotic translation initiation factor 3 complex	6	4.00E-02	2.90E-01
	GOTERM_BP_FAT	regulation of translational initiation	10	8.30E-02	6.30E-01
**Annotation Cluster 36**	**Enrichment Score: 2.06**		**Count**	**P_Value**	**Benjamini**
	GOTERM_BP_FAT	transcription, DNA-dependent	55	5.90E-03	1.40E-01
	GOTERM_BP_FAT	RNA biosynthetic process	55	7.80E-03	1.70E-01
	GOTERM_BP_FAT	transcription from RNA polymerase II promoter	44	1.40E-02	2.50E-01
**Annotation Cluster 37**	**Enrichment Score: 2.05**		**Count**	**P_Value**	**Benjamini**
	GOTERM_BP_FAT	leukocyte mediated immunity	24	5.80E-04	2.80E-02
	GOTERM_BP_FAT	immune effector process	32	1.10E-03	4.30E-02
	GOTERM_BP_FAT	lymphocyte mediated immunity	20	1.40E-03	4.90E-02
	GOTERM_BP_FAT	B cell mediated immunity	15	1.20E-02	2.20E-01
	GOTERM_BP_FAT	immunoglobulin mediated immune response	14	2.10E-02	3.10E-01
	GOTERM_BP_FAT	adaptive immune response based on somatic recombination of immune receptors built from immunoglobulin superfamily domains	18	2.10E-02	3.10E-01
	GOTERM_BP_FAT	adaptive immune response	18	2.10E-02	3.10E-01
	GOTERM_BP_FAT	humoral immune response mediated by circulating immunoglobulin	6	3.90E-01	9.70E-01
**Annotation Cluster 38**	**Enrichment Score: 2.02**		**Count**	**P_Value**	**Benjamini**
	GOTERM_BP_FAT	positive regulation of immune system process	47	4.60E-03	1.20E-01
	GOTERM_BP_FAT	positive regulation of response to stimulus	46	6.40E-03	1.50E-01

ILMN_3249425	NA	-0.409768274
ILMN_1771878	SUSD5	-0.409819129
ILMN_1771538	PSCA	-0.409822459
ILMN_1805792	TRIO	-0.409832336
ILMN_1778557	CDK13	-0.409848931
ILMN_2356441	CACNG6	-0.409890521
ILMN_1745813	KIAA1279	-0.409915645
ILMN_1704302	C10orf140	-0.409982644
ILMN_1812233	KALRN	-0.410071225
ILMN_1744056	TAAR1	-0.410073148
ILMN_1674975	MANEAL	-0.410099542
ILMN_3242462	UHRF1BP1	-0.410115591
ILMN_2237746	ING3	-0.410150081
ILMN_2170353	CR613620	-0.410158071
ILMN_1782551	E2F5	-0.410291471
ILMN_1771268	BICD2	-0.410320718
ILMN_1798475	CSF2RB	-0.410331802
ILMN_1833524	CR741512	-0.410405346
ILMN_1737635	RAD1	-0.410421899
ILMN_1674050	COL8A2	-0.410447736
ILMN_2125618	SMEK3P	-0.41046641
ILMN_2380698	DSTN	-0.410473475
ILMN_2403237	CHN2	-0.410482743
ILMN_1709440	CPM	-0.410555572
ILMN_2408566	NR4A1	-0.410572275
ILMN_2352159	BBS9	-0.410576139
ILMN_2341343	AGTR1	-0.410608277
ILMN_1741592	SLC27A6	-0.410711627
ILMN_1858001	BE894306	-0.410857358
ILMN_1797318	HSPA14	-0.41086757
ILMN_1681755	DKFZp434F1622	-0.410883008
ILMN_3234959	FLJ35424	-0.410885357
ILMN_1776129	ASAH2	-0.410896677
ILMN_1774878	OTOGL	-0.410912988
ILMN_3308778	MIR488	-0.410989035
ILMN_1803033	MGST1	-0.411011975
ILMN_1673605	PRSS57	-0.411037649
ILMN_3243639	DUX2	-0.411045732
ILMN_1832208	TMEM33	-0.411119289
ILMN_1659781	CSMD3	-0.411134263
ILMN_1789294	OPRL1	-0.411163527
ILMN_1722829	HLF	-0.411183356
ILMN_1671626	DLGAP3	-0.411190821
ILMN_2386967	VPS54	-0.411256009
ILMN_1723761	ZFATAS	-0.41125828
ILMN_1810866	MRPS12	-0.411273493
ILMN_1681374	ALDOA	-0.411275026
ILMN_1812254	KIF5A	-0.411275574
ILMN_2342559	SPOCK3	-0.411286103
ILMN_2404135	RIOK3	-0.411469298
ILMN_2258977	XAGE1A	-0.41147957
ILMN_1796847	PITX2	-0.411490435
ILMN_3309299	MIR559	-0.411556847
ILMN_1652188	EPG5	-0.41159775
ILMN_1826215	LOC100128922	-0.411602097
ILMN_1695773	STK24	-0.411605734
ILMN_1695797	NCOA6	-0.411650861
ILMN_2231242	HMGB1	-0.411683661
ILMN_1740949	LRRC19	-0.411784407
ILMN_1730745	CCDC73	-0.411790568
ILMN_2390453	BCKDHB	-0.411806902
ILMN_2394193	METTL21D	-0.411876576
ILMN_1750879	TSPY3	-0.411878777
ILMN_1748899	TMED6	-0.411882633
ILMN_3243102	LOC728989	-0.41191828
ILMN_1764944	SLC6A10P	-0.411941889
ILMN_1694491	CCNG1	-0.41196735
ILMN_1849745	AI446524	-0.412009292
ILMN_1784654	RFPL3	-0.412022028
ILMN_1788895	SRD5A2	-0.412064535
ILMN_3292122	NA	-0.412077231
ILMN_1737418	ACSL6	-0.412085054
ILMN_1772207	FAM36A	-0.412100168
ILMN_3242896	DKFZp686K04236	-0.412207046
ILMN_1798615	SOHLH2	-0.412211572
ILMN_1660232	PEX5	-0.412215223
ILMN_1655905	DEFB131	-0.412233381
ILMN_1662603	NA	-0.412320857
ILMN_1689770	ARHGEF10L	-0.412336612
ILMN_1655624	LOC100130673	-0.412417419
ILMN_1653129	CSTF2	-0.412435776
ILMN_1687913	ELFN1	-0.41246446
ILMN_3273268	FAM75C1	-0.41247614
ILMN_1898826	BG564551	-0.412592797
ILMN_3201334	LOC100505502	-0.412614451
ILMN_1713613	PIAS2	-0.412670176
ILMN_2226324	BRP44L	-0.41267415
ILMN_1836193	NA	-0.412703862
ILMN_3275422	LOC648809	-0.412725089
ILMN_1662331	PDSS2	-0.412812625
ILMN_1910308	R00495	-0.41285712
ILMN_1728338	AK126539	-0.412871298
ILMN_1886639	DB027317	-0.412909083
ILMN_1856662	BM680619	-0.412938364
ILMN_1752550	NPAS3	-0.412943996

	GOTERM_BP_FAT	protein amino acid phosphorylation	82	1.10E-01	9.80E-01
	SP_PIR_KEYWORDS	kinase	83	1.10E-01	8.50E-01
	GOTERM_BP_FAT	phosphorylation	95	1.50E-01	9.80E-01
	SMART	S_TKc	32	1.60E-01	9.40E-01
	INTERPRO	Protein kinase, ATP binding site	56	1.70E-01	1.00E+00
	UP_SEQ_FEATURE	domain:Protein kinase	57	1.70E-01	1.00E+00
	GOTERM_MF_FAT	protein kinase activity	73	1.90E-01	9.80E-01
	UP_SEQ_FEATURE	active site:Proton acceptor	77	2.10E-01	1.00E+00
	SP_PIR_KEYWORDS	transferase	156	2.20E-01	9.00E-01
	UP_SEQ_FEATURE	binding site:ATP	63	2.60E-01	1.00E+00
	INTERPRO	Serine/threonine protein kinase	32	2.60E-01	1.00E+00
	UP_SEQ_FEATURE	nucleotide phosphate-binding region:ATP	108	2.80E-01	1.00E+00
	INTERPRO	Serine/threonine protein kinase-related	42	3.30E-01	1.00E+00
	GOTERM_MF_FAT	protein serine/threonine kinase activity	50	3.50E-01	9.90E-01
	SP_PIR_KEYWORDS	serine/threonine-protein kinase	41	5.20E-01	9.70E-01
	INTERPRO	Serine/threonine protein kinase, active site	38	5.60E-01	1.00E+00
**Annotation Cluster 35**	**Enrichment Score: 0.97**		**Count**	**P_Value**	**Benjamini**
	GOTERM_BP_FAT	UDP-glucose metabolic process	3	3.10E-02	9.60E-01
	GOTERM_BP_FAT	nucleotide-sugar metabolic process	4	1.80E-01	9.80E-01
	KEGG_PATHWAY	Amino sugar and nucleotide sugar metabolism	8	2.20E-01	9.50E-01
**Annotation Cluster 36**	**Enrichment Score: 0.94**		**Count**	**P_Value**	**Benjamini**
	SMART	RHOD	6	8.30E-02	9.60E-01
	INTERPRO	Rhodanese-like	6	1.00E-01	1.00E+00
	UP_SEQ_FEATURE	domain:Rhodanese	5	1.70E-01	1.00E+00
**Annotation Cluster 37**	**Enrichment Score: 0.92**		**Count**	**P_Value**	**Benjamini**
	GOTERM_BP_FAT	regulation of defense response to virus	6	4.40E-02	9.80E-01
	GOTERM_BP_FAT	regulation of defense response to virus by host	4	6.10E-02	9.80E-01
	GOTERM_BP_FAT	regulation of response to biotic stimulus	7	8.00E-02	9.80E-01
	GOTERM_BP_FAT	regulation of multi-organism process	6	2.30E-01	9.90E-01
	GOTERM_BP_FAT	regulation of immune effector process	12	5.10E-01	1.00E+00
**Annotation Cluster 38**	**Enrichment Score: 0.9**		**Count**	**P_Value**	**Benjamini**
	GOTERM_BP_FAT	phosphatidylcholine metabolic process	6	6.40E-02	9.80E-01
	GOTERM_BP_FAT	biogenic amine metabolic process	15	1.40E-01	9.80E-01
	GOTERM_BP_FAT	ethanolamine and derivative metabolic process	7	1.50E-01	9.80E-01
	GOTERM_BP_FAT	glycerophospholipid metabolic process	17	1.80E-01	9.80E-01
**Annotation Cluster 39**	**Enrichment Score: 0.9**		**Count**	**P_Value**	**Benjamini**
	GOTERM_BP_FAT	cerebellar granular layer formation	3	5.80E-02	9.80E-01
	GOTERM_BP_FAT	cerebellar granule cell differentiation	3	5.80E-02	9.80E-01
	GOTERM_BP_FAT	cerebellar granular layer morphogenesis	3	5.80E-02	9.80E-01
	GOTERM_BP_FAT	cerebellar granular layer development	3	5.80E-02	9.80E-01
	GOTERM_BP_FAT	cerebellar cortex morphogenesis	5	8.10E-02	9.80E-01
	GOTERM_BP_FAT	cerebellar cortex development	5	1.20E-01	9.80E-01
	GOTERM_BP_FAT	cerebellar cortex formation	4	1.30E-01	9.80E-01
	GOTERM_BP_FAT	cerebellum morphogenesis	5	1.30E-01	9.80E-01
	GOTERM_BP_FAT	cell differentiation in hindbrain	4	1.50E-01	9.80E-01
	GOTERM_BP_FAT	cerebellum development	6	2.10E-01	9.90E-01
	GOTERM_BP_FAT	hindbrain morphogenesis	5	2.20E-01	9.90E-01
	GOTERM_BP_FAT	metencephalon development	6	3.10E-01	9.90E-01
	GOTERM_BP_FAT	hindbrain development	8	4.50E-01	1.00E+00
**Annotation Cluster 40**	**Enrichment Score: 0.9**		**Count**	**P_Value**	**Benjamini**
	SMART	CLH	3	5.20E-02	9.30E-01
	INTERPRO	Clathrin, heavy chain/VPS, 7-fold repeat	3	5.80E-02	1.00E+00
	GOTERM_CC_FAT	Golgi-associated vesicle	6	6.50E-01	9.80E-01
**Annotation Cluster 41**	**Enrichment Score: 0.9**		**Count**	**P_Value**	**Benjamini**
	GOTERM_CC_FAT	chromatin	31	3.70E-02	8.20E-01
	GOTERM_CC_FAT	chromosome	57	1.80E-01	9.40E-01
	GOTERM_CC_FAT	chromosomal part	46	3.00E-01	9.50E-01
**Annotation Cluster 42**	**Enrichment Score: 0.89**		**Count**	**P_Value**	**Benjamini**
	SP_PIR_KEYWORDS	gtp-binding	44	7.40E-02	8.40E-01
	GOTERM_MF_FAT	guanyl ribonucleotide binding	51	7.70E-02	9.10E-01
	GOTERM_MF_FAT	guanyl nucleotide binding	51	7.70E-02	9.10E-01
	UP_SEQ_FEATURE	nucleotide phosphate-binding region:GTP	40	8.00E-02	1.00E+00
	GOTERM_MF_FAT	GTP binding	48	1.30E-01	9.50E-01
	GOTERM_MF_FAT	GTPase activity	27	2.50E-01	9.90E-01
	INTERPRO	Small GTP-binding protein	19	5.10E-01	1.00E+00
**Annotation Cluster 43**	**Enrichment Score: 0.85**		**Count**	**P_Value**	**Benjamini**
	GOTERM_BP_FAT	hormone metabolic process	18	5.40E-02	9.80E-01
	GOTERM_BP_FAT	regulation of hormone levels	22	1.20E-01	9.70E-01
	GOTERM_BP_FAT	cellular hormone metabolic process	8	4.40E-01	1.00E+00
**Annotation Cluster 44**	**Enrichment Score: 0.85**		**Count**	**P_Value**	**Benjamini**
	GOTERM_BP_FAT	endochondral bone morphogenesis	6	2.10E-02	9.50E-01
	GOTERM_BP_FAT	endochondral ossification	5	3.10E-02	9.60E-01
	GOTERM_BP_FAT	bone morphogenesis	6	5.30E-02	9.80E-01
	GOTERM_BP_FAT	bone development	16	3.20E-01	9.90E-01
	GOTERM_BP_FAT	ossification	15	3.30E-01	9.90E-01
	GOTERM_BP_FAT	skeletal system development	36	4.40E-01	1.00E+00
	GOTERM_BP_FAT	skeletal system morphogenesis	12	6.50E-01	1.00E+00
**Annotation Cluster 45**	**Enrichment Score: 0.84**		**Count**	**P_Value**	**Benjamini**
	UP_SEQ_FEATURE	domain:LEM	3	1.20E-01	1.00E+00
	SMART	LEM	3	1.50E-01	9.50E-01
	INTERPRO	Lamino-associated polypeptide 2/emerin	3	1.60E-01	1.00E+00
**Annotation Cluster 46**	**Enrichment Score: 0.84**		**Count**	**P_Value**	**Benjamini**
	UP_SEQ_FEATURE	zinc finger region:A20-type	3	1.20E-01	1.00E+00
	SMART	ZnF_A20	3	1.50E-01	9.50E-01
	INTERPRO	Zinc finger, A20-type	3	1.60E-01	1.00E+00
**Annotation Cluster 47**	**Enrichment Score: 0.83**		**Count**	**P_Value**	**Benjamini**
	KEGG_PATHWAY	Citrate cycle (TCA cycle)	8	5.10E-02	8.60E-01
	GOTERM_BP_FAT	tricarboxylic acid cycle	6	8.90E-02	9.80E-01
	GOTERM_BP_FAT	acetyl-CoA catabolic process	6	8.90E-02	9.80E-01
	GOTERM_BP_FAT	acetyl-CoA metabolic process	7	1.00E-01	9.80E-01
	GOTERM_BP_FAT	cofactor catabolic process	7	1.00E-01	9.80E-01
	GOTERM_BP_FAT	coenzyme catabolic process	6	1.30E-01	9.80E-01
	GOTERM_BP_FAT	aerobic respiration	7	1.60E-01	9.80E-01
	SP_PIR_KEYWORDS	tricarboxylic acid cycle	5	1.70E-01	8.70E-01
	GOTERM_BP_FAT	energy derivation by oxidation of organic compounds	18	3.60E-01	1.00E+00

ILMN_1676361	ARHGAP22	0.780565106
ILMN_3210538	NUDT3	0.780484257
ILMN_2101885	TUBB	0.780271925
ILMN_1687805	RPS12	0.780215735
ILMN_2392940	CEP250	0.779958173
ILMN_2368530	IL32	0.779947832
ILMN_1778226	EXTL3	0.779266044
ILMN_1703692	TUBB	0.77913614
ILMN_1770892	YY1	0.779083851
ILMN_1662174	ORMDL3	0.778951889
ILMN_2354334	ATXN2L	0.778812581
ILMN_1666078	HLA-H	0.778708791
ILMN_2141453	RPL18A	0.778578355
ILMN_2400512	PRKCSH	0.778542937
ILMN_1731233	GZMH	0.778437598
ILMN_1786347	TNPO1	0.778355095
ILMN_1726222	FLOT2	0.778227216
ILMN_1785902	C1QC	0.77808631
ILMN_2371685	UBE2E1	0.778036682
ILMN_1815656	SERINC3	0.778007044
ILMN_1772888	AX746718	0.777938563
ILMN_3247802	PRRC2B	0.777879502
ILMN_1678422	DHX58	0.777853097
ILMN_1710756	ENO1	0.777850668
ILMN_1765649	IRF3	0.77773112
ILMN_1768311	NPIPL3	0.777621226
ILMN_1750805	ARHGAP30	0.777390041
ILMN_2388155	CASP3	0.777354802
ILMN_3307693	WFDC2	0.776754166
ILMN_2177965	RPS19BP1	0.776634451
ILMN_2319414	BTF3	0.776434929
ILMN_1693352	MRPL20	0.776352647
ILMN_1652797	FAM174B	0.776220261
ILMN_2300186	DYNLL1	0.77620392
ILMN_3250389	GPAA1	0.776150121
ILMN_1758963	NADK	0.776113508
ILMN_1767509	DEF8	0.775854204
ILMN_1786197	NR2F1	0.77579258
ILMN_1810037	RUSC2	0.775662603
ILMN_1758398	GUK1	0.775375982
ILMN_1801118	SNRNP25	0.77536368
ILMN_2384544	ADAM15	0.775174467
ILMN_2330948	ARF1	0.774739379
ILMN_1711994	TCIRG1	0.77455265
ILMN_1717101	MINK1	0.774295441
ILMN_1787949	RPS15A	0.774200408
ILMN_3288161	RPS27	0.774133502
ILMN_2399503	UBN1	0.773880138
ILMN_1652768	RPS2	0.773787861
ILMN_1658411	CHD4	0.773658832
ILMN_2399036	SEPN1	0.77365667
ILMN_2215640	TUBA3D	0.773601924
ILMN_1711853	MED24	0.77351025
ILMN_1795933	CHMP7	0.773417413
ILMN_1666819	PHLDB1	0.773155687
ILMN_1792314	ACTR1A	0.773091396
ILMN_1810085	ABCF3	0.773051203
ILMN_1777296	ACTB	0.773021411
ILMN_2133675	SGSH	0.772784884
ILMN_3241462	RPS18	0.772665206
ILMN_2130180	RPL13P5	0.772606093
ILMN_2323491	NUP62	0.772599457
ILMN_1718206	KIAA0100	0.772408616
ILMN_1653498	IGSF6	0.772385033
ILMN_2309848	FXYD5	0.772374581
ILMN_2407482	ITPA	0.772308891
ILMN_2088847	OTUD5	0.771954346
ILMN_1796216	VASH1	0.771791281
ILMN_1694730	RNPEPL1	0.771732489
ILMN_1753534	RPS18	0.771545942
ILMN_2355559	PSAP	0.771455305
ILMN_2403994	RALY	0.771364184
ILMN_2038778	GAPDH	0.771202286
ILMN_1807972	MICAL1	0.771072148
ILMN_2371911	MUC1	0.771021162
ILMN_1772189	ABCD1	0.770993054
ILMN_2405592	TMEM93	0.770698287
ILMN_1664610	RPS17	0.770564138
ILMN_2112301	DRAP1	0.770554021
ILMN_1694475	GTPBP2	0.770480273
ILMN_2401978	STAT3	0.770386192
ILMN_2331062	CBFA2T2	0.77036556
ILMN_1729161	NOTCH1	0.770320625
ILMN_2208903	CD52	0.770023683
ILMN_2319994	RPL3	0.76988788
ILMN_3246962	SPECC1L	0.769831502
ILMN_3239460	OAZ2	0.769820984
ILMN_1780987	RFXANK	0.769677987
ILMN_1798819	DTNB	0.769550915
ILMN_1659800	BCL11A	0.769435097
ILMN_1730773	RPL10	0.769426962
ILMN_2392043	SPI1	0.76940794

	GOTERM_BP_FAT	positive regulation of immune response	31	7.60E-03	1.70E-01
	GOTERM_BP_FAT	activation of immune response	20	3.60E-02	4.20E-01
**Annotation Cluster 39**	**Enrichment Score: 1.95**		**Count**	**P_Value**	**Benjamini**
	INTERPRO	Tubulin/FtsZ, 2-layer sandwich domain	10	8.50E-04	2.10E-01
	UP_SEQ_FEATURE	site:Involved in polymerization	5	9.70E-04	3.50E-01
	GOTERM_BP_FAT	protein polymerization	16	1.20E-03	4.40E-02
	KEGG_PATHWAY	Pathogenic Escherichia coli infection	19	1.20E-03	4.00E-02
	INTERPRO	Tubulin	10	1.80E-03	2.60E-01
	INTERPRO	Tubulin/FtsZ, GTPase domain	10	1.80E-03	2.60E-01
	INTERPRO	Alpha tubulin	6	2.60E-03	3.40E-01
	INTERPRO	Tubulin, conserved site	9	5.40E-03	4.80E-01
	PIR_SUPERFAMILY	PIRSF002306:tubulin	9	6.00E-03	9.60E-01
	INTERPRO	Beta tubulin	3	4.10E-01	1.00E+00
	GOTERM_BP_FAT	microtubule-based movement	15	6.40E-01	1.00E+00
	INTERPRO	Beta tubulin, autoregulation binding site	3	6.80E-01	1.00E+00
	GOTERM_BP_FAT	microtubule-based process	28	9.00E-01	1.00E+00
**Annotation Cluster 40**	**Enrichment Score: 1.92**		**Count**	**P_Value**	**Benjamini**
	GOTERM_BP_FAT	'de novo' protein folding	8	2.60E-03	7.60E-02
	GOTERM_BP_FAT	'de novo' posttranslational protein folding	6	2.00E-02	3.00E-01
	GOTERM_BP_FAT	chaperone mediated protein folding requiring cofactor	5	3.30E-02	4.00E-01
**Annotation Cluster 41**	**Enrichment Score: 1.87**		**Count**	**P_Value**	**Benjamini**
	GOTERM_BP_FAT	in utero embryonic development	39	1.30E-03	4.80E-02
	GOTERM_BP_FAT	chordate embryonic development	56	4.00E-02	4.40E-01
	GOTERM_BP_FAT	embryonic development ending in birth or egg hatching	56	4.70E-02	4.80E-01
**Annotation Cluster 42**	**Enrichment Score: 1.85**		**Count**	**P_Value**	**Benjamini**
	GOTERM_BP_FAT	erythrocyte homeostasis	15	3.40E-03	9.30E-02
	GOTERM_BP_FAT	myeloid cell differentiation	22	8.70E-03	1.80E-01
	GOTERM_BP_FAT	erythrocyte differentiation	12	2.10E-02	3.10E-01
	GOTERM_BP_FAT	homeostasis of number of cells	20	6.30E-02	5.60E-01
**Annotation Cluster 43**	**Enrichment Score: 1.79**		**Count**	**P_Value**	**Benjamini**
	GOTERM_BP_FAT	positive regulation of developmental process	57	7.00E-04	3.10E-02
	GOTERM_BP_FAT	positive regulation of cell development	20	1.20E-03	4.50E-02
	GOTERM_BP_FAT	positive regulation of cell differentiation	48	1.20E-03	4.60E-02
	GOTERM_BP_FAT	positive regulation of cell projection organization	15	2.20E-03	6.60E-02
	GOTERM_BP_FAT	positive regulation of neurogenesis	17	3.30E-03	9.00E-02
	GOTERM_BP_FAT	regulation of cell morphogenesis	29	6.10E-03	1.40E-01
	GOTERM_BP_FAT	regulation of cell projection organization	21	1.10E-02	2.10E-01
	GOTERM_BP_FAT	regulation of cell development	40	1.10E-02	2.10E-01
	GOTERM_BP_FAT	positive regulation of axonogenesis	9	1.20E-02	2.20E-01
	GOTERM_BP_FAT	regulation of cell morphogenesis involved in differentiation	18	2.10E-02	3.10E-01
	GOTERM_BP_FAT	regulation of axonogenesis	14	3.20E-02	3.90E-01
	GOTERM_BP_FAT	regulation of neuron projection development	16	3.70E-02	4.20E-01
	GOTERM_BP_FAT	regulation of nervous system development	33	9.50E-02	6.70E-01
	GOTERM_BP_FAT	positive regulation of cellular component organization	31	1.10E-01	7.10E-01
	GOTERM_BP_FAT	regulation of axon extension	6	1.10E-01	7.20E-01
	GOTERM_BP_FAT	regulation of neurogenesis	28	1.40E-01	7.80E-01
	GOTERM_BP_FAT	positive regulation of developmental growth	5	1.50E-01	7.90E-01
	GOTERM_BP_FAT	regulation of neuron differentiation	23	1.50E-01	8.00E-01
**Annotation Cluster 44**	**Enrichment Score: 1.77**		**Count**	**P_Value**	**Benjamini**
	SP_PIR_KEYWORDS	elongation factor	8	9.20E-03	9.30E-02
	GOTERM_CC_FAT	eukaryotic translation elongation factor 1 complex	4	1.90E-02	1.90E-01
	GOTERM_MF_FAT	translation elongation factor activity	8	2.70E-02	4.60E-01
**Annotation Cluster 45**	**Enrichment Score: 1.77**		**Count**	**P_Value**	**Benjamini**
	PIR_SUPERFAMILY	PIRSF002034:ubiquitin	6	2.40E-04	2.20E-01
	INTERPRO	Ubiquitin supergroup	17	4.60E-04	1.50E-01
	UP_SEQ_FEATURE	cross-link:Glycyl lysine isopeptide (Gly-Lys) (interchain with K-? in acceptor proteins)	7	8.40E-04	3.60E-01
	UP_SEQ_FEATURE	binding site:Activating enzyme	5	9.70E-04	3.50E-01
	INTERPRO	Ubiquitin subgroup	7	1.10E-03	2.30E-01
	INTERPRO	Ubiquitin	15	1.20E-03	2.40E-01
	SMART	UBQ	15	1.80E-03	2.10E-01
	GOTERM_BP_FAT	regulation of skeletal muscle fiber development	10	3.30E-03	9.10E-02
	SP_PIR_KEYWORDS	protein degradation	8	4.90E-03	5.60E-02
	GOTERM_BP_FAT	regulation of striated muscle cell differentiation	11	6.40E-03	1.50E-01
	UP_SEQ_FEATURE	compositionally biased region:Lys-rich (highly basic)	4	6.40E-03	8.40E-01
	UP_SEQ_FEATURE	site:Essential for function	4	6.40E-03	8.40E-01
	UP_SEQ_FEATURE	chain:Ubiquitin	4	6.40E-03	8.40E-01
	UP_SEQ_FEATURE	chain:40S ribosomal protein S27a	4	6.40E-03	8.40E-01
	INTERPRO	Ribosomal protein S27a	4	7.30E-03	5.50E-01
	INTERPRO	Ribosomal protein L40e	4	7.30E-03	5.50E-01
	GOTERM_BP_FAT	regulation of synaptic growth at neuromuscular junction	5	7.60E-03	1.70E-01
	PIR_SUPERFAMILY	PIRSF038431:ribosomal protein S27Ae	4	8.90E-03	9.50E-01
	PIR_SUPERFAMILY	PIRSF002042:polyubiquitin 3	4	8.90E-03	9.50E-01
	GOTERM_BP_FAT	regulation of skeletal muscle tissue development	10	1.00E-02	2.00E-01
	GOTERM_BP_FAT	regulation of striated muscle tissue development	14	1.10E-02	2.10E-01
	GOTERM_BP_FAT	regulation of muscle development	14	1.30E-02	2.30E-01
	GOTERM_BP_FAT	negative regulation of striated muscle development	5	1.40E-02	2.40E-01
	SP_PIR_KEYWORDS	polyprotein	4	1.40E-02	1.30E-01
	GOTERM_BP_FAT	regulation of developmental growth	13	1.70E-02	2.70E-01
	GOTERM_BP_FAT	long-term strengthening of neuromuscular junction	4	1.90E-02	2.80E-01
	GOTERM_BP_FAT	negative regulation of muscle development	5	2.20E-02	3.10E-01
	GOTERM_BP_FAT	regulation of muscle cell differentiation	11	2.70E-02	3.60E-01
	GOTERM_BP_FAT	ER-associated protein catabolic process	7	8.60E-02	6.40E-01
	SP_PIR_KEYWORDS	pseudogene	4	1.10E-01	4.70E-01
	UP_SEQ_FEATURE	domain:Ubiquitin-like	9	1.30E-01	1.00E+00
	INTERPRO	Ubiquitin conserved site	8	1.30E-01	9.80E-01
	SP_PIR_KEYWORDS	thiolester bond	8	1.40E-01	5.20E-01
	GOTERM_BP_FAT	regulation of myoblast differentiation	4	1.70E-01	8.20E-01
	GOTERM_BP_FAT	negative regulation of myoblast differentiation	3	1.80E-01	8.40E-01
	GOTERM_BP_FAT	regulation of synapse structure and activity	6	2.00E-01	8.60E-01
	GOTERM_BP_FAT	regulation of synaptogenesis	5	2.10E-01	8.70E-01
	GOTERM_BP_FAT	positive regulation of synaptic transmission	7	2.90E-01	9.30E-01
	GOTERM_BP_FAT	regulation of synapse organization	5	3.00E-01	9.30E-01

ILMN_1670870	ALCAM	-0.412996776
ILMN_3210565	HIGD1A	-0.413118496
ILMN_3297876	VSTM2B	-0.413201209
ILMN_1714083	KLHL8	-0.413224445
ILMN_1712405	LOC100506085	-0.413228518
ILMN_1844361	CA944693	-0.413252565
ILMN_1713744	DKFZp761F2014	-0.413271021
ILMN_1776842	DKFZp451A211	-0.413292205
ILMN_1731498	GPR156	-0.4132939
ILMN_1708298	FLJ38668	-0.413310988
ILMN_1674508	MDM1	-0.41333016
ILMN_2397223	LGALS14	-0.413357704
ILMN_3244276	LOC415056	-0.413376935
ILMN_2123431	RPS6KC1	-0.413406812
ILMN_1687275	JUB	-0.413412217
ILMN_1894484	BX111257	-0.41342727
ILMN_1710522	RUNX1T1	-0.413431952
ILMN_1681651	FOLH1	-0.413474798
ILMN_1748926	TMEM209	-0.41353305
ILMN_1811472	KIF23	-0.413551308
ILMN_3249152	VSX2	-0.413576721
ILMN_1707484	GEMIN6	-0.41361845
ILMN_2158594	C10orf28	-0.413620924
ILMN_1684549	RBM39	-0.413632488
ILMN_1765986	INHBC	-0.413657481
ILMN_1713873	THSD7B	-0.413667109
ILMN_1811927	OR2W3	-0.413698385
ILMN_1657266	LOC645323	-0.413721597
ILMN_3244200	FLJ00326	-0.413812521
ILMN_1762024	TAAR9	-0.413850833
ILMN_1763419	TOP1	-0.413943351
ILMN_2361737	TRIM36	-0.414002801
ILMN_1777214	C2orf67	-0.414030989
ILMN_1791588	S71824	-0.414126197
ILMN_1729112	CHPT1	-0.414215016
ILMN_1677824	RAB4A	-0.414219792
ILMN_1664833	MRPL50	-0.414246458
ILMN_1771523	PRKACB	-0.414298387
ILMN_3227582	AK311627	-0.414334066
ILMN_1740996	CA3	-0.414412618
ILMN_3309869	MIR504	-0.41454217
ILMN_2128967	C11orf1	-0.414555872
ILMN_2202368	RBMY2EP	-0.41459383
ILMN_1854109	BC021185	-0.414601782
ILMN_1895378	CK905761	-0.414686253
ILMN_1670217	MCF2L2	-0.41476031
ILMN_3302813	MUC3A	-0.414760587
ILMN_1780041	OR4C3	-0.414778342
ILMN_3310025	MIR2117	-0.414804875
ILMN_1694027	SESN3	-0.414859569
ILMN_1873412	NA	-0.414885935
ILMN_2323979	WARS2	-0.415021658
ILMN_1662491	BI827797	-0.41504115
ILMN_1774110	CHN2	-0.415126523
ILMN_1766316	GOLGA8G	-0.415145923
ILMN_1679949	SLC25A23	-0.415273941
ILMN_3244461	LRRC16A	-0.415275096
ILMN_1760651	LOC253724	-0.415332144
ILMN_1685357	RTN2	-0.415368994
ILMN_1772677	CNOT4	-0.415373352
ILMN_1775486	SSPN	-0.415404417
ILMN_1807244	STGC3	-0.415416395
ILMN_1679527	IAPP	-0.415546722
ILMN_3237035	SNORA54	-0.415691542
ILMN_1777017	FOXK1	-0.415702304
ILMN_1768004	PDCD4	-0.415746344
ILMN_1725314	GBP3	-0.415820056
ILMN_2151048	STAG1	-0.415840446
ILMN_1788774	TYR	-0.415861118
ILMN_1659857	SNAP29	-0.415967834
ILMN_3237817	REXO1L1	-0.416047244
ILMN_1669443	LOC642384	-0.416063962
ILMN_1754234	ZMYND11	-0.416169894
ILMN_1713764	AK096649	-0.416181545
ILMN_1785387	ERMAP	-0.416210539
ILMN_1853280	DA778026	-0.416246306
ILMN_3309699	MIR29B1	-0.416333861
ILMN_1904400	AW136452	-0.416345798
ILMN_1724254	INSL6	-0.416350016
ILMN_1684184	DYRK2	-0.41640553
ILMN_1667388	OR13C8	-0.416488441
ILMN_1667213	DFFA	-0.416489707
ILMN_1740048	MYOD1	-0.416508333
ILMN_1683737	SNX32	-0.416513401
ILMN_1899842	ELK4	-0.41652031
ILMN_1690352	ADO	-0.416629553
ILMN_2347097	KLK15	-0.416639946
ILMN_1782938	SLC16A10	-0.416707259
ILMN_2369953	ARL4A	-0.416751669
ILMN_1797642	KIAA1569	-0.416754414
ILMN_2336585	CTAG2	-0.416795974
ILMN_1843478	AI494041	-0.416805589

	GOTERM_BP_FAT	cellular respiration	9	8.20E-01	1.00E+00
**Annotation Cluster 48**	**Enrichment Score: 0.81**		**Count**	**P_Value**	**Benjamini**
	GOTERM_BP_FAT	myoblast fusion	4	1.00E-01	9.80E-01
	GOTERM_BP_FAT	syncytium formation by plasma membrane fusion	4	1.30E-01	9.80E-01
	GOTERM_BP_FAT	syncytium formation	4	1.80E-01	9.80E-01
	GOTERM_BP_FAT	myotube differentiation	4	2.40E-01	9.90E-01
**Annotation Cluster 49**	**Enrichment Score: 0.81**		**Count**	**P_Value**	**Benjamini**
	GOTERM_MF_FAT	acid-thiol ligase activity	6	5.50E-02	8.70E-01
	GOTERM_MF_FAT	ligase activity, forming carbon-sulfur bonds	7	6.20E-02	8.80E-01
	GOTERM_MF_FAT	CoA-ligase activity	3	2.10E-01	9.80E-01
	SP_PIR_KEYWORDS	fatty acid metabolism	9	3.20E-01	9.20E-01
	INTERPRO	AMP-dependent synthetase and ligase	5	4.00E-01	1.00E+00
**Annotation Cluster 50**	**Enrichment Score: 0.8**		**Count**	**P_Value**	**Benjamini**
	GOTERM_CC_FAT	cytoskeleton	166	6.90E-02	8.90E-01
	GOTERM_CC_FAT	cytoskeletal part	114	1.30E-01	9.20E-01
	GOTERM_CC_FAT	intracellular non-membrane-bounded organelle	290	2.60E-01	9.50E-01
	GOTERM_CC_FAT	non-membrane-bounded organelle	290	2.60E-01	9.50E-01
**Annotation Cluster 51**	**Enrichment Score: 0.8**		**Count**	**P_Value**	**Benjamini**
	SMART	LA	3	1.50E-01	9.50E-01
	UP_SEQ_FEATURE	domain:HTH La-type RNA-binding	3	1.60E-01	1.00E+00
	INTERPRO	RNA-binding protein Lupus La	3	1.60E-01	1.00E+00
**Annotation Cluster 52**	**Enrichment Score: 0.8**		**Count**	**P_Value**	**Benjamini**
	GOTERM_BP_FAT	glucose metabolic process	22	1.30E-01	9.80E-01
	GOTERM_BP_FAT	monosaccharide metabolic process	30	1.40E-01	9.80E-01
	GOTERM_BP_FAT	hexose metabolic process	25	2.20E-01	9.90E-01
**Annotation Cluster 53**	**Enrichment Score: 0.8**		**Count**	**P_Value**	**Benjamini**
	GOTERM_CC_FAT	autophagic vacuole	6	1.70E-02	9.20E-01
	SP_PIR_KEYWORDS	autophagy	5	4.10E-01	9.40E-01
	GOTERM_BP_FAT	autophagy	5	5.80E-01	1.00E+00
**Annotation Cluster 54**	**Enrichment Score: 0.79**		**Count**	**P_Value**	**Benjamini**
	GOTERM_BP_FAT	glutathione metabolic process	7	6.90E-02	9.80E-01
	GOTERM_BP_FAT	sulfur metabolic process	18	9.90E-02	9.80E-01
	GOTERM_BP_FAT	peptide metabolic process	10	1.00E-01	9.80E-01
	GOTERM_BP_FAT	sulfur compound biosynthetic process	9	1.50E-01	9.80E-01
	GOTERM_BP_FAT	glutathione biosynthetic process	3	2.90E-01	9.90E-01
	GOTERM_BP_FAT	peptide biosynthetic process	3	5.50E-01	1.00E+00
**Annotation Cluster 55**	**Enrichment Score: 0.79**		**Count**	**P_Value**	**Benjamini**
	UP_SEQ_FEATURE	metal ion-binding site:Iron	10	4.80E-02	1.00E+00
	SMART	PP2Ac	4	1.30E-01	9.60E-01
	INTERPRO	Serine/threonine-specific protein phosphatase and bis(5-nucleosyl)-tetraphosphatase	4	1.50E-01	1.00E+00
	INTERPRO	Metallophosphoesterase	6	1.70E-01	1.00E+00
	UP_SEQ_FEATURE	metal ion-binding site:Manganese	4	6.90E-01	1.00E+00
**Annotation Cluster 56**	**Enrichment Score: 0.79**		**Count**	**P_Value**	**Benjamini**
	SMART	14_3_3	3	1.50E-01	9.50E-01
	UP_SEQ_FEATURE	site:Interaction with phosphoserine on interacting protein	3	1.60E-01	1.00E+00
	INTERPRO	14-3-3 protein	3	1.60E-01	1.00E+00
	PIR_SUPERFAMILY	PIRSF000868:14-3-3	3	1.70E-01	1.00E+00
	PIR_SUPERFAMILY	PIRSF000868:14-3-3 protein	3	1.70E-01	1.00E+00
**Annotation Cluster 57**	**Enrichment Score: 0.78**		**Count**	**P_Value**	**Benjamini**
	GOTERM_MF_FAT	inhibitory MHC class I receptor activity	3	3.20E-02	8.20E-01
	PIR_SUPERFAMILY	PIRSF016955:killer cell immunoglobulin-like receptor	3	2.50E-01	1.00E+00
	GOTERM_MF_FAT	MHC class I receptor activity	3	5.60E-01	1.00E+00
**Annotation Cluster 58**	**Enrichment Score: 0.77**		**Count**	**P_Value**	**Benjamini**
	PIR_SUPERFAMILY	PIRSF036841:ring finger protein 13	3	1.30E-01	1.00E+00
	UP_SEQ_FEATURE	zinc finger region:RING-type; atypical	7	1.40E-01	1.00E+00
	UP_SEQ_FEATURE	domain:PA	4	1.50E-01	1.00E+00
	INTERPRO	Protease-associated PA	4	3.00E-01	1.00E+00
**Annotation Cluster 59**	**Enrichment Score: 0.77**		**Count**	**P_Value**	**Benjamini**
	GOTERM_BP_FAT	centrosome cycle	6	4.40E-02	9.80E-01
	GOTERM_BP_FAT	centrosome duplication	3	2.50E-01	9.90E-01
	GOTERM_BP_FAT	centrosome organization	6	2.50E-01	9.90E-01
	GOTERM_BP_FAT	microtubule organizing center organization	6	3.10E-01	9.90E-01
**Annotation Cluster 60**	**Enrichment Score: 0.75**		**Count**	**P_Value**	**Benjamini**
	GOTERM_BP_FAT	histone ubiquitination	5	1.20E-01	9.80E-01
	GOTERM_BP_FAT	histone monoubiquitination	3	1.60E-01	9.80E-01
	GOTERM_BP_FAT	histone H2B ubiquitination	3	2.00E-01	9.90E-01
	GOTERM_BP_FAT	protein monoubiquitination	3	2.50E-01	9.90E-01
**Annotation Cluster 61**	**Enrichment Score: 0.75**		**Count**	**P_Value**	**Benjamini**
	UP_SEQ_FEATURE	domain:COS	4	1.00E-01	1.00E+00
	INTERPRO	COS domain	4	1.30E-01	1.00E+00
	UP_SEQ_FEATURE	domain:Fibronectin type-III	8	4.30E-01	1.00E+00
**Annotation Cluster 62**	**Enrichment Score: 0.75**		**Count**	**P_Value**	**Benjamini**
	GOTERM_MF_FAT	phospholipase activity	14	1.00E-01	9.30E-01
	GOTERM_BP_FAT	lipid catabolic process	25	1.00E-01	9.80E-01
	GOTERM_MF_FAT	lipase activity	15	1.80E-01	9.80E-01
	GOTERM_MF_FAT	carboxylesterase activity	14	2.20E-01	9.80E-01
	SP_PIR_KEYWORDS	lipid degradation	11	4.10E-01	9.40E-01
**Annotation Cluster 63**	**Enrichment Score: 0.75**		**Count**	**P_Value**	**Benjamini**
	BIOCARTA	Aspirin Blocks Signaling Pathway Involved in Platelet Activation	8	2.40E-03	4.30E-01
	BIOCARTA	Erk1/Erk2 Mapk Signaling pathway	8	3.00E-02	9.70E-01
	BIOCARTA	Phospholipids as signalling intermediaries	7	3.00E-02	9.00E-01
	BIOCARTA	Roles of ?-arrestin-dependent Recruitment of Src Kinases in GPCR Signaling	6	3.70E-02	8.20E-01
	BIOCARTA	Role of ?-arrestins in the activation and targeting of MAP kinases	5	5.50E-02	8.80E-01
	BIOCARTA	CCR3 signaling in Eosinophils	6	6.70E-02	8.90E-01
	SP_PIR_KEYWORDS	signal transduction	7	1.50E-01	8.70E-01
	BIOCARTA	uCalpain and friends in Cell spread	4	1.60E-01	9.80E-01
	BIOCARTA	PKC-catalyzed phosphorylation of inhibitory phosphoprotein of myosin phosphatase	4	1.60E-01	9.80E-01
	BIOCARTA	Attenuation of GPCR Signaling	3	1.70E-01	9.70E-01
	BIOCARTA	Signaling Pathway from G-Protein Families	5	1.80E-01	9.70E-01
	BIOCARTA	Erk and PI-3 Kinase Are Necessary for Collagen Binding in Corneal Epithelia	5	2.10E-01	9.80E-01
	BIOCARTA	mCalpain and friends in Cell motility	4	2.10E-01	9.70E-01
	BIOCARTA	CXCR4 Signaling Pathway	5	2.30E-01	9.80E-01

ILMN_1700628	DDX24	0.769278801
ILMN_2364376	ILK	0.76892612
ILMN_2128428	DAB2	0.76891676
ILMN_1737170	FLII	0.768501793
ILMN_1708077	DAXX	0.768491106
ILMN_1862018	ATXN7L3	0.768406132
ILMN_1810844	RARRES2	0.768397201
ILMN_1683658	FKBP1A	0.768328505
ILMN_2410986	STAT3	0.768291334
ILMN_1774828	VEZT	0.768213139
ILMN_1763884	CORO7	0.768084654
ILMN_3232447	AI880175	0.768028287
ILMN_1738675	PTPN6	0.767666604
ILMN_2349061	IRF7	0.767648353
ILMN_1736178	AEBP1	0.767636916
ILMN_1699603	MRPL12	0.767568354
ILMN_3221499	ZNF735	0.767552558
ILMN_1730084	COMT	0.767517185
ILMN_2146566	CLASRP	0.76714661
ILMN_2189424	MRPL20	0.767071215
ILMN_1761155	PABPC1	0.766818243
ILMN_2393450	INF2	0.766651565
ILMN_1733155	GIT1	0.766626893
ILMN_1726589	CD248	0.766522016
ILMN_2395711	ARRB2	0.766330425
ILMN_1778078	PTPRA	0.766328791
ILMN_3217815	RPS2	0.766213836
ILMN_2372922	PPP2R4	0.766156978
ILMN_1766275	PIK3CD	0.766095938
ILMN_2128750	PTTG1IP	0.765963971
ILMN_1677138	POLR2J2	0.765735548
ILMN_1706959	TIMM22	0.76530121
ILMN_1793371	KIAA0430	0.765231772
ILMN_2038772	RPS9	0.765190139
ILMN_2058251	VIM	0.764893517
ILMN_1767651	TECPR1	0.764813359
ILMN_1708743	NT5DC2	0.764702922
ILMN_1791211	DOK2	0.764683695
ILMN_1665384	SH3BP5L	0.764653302
ILMN_1778374	BSG	0.764583833
ILMN_3212833	RPL7A	0.764522098
ILMN_1713491	VAMP2	0.76444178
ILMN_1726392	NIN	0.7643455
ILMN_1701558	MAP1A	0.764322918
ILMN_1692473	PRMT1	0.764226141
ILMN_3192791	HNRNPM	0.764038255
ILMN_1790797	VPS28	0.763755018
ILMN_1694504	RNF220	0.763260422
ILMN_1815261	PDIA4	0.763096431
ILMN_1799062	NFKB2	0.763063543
ILMN_1686626	DDX39B	0.762974327
ILMN_2408400	NSUN5	0.762875397
ILMN_1806937	BABAM1	0.762820219
ILMN_1667893	TNS3	0.762800879
ILMN_1743655	TMED9	0.762756591
ILMN_1683415	CAMK2D	0.762638161
ILMN_1761159	ESYT1	0.762562585
ILMN_2315979	LBH	0.76207024
ILMN_1688749	RPS2	0.761808173
ILMN_1674236	HSPB1	0.76179783
ILMN_1720287	E4F1	0.761696832
ILMN_1695829	TTYH2	0.76162341
ILMN_1709439	CHMP1A	0.761609338
ILMN_1701832	RPL19	0.761446189
ILMN_1780940	PPP2R5D	0.761372969
ILMN_3270833	NA	0.761344641
ILMN_3214389	HLA-DQB1	0.76128883
ILMN_1756445	PMF1	0.761287994
ILMN_3291098	RPL29	0.761043879
ILMN_1684051	WASF2	0.760934372
ILMN_1750178	FAM21C	0.760806481
ILMN_1794740	CD151	0.76078523
ILMN_2349138	CDC42SE1	0.760631804
ILMN_1703622	PPIB	0.760487785
ILMN_2085862	SLC15A3	0.76044275
ILMN_1669456	NME3	0.760377893
ILMN_1807074	MIF	0.760324495
ILMN_3281599	RPL3	0.76031587
ILMN_1665985	SPDYE7P	0.760107584
ILMN_2333670	RNASE1	0.759898058
ILMN_2290808	RPL21	0.759883
ILMN_1790455	BCAM	0.75980712
ILMN_1685625	UCP2	0.75976714
ILMN_1773080	OAZ1	0.759708288
ILMN_1758823	SMARCB1	0.759572163
ILMN_1651878	EVC	0.75945475
ILMN_1746579	UBE2O	0.759452195
ILMN_1778668	TAGLN	0.759167503

	GOTERM_BP_FAT	negative regulation of muscle cell differentiation	4	3.50E-01	9.60E-01
	GOTERM_BP_FAT	positive regulation of transmission of nerve impulse	7	3.60E-01	9.60E-01
	GOTERM_BP_FAT	positive regulation of neurological system process	7	4.10E-01	9.70E-01
**Annotation Cluster 46**	**Enrichment Score: 1.76**		**Count**	**P_Value**	**Benjamini**
	GOTERM_CC_FAT	nuclear inner membrane	9	7.60E-03	1.00E-01
	GOTERM_CC_FAT	lamin filament	4	1.90E-02	1.90E-01
	GOTERM_CC_FAT	nuclear lamina	4	3.50E-02	2.70E-01
**Annotation Cluster 47**	**Enrichment Score: 1.76**		**Count**	**P_Value**	**Benjamini**
	GOTERM_BP_FAT	antigen processing and presentation of peptide antigen	13	1.00E-04	6.90E-03
	GOTERM_MF_FAT	MHC protein binding	12	1.20E-04	1.60E-02
	UP_SEQ_FEATURE	region of interest:Alpha-1	10	2.60E-04	1.60E-01
	UP_SEQ_FEATURE	region of interest:Alpha-2	10	2.60E-04	1.60E-01
	GOTERM_MF_FAT	MHC class I protein binding	9	3.60E-04	3.60E-02
	KEGG_PATHWAY	Viral myocarditis	23	4.60E-04	4.00E-02
	UP_SEQ_FEATURE	domain:Ig-like C1-type	14	5.90E-04	3.00E-01
	GOTERM_BP_FAT	antigen processing and presentation	23	8.60E-04	3.70E-02
	GOTERM_BP_FAT	antigen processing and presentation of exogenous antigen	8	1.00E-03	4.10E-02
	UP_SEQ_FEATURE	region of interest:Connecting peptide	13	1.30E-03	4.10E-01
	UP_SEQ_FEATURE	region of interest:Alpha-3	6	2.10E-03	5.60E-01
	GOTERM_CC_FAT	MHC class I peptide loading complex	6	3.30E-03	5.40E-02
	GOTERM_BP_FAT	antigen processing and presentation of peptide antigen via MHC class I	8	4.00E-03	1.00E-01
	GOTERM_BP_FAT	antigen processing and presentation of exogenous peptide antigen	6	9.20E-03	1.90E-01
	INTERPRO	Immunoglobulin C1-set	17	9.30E-03	5.80E-01
	KEGG_PATHWAY	Cell adhesion molecules (CAMs)	31	1.10E-02	1.40E-01
	KEGG_PATHWAY	Allograft rejection	12	1.30E-02	1.50E-01
	GOTERM_BP_FAT	antigen processing and presentation of peptide antigen via MHC class II	5	1.40E-02	2.40E-01
	GOTERM_BP_FAT	antigen processing and presentation of exogenous peptide antigen via MHC class II	5	1.40E-02	2.40E-01
	SMART	IGc1	17	1.40E-02	4.70E-01
	KEGG_PATHWAY	Antigen processing and presentation	21	1.90E-02	1.70E-01
	PIR_SUPERFAMILY	PIRSF001990:class I histocompatibility antigen	5	2.40E-02	9.90E-01
	SP_PIR_KEYWORDS	mhc i	5	2.40E-02	1.80E-01
	KEGG_PATHWAY	Intestinal immune network for IgA production	14	2.50E-02	2.00E-01
	SP_PIR_KEYWORDS	mhc ii	9	2.70E-02	2.00E-01
	GOTERM_MF_FAT	MHC class II receptor activity	7	2.70E-02	4.50E-01
	KEGG_PATHWAY	Type I diabetes mellitus	12	4.10E-02	2.60E-01
	INTERPRO	Immunoglobulin/major histocompatibility complex, conserved site	18	4.30E-02	9.20E-01
	GOTERM_CC_FAT	TAP complex	4	5.50E-02	3.50E-01
	KEGG_PATHWAY	Graft-versus-host disease	11	5.70E-02	3.30E-01
	GOTERM_MF_FAT	MHC class I receptor activity	6	5.70E-02	6.40E-01
	GOTERM_BP_FAT	antigen processing and presentation of peptide or polysaccharide antigen via MHC class II	9	6.10E-02	5.50E-01
	GOTERM_CC_FAT	MHC protein complex	13	7.10E-02	4.20E-01
	SP_PIR_KEYWORDS	transplantation antigen	3	7.40E-02	3.70E-01
	INTERPRO	MHC class I, alpha chain, C-terminal	3	8.00E-02	9.50E-01
	PIR_SUPERFAMILY	PIRSF001991:class II histocompatibility antigen	7	1.30E-01	1.00E+00
	KEGG_PATHWAY	Autoimmune thyroid disease	12	1.30E-01	5.00E-01
	KEGG_PATHWAY	Asthma	8	1.30E-01	4.90E-01
	INTERPRO	MHC class II, alpha/beta chain, N-terminal	6	1.40E-01	9.80E-01
	BBID	70.Signal_peptides_(MHC)_class_I_molecules	3	1.40E-01	9.90E-01
	GOTERM_CC_FAT	MHC class II protein complex	7	1.80E-01	6.50E-01
	INTERPRO	MHC class II, alpha chain, N-terminal	4	1.90E-01	9.90E-01
	INTERPRO	MHC class I, alpha chain, alpha1 and alpha2	6	2.50E-01	1.00E+00
	INTERPRO	MHC class I-like antigen recognition	6	2.70E-01	1.00E+00
	GOTERM_CC_FAT	MHC class I protein complex	6	3.20E-01	8.00E-01
	UP_SEQ_FEATURE	region of interest:Beta-2	3	4.40E-01	1.00E+00
	UP_SEQ_FEATURE	region of interest:Beta-1	3	4.40E-01	1.00E+00
	INTERPRO	MHC class II, beta chain, N-terminal	3	6.50E-01	1.00E+00
**Annotation Cluster 48**	**Enrichment Score: 1.7**		**Count**	**P_Value**	**Benjamini**
	UP_SEQ_FEATURE	domain:SAP	8	1.60E-02	9.40E-01
	INTERPRO	DNA-binding SAP	8	2.00E-02	8.00E-01
	SMART	SAP	8	2.50E-02	6.00E-01
**Annotation Cluster 49**	**Enrichment Score: 1.7**		**Count**	**P_Value**	**Benjamini**
	GOTERM_BP_FAT	nuclear export	16	9.60E-03	1.90E-01
	SP_PIR_KEYWORDS	translocation	17	1.40E-02	1.20E-01
	GOTERM_BP_FAT	nucleic acid transport	22	1.40E-02	2.40E-01
	GOTERM_BP_FAT	establishment of RNA localization	22	1.40E-02	2.40E-01
	GOTERM_BP_FAT	RNA transport	22	1.40E-02	2.40E-01
	GOTERM_BP_FAT	mRNA transport	20	1.70E-02	2.70E-01
	SP_PIR_KEYWORDS	nuclear pore complex	11	1.80E-02	1.50E-01
	GOTERM_BP_FAT	RNA localization	22	1.90E-02	2.90E-01
	GOTERM_BP_FAT	nucleobase, nucleoside, nucleotide and nucleic acid transport	24	2.10E-02	3.10E-01
	GOTERM_BP_FAT	mRNA export from nucleus	10	2.40E-02	3.30E-01
	SP_PIR_KEYWORDS	mRNA transport	16	2.90E-02	2.00E-01
	GOTERM_CC_FAT	nuclear pore	18	3.00E-02	2.50E-01
	GOTERM_BP_FAT	RNA export from nucleus	11	3.70E-02	4.20E-01
	GOTERM_CC_FAT	pore complex	20	4.50E-02	3.10E-01
**Annotation Cluster 50**	**Enrichment Score: 1.67**		**Count**	**P_Value**	**Benjamini**
	SP_PIR_KEYWORDS	GTPase activation	36	2.70E-04	5.00E-03
	GOTERM_BP_FAT	regulation of Ras protein signal transduction	42	6.00E-03	1.40E-01
	GOTERM_MF_FAT	small GTPase regulator activity	49	1.50E-02	3.50E-01
	GOTERM_MF_FAT	GTPase regulator activity	68	1.60E-02	3.60E-01
	GOTERM_MF_FAT	nucleoside-triphosphatase regulator activity	69	1.80E-02	3.70E-01
	GOTERM_BP_FAT	regulation of small GTPase mediated signal transduction	46	2.00E-02	3.00E-01
	GOTERM_MF_FAT	GTPase activator activity	40	2.20E-02	4.10E-01
	GOTERM_MF_FAT	enzyme activator activity	56	3.20E-02	4.90E-01
	GOTERM_BP_FAT	regulation of Rho protein signal transduction	20	5.80E-02	5.40E-01
	SP_PIR_KEYWORDS	guanine-nucleotide releasing factor	19	2.00E-01	6.30E-01
	GOTERM_MF_FAT	guanyl-nucleotide exchange factor activity	23	3.10E-01	9.70E-01
**Annotation Cluster 51**	**Enrichment Score: 1.62**		**Count**	**P_Value**	**Benjamini**

ILMN_1676118	RPL23AP7	-0.416834548
ILMN_3308758	MIR1207	-0.416880971
ILMN_3244592	SNORA68	-0.41689948
ILMN_1786984	DCTN3	-0.416938312
ILMN_3308245	MIR524	-0.41698299
ILMN_2321339	POLR3H	-0.417032617
ILMN_1784630	KBTBD11	-0.41703786
ILMN_1664994	MINPP1	-0.417089747
ILMN_2375032	BEND3	-0.417099143
ILMN_2401701	PCGF6	-0.417109376
ILMN_1675937	ANKRD9	-0.417133456
ILMN_1687123	C21orf99	-0.417215758
ILMN_1900795	AF070564	-0.417218275
ILMN_2351906	ACPT	-0.417266712
ILMN_1688234	ASS1	-0.417292674
ILMN_2238132	TTYH2	-0.417312924
ILMN_1694487	LOC645427	-0.417325944
ILMN_2231478	TSPYL3	-0.417374219
ILMN_3307972	SKCG-1	-0.417613374
ILMN_1690704	CBY3	-0.417642185
ILMN_2241775	TROVE2	-0.417645294
ILMN_1768489	TTTY20	-0.417647576
ILMN_1721564	C3orf24	-0.417676562
ILMN_1743104	RBM4B	-0.417694835
ILMN_3309339	MIR100	-0.417945079
ILMN_2127722	FAM174A	-0.417950893
ILMN_1758128	CYGB	-0.417967253
ILMN_3308465	MIR376A1	-0.417987753
ILMN_1797965	PDP2	-0.418011751
ILMN_1801467	KCNK4	-0.418029897
ILMN_1796655	SPO11	-0.418046672
ILMN_1789323	LINC00477	-0.418064903
ILMN_1770665	HERC4	-0.418333135
ILMN_1727178	C11orf92	-0.418358379
ILMN_1684108	IRX4	-0.418375933
ILMN_3238793	C9orf129	-0.418413663
ILMN_1835210	BM503295	-0.418430631
ILMN_1727567	OLIG2	-0.418464054
ILMN_1752214	BC141517	-0.418512538
ILMN_1749108	H1FOO	-0.418536795
ILMN_3292711	LOC100128437	-0.418579011
ILMN_1705423	TBC1D29	-0.418718318
ILMN_2394287	ATP6V0A4	-0.41874763
ILMN_1665996	BCL7A	-0.418761356
ILMN_1879811	BI830246	-0.41877747
ILMN_1720041	MAGEC1	-0.418887745
ILMN_3250932	SCAND2	-0.418917705
ILMN_1807579	AX747879	-0.41894642
ILMN_2412141	TAAR2	-0.418974128
ILMN_1788387	UGGT2	-0.418981141
ILMN_2402303	SSX4B	-0.419005514
ILMN_1672871	CELF5	-0.419008891
ILMN_1789751	MFSD1	-0.419033131
ILMN_3310466	MIR572	-0.419052543
ILMN_1683670	SLC10A2	-0.419095638
ILMN_3235046	LOC100132354	-0.419139648
ILMN_3237263	LOC728405	-0.419163003
ILMN_1723418	CEL	-0.419231993
ILMN_1696699	FAM184A	-0.419334626
ILMN_1763187	PAK4	-0.419450804
ILMN_2211790	ADAMTS7	-0.419468599
ILMN_1730768	LOC400456	-0.419482668
ILMN_1736103	ITPR2	-0.419488284
ILMN_1659378	TFAP2D	-0.419502913
ILMN_3309269	MIR557	-0.419597425
ILMN_1683377	MDH1B	-0.419617169
ILMN_2116605	COL19A1	-0.419685175
ILMN_1697959	SLC35B4	-0.419719641
ILMN_1689059	ZNF329	-0.419720233
ILMN_1742328	IFNA14	-0.419720608
ILMN_1667753	PPP1R1C	-0.419810248
ILMN_1800989	HTR6	-0.419831006
ILMN_1726391	MRPL39	-0.419864186
ILMN_1665585	LOC100287166	-0.419965309
ILMN_1759631	SERPINA4	-0.420052556
ILMN_3237520	C6orf127	-0.420089566
ILMN_1750062	PPARGC1A	-0.420099403
ILMN_1730504	AGPAT4	-0.420109525
ILMN_1691911	CDON	-0.420152051
ILMN_1906437	LOC100240735	-0.420360117
ILMN_1691243	NA	-0.420473688
ILMN_2415439	NAE1	-0.420525952
ILMN_2229464	LRRC37A2	-0.420607237
ILMN_1894780	H05469	-0.420641242
ILMN_1811272	AF385432	-0.420694654
ILMN_1762466	BBS4	-0.420729147
ILMN_1712075	KIAA0353	-0.42085462
ILMN_1718061	TMCO7	-0.420877648

	BIOCARTA	Thrombin signaling and protease-activated receptors	4	2.80E-01	9.80E-01
	BIOCARTA	Integrin Signaling Pathway	6	3.00E-01	9.90E-01
	BIOCARTA	Signaling of Hepatocyte Growth Factor Receptor	6	3.00E-01	9.90E-01
	BIOCARTA	Agrin in Postsynaptic Differentiation	5	3.30E-01	9.90E-01
	BIOCARTA	Angiotensin II mediated activation of JNK Pathway via Pyk2 dependent signaling	5	3.30E-01	9.90E-01
	BIOCARTA	?-arrestins in GPCR Desensitization	3	3.80E-01	9.90E-01
	BIOCARTA	Bioactive Peptide Induced Signaling Pathway	5	4.10E-01	9.90E-01
	BIOCARTA	Cadmium induces DNA synthesis and proliferation in macrophages	3	4.20E-01	9.90E-01
	KEGG_PATHWAY	VEGF signaling pathway	10	4.60E-01	9.50E-01
	BIOCARTA	fMLP induced chemokine gene expression in HMC-1 cells	4	5.20E-01	9.90E-01
	BIOCARTA	Links between Pyk2 and Map Kinases	4	5.40E-01	9.90E-01
	KEGG_PATHWAY	ErbB signaling pathway	9	7.70E-01	9.70E-01
	KEGG_PATHWAY	Fc epsilon RI signaling pathway	6	9.50E-01	9.90E-01
**Annotation Cluster 64**	**Enrichment Score: 0.74**		**Count**	**P_Value**	**Benjamini**
	GOTERM_BP_FAT	membrane lipid metabolic process	14	8.40E-02	9.80E-01
	GOTERM_BP_FAT	sphingolipid metabolic process	13	9.60E-02	9.80E-01
	GOTERM_BP_FAT	sphingoid metabolic process	8	1.90E-01	9.80E-01
	KEGG_PATHWAY	Sphingolipid metabolism	7	2.70E-01	9.50E-01
	GOTERM_BP_FAT	ceramide metabolic process	7	2.80E-01	9.90E-01
	GOTERM_MF_FAT	ceramidase activity	3	2.90E-01	9.90E-01
**Annotation Cluster 65**	**Enrichment Score: 0.74**		**Count**	**P_Value**	**Benjamini**
	GOTERM_BP_FAT	nucleoside monophosphate metabolic process	11	1.60E-01	9.80E-01
	GOTERM_BP_FAT	cGMP metabolic process	4	1.80E-01	9.80E-01
	GOTERM_BP_FAT	cyclic nucleotide metabolic process	7	2.10E-01	9.90E-01
**Annotation Cluster 66**	**Enrichment Score: 0.74**		**Count**	**P_Value**	**Benjamini**
	GOTERM_MF_FAT	di-, tri-valent inorganic cation transmembrane transporter activity	8	1.20E-01	9.50E-01
	GOTERM_BP_FAT	transition metal ion transport	12	1.30E-01	9.70E-01
	GOTERM_BP_FAT	copper ion transport	4	2.40E-01	9.90E-01
	GOTERM_MF_FAT	transition metal ion transmembrane transporter activity	5	3.30E-01	9.90E-01
**Annotation Cluster 67**	**Enrichment Score: 0.73**		**Count**	**P_Value**	**Benjamini**
	SMART	BRK	3	1.10E-01	9.60E-01
	INTERPRO	BRK domain	3	1.30E-01	1.00E+00
	INTERPRO	SNF2-related	5	4.40E-01	1.00E+00
**Annotation Cluster 68**	**Enrichment Score: 0.73**		**Count**	**P_Value**	**Benjamini**
	GOTERM_BP_FAT	cysteine metabolic process	3	1.30E-01	9.80E-01
	GOTERM_BP_FAT	serine family amino acid metabolic process	6	1.30E-01	9.80E-01
	GOTERM_BP_FAT	sulfur compound biosynthetic process	9	1.50E-01	9.80E-01
	GOTERM_BP_FAT	sulfur amino acid metabolic process	4	4.50E-01	1.00E+00
**Annotation Cluster 69**	**Enrichment Score: 0.73**		**Count**	**P_Value**	**Benjamini**
	SMART	FBOX	11	1.40E-01	9.60E-01
	INTERPRO	Cyclin-like F-box	11	2.00E-01	1.00E+00
	UP_SEQ_FEATURE	domain:F-box	11	2.20E-01	1.00E+00
**Annotation Cluster 70**	**Enrichment Score: 0.72**		**Count**	**P_Value**	**Benjamini**
	GOTERM_BP_FAT	cellular macromolecular complex subunit organization	48	6.90E-02	9.80E-01
	GOTERM_BP_FAT	cellular macromolecular complex assembly	43	7.90E-02	9.80E-01
	GOTERM_BP_FAT	macromolecular complex subunit organization	87	1.00E-01	9.80E-01
	GOTERM_BP_FAT	macromolecular complex assembly	78	2.20E-01	9.90E-01
	GOTERM_BP_FAT	protein complex assembly	53	6.10E-01	1.00E+00
	GOTERM_BP_FAT	protein complex biogenesis	53	6.10E-01	1.00E+00
**Annotation Cluster 71**	**Enrichment Score: 0.72**		**Count**	**P_Value**	**Benjamini**
	SP_PIR_KEYWORDS	cGMP-binding	4	5.90E-02	8.00E-01
	UP_SEQ_FEATURE	binding site:cGMP	3	1.20E-01	1.00E+00
	GOTERM_MF_FAT	cyclic nucleotide binding	5	2.30E-01	9.80E-01
	GOTERM_MF_FAT	cGMP binding	3	2.50E-01	9.90E-01
	GOTERM_MF_FAT	GMP binding	3	2.90E-01	9.90E-01
	SP_PIR_KEYWORDS	cGMP	4	3.80E-01	9.30E-01
**Annotation Cluster 72**	**Enrichment Score: 0.71**		**Count**	**P_Value**	**Benjamini**
	UP_SEQ_FEATURE	domain:WW 2	8	2.80E-02	1.00E+00
	UP_SEQ_FEATURE	domain:WW 1	8	2.80E-02	1.00E+00
	UP_SEQ_FEATURE	domain:WW 4	3	8.90E-02	1.00E+00
	SMART	WW	9	1.50E-01	9.50E-01
	INTERPRO	WW/Rsp5/WWP	9	1.90E-01	1.00E+00
	UP_SEQ_FEATURE	domain:WW 3	3	2.40E-01	1.00E+00
	SMART	HECTc	5	3.00E-01	9.70E-01
	UP_SEQ_FEATURE	domain:HECT	5	3.40E-01	1.00E+00
	INTERPRO	HECT	5	3.40E-01	1.00E+00
	GOTERM_BP_FAT	viral reproductive process	5	9.10E-01	1.00E+00
	GOTERM_BP_FAT	viral reproduction	5	9.50E-01	1.00E+00
**Annotation Cluster 73**	**Enrichment Score: 0.71**		**Count**	**P_Value**	**Benjamini**
	GOTERM_MF_FAT	inorganic cation transmembrane transporter activity	25	3.10E-02	8.40E-01
	GOTERM_BP_FAT	ATP biosynthetic process	16	4.70E-02	9.80E-01
	GOTERM_BP_FAT	purine nucleoside triphosphate biosynthetic process	17	5.70E-02	9.80E-01
	UP_SEQ_FEATURE	metal ion-binding site:Magnesium	19	5.90E-02	1.00E+00
	GOTERM_BP_FAT	nucleoside triphosphate biosynthetic process	17	7.10E-02	9.80E-01
	INTERPRO	ATPase, P-type phosphorylation site	8	8.10E-02	1.00E+00
	INTERPRO	ATPase, P-type, ATPase-associated region	8	8.10E-02	1.00E+00
	INTERPRO	ATPase, P-type, K/Mg/Cd/Cu/Zn/Na/Ca/Na/H-transporter	8	8.10E-02	1.00E+00
	GOTERM_MF_FAT	cation-transporting ATPase activity	8	8.30E-02	9.20E-01
	GOTERM_BP_FAT	ATP metabolic process	17	8.70E-02	9.80E-01
	GOTERM_BP_FAT	purine ribonucleoside triphosphate biosynthetic process	16	9.20E-02	9.80E-01
	GOTERM_MF_FAT	ATPase activity, coupled to transmembrane movement of ions	13	9.30E-02	9.20E-01
	GOTERM_BP_FAT	nucleoside triphosphate metabolic process	20	9.80E-02	9.80E-01
	GOTERM_BP_FAT	ribonucleoside triphosphate biosynthetic process	16	9.90E-02	9.80E-01
	GOTERM_BP_FAT	ribonucleotide biosynthetic process	19	1.00E-01	9.80E-01
	GOTERM_CC_FAT	proton-transporting two-sector ATPase complex	9	1.00E-01	9.30E-01
	UP_SEQ_FEATURE	active site:4-aspartylphosphate intermediate	8	1.10E-01	1.00E+00
	INTERPRO	Haloacid dehalogenase-like hydrolase	8	1.10E-01	1.00E+00
	GOTERM_MF_FAT	di-, tri-valent inorganic cation transmembrane transporter activity	8	1.20E-01	9.50E-01

ILMN_1761068	DKFZp434K1323	0.759113489
ILMN_1798224	KDM5C	0.758982766
ILMN_1787509	PRIC285	0.758855132
ILMN_2059549	SYK	0.758671164
ILMN_3248649	MYO15B	0.758428679
ILMN_1787844	C19orf10	0.758341435
ILMN_1756992	MUC1	0.758300389
ILMN_2349696	CNIH2	0.758288412
ILMN_1733799	FAM195B	0.758241702
ILMN_3241446	ODF3B	0.758165545
ILMN_2306189	MAGED1	0.757975406
ILMN_1711909	EDEM2	0.757927036
ILMN_1804530	ARPC1B	0.757887861
ILMN_1796755	ITGB5	0.757816349
ILMN_1815023	PIM1	0.757678386
ILMN_2340131	MAPK10	0.757009895
ILMN_1768973	HIST2H2AC	0.757009777
ILMN_1712755	LRRC41	0.757003905
ILMN_1800317	WNT5A	0.756873715
ILMN_3246766	NPIPL3	0.75638424
ILMN_1651378	AUP1	0.756316929
ILMN_1812403	BCAP31	0.755498888
ILMN_1724990	C3orf75	0.755437233
ILMN_1810467	PPP2R1A	0.755279821
ILMN_2347234	PRMT1	0.755278681
ILMN_1802706	IDH3G	0.755274672
ILMN_1766222	LARP4B	0.75493525
ILMN_1721977	NAA10	0.754893092
ILMN_1776678	GIMAP7	0.754823661
ILMN_1782292	LAMP1	0.754713332
ILMN_1804444	PDIA2	0.754706698
ILMN_2198893	MAP2K2	0.754674051
ILMN_2114422	NOD1	0.754510983
ILMN_1672650	PKM2	0.754413707
ILMN_1806017	PSME1	0.754264268
ILMN_1681679	TSPO	0.754116091
ILMN_1689004	TNFRSF12A	0.754010636
ILMN_2399392	SIL1	0.75399574
ILMN_1775522	MAGED1	0.753701943
ILMN_1663080	LFNG	0.753468439
ILMN_2206746	BGN	0.753434943
ILMN_1677484	SNAPC4	0.753429177
ILMN_1708881	RAB20	0.753419869
ILMN_1761844	ZCCHC17	0.753381459
ILMN_1771862	TXNDC11	0.75318273
ILMN_1659771	RPL13AP3	0.753133201
ILMN_2410772	KEAP1	0.753047726
ILMN_2358914	SLC35C2	0.752972172
ILMN_1730670	FSTL3	0.752941615
ILMN_1688959	CD27	0.752626113
ILMN_1772487	SUGP2	0.752475521
ILMN_1733811	JUP	0.752368762
ILMN_3209220	NA	0.752230086
ILMN_3242900	HIST2H2AA4	0.7521808
ILMN_1778144	SLC48A1	0.752087304
ILMN_1886655	TCRVB	0.75191266
ILMN_1724059	GAS2L1	0.751852332
ILMN_1796336	MAP1S	0.7517574
ILMN_1751871	STK11	0.751726854
ILMN_1687867	KRT8	0.751721383
ILMN_1751034	ITPRIPL2	0.751612733
ILMN_1677505	CCL21	0.751533959
ILMN_1751368	HNRNPD	0.751460303
ILMN_1726434	UNC45A	0.751393254
ILMN_1765446	EMP3	0.751337857
ILMN_1814194	TCF4	0.751320118
ILMN_1760143	ADRM1	0.751127854
ILMN_1654563	EFNB1	0.750694334
ILMN_1691942	CCNI	0.750507801
ILMN_1789909	TBC1D9B	0.750358301
ILMN_2324672	USF2	0.750054682
ILMN_1744113	SCNM1	0.750003388
ILMN_1755115	RPL23	0.749719903
ILMN_1716876	MED27	0.749601114
ILMN_1707810	RPS5	0.749530339
ILMN_1658283	RPL17	0.749480923
ILMN_1741398	TTI1	0.749391475
ILMN_1773849	ATP6V0C	0.749354628
ILMN_1663113	TTLL12	0.74928163
ILMN_1752269	ACSS1	0.749241449

	GOTERM_BP_FAT	angiogenesis	31	1.00E-02	2.00E-01
	GOTERM_BP_FAT	blood vessel morphogenesis	39	2.70E-02	3.60E-01
	GOTERM_BP_FAT	blood vessel development	44	3.00E-02	3.80E-01
	GOTERM_BP_FAT	vasculature development	44	4.20E-02	4.50E-01
**Annotation Cluster 52**	**Enrichment Score: 1.61**		**Count**	**P_Value**	**Benjamini**
	GOTERM_MF_FAT	SH3 domain binding	24	2.00E-03	1.20E-01
	SP_PIR_KEYWORDS	sh3-binding	13	5.80E-02	3.20E-01
	UP_SEQ_FEATURE	short sequence motif:SH3-binding	11	1.20E-01	1.00E+00
**Annotation Cluster 53**	**Enrichment Score: 1.61**		**Count**	**P_Value**	**Benjamini**
	UP_SEQ_FEATURE	domain:BAR	7	1.20E-02	9.10E-01
	INTERPRO	BAR	6	3.20E-02	9.10E-01
	SMART	BAR	6	3.80E-02	6.80E-01
**Annotation Cluster 54**	**Enrichment Score: 1.61**		**Count**	**P_Value**	**Benjamini**
	INTERPRO	Ets	10	7.50E-03	5.50E-01
	SMART	ETS	10	1.00E-02	4.00E-01
	UP_SEQ_FEATURE	DNA-binding region:ETS	9	1.20E-02	9.10E-01
	INTERPRO	Winged helix repressor DNA-binding	26	4.10E-01	1.00E+00
**Annotation Cluster 55**	**Enrichment Score: 1.57**		**Count**	**P_Value**	**Benjamini**
	GOTERM_MF_FAT	intramolecular oxidoreductase activity	13	2.10E-03	1.20E-01
	UP_SEQ_FEATURE	domain:Thioredoxin 1	6	2.10E-03	5.60E-01
	UP_SEQ_FEATURE	domain:Thioredoxin 2	6	2.10E-03	5.60E-01
	INTERPRO	Disulphide isomerase	5	3.10E-03	3.70E-01
	INTERPRO	Thioredoxin-like	12	3.70E-03	4.10E-01
	GOTERM_MF_FAT	intramolecular oxidoreductase activity, interconverting keto- and enol-groups	6	5.00E-03	1.80E-01
	GOTERM_MF_FAT	intramolecular oxidoreductase activity, transposing S-S bonds	5	2.00E-02	3.80E-01
	GOTERM_MF_FAT	protein disulfide isomerase activity	5	2.00E-02	3.80E-01
	INTERPRO	Thioredoxin-like subdomain	5	2.80E-02	8.80E-01
	INTERPRO	Thioredoxin fold	20	4.70E-02	9.20E-01
	PIR_SUPERFAMILY	PIRSF001487:protein disulfide-isomerase	4	5.70E-02	1.00E+00
	SP_PIR_KEYWORDS	Redox-active center	10	5.90E-02	3.20E-01
	GOTERM_BP_FAT	cell redox homeostasis	14	6.50E-02	5.70E-01
	UP_SEQ_FEATURE	site:Lowers pKa of C-terminal Cys of first active site	3	7.40E-02	1.00E+00
	UP_SEQ_FEATURE	site:Lowers pKa of C-terminal Cys of second active site	3	7.40E-02	1.00E+00
	INTERPRO	Protein disulphide isomerase	3	8.00E-02	9.50E-01
	INTERPRO	Thioredoxin domain	7	1.20E-01	9.70E-01
	UP_SEQ_FEATURE	site:Contributes to redox potential value	3	2.00E-01	1.00E+00
	INTERPRO	Thioredoxin, conserved site	6	2.20E-01	1.00E+00
	SP_PIR_KEYWORDS	redox-active disulfide	3	2.50E-01	6.90E-01
**Annotation Cluster 56**	**Enrichment Score: 1.53**		**Count**	**P_Value**	**Benjamini**
	INTERPRO	Ribosomal protein L7Ae/L30e/S12e/Gadd45	6	7.60E-03	5.40E-01
	INTERPRO	Ribosomal protein L7Ae conserved site	3	4.40E-02	9.10E-01
	INTERPRO	Ribosomal protein L7A/RS6 family	3	4.40E-02	9.10E-01
	PIR_SUPERFAMILY	PIRSF002165:ribosomal protein, L7Ab/L7Aa/L7Ae/L8e types/H/ACA ribonucleoprotein complex, NHP2 subunit	3	5.00E-02	1.00E+00
**Annotation Cluster 57**	**Enrichment Score: 1.5**		**Count**	**P_Value**	**Benjamini**
	UP_SEQ_FEATURE	zinc finger region:C4-type	18	2.50E-03	5.70E-01
	GOTERM_BP_FAT	regulation of ARF protein signal transduction	13	9.70E-03	1.90E-01
	UP_SEQ_FEATURE	domain:Arf-GAP	9	1.50E-02	9.40E-01
	INTERPRO	Arf GTPase activating protein	9	1.90E-02	8.00E-01
	GOTERM_BP_FAT	regulation of ARF GTPase activity	9	2.40E-02	3.30E-01
	SMART	ArfGap	9	2.50E-02	6.40E-01
	GOTERM_MF_FAT	ARF GTPase activator activity	9	2.60E-02	4.50E-01
	GOTERM_BP_FAT	regulation of GTPase activity	22	1.30E-01	7.50E-01
	GOTERM_BP_FAT	regulation of Ras GTPase activity	19	1.40E-01	7.70E-01
	GOTERM_BP_FAT	regulation of hydrolase activity	46	4.90E-01	9.90E-01
**Annotation Cluster 58**	**Enrichment Score: 1.48**		**Count**	**P_Value**	**Benjamini**
	GOTERM_BP_FAT	positive regulation of adaptive immune response	12	1.40E-03	4.90E-02
	GOTERM_BP_FAT	positive regulation of adaptive immune response based on somatic recombination of immune receptors built from immunoglobulin superfamily domains	11	3.80E-03	1.00E-01
	GOTERM_BP_FAT	positive regulation of T cell mediated immunity	7	9.00E-03	1.90E-01
	GOTERM_BP_FAT	positive regulation of immune effector process	13	9.70E-03	1.90E-01
	GOTERM_BP_FAT	regulation of immune effector process	23	1.10E-02	2.10E-01
	GOTERM_BP_FAT	regulation of adaptive immune response	15	1.20E-02	2.20E-01
	GOTERM_BP_FAT	regulation of adaptive immune response based on somatic recombination of immune receptors built from immunoglobulin superfamily domains	14	2.40E-02	3.30E-01
	GOTERM_BP_FAT	regulation of leukocyte mediated immunity	15	2.50E-02	3.40E-01
	GOTERM_BP_FAT	positive regulation of leukocyte mediated immunity	10	2.80E-02	3.70E-01
	GOTERM_BP_FAT	positive regulation of lymphocyte mediated immunity	10	2.80E-02	3.70E-01
	GOTERM_BP_FAT	regulation of lymphocyte mediated immunity	13	4.60E-02	4.80E-01
	GOTERM_BP_FAT	regulation of T cell mediated immunity	7	4.90E-02	4.90E-01
	GOTERM_BP_FAT	positive regulation of defense response	14	1.60E-01	8.10E-01
	GOTERM_BP_FAT	regulation of production of molecular mediator of immune response	9	1.60E-01	8.20E-01
	GOTERM_BP_FAT	positive regulation of B cell mediated immunity	4	1.70E-01	8.20E-01
	GOTERM_BP_FAT	positive regulation of immunoglobulin mediated immune response	4	1.70E-01	8.20E-01
	GOTERM_BP_FAT	regulation of immunoglobulin mediated immune response	6	2.00E-01	8.60E-01
	GOTERM_BP_FAT	regulation of B cell mediated immunity	6	2.00E-01	8.60E-01
**Annotation Cluster 59**	**Enrichment Score: 1.48**		**Count**	**P_Value**	**Benjamini**
	GOTERM_MF_FAT	identical protein binding	107	3.20E-03	1.40E-01
	GOTERM_MF_FAT	protein dimerization activity	82	8.10E-02	7.40E-01
	GOTERM_MF_FAT	protein homodimerization activity	51	1.40E-01	8.60E-01
**Annotation Cluster 60**	**Enrichment Score: 1.48**		**Count**	**P_Value**	**Benjamini**
	UP_SEQ_FEATURE	domain:Saposin B-type	4	1.50E-02	9.40E-01
	INTERPRO	Saposin B	4	4.70E-02	9.20E-01

ILMN_1859744	AI939525	-0.420896686
ILMN_1768922	ZNF790	-0.420930585
ILMN_3184595	NA	-0.420965069
ILMN_1663118	RASGEF1C	-0.421011499
ILMN_1695108	RSPO2	-0.421060597
ILMN_1720274	C2orf53	-0.421112694
ILMN_1672332	LOC646862	-0.42116657
ILMN_3187758	SELK	-0.421219375
ILMN_1681598	PCDHGC3	-0.421302283
ILMN_1757281	GPRIN1	-0.421305186
ILMN_1684730	POM121L2	-0.421363856
ILMN_3284078	KRTAP16-1	-0.421364059
ILMN_2073969	ABHD13	-0.421375678
ILMN_1655117	WDR19	-0.421396867
ILMN_2292875	EFTUD1	-0.421516702
ILMN_2351272	DZIP1	-0.421518591
ILMN_1669208	TSPO2	-0.421558258
ILMN_3186439	NA	-0.421562546
ILMN_1696407	SRSF2	-0.421579849
ILMN_1750295	ACCN5	-0.421635823
ILMN_1671355	SLC7A5P1	-0.421637868
ILMN_1705177	DNAH14	-0.421685041
ILMN_1900154	BU536494	-0.421703168
ILMN_1832920	LOC115110	-0.421741186
ILMN_3305933	LOC730668	-0.421755043
ILMN_3274006	BC153822	-0.421773219
ILMN_1713892	C4orf34	-0.421782949
ILMN_1893367	BX094716	-0.421802516
ILMN_3308210	MIR195	-0.421824797
ILMN_1691076	TEX11	-0.421833839
ILMN_1739045	RWDD4	-0.421861943
ILMN_1726603	ATP5I	-0.421882992
ILMN_1743319	PAFAH1B2	-0.421915585
ILMN_1722559	NEUROD6	-0.421983046
ILMN_1660440	FAM108B1	-0.422013607
ILMN_3289764	SP9	-0.422021701
ILMN_2186482	TICAM2	-0.422054381
ILMN_1703894	SLX1A	-0.422156804
ILMN_1679318	ARSJ	-0.42220231
ILMN_1850240	AW262671	-0.422249302
ILMN_1793578	ZFP37	-0.422309081
ILMN_1774432	DTD1	-0.422310182
ILMN_1731181	TEX2	-0.422384271
ILMN_1749375	TNFAIP8	-0.422386726
ILMN_2192694	EIF3M	-0.422432994
ILMN_1702889	OR2AK2	-0.422448739
ILMN_2103133	PDZD11	-0.422459642
ILMN_2412564	NCBP2	-0.422476376
ILMN_1762629	ANO9	-0.422531645
ILMN_1773650	LRRN3	-0.422551983
ILMN_3234513	CCDC72	-0.422560887
ILMN_1658083	ABT1	-0.422598472
ILMN_1777476	SPDYC	-0.422601802
ILMN_1685104	C9orf6	-0.422604869
ILMN_1685413	ALG8	-0.422615704
ILMN_2210129	PRIM1	-0.422629432
ILMN_3262321	PATE4	-0.422634123
ILMN_1814327	AGTR1	-0.422688808
ILMN_1677608	NEUROD4	-0.422700162
ILMN_1766678	NKX2-6	-0.422777814
ILMN_2046751	PGLYRP3	-0.422825113
ILMN_1660970	ADAMTS12	-0.422826522
ILMN_3185130	LOC100128770	-0.422834069
ILMN_1667058	CLU	-0.422855399
ILMN_1713315	AMAC1L2	-0.422968903
ILMN_2156250	SOCS3	-0.423003588
ILMN_1751389	LOC100288974	-0.423129224
ILMN_1668270	ZDHHC18	-0.423145952
ILMN_2233099	SSRP1	-0.423168489
ILMN_1778673	GOLGA7	-0.423298303
ILMN_1781390	FGF17	-0.423437624
ILMN_1787658	MTMR7	-0.423600942
ILMN_1754658	CDRT1	-0.423640229
ILMN_1770676	CAPRIN1	-0.423673102
ILMN_1656902	HECTD3	-0.423703093
ILMN_2132379	TDGF1	-0.423794741
ILMN_1662482	LPPR4	-0.423823411
ILMN_1822180	AW188305	-0.42394859
ILMN_1676998	SCN2B	-0.423996991
ILMN_3244779	LOC100286979	-0.424107709

	GOTERM_BP_FAT	purine nucleotide metabolic process	26	1.30E-01	9.70E-01
	GOTERM_BP_FAT	nucleotide biosynthetic process	26	1.30E-01	9.70E-01
	GOTERM_MF_FAT	calcium ion transmembrane transporter activity	4	1.30E-01	9.50E-01
	GOTERM_MF_FAT	hydrolase activity, acting on acid anhydrides, catalyzing transmembrane movement of substances	17	1.30E-01	9.60E-01
	GOTERM_BP_FAT	ribonucleotide metabolic process	21	1.50E-01	9.80E-01
	GOTERM_BP_FAT	purine nucleoside triphosphate metabolic process	18	1.50E-01	9.80E-01
	GOTERM_BP_FAT	purine nucleotide biosynthetic process	21	1.50E-01	9.80E-01
	GOTERM_MF_FAT	P-P-bond-hydrolysis-driven transmembrane transporter activity	18	1.50E-01	9.70E-01
	GOTERM_MF_FAT	primary active transmembrane transporter activity	18	1.50E-01	9.70E-01
	GOTERM_CC_FAT	proton-transporting V-type ATPase complex	5	1.60E-01	9.30E-01
	GOTERM_BP_FAT	nucleobase, nucleoside and nucleotide biosynthetic process	26	1.70E-01	9.80E-01
	GOTERM_BP_FAT	nucleobase, nucleoside, nucleotide and nucleic acid biosynthetic process	26	1.70E-01	9.80E-01
	GOTERM_BP_FAT	purine ribonucleotide biosynthetic process	17	1.70E-01	9.80E-01
	GOTERM_BP_FAT	purine ribonucleoside triphosphate metabolic process	17	1.70E-01	9.80E-01
	GOTERM_BP_FAT	nitrogen compound biosynthetic process	41	1.80E-01	9.80E-01
	INTERPRO	ATPase, P-type, phospholipid-translocating, flippase	4	1.80E-01	1.00E+00
	GOTERM_BP_FAT	ribonucleoside triphosphate metabolic process	17	1.80E-01	9.80E-01
	GOTERM_MF_FAT	ATPase activity, coupled to transmembrane movement of substances	16	1.90E-01	9.80E-01
	GOTERM_MF_FAT	phospholipid transporter activity	6	1.90E-01	9.80E-01
	GOTERM_MF_FAT	ATPase activity, coupled to movement of substances	16	1.90E-01	9.80E-01
	GOTERM_MF_FAT	ATPase activity, coupled to transmembrane movement of ions, phosphorylative mechanism	9	2.00E-01	9.80E-01
	GOTERM_BP_FAT	purine ribonucleotide metabolic process	19	2.10E-01	9.90E-01
	GOTERM_MF_FAT	phospholipid-translocating ATPase activity	4	2.10E-01	9.80E-01
	GOTERM_MF_FAT	aminophospholipid transporter activity	4	2.10E-01	9.80E-01
	GOTERM_MF_FAT	monovalent inorganic cation transmembrane transporter activity	15	2.20E-01	9.80E-01
	GOTERM_BP_FAT	hydrogen transport	10	2.20E-01	9.90E-01
	GOTERM_MF_FAT	lipid transporter activity	10	2.30E-01	9.80E-01
	SP_PIR_KEYWORDS	Hydrogen ion transport	8	2.30E-01	9.00E-01
	GOTERM_BP_FAT	ATP synthesis coupled proton transport	7	2.50E-01	9.90E-01
	GOTERM_BP_FAT	energy coupled proton transport, down electrochemical gradient	7	2.50E-01	9.90E-01
	GOTERM_MF_FAT	calcium-transporting ATPase activity	3	2.50E-01	9.90E-01
	GOTERM_CC_FAT	proton-transporting V-type ATPase, V1 domain	3	2.50E-01	9.50E-01
	GOTERM_MF_FAT	hydrogen ion transmembrane transporter activity	13	2.50E-01	9.90E-01
	GOTERM_BP_FAT	ion transmembrane transport	8	2.60E-01	9.90E-01
	GOTERM_BP_FAT	phospholipid transport	6	2.70E-01	9.90E-01
	KEGG_PATHWAY	Vibrio cholerae infection	9	2.80E-01	9.40E-01
	GOTERM_CC_FAT	vacuolar proton-transporting V-type ATPase complex	3	2.90E-01	9.60E-01
	GOTERM_MF_FAT	ATPase activity	40	3.10E-01	9.90E-01
	GOTERM_BP_FAT	proton transport	9	3.20E-01	9.90E-01
	GOTERM_CC_FAT	mitochondrial proton-transporting ATP synthase complex	4	3.40E-01	9.50E-01
	GOTERM_MF_FAT	ATPase activity, coupled	32	3.80E-01	9.90E-01
	GOTERM_CC_FAT	proton-transporting ATP synthase complex	4	4.00E-01	9.60E-01
	SP_PIR_KEYWORDS	cf(0)	3	4.00E-01	9.40E-01
	GOTERM_MF_FAT	proton-transporting ATPase activity, rotational mechanism	4	4.50E-01	1.00E+00
	INTERPRO	ATPase, P-type cation-transporter, C-terminal	3	4.80E-01	1.00E+00
	GOTERM_CC_FAT	proton-transporting two-sector ATPase complex, proton-transporting domain	4	4.80E-01	9.70E-01
	GOTERM_CC_FAT	proton-transporting ATP synthase complex, coupling factor F(o)	3	4.90E-01	9.70E-01
	PIR_SUPERFAMILY	PIRSF001297:Na(+)/K(+)-transporting ATPase alpha chain	3	4.90E-01	1.00E+00
	GOTERM_MF_FAT	hydrogen ion transporting ATP synthase activity, rotational mechanism	3	5.20E-01	1.00E+00
	INTERPRO	ATPase, P-type cation-transporter, N-terminal	3	5.50E-01	1.00E+00
	GOTERM_CC_FAT	proton-transporting two-sector ATPase complex, catalytic domain	3	5.90E-01	9.70E-01
	GOTERM_BP_FAT	lipid transport	15	6.80E-01	1.00E+00
	GOTERM_BP_FAT	lipid localization	16	7.00E-01	1.00E+00
	KEGG_PATHWAY	Epithelial cell signaling in Helicobacter pylori infection	7	7.90E-01	9.70E-01
	KEGG_PATHWAY	Oxidative phosphorylation	13	8.00E-01	9.70E-01
	GOTERM_BP_FAT	oxidative phosphorylation	8	9.10E-01	1.00E+00
**Annotation Cluster 74**	**Enrichment Score: 0.7**		**Count**	**P_Value**	**Benjamini**
	GOTERM_MF_FAT	CoA hydrolase activity	5	1.00E-01	9.30E-01
	GOTERM_MF_FAT	acyl-CoA thioesterase activity	4	1.30E-01	9.50E-01
	GOTERM_MF_FAT	palmitoyl-CoA hydrolase activity	3	2.10E-01	9.80E-01
	SP_PIR_KEYWORDS	serine esterase	4	5.70E-01	9.70E-01
**Annotation Cluster 75**	**Enrichment Score: 0.7**		**Count**	**P_Value**	**Benjamini**
	UP_SEQ_FEATURE	site:Cleavage; by anthrax lethal factor	3	8.90E-02	1.00E+00
	BBID	100.MAPK_signaling_cascades	5	9.70E-02	9.90E-01
	KEGG_PATHWAY	Fc epsilon RI signaling pathway	6	9.50E-01	9.90E-01
**Annotation Cluster 76**	**Enrichment Score: 0.69**		**Count**	**P_Value**	**Benjamini**
	INTERPRO	Adenylate kinase, subfamily	3	3.10E-02	9.90E-01
	INTERPRO	Adenylate kinase, zinc-finger lid region	3	3.10E-02	9.90E-01
	PIR_SUPERFAMILY	PIRSF000734:adenylate kinase	3	9.30E-02	1.00E+00
	GOTERM_MF_FAT	adenylate kinase activity	3	2.10E-01	9.80E-01
	INTERPRO	Adenylate kinase	3	2.50E-01	1.00E+00
	UP_SEQ_FEATURE	nucleotide phosphate-binding region:AMP	3	4.00E-01	1.00E+00
	UP_SEQ_FEATURE	binding site:AMP	3	4.00E-01	1.00E+00
	GOTERM_MF_FAT	nucleotide kinase activity	4	4.50E-01	1.00E+00
	GOTERM_MF_FAT	phosphotransferase activity, phosphate group as acceptor	5	4.80E-01	1.00E+00
	GOTERM_MF_FAT	nucleobase, nucleoside, nucleotide kinase activity	5	6.90E-01	1.00E+00
**Annotation Cluster 77**	**Enrichment Score: 0.69**		**Count**	**P_Value**	**Benjamini**
	GOTERM_MF_FAT	cofactor transporter activity	5	4.20E-02	8.30E-01
	GOTERM_MF_FAT	vitamin transporter activity	4	1.30E-01	9.50E-01
	GOTERM_BP_FAT	vitamin transport	4	4.80E-01	1.00E+00

ILMN_1738523	MYD88	0.749143118
ILMN_3188110	C19orf60	0.749113677
ILMN_2219134	RPS15	0.749081538
ILMN_1659762	BTF3	0.748956648
ILMN_1674337	FKBP2	0.748912493
ILMN_1811624	THADA	0.748883475
ILMN_1676765	RPL18A	0.748863715
ILMN_2336902	TRIM6	0.74874964
ILMN_1754145	CAPRIN1	0.748636943
ILMN_1792256	TBX2	0.748422142
ILMN_1695598	RPL18	0.748241343
ILMN_2402272	TCEAL8	0.748072527
ILMN_1732328	RPL22	0.747994322
ILMN_1742167	TUBA1C	0.747880886
ILMN_1679520	AGPAT1	0.747821968
ILMN_1789136	SERF2	0.747787471
ILMN_1690085	STK11IP	0.747659456
ILMN_1699015	H3F3A	0.747575539
ILMN_3202432	RPSA	0.747469134
ILMN_1805161	LZTR1	0.747419021
ILMN_2175912	ITGB2	0.747379246
ILMN_1752197	PDDC1	0.747305129
ILMN_1755658	ABI3	0.747162783
ILMN_1725534	ACTN4	0.747091663
ILMN_3247781	AK097701	0.746960724
ILMN_2051519	RPL37A	0.746839627
ILMN_1702044	CNTROB	0.746622693
ILMN_1732296	ID3	0.746592473
ILMN_1689585	C20orf194	0.746450291
ILMN_2148819	TUBA1A	0.746400147
ILMN_1737344	DDX41	0.74578133
ILMN_1790891	CKAP4	0.745769159
ILMN_1803813	ASTE1	0.745701066
ILMN_3241657	TMEM204	0.745431323
ILMN_1807535	YWHAE	0.745411392
ILMN_1765371	LUC7L	0.745317437
ILMN_1677906	PTMA	0.745313514
ILMN_1681543	RHBDD1	0.745305825
ILMN_1756595	SH3TC1	0.745104216
ILMN_3243297	RPL6	0.745100352
ILMN_1689989	CDC42BPB	0.745035512
ILMN_1730940	KLHDC3	0.744992527
ILMN_1678827	SPDYE7P	0.744941915
ILMN_1683204	GMEB2	0.744705211
ILMN_2261416	CD3D	0.744578995
ILMN_2411076	MATR3	0.744547337
ILMN_2384591	HN1	0.744528533
ILMN_2335398	CECR5	0.744517035
ILMN_2408179	PRRC2A	0.744364107
ILMN_1738347	RNPEP	0.744262065
ILMN_2230624	RPL18	0.744246643
ILMN_2262288	EEF1G	0.744154237
ILMN_1703288	C19orf6	0.744096924
ILMN_1666050	TMUB1	0.743526518
ILMN_1664861	ID1	0.74342345
ILMN_1685631	MAU2	0.743354483
ILMN_1814998	POTEKP	0.743325444
ILMN_2343775	RPL38	0.743226412
ILMN_1698940	RPL15	0.74309253
ILMN_1742521	GRB2	0.743086328
ILMN_1776963	LMAN2	0.743083332
ILMN_2356284	PFDN5	0.743081719
ILMN_3248069	RPL3	0.742726182
ILMN_1724422	SELL	0.742701863
ILMN_1678235	KIAA1267	0.742578781
ILMN_2333440	TM9SF1	0.742534842
ILMN_1758100	GALR3	0.742414912
ILMN_1659744	PRMT1	0.74238718
ILMN_1694778	KRT18	0.742292768
ILMN_1699931	HCST	0.742244256
ILMN_1751530	GADD45GIP1	0.742126611
ILMN_1812392	TMSB10	0.741990104
ILMN_2054019	ISG15	0.741885968
ILMN_2095597	CDH16	0.741683981
ILMN_1809013	MYL6	0.741657101
ILMN_2154671	COX6B1	0.74161683
ILMN_1659095	RPL7A	0.741545594
ILMN_1669281	CLN3	0.741462253
ILMN_3248487	RPL7A	0.741423171
ILMN_1760089	RPS2	0.741379833
ILMN_1728626	WDR5	0.741376304
ILMN_2415011	DCTD	0.741303668
ILMN_1728049	S100A16	0.741253522
ILMN_2316540	MRPL11	0.74122786
ILMN_1738684	NRXN2	0.741223652
ILMN_1724907	NUDT3	0.740878563
ILMN_2096654	COMMD4	0.740786308
ILMN_2280548	FYB	0.740768247
ILMN_1651899	RPL19	0.740625479
ILMN_1663799	RPL32	0.740617837
ILMN_1653797	C6orf62	0.740588198
ILMN_3241136	RNASEK	0.740543971
ILMN_1775327	PKM2	0.740476148
ILMN_1786176	CD37	0.740269633
ILMN_1734184	PLBD2	0.740196157
ILMN_2207539	RPS17	0.740116232
ILMN_2321416	DIAPH1	0.740019288

	SMART	SapB	4	5.30E-02	7.30E-01
**Annotation Cluster 61**	**Enrichment Score: 1.48**		**Count**	**P_Value**	**Benjamini**
	GOTERM_CC_FAT	membrane-bounded vesicle	95	1.50E-02	1.60E-01
	GOTERM_CC_FAT	vesicle	108	2.60E-02	2.40E-01
	GOTERM_CC_FAT	cytoplasmic membrane-bounded vesicle	89	3.80E-02	2.80E-01
	GOTERM_CC_FAT	cytoplasmic vesicle	99	8.30E-02	4.60E-01
**Annotation Cluster 62**	**Enrichment Score: 1.47**		**Count**	**P_Value**	**Benjamini**
	GOTERM_CC_FAT	membrane coat	19	1.10E-03	2.20E-02
	GOTERM_CC_FAT	coated membrane	19	1.10E-03	2.20E-02
	GOTERM_CC_FAT	clathrin coat	14	1.10E-03	2.20E-02
	GOTERM_CC_FAT	clathrin adaptor complex	11	3.10E-03	5.30E-02
	GOTERM_CC_FAT	AP-type membrane coat adaptor complex	11	4.20E-03	6.40E-02
	GOTERM_CC_FAT	vesicle coat	11	2.40E-02	2.30E-01
	GOTERM_CC_FAT	Golgi-associated vesicle	12	7.00E-02	4.20E-01
	GOTERM_CC_FAT	Golgi-associated vesicle membrane	8	8.30E-02	4.60E-01
	GOTERM_CC_FAT	clathrin vesicle coat	6	9.90E-02	4.90E-01
	GOTERM_CC_FAT	coated vesicle membrane	15	1.00E-01	5.00E-01
	GOTERM_CC_FAT	vesicle membrane	26	1.50E-01	5.90E-01
	GOTERM_CC_FAT	coated vesicle	27	1.60E-01	6.10E-01
	GOTERM_CC_FAT	cytoplasmic vesicle part	30	2.20E-01	7.00E-01
	GOTERM_CC_FAT	cytoplasmic vesicle membrane	23	2.30E-01	7.00E-01
	GOTERM_CC_FAT	clathrin coated vesicle membrane	10	2.70E-01	7.60E-01
	GOTERM_CC_FAT	clathrin-coated vesicle	20	4.00E-01	8.70E-01
**Annotation Cluster 63**	**Enrichment Score: 1.46**		**Count**	**P_Value**	**Benjamini**
	GOTERM_BP_FAT	cellular response to stress	106	1.50E-04	9.20E-03
	GOTERM_BP_FAT	DNA metabolic process	82	3.60E-02	4.20E-01
	GOTERM_BP_FAT	response to DNA damage stimulus	60	7.70E-02	6.10E-01
	SP_PIR_KEYWORDS	dna repair	30	1.10E-01	4.70E-01
	GOTERM_BP_FAT	DNA repair	44	1.80E-01	8.40E-01
	SP_PIR_KEYWORDS	DNA damage	30	2.00E-01	6.30E-01
**Annotation Cluster 64**	**Enrichment Score: 1.45**		**Count**	**P_Value**	**Benjamini**
	GOTERM_MF_FAT	ATPase activity	63	1.70E-03	1.10E-01
	GOTERM_MF_FAT	ATPase activity, coupled	53	2.00E-03	1.20E-01
	GOTERM_MF_FAT	ATP-dependent helicase activity	24	2.70E-03	1.30E-01
	GOTERM_MF_FAT	purine NTP-dependent helicase activity	24	2.70E-03	1.30E-01
	SP_PIR_KEYWORDS	helicase	28	4.80E-03	5.60E-02
	GOTERM_MF_FAT	RNA-dependent ATPase activity	8	1.70E-02	3.60E-01
	GOTERM_MF_FAT	helicase activity	28	1.90E-02	3.80E-01
	GOTERM_MF_FAT	RNA helicase activity	9	2.60E-02	4.50E-01
	GOTERM_MF_FAT	ATP-dependent DNA helicase activity	8	3.40E-02	5.00E-01
	GOTERM_MF_FAT	ATP-dependent RNA helicase activity	7	4.40E-02	5.80E-01
	INTERPRO	DEAD-like helicase, N-terminal	21	5.30E-02	9.20E-01
	GOTERM_MF_FAT	DNA helicase activity	10	6.40E-02	6.70E-01
	UP_SEQ_FEATURE	domain:Helicase ATP-binding	20	6.90E-02	1.00E+00
	UP_SEQ_FEATURE	short sequence motif:Q motif	9	7.10E-02	1.00E+00
	INTERPRO	DNA/RNA helicase, DEAD/DEAH box type, N-terminal	13	7.70E-02	9.60E-01
	INTERPRO	DNA/RNA helicase, C-terminal	20	7.70E-02	9.50E-01
	INTERPRO	Helicase, superfamily 1 and 2, ATP-binding	20	7.70E-02	9.50E-01
	SMART	DEXDc	21	7.70E-02	7.70E-01
	UP_SEQ_FEATURE	domain:Helicase C-terminal	19	8.00E-02	1.00E+00
	INTERPRO	RNA helicase, DEAD-box type, Q motif	9	8.70E-02	9.60E-01
	UP_SEQ_FEATURE	short sequence motif:DEAD box	8	9.60E-02	1.00E+00
	SMART	HELICc	20	1.00E-01	8.00E-01
	PIR_SUPERFAMILY	PIRSF001321:ATP-dependent RNA helicase	4	1.80E-01	1.00E+00
	GOTERM_MF_FAT	DNA-dependent ATPase activity	11	1.90E-01	9.00E-01
	INTERPRO	RNA helicase, ATP-dependent, DEAD-box, conserved site	6	3.80E-01	1.00E+00
**Annotation Cluster 65**	**Enrichment Score: 1.44**		**Count**	**P_Value**	**Benjamini**
	GOTERM_BP_FAT	viral reproductive process	16	1.30E-02	2.30E-01
	GOTERM_BP_FAT	viral infectious cycle	12	2.10E-02	3.10E-01
	GOTERM_BP_FAT	viral reproduction	16	4.20E-02	4.50E-01
	GOTERM_BP_FAT	initiation of viral infection	6	1.50E-01	8.00E-01
**Annotation Cluster 66**	**Enrichment Score: 1.44**		**Count**	**P_Value**	**Benjamini**
	GOTERM_BP_FAT	lung development	22	1.70E-02	2.70E-01
	GOTERM_BP_FAT	respiratory system development	23	2.40E-02	3.30E-01
	GOTERM_BP_FAT	respiratory tube development	22	2.40E-02	3.30E-01
	GOTERM_BP_FAT	tube development	35	1.80E-01	8.40E-01
**Annotation Cluster 67**	**Enrichment Score: 1.43**		**Count**	**P_Value**	**Benjamini**
	SP_PIR_KEYWORDS	Isomerase	28	9.10E-04	1.40E-02
	UP_SEQ_FEATURE	domain:PPIase FKBP-type	6	9.80E-03	9.00E-01
	SP_PIR_KEYWORDS	Rotamase	10	2.80E-02	2.00E-01
	INTERPRO	Peptidyl-prolyl cis-trans isomerase, FKBP-type	7	3.20E-02	9.00E-01
	GOTERM_MF_FAT	peptidyl-prolyl cis-trans isomerase activity	10	4.10E-02	5.50E-01
	GOTERM_MF_FAT	cis-trans isomerase activity	10	5.50E-02	6.40E-01
	UP_SEQ_FEATURE	domain:PPIase cyclophilin-type	4	3.70E-01	1.00E+00
	INTERPRO	Peptidyl-prolyl cis-trans isomerase, cyclophilin-type	4	5.00E-01	1.00E+00
**Annotation Cluster 68**	**Enrichment Score: 1.43**		**Count**	**P_Value**	**Benjamini**
	GOTERM_CC_FAT	NuRD complex	8	6.10E-04	1.40E-02
	GOTERM_CC_FAT	Sin3-type complex	3	2.90E-01	7.80E-01
	GOTERM_CC_FAT	Sin3 complex	3	2.90E-01	7.80E-01
**Annotation Cluster 69**	**Enrichment Score: 1.42**		**Count**	**P_Value**	**Benjamini**
	UP_SEQ_FEATURE	domain:FCH	7	2.70E-02	9.90E-01
	INTERPRO	Fps/Fes/Fer/CIP4 homology	7	4.10E-02	9.10E-01
	SMART	FCH	7	4.90E-02	7.50E-01
**Annotation Cluster 70**	**Enrichment Score: 1.41**		**Count**	**P_Value**	**Benjamini**
	GOTERM_BP_FAT	negative regulation of response to stimulus	23	1.00E-02	2.00E-01
	GOTERM_BP_FAT	regulation of inflammatory response	18	1.90E-02	2.80E-01
	GOTERM_BP_FAT	negative regulation of defense response	10	4.00E-02	4.40E-01
	GOTERM_BP_FAT	negative regulation of inflammatory response	8	1.00E-01	7.00E-01
	GOTERM_BP_FAT	negative regulation of response to external stimulus	11	1.10E-01	7.20E-01
**Annotation Cluster 71**	**Enrichment Score: 1.39**		**Count**	**P_Value**	**Benjamini**
	GOTERM_BP_FAT	regulation of cell activation	40	6.00E-04	2.90E-02
	GOTERM_BP_FAT	regulation of lymphocyte activation	35	7.40E-04	3.30E-02
	GOTERM_BP_FAT	regulation of leukocyte activation	38	8.10E-04	3.50E-02
	GOTERM_BP_FAT	regulation of T cell activation	29	1.10E-03	4.30E-02
	GOTERM_BP_FAT	positive regulation of lymphocyte activation	24	3.30E-03	9.00E-02
	GOTERM_BP_FAT	positive regulation of T cell activation	20	3.90E-03	1.00E-01
	GOTERM_BP_FAT	positive regulation of immune system process	47	4.60E-03	1.20E-01

ILMN_1787386	ADAMTS13	-0.424117354
ILMN_1675584	HTRA4	-0.42413881
ILMN_2355463	CYFIP1	-0.424204293
ILMN_1802105	STYK1	-0.424225734
ILMN_1770203	TUBGCP6	-0.42426629
ILMN_1736223	IQCH	-0.424272159
ILMN_1655348	GPR1	-0.424277053
ILMN_2079059	KRTAP4-11	-0.424298365
ILMN_1785574	HTRA2	-0.424354666
ILMN_1767542	THAP10	-0.424389436
ILMN_1693303	TAS2R41	-0.424410513
ILMN_1712999	MGAM	-0.424427509
ILMN_1859160	BX110173	-0.42445343
ILMN_1878007	FUT9	-0.424469342
ILMN_2148193	MPPED2	-0.42458187
ILMN_1801053	C15orf2	-0.424587832
ILMN_2253805	GFRA1	-0.424607489
ILMN_1901646	BX109627	-0.424864169
ILMN_2076658	MRPL1	-0.424946744
ILMN_1803673	LOC113230	-0.424988471
ILMN_1658109	LOC221442	-0.425011137
ILMN_1771233	CHMP4B	-0.425019744
ILMN_1689634	NA	-0.425068716
ILMN_1688346	ZNF800	-0.425144867
ILMN_1664216	NKIRAS1	-0.425154091
ILMN_1711069	YPEL5	-0.425170275
ILMN_2043306	EPB41L5	-0.425258071
ILMN_1672076	WFDC10B	-0.425299372
ILMN_1874770	DA691010	-0.425337705
ILMN_2195060	OR8K5	-0.425405996
ILMN_1871136	DR731427	-0.425447413
ILMN_1737685	CRLS1	-0.425494414
ILMN_1834233	AW138182	-0.425588095
ILMN_1699852	CES4A	-0.425595728
ILMN_2081863	NDUFB4	-0.425625577
ILMN_1760414	AADAC	-0.425643518
ILMN_1736510	FOXN2	-0.4256478
ILMN_1745851	LOC389676	-0.425650127
ILMN_3238823	CXorf49	-0.42571376
ILMN_2222984	NT5C1B	-0.425742622
ILMN_2286800	DNM1L	-0.425744372
ILMN_2089902	NUS1	-0.425745231
ILMN_1712751	HADHA	-0.425747776
ILMN_3244638	DDX3Y	-0.425795046
ILMN_1891885	AI219815	-0.425886906
ILMN_2306565	MTX2	-0.425912992
ILMN_1849267	DB026495	-0.425920313
ILMN_1760955	C1orf100	-0.425923315
ILMN_1754241	C16orf73	-0.425931476
ILMN_1737172	BC035647	-0.425957153
ILMN_1912185	GPC6	-0.426032637
ILMN_2178994	TUBB8	-0.42619813
ILMN_2153547	OR4C15	-0.426243844
ILMN_1700048	BX107512	-0.426253652
ILMN_1894862	AW130728	-0.426269146
ILMN_1806294	RPS6KA3	-0.426313921
ILMN_2376258	SMARCA1	-0.4264026
ILMN_3236671	CLRN2	-0.426436531
ILMN_1760798	RYR2	-0.426640324
ILMN_1769810	ARL6IP5	-0.426696823
ILMN_1703826	UNC5D	-0.426706936
ILMN_1824498	BQ889978	-0.426773639
ILMN_1782881	DLEU7	-0.426873294
ILMN_1718968	MT1E	-0.426940247
ILMN_3309965	MIRLET7F2	-0.427010655
ILMN_2405023	PPP1CB	-0.427108504
ILMN_1722527	LECT1	-0.427112236
ILMN_1903159	PPM1A	-0.427159427
ILMN_1885762	AI140602	-0.427257499
ILMN_3309799	MIR569	-0.427263382
ILMN_1788701	PSIP1	-0.427268444
ILMN_1690971	SSX3	-0.427355744
ILMN_1657977	MSRB2	-0.427383894
ILMN_1809035	SYT7	-0.427498519
ILMN_1866868	AA766245	-0.427506992
ILMN_2224454	TTY7	-0.427510999
ILMN_1741176	CHMP2B	-0.427548572
ILMN_1850372	AI798827	-0.427569658
ILMN_1813581	CNR1	-0.427593645
ILMN_1756311	UFSP2	-0.427619797
ILMN_1852040	AW263556	-0.427624859
ILMN_1657191	KRTAP10-5	-0.427635264
ILMN_1874577	LOC440117	-0.427692003
ILMN_1658825	FAM75C1	-0.427793933
ILMN_2128741	RDH11	-0.427811753
ILMN_1785379	ZNF8	-0.427831674
ILMN_2227790	GRIN2C	-0.427835691
ILMN_3241451	MRAP2	-0.427847201
ILMN_1787196	LYZL1	-0.427889431
ILMN_2052331	MYOC	-0.427896646
ILMN_1812461	WISP2	-0.427907155
ILMN_1769546	RIN2	-0.427933158
ILMN_1891857	RAB2A	-0.428033007
ILMN_1688723	MSX2P1	-0.428064247
ILMN_1887505	BX648650	-0.428068483
ILMN_1676946	AP3M2	-0.428089825
ILMN_1898130	CN291113	-0.42809942

	GOTERM_BP_FAT	cofactor transport	3	6.40E-01	1.00E+00
**Annotation Cluster 78**	**Enrichment Score: 0.69**		**Count**	**P_Value**	**Benjamini**
	INTERPRO	Nucleosome assembly protein (NAP)	6	2.10E-02	9.90E-01
	GOTERM_BP_FAT	nucleosome organization	16	6.40E-02	9.80E-01
	GOTERM_BP_FAT	chromatin assembly or disassembly	20	7.70E-02	9.80E-01
	GOTERM_BP_FAT	nucleosome assembly	14	1.00E-01	9.80E-01
	GOTERM_BP_FAT	DNA packaging	18	1.10E-01	9.70E-01
	GOTERM_BP_FAT	chromatin assembly	14	1.30E-01	9.70E-01
	GOTERM_BP_FAT	protein-DNA complex assembly	14	1.60E-01	9.80E-01
	SMART	H2A	4	2.90E-01	9.70E-01
	GOTERM_CC_FAT	protein-DNA complex	12	3.20E-01	9.50E-01
	INTERPRO	Histone H2A	4	3.30E-01	1.00E+00
	SP_PIR_KEYWORDS	chromosomal protein	18	3.50E-01	9.30E-01
	GOTERM_CC_FAT	nucleosome	9	3.70E-01	9.50E-01
	SP_PIR_KEYWORDS	nucleosome core	7	3.90E-01	9.30E-01
	INTERPRO	Histone-fold	7	5.20E-01	1.00E+00
	INTERPRO	Histone core	6	5.30E-01	1.00E+00
	KEGG_PATHWAY	Systemic lupus erythematosus	9	8.80E-01	9.80E-01
**Annotation Cluster 79**	**Enrichment Score: 0.68**		**Count**	**P_Value**	**Benjamini**
	INTERPRO	Guanine nucleotide binding protein (G-protein), alpha subunit	5	8.10E-02	1.00E+00
	PIR_SUPERFAMILY	PIRSF002400:GTP-binding regulatory protein Gs alpha chain	5	8.50E-02	1.00E+00
	SP_PIR_KEYWORDS	signal transduction	7	1.50E-01	8.70E-01
	SP_PIR_KEYWORDS	heterotrimer	6	1.60E-01	8.70E-01
	INTERPRO	G-protein alpha subunit, group I	3	2.00E-01	1.00E+00
	SP_PIR_KEYWORDS	ADP-ribosylation	6	2.60E-01	9.00E-01
	SP_PIR_KEYWORDS	GTP binding	10	2.60E-01	9.00E-01
	SP_PIR_KEYWORDS	myristylation	6	3.90E-01	9.40E-01
	SP_PIR_KEYWORDS	thiolester bond	4	7.40E-01	9.90E-01
**Annotation Cluster 80**	**Enrichment Score: 0.68**		**Count**	**P_Value**	**Benjamini**
	UP_SEQ_FEATURE	domain:DRBM 3	4	9.90E-03	1.00E+00
	GOTERM_BP_FAT	RNA interference, production of siRNA	3	3.10E-02	9.60E-01
	GOTERM_MF_FAT	double-stranded RNA binding	8	5.60E-02	8.70E-01
	UP_SEQ_FEATURE	domain:DRBM 2	4	1.20E-01	1.00E+00
	UP_SEQ_FEATURE	domain:DRBM 1	4	1.20E-01	1.00E+00
	GOTERM_BP_FAT	RNA interference	3	1.30E-01	9.80E-01
	SP_PIR_KEYWORDS	rna-mediated gene silencing	6	1.50E-01	8.60E-01
	SMART	DSRM	5	1.70E-01	9.40E-01
	INTERPRO	Double-stranded RNA binding	5	2.00E-01	1.00E+00
	GOTERM_BP_FAT	gene silencing	9	2.40E-01	9.90E-01
	INTERPRO	Double-stranded RNA-binding-like	4	2.70E-01	1.00E+00
	GOTERM_BP_FAT	posttranscriptional gene silencing by RNA	4	3.90E-01	1.00E+00
	GOTERM_BP_FAT	posttranscriptional gene silencing	4	3.90E-01	1.00E+00
	GOTERM_BP_FAT	dsRNA fragmentation	3	4.10E-01	1.00E+00
	GOTERM_BP_FAT	gene silencing by RNA	5	4.70E-01	1.00E+00
	GOTERM_BP_FAT	gene silencing by miRNA	3	5.50E-01	1.00E+00
	GOTERM_BP_FAT	regulation of gene expression, epigenetic	9	5.80E-01	1.00E+00
	GOTERM_BP_FAT	chromatin silencing	3	5.80E-01	1.00E+00
	GOTERM_BP_FAT	negative regulation of gene expression, epigenetic	3	6.70E-01	1.00E+00
	GOTERM_BP_FAT	response to dsRNA	3	7.60E-01	1.00E+00
**Annotation Cluster 81**	**Enrichment Score: 0.67**		**Count**	**P_Value**	**Benjamini**
	INTERPRO	Quinone oxidoreductase/zeta-crystallin, conserved site	3	1.60E-01	1.00E+00
	INTERPRO	Alcohol dehydrogenase GroES-like	4	1.80E-01	1.00E+00
	INTERPRO	Alcohol dehydrogenase, zinc-binding	4	2.70E-01	1.00E+00
	INTERPRO	Alcohol dehydrogenase superfamily, zinc-containing	4	2.70E-01	1.00E+00
**Annotation Cluster 82**	**Enrichment Score: 0.67**		**Count**	**P_Value**	**Benjamini**
	UP_SEQ_FEATURE	domain:CS	4	1.80E-01	1.00E+00
	INTERPRO	CS	4	2.40E-01	1.00E+00
	INTERPRO	CS domain	4	2.40E-01	1.00E+00
**Annotation Cluster 83**	**Enrichment Score: 0.67**		**Count**	**P_Value**	**Benjamini**
	UP_SEQ_FEATURE	short sequence motif:GXSXG	3	2.00E-01	1.00E+00
	UP_SEQ_FEATURE	domain:Patatin	3	2.00E-01	1.00E+00
	INTERPRO	Patatin	3	2.50E-01	1.00E+00
**Annotation Cluster 84**	**Enrichment Score: 0.66**		**Count**	**P_Value**	**Benjamini**
	GOTERM_BP_FAT	cell growth	11	1.10E-01	9.70E-01
	GOTERM_BP_FAT	regulation of cell size	28	1.40E-01	9.80E-01
	GOTERM_BP_FAT	growth	19	6.70E-01	1.00E+00
**Annotation Cluster 85**	**Enrichment Score: 0.66**		**Count**	**P_Value**	**Benjamini**
	SP_PIR_KEYWORDS	cell junction	53	5.70E-02	8.20E-01
	GOTERM_CC_FAT	cell junction	67	7.90E-02	9.10E-01
	GOTERM_CC_FAT	postsynaptic density	12	1.40E-01	9.20E-01
	SP_PIR_KEYWORDS	synapse	26	3.00E-01	9.20E-01
	GOTERM_CC_FAT	synapse	42	3.40E-01	9.50E-01
	SP_PIR_KEYWORDS	postsynaptic cell membrane	14	3.60E-01	9.30E-01
	GOTERM_CC_FAT	synapse part	28	4.80E-01	9.70E-01
	GOTERM_CC_FAT	postsynaptic membrane	16	4.90E-01	9.70E-01
**Annotation Cluster 86**	**Enrichment Score: 0.66**		**Count**	**P_Value**	**Benjamini**
	GOTERM_BP_FAT	negative regulation of catalytic activity	37	1.10E-01	9.80E-01
	GOTERM_BP_FAT	negative regulation of MAP kinase activity	7	1.80E-01	9.80E-01
	GOTERM_BP_FAT	negative regulation of protein kinase activity	13	2.10E-01	9.90E-01
	GOTERM_BP_FAT	negative regulation of transferase activity	14	2.10E-01	9.90E-01
	GOTERM_BP_FAT	negative regulation of kinase activity	13	2.40E-01	9.90E-01
	GOTERM_BP_FAT	regulation of protein kinase activity	38	5.10E-01	1.00E+00
**Annotation Cluster 87**	**Enrichment Score: 0.66**		**Count**	**P_Value**	**Benjamini**
	GOTERM_BP_FAT	lipid biosynthetic process	47	2.30E-02	9.60E-01
	GOTERM_BP_FAT	organophosphate metabolic process	30	5.00E-02	9.80E-01
	GOTERM_BP_FAT	phospholipid metabolic process	28	7.00E-02	9.80E-01
	GOTERM_BP_FAT	glycerolipid biosynthetic process	14	7.70E-02	9.80E-01
	GOTERM_BP_FAT	glycerolipid metabolic process	24	8.90E-02	9.80E-01
	GOTERM_BP_FAT	preassembly of GPI anchor in ER membrane	5	9.80E-02	9.80E-01
	GOTERM_BP_FAT	phospholipid biosynthetic process	16	1.20E-01	9.70E-01
	GOTERM_BP_FAT	glycerophospholipid metabolic process	17	1.80E-01	9.80E-01
	GOTERM_BP_FAT	glycerophospholipid biosynthetic process	10	2.90E-01	9.90E-01
	GOTERM_BP_FAT	phosphoinositide biosynthetic process	6	4.20E-01	1.00E+00
	SP_PIR_KEYWORDS	gpi-anchor biosynthesis	4	4.70E-01	9.50E-01
	GOTERM_BP_FAT	GPI anchor biosynthetic process	5	4.90E-01	1.00E+00
	GOTERM_BP_FAT	protein amino acid lipidation	7	5.00E-01	1.00E+00
	GOTERM_BP_FAT	GPI anchor metabolic process	5	5.20E-01	1.00E+00

ILMN_2321064	BAX	0.739890518
ILMN_1690125	PDLIM7	0.739634415
ILMN_1765523	TOLLIP	0.739566202
ILMN_1652631	GLIPR2	0.739411309
ILMN_2337655	WARS	0.738993733
ILMN_1660869	SERF2-C15ORF63	0.738460636
ILMN_3284584	RPL10	0.738444294
ILMN_1666522	ZDHHC14	0.738322719
ILMN_1801909	IGHMBP2	0.738254491
ILMN_1670841	CPNE1	0.738134936
ILMN_1655126	LOC375133	0.738104634
ILMN_1705302	FCGRT	0.737929733
ILMN_1714622	TNRC6A	0.737815755
ILMN_1710697	BUD31	0.737719804
ILMN_1733045	RAB36	0.737485946
ILMN_2344130	PSMD4	0.737266157
ILMN_1782977	UBA52	0.73716794
ILMN_1793632	TMEM222	0.73713849
ILMN_2339863	VPS28	0.73713121
ILMN_2361427	PMS2P3	0.737126013
ILMN_1712435	NA	0.737115708
ILMN_3245403	RPL17	0.736920197
ILMN_1737586	EIF4H	0.736827318
ILMN_2351795	C18orf1	0.7367946
ILMN_2225887	ATP5EP2	0.736462159
ILMN_2309180	SMARCD3	0.736423232
ILMN_3219558	TUBA1C	0.736323324
ILMN_2389582	HNRNPL	0.736218413
ILMN_1802205	RHOB	0.736057886
ILMN_1671911	MTA1	0.736053868
ILMN_2226955	VOPP1	0.736035768
ILMN_3247452	OST4	0.736020172
ILMN_1733703	GTSE1	0.735948768
ILMN_3247906	RNF114	0.735792395
ILMN_1797530	CHCHD5	0.73573271
ILMN_2112493	DAP	0.735726116
ILMN_2320888	CXCR4	0.735719408
ILMN_1733991	UBL7	0.735629463
ILMN_1658425	DAG1	0.735618369
ILMN_2206716	JTB	0.735598805
ILMN_1742166	GRWD1	0.735534054
ILMN_1699570	TPD52L2	0.735184095
ILMN_1772113	U2AF1	0.735180067
ILMN_1675479	ZNF646	0.735157336
ILMN_1701696	SRP14	0.734987259
ILMN_1697777	TSTA3	0.734888093
ILMN_1802053	ZNF91	0.73472377
ILMN_1713394	RPS16	0.734627919
ILMN_1713402	FAM160A2	0.734529733
ILMN_2083559	KIAA0415	0.734276087
ILMN_1788099	LSM4	0.734200543
ILMN_1740395	RAVER1	0.73409799
ILMN_1738677	PRPF8	0.734073742
ILMN_2143685	CLDN7	0.733952055
ILMN_2384496	ST6GAL1	0.73394705
ILMN_1778132	C2CD2L	0.733831375
ILMN_2169025	JOSD2	0.733799154
ILMN_1756982	CLIC1	0.733788964
ILMN_2075847	ZC3H10	0.733694641
ILMN_2354391	RAB4B-EGLN2	0.733632767
ILMN_2173451	GPI	0.733626254
ILMN_1680626	PDIA6	0.733598848
ILMN_1808202	C19orf22	0.73359855
ILMN_1790951	C19orf50	0.733550828
ILMN_2293131	ARHGEF1	0.733518636
ILMN_2140389	TMEM185A	0.733435005
ILMN_2325234	PQBP1	0.733229163
ILMN_2287888	PSMC4	0.733213151
ILMN_1739794	CD3E	0.733159787
ILMN_1774334	HIGD2A	0.732927988
ILMN_1798083	CHERP	0.732905648
ILMN_1764794	PSMB2	0.732893804
ILMN_1760714	RPS3	0.732831237
ILMN_1713369	RPL13A	0.732680312
ILMN_1756696	USF2	0.732606733
ILMN_2356786	ADD1	0.732451155
ILMN_1696065	SDF4	0.732350935
ILMN_2407589	CTDP1	0.732307437
ILMN_1761138	C9orf142	0.732074295
ILMN_1737935	MACF1	0.731992143
ILMN_1690653	CDK2AP2	0.731766512
ILMN_1659905	FKBP10	0.73163287
ILMN_3202734	RPL17	0.73158821
ILMN_1742432	COBRA1	0.731556516
ILMN_1706273	MOB2	0.731546943
ILMN_1808041	RPL10A	0.731483283
ILMN_1731287	ARFGAP3	0.73135961
ILMN_2137066	ZNF7	0.731315655
ILMN_1753008	REXO1	0.731301258
ILMN_1755234	SSH3	0.731294931
ILMN_1652594	ACTR5	0.731041723
ILMN_1663444	LIN7B	0.730886503
ILMN_1754660	ZCCHC24	0.730869661
ILMN_1797362	LIMK1	0.730828199
ILMN_2376205	LTB	0.730798793
ILMN_1761797	CSTB	0.730770844

	GOTERM_BP_FAT	positive regulation of leukocyte activation	25	5.10E-03	1.20E-01
	GOTERM_BP_FAT	positive regulation of cell activation	25	9.20E-03	1.90E-01
	GOTERM_BP_FAT	positive regulation of alpha-beta T cell activation	9	1.60E-02	2.60E-01
	GOTERM_BP_FAT	regulation of T cell differentiation	13	3.10E-02	3.90E-01
	GOTERM_BP_FAT	regulation of lymphocyte differentiation	15	3.30E-02	4.00E-01
	GOTERM_BP_FAT	positive regulation of lymphocyte differentiation	10	3.40E-02	4.00E-01
	GOTERM_BP_FAT	regulation of alpha-beta T cell activation	10	4.00E-02	4.40E-01
	GOTERM_BP_FAT	regulation of lymphocyte proliferation	18	4.10E-02	4.50E-01
	GOTERM_BP_FAT	regulation of leukocyte proliferation	18	4.60E-02	4.80E-01
	GOTERM_BP_FAT	regulation of mononuclear cell proliferation	18	4.60E-02	4.80E-01
	GOTERM_BP_FAT	positive regulation of T cell differentiation	9	5.20E-02	5.00E-01
	GOTERM_BP_FAT	positive regulation of lymphocyte proliferation	13	5.20E-02	5.00E-01
	GOTERM_BP_FAT	positive regulation of leukocyte proliferation	13	5.80E-02	5.40E-01
	GOTERM_BP_FAT	positive regulation of mononuclear cell proliferation	13	5.80E-02	5.40E-01
	GOTERM_BP_FAT	regulation of T cell proliferation	13	1.10E-01	7.10E-01
	GOTERM_BP_FAT	positive regulation of alpha-beta T cell differentiation	6	1.10E-01	7.20E-01
	GOTERM_BP_FAT	regulation of B cell activation	11	1.30E-01	7.50E-01
	GOTERM_BP_FAT	positive regulation of T cell proliferation	9	1.30E-01	7.60E-01
	GOTERM_BP_FAT	positive regulation of B cell activation	8	1.40E-01	7.70E-01
	GOTERM_BP_FAT	regulation of B cell proliferation	7	1.50E-01	8.00E-01
	GOTERM_BP_FAT	regulation of production of molecular mediator of immune response	9	1.60E-01	8.20E-01
	GOTERM_BP_FAT	regulation of immunoglobulin mediated immune response	6	2.00E-01	8.60E-01
	GOTERM_BP_FAT	regulation of B cell mediated immunity	6	2.00E-01	8.60E-01
	GOTERM_BP_FAT	regulation of alpha-beta T cell differentiation	6	2.30E-01	8.90E-01
	GOTERM_BP_FAT	regulation of immunoglobulin production	6	2.50E-01	9.10E-01
	GOTERM_BP_FAT	positive regulation of B cell proliferation	5	2.70E-01	9.20E-01
	GOTERM_BP_FAT	positive regulation of isotype switching	3	3.40E-01	9.50E-01
	GOTERM_BP_FAT	positive regulation of DNA recombination	3	3.40E-01	9.50E-01
	GOTERM_BP_FAT	regulation of isotype switching	3	5.30E-01	9.90E-01
	GOTERM_BP_FAT	regulation of DNA recombination	3	8.80E-01	1.00E+00
**Annotation Cluster 72**	**Enrichment Score: 1.38**		**Count**	**P_Value**	**Benjamini**
	GOTERM_CC_FAT	cytoskeleton	214	9.90E-03	1.30E-01
	GOTERM_CC_FAT	microtubule cytoskeleton	87	6.10E-02	3.80E-01
	GOTERM_CC_FAT	cytoskeletal part	141	1.20E-01	5.30E-01
**Annotation Cluster 73**	**Enrichment Score: 1.38**		**Count**	**P_Value**	**Benjamini**
	GOTERM_BP_FAT	regulation of lymphocyte activation	35	7.40E-04	3.30E-02
	GOTERM_BP_FAT	regulation of T cell activation	29	1.10E-03	4.30E-02
	GOTERM_BP_FAT	negative regulation of immune system process	22	2.10E-03	6.50E-02
	GOTERM_BP_FAT	negative regulation of cell activation	15	2.50E-02	3.40E-01
	GOTERM_BP_FAT	regulation of lymphocyte proliferation	18	4.10E-02	4.50E-01
	GOTERM_BP_FAT	negative regulation of T cell activation	11	4.30E-02	4.60E-01
	GOTERM_BP_FAT	regulation of leukocyte proliferation	18	4.60E-02	4.80E-01
	GOTERM_BP_FAT	regulation of mononuclear cell proliferation	18	4.60E-02	4.80E-01
	GOTERM_BP_FAT	negative regulation of lymphocyte activation	13	4.60E-02	4.80E-01
	GOTERM_BP_FAT	negative regulation of leukocyte activation	13	6.50E-02	5.70E-01
	GOTERM_BP_FAT	regulation of T cell proliferation	13	1.10E-01	7.10E-01
	GOTERM_BP_FAT	negative regulation of leukocyte proliferation	7	2.40E-01	9.00E-01
	GOTERM_BP_FAT	negative regulation of lymphocyte proliferation	7	2.40E-01	9.00E-01
	GOTERM_BP_FAT	negative regulation of mononuclear cell proliferation	7	2.40E-01	9.00E-01
	GOTERM_BP_FAT	negative regulation of T cell proliferation	6	2.80E-01	9.30E-01
	GOTERM_BP_FAT	regulation of activated T cell proliferation	3	4.80E-01	9.90E-01
**Annotation Cluster 74**	**Enrichment Score: 1.37**		**Count**	**P_Value**	**Benjamini**
	BBID	73.Integrins_and_other_cell-surface_receptors	8	1.70E-02	7.50E-01
	PIR_SUPERFAMILY	PIRSF002419:CD9 antigen	7	2.60E-02	9.90E-01
	INTERPRO	Tetraspanin, conserved site	8	3.80E-02	9.20E-01
	INTERPRO	Tetraspanin, subgroup	8	7.50E-02	9.60E-01
	INTERPRO	Tetraspanin	8	1.10E-01	9.70E-01
**Annotation Cluster 75**	**Enrichment Score: 1.36**		**Count**	**P_Value**	**Benjamini**
	GOTERM_CC_FAT	clathrin coat	14	1.10E-03	2.20E-02
	GOTERM_CC_FAT	clathrin adaptor complex	11	3.10E-03	5.30E-02
	GOTERM_CC_FAT	AP-type membrane coat adaptor complex	11	4.20E-03	6.40E-02
	INTERPRO	Clathrin adaptor, mu subunit, C-terminal	5	4.00E-02	9.20E-01
	INTERPRO	Clathrin adaptor, mu subunit, conserved site	4	4.70E-02	9.20E-01
	INTERPRO	Clathrin adaptor, alpha/beta/gamma-adaptin, appendage, Ig-like subdomain	4	9.40E-02	9.60E-01
	SMART	Alpha_adaptinC2	4	1.00E-01	7.90E-01
	UP_SEQ_FEATURE	domain:MHD	4	1.10E-01	1.00E+00
	INTERPRO	Clathrin adaptor, mu subunit	3	1.70E-01	9.90E-01
	PIR_SUPERFAMILY	PIRSF005992:Clathrin_mu	3	1.90E-01	1.00E+00
	PIR_SUPERFAMILY	PIRSF005992:adaptor protein complex, mu subunit	3	1.90E-01	1.00E+00
	INTERPRO	Clathrin/coatomer adaptor, adaptin-like, N-terminal	4	2.60E-01	1.00E+00
**Annotation Cluster 76**	**Enrichment Score: 1.32**		**Count**	**P_Value**	**Benjamini**
	GOTERM_CC_FAT	microtubule organizing center	45	3.80E-02	2.80E-01
	GOTERM_CC_FAT	centrosome	40	4.80E-02	3.30E-01
	GOTERM_CC_FAT	microtubule cytoskeleton	87	6.10E-02	3.80E-01
**Annotation Cluster 77**	**Enrichment Score: 1.3**		**Count**	**P_Value**	**Benjamini**
	UP_SEQ_FEATURE	domain:BRCT	6	2.00E-02	9.70E-01
	INTERPRO	BRCT	7	7.30E-02	9.60E-01
	SMART	BRCT	7	8.70E-02	7.90E-01
**Annotation Cluster 78**	**Enrichment Score: 1.29**		**Count**	**P_Value**	**Benjamini**
	UP_SEQ_FEATURE	domain:Bromo	9	1.50E-02	9.40E-01
	UP_SEQ_FEATURE	zinc finger region:PHD-type	12	4.40E-02	1.00E+00
	INTERPRO	Bromodomain	10	5.80E-02	9.40E-01
	SMART	BROMO	10	7.30E-02	7.70E-01
	INTERPRO	Bromodomain, conserved site	9	7.60E-02	9.60E-01
	SP_PIR_KEYWORDS	bromodomain	9	9.00E-02	4.10E-01
**Annotation Cluster 79**	**Enrichment Score: 1.29**		**Count**	**P_Value**	**Benjamini**
	GOTERM_BP_FAT	T cell selection	8	8.10E-03	1.70E-01
	GOTERM_BP_FAT	thymic T cell selection	6	2.00E-02	3.00E-01
	GOTERM_BP_FAT	positive thymic T cell selection	4	5.30E-02	5.10E-01
	GOTERM_BP_FAT	negative thymic T cell selection	4	7.70E-02	6.10E-01
	GOTERM_BP_FAT	positive T cell selection	4	1.00E-01	7.00E-01
	GOTERM_BP_FAT	negative T cell selection	4	1.00E-01	7.00E-01
	GOTERM_BP_FAT	T cell differentiation in the thymus	7	1.40E-01	7.70E-01
**Annotation Cluster 80**	**Enrichment Score: 1.28**		**Count**	**P_Value**	**Benjamini**
	GOTERM_BP_FAT	defense response	98	3.30E-02	4.00E-01
	GOTERM_BP_FAT	inflammatory response	55	4.20E-02	4.50E-01

ILMN_3232869	NA	-0.428259945
ILMN_1782651	NLRP14	-0.428335873
ILMN_2312386	PAIP1	-0.428363785
ILMN_1752557	NEU2	-0.428389602
ILMN_1888925	BQ184051	-0.428480908
ILMN_3259148	AB058757	-0.428515085
ILMN_1713846	PPM1H	-0.428520709
ILMN_1738217	NPIPL1	-0.428545148
ILMN_1654979	EPHA6	-0.428585569
ILMN_1658452	FAM53A	-0.428653755
ILMN_3242743	NA	-0.428786445
ILMN_3307132	AK127120	-0.428876979
ILMN_1766974	ZNF107	-0.428912359
ILMN_3300663	SLC35E2B	-0.429045317
ILMN_3272299	EML6	-0.429070749
ILMN_1769099	ZNF253	-0.429091607
ILMN_1673138	ZBTB33	-0.429101869
ILMN_1833212	C9orf130	-0.429171819
ILMN_1735062	KCNG2	-0.429236939
ILMN_1785404	FGFBP1	-0.429287969
ILMN_1758111	PIBF1	-0.429288058
ILMN_1745788	CX3CR1	-0.429344508
ILMN_1815705	LZTFL1	-0.429349175
ILMN_1726054	C13orf26	-0.429387652
ILMN_1808457	TBX18	-0.429463347
ILMN_1721741	AY358864	-0.429477547
ILMN_1729314	PRG2	-0.429508499
ILMN_1693323	C6orf162	-0.429514342
ILMN_2128923	HCCS	-0.429518411
ILMN_2376980	FSD1	-0.429532381
ILMN_1751941	TMEM106A	-0.429538597
ILMN_1763732	ZC3H13	-0.429599387
ILMN_1871756	AA609726	-0.429721419
ILMN_2415583	HS6ST2	-0.429750202
ILMN_1739178	TBXA2R	-0.429766709
ILMN_2159322	SLK	-0.429772553
ILMN_1665458	PDHA2	-0.429796515
ILMN_1916404	AI948987	-0.429803823
ILMN_1833010	BF001957	-0.429861286
ILMN_3218421	NA	-0.429888246
ILMN_1777453	NPB	-0.429982509
ILMN_1658806	SLC5A10	-0.430035052
ILMN_1767632	FLJ44838	-0.430089556
ILMN_3245659	ERI1	-0.430203769
ILMN_1845162	MYO1H	-0.430265648
ILMN_1897405	CA420307	-0.430344795
ILMN_1663932	TP53TG3b	-0.430352466
ILMN_1657680	CCDC69	-0.430402929
ILMN_1761511	APOH	-0.430404287
ILMN_2372384	MGA	-0.430417436
ILMN_1687371	LOC100131257	-0.430581356
ILMN_1756878	SLC39A9	-0.430602736
ILMN_3305180	LOC100631378	-0.43063044
ILMN_1658710	FAM19A4	-0.430731411
ILMN_1740463	AX747015	-0.430754359
ILMN_2107655	SNORD116-2	-0.430796517
ILMN_1854544	LOC100289673	-0.430803434
ILMN_3306168	MOBKL3	-0.430836961
ILMN_1758333	HMGXB4	-0.430896013
ILMN_1716803	RALYL	-0.431009779
ILMN_1846110	NA	-0.431125632
ILMN_1749009	REXO2	-0.431199544
ILMN_3239946	LOC729375	-0.431351493
ILMN_1758542	BMP1	-0.431364816
ILMN_2088825	ACAP2	-0.431389727
ILMN_1667750	SLC4A5	-0.431401101
ILMN_3177615	LOC100128816	-0.431473698
ILMN_2351902	SMN1	-0.431495602
ILMN_1686884	IL1RAP	-0.431499851
ILMN_1667561	IFRD1	-0.431546459
ILMN_1664622	EFR3B	-0.431565379
ILMN_1780625	HR	-0.431652883
ILMN_1892782	NA	-0.431655352
ILMN_1752935	TMEM30B	-0.431679243
ILMN_1775759	NRAS	-0.431696171
ILMN_1707342	LRIG1	-0.431699745
ILMN_1735278	OR13C3	-0.431743776
ILMN_1652459	ATAD3B	-0.431759749
ILMN_1798535	SMR3A	-0.43178259
ILMN_1689510	AK128283	-0.431878408
ILMN_1798853	CTSL1P2	-0.431959947
ILMN_3248970	KIAA0393	-0.431960765
ILMN_3243773	GALNTL6	-0.431978149
ILMN_3190834	ELMOD3	-0.43200874
ILMN_2300894	KCNQ2	-0.432046141
ILMN_1730931	RUFY3	-0.432060268
ILMN_3201183	NA	-0.432079676
ILMN_1766128	THAP3	-0.432087015
ILMN_1913510	AK023519	-0.432105989
ILMN_2154836	BTG3	-0.43211068
ILMN_1694917	SERPINB11	-0.432125266
ILMN_1671337	SLC2A5	-0.432162772
ILMN_1791587	OR2Y1	-0.432165773
ILMN_2221769	PRAMEF11	-0.432175952
ILMN_3272689	NA	-0.432227101
ILMN_3226405	PRAMEF11	-0.432290462

	KEGG_PATHWAY	Glycosylphosphatidylinositol(GPI)-anchor biosynthesis	4	5.40E-01	9.50E-01
	GOTERM_BP_FAT	lipoprotein biosynthetic process	7	5.90E-01	1.00E+00
	GOTERM_BP_FAT	phosphoinositide metabolic process	8	6.70E-01	1.00E+00
	GOTERM_BP_FAT	lipoprotein metabolic process	7	8.60E-01	1.00E+00
**Annotation Cluster 88**	**Enrichment Score: 0.65**		**Count**	**P_Value**	**Benjamini**
	GOTERM_BP_FAT	glutamine metabolic process	5	1.30E-01	9.80E-01
	GOTERM_BP_FAT	glutamine family amino acid metabolic process	9	1.80E-01	9.80E-01
	KEGG_PATHWAY	Alanine, aspartate and glutamate metabolism	5	4.60E-01	9.50E-01
**Annotation Cluster 89**	**Enrichment Score: 0.64**		**Count**	**P_Value**	**Benjamini**
	GOTERM_BP_FAT	regulation of action potential in neuron	10	1.10E-01	9.80E-01
	GOTERM_BP_FAT	myelination	7	2.10E-01	9.90E-01
	GOTERM_BP_FAT	regulation of membrane potential	18	2.50E-01	9.90E-01
	GOTERM_BP_FAT	axon ensheathment	7	2.80E-01	9.90E-01
	GOTERM_BP_FAT	ensheathment of neurons	7	2.80E-01	9.90E-01
	GOTERM_BP_FAT	regulation of action potential	10	2.90E-01	9.90E-01
**Annotation Cluster 90**	**Enrichment Score: 0.64**		**Count**	**P_Value**	**Benjamini**
	SMART	PTB	7	1.70E-01	9.40E-01
	INTERPRO	Phosphotyrosine interaction region	7	2.10E-01	1.00E+00
	UP_SEQ_FEATURE	domain:PID	5	3.40E-01	1.00E+00
**Annotation Cluster 91**	**Enrichment Score: 0.63**		**Count**	**P_Value**	**Benjamini**
	SMART	PDZ	20	1.50E-01	9.40E-01
	INTERPRO	PDZ/DHR/GLGF	20	2.30E-01	1.00E+00
	UP_SEQ_FEATURE	domain:PDZ	14	3.70E-01	1.00E+00
**Annotation Cluster 92**	**Enrichment Score: 0.62**		**Count**	**P_Value**	**Benjamini**
	GOTERM_BP_FAT	cellular lipid catabolic process	14	5.50E-02	9.80E-01
	GOTERM_BP_FAT	fatty acid catabolic process	7	1.80E-01	9.80E-01
	GOTERM_BP_FAT	lipid oxidation	7	2.30E-01	9.90E-01
	GOTERM_BP_FAT	fatty acid oxidation	7	2.30E-01	9.90E-01
	GOTERM_BP_FAT	fatty acid beta-oxidation	4	5.80E-01	1.00E+00
	GOTERM_BP_FAT	lipid modification	8	6.10E-01	1.00E+00
**Annotation Cluster 93**	**Enrichment Score: 0.62**		**Count**	**P_Value**	**Benjamini**
	INTERPRO	BTB/POZ	19	1.20E-01	1.00E+00
	UP_SEQ_FEATURE	domain:BTB	20	1.60E-01	1.00E+00
	SMART	BTB	23	1.70E-01	9.30E-01
	INTERPRO	BTB/POZ-like	23	2.60E-01	1.00E+00
	INTERPRO	BTB/POZ fold	22	3.60E-01	1.00E+00
	INTERPRO	Kelch related	9	6.10E-01	1.00E+00
**Annotation Cluster 94**	**Enrichment Score: 0.61**		**Count**	**P_Value**	**Benjamini**
	INTERPRO	ATP-grasp fold, subdomain 2	5	9.80E-02	1.00E+00
	UP_SEQ_FEATURE	domain:ATP-grasp	3	3.60E-01	1.00E+00
	INTERPRO	ATP-grasp fold	3	4.10E-01	1.00E+00
**Annotation Cluster 95**	**Enrichment Score: 0.61**		**Count**	**P_Value**	**Benjamini**
	GOTERM_BP_FAT	calcium ion homeostasis	27	9.50E-02	9.80E-01
	GOTERM_BP_FAT	metal ion homeostasis	28	1.40E-01	9.80E-01
	GOTERM_BP_FAT	cellular calcium ion homeostasis	25	1.60E-01	9.80E-01
	GOTERM_BP_FAT	negative regulation of sequestering of calcium ion	4	1.80E-01	9.80E-01
	GOTERM_BP_FAT	regulation of sequestering of calcium ion	4	1.80E-01	9.80E-01
	GOTERM_BP_FAT	release of sequestered calcium ion into cytosol	4	1.80E-01	9.80E-01
	GOTERM_BP_FAT	cellular metal ion homeostasis	26	1.90E-01	9.90E-01
	GOTERM_BP_FAT	calcium ion transport into cytosol	4	3.30E-01	9.90E-01
	GOTERM_BP_FAT	cytosolic calcium ion transport	4	3.60E-01	1.00E+00
	GOTERM_BP_FAT	cytosolic calcium ion homeostasis	11	8.20E-01	1.00E+00
	GOTERM_BP_FAT	elevation of cytosolic calcium ion concentration	10	8.40E-01	1.00E+00
**Annotation Cluster 96**	**Enrichment Score: 0.6**		**Count**	**P_Value**	**Benjamini**
	GOTERM_BP_FAT	microtubule cytoskeleton organization	21	1.50E-01	9.80E-01
	GOTERM_BP_FAT	microtubule-based process	32	2.20E-01	9.90E-01
	GOTERM_BP_FAT	cytoskeleton organization	48	4.80E-01	1.00E+00
**Annotation Cluster 97**	**Enrichment Score: 0.6**		**Count**	**P_Value**	**Benjamini**
	SP_PIR_KEYWORDS	cytoplasmic vesicle	32	1.20E-01	8.50E-01
	GOTERM_CC_FAT	vesicle	78	2.70E-01	9.50E-01
	GOTERM_CC_FAT	membrane-bounded vesicle	66	3.10E-01	9.50E-01
	GOTERM_CC_FAT	cytoplasmic membrane-bounded vesicle	64	3.10E-01	9.50E-01
	GOTERM_CC_FAT	cytoplasmic vesicle	74	3.20E-01	9.50E-01
**Annotation Cluster 98**	**Enrichment Score: 0.6**		**Count**	**P_Value**	**Benjamini**
	GOTERM_BP_FAT	G1/S transition of mitotic cell cycle	11	6.80E-02	9.80E-01
	GOTERM_BP_FAT	interphase of mitotic cell cycle	14	2.90E-01	9.90E-01
	GOTERM_BP_FAT	interphase	14	3.30E-01	9.90E-01
	GOTERM_BP_FAT	mitotic cell cycle	39	6.10E-01	1.00E+00
**Annotation Cluster 99**	**Enrichment Score: 0.59**		**Count**	**P_Value**	**Benjamini**
	SP_PIR_KEYWORDS	phenylalanine catabolism	3	1.20E-01	8.50E-01
	GOTERM_BP_FAT	L-phenylalanine metabolic process	3	1.30E-01	9.80E-01
	GOTERM_BP_FAT	L-phenylalanine catabolic process	3	1.30E-01	9.80E-01
	GOTERM_BP_FAT	tyrosine metabolic process	3	2.50E-01	9.90E-01
	GOTERM_BP_FAT	aromatic amino acid family catabolic process	3	3.70E-01	1.00E+00
	GOTERM_BP_FAT	aromatic amino acid family metabolic process	3	6.10E-01	1.00E+00
	GOTERM_BP_FAT	aromatic compound catabolic process	3	6.40E-01	1.00E+00
**Annotation Cluster 100**	**Enrichment Score: 0.59**		**Count**	**P_Value**	**Benjamini**
	SP_PIR_KEYWORDS	signal transduction	7	1.50E-01	8.70E-01
	BIOCARTA	How Progesterone Initiates the Oocyte Maturation	5	1.60E-01	9.70E-01
	SP_PIR_KEYWORDS	heterotrimer	6	1.60E-01	8.70E-01
	BIOCARTA	Signaling Pathway from G-Protein Families	5	1.80E-01	9.70E-01
	GOTERM_CC_FAT	heterotrimeric G-protein complex	4	6.20E-01	9.70E-01
	GOTERM_CC_FAT	extrinsic to plasma membrane	6	6.40E-01	9.80E-01
**Annotation Cluster 101**	**Enrichment Score: 0.59**		**Count**	**P_Value**	**Benjamini**
	GOTERM_MF_FAT	cytoskeletal protein binding	64	1.00E-01	9.40E-01
	GOTERM_MF_FAT	actin binding	38	3.80E-01	9.90E-01
	SP_PIR_KEYWORDS	actin-binding	28	4.40E-01	9.50E-01
**Annotation Cluster 102**	**Enrichment Score: 0.58**		**Count**	**P_Value**	**Benjamini**
	GOTERM_CC_FAT	microtubule cytoskeleton	66	2.20E-01	9.40E-01
	GOTERM_CC_FAT	microtubule	34	2.70E-01	9.60E-01
	SP_PIR_KEYWORDS	microtubule	28	3.00E-01	9.20E-01
**Annotation Cluster 103**	**Enrichment Score: 0.58**		**Count**	**P_Value**	**Benjamini**
	GOTERM_BP_FAT	negative regulation of protein modification process	19	7.70E-02	9.80E-01
	GOTERM_BP_FAT	regulation of cellular protein metabolic process	60	1.10E-01	9.70E-01
	GOTERM_BP_FAT	regulation of protein modification process	39	1.20E-01	9.70E-01
	GOTERM_BP_FAT	positive regulation of protein metabolic process	28	4.30E-01	1.00E+00

ILMN_1657612	RPL6	0.730740614
ILMN_1737015	RPL39	0.730398658
ILMN_1802252	GAPDH	0.730307584
ILMN_1783303	HSP90AB4P	0.729849286
ILMN_1658351	FIS1	0.729722964
ILMN_1693233	KIAA0513	0.729509954
ILMN_1740385	CEP164	0.729366497
ILMN_1808395	ACAP1	0.72924889
ILMN_1785424	ABLIM1	0.72923244
ILMN_1686623	CSF1R	0.729178342
ILMN_2398388	APH1A	0.729146156
ILMN_2405009	C1orf151	0.728968729
ILMN_1803476	KCTD20	0.728749607
ILMN_1685415	HBP1	0.728599642
ILMN_1790692	GNLY	0.728549165
ILMN_1660376	RPL13A	0.728529068
ILMN_1727249	FAM21C	0.728160083
ILMN_2203950	HLA-A	0.72790687
ILMN_1745112	FAM102A	0.727886698
ILMN_1800451	MED16	0.727865169
ILMN_1651767	MKL1	0.72777291
ILMN_3279306	RPL17	0.727761711
ILMN_1652313	RNF215	0.727662846
ILMN_1752802	CLPTM1L	0.727652757
ILMN_1688381	BPTF	0.727549807
ILMN_1689456	ZBTB20	0.727509872
ILMN_2328224	MADD	0.727414894
ILMN_3302905	RPL23A	0.727227199
ILMN_1742238	SET	0.727091517
ILMN_1797005	PGLS	0.727061288
ILMN_1726169	EDF1	0.7270302
ILMN_1747589	HIST2H2AB	0.726897749
ILMN_1730797	RUNX1	0.726892018
ILMN_1736597	TKT	0.726868258
ILMN_1719972	PLXNA3	0.7267651
ILMN_1682993	NKG7	0.726748877
ILMN_2415179	CLSTN1	0.726714589
ILMN_1762787	RNF26	0.726667157
ILMN_1798867	CAMTA2	0.726639999
ILMN_1708991	CCNY	0.726567618
ILMN_2362368	U2AF1	0.726549068
ILMN_1812191	C12orf57	0.726548015
ILMN_1801504	RUNX1	0.726522574
ILMN_1669607	PHKG2	0.726478066
ILMN_1703866	SUPT5H	0.726406396
ILMN_1686725	DICER1	0.726314052
ILMN_2188533	RPL12	0.726058474
ILMN_2360710	TPM1	0.725850368
ILMN_3205656	GAPDH	0.725803486
ILMN_1735788	TRIOBP	0.725778112
ILMN_1771651	MON1B	0.725753881
ILMN_2393765	IGLL1	0.725656482
ILMN_2319996	RPL3	0.725546927
ILMN_1735827	NISCH	0.725520364
ILMN_1752755	VWF	0.725387195
ILMN_1798081	PTPRF	0.725266032
ILMN_1812616	MYO1C	0.725145118
ILMN_1785061	EPHB2	0.724979615
ILMN_1699887	ST14	0.724950309
ILMN_1803772	POLD4	0.724915578
ILMN_1675222	PLXND1	0.724852498
ILMN_1695334	PYGO2	0.72482679
ILMN_1809003	CBR1	0.724779287
ILMN_1804327	NAP1L4	0.724721866
ILMN_1790782	MED16	0.724694927
ILMN_1804652	PLEKHH3	0.724647986
ILMN_1784299	C19orf43	0.724614574
ILMN_2300396	COMMD5	0.724577438
ILMN_3242293	SPDYE7P	0.724523554
ILMN_1756674	ATP5E	0.724508499
ILMN_1718607	TSPAN4	0.724488193
ILMN_2167416	MR1	0.724215407
ILMN_2154115	PSD4	0.724198124
ILMN_1794781	VAV2	0.724195197
ILMN_1761442	WDR24	0.724169156
ILMN_1658206	OSCP1	0.724032736
ILMN_1653652	PTPRC	0.724031071
ILMN_1660810	TTC17	0.72373663
ILMN_1689445	POLR2H	0.723525328
ILMN_2393712	CTTN	0.723113826
ILMN_1730777	KRT19	0.722962193
ILMN_1708151	LAGE3	0.722882173
ILMN_1722102	ANAPC11	0.72282853
ILMN_1713990	TRIP6	0.722752855
ILMN_1789616	NUPL2	0.722659607
ILMN_1709809	NHP2L1	0.722440577
ILMN_1703305	TWF2	0.722303972
ILMN_1343295	GAPDH	0.722259911
ILMN_1782633	BOLA2B	0.722248619
ILMN_3251404	NUCKS1	0.722149451
ILMN_3235254	SPDYE8P	0.721926034
ILMN_2383489	CDK5RAP3	0.721677057
ILMN_3211187	TUBB2A	0.721569085
ILMN_2399328	COBRA1	0.72152988
ILMN_1686082	C14orf79	0.721512515
ILMN_1802458	AGTRAP	0.721494231

	GOTERM_BP_FAT	response to wounding	81	1.10E-01	7.00E-01
**Annotation Cluster 81**	**Enrichment Score: 1.23**		**Count**	**P_Value**	**Benjamini**
	SP_PIR_KEYWORDS	methylated carboxyl end	6	3.80E-03	4.70E-02
	SP_PIR_KEYWORDS	ADP-ribosylation	10	1.40E-02	1.20E-01
	INTERPRO	Ras small GTPase, Rho type	6	9.80E-02	9.70E-01
	SMART	RHO	6	1.10E-01	8.10E-01
	GOTERM_BP_FAT	regulation of cell projection assembly	5	2.10E-01	8.70E-01
	PIR_SUPERFAMILY	PIRSF037169:small GTPase, Rho type	4	3.30E-01	1.00E+00
**Annotation Cluster 82**	**Enrichment Score: 1.22**		**Count**	**P_Value**	**Benjamini**
	GOTERM_BP_FAT	regulation of actin filament polymerization	15	8.80E-03	1.80E-01
	GOTERM_BP_FAT	regulation of actin cytoskeleton organization	21	1.10E-02	2.10E-01
	GOTERM_BP_FAT	regulation of actin polymerization or depolymerization	16	1.10E-02	2.10E-01
	GOTERM_BP_FAT	regulation of actin filament length	16	1.50E-02	2.50E-01
	GOTERM_BP_FAT	regulation of actin filament-based process	21	1.60E-02	2.60E-01
	GOTERM_BP_FAT	negative regulation of cellular component organization	29	1.80E-02	2.80E-01
	GOTERM_BP_FAT	barbed-end actin filament capping	4	1.90E-02	2.80E-01
	GOTERM_BP_FAT	negative regulation of actin filament polymerization	9	2.40E-02	3.30E-01
	GOTERM_BP_FAT	negative regulation of protein complex assembly	10	2.80E-02	3.70E-01
	GOTERM_BP_FAT	negative regulation of protein polymerization	9	3.00E-02	3.80E-01
	GOTERM_BP_FAT	regulation of cellular component biogenesis	28	3.10E-02	3.90E-01
	GOTERM_BP_FAT	regulation of cytoskeleton organization	27	3.20E-02	3.90E-01
	GOTERM_BP_FAT	regulation of protein polymerization	15	5.80E-02	5.40E-01
	GOTERM_BP_FAT	regulation of cellular component size	46	5.90E-02	5.40E-01
	UP_SEQ_FEATURE	repeat:Gelsolin-like 2	4	6.20E-02	1.00E+00
	UP_SEQ_FEATURE	repeat:Gelsolin-like 1	4	6.20E-02	1.00E+00
	UP_SEQ_FEATURE	repeat:Gelsolin-like 3	4	6.20E-02	1.00E+00
	INTERPRO	Gelsolin	4	6.90E-02	9.50E-01
	SMART	GEL	4	7.70E-02	7.60E-01
	GOTERM_BP_FAT	regulation of organelle organization	37	8.50E-02	6.40E-01
	INTERPRO	Gelsolin region	5	9.00E-02	9.60E-01
	GOTERM_BP_FAT	negative regulation of cytoskeleton organization	12	1.00E-01	6.90E-01
	GOTERM_BP_FAT	negative regulation of organelle organization	16	1.20E-01	7.20E-01
	GOTERM_BP_FAT	regulation of protein complex assembly	17	1.30E-01	7.60E-01
	GOTERM_BP_FAT	actin filament capping	6	1.50E-01	8.00E-01
	GOTERM_BP_FAT	negative regulation of actin filament depolymerization	6	2.00E-01	8.60E-01
	UP_SEQ_FEATURE	repeat:Gelsolin-like 4	3	2.00E-01	1.00E+00
	UP_SEQ_FEATURE	repeat:Gelsolin-like 5	3	2.00E-01	1.00E+00
	GOTERM_BP_FAT	regulation of protein complex disassembly	10	2.20E-01	8.90E-01
	GOTERM_BP_FAT	regulation of actin filament depolymerization	6	2.80E-01	9.30E-01
	GOTERM_BP_FAT	negative regulation of protein complex disassembly	8	2.90E-01	9.30E-01
	SP_PIR_KEYWORDS	actin capping	4	4.10E-01	8.40E-01
**Annotation Cluster 83**	**Enrichment Score: 1.2**		**Count**	**P_Value**	**Benjamini**
	GOTERM_MF_FAT	protein complex scaffold	8	3.40E-02	5.00E-01
	GOTERM_MF_FAT	MAP-kinase scaffold activity	3	8.30E-02	7.40E-01
	GOTERM_MF_FAT	receptor signaling complex scaffold activity	6	8.70E-02	7.50E-01
**Annotation Cluster 84**	**Enrichment Score: 1.2**		**Count**	**P_Value**	**Benjamini**
	GOTERM_BP_FAT	cell cycle	137	2.30E-04	1.40E-02
	GOTERM_BP_FAT	cell cycle process	104	3.20E-04	1.80E-02
	GOTERM_BP_FAT	mitotic cell cycle	67	6.00E-03	1.40E-01
	GOTERM_BP_FAT	cell division	50	5.10E-02	5.00E-01
	SP_PIR_KEYWORDS	cell cycle	68	5.90E-02	3.30E-01
	SP_PIR_KEYWORDS	cell division	41	7.50E-02	3.70E-01
	SP_PIR_KEYWORDS	mitosis	27	2.10E-01	6.40E-01
	GOTERM_BP_FAT	cell cycle phase	59	3.30E-01	9.50E-01
	GOTERM_BP_FAT	organelle fission	32	4.70E-01	9.80E-01
	GOTERM_BP_FAT	M phase	45	4.80E-01	9.90E-01
	GOTERM_BP_FAT	M phase of mitotic cell cycle	31	4.90E-01	9.90E-01
	GOTERM_BP_FAT	mitosis	30	5.30E-01	9.90E-01
	GOTERM_BP_FAT	nuclear division	30	5.30E-01	9.90E-01
**Annotation Cluster 85**	**Enrichment Score: 1.2**		**Count**	**P_Value**	**Benjamini**
	GOTERM_BP_FAT	response to nutrient	29	1.50E-02	2.50E-01
	GOTERM_BP_FAT	response to nutrient levels	35	5.90E-02	5.40E-01
	GOTERM_BP_FAT	response to extracellular stimulus	38	7.00E-02	5.80E-01
	GOTERM_BP_FAT	response to vitamin	12	2.50E-01	9.00E-01
**Annotation Cluster 86**	**Enrichment Score: 1.2**		**Count**	**P_Value**	**Benjamini**
	GOTERM_CC_FAT	nuclear chromatin	15	1.20E-02	1.40E-01
	GOTERM_CC_FAT	heterochromatin	12	1.60E-02	1.70E-01
	GOTERM_CC_FAT	nuclear heterochromatin	8	8.30E-02	4.60E-01
	GOTERM_CC_FAT	nuclear chromosome part	22	1.30E-01	5.60E-01
	GOTERM_CC_FAT	chromosomal part	59	1.80E-01	6.40E-01
	GOTERM_CC_FAT	nuclear chromosome	27	1.80E-01	6.40E-01
**Annotation Cluster 87**	**Enrichment Score: 1.16**		**Count**	**P_Value**	**Benjamini**
	GOTERM_BP_FAT	lymphocyte proliferation	11	4.30E-02	4.60E-01
	GOTERM_BP_FAT	mononuclear cell proliferation	11	5.70E-02	5.30E-01
	GOTERM_BP_FAT	leukocyte proliferation	11	5.70E-02	5.30E-01
	GOTERM_BP_FAT	B cell proliferation	5	8.10E-02	6.30E-01
	GOTERM_BP_FAT	T cell proliferation	7	1.40E-01	7.70E-01
**Annotation Cluster 88**	**Enrichment Score: 1.16**		**Count**	**P_Value**	**Benjamini**
	GOTERM_BP_FAT	regulation of endocytosis	15	2.50E-02	3.40E-01
	GOTERM_BP_FAT	regulation of vesicle-mediated transport	19	7.60E-02	6.10E-01
	GOTERM_BP_FAT	positive regulation of endocytosis	7	1.70E-01	8.30E-01
**Annotation Cluster 89**	**Enrichment Score: 1.15**		**Count**	**P_Value**	**Benjamini**
	GOTERM_BP_FAT	negative regulation of protein kinase activity	18	6.10E-02	5.50E-01
	GOTERM_BP_FAT	negative regulation of transferase activity	19	7.60E-02	6.10E-01
	GOTERM_BP_FAT	negative regulation of kinase activity	18	7.90E-02	6.10E-01
**Annotation Cluster 90**	**Enrichment Score: 1.13**		**Count**	**P_Value**	**Benjamini**
	UP_SEQ_FEATURE	domain:VWFA	13	6.60E-02	1.00E+00
	INTERPRO	von Willebrand factor, type A	16	6.80E-02	9.50E-01
	SMART	VWA	16	9.10E-02	7.90E-01
**Annotation Cluster 91**	**Enrichment Score: 1.13**		**Count**	**P_Value**	**Benjamini**
	GOTERM_BP_FAT	immune response-regulating signal transduction	14	2.80E-02	3.60E-01
	GOTERM_BP_FAT	activation of immune response	20	3.60E-02	4.20E-01
	GOTERM_BP_FAT	immune response-regulating cell surface receptor signaling pathway	11	4.30E-02	4.60E-01
	GOTERM_BP_FAT	antigen receptor-mediated signaling pathway	9	6.10E-02	5.50E-01
	GOTERM_BP_FAT	immune response-activating signal transduction	12	7.40E-02	6.00E-01
	GOTERM_BP_FAT	B cell receptor signaling pathway	4	1.00E-01	7.00E-01

ILMN_1712523	MAP6	-0.432305905
ILMN_1798557	FAM153A	-0.432401124
ILMN_2172933	LCN12	-0.432421757
ILMN_3272764	NA	-0.432462818
ILMN_2312106	PRAMEF10	-0.432544283
ILMN_1750974	S100A9	-0.432548586
ILMN_1777905	NA	-0.432598373
ILMN_1778717	DBF4	-0.432717105
ILMN_1700306	OCIAD2	-0.432744868
ILMN_3308235	MIR608	-0.432790709
ILMN_1664660	GAGE8	-0.432836359
ILMN_2357730	CDK20	-0.432837531
ILMN_1668012	SLC25A13	-0.432843898
ILMN_3201163	NA	-0.432919028
ILMN_1668740	LOC100287221	-0.432979918
ILMN_1838702	BG188175	-0.433001122
ILMN_2343563	ANAPC11	-0.433057001
ILMN_1828245	AW070200	-0.433082296
ILMN_3251625	ZNF503	-0.433103876
ILMN_1762526	SMC1B	-0.433236043
ILMN_2120917	CTR9	-0.433242368
ILMN_1828083	DB338706	-0.433270795
ILMN_3231080	FAM182A	-0.433273624
ILMN_1792138	UQCRH	-0.43328664
ILMN_3308505	MIR129-2	-0.433300545
ILMN_1785650	OR11L1	-0.433389271
ILMN_3297675	GATSL2	-0.433393599
ILMN_1814429	EYS	-0.433400488
ILMN_1662184	C5orf34	-0.433411696
ILMN_1881783	BX090917	-0.433445686
ILMN_1745887	FBXO21	-0.433455559
ILMN_1808110	TCEB3	-0.433517372
ILMN_1707816	ZNF527	-0.433523923
ILMN_1801891	HDX	-0.43360293
ILMN_1713983	NCRNA00169	-0.433645523
ILMN_1785564	FGF16	-0.433686112
ILMN_1781221	SPDYA	-0.433733857
ILMN_1757384	RAN	-0.433743261
ILMN_2402947	USP14	-0.433817481
ILMN_1804597	DCAF17	-0.433840165
ILMN_3183170	AK126852	-0.433857008
ILMN_2411935	KREMEN2	-0.433943196
ILMN_1697512	SLC32A1	-0.433946007
ILMN_3291253	SBF1P1	-0.43399392
ILMN_1681304	PAN3	-0.434075855
ILMN_1663980	MMP19	-0.434109157
ILMN_1915752	BX119185	-0.434138242
ILMN_2327181	GEMIN7	-0.434187009
ILMN_1742131	CPNE7	-0.434220155
ILMN_3277282	LOC115110	-0.434238359
ILMN_2124236	C3orf42	-0.434250906
ILMN_1676893	ADCY3	-0.434311164
ILMN_1705225	PRTG	-0.434342192
ILMN_1796629	EDNRA	-0.434347015
ILMN_2399769	WLS	-0.434353462
ILMN_2115669	SEMA4C	-0.434361155
ILMN_1678718	AK307134	-0.434406058
ILMN_1741200	RFX5	-0.434442976
ILMN_1655387	PRDM16	-0.434472183
ILMN_1767960	NSUN7	-0.434526268
ILMN_1832209	AA906426	-0.434534979
ILMN_1739659	ZDHHC6	-0.434605591
ILMN_2179739	HCRT	-0.434678465
ILMN_1707954	N39814	-0.434683301
ILMN_1689098	ATP1A3	-0.434720181
ILMN_3307616	EPHA10	-0.434737165
ILMN_3301502	BC112987	-0.434768092
ILMN_1667405	C9orf100	-0.434890558
ILMN_1736453	RCVRN	-0.434899172
ILMN_1913498	BM665736	-0.434907575
ILMN_2063584	CLIC4	-0.434969808
ILMN_3310955	MIR758	-0.435009197
ILMN_1849997	AK123174	-0.435027152
ILMN_1786989	NKX6-2	-0.435167323
ILMN_1772055	VARS	-0.435170074
ILMN_1670189	FAM21C	-0.435187275
ILMN_2228780	SLC5A11	-0.435200944
ILMN_3206986	NA	-0.435255744
ILMN_1780773	LOC400027	-0.435260464
ILMN_1725485	RGS17	-0.435276808
ILMN_1686832	NR6A1	-0.43546439
ILMN_1797367	TSC1	-0.435579515
ILMN_1694389	MYL10	-0.435609902
ILMN_2077008	WDR38	-0.435614998
ILMN_1804090	SLC25A10	-0.435648776
ILMN_2224230	TTTY21	-0.435666477
ILMN_1773835	ZNF585B	-0.435692079
ILMN_1680963	LOC100132686	-0.43569364
ILMN_1833415	BC021736	-0.435768477
ILMN_2136446	CTNNAL1	-0.435792767
ILMN_1711839	AK130343	-0.435813286
ILMN_1657607	NRG1	-0.435853208
ILMN_1914707	DA886662	-0.43590821
ILMN_1742787	BHLHE23	-0.435950728
ILMN_1799969	SNAPIN	-0.435967944
ILMN_2341229	CD34	-0.435985153

	GOTERM_BP_FAT	positive regulation of protein modification process	21	5.20E-01	1.00E+00
	GOTERM_BP_FAT	positive regulation of cellular protein metabolic process	25	5.90E-01	1.00E+00
	GOTERM_BP_FAT	positive regulation of protein ubiquitination	9	6.80E-01	1.00E+00
**Annotation Cluster 104**	**Enrichment Score: 0.58**		**Count**	**P_Value**	**Benjamini**
	GOTERM_BP_FAT	energy reserve metabolic process	9	7.90E-02	9.80E-01
	SP_PIR_KEYWORDS	carbohydrate metabolism	13	9.60E-02	8.40E-01
	SP_PIR_KEYWORDS	glycogen metabolism	5	1.90E-01	8.90E-01
	SP_PIR_KEYWORDS	glycogen storage disease	4	2.60E-01	9.00E-01
	GOTERM_BP_FAT	glycogen metabolic process	6	3.10E-01	9.90E-01
	GOTERM_BP_FAT	glucan metabolic process	6	3.30E-01	9.90E-01
	GOTERM_BP_FAT	cellular glucan metabolic process	6	3.30E-01	9.90E-01
	GOTERM_BP_FAT	energy derivation by oxidation of organic compounds	18	3.60E-01	1.00E+00
	GOTERM_BP_FAT	cellular polysaccharide metabolic process	7	5.00E-01	1.00E+00
	GOTERM_BP_FAT	polysaccharide metabolic process	11	7.50E-01	1.00E+00
**Annotation Cluster 105**	**Enrichment Score: 0.58**		**Count**	**P_Value**	**Benjamini**
	INTERPRO	SM22/calponin	4	1.00E-01	1.00E+00
	INTERPRO	Calponin repeat	3	1.30E-01	1.00E+00
	UP_SEQ_FEATURE	domain:CH	7	2.80E-01	1.00E+00
	SMART	CH	9	3.90E-01	9.70E-01
	INTERPRO	Calponin-like actin-binding	9	4.70E-01	1.00E+00
	COG_ONTOLOGY	Cytoskeleton	4	5.40E-01	9.70E-01
**Annotation Cluster 106**	**Enrichment Score: 0.57**		**Count**	**P_Value**	**Benjamini**
	GOTERM_BP_FAT	chromatin assembly or disassembly	20	7.70E-02	9.80E-01
	GOTERM_BP_FAT	chromatin organization	49	1.10E-01	9.70E-01
	GOTERM_BP_FAT	chromosome organization	58	2.20E-01	9.90E-01
	GOTERM_BP_FAT	covalent chromatin modification	15	4.70E-01	1.00E+00
	GOTERM_BP_FAT	chromatin modification	30	5.30E-01	1.00E+00
	SP_PIR_KEYWORDS	chromatin regulator	20	8.00E-01	9.90E-01
**Annotation Cluster 107**	**Enrichment Score: 0.57**		**Count**	**P_Value**	**Benjamini**
	SP_PIR_KEYWORDS	iron transport	6	8.50E-02	8.60E-01
	GOTERM_BP_FAT	cellular iron ion homeostasis	5	4.20E-01	1.00E+00
	GOTERM_BP_FAT	iron ion homeostasis	5	5.40E-01	1.00E+00
**Annotation Cluster 108**	**Enrichment Score: 0.57**		**Count**	**P_Value**	**Benjamini**
	UP_SEQ_FEATURE	repeat:Solcar 1	10	1.10E-01	1.00E+00
	UP_SEQ_FEATURE	repeat:Solcar 2	10	1.10E-01	1.00E+00
	INTERPRO	Mitochondrial substrate/solute carrier	10	1.30E-01	1.00E+00
	PIR_SUPERFAMILY	PIRSF002458:ADP,ATP carrier protein	5	4.10E-01	1.00E+00
	UP_SEQ_FEATURE	repeat:Solcar 3	7	4.50E-01	1.00E+00
	INTERPRO	Mitochondrial carrier protein	4	5.60E-01	1.00E+00
	INTERPRO	Mitochondrial substrate carrier	6	6.60E-01	1.00E+00
**Annotation Cluster 109**	**Enrichment Score: 0.57**		**Count**	**P_Value**	**Benjamini**
	GOTERM_MF_FAT	non-membrane spanning protein tyrosine kinase activity	9	6.60E-02	8.80E-01
	PIR_SUPERFAMILY	PIRSF000601:tyrosine-protein kinase, proto-oncogene SRC type	4	1.30E-01	1.00E+00
	SMART	SH2	13	4.20E-01	9.80E-01
	UP_SEQ_FEATURE	domain:SH2	12	4.30E-01	1.00E+00
	SP_PIR_KEYWORDS	SH2 domain	13	5.00E-01	9.60E-01
	INTERPRO	SH2 motif	13	5.20E-01	1.00E+00
**Annotation Cluster 110**	**Enrichment Score: 0.56**		**Count**	**P_Value**	**Benjamini**
	KEGG_PATHWAY	Starch and sucrose metabolism	9	9.00E-02	9.20E-01
	INTERPRO	Glycoside hydrolase, subgroup, catalytic core	6	2.70E-01	1.00E+00
	SP_PIR_KEYWORDS	glycosidase	7	8.50E-01	9.90E-01
**Annotation Cluster 111**	**Enrichment Score: 0.56**		**Count**	**P_Value**	**Benjamini**
	GOTERM_MF_FAT	identical protein binding	77	1.80E-01	9.80E-01
	GOTERM_MF_FAT	protein homodimerization activity	42	2.00E-01	9.80E-01
	GOTERM_MF_FAT	protein dimerization activity	58	5.70E-01	1.00E+00
**Annotation Cluster 112**	**Enrichment Score: 0.56**		**Count**	**P_Value**	**Benjamini**
	GOTERM_MF_FAT	cyclic nucleotide binding	5	2.30E-01	9.80E-01
	SMART	cNMP	6	2.60E-01	9.60E-01
	INTERPRO	Cyclic nucleotide-binding, conserved site	6	2.70E-01	1.00E+00
	INTERPRO	RmlC-like jelly roll fold	6	3.10E-01	1.00E+00
	INTERPRO	Cyclic nucleotide-binding	6	3.10E-01	1.00E+00
**Annotation Cluster 113**	**Enrichment Score: 0.56**		**Count**	**P_Value**	**Benjamini**
	GOTERM_BP_FAT	central nervous system neuron axonogenesis	4	2.10E-01	9.90E-01
	GOTERM_BP_FAT	central nervous system neuron development	6	2.50E-01	9.90E-01
	GOTERM_BP_FAT	central nervous system neuron differentiation	6	4.20E-01	1.00E+00
**Annotation Cluster 114**	**Enrichment Score: 0.55**		**Count**	**P_Value**	**Benjamini**
	UP_SEQ_FEATURE	short sequence motif:DEAH box	9	6.80E-02	1.00E+00
	UP_SEQ_FEATURE	domain:Chromo 2	3	2.40E-01	1.00E+00
	UP_SEQ_FEATURE	domain:Chromo 1	3	3.60E-01	1.00E+00
	SMART	CHROMO	5	3.90E-01	9.70E-01
	INTERPRO	Chromo domain	5	4.40E-01	1.00E+00
	INTERPRO	SNF2-related	5	4.40E-01	1.00E+00
**Annotation Cluster 115**	**Enrichment Score: 0.55**		**Count**	**P_Value**	**Benjamini**
	PIR_SUPERFAMILY	PIRSF038059:suppressor of cytokine signaling (SOCS) 4-7	3	3.20E-02	1.00E+00
	SMART	SOCS	6	3.20E-01	9.70E-01
	UP_SEQ_FEATURE	domain:SOCS box	6	3.70E-01	1.00E+00
	INTERPRO	SOCS protein, C-terminal	6	3.80E-01	1.00E+00
	SP_PIR_KEYWORDS	Signal transduction inhibitor	5	5.30E-01	9.70E-01
	SP_PIR_KEYWORDS	growth regulation	6	6.60E-01	9.80E-01
**Annotation Cluster 116**	**Enrichment Score: 0.55**		**Count**	**P_Value**	**Benjamini**
	GOTERM_BP_FAT	striated muscle cell differentiation	18	1.00E-02	8.30E-01
	GOTERM_BP_FAT	muscle cell development	8	3.80E-01	1.00E+00
	GOTERM_BP_FAT	striated muscle cell development	7	4.80E-01	1.00E+00
	GOTERM_BP_FAT	skeletal muscle fiber development	4	5.80E-01	1.00E+00
	GOTERM_BP_FAT	neuromuscular junction development	3	6.40E-01	1.00E+00
	GOTERM_BP_FAT	muscle fiber development	4	7.30E-01	1.00E+00
**Annotation Cluster 117**	**Enrichment Score: 0.55**		**Count**	**P_Value**	**Benjamini**
	GOTERM_BP_FAT	DNA metabolic process	61	1.90E-01	9.90E-01
	GOTERM_BP_FAT	DNA repair	36	1.90E-01	9.80E-01
	GOTERM_BP_FAT	response to DNA damage stimulus	46	2.00E-01	9.80E-01
	SP_PIR_KEYWORDS	DNA damage	25	3.10E-01	9.20E-01
	GOTERM_BP_FAT	cellular response to stress	63	4.10E-01	1.00E+00
	SP_PIR_KEYWORDS	dna repair	21	5.30E-01	9.70E-01
**Annotation Cluster 118**	**Enrichment Score: 0.54**		**Count**	**P_Value**	**Benjamini**
	GOTERM_BP_FAT	neurotransmitter metabolic process	5	2.00E-01	9.90E-01
	GOTERM_BP_FAT	neurotransmitter biosynthetic process	3	2.00E-01	9.90E-01

ILMN_1805812	TOR1A	0.72135148
ILMN_1683597	RPL23A	0.721261586
ILMN_2399140	RAB5C	0.721179209
ILMN_2312709	LCMT1	0.721169216
ILMN_1724532	C11orf49	0.721095522
ILMN_1656420	C16orf67	0.721013331
ILMN_1760493	LIMS2	0.720817288
ILMN_1653404	NKIRAS2	0.720628198
ILMN_1664706	HIST2H3D	0.720585865
ILMN_1765082	RBM10	0.720585208
ILMN_1770281	ERC1	0.720505797
ILMN_1657750	CNNM1	0.720505016
ILMN_2353642	ATP6V0B	0.720438369
ILMN_1813836	DARS	0.720315758
ILMN_1778111	FLJ45340	0.720312126
ILMN_2246894	EDF1	0.719921976
ILMN_3219340	RPL29	0.719689217
ILMN_1764770	OXNAD1	0.719612125
ILMN_2325763	VCAM1	0.719611096
ILMN_1656920	CRIP1	0.719097703
ILMN_1804854	CTNNA1	0.719036017
ILMN_1766125	LONP1	0.718863713
ILMN_1792571	ARHGEF15	0.718239915
ILMN_1713884	C16orf42	0.718139537
ILMN_3244029	ANKRD52	0.718026121
ILMN_3238570	EIF3CL	0.717894547
ILMN_1693430	NME1	0.717879579
ILMN_1723871	OTUB1	0.717731459
ILMN_1728845	SMARCD1	0.717617061
ILMN_1676792	RPS11	0.717603756
ILMN_1716913	TRAPPC1	0.717575633
ILMN_2252309	DPP7	0.717544221
ILMN_1779886	TBC1D14	0.717473712
ILMN_2402806	TRPC4AP	0.717451582
ILMN_1800843	SCAMP4	0.71744772
ILMN_2367384	EPHB2	0.717412277
ILMN_1812559	SLC7A6	0.71739314
ILMN_1773620	SMARCC2	0.717355946
ILMN_1745963	FOLR2	0.717254042
ILMN_1733863	FAM100A	0.71688483
ILMN_2358652	NXF1	0.716562212
ILMN_1679838	WBP5	0.716502688
ILMN_1663132	ADCK2	0.716461666
ILMN_1788689	PHIP	0.716431965
ILMN_2353697	HINFP	0.716148564
ILMN_1778560	ZNF3	0.716094649
ILMN_1801121	SENP2	0.716066822
ILMN_1814799	S100A13	0.716053904
ILMN_2336186	LCMT1	0.715848835
ILMN_2083789	C14orf105	0.715831144
ILMN_1706992	ADAMTSL1	0.715713169
ILMN_1655418	CAPNS1	0.715669409
ILMN_2233256	ZNF607	0.715587836
ILMN_1657095	STARD3	0.715563051
ILMN_1774844	MAPKAPK2	0.715444497
ILMN_2067370	SNRPF	0.715404011
ILMN_1663667	FLAD1	0.715398763
ILMN_1667432	HYAL3	0.715337448
ILMN_1794677	TMC6	0.715169627
ILMN_1731991	SCYL1	0.715144378
ILMN_1722900	EIF4A1	0.715052278
ILMN_2302716	ALDH18A1	0.714881515
ILMN_1700147	VPREB3	0.714847053
ILMN_1708728	H2AFJ	0.71481995
ILMN_1739222	ETV5	0.714815334
ILMN_2053546	RPL19	0.714641707
ILMN_2410771	KEAP1	0.714606607
ILMN_1753275	DENND1A	0.714537554
ILMN_1696749	LMNA	0.714461714
ILMN_1713006	CDC34	0.714268583
ILMN_1695851	PARVG	0.714081512
ILMN_3227912	RPL15	0.714067204
ILMN_3233179	HS6ST1	0.713988857
ILMN_1737157	GRAMD1A	0.71391632
ILMN_1685873	ZC3H7B	0.713889087
ILMN_2407434	DCLK2	0.713866359
ILMN_1744665	EP300	0.713856083
ILMN_1659029	FAM116B	0.713813112
ILMN_1664614	FAU	0.713670468
ILMN_2399694	NOMO3	0.713581054
ILMN_2396148	HIP1R	0.713567092
ILMN_3303101	AK027616	0.713366175
ILMN_2377980	PPP1CA	0.713348549
ILMN_1754199	BCL7B	0.713209495
ILMN_3307827	DNAJC4	0.713067655
ILMN_1739450	NFE2L1	0.712963889
ILMN_1716608	NGF	0.712878312
ILMN_1703949	KPNB1	0.71281946
ILMN_1716973	POLM	0.712710169
ILMN_1811313	SLIT3	0.712649869
ILMN_1704941	PPCDC	0.712647346
ILMN_2352042	MRPL4	0.712548643
ILMN_1753790	ZNF259	0.712454778

	GOTERM_BP_FAT	immune response-activating cell surface receptor signaling pathway	9	1.30E-03	7.60E-01
	GOTERM_BP_FAT	T cell receptor signaling pathway	5	3.60E-01	9.60E-01
**Annotation Cluster 92**	**Enrichment Score: 1.13**		**Count**	**P_Value**	**Benjamini**
	GOTERM_CC_FAT	leading edge membrane	8	2.60E-02	2.30E-01
	GOTERM_CC_FAT	ruffle membrane	5	6.50E-02	4.00E-01
	GOTERM_CC_FAT	cell projection membrane	12	2.50E-01	7.20E-01
**Annotation Cluster 93**	**Enrichment Score: 1.12**		**Count**	**P_Value**	**Benjamini**
	GOTERM_BP_FAT	positive regulation of axonogenesis	9	1.20E-02	2.20E-01
	GOTERM_BP_FAT	positive regulation of cell growth	11	4.30E-02	4.60E-01
	GOTERM_BP_FAT	positive regulation of cell size	11	9.30E-02	6.60E-01
	GOTERM_BP_FAT	positive regulation of axon extension	4	1.00E-01	7.00E-01
	GOTERM_BP_FAT	regulation of axon extension	6	1.10E-01	7.20E-01
	GOTERM_BP_FAT	positive regulation of developmental growth	5	1.50E-01	7.90E-01
	GOTERM_BP_FAT	positive regulation of growth	15	1.60E-01	8.10E-01
**Annotation Cluster 94**	**Enrichment Score: 1.1**		**Count**	**P_Value**	**Benjamini**
	GOTERM_CC_FAT	cortical cytoskeleton	13	2.50E-02	2.30E-01
	GOTERM_CC_FAT	cell cortex	27	7.50E-02	4.30E-01
	GOTERM_CC_FAT	cell cortex part	16	1.40E-01	5.60E-01
	GOTERM_CC_FAT	cortical actin cytoskeleton	7	1.60E-01	6.10E-01
**Annotation Cluster 95**	**Enrichment Score: 1.09**		**Count**	**P_Value**	**Benjamini**
	GOTERM_BP_FAT	regulation of Ras protein signal transduction	42	6.00E-03	1.40E-01
	INTERPRO	Guanine-nucleotide dissociation stimulator, CDC24, conserved site	14	4.90E-02	9.20E-01
	UP_SEQ_FEATURE	domain:DH	14	5.60E-02	1.00E+00
	GOTERM_BP_FAT	regulation of Rho protein signal transduction	20	5.80E-02	5.40E-01
	INTERPRO	Dbl homology (DH) domain	14	8.90E-02	9.60E-01
	SMART	RhoGEF	14	1.20E-01	8.00E-01
	GOTERM_MF_FAT	Rho guanyl-nucleotide exchange factor activity	14	1.50E-01	8.70E-01
	GOTERM_MF_FAT	Ras guanyl-nucleotide exchange factor activity	16	1.50E-01	8.70E-01
	SP_PIR_KEYWORDS	guanine-nucleotide releasing factor	19	2.00E-01	6.30E-01
	GOTERM_MF_FAT	guanyl-nucleotide exchange factor activity	23	3.10E-01	9.70E-01
**Annotation Cluster 96**	**Enrichment Score: 1.08**		**Count**	**P_Value**	**Benjamini**
	GOTERM_MF_FAT	protein tyrosine kinase activity	32	2.00E-02	3.90E-01
	SP_PIR_KEYWORDS	autophosphorylation	12	2.90E-02	2.00E-01
	INTERPRO	Tyrosine protein kinase	23	3.80E-02	9.10E-01
	SP_PIR_KEYWORDS	tyrosine-specific protein kinase	11	5.70E-02	3.20E-01
	SMART	TyrKc	23	5.70E-02	7.40E-01
	SP_PIR_KEYWORDS	tyrosine-protein kinase	20	7.20E-02	3.60E-01
	GOTERM_MF_FAT	transmembrane receptor protein tyrosine kinase activity	13	1.50E-01	8.70E-01
	GOTERM_MF_FAT	non-membrane spanning protein tyrosine kinase activity	7	4.30E-01	9.90E-01
	INTERPRO	Tyrosine protein kinase, active site	13	5.80E-01	1.00E+00
**Annotation Cluster 97**	**Enrichment Score: 1.08**		**Count**	**P_Value**	**Benjamini**
	GOTERM_BP_FAT	regulation of cell growth	36	3.20E-02	3.90E-01
	GOTERM_BP_FAT	regulation of cellular component size	46	5.90E-02	5.40E-01
	GOTERM_BP_FAT	regulation of cell size	31	3.10E-01	9.40E-01
**Annotation Cluster 98**	**Enrichment Score: 1.06**		**Count**	**P_Value**	**Benjamini**
	GOTERM_MF_FAT	transcription activator activity	78	3.40E-04	3.70E-02
	GOTERM_BP_FAT	positive regulation of macromolecule metabolic process	143	1.90E-03	6.00E-02
	GOTERM_BP_FAT	regulation of transcription from RNA polymerase II promoter	121	4.80E-03	1.20E-01
	GOTERM_BP_FAT	positive regulation of macromolecule biosynthetic process	95	1.90E-01	8.60E-01
	GOTERM_BP_FAT	positive regulation of cellular biosynthetic process	98	2.30E-01	8.90E-01
	GOTERM_BP_FAT	positive regulation of biosynthetic process	99	2.50E-01	9.10E-01
	GOTERM_BP_FAT	positive regulation of nitrogen compound metabolic process	92	2.50E-01	9.10E-01
	GOTERM_BP_FAT	positive regulation of transcription, DNA-dependent	69	2.60E-01	9.10E-01
	GOTERM_BP_FAT	positive regulation of nucleobase, nucleoside, nucleotide and nucleic acid metabolic process	89	2.70E-01	9.20E-01
	GOTERM_BP_FAT	positive regulation of RNA metabolic process	69	2.90E-01	9.30E-01
	GOTERM_BP_FAT	positive regulation of transcription	79	3.50E-01	9.60E-01
	GOTERM_BP_FAT	positive regulation of gene expression	79	4.50E-01	9.80E-01
	GOTERM_BP_FAT	positive regulation of transcription from RNA polymerase II promoter	50	5.20E-01	9.90E-01
**Annotation Cluster 99**	**Enrichment Score: 1.06**		**Count**	**P_Value**	**Benjamini**
	GOTERM_BP_FAT	glucose catabolic process	15	1.70E-02	2.60E-01
	SP_PIR_KEYWORDS	glycolysis	11	3.40E-02	2.30E-01
	BIOCARTA	Glycolysis Pathway	6	4.40E-02	9.10E-01
	GOTERM_BP_FAT	hexose catabolic process	15	6.40E-02	5.60E-01
	GOTERM_BP_FAT	hexose metabolic process	34	6.50E-02	5.70E-01
	SP_PIR_KEYWORDS	gluconeogenesis	6	6.90E-02	3.60E-01
	GOTERM_BP_FAT	monosaccharide catabolic process	15	7.80E-02	6.10E-01
	GOTERM_BP_FAT	glycolysis	11	8.30E-02	6.30E-01
	GOTERM_BP_FAT	cellular carbohydrate catabolic process	17	8.80E-02	6.40E-01
	GOTERM_BP_FAT	glucose metabolic process	27	1.00E-01	6.90E-01
	GOTERM_BP_FAT	alcohol catabolic process	16	1.10E-01	7.00E-01
	GOTERM_BP_FAT	monosaccharide metabolic process	35	2.00E-01	8.60E-01
	GOTERM_BP_FAT	carbohydrate catabolic process	18	2.70E-01	9.20E-01
	KEGG_PATHWAY	Glycolysis / Gluconeogenesis	10	5.60E-01	8.40E-01
**Annotation Cluster 100**	**Enrichment Score: 1.06**		**Count**	**P_Value**	**Benjamini**
	GOTERM_BP_FAT	negative regulation of actin filament polymerization	9	2.40E-02	3.30E-01
	GOTERM_BP_FAT	negative regulation of protein polymerization	9	3.00E-02	3.80E-01
	INTERPRO	Thymosin beta-4, chordata	3	1.20E-01	9.80E-01
	INTERPRO	Thymosin beta-4	3	1.20E-01	9.80E-01
	SMART	THY	3	1.30E-01	8.10E-01
	PIR_SUPERFAMILY	PIRSF001828:Thymosin_beta	3	1.40E-01	1.00E+00
	GOTERM_BP_FAT	sequestering of actin monomers	3	1.80E-01	8.40E-01
**Annotation Cluster 101**	**Enrichment Score: 1.06**		**Count**	**P_Value**	**Benjamini**
	GOTERM_BP_FAT	regulation of inflammatory response	18	1.90E-02	2.80E-01
	GOTERM_BP_FAT	regulation of response to external stimulus	29	6.60E-02	5.70E-01
	GOTERM_BP_FAT	positive regulation of inflammatory response	8	9.10E-02	6.60E-01
	GOTERM_BP_FAT	negative regulation of response to external stimulus	11	1.10E-01	7.20E-01
	GOTERM_BP_FAT	positive regulation of defense response	14	1.60E-01	8.10E-01
	GOTERM_BP_FAT	positive regulation of response to external stimulus	12	2.20E-01	8.80E-01
**Annotation Cluster 102**	**Enrichment Score: 1.04**		**Count**	**P_Value**	**Benjamini**
	GOTERM_BP_FAT	T cell selection	8	8.10E-03	1.70E-01
	GOTERM_BP_FAT	thymic T cell selection	6	2.00E-02	3.00E-01
	BIOCARTA	Activation of Csk by cAMP-dependent Protein Kinase Inhibits Signaling through the T Cell Receptor	8	3.10E-02	9.40E-01
	OMIM_DISEASE	Severe combined immunodeficiency, T cell-negative, B-cell/natural killer-cell positive	3	3.30E-02	1.00E+00

ILMN_1779780	CHTF8	-0.43608213
ILMN_2264634	UTY	-0.436119098
ILMN_1670956	GCOM1	-0.436159586
ILMN_1733116	UBXN7	-0.436176279
ILMN_2225956	C4orf39	-0.436184678
ILMN_1692589	LOC728723	-0.43635144
ILMN_1725683	FUT10	-0.436355785
ILMN_3184724	C7orf58	-0.436471374
ILMN_1666881	AK125965	-0.436521652
ILMN_1691592	SUMF2	-0.436552903
ILMN_1754827	LRRC45	-0.436573051
ILMN_2355042	CLUAP1	-0.436586154
ILMN_1727909	CNTN5	-0.436599502
ILMN_1783443	ALOX15	-0.436611258
ILMN_2056975	HPRT1	-0.436637968
ILMN_1788291	LHFPL5	-0.436647352
ILMN_1748124	TSC22D3	-0.436729844
ILMN_1667948	SPATA18	-0.436771237
ILMN_1813400	CBR4	-0.436800402
ILMN_1743535	PIKFYVE	-0.43682001
ILMN_3272347	AK123101	-0.43691845
ILMN_1680967	SARNP	-0.436926238
ILMN_1684017	GYG2	-0.436933296
ILMN_2083469	IRS2	-0.436986882
ILMN_1783852	CD164	-0.437004022
ILMN_1784595	SBF2	-0.437014345
ILMN_1828111	BE466189	-0.43733202
ILMN_1718352	AK125467	-0.437372516
ILMN_1711102	B3GNT2	-0.437430702
ILMN_1670899	FBN2	-0.4374364
ILMN_1739366	TBR1	-0.437445259
ILMN_1716448	OR10A3	-0.437446785
ILMN_1691175	ADH6	-0.437499867
ILMN_3310366	MIR365-2	-0.437505934
ILMN_1799104	SPAG9	-0.437517658
ILMN_2092589	FAM5C	-0.437519592
ILMN_2260813	TSPYL2	-0.437543188
ILMN_2283814	C21orf34	-0.437632591
ILMN_1895504	LOC100288238	-0.43768678
ILMN_1727558	MRPL27	-0.437761484
ILMN_3247636	SCARNA14	-0.43778702
ILMN_1698769	FAM75D1	-0.437803395
ILMN_1905829	BC063871	-0.437810931
ILMN_1688537	OR10J3	-0.437831073
ILMN_3307858	STIM2	-0.437840772
ILMN_1681643	PCP4L1	-0.437855398
ILMN_3307149	FLJ34503	-0.437869671
ILMN_1768162	LOC100507271	-0.438037956
ILMN_1742798	TRA2B	-0.438080332
ILMN_2082130	C1orf123	-0.438107331
ILMN_1727287	PHF20L1	-0.438162845
ILMN_2166812	KIR2DS5	-0.438172997
ILMN_1818617	AL133627	-0.438185037
ILMN_1717909	ANO1	-0.43820741
ILMN_1674089	PRAMEF7	-0.438229691
ILMN_2075221	C17orf47	-0.438335282
ILMN_2408771	SPPL2B	-0.438358631
ILMN_1685580	CBLB	-0.438390951
ILMN_1700834	SLK	-0.438397889
ILMN_1779897	NPR1	-0.438416636
ILMN_2118612	FAM120C	-0.43848772
ILMN_1732154	BCAN	-0.438491018
ILMN_1796969	POU4F3	-0.438499174
ILMN_3282184	TRIM43B	-0.438517329
ILMN_1882045	CF890951	-0.438519713
ILMN_1705550	FOXD4L6	-0.438527561
ILMN_2174023	FAM75A3	-0.438533853
ILMN_1733311	PIGB	-0.438598937
ILMN_3228090	ZNF716	-0.438602769
ILMN_1675756	KCNJ15	-0.438646786
ILMN_1801240	OR5D14	-0.438664991
ILMN_1737396	PSMD14	-0.438684379
ILMN_2075578	FAM126A	-0.438752254
ILMN_1707311	CCDC54	-0.438795326
ILMN_1846742	AA459851	-0.438805928
ILMN_1753502	IGSF11	-0.438877772
ILMN_1675237	BC113726	-0.439005335
ILMN_2243687	LAMP2	-0.439021241
ILMN_1829253	AA398827	-0.439059711
ILMN_2124187	TSC22D2	-0.439276172
ILMN_3191988	NA	-0.439280143
ILMN_1657476	C7orf72	-0.439303325
ILMN_1721337	MRPS18B	-0.439338505
ILMN_1698484	RHOV	-0.439462304
ILMN_1714650	RASGRP4	-0.439485965
ILMN_1672807	CA5B	-0.43949023
ILMN_3307247	LOC440742	-0.43949744
ILMN_1853116	BC031013	-0.439545137
ILMN_3247400	LOC572558	-0.439592331
ILMN_1673493	GABRB3	-0.439654556
ILMN_1710884	OR5M10	-0.439693608
ILMN_3234910	CLIC5	-0.439718468
ILMN_1809347	SRP14	-0.439747418

	GOTERM_BP_FAT	regulation of neurotransmitter levels	8	5.70E-01	1.00E+00
**Annotation Cluster 119**	**Enrichment Score: 0.54**		**Count**	**P_Value**	**Benjamini**
	SP_PIR_KEYWORDS	molecular chaperone	4	1.50E-01	8.60E-01
	INTERPRO	Chaperone, tailless complex polypeptide 1	3	3.30E-01	1.00E+00
	INTERPRO	Chaperonin Cpn60/TCP-1	3	4.80E-01	1.00E+00
**Annotation Cluster 120**	**Enrichment Score: 0.54**		**Count**	**P_Value**	**Benjamini**
	SP_PIR_KEYWORDS	Chaperone	23	1.00E-01	8.30E-01
	GOTERM_MF_FAT	unfolded protein binding	14	4.60E-01	1.00E+00
	GOTERM_BP_FAT	protein folding	20	5.10E-01	1.00E+00
**Annotation Cluster 121**	**Enrichment Score: 0.54**		**Count**	**P_Value**	**Benjamini**
	SP_PIR_KEYWORDS	protein transport	66	2.20E-02	6.20E-01
	GOTERM_BP_FAT	protein localization	106	1.10E-01	9.70E-01
	GOTERM_BP_FAT	establishment of protein localization	93	1.20E-01	9.80E-01
	GOTERM_BP_FAT	protein transport	92	1.20E-01	9.80E-01
	GOTERM_BP_FAT	intracellular transport	69	6.00E-01	1.00E+00
	GOTERM_BP_FAT	cellular macromolecule localization	39	8.50E-01	1.00E+00
	GOTERM_BP_FAT	cellular protein localization	38	8.80E-01	1.00E+00
	GOTERM_BP_FAT	intracellular protein transport	33	9.20E-01	1.00E+00
	GOTERM_BP_FAT	protein targeting	18	9.20E-01	1.00E+00
**Annotation Cluster 122**	**Enrichment Score: 0.54**		**Count**	**P_Value**	**Benjamini**
	GOTERM_CC_FAT	cell-cell adherens junction	7	1.70E-01	9.40E-01
	GOTERM_CC_FAT	fascia adherens	3	2.90E-01	9.60E-01
	GOTERM_CC_FAT	intercalated disc	3	4.90E-01	9.70E-01
**Annotation Cluster 123**	**Enrichment Score: 0.54**		**Count**	**P_Value**	**Benjamini**
	SMART	R3H	3	2.60E-01	9.70E-01
	INTERPRO	Single-stranded nucleic acid binding R3H	3	2.90E-01	1.00E+00
	UP_SEQ_FEATURE	domain:R3H	3	3.20E-01	1.00E+00
**Annotation Cluster 124**	**Enrichment Score: 0.53**		**Count**	**P_Value**	**Benjamini**
	GOTERM_BP_FAT	membrane lipid metabolic process	14	8.40E-02	9.80E-01
	GOTERM_BP_FAT	sphingolipid metabolic process	13	9.60E-02	9.80E-01
	GOTERM_BP_FAT	glycolipid metabolic process	6	2.30E-01	9.90E-01
	GOTERM_BP_FAT	glycosphingolipid metabolic process	5	2.90E-01	9.90E-01
	GOTERM_BP_FAT	sphingolipid biosynthetic process	5	4.20E-01	1.00E+00
	GOTERM_BP_FAT	membrane lipid biosynthetic process	5	4.90E-01	1.00E+00
	GOTERM_CC_FAT	intrinsic to Golgi membrane	6	5.60E-01	9.70E-01
	GOTERM_CC_FAT	integral to Golgi membrane	4	8.60E-01	9.90E-01
**Annotation Cluster 125**	**Enrichment Score: 0.52**		**Count**	**P_Value**	**Benjamini**
	SMART	TUDOR	5	2.30E-01	9.70E-01
	INTERPRO	Tudor domain	5	2.70E-01	1.00E+00
	UP_SEQ_FEATURE	domain:Tudor	3	3.20E-01	1.00E+00
	INTERPRO	Tudor subgroup	3	4.10E-01	1.00E+00
**Annotation Cluster 126**	**Enrichment Score: 0.51**		**Count**	**P_Value**	**Benjamini**
	GOTERM_BP_FAT	metal ion transport	59	1.10E-01	9.70E-01
	GOTERM_BP_FAT	monovalent inorganic cation transport	41	1.40E-01	9.80E-01
	GOTERM_BP_FAT	potassium ion transport	20	3.40E-01	9.90E-01
	GOTERM_MF_FAT	alkali metal ion binding	27	3.90E-01	9.90E-01
	GOTERM_BP_FAT	sodium ion transport	16	4.00E-01	1.00E+00
	SP_PIR_KEYWORDS	Sodium transport	14	4.40E-01	9.50E-01
	SP_PIR_KEYWORDS	Sodium	14	4.40E-01	9.50E-01
	GOTERM_MF_FAT	sodium ion binding	14	4.90E-01	1.00E+00
**Annotation Cluster 127**	**Enrichment Score: 0.51**		**Count**	**P_Value**	**Benjamini**
	GOTERM_BP_FAT	proximal/distal pattern formation	5	2.20E-01	9.90E-01
	GOTERM_BP_FAT	embryonic forelimb morphogenesis	4	3.30E-01	9.90E-01
	GOTERM_BP_FAT	forelimb morphogenesis	4	4.20E-01	1.00E+00
**Annotation Cluster 128**	**Enrichment Score: 0.5**		**Count**	**P_Value**	**Benjamini**
	GOTERM_BP_FAT	lung development	14	2.50E-01	9.90E-01
	GOTERM_BP_FAT	respiratory tube development	14	2.80E-01	9.90E-01
	GOTERM_BP_FAT	respiratory system development	14	3.60E-01	1.00E+00
	GOTERM_BP_FAT	tube development	26	3.90E-01	1.00E+00
**Annotation Cluster 129**	**Enrichment Score: 0.5**		**Count**	**P_Value**	**Benjamini**
	SMART	MAM	4	2.60E-01	9.70E-01
	INTERPRO	MAM	4	3.00E-01	1.00E+00
	UP_SEQ_FEATURE	domain:MAM	3	4.00E-01	1.00E+00
**Annotation Cluster 130**	**Enrichment Score: 0.5**		**Count**	**P_Value**	**Benjamini**
	KEGG_PATHWAY	Retinol metabolism	9	2.50E-01	9.50E-01
	GOTERM_BP_FAT	estrogen metabolic process	3	2.90E-01	9.90E-01
	GOTERM_BP_FAT	cellular hormone metabolic process	8	4.40E-01	1.00E+00
**Annotation Cluster 131**	**Enrichment Score: 0.5**		**Count**	**P_Value**	**Benjamini**
	GOTERM_BP_FAT	response to corticosteroid stimulus	13	1.90E-01	9.80E-01
	GOTERM_BP_FAT	response to steroid hormone stimulus	23	3.80E-01	1.00E+00
	GOTERM_BP_FAT	response to glucocorticoid stimulus	10	4.50E-01	1.00E+00
**Annotation Cluster 132**	**Enrichment Score: 0.5**		**Count**	**P_Value**	**Benjamini**
	SMART	RINGv	3	3.00E-01	9.70E-01
	UP_SEQ_FEATURE	zinc finger region:RING-CH-type	3	3.20E-01	1.00E+00
	INTERPRO	Zinc finger, RING-CH-type	3	3.30E-01	1.00E+00
**Annotation Cluster 133**	**Enrichment Score: 0.5**		**Count**	**P_Value**	**Benjamini**
	INTERPRO	C2 membrane targeting protein	17	9.30E-02	1.00E+00
	SMART	C2	18	2.00E-01	9.50E-01
	INTERPRO	C2 calcium-dependent membrane targeting	18	2.80E-01	1.00E+00
	PIR_SUPERFAMILY	PIRSF002385:B/K protein/synaptotagmin	3	4.50E-01	1.00E+00
	UP_SEQ_FEATURE	domain:C2	10	4.70E-01	1.00E+00
	UP_SEQ_FEATURE	domain:C2 2	7	5.40E-01	1.00E+00
	UP_SEQ_FEATURE	domain:C2 1	7	5.40E-01	1.00E+00
**Annotation Cluster 134**	**Enrichment Score: 0.49**		**Count**	**P_Value**	**Benjamini**
	GOTERM_CC_FAT	adherens junction	23	1.10E-01	9.20E-01
	GOTERM_CC_FAT	anchoring junction	25	1.10E-01	9.20E-01
	GOTERM_CC_FAT	basolateral plasma membrane	23	5.20E-01	9.70E-01
	GOTERM_CC_FAT	focal adhesion	12	5.40E-01	9.70E-01
	GOTERM_CC_FAT	cell-substrate junction	13	5.50E-01	9.70E-01
	GOTERM_CC_FAT	cell-substrate adherens junction	12	6.00E-01	9.70E-01
**Annotation Cluster 135**	**Enrichment Score: 0.49**		**Count**	**P_Value**	**Benjamini**
	SMART	PlsC	4	2.60E-01	9.70E-01
	SP_PIR_KEYWORDS	phospholipid biosynthesis	7	2.70E-01	9.00E-01

ILMN_2369580	C16orf35	0.712439732
ILMN_1789510	STIP1	0.712271416
ILMN_1692168	UBE2Z	0.712195695
ILMN_1813669	ANKS1A	0.712054629
ILMN_1795227	DYNLL1	0.711828081
ILMN_1728163	CTDSP1	0.71175555
ILMN_1699703	ARCN1	0.71161254
ILMN_3243700	RPS8	0.711544341
ILMN_1678329	TNRC18	0.711449205
ILMN_2121207	FAM125A	0.711383192
ILMN_1730622	EVL	0.71137422
ILMN_1803036	TARBP1	0.711245456
ILMN_1720799	TECR	0.710984938
ILMN_1721225	C20orf4	0.710812604
ILMN_3289171	RPS27	0.710729327
ILMN_1675156	CDC42	0.710657034
ILMN_1681269	DYRK4	0.710472999
ILMN_1739199	WASH2P	0.710470181
ILMN_3251100	MEF2A	0.710338722
ILMN_1760682	STAG3L2	0.710292459
ILMN_1651262	HNRNPAB	0.710229282
ILMN_1686954	RPS10	0.710030708
ILMN_1753254	SLC12A7	0.7099978
ILMN_2262198	PMS2CL	0.709931654
ILMN_1671054	HLA-A	0.709830158
ILMN_1664028	CENPB	0.709789272
ILMN_3299520	PRKCB	0.709611469
ILMN_1780298	FAM86A	0.709575137
ILMN_2181968	CBL	0.709530485
ILMN_1663313	AMY1A	0.709360339
ILMN_3238375	CENPT	0.709230526
ILMN_2380163	PTPRF	0.70890466
ILMN_1808500	CEP68	0.708828908
ILMN_3241469	RNF181	0.708644084
ILMN_1803277	MVP	0.708256704
ILMN_1774074	RXRB	0.707998818
ILMN_2378952	GPX4	0.707983345
ILMN_1675460	RPL29	0.707909625
ILMN_1760778	ENG	0.70778025
ILMN_2382829	PRDX2	0.707733136
ILMN_1802251	PTTG1IP	0.707573612
ILMN_1778684	BRE	0.707548634
ILMN_3297317	RPL13AP5	0.707479084
ILMN_1723895	GTF3C5	0.707438165
ILMN_1726245	TGFBR2	0.707408106
ILMN_1703593	BAIAP2L1	0.707253559
ILMN_3204734	PILRB	0.707071166
ILMN_1800425	SLC9A1	0.706943657
ILMN_1815057	PDGFRB	0.706935348
ILMN_1683441	NCAPD3	0.706821779
ILMN_1781281	B9D1	0.706782355
ILMN_2175712	NDUFA11	0.706759502
ILMN_1740429	FTL	0.70665256
ILMN_2112673	POTEKP	0.70663258
ILMN_2109489	GZMB	0.706494782
ILMN_3195401	GABPB1	0.706453998
ILMN_1787815	TRIB3	0.705511361
ILMN_3193623	RPL17	0.705503693
ILMN_1674608	FAM169B	0.705460214
ILMN_1726990	DOM3Z	0.705379976
ILMN_1760858	RAB8A	0.705225433
ILMN_1811104	MDSRP	0.705224441
ILMN_1655987	STAB1	0.705203827
ILMN_1719986	PIK3IP1	0.704946162
ILMN_1805999	MYO1D	0.704863137
ILMN_2334989	CCT3	0.704862075
ILMN_2151817	PFN1	0.704850406
ILMN_1779399	SNRPA	0.704760345
ILMN_1755536	PFDN5	0.704740593
ILMN_3195203	C17orf101	0.704711346
ILMN_1655563	CTIF	0.704629425
ILMN_1679482	TUBA3C	0.704415773
ILMN_1808777	EHD2	0.704360272
ILMN_1674768	LOC375133	0.704121918
ILMN_1665117	C6orf89	0.704012997
ILMN_3200362	NBPF1	0.703889553
ILMN_1795495	ERCC5	0.703843462
ILMN_1716056	LMF2	0.703729544
ILMN_1749109	PSAP	0.703548706
ILMN_1686664	MT2A	0.703502977
ILMN_1783909	COL6A2	0.70340899
ILMN_1776047	HSP90AB3P	0.703397603
ILMN_1781623	TEX264	0.703188699
ILMN_1724066	PLCB2	0.703049068
ILMN_1651544	HDAC8	0.702988239
ILMN_1721241	CYTH2	0.70288524
ILMN_1680437	EEF1G	0.702822882
ILMN_3242330	FAM65C	0.702798498
ILMN_1741491	ZNHIT1	0.702625337
ILMN_2338348	UBASH3A	0.702585529
ILMN_1733023	CCT6P1	0.702434205
ILMN_1722872	MYH9	0.702385945
ILMN_1670638	PITPNC1	0.702351978
ILMN_1809437	RHBDD2	0.702329845

	BIOCARTA	Lck and Fyn tyrosine kinases in initiation of TCR Activation	6	4.40E-02	9.10E-01
	GOTERM_CC_FAT	T cell receptor complex	5	6.50E-02	4.00E-01
	KEGG_PATHWAY	Primary immunodeficiency	10	6.80E-02	3.60E-01
	GOTERM_BP_FAT	T cell differentiation in the thymus	7	1.40E-01	7.70E-01
	SP_PIR_KEYWORDS	T-cell	7	1.60E-01	5.80E-01
	BIOCARTA	T Cytotoxic Cell Surface Molecules	5	1.90E-01	9.40E-01
	BIOCARTA	T Helper Cell Surface Molecules	5	1.90E-01	9.40E-01
	BIOCARTA	CTL mediated immune response against target cells	5	3.30E-01	9.50E-01
	BIOCARTA	HIV Induced T Cell Apoptosis	3	5.40E-01	9.80E-01
	BIOCARTA	IL 17 Signaling Pathway	4	5.80E-01	9.90E-01
**Annotation Cluster 103**	**Enrichment Score: 1.04**		**Count**	**P_Value**	**Benjamini**
	SP_PIR_KEYWORDS	microfilament	3	4.00E-02	2.50E-01
	INTERPRO	Actin, conserved site	6	4.20E-02	9.20E-01
	PIR_SUPERFAMILY	PIRSF002337:Actin	6	6.90E-02	1.00E+00
	INTERPRO	Actin/actin-like	8	2.20E-01	1.00E+00
	SMART	ACTIN	8	2.50E-01	9.20E-01
**Annotation Cluster 104**	**Enrichment Score: 1.04**		**Count**	**P_Value**	**Benjamini**
	INTERPRO	Ubiquitin-associated/translation elongation factor EF1B, N-terminal, eukaryote	10	7.50E-02	9.60E-01
	SMART	UBA	10	9.40E-02	7.90E-01
	INTERPRO	Ubiquitin-associated/translation elongation factor EF1B, N-terminal	9	9.80E-02	9.60E-01
	UP_SEQ_FEATURE	domain:UBA	9	1.00E-01	1.00E+00
**Annotation Cluster 105**	**Enrichment Score: 1.03**		**Count**	**P_Value**	**Benjamini**
	GOTERM_BP_FAT	regulation of protein amino acid phosphorylation	36	6.00E-03	1.40E-01
	GOTERM_BP_FAT	regulation of peptidyl-tyrosine phosphorylation	16	2.90E-02	3.80E-01
	GOTERM_BP_FAT	positive regulation of peptidyl-tyrosine phosphorylation	10	1.60E-01	8.10E-01
	GOTERM_BP_FAT	positive regulation of phosphorus metabolic process	18	1.60E-01	8.10E-01
	GOTERM_BP_FAT	positive regulation of phosphate metabolic process	18	1.60E-01	8.10E-01
	GOTERM_BP_FAT	positive regulation of protein amino acid phosphorylation	15	2.80E-01	9.20E-01
	GOTERM_BP_FAT	positive regulation of phosphorylation	16	2.90E-01	9.30E-01
**Annotation Cluster 106**	**Enrichment Score: 1.03**		**Count**	**P_Value**	**Benjamini**
	GOTERM_BP_FAT	regulation of lymphocyte apoptosis	5	6.20E-02	5.60E-01
	GOTERM_BP_FAT	regulation of B cell apoptosis	4	7.70E-02	6.10E-01
	GOTERM_BP_FAT	positive regulation of B cell apoptosis	3	8.70E-02	6.40E-01
	GOTERM_BP_FAT	apoptotic mitochondrial changes	8	1.00E-01	7.00E-01
	GOTERM_BP_FAT	apoptotic nuclear changes	7	1.20E-01	7.30E-01
	GOTERM_BP_FAT	positive regulation of lymphocyte apoptosis	3	1.30E-01	7.60E-01
**Annotation Cluster 107**	**Enrichment Score: 1.02**		**Count**	**P_Value**	**Benjamini**
	INTERPRO	WD40/YVTN repeat-like	50	3.40E-02	9.10E-01
	UP_SEQ_FEATURE	repeat:WD 3	43	4.10E-02	9.90E-01
	UP_SEQ_FEATURE	repeat:WD 1	44	4.50E-02	1.00E+00
	UP_SEQ_FEATURE	repeat:WD 2	44	4.50E-02	1.00E+00
	SP_PIR_KEYWORDS	wd repeat	44	4.60E-02	2.70E-01
	INTERPRO	WD40 repeat, conserved site	45	6.30E-02	9.40E-01
	INTERPRO	WD40 repeat	43	7.30E-02	9.60E-01
	UP_SEQ_FEATURE	repeat:WD 5	37	7.40E-02	1.00E+00
	INTERPRO	WD40 repeat, subgroup	39	9.00E-02	9.60E-01
	INTERPRO	WD40 repeat, region	38	1.00E-01	9.70E-01
	SMART	WD40	43	1.20E-01	8.10E-01
	INTERPRO	WD40 repeat 2	35	1.60E-01	9.90E-01
	UP_SEQ_FEATURE	repeat:WD 4	37	1.60E-01	1.00E+00
	UP_SEQ_FEATURE	repeat:WD 6	25	4.70E-01	1.00E+00
	UP_SEQ_FEATURE	repeat:WD 7	17	7.40E-01	1.00E+00
**Annotation Cluster 108**	**Enrichment Score: 1.02**		**Count**	**P_Value**	**Benjamini**
	GOTERM_BP_FAT	gene silencing	16	4.90E-03	1.20E-01
	GOTERM_BP_FAT	regulation of gene expression, epigenetic	19	1.00E-02	2.00E-01
	SP_PIR_KEYWORDS	rna-mediated gene silencing	8	3.70E-02	2.40E-01
	GOTERM_BP_FAT	gene silencing by RNA	9	6.10E-02	5.50E-01
	GOTERM_CC_FAT	RNA-induced silencing complex	3	8.90E-02	4.70E-01
	GOTERM_CC_FAT	RNAi effector complex	3	8.90E-02	4.70E-01
	GOTERM_BP_FAT	RNA interference	3	1.80E-01	8.40E-01
	GOTERM_BP_FAT	posttranscriptional gene silencing	5	3.00E-01	9.30E-01
	GOTERM_BP_FAT	posttranscriptional gene silencing by RNA	5	3.00E-01	9.30E-01
	UP_SEQ_FEATURE	domain:PAZ	3	3.00E-01	1.00E+00
	INTERPRO	Argonaute and Dicer protein, PAZ	3	3.20E-01	1.00E+00
	GOTERM_BP_FAT	gene silencing by miRNA	4	3.90E-01	9.70E-01
**Annotation Cluster 109**	**Enrichment Score: 1.02**		**Count**	**P_Value**	**Benjamini**
	GOTERM_BP_FAT	positive regulation of alpha-beta T cell activation	9	1.60E-02	2.60E-01
	GOTERM_BP_FAT	regulation of calcium-mediated signaling	7	3.90E-02	4.40E-01
	GOTERM_BP_FAT	positive regulation of calcium-mediated signaling	6	6.30E-02	5.60E-01
	GOTERM_BP_FAT	positive regulation of alpha-beta T cell proliferation	4	1.00E-01	7.00E-01
	GOTERM_BP_FAT	positive regulation of T cell proliferation	9	1.30E-01	7.60E-01
	GOTERM_BP_FAT	leukocyte adhesion	7	1.50E-01	8.00E-01
	GOTERM_BP_FAT	regulation of alpha-beta T cell proliferation	4	2.40E-01	9.00E-01
	GOTERM_BP_FAT	regulation of B cell differentiation	4	3.50E-01	9.60E-01
**Annotation Cluster 110**	**Enrichment Score: 1.02**		**Count**	**P_Value**	**Benjamini**
	GOTERM_BP_FAT	retrograde vesicle-mediated transport, Golgi to ER	8	1.50E-02	2.50E-01
	GOTERM_BP_FAT	Golgi transport vesicle coating	5	3.30E-02	4.00E-01
	GOTERM_BP_FAT	COPI coating of Golgi vesicle	5	3.30E-02	4.00E-01
	GOTERM_BP_FAT	Golgi vesicle budding	5	3.30E-02	4.00E-01
	GOTERM_BP_FAT	vesicle targeting	7	6.00E-02	5.50E-01
	GOTERM_BP_FAT	vesicle targeting, to, from or within Golgi	5	6.20E-02	5.60E-01
	GOTERM_BP_FAT	vesicle coating	5	1.30E-01	7.50E-01
	GOTERM_CC_FAT	COPI-coated vesicle	5	1.30E-01	5.60E-01
	GOTERM_BP_FAT	establishment of vesicle localization	8	1.40E-01	7.70E-01
	GOTERM_BP_FAT	organelle localization	17	1.50E-01	7.90E-01
	GOTERM_BP_FAT	membrane budding	5	1.50E-01	7.90E-01
	GOTERM_CC_FAT	COPI vesicle coat	4	1.70E-01	6.30E-01
	GOTERM_BP_FAT	establishment of organelle localization	13	1.90E-01	8.50E-01
	GOTERM_BP_FAT	vesicle localization	8	2.10E-01	8.70E-01
	GOTERM_CC_FAT	COPI coated vesicle membrane	4	2.10E-01	6.90E-01
	GOTERM_BP_FAT	vesicle organization	9	3.80E-01	9.60E-01
**Annotation Cluster 111**	**Enrichment Score: 1.02**		**Count**	**P_Value**	**Benjamini**
	INTERPRO	Citron-like	5	9.00E-02	9.60E-01
	UP_SEQ_FEATURE	domain:CNH	5	9.80E-02	1.00E+00

ILMN_2232066	ABCB11	-0.439831383
ILMN_1904791	AK023763	-0.439964325
ILMN_1733746	REEP1	-0.439969482
ILMN_1690753	RBMY1A1	-0.440043788
ILMN_3311180	SKCG-1	-0.440076423
ILMN_2054271	GPR135	-0.440147566
ILMN_1651535	TMEM217	-0.440257381
ILMN_1762095	TMTC4	-0.440319827
ILMN_1742901	RSPH10B2	-0.44032704
ILMN_1738867	TYMP	-0.440363918
ILMN_3308632	MIR617	-0.440463093
ILMN_1777665	SLC22A13	-0.44047253
ILMN_1730085	LCORL	-0.440486382
ILMN_2128048	PCDHB3	-0.440506452
ILMN_3246014	C1orf130	-0.440568885
ILMN_1903082	AI769787	-0.440596157
ILMN_1685952	ACSM3	-0.44061411
ILMN_1874613	AW979088	-0.440651233
ILMN_1801943	NAV2	-0.440670235
ILMN_3240715	PP1164	-0.440698803
ILMN_3246673	AMAC1L3	-0.440776846
ILMN_1759493	ANXA8	-0.440784885
ILMN_1700763	BC153822	-0.44080103
ILMN_3255871	H03055	-0.440870857
ILMN_1790384	AK124197	-0.440932927
ILMN_3221072	GCSH	-0.440984666
ILMN_3235891	AK122833	-0.441045563
ILMN_3240446	FKHRL1P2	-0.441105629
ILMN_1712352	DOCK3	-0.441147848
ILMN_1749996	KCNN2	-0.441176515
ILMN_1728746	GAB2	-0.441220783
ILMN_3211746	AK127966	-0.441382424
ILMN_3207615	TRIM64C	-0.441408399
ILMN_1674661	CIRBP	-0.441408412
ILMN_1776240	DUSP13	-0.441446191
ILMN_3307695	THEM5	-0.441454301
ILMN_1703599	HYDIN	-0.441467537
ILMN_2143220	ANGPTL3	-0.441479773
ILMN_1712197	KCNMB3	-0.441487542
ILMN_1909135	BX952833	-0.441488486
ILMN_2361575	SNX14	-0.441488586
ILMN_1842191	AI827529	-0.441494661
ILMN_3190833	CCRL2	-0.441572955
ILMN_1737242	C8G	-0.441579568
ILMN_1796835	TMEM56	-0.441618944
ILMN_3238462	LOC100506874	-0.441622702
ILMN_2185077	C14orf39	-0.441679986
ILMN_1695949	SIGLEC12	-0.441688566
ILMN_1701565	UPK3B	-0.441726138
ILMN_3240465	DKFZp434F1719	-0.441832231
ILMN_1709360	ARAP2	-0.441844139
ILMN_3226324	BBIP1	-0.441851699
ILMN_3309349	SNHG8	-0.441876326
ILMN_1654386	C14orf182	-0.441955043
ILMN_3246694	KRTAP21-1	-0.442021286
ILMN_3308078	MIR31	-0.442027231
ILMN_1790043	NXF4	-0.442041196
ILMN_1669747	UNC13B	-0.442058824
ILMN_2344283	FMO3	-0.44211933
ILMN_1903651	BX417275	-0.442136871
ILMN_1724826	DNM1L	-0.442223782
ILMN_2402629	METTL9	-0.442227831
ILMN_3299074	NOX4	-0.442276259
ILMN_1720207	GMPPB	-0.4423306
ILMN_1797209	TSGA14	-0.442358382
ILMN_3238960	PRSS3	-0.442450719
ILMN_1811957	CAMSAP1	-0.442577249
ILMN_3215006	LOC100131831	-0.442583681
ILMN_1876466	NA	-0.442583967
ILMN_1781381	AK123658	-0.442603723
ILMN_3224820	HM801842	-0.442609643
ILMN_3249524	FLJ40434	-0.442708382
ILMN_1856044	AA909740	-0.442721675
ILMN_3307178	SPRNP1	-0.44273019
ILMN_1774256	PRSS21	-0.442745298
ILMN_1810387	PLAA	-0.442776337
ILMN_1740076	SLC16A12	-0.442777699
ILMN_1856986	BX109078	-0.442787563
ILMN_1673395	PIGO	-0.442821529
ILMN_1800611	CEP72	-0.442848258
ILMN_3309224	MIR23A	-0.442887876
ILMN_2257833	BBS7	-0.44304955
ILMN_2216815	MAP7	-0.443057762
ILMN_1744295	CDKN2A	-0.4430874
ILMN_1787716	AK125940	-0.443106683
ILMN_1660085	PRTG	-0.443168942
ILMN_1788832	MIB1	-0.4432983
ILMN_1677165	SF3B1	-0.443345965
ILMN_2121272	PDE10A	-0.443349406
ILMN_2215545	C3orf26	-0.443370626
ILMN_3251672	DSG2	-0.443408839
ILMN_1688218	RASGRP4	-0.443422307
ILMN_3211343	AX747968	-0.443453928
ILMN_3248440	SNORA81	-0.443476194

	INTERPRO	Phospholipid/glycerol acyltransferase	4	3.00E-01	1.00E+00
	UP_SEQ_FEATURE	short sequence motif:HXXXXD motif	3	5.10E-01	1.00E+00
**Annotation Cluster 136**	**Enrichment Score: 0.49**		**Count**	**P_Value**	**Benjamini**
	SP_PIR_KEYWORDS	fmn	4	2.30E-01	9.00E-01
	GOTERM_MF_FAT	FMN binding	3	3.70E-01	9.90E-01
	UP_SEQ_FEATURE	nucleotide phosphate-binding region:FMN	3	4.00E-01	1.00E+00
**Annotation Cluster 137**	**Enrichment Score: 0.48**		**Count**	**P_Value**	**Benjamini**
	GOTERM_BP_FAT	angiogenesis	20	2.20E-01	9.90E-01
	SP_PIR_KEYWORDS	angiogenesis	10	2.50E-01	9.00E-01
	GOTERM_BP_FAT	blood vessel morphogenesis	26	3.10E-01	9.90E-01
	GOTERM_BP_FAT	blood vessel development	28	4.50E-01	1.00E+00
	GOTERM_BP_FAT	vasculature development	28	5.00E-01	1.00E+00
**Annotation Cluster 138**	**Enrichment Score: 0.48**		**Count**	**P_Value**	**Benjamini**
	BIOCARTA	AKT Signaling Pathway	5	2.10E-01	9.80E-01
	BIOCARTA	mTOR Signaling Pathway	5	3.30E-01	9.90E-01
	BIOCARTA	Inactivation of Gsk3 by AKT causes accumulation of b-catenin in Alveolar Macrophages	5	3.80E-01	9.90E-01
	BIOCARTA	Skeletal muscle hypertrophy is regulated via AKT/mTOR pathway	4	4.60E-01	9.90E-01
**Annotation Cluster 139**	**Enrichment Score: 0.48**		**Count**	**P_Value**	**Benjamini**
	GOTERM_MF_FAT	solute:cation antiporter activity	6	2.10E-01	9.80E-01
	GOTERM_MF_FAT	solute:hydrogen antiporter activity	5	2.30E-01	9.80E-01
	INTERPRO	Na+/H+ exchanger, subfamily	3	2.50E-01	1.00E+00
	INTERPRO	Na+/H+ exchanger, conserved region	3	2.50E-01	1.00E+00
	GOTERM_MF_FAT	monovalent cation:hydrogen antiporter activity	3	2.50E-01	9.90E-01
	GOTERM_MF_FAT	sodium:hydrogen antiporter activity	3	2.50E-01	9.90E-01
	GOTERM_MF_FAT	cation:cation antiporter activity	4	2.70E-01	9.90E-01
	INTERPRO	Cation/H+ exchanger, conserved region	3	2.90E-01	1.00E+00
	GOTERM_MF_FAT	antiporter activity	9	4.40E-01	1.00E+00
	INTERPRO	Cation/H+ exchanger	3	4.50E-01	1.00E+00
	GOTERM_BP_FAT	regulation of pH	5	4.90E-01	1.00E+00
	GOTERM_MF_FAT	solute:solute antiporter activity	7	5.20E-01	1.00E+00
	GOTERM_BP_FAT	monovalent inorganic cation homeostasis	6	5.30E-01	1.00E+00
	SP_PIR_KEYWORDS	antiport	5	5.30E-01	9.70E-01
**Annotation Cluster 140**	**Enrichment Score: 0.47**		**Count**	**P_Value**	**Benjamini**
	GOTERM_CC_FAT	nuclear chromatin	9	2.30E-01	9.50E-01
	GOTERM_CC_FAT	nuclear chromosome	21	2.90E-01	9.60E-01
	GOTERM_CC_FAT	nuclear chromosome part	14	5.60E-01	9.70E-01
**Annotation Cluster 141**	**Enrichment Score: 0.47**		**Count**	**P_Value**	**Benjamini**
	GOTERM_BP_FAT	dorsal/ventral axis specification	3	2.00E-01	9.90E-01
	GOTERM_BP_FAT	axis specification	6	2.10E-01	9.90E-01
	GOTERM_BP_FAT	dorsal/ventral pattern formation	5	8.90E-01	1.00E+00
**Annotation Cluster 142**	**Enrichment Score: 0.47**		**Count**	**P_Value**	**Benjamini**
	INTERPRO	Acyl-CoA dehydrogenase/oxidase, N-terminal	4	1.80E-01	1.00E+00
	INTERPRO	Acyl-CoA oxidase/dehydrogenase, central region	4	2.10E-01	1.00E+00
	INTERPRO	Acyl-CoA oxidase/dehydrogenase, type1/2, C-terminal	4	2.10E-01	1.00E+00
	GOTERM_MF_FAT	acyl-CoA dehydrogenase activity	4	2.10E-01	9.80E-01
	SP_PIR_KEYWORDS	Flavoprotein	15	2.30E-01	9.00E-01
	INTERPRO	Acyl-CoA dehydrogenase, N-terminal	3	3.30E-01	1.00E+00
	GOTERM_MF_FAT	FAD binding	10	3.60E-01	9.90E-01
	INTERPRO	Acyl-CoA oxidase/dehydrogenase, type 1	3	4.10E-01	1.00E+00
	UP_SEQ_FEATURE	binding site:Substrate; via carbonyl oxygen	4	5.20E-01	1.00E+00
	SP_PIR_KEYWORDS	FAD	13	5.40E-01	9.70E-01
	UP_SEQ_FEATURE	nucleotide phosphate-binding region:FAD	7	6.30E-01	1.00E+00
	UP_SEQ_FEATURE	binding site:Substrate; via amide nitrogen	3	7.10E-01	1.00E+00
**Annotation Cluster 143**	**Enrichment Score: 0.47**		**Count**	**P_Value**	**Benjamini**
	UP_SEQ_FEATURE	domain:WH2	4	2.90E-01	1.00E+00
	SMART	WH2	4	3.50E-01	9.70E-01
	INTERPRO	Actin-binding WH2	4	3.90E-01	1.00E+00
**Annotation Cluster 144**	**Enrichment Score: 0.46**		**Count**	**P_Value**	**Benjamini**
	SMART	ANK	30	1.40E-01	9.60E-01
	UP_SEQ_FEATURE	repeat:ANK 9	7	1.90E-01	1.00E+00
	SP_PIR_KEYWORDS	ank repeat	30	2.30E-01	9.00E-01
	INTERPRO	Ankyrin	30	2.40E-01	1.00E+00
	UP_SEQ_FEATURE	repeat:ANK 7	9	2.60E-01	1.00E+00
	UP_SEQ_FEATURE	repeat:ANK 8	7	2.80E-01	1.00E+00
	UP_SEQ_FEATURE	repeat:ANK 1	28	3.10E-01	1.00E+00
	UP_SEQ_FEATURE	repeat:ANK 2	28	3.20E-01	1.00E+00
	UP_SEQ_FEATURE	repeat:ANK 3	21	5.70E-01	1.00E+00
	UP_SEQ_FEATURE	repeat:ANK 4	16	7.00E-01	1.00E+00
	UP_SEQ_FEATURE	repeat:ANK 6	9	7.60E-01	1.00E+00
	UP_SEQ_FEATURE	repeat:ANK 5	10	9.40E-01	1.00E+00
**Annotation Cluster 145**	**Enrichment Score: 0.46**		**Count**	**P_Value**	**Benjamini**
	GOTERM_BP_FAT	RNA localization	15	1.70E-01	9.80E-01
	GOTERM_BP_FAT	nuclear export	9	3.00E-01	9.90E-01
	GOTERM_BP_FAT	establishment of RNA localization	13	3.30E-01	9.90E-01
	GOTERM_BP_FAT	RNA transport	13	3.30E-01	9.90E-01
	GOTERM_BP_FAT	nucleic acid transport	13	3.30E-01	9.90E-01
	SP_PIR_KEYWORDS	mRNA transport	10	3.70E-01	9.30E-01
	GOTERM_BP_FAT	nucleobase, nucleoside, nucleotide and nucleic acid transport	14	4.20E-01	1.00E+00
	GOTERM_BP_FAT	RNA export from nucleus	6	4.40E-01	1.00E+00
	GOTERM_BP_FAT	mRNA transport	10	5.80E-01	1.00E+00
**Annotation Cluster 146**	**Enrichment Score: 0.46**		**Count**	**P_Value**	**Benjamini**
	GOTERM_BP_FAT	nucleus organization	9	1.80E-01	9.80E-01
	GOTERM_BP_FAT	DNA catabolic process, endonucleolytic	5	2.90E-01	9.90E-01
	GOTERM_BP_FAT	DNA fragmentation involved in apoptosis	4	3.30E-01	9.90E-01
	GOTERM_BP_FAT	cellular component disassembly	8	3.30E-01	9.90E-01
	GOTERM_BP_FAT	cell structure disassembly during apoptosis	4	4.50E-01	1.00E+00
	GOTERM_BP_FAT	DNA catabolic process	8	4.50E-01	1.00E+00
	GOTERM_BP_FAT	apoptotic nuclear changes	4	5.30E-01	1.00E+00
**Annotation Cluster 147**	**Enrichment Score: 0.46**		**Count**	**P_Value**	**Benjamini**
	BBID	26.cyclin-CDK_complexes	4	1.30E-01	9.70E-01
	SP_PIR_KEYWORDS	cell cycle control	6	2.20E-01	9.00E-01
	BIOCARTA	Cell Cycle	5	3.80E-01	9.90E-01
	GOTERM_BP_FAT	regulation of cyclin-dependent protein kinase activity	7	5.20E-01	1.00E+00
	BIOCARTA	Cyclins and Cell Cycle Regulation	4	5.20E-01	9.90E-01

ILMN_1778561	WEE1	0.702204115
ILMN_1767556	C10orf10	0.702061648
ILMN_2232936	UQCRH	0.702050315
ILMN_3239397	NA	0.701964741
ILMN_1736500	GNB2L1	0.701788952
ILMN_1668090	STK25	0.701760846
ILMN_3302356	RPS14	0.701685109
ILMN_1793386	MED12	0.701490529
ILMN_2355953	LILRB4	0.701410093
ILMN_1668721	CCND3	0.701402337
ILMN_1687358	TMPRSS4	0.701258218
ILMN_1707901	DHRS7B	0.701250343
ILMN_2149566	VPS25	0.701231327
ILMN_1766110	PHF6	0.701128861
ILMN_1775008	NCAPD2	0.701061554
ILMN_1844692	FOXO3	0.701030179
ILMN_1785711	NEDD8	0.700991782
ILMN_2111229	BZRAP1	0.700964056
ILMN_2408576	FAM129B	0.700926326
ILMN_1796900	NUDCD3	0.700843356
ILMN_1704385	H3F3A	0.700745281
ILMN_1746604	IL6ST	0.700737445
ILMN_1653165	AAMP	0.700502268
ILMN_1739640	DCHS1	0.700418729
ILMN_1813314	HIST1H2BK	0.700388564
ILMN_2258816	SAMD3	0.700323247
ILMN_3245236	FBRS	0.70027578
ILMN_1695827	PPP1CA	0.70023217
ILMN_1804601	DKFZp686O16217	0.700199015
ILMN_2119535	RIPK1	0.700166642
ILMN_1685122	COL9A2	0.699841767
ILMN_2375599	RNH1	0.699728564
ILMN_1769191	GNAS	0.699673301
ILMN_1739283	UPF2	0.699650078
ILMN_1740749	RPL7A	0.699587198
ILMN_1749345	STX5	0.699281098
ILMN_1653599	ATP5D	0.699201793
ILMN_1696485	HNRNPAB	0.699164936
ILMN_1797566	SNAP47	0.698941458
ILMN_1745994	GAS7	0.698881456
ILMN_2332990	DIABLO	0.698876969
ILMN_1778941	HNF1B	0.698762754
ILMN_1669772	LRP1	0.698624053
ILMN_2377025	TCOF1	0.698620488
ILMN_2154287	ACCS	0.698553776
ILMN_2098126	CCL5	0.698199295
ILMN_3245441	AK299972	0.698013645
ILMN_1682818	ARPC4	0.697980044
ILMN_1656720	PTPN11	0.69796538
ILMN_1807234	ZNF700	0.697893954
ILMN_1735467	NPIPL2	0.697891101
ILMN_1718303	PVRL2	0.697817979
ILMN_1658624	UBXN6	0.697334632
ILMN_1692742	DENND3	0.697137203
ILMN_1663416	RPL17	0.696919711
ILMN_2330213	CUX1	0.696826757
ILMN_1700349	ADCK4	0.696805558
ILMN_2325837	CD3D	0.696722503
ILMN_1678547	CPNE9	0.696617386
ILMN_1806242	PSD	0.69660384
ILMN_1719449	DCLK2	0.69646375
ILMN_1779735	C7orf59	0.696429547
ILMN_1758831	RNF31	0.696369303
ILMN_1658486	MRPL54	0.696355015
ILMN_1718900	KCTD2	0.696252633
ILMN_1764383	MCOLN1	0.69619835
ILMN_1714159	LUZP1	0.696176582
ILMN_1738132	HOXA11	0.696158559
ILMN_3249400	abParts	0.696156237
ILMN_1800091	RARRES1	0.696141806
ILMN_2331501	UBC	0.696126166
ILMN_2197128	OSR1	0.695965019
ILMN_1674780	SF3B1	0.695839994
ILMN_2347068	MKNK2	0.695789016
ILMN_3230608	NBPF14	0.695745547
ILMN_1804789	KIAA1967	0.695735692
ILMN_1708093	ARHGEF5	0.69559438
ILMN_3241051	RPL32	0.695479706
ILMN_1683609	UBA1	0.69538271
ILMN_1728984	PA2G4	0.695308849
ILMN_1652161	PNKD	0.695230244
ILMN_3215569	NTAN1	0.695197857
ILMN_2348090	MRPL55	0.695190001
ILMN_1734353	GPX4	0.695187585
ILMN_1682792	BYSL	0.695068912
ILMN_1795218	DHX30	0.694824287
ILMN_1704079	RBM38	0.694736392
ILMN_1699980	TSPAN18	0.694722499
ILMN_1726410	APRT	0.694565054
ILMN_2232368	PCIF1	0.694554088
ILMN_1796409	C1QB	0.694507802

	SMART	CNH	5	1.00E-01	7.90E-01
**Annotation Cluster 112**	**Enrichment Score: 1**		**Count**	**P_Value**	**Benjamini**
	GOTERM_BP_FAT	protein modification by small protein conjugation	29	6.80E-03	1.50E-01
	GOTERM_BP_FAT	protein modification by small protein conjugation or removal	33	9.90E-03	2.00E-01
	GOTERM_BP_FAT	protein ubiquitination	24	3.70E-02	4.20E-01
	GOTERM_CC_FAT	ubiquitin ligase complex	19	5.00E-02	3.30E-01
	GOTERM_MF_FAT	small conjugating protein ligase activity	28	1.20E-01	8.30E-01
	GOTERM_MF_FAT	ubiquitin-protein ligase activity	24	1.80E-01	8.90E-01
	GOTERM_MF_FAT	acid-amino acid ligase activity	30	2.80E-01	9.60E-01
	KEGG_PATHWAY	Ubiquitin mediated proteolysis	23	4.00E-01	7.30E-01
	GOTERM_MF_FAT	ligase activity, forming carbon-nitrogen bonds	31	5.10E-01	9.90E-01
	SP_PIR_KEYWORDS	ubl conjugation pathway	60	6.50E-01	9.50E-01
**Annotation Cluster 113**	**Enrichment Score: 0.97**		**Count**	**P_Value**	**Benjamini**
	GOTERM_BP_FAT	positive regulation of T cell mediated immunity	7	9.00E-03	1.90E-01
	GOTERM_BP_FAT	positive regulation of immune effector process	13	9.70E-03	1.90E-01
	GOTERM_BP_FAT	regulation of interleukin-2 production	9	3.60E-02	4.20E-01
	GOTERM_BP_FAT	regulation of T cell mediated immunity	7	4.90E-02	4.90E-01
	GOTERM_BP_FAT	positive regulation of production of molecular mediator of immune response	5	1.30E-01	7.50E-01
	GOTERM_BP_FAT	positive regulation of T cell cytokine production	3	1.30E-01	7.60E-01
	GOTERM_BP_FAT	regulation of production of molecular mediator of immune response	9	1.60E-01	8.20E-01
	GOTERM_BP_FAT	regulation of T cell cytokine production	3	1.80E-01	8.40E-01
	GOTERM_BP_FAT	positive regulation of immunoglobulin production	3	1.80E-01	8.40E-01
	GOTERM_BP_FAT	positive regulation of interleukin-2 production	3	2.30E-01	8.90E-01
	GOTERM_BP_FAT	regulation of immunoglobulin production	6	2.50E-01	9.10E-01
	GOTERM_BP_FAT	positive regulation of cytokine production during immune response	3	3.90E-01	9.70E-01
	GOTERM_BP_FAT	regulation of cytokine production during immune response	3	7.10E-01	1.00E+00
**Annotation Cluster 114**	**Enrichment Score: 0.96**		**Count**	**P_Value**	**Benjamini**
	GOTERM_BP_FAT	purine nucleoside triphosphate catabolic process	8	1.50E-02	2.50E-01
	GOTERM_BP_FAT	ribonucleoside triphosphate catabolic process	7	3.10E-02	3.80E-01
	GOTERM_BP_FAT	purine ribonucleoside triphosphate catabolic process	7	3.10E-02	3.80E-01
	GOTERM_BP_FAT	nucleoside triphosphate catabolic process	8	3.10E-02	3.90E-01
	GOTERM_BP_FAT	ATP catabolic process	6	5.00E-02	4.90E-01
	GOTERM_BP_FAT	purine ribonucleotide catabolic process	7	7.30E-02	6.00E-01
	GOTERM_BP_FAT	ribonucleotide catabolic process	7	1.00E-01	6.90E-01
	GOTERM_BP_FAT	purine nucleotide catabolic process	8	1.40E-01	7.70E-01
	GOTERM_BP_FAT	heterocycle catabolic process	14	2.10E-01	8.70E-01
	GOTERM_BP_FAT	nucleobase, nucleoside, nucleotide and nucleic acid catabolic process	10	3.50E-01	9.60E-01
	GOTERM_BP_FAT	nucleobase, nucleoside and nucleotide catabolic process	10	3.50E-01	9.60E-01
	GOTERM_BP_FAT	nucleotide catabolic process	9	3.60E-01	9.60E-01
	GOTERM_BP_FAT	nitrogen compound catabolic process	11	3.90E-01	9.70E-01
	GOTERM_MF_FAT	ADP binding	4	4.50E-01	9.90E-01
**Annotation Cluster 115**	**Enrichment Score: 0.96**		**Count**	**P_Value**	**Benjamini**
	GOTERM_CC_FAT	integral to organelle membrane	27	1.10E-02	1.30E-01
	GOTERM_CC_FAT	intrinsic to organelle membrane	29	2.90E-02	2.50E-01
	GOTERM_CC_FAT	integral to endoplasmic reticulum membrane	11	8.80E-02	4.70E-01
	GOTERM_CC_FAT	integral to Golgi membrane	10	1.10E-01	5.10E-01
	GOTERM_CC_FAT	intrinsic to Golgi membrane	10	1.50E-01	6.00E-01
	KEGG_PATHWAY	Glycosphingolipid biosynthesis	5	1.80E-01	5.40E-01
	GOTERM_BP_FAT	glycolipid metabolic process	7	2.20E-01	8.80E-01
	GOTERM_CC_FAT	intrinsic to endoplasmic reticulum membrane	12	2.30E-01	7.10E-01
	INTERPRO	Glycosyl transferase, family 29	5	2.40E-01	1.00E+00
	GOTERM_MF_FAT	sialyltransferase activity	5	2.50E-01	9.40E-01
**Annotation Cluster 116**	**Enrichment Score: 0.96**		**Count**	**P_Value**	**Benjamini**
	INTERPRO	Ribosomal protein L13, eukaryotic/archaeal	3	8.00E-02	9.50E-01
	INTERPRO	Ribosomal protein L13	3	1.20E-01	9.80E-01
	PIR_SUPERFAMILY	PIRSF002181:Ribosomal_L13	3	1.40E-01	1.00E+00
**Annotation Cluster 117**	**Enrichment Score: 0.95**		**Count**	**P_Value**	**Benjamini**
	SP_PIR_KEYWORDS	tpr repeat	29	2.70E-02	2.00E-01
	INTERPRO	Tetratricopeptide-like helical	29	3.60E-02	9.20E-01
	UP_SEQ_FEATURE	repeat:TPR 9	8	6.30E-02	1.00E+00
	UP_SEQ_FEATURE	repeat:TPR 2	26	6.40E-02	1.00E+00
	UP_SEQ_FEATURE	repeat:TPR 1	26	6.40E-02	1.00E+00
	UP_SEQ_FEATURE	repeat:TPR 3	24	6.90E-02	1.00E+00
	UP_SEQ_FEATURE	repeat:TPR 4	18	7.40E-02	1.00E+00
	INTERPRO	Tetratricopeptide repeat	21	9.60E-02	9.60E-01
	INTERPRO	Tetratricopeptide region	21	1.20E-01	9.80E-01
	SMART	TPR	21	1.30E-01	8.10E-01
	INTERPRO	Tetratricopeptide TPR-1	17	1.90E-01	9.90E-01
	UP_SEQ_FEATURE	repeat:TPR 7	10	2.80E-01	1.00E+00
	UP_SEQ_FEATURE	repeat:TPR 6	11	3.10E-01	1.00E+00
	UP_SEQ_FEATURE	repeat:TPR 8	8	3.60E-01	1.00E+00
	UP_SEQ_FEATURE	repeat:TPR 5	11	4.40E-01	1.00E+00
**Annotation Cluster 118**	**Enrichment Score: 0.95**		**Count**	**P_Value**	**Benjamini**
	GOTERM_BP_FAT	macromolecule catabolic process	122	3.00E-02	3.80E-01
	GOTERM_BP_FAT	cellular macromolecule catabolic process	113	3.80E-02	4.30E-01
	GOTERM_BP_FAT	ubiquitin-dependent protein catabolic process	42	5.30E-02	5.10E-01
	GOTERM_BP_FAT	protein catabolic process	96	6.80E-02	5.70E-01
	GOTERM_BP_FAT	proteolysis involved in cellular protein catabolic process	91	1.00E-01	6.90E-01
	GOTERM_BP_FAT	cellular protein catabolic process	91	1.10E-01	7.10E-01
	GOTERM_BP_FAT	modification-dependent protein catabolic process	85	1.60E-01	8.10E-01
	GOTERM_BP_FAT	modification-dependent macromolecule catabolic process	85	1.60E-01	8.10E-01
	GOTERM_BP_FAT	proteolysis	144	3.70E-01	9.60E-01
	SP_PIR_KEYWORDS	ubl conjugation pathway	60	6.50E-01	9.50E-01
**Annotation Cluster 119**	**Enrichment Score: 0.95**		**Count**	**P_Value**	**Benjamini**
	GOTERM_BP_FAT	nucleocytoplasmic transport	32	1.20E-02	2.20E-01
	GOTERM_BP_FAT	nuclear transport	32	1.40E-02	2.40E-01
	GOTERM_BP_FAT	protein targeting	41	1.50E-02	2.50E-01
	GOTERM_BP_FAT	protein localization in organelle	25	1.50E-01	7.90E-01
	GOTERM_BP_FAT	nuclear import	15	2.70E-01	9.20E-01
	GOTERM_BP_FAT	protein import into nucleus	14	3.40E-01	9.50E-01
	GOTERM_BP_FAT	protein import	20	3.60E-01	9.60E-01

ILMN_1656170	JAKMIP1	-0.443505301
ILMN_1704790	FADS6	-0.443508534
ILMN_2224955	XCL1	-0.443522363
ILMN_2051563	HIGD2B	-0.443558473
ILMN_2310703	RPS26	-0.443579728
ILMN_1686557	TIMM13	-0.443590721
ILMN_1792088	C1orf101	-0.443596714
ILMN_1679232	KIDINS220	-0.443638681
ILMN_3239673	POM121C	-0.443648152
ILMN_1677303	C5orf42	-0.443800556
ILMN_1731639	CASP3	-0.443924705
ILMN_3247979	LOC100132024	-0.44402494
ILMN_3307252	STMN1	-0.444058039
ILMN_2113417	CRYGC	-0.444089503
ILMN_1736340	ANGEL2	-0.444100488
ILMN_1699684	PBLD	-0.444269621
ILMN_3177122	LOC100127910	-0.444302694
ILMN_1804005	FBP2	-0.444370216
ILMN_2112942	KRTAP6-1	-0.444499187
ILMN_1719089	EXO1	-0.444520538
ILMN_1658563	KRT40	-0.444536418
ILMN_3249059	TRG11	-0.444563112
ILMN_3309159	MIR199B	-0.444574744
ILMN_2234187	CDO1	-0.444585031
ILMN_1672843	FBXO8	-0.444601413
ILMN_3309754	DNM3OS	-0.444611257
ILMN_1688160	WDR27	-0.444630754
ILMN_1681203	TWF1	-0.444658451
ILMN_1671366	LOC285780	-0.444681556
ILMN_1657603	AGBL1	-0.444687196
ILMN_1703091	C2orf27B	-0.444757214
ILMN_1747696	TPM3	-0.444770424
ILMN_1658548	LOC401180	-0.444803037
ILMN_1690423	SLC29A4	-0.444876832
ILMN_1907705	BM717102	-0.444902805
ILMN_1813325	PDE11A	-0.444921785
ILMN_1899048	AW295241	-0.44492497
ILMN_1800164	PPFIA1	-0.445038026
ILMN_1729179	SPATA5L1	-0.4450694
ILMN_1715376	LRRIQ3	-0.44511096
ILMN_1805180	CDAN1	-0.445111279
ILMN_1796926	PABPC5	-0.445300428
ILMN_1701831	GSTA1	-0.445338498
ILMN_3214579	LOC100131733	-0.445371657
ILMN_1750188	LAT	-0.445436292
ILMN_1748281	MAPK10	-0.445464811
ILMN_1711608	SSBP2	-0.445554373
ILMN_1710312	TMEM31	-0.445649649
ILMN_1877939	BX109006	-0.445879268
ILMN_1657870	ABL2	-0.445957138
ILMN_3204975	LOC201651	-0.446074298
ILMN_1803676	ENOSF1	-0.446170381
ILMN_3270641	HNRNPH3	-0.446179172
ILMN_1757183	LOC100192378	-0.446223234
ILMN_1814353	ARGFXP2	-0.446301019
ILMN_1692674	NKD1	-0.446349667
ILMN_2083595	BC132841	-0.446391702
ILMN_1693766	CEP135	-0.446428724
ILMN_2253286	PRKCZ	-0.446477273
ILMN_3191541	PTPRQ	-0.446495565
ILMN_1748141	AMOTL1	-0.446496285
ILMN_1685323	CXADR	-0.446526554
ILMN_2184525	LOC220115	-0.446636897
ILMN_3308500	MIR611	-0.446703416
ILMN_1823034	NA	-0.446722288
ILMN_1719998	DKFZp779O012	-0.446725303
ILMN_2393046	NHLRC3	-0.446756266
ILMN_2043265	ZFP106	-0.446806903
ILMN_3241878	RNU6ATAC	-0.446833868
ILMN_1808970	MORC1	-0.44686371
ILMN_2053567	FASTKD2	-0.446868207
ILMN_2148507	ANKRD13C	-0.447009162
ILMN_1813701	RASAL2	-0.447016925
ILMN_1776542	SLC35C2	-0.447091431
ILMN_1742009	C3orf30	-0.44712452
ILMN_1884828	AA770086	-0.447191044
ILMN_3228301	TMEM191C	-0.447193649
ILMN_1780117	CXorf48	-0.447328691
ILMN_1698849	CHRNA2	-0.447412221
ILMN_1795254	OR2T2	-0.447497026
ILMN_1806647	HEATR2	-0.447509548
ILMN_1675437	FLJ34503	-0.447512098
ILMN_3246724	CISD3	-0.447550123
ILMN_1706028	VCX3B	-0.447584249
ILMN_1663948	KIAA1013	-0.447607147
ILMN_3277872	BMS1	-0.447630518
ILMN_1781102	METTL21D	-0.447637794
ILMN_1781841	LRRN2	-0.447748083
ILMN_3245031	OR7E156P	-0.44778119
ILMN_1654766	NHEDC2	-0.447797351
ILMN_3294028	BM714821	-0.447801271

	SP_PIR_KEYWORDS	cyclin	6	6.10E-01	9.70E-01
**Annotation Cluster 148**	**Enrichment Score: 0.46**		**Count**	**P_Value**	**Benjamini**
	KEGG_PATHWAY	Regulation of autophagy	9	3.60E-02	9.00E-01
	INTERPRO	Four-helical cytokine, core	10	5.00E-02	1.00E+00
	INTERPRO	Interferon alpha	4	1.50E-01	1.00E+00
	SP_PIR_KEYWORDS	antiviral	4	1.70E-01	8.70E-01
	PIR_SUPERFAMILY	PIRSF001934:interferon alpha	4	2.10E-01	1.00E+00
	SMART	IFabd	4	2.30E-01	9.60E-01
	GOTERM_MF_FAT	interferon-alpha/beta receptor binding	3	2.50E-01	9.90E-01
	INTERPRO	Interferon alpha/beta/delta	4	2.70E-01	1.00E+00
	SP_PIR_KEYWORDS	leukocyte	3	4.80E-01	9.60E-01
	KEGG_PATHWAY	Antigen processing and presentation	10	5.90E-01	9.60E-01
	KEGG_PATHWAY	Autoimmune thyroid disease	6	6.90E-01	9.60E-01
	KEGG_PATHWAY	Cytosolic DNA-sensing pathway	5	8.80E-01	9.80E-01
	KEGG_PATHWAY	Natural killer cell mediated cytotoxicity	12	8.90E-01	9.80E-01
	SP_PIR_KEYWORDS	Antiviral defense	5	9.00E-01	1.00E+00
	GOTERM_BP_FAT	response to virus	9	9.00E-01	1.00E+00
	KEGG_PATHWAY	Toll-like receptor signaling pathway	8	9.40E-01	9.90E-01
	KEGG_PATHWAY	RIG-I-like receptor signaling pathway	5	9.60E-01	9.90E-01
**Annotation Cluster 149**	**Enrichment Score: 0.45**		**Count**	**P_Value**	**Benjamini**
	GOTERM_MF_FAT	monocarboxylic acid binding	9	2.20E-01	9.80E-01
	GOTERM_MF_FAT	fatty acid binding	6	3.80E-01	9.90E-01
	GOTERM_MF_FAT	acyl-CoA binding	3	5.20E-01	1.00E+00
**Annotation Cluster 150**	**Enrichment Score: 0.45**		**Count**	**P_Value**	**Benjamini**
	GOTERM_BP_FAT	negative regulation of protein modification process	19	7.70E-02	9.80E-01
	GOTERM_BP_FAT	negative regulation of cellular protein metabolic process	26	9.60E-02	9.80E-01
	GOTERM_BP_FAT	regulation of protein ubiquitination	16	1.10E-01	9.80E-01
	GOTERM_BP_FAT	regulation of protein modification process	39	1.20E-01	9.70E-01
	GOTERM_BP_FAT	negative regulation of protein metabolic process	26	1.30E-01	9.80E-01
	GOTERM_BP_FAT	negative regulation of protein ubiquitination	12	1.60E-01	9.80E-01
	GOTERM_BP_FAT	proteasomal protein catabolic process	14	2.80E-01	9.90E-01
	GOTERM_BP_FAT	proteasomal ubiquitin-dependent protein catabolic process	14	2.80E-01	9.90E-01
	GOTERM_BP_FAT	regulation of ubiquitin-protein ligase activity	11	3.10E-01	9.90E-01
	GOTERM_BP_FAT	regulation of ligase activity	11	3.50E-01	1.00E+00
	GOTERM_BP_FAT	negative regulation of ubiquitin-protein ligase activity	9	4.20E-01	1.00E+00
	GOTERM_BP_FAT	negative regulation of ligase activity	9	4.20E-01	1.00E+00
	GOTERM_BP_FAT	negative regulation of ubiquitin-protein ligase activity during mitotic cell cycle	8	5.40E-01	1.00E+00
	GOTERM_BP_FAT	positive regulation of ubiquitin-protein ligase activity	8	6.20E-01	1.00E+00
	GOTERM_BP_FAT	regulation of ubiquitin-protein ligase activity during mitotic cell cycle	8	6.40E-01	1.00E+00
	GOTERM_CC_FAT	proteasome core complex	3	6.50E-01	9.80E-01
	GOTERM_BP_FAT	positive regulation of ligase activity	8	6.70E-01	1.00E+00
	GOTERM_BP_FAT	positive regulation of protein ubiquitination	9	6.80E-01	1.00E+00
	GOTERM_BP_FAT	anaphase-promoting complex-dependent proteasomal ubiquitin-dependent protein catabolic process	7	7.00E-01	1.00E+00
	SP_PIR_KEYWORDS	proteasome	6	7.10E-01	9.80E-01
	GOTERM_BP_FAT	positive regulation of ubiquitin-protein ligase activity during mitotic cell cycle	7	7.40E-01	1.00E+00
	KEGG_PATHWAY	Proteasome	5	7.90E-01	9.70E-01
	GOTERM_CC_FAT	proteasome complex	6	8.00E-01	9.90E-01
**Annotation Cluster 151**	**Enrichment Score: 0.45**		**Count**	**P_Value**	**Benjamini**
	SMART	PTBI	3	3.40E-01	9.70E-01
	GOTERM_MF_FAT	insulin receptor binding	5	3.50E-01	9.90E-01
	UP_SEQ_FEATURE	domain:IRS-type PTB	3	3.60E-01	1.00E+00
	INTERPRO	Insulin receptor substrate-1, PTB	3	3.70E-01	1.00E+00
**Annotation Cluster 152**	**Enrichment Score: 0.44**		**Count**	**P_Value**	**Benjamini**
	GOTERM_MF_FAT	transmembrane receptor protein serine/threonine kinase activity	4	3.00E-01	9.90E-01
	GOTERM_MF_FAT	transforming growth factor beta receptor activity	4	3.00E-01	9.90E-01
	GOTERM_BP_FAT	BMP signaling pathway	7	3.20E-01	9.90E-01
	GOTERM_MF_FAT	growth factor binding	12	5.80E-01	1.00E+00
**Annotation Cluster 153**	**Enrichment Score: 0.44**		**Count**	**P_Value**	**Benjamini**
	GOTERM_BP_FAT	erythrocyte differentiation	8	1.70E-01	9.80E-01
	GOTERM_BP_FAT	erythrocyte homeostasis	8	2.60E-01	9.90E-01
	GOTERM_BP_FAT	myeloid cell differentiation	11	5.30E-01	1.00E+00
	GOTERM_BP_FAT	homeostasis of number of cells	10	7.50E-01	1.00E+00
**Annotation Cluster 154**	**Enrichment Score: 0.44**		**Count**	**P_Value**	**Benjamini**
	GOTERM_BP_FAT	muscle filament sliding	3	2.50E-01	9.90E-01
	GOTERM_BP_FAT	actin-mediated cell contraction	3	2.50E-01	9.90E-01
	GOTERM_BP_FAT	actin-myosin filament sliding	3	2.50E-01	9.90E-01
	SP_PIR_KEYWORDS	muscle protein	8	4.40E-01	9.50E-01
	GOTERM_BP_FAT	cytoskeleton-dependent intracellular transport	7	4.80E-01	1.00E+00
	GOTERM_BP_FAT	actin filament-based movement	3	7.20E-01	1.00E+00
**Annotation Cluster 155**	**Enrichment Score: 0.44**		**Count**	**P_Value**	**Benjamini**
	GOTERM_BP_FAT	cellular protein complex disassembly	4	2.70E-01	9.90E-01
	GOTERM_BP_FAT	protein complex disassembly	4	2.70E-01	9.90E-01
	GOTERM_BP_FAT	cellular component disassembly	8	3.30E-01	9.90E-01
	GOTERM_BP_FAT	macromolecular complex disassembly	4	5.30E-01	1.00E+00
	GOTERM_BP_FAT	cellular macromolecular complex disassembly	4	5.30E-01	1.00E+00
**Annotation Cluster 156**	**Enrichment Score: 0.44**		**Count**	**P_Value**	**Benjamini**
	UP_SEQ_FEATURE	domain:Laminin EGF-like 5	3	1.20E-01	1.00E+00
	UP_SEQ_FEATURE	domain:Laminin EGF-like 1	5	2.90E-01	1.00E+00
	UP_SEQ_FEATURE	domain:Laminin EGF-like 2	5	3.10E-01	1.00E+00
	SMART	EGF_Lam	6	3.90E-01	9.70E-01
	SP_PIR_KEYWORDS	laminin egf-like domain	5	3.90E-01	9.40E-01
	UP_SEQ_FEATURE	domain:Laminin EGF-like 4	3	4.00E-01	1.00E+00
	UP_SEQ_FEATURE	domain:Laminin EGF-like 3	4	4.10E-01	1.00E+00
	INTERPRO	EGF-like, laminin	6	4.40E-01	1.00E+00
	SMART	LamNT	3	4.80E-01	9.80E-01
	UP_SEQ_FEATURE	domain:Laminin N-terminal	3	5.10E-01	1.00E+00
	INTERPRO	Laminin, N-terminal	3	5.20E-01	1.00E+00
**Annotation Cluster 157**	**Enrichment Score: 0.43**		**Count**	**P_Value**	**Benjamini**
	INTERPRO	Pleckstrin homology-type	36	3.20E-01	1.00E+00
	SMART	PH	31	3.30E-01	9.70E-01
	UP_SEQ_FEATURE	domain:PH	28	3.60E-01	1.00E+00

ILMN_1793934	PISD	0.694466229		GOTERM_BP_FAT	protein import into nucleus, translocation	6	4.70E-01	9.80E-01
ILMN_1651504	FAM193A	0.694288895		GOTERM_BP_FAT	protein localization in nucleus	14	4.70E-01	9.80E-01
ILMN_2207533	RPS17	0.694219264	**Annotation Cluster 120**	**Enrichment Score: 0.95**		**Count**	**P_Value**	**Benjamini**
ILMN_1715705	SSNA1	0.694206495		GOTERM_BP_FAT	positive regulation of T cell mediated immunity	7	9.00E-03	1.90E-01
ILMN_1796177	GIPC1	0.694146954		GOTERM_BP_FAT	regulation of response to biotic stimulus	9	3.00E-02	3.80E-01
ILMN_2412192	CFH	0.694125476		GOTERM_BP_FAT	regulation of T cell mediated immunity	7	4.90E-02	4.90E-01
ILMN_1673682	GATAD2A	0.694054626		GOTERM_BP_FAT	positive regulation of response to tumor cell	3	2.90E-01	9.30E-01
ILMN_3310431	MIR874	0.6940248		GOTERM_BP_FAT	regulation of response to tumor cell	3	2.90E-01	9.30E-01
ILMN_1790637	C11orf80	0.69381097		GOTERM_BP_FAT	positive regulation of response to biotic stimulus	3	2.90E-01	9.30E-01
ILMN_3206132	RPS8	0.693780645		GOTERM_BP_FAT	regulation of immune response to tumor cell	3	2.90E-01	9.30E-01
ILMN_1697448	TXNIP	0.693559306		GOTERM_BP_FAT	positive regulation of immune response to tumor cell	3	2.90E-01	9.30E-01
ILMN_1771957	MAN1B1	0.693454847	**Annotation Cluster 121**	**Enrichment Score: 0.95**		**Count**	**P_Value**	**Benjamini**
ILMN_2329165	MYO1C	0.693441625		UP_SEQ_FEATURE	domain:SH3 1	9	6.20E-02	1.00E+00
ILMN_1678605	CDC123	0.693249623		UP_SEQ_FEATURE	domain:SH3 2	9	7.10E-02	1.00E+00
ILMN_3243238	TPSAB1	0.693151723		INTERPRO	Neutrophil cytosol factor 2	7	8.60E-02	9.60E-01
ILMN_1739847	EIF3D	0.693127913		UP_SEQ_FEATURE	domain:SH3 3	5	1.90E-01	1.00E+00
ILMN_1667476	LTBR	0.692938344		UP_SEQ_FEATURE	domain:SH3 4	3	2.50E-01	1.00E+00
ILMN_1752968	LAMB2	0.692886946	**Annotation Cluster 122**	**Enrichment Score: 0.94**		**Count**	**P_Value**	**Benjamini**
ILMN_1709880	RPLP0	0.692798447		GOTERM_CC_FAT	vesicle coat	11	2.40E-02	2.30E-01
ILMN_1747460	TMEM184B	0.692786108		GOTERM_CC_FAT	transport vesicle	15	5.10E-02	3.30E-01
ILMN_1798679	ANO9	0.692722927		GOTERM_CC_FAT	Golgi-associated vesicle	12	7.00E-02	4.20E-01
ILMN_1752526	RNF144B	0.692567269		GOTERM_CC_FAT	Golgi-associated vesicle membrane	8	8.30E-02	4.60E-01
ILMN_2047511	ADAP1	0.692514576		GOTERM_CC_FAT	trans-Golgi network transport vesicle	7	9.10E-02	4.70E-01
ILMN_1814971	TCF25	0.692385924		GOTERM_CC_FAT	clathrin vesicle coat	6	9.90E-02	4.90E-01
ILMN_2406410	RHBDD2	0.692371389		GOTERM_CC_FAT	AP-1 adaptor complex	3	1.40E-01	5.70E-01
ILMN_1713163	SMARCA5	0.69221211		GOTERM_CC_FAT	clathrin coat of trans-Golgi network vesicle	4	2.50E-01	7.20E-01
ILMN_1701613	RARRES3	0.692141628		GOTERM_CC_FAT	transport vesicle membrane	6	2.60E-01	7.50E-01
ILMN_1685763	WDR45L	0.692075599		GOTERM_CC_FAT	clathrin coated vesicle membrane	10	2.70E-01	7.60E-01
ILMN_1788149	NEK11	0.691901095		GOTERM_CC_FAT	trans-Golgi network transport vesicle membrane	4	2.80E-01	7.70E-01
ILMN_1710216	AVEN	0.691890678	**Annotation Cluster 123**	**Enrichment Score: 0.94**		**Count**	**P_Value**	**Benjamini**
ILMN_2205245	GPN2	0.691881428		GOTERM_BP_FAT	regulation of cell motion	38	1.20E-02	2.10E-01
ILMN_2325194	PHF6	0.691769677		GOTERM_BP_FAT	negative regulation of cell motion	15	3.30E-02	4.00E-01
ILMN_1756071	MFGE8	0.691769151		GOTERM_BP_FAT	regulation of cell migration	30	8.10E-02	6.20E-01
ILMN_1758055	YIF1B	0.691688991		GOTERM_BP_FAT	regulation of locomotion	33	9.50E-02	6.70E-01
ILMN_1729915	PILRA	0.691561592		GOTERM_BP_FAT	negative regulation of locomotion	12	1.70E-01	8.30E-01
ILMN_1724659	CIZ1	0.691559985		GOTERM_BP_FAT	negative regulation of cell migration	11	2.10E-01	8.70E-01
ILMN_1743763	TJAP1	0.691483609		GOTERM_BP_FAT	positive regulation of cell motion	17	2.20E-01	8.80E-01
ILMN_1738103	COPE	0.691473888		GOTERM_BP_FAT	positive regulation of cell migration	14	3.90E-01	9.70E-01
ILMN_1691843	RNPS1	0.691352724		GOTERM_BP_FAT	positive regulation of locomotion	15	4.20E-01	9.70E-01
ILMN_1660368	TRRAP	0.691302784	**Annotation Cluster 124**	**Enrichment Score: 0.93**		**Count**	**P_Value**	**Benjamini**
ILMN_1711414	MRPS27	0.691226588		SP_PIR_KEYWORDS	cell adhesion	62	7.50E-02	3.60E-01
ILMN_2127605	LRP3	0.691187833		GOTERM_BP_FAT	cell adhesion	103	1.50E-01	7.90E-01
ILMN_2070072	RPS7	0.691033314		GOTERM_BP_FAT	biological adhesion	103	1.50E-01	7.90E-01
ILMN_2382657	ARHGAP9	0.690935078	**Annotation Cluster 125**	**Enrichment Score: 0.92**		**Count**	**P_Value**	**Benjamini**
ILMN_1719286	CTSA	0.690920825		GOTERM_CC_FAT	histone methyltransferase complex	9	4.00E-03	6.40E-02
ILMN_1716983	LILRA2	0.690834909		GOTERM_CC_FAT	methyltransferase complex	9	4.00E-03	6.40E-02
ILMN_1700419	TMEM208	0.690780155		GOTERM_CC_FAT	MLL5-L complex	5	1.40E-02	1.60E-01
ILMN_1777129	CENPT	0.690667649		GOTERM_MF_FAT	protein methyltransferase activity	13	2.20E-02	4.10E-01
ILMN_3185223	NA	0.690566702		SP_PIR_KEYWORDS	s-adenosyl-l-methionine	20	3.80E-02	2.40E-01
ILMN_1808059	BCAS4	0.690435278		GOTERM_BP_FAT	protein amino acid alkylation	10	6.30E-02	5.60E-01
ILMN_1723021	ICMT	0.690385327		GOTERM_BP_FAT	protein amino acid methylation	10	6.30E-02	5.60E-01
ILMN_1782419	GNG11	0.690352743		GOTERM_BP_FAT	biopolymer methylation	15	6.40E-02	5.60E-01
ILMN_1660880	RNH1	0.690200071		UP_SEQ_FEATURE	binding site:S-adenosyl-L-methionine	11	8.20E-02	1.00E+00
ILMN_1672741	ITSN1	0.690177278		GOTERM_MF_FAT	histone methyltransferase activity	9	1.20E-01	8.30E-01
ILMN_1681591	PTPN1	0.690095849		GOTERM_BP_FAT	methylation	15	1.20E-01	7.40E-01
ILMN_1715698	RNASEK	0.690070735		UP_SEQ_FEATURE	region of interest:S-adenosyl-L-methionine binding	8	1.20E-01	1.00E+00
ILMN_1704015	SYNGAP1	0.690014695		GOTERM_MF_FAT	N-methyltransferase activity	11	1.40E-01	8.50E-01
ILMN_1667315	STAG3L1	0.689889352		INTERPRO	Post-SET zinc-binding region	5	1.60E-01	9.90E-01
ILMN_1779648	HIST3H2A	0.68987261		INTERPRO	MLL Transcription Factor	3	1.70E-01	9.90E-01
ILMN_2365544	NHP2	0.689812136		SMART	PostSET	5	1.80E-01	8.80E-01
ILMN_2375825	CD37	0.689696843		GOTERM_BP_FAT	DNA methylation	6	2.00E-01	8.60E-01
ILMN_1814985	PDLIM7	0.689583877		GOTERM_BP_FAT	DNA alkylation	6	2.00E-01	8.60E-01
ILMN_1745415	BBX	0.689492791		GOTERM_MF_FAT	lysine N-methyltransferase activity	7	2.20E-01	9.30E-01
ILMN_1711699	HIP1R	0.689300878		GOTERM_MF_FAT	protein-lysine N-methyltransferase activity	7	2.20E-01	9.30E-01
ILMN_1809208	KIAA1543	0.689265539		GOTERM_MF_FAT	histone-lysine N-methyltransferase activity	7	2.20E-01	9.30E-01
ILMN_1718646	MMP15	0.689126296		SP_PIR_KEYWORDS	methyltransferase	22	2.20E-01	6.60E-01
ILMN_1695354	BMF	0.689124585		GOTERM_BP_FAT	DNA modification	7	2.60E-01	9.20E-01
ILMN_1665895	LOC653160	0.689118285		GOTERM_BP_FAT	one-carbon metabolic process	18	3.00E-01	9.40E-01
ILMN_1797604	CAP1	0.689088772		UP_SEQ_FEATURE	domain:Post-SET	4	3.00E-01	1.00E+00
ILMN_2357809	G3BP1	0.689058574		GOTERM_BP_FAT	histone methylation	5	3.30E-01	9.50E-01
ILMN_1751330	RBCK1	0.688909808		UP_SEQ_FEATURE	domain:SET	8	3.80E-01	1.00E+00
ILMN_1800573	RPS21	0.688909574		INTERPRO	SET	8	4.20E-01	1.00E+00
ILMN_2303166	MBNL3	0.688885937		SMART	SET	8	4.70E-01	9.70E-01
ILMN_2342066	METRNL	0.688755307		KEGG_PATHWAY	Lysine degradation	5	9.10E-01	9.70E-01
ILMN_2376289	DBNL	0.688464839	**Annotation Cluster 126**	**Enrichment Score: 0.92**		**Count**	**P_Value**	**Benjamini**
ILMN_1728535	COPZ1	0.688399794		GOTERM_BP_FAT	regulation of response to biotic stimulus	9	3.00E-02	3.80E-01
ILMN_1703370	ZDHHC12	0.688352739		GOTERM_BP_FAT	regulation of multi-organism process	8	1.00E-01	7.00E-01
ILMN_1683475	TOMM40	0.688239125		GOTERM_BP_FAT	regulation of defense response to virus	5	2.40E-01	8.90E-01
ILMN_2340217	PTPRC	0.688190568		GOTERM_BP_FAT	regulation of defense response to virus by virus	3	2.90E-01	9.30E-01
ILMN_1664012	CANT1	0.68800571	**Annotation Cluster 127**	**Enrichment Score: 0.91**		**Count**	**P_Value**	**Benjamini**
ILMN_1782257	METTL17	0.687953005		GOTERM_MF_FAT	nuclear hormone receptor binding	19	7.80E-03	2.30E-01
ILMN_2038773	UBC	0.687900831		GOTERM_MF_FAT	hormone receptor binding	19	2.90E-02	4.60E-01
ILMN_1715908	NCAPH2	0.687870665		GOTERM_BP_FAT	steroid hormone receptor signaling pathway	12	1.40E-01	7.70E-01
ILMN_1783806	DTNBP1	0.687862023		GOTERM_BP_FAT	intracellular receptor-mediated signaling pathway	14	1.80E-01	8.40E-01
ILMN_1775243	RPL35	0.687747131		GOTERM_BP_FAT	androgen receptor signaling pathway	7	3.40E-01	9.50E-01
ILMN_3248562	SNORD49A	0.687741596		GOTERM_MF_FAT	androgen receptor binding	5	4.30E-01	9.90E-01
ILMN_2139100	SHISA5	0.687558011		GOTERM_MF_FAT	steroid hormone receptor binding	6	5.50E-01	1.00E+00
ILMN_1715636	EIF3B	0.687317218	**Annotation Cluster 128**	**Enrichment Score: 0.9**		**Count**	**P_Value**	**Benjamini**
ILMN_3275193	LOC643955	0.687285275		UP_SEQ_FEATURE	region of interest:Beta-galactoside binding 1	3	4.00E-02	9.90E-01
ILMN_1706539	KDM3B	0.687196685		UP_SEQ_FEATURE	region of interest:Beta-galactoside binding 2	3	4.00E-02	9.90E-01
ILMN_1755235	XPO6	0.687189921		UP_SEQ_FEATURE	domain:Galectin 2	3	1.60E-01	1.00E+00
ILMN_1799011	CEP250	0.687106734		UP_SEQ_FEATURE	domain:Galectin 1	3	1.60E-01	1.00E+00

ILMN_2367710	PKN1	-0.447812901		INTERPRO	Pleckstrin homology	31	4.80E-01	1.00E+00
ILMN_1776442	ACTRT2	-0.447958898	**Annotation Cluster 158**	**Enrichment Score: 0.43**		**Count**	**P_Value**	**Benjamini**
ILMN_1790810	HCN4	-0.448008569		GOTERM_MF_FAT	lysine N-acetyltransferase activity	8	1.00E-01	9.30E-01
ILMN_2168449	DHX15	-0.448008777		GOTERM_MF_FAT	histone acetyltransferase activity	8	1.00E-01	9.30E-01
ILMN_1810684	TINAG	-0.448081753		SP_PIR_KEYWORDS	Acyltransferase	20	2.50E-01	9.00E-01
ILMN_1665748	DCC	-0.448104212		GOTERM_MF_FAT	acetyltransferase activity	12	2.60E-01	9.90E-01
ILMN_3244597	SNORA38	-0.448238503		GOTERM_BP_FAT	histone H3 acetylation	5	2.90E-01	9.90E-01
ILMN_1694875	APPBP2	-0.448291002		GOTERM_MF_FAT	N-acetyltransferase activity	10	3.20E-01	9.90E-01
ILMN_1805658	LTV1	-0.448317062		INTERPRO	Acyl-CoA N-acyltransferase	5	4.20E-01	1.00E+00
ILMN_1717184	MZT2B	-0.448319024		GOTERM_BP_FAT	covalent chromatin modification	15	4.70E-01	1.00E+00
ILMN_3179396	MUC7	-0.448476914		GOTERM_MF_FAT	N-acyltransferase activity	10	5.30E-01	1.00E+00
ILMN_3308118	MIR125B1	-0.44855093		GOTERM_BP_FAT	histone modification	14	5.40E-01	1.00E+00
ILMN_1746871	TNRC6C	-0.448560495		GOTERM_BP_FAT	histone acetylation	6	5.90E-01	1.00E+00
ILMN_3310276	MIR425	-0.448585482		GOTERM_BP_FAT	protein amino acid acetylation	6	6.60E-01	1.00E+00
ILMN_1712019	ANKRD17	-0.448612328		GOTERM_BP_FAT	protein amino acid acylation	6	7.80E-01	1.00E+00
ILMN_1702739	TMEM146	-0.448618825		GOTERM_CC_FAT	histone acetyltransferase complex	5	8.00E-01	9.90E-01
ILMN_2399463	VAV3	-0.448622698	**Annotation Cluster 159**	**Enrichment Score: 0.43**		**Count**	**P_Value**	**Benjamini**
ILMN_1910322	LOC100131034	-0.448746157		SMART	GuKc	6	1.10E-01	9.60E-01
ILMN_3199958	PCDH11Y	-0.448951354		INTERPRO	Guanylate kinase/L-type calcium channel region	6	1.30E-01	1.00E+00
ILMN_1706691	CCDC87	-0.448974168		UP_SEQ_FEATURE	domain:PDZ 1	6	4.10E-01	1.00E+00
ILMN_1682763	ALB	-0.448976182		UP_SEQ_FEATURE	domain:PDZ 2	6	4.10E-01	1.00E+00
ILMN_1760635	RAD51C	-0.449013202		UP_SEQ_FEATURE	domain:PDZ 4	3	4.80E-01	1.00E+00
ILMN_1733035	MZT2A	-0.449051629		UP_SEQ_FEATURE	domain:PDZ 3	4	5.80E-01	1.00E+00
ILMN_1902378	DB299844	-0.449084309		UP_SEQ_FEATURE	domain:Guanylate kinase-like	3	7.10E-01	1.00E+00
ILMN_3305763	LOC728460	-0.449111853		INTERPRO	Guanylate kinase	3	7.20E-01	1.00E+00
ILMN_3240425	LOC348926	-0.449116943	**Annotation Cluster 160**	**Enrichment Score: 0.43**		**Count**	**P_Value**	**Benjamini**
ILMN_3308415	MIR337	-0.449117699		INTERPRO	Voltage-dependent calcium channel, L-type, beta subunit	3	5.80E-02	1.00E+00
ILMN_2399300	NAV2	-0.449153243		PIR_SUPERFAMILY	PIRSF015600:human voltage-dependent calcium channel beta chain	3	6.00E-02	1.00E+00
ILMN_1690621	GPR98	-0.449159777		SMART	GuKc	6	1.10E-01	9.60E-01
ILMN_3190972	DSTNP2	-0.449177351		INTERPRO	Guanylate kinase/L-type calcium channel region	6	1.30E-01	1.00E+00
ILMN_1757569	GSK3A	-0.449207462		GOTERM_BP_FAT	di-, tri-valent inorganic cation transport	23	2.40E-01	9.90E-01
ILMN_1759879	LOC220115	-0.449216282		GOTERM_BP_FAT	calcium ion transport	17	4.40E-01	1.00E+00
ILMN_1780222	SLC6A7	-0.449283042		KEGG_PATHWAY	Arrhythmogenic right ventricular cardiomyopathy (ARVC)	10	4.80E-01	9.50E-01
ILMN_1681515	CRLF1	-0.44929925		SP_PIR_KEYWORDS	calcium transport	10	5.40E-01	9.70E-01
ILMN_1696975	USP1	-0.449304164		GOTERM_MF_FAT	voltage-gated calcium channel activity	4	5.90E-01	1.00E+00
ILMN_1807101	F11	-0.449321796		GOTERM_CC_FAT	voltage-gated calcium channel complex	3	7.00E-01	9.80E-01
ILMN_2297997	LIPT1	-0.449324143		KEGG_PATHWAY	Hypertrophic cardiomyopathy (HCM)	9	7.50E-01	9.70E-01
ILMN_1662453	PRDM6	-0.449338283		GOTERM_MF_FAT	calcium channel activity	8	7.50E-01	1.00E+00
ILMN_1727668	LOC100131180	-0.449388055		SP_PIR_KEYWORDS	calcium channel	6	7.80E-01	9.90E-01
ILMN_3310476	MIR508	-0.449438747		KEGG_PATHWAY	Cardiac muscle contraction	8	7.80E-01	9.70E-01
ILMN_1853592	TFAP2B	-0.449498603		GOTERM_CC_FAT	calcium channel complex	3	8.00E-01	9.90E-01
ILMN_2382127	PPFIA1	-0.44958249		KEGG_PATHWAY	Dilated cardiomyopathy	9	8.20E-01	9.70E-01
ILMN_1860789	BM974067	-0.44963838	**Annotation Cluster 161**	**Enrichment Score: 0.43**		**Count**	**P_Value**	**Benjamini**
ILMN_2077733	NAA25	-0.449639227		GOTERM_BP_FAT	positive regulation of mesenchymal cell proliferation	4	2.70E-01	9.90E-01
ILMN_1900456	NA	-0.449660815		GOTERM_BP_FAT	regulation of mesenchymal cell proliferation	4	3.00E-01	9.90E-01
ILMN_1790688	AHNAK2	-0.44967952		GOTERM_BP_FAT	positive regulation of epithelial cell proliferation	5	6.40E-01	1.00E+00
ILMN_1665714	C4orf22	-0.449681545	**Annotation Cluster 162**	**Enrichment Score: 0.43**		**Count**	**P_Value**	**Benjamini**
ILMN_2208373	TMEM164	-0.449697587		UP_SEQ_FEATURE	domain:VWFA 1	4	2.30E-01	1.00E+00
ILMN_1694182	C21orf84	-0.449779612		UP_SEQ_FEATURE	domain:VWFA 2	4	2.60E-01	1.00E+00
ILMN_1786657	LOC283682	-0.44978002		SMART	VWA	10	3.80E-01	9.70E-01
ILMN_1662243	ING1	-0.449789464		INTERPRO	von Willebrand factor, type A	10	4.60E-01	1.00E+00
ILMN_1688504	OR1L8	-0.449815763		UP_SEQ_FEATURE	domain:VWFA	7	6.50E-01	1.00E+00
ILMN_1784175	KIAA1383	-0.449869106	**Annotation Cluster 163**	**Enrichment Score: 0.43**		**Count**	**P_Value**	**Benjamini**
ILMN_1868866	NA	-0.450002232		OMIM_DISEASE	Common variants at 30 loci contribute to polygenic dyslipidemia	9	6.40E-02	1.00E+00
ILMN_1740837	NEUROD1	-0.450006878		GOTERM_BP_FAT	glycerol ether biosynthetic process	4	1.50E-01	9.80E-01
ILMN_1765605	APOC4-APOC2	-0.450022578		GOTERM_BP_FAT	very-low-density lipoprotein particle remodeling	3	1.60E-01	9.80E-01
ILMN_1684186	C1orf141	-0.450060516		GOTERM_BP_FAT	triglyceride metabolic process	8	1.70E-01	9.80E-01
ILMN_1765085	TRMT6	-0.450065208		GOTERM_BP_FAT	glycerol ether metabolic process	9	1.70E-01	9.80E-01
ILMN_1708249	NR1D1	-0.45007284		GOTERM_BP_FAT	organic ether metabolic process	9	1.90E-01	9.80E-01
ILMN_1762670	C20orf70	-0.450084734		GOTERM_BP_FAT	triglyceride homeostasis	3	2.00E-01	9.90E-01
ILMN_3244678	SNORA5B	-0.450119526		GOTERM_BP_FAT	triglyceride-rich lipoprotein particle remodeling	3	2.50E-01	9.90E-01
ILMN_1891042	AI807473	-0.450152778		GOTERM_BP_FAT	low-density lipoprotein particle remodeling	3	2.50E-01	9.90E-01
ILMN_1704176	PSKH2	-0.450204164		GOTERM_BP_FAT	triglyceride biosynthetic process	3	2.50E-01	9.90E-01
ILMN_1739259	UBE4A	-0.450214582		GOTERM_BP_FAT	acylglycerol metabolic process	8	2.60E-01	9.90E-01
ILMN_3251278	DPPA3	-0.450233268		GOTERM_BP_FAT	neutral lipid metabolic process	8	2.80E-01	9.90E-01
ILMN_1851540	FGF12	-0.450331446		GOTERM_BP_FAT	macromolecular complex remodeling	4	3.60E-01	1.00E+00
ILMN_2354478	CYFIP2	-0.450375375		GOTERM_BP_FAT	protein-lipid complex remodeling	4	3.60E-01	1.00E+00
ILMN_1718783	EXTL1	-0.450445943		GOTERM_BP_FAT	plasma lipoprotein particle remodeling	4	3.60E-01	1.00E+00
ILMN_1674648	LOC338588	-0.450465728		OMIM_DISEASE	Genome-wide association analysis of metabolic traits in a birth cohort from a founder population	4	3.60E-01	1.00E+00
ILMN_1696708	C14orf166	-0.450484354		GOTERM_BP_FAT	high-density lipoprotein particle remodeling	3	3.70E-01	1.00E+00
ILMN_1814316	RBFOX1	-0.450537026		GOTERM_BP_FAT	neutral lipid biosynthetic process	3	3.70E-01	1.00E+00
ILMN_1733419	CCNE1	-0.450620195		GOTERM_BP_FAT	acylglycerol biosynthetic process	3	3.70E-01	1.00E+00
ILMN_1887977	BX094636	-0.450630545		GOTERM_BP_FAT	reverse cholesterol transport	3	5.20E-01	1.00E+00
ILMN_1662091	FLJ43860	-0.450695817		OMIM_DISEASE	Newly identified loci that influence lipid concentrations and risk of coronary artery disease	4	5.40E-01	1.00E+00
ILMN_2207170	WWC2	-0.450750891		GOTERM_BP_FAT	lipid homeostasis	6	6.40E-01	1.00E+00
ILMN_1769301	DNAH10OS	-0.450785348		OMIM_DISEASE	Six new loci associated with blood low-density lipoprotein cholesterol, high-density lipoprotein cholesterol or triglycerides in humans	4	6.50E-01	1.00E+00
ILMN_1670572	IDO2	-0.450831449		GOTERM_CC_FAT	protein-lipid complex	4	7.40E-01	9.80E-01
ILMN_1704101	HSF4	-0.450869197		GOTERM_CC_FAT	plasma lipoprotein particle	4	7.40E-01	9.80E-01
ILMN_1766219	HGC6.1.1	-0.450961747		GOTERM_BP_FAT	regulation of foam cell differentiation	3	7.60E-01	1.00E+00
ILMN_1663099	UBE2B	-0.451014558		GOTERM_CC_FAT	high-density lipoprotein particle	3	7.70E-01	9.90E-01
ILMN_1707137	C17orf97	-0.451016527		GOTERM_BP_FAT	sterol transport	4	7.70E-01	1.00E+00
ILMN_1698007	CCRL1	-0.451024822		GOTERM_BP_FAT	cholesterol transport	4	7.70E-01	1.00E+00
ILMN_1758943	FAM5C	-0.451070859		GOTERM_BP_FAT	sterol homeostasis	4	7.80E-01	1.00E+00
ILMN_2266309	CEPT1	-0.451112198		GOTERM_BP_FAT	cholesterol homeostasis	4	7.80E-01	1.00E+00
ILMN_1671398	NPAS3	-0.451124818		OMIM_DISEASE	Loci influencing lipid levels and coronary heart disease risk in 16 European population cohorts	3	7.90E-01	1.00E+00
ILMN_1734775	TISP43	-0.45113568	**Annotation Cluster 164**	**Enrichment Score: 0.43**		**Count**	**P_Value**	**Benjamini**
ILMN_2274199	SUPT3H	-0.451147358		GOTERM_BP_FAT	amino sugar metabolic process	5	2.70E-01	9.90E-01
ILMN_1652066	SNED1	-0.451164905		GOTERM_BP_FAT	glucosamine metabolic process	4	3.60E-01	1.00E+00
ILMN_3290889	AX748201	-0.451171704		GOTERM_BP_FAT	N-acetylglucosamine metabolic process	4	3.60E-01	1.00E+00
ILMN_3266944	RPS29	-0.451173746		GOTERM_CC_FAT	intrinsic to Golgi membrane	6	5.60E-01	9.70E-01
ILMN_2082810	BRD7	-0.451181175	**Annotation Cluster 165**	**Enrichment Score: 0.43**		**Count**	**P_Value**	**Benjamini**
ILMN_1732197	MN1	-0.451273067		GOTERM_MF_FAT	kinase regulator activity	13	2.90E-01	9.90E-01

ILMN_1775111	SND1	0.686717255
ILMN_2113333	LGALS9B	0.686662288
ILMN_1773347	CARD14	0.686631498
ILMN_2411723	RPSA	0.686628097
ILMN_1677877	UBE2L3	0.686490093
ILMN_1745242	PLSCR1	0.686490004
ILMN_1783636	COX6A1	0.686451705
ILMN_1742001	CD160	0.686319965
ILMN_1663379	FBXL15	0.686314752
ILMN_1771447	ZNF833P	0.686283476
ILMN_2340027	TSPAN4	0.686245124
ILMN_1670037	POLR2L	0.686141665
ILMN_1679401	TRPM4	0.686131347
ILMN_1683447	RPL35	0.685977161
ILMN_1652464	TUBA3E	0.68591112
ILMN_2412024	JMJD6	0.685785596
ILMN_1655625	GPATCH1	0.685732948
ILMN_2391765	C6orf48	0.685655911
ILMN_2334193	SLC22A2	0.685628417
ILMN_1795286	C6orf47	0.685560508
ILMN_1708534	PAX8	0.685397814
ILMN_3222402	FLJ45340	0.685381068
ILMN_1815734	FCHSD2	0.685305967
ILMN_1813604	NDUFB7	0.685281181
ILMN_1749579	PKHD1	0.685128982
ILMN_2370872	GRINA	0.685109859
ILMN_1763000	ADAP2	0.685053322
ILMN_1751075	SETD4	0.684873964
ILMN_1735014	KLF6	0.684850944
ILMN_1810577	RPS4X	0.684811427
ILMN_1791067	TESK1	0.684715357
ILMN_1803686	ADA	0.684715039
ILMN_1702592	WDR73	0.684679673
ILMN_2317923	TMEM132A	0.684677645
ILMN_1746588	TALDO1	0.684628684
ILMN_1705733	RPRD2	0.684532078
ILMN_2050112	RPSA	0.684492304
ILMN_1779034	NADSYN1	0.684450156
ILMN_3191596	STAG3L2	0.684346439
ILMN_1651958	MGP	0.684329929
ILMN_1785161	CHCHD6	0.684313981
ILMN_1770719	KIAA0664	0.684275455
ILMN_2116714	SLC39A1	0.684062935
ILMN_1737805	TPCN1	0.683876536
ILMN_3207060	RPL4	0.683759877
ILMN_1666670	RBX1	0.683743884
ILMN_2359453	ERGIC3	0.68366081
ILMN_1664922	FLNB	0.683633582
ILMN_1755850	ZNF350	0.683521295
ILMN_1712975	YIF1A	0.683513172
ILMN_1795918	AGAP3	0.683504337
ILMN_1811063	RPL29	0.683283692
ILMN_1750100	TUBB8	0.683042307
ILMN_1683700	TNFSF13	0.683012708
ILMN_1700690	VAT1	0.68295504
ILMN_2268026	C15orf44	0.682931494
ILMN_1671971	KRT8	0.6829028
ILMN_1733861	ARHGEF11	0.682879575
ILMN_1814823	FTL	0.682873452
ILMN_1796912	ARHGEF7	0.682848631
ILMN_1737576	RASA1	0.682789539
ILMN_1710885	RPSA	0.682731828
ILMN_3238001	LOC349114	0.682662627
ILMN_1775939	SF3B2	0.682631349
ILMN_1715301	FXYD2	0.682449405
ILMN_1704294	CDH3	0.682098062
ILMN_1708029	TMEM8B	0.682072756
ILMN_1713993	UBAC2	0.681886723
ILMN_2286870	CSNK1D	0.681829554
ILMN_1716488	PACS1	0.681747234
ILMN_2405031	TRIM24	0.681644452
ILMN_1662409	PRR13	0.681468652
ILMN_2165753	HLA-A	0.681360286
ILMN_1664440	TP53BP1	0.681355726
ILMN_1782403	PRR11	0.681324401
ILMN_1676600	SEC24C	0.681277727
ILMN_1728197	CLDN5	0.68126484
ILMN_1696434	LAMA1	0.681250869
ILMN_1720083	EHD4	0.681179854
ILMN_1707631	MED10	0.68102544
ILMN_1701477	CCDC101	0.68098973
ILMN_1669215	SRPK2	0.680880702
ILMN_1743755	C6orf226	0.680831211
ILMN_1720941	EVC	0.680770855
ILMN_1777190	CFD	0.680639226
ILMN_2389876	TGFB1I1	0.680518629
ILMN_1704672	OBFC2B	0.680479917
ILMN_2399174	TRAK1	0.680359331
ILMN_1675124	DDX17	0.68020088
ILMN_1677532	TARDBP	0.680106053
ILMN_2103591	MORC2	0.680078405
ILMN_1663145	NOMO3	0.680036137
ILMN_2234758	SRP14	0.679898981
ILMN_2092693	LSM12	0.679857254

	INTERPRO	Galectin, carbohydrate recognition domain	4	2.90E-01	1.00E+00
	SMART	GLECT	4	3.20E-01	9.50E-01
**Annotation Cluster 129**	**Enrichment Score: 0.9**		**Count**	**P_Value**	**Benjamini**
	SP_PIR_KEYWORDS	Aminoacyl-tRNA synthetase	10	3.90E-02	2.50E-01
	GOTERM_MF_FAT	ligase activity, forming carbon-oxygen bonds	11	7.20E-02	7.00E-01
	GOTERM_MF_FAT	ligase activity, forming aminoacyl-tRNA and related compounds	11	7.20E-02	7.00E-01
	GOTERM_MF_FAT	aminoacyl-tRNA ligase activity	11	7.20E-02	7.00E-01
	GOTERM_BP_FAT	amino acid activation	11	7.30E-02	6.00E-01
	GOTERM_BP_FAT	tRNA aminoacylation for protein translation	11	7.30E-02	6.00E-01
	GOTERM_BP_FAT	tRNA aminoacylation	11	7.30E-02	6.00E-01
	UP_SEQ_FEATURE	short sequence motif:"KMSKS" region	5	1.20E-01	1.00E+00
	KEGG_PATHWAY	Aminoacyl-tRNA biosynthesis	10	1.50E-01	5.00E-01
	UP_SEQ_FEATURE	short sequence motif:"HIGH" region	5	1.70E-01	1.00E+00
	INTERPRO	Rossmann-like alpha/beta/alpha sandwich fold	8	2.30E-01	1.00E+00
	GOTERM_BP_FAT	tRNA metabolic process	19	2.90E-01	9.30E-01
	INTERPRO	Aminoacyl-tRNA synthetase, class II, conserved region	4	3.60E-01	1.00E+00
	INTERPRO	Aminoacyl-tRNA synthetase, class I, conserved site	5	6.40E-01	1.00E+00
**Annotation Cluster 130**	**Enrichment Score: 0.89**		**Count**	**P_Value**	**Benjamini**
	INTERPRO	Protein kinase C-like, phorbol ester/diacylglycerol binding	15	3.80E-02	9.10E-01
	SMART	C1	15	5.20E-02	7.40E-01
	INTERPRO	Protein kinase C, phorbol ester/diacylglycerol binding	11	9.20E-02	9.60E-01
	GOTERM_MF_FAT	diacylglycerol binding	13	1.60E-01	8.80E-01
	UP_SEQ_FEATURE	zinc finger region:Phorbol-ester/DAG-type	8	2.60E-01	1.00E+00
	UP_SEQ_FEATURE	zinc finger region:Phorbol-ester/DAG-type 2	5	2.70E-01	1.00E+00
	UP_SEQ_FEATURE	zinc finger region:Phorbol-ester/DAG-type 1	5	2.70E-01	1.00E+00
**Annotation Cluster 131**	**Enrichment Score: 0.89**		**Count**	**P_Value**	**Benjamini**
	UP_SEQ_FEATURE	glycosylation site:O-linked (Xyl...) (glycosaminoglycan)	5	7.90E-02	1.00E+00
	SP_PIR_KEYWORDS	chondroitin sulfate proteoglycan	5	1.40E-01	5.30E-01
	SP_PIR_KEYWORDS	proteoglycan	10	2.00E-01	6.30E-01
**Annotation Cluster 132**	**Enrichment Score: 0.89**		**Count**	**P_Value**	**Benjamini**
	GOTERM_BP_FAT	positive regulation of MAPKKK cascade	11	9.30E-02	6.60E-01
	GOTERM_BP_FAT	positive regulation of stress-activated protein kinase signaling pathway	6	1.30E-01	7.60E-01
	GOTERM_BP_FAT	positive regulation of JNK cascade	5	1.80E-01	8.30E-01
**Annotation Cluster 133**	**Enrichment Score: 0.88**		**Count**	**P_Value**	**Benjamini**
	INTERPRO	Histone H3	3	1.20E-01	9.80E-01
	SMART	H3	3	1.30E-01	8.10E-01
	PIR_SUPERFAMILY	PIRSF002051:histone H3	3	1.40E-01	1.00E+00
**Annotation Cluster 134**	**Enrichment Score: 0.88**		**Count**	**P_Value**	**Benjamini**
	UP_SEQ_FEATURE	repeat:HEAT 3	12	3.40E-02	9.90E-01
	INTERPRO	Armadillo-like helical	24	5.00E-02	9.20E-01
	UP_SEQ_FEATURE	repeat:HEAT 7	7	6.20E-02	1.00E+00
	UP_SEQ_FEATURE	repeat:HEAT 8	6	7.10E-02	1.00E+00
	INTERPRO	HEAT	12	8.00E-02	9.60E-01
	UP_SEQ_FEATURE	repeat:HEAT 2	12	8.60E-02	1.00E+00
	UP_SEQ_FEATURE	repeat:HEAT 1	12	8.60E-02	1.00E+00
	UP_SEQ_FEATURE	repeat:HEAT 5	8	1.20E-01	1.00E+00
	UP_SEQ_FEATURE	repeat:HEAT 4	9	1.40E-01	1.00E+00
	UP_SEQ_FEATURE	repeat:HEAT 6	7	1.50E-01	1.00E+00
	UP_SEQ_FEATURE	domain:Importin N-terminal	4	2.40E-01	1.00E+00
	UP_SEQ_FEATURE	repeat:HEAT 9	4	2.70E-01	1.00E+00
	UP_SEQ_FEATURE	repeat:HEAT 10	4	2.70E-01	1.00E+00
	INTERPRO	Importin-beta, N-terminal	4	2.90E-01	1.00E+00
	UP_SEQ_FEATURE	repeat:HEAT 11	3	3.90E-01	1.00E+00
	GOTERM_BP_FAT	protein import into nucleus, docking	4	3.90E-01	9.70E-01
**Annotation Cluster 135**	**Enrichment Score: 0.88**		**Count**	**P_Value**	**Benjamini**
	GOTERM_CC_FAT	organelle membrane	183	7.40E-04	1.70E-02
	GOTERM_CC_FAT	organelle envelope	103	1.30E-02	1.60E-01
	GOTERM_CC_FAT	envelope	103	1.50E-02	1.60E-01
	GOTERM_CC_FAT	organelle inner membrane	50	2.20E-01	7.00E-01
	SP_PIR_KEYWORDS	mitochondrion inner membrane	27	3.00E-01	7.50E-01
	GOTERM_CC_FAT	mitochondrial envelope	58	4.60E-01	9.00E-01
	SP_PIR_KEYWORDS	mitochondrion	102	4.90E-01	8.90E-01
	GOTERM_CC_FAT	mitochondrial part	81	5.00E-01	9.20E-01
	GOTERM_CC_FAT	mitochondrial membrane	53	5.60E-01	9.40E-01
	GOTERM_CC_FAT	mitochondrial inner membrane	41	5.80E-01	9.40E-01
	GOTERM_CC_FAT	mitochondrion	144	5.90E-01	9.40E-01
**Annotation Cluster 136**	**Enrichment Score: 0.88**		**Count**	**P_Value**	**Benjamini**
	GOTERM_BP_FAT	natural killer cell mediated cytotoxicity	4	7.70E-02	6.10E-01
	GOTERM_BP_FAT	natural killer cell mediated immunity	4	7.70E-02	6.10E-01
	GOTERM_BP_FAT	leukocyte mediated cytotoxicity	4	1.40E-01	7.70E-01
	GOTERM_BP_FAT	cell killing	5	3.90E-01	9.70E-01
**Annotation Cluster 137**	**Enrichment Score: 0.85**		**Count**	**P_Value**	**Benjamini**
	KEGG_PATHWAY	Pentose phosphate pathway	8	6.90E-02	3.40E-01
	GOTERM_MF_FAT	monosaccharide binding	9	1.30E-01	8.50E-01
	GOTERM_BP_FAT	fructose metabolic process	5	1.80E-01	8.30E-01
	GOTERM_BP_FAT	fructose 6-phosphate metabolic process	3	2.30E-01	8.90E-01
**Annotation Cluster 138**	**Enrichment Score: 0.84**		**Count**	**P_Value**	**Benjamini**
	UP_SEQ_FEATURE	domain:EH	3	1.10E-01	1.00E+00
	INTERPRO	EPS15 homology (EH)	4	1.50E-01	9.90E-01
	SMART	EH	4	1.70E-01	8.70E-01
**Annotation Cluster 139**	**Enrichment Score: 0.84**		**Count**	**P_Value**	**Benjamini**
	INTERPRO	Calponin-like actin-binding	18	5.30E-03	4.90E-01
	SMART	CH	18	8.20E-03	3.80E-01
	UP_SEQ_FEATURE	domain:Actin-binding	7	2.70E-02	9.90E-01
	UP_SEQ_FEATURE	domain:CH 1	8	3.80E-02	9.90E-01
	UP_SEQ_FEATURE	domain:CH 2	8	3.80E-02	9.90E-01
	UP_SEQ_FEATURE	domain:CH	10	6.10E-02	1.00E+00
	INTERPRO	Actinin-type, actin-binding, conserved site	7	6.10E-02	9.40E-01
	UP_SEQ_FEATURE	repeat:Spectrin 4	6	1.00E-01	1.00E+00
	INTERPRO	Spectrin repeat	6	1.20E-01	9.70E-01
	UP_SEQ_FEATURE	repeat:Spectrin 3	6	1.20E-01	1.00E+00
	UP_SEQ_FEATURE	repeat:Spectrin 2	6	2.00E-01	1.00E+00
	UP_SEQ_FEATURE	repeat:Spectrin 1	6	2.00E-01	1.00E+00
	UP_SEQ_FEATURE	repeat:Spectrin 8	4	2.00E-01	1.00E+00

ILMN_1855868	BX093310	-0.451331075
ILMN_1721727	USP6	-0.451404358
ILMN_2152828	KIF16B	-0.451493441
ILMN_2231985	HSPA13	-0.451513311
ILMN_2346451	ACTR3B	-0.45152437
ILMN_1664698	UNC119	-0.451559593
ILMN_1796813	EED	-0.451559871
ILMN_2339779	ATP6V1E1	-0.451584432
ILMN_1770245	EPB41L5	-0.451598535
ILMN_1804470	C17orf109	-0.451601199
ILMN_2179533	C4A	-0.451608326
ILMN_1839138	BU632831	-0.451610381
ILMN_1779423	MRPS14	-0.451646238
ILMN_1663156	DDX31	-0.451662968
ILMN_1718236	LL0XNC01-19D8.1	-0.451680163
ILMN_1689831	FLJ11235	-0.451680904
ILMN_1902112	AA725558	-0.451820475
ILMN_3265228	LOC653581	-0.45182171
ILMN_1686750	MGEA5	-0.451876182
ILMN_1653783	MLC1	-0.45190179
ILMN_1685260	DNM1L	-0.451901885
ILMN_3311115	MIR663B	-0.451939116
ILMN_2373073	LIG4	-0.452046064
ILMN_2204826	SEPP1	-0.452164644
ILMN_1668498	CWF19L2	-0.452190494
ILMN_1896431	AK127458	-0.452238922
ILMN_1714461	RNF14	-0.452276499
ILMN_3300937	C13orf25 v_1	-0.452305745
ILMN_1779584	UTP18	-0.452364263
ILMN_1782609	STAG2	-0.45237227
ILMN_3248146	LOC100128542	-0.452375432
ILMN_1848552	NFIB	-0.452412585
ILMN_1686804	CDK20	-0.452480917
ILMN_1862939	BX100265	-0.452678126
ILMN_1688629	ZNF274	-0.452718779
ILMN_1773850	FXC1	-0.452756995
ILMN_3236346	ZNF286B	-0.452806789
ILMN_1818248	NCRNA00160	-0.452812817
ILMN_1671642	CSNK1A1L	-0.452891287
ILMN_1808487	PLA2G12B	-0.4529136
ILMN_1731314	OR10H3	-0.452948706
ILMN_1755047	LRRC2	-0.45300658
ILMN_3247592	ZNF830	-0.453018831
ILMN_1811278	RNF186	-0.453047927
ILMN_1784395	NA	-0.453048809
ILMN_1783645	DKFZp666P158	-0.453100566
ILMN_1661940	CAMTA1	-0.453156789
ILMN_1738816	FOXO1	-0.453196211
ILMN_1659631	LOC440905	-0.453198176
ILMN_1764186	ATXN1L	-0.453309646
ILMN_1799293	MINA53	-0.453346269
ILMN_1663367	SERHL	-0.45352334
ILMN_1761438	truncated AKR	-0.453560044
ILMN_2087629	NOL4	-0.453572276
ILMN_3299416	LOC729020	-0.453577253
ILMN_3191746	BCAR4	-0.453581402
ILMN_2339748	RNF13	-0.453588655
ILMN_1786388	RNF113A	-0.45358978
ILMN_1670959	CEACAM5	-0.453723327
ILMN_1738767	PLP2	-0.453741479
ILMN_1718018	PFN3	-0.453795538
ILMN_1695969	TMPRSS15	-0.4538438
ILMN_1859672	AI638857	-0.453955341
ILMN_2149494	NPL	-0.453958514
ILMN_1698060	FLJ43860	-0.453968512
ILMN_1658605	NA	-0.454033952
ILMN_1736060	GLT6D1	-0.454051797
ILMN_3237956	ZC3H12C	-0.454075711
ILMN_1806800	TRADD	-0.454086329
ILMN_1808272	KCNJ10	-0.454187942
ILMN_1893798	CK823348	-0.454212595
ILMN_2128931	FAT2	-0.454283376
ILMN_3224722	NA	-0.454290694
ILMN_1767274	FBLL1	-0.454352567
ILMN_1690048	OR4F21	-0.454415141
ILMN_3275685	LOC727721	-0.454522545
ILMN_1752791	PARP8	-0.454564307
ILMN_1769673	ZNF655	-0.45460196
ILMN_2038777	ACTB	-0.45465125
ILMN_1880796	BE439524	-0.454668251
ILMN_3199911	BX106402	-0.454680283
ILMN_3185278	LOC100128542	-0.454783315
ILMN_1815938	TRPM3	-0.454826748
ILMN_1660400	CELF4	-0.454880016
ILMN_1717904	SORBS3	-0.454882914
ILMN_1888546	AI039233	-0.454979676
ILMN_1808238	RBPMS2	-0.455000533
ILMN_1803819	IQGAP1	-0.455007066
ILMN_1719788	LDHAL6B	-0.455027337
ILMN_1804329	TUSC2	-0.455035191
ILMN_1730477	TAS2R43	-0.45505705
ILMN_1666453	STK3	-0.45509784
ILMN_2403911	ARFIP1	-0.4551006
ILMN_1664118	CADM2	-0.455124987

	GOTERM_MF_FAT	protein kinase inhibitor activity	6	3.40E-01	9.90E-01
	GOTERM_MF_FAT	kinase inhibitor activity	6	3.60E-01	9.90E-01
	GOTERM_MF_FAT	protein kinase regulator activity	11	3.60E-01	9.90E-01
	GOTERM_MF_FAT	protein serine/threonine kinase inhibitor activity	3	5.60E-01	1.00E+00
**Annotation Cluster 166**	**Enrichment Score: 0.42**		**Count**	**P_Value**	**Benjamini**
	GOTERM_BP_FAT	telencephalon development	10	2.50E-01	9.90E-01
	GOTERM_BP_FAT	pallium development	7	3.60E-01	1.00E+00
	GOTERM_BP_FAT	hippocampus development	4	4.20E-01	1.00E+00
	GOTERM_BP_FAT	limbic system development	5	4.40E-01	1.00E+00
	GOTERM_BP_FAT	cerebral cortex development	5	4.70E-01	1.00E+00
**Annotation Cluster 167**	**Enrichment Score: 0.42**		**Count**	**P_Value**	**Benjamini**
	SP_PIR_KEYWORDS	dioxygenase	10	2.50E-01	9.00E-01
	UP_SEQ_FEATURE	metal ion-binding site:Iron; catalytic	5	2.70E-01	1.00E+00
	GOTERM_MF_FAT	oxidoreductase activity, acting on single donors with incorporation of molecular oxygen, incorporation of two atoms of oxygen	9	4.10E-01	1.00E+00
	GOTERM_MF_FAT	oxidoreductase activity, acting on single donors with incorporation of molecular oxygen	9	4.30E-01	1.00E+00
	GOTERM_MF_FAT	L-ascorbic acid binding	3	6.70E-01	1.00E+00
**Annotation Cluster 168**	**Enrichment Score: 0.42**		**Count**	**P_Value**	**Benjamini**
	INTERPRO	HEAT	9	2.20E-01	1.00E+00
	UP_SEQ_FEATURE	repeat:HEAT 14	3	2.40E-01	1.00E+00
	UP_SEQ_FEATURE	repeat:HEAT 1	9	2.60E-01	1.00E+00
	UP_SEQ_FEATURE	repeat:HEAT 2	9	2.60E-01	1.00E+00
	UP_SEQ_FEATURE	repeat:HEAT 12	3	2.80E-01	1.00E+00
	UP_SEQ_FEATURE	repeat:HEAT 13	3	2.80E-01	1.00E+00
	UP_SEQ_FEATURE	repeat:HEAT 11	3	3.20E-01	1.00E+00
	UP_SEQ_FEATURE	repeat:HEAT 6	5	3.90E-01	1.00E+00
	UP_SEQ_FEATURE	repeat:HEAT 7	4	4.70E-01	1.00E+00
	UP_SEQ_FEATURE	repeat:HEAT 4	6	4.80E-01	1.00E+00
	UP_SEQ_FEATURE	repeat:HEAT 10	3	4.80E-01	1.00E+00
	UP_SEQ_FEATURE	repeat:HEAT 9	3	4.80E-01	1.00E+00
	UP_SEQ_FEATURE	repeat:HEAT 5	5	5.10E-01	1.00E+00
	INTERPRO	Armadillo-like helical	14	6.10E-01	1.00E+00
	UP_SEQ_FEATURE	repeat:HEAT 8	3	6.10E-01	1.00E+00
	UP_SEQ_FEATURE	repeat:HEAT 3	6	6.20E-01	1.00E+00
**Annotation Cluster 169**	**Enrichment Score: 0.41**		**Count**	**P_Value**	**Benjamini**
	SP_PIR_KEYWORDS	amino-acid transport	7	1.70E-01	8.70E-01
	INTERPRO	Amino acid permease-associated region	5	2.00E-01	1.00E+00
	GOTERM_BP_FAT	amino acid transport	13	2.40E-01	9.90E-01
	GOTERM_BP_FAT	amine transport	15	3.70E-01	1.00E+00
	GOTERM_BP_FAT	carboxylic acid transport	18	3.90E-01	1.00E+00
	GOTERM_BP_FAT	organic acid transport	18	4.00E-01	1.00E+00
	INTERPRO	Amino acid/polyamine transporter I	3	4.80E-01	1.00E+00
	PIR_SUPERFAMILY	PIRSF006060:AA_transporter	3	4.90E-01	1.00E+00
	GOTERM_MF_FAT	amine transmembrane transporter activity	9	5.40E-01	1.00E+00
	GOTERM_MF_FAT	L-amino acid transmembrane transporter activity	5	5.90E-01	1.00E+00
	GOTERM_MF_FAT	amino acid transmembrane transporter activity	6	7.70E-01	1.00E+00
**Annotation Cluster 170**	**Enrichment Score: 0.41**		**Count**	**P_Value**	**Benjamini**
	GOTERM_BP_FAT	response to estradiol stimulus	8	3.50E-01	1.00E+00
	GOTERM_BP_FAT	response to steroid hormone stimulus	23	3.80E-01	1.00E+00
	GOTERM_BP_FAT	response to estrogen stimulus	13	4.40E-01	1.00E+00
**Annotation Cluster 171**	**Enrichment Score: 0.41**		**Count**	**P_Value**	**Benjamini**
	GOTERM_BP_FAT	cell-cell signaling	69	3.00E-01	9.90E-01
	GOTERM_BP_FAT	transmission of nerve impulse	40	4.00E-01	1.00E+00
	GOTERM_BP_FAT	synaptic transmission	33	5.00E-01	1.00E+00
**Annotation Cluster 172**	**Enrichment Score: 0.41**		**Count**	**P_Value**	**Benjamini**
	GOTERM_CC_FAT	Sin3 complex	3	2.10E-01	9.50E-01
	GOTERM_CC_FAT	Sin3-type complex	3	2.10E-01	9.50E-01
	GOTERM_CC_FAT	NuRD complex	3	4.20E-01	9.60E-01
	GOTERM_CC_FAT	histone deacetylase complex	6	4.50E-01	9.70E-01
	GOTERM_CC_FAT	chromatin remodeling complex	8	6.50E-01	9.80E-01
	GOTERM_CC_FAT	transcriptional repressor complex	5	6.60E-01	9.80E-01
**Annotation Cluster 173**	**Enrichment Score: 0.41**		**Count**	**P_Value**	**Benjamini**
	GOTERM_CC_FAT	vacuole	34	1.30E-01	9.30E-01
	SP_PIR_KEYWORDS	lysosome	17	5.00E-01	9.60E-01
	GOTERM_CC_FAT	lytic vacuole	23	5.90E-01	9.70E-01
	GOTERM_CC_FAT	lysosome	23	5.90E-01	9.70E-01
**Annotation Cluster 174**	**Enrichment Score: 0.41**		**Count**	**P_Value**	**Benjamini**
	GOTERM_MF_FAT	gap junction channel activity	3	2.90E-01	9.90E-01
	GOTERM_MF_FAT	wide pore channel activity	3	3.30E-01	9.90E-01
	SMART	CNX	4	3.50E-01	9.70E-01
	INTERPRO	Connexins	4	3.60E-01	1.00E+00
	INTERPRO	Gap junction protein, cysteine-rich domain	4	3.60E-01	1.00E+00
	INTERPRO	Connexin, N-terminal	4	3.90E-01	1.00E+00
	INTERPRO	Connexin region	4	3.90E-01	1.00E+00
	INTERPRO	Connexin, conserved site	4	3.90E-01	1.00E+00
	PIR_SUPERFAMILY	PIRSF002292:gap junction protein	3	4.20E-01	1.00E+00
	GOTERM_CC_FAT	connexon complex	4	4.30E-01	9.60E-01
	SP_PIR_KEYWORDS	gap junction	4	4.90E-01	9.60E-01
	GOTERM_CC_FAT	gap junction	4	6.20E-01	9.70E-01
**Annotation Cluster 175**	**Enrichment Score: 0.4**		**Count**	**P_Value**	**Benjamini**
	GOTERM_CC_FAT	vacuolar proton-transporting V-type ATPase complex	3	2.90E-01	9.60E-01
	GOTERM_CC_FAT	vacuolar membrane	8	4.00E-01	9.60E-01
	GOTERM_CC_FAT	vacuolar part	8	5.40E-01	9.70E-01
**Annotation Cluster 176**	**Enrichment Score: 0.4**		**Count**	**P_Value**	**Benjamini**
	GOTERM_BP_FAT	midgut development	3	9.00E-02	9.80E-01
	GOTERM_BP_FAT	morphogenesis of embryonic epithelium	9	2.70E-01	9.90E-01
	GOTERM_BP_FAT	embryonic epithelial tube formation	7	3.00E-01	9.90E-01
	GOTERM_BP_FAT	tube lumen formation	7	3.20E-01	9.90E-01
	GOTERM_BP_FAT	mesenchymal cell development	7	4.60E-01	1.00E+00
	GOTERM_BP_FAT	mesenchymal cell differentiation	7	4.60E-01	1.00E+00
	GOTERM_BP_FAT	neural crest cell differentiation	5	4.70E-01	1.00E+00
	GOTERM_BP_FAT	neural crest cell development	5	4.70E-01	1.00E+00
	GOTERM_BP_FAT	mesenchyme development	7	4.80E-01	1.00E+00
	GOTERM_BP_FAT	ameboidal cell migration	5	5.60E-01	1.00E+00
	GOTERM_BP_FAT	neural crest cell migration	3	7.20E-01	1.00E+00

ILMN_1737298	MAT2A	0.679827006		UP_SEQ_FEATURE	repeat:Spectrin 9	4	2.00E-01	1.00E+00
ILMN_1665943	MAP4K1	0.679808081		UP_SEQ_FEATURE	repeat:Spectrin 6	4	2.00E-01	1.00E+00
ILMN_1679358	ZDHHC5	0.679527043		UP_SEQ_FEATURE	repeat:Spectrin 7	4	2.00E-01	1.00E+00
ILMN_1677800	SGTA	0.679354713		UP_SEQ_FEATURE	repeat:Spectrin 5	4	2.40E-01	1.00E+00
ILMN_1673804	ZNF426	0.679337048		INTERPRO	Spectrin/alpha-actinin	6	3.50E-01	1.00E+00
ILMN_1789839	GTF3C1	0.679195114		SMART	SPEC	6	3.90E-01	9.70E-01
ILMN_1746968	PHF1	0.679013586		UP_SEQ_FEATURE	repeat:Spectrin 16	3	4.40E-01	1.00E+00
ILMN_1743910	PANK4	0.678918564		UP_SEQ_FEATURE	repeat:Spectrin 17	3	4.40E-01	1.00E+00
ILMN_1674038	CTSD	0.678821669		UP_SEQ_FEATURE	repeat:Spectrin 14	3	4.40E-01	1.00E+00
ILMN_1755883	RPS27A	0.678720553		UP_SEQ_FEATURE	repeat:Spectrin 15	3	4.40E-01	1.00E+00
ILMN_1709114	TAB1	0.678693357		UP_SEQ_FEATURE	repeat:Spectrin 12	3	4.40E-01	1.00E+00
ILMN_1768284	P2RY8	0.678472555		UP_SEQ_FEATURE	repeat:Spectrin 13	3	4.40E-01	1.00E+00
ILMN_1666449	LOC162427	0.678254572		UP_SEQ_FEATURE	repeat:Spectrin 11	3	4.40E-01	1.00E+00
ILMN_1706434	CSDAP1	0.678202914		UP_SEQ_FEATURE	repeat:Spectrin 10	3	4.40E-01	1.00E+00
ILMN_3248008	TUBB	0.678176598	**Annotation Cluster 140**	**Enrichment Score: 0.84**		**Count**	**P_Value**	**Benjamini**
ILMN_1700109	PTOV1	0.6781494		GOTERM_BP_FAT	response to organic substance	123	1.90E-03	6.00E-02
ILMN_1807493	ACVRL1	0.678119532		GOTERM_BP_FAT	cellular response to hormone stimulus	24	1.00E-01	6.90E-01
ILMN_1689446	EIF3G	0.677980343		GOTERM_BP_FAT	response to hormone stimulus	56	1.70E-01	8.20E-01
ILMN_2391912	SEC14L1	0.677749592		GOTERM_BP_FAT	insulin receptor signaling pathway	8	2.10E-01	8.70E-01
ILMN_1687857	ST6GALNAC4	0.677553537		GOTERM_BP_FAT	response to peptide hormone stimulus	25	2.20E-01	8.80E-01
ILMN_1697544	SLC25A29	0.677453833		GOTERM_BP_FAT	response to endogenous stimulus	59	2.70E-01	9.20E-01
ILMN_1801130	STOML1	0.677406535		GOTERM_BP_FAT	cellular response to insulin stimulus	12	2.80E-01	9.20E-01
ILMN_1740160	PLCG1	0.677277017		GOTERM_BP_FAT	response to insulin stimulus	16	3.40E-01	9.50E-01
ILMN_1801307	TNFSF10	0.677247548		GOTERM_BP_FAT	response to steroid hormone stimulus	23	7.90E-01	1.00E+00
ILMN_2147133	NBPF1	0.677063664	**Annotation Cluster 141**	**Enrichment Score: 0.83**		**Count**	**P_Value**	**Benjamini**
ILMN_1738369	TUFM	0.676960033		INTERPRO	Cell surface receptor IPT/TIG	10	2.20E-02	8.20E-01
ILMN_1693650	FES	0.676938331		SMART	IPT	10	2.90E-02	6.00E-01
ILMN_1769705	H3F3C	0.676923188		INTERPRO	Plexin/semaphorin/integrin	11	5.10E-02	9.20E-01
ILMN_1661306	RPL29	0.676862337		UP_SEQ_FEATURE	domain:IPT/TIG 3	5	6.20E-02	1.00E+00
ILMN_1786852	ZCCHC3	0.676740179		UP_SEQ_FEATURE	domain:IPT/TIG 2	5	6.20E-02	1.00E+00
ILMN_1693986	WHAMML2	0.676705172		SMART	PSI	11	6.50E-02	7.50E-01
ILMN_1807169	TINAGL1	0.676688423		UP_SEQ_FEATURE	domain:IPT/TIG 4	3	2.50E-01	1.00E+00
ILMN_1783226	SSR2	0.676511775		INTERPRO	Plexin cytoplasmic region	3	3.20E-01	1.00E+00
ILMN_1651385	MFN2	0.676415783		INTERPRO	Plexin	6	3.50E-01	1.00E+00
ILMN_3249419	FLJ14186	0.676375007		UP_SEQ_FEATURE	domain:IPT/TIG 1	3	3.90E-01	1.00E+00
ILMN_1732615	ASMTL	0.676373858		UP_SEQ_FEATURE	domain:Sema	5	5.30E-01	1.00E+00
ILMN_2406169	PKIG	0.676299555		INTERPRO	Semaphorin/CD100 antigen	5	5.60E-01	1.00E+00
ILMN_1686981	SULF2	0.676187438		SMART	Sema	5	6.00E-01	9.90E-01
ILMN_3181411	ATL1	0.67616968	**Annotation Cluster 142**	**Enrichment Score: 0.83**		**Count**	**P_Value**	**Benjamini**
ILMN_1725193	IGFBP2	0.676160599		GOTERM_CC_FAT	endoplasmic reticulum part	62	1.40E-02	1.60E-01
ILMN_1654032	ZER1	0.676066546		GOTERM_CC_FAT	nuclear envelope-endoplasmic reticulum network	40	4.50E-01	9.00E-01
ILMN_1675266	NAGPA	0.676063654		GOTERM_CC_FAT	endoplasmic reticulum membrane	37	5.20E-01	9.20E-01
ILMN_3295847	RPL6	0.675929137	**Annotation Cluster 143**	**Enrichment Score: 0.83**		**Count**	**P_Value**	**Benjamini**
ILMN_1815519	EPN2	0.675922864		GOTERM_BP_FAT	negative regulation of cellular catabolic process	8	1.10E-02	2.10E-01
ILMN_1688534	EIF2B5	0.675901234		GOTERM_BP_FAT	regulation of proteolysis	14	1.80E-02	2.80E-01
ILMN_1696757	TTC14	0.67582851		GOTERM_BP_FAT	regulation of cellular catabolic process	13	9.90E-02	6.80E-01
ILMN_3251728	MTMR10	0.675820703		GOTERM_BP_FAT	positive regulation of proteolysis	7	1.00E-01	6.90E-01
ILMN_3283573	RPL4	0.675768654		GOTERM_BP_FAT	negative regulation of catabolic process	9	1.10E-01	7.00E-01
ILMN_1675992	DST	0.67572379		GOTERM_BP_FAT	positive regulation of protein catabolic process	7	1.40E-01	7.70E-01
ILMN_2183510	MANF	0.675488921		GOTERM_BP_FAT	negative regulation of proteolysis	6	1.50E-01	8.00E-01
ILMN_1682929	SYTL2	0.675266457		GOTERM_BP_FAT	regulation of proteasomal ubiquitin-dependent protein catabolic process	4	1.70E-01	8.20E-01
ILMN_1665390	SPDYE7P	0.675218732		GOTERM_BP_FAT	regulation of membrane protein ectodomain proteolysis	4	2.40E-01	9.00E-01
ILMN_2348503	PPIE	0.674954781		GOTERM_BP_FAT	regulation of protein catabolic process	10	2.40E-01	9.00E-01
ILMN_1715832	PIK3R4	0.674874204		GOTERM_BP_FAT	regulation of catabolic process	16	2.80E-01	9.20E-01
ILMN_1669433	KIAA0913	0.674832191		GOTERM_BP_FAT	positive regulation of membrane protein ectodomain proteolysis	3	3.40E-01	9.50E-01
ILMN_1754746	CCNB3	0.67475101		GOTERM_BP_FAT	negative regulation of protein catabolic process	4	3.90E-01	9.70E-01
ILMN_1812557	CDK5RAP3	0.674746479		GOTERM_BP_FAT	positive regulation of catabolic process	8	4.70E-01	9.80E-01
ILMN_1737964	HIATL1	0.674740263		GOTERM_BP_FAT	positive regulation of cellular catabolic process	6	5.20E-01	9.90E-01
ILMN_2383419	GMEB1	0.674735708	**Annotation Cluster 144**	**Enrichment Score: 0.81**		**Count**	**P_Value**	**Benjamini**
ILMN_1768812	FXYD6	0.674594413		GOTERM_MF_FAT	oxidoreductase activity, acting on paired donors, with incorporation or reduction of molecular oxygen, 2-oxoglutarate as one donor, and incorporation of one atom each of oxygen into both donors	8	5.90E-02	6.50E-01
ILMN_1715305	TRIM6	0.674593136		GOTERM_MF_FAT	procollagen-proline dioxygenase activity	3	2.20E-01	9.30E-01
ILMN_2397721	GLB1	0.674552325		GOTERM_MF_FAT	peptidyl-proline dioxygenase activity	3	2.70E-01	9.60E-01
ILMN_1792135	SLC35A2	0.674514242	**Annotation Cluster 145**	**Enrichment Score: 0.81**		**Count**	**P_Value**	**Benjamini**
ILMN_1666376	TRIM56	0.674371914		GOTERM_BP_FAT	small GTPase mediated signal transduction	56	9.20E-03	1.90E-01
ILMN_1809495	COX8A	0.674359617		GOTERM_MF_FAT	GTPase activity	40	1.20E-02	3.00E-01
ILMN_2257015	AGER	0.674284093		UP_SEQ_FEATURE	nucleotide phosphate-binding region:GTP	48	3.00E-02	9.90E-01
ILMN_1710937	IFI16	0.674261606		UP_SEQ_FEATURE	short sequence motif:Effector region	18	6.30E-02	1.00E+00
ILMN_2124386	RGL2	0.674245626		SP_PIR_KEYWORDS	prenylation	26	8.30E-02	3.90E-01
ILMN_1734742	ARHGDIA	0.674235917		SP_PIR_KEYWORDS	gtp-binding	49	9.20E-02	4.10E-01
ILMN_1727041	EWSR1	0.674058724		UP_SEQ_FEATURE	lipid moiety-binding region:S-geranylgeranyl cysteine	18	1.10E-01	1.00E+00
ILMN_3238221	FLJ42627	0.674022751		GOTERM_MF_FAT	GTP binding	55	1.90E-01	9.00E-01
ILMN_1739103	MPZL1	0.673915791		GOTERM_MF_FAT	guanyl nucleotide binding	55	2.50E-01	9.40E-01
ILMN_1763452	EVI2B	0.673891872		GOTERM_MF_FAT	guanyl ribonucleotide binding	55	2.50E-01	9.40E-01
ILMN_2326071	MYL6	0.673865263		INTERPRO	RNA polymerase sigma factor 54, interaction	3	4.10E-01	1.00E+00
ILMN_1732609	KIAA1539	0.673851422		INTERPRO	Ras GTPase	19	4.40E-01	1.00E+00
ILMN_2062524	RBBP4	0.673761966		INTERPRO	Small GTP-binding protein	23	4.50E-01	1.00E+00
ILMN_1797298	ARMC7	0.673370934		INTERPRO	Ras small GTPase, Rab type	9	5.10E-01	1.00E+00
ILMN_2160388	RPL24	0.673342293		INTERPRO	Ras	17	5.20E-01	1.00E+00
ILMN_1688011	UPF1	0.673232877		SMART	RAB	9	5.60E-01	9.90E-01
ILMN_1785272	COL1A2	0.672931436		PIR_SUPERFAMILY	PIRSF001710:Ras-related protein Rab	3	8.60E-01	1.00E+00
ILMN_1759915	ARPC1A	0.672786001	**Annotation Cluster 146**	**Enrichment Score: 0.81**		**Count**	**P_Value**	**Benjamini**
ILMN_2316806	RWDD1	0.67265973		INTERPRO	MIF4G-like, type 3	4	1.20E-01	9.80E-01
ILMN_1660554	VWA1	0.672628474		INTERPRO	MIF4-like, type 1/2/3	4	1.20E-01	9.80E-01
ILMN_1805466	SOX9	0.672350778		SMART	MIF4G	4	1.40E-01	8.10E-01
ILMN_2058782	IFI27	0.672182059		UP_SEQ_FEATURE	domain:MIF4G	3	3.00E-01	1.00E+00
ILMN_1804384	ASMTL	0.672147564	**Annotation Cluster 147**	**Enrichment Score: 0.77**		**Count**	**P_Value**	**Benjamini**
ILMN_3282436	EIF3F	0.672028851		SP_PIR_KEYWORDS	ascorbic acid	5	2.60E-03	3.40E-02

ILMN_1866314	BX118075	-0.455172406		GOTERM_BP_FAT	gut development	4	7.50E-01	1.00E+00
ILMN_1687073	KIAA0889	-0.455175667	**Annotation Cluster 177**	**Enrichment Score: 0.4**		**Count**	**P_Value**	**Benjamini**
ILMN_3294126	RPL39	-0.455194247		GOTERM_BP_FAT	negative regulation of specific transcription from RNA polymerase II promoter	7	2.60E-01	9.90E-01
ILMN_1690530	FER1L5	-0.455260278		BIOCARTA	Control of Gene Expression by Vitamin D Receptor	5	3.30E-01	9.90E-01
ILMN_3287849	LOC440419	-0.455368959		GOTERM_BP_FAT	regulation of gene-specific transcription	17	3.50E-01	1.00E+00
ILMN_3241154	POM121L1P	-0.455371985		GOTERM_BP_FAT	negative regulation of gene-specific transcription	7	4.00E-01	1.00E+00
ILMN_1713024	PCDHA@	-0.455400301		GOTERM_BP_FAT	regulation of specific transcription from RNA polymerase II promoter	12	4.10E-01	1.00E+00
ILMN_1681728	BM665414	-0.455413589		GOTERM_BP_FAT	positive regulation of specific transcription from RNA polymerase II promoter	7	5.70E-01	1.00E+00
ILMN_1659484	MAGEC3	-0.455419505		GOTERM_BP_FAT	positive regulation of gene-specific transcription	10	5.80E-01	1.00E+00
ILMN_2364819	USP44	-0.455473525	**Annotation Cluster 178**	**Enrichment Score: 0.4**		**Count**	**P_Value**	**Benjamini**
ILMN_1798206	KRTAP11-1	-0.455496613		INTERPRO	Carbonic anhydrase, alpha-class, catalytic domain	4	3.00E-01	1.00E+00
ILMN_2175020	RPL32P3	-0.455515513		PIR_SUPERFAMILY	PIRSF001392:carbonate dehydratase	3	4.50E-01	1.00E+00
ILMN_1724211	GPRC5C	-0.455548942		INTERPRO	Carbonic anhydrase, alpha-class, conserved site	3	4.80E-01	1.00E+00
ILMN_1769358	NPBWR2	-0.455582418	**Annotation Cluster 179**	**Enrichment Score: 0.4**		**Count**	**P_Value**	**Benjamini**
ILMN_2173909	ZNF14	-0.45568314		GOTERM_BP_FAT	regulation of binding	21	1.90E-01	9.90E-01
ILMN_1688633	CLCN2	-0.455761339		GOTERM_BP_FAT	positive regulation of DNA binding	11	2.00E-01	9.90E-01
ILMN_1709346	CLTB	-0.455788656		GOTERM_BP_FAT	regulation of DNA binding	17	2.10E-01	9.90E-01
ILMN_1796772	ARHGAP28	-0.455871772		GOTERM_BP_FAT	positive regulation of binding	11	3.10E-01	9.90E-01
ILMN_1731966	LEPR	-0.455916835		GOTERM_BP_FAT	positive regulation of transcription factor activity	8	4.50E-01	1.00E+00
ILMN_1765132	LACTB	-0.455946565		GOTERM_BP_FAT	regulation of transcription factor activity	12	5.40E-01	1.00E+00
ILMN_3264466	FAM54B	-0.456016739		GOTERM_BP_FAT	negative regulation of binding	7	6.10E-01	1.00E+00
ILMN_1900782	BE466650	-0.45614319		GOTERM_BP_FAT	negative regulation of DNA binding	6	6.40E-01	1.00E+00
ILMN_1853881	BG118017	-0.456283849		GOTERM_BP_FAT	positive regulation of NF-kappaB transcription factor activity	5	6.40E-01	1.00E+00
ILMN_1890421	DB311601	-0.456297432		GOTERM_BP_FAT	negative regulation of transcription factor activity	5	7.20E-01	1.00E+00
ILMN_1783847	FCHSD1	-0.456329456	**Annotation Cluster 180**	**Enrichment Score: 0.39**		**Count**	**P_Value**	**Benjamini**
ILMN_1768049	FOXP3	-0.45639319		SP_PIR_KEYWORDS	GTP binding	10	2.60E-01	9.00E-01
ILMN_1789096	OSTalpha	-0.456407575		SP_PIR_KEYWORDS	P-loop	14	4.40E-01	9.50E-01
ILMN_1781031	CTBP2	-0.456452811		SP_PIR_KEYWORDS	nucleotide binding	12	5.70E-01	9.70E-01
ILMN_2166706	FBXO22OS	-0.456483928	**Annotation Cluster 181**	**Enrichment Score: 0.39**		**Count**	**P_Value**	**Benjamini**
ILMN_1662865	CPPED1	-0.456486631		GOTERM_BP_FAT	DNA damage response, signal transduction by p53 class mediator resulting in cell cycle arrest	3	1.30E-01	9.80E-01
ILMN_1794528	KIAA1751	-0.456533986		GOTERM_BP_FAT	G1/S transition checkpoint	4	2.70E-01	9.90E-01
ILMN_1805449	TAPBPL	-0.456631368		GOTERM_BP_FAT	G1 DNA damage checkpoint	3	3.30E-01	9.90E-01
ILMN_1662880	FIS	-0.456661693		GOTERM_BP_FAT	DNA damage checkpoint	7	4.00E-01	1.00E+00
ILMN_1803454	PCGF3	-0.456663669		GOTERM_BP_FAT	DNA integrity checkpoint	7	4.80E-01	1.00E+00
ILMN_1897911	BF512455	-0.456745054		GOTERM_BP_FAT	mitotic cell cycle checkpoint	6	4.80E-01	1.00E+00
ILMN_1701497	CD5L	-0.456758987		GOTERM_BP_FAT	cell cycle checkpoint	11	5.00E-01	1.00E+00
ILMN_1693092	SYNPO2	-0.456777744		GOTERM_BP_FAT	DNA damage response, signal transduction	8	7.60E-01	1.00E+00
ILMN_1765355	NOX1	-0.456786834		GOTERM_BP_FAT	DNA damage response, signal transduction by p53 class mediator	3	7.80E-01	1.00E+00
ILMN_1846922	BC042566	-0.456799136	**Annotation Cluster 182**	**Enrichment Score: 0.39**		**Count**	**P_Value**	**Benjamini**
ILMN_2059606	CCDC117	-0.456800237		SMART	BBOX	12	9.70E-02	9.70E-01
ILMN_2097052	CDY2B	-0.456858401		INTERPRO	Zinc finger, B-box	12	1.40E-01	1.00E+00
ILMN_1818538	DA855529	-0.456915689		UP_SEQ_FEATURE	zinc finger region:B box-type 2	4	2.30E-01	1.00E+00
ILMN_1693487	OR2W1	-0.457018656		UP_SEQ_FEATURE	zinc finger region:B box-type	8	3.60E-01	1.00E+00
ILMN_1687875	UNC5C	-0.457030781		UP_SEQ_FEATURE	zinc finger region:B box-type 1	3	3.60E-01	1.00E+00
ILMN_1825242	AA918474	-0.457033634		INTERPRO	B302 (SPRY)-like	11	4.40E-01	1.00E+00
ILMN_1708393	C1orf185	-0.457046349		INTERPRO	Butyrophylin-like	8	4.90E-01	1.00E+00
ILMN_1699728	BTD	-0.457056385		UP_SEQ_FEATURE	domain:B30.2/SPRY	10	5.00E-01	1.00E+00
ILMN_1814769	UGT2B15	-0.457059252		INTERPRO	SPla/RYanodine receptor SPRY	10	5.40E-01	1.00E+00
ILMN_1697289	ZNF713	-0.457227176		SMART	SPRY	6	6.50E-01	9.90E-01
ILMN_1683664	FAM60A	-0.457236303		INTERPRO	SPla/RYanodine receptor subgroup	6	7.10E-01	1.00E+00
ILMN_1672325	PYDC2	-0.457251594		SMART	PRY	4	8.70E-01	1.00E+00
ILMN_1777156	GTPBP3	-0.457270138		INTERPRO	SPRY-associated	4	9.00E-01	1.00E+00
ILMN_1708989	TBX1	-0.45730284	**Annotation Cluster 183**	**Enrichment Score: 0.39**		**Count**	**P_Value**	**Benjamini**
ILMN_1876039	AA812958	-0.457401986		GOTERM_MF_FAT	protein serine/threonine/tyrosine kinase activity	4	2.10E-01	9.80E-01
ILMN_1827966	AK023961	-0.457439321		GOTERM_MF_FAT	MAP kinase kinase activity	3	4.10E-01	1.00E+00
ILMN_2103720	MRPL15	-0.457675346		BIOCARTA	MAPKinase Signaling Pathway	8	7.80E-01	1.00E+00
ILMN_1683097	RPS6KA5	-0.457741257	**Annotation Cluster 184**	**Enrichment Score: 0.39**		**Count**	**P_Value**	**Benjamini**
ILMN_1860963	BM715829	-0.457754487		SMART	FHA	5	3.70E-01	9.80E-01
ILMN_1693771	ASPH	-0.457794593		INTERPRO	Forkhead-associated	5	4.20E-01	1.00E+00
ILMN_1695991	COLQ	-0.457803983		UP_SEQ_FEATURE	domain:FHA	5	4.40E-01	1.00E+00
ILMN_3235221	LOC644936	-0.457820283	**Annotation Cluster 185**	**Enrichment Score: 0.39**		**Count**	**P_Value**	**Benjamini**
ILMN_1770692	WDR12	-0.457858169		GOTERM_BP_FAT	positive regulation of cholesterol biosynthetic process	3	5.80E-02	9.80E-01
ILMN_1806492	TNR	-0.457954323		GOTERM_BP_FAT	positive regulation of steroid biosynthetic process	3	1.60E-01	9.80E-01
ILMN_2156687	DFNB59	-0.457999732		GOTERM_BP_FAT	regulation of cholesterol biosynthetic process	3	2.50E-01	9.90E-01
ILMN_1695058	SLC38A5	-0.458073392		GOTERM_BP_FAT	regulation of steroid biosynthetic process	5	2.70E-01	9.90E-01
ILMN_1818935	BQ575873	-0.458134297		GOTERM_BP_FAT	positive regulation of lipid biosynthetic process	4	3.90E-01	1.00E+00
ILMN_1765019	SACM1L	-0.458283138		GOTERM_BP_FAT	positive regulation of steroid metabolic process	3	4.50E-01	1.00E+00
ILMN_1728975	SCO1	-0.458447002		GOTERM_BP_FAT	regulation of lipid biosynthetic process	6	5.30E-01	1.00E+00
ILMN_1655521	DMRTA1	-0.4584615		GOTERM_BP_FAT	regulation of steroid metabolic process	5	5.40E-01	1.00E+00
ILMN_1694078	AY358310	-0.458474593		GOTERM_BP_FAT	positive regulation of lipid metabolic process	6	5.50E-01	1.00E+00
ILMN_1693878	GMPPA	-0.45851713		GOTERM_BP_FAT	regulation of fatty acid oxidation	3	8.10E-01	1.00E+00
ILMN_1704497	KRT25	-0.458606697		GOTERM_BP_FAT	regulation of lipid metabolic process	10	8.50E-01	1.00E+00
ILMN_2206420	UGT1A1	-0.458608053		GOTERM_BP_FAT	regulation of cellular ketone metabolic process	5	8.70E-01	1.00E+00
ILMN_1800612	VBP1	-0.458637544		GOTERM_BP_FAT	regulation of fatty acid metabolic process	4	9.00E-01	1.00E+00
ILMN_1843425	AF520419	-0.45864635	**Annotation Cluster 186**	**Enrichment Score: 0.39**		**Count**	**P_Value**	**Benjamini**
ILMN_1737813	ERI3	-0.458723363		GOTERM_MF_FAT	retinol dehydrogenase activity	3	2.90E-01	9.90E-01
ILMN_1900457	NA	-0.458852105		INTERPRO	NAD(P)-binding domain	19	3.20E-01	1.00E+00
ILMN_2162328	PTS	-0.458860781		PIR_SUPERFAMILY	PIRSF000092:short-chain dehydrogenase	3	4.20E-01	1.00E+00
ILMN_2103003	OR4N3P	-0.458872091		INTERPRO	Glucose/ribitol dehydrogenase	7	5.00E-01	1.00E+00
ILMN_1673716	KIF6	-0.458876574		INTERPRO	Short-chain dehydrogenase/reductase SDR	7	6.10E-01	1.00E+00
ILMN_1763092	MCCD1	-0.458893651	**Annotation Cluster 187**	**Enrichment Score: 0.38**		**Count**	**P_Value**	**Benjamini**
ILMN_1756721	FSCB	-0.458918457		UP_SEQ_FEATURE	domain:MAGE	6	2.40E-01	1.00E+00
ILMN_1688935	NA	-0.458979036		INTERPRO	MAGE protein	6	3.10E-01	1.00E+00
ILMN_1756102	RFX3	-0.458982059		SP_PIR_KEYWORDS	tumor antigen	5	5.70E-01	9.70E-01
ILMN_1680673	NT5DC1	-0.459061119		PIR_SUPERFAMILY	PIRSF005491:tumor associated protein MAGE	3	6.80E-01	1.00E+00
ILMN_1786658	BOLA3	-0.459127312	**Annotation Cluster 188**	**Enrichment Score: 0.38**		**Count**	**P_Value**	**Benjamini**
ILMN_1903568	CR625988	-0.459134649		KEGG_PATHWAY	Steroid biosynthesis	5	1.10E-01	9.20E-01

						Count	P_Value	Benjamini
ILMN_2382906	PLCG1	0.671705455		GOTERM_MF_FAT	oxidoreductase activity, acting on paired donors, with incorporation or reduction of molecular oxygen, 2-oxoglutarate as one donor, and incorporation of one atom each of oxygen into both donors	8	5.90E-02	6.50E-01
ILMN_1798189	COX7C	0.671666584		SP_PIR_KEYWORDS	vitamin c	6	6.90E-02	3.60E-01
ILMN_1674640	CXCR6	0.671613346		UP_SEQ_FEATURE	domain:Fe2OG dioxygenase	6	1.00E-01	1.00E+00
ILMN_1686748	TMEM9	0.671499677		INTERPRO	Prolyl 4-hydroxylase, alpha subunit	5	1.10E-01	9.70E-01
ILMN_1705301	TEAD4	0.671491247		GOTERM_MF_FAT	L-ascorbic acid binding	6	1.20E-01	8.30E-01
ILMN_1770206	GEMIN4	0.671455025		SMART	P4Hc	5	1.30E-01	8.00E-01
ILMN_1713829	PTGES	0.671358329		INTERPRO	Oxoglutarate and iron-dependent oxygenase	5	1.60E-01	9.90E-01
ILMN_3267760	RPL10A	0.671099039		SP_PIR_KEYWORDS	dioxygenase	12	1.70E-01	5.80E-01
ILMN_2325394	MSH5	0.671069998		UP_SEQ_FEATURE	metal ion-binding site:Iron	9	1.90E-01	1.00E+00
ILMN_1738759	PIGT	0.670970006		GOTERM_MF_FAT	oxidoreductase activity, acting on single donors with incorporation of molecular oxygen, incorporation of two atoms of oxygen	12	2.20E-01	9.30E-01
ILMN_1805693	GMIP	0.670826438		GOTERM_MF_FAT	oxidoreductase activity, acting on single donors with incorporation of molecular oxygen	12	2.40E-01	9.40E-01
ILMN_3234142	LOC728855	0.670733624		GOTERM_MF_FAT	vitamin binding	13	9.20E-01	1.00E+00
ILMN_2352448	NSUN5P1	0.670725248		GOTERM_MF_FAT	carboxylic acid binding	14	9.40E-01	1.00E+00
ILMN_1806705	ASB6	0.67072202		SP_PIR_KEYWORDS	iron	24	9.90E-01	1.00E+00
ILMN_1657836	PLEKHG2	0.670628443		GOTERM_MF_FAT	iron ion binding	26	1.00E+00	1.00E+00
ILMN_2214232	AIM2	0.670529878	**Annotation Cluster 148**	**Enrichment Score: 0.77**		**Count**	**P_Value**	**Benjamini**
ILMN_2383435	PSMD10	0.670509576		SP_PIR_KEYWORDS	cell binding	7	5.80E-03	6.30E-02
ILMN_1694587	EEF1B2	0.670409492		SP_PIR_KEYWORDS	collagen	18	6.10E-02	3.30E-01
ILMN_1669362	IGFBP6	0.670382976		SP_PIR_KEYWORDS	hydroxyproline	9	7.00E-02	3.60E-01
ILMN_2059689	TMEM54	0.670365533		SP_PIR_KEYWORDS	triple helix	8	7.20E-02	3.60E-01
ILMN_1693630	C16orf7	0.67028484		SP_PIR_KEYWORDS	hydroxylysine	8	7.20E-02	3.60E-01
ILMN_1652412	PHKB	0.670079773		SP_PIR_KEYWORDS	hydroxylation	13	1.40E-01	5.30E-01
ILMN_1814966	PIAS3	0.670079012		GOTERM_CC_FAT	collagen	8	1.80E-01	6.40E-01
ILMN_2220283	HNRNPA1	0.670030498		INTERPRO	Collagen triple helix repeat	14	2.60E-01	1.00E+00
ILMN_1726025	ASXL1	0.670025962		UP_SEQ_FEATURE	glycosylation site:O-linked (Gal...)	3	3.00E-01	1.00E+00
ILMN_1694398	EIF3F	0.66986		UP_SEQ_FEATURE	region of interest:Triple-helical region	5	3.00E-01	1.00E+00
ILMN_1657509	TSEN54	0.669749613		UP_SEQ_FEATURE	domain:VWFA 2	4	3.40E-01	1.00E+00
ILMN_1707070	PCOLCE	0.669425304		GOTERM_CC_FAT	anchoring collagen	3	3.40E-01	8.20E-01
ILMN_1783728	TBRG4	0.669351647		UP_SEQ_FEATURE	domain:VWFA 1	3	5.90E-01	1.00E+00
ILMN_1677122	FAM168B	0.669104591		SP_PIR_KEYWORDS	trimer	4	6.20E-01	9.40E-01
ILMN_2251184	ERP29	0.66903646		SP_PIR_KEYWORDS	pyroglutamic acid	5	8.20E-01	9.90E-01
ILMN_1756573	NDUFA4L2	0.66902643	**Annotation Cluster 149**	**Enrichment Score: 0.77**		**Count**	**P_Value**	**Benjamini**
ILMN_1670878	YTHDC1	0.66900312		UP_SEQ_FEATURE	DNA-binding region:ETS	9	1.20E-02	9.10E-01
ILMN_2413064	ST6GALNAC4	0.668948591		UP_SEQ_FEATURE	domain:PNT	3	3.90E-01	1.00E+00
ILMN_2316104	IQCB1	0.668866984		INTERPRO	Sterile alpha motif/pointed	3	4.10E-01	1.00E+00
ILMN_3234831	RPL17	0.668859052		SMART	SAM_PNT	3	4.40E-01	9.70E-01
ILMN_1673711	HSP90AB1	0.668857289	**Annotation Cluster 150**	**Enrichment Score: 0.76**		**Count**	**P_Value**	**Benjamini**
ILMN_1807600	NPLOC4	0.66874258		GOTERM_BP_FAT	regulation of macroautophagy	3	8.70E-02	6.40E-01
ILMN_1711272	SERPING1	0.668734692		GOTERM_BP_FAT	regulation of autophagy	3	1.80E-01	8.40E-01
ILMN_1806908	PRKCB	0.668697411		GOTERM_BP_FAT	regulation of response to nutrient levels	4	2.40E-01	9.00E-01
ILMN_3268028	BMS1	0.668675846		GOTERM_BP_FAT	regulation of response to extracellular stimulus	4	2.40E-01	9.00E-01
ILMN_1758523	ABCA3	0.668606288	**Annotation Cluster 151**	**Enrichment Score: 0.76**		**Count**	**P_Value**	**Benjamini**
ILMN_1785528	DKFZp434K1323	0.668567798		GOTERM_BP_FAT	protein retention in ER lumen	4	5.30E-02	5.10E-01
ILMN_1787518	GSN	0.668482276		GOTERM_BP_FAT	maintenance of location	13	1.30E-01	7.60E-01
ILMN_1682332	GYPC	0.668413977		GOTERM_BP_FAT	maintenance of protein location	10	1.70E-01	8.30E-01
ILMN_1699636	ACIN1	0.668329109		GOTERM_BP_FAT	maintenance of location in cell	9	3.00E-01	9.30E-01
ILMN_2286514	GTPBP8	0.668280969		GOTERM_BP_FAT	maintenance of protein location in cell	7	4.60E-01	9.80E-01
ILMN_2041222	FLJ40504	0.66815541	**Annotation Cluster 152**	**Enrichment Score: 0.76**		**Count**	**P_Value**	**Benjamini**
ILMN_2368318	FGR	0.668110343		KEGG_PATHWAY	Fc gamma R-mediated phagocytosis	21	6.90E-02	3.50E-01
ILMN_1802376	FAM48A	0.668084297		KEGG_PATHWAY	Natural killer cell mediated cytotoxicity	26	1.30E-01	4.80E-01
ILMN_1725705	CLPP	0.667888001		KEGG_PATHWAY	B cell receptor signaling pathway	16	1.50E-01	5.00E-01
ILMN_2298958	MRRF	0.667819691		KEGG_PATHWAY	Non-small cell lung cancer	12	1.80E-01	5.50E-01
ILMN_1684647	ILKAP	0.667750134		KEGG_PATHWAY	T cell receptor signaling pathway	21	1.80E-01	5.30E-01
ILMN_1717524	LIPT1	0.667663108		KEGG_PATHWAY	Glioma	13	2.30E-01	5.80E-01
ILMN_1673708	HDAC9	0.667521467		KEGG_PATHWAY	Fc epsilon RI signaling pathway	15	2.70E-01	6.30E-01
ILMN_2379326	MAP3K7	0.667427586		KEGG_PATHWAY	VEGF signaling pathway	14	3.30E-01	6.80E-01
ILMN_3228639	NA	0.66726768	**Annotation Cluster 153**	**Enrichment Score: 0.76**		**Count**	**P_Value**	**Benjamini**
ILMN_1845157	TTC15	0.667206659		GOTERM_BP_FAT	mRNA catabolic process	10	1.10E-01	7.00E-01
ILMN_2390974	DNAJB2	0.66700416		SP_PIR_KEYWORDS	nonsense-mediated mrna decay	7	1.10E-01	4.70E-01
ILMN_2118773	ASAH2B	0.666966892		GOTERM_BP_FAT	nuclear-transcribed mRNA catabolic process, nonsense-mediated decay	7	2.00E-01	8.60E-01
ILMN_1667925	PDCL3	0.6669445		GOTERM_BP_FAT	RNA catabolic process	12	2.30E-01	8.90E-01
ILMN_3248122	MED1	0.666930575		GOTERM_BP_FAT	nuclear-transcribed mRNA catabolic process	7	3.10E-01	9.40E-01
ILMN_1705522	SPAG1	0.666923975	**Annotation Cluster 154**	**Enrichment Score: 0.76**		**Count**	**P_Value**	**Benjamini**
ILMN_1802646	EPHB6	0.666875852		GOTERM_BP_FAT	regulation of T cell tolerance induction	3	1.30E-01	7.60E-01
ILMN_1698307	DBNL	0.666543185		GOTERM_BP_FAT	positive regulation of T cell tolerance induction	3	1.30E-01	7.60E-01
ILMN_2317751	REC8	0.666487316		GOTERM_BP_FAT	positive regulation of tolerance induction	3	2.30E-01	8.90E-01
ILMN_2309245	BIN1	0.666460964		GOTERM_BP_FAT	regulation of tolerance induction	3	2.30E-01	8.90E-01
ILMN_1703565	GLTSCR2	0.666450318	**Annotation Cluster 155**	**Enrichment Score: 0.76**		**Count**	**P_Value**	**Benjamini**
ILMN_1779257	CD40	0.666326866		GOTERM_BP_FAT	nucleus organization	13	3.50E-02	4.10E-01
ILMN_1773389	PLTP	0.666260043		GOTERM_BP_FAT	DNA catabolic process, endonucleolytic	8	4.70E-02	4.80E-01
ILMN_1679405	DDX56	0.666245243		GOTERM_BP_FAT	apoptotic nuclear changes	7	1.20E-01	7.30E-01
ILMN_1790460	ZNF574	0.666229046		GOTERM_BP_FAT	DNA catabolic process	12	1.60E-01	8.10E-01
ILMN_1715500	SS18L1	0.666144362		GOTERM_BP_FAT	cell structure disassembly during apoptosis	6	1.80E-01	8.30E-01
ILMN_1822775	DB336446	0.665810107		GOTERM_BP_FAT	DNA fragmentation involved in apoptosis	5	2.40E-01	8.90E-01
ILMN_1661337	SRM	0.665496289		GOTERM_BP_FAT	cellular component disassembly	9	4.00E-01	9.70E-01
ILMN_2366719	NSFL1C	0.66540831		BIOCARTA	Caspase Cascade in Apoptosis	6	5.10E-01	9.80E-01
ILMN_1676721	NF2	0.665246719		BIOCARTA	Apoptotic DNA fragmentation and tissue homeostasis	3	6.00E-01	9.90E-01
ILMN_1682957	PACSIN3	0.665223496	**Annotation Cluster 156**	**Enrichment Score: 0.75**		**Count**	**P_Value**	**Benjamini**
ILMN_1793990	ID2	0.66515368		GOTERM_CC_FAT	Golgi trans cisterna	3	8.90E-02	4.70E-01
ILMN_2219351	CENPO	0.664897585		GOTERM_CC_FAT	Golgi stack	10	1.50E-01	6.00E-01
ILMN_1728355	PSMD4	0.664887716		GOTERM_CC_FAT	organelle subcompartment	6	2.10E-01	6.80E-01
ILMN_1727300	ZNF444	0.664871563		GOTERM_CC_FAT	Golgi cisterna	5	3.40E-01	8.20E-01
ILMN_3279935	RPL17	0.664831025	**Annotation Cluster 157**	**Enrichment Score: 0.75**		**Count**	**P_Value**	**Benjamini**
ILMN_1683781	ADAMTS15	0.664823528		GOTERM_BP_FAT	ribonucleoside metabolic process	11	7.30E-02	6.00E-01
ILMN_2367020	SEC61G	0.664759946		GOTERM_BP_FAT	purine nucleoside metabolic process	8	7.80E-02	6.10E-01
ILMN_1741459	CDK10	0.664736578		GOTERM_BP_FAT	purine ribonucleoside metabolic process	8	7.80E-02	6.10E-01
ILMN_1703430	CCDC94	0.664696712		GOTERM_BP_FAT	nucleoside biosynthetic process	3	2.90E-01	9.30E-01
ILMN_3241870	FRMD8	0.664693711		GOTERM_BP_FAT	purine nucleoside biosynthetic process	3	2.90E-01	9.30E-01
ILMN_1765876	ARHGAP24	0.664683073		GOTERM_BP_FAT	ribonucleoside biosynthetic process	3	2.90E-01	9.30E-01
ILMN_3226807	NHP2	0.664459917		GOTERM_BP_FAT	purine ribonucleoside biosynthetic process	3	2.90E-01	9.30E-01
ILMN_2356578	TH	0.664391026		GOTERM_BP_FAT	nucleoside metabolic process	11	3.30E-01	9.50E-01
ILMN_2339028	PKD1	0.664353106	**Annotation Cluster 158**	**Enrichment Score: 0.75**		**Count**	**P_Value**	**Benjamini**

						Count	P_Value	Benjamini
ILMN_3233385	TP53TG3b	-0.459150921		GOTERM_BP_FAT	steroid metabolic process	26	2.30E-01	9.90E-01
ILMN_1865290	BU618600	-0.459196581		SP_PIR_KEYWORDS	sterol biosynthesis	5	2.60E-01	9.00E-01
ILMN_1781517	LIPF	-0.459247504		GOTERM_BP_FAT	steroid biosynthetic process	12	2.90E-01	9.90E-01
ILMN_1769151	AK123300	-0.459405242		SP_PIR_KEYWORDS	Steroid biosynthesis	6	4.10E-01	9.40E-01
ILMN_1755504	CALCOCO2	-0.459428284		GOTERM_BP_FAT	sterol biosynthetic process	5	5.20E-01	1.00E+00
ILMN_1693192	PI3	-0.459617215		SP_PIR_KEYWORDS	Cholesterol biosynthesis	3	6.10E-01	9.70E-01
ILMN_1788239	AMDHD1	-0.459628056		GOTERM_BP_FAT	sterol metabolic process	11	6.40E-01	1.00E+00
ILMN_2105549	TMEM220	-0.45962916		SP_PIR_KEYWORDS	lipid synthesis	10	7.10E-01	9.80E-01
ILMN_1904301	MECP2	-0.459632711		GOTERM_BP_FAT	cholesterol metabolic process	9	7.70E-01	1.00E+00
ILMN_1766377	PCDHB6	-0.459747086		GOTERM_BP_FAT	cholesterol biosynthetic process	3	7.80E-01	1.00E+00
ILMN_1672324	EBLN1	-0.459751472	**Annotation Cluster 189**	**Enrichment Score: 0.37**		**Count**	**P_Value**	**Benjamini**
ILMN_2388669	GRIA3	-0.459776539		GOTERM_BP_FAT	ectoderm development	26	2.10E-01	9.90E-01
ILMN_1658259	DRG1	-0.459786374		GOTERM_BP_FAT	epidermal cell differentiation	11	2.30E-01	9.90E-01
ILMN_1722620	MGC39584	-0.460016872		GOTERM_BP_FAT	keratinocyte differentiation	10	2.60E-01	9.90E-01
ILMN_1915480	BX107598	-0.460041031		GOTERM_BP_FAT	epidermis development	21	4.90E-01	1.00E+00
ILMN_2154101	UPRT	-0.460086166		SP_PIR_KEYWORDS	keratinization	5	6.10E-01	9.70E-01
ILMN_1699447	TRAF5	-0.460093358		GOTERM_BP_FAT	keratinization	5	6.80E-01	1.00E+00
ILMN_1774945	NRG3	-0.460106803		GOTERM_BP_FAT	epithelial cell differentiation	11	9.30E-01	1.00E+00
ILMN_2206592	PRAMEF11	-0.46013135	**Annotation Cluster 190**	**Enrichment Score: 0.37**		**Count**	**P_Value**	**Benjamini**
ILMN_1707887	LOC644172	-0.460183384		UP_SEQ_FEATURE	repeat:ANK 9	7	1.90E-01	1.00E+00
ILMN_1746194	DUSP21	-0.460189579		UP_SEQ_FEATURE	repeat:ANK 7	9	2.60E-01	1.00E+00
ILMN_2246105	C3orf23	-0.460242904		UP_SEQ_FEATURE	repeat:ANK 8	7	2.80E-01	1.00E+00
ILMN_3280594	LOC120824	-0.460256891		UP_SEQ_FEATURE	repeat:ANK 13	3	4.00E-01	1.00E+00
ILMN_1659426	CCDC36	-0.460257243		UP_SEQ_FEATURE	repeat:ANK 15	3	4.00E-01	1.00E+00
ILMN_1726602	PPEF2	-0.460281973		UP_SEQ_FEATURE	repeat:ANK 14	3	4.00E-01	1.00E+00
ILMN_3235851	SCARNA9L	-0.460287208		UP_SEQ_FEATURE	repeat:ANK 11	4	4.40E-01	1.00E+00
ILMN_1747573	C3orf65	-0.460305301		UP_SEQ_FEATURE	repeat:ANK 12	3	5.10E-01	1.00E+00
ILMN_1688569	KCNQ1	-0.460364151		UP_SEQ_FEATURE	repeat:ANK 10	4	5.20E-01	1.00E+00
ILMN_3310760	MIR519A2	-0.46042833		UP_SEQ_FEATURE	repeat:ANK 6	9	7.60E-01	1.00E+00
ILMN_1748802	OR2B3	-0.460516029		UP_SEQ_FEATURE	repeat:ANK 5	10	9.40E-01	1.00E+00
ILMN_1768894	OR8G1	-0.46053136	**Annotation Cluster 191**	**Enrichment Score: 0.37**		**Count**	**P_Value**	**Benjamini**
ILMN_1801869	WDR75	-0.460609346		INTERPRO	Four-helical cytokine, core	10	5.00E-02	1.00E+00
ILMN_1805404	GRIN1	-0.460622487		KEGG_PATHWAY	Jak-STAT signaling pathway	21	2.80E-01	9.50E-01
ILMN_1880223	AL833666	-0.460720251		KEGG_PATHWAY	Autoimmune thyroid disease	6	6.90E-01	9.60E-01
ILMN_1688627	AGBL2	-0.460738451		KEGG_PATHWAY	Cytokine-cytokine receptor interaction	26	8.30E-01	9.70E-01
ILMN_1775053	LOC644838	-0.460783545		GOTERM_MF_FAT	cytokine activity	18	8.50E-01	1.00E+00
ILMN_1789543	FBF1	-0.460834808		SP_PIR_KEYWORDS	cytokine	16	8.60E-01	9.90E-01
ILMN_1700771	PRUNE2	-0.460985719	**Annotation Cluster 192**	**Enrichment Score: 0.37**		**Count**	**P_Value**	**Benjamini**
ILMN_1787434	OR10H1	-0.460994076		GOTERM_BP_FAT	regulation of neuron projection development	11	2.00E-01	9.90E-01
ILMN_1704557	RPS6KB1	-0.460994943		GOTERM_BP_FAT	regulation of cell morphogenesis	18	2.20E-01	9.90E-01
ILMN_3234879	OTOA	-0.461058203		GOTERM_BP_FAT	regulation of cell morphogenesis involved in differentiation	11	3.00E-01	9.90E-01
ILMN_2357062	IL1RAP	-0.461084253		GOTERM_BP_FAT	regulation of neuron differentiation	17	3.30E-01	9.90E-01
ILMN_1660590	NA	-0.461108912		GOTERM_BP_FAT	regulation of cell projection organization	12	3.40E-01	1.00E+00
ILMN_1861767	BX118413	-0.461164416		GOTERM_BP_FAT	negative regulation of cell projection organization	5	3.40E-01	1.00E+00
ILMN_1698171	INCENP	-0.461225319		GOTERM_BP_FAT	negative regulation of cell development	7	4.00E-01	1.00E+00
ILMN_1781906	RBM17	-0.461261673		GOTERM_BP_FAT	regulation of axonogenesis	8	4.00E-01	1.00E+00
ILMN_3309644	MIR139	-0.461287599		GOTERM_BP_FAT	negative regulation of axonogenesis	4	5.00E-01	1.00E+00
ILMN_1810335	DGKB	-0.461299486		GOTERM_BP_FAT	negative regulation of neurogenesis	6	5.30E-01	1.00E+00
ILMN_1675671	ICOSLG	-0.461312451		GOTERM_BP_FAT	regulation of neurogenesis	18	6.00E-01	1.00E+00
ILMN_1792107	ERCC8	-0.461406617		GOTERM_BP_FAT	regulation of nervous system development	20	6.60E-01	1.00E+00
ILMN_2413141	C6orf162	-0.461444343		GOTERM_BP_FAT	regulation of cell development	20	7.70E-01	1.00E+00
ILMN_1687316	NCDN	-0.46144502		GOTERM_BP_FAT	negative regulation of cellular component organization	11	9.40E-01	1.00E+00
ILMN_3243961	ZNF252	-0.461524156	**Annotation Cluster 193**	**Enrichment Score: 0.37**		**Count**	**P_Value**	**Benjamini**
ILMN_3307752	DLEU7	-0.461554441		UP_SEQ_FEATURE	domain:PLAT	4	3.80E-01	1.00E+00
ILMN_2351930	TRIM33	-0.46159732		SMART	LH2	4	4.30E-01	9.80E-01
ILMN_1878928	BC031939	-0.461628446		INTERPRO	Lipoxygenase, LH2	4	4.80E-01	1.00E+00
ILMN_1694634	MOGAT3	-0.461645812	**Annotation Cluster 194**	**Enrichment Score: 0.37**		**Count**	**P_Value**	**Benjamini**
ILMN_3191893	NA	-0.461676571		GOTERM_BP_FAT	DNA geometric change	4	3.00E-01	9.90E-01
ILMN_1701661	CYP7B1	-0.461702175		GOTERM_BP_FAT	DNA duplex unwinding	4	3.00E-01	9.90E-01
ILMN_1725556	TMEM229A	-0.461713508		GOTERM_BP_FAT	DNA unwinding during replication	3	4.50E-01	1.00E+00
ILMN_1825421	BC033844	-0.461734635		GOTERM_BP_FAT	DNA-dependent DNA replication	5	8.80E-01	1.00E+00
ILMN_1813117	ITGA9	-0.461791634	**Annotation Cluster 195**	**Enrichment Score: 0.36**		**Count**	**P_Value**	**Benjamini**
ILMN_1693259	PDCD6IP	-0.46182869		UP_SEQ_FEATURE	domain:TTL	3	4.00E-01	1.00E+00
ILMN_1884357	AI734261	-0.46186157		INTERPRO	Tubulin-tyrosine ligase	3	4.50E-01	1.00E+00
ILMN_1797408	NKAPL	-0.46188383		GOTERM_MF_FAT	tubulin-tyrosine ligase activity	3	4.50E-01	1.00E+00
ILMN_1701127	CC2D1A	-0.462011904	**Annotation Cluster 196**	**Enrichment Score: 0.36**		**Count**	**P_Value**	**Benjamini**
ILMN_1890902	BF056964	-0.462012129		GOTERM_BP_FAT	nucleoside monophosphate metabolic process	11	1.60E-01	9.80E-01
ILMN_1812659	ZNF705G	-0.462048694		GOTERM_BP_FAT	ribonucleoside monophosphate metabolic process	4	5.00E-01	1.00E+00
ILMN_1724977	MDK	-0.462097864		GOTERM_BP_FAT	nucleoside monophosphate biosynthetic process	6	6.20E-01	1.00E+00
ILMN_1815345	EIF3J	-0.462207959		GOTERM_BP_FAT	ribonucleoside monophosphate biosynthetic process	3	7.20E-01	1.00E+00
ILMN_1807093	CR608006	-0.462239629	**Annotation Cluster 197**	**Enrichment Score: 0.36**		**Count**	**P_Value**	**Benjamini**
ILMN_3236316	LOC729121	-0.462288516		GOTERM_BP_FAT	positive regulation of osteoblast differentiation	6	1.20E-01	9.80E-01
ILMN_1741096	FDFT1	-0.462307934		GOTERM_BP_FAT	regulation of osteoblast differentiation	7	3.00E-01	9.90E-01
ILMN_1763202	LOC441179	-0.462376636		GOTERM_BP_FAT	regulation of bone mineralization	5	4.00E-01	1.00E+00
ILMN_2122076	GPR149	-0.462392199		GOTERM_BP_FAT	regulation of biomineral formation	5	4.40E-01	1.00E+00
ILMN_1707741	TBC1D26	-0.462413744		GOTERM_BP_FAT	regulation of ossification	10	4.50E-01	1.00E+00
ILMN_1685521	KLRF1	-0.462441853		GOTERM_BP_FAT	positive regulation of bone mineralization	3	5.80E-01	1.00E+00
ILMN_1734707	CHST13	-0.462446699		GOTERM_BP_FAT	positive regulation of biomineral formation	3	6.10E-01	1.00E+00
ILMN_1907966	BI464833	-0.462450978		BIOCARTA	ALK in cardiac myocytes	4	7.30E-01	1.00E+00
ILMN_3242226	LOC399939	-0.462453068		GOTERM_BP_FAT	positive regulation of ossification	3	7.40E-01	1.00E+00
ILMN_1708110	TMEM144	-0.462466438	**Annotation Cluster 198**	**Enrichment Score: 0.36**		**Count**	**P_Value**	**Benjamini**
ILMN_2394552	WNK3	-0.462536854		SP_PIR_KEYWORDS	hormone receptor	5	2.40E-01	9.00E-01
ILMN_1741662	TMC5	-0.462547316		GOTERM_MF_FAT	peptide hormone binding	4	5.60E-01	1.00E+00
ILMN_2244484	CCBL2	-0.462603511		GOTERM_MF_FAT	hormone binding	6	6.10E-01	1.00E+00
ILMN_1726421	METTL9	-0.462630235	**Annotation Cluster 199**	**Enrichment Score: 0.35**		**Count**	**P_Value**	**Benjamini**
ILMN_2364574	MAPK8	-0.462653224		GOTERM_CC_FAT	microbody	15	2.10E-01	9.50E-01
ILMN_1750051	FAM123B	-0.462771748		GOTERM_CC_FAT	peroxisome	15	2.10E-01	9.50E-01
ILMN_1854765	AK130706	-0.462789103		SP_PIR_KEYWORDS	peroxisome	12	3.40E-01	9.30E-01
ILMN_1839665	BQ186372	-0.462810517		GOTERM_CC_FAT	microbody membrane	4	6.60E-01	9.80E-01
ILMN_1796574	BEND4	-0.462813826		GOTERM_CC_FAT	peroxisomal membrane	4	6.60E-01	9.80E-01
ILMN_1711988	KCNK12	-0.462849309		GOTERM_CC_FAT	peroxisomal part	5	6.90E-01	9.80E-01
ILMN_3248990	UBTFL1	-0.462870419		GOTERM_CC_FAT	microbody part	5	6.90E-01	9.80E-01

ILMN_1781752	CLEC16A	0.664237109
ILMN_1721116	USP10	0.664217881
ILMN_2108709	ANKRD11	0.664214754
ILMN_1792344	LOC653075	0.664161733
ILMN_2391458	ACSL4	0.664146908
ILMN_2206098	ATG10	0.66410867
ILMN_1674036	BIRC7	0.664008883
ILMN_2282641	TBXAS1	0.663984355
ILMN_1714327	SETD1A	0.663932846
ILMN_1680134	CARM1	0.663893163
ILMN_1753584	KRT8	0.66375219
ILMN_1674421	TM9SF4	0.66368151
ILMN_1801845	DNAL4	0.663586189
ILMN_1741997	SNRPC	0.663274815
ILMN_2157435	DYNLRB1	0.663211701
ILMN_1653342	DUS1L	0.66306881
ILMN_1809928	COL6A2	0.662983404
ILMN_2366864	JUP	0.662950866
ILMN_1651429	SELM	0.662832499
ILMN_1769779	PTP4A3	0.662799159
ILMN_1782829	GLTSCR1	0.662699983
ILMN_1765043	RPL38	0.662686337
ILMN_2211065	TMEM91	0.662447488
ILMN_2377496	ERCC1	0.662353905
ILMN_1757631	DBNDD1	0.662336505
ILMN_1770085	BTG2	0.662251178
ILMN_2356909	MOSPD3	0.662164961
ILMN_1663577	S100PBP	0.662099212
ILMN_1746012	MBD6	0.662054439
ILMN_1783798	GAS8	0.66192612
ILMN_1659564	SEC61A1	0.66185139
ILMN_3244323	hCG_20425	0.66162293
ILMN_3286389	LOC653075	0.661591243
ILMN_1757439	RBM14	0.6612937
ILMN_1795025	NFX1	0.661171438
ILMN_1731135	RAB11B	0.661149939
ILMN_1787567	TSC22D1	0.660853624
ILMN_1775677	TYSND1	0.660845479
ILMN_1757467	H1F0	0.660696733
ILMN_3210917	SET	0.660677071
ILMN_1672908	TWIST1	0.660586019
ILMN_2189936	RPL36AL	0.660584582
ILMN_1722634	NUCB1	0.660555595
ILMN_1757134	MPV17L2	0.660542967
ILMN_1792951	ZHX2	0.660517834
ILMN_1654118	BCL2L1	0.660482004
ILMN_2134538	FTH1	0.660388058
ILMN_3244798	AV762097	0.660267415
ILMN_2227533	ABHD14B	0.660139969
ILMN_1685387	PIGR	0.660135579
ILMN_1800634	NME4	0.659963146
ILMN_1786396	ZZEF1	0.659882909
ILMN_1808047	PHC2	0.659878124
ILMN_1794072	B3GAT1	0.659796348
ILMN_2181089	VPRBP	0.659730007
ILMN_1784218	DDX23	0.659720074
ILMN_1768181	TOR3A	0.659512821
ILMN_1675239	NDUFA7	0.659483457
ILMN_1798181	IRF7	0.659434799
ILMN_3208973	LAMR1P15	0.659254689
ILMN_1682928	CPVL	0.659195443
ILMN_1651354	SPP1	0.659191842
ILMN_1801833	ARHGAP24	0.65907067
ILMN_1676128	DNMT3A	0.65906758
ILMN_1747903	DNAJC11	0.658741051
ILMN_1774584	C2orf28	0.658729134
ILMN_1653042	HSD3B7	0.658560962
ILMN_2143314	SPIB	0.658543569
ILMN_2078389	SLC4A2	0.658516728
ILMN_1762594	NOD2	0.658424222
ILMN_1753468	CD63	0.658360794
ILMN_1677104	TRAF5	0.658153809
ILMN_2374159	HERPUD1	0.658092516
ILMN_1674160	BIN1	0.65795398
ILMN_1777314	CNPY4	0.657906899
ILMN_2234016	FTH1	0.657648232
ILMN_1668185	ZNF282	0.657619621
ILMN_2358760	HPN	0.657599453
ILMN_2289593	FXYD2	0.65750512
ILMN_2129161	LRRC32	0.657495506
ILMN_1748767	H3F3A	0.65742503
ILMN_3299407	RPS8	0.657329729
ILMN_1654031	TPTE2	0.6573058
ILMN_1769787	SELO	0.657252725
ILMN_1709630	CCDC107	0.657210926
ILMN_3248975	PPP4C	0.657016089
ILMN_2263144	TMEM223	0.656826455
ILMN_1669584	ILF3	0.656789233
ILMN_1652525	FAM125B	0.656666969
ILMN_1766184	LGALS9C	0.656643706
ILMN_2352216	FBXL6	0.656535507
ILMN_1763723	PTDSS2	0.656533806
ILMN_1670256	PDK1	0.656499565
ILMN_1790909	NFE2L2	0.656085042

	GOTERM_CC_FAT	microtubule cytoskeleton	87	6.10E-02	3.80E-01
	SP_PIR_KEYWORDS	microtubule	37	6.30E-02	3.30E-01
	GOTERM_CC_FAT	microtubule	41	3.00E-01	7.80E-01
	GOTERM_BP_FAT	microtubule-based process	28	9.00E-01	1.00E+00
**Annotation Cluster 159**	**Enrichment Score: 0.74**		**Count**	**P_Value**	**Benjamini**
	GOTERM_BP_FAT	regulation of binding	31	1.60E-02	2.60E-01
	GOTERM_BP_FAT	positive regulation of binding	16	8.30E-02	6.30E-01
	GOTERM_BP_FAT	regulation of DNA binding	22	1.10E-01	7.10E-01
	GOTERM_BP_FAT	negative regulation of binding	12	1.50E-01	7.90E-01
	GOTERM_BP_FAT	negative regulation of DNA binding	10	2.20E-01	8.90E-01
	GOTERM_BP_FAT	negative regulation of transcription factor activity	9	2.40E-01	8.90E-01
	GOTERM_BP_FAT	regulation of transcription factor activity	17	2.80E-01	9.20E-01
	GOTERM_BP_FAT	positive regulation of DNA binding	12	3.10E-01	9.40E-01
	GOTERM_BP_FAT	positive regulation of transcription factor activity	9	5.40E-01	9.90E-01
	GOTERM_BP_FAT	positive regulation of NF-kappaB transcription factor activity	6	6.40E-01	1.00E+00
**Annotation Cluster 160**	**Enrichment Score: 0.74**		**Count**	**P_Value**	**Benjamini**
	GOTERM_CC_FAT	leading edge membrane	8	2.60E-02	2.30E-01
	GOTERM_CC_FAT	axolemma	3	1.90E-01	6.50E-01
	GOTERM_CC_FAT	neuron projection membrane	3	2.90E-01	7.80E-01
	GOTERM_CC_FAT	axon part	7	7.50E-01	9.80E-01
**Annotation Cluster 161**	**Enrichment Score: 0.74**		**Count**	**P_Value**	**Benjamini**
	UP_SEQ_FEATURE	domain:WW	7	4.20E-02	9.90E-01
	INTERPRO	WW/Rsp5/WWP	9	3.50E-01	1.00E+00
	SMART	WW	9	4.00E-01	9.60E-01
**Annotation Cluster 162**	**Enrichment Score: 0.74**		**Count**	**P_Value**	**Benjamini**
	GOTERM_BP_FAT	icosanoid biosynthetic process	10	1.60E-02	2.60E-01
	GOTERM_BP_FAT	unsaturated fatty acid biosynthetic process	10	2.80E-02	3.70E-01
	GOTERM_BP_FAT	icosanoid metabolic process	12	3.90E-02	4.40E-01
	GOTERM_BP_FAT	unsaturated fatty acid metabolic process	12	6.50E-02	5.70E-01
	INTERPRO	Peptidase M1, leukotriene A4 hydrolase, aminopeptidase C-terminal	3	8.00E-02	9.50E-01
	GOTERM_MF_FAT	aminopeptidase activity	8	8.20E-02	7.40E-01
	SP_PIR_KEYWORDS	Aminopeptidase	7	8.40E-02	3.90E-01
	GOTERM_BP_FAT	prostanoid metabolic process	6	9.50E-02	6.70E-01
	GOTERM_BP_FAT	prostaglandin metabolic process	6	9.50E-02	6.70E-01
	GOTERM_BP_FAT	prostanoid biosynthetic process	4	1.70E-01	8.20E-01
	GOTERM_BP_FAT	prostaglandin biosynthetic process	4	1.70E-01	8.20E-01
	INTERPRO	Peptidase M1, membrane alanine aminopeptidase	4	1.90E-01	9.90E-01
	INTERPRO	Peptidase M1, membrane alanine aminopeptidase, N-terminal	4	2.20E-01	1.00E+00
	GOTERM_BP_FAT	alkene biosynthetic process	5	2.40E-01	8.90E-01
	GOTERM_BP_FAT	leukotriene biosynthetic process	5	2.40E-01	8.90E-01
	GOTERM_MF_FAT	exopeptidase activity	13	2.80E-01	9.60E-01
	GOTERM_BP_FAT	leukotriene metabolic process	5	3.30E-01	9.50E-01
	GOTERM_BP_FAT	cellular alkene metabolic process	5	3.60E-01	9.60E-01
	GOTERM_BP_FAT	fatty acid biosynthetic process	12	4.70E-01	9.80E-01
	GOTERM_BP_FAT	carboxylic acid biosynthetic process	20	6.70E-01	1.00E+00
	GOTERM_BP_FAT	organic acid biosynthetic process	20	6.70E-01	1.00E+00
	BIOCARTA	Eicosanoid Metabolism	4	8.00E-01	1.00E+00
	GOTERM_BP_FAT	fatty acid metabolic process	22	8.80E-01	1.00E+00
	UP_SEQ_FEATURE	metal ion-binding site:Zinc; catalytic	12	8.90E-01	1.00E+00
**Annotation Cluster 163**	**Enrichment Score: 0.74**		**Count**	**P_Value**	**Benjamini**
	GOTERM_BP_FAT	positive regulation of stress-activated protein kinase signaling pathway	6	1.30E-01	7.60E-01
	GOTERM_BP_FAT	regulation of stress-activated MAPK cascade	4	1.40E-01	7.70E-01
	GOTERM_BP_FAT	positive regulation of stress-activated MAPK cascade	3	3.40E-01	9.50E-01
**Annotation Cluster 164**	**Enrichment Score: 0.74**		**Count**	**P_Value**	**Benjamini**
	GOTERM_BP_FAT	positive regulation of myeloid leukocyte differentiation	6	7.80E-02	6.10E-01
	GOTERM_BP_FAT	positive regulation of myeloid cell differentiation	8	1.40E-01	7.70E-01
	GOTERM_BP_FAT	regulation of myeloid leukocyte differentiation	9	1.60E-01	8.20E-01
	GOTERM_BP_FAT	positive regulation of osteoclast differentiation	3	1.80E-01	8.40E-01
	GOTERM_BP_FAT	regulation of myeloid cell differentiation	13	2.10E-01	8.70E-01
	GOTERM_BP_FAT	regulation of osteoclast differentiation	4	5.70E-01	9.90E-01
**Annotation Cluster 165**	**Enrichment Score: 0.73**		**Count**	**P_Value**	**Benjamini**
	GOTERM_BP_FAT	heme metabolic process	7	6.00E-02	5.50E-01
	GOTERM_BP_FAT	heme biosynthetic process	6	6.30E-02	5.60E-01
	GOTERM_BP_FAT	heterocycle biosynthetic process	13	8.10E-02	6.20E-01
	GOTERM_BP_FAT	tetrapyrrole biosynthetic process	6	1.10E-01	7.20E-01
	GOTERM_BP_FAT	porphyrin biosynthetic process	6	1.10E-01	7.20E-01
	GOTERM_BP_FAT	pigment metabolic process	10	1.30E-01	7.60E-01
	GOTERM_BP_FAT	pigment biosynthetic process	9	1.30E-01	7.60E-01
	GOTERM_BP_FAT	tetrapyrrole metabolic process	7	1.70E-01	8.30E-01
	GOTERM_BP_FAT	porphyrin metabolic process	7	1.70E-01	8.30E-01
	SP_PIR_KEYWORDS	heme biosynthesis	4	2.00E-01	6.30E-01
	SP_PIR_KEYWORDS	porphyrin biosynthesis	3	3.40E-01	7.90E-01
	GOTERM_BP_FAT	cofactor biosynthetic process	13	6.40E-01	1.00E+00
	BIOCARTA	Hemoglobin's Chaperone	3	7.10E-01	1.00E+00
	KEGG_PATHWAY	Porphyrin and chlorophyll metabolism	4	8.90E-01	9.70E-01
**Annotation Cluster 166**	**Enrichment Score: 0.73**		**Count**	**P_Value**	**Benjamini**
	UP_SEQ_FEATURE	domain:MATH	4	1.70E-01	1.00E+00
	INTERPRO	MATH	4	1.90E-01	9.90E-01
	SMART	MATH	4	2.00E-01	9.00E-01
**Annotation Cluster 167**	**Enrichment Score: 0.72**		**Count**	**P_Value**	**Benjamini**
	GOTERM_MF_FAT	phosphatidylinositol transporter activity	3	1.30E-01	8.40E-01
	INTERPRO	Phosphatidylinositol transfer protein	3	1.70E-01	9.90E-01
	GOTERM_MF_FAT	phospholipid transporter activity	6	3.10E-01	9.70E-01
**Annotation Cluster 168**	**Enrichment Score: 0.72**		**Count**	**P_Value**	**Benjamini**
	UP_SEQ_FEATURE	domain:Ras-associating	8	1.10E-01	1.00E+00
	INTERPRO	Ras-association	8	2.30E-01	1.00E+00
	SMART	RA	8	2.70E-01	9.30E-01
**Annotation Cluster 169**	**Enrichment Score: 0.72**		**Count**	**P_Value**	**Benjamini**
	UP_SEQ_FEATURE	domain:RUN	5	1.40E-01	1.00E+00
	INTERPRO	RUN	5	2.10E-01	1.00E+00
	SMART	RUN	5	2.40E-01	9.20E-01
**Annotation Cluster 170**	**Enrichment Score: 0.72**		**Count**	**P_Value**	**Benjamini**
	GOTERM_CC_FAT	SWI/SNF complex	5	1.30E-01	5.60E-01
	GOTERM_CC_FAT	SWI/SNF-type complex	5	1.30E-01	5.60E-01

ILMN_1767230	AXDND1	-0.462985533
ILMN_1817255	LOC144438	-0.463031801
ILMN_1768930	U2AF2	-0.463062918
ILMN_1860288	MBTPS2	-0.463085596
ILMN_3242977	FLJ33630	-0.463121596
ILMN_1734229	SPPL2A	-0.463267776
ILMN_3292114	LOC100131510	-0.4632744
ILMN_1796583	CLRN2	-0.463348463
ILMN_1718042	ZNF549	-0.46335229
ILMN_1748819	MRPL22	-0.463357528
ILMN_1683390	FLCN	-0.463378395
ILMN_1790603	ATPAF1	-0.463427045
ILMN_1795000	OR8J3	-0.463438428
ILMN_2413259	SOCS4	-0.463474005
ILMN_2089484	CYP4F3	-0.463475685
ILMN_1661966	UGCGL1	-0.463507271
ILMN_1736630	LOC100132024	-0.46356997
ILMN_1895518	AW468861	-0.463575816
ILMN_1810330	LOC440131	-0.463577729
ILMN_1804361	SUFU	-0.4636211
ILMN_1672957	RPL32P3	-0.463641722
ILMN_1744059	DCTN6	-0.463671021
ILMN_1695036	TPPP3	-0.463706361
ILMN_1733696	IMP3	-0.463724496
ILMN_2069821	C4orf32	-0.463785453
ILMN_1660727	ENPP5	-0.463798942
ILMN_3255965	NA	-0.463835049
ILMN_2221336	QRFPR	-0.463852421
ILMN_2382717	OPN3	-0.463866346
ILMN_1724367	NDUFB1	-0.463939172
ILMN_1670625	DKFZp434A171	-0.463962596
ILMN_1774930	CDC14A	-0.463997283
ILMN_3236182	GCNT4	-0.464006634
ILMN_1742203	DYDC1	-0.464010982
ILMN_1656218	AK131040	-0.464012025
ILMN_1657436	FGFR1OP2	-0.464128654
ILMN_1750447	GOLGA6A	-0.464145869
ILMN_1808537	LRRC66	-0.464304091
ILMN_1774589	IQCC	-0.464308049
ILMN_1704255	FAM159B	-0.464316652
ILMN_2138745	C14orf37	-0.464334062
ILMN_3305788	NA	-0.464452296
ILMN_1707694	FAM22A	-0.464482571
ILMN_3235931	LOC100132287	-0.464488957
ILMN_1744701	RGSL1	-0.464522237
ILMN_1715742	SLC22A1	-0.464539519
ILMN_2334322	MIER1	-0.464542661
ILMN_1653876	SPG21	-0.464556224
ILMN_3243986	FAM72B	-0.464608865
ILMN_2211189	IKBKAP	-0.464716851
ILMN_1694952	SSX4B	-0.464773608
ILMN_1694734	RUNX1T1	-0.464779476
ILMN_1732860	FLJ26850	-0.464843623
ILMN_1751816	MCTS1	-0.464859519
ILMN_1705085	C8orf77	-0.464880181
ILMN_1687439	SNX20	-0.464892833
ILMN_1685057	SLC22A4	-0.464940871
ILMN_2060578	INSL5	-0.464970899
ILMN_1684746	IPO11	-0.465074254
ILMN_1760431	GABRR1	-0.465116271
ILMN_1660323	PDXDC1	-0.465180109
ILMN_1678246	SLC25A32	-0.465208692
ILMN_1752213	TMEM60	-0.465233708
ILMN_2189668	NUDT11	-0.465240638
ILMN_1807304	MBNL1	-0.465256516
ILMN_3282561	LOC100132272	-0.465354651
ILMN_1888015	AI525756	-0.465355237
ILMN_1785615	SUMO1P1	-0.465386407
ILMN_1672962	LOC645485	-0.465402467
ILMN_1670805	PRR20A	-0.465523449
ILMN_1717485	AL832378	-0.465565336
ILMN_3243414	LOC100133161	-0.465574713
ILMN_2410371	CD79A	-0.46560942
ILMN_1715638	GAGE8	-0.465625119
ILMN_1853210	AW117187	-0.465626213
ILMN_1765310	TCEAL2	-0.465722059
ILMN_1752728	FUCA1	-0.465825871
ILMN_1706615	SLC22A7	-0.465833951
ILMN_1741518	LOC100128035	-0.465848794
ILMN_3248228	PROX2	-0.465880704
ILMN_1849859	BU616091	-0.46589324
ILMN_1793508	C7orf36	-0.466004572
ILMN_3240893	SLMO2	-0.466006216
ILMN_1739486	BX647938	-0.466025033
ILMN_1726642	BEND3	-0.466051337
ILMN_1663131	LYST	-0.46606158
ILMN_1893697	AA532505	-0.466078333
ILMN_1868572	C6orf168	-0.466098715
ILMN_3269520	LOC100130673	-0.466099176
ILMN_1911806	BQ940594	-0.466107422
ILMN_1813344	C20orf7	-0.466112309
ILMN_3247434	LOC100128977	-0.466138241
ILMN_1729272	DIP2A	-0.466170826
ILMN_1817651	BX091227	-0.466197974

ILMN_2105966	SLC35A4	0.656083511
ILMN_1814917	TLE2	0.656022695
ILMN_2394027	CLK3	0.655989811
ILMN_2330552	CDK11A	0.655953672
ILMN_1747593	ISLR	0.655785374
ILMN_2382500	MS4A14	0.655730543
ILMN_1685433	COL8A1	0.655630543
ILMN_2390919	FBLN2	0.65550337
ILMN_2212690	ZC3H7A	0.655449582
ILMN_1654060	MKNK2	0.655429293
ILMN_1794165	PGD	0.655361599
ILMN_1757877	HCFC1R1	0.655289821
ILMN_1674811	OASL	0.655200294
ILMN_1776953	MYL9	0.655087788
ILMN_1684258	RPS28	0.655071645
ILMN_1677635	TUBA4B	0.654993849
ILMN_1744487	C1QTNF5	0.654760753
ILMN_1803846	EIF1	0.654706558
ILMN_1752249	FAM38A	0.654613797
ILMN_1684391	PLOD1	0.654601479
ILMN_2189933	RPL36AL	0.654522637
ILMN_1703718	CCT7	0.654390059
ILMN_1769129	CCL19	0.654366457
ILMN_1748812	TMEM130	0.654139742
ILMN_1712155	RPL6	0.65410387
ILMN_1810420	DYSF	0.654016911
ILMN_1712515	FOXO3	0.653909675
ILMN_1872419	AW276479	0.653862958
ILMN_1716687	TPM1	0.653662339
ILMN_1694259	NSA2	0.653494417
ILMN_1802799	AKIRIN1	0.653447736
ILMN_1675130	NFIC	0.653279278
ILMN_2241953	PILRA	0.653184116
ILMN_1730698	ODF2	0.653115176
ILMN_1719256	CKS1B	0.653075236
ILMN_1693334	P4HA1	0.653058128
ILMN_3240375	abParts	0.652854973
ILMN_1696843	NPIPL3	0.652731688
ILMN_1739840	LRRC8A	0.652657803
ILMN_1704253	C6orf106	0.652631303
ILMN_2174081	ZNF133	0.652535356
ILMN_1680274	abParts	0.652494909
ILMN_1719611	CCT6P3	0.652401612
ILMN_2347145	DCN	0.652396222
ILMN_1656082	H3F3A	0.652210278
ILMN_1778337	HNF1B	0.652195861
ILMN_1753890	IFT20	0.652166796
ILMN_1771599	PLOD2	0.65209355
ILMN_2389501	HSD11B1	0.652047615
ILMN_1689007	SUGP2	0.652012182
ILMN_1759436	NOSIP	0.651984083
ILMN_1685378	RPS7	0.651926927
ILMN_3261197	ATP5E	0.65187189
ILMN_1759419	ILVBL	0.651656546
ILMN_2082244	FOXK1	0.65164199
ILMN_1761463	EFHD2	0.651601653
ILMN_2191428	UBB	0.651552987
ILMN_1725700	MOV10	0.651521456
ILMN_1716336	CKAP5	0.651433031
ILMN_3251536	FAM127C	0.651403377
ILMN_1763603	FAM177A1	0.651364419
ILMN_1670134	FADS1	0.651333747
ILMN_1688279	PVRIG	0.651304312
ILMN_1678290	HMG20A	0.651138322
ILMN_1739508	abParts	0.651073095
ILMN_1759325	C16orf91	0.650995539
ILMN_1805519	DQ893812	0.650963974
ILMN_3235709	HNF1B	0.650881266
ILMN_1659189	C9orf89	0.650851083
ILMN_1780397	TRAF3IP3	0.650726968
ILMN_1687442	TNIK	0.65054193
ILMN_1692706	DCUN1D2	0.650508496
ILMN_2088990	CXorf21	0.650410073
ILMN_2041577	GPR172A	0.650382138
ILMN_2069128	EPB41L2	0.650359696
ILMN_1815158	GPS2	0.650296365
ILMN_1737406	KLF6	0.650223224
ILMN_1712687	PAK2	0.650203508
ILMN_2396956	AKAP13	0.65012727
ILMN_3180557	CYB561D1	0.650100296
ILMN_1766221	B4GALT1	0.650052639
ILMN_3278548	RPL7	0.650024561
ILMN_1811579	HOMER3	0.649891391
ILMN_2398432	BRMS1	0.649592127
ILMN_1802523	HS3ST5	0.649280407
ILMN_1761733	HLA-DMB	0.649268095
ILMN_1737862	PSMB4	0.649195609
ILMN_3307930	RAN	0.649149211
ILMN_1757627	ZMYND19	0.649104675
ILMN_2136133	PABPC1	0.648992521
ILMN_1695962	SLC12A9	0.648974306
ILMN_1669308	BRPF1	0.648970928
ILMN_1793522	PRKAB1	0.648924796
ILMN_1764230	GNPTG	0.648791896

	BIOCARTA	Chromatin Remodeling by hSWI/SNF ATP-dependent Complexes	5	4.20E-01	9.70E-01
**Annotation Cluster 171**	**Enrichment Score: 0.72**		**Count**	**P_Value**	**Benjamini**
	UP_SEQ_FEATURE	domain:PSI	6	9.80E-03	9.00E-01
	INTERPRO	Plexin/semaphorin/integrin	11	5.10E-02	9.20E-01
	SMART	PSI	11	6.50E-02	7.50E-01
	INTERPRO	Integrin beta subunit, N-terminal	4	6.90E-02	9.50E-01
	SMART	INB	4	7.70E-02	7.60E-01
	UP_SEQ_FEATURE	region of interest:Cysteine-rich tandem repeats	4	8.50E-02	1.00E+00
	INTERPRO	Integrin beta subunit, C-terminal	4	9.40E-02	9.60E-01
	INTERPRO	Integrin beta subunit	4	9.40E-02	9.60E-01
	GOTERM_CC_FAT	integrin complex	7	1.80E-01	6.50E-01
	INTERPRO	Integrin beta subunit, tail	3	2.20E-01	1.00E+00
	INTERPRO	Integrin beta subunit, subgroup	3	2.20E-01	1.00E+00
	PIR_SUPERFAMILY	PIRSF002512:integrin, beta subunit	3	2.40E-01	1.00E+00
	PIR_SUPERFAMILY	PIRSF002512:Integrin_B	3	2.40E-01	1.00E+00
	GOTERM_BP_FAT	integrin-mediated signaling pathway	12	3.10E-01	9.40E-01
	INTERPRO	Plexin	6	3.50E-01	1.00E+00
	SP_PIR_KEYWORDS	integrin	7	3.70E-01	8.10E-01
	UP_SEQ_FEATURE	repeat:I	4	6.30E-01	1.00E+00
	UP_SEQ_FEATURE	repeat:IV	4	6.30E-01	1.00E+00
	UP_SEQ_FEATURE	repeat:III	4	6.30E-01	1.00E+00
	UP_SEQ_FEATURE	repeat:II	4	6.30E-01	1.00E+00
	GOTERM_BP_FAT	cell-matrix adhesion	12	6.40E-01	1.00E+00
	INTERPRO	EGF, extracellular	8	7.00E-01	1.00E+00
**Annotation Cluster 172**	**Enrichment Score: 0.71**		**Count**	**P_Value**	**Benjamini**
	GOTERM_CC_FAT	organellar large ribosomal subunit	6	8.10E-02	4.60E-01
	GOTERM_CC_FAT	mitochondrial large ribosomal subunit	6	8.10E-02	4.60E-01
	GOTERM_CC_FAT	organellar ribosome	8	4.60E-01	9.00E-01
	GOTERM_CC_FAT	mitochondrial ribosome	8	4.60E-01	9.00E-01
**Annotation Cluster 173**	**Enrichment Score: 0.7**		**Count**	**P_Value**	**Benjamini**
	GOTERM_BP_FAT	covalent chromatin modification	26	2.30E-02	3.20E-01
	GOTERM_BP_FAT	histone modification	25	2.80E-02	3.70E-01
	GOTERM_BP_FAT	protein amino acid acetylation	10	2.40E-01	9.00E-01
	GOTERM_BP_FAT	histone acetylation	9	3.00E-01	9.30E-01
	GOTERM_CC_FAT	histone acetyltransferase complex	9	3.50E-01	8.30E-01
	GOTERM_BP_FAT	protein amino acid acylation	10	3.90E-01	9.70E-01
	GOTERM_BP_FAT	histone H4 acetylation	4	5.00E-01	9.90E-01
	GOTERM_BP_FAT	histone H3 acetylation	3	8.80E-01	1.00E+00
**Annotation Cluster 174**	**Enrichment Score: 0.69**		**Count**	**P_Value**	**Benjamini**
	GOTERM_CC_FAT	anchored to external side of plasma membrane	3	1.40E-01	5.70E-01
	GOTERM_CC_FAT	anchored to plasma membrane	6	1.60E-01	6.10E-01
	GOTERM_CC_FAT	intrinsic to external side of plasma membrane	3	3.90E-01	8.60E-01
**Annotation Cluster 175**	**Enrichment Score: 0.67**		**Count**	**P_Value**	**Benjamini**
	GOTERM_BP_FAT	regulation of RNA splicing	6	1.10E-01	7.20E-01
	GOTERM_BP_FAT	regulation of mRNA processing	5	1.80E-01	8.30E-01
	GOTERM_BP_FAT	regulation of nuclear mRNA splicing, via spliceosome	3	4.80E-01	9.90E-01
**Annotation Cluster 176**	**Enrichment Score: 0.67**		**Count**	**P_Value**	**Benjamini**
	SP_PIR_KEYWORDS	molecular chaperone	5	6.20E-02	3.30E-01
	INTERPRO	Chaperone, tailless complex polypeptide 1	4	1.50E-01	9.90E-01
	GOTERM_CC_FAT	chaperonin-containing T-complex	3	2.40E-01	7.20E-01
	INTERPRO	Chaperonin Cpn60/TCP-1	4	2.90E-01	1.00E+00
	INTERPRO	Chaperonin TCP-1, conserved site	3	3.70E-01	1.00E+00
	PIR_SUPERFAMILY	PIRSF002584:molecular chaperone t-complex-type	3	4.00E-01	1.00E+00
**Annotation Cluster 177**	**Enrichment Score: 0.66**		**Count**	**P_Value**	**Benjamini**
	GOTERM_BP_FAT	negative regulation of tyrosine phosphorylation of Stat3 protein	3	8.70E-02	6.40E-01
	GOTERM_BP_FAT	negative regulation of phosphate metabolic process	11	9.30E-02	6.60E-01
	GOTERM_BP_FAT	negative regulation of phosphorus metabolic process	11	9.30E-02	6.60E-01
	GOTERM_BP_FAT	negative regulation of peptidyl-tyrosine phosphorylation	4	1.00E-01	7.00E-01
	GOTERM_BP_FAT	negative regulation of protein amino acid phosphorylation	8	1.20E-01	7.30E-01
	GOTERM_BP_FAT	regulation of tyrosine phosphorylation of Stat3 protein	5	1.80E-01	8.30E-01
	GOTERM_BP_FAT	negative regulation of tyrosine phosphorylation of STAT protein	3	1.80E-01	8.40E-01
	GOTERM_BP_FAT	negative regulation of JAK-STAT cascade	3	2.30E-01	8.90E-01
	GOTERM_BP_FAT	negative regulation of phosphorylation	9	2.40E-01	8.90E-01
	GOTERM_BP_FAT	regulation of JAK-STAT cascade	7	3.40E-01	9.50E-01
	GOTERM_BP_FAT	negative regulation of protein kinase cascade	7	3.40E-01	9.50E-01
	GOTERM_BP_FAT	regulation of tyrosine phosphorylation of STAT protein	6	3.90E-01	9.70E-01
	GOTERM_BP_FAT	positive regulation of tyrosine phosphorylation of STAT protein	3	8.30E-01	1.00E+00
	GOTERM_BP_FAT	positive regulation of JAK-STAT cascade	3	8.80E-01	1.00E+00
**Annotation Cluster 178**	**Enrichment Score: 0.66**		**Count**	**P_Value**	**Benjamini**
	UP_SEQ_FEATURE	domain:W2	3	2.00E-01	1.00E+00
	INTERPRO	eIF4-gamma/eIF5/eIF2-epsilon	3	2.20E-01	1.00E+00
	SMART	eIF5C	3	2.30E-01	9.20E-01
**Annotation Cluster 179**	**Enrichment Score: 0.66**		**Count**	**P_Value**	**Benjamini**
	GOTERM_BP_FAT	amyloid precursor protein catabolic process	5	2.20E-02	3.10E-01
	GOTERM_BP_FAT	amyloid precursor protein metabolic process	5	4.60E-02	4.80E-01
	GOTERM_BP_FAT	membrane protein ectodomain proteolysis	5	1.30E-01	7.50E-01
	GOTERM_BP_FAT	Notch receptor processing	3	1.80E-01	8.40E-01
	GOTERM_BP_FAT	glycoprotein catabolic process	5	2.10E-01	8.70E-01
	GOTERM_BP_FAT	membrane protein proteolysis	5	2.70E-01	9.20E-01
	GOTERM_BP_FAT	membrane protein intracellular domain proteolysis	3	4.40E-01	9.80E-01
	KEGG_PATHWAY	Notch signaling pathway	8	5.70E-01	8.30E-01
	SP_PIR_KEYWORDS	notch signaling pathway	4	8.90E-01	9.90E-01
	GOTERM_BP_FAT	Notch signaling pathway	5	9.30E-01	1.00E+00
**Annotation Cluster 180**	**Enrichment Score: 0.66**		**Count**	**P_Value**	**Benjamini**
	UP_SEQ_FEATURE	short sequence motif:LXXLL motif 3	4	1.40E-01	1.00E+00
	UP_SEQ_FEATURE	short sequence motif:LXXLL motif 5	3	2.00E-01	1.00E+00
	UP_SEQ_FEATURE	short sequence motif:LXXLL motif 2	5	2.40E-01	1.00E+00
	UP_SEQ_FEATURE	short sequence motif:LXXLL motif 1	5	2.40E-01	1.00E+00
	UP_SEQ_FEATURE	short sequence motif:LXXLL motif 4	3	3.00E-01	1.00E+00
**Annotation Cluster 181**	**Enrichment Score: 0.66**		**Count**	**P_Value**	**Benjamini**
	UP_SEQ_FEATURE	domain:UBX	4	2.00E-01	1.00E+00
	INTERPRO	UBX	4	2.20E-01	1.00E+00
	SMART	UBX	4	2.40E-01	9.20E-01
**Annotation Cluster 182**	**Enrichment Score: 0.66**		**Count**	**P_Value**	**Benjamini**

ILMN_3309614	MIR1-1	-0.466386075
ILMN_1904135	AK023536	-0.466390952
ILMN_1790062	FAM105B	-0.466414379
ILMN_1794807	CACHD1	-0.466438363
ILMN_3309814	MIR208B	-0.466441332
ILMN_1903750	U10515	-0.466481294
ILMN_1693042	WDR90	-0.46651748
ILMN_3237446	SCARNA16	-0.466536328
ILMN_2089977	FKBP9L	-0.46654968
ILMN_3235168	MUL1	-0.466593345
ILMN_1698318	LGALS14	-0.466644467
ILMN_3242059	C6orf221	-0.466701561
ILMN_1739559	FAM187B	-0.466820572
ILMN_1675695	PDS5B	-0.466856482
ILMN_1839956	NA	-0.466871559
ILMN_1785535	ARPC4	-0.466891826
ILMN_1655039	SLC27A4	-0.467008949
ILMN_1668531	BC029817	-0.467026949
ILMN_1656867	PLA2G2C	-0.467083318
ILMN_1692026	SUV420H1	-0.467086463
ILMN_1740648	CYP2D6	-0.467183257
ILMN_3242856	BEND3	-0.467188233
ILMN_1692688	RGS12	-0.467251711
ILMN_1657860	C1orf151	-0.467281156
ILMN_2353358	LGALS8	-0.467350998
ILMN_1775268	HECW2	-0.467379419
ILMN_2187727	NOC3L	-0.467394014
ILMN_1732491	OFCC1	-0.467455284
ILMN_1812100	HEATR7A	-0.46747241
ILMN_1673099	PTPN20A	-0.467494038
ILMN_3245559	CDK2AP1	-0.467548429
ILMN_1791297	LOC649305	-0.467600865
ILMN_3262015	AK024162	-0.467611758
ILMN_1875967	BG222050	-0.467630011
ILMN_1758938	SLC31A2	-0.467666404
ILMN_1693843	HELT	-0.467666816
ILMN_1753450	ATXN3L	-0.467688251
ILMN_2152387	DOCK7	-0.467929693
ILMN_3310136	MIR605	-0.467983259
ILMN_1676629	INSIG2	-0.468034473
ILMN_1714643	MGAM	-0.468041434
ILMN_1704369	LIMA1	-0.468054209
ILMN_3208428	NA	-0.468087847
ILMN_2346997	RAB23	-0.46809061
ILMN_2157544	GBF1	-0.468097319
ILMN_1729775	OPA1	-0.468130591
ILMN_2166686	NUFIP1	-0.468147386
ILMN_1853705	AW118645	-0.468161779
ILMN_1667229	HCG22	-0.468185191
ILMN_2283196	ZSWIM3	-0.4683098
ILMN_1674190	LOC100507206	-0.468312809
ILMN_1769011	KIAA1456	-0.468333468
ILMN_2352926	BPGM	-0.468335546
ILMN_1794070	ANKRD13B	-0.468526692
ILMN_3261292	EBF4	-0.46853916
ILMN_2184556	SLC4A4	-0.468540888
ILMN_1655296	UTRN	-0.468581747
ILMN_3237085	NTF4	-0.468607042
ILMN_1809639	TMEM26	-0.468622759
ILMN_1764396	HDAC4	-0.468661224
ILMN_1752843	GRM4	-0.468744452
ILMN_1793832	GRB14	-0.46876901
ILMN_3239541	BC131768	-0.468787874
ILMN_1742040	GDPD4	-0.468923703
ILMN_1667018	ACE2	-0.46894057
ILMN_3239177	MEX3A	-0.468984692
ILMN_3246909	LOC256880	-0.469011429
ILMN_1779070	APOLIV	-0.469094128
ILMN_1799299	PRPF38B	-0.469096519
ILMN_3269652	TUBBP5	-0.469115687
ILMN_1892652	DB335929	-0.469156047
ILMN_2390457	ESR2	-0.46916113
ILMN_3307791	FAM69A	-0.469177238
ILMN_1725401	DEFB104B	-0.469219297
ILMN_1670124	YDJC	-0.469274602
ILMN_1690911	C16orf87	-0.469336638
ILMN_1655165	RNF138	-0.469384277
ILMN_1722445	MC4R	-0.469395113
ILMN_1685661	RRP15	-0.469403129
ILMN_1757427	XAGE1D	-0.469407343
ILMN_1695506	DKK2	-0.469412407
ILMN_3251526	USP13	-0.46942161
ILMN_2160764	HBP1	-0.469506567
ILMN_1766643	HOPX	-0.469512928
ILMN_1677269	MAGEA2	-0.469598079
ILMN_1811277	TRIM13	-0.469626386
ILMN_1772557	SLC22A10	-0.469644149
ILMN_2301955	THAP1	-0.469789765
ILMN_3227031	MS4A18	-0.469792946
ILMN_3238130	DUX2	-0.469858965
ILMN_1739144	SI	-0.469900225
ILMN_1759690	PRB2	-0.469920712
ILMN_1757272	THAP1	-0.469989041
ILMN_1666546	DUSP14	-0.469993341

ILMN_1740587	RPS11	0.648719151
ILMN_2383306	GPATCH4	0.648604602
ILMN_1697554	SASH3	0.648502748
ILMN_1680624	CREG1	0.648359421
ILMN_1690566	RASSF4	0.648292113
ILMN_1651850	RPS16	0.648245488
ILMN_2364022	SLC16A3	0.648036352
ILMN_1790472	SLC25A28	0.647999833
ILMN_1670561	TCEAL8	0.647988804
ILMN_2364384	PPARG	0.647916434
ILMN_1758798	NPIPL2	0.647913515
ILMN_1657708	MGLL	0.647911016
ILMN_1674519	ZP2	0.647888247
ILMN_1767475	CERK	0.647765033
ILMN_1763207	BATF3	0.647689072
ILMN_1691117	DNTTIP1	0.647501964
ILMN_1780806	ANKRD36B	0.647232295
ILMN_2325168	ARRB1	0.64723057
ILMN_1659058	PPP1R10	0.647122928
ILMN_1690105	STAT1	0.646980823
ILMN_1656482	OSBPL2	0.646968308
ILMN_1774207	ANGPT2	0.646942108
ILMN_1811195	ZNF211	0.646900036
ILMN_1666269	CTSZ	0.646876047
ILMN_1766803	TUBGCP6	0.646779075
ILMN_1689932	WDR85	0.646778371
ILMN_1749722	RNF213	0.6467514
ILMN_1660341	LRPAP1	0.646609936
ILMN_1751328	FAM83H	0.646493113
ILMN_3285819	NA	0.64642578
ILMN_1743097	XRCC6	0.646388804
ILMN_1766185	AXIN1	0.646235652
ILMN_1728024	TUBG2	0.64622909
ILMN_1800261	TUBA1B	0.646190746
ILMN_1730048	C7orf26	0.646100155
ILMN_1667551	SMA4	0.646085609
ILMN_1652749	ERF	0.646050754
ILMN_2363165	TACC2	0.646049908
ILMN_1803560	LAT2	0.645981039
ILMN_1703412	LATS2	0.645972871
ILMN_1691449	CHCHD2	0.645829087
ILMN_1666169	NBR2	0.64571977
ILMN_1722820	KDELR3	0.645574177
ILMN_1665554	BRF2	0.645567397
ILMN_1731666	ZNF641	0.645527247
ILMN_2056551	RBMX2	0.64539567
ILMN_1689725	RPLP1	0.645324939
ILMN_2311166	ITGB5	0.645162054
ILMN_2294878	NSUN5	0.645125427
ILMN_3269537	RPS2	0.645118614
ILMN_1809141	ING4	0.645115556
ILMN_2194828	C16orf53	0.645004757
ILMN_1727761	GMEB1	0.644906018
ILMN_1778013	PDPR	0.644781906
ILMN_2226763	H2AFB2	0.644766692
ILMN_2305721	POMT1	0.644241239
ILMN_1715332	TTC21A	0.644098518
ILMN_1734608	ZNF77	0.644020008
ILMN_1684663	ZDHHC13	0.644014898
ILMN_2370414	CHD3	0.643992057
ILMN_3304012	NA	0.643770383
ILMN_1722059	SAFB	0.643514246
ILMN_1812769	UBXN1	0.643509069
ILMN_1769665	RAB5C	0.64348971
ILMN_1719237	SPDYE8P	0.64337813
ILMN_3307799	PSMD4	0.643136118
ILMN_1703053	ZFP91	0.643070727
ILMN_1815874	NANS	0.64306804
ILMN_1652223	WDR91	0.642961922
ILMN_2328835	IP6K2	0.642952858
ILMN_2320964	ADAR	0.642800995
ILMN_2101930	POLG	0.642760054
ILMN_1675626	PRPF38A	0.642713365
ILMN_2349129	DPP3	0.642640033
ILMN_1809889	CCDC117	0.642577425
ILMN_2357976	DDX39B	0.642577256
ILMN_1788377	COL27A1	0.642557904
ILMN_2047112	DPCD	0.642517811
ILMN_2134062	ALKBH3	0.641789203
ILMN_1691131	LSMD1	0.641742692
ILMN_1813025	AK125786	0.641719946
ILMN_1779071	FEZ1	0.641517956
ILMN_2151277	LPGAT1	0.641485064
ILMN_2056032	CD99	0.641476689
ILMN_1812067	RER1	0.641472642
ILMN_1690802	TRMT112	0.641450614
ILMN_1702065	MFSD5	0.641366445
ILMN_1716080	CBL	0.641237401
ILMN_1713301	DGCR2	0.641171187
ILMN_2325506	BCAS4	0.641168308
ILMN_1687037	IFT140	0.640959966
ILMN_2287296	SYF2	0.640923742
ILMN_3283772	GAPDH	0.640852411
ILMN_1699440	ZBTB47	0.640730326

	INTERPRO	PAK-box/P21-Rho-binding	5	1.80E-01	9.90E-01
	UP_SEQ_FEATURE	domain:CRIB	5	1.90E-01	1.00E+00
	SMART	PBD	5	2.10E-01	9.00E-01
	SP_PIR_KEYWORDS	cell shape	5	3.30E-01	7.80E-01
**Annotation Cluster 183**	**Enrichment Score: 0.65**		**Count**	**P_Value**	**Benjamini**
	UP_SEQ_FEATURE	repeat:HAT 5	4	1.40E-01	1.00E+00
	UP_SEQ_FEATURE	repeat:HAT 4	4	1.70E-01	1.00E+00
	UP_SEQ_FEATURE	repeat:HAT 1	4	2.00E-01	1.00E+00
	UP_SEQ_FEATURE	repeat:HAT 3	4	2.00E-01	1.00E+00
	UP_SEQ_FEATURE	repeat:HAT 2	4	2.00E-01	1.00E+00
	UP_SEQ_FEATURE	repeat:HAT 7	3	2.00E-01	1.00E+00
	UP_SEQ_FEATURE	repeat:HAT 6	3	3.00E-01	1.00E+00
	INTERPRO	RNA-processing protein, HAT helix	3	3.20E-01	1.00E+00
	SMART	HAT	3	3.40E-01	9.60E-01
**Annotation Cluster 184**	**Enrichment Score: 0.65**		**Count**	**P_Value**	**Benjamini**
	GOTERM_BP_FAT	branching morphogenesis of a tube	13	1.40E-01	7.80E-01
	GOTERM_BP_FAT	morphogenesis of a branching structure	14	1.70E-01	8.20E-01
	GOTERM_BP_FAT	tube development	35	1.80E-01	8.40E-01
	GOTERM_BP_FAT	patterning of blood vessels	5	3.00E-01	9.30E-01
	GOTERM_BP_FAT	tube morphogenesis	19	4.10E-01	9.70E-01
**Annotation Cluster 185**	**Enrichment Score: 0.65**		**Count**	**P_Value**	**Benjamini**
	GOTERM_BP_FAT	regulation of lipid kinase activity	4	1.40E-01	7.70E-01
	GOTERM_BP_FAT	regulation of phosphoinositide 3-kinase activity	3	1.80E-01	8.40E-01
	GOTERM_BP_FAT	positive regulation of phosphoinositide 3-kinase activity	3	1.80E-01	8.40E-01
	GOTERM_BP_FAT	positive regulation of lipid metabolic process	7	5.80E-01	9.90E-01
**Annotation Cluster 186**	**Enrichment Score: 0.64**		**Count**	**P_Value**	**Benjamini**
	GOTERM_MF_FAT	protein binding, bridging	19	5.30E-02	6.20E-01
	GOTERM_MF_FAT	SH3/SH2 adaptor activity	8	4.40E-01	9.90E-01
	GOTERM_MF_FAT	molecular adaptor activity	10	5.00E-01	9.90E-01
**Annotation Cluster 187**	**Enrichment Score: 0.64**		**Count**	**P_Value**	**Benjamini**
	INTERPRO	Tropomyosin	3	8.00E-02	9.50E-01
	GOTERM_CC_FAT	muscle thin filament tropomyosin	3	8.90E-02	4.70E-01
	PIR_SUPERFAMILY	PIRSF002327:tropomyosin	3	9.10E-02	1.00E+00
	SP_PIR_KEYWORDS	muscle protein	9	4.40E-01	8.60E-01
	GOTERM_CC_FAT	striated muscle thin filament	3	6.10E-01	9.50E-01
	SP_PIR_KEYWORDS	skeletal muscle	3	8.00E-01	9.90E-01
**Annotation Cluster 188**	**Enrichment Score: 0.64**		**Count**	**P_Value**	**Benjamini**
	GOTERM_BP_FAT	phosphoinositide phosphorylation	5	1.30E-01	7.50E-01
	GOTERM_BP_FAT	lipid phosphorylation	5	1.50E-01	7.90E-01
	UP_SEQ_FEATURE	domain:FAT	3	1.60E-01	1.00E+00
	UP_SEQ_FEATURE	domain:FATC	3	1.60E-01	1.00E+00
	UP_SEQ_FEATURE	domain:PI3K/PI4K	5	1.70E-01	1.00E+00
	INTERPRO	PIK-related kinase, FATC	3	1.70E-01	9.90E-01
	INTERPRO	PIK-related kinase	3	1.70E-01	9.90E-01
	INTERPRO	Phosphatidylinositol 3- and 4-kinase, conserved site	5	1.80E-01	9.90E-01
	INTERPRO	Phosphatidylinositol 3- and 4-kinase, catalytic	5	1.80E-01	9.90E-01
	SMART	PI3Kc	5	2.10E-01	9.00E-01
	GOTERM_CC_FAT	phosphoinositide 3-kinase complex	3	4.90E-01	9.10E-01
	KEGG_PATHWAY	Type II diabetes mellitus	7	7.30E-01	9.10E-01
	KEGG_PATHWAY	mTOR signaling pathway	7	8.10E-01	9.30E-01
**Annotation Cluster 189**	**Enrichment Score: 0.64**		**Count**	**P_Value**	**Benjamini**
	GOTERM_BP_FAT	response to vitamin A	9	1.80E-01	8.40E-01
	GOTERM_BP_FAT	response to vitamin	12	2.50E-01	9.00E-01
	GOTERM_BP_FAT	response to retinoic acid	7	2.60E-01	9.20E-01
**Annotation Cluster 190**	**Enrichment Score: 0.64**		**Count**	**P_Value**	**Benjamini**
	GOTERM_MF_FAT	transforming growth factor beta binding	4	1.30E-01	8.40E-01
	GOTERM_MF_FAT	activin binding	4	1.60E-01	8.70E-01
	GOTERM_MF_FAT	transmembrane receptor protein serine/threonine kinase activity	5	1.90E-01	9.00E-01
	GOTERM_MF_FAT	transforming growth factor beta receptor activity	5	1.90E-01	9.00E-01
	GOTERM_MF_FAT	type I transforming growth factor beta receptor binding	3	2.20E-01	9.30E-01
	GOTERM_MF_FAT	transforming growth factor beta receptor binding	4	3.80E-01	9.80E-01
	GOTERM_MF_FAT	SMAD binding	7	5.50E-01	9.90E-01
**Annotation Cluster 191**	**Enrichment Score: 0.64**		**Count**	**P_Value**	**Benjamini**
	GOTERM_CC_FAT	basolateral plasma membrane	35	9.50E-02	4.80E-01
	GOTERM_CC_FAT	cell-substrate junction	20	1.60E-01	6.20E-01
	GOTERM_CC_FAT	adherens junction	26	1.80E-01	6.50E-01
	GOTERM_CC_FAT	focal adhesion	18	2.00E-01	6.70E-01
	GOTERM_CC_FAT	cell-substrate adherens junction	18	2.40E-01	7.30E-01
	GOTERM_CC_FAT	anchoring junction	27	2.80E-01	7.60E-01
	GOTERM_CC_FAT	cell junction	58	9.60E-01	1.00E+00
**Annotation Cluster 192**	**Enrichment Score: 0.63**		**Count**	**P_Value**	**Benjamini**
	GOTERM_MF_FAT	histone deacetylase activity	5	1.20E-01	8.20E-01
	GOTERM_MF_FAT	protein deacetylase activity	5	1.20E-01	8.20E-01
	GOTERM_BP_FAT	protein amino acid deacetylation	6	1.50E-01	8.00E-01
	GOTERM_BP_FAT	histone deacetylation	5	1.80E-01	8.30E-01
	GOTERM_MF_FAT	deacetylase activity	5	1.90E-01	9.00E-01
	GOTERM_BP_FAT	chromatin silencing	5	2.10E-01	8.70E-01
	COG_ONTOLOGY	Chromatin structure and dynamics / Secondary metabolites biosynthesis, transport, and catabolism	3	2.10E-01	9.50E-01
	GOTERM_BP_FAT	negative regulation of gene expression, epigenetic	5	3.00E-01	9.30E-01
	UP_SEQ_FEATURE	region of interest:Histone deacetylase	3	3.50E-01	1.00E+00
	INTERPRO	Histone deacetylase superfamily	3	4.10E-01	1.00E+00
	GOTERM_MF_FAT	hydrolase activity, acting on carbon-nitrogen (but not peptide) bonds, in linear amides	7	7.70E-01	1.00E+00
**Annotation Cluster 193**	**Enrichment Score: 0.63**		**Count**	**P_Value**	**Benjamini**
	GOTERM_CC_FAT	vacuolar part	13	1.40E-01	5.70E-01
	GOTERM_CC_FAT	lysosomal membrane	6	2.90E-01	7.80E-01
	GOTERM_CC_FAT	vacuolar membrane	10	3.30E-01	8.10E-01
**Annotation Cluster 194**	**Enrichment Score: 0.62**		**Count**	**P_Value**	**Benjamini**
	BIOCARTA	Activation of Csk by cAMP-dependent Protein Kinase Inhibits Signaling through the T Cell Receptor	8	3.10E-02	9.40E-01
	BIOCARTA	Lck and Fyn tyrosine kinases in initiation of TCR Activation	6	4.40E-02	9.10E-01
	KEGG_PATHWAY	Asthma	8	1.30E-01	4.90E-01
	BIOCARTA	B Lymphocyte Cell Surface Molecules	5	1.50E-01	9.30E-01
	BIOCARTA	Bystander B Cell Activation	4	1.90E-01	9.30E-01
	BIOCARTA	Antigen Dependent B Cell Activation	4	4.20E-01	9.70E-01
	BIOCARTA	The Co-Stimulatory Signal During T-cell Activation	5	5.20E-01	9.80E-01

ILMN_1726967	TWSG1	-0.47004142
ILMN_1810107	C17orf74	-0.470054119
ILMN_1691181	TMX1	-0.470125971
ILMN_1800543	SCFD1	-0.470167777
ILMN_1688240	PHOSPHO1	-0.470177798
ILMN_1692762	NA	-0.470233624
ILMN_1670245	KLF3	-0.470269252
ILMN_3269956	NA	-0.470305978
ILMN_1726701	BC038536	-0.470319584
ILMN_1707585	SLC46A2	-0.470393928
ILMN_1881598	AW451118	-0.47039514
ILMN_1837271	CA449813	-0.470425997
ILMN_1746428	RGPD1	-0.470431857
ILMN_1745949	CEACAM21	-0.470562241
ILMN_1671890	UBTFL1	-0.470572197
ILMN_1787794	BMF	-0.470589425
ILMN_1885728	KIAA1147	-0.470634556
ILMN_1870923	AI125404	-0.470648949
ILMN_3304072	PRG4	-0.470650171
ILMN_1833175	CD242195	-0.470660412
ILMN_1914874	KRTAP25-1	-0.470678994
ILMN_1808163	C11orf24	-0.47071481
ILMN_1662413	HGSNAT	-0.470745829
ILMN_1696183	HBQ1	-0.470804271
ILMN_1671547	HMGA2	-0.470814597
ILMN_1736546	SLC16A14	-0.470840134
ILMN_1714280	FOXL2	-0.47088854
ILMN_1695181	SCRIB	-0.47089567
ILMN_1772214	CRYBA1	-0.470958407
ILMN_1861915	CA392115	-0.470972771
ILMN_2145781	RBMY2FP	-0.471052682
ILMN_1734552	LRRC20	-0.471073155
ILMN_1723141	OTUD1	-0.471203258
ILMN_1704374	BC043355	-0.471243422
ILMN_1822408	BU736534	-0.471280693
ILMN_2360047	MAGEA11	-0.47131226
ILMN_1760222	C14orf144	-0.471452385
ILMN_1803073	DNAJC12	-0.471564801
ILMN_1654340	NA	-0.471582102
ILMN_3239367	HYDIN	-0.471593006
ILMN_2229379	KIT	-0.471602468
ILMN_1751072	SRPRB	-0.471618649
ILMN_2203073	RNF151	-0.471660458
ILMN_1776337	CHORDC1	-0.471673192
ILMN_1711289	GYS1	-0.47170598
ILMN_2072622	LOC100288413	-0.471747066
ILMN_1750902	LYZL6	-0.471768405
ILMN_1867214	LOC400654	-0.471772912
ILMN_1888373	AI859116	-0.471795519
ILMN_1748719	SEC16B	-0.471795641
ILMN_1703703	AB231702	-0.471879148
ILMN_1784707	LOC644277	-0.471892068
ILMN_1681930	TMEM232	-0.471896475
ILMN_1661833	ANKRD12	-0.471917578
ILMN_1725312	RASA1	-0.471934084
ILMN_2081883	IQCK	-0.471976033
ILMN_1684494	BX248300	-0.472119338
ILMN_1784428	MGC57346	-0.472267522
ILMN_1775587	STX19	-0.472268977
ILMN_2071826	RNF152	-0.472339409
ILMN_1776112	SLC10A1	-0.472349113
ILMN_1916313	BX111153	-0.472353236
ILMN_1657790	SRSF11	-0.47235351
ILMN_1849418	AW661821	-0.472416738
ILMN_1864558	BC034632	-0.472456835
ILMN_1895935	CB242622	-0.47250937
ILMN_1668463	SON	-0.472526275
ILMN_1707312	NFIL3	-0.472577389
ILMN_2360988	SEMA6B	-0.472582451
ILMN_3240108	SNORD90	-0.472643537
ILMN_1791186	ARHGAP18	-0.472646883
ILMN_1801405	ROPN1B	-0.472689156
ILMN_2397230	USP16	-0.472716629
ILMN_1725594	FAM188A	-0.472720506
ILMN_1769104	ZCCHC10	-0.472740977
ILMN_1657873	XPO4	-0.472861561
ILMN_1886117	AI187771	-0.472952411
ILMN_2379110	WDR16	-0.472991808
ILMN_1790706	OR10C1	-0.473013376
ILMN_1676490	MIP	-0.473402353
ILMN_1881561	AI204073	-0.473448414
ILMN_2117526	DMGDH	-0.473499684
ILMN_1742534	COL4A5	-0.473529648
ILMN_2322458	OPA1	-0.473550389
ILMN_1676846	ABCE1	-0.473646518
ILMN_1685079	TELO2	-0.473665111
ILMN_1840469	BE217788	-0.473704533
ILMN_1705035	FBXL7	-0.473705225
ILMN_1695972	CCDC89	-0.473725903
ILMN_1746788	TRIM10	-0.473751454
ILMN_1847612	AA554377	-0.473759238
ILMN_1797219	CLCA1	-0.47377604
ILMN_1716680	GRM7	-0.473892278
ILMN_1782045	FKBP4	-0.473919172

ILMN_1675038	PRMT2	0.640461759
ILMN_2311989	CUTA	0.640418616
ILMN_1812721	HIP1R	0.640279458
ILMN_1789775	WDR74	0.640277022
ILMN_1680104	SLC35C1	0.640264772
ILMN_1810560	NUPR1	0.640214158
ILMN_1739946	VKORC1	0.640204728
ILMN_1701621	TYMP	0.640118228
ILMN_1689160	DPEP2	0.640066393
ILMN_3297945	UBA1	0.640064819
ILMN_3305938	SGK1	0.640042001
ILMN_1801383	SMG1	0.639796237
ILMN_1715169	HLA-DRB1	0.639780211
ILMN_2399264	sept-06	0.639680881
ILMN_1676036	TUBBP5	0.639590345
ILMN_3234841	RPL23AP7	0.639342661
ILMN_1667222	MTX1	0.639324629
ILMN_1696911	FTH1	0.63928477
ILMN_1660292	MRPS21	0.63924818
ILMN_1747204	HTRA2	0.639062979
ILMN_1761566	C5orf32	0.639011137
ILMN_1651819	GALNT11	0.638855523
ILMN_1807283	NDST1	0.638844663
ILMN_1794522	EIF5A	0.638830659
ILMN_1681356	PDE2A	0.638809858
ILMN_1745533	FAM117A	0.638766839
ILMN_1763842	PTRH1	0.638691536
ILMN_3237636	C16orf67	0.638690652
ILMN_1800420	RNF214	0.638631004
ILMN_1667716	TMEM101	0.63860242
ILMN_2302358	ABCC5	0.638320677
ILMN_1659024	TMCC2	0.638141021
ILMN_2386354	CSNK2A1	0.638134384
ILMN_1685928	WDR34	0.638101024
ILMN_1718129	MAP2K5	0.638005281
ILMN_3214532	RPL21	0.637949219
ILMN_1713749	CORO1A	0.637824404
ILMN_1738420	TMEM201	0.637608654
ILMN_1725414	ABCC6	0.637555032
ILMN_1710434	TBC1D10C	0.63752807
ILMN_1767006	PSMB8	0.637496823
ILMN_2331205	CHKB	0.637166824
ILMN_1717078	DNAJC16	0.637107692
ILMN_2161746	TRIP10	0.637068321
ILMN_1671295	MST151	0.63697052
ILMN_2342630	LPHN1	0.636968514
ILMN_1796244	CD2BP2	0.636915292
ILMN_1683082	RPUSD1	0.636882451
ILMN_1710571	PAPD5	0.636868402
ILMN_2395285	SNRNP35	0.636796035
ILMN_1736575	TRIM28	0.63665085
ILMN_1796762	CCDC102A	0.636502033
ILMN_1680091	POP7	0.63649984
ILMN_2178775	ZNF585B	0.636432
ILMN_1677237	CHCHD2	0.636380577
ILMN_1707783	CCDC72	0.636344863
ILMN_2354649	SRSF10	0.636288732
ILMN_1703538	AIF1	0.636287556
ILMN_1751958	NSUN5	0.636128946
ILMN_1876778	AI819186	0.636125063
ILMN_1670096	NRBP1	0.636123175
ILMN_1661888	MEF2A	0.635826579
ILMN_2354617	DYRK1B	0.635806682
ILMN_1756898	COQ9	0.635770368
ILMN_1736863	TMEM140	0.635741966
ILMN_1729433	LIPH	0.635610849
ILMN_1664541	POLR2J4	0.635376507
ILMN_2211800	HMGB1	0.635340699
ILMN_1709795	RAC2	0.635233284
ILMN_3236021	ANKRD36	0.635108669
ILMN_2386016	C2orf28	0.635089333
ILMN_3236752	COAS3	0.635076305
ILMN_2396991	HCST	0.635041369
ILMN_1651776	FHOD1	0.63491931
ILMN_1813682	MRPL53	0.634909454
ILMN_1767377	SMA4	0.634905945
ILMN_1666409	PSMB6	0.634878999
ILMN_1718771	CCDC24	0.634843087
ILMN_1777725	LSM14B	0.634811991
ILMN_1682960	DTNBP1	0.634762046
ILMN_2361807	OS9	0.634706209
ILMN_1712708	TRIM47	0.634688648
ILMN_1769473	SETD2	0.634566993
ILMN_2099487	C17orf108	0.634399624
ILMN_2410713	FGFR4	0.634056766
ILMN_3301749	SPNS2	0.63392257
ILMN_1695356	RNF166	0.633745788
ILMN_1762312	FOXRED1	0.633738443
ILMN_1790625	CBX3	0.633642355
ILMN_3176040	RPS27A	0.63363912
ILMN_3273456	PABPC4	0.633604509
ILMN_1722491	APRT	0.633568265
ILMN_1761996	SRSF5	0.633472161
ILMN_1740298	SIGLEC5	0.633340891
ILMN_2413779	SEZ6L2	0.633328612

	BIOCARTA	IL 5 Signaling Pathway	3	6.00E-01	9.90E-01
	BIOCARTA	Th1/Th2 Differentiation	3	9.20E-01	1.00E+00
	BIOCARTA	Cytokines and Inflammatory Response	3	9.70E-01	1.00E+00
**Annotation Cluster 195**	**Enrichment Score: 0.62**		**Count**	**P_Value**	**Benjamini**
	GOTERM_BP_FAT	DNA damage response, signal transduction by p53 class mediator resulting in transcription of p21 class mediator	3	2.30E-01	8.90E-01
	GOTERM_BP_FAT	DNA damage response, signal transduction resulting in transcription	3	2.30E-01	8.90E-01
	GOTERM_BP_FAT	DNA damage response, signal transduction by p53 class mediator	6	2.50E-01	9.10E-01
**Annotation Cluster 196**	**Enrichment Score: 0.62**		**Count**	**P_Value**	**Benjamini**
	GOTERM_MF_FAT	fructose binding	3	1.70E-01	8.90E-01
	GOTERM_BP_FAT	fructose metabolic process	5	1.80E-01	8.30E-01
	GOTERM_MF_FAT	phosphofructokinase activity	3	2.20E-01	9.30E-01
	KEGG_PATHWAY	Fructose and mannose metabolism	8	2.40E-01	5.90E-01
	GOTERM_MF_FAT	carbohydrate kinase activity	4	4.80E-01	9.90E-01
**Annotation Cluster 197**	**Enrichment Score: 0.61**		**Count**	**P_Value**	**Benjamini**
	GOTERM_BP_FAT	response to lipopolysaccharide	15	1.30E-01	7.60E-01
	GOTERM_BP_FAT	response to molecule of bacterial origin	16	1.60E-01	8.00E-01
	GOTERM_BP_FAT	response to bacterium	24	7.30E-01	1.00E+00
**Annotation Cluster 198**	**Enrichment Score: 0.6**		**Count**	**P_Value**	**Benjamini**
	GOTERM_CC_FAT	stress fiber	6	2.10E-01	6.80E-01
	GOTERM_CC_FAT	actin filament bundle	6	2.60E-01	7.50E-01
	GOTERM_CC_FAT	actomyosin	6	2.90E-01	7.80E-01
**Annotation Cluster 199**	**Enrichment Score: 0.6**		**Count**	**P_Value**	**Benjamini**
	GOTERM_MF_FAT	insulin-like growth factor binding	7	9.20E-02	7.60E-01
	SP_PIR_KEYWORDS	Growth factor binding	5	1.20E-01	4.80E-01
	INTERPRO	Insulin-like growth factor binding protein	3	1.70E-01	9.90E-01
	UP_SEQ_FEATURE	domain:Thyroglobulin type-1	4	2.00E-01	1.00E+00
	UP_SEQ_FEATURE	domain:IGFBP N-terminal	5	2.20E-01	1.00E+00
	INTERPRO	Insulin-like growth factor-binding protein, IGFBP	5	2.40E-01	1.00E+00
	SMART	IB	5	2.70E-01	9.30E-01
	INTERPRO	Thyroglobulin type-1	4	3.60E-01	1.00E+00
	SMART	TY	4	3.90E-01	9.60E-01
	INTERPRO	Insulin-like growth factor binding protein, N-terminal	3	4.60E-01	1.00E+00
	BIOCARTA	Ghrelin	3	7.50E-01	1.00E+00
**Annotation Cluster 200**	**Enrichment Score: 0.6**		**Count**	**P_Value**	**Benjamini**
	GOTERM_BP_FAT	cell motion	73	1.10E-01	7.10E-01
	GOTERM_BP_FAT	cell migration	41	2.80E-01	9.30E-01
	GOTERM_BP_FAT	localization of cell	44	3.60E-01	9.60E-01
	GOTERM_BP_FAT	cell motility	44	3.60E-01	9.60E-01
**Annotation Cluster 201**	**Enrichment Score: 0.6**		**Count**	**P_Value**	**Benjamini**
	SP_PIR_KEYWORDS	antiport	8	1.40E-01	5.20E-01
	GOTERM_MF_FAT	solute:hydrogen antiporter activity	6	1.60E-01	8.80E-01
	GOTERM_MF_FAT	solute:cation antiporter activity	7	1.80E-01	8.90E-01
	GOTERM_MF_FAT	antiporter activity	12	2.50E-01	9.50E-01
	INTERPRO	Cation/H+ exchanger	4	2.60E-01	1.00E+00
	GOTERM_MF_FAT	solute:solute antiporter activity	9	3.90E-01	9.80E-01
	GOTERM_MF_FAT	cation:cation antiporter activity	3	6.60E-01	1.00E+00
**Annotation Cluster 202**	**Enrichment Score: 0.59**		**Count**	**P_Value**	**Benjamini**
	SP_PIR_KEYWORDS	igg-binding protein	3	2.00E-01	6.30E-01
	GOTERM_MF_FAT	IgG binding	3	2.70E-01	9.60E-01
	GOTERM_MF_FAT	immunoglobulin binding	4	3.00E-01	9.60E-01
**Annotation Cluster 203**	**Enrichment Score: 0.59**		**Count**	**P_Value**	**Benjamini**
	UP_SEQ_FEATURE	zinc finger region:C3H1-type 2	7	1.50E-01	1.00E+00
	UP_SEQ_FEATURE	zinc finger region:C3H1-type 1	7	1.50E-01	1.00E+00
	UP_SEQ_FEATURE	zinc finger region:C3H1-type 3	5	2.20E-01	1.00E+00
	INTERPRO	Zinc finger, CCCH-type	9	3.30E-01	1.00E+00
	SMART	ZnF_C3H1	9	3.80E-01	9.70E-01
	UP_SEQ_FEATURE	zinc finger region:C3H1-type 4	3	4.80E-01	1.00E+00
**Annotation Cluster 204**	**Enrichment Score: 0.59**		**Count**	**P_Value**	**Benjamini**
	KEGG_PATHWAY	Endometrial cancer	13	8.00E-02	3.70E-01
	KEGG_PATHWAY	Acute myeloid leukemia	14	8.50E-02	3.80E-01
	KEGG_PATHWAY	Non-small cell lung cancer	12	1.80E-01	5.50E-01
	KEGG_PATHWAY	ErbB signaling pathway	17	2.20E-01	5.90E-01
	KEGG_PATHWAY	Chronic myeloid leukemia	15	2.30E-01	5.80E-01
	KEGG_PATHWAY	Glioma	13	2.30E-01	5.80E-01
	KEGG_PATHWAY	Prostate cancer	14	5.90E-01	8.40E-01
	BIOCARTA	Trefoil Factors Initiate Mucosal Healing	5	6.40E-01	9.90E-01
	KEGG_PATHWAY	Melanoma	7	9.70E-01	9.90E-01
**Annotation Cluster 205**	**Enrichment Score: 0.58**		**Count**	**P_Value**	**Benjamini**
	GOTERM_MF_FAT	tumor necrosis factor receptor superfamily binding	9	3.80E-02	5.30E-01
	GOTERM_MF_FAT	tumor necrosis factor receptor binding	5	2.80E-01	9.60E-01
	INTERPRO	Tumor Necrosis Factor	3	6.50E-01	1.00E+00
	SMART	TNF	3	6.80E-01	9.90E-01
**Annotation Cluster 206**	**Enrichment Score: 0.58**		**Count**	**P_Value**	**Benjamini**
	SP_PIR_KEYWORDS	heterotrimer	9	1.50E-02	1.30E-01
	SP_PIR_KEYWORDS	basement membrane	9	9.00E-02	4.10E-01
	UP_SEQ_FEATURE	domain:Laminin G-like 2	7	1.30E-01	1.00E+00
	UP_SEQ_FEATURE	domain:Laminin G-like 1	7	1.30E-01	1.00E+00
	SP_PIR_KEYWORDS	laminin egf-like domain	7	1.40E-01	5.40E-01
	INTERPRO	Concanavalin A-like lectin/glucanase, subgroup	14	1.70E-01	9.90E-01
	UP_SEQ_FEATURE	domain:Laminin EGF-like 1	6	2.00E-01	1.00E+00
	UP_SEQ_FEATURE	domain:Laminin EGF-like 12	3	2.00E-01	1.00E+00
	UP_SEQ_FEATURE	domain:Laminin EGF-like 13	3	2.00E-01	1.00E+00
	UP_SEQ_FEATURE	domain:Laminin G-like 3	5	2.20E-01	1.00E+00
	INTERPRO	Laminin G, subdomain 1	3	2.20E-01	1.00E+00
	UP_SEQ_FEATURE	domain:Laminin EGF-like 2	6	2.20E-01	1.00E+00
	UP_SEQ_FEATURE	domain:Laminin G-like 4	4	2.70E-01	1.00E+00
	UP_SEQ_FEATURE	domain:Laminin G-like 5	3	3.00E-01	1.00E+00
	UP_SEQ_FEATURE	domain:Laminin EGF-like 11	3	3.00E-01	1.00E+00
	UP_SEQ_FEATURE	domain:Laminin N-terminal	4	3.00E-01	1.00E+00
	INTERPRO	Laminin, N-terminal	4	3.30E-01	1.00E+00
	INTERPRO	Laminin G	8	3.40E-01	1.00E+00
	GOTERM_CC_FAT	laminin complex	3	3.40E-01	8.20E-01
	UP_SEQ_FEATURE	domain:Laminin EGF-like 9	3	3.50E-01	1.00E+00
	SMART	LamNT	4	3.60E-01	9.60E-01
	SMART	LamG	8	3.80E-01	9.70E-01

ILMN_1735792	RHBDF2	-0.47393291
ILMN_1805720	SDHAF1	-0.474196797
ILMN_1690276	ZNF354C	-0.474207661
ILMN_1718107	MAGEA12	-0.474373924
ILMN_1671997	LOC388428	-0.474382216
ILMN_1851906	AW665857	-0.474389706
ILMN_3200421	GCSH	-0.474403659
ILMN_1719083	OR56A3	-0.474481903
ILMN_1801491	SLC26A2	-0.474556968
ILMN_1797204	WDR64	-0.474652961
ILMN_1813620	PARP6	-0.474684817
ILMN_1883255	AA470148	-0.474688734
ILMN_1810945	CYP2F1	-0.474711759
ILMN_2399431	PRPS2	-0.474736164
ILMN_1683817	UBE2Q2	-0.474750651
ILMN_2263718	SPAG9	-0.474783317
ILMN_1887177	ODC1	-0.474880378
ILMN_1804277	SPRED1	-0.474888007
ILMN_1663518	FAM153A	-0.47494422
ILMN_1701933	SNCA	-0.474954669
ILMN_1709768	LOC100128818	-0.47502575
ILMN_1709937	KCNN4	-0.475037004
ILMN_1767433	TBC1D5	-0.475071976
ILMN_1816395	CR736179	-0.475104769
ILMN_1675221	DGKZ	-0.47511494
ILMN_1687315	RXRA	-0.475209409
ILMN_1735195	PRAMEF10	-0.475231117
ILMN_1681053	LOC100124692	-0.475268856
ILMN_2330112	CNIH	-0.47527494
ILMN_2262901	RUFY3	-0.475300143
ILMN_3194755	BE780475	-0.475309642
ILMN_1666617	C6orf155	-0.475310114
ILMN_1785795	METAP1	-0.475392807
ILMN_1793201	HAGHL	-0.475488259
ILMN_1776266	CRYGB	-0.475523349
ILMN_1720113	PTPRO	-0.47554397
ILMN_2295511	LUC7L	-0.475560967
ILMN_1710873	ZNF330	-0.475584244
ILMN_1653504	S1PR1	-0.475612704
ILMN_3206537	UBTFL1	-0.475718774
ILMN_1730019	BRD1	-0.475786939
ILMN_1656779	FAM75D3	-0.475792148
ILMN_2193752	MBLAC2	-0.47596555
ILMN_1785821	TDP2	-0.475976199
ILMN_1677085	RGS19	-0.476120237
ILMN_1670681	PHOX2A	-0.476371349
ILMN_3262976	FLJ46446	-0.476391619
ILMN_2250830	SPDYE1	-0.476483911
ILMN_1744539	GOLGA6L6	-0.476493316
ILMN_2205767	CTXN3	-0.476494973
ILMN_1665945	ACBD3	-0.476524865
ILMN_1713397	NCCRP1	-0.476528434
ILMN_1801441	RFTN2	-0.476533485
ILMN_1681437	DCXR	-0.476610378
ILMN_1811048	GPR107	-0.476657237
ILMN_1769720	STAU2	-0.4766948
ILMN_1755027	NLRP8	-0.4768509
ILMN_1911487	BX108993	-0.476867295
ILMN_1692396	CXorf58	-0.476905076
ILMN_1733298	C1orf158	-0.476910465
ILMN_2344047	ZNF398	-0.476938799
ILMN_1787115	WWTR1	-0.477014359
ILMN_2311548	PTRH2	-0.477063219
ILMN_2393461	DDO	-0.477082285
ILMN_1699610	CCT6B	-0.47708757
ILMN_3309759	VTRNA1-1	-0.477105043
ILMN_1813800	NUFIP1	-0.47711128
ILMN_1772278	TANC1	-0.477124925
ILMN_1756408	PARVA	-0.477168408
ILMN_1656492	NA	-0.477174613
ILMN_1875763	AI290919	-0.477198607
ILMN_3245038	AQP7	-0.477305008
ILMN_1787591	XPA	-0.477328145
ILMN_2359789	RAC1	-0.477341172
ILMN_1739086	SNORD43	-0.477407907
ILMN_1712945	DYNC2LI1	-0.477443501
ILMN_1666718	NA	-0.47750202
ILMN_1732269	GP6	-0.477531298
ILMN_3211615	FAM157B	-0.477705882
ILMN_1840294	D59687	-0.477751754
ILMN_3207821	FAM159B	-0.477751758
ILMN_1703074	CPD	-0.47792571
ILMN_1889752	LOC221710	-0.477944959
ILMN_2347943	ZNF384	-0.478026335
ILMN_1693891	MOGAT2	-0.478043788
ILMN_1746618	PAQR7	-0.478046776
ILMN_1827283	BX104024	-0.478067893
ILMN_1882041	H16080	-0.478091843
ILMN_1722906	SPATC1	-0.478211662
ILMN_1770725	AIF1L	-0.478256916
ILMN_2374692	WAC	-0.478357269
ILMN_3227782	MARVELD1	-0.478385219
ILMN_3271872	BX117604	-0.478402908
ILMN_1911721	AK090694	-0.478485801
ILMN_1661021	AK125823	-0.478522204

ILMN_1757604	TPM2	0.633319315
ILMN_1711566	TIMP1	0.633212006
ILMN_2408645	SPCS2	0.633097681
ILMN_2260313	CRYBA2	0.632745288
ILMN_1670948	TSR2	0.632711628
ILMN_1732717	FAM71E1	0.632684355
ILMN_3307950	CDK5RAP3	0.632539428
ILMN_1805737	PFKP	0.632511352
ILMN_1782741	CD300LB	0.632347748
ILMN_1802743	MLL2	0.632287022
ILMN_1745573	TTC13	0.63219832
ILMN_1745787	POLL	0.632127813
ILMN_2059211	KIAA0195	0.63206416
ILMN_1670844	OPN3	0.632029846
ILMN_1794333	POU2F1	0.632022883
ILMN_2088124	TMEM154	0.632022516
ILMN_1747622	CD33	0.631911285
ILMN_1717737	FKBP7	0.631692684
ILMN_1780825	RRAS	0.631667707
ILMN_1657283	ALKBH5	0.631644826
ILMN_1667408	EPB41	0.631582362
ILMN_1797384	UROS	0.631518903
ILMN_1805665	FLRT3	0.631500397
ILMN_1716651	RUNX2	0.631329228
ILMN_1775999	ATAT1	0.631328563
ILMN_2205050	PRKX	0.631309903
ILMN_1696035	ERP29	0.631292837
ILMN_3242485	TLK2	0.631292722
ILMN_2318811	RANBP3	0.63121831
ILMN_3209399	KRT18	0.631169044
ILMN_1690682	B3GALT4	0.631008398
ILMN_1665583	TUBB	0.630842464
ILMN_1789642	DNAJC5	0.630812919
ILMN_1748093	PAFAH1B3	0.630735107
ILMN_1656826	SH3RF1	0.630601495
ILMN_1679655	WDR82	0.63057194
ILMN_1808435	OGT	0.630433502
ILMN_1775224	NOS3	0.630409413
ILMN_1805512	WDR13	0.630297603
ILMN_1799387	INO80	0.630285592
ILMN_1810559	RHOQ	0.63016679
ILMN_3225784	LAMR1P15	0.630147187
ILMN_2160476	CCL22	0.630108488
ILMN_1784651	NAGA	0.629970984
ILMN_1686254	FAM127B	0.629889324
ILMN_1708375	IRF1	0.629661595
ILMN_1700031	PRAME	0.629475057
ILMN_1796316	MMP9	0.629456365
ILMN_3266666	NSA2	0.629425867
ILMN_1753524	HIST1H2AB	0.629366542
ILMN_3240793	OR2A4	0.629280973
ILMN_1770388	VPS39	0.629272951
ILMN_1760779	ENSA	0.629116444
ILMN_1728199	POLE	0.629090728
ILMN_1664644	ATG16L2	0.628866171
ILMN_1713605	RPAP1	0.628861016
ILMN_2046856	RPS15	0.628699157
ILMN_1765459	S100A13	0.628694702
ILMN_2141398	VEZT	0.628692364
ILMN_1802203	ARF1	0.628646144
ILMN_1674034	H2AFY	0.628565156
ILMN_3273198	NA	0.628544975
ILMN_2404049	RBM38	0.628471469
ILMN_1748438	POLR2G	0.628465046
ILMN_1692539	SH3BP1	0.628403344
ILMN_2280568	ANKRD36	0.628341658
ILMN_1783606	MLL5	0.628214752
ILMN_1762316	CPSF3L	0.628090266
ILMN_3228585	SLC35E2B	0.628037558
ILMN_1659845	KIAA0355	0.627971069
ILMN_3262031	BRD7	0.627869224
ILMN_2138435	MRPS27	0.627859046
ILMN_1694731	CLCN7	0.62756474
ILMN_1732151	COL6A1	0.627479873
ILMN_1676302	FAM113A	0.627434723
ILMN_2373062	RHBDF2	0.627426734
ILMN_2352190	CLIP2	0.627369784
ILMN_1719835	OSCP1	0.627332289
ILMN_1783712	C16orf88	0.627149775
ILMN_1698015	TBX1	0.627111249
ILMN_3211302	RPL17	0.627052608
ILMN_2347693	NCOA3	0.627025326
ILMN_1709486	SRPX	0.626789186
ILMN_1659792	HOXD9	0.626687339
ILMN_1661138	GON4L	0.626296389
ILMN_1673649	HYOU1	0.626285491
ILMN_1663618	STAT3	0.62627495
ILMN_1710524	PARD3	0.62626597
ILMN_2075334	HIST1H4C	0.6261659
ILMN_2377733	CASP8	0.626149099
ILMN_2334210	ITGB4	0.626009906
ILMN_1811754	NDUFB10	0.626009762
ILMN_2155480	RAB43	0.62586857
ILMN_1740170	CHCHD10	0.625863693

	UP_SEQ_FEATURE	domain:Laminin EGF-like 8	3	3.90E-01	1.00E+00
	UP_SEQ_FEATURE	domain:Laminin EGF-like 10	3	3.90E-01	1.00E+00
	GOTERM_CC_FAT	basal lamina	4	4.00E-01	8.60E-01
	INTERPRO	EGF-like, laminin	7	4.20E-01	1.00E+00
	UP_SEQ_FEATURE	domain:Laminin EGF-like 7	3	4.40E-01	1.00E+00
	INTERPRO	Laminin G, subdomain 2	7	4.40E-01	1.00E+00
	SMART	EGF_Lam	7	4.60E-01	9.70E-01
	GOTERM_CC_FAT	basement membrane	12	4.70E-01	9.00E-01
	UP_SEQ_FEATURE	domain:Laminin EGF-like 4	3	4.80E-01	1.00E+00
	UP_SEQ_FEATURE	domain:Laminin EGF-like 6	3	4.80E-01	1.00E+00
	UP_SEQ_FEATURE	domain:Laminin EGF-like 3	4	5.10E-01	1.00E+00
**Annotation Cluster 207**	**Enrichment Score: 0.58**		**Count**	**P_Value**	**Benjamini**
	BIOCARTA	Role of Erk5 in Neuronal Survival	7	2.50E-02	9.70E-01
	BIOCARTA	TPO Signaling Pathway	8	7.30E-02	9.40E-01
	BIOCARTA	Erk1/Erk2 Mapk Signaling pathway	10	9.00E-02	9.50E-01
	BIOCARTA	Nerve growth factor pathway (NGF)	7	9.20E-02	9.20E-01
	BIOCARTA	PDGF Signaling Pathway	9	9.20E-02	9.00E-01
	BIOCARTA	Role of ERBB2 in Signal Transduction and Oncology	8	9.30E-02	8.80E-01
	BIOCARTA	T Cell Receptor Signaling Pathway	11	1.00E-01	8.90E-01
	BIOCARTA	IGF-1 Signaling Pathway	7	1.40E-01	9.30E-01
	BIOCARTA	IL 6 signaling pathway	7	1.40E-01	9.30E-01
	BIOCARTA	Fc Epsilon Receptor I Signaling in Mast Cells	9	2.10E-01	9.30E-01
	BIOCARTA	EGF Signaling Pathway	8	2.20E-01	9.40E-01
	BIOCARTA	BCR Signaling Pathway	8	2.20E-01	9.40E-01
	BIOCARTA	EPO Signaling Pathway	6	2.60E-01	9.40E-01
	BIOCARTA	Inhibition of Cellular Proliferation by Gleevec	6	3.00E-01	9.60E-01
	BIOCARTA	Insulin Signaling Pathway	6	3.00E-01	9.60E-01
	BIOCARTA	Role of MAL in Rho-Mediated Activation of SRF	6	3.00E-01	9.60E-01
	BIOCARTA	Signaling of Hepatocyte Growth Factor Receptor	9	3.30E-01	9.50E-01
	BIOCARTA	Trka Receptor Signaling Pathway	4	3.60E-01	9.60E-01
	BIOCARTA	Growth Hormone Signaling Pathway	6	4.30E-01	9.70E-01
	BIOCARTA	Sprouty regulation of tyrosine kinase signals	4	5.80E-01	9.90E-01
	BIOCARTA	IL 2 signaling pathway	5	6.00E-01	9.90E-01
	BIOCARTA	Angiotensin II mediated activation of JNK Pathway via Pyk2 dependent signaling	6	6.20E-01	9.90E-01
	BIOCARTA	Links between Pyk2 and Map Kinases	5	7.70E-01	1.00E+00
	BIOCARTA	IL 3 signaling pathway	3	7.90E-01	1.00E+00
	BIOCARTA	Signaling Pathway from G-Protein Families	4	8.00E-01	1.00E+00
	BIOCARTA	Integrin Signaling Pathway	6	8.20E-01	1.00E+00
	BIOCARTA	Bioactive Peptide Induced Signaling Pathway	5	8.60E-01	1.00E+00
	BIOCARTA	fMLP induced chemokine gene expression in HMC-1 cells	4	8.90E-01	1.00E+00
**Annotation Cluster 208**	**Enrichment Score: 0.58**		**Count**	**P_Value**	**Benjamini**
	GOTERM_BP_FAT	actin filament-based movement	6	1.80E-01	8.30E-01
	GOTERM_MF_FAT	actin-dependent ATPase activity	3	2.70E-01	9.60E-01
	GOTERM_BP_FAT	cytoskeleton-dependent intracellular transport	9	3.80E-01	9.60E-01
**Annotation Cluster 209**	**Enrichment Score: 0.58**		**Count**	**P_Value**	**Benjamini**
	GOTERM_BP_FAT	negative regulation of phosphatase activity	3	8.70E-02	6.40E-01
	GOTERM_BP_FAT	regulation of dephosphorylation	6	2.50E-01	9.10E-01
	GOTERM_BP_FAT	regulation of phosphatase activity	4	4.30E-01	9.80E-01
	GOTERM_BP_FAT	regulation of phosphoprotein phosphatase activity	3	5.30E-01	9.90E-01
**Annotation Cluster 210**	**Enrichment Score: 0.58**		**Count**	**P_Value**	**Benjamini**
	GOTERM_BP_FAT	regulation of interleukin-2 production	9	3.60E-02	4.20E-01
	GOTERM_BP_FAT	regulation of interleukin-2 biosynthetic process	5	2.40E-01	8.90E-01
	GOTERM_BP_FAT	positive regulation of cytokine biosynthetic process	8	4.50E-01	9.80E-01
	GOTERM_BP_FAT	positive regulation of interleukin-2 biosynthetic process	3	5.30E-01	9.90E-01
	GOTERM_BP_FAT	regulation of cytokine biosynthetic process	10	6.50E-01	1.00E+00
**Annotation Cluster 211**	**Enrichment Score: 0.57**		**Count**	**P_Value**	**Benjamini**
	GOTERM_BP_FAT	UV protection	5	4.60E-02	4.80E-01
	GOTERM_BP_FAT	nucleotide-excision repair, DNA incision	3	1.80E-01	8.40E-01
	SP_PIR_KEYWORDS	xeroderma pigmentosum	3	2.50E-01	6.90E-01
	GOTERM_BP_FAT	response to UV	10	3.70E-01	9.60E-01
	GOTERM_BP_FAT	nucleotide-excision repair	9	4.40E-01	9.80E-01
	KEGG_PATHWAY	Nucleotide excision repair	8	5.00E-01	8.10E-01
	GOTERM_BP_FAT	nucleotide-excision repair, DNA damage removal	4	5.70E-01	9.90E-01
**Annotation Cluster 212**	**Enrichment Score: 0.57**		**Count**	**P_Value**	**Benjamini**
	UP_SEQ_FEATURE	domain:FCP1 homology	3	2.50E-01	1.00E+00
	INTERPRO	NLI interacting factor	3	2.70E-01	1.00E+00
	SMART	CPDc	3	2.90E-01	9.40E-01
**Annotation Cluster 213**	**Enrichment Score: 0.57**		**Count**	**P_Value**	**Benjamini**
	KEGG_PATHWAY	Dilated cardiomyopathy	19	1.40E-01	4.90E-01
	KEGG_PATHWAY	Arrhythmogenic right ventricular cardiomyopathy (ARVC)	14	3.50E-01	7.00E-01
	KEGG_PATHWAY	Hypertrophic cardiomyopathy (HCM)	15	4.00E-01	7.40E-01
**Annotation Cluster 214**	**Enrichment Score: 0.57**		**Count**	**P_Value**	**Benjamini**
	GOTERM_BP_FAT	regulation of specific transcription from RNA polymerase II promoter	17	1.70E-01	8.20E-01
	GOTERM_BP_FAT	positive regulation of specific transcription from RNA polymerase II promoter	11	2.10E-01	8.70E-01
	GOTERM_BP_FAT	positive regulation of gene-specific transcription	15	2.50E-01	9.10E-01
	GOTERM_BP_FAT	negative regulation of specific transcription from RNA polymerase II promoter	8	2.90E-01	9.30E-01
	GOTERM_BP_FAT	regulation of gene-specific transcription	21	3.10E-01	9.40E-01
	GOTERM_BP_FAT	negative regulation of gene-specific transcription	8	4.50E-01	9.80E-01
**Annotation Cluster 215**	**Enrichment Score: 0.57**		**Count**	**P_Value**	**Benjamini**
	GOTERM_BP_FAT	regulation of axonogenesis	14	3.20E-02	3.90E-01
	GOTERM_BP_FAT	negative regulation of cell projection organization	6	3.10E-01	9.40E-01
	GOTERM_BP_FAT	negative regulation of axonogenesis	5	4.20E-01	9.80E-01
	GOTERM_BP_FAT	negative regulation of neurogenesis	7	5.50E-01	9.90E-01
	GOTERM_BP_FAT	negative regulation of cell development	7	6.20E-01	1.00E+00
**Annotation Cluster 216**	**Enrichment Score: 0.56**		**Count**	**P_Value**	**Benjamini**
	GOTERM_MF_FAT	Rho GTPase binding	9	1.10E-01	8.10E-01
	GOTERM_MF_FAT	GTPase binding	17	3.20E-01	9.70E-01
	GOTERM_MF_FAT	Ras GTPase binding	14	3.90E-01	9.80E-01
	GOTERM_MF_FAT	small GTPase binding	15	4.20E-01	9.90E-01
**Annotation Cluster 217**	**Enrichment Score: 0.56**		**Count**	**P_Value**	**Benjamini**
	UP_SEQ_FEATURE	domain:LIM zinc-binding 3	6	1.80E-01	1.00E+00
	SP_PIR_KEYWORDS	LIM domain	12	2.30E-01	6.70E-01
	UP_SEQ_FEATURE	domain:LIM zinc-binding 1	9	2.50E-01	1.00E+00
	UP_SEQ_FEATURE	domain:LIM zinc-binding 2	9	2.50E-01	1.00E+00

ILMN_2202967	ARL15	-0.478566998
ILMN_1656940	ABLIM3	-0.47858447
ILMN_2304577	KCNK7	-0.478593948
ILMN_1752646	NA	-0.478634718
ILMN_1844083	C12orf71	-0.478683997
ILMN_1765645	C17orf50	-0.478697378
ILMN_2058141	HMGN2	-0.478765255
ILMN_1785731	MAPK8IP2	-0.47876775
ILMN_3222974	PRKCB	-0.478796715
ILMN_1853344	CA393467	-0.478936197
ILMN_1777797	AFM	-0.478952553
ILMN_1786631	SIX6	-0.478970804
ILMN_2044645	CGB1	-0.478994848
ILMN_2290089	UBN1	-0.479183682
ILMN_1737283	TMSB15B	-0.479224153
ILMN_1679295	ARL5C	-0.479260297
ILMN_2415949	MRRF	-0.479390461
ILMN_1690703	C21orf34	-0.479474718
ILMN_1657455	CEACAM4	-0.479481595
ILMN_1755364	RALA	-0.479513542
ILMN_1651347	SERTAD2	-0.479554431
ILMN_1678535	ESR1	-0.479562232
ILMN_1788267	PPP1R14D	-0.47956753
ILMN_2093027	MYO1B	-0.479590758
ILMN_1720496	GUCY1A2	-0.479623285
ILMN_1734762	OPA3	-0.479646497
ILMN_1722151	EIF6	-0.479673407
ILMN_1833478	AV700714	-0.479679436
ILMN_2181992	MTRF1	-0.479758204
ILMN_2052487	GPR45	-0.479839027
ILMN_1699384	PPP2R5D	-0.47985094
ILMN_1660865	AIDA	-0.479860684
ILMN_1667374	C14orf149	-0.479990939
ILMN_1685696	C3orf55	-0.480089797
ILMN_1900604	BX108309	-0.480116146
ILMN_2195721	KRTAP21-1	-0.480139906
ILMN_2239408	ANG	-0.480202203
ILMN_1779061	ST6GALNAC3	-0.480211648
ILMN_1755882	RNF219	-0.480264477
ILMN_2233552	POLD3	-0.480310962
ILMN_2156971	PRY	-0.480356111
ILMN_1722532	KDM3A	-0.480375155
ILMN_1723632	PIGC	-0.480380032
ILMN_1705774	TIGD5	-0.480432794
ILMN_1704135	LOC388242	-0.48043877
ILMN_2111255	VIT	-0.480491064
ILMN_1788131	CYP26A1	-0.48050802
ILMN_2222317	DNAJB4	-0.480544885
ILMN_1670255	MAGEA9B	-0.480612804
ILMN_2397846	SNCB	-0.480617184
ILMN_1813741	KCNJ16	-0.48063397
ILMN_1653814	HBBP1	-0.480636092
ILMN_3242920	GAGE12I	-0.480789041
ILMN_1811380	KCNA10	-0.480816288
ILMN_1763749	GUCA2A	-0.480822234
ILMN_1709645	CBX2	-0.480832856
ILMN_2076758	MBD3L2	-0.480858701
ILMN_1686968	ZNF362	-0.480883101
ILMN_1721984	LOC80154	-0.48089932
ILMN_1782377	IMAA	-0.480925638
ILMN_1778053	SLC39A7	-0.481006236
ILMN_1719158	CTBP1	-0.481081124
ILMN_1654250	MRPL21	-0.481081197
ILMN_3297392	UBE2K	-0.481109837
ILMN_2330267	ABCE1	-0.481120093
ILMN_1762714	VGLL2	-0.481212183
ILMN_2412380	TSC22D1	-0.481301499
ILMN_3309914	MIR660	-0.481319389
ILMN_1717265	SRRM3	-0.481335347
ILMN_1755120	MAN1A2	-0.481362114
ILMN_1750052	NOP14	-0.481368946
ILMN_1774219	C2orf51	-0.481372882
ILMN_1680081	B3GNT6	-0.481466961
ILMN_1814686	BCMO1	-0.481526208
ILMN_2212878	ESM1	-0.481577044
ILMN_1876620	NA	-0.481597411
ILMN_1754055	APOA5	-0.481608157
ILMN_1723630	LOC100128505	-0.481632441
ILMN_1738246	OR10A7	-0.481675308
ILMN_2058975	KIAA0776	-0.481711759
ILMN_3297082	LOC729080	-0.481722115
ILMN_1655347	SCGB1A1	-0.48174791
ILMN_2064926	ITFG1	-0.481811211
ILMN_1792603	GOLGA6L10	-0.481938475
ILMN_3229046	PATL2	-0.481991414
ILMN_1735918	FJH1	-0.482053976
ILMN_2152095	DROSHA	-0.482060391
ILMN_1754137	CFHR1	-0.482074657
ILMN_2041788	PLS3	-0.482141481
ILMN_1750180	HIST1H2BB	-0.482184388
ILMN_1791328	STK39	-0.48219161
ILMN_1671209	SYTL2	-0.48219418
ILMN_1818562	BC040572	-0.482196563
ILMN_3308400	MIR516A2	-0.48221901

ILMN_2345837	CLTA	0.625797514
ILMN_1792473	AIF1	0.625785042
ILMN_1708167	CDCP1	0.625724657
ILMN_1763837	ANPEP	0.625719521
ILMN_1677226	ARVCF	0.625349346
ILMN_1804117	FAM89B	0.625106039
ILMN_1716400	FOXM1	0.62490057
ILMN_1809323	C9orf125	0.62481904
ILMN_1737255	C22orf36	0.624774209
ILMN_3247661	FLJ45340	0.624768496
ILMN_1750429	DKFZp686E14208	0.624763973
ILMN_3224856	LOC100133161	0.624760764
ILMN_3272768	HSPC157	0.624693038
ILMN_2402972	GGA3	0.624672294
ILMN_1715508	NNMT	0.624649278
ILMN_2311779	TMUB2	0.624490175
ILMN_1689389	SF3B5	0.624402482
ILMN_1740620	LOC161527	0.624360526
ILMN_2358457	ATF4	0.624249583
ILMN_3198464	BC053580	0.624219975
ILMN_2109416	NAPSB	0.624179514
ILMN_1803997	SDCCAG3	0.6241661
ILMN_3214945	SMN1	0.623998723
ILMN_3229324	SGK1	0.623964063
ILMN_1659843	DNAJC3	0.6239674
ILMN_1679693	TMEM87B	0.623894029
ILMN_1769757	C14orf104	0.62355159
ILMN_1781999	ABCF2	0.623505721
ILMN_1766593	PHRF1	0.623462305
ILMN_1685125	CR626459	0.623278424
ILMN_1714352	DMWD	0.623214998
ILMN_1793959	ADPGK	0.623188836
ILMN_1703743	AATF	0.623183471
ILMN_2049303	ECI1	0.623176948
ILMN_1746408	MIDN	0.622996925
ILMN_2383097	RPL17	0.622989389
ILMN_1794213	ABHD14A	0.622600644
ILMN_1691090	MPV17	0.622500707
ILMN_3222058	LOC349114	0.622360976
ILMN_1671005	IRF2BP2	0.622230176
ILMN_1705991	SMA4	0.62218908
ILMN_1677534	SCAP	0.622158898
ILMN_1723480	BST2	0.621860309
ILMN_2364131	TTPAL	0.621797408
ILMN_3305304	POLD2	0.621682804
ILMN_2415926	THOC3	0.621612402
ILMN_3235044	LOC100288615	0.621563462
ILMN_1705737	IMPDH2	0.621411295
ILMN_1695414	ASF1B	0.621357769
ILMN_1797594	NFAT5	0.621331236
ILMN_2292178	CLEC12A	0.621270542
ILMN_1715715	CEBPA	0.621197987
ILMN_1781099	ISY1	0.621160077
ILMN_2402674	DPM3	0.621115971
ILMN_1693317	TTC15	0.620977594
ILMN_1771057	XAB2	0.620966852
ILMN_1674629	C9orf3	0.620953797
ILMN_1737580	PPPDE2	0.620775065
ILMN_2110271	OLFM2	0.620749721
ILMN_1741617	SCRT1	0.620644807
ILMN_1713561	C20orf103	0.620590608
ILMN_1712419	DCDC2	0.620437464
ILMN_1698803	ZAK	0.620419111
ILMN_1697218	MED22	0.620356201
ILMN_2391750	SFMBT1	0.620244592
ILMN_1672446	RPL11	0.620215588
ILMN_1728802	SDCCAG8	0.620192568
ILMN_1740938	APOE	0.620173322
ILMN_1755749	PGK1	0.620161189
ILMN_1802096	ABTB1	0.619776798
ILMN_2308689	AGBL5	0.619765569
ILMN_1716563	PRKCB	0.619755632
ILMN_2073235	FTH1	0.61970774
ILMN_1809040	LDLRAP1	0.619694508
ILMN_1688103	CTNNBIP1	0.619639588
ILMN_1797522	DUSP3	0.619567878
ILMN_1809010	PSMC3	0.619544418
ILMN_1811823	MED25	0.619513844
ILMN_1696704	TLE4	0.619392467
ILMN_1662905	NME1	0.619236939
ILMN_2370208	CMTM3	0.619107841
ILMN_1815668	GTF2I	0.619037669
ILMN_1701596	RPS20	0.618988482
ILMN_1763460	NHP2L1	0.618947575
ILMN_1676611	PHPT1	0.6188947
ILMN_1763524	LZTS2	0.618847218
ILMN_3289352	RPL6	0.618778872
ILMN_1771593	RRM1	0.618762992
ILMN_1713406	DKFZp434K1323	0.618661597
ILMN_2403730	ATP6V1H	0.618481454
ILMN_1697682	RECQL5	0.618474485
ILMN_1693438	FBXL12	0.618338249
ILMN_1668283	HYAL2	0.618273381
ILMN_1745130	RBFOX2	0.618205735
ILMN_1688000	POLDIP3	0.618165194
ILMN_2337923	TPD52L1	0.618136889
ILMN_1683234	DNAJC1	0.618045672

	INTERPRO	Zinc finger, LIM-type	12	3.10E-01	1.00E+00
	SMART	LIM	12	3.70E-01	9.60E-01
	UP_SEQ_FEATURE	domain:LIM zinc-binding 4	3	3.90E-01	1.00E+00
**Annotation Cluster 218**	**Enrichment Score: 0.55**		**Count**	**P_Value**	**Benjamini**
	GOTERM_BP_FAT	virus-host interaction	5	2.70E-01	9.20E-01
	GOTERM_BP_FAT	intronless viral mRNA export from host nucleus	3	2.90E-01	9.30E-01
	GOTERM_CC_FAT	transcription export complex	3	2.90E-01	7.80E-01
**Annotation Cluster 219**	**Enrichment Score: 0.55**		**Count**	**P_Value**	**Benjamini**
	GOTERM_BP_FAT	positive regulation of tumor necrosis factor production	5	1.30E-01	7.50E-01
	GOTERM_MF_FAT	peptidoglycan binding	3	3.30E-01	9.70E-01
	GOTERM_BP_FAT	regulation of tumor necrosis factor production	6	3.90E-01	9.70E-01
	GOTERM_BP_FAT	positive regulation of cytokine production	14	4.10E-01	9.70E-01
**Annotation Cluster 220**	**Enrichment Score: 0.55**		**Count**	**P_Value**	**Benjamini**
	INTERPRO	Phosphorylated immunoreceptor signaling ITAM	3	2.70E-01	1.00E+00
	SMART	ITAM	3	2.90E-01	9.40E-01
	UP_SEQ_FEATURE	domain:ITAM	3	3.00E-01	1.00E+00
**Annotation Cluster 221**	**Enrichment Score: 0.55**		**Count**	**P_Value**	**Benjamini**
	UP_SEQ_FEATURE	DNA-binding region:A.T hook	5	4.80E-02	1.00E+00
	INTERPRO	AT hook, DNA-binding, conserved site	4	6.80E-01	1.00E+00
	SMART	AT_hook	4	7.10E-01	9.90E-01
**Annotation Cluster 222**	**Enrichment Score: 0.54**		**Count**	**P_Value**	**Benjamini**
	INTERPRO	Low density lipoprotein-receptor, class A, cysteine-rich	12	4.40E-02	9.10E-01
	SMART	LDLa	12	5.80E-02	7.20E-01
	UP_SEQ_FEATURE	domain:LDL-receptor class A 1	6	2.20E-01	1.00E+00
	UP_SEQ_FEATURE	domain:LDL-receptor class A 2	6	2.20E-01	1.00E+00
	UP_SEQ_FEATURE	domain:LDL-receptor class A 4	4	3.00E-01	1.00E+00
	UP_SEQ_FEATURE	domain:LDL-receptor class A	4	4.40E-01	1.00E+00
	UP_SEQ_FEATURE	domain:CUB 1	6	4.50E-01	1.00E+00
	UP_SEQ_FEATURE	domain:CUB 2	6	4.50E-01	1.00E+00
	UP_SEQ_FEATURE	domain:LDL-receptor class A 3	4	4.80E-01	1.00E+00
	INTERPRO	CUB	6	7.90E-01	1.00E+00
	SMART	CUB	6	8.20E-01	1.00E+00
**Annotation Cluster 223**	**Enrichment Score: 0.54**		**Count**	**P_Value**	**Benjamini**
	GOTERM_BP_FAT	cell activation during immune response	8	1.90E-01	8.50E-01
	GOTERM_BP_FAT	leukocyte activation during immune response	8	1.90E-01	8.50E-01
	GOTERM_BP_FAT	leukocyte degranulation	4	2.00E-01	8.70E-01
	GOTERM_BP_FAT	lymphocyte activation during immune response	5	2.10E-01	8.70E-01
	GOTERM_BP_FAT	T cell activation during immune response	4	2.40E-01	9.00E-01
	GOTERM_BP_FAT	myeloid leukocyte mediated immunity	4	3.20E-01	9.40E-01
	GOTERM_BP_FAT	myeloid leukocyte activation	8	4.00E-01	9.70E-01
	GOTERM_BP_FAT	regulated secretory pathway	4	4.30E-01	9.80E-01
	GOTERM_BP_FAT	myeloid cell activation during immune response	3	7.10E-01	1.00E+00
**Annotation Cluster 224**	**Enrichment Score: 0.54**		**Count**	**P_Value**	**Benjamini**
	GOTERM_MF_FAT	ubiquitin binding	6	2.60E-01	9.50E-01
	GOTERM_MF_FAT	small conjugating protein binding	6	2.90E-01	9.60E-01
	GOTERM_MF_FAT	polyubiquitin binding	3	3.30E-01	9.70E-01
**Annotation Cluster 225**	**Enrichment Score: 0.53**		**Count**	**P_Value**	**Benjamini**
	GOTERM_BP_FAT	apoptotic mitochondrial changes	8	1.00E-01	7.00E-01
	BBID	127.Mito-stress	4	1.50E-01	9.60E-01
	GOTERM_BP_FAT	induction of apoptosis by intracellular signals	11	1.70E-01	8.20E-01
	GOTERM_BP_FAT	negative regulation of survival gene product expression	3	2.30E-01	8.90E-01
	GOTERM_BP_FAT	release of cytochrome c from mitochondria	5	3.00E-01	9.30E-01
	GOTERM_BP_FAT	lymphocyte apoptosis	3	3.90E-01	9.70E-01
	BIOCARTA	Role of Mitochondria in Apoptotic Signaling	6	4.30E-01	9.70E-01
	BIOCARTA	Apoptotic Signaling in Response to DNA Damage	6	4.30E-01	9.70E-01
	GOTERM_BP_FAT	regulation of survival gene product expression	4	5.40E-01	9.90E-01
	KEGG_PATHWAY	Amyotrophic lateral sclerosis (ALS)	8	7.00E-01	8.90E-01
**Annotation Cluster 226**	**Enrichment Score: 0.53**		**Count**	**P_Value**	**Benjamini**
	SP_PIR_KEYWORDS	myristylation	11	1.50E-02	1.30E-01
	INTERPRO	ADP-ribosylation factor	4	5.40E-01	1.00E+00
	SMART	ARF	4	5.70E-01	9.90E-01
	PIR_SUPERFAMILY	PIRSF001711:ADP-ribosylation factor	4	6.50E-01	1.00E+00
	INTERPRO	ARF/SAR superfamily	4	7.50E-01	1.00E+00
**Annotation Cluster 227**	**Enrichment Score: 0.53**		**Count**	**P_Value**	**Benjamini**
	GOTERM_BP_FAT	epithelial to mesenchymal transition	7	2.40E-02	3.30E-01
	GOTERM_BP_FAT	mesenchymal cell differentiation	10	2.20E-01	8.90E-01
	GOTERM_BP_FAT	mesenchymal cell development	10	2.20E-01	8.90E-01
	GOTERM_BP_FAT	mesenchyme development	10	2.40E-01	9.00E-01
	GOTERM_BP_FAT	ameboidal cell migration	7	3.60E-01	9.60E-01
	GOTERM_BP_FAT	neural crest cell migration	3	8.30E-01	1.00E+00
	GOTERM_BP_FAT	neural crest cell differentiation	4	8.30E-01	1.00E+00
	GOTERM_BP_FAT	neural crest cell development	4	8.30E-01	1.00E+00
**Annotation Cluster 228**	**Enrichment Score: 0.53**		**Count**	**P_Value**	**Benjamini**
	UP_SEQ_FEATURE	domain:SAND	3	2.50E-01	1.00E+00
	INTERPRO	SAND	3	2.70E-01	1.00E+00
	SMART	SAND	3	2.90E-01	9.40E-01
	INTERPRO	SAND-like	3	4.10E-01	1.00E+00
**Annotation Cluster 229**	**Enrichment Score: 0.52**		**Count**	**P_Value**	**Benjamini**
	BIOCARTA	TNFR1 Signaling Pathway	11	4.80E-02	8.90E-01
	GOTERM_BP_FAT	activation of pro-apoptotic gene products	7	6.00E-02	5.50E-01
	BIOCARTA	Ceramide Signaling Pathway	7	2.10E-01	9.40E-01
	GOTERM_BP_FAT	response to tumor necrosis factor	4	2.40E-01	9.00E-01
	BIOCARTA	SODD/TNFR1 Signaling Pathway	4	2.40E-01	9.50E-01
	BIOCARTA	HIV-I Nef	13	3.20E-01	9.50E-01
	BBID	40.Deg_of_Chrom_DNA_TNF-ind_apoptosis	4	3.60E-01	1.00E+00
	BBID	150.caspase_and_NFKB_activation	5	3.70E-01	9.90E-01
	BIOCARTA	FAS signaling pathway (CD95)	8	3.90E-01	9.70E-01
	BBID	44.Sig_Trans_TNFR1-DR3-DR4_DR5	3	4.40E-01	9.90E-01
	BIOCARTA	Caspase Cascade in Apoptosis	6	5.10E-01	9.80E-01
	BIOCARTA	Induction of apoptosis through DR3 and DR4/5 Death Receptors	7	5.40E-01	9.80E-01
	BIOCARTA	Keratinocyte Differentiation	7	7.70E-01	1.00E+00
	BIOCARTA	TNF/Stress Related Signaling	3	9.60E-01	1.00E+00
**Annotation Cluster 230**	**Enrichment Score: 0.52**		**Count**	**P_Value**	**Benjamini**
	INTERPRO	Peptidase C1A, papain C-terminal	5	9.00E-02	9.60E-01
	INTERPRO	Peptidase C1A, papain	5	9.00E-02	9.60E-01
	SMART	Pept_C1	5	1.00E-01	7.90E-01
	PIR_SUPERFAMILY	PIRSF001182:papain	3	3.50E-01	1.00E+00

ILMN_1687046	OR10A2	-0.482291791
ILMN_2154603	ZNF830	-0.48234456
ILMN_2163835	PRDM14	-0.482390407
ILMN_3266265	LOXHD1	-0.48241216
ILMN_1798914	C1orf65	-0.482470499
ILMN_1893915	AW195332	-0.482482882
ILMN_2204726	UBR5	-0.482624112
ILMN_1760246	BSN	-0.482688544
ILMN_3249444	POM121-like 1	-0.482715865
ILMN_2260933	C20orf71	-0.48279389
ILMN_3254046	BI599339	-0.482872776
ILMN_1656495	NOTO	-0.482918493
ILMN_2073263	C11orf61	-0.482928147
ILMN_1897107	AI056628	-0.483026736
ILMN_1676239	LOC653550	-0.483077145
ILMN_1892996	BQ011293	-0.483130494
ILMN_1789112	TMEM145	-0.483306092
ILMN_2114185	C1orf104	-0.483338188
ILMN_1800935	ARV1	-0.483354924
ILMN_1663129	MAMLD1	-0.483388175
ILMN_1798166	POM121C	-0.483419832
ILMN_1686405	KDR	-0.483474217
ILMN_1736300	KIAA1445	-0.483485054
ILMN_1738009	PRRT4	-0.483507903
ILMN_1736162	GC	-0.483562334
ILMN_1770658	NEB	-0.483569172
ILMN_1798923	MYH1	-0.483623637
ILMN_1804907	OR1F2	-0.483646653
ILMN_1798690	ADAMTSL3	-0.483650903
ILMN_1685043	CYP3A7	-0.48381583
ILMN_1747205	JDP2	-0.483875267
ILMN_1655892	BE266556	-0.483941115
ILMN_1651636	CYP2A13	-0.48395582
ILMN_1840934	AJ420430	-0.484006514
ILMN_2287543	SHISA3	-0.484009161
ILMN_1797878	OR7G3	-0.48406013
ILMN_1701386	STRADB	-0.484131496
ILMN_1739925	SLC28A1	-0.484175517
ILMN_2077623	RRAS2	-0.484193122
ILMN_1762582	ARNT	-0.48421571
ILMN_1723414	HACL1	-0.484254684
ILMN_1747151	ANKRD23	-0.484305847
ILMN_1713550	LGR6	-0.484320001
ILMN_2373515	HSP90AA1	-0.484335026
ILMN_2103014	CCDC104	-0.48443129
ILMN_1662010	DLK2	-0.484441682
ILMN_1806473	BEX5	-0.484443842
ILMN_1729529	BAI1	-0.484451211
ILMN_2164173	DYDC1	-0.484452988
ILMN_3308445	MIR642A	-0.484564696
ILMN_1676289	NCAM1	-0.484626772
ILMN_1735108	ANKS6	-0.484632481
ILMN_1676997	TREX1	-0.484653136
ILMN_1753415	LY6H	-0.484671046
ILMN_1660261	TDRD6	-0.484747081
ILMN_1654637	SETD5	-0.484757961
ILMN_1817715	CK001807	-0.485010512
ILMN_1659773	PCDHA@	-0.485020768
ILMN_3235833	NA	-0.485034538
ILMN_1736533	RND2	-0.48504642
ILMN_1787988	UBE2D1	-0.485069562
ILMN_1887349	BU753709	-0.485105833
ILMN_1909276	BX105693	-0.485121211
ILMN_1658299	CCR2	-0.485137193
ILMN_1664016	ARHGEF18	-0.485320747
ILMN_1722640	LOC284801	-0.485351539
ILMN_1755419	EIF1AX	-0.485387193
ILMN_1804137	ATP11C	-0.485475747
ILMN_1710326	CLDND1	-0.485587847
ILMN_1667804	FAM182B	-0.485651718
ILMN_1803261	OPRM1	-0.48571115
ILMN_1675946	ISG20L2	-0.485805273
ILMN_1916256	TCRA	-0.485927899
ILMN_3298694	TYW1B	-0.485939855
ILMN_1661853	TRIM49L2	-0.485946961
ILMN_1778236	PTPN11	-0.485985263
ILMN_1791283	AK126240	-0.486021395
ILMN_1755369	RAB3A	-0.486053789
ILMN_1737514	KYNU	-0.486059831
ILMN_1769931	SFPQ	-0.486100916
ILMN_2101526	GGCT	-0.48622118
ILMN_1843007	BU752765	-0.486285817
ILMN_1825443	BX089525	-0.486435449
ILMN_1745567	SMA4	-0.486471918
ILMN_1680776	C12orf70	-0.486568851
ILMN_1756367	WNK3	-0.486592904
ILMN_1771841	FOSL1	-0.486604152
ILMN_1718446	FAM69B	-0.486607736
ILMN_1893852	AW340373	-0.48662648
ILMN_2112599	C16orf80	-0.486636961
ILMN_1860645	CD706264	-0.486674586
ILMN_3229515	CSN1S2BP	-0.486700829
ILMN_1749804	ADSSL1	-0.486702768
ILMN_1667707	SPCS3	-0.486768999
ILMN_1663975	CNNM2	-0.486789474
ILMN_1741812	AK056801	-0.486854333
ILMN_1752294	PCDH9	-0.486907116

ILMN_1729767	TARBP2	0.618013865
ILMN_1897310	LETM1	0.617997
ILMN_3296816	LOC338739	0.617812825
ILMN_1703433	PLSCR3	0.617624725
ILMN_1733581	C16orf35	0.617435518
ILMN_3287068	LAMR1P15	0.617378478
ILMN_2140700	CRIPAK	0.617368782
ILMN_1737644	TMEM219	0.617297151
ILMN_2396982	BCL2L12	0.617245929
ILMN_2334693	NARF	0.617237576
ILMN_2301624	MACF1	0.617220349
ILMN_2374449	SPP1	0.617132229
ILMN_1686388	TPM3	0.61705512
ILMN_2361186	PHF20L1	0.617052259
ILMN_2123402	CNPY2	0.616868674
ILMN_2370336	MS4A4A	0.616689646
ILMN_1769201	ELF3	0.616648338
ILMN_3304435	LOC728640	0.616521564
ILMN_1708936	EXOSC3	0.616501325
ILMN_1685830	SLC7A2	0.616372785
ILMN_1722738	ROGDI	0.616352259
ILMN_3243291	HNRNPA1	0.616172737
ILMN_2087656	SLCO2B1	0.616167402
ILMN_1803392	P2RX5-TAX1BP3	0.616156047
ILMN_1715947	HNRNPA1	0.616038903
ILMN_1790228	FURIN	0.615918446
ILMN_1659913	ISG20	0.615825232
ILMN_2123567	SENP2	0.615820735
ILMN_3309089	MIR196B	0.615686153
ILMN_1677314	MUC1	0.615615678
ILMN_2403458	SMARCB1	0.615568416
ILMN_1723042	CLDN3	0.615566622
ILMN_3240838	SLC25A6	0.615523631
ILMN_2041046	CKS1B	0.61551412
ILMN_1711078	CDK11A	0.615483088
ILMN_1773059	GPR124	0.615379201
ILMN_2208802	NPIPL3	0.615258485
ILMN_1693014	CEBPB	0.615145393
ILMN_1811790	FOXS1	0.615093188
ILMN_2054145	PAK1IP1	0.615027205
ILMN_1681467	RAB11FIP4	0.614937828
ILMN_1811117	DKFZp667P0924	0.614929997
ILMN_1788062	SH3GL1	0.614908688
ILMN_1695420	CLTA	0.614829608
ILMN_1654065	ATOH8	0.614814588
ILMN_1777584	KARS	0.614782688
ILMN_1755462	UGCGL1	0.614714292
ILMN_1793267	ETHE1	0.614645399
ILMN_1691717	RHBDF2	0.614571358
ILMN_1764723	SH3PXD2B	0.614105043
ILMN_2386269	U2AF1L4	0.613988701
ILMN_1738681	NUP62	0.613986277
ILMN_2203588	MYL5	0.613890043
ILMN_2299045	BMP2K	0.613754389
ILMN_1777881	TSPAN17	0.613693348
ILMN_2380850	SDCCAG3	0.613670142
ILMN_1774823	RPL34	0.613361396
ILMN_1801421	EMD	0.613312834
ILMN_1709032	FYCO1	0.613274137
ILMN_1903914	AK129699	0.613260843
ILMN_1680434	MAPK8IP1	0.613107427
ILMN_1665428	GSDMD	0.613045137
ILMN_2163723	KRT7	0.612879754
ILMN_1718537	HPS6	0.61283754
ILMN_1713037	PFKL	0.612676316
ILMN_2083066	IGLL3P	0.612643105
ILMN_1671106	GJA4	0.612628393
ILMN_1703326	GPR44	0.612590433
ILMN_2364529	EZH2	0.612517783
ILMN_1677589	hCG_1818547	0.612489948
ILMN_1738529	BCS1L	0.612305778
ILMN_1655821	CAPG	0.612230463
ILMN_1747023	PTGIR	0.612093773
ILMN_1655952	WASH3P	0.611877331
ILMN_1751161	COL7A1	0.611867
ILMN_2351298	WIPF1	0.611747531
ILMN_3246159	C20orf191	0.611726723
ILMN_2176502	UNC5B	0.611533032
ILMN_3240321	AEN	0.611496932
ILMN_3248113	SNRNP70	0.6114509
ILMN_1780769	TUBB2C	0.611443455
ILMN_3239055	TMEM208	0.611264163
ILMN_1761764	ALKBH7	0.611168576
ILMN_1725992	RAPGEF6	0.610998934
ILMN_1653278	MUC20	0.610985416
ILMN_2313730	RHOC	0.610908254
ILMN_1815718	BTRC	0.610708871
ILMN_2244841	ALDH4A1	0.610660181

	INTERPRO	Peptidase, cysteine peptidase active site	6	4.80E-01	1.00E+00
	GOTERM_MF_FAT	cysteine-type endopeptidase activity	10	5.90E-01	1.00E+00
	GOTERM_MF_FAT	cysteine-type peptidase activity	14	9.30E-01	1.00E+00
	SP_PIR_KEYWORDS	thiol protease	11	9.50E-01	1.00E+00
**Annotation Cluster 231**	**Enrichment Score: 0.52**		**Count**	**P_Value**	**Benjamini**
	GOTERM_MF_FAT	ATPase activity, coupled	53	2.00E-03	1.20E-01
	GOTERM_BP_FAT	purine nucleoside triphosphate metabolic process	23	7.70E-02	6.10E-01
	GOTERM_BP_FAT	purine ribonucleoside triphosphate metabolic process	22	8.60E-02	6.40E-01
	GOTERM_BP_FAT	ribonucleoside triphosphate metabolic process	22	9.20E-02	6.60E-01
	GOTERM_MF_FAT	ATPase activity, coupled to movement of substances	20	1.10E-01	8.10E-01
	GOTERM_BP_FAT	nucleoside triphosphate metabolic process	23	1.40E-01	7.70E-01
	GOTERM_BP_FAT	purine ribonucleotide metabolic process	24	1.40E-01	7.70E-01
	GOTERM_MF_FAT	P-P-bond-hydrolysis-driven transmembrane transporter activity	21	1.50E-01	8.70E-01
	GOTERM_MF_FAT	primary active transmembrane transporter activity	21	1.50E-01	8.70E-01
	GOTERM_MF_FAT	ATPase activity, coupled to transmembrane movement of substances	19	1.60E-01	8.70E-01
	GOTERM_MF_FAT	hydrolase activity, acting on acid anhydrides, catalyzing transmembrane movement of substances	19	1.80E-01	8.90E-01
	GOTERM_BP_FAT	ATP metabolic process	18	2.20E-01	8.80E-01
	GOTERM_BP_FAT	ribonucleotide metabolic process	24	2.20E-01	8.80E-01
	GOTERM_MF_FAT	hydrogen ion transmembrane transporter activity	15	2.60E-01	9.50E-01
	GOTERM_BP_FAT	energy coupled proton transport, down electrochemical gradient	8	2.70E-01	9.20E-01
	GOTERM_BP_FAT	ATP synthesis coupled proton transport	8	2.70E-01	9.20E-01
	GOTERM_BP_FAT	purine ribonucleoside triphosphate biosynthetic process	16	3.10E-01	9.40E-01
	GOTERM_MF_FAT	cation-transporting ATPase activity	7	3.10E-01	9.70E-01
	GOTERM_BP_FAT	hydrogen transport	11	3.20E-01	9.40E-01
	GOTERM_BP_FAT	ribonucleoside triphosphate biosynthetic process	16	3.20E-01	9.50E-01
	GOTERM_BP_FAT	purine nucleoside triphosphate biosynthetic process	16	3.20E-01	9.50E-01
	GOTERM_BP_FAT	nitrogen compound biosynthetic process	47	3.30E-01	9.50E-01
	GOTERM_BP_FAT	purine nucleotide metabolic process	28	3.30E-01	9.50E-01
	GOTERM_MF_FAT	proton-transporting ATPase activity, rotational mechanism	5	3.40E-01	9.70E-01
	SP_PIR_KEYWORDS	Hydrogen ion transport	8	3.60E-01	8.00E-01
	GOTERM_MF_FAT	monovalent inorganic cation transmembrane transporter activity	16	3.60E-01	9.70E-01
	GOTERM_BP_FAT	nucleoside triphosphate biosynthetic process	16	3.70E-01	9.60E-01
	GOTERM_BP_FAT	purine ribonucleotide biosynthetic process	18	3.70E-01	9.60E-01
	GOTERM_BP_FAT	nucleobase, nucleoside and nucleotide biosynthetic process	28	4.00E-01	9.70E-01
	GOTERM_BP_FAT	nucleobase, nucleoside, nucleotide and nucleic acid biosynthetic process	28	4.00E-01	9.70E-01
	GOTERM_BP_FAT	proton transport	10	4.10E-01	9.70E-01
	GOTERM_BP_FAT	ribonucleotide biosynthetic process	18	4.70E-01	9.80E-01
	GOTERM_BP_FAT	ion transmembrane transport	8	4.70E-01	9.80E-01
	GOTERM_BP_FAT	purine nucleotide biosynthetic process	21	4.90E-01	9.90E-01
	GOTERM_BP_FAT	ATP biosynthetic process	13	5.10E-01	9.90E-01
	GOTERM_BP_FAT	oxidative phosphorylation	14	5.40E-01	9.90E-01
	GOTERM_CC_FAT	proton-transporting ATP synthase complex	4	5.50E-01	9.30E-01
	GOTERM_CC_FAT	proton-transporting two-sector ATPase complex	7	5.70E-01	9.40E-01
	GOTERM_BP_FAT	nucleotide biosynthetic process	25	5.80E-01	9.90E-01
	GOTERM_MF_FAT	ATPase activity, coupled to transmembrane movement of ions	10	6.20E-01	1.00E+00
	GOTERM_MF_FAT	hydrogen ion transporting ATP synthase activity, rotational mechanism	3	6.30E-01	1.00E+00
	GOTERM_CC_FAT	proton-transporting two-sector ATPase complex, proton-transporting domain	4	6.40E-01	9.60E-01
	GOTERM_MF_FAT	inorganic cation transmembrane transporter activity	19	6.70E-01	1.00E+00
	GOTERM_CC_FAT	proton-transporting two-sector ATPase complex, catalytic domain	3	7.10E-01	9.70E-01
	GOTERM_CC_FAT	mitochondrial proton-transporting ATP synthase complex	3	7.40E-01	9.80E-01
	GOTERM_CC_FAT	proton-transporting V-type ATPase complex	3	7.70E-01	9.80E-01
	GOTERM_CC_FAT	mitochondrial respiratory chain	8	7.70E-01	9.80E-01
	GOTERM_CC_FAT	mitochondrial membrane part	14	8.70E-01	1.00E+00
	GOTERM_BP_FAT	monovalent inorganic cation transport	22	1.00E+00	1.00E+00
**Annotation Cluster 232**	**Enrichment Score: 0.51**		**Count**	**P_Value**	**Benjamini**
	GOTERM_CC_FAT	BRCA1-A complex	4	5.50E-02	3.50E-01
	GOTERM_BP_FAT	G2/M transition checkpoint	5	1.50E-01	7.90E-01
	GOTERM_BP_FAT	regulation of DNA repair	5	3.30E-01	9.50E-01
	GOTERM_BP_FAT	response to ionizing radiation	10	3.90E-01	9.70E-01
	GOTERM_BP_FAT	G2/M transition DNA damage checkpoint	3	5.30E-01	9.90E-01
	GOTERM_BP_FAT	positive regulation of DNA repair	3	6.10E-01	1.00E+00
	GOTERM_BP_FAT	double-strand break repair	8	7.30E-01	1.00E+00
**Annotation Cluster 233**	**Enrichment Score: 0.51**		**Count**	**P_Value**	**Benjamini**
	UP_SEQ_FEATURE	repeat:TPR 9	8	6.30E-02	1.00E+00
	UP_SEQ_FEATURE	repeat:TPR 12	3	3.50E-01	1.00E+00
	UP_SEQ_FEATURE	repeat:TPR 8	8	3.60E-01	1.00E+00
	UP_SEQ_FEATURE	repeat:TPR 11	3	4.80E-01	1.00E+00
	UP_SEQ_FEATURE	repeat:TPR 10	3	7.20E-01	1.00E+00
**Annotation Cluster 234**	**Enrichment Score: 0.51**		**Count**	**P_Value**	**Benjamini**
	GOTERM_CC_FAT	nuclear heterochromatin	8	8.30E-02	4.60E-01
	GOTERM_CC_FAT	sex chromatin	3	5.80E-01	9.40E-01
	GOTERM_CC_FAT	sex chromosome	3	6.10E-01	9.50E-01
**Annotation Cluster 235**	**Enrichment Score: 0.51**		**Count**	**P_Value**	**Benjamini**
	GOTERM_BP_FAT	interphase	18	2.30E-01	8.90E-01
	GOTERM_BP_FAT	interphase of mitotic cell cycle	17	2.80E-01	9.20E-01
	GOTERM_BP_FAT	G1/S transition of mitotic cell cycle	9	4.60E-01	9.80E-01
**Annotation Cluster 236**	**Enrichment Score: 0.51**		**Count**	**P_Value**	**Benjamini**
	INTERPRO	D111/G-patch	5	3.00E-01	1.00E+00
	UP_SEQ_FEATURE	domain:G-patch	5	3.00E-01	1.00E+00
	SMART	G_patch	5	3.30E-01	9.60E-01
**Annotation Cluster 237**	**Enrichment Score: 0.51**		**Count**	**P_Value**	**Benjamini**
	GOTERM_BP_FAT	respiratory burst	6	2.00E-02	3.00E-01
	SP_PIR_KEYWORDS	antioxidant	4	8.40E-02	3.90E-01
	UP_SEQ_FEATURE	active site:Cysteine sulfenic acid (-SOH) intermediate	3	1.60E-01	1.00E+00
	GOTERM_BP_FAT	cellular response to oxidative stress	9	2.00E-01	8.60E-01
	INTERPRO	Alkyl hydroperoxide reductase/ Thiol specific antioxidant/ Mal allergen	3	2.20E-01	1.00E+00
	GOTERM_BP_FAT	hydrogen peroxide metabolic process	6	2.30E-01	8.90E-01
	GOTERM_MF_FAT	peroxiredoxin activity	3	2.70E-01	9.60E-01

ILMN_1780996	ETAA1	-0.486932571
ILMN_1781472	CDC42BPA	-0.486958384
ILMN_1656630	AY831680	-0.48705526
ILMN_1910805	BE326664	-0.487184216
ILMN_1701507	EHHADH	-0.487243649
ILMN_2295844	VAMP1	-0.487351562
ILMN_1748661	AKT1	-0.487363086
ILMN_1653878	CACNB2	-0.487430352
ILMN_1838137	BF514549	-0.487446463
ILMN_2373779	COPS8	-0.487453259
ILMN_1885199	AI733327	-0.487462655
ILMN_2373010	TMEM70	-0.487528536
ILMN_1749343	PLXNA4	-0.487595114
ILMN_1795822	DIS3L	-0.487704226
ILMN_3187648	BC153822	-0.487710386
ILMN_3183069	C17orf107	-0.487716017
ILMN_3269324	AK094963	-0.487768976
ILMN_1802414	CA13	-0.487832083
ILMN_1657050	ARSK	-0.487918423
ILMN_2120575	HTATSF1	-0.487960094
ILMN_1721541	WIF1	-0.488027774
ILMN_1701289	LOC146880	-0.488041555
ILMN_3236661	SDR16C5	-0.488041593
ILMN_1782937	GSTA1	-0.488137579
ILMN_1730620	ARRB1	-0.488222874
ILMN_2131123	AMY2A	-0.488223214
ILMN_1887928	BQ775960	-0.488266937
ILMN_3240173	MTSS1L	-0.488269971
ILMN_1728785	GPR116	-0.488298714
ILMN_3239408	REXO1L1	-0.48829914
ILMN_1810652	LMBRD2	-0.488340651
ILMN_3277332	OR7E14P	-0.488479804
ILMN_2052598	ARMC10	-0.48848483
ILMN_1702837	PSMD1	-0.488511173
ILMN_2076484	FAM126B	-0.488627404
ILMN_3238299	NHEDC1	-0.488701963
ILMN_1745570	KLK7	-0.488782955
ILMN_1657010	FAM90A12	-0.488823777
ILMN_1727438	KCND3	-0.488921108
ILMN_1723846	METTL21B	-0.488971905
ILMN_2059294	RTCD1	-0.48900466
ILMN_1671154	TMPRSS13	-0.489071031
ILMN_1683453	FRAS1	-0.48908457
ILMN_1664449	ALG5	-0.489181354
ILMN_1740627	TIAM2	-0.489213818
ILMN_1720235	ADSSL1	-0.489220096
ILMN_1915734	DA722162	-0.489237793
ILMN_1759448	CPZ	-0.489256511
ILMN_1679881	WRN	-0.489376618
ILMN_1708145	LOC100132966	-0.489432097
ILMN_1714782	OPRK1	-0.489484764
ILMN_1741957	RABEPK	-0.489512287
ILMN_1699562	EFCAB1	-0.48957376
ILMN_1802450	CNOT6L	-0.489641299
ILMN_1764605	FGF6	-0.489696579
ILMN_1761829	HK1	-0.489792445
ILMN_1845693	COL29A1	-0.489856182
ILMN_1780783	SOCS7	-0.489859893
ILMN_1800465	DHRS4L2	-0.49000246
ILMN_1659099	ROCK2	-0.490036548
ILMN_1766028	TBC1D26	-0.490047284
ILMN_2409596	RAB11FIP1	-0.490097181
ILMN_1803376	AEBP2	-0.490111163
ILMN_1800923	OR4M1	-0.490172709
ILMN_3251132	TMOD2	-0.49021148
ILMN_3301206	MAGIX	-0.490231692
ILMN_1665022	SMN1	-0.490261201
ILMN_1716357	AK097401	-0.490287181
ILMN_1809760	KLHL1	-0.490347764
ILMN_2156699	ACOT6	-0.490390855
ILMN_1783705	GRXCR2	-0.490566368
ILMN_1886537	AI859685	-0.490588762
ILMN_1775192	BCLAF1	-0.490633872
ILMN_1820327	NA	-0.490658947
ILMN_1726112	PKP3	-0.490763684
ILMN_1709256	PLEC	-0.490861396
ILMN_1783497	PANK1	-0.490873962
ILMN_2043109	TOP1P2	-0.490889484
ILMN_1724166	OR56B1	-0.490978835
ILMN_3191416	LOC100131199	-0.490995585
ILMN_2372681	MAGEA6	-0.491001816
ILMN_2086612	DKFZp686H12134	-0.491019129
ILMN_1703648	TMEM82	-0.491078523
ILMN_2057220	HRSP12	-0.491083967
ILMN_1781419	C11orf73	-0.491103443
ILMN_2279144	PHF16	-0.491124319
ILMN_1795673	C9orf173	-0.491212072
ILMN_1740041	HERC2P7	-0.491230771

ILMN_1653115	ECH1	0.610550226
ILMN_2043615	C17orf90	0.610447793
ILMN_1766713	HSPD1	0.610421587
ILMN_1740500	TSPYL4	0.610416845
ILMN_2376408	DPM3	0.610354929
ILMN_1677261	LZTS1	0.610230505
ILMN_2335604	SKAP1	0.610019676
ILMN_1655710	RPS25	0.610002765
ILMN_2139943	RPS3A	0.609990545
ILMN_1760509	EOMES	0.609953246
ILMN_1741259	RPS2	0.609900608
ILMN_1792733	FOXA3	0.609897719
ILMN_1738819	EFTUD2	0.609666627
ILMN_1688246	LOC642852	0.60962932
ILMN_1740742	UROD	0.609508182
ILMN_1724612	SLC25A35	0.609464329
ILMN_1726456	SLC3A2	0.609364313
ILMN_1775542	FAIM3	0.609340482
ILMN_1722524	GALNT14	0.609256221
ILMN_2340721	TMEM134	0.609251119
ILMN_1778333	MMP24	0.609094517
ILMN_3236135	FAM86B1	0.608972058
ILMN_2209260	KRTAP1-3	0.608930002
ILMN_2074258	BARD1	0.608921187
ILMN_1785060	TSPAN14	0.608864415
ILMN_2304624	EIF4H	0.608769001
ILMN_1797453	ARMC8	0.608606675
ILMN_1690064	RUNDC2B	0.608419098
ILMN_1815024	PRDX5	0.608334596
ILMN_1753241	SNTA1	0.608229652
ILMN_1694571	SPDYE7P	0.608118275
ILMN_2409055	FAM13C	0.608034601
ILMN_1717793	C19orf33	0.607808158
ILMN_3208233	RBMX	0.607798713
ILMN_1772991	MIR671	0.607705427
ILMN_1675541	EEF1B2	0.607692853
ILMN_1749448	GLMN	0.607692322
ILMN_2352131	ERBB2	0.607672202
ILMN_2380237	C1QTNF1	0.607661626
ILMN_2307656	AGTRAP	0.607639506
ILMN_1803739	RPGRIP1	0.607533186
ILMN_1698950	PWP2	0.607450684
ILMN_1699878	RAB27A	0.607376406
ILMN_1656625	RPS24	0.607255827
ILMN_2234310	GLTPD1	0.607217285
ILMN_1806320	PPFIBP1	0.607151322
ILMN_1769031	MTOR	0.607029644
ILMN_2198393	KATNA1	0.60699289
ILMN_1767253	RRP12	0.606923438
ILMN_1656904	SLC1A4	0.606869559
ILMN_1723177	ANAPC5	0.606834762
ILMN_2295506	LUC7L	0.606634645
ILMN_1789176	PSMB1	0.606591381
ILMN_1774287	CFB	0.606530067
ILMN_1768482	CD8A	0.60652102
ILMN_3292224	HNRNPA1	0.606494982
ILMN_1768773	RAB4B-EGLN2	0.606492562
ILMN_2396269	CDH24	0.606468537
ILMN_1658053	DYNLRB1	0.60636705
ILMN_1658373	PAN2	0.606187893
ILMN_1727809	STK35	0.606116094
ILMN_1682736	FT002	0.606066042
ILMN_2312296	PCBP2	0.606053262
ILMN_1759003	SNX12	0.605960101
ILMN_3224952	RPS16	0.605920918
ILMN_2169736	PGBD4	0.605797384
ILMN_1709026	C6orf145	0.605635045
ILMN_2399571	CBY1	0.605538674
ILMN_1674533	TRPV6	0.605479305
ILMN_1730734	TMEM205	0.605425575
ILMN_1654072	CX3CL1	0.605355362
ILMN_1696601	VARS	0.605345003
ILMN_2376416	UBXN11	0.605295637
ILMN_1680102	C11orf45	0.605194914
ILMN_1791840	RALBP1	0.605130631
ILMN_1766560	ANKHD1	0.605063042
ILMN_1738750	TFCP2	0.604962072
ILMN_1683980	PLEKHM2	0.604889263
ILMN_1789809	RPL13AP6	0.60471905
ILMN_1678831	GCC2	0.604660433
ILMN_2313946	CPNE7	0.604640291
ILMN_1699226	UBR4	0.604636177
ILMN_1787410	EIF6	0.604273031
ILMN_1733927	TCEB2	0.604239323
ILMN_2353732	CD8A	0.60420109
ILMN_2314080	CTAGE5	0.604185247
ILMN_1708025	RBPMS	0.604123918
ILMN_1751726	RPLP0	0.604063322
ILMN_1745217	KIAA1310	0.604058665
ILMN_1657857	TMEM14C	0.604042502
ILMN_2326737	PPIE	0.60399066
ILMN_1787879	ARL2	0.603955588
ILMN_3287687	TUBB2A	0.60380977
ILMN_3188076	RPS7	0.603804338
ILMN_1652407	ZMYND8	0.60378994
ILMN_2226917	KIAA0247	0.603714965

	GOTERM_BP_FAT	response to reactive oxygen species	13	2.80E-01	9.20E-01
	SP_PIR_KEYWORDS	peroxidase	5	3.60E-01	8.00E-01
	GOTERM_BP_FAT	cellular response to reactive oxygen species	6	3.60E-01	9.60E-01
	UP_SEQ_FEATURE	domain:Thioredoxin	6	3.70E-01	1.00E+00
	GOTERM_BP_FAT	hydrogen peroxide catabolic process	4	3.90E-01	9.70E-01
	GOTERM_MF_FAT	oxidoreductase activity, acting on peroxide as acceptor	6	3.90E-01	9.80E-01
	GOTERM_MF_FAT	peroxidase activity	6	3.90E-01	9.80E-01
	GOTERM_MF_FAT	antioxidant activity	8	4.00E-01	9.80E-01
	GOTERM_MF_FAT	glutathione peroxidase activity	3	4.20E-01	9.90E-01
	GOTERM_BP_FAT	superoxide metabolic process	5	4.20E-01	9.80E-01
	GOTERM_BP_FAT	cellular response to hydrogen peroxide	4	4.30E-01	9.80E-01
	GOTERM_BP_FAT	response to hydrogen peroxide	9	4.60E-01	9.80E-01
	GOTERM_BP_FAT	response to oxidative stress	23	5.00E-01	9.90E-01
	GOTERM_BP_FAT	oxygen and reactive oxygen species metabolic process	10	5.30E-01	9.90E-01
	GOTERM_BP_FAT	peptide metabolic process	8	5.60E-01	9.90E-01
	GOTERM_BP_FAT	response to inorganic substance	26	6.90E-01	1.00E+00
	GOTERM_BP_FAT	glutathione metabolic process	4	7.30E-01	1.00E+00
**Annotation Cluster 238**	**Enrichment Score: 0.5**		**Count**	**P_Value**	**Benjamini**
	INTERPRO	Octicosapeptide/Phox/Bem1p	4	2.60E-01	1.00E+00
	SMART	PB1	4	2.80E-01	9.30E-01
	UP_SEQ_FEATURE	domain:OPR	3	4.40E-01	1.00E+00
**Annotation Cluster 239**	**Enrichment Score: 0.5**		**Count**	**P_Value**	**Benjamini**
	UP_SEQ_FEATURE	DNA-binding region:Tryptophan pentad repeat	3	3.00E-01	1.00E+00
	INTERPRO	Interferon regulatory factor	3	3.20E-01	1.00E+00
	INTERPRO	Interferon regulatory factor, conserved site	3	3.20E-01	1.00E+00
	SMART	IRF	3	3.40E-01	9.60E-01
**Annotation Cluster 240**	**Enrichment Score: 0.5**		**Count**	**P_Value**	**Benjamini**
	GOTERM_CC_FAT	NuA4 histone acetyltransferase complex	4	2.80E-01	7.70E-01
	GOTERM_CC_FAT	H4/H2A histone acetyltransferase complex	4	3.20E-01	8.10E-01
	GOTERM_CC_FAT	histone acetyltransferase complex	9	3.50E-01	8.30E-01
**Annotation Cluster 241**	**Enrichment Score: 0.49**		**Count**	**P_Value**	**Benjamini**
	INTERPRO	Translation elongation factor EFTu/EF1A, domain 2	4	2.90E-01	1.00E+00
	INTERPRO	Translation elongation factor EFTu/EF1A, C-terminal	3	3.20E-01	1.00E+00
	INTERPRO	Protein synthesis factor, GTP-binding	4	3.60E-01	1.00E+00
**Annotation Cluster 242**	**Enrichment Score: 0.49**		**Count**	**P_Value**	**Benjamini**
	SP_PIR_KEYWORDS	SH2 domain	18	1.80E-01	6.00E-01
	INTERPRO	SH2 motif	17	3.20E-01	1.00E+00
	SMART	SH2	17	3.90E-01	9.60E-01
	UP_SEQ_FEATURE	domain:SH2	13	5.00E-01	1.00E+00
**Annotation Cluster 243**	**Enrichment Score: 0.49**		**Count**	**P_Value**	**Benjamini**
	UP_SEQ_FEATURE	short sequence motif:BH2	4	2.00E-01	1.00E+00
	INTERPRO	Apoptosis regulator Bcl-2, BH	5	3.00E-01	1.00E+00
	SMART	BCL	5	3.30E-01	9.60E-01
	UP_SEQ_FEATURE	short sequence motif:BH3	4	3.40E-01	1.00E+00
	UP_SEQ_FEATURE	short sequence motif:BH1	3	3.90E-01	1.00E+00
	INTERPRO	BCL2-like apoptosis inhibitor	3	4.60E-01	1.00E+00
**Annotation Cluster 244**	**Enrichment Score: 0.49**		**Count**	**P_Value**	**Benjamini**
	GOTERM_BP_FAT	regulation of establishment of protein localization	22	1.10E-01	7.10E-01
	GOTERM_BP_FAT	regulation of intracellular protein transport	11	1.50E-01	8.00E-01
	GOTERM_BP_FAT	regulation of protein transport	20	1.70E-01	8.20E-01
	GOTERM_BP_FAT	regulation of protein localization	23	2.00E-01	8.60E-01
	GOTERM_BP_FAT	regulation of protein import into nucleus	9	2.20E-01	8.80E-01
	GOTERM_BP_FAT	regulation of nucleocytoplasmic transport	11	2.30E-01	8.90E-01
	GOTERM_BP_FAT	positive regulation of protein transport	12	2.60E-01	9.20E-01
	GOTERM_BP_FAT	regulation of intracellular transport	12	3.30E-01	9.50E-01
	GOTERM_BP_FAT	regulation of transcription factor import into nucleus	6	3.60E-01	9.60E-01
	GOTERM_BP_FAT	regulation of NF-kappaB import into nucleus	4	4.30E-01	9.80E-01
	GOTERM_BP_FAT	positive regulation of nucleocytoplasmic transport	4	5.70E-01	9.90E-01
	GOTERM_BP_FAT	positive regulation of intracellular transport	5	5.70E-01	9.90E-01
	GOTERM_BP_FAT	positive regulation of protein import into nucleus	3	6.80E-01	1.00E+00
	GOTERM_BP_FAT	positive regulation of transport	27	7.80E-01	1.00E+00
	GOTERM_BP_FAT	regulation of cellular localization	30	7.80E-01	1.00E+00
**Annotation Cluster 245**	**Enrichment Score: 0.47**		**Count**	**P_Value**	**Benjamini**
	GOTERM_MF_FAT	transcription regulator activity	223	1.50E-02	3.50E-01
	SP_PIR_KEYWORDS	Transcription	268	1.10E-01	4.70E-01
	SP_PIR_KEYWORDS	transcription regulation	259	1.70E-01	5.90E-01
	GOTERM_MF_FAT	transcription factor activity	130	3.80E-01	9.80E-01
	GOTERM_BP_FAT	regulation of RNA metabolic process	236	6.30E-01	1.00E+00
	GOTERM_BP_FAT	regulation of transcription	339	6.40E-01	1.00E+00
	GOTERM_BP_FAT	transcription	273	6.50E-01	1.00E+00
	GOTERM_BP_FAT	regulation of transcription, DNA-dependent	226	7.60E-01	1.00E+00
	GOTERM_MF_FAT	DNA binding	280	9.30E-01	1.00E+00
	SP_PIR_KEYWORDS	dna-binding	207	9.30E-01	1.00E+00
**Annotation Cluster 246**	**Enrichment Score: 0.47**		**Count**	**P_Value**	**Benjamini**
	INTERPRO	Ephrin, conserved site	3	2.70E-01	1.00E+00
	INTERPRO	Ephrin	3	2.70E-01	1.00E+00
	INTERPRO	Cupredoxin	3	5.40E-01	1.00E+00
**Annotation Cluster 247**	**Enrichment Score: 0.47**		**Count**	**P_Value**	**Benjamini**
	GOTERM_BP_FAT	protein maturation	24	4.70E-02	4.80E-01
	SP_PIR_KEYWORDS	innate immunity	14	1.40E-01	5.30E-01
	GOTERM_BP_FAT	protein maturation by peptide bond cleavage	16	1.60E-01	8.00E-01
	SP_PIR_KEYWORDS	complement alternate pathway	4	2.00E-01	6.30E-01
	GOTERM_BP_FAT	humoral immune response	14	2.40E-01	9.00E-01
	GOTERM_BP_FAT	protein processing	18	3.00E-01	9.40E-01
	GOTERM_BP_FAT	acute inflammatory response	16	3.10E-01	9.40E-01
	GOTERM_BP_FAT	complement activation	8	3.10E-01	9.40E-01
	GOTERM_BP_FAT	complement activation, alternative pathway	4	3.20E-01	9.40E-01
	GOTERM_BP_FAT	activation of plasma proteins involved in acute inflammatory response	8	3.40E-01	9.50E-01
	GOTERM_BP_FAT	humoral immune response mediated by circulating immunoglobulin	6	3.90E-01	9.70E-01
	SP_PIR_KEYWORDS	complement pathway	5	4.70E-01	8.80E-01
	GOTERM_BP_FAT	complement activation, classical pathway	5	5.40E-01	9.90E-01
	BIOCARTA	Alternative Complement Pathway	3	6.00E-01	9.90E-01
	BIOCARTA	Complement Pathway	4	6.70E-01	9.90E-01
	SP_PIR_KEYWORDS	acute phase	4	7.00E-01	9.60E-01
	KEGG_PATHWAY	Complement and coagulation cascades	9	8.30E-01	9.40E-01
	SP_PIR_KEYWORDS	plasma	7	9.70E-01	1.00E+00

ILMN_1704627	BM723461	-0.491254183
ILMN_2202320	BRWD1-IT2	-0.49134899
ILMN_1813955	SERTAD3	-0.491361493
ILMN_1664448	PEX12	-0.491398755
ILMN_1725387	TMEM200A	-0.491443294
ILMN_2215043	RNF32	-0.491472247
ILMN_3225244	TSEN15	-0.491500431
ILMN_1746819	C5	-0.491523781
ILMN_1669669	KCMF1	-0.491529827
ILMN_1730032	BOK	-0.491531516
ILMN_1712066	EXPH5	-0.491583942
ILMN_1673713	LOC646168	-0.491640506
ILMN_1760412	SHISA2	-0.491661543
ILMN_1728532	FAM72A	-0.491695174
ILMN_1776524	LOC388692	-0.491724449
ILMN_1666966	IGF2	-0.491775952
ILMN_1813775	GAK	-0.491795733
ILMN_2045453	CFHR2	-0.491837928
ILMN_2107184	SNRK	-0.491846243
ILMN_1801778	MPPED1	-0.491850239
ILMN_3240993	NA	-0.491861244
ILMN_1652409	SPATA7	-0.491868132
ILMN_1664014	STOX1	-0.491884019
ILMN_1753729	KNG1	-0.491894172
ILMN_2372379	MGA	-0.49191354
ILMN_1754103	CLDN11	-0.491950576
ILMN_1800049	MAPT	-0.491972058
ILMN_1796455	RYR3	-0.49199601
ILMN_2232430	NMD3	-0.492064549
ILMN_1804328	WWP1	-0.492082006
ILMN_1703545	CSHL1	-0.492091384
ILMN_1783168	NETO1	-0.49215635
ILMN_1811478	MFI2	-0.492396909
ILMN_1780504	C19orf26	-0.492401509
ILMN_1671682	LOC554249	-0.492423831
ILMN_1791933	CCDC82	-0.49243437
ILMN_2293511	CTNND1	-0.492452557
ILMN_1695418	IQGAP3	-0.492622316
ILMN_3309939	MIR302F	-0.492627887
ILMN_3304064	DAMS	-0.492643715
ILMN_1773493	TIMM23	-0.492752372
ILMN_1740576	BC169297	-0.492798836
ILMN_1866290	BI827719	-0.492838246
ILMN_2204526	TCTEX1D4	-0.492955734
ILMN_1665630	HMGN2	-0.493048252
ILMN_1707551	AFMID	-0.49309896
ILMN_1771065	AB621825	-0.493130053
ILMN_3236481	HIGD1A	-0.493170178
ILMN_1730448	DEFB108B	-0.493405901
ILMN_1680284	ZNF681	-0.493426146
ILMN_3295874	NA	-0.493426376
ILMN_1684339	LRIT1	-0.493495651
ILMN_2177234	DEFB104B	-0.493621684
ILMN_1784302	LIPJ	-0.493629619
ILMN_1672591	SCN9A	-0.493839753
ILMN_1897796	BQ773461	-0.49387516
ILMN_3221852	LOC728405	-0.4938893
ILMN_1852041	CA391357	-0.493975241
ILMN_1669033	NCOA1	-0.493986715
ILMN_2180252	FAM70A	-0.494004826
ILMN_1737806	DKFZp686K1684	-0.494008084
ILMN_1902060	CD000292	-0.494046447
ILMN_1693674	TRDN	-0.494063022
ILMN_1774757	ZNF496	-0.494112853
ILMN_1661066	NCRNA00087	-0.494155953
ILMN_1887508	AI206543	-0.494255035
ILMN_3240586	PLD6	-0.494263446
ILMN_3241904	MAN1C1	-0.494341248
ILMN_3310548	MIR889	-0.494371811
ILMN_1656066	TNPO2	-0.494463923
ILMN_1790160	KIT	-0.49447714
ILMN_1741389	B3GNT8	-0.494486965
ILMN_3280496	RPL26	-0.494539906
ILMN_1779264	PSMG1	-0.494569709
ILMN_1669123	AK125437	-0.494589886
ILMN_1728107	GNG7	-0.494673002
ILMN_2385688	RABL2B	-0.49480939
ILMN_1770336	AGRP	-0.494880584
ILMN_2066151	TEK	-0.494902748
ILMN_1758343	LOC100129233	-0.494962869
ILMN_1788468	ARGLU1	-0.49500168
ILMN_2385161	CUL4B	-0.495108415
ILMN_3187619	NA	-0.495115324
ILMN_3205271	HMGB1	-0.495191867
ILMN_1782173	CBX3	-0.495206971
ILMN_1731518	PLD6	-0.495374389
ILMN_1740213	ELOVL7	-0.495400922
ILMN_1724234	TRPV1	-0.495465121
ILMN_1682273	SHISA3	-0.495512896
ILMN_1703408	FZD3	-0.495533114
ILMN_1658427	SMPD4	-0.495683973
ILMN_1687392	NRK	-0.495689272
ILMN_1801044	DOCK4	-0.495698258
ILMN_1911594	AV647465	-0.495711091
ILMN_2402463	SHMT1	-0.495716464
ILMN_3311045	MIR216A	-0.495721489

ILMN_3237368	MAFG	0.60368571
ILMN_1659727	CDAN1	0.603668155
ILMN_1708041	PLEKHF1	0.603557258
ILMN_1739558	CRELD1	0.603460465
ILMN_1690806	CR613620	0.603453994
ILMN_1811049	POU2AF1	0.603450557
ILMN_1767337	SFXN5	0.603394433
ILMN_1801101	ZBTB48	0.603331234
ILMN_2114876	RPL11	0.603318644
ILMN_1664243	USE1	0.603269604
ILMN_1782788	CSDA	0.603185596
ILMN_1781580	BRI3	0.60314657
ILMN_1673944	MANBAL	0.603115857
ILMN_2340565	ATP2C1	0.60311099
ILMN_1736623	NCKIPSD	0.602768278
ILMN_1786972	SARS	0.602647655
ILMN_1753312	PLXDC2	0.602556315
ILMN_1670456	RBM42	0.602512248
ILMN_2361163	SSBP3	0.602453977
ILMN_1812995	CTSL1	0.602318078
ILMN_1689868	TMEM80	0.602247033
ILMN_2387505	AP1B1	0.602218145
ILMN_1670398	BCR	0.602190015
ILMN_1691930	CBX6	0.602051977
ILMN_1790819	MEMO1	0.60194508
ILMN_2081269	OR2A9P	0.60192587
ILMN_2398903	HAX1	0.60173596
ILMN_1744822	BECN1	0.601725771
ILMN_1811682	CLIP2	0.601633363
ILMN_3247424	ADAP1	0.601546688
ILMN_1735333	NBR1	0.601467656
ILMN_3243381	MLEC	0.601423953
ILMN_1780153	QTRT1	0.601366167
ILMN_1688295	ZNF219	0.601348002
ILMN_2333367	FKBP1A	0.601345885
ILMN_1795183	RNASE1	0.601313435
ILMN_1655572	C6orf59	0.601236528
ILMN_1657435	MT1M	0.601142974
ILMN_1657884	NME1	0.601015529
ILMN_2394381	CLN3	0.600953166
ILMN_1691290	CELSR3	0.600932254
ILMN_1697864	CXorf38	0.600909598
ILMN_1747442	P4HA1	0.600816601
ILMN_1707748	PIM3	0.600742006
ILMN_2114568	GBP5	0.60068657
ILMN_2054392	PPIL1	0.600685305
ILMN_1740216	ERCC3	0.600643528
ILMN_1705783	NXF1	0.600628355
ILMN_1724194	NPEPL1	0.6006186
ILMN_1739335	OST4	0.600561463
ILMN_1711810	PNKD	0.600553256
ILMN_1774132	MAP6	0.600424134
ILMN_2339627	COPE	0.600410738
ILMN_1694950	MRPL28	0.600359062
ILMN_1719165	BRMS1	0.600342873
ILMN_1765257	CINP	0.600309571
ILMN_1750761	NCR1	0.600163747
ILMN_1726659	THOP1	0.60015062
ILMN_1652929	POGZ	0.599864672
ILMN_2394250	PLEKHA1	0.599834206
ILMN_1753482	FAM86C	0.59980756
ILMN_1785831	ZDHHC13	0.599785155
ILMN_1778991	NFIB	0.599686543
ILMN_1782098	SMO	0.599485382
ILMN_2141482	SERPINF1	0.599363814
ILMN_1656998	CHMP4B	0.599265175
ILMN_1778032	SURF6	0.599204428
ILMN_1706664	RIMKLA	0.59918758
ILMN_1753353	SLBP	0.599167167
ILMN_2372639	TRAPPC5	0.599159135
ILMN_3185709	IP6K2	0.59891642
ILMN_2400759	CPVL	0.59882385
ILMN_1658619	WWC1	0.598776047
ILMN_1718023	APEH	0.59870449
ILMN_1751431	WIBG	0.598693554
ILMN_2345353	LST1	0.59862278
ILMN_2075440	PDIA3P	0.598604353
ILMN_2242463	CTSC	0.598562706
ILMN_1787718	SLC27A1	0.598508998
ILMN_1786310	MVK	0.598455581
ILMN_1724407	TACC3	0.598432535
ILMN_1775692	EIF4G3	0.598287317
ILMN_1700541	FBLN1	0.59823139
ILMN_1745583	EXOC1	0.597884304
ILMN_1784005	RAB13	0.597832028
ILMN_1784141	JAKMIP1	0.597786862
ILMN_1789196	TPM2	0.597686251
ILMN_1677968	KIAA0649	0.597619344
ILMN_1652199	abParts	0.59756277
ILMN_2152581	STK38	0.597365722
ILMN_1696066	CARS	0.597360402
ILMN_2208413	ARHGAP15	0.59728746
ILMN_1811765	ADAMTS2	0.597227504
ILMN_1869109	NUCKS1	0.597162893
ILMN_1668960	MID1IP1	0.597094812
ILMN_1665865	IGFBP4	0.597056285
ILMN_2097546	NUDC	0.597029011

**Annotation Cluster 248**	**Enrichment Score: 0.47**		**Count**	**P_Value**	**Benjamini**
	UP_SEQ_FEATURE	domain:SEC7	4	3.00E-01	1.00E+00
	INTERPRO	SEC7-like	4	3.30E-01	1.00E+00
	SMART	Sec7	4	3.60E-01	9.60E-01
	GOTERM_MF_FAT	ARF guanyl-nucleotide exchange factor activity	4	3.80E-01	9.80E-01
**Annotation Cluster 249**	**Enrichment Score: 0.47**		**Count**	**P_Value**	**Benjamini**
	BIOCARTA	Role of Erk5 in Neuronal Survival	7	2.50E-02	9.70E-01
	BIOCARTA	Ras Signaling Pathway	8	7.30E-02	9.40E-01
	BIOCARTA	Inhibition of Cellular Proliferation by Gleevec	6	3.00E-01	9.60E-01
	BIOCARTA	Transcription factor CREB and its extracellular signals	5	4.70E-01	9.80E-01
	BIOCARTA	PTEN dependent cell cycle arrest and apoptosis	5	6.40E-01	9.90E-01
	BIOCARTA	Trefoil Factors Initiate Mucosal Healing	5	6.40E-01	9.90E-01
	BIOCARTA	Multiple antiapoptotic pathways from IGF-1R signaling lead to BAD phosphorylation	4	7.10E-01	1.00E+00
	BIOCARTA	Phospholipids as signalling intermediaries	4	8.50E-01	1.00E+00
	BIOCARTA	Influence of Ras and Rho proteins on G1 to S Transition	3	9.60E-01	1.00E+00
**Annotation Cluster 250**	**Enrichment Score: 0.47**		**Count**	**P_Value**	**Benjamini**
	GOTERM_MF_FAT	insulin receptor binding	7	1.40E-01	8.60E-01
	UP_SEQ_FEATURE	domain:IRS-type PTB	3	4.40E-01	1.00E+00
	INTERPRO	Insulin receptor substrate-1, PTB	3	4.60E-01	1.00E+00
	SMART	PTBI	3	4.80E-01	9.70E-01
**Annotation Cluster 251**	**Enrichment Score: 0.47**		**Count**	**P_Value**	**Benjamini**
	GOTERM_MF_FAT	DNA-directed DNA polymerase activity	7	2.20E-01	9.30E-01
	SP_PIR_KEYWORDS	dna-directed dna polymerase	5	2.40E-01	6.80E-01
	SP_PIR_KEYWORDS	nucleotidyltransferase	11	4.30E-01	8.60E-01
	GOTERM_MF_FAT	nucleotidyltransferase activity	18	4.40E-01	9.90E-01
	GOTERM_MF_FAT	DNA polymerase activity	7	4.60E-01	9.90E-01
**Annotation Cluster 252**	**Enrichment Score: 0.46**		**Count**	**P_Value**	**Benjamini**
	SP_PIR_KEYWORDS	extracellular matrix	41	2.20E-02	1.70E-01
	GOTERM_CC_FAT	proteinaceous extracellular matrix	43	5.70E-01	9.40E-01
	GOTERM_CC_FAT	extracellular matrix part	16	6.10E-01	9.50E-01
	GOTERM_CC_FAT	extracellular matrix	45	6.50E-01	9.60E-01
	GOTERM_CC_FAT	extracellular region part	108	9.90E-01	1.00E+00
**Annotation Cluster 253**	**Enrichment Score: 0.46**		**Count**	**P_Value**	**Benjamini**
	UP_SEQ_FEATURE	domain:ABC transporter 2	8	8.40E-02	1.00E+00
	UP_SEQ_FEATURE	domain:ABC transporter 1	8	8.40E-02	1.00E+00
	UP_SEQ_FEATURE	nucleotide phosphate-binding region:ATP 1	7	2.40E-01	1.00E+00
	UP_SEQ_FEATURE	nucleotide phosphate-binding region:ATP 2	7	2.40E-01	1.00E+00
	INTERPRO	ABC transporter-like	9	2.50E-01	1.00E+00
	PIR_SUPERFAMILY	PIRSF002775:human multidrug resistance protein cMOAT2	3	3.50E-01	1.00E+00
	INTERPRO	ATPase, AAA+ type, core	21	3.60E-01	1.00E+00
	INTERPRO	ABC transporter, conserved site	9	4.30E-01	1.00E+00
	SMART	AAA	21	4.40E-01	9.70E-01
	INTERPRO	ABC transporter integral membrane type 1	5	4.80E-01	1.00E+00
	UP_SEQ_FEATURE	domain:ABC transmembrane type-1 1	3	5.60E-01	1.00E+00
	UP_SEQ_FEATURE	domain:ABC transmembrane type-1 2	3	5.60E-01	1.00E+00
	INTERPRO	ABC transporter, transmembrane region	4	6.00E-01	1.00E+00
	COG_ONTOLOGY	Defense mechanisms	3	6.90E-01	9.80E-01
	KEGG_PATHWAY	ABC transporters	6	8.10E-01	9.40E-01
**Annotation Cluster 254**	**Enrichment Score: 0.46**		**Count**	**P_Value**	**Benjamini**
	GOTERM_BP_FAT	cell fate specification	11	1.30E-01	7.50E-01
	GOTERM_BP_FAT	cell-cell signaling involved in cell fate specification	4	4.70E-01	9.80E-01
	GOTERM_BP_FAT	developmental induction	4	4.70E-01	9.80E-01
	GOTERM_BP_FAT	induction of an organ	3	5.30E-01	9.90E-01
**Annotation Cluster 255**	**Enrichment Score: 0.46**		**Count**	**P_Value**	**Benjamini**
	GOTERM_BP_FAT	regulation of interleukin-10 production	4	1.70E-01	8.20E-01
	GOTERM_BP_FAT	positive regulation of interferon-gamma production	3	4.40E-01	9.80E-01
	GOTERM_BP_FAT	regulation of interferon-gamma production	5	5.70E-01	9.90E-01
**Annotation Cluster 256**	**Enrichment Score: 0.46**		**Count**	**P_Value**	**Benjamini**
	UP_SEQ_FEATURE	domain:CUE	3	3.00E-01	1.00E+00
	INTERPRO	Ubiquitin system component Cue	3	3.70E-01	1.00E+00
	SMART	CUE	3	3.90E-01	9.70E-01
**Annotation Cluster 257**	**Enrichment Score: 0.46**		**Count**	**P_Value**	**Benjamini**
	GOTERM_BP_FAT	pentose-phosphate shunt	4	1.00E-01	7.00E-01
	GOTERM_BP_FAT	oxidoreduction coenzyme metabolic process	10	2.40E-01	9.00E-01
	GOTERM_BP_FAT	nicotinamide nucleotide metabolic process	8	2.70E-01	9.20E-01
	GOTERM_BP_FAT	nicotinamide metabolic process	8	2.70E-01	9.20E-01
	GOTERM_BP_FAT	alkaloid metabolic process	8	2.90E-01	9.30E-01
	GOTERM_BP_FAT	pyridine nucleotide metabolic process	8	3.10E-01	9.40E-01
	GOTERM_BP_FAT	cofactor metabolic process	29	3.40E-01	9.50E-01
	GOTERM_BP_FAT	NADP metabolic process	4	3.50E-01	9.60E-01
	GOTERM_BP_FAT	cellular amide metabolic process	9	4.60E-01	9.80E-01
	GOTERM_BP_FAT	coenzyme metabolic process	20	6.50E-01	1.00E+00
	GOTERM_BP_FAT	NAD metabolic process	4	6.60E-01	1.00E+00
	GOTERM_BP_FAT	secondary metabolic process	9	8.30E-01	1.00E+00
**Annotation Cluster 258**	**Enrichment Score: 0.45**		**Count**	**P_Value**	**Benjamini**
	GOTERM_BP_FAT	cell cycle checkpoint	16	2.10E-01	8.80E-01
	GOTERM_BP_FAT	DNA damage response, signal transduction	14	2.50E-01	9.10E-01
	GOTERM_BP_FAT	DNA damage checkpoint	9	3.00E-01	9.30E-01
	GOTERM_BP_FAT	DNA integrity checkpoint	9	3.80E-01	9.60E-01
	GOTERM_BP_FAT	G2/M transition DNA damage checkpoint	3	5.30E-01	9.90E-01
	GOTERM_BP_FAT	positive regulation of DNA repair	3	6.10E-01	1.00E+00
**Annotation Cluster 259**	**Enrichment Score: 0.45**		**Count**	**P_Value**	**Benjamini**
	SP_PIR_KEYWORDS	calmodulin-binding	19	1.90E-01	6.30E-01
	GOTERM_MF_FAT	calmodulin binding	21	3.40E-01	9.70E-01
	UP_SEQ_FEATURE	region of interest:Calmodulin-binding	7	6.70E-01	1.00E+00
**Annotation Cluster 260**	**Enrichment Score: 0.45**		**Count**	**P_Value**	**Benjamini**
	GOTERM_BP_FAT	endothelial cell development	3	2.30E-01	8.90E-01
	GOTERM_BP_FAT	endothelial cell differentiation	4	3.50E-01	9.60E-01
	GOTERM_BP_FAT	epithelial cell development	4	5.40E-01	9.90E-01
**Annotation Cluster 261**	**Enrichment Score: 0.45**		**Count**	**P_Value**	**Benjamini**
	GOTERM_BP_FAT	ceramide metabolic process	9	1.80E-01	8.40E-01
	GOTERM_BP_FAT	glycolipid metabolic process	7	2.20E-01	8.80E-01
	GOTERM_BP_FAT	sphingoid metabolic process	9	2.40E-01	8.90E-01
	GOTERM_BP_FAT	glycosphingolipid metabolic process	6	2.50E-01	9.10E-01
	GOTERM_BP_FAT	membrane lipid metabolic process	14	2.70E-01	9.20E-01
	GOTERM_BP_FAT	sphingolipid metabolic process	13	2.80E-01	9.20E-01
	GOTERM_BP_FAT	sphingolipid biosynthetic process	6	3.90E-01	9.70E-01

ILMN_1678968	GEM	-0.49583411
ILMN_2128489	UQCRB	-0.495862925
ILMN_1779951	ANKRD7	-0.495895963
ILMN_1801143	LOXHD1	-0.495950648
ILMN_1792931	LYSMD1	-0.496070999
ILMN_1882560	NA	-0.496119898
ILMN_1652439	KRTAP3-1	-0.496200121
ILMN_1777657	SLC22A9	-0.496237243
ILMN_1811696	FDXR	-0.496269776
ILMN_2351262	MAPK8IP2	-0.496279996
ILMN_3305273	PSAT1	-0.496465607
ILMN_3309664	MIR300	-0.496497568
ILMN_1775646	L3MBTL4	-0.496570073
ILMN_1806655	ARHGAP6	-0.496658217
ILMN_1767649	PCDHGC3	-0.496692056
ILMN_1768205	SLFN12L	-0.496734832
ILMN_2275060	GHRHR	-0.496769983
ILMN_1771898	NBPF3	-0.49683929
ILMN_1654063	HYDIN	-0.496866963
ILMN_1874172	BX097339	-0.496884903
ILMN_3306028	BM932296	-0.497038992
ILMN_1651752	CXorf21	-0.497043976
ILMN_1779997	MEIS3	-0.497058848
ILMN_1712461	CBLN1	-0.497083712
ILMN_1669015	XPNPEP1	-0.497095622
ILMN_1730117	TMC5	-0.497099582
ILMN_1802146	ZNF74	-0.497124265
ILMN_2200331	H2AFX	-0.497161221
ILMN_1810115	KCTD2	-0.497168956
ILMN_1701881	ZNF365	-0.497180095
ILMN_1793231	BF056868	-0.49726785
ILMN_1669940	TMEM38B	-0.497279877
ILMN_1769752	FER1L5	-0.49731926
ILMN_1847029	CX787363	-0.497337425
ILMN_3307818	C9orf106	-0.497353678
ILMN_3240524	MFSD6	-0.497381559
ILMN_1789500	KIAA1875	-0.497394456
ILMN_1760180	MID2	-0.497448803
ILMN_1815051	API5	-0.497524899
ILMN_1652023	AGFG2	-0.49752636
ILMN_1679647	CHIA	-0.497549809
ILMN_1766320	IL3	-0.497600278
ILMN_1796595	C5orf22	-0.497606242
ILMN_1677880	MYADML2	-0.497620725
ILMN_2292863	RASA1	-0.49769163
ILMN_1837935	TNPO1	-0.497698456
ILMN_1799259	RASSF2	-0.497736154
ILMN_2372398	ALDH5A1	-0.497831697
ILMN_1653429	SLC35A3	-0.497861577
ILMN_1817816	BC032017	-0.497863425
ILMN_1680223	PNPLA8	-0.497874574
ILMN_1811363	NOVA1	-0.497953127
ILMN_2122669	TOPORS	-0.497986294
ILMN_1810628	PRUNE2	-0.498011004
ILMN_1787648	GPR125	-0.498110341
ILMN_3277152	TAS2R19	-0.498216763
ILMN_1888330	AI809531	-0.49823388
ILMN_1761072	AIDA	-0.498258196
ILMN_1813055	LOC401093	-0.498320978
ILMN_1890924	BX094420	-0.498340873
ILMN_1665730	ABCB10	-0.49845364
ILMN_1754132	FLJ45079	-0.498506496
ILMN_1801098	TSLP	-0.498636424
ILMN_3241953	A2LD1	-0.498704586
ILMN_1754304	SNRNP48	-0.498709153
ILMN_1727134	KLHDC5	-0.498722239
ILMN_1680754	A4GNT	-0.498740105
ILMN_2212156	PPIG	-0.498827256
ILMN_1862684	BQ225423	-0.498859517
ILMN_1693273	AK127961	-0.498864069
ILMN_1682224	MUC3A	-0.498944122
ILMN_1883982	KSR2	-0.498952769
ILMN_1668285	APBA1	-0.498954249
ILMN_1728734	PSG5	-0.498995267
ILMN_1765198	HEATR4	-0.499011374
ILMN_1810319	TTY7	-0.49901487
ILMN_1668657	C2orf69	-0.499078059
ILMN_2394366	FAM123A	-0.49908604
ILMN_1880704	AA484452	-0.49908693
ILMN_2130411	KDELR1	-0.499111369
ILMN_1753745	HDDC2	-0.499136784
ILMN_3283960	BC046381	-0.499165908
ILMN_1707258	UCN	-0.499198496
ILMN_1696127	KIAA0240	-0.499269975
ILMN_3182712	NA	-0.499392206
ILMN_1882000	TXLNG	-0.499400887
ILMN_2173740	ASB8	-0.499447764
ILMN_1729868	SPRYD4	-0.499449907
ILMN_1681008	CGRRF1	-0.499567087
ILMN_3296181	AX747752	-0.499615619
ILMN_3262348	IP6K2	-0.499683462
ILMN_1767837	GOLT1B	-0.499739895
ILMN_1691705	ZNF98	-0.499789902
ILMN_1699606	ASB3	-0.499801177
ILMN_1676296	PPAP2A	-0.49983008
ILMN_1753026	LPPR1	-0.499849499
ILMN_2080760	SNX22	-0.499888766

ILMN_3182762	RPL21	0.596797538
ILMN_1658231	NACC1	0.596748414
ILMN_1703263	SP140	0.596726389
ILMN_1720053	ZFAND3	0.596671506
ILMN_1698491	MBD3	0.596636286
ILMN_1664464	PTGDS	0.596546765
ILMN_1653861	SCMH1	0.596542778
ILMN_2384133	DFFB	0.596467807
ILMN_1785618	SMTN	0.596417042
ILMN_1733932	SNUPN	0.596348252
ILMN_2211122	TRAPPC2	0.596258973
ILMN_1724341	ALG13	0.596258318
ILMN_1659240	MTMR14	0.596196159
ILMN_3238803	RASAL3	0.596116657
ILMN_2229922	C12orf35	0.595954254
ILMN_1675106	YIPF2	0.595751496
ILMN_1691436	BLVRA	0.595695716
ILMN_2392635	ABCF1	0.595633076
ILMN_1659769	MRAP	0.595605399
ILMN_1732921	ITGB8	0.595445735
ILMN_1655195	SMA4	0.595224509
ILMN_1794912	ATP5H	0.595189682
ILMN_1669966	NDUFS7	0.595163172
ILMN_1750321	TRANK1	0.595124349
ILMN_1697417	CEP170	0.595078471
ILMN_1776119	ABCC10	0.595068996
ILMN_1755710	EFNA4	0.594975865
ILMN_2399190	TNFSF13	0.594930027
ILMN_1654893	SCAMP2	0.594893003
ILMN_1769671	RYK	0.594821577
ILMN_1783843	MIIP	0.594771603
ILMN_1725612	NUP50	0.594726005
ILMN_2359456	ERGIC3	0.594530774
ILMN_1656310	IDO1	0.594441505
ILMN_1657983	TERF2IP	0.594430348
ILMN_3229467	EBPL	0.594381302
ILMN_1693762	LRRC48	0.594349265
ILMN_1808707	FSCN1	0.594316489
ILMN_1720373	SLC7A5	0.59429729
ILMN_1748651	PSMB3	0.594245679
ILMN_1738263	PIGU	0.593881209
ILMN_3234837	PKDCC	0.593854248
ILMN_1675848	MYL12A	0.593763397
ILMN_1681798	sept-06	0.593757192
ILMN_3247679	SNORA52	0.593637355
ILMN_3274666	CR601848	0.593615661
ILMN_1703228	AGFG2	0.593588092
ILMN_2094718	TPT1	0.59357182
ILMN_1660817	DDB2	0.593493035
ILMN_1782352	VENTX	0.593444383
ILMN_1900123	BX105859	0.593358623
ILMN_2220739	TMCO3	0.593357565
ILMN_2328433	NOP2	0.593329782
ILMN_1707503	C1orf144	0.593307181
ILMN_1733603	NDUFC1	0.593216444
ILMN_1676256	TPSAB1	0.593166469
ILMN_1725241	GSTK1	0.593042652
ILMN_1746578	SLC23A2	0.592968599
ILMN_1739345	C11orf48	0.592918181
ILMN_1813491	SPTLC1	0.592915828
ILMN_2109536	GOLGA8F	0.592861775
ILMN_1789733	CLIP3	0.592800721
ILMN_1711023	CDK5RAP1	0.592665156
ILMN_1771815	C20orf43	0.592601965
ILMN_1778319	DMKN	0.592557893
ILMN_1753805	PRKD2	0.592528237
ILMN_2330806	MYO18A	0.592502312
ILMN_1678966	SNRPF	0.592392402
ILMN_1681103	AQP1	0.592354606
ILMN_1810680	BOLA2B	0.592345175
ILMN_1700081	FST	0.592324328
ILMN_3247064	SNRNP40	0.592299215
ILMN_1659610	TJP3	0.591983311
ILMN_1814402	NFIC	0.591843276
ILMN_1802338	HHLA3	0.591831182
ILMN_1815366	ENGASE	0.591751019
ILMN_1678766	DYNLT1	0.591737109
ILMN_2339266	LAMA2	0.591735944
ILMN_2366391	PRDX1	0.591712015
ILMN_3179371	HNRNPK	0.591651785
ILMN_1656718	DEF8	0.591648181
ILMN_1661335	SPTBN1	0.591532043
ILMN_1727574	ZNF827	0.591502905
ILMN_1692398	CNTNAP1	0.591488393
ILMN_1769545	PRPF19	0.591463425
ILMN_1700203	KIAA1984	0.591462112
ILMN_1713901	KDELR3	0.591295624
ILMN_1682227	ZNF404	0.591250389
ILMN_1721113	HLA-C	0.591187187
ILMN_1651735	TGOLN2	0.591168427
ILMN_2255579	RAB37	0.591168297
ILMN_2048326	RPS27A	0.591032097
ILMN_1817239	DA160411	0.590962137
ILMN_3311070	MIR33B	0.590948731

	GOTERM_BP_FAT	sphingomyelin metabolic process	3	4.40E-01	9.80E-01
	GOTERM_BP_FAT	membrane lipid biosynthetic process	6	4.70E-01	9.80E-01
	GOTERM_BP_FAT	ceramide biosynthetic process	3	6.10E-01	1.00E+00
	GOTERM_BP_FAT	sphingoid biosynthetic process	3	6.40E-01	1.00E+00
	KEGG_PATHWAY	Sphingolipid metabolism	6	7.20E-01	9.00E-01
**Annotation Cluster 262**	**Enrichment Score: 0.45**		**Count**	**P_Value**	**Benjamini**
	GOTERM_CC_FAT	Srb-mediator complex	7	1.80E-01	6.50E-01
	GOTERM_MF_FAT	RNA polymerase II transcription mediator activity	6	3.10E-01	9.70E-01
	GOTERM_MF_FAT	general RNA polymerase II transcription factor activity	6	7.90E-01	1.00E+00
**Annotation Cluster 263**	**Enrichment Score: 0.44**		**Count**	**P_Value**	**Benjamini**
	BIOCARTA	Synaptic Proteins at the Synaptic Junction	7	1.20E-01	9.00E-01
	UP_SEQ_FEATURE	domain:Guanylate kinase-like	5	3.00E-01	1.00E+00
	INTERPRO	Guanylate kinase	5	3.30E-01	1.00E+00
	INTERPRO	Guanylate kinase/L-type calcium channel region	5	4.20E-01	1.00E+00
	UP_SEQ_FEATURE	domain:PDZ 3	5	4.40E-01	1.00E+00
	SMART	GuKc	5	4.50E-01	9.70E-01
	UP_SEQ_FEATURE	domain:PDZ 1	6	5.40E-01	1.00E+00
	UP_SEQ_FEATURE	domain:PDZ 2	6	5.40E-01	1.00E+00
**Annotation Cluster 264**	**Enrichment Score: 0.44**		**Count**	**P_Value**	**Benjamini**
	BIOCARTA	Role of Erk5 in Neuronal Survival	7	2.50E-02	9.70E-01
	BIOCARTA	The IGF-1 Receptor and Longevity	5	1.50E-01	9.30E-01
	BIOCARTA	IL-2 Receptor Beta Chain in T cell Activation	10	1.70E-01	9.30E-01
	BIOCARTA	Regulation of BAD phosphorylation	6	2.60E-01	9.40E-01
	BIOCARTA	Inhibition of Cellular Proliferation by Gleevec	6	3.00E-01	9.60E-01
	BIOCARTA	Control of skeletal myogenesis by HDAC & calcium/calmodulin-dependent kinase (CaMK)	5	4.20E-01	9.70E-01
	BIOCARTA	Growth Hormone Signaling Pathway	6	4.30E-01	9.70E-01
	BIOCARTA	Human Cytomegalovirus and Map Kinase Pathways	4	5.30E-01	9.80E-01
	BIOCARTA	Trefoil Factors Initiate Mucosal Healing	5	6.40E-01	9.90E-01
	KEGG_PATHWAY	mTOR signaling pathway	7	8.10E-01	9.30E-01
	BIOCARTA	Phospholipids as signalling intermediaries	4	8.50E-01	1.00E+00
	BIOCARTA	NFAT and Hypertrophy of the heart (Transcription in the broken heart)	5	9.50E-01	1.00E+00
	KEGG_PATHWAY	Melanoma	7	9.70E-01	9.90E-01
**Annotation Cluster 265**	**Enrichment Score: 0.44**		**Count**	**P_Value**	**Benjamini**
	UP_SEQ_FEATURE	zinc finger region:RanBP2-type	4	3.40E-01	1.00E+00
	INTERPRO	Zinc finger, RanBP2-type	5	3.60E-01	1.00E+00
	SMART	ZnF_RBZ	5	3.90E-01	9.60E-01
**Annotation Cluster 266**	**Enrichment Score: 0.44**		**Count**	**P_Value**	**Benjamini**
	UP_SEQ_FEATURE	active site:S-palmitoyl cysteine intermediate	3	3.00E-01	1.00E+00
	UP_SEQ_FEATURE	zinc finger region:DHHC-type	5	3.90E-01	1.00E+00
	INTERPRO	Zinc finger, DHHC-type	5	4.20E-01	1.00E+00
**Annotation Cluster 267**	**Enrichment Score: 0.44**		**Count**	**P_Value**	**Benjamini**
	INTERPRO	S100/CaBP-9k-type, calcium binding, subdomain	6	2.50E-01	1.00E+00
	PIR_SUPERFAMILY	PIRSF002353:S-100 protein	5	2.50E-01	1.00E+00
	INTERPRO	S100/CaBP-9k-type, calcium binding	6	2.70E-01	1.00E+00
	SP_PIR_KEYWORDS	EF hand	10	2.90E-01	7.50E-01
	UP_SEQ_FEATURE	calcium-binding region:1; low affinity	3	6.60E-01	1.00E+00
	UP_SEQ_FEATURE	calcium-binding region:2; high affinity	3	7.40E-01	1.00E+00
**Annotation Cluster 268**	**Enrichment Score: 0.43**		**Count**	**P_Value**	**Benjamini**
	BIOCARTA	Roles of ?-arrestin-dependent Recruitment of Src Kinases in GPCR Signaling	6	2.60E-01	9.40E-01
	BIOCARTA	Role of ?-arrestins in the activation and targeting of MAP kinases	5	2.80E-01	9.50E-01
	BIOCARTA	?-arrestins in GPCR Desensitization	3	7.10E-01	1.00E+00
**Annotation Cluster 269**	**Enrichment Score: 0.43**		**Count**	**P_Value**	**Benjamini**
	GOTERM_MF_FAT	CARD domain binding	4	5.00E-02	6.10E-01
	UP_SEQ_FEATURE	domain:CARD	4	7.00E-01	1.00E+00
	INTERPRO	Caspase Recruitment	4	7.30E-01	1.00E+00
	SMART	CARD	4	7.60E-01	1.00E+00
**Annotation Cluster 270**	**Enrichment Score: 0.43**		**Count**	**P_Value**	**Benjamini**
	SP_PIR_KEYWORDS	zymogen	35	3.50E-02	2.30E-01
	GOTERM_BP_FAT	proteolysis	144	3.70E-01	9.60E-01
	SP_PIR_KEYWORDS	Protease	60	4.80E-01	8.80E-01
	GOTERM_MF_FAT	endopeptidase activity	50	4.80E-01	9.90E-01
	GOTERM_MF_FAT	serine-type peptidase activity	24	5.20E-01	9.90E-01
	GOTERM_MF_FAT	serine hydrolase activity	24	5.50E-01	1.00E+00
	GOTERM_MF_FAT	peptidase activity, acting on L-amino acid peptides	69	6.50E-01	1.00E+00
	GOTERM_MF_FAT	peptidase activity	72	6.60E-01	1.00E+00
**Annotation Cluster 271**	**Enrichment Score: 0.43**		**Count**	**P_Value**	**Benjamini**
	GOTERM_BP_FAT	endochondral bone morphogenesis	5	1.50E-01	7.90E-01
	GOTERM_BP_FAT	bone morphogenesis	5	2.70E-01	9.20E-01
	GOTERM_BP_FAT	regulation of chondrocyte differentiation	4	2.80E-01	9.20E-01
	GOTERM_BP_FAT	endochondral ossification	3	4.80E-01	9.90E-01
	GOTERM_BP_FAT	cartilage development	10	6.50E-01	1.00E+00
	GOTERM_BP_FAT	bone development	15	7.60E-01	1.00E+00
**Annotation Cluster 272**	**Enrichment Score: 0.43**		**Count**	**P_Value**	**Benjamini**
	PIR_SUPERFAMILY	PIRSF002583:heat shock protein, HSP90/HTPG types	3	2.40E-01	1.00E+00
	INTERPRO	ATP-binding region, ATPase-like	6	3.30E-01	1.00E+00
	SMART	HATPase_c	6	3.60E-01	9.60E-01
	INTERPRO	Heat shock protein Hsp90	3	4.60E-01	1.00E+00
	SP_PIR_KEYWORDS	stress response	9	5.50E-01	9.20E-01
**Annotation Cluster 273**	**Enrichment Score: 0.43**		**Count**	**P_Value**	**Benjamini**
	GOTERM_BP_FAT	negative regulation of protein kinase cascade	7	3.40E-01	9.50E-01
	GOTERM_BP_FAT	negative regulation of JNK cascade	3	3.90E-01	9.70E-01
	GOTERM_BP_FAT	negative regulation of stress-activated protein kinase signaling pathway	3	3.90E-01	9.70E-01
	GOTERM_BP_FAT	negative regulation of MAPKKK cascade	4	3.90E-01	9.70E-01
**Annotation Cluster 274**	**Enrichment Score: 0.42**		**Count**	**P_Value**	**Benjamini**
	INTERPRO	Synaptobrevin, metazoa/fungi	3	1.70E-01	9.90E-01
	PIR_SUPERFAMILY	PIRSF005409:Synaptobrevin_euk	3	1.90E-01	1.00E+00
	UP_SEQ_FEATURE	domain:v-SNARE coiled-coil homology	3	4.40E-01	1.00E+00
	INTERPRO	Synaptobrevin	3	4.60E-01	1.00E+00
	KEGG_PATHWAY	SNARE interactions in vesicular transport	7	5.20E-01	8.20E-01
	UP_SEQ_FEATURE	topological domain:Vesicular	4	8.70E-01	1.00E+00
**Annotation Cluster 275**	**Enrichment Score: 0.42**		**Count**	**P_Value**	**Benjamini**
	GOTERM_BP_FAT	alpha-beta T cell differentiation	4	2.80E-01	9.20E-01
	GOTERM_BP_FAT	alpha-beta T cell activation	4	4.30E-01	9.80E-01
	GOTERM_BP_FAT	positive regulation of cytokine biosynthetic process	8	4.50E-01	9.80E-01

ILMN_1740305	DLEC1	-0.500020439
ILMN_1731326	PRR23C	-0.500174805
ILMN_2390318	CIDEA	-0.500190252
ILMN_1652037	UPP2	-0.500306247
ILMN_1709112	CHM	-0.500334664
ILMN_1656939	LOC400099	-0.500350912
ILMN_1793433	RAB10	-0.500374101
ILMN_3235340	ACER2	-0.500429574
ILMN_1725910	CNTFR	-0.500460777
ILMN_2389080	IL4	-0.500470675
ILMN_1707965	LOC100192378	-0.500476814
ILMN_1728803	HMP19	-0.500650058
ILMN_1744102	AK128005	-0.50076465
ILMN_1750092	SEPSECS	-0.500796234
ILMN_2249677	LSR	-0.500799466
ILMN_2288285	CDY1B	-0.500821579
ILMN_1757807	AGXT2L1	-0.500946964
ILMN_1910948	AK091312	-0.500962313
ILMN_1716524	RAB7A	-0.500969516
ILMN_1677311	AW665104	-0.501079047
ILMN_1904256	CR736208	-0.501175829
ILMN_1860487	DA196703	-0.50126068
ILMN_1703092	RECQL4	-0.501289048
ILMN_1658675	CDH23	-0.501301003
ILMN_3237044	SNORD36B	-0.501308836
ILMN_1889144	BI047056	-0.501335765
ILMN_1681612	DMBT1	-0.501355145
ILMN_2362293	FBXO38	-0.501368591
ILMN_1720838	DECR1	-0.501409463
ILMN_1751636	ANKS3	-0.501414166
ILMN_1733736	DKFZp434O171	-0.501418873
ILMN_3261382	TMEM221	-0.501427898
ILMN_1722753	GJA5	-0.501446578
ILMN_3301607	C13orf18	-0.50145178
ILMN_1763408	CCBL2	-0.501464134
ILMN_1809456	CNTFR	-0.501489139
ILMN_2178116	MMP20	-0.501491544
ILMN_1733675	MPP1	-0.501546967
ILMN_3238854	RGPD8	-0.50156417
ILMN_1811178	SCAPER	-0.50162557
ILMN_1677734	WFDC9	-0.501635867
ILMN_1678437	FRY	-0.501709647
ILMN_2126055	ASB5	-0.501799086
ILMN_1720998	CA12	-0.501938692
ILMN_1800253	ZNF711	-0.502077217
ILMN_2372316	PKD1L2	-0.502252429
ILMN_1651966	FGFR3	-0.502280151
ILMN_2187830	CCNH	-0.502367249
ILMN_1780894	ANTXR1	-0.502368544
ILMN_1767503	GABRB3	-0.502384548
ILMN_1793367	LOC729966	-0.502430667
ILMN_1704134	NA	-0.502468582
ILMN_1737152	DAO	-0.502471485
ILMN_1796240	TRAPPC8	-0.502484582
ILMN_1788005	PLEC	-0.502554764
ILMN_1681367	ZNF642	-0.502606215
ILMN_1731644	SETDB2	-0.502630714
ILMN_1849206	DA724733	-0.50286743
ILMN_1808715	LOC389300	-0.502884306
ILMN_2153029	CGB5	-0.502896108
ILMN_1687589	CPT1A	-0.502970028
ILMN_1678493	CHN1	-0.502990482
ILMN_1794890	SLC38A4	-0.503000428
ILMN_1743131	TOX4	-0.503080498
ILMN_1704354	NCDN	-0.503166276
ILMN_1865078	AL832552	-0.503187368
ILMN_3292840	NA	-0.503234567
ILMN_1849134	BQ012728	-0.503244928
ILMN_1789395	OR9G1	-0.503280638
ILMN_1799985	C13orf40	-0.503298404
ILMN_1778617	TAF9	-0.503302636
ILMN_1684114	LOC286016	-0.50334899
ILMN_3250879	ANGPT2	-0.50342157
ILMN_1771019	MTMR4	-0.503485974
ILMN_1732127	RBKS	-0.503542201
ILMN_1715476	CKMT1B	-0.503558061
ILMN_1717869	BF355127	-0.503581393
ILMN_1695378	SREBF1	-0.503588982
ILMN_3232949	FAM71E2	-0.503604646
ILMN_3308515	SNORD116-13	-0.503673201
ILMN_1726327	AMY1A	-0.503719381
ILMN_1761946	PROM2	-0.503744174
ILMN_2406106	CACNB2	-0.503758297
ILMN_1748147	MTO1	-0.50376076
ILMN_3202710	AK126532	-0.50379613
ILMN_3251375	WBP11P1	-0.503904017
ILMN_1739751	SLC26A11	-0.503933397
ILMN_1821352	BX096603	-0.503971916
ILMN_2128358	TPP2	-0.503983841
ILMN_1736490	BC156836	-0.504065871
ILMN_1677845	RTL1	-0.504179241
ILMN_2193980	ABCB6	-0.504210152
ILMN_1678919	YOD1	-0.504221555
ILMN_1888558	AI378936	-0.504317515

ILMN_1753345	SCAMP5	0.590758952
ILMN_1741674	PPP1R9A	0.590629022
ILMN_1652445	RAC1	0.590518176
ILMN_1713985	MAF1	0.590351914
ILMN_2390472	TTC14	0.590257967
ILMN_1751028	SERPINH1	0.590117785
ILMN_1703564	DYNLRB1	0.590117538
ILMN_1669286	YWHAZ	0.59004677
ILMN_1806408	ACADVL	0.590043141
ILMN_3297644	TMEM214	0.590041971
ILMN_1757757	TM6SF2	0.590019798
ILMN_1805796	FLYWCH2	0.589967161
ILMN_2355549	GSTT2B	0.589948059
ILMN_1794726	RNF167	0.589934921
ILMN_3231621	LOC349114	0.5898661
ILMN_2221808	KIAA1647	0.589782298
ILMN_1679178	ATP5D	0.589758601
ILMN_1682099	TNFAIP8L3	0.589710944
ILMN_2379718	RAB24	0.589480592
ILMN_1739618	ZNF408	0.589416408
ILMN_1690034	SOD3	0.589350703
ILMN_1752285	RPL4	0.589153595
ILMN_2352574	ZNF274	0.589152035
ILMN_1793672	SIX5	0.588906287
ILMN_1795400	TBCD	0.58875539
ILMN_1796712	S100A10	0.588572803
ILMN_1754579	SYNE2	0.588513368
ILMN_1793017	DGKQ	0.588422207
ILMN_1687538	ETS1	0.588272491
ILMN_2403889	PRMT5	0.588156371
ILMN_3226467	AK023375	0.588112626
ILMN_1669842	CHAF1A	0.588112356
ILMN_3225634	RPL21	0.588027769
ILMN_2087080	RPL5	0.588005479
ILMN_1771697	VRK3	0.587932698
ILMN_1757914	C19orf56	0.587768615
ILMN_1757370	SMPD1	0.587614508
ILMN_1692849	ANKRD58	0.587599521
ILMN_1774259	FAM63B	0.587582795
ILMN_1653718	ZFAND2B	0.58757834
ILMN_1675612	BLCAP	0.587577099
ILMN_1699711	DEDD	0.587552608
ILMN_2368713	UBE2V1	0.587546277
ILMN_3238018	NA	0.587534546
ILMN_1663390	CDC20	0.58741237
ILMN_1751803	LSM10	0.587321385
ILMN_1656042	KIAA0319L	0.587303036
ILMN_1735594	CDC42SE2	0.587123994
ILMN_3237946	PXDN	0.587091132
ILMN_1701875	ZYX	0.587077588
ILMN_1757019	NTN4	0.586987923
ILMN_1700674	RPL23AP53	0.586936042
ILMN_2339655	MAPK7	0.586890573
ILMN_1709042	RBM4	0.586240268
ILMN_1806733	COL18A1	0.586231118
ILMN_2399208	SCAMP3	0.586092806
ILMN_2333594	SUMO2	0.586066717
ILMN_1805200	DNM1	0.585722076
ILMN_1756126	STUB1	0.585716818
ILMN_1747499	EMID1	0.585677357
ILMN_1705602	KLHL17	0.585496213
ILMN_2261600	FCGR1B	0.585489826
ILMN_3239284	B9D1	0.585467467
ILMN_1675048	IGF1R	0.585431802
ILMN_1740505	SGMS1	0.585396633
ILMN_1696420	BRD7	0.585115196
ILMN_1779015	ZNF467	0.585022866
ILMN_1762608	EPG5	0.584995895
ILMN_2369018	EVI2A	0.584911703
ILMN_1682312	CYBB	0.584603609
ILMN_1775304	DNAJB1	0.584494301
ILMN_1754179	AP1G2	0.58446575
ILMN_1652331	LOC652276	0.584330705
ILMN_1657771	CRTC2	0.584320811
ILMN_1700047	ALAS1	0.584320176
ILMN_3306742	SIGMAR1	0.584257475
ILMN_3237966	KIAA1004	0.584178941
ILMN_1728009	TMEM171	0.584157147
ILMN_1712639	AIFM2	0.584127333
ILMN_1765833	SLC9A3R2	0.584054185
ILMN_1722622	CD163	0.584036946
ILMN_1808712	RNF40	0.584013114
ILMN_1747504	AHCTF1	0.583885626
ILMN_1695821	ACTR1B	0.583828644
ILMN_1709953	DONSON	0.583715903
ILMN_2336728	DLG3	0.58366225
ILMN_3241756	FAM136A	0.583661301
ILMN_1741404	MSC	0.583638945
ILMN_3250147	ZNF626	0.583619525
ILMN_2049417	TMEM86B	0.583571334
ILMN_2391931	SLC35A2	0.583469731
ILMN_1691736	ST6GALNAC6	0.583441936
ILMN_2406873	ARFGAP1	0.583440004
ILMN_1684183	RAD9A	0.583268412
ILMN_1774596	BSCL2	0.583261012

**Annotation Cluster 276**	**Enrichment Score: 0.42**		**Count**	**P_Value**	**Benjamini**
	GOTERM_BP_FAT	positive regulation of endocytosis	7	1.70E-01	8.30E-01
	GOTERM_BP_FAT	positive regulation of phagocytosis	3	4.80E-01	9.90E-01
	GOTERM_BP_FAT	regulation of phagocytosis	3	6.40E-01	1.00E+00
**Annotation Cluster 277**	**Enrichment Score: 0.42**		**Count**	**P_Value**	**Benjamini**
	SP_PIR_KEYWORDS	selenium	6	3.20E-01	7.70E-01
	GOTERM_MF_FAT	selenium binding	6	3.90E-01	9.80E-01
	SP_PIR_KEYWORDS	selenocysteine	5	4.40E-01	8.60E-01
**Annotation Cluster 278**	**Enrichment Score: 0.42**		**Count**	**P_Value**	**Benjamini**
	UP_SEQ_FEATURE	repeat:3-3	3	1.60E-01	1.00E+00
	UP_SEQ_FEATURE	repeat:3-2	3	2.00E-01	1.00E+00
	UP_SEQ_FEATURE	repeat:3-1	3	2.00E-01	1.00E+00
	UP_SEQ_FEATURE	repeat:1-2	5	3.90E-01	1.00E+00
	UP_SEQ_FEATURE	repeat:1-1	5	4.20E-01	1.00E+00
	UP_SEQ_FEATURE	repeat:2-1	4	6.00E-01	1.00E+00
	UP_SEQ_FEATURE	repeat:1-4	3	6.30E-01	1.00E+00
	UP_SEQ_FEATURE	repeat:2-2	4	6.50E-01	1.00E+00
	UP_SEQ_FEATURE	repeat:1-3	3	6.60E-01	1.00E+00
**Annotation Cluster 279**	**Enrichment Score: 0.42**		**Count**	**P_Value**	**Benjamini**
	INTERPRO	Single-stranded nucleic acid binding R3H	3	3.70E-01	1.00E+00
	SMART	R3H	3	3.90E-01	9.70E-01
	UP_SEQ_FEATURE	domain:R3H	3	3.90E-01	1.00E+00
**Annotation Cluster 280**	**Enrichment Score: 0.42**		**Count**	**P_Value**	**Benjamini**
	GOTERM_BP_FAT	immunoglobulin production	7	2.00E-01	8.60E-01
	GOTERM_BP_FAT	production of molecular mediator of immune response	7	2.20E-01	8.80E-01
	GOTERM_BP_FAT	somatic diversification of immune receptors	6	2.80E-01	9.30E-01
	GOTERM_BP_FAT	somatic diversification of immunoglobulins	5	3.30E-01	9.50E-01
	GOTERM_BP_FAT	somatic hypermutation of immunoglobulin genes	3	3.40E-01	9.50E-01
	GOTERM_BP_FAT	somatic diversification of immune receptors via somatic mutation	3	3.40E-01	9.50E-01
	GOTERM_BP_FAT	immunoglobulin production during immune response	3	5.30E-01	9.90E-01
	GOTERM_BP_FAT	somatic cell DNA recombination	4	6.30E-01	1.00E+00
	GOTERM_BP_FAT	somatic diversification of immune receptors via germline recombination within a single locus	4	6.30E-01	1.00E+00
	GOTERM_BP_FAT	somatic recombination of immunoglobulin gene segments	3	7.10E-01	1.00E+00
**Annotation Cluster 281**	**Enrichment Score: 0.42**		**Count**	**P_Value**	**Benjamini**
	INTERPRO	26S proteasome subunit P45	3	2.20E-01	1.00E+00
	INTERPRO	ATPase, AAA-type, conserved site	6	4.30E-01	1.00E+00
	INTERPRO	ATPase, AAA-type, core	7	6.00E-01	1.00E+00
**Annotation Cluster 282**	**Enrichment Score: 0.42**		**Count**	**P_Value**	**Benjamini**
	INTERPRO	CS domain	4	3.30E-01	1.00E+00
	INTERPRO	CS	4	3.30E-01	1.00E+00
	UP_SEQ_FEATURE	domain:CS	3	5.20E-01	1.00E+00
**Annotation Cluster 283**	**Enrichment Score: 0.42**		**Count**	**P_Value**	**Benjamini**
	GOTERM_BP_FAT	regulation of cell-cell adhesion	5	3.30E-01	9.50E-01
	GOTERM_BP_FAT	negative regulation of cell-cell adhesion	3	3.40E-01	9.50E-01
	GOTERM_BP_FAT	negative regulation of cell adhesion	7	5.10E-01	9.90E-01
**Annotation Cluster 284**	**Enrichment Score: 0.42**		**Count**	**P_Value**	**Benjamini**
	GOTERM_MF_FAT	deoxyribonuclease activity	9	6.20E-02	6.70E-01
	GOTERM_MF_FAT	nuclease activity	24	2.90E-01	9.60E-01
	SP_PIR_KEYWORDS	nuclease	15	4.00E-01	8.30E-01
	SP_PIR_KEYWORDS	Endonuclease	8	6.60E-01	9.60E-01
	GOTERM_MF_FAT	endonuclease activity	12	7.50E-01	1.00E+00
	GOTERM_MF_FAT	endoribonuclease activity	5	8.70E-01	1.00E+00
**Annotation Cluster 285**	**Enrichment Score: 0.41**		**Count**	**P_Value**	**Benjamini**
	UP_SEQ_FEATURE	repeat:UIM 2	4	2.70E-01	1.00E+00
	UP_SEQ_FEATURE	repeat:UIM 1	4	2.70E-01	1.00E+00
	INTERPRO	Ubiquitin interacting motif	4	5.40E-01	1.00E+00
	SMART	UIM	4	5.70E-01	9.90E-01
**Annotation Cluster 286**	**Enrichment Score: 0.41**		**Count**	**P_Value**	**Benjamini**
	UP_SEQ_FEATURE	domain:AGC-kinase C-terminal	9	3.50E-01	1.00E+00
	INTERPRO	AGC-kinase, C-terminal	9	3.90E-01	1.00E+00
	INTERPRO	Protein kinase, C-terminal	7	3.90E-01	1.00E+00
	SMART	S_TK_X	9	4.40E-01	9.70E-01
**Annotation Cluster 287**	**Enrichment Score: 0.4**		**Count**	**P_Value**	**Benjamini**
	PIR_SUPERFAMILY	PIRSF000735:nucleoside diphosphate kinase	3	9.10E-02	1.00E+00
	UP_SEQ_FEATURE	active site:Pros-phosphohistidine intermediate	3	1.60E-01	1.00E+00
	GOTERM_BP_FAT	GTP metabolic process	4	2.00E-01	8.70E-01
	SP_PIR_KEYWORDS	nucleotide metabolism	5	2.10E-01	6.40E-01
	INTERPRO	Nucleoside diphosphate kinase, core	3	2.70E-01	1.00E+00
	SMART	NDK	3	2.90E-01	9.40E-01
	GOTERM_MF_FAT	nucleoside diphosphate kinase activity	3	3.30E-01	9.70E-01
	GOTERM_BP_FAT	GTP biosynthetic process	3	3.40E-01	9.50E-01
	GOTERM_BP_FAT	UTP metabolic process	3	3.40E-01	9.50E-01
	GOTERM_BP_FAT	UTP biosynthetic process	3	3.40E-01	9.50E-01
	GOTERM_BP_FAT	pyrimidine ribonucleoside triphosphate biosynthetic process	3	3.90E-01	9.70E-01
	GOTERM_BP_FAT	pyrimidine ribonucleoside triphosphate metabolic process	3	3.90E-01	9.70E-01
	GOTERM_BP_FAT	CTP metabolic process	3	3.90E-01	9.70E-01
	GOTERM_BP_FAT	CTP biosynthetic process	3	3.90E-01	9.70E-01
	GOTERM_BP_FAT	pyrimidine nucleoside triphosphate biosynthetic process	3	4.80E-01	9.90E-01
	GOTERM_BP_FAT	pyrimidine nucleoside triphosphate metabolic process	3	5.70E-01	9.90E-01
	GOTERM_BP_FAT	pyrimidine ribonucleotide biosynthetic process	3	6.10E-01	1.00E+00
	GOTERM_MF_FAT	phosphotransferase activity, phosphate group as acceptor	5	6.30E-01	1.00E+00
	GOTERM_BP_FAT	pyrimidine ribonucleotide metabolic process	3	6.40E-01	1.00E+00
	GOTERM_MF_FAT	nucleobase, nucleoside, nucleotide kinase activity	6	6.60E-01	1.00E+00
	GOTERM_BP_FAT	pyrimidine ribonucleoside metabolic process	3	6.80E-01	1.00E+00
	GOTERM_BP_FAT	pyrimidine nucleotide metabolic process	5	7.20E-01	1.00E+00
	GOTERM_BP_FAT	pyrimidine nucleotide biosynthetic process	3	8.10E-01	1.00E+00
	GOTERM_BP_FAT	pyrimidine nucleoside metabolic process	3	8.60E-01	1.00E+00
**Annotation Cluster 288**	**Enrichment Score: 0.4**		**Count**	**P_Value**	**Benjamini**
	GOTERM_MF_FAT	ligand-dependent nuclear receptor transcription coactivator activity	8	1.70E-01	8.90E-01
	GOTERM_MF_FAT	vitamin D receptor binding	5	3.40E-01	9.70E-01
	GOTERM_MF_FAT	thyroid hormone receptor activator activity	3	4.20E-01	9.90E-01
	GOTERM_MF_FAT	thyroid hormone receptor coactivator activity	3	4.20E-01	9.90E-01
	GOTERM_MF_FAT	receptor regulator activity	5	5.50E-01	1.00E+00
	GOTERM_MF_FAT	receptor activator activity	3	6.60E-01	1.00E+00
**Annotation Cluster 289**	**Enrichment Score: 0.4**		**Count**	**P_Value**	**Benjamini**

ILMN_3283869	FRG1B	-0.504418056
ILMN_1689380	FBXO44	-0.504462622
ILMN_1749822	TSG1	-0.504471861
ILMN_1774617	NAB1	-0.504509169
ILMN_2071405	FBXL3	-0.504582884
ILMN_2277246	SDCBP2	-0.50468522
ILMN_2155816	DIRAS2	-0.504725846
ILMN_1692790	ITGB3BP	-0.504731483
ILMN_1891311	AA744242	-0.504731716
ILMN_2149292	TMEM40	-0.504769301
ILMN_1678312	DQ577523	-0.504790052
ILMN_1839750	NA	-0.504847741
ILMN_3241149	TMEM236	-0.504931745
ILMN_1665647	CD180	-0.504976282
ILMN_2092903	C20orf141	-0.505017183
ILMN_1750246	ZBTB1	-0.505081211
ILMN_1717168	PCDHGC3	-0.505165696
ILMN_2338849	BIRC7	-0.505173292
ILMN_1651495	BG620193	-0.505197916
ILMN_1758093	C14orf101	-0.505256042
ILMN_1666371	LOC648691	-0.505292078
ILMN_1837692	BF514454	-0.505393678
ILMN_1691339	CLEC1A	-0.50541832
ILMN_1788122	GSTA5	-0.505440943
ILMN_3239925	LOC25845	-0.505470946
ILMN_2192316	TOP1	-0.505474113
ILMN_1673537	WFDC3	-0.505544985
ILMN_1882519	DA375949	-0.505605368
ILMN_1720710	HSPB3	-0.505614628
ILMN_1789407	CDRT4	-0.505637331
ILMN_1701058	TATDN1	-0.505692451
ILMN_1699912	SPDYE7P	-0.50569306
ILMN_1686083	MAGEA11	-0.505733544
ILMN_1800697	LDB2	-0.505750611
ILMN_1812473	MLLT3	-0.505793252
ILMN_1682208	CACNB2	-0.505800295
ILMN_3309219	MIR940	-0.50591876
ILMN_1687291	CDNF	-0.505970514
ILMN_3201026	LOC283028	-0.505989615
ILMN_1764607	UGT1A1	-0.506011614
ILMN_1780357	PRRT1	-0.506035317
ILMN_1690693	CTPS2	-0.506115613
ILMN_3250049	SLX1B-SULT1A4	-0.506115881
ILMN_1660307	BC073927	-0.506118462
ILMN_1736078	THBS4	-0.506187408
ILMN_1655613	GSTA2	-0.506250387
ILMN_1678671	KLHL24	-0.506256116
ILMN_2356838	CEPT1	-0.506402248
ILMN_1667201	POC1B	-0.506411454
ILMN_1750724	ZNF879	-0.506459148
ILMN_3310371	MIR939	-0.506650607
ILMN_2323385	TRIM4	-0.506815613
ILMN_1810210	DACH2	-0.506844006
ILMN_1815599	FOXE3	-0.506883389
ILMN_1656915	PSD3	-0.506892145
ILMN_2244631	STEAP1	-0.507217322
ILMN_1668779	GPR17	-0.507265127
ILMN_3309059	MIR93	-0.507271921
ILMN_3246826	AQPEP	-0.507419289
ILMN_1811954	NRG1	-0.507434765
ILMN_2149053	RIF1	-0.507451582
ILMN_3308190	MIR641	-0.507556841
ILMN_1767142	ZNF280D	-0.507647473
ILMN_1700762	PBX4	-0.507694577
ILMN_1857067	BX113978	-0.507727174
ILMN_1855107	AW592785	-0.507733326
ILMN_1721011	LCN1	-0.507737077
ILMN_3250032	XPR1	-0.507775456
ILMN_1732981	ZBTB32	-0.507790524
ILMN_2379560	CDC14B	-0.507796608
ILMN_1792383	PFN4	-0.507881276
ILMN_1812678	AGMO	-0.507891165
ILMN_1720606	LRCH2	-0.507917489
ILMN_1840806	BX105881	-0.507935421
ILMN_1690733	EGF	-0.507992945
ILMN_3310176	MIR593	-0.508058914
ILMN_1825067	AW665831	-0.508074029
ILMN_2231436	FAM26D	-0.508119229
ILMN_1705153	NEFH	-0.508137843
ILMN_3253599	FIS	-0.508169014
ILMN_1894388	NA	-0.508186395
ILMN_1699576	CDON	-0.508231246
ILMN_3242507	BX282781	-0.508232021
ILMN_1728512	YWHAH	-0.508269254
ILMN_1653031	ZNF580	-0.508273812
ILMN_1681032	C16orf54	-0.508332082
ILMN_1651599	ENTPD2	-0.508369318
ILMN_1784870	CES5A	-0.508402391
ILMN_1755741	DACH1	-0.508424037
ILMN_1806633	LOC348926	-0.508486526
ILMN_3250077	EIF4E	-0.508658524
ILMN_1660007	CCDC155	-0.508674308
ILMN_1696450	KLK1	-0.508823651
ILMN_2072401	OR11H1	-0.508837063
ILMN_1672545	GIPC2	-0.508837445

ILMN_2199284	ANAPC7	0.583244201
ILMN_1812526	TGFB2	0.583193342
ILMN_1654195	GCC2	0.58318603
ILMN_3243859	NDUFB9	0.583161978
ILMN_1824052	AA909514	0.583128652
ILMN_1708900	TMEM161A	0.583087238
ILMN_1699737	TRAP1	0.582973377
ILMN_2103388	ZBTB49	0.582972897
ILMN_1708451	PIGP	0.582964946
ILMN_2371053	EFNA1	0.582640507
ILMN_3200322	TOMM20	0.582635134
ILMN_1657194	TSNAXIP1	0.582601218
ILMN_2390114	AP3D1	0.582578054
ILMN_1748290	FAM175A	0.582386104
ILMN_1695983	DDX42	0.582262468
ILMN_1668535	JOSD1	0.5822163
ILMN_1806956	ATAD5	0.58211571
ILMN_1698067	NBPF1	0.582064201
ILMN_3240721	TDG	0.582058646
ILMN_1792681	CCDC86	0.581826461
ILMN_2147078	PIWIL4	0.581793283
ILMN_1669032	PPIC	0.581734773
ILMN_1659158	SAMD3	0.581671553
ILMN_1666910	GAB1	0.581634872
ILMN_1698766	PYCARD	0.581578172
ILMN_1652198	CCM2	0.58139921
ILMN_2402556	ZHX1	0.581386367
ILMN_1797451	SRGAP2P2	0.581380395
ILMN_1738326	EIF4E2	0.58134671
ILMN_1744109	OR2A42	0.581282412
ILMN_2345908	DDX11	0.581238322
ILMN_1684699	ELF5	0.581168634
ILMN_1754839	DHX15	0.581080154
ILMN_1807662	IGF2R	0.581018055
ILMN_1761788	MOXD1	0.581016538
ILMN_1690494	RPL6	0.58095219
ILMN_2243912	RAB37	0.580889438
ILMN_1764456	TECPR2	0.580762009
ILMN_3208881	SETD8	0.580551373
ILMN_1656807	RPL27	0.580496793
ILMN_1656017	NRF1	0.580485281
ILMN_1742824	SPATA13	0.580205101
ILMN_2087646	HLX	0.580169992
ILMN_1717063	FBXO9	0.580124518
ILMN_1673638	RPS3A	0.580076467
ILMN_3243890	NDUFA2	0.580005505
ILMN_1816342	MTRNR2L1	0.579994594
ILMN_1675979	RBMS3	0.579910656
ILMN_1778064	FICD	0.57990717
ILMN_1740900	BMP4	0.579864036
ILMN_2093343	PLAC8	0.579654265
ILMN_2317658	SLC3A2	0.579638798
ILMN_1696814	SEPSECS	0.579541657
ILMN_2327947	SLC25A25	0.579425099
ILMN_1793563	DCTN1	0.579402523
ILMN_2308582	CYB5R3	0.579374741
ILMN_3183573	NA	0.579366133
ILMN_1794374	PER2	0.579321984
ILMN_1669674	CNPY3	0.579200192
ILMN_1666894	CSPG4	0.57918953
ILMN_2398711	SIRT2	0.579189159
ILMN_1676213	SRPX2	0.579188757
ILMN_2300695	IKZF3	0.579132176
ILMN_1698323	PLEKHB2	0.579119712
ILMN_1711368	TRAPPC9	0.579019574
ILMN_1749752	GAR1	0.578980616
ILMN_1666280	COX11	0.578877596
ILMN_1731609	CHMP6	0.578875738
ILMN_1695792	CUL4A	0.578854416
ILMN_1715674	ITPK1	0.578848896
ILMN_1664294	LEPRE1	0.57852179
ILMN_1704383	TRIM37	0.578495202
ILMN_1762260	C3	0.578448725
ILMN_1703153	GLYR1	0.578371372
ILMN_1791754	CPT1B	0.578284899
ILMN_1759952	PSMA5	0.578211504
ILMN_1685202	KRBA2	0.57816883
ILMN_1743130	PTGFRN	0.57808104
ILMN_1717565	CLEC18A	0.577776739
ILMN_1768969	LBR	0.577725289
ILMN_1734895	SFT2D1	0.577550675
ILMN_1798006	ANKRD35	0.577532433
ILMN_1759789	KAT5	0.577526783
ILMN_1763640	NCKAP5L	0.577469662
ILMN_2173919	MYO9A	0.577469554
ILMN_1756937	ST8SIA4	0.577436823
ILMN_3235984	FLJ45340	0.577394705
ILMN_1803624	TMEM147	0.577355314
ILMN_1673604	YIPF3	0.577327078
ILMN_2411897	KLF10	0.577230304
ILMN_2311674	ADCY6	0.577174644
ILMN_1689786	ASMTL	0.576994755
ILMN_1813840	KDM1A	0.57678522
ILMN_1651229	IPO13	0.576587199
ILMN_2133638	CTDNEP1	0.576556523
ILMN_1668249	LMF1	0.576524858
ILMN_1682864	SPSB3	0.576477684
ILMN_2124951	RBMX	0.576463745

	UP_SEQ_FEATURE	domain:GST C-terminal	7	2.00E-01	1.00E+00
	INTERPRO	Glutathione S-transferase, N-terminal	5	3.00E-01	1.00E+00
	UP_SEQ_FEATURE	domain:GST N-terminal	4	4.80E-01	1.00E+00
	INTERPRO	Glutathione S-transferase, C-terminal	4	5.00E-01	1.00E+00
	INTERPRO	Glutathione S-transferase, C-terminal-like	5	5.10E-01	1.00E+00
	INTERPRO	Glutathione S-transferase/chloride channel, C-terminal	5	5.30E-01	1.00E+00
**Annotation Cluster 290**	**Enrichment Score: 0.4**		**Count**	**P_Value**	**Benjamini**
	GOTERM_BP_FAT	cell fate specification	11	1.30E-01	7.50E-01
	GOTERM_BP_FAT	dorsal/ventral neural tube patterning	4	2.80E-01	9.20E-01
	GOTERM_BP_FAT	neural tube patterning	4	5.40E-01	9.90E-01
	GOTERM_BP_FAT	neural tube development	10	5.50E-01	9.90E-01
	GOTERM_BP_FAT	dorsal/ventral pattern formation	5	9.60E-01	1.00E+00
**Annotation Cluster 291**	**Enrichment Score: 0.39**		**Count**	**P_Value**	**Benjamini**
	GOTERM_BP_FAT	mitochondrial transport	12	3.00E-01	9.30E-01
	GOTERM_BP_FAT	protein localization in mitochondrion	6	4.70E-01	9.80E-01
	GOTERM_BP_FAT	protein targeting to mitochondrion	6	4.70E-01	9.80E-01
**Annotation Cluster 292**	**Enrichment Score: 0.39**		**Count**	**P_Value**	**Benjamini**
	INTERPRO	Chromo domain	6	3.80E-01	1.00E+00
	SMART	CHROMO	6	4.20E-01	9.70E-01
	UP_SEQ_FEATURE	domain:Chromo 1	3	4.40E-01	1.00E+00
**Annotation Cluster 293**	**Enrichment Score: 0.39**		**Count**	**P_Value**	**Benjamini**
	UP_SEQ_FEATURE	domain:CBS 1	4	3.70E-01	1.00E+00
	UP_SEQ_FEATURE	domain:CBS 2	4	3.70E-01	1.00E+00
	SP_PIR_KEYWORDS	CBS domain	4	4.10E-01	8.40E-01
	INTERPRO	Cystathionine beta-synthase, core	4	4.40E-01	1.00E+00
	SMART	CBS	4	4.70E-01	9.70E-01
**Annotation Cluster 294**	**Enrichment Score: 0.39**		**Count**	**P_Value**	**Benjamini**
	GOTERM_BP_FAT	chromosome condensation	7	1.00E-01	6.90E-01
	GOTERM_BP_FAT	mitotic chromosome condensation	3	5.30E-01	9.90E-01
	GOTERM_BP_FAT	mitotic sister chromatid segregation	5	7.20E-01	1.00E+00
	GOTERM_BP_FAT	sister chromatid segregation	5	7.40E-01	1.00E+00
**Annotation Cluster 295**	**Enrichment Score: 0.39**		**Count**	**P_Value**	**Benjamini**
	GOTERM_BP_FAT	cell morphogenesis involved in differentiation	37	2.60E-01	9.10E-01
	GOTERM_BP_FAT	cellular component morphogenesis	58	2.70E-01	9.20E-01
	GOTERM_BP_FAT	cell morphogenesis	52	2.80E-01	9.30E-01
	GOTERM_BP_FAT	neuron projection morphogenesis	32	3.00E-01	9.40E-01
	GOTERM_BP_FAT	cell part morphogenesis	37	3.70E-01	9.60E-01
	GOTERM_BP_FAT	cell projection organization	52	3.80E-01	9.60E-01
	GOTERM_BP_FAT	neuron projection development	36	4.40E-01	9.80E-01
	GOTERM_BP_FAT	axon guidance	16	4.40E-01	9.80E-01
	GOTERM_BP_FAT	cell projection morphogenesis	34	4.80E-01	9.80E-01
	GOTERM_BP_FAT	axonogenesis	27	4.90E-01	9.90E-01
	GOTERM_BP_FAT	neuron development	45	5.70E-01	9.90E-01
	GOTERM_BP_FAT	cell morphogenesis involved in neuron differentiation	27	6.60E-01	1.00E+00
	GOTERM_BP_FAT	neuron differentiation	56	6.80E-01	1.00E+00
**Annotation Cluster 296**	**Enrichment Score: 0.38**		**Count**	**P_Value**	**Benjamini**
	INTERPRO	Phosphoinositide 3-kinase accessory region PIK	3	3.70E-01	1.00E+00
	SMART	PI3Ka	3	3.90E-01	9.70E-01
	GOTERM_MF_FAT	inositol or phosphatidylinositol kinase activity	6	5.30E-01	9.90E-01
**Annotation Cluster 297**	**Enrichment Score: 0.37**		**Count**	**P_Value**	**Benjamini**
	GOTERM_BP_FAT	cell chemotaxis	8	2.50E-01	9.10E-01
	GOTERM_BP_FAT	leukocyte migration	10	3.40E-01	9.50E-01
	GOTERM_BP_FAT	leukocyte chemotaxis	6	5.50E-01	9.90E-01
	GOTERM_BP_FAT	neutrophil chemotaxis	3	7.10E-01	1.00E+00

ILMN_1673793	SUN3	-0.508853843
ILMN_1818856	BE549596	-0.508867708
ILMN_1871488	BQ027500	-0.508918321
ILMN_1812134	C11orf30	-0.508932418
ILMN_1806434	PAQR5	-0.508946491
ILMN_1670420	METAP2	-0.508972445
ILMN_1701434	RAP1B	-0.508975713
ILMN_1796075	VPS41	-0.508979302
ILMN_1727309	FAM82A2	-0.509032802
ILMN_1724391	ICA1L	-0.509067363
ILMN_2126344	SEC16A	-0.509287181
ILMN_1673843	CST2	-0.509333744
ILMN_2398378	PML	-0.509375361
ILMN_3281855	SSBP3	-0.509377631
ILMN_1775034	MEIG1	-0.509427359
ILMN_3245834	USP17L2	-0.509441486
ILMN_1696397	HYDIN	-0.509452498
ILMN_1773333	TMCO2	-0.509461002
ILMN_1855386	CR622974	-0.509491676
ILMN_1761049	EIF2C3	-0.509498393
ILMN_1792959	KCNMB2	-0.509514211
ILMN_1781256	LEFTY2	-0.509571813
ILMN_1743594	UBXN7	-0.50957889
ILMN_2390416	BAG6	-0.509618643
ILMN_2375386	RNPS1	-0.509717163
ILMN_1803348	EHBP1	-0.509729837
ILMN_1669366	IFT88	-0.509777236
ILMN_1764043	TTL	-0.509792958
ILMN_1721549	PLA2G1B	-0.50981434
ILMN_1655748	ZNF323	-0.50982646
ILMN_1712530	AKAP1	-0.509903957
ILMN_3305942	LOC729970	-0.510008881
ILMN_3283180	NA	-0.510158445
ILMN_2186678	C6orf211	-0.510222567
ILMN_1716999	ZSCAN1	-0.510266089
ILMN_2218104	PAH	-0.51026801
ILMN_1685012	EAF1	-0.510330591
ILMN_2207726	FAM46D	-0.510350389
ILMN_1764166	BCKDHB	-0.510468004
ILMN_2108590	THSD7B	-0.510526583
ILMN_1877421	BX092644	-0.510531852
ILMN_1725105	EEF1E1	-0.510564276
ILMN_1886646	AI810925	-0.510568877
ILMN_1851912	BC037850	-0.510603265
ILMN_1757754	SUDS3	-0.510632797
ILMN_2374191	FNTA	-0.510670948
ILMN_1657077	SOCS6	-0.510679853
ILMN_1705659	FAM71C	-0.510721725
ILMN_1726435	NA	-0.510877993
ILMN_1748538	ALDH1A2	-0.510893713
ILMN_1910732	AI829589	-0.510991912
ILMN_1660778	ANKH	-0.511016345
ILMN_1724699	ACAD8	-0.511065076
ILMN_1669773	ANGPTL1	-0.511075038
ILMN_2200210	GJA10	-0.511143405
ILMN_2174834	OR4C3	-0.511197128
ILMN_1744832	LOC399715	-0.511236675
ILMN_3211472	LOC221442	-0.511288591
ILMN_1688380	AA213559	-0.511302205
ILMN_3247552	KNCN	-0.511430193
ILMN_1748352	CTSL2	-0.511568058
ILMN_1745817	NELL1	-0.511657934
ILMN_3241944	Nbla00301	-0.511663456
ILMN_1681994	AJ005282	-0.511710008
ILMN_1716377	HMGCLL1	-0.511839757
ILMN_1680727	GLRX2	-0.511840971
ILMN_1699674	ZNF703	-0.511859723
ILMN_1879041	BM673750	-0.511876616
ILMN_1701630	FAM74A4	-0.51191006
ILMN_2151488	RMI1	-0.511917017
ILMN_3256789	NA	-0.51195306
ILMN_2173500	FAM69C	-0.511991276
ILMN_1811544	LPAL2	-0.512049182
ILMN_1674349	HRASLS5	-0.512084775
ILMN_2311020	DNAJC12	-0.512166627
ILMN_1707652	KRTAP1-5	-0.512270831
ILMN_2340643	INSC	-0.512309445
ILMN_1727626	KRT39	-0.512322541
ILMN_3243863	DB462629	-0.512340386
ILMN_1915188	MID2	-0.512341812
ILMN_1779412	OR2AT4	-0.512374948
ILMN_2319952	VDR	-0.512431905
ILMN_1806306	SV2B	-0.51250894
ILMN_1688702	PJA2	-0.512509588
ILMN_1776383	LOC642426	-0.512633512
ILMN_1759277	OIP5	-0.512657494
ILMN_1762134	ECHDC1	-0.512674063
ILMN_2372698	RGN	-0.512710403
ILMN_1672389	CRYZ	-0.512736857
ILMN_1680378	RBM45	-0.512791437
ILMN_1738418	SPEF1	-0.512878695
ILMN_1675329	TBC1D3	-0.512977987
ILMN_1790106	PLP1	-0.51298346
ILMN_3244402	RIMBP3	-0.513026692
ILMN_1652017	PPEF1	-0.513039306
ILMN_3186401	FAM71E2	-0.513041442
ILMN_1751943	MME	-0.513054003
ILMN_1732300	POLR2C	-0.513061301

ILMN_1789186	OBFC1	0.576307591
ILMN_1726108	LASS2	0.576265733
ILMN_1758129	OR6F1	0.576170467
ILMN_3243782	ANKRD36	0.576163744
ILMN_1727402	HCLS1	0.576072548
ILMN_1815130	MICALL1	0.576060197
ILMN_1683065	C2orf64	0.576007736
ILMN_2405254	GRB7	0.576004985
ILMN_1807492	PSMC1	0.575901719
ILMN_1712605	ZNF829	0.57589852
ILMN_1780292	MSH5	0.575721969
ILMN_1761801	ZNF761	0.575714614
ILMN_1812353	CSPP1	0.575702784
ILMN_2346339	FOLR1	0.575668728
ILMN_1757847	C11orf68	0.575664049
ILMN_2110908	MYC	0.575622953
ILMN_2256953	CASZ1	0.575584166
ILMN_1864900	MIAT	0.575542942
ILMN_2143250	FAR1	0.575542281
ILMN_2119774	CYP2R1	0.575534704
ILMN_2147105	NPIPL2	0.57543748
ILMN_1687074	CDC14B	0.575349925
ILMN_3201115	PGAM1	0.575255011
ILMN_2084059	SLC12A4	0.575232785
ILMN_2306692	MINK1	0.575181906
ILMN_2066060	HLA-DRB6	0.575176307
ILMN_1806037	TK1	0.575118776
ILMN_3258321	SNRPG	0.575005844
ILMN_1773847	DYNC1I2	0.575004184
ILMN_1786139	VKORC1	0.574996115
ILMN_2323633	TPD52L2	0.57492366
ILMN_1780236	PMM1	0.574690826
ILMN_2369682	HNRNPA2B1	0.574684042
ILMN_2255310	RPS15A	0.574600783
ILMN_2365465	XBP1	0.57452938
ILMN_1778650	VILL	0.574502209
ILMN_1813338	LAG3	0.574448777
ILMN_1732060	ARHGAP1	0.574297905
ILMN_1790917	IFFO1	0.573971111
ILMN_1777444	STX5	0.573915084
ILMN_3229033	TDG	0.573861161
ILMN_1657495	MLEC	0.573803123
ILMN_1713934	LITAF	0.573635345
ILMN_1756352	LAMTOR2	0.573593533
ILMN_1717990	CALD1	0.573583008
ILMN_1694057	EIF3K	0.573569342
ILMN_1780315	PUSL1	0.573455516
ILMN_1805922	EBPL	0.573166062
ILMN_2358980	ILK	0.573105742
ILMN_2352009	ACADVL	0.573096595
ILMN_1727051	DDX19A	0.573071455
ILMN_1659075	HLA-DOA	0.572990707
ILMN_1748797	GRB2	0.572973913
ILMN_2398489	SIGMAR1	0.572832512
ILMN_1734410	BNIP1	0.572741785
ILMN_1809433	XBP1	0.572580125
ILMN_1758229	QSOX1	0.57257423
ILMN_2414007	NME1	0.572514311
ILMN_1717855	PFDN1	0.572354045
ILMN_1809859	PCGF2	0.572311987
ILMN_3202885	LOC650368	0.572228662
ILMN_1796063	TRIM44	0.5722189
ILMN_1672417	PTPRCAP	0.572198816
ILMN_1660871	NEK6	0.57200875
ILMN_1779512	AP4M1	0.571894228
ILMN_2388605	ACTR2	0.571893363
ILMN_1764489	TBL2	0.57179172
ILMN_1714527	VAMP3	0.571790763
ILMN_1679929	KLF13	0.571751061
ILMN_1742224	SLTM	0.571732895
ILMN_1717326	SLC29A3	0.57162815
ILMN_1808999	ARHGEF19	0.571534603
ILMN_1738237	HS1BP3	0.571534102
ILMN_3305397	RPL7	0.571530501
ILMN_1768282	SNX21	0.571519386
ILMN_1762766	PTPRA	0.571410414
ILMN_1671565	RNASET2	0.571406173
ILMN_1743065	PTPLAD1	0.571390805
ILMN_1657554	TSPYL2	0.571083423
ILMN_1653292	PFKFB4	0.571042985
ILMN_2144654	SPDYE2	0.571000907
ILMN_1651950	TPST1	0.570981415
ILMN_1672080	NR2F6	0.570880843
ILMN_1654939	TMED2	0.570876639
ILMN_1692779	PRPF39	0.570863206
ILMN_2299612	TMEM150A	0.570840572
ILMN_1688452	LCMT1	0.570750815
ILMN_2167729	ZNF391	0.570626513
ILMN_1726342	BIN2	0.570489117
ILMN_1708485	BIN3	0.570468957
ILMN_1655161	MORN1	0.570328028
ILMN_2284941	ABCF2	0.570268102
ILMN_2163187	FLJ35776	0.570190158
ILMN_1787345	FKBP11	0.570162591
ILMN_1763144	NEU1	0.570127411
ILMN_1687958	SLC25A22	0.570082373
ILMN_2384241	TGFBR2	0.570030767
ILMN_2407811	NEURL4	0.569973882

ILMN_1655545	ZMYND12	-0.513103452
ILMN_1754491	IYD	-0.513147027
ILMN_1738410	BC031945	-0.513150758
ILMN_1651259	UGP2	-0.513199548
ILMN_1810797	WASF3	-0.513353295
ILMN_2047480	CXorf36	-0.513358231
ILMN_1802942	AJ292264	-0.513475917
ILMN_1662081	KRTAP3-2	-0.513583807
ILMN_1699455	FAM90A7	-0.51361299
ILMN_1748092	F8A1	-0.513613552
ILMN_1693665	LIMS1	-0.513682537
ILMN_1732080	SUMO1P3	-0.513683133
ILMN_2089458	SASS6	-0.513743727
ILMN_1737972	TSPYL5	-0.513851161
ILMN_1682165	NT5C2	-0.513887801
ILMN_2181060	CKAP2	-0.513908396
ILMN_1668708	SPATA3	-0.513943264
ILMN_2264681	LETMD1	-0.513996245
ILMN_1885147	AW292656	-0.514037441
ILMN_3251440	PIK3C2A	-0.514158172
ILMN_1657478	MAGEL2	-0.514178261
ILMN_1892243	AA720941	-0.514184423
ILMN_1698186	IFNA2	-0.514248817
ILMN_1867345	AI088589	-0.514252143
ILMN_1807147	RC3H1	-0.51431295
ILMN_3247190	TNS1	-0.51433498
ILMN_1795170	SLITRK2	-0.514337205
ILMN_1771084	ACSM3	-0.514382624
ILMN_1854165	BM931599	-0.514463168
ILMN_1692938	PSAT1	-0.514522818
ILMN_1760339	ZNF774	-0.514536333
ILMN_1714010	KRTAP4-4	-0.51474304
ILMN_1727466	KCNMB4	-0.514757214
ILMN_1692638	MYH2	-0.514835492
ILMN_1813938	CHCHD4	-0.514857877
ILMN_1697611	AMBN	-0.514891794
ILMN_2183216	TDP2	-0.515065882
ILMN_3243324	FLJ35024	-0.515123038
ILMN_3213640	RPPH1	-0.51514415
ILMN_3178368	GAFA2	-0.515223867
ILMN_3240147	CXorf51	-0.515240753
ILMN_1868513	BM727329	-0.515269199
ILMN_1869146	BQ007862	-0.515276746
ILMN_1656988	LOC646508	-0.515348956
ILMN_2191803	MYPN	-0.515351781
ILMN_1733781	FSD1	-0.515437746
ILMN_1703226	KTI12	-0.515442746
ILMN_1855966	DB338170	-0.515477257
ILMN_1669805	C1orf87	-0.515519371
ILMN_1656981	LOC100505679	-0.51553131
ILMN_1734826	NUP88	-0.515571
ILMN_1882764	LOC729852	-0.515638683
ILMN_1881081	HIST1H3F	-0.515763676
ILMN_1839647	C21orf130	-0.515831171
ILMN_1846249	AV647692	-0.51583872
ILMN_1726198	CTSL3	-0.515842171
ILMN_1739513	CASP14	-0.515869329
ILMN_1661417	MAGED2	-0.515887164
ILMN_1757237	ASAP2	-0.515928143
ILMN_1791749	CLEC2L	-0.515999429
ILMN_1880885	AL042883	-0.516009536
ILMN_2386444	ANGPTL4	-0.51602042
ILMN_2120210	RCAN2	-0.516182882
ILMN_1720484	CRTAP	-0.516215196
ILMN_3236120	CB991063	-0.516223508
ILMN_2395506	SOX6	-0.516226364
ILMN_1695579	CIT	-0.516301463
ILMN_1739690	NPFFR2	-0.516305548
ILMN_3188479	CCR2	-0.516334106
ILMN_3307509	C17orf54	-0.516511873
ILMN_1681732	C1orf226	-0.516534935
ILMN_1714384	PCCA	-0.51654711
ILMN_1705617	EFEMP2	-0.516602675
ILMN_1778079	sept-04	-0.516609984
ILMN_1776826	ARHGAP42	-0.516623018
ILMN_2298624	RREB1	-0.516647135
ILMN_1753006	TACR2	-0.516673993
ILMN_1775716	GOLGA8G	-0.516716981
ILMN_1799860	PIGM	-0.51676509
ILMN_1658152	MAP3K10	-0.516765601
ILMN_1695288	PDE5A	-0.516784259
ILMN_1745473	PDE4DIP	-0.516829108
ILMN_1731157	MYOZ1	-0.516837118
ILMN_1872450	AI912163	-0.516843255
ILMN_2220845	TMPRSS15	-0.516845441
ILMN_1767481	XRCC6BP1	-0.516878089
ILMN_1775175	RPL3L	-0.51698198
ILMN_1817524	NA	-0.517078649
ILMN_2094587	USP8	-0.517112111
ILMN_1661566	FSTL4	-0.517211147
ILMN_1744611	DCAF13	-0.517217219
ILMN_1786785	SLC18A1	-0.517315363
ILMN_1776777	ADAR	-0.517355364
ILMN_1758272	MYPN	-0.517376046
ILMN_1804150	HIBADH	-0.517410915
ILMN_1728830	GRK5	-0.517522324
ILMN_1730401	LOC442421	-0.517553874
ILMN_3209156	LOC100131372	-0.517565844

ILMN_2146418	CRIM1	0.569915777
ILMN_1754990	RPS27A	0.569878898
ILMN_3292678	RPL21	0.569772528
ILMN_1745686	MFHAS1	0.56977169
ILMN_1729430	FBXO18	0.569630417
ILMN_1700515	C17orf58	0.569597399
ILMN_1763907	CENPW	0.569585866
ILMN_2115154	NUPL2	0.569466251
ILMN_1670383	PCDH7	0.569455878
ILMN_1808946	POLR2J4	0.569428458
ILMN_3235326	LOC388796	0.569274839
ILMN_1703487	LMO4	0.569267353
ILMN_2248725	TYSND1	0.569205539
ILMN_3238481	SNORD60	0.569158412
ILMN_1667298	C17orf108	0.568797227
ILMN_1721967	OR2A7	0.56877773
ILMN_1757723	C6orf106	0.568710707
ILMN_1756086	INTS3	0.568701397
ILMN_1745075	RPLP0	0.568695043
ILMN_1805247	SYNRG	0.568612894
ILMN_2285996	KIAA0101	0.568440623
ILMN_2361862	VLDLR	0.568423196
ILMN_1665831	CLPTM1	0.568343188
ILMN_1762899	EGR1	0.568195469
ILMN_1673757	CASP8	0.568186399
ILMN_1780663	KLHL30	0.568178901
ILMN_1811574	MAPK8IP3	0.568175869
ILMN_1716728	C6orf64	0.568169984
ILMN_1698258	DNAJC8	0.568094994
ILMN_3242883	AGAP8	0.568088538
ILMN_2166831	RPS4X	0.568073682
ILMN_1712487	EIF2D	0.567981059
ILMN_1668639	TBC1D10B	0.567965126
ILMN_2114720	SLPI	0.567273158
ILMN_1693009	FGL2	0.567243378
ILMN_3263329	GAR1	0.566981855
ILMN_2179022	CDRT15P	0.566968074
ILMN_1720857	SMA4	0.566946573
ILMN_1763080	QARS	0.566888226
ILMN_1789639	FMOD	0.566654627
ILMN_3283563	AK130934	0.566594327
ILMN_2075436	PDIA3P	0.566570845
ILMN_1757440	FAM69B	0.566537815
ILMN_1735155	GLB1	0.566469651
ILMN_1691201	FAM21C	0.566451928
ILMN_1679025	RPS3A	0.566449321
ILMN_1343291	EEF1A1	0.566440022
ILMN_1674402	TMEM71	0.566301502
ILMN_1734445	PKDCC	0.56611728
ILMN_2366388	PRDX1	0.566101287
ILMN_1811328	DPP7	0.566048885
ILMN_2365307	CD276	0.56599789
ILMN_1680831	BAZ1B	0.565910189
ILMN_1655311	C15orf61	0.565805451
ILMN_1658579	CDK5RAP3	0.565765018
ILMN_1832106	NA	0.56562624
ILMN_1684571	RSPH1	0.565598358
ILMN_1733221	SEMA4D	0.565462792
ILMN_2181363	UBE3C	0.565328298
ILMN_2297864	MTMR14	0.565287784
ILMN_2336094	ODZ3	0.565090228
ILMN_2382505	SLC22A18	0.564976445
ILMN_1734138	TATDN2	0.564856304
ILMN_1700461	AARSD1	0.564748499
ILMN_1669592	MARCH2	0.564698427
ILMN_1701375	FBXW5	0.564620738
ILMN_2067890	CXCL11	0.564574175
ILMN_2381834	TRIM69	0.564518666
ILMN_1807372	ADORA2A	0.564492871
ILMN_1677487	ANKZF1	0.564474946
ILMN_1761199	SLCO2B1	0.564428588
ILMN_1740165	C14orf102	0.564426937
ILMN_1784320	ELMO1	0.564424449
ILMN_1700606	RNF43	0.56437674
ILMN_2183692	PHYHD1	0.564337519
ILMN_1807925	GNG2	0.564237686
ILMN_1684897	SPDYE1	0.564199484
ILMN_2415189	ATP1A1	0.564104026
ILMN_3289144	BC012174	0.563927576
ILMN_1708143	FAM127A	0.563926557
ILMN_1662954	CCT7	0.563899685
ILMN_1781514	PCDH17	0.5638909
ILMN_1812091	FAM20A	0.563876756
ILMN_3195253	RPL7	0.563827974
ILMN_1665219	LTBP4	0.563805208
ILMN_1808779	CSTF3	0.563790735
ILMN_1815148	MAN2A2	0.56362551
ILMN_3234993	ROMO1	0.56330711
ILMN_1773276	WHAMML2	0.563228656
ILMN_1708101	LMNB2	0.563173824
ILMN_2072603	MRPL14	0.562972643
ILMN_1794707	ATHL1	0.562934007
ILMN_1696087	PHB2	0.562858664
ILMN_2373444	ADORA1	0.562792727
ILMN_1672547	MYO9B	0.56275611
ILMN_3233748	NA	0.562651458
ILMN_1819608	ARRB1	0.562645878
ILMN_1750507	RPL9	0.562574789

ILMN_2376263	SMARCA1	-0.517587979
ILMN_1696223	ATP7B	-0.517695667
ILMN_1711245	SCG3	-0.517800139
ILMN_2045729	WDR12	-0.517832813
ILMN_1795839	SCCPDH	-0.517859816
ILMN_3245351	UNC119B	-0.517875729
ILMN_1730282	LOC100289656	-0.517937469
ILMN_1661594	C2orf42	-0.517957374
ILMN_1671619	HMX2	-0.517993271
ILMN_1781104	MAP2K7	-0.518078541
ILMN_1698551	RCN1	-0.518103455
ILMN_1698379	MAGEC1	-0.51818171
ILMN_1734486	TSEN15	-0.518198737
ILMN_2054725	SUGT1	-0.518276707
ILMN_3180807	BX649083	-0.518320643
ILMN_2135944	CT45A3	-0.518456058
ILMN_3298163	CR594811	-0.518470765
ILMN_1800873	SEZ6L2	-0.518493714
ILMN_1714891	OR2A14	-0.518548434
ILMN_1730646	WDR87	-0.518559621
ILMN_1861652	BQ575680	-0.518616814
ILMN_1661220	TRIM16	-0.51882035
ILMN_1801045	SPIN3	-0.518924112
ILMN_1759870	ING5	-0.518982796
ILMN_1755954	CPEB3	-0.519002059
ILMN_1729639	CLDN20	-0.519042471
ILMN_2395856	TMEM218	-0.519075335
ILMN_2406335	ANKRD17	-0.519125613
ILMN_1750850	KLK14	-0.519125867
ILMN_1696028	ETNK1	-0.519202269
ILMN_3226388	PSG4	-0.519206953
ILMN_1682110	HOXA10	-0.519229026
ILMN_2352023	DSTYK	-0.51929627
ILMN_1864157	CR627150	-0.51932394
ILMN_1658636	MRE11A	-0.519329081
ILMN_3194508	ASAP2	-0.519333324
ILMN_1865568	AL043551	-0.519412699
ILMN_2172735	EXOC3L1	-0.5195244
ILMN_3252033	AK098731	-0.519525878
ILMN_1800642	RELN	-0.519540539
ILMN_1667195	NA	-0.519581159
ILMN_1727076	OR10H4	-0.519703853
ILMN_2091217	FAM150A	-0.519737709
ILMN_1682062	RYR1	-0.519803485
ILMN_2279339	WTAP	-0.519823836
ILMN_1693932	LOC285733	-0.519824637
ILMN_3236437	PCAN1	-0.519847087
ILMN_1755631	WISP3	-0.519878787
ILMN_1904188	AW269209	-0.519909972
ILMN_1695998	YKT6	-0.519942104
ILMN_1752927	FAM160B1	-0.5200251
ILMN_1816458	DB298233	-0.520102719
ILMN_2152028	AK027082	-0.520109113
ILMN_1826447	BX107250	-0.520208094
ILMN_1781761	ENPP4	-0.520250482
ILMN_1796029	AB051447	-0.520376542
ILMN_1675520	CPAMD8	-0.520405232
ILMN_1781222	XGPY2	-0.520432007
ILMN_1799628	NBR1	-0.520467557
ILMN_1660882	CHRM4	-0.52051197
ILMN_1798187	MYST2	-0.520557324
ILMN_2098571	MYNN	-0.520667591
ILMN_1674795	hCG_1997999	-0.520726294
ILMN_1765734	TGIF2LX	-0.520754674
ILMN_1703507	PPIP5K1	-0.520767727
ILMN_1672884	MTFMT	-0.52080178
ILMN_1710512	PFN2	-0.520842147
ILMN_2402581	GCOM1	-0.520902885
ILMN_1659937	ZBTB24	-0.52099169
ILMN_1656225	DUPD1	-0.521074483
ILMN_1661458	ARF1	-0.521097549
ILMN_1789095	BMPR2	-0.521114769
ILMN_3178050	SPECC1	-0.521179764
ILMN_1680860	LEMD2	-0.52121908
ILMN_1703233	TBC1D3	-0.52128896
ILMN_2363097	SLC9A6	-0.521390488
ILMN_1699320	LOC339524	-0.521399019
ILMN_3274299	BC029572	-0.521403697
ILMN_1674098	SORBS2	-0.52142163
ILMN_2073409	C1orf81	-0.521474577
ILMN_1768064	OR52I2	-0.521497976
ILMN_1773886	MED21	-0.521509254
ILMN_1758918	BRD2	-0.521528043
ILMN_1656746	DKFZp666C185	-0.521580109
ILMN_1785069	KIAA1845	-0.521654305
ILMN_1699676	C14orf147	-0.521681578
ILMN_1854359	BX112865	-0.521701298
ILMN_2189314	HDGFL1	-0.521784898
ILMN_1658315	TSPY8	-0.521850137
ILMN_3223953	NA	-0.521852761
ILMN_3273491	AI241366	-0.521937425
ILMN_1725234	CYP19A1	-0.521951705
ILMN_2181540	YY1	-0.521971312
ILMN_1673503	CACNB4	-0.521983935
ILMN_1708182	ODF1	-0.522040607
ILMN_2194467	SGCB	-0.522146047
ILMN_1776939	MS4A1	-0.522171848
ILMN_1907042	EU154351	-0.522235831

ILMN_1666057	REEP2	0.562557866
ILMN_3249327	DQ581928	0.562459225
ILMN_2363106	RBM23	0.562454471
ILMN_1745223	CDC42EP4	0.562404466
ILMN_1729640	ESRP2	0.562390562
ILMN_1758337	ZNF213	0.562312648
ILMN_1749634	PLRG1	0.562247861
ILMN_1763264	MRPL2	0.562209736
ILMN_1763228	MEF2D	0.562206792
ILMN_1772605	FRS3	0.562180237
ILMN_3278879	NA	0.562101514
ILMN_1809439	HMGB1	0.562031581
ILMN_1763347	PIK3CB	0.561880748
ILMN_1804263	ZNF76	0.561863638
ILMN_1703441	ZNF593	0.56181391
ILMN_3251629	EIF4A1	0.561665242
ILMN_1676665	CUEDC1	0.561589605
ILMN_1721833	IER5	0.561582916
ILMN_1809467	VAMP5	0.561564933
ILMN_3224290	UBE2V1	0.561482732
ILMN_3216336	TPT1	0.56140715
ILMN_1665601	THAP4	0.56138817
ILMN_1653161	SNCG	0.561355569
ILMN_2383871	ZNF74	0.561330117
ILMN_1683678	SPATS2L	0.561256825
ILMN_1656316	ZMYM3	0.561236009
ILMN_1691668	C6orf154	0.561061396
ILMN_1808011	FAM46B	0.560995579
ILMN_1760922	GIT2	0.560925396
ILMN_1693685	NCRNA00116	0.560786693
ILMN_1777344	RPS13	0.560657981
ILMN_1753064	TTC13	0.560449504
ILMN_1727862	HPS4	0.560382877
ILMN_3251361	NBPF1	0.560373609
ILMN_2326712	BACE2	0.560362052
ILMN_2046730	S100A10	0.560224592
ILMN_2206411	CTAG1B	0.560218804
ILMN_1750678	TIMD4	0.560211894
ILMN_3243972	SNORA70B	0.560193975
ILMN_1722923	SRGAP3	0.560150555
ILMN_3239965	IDO1	0.56011791
ILMN_1773352	CCL5	0.560115777
ILMN_1709334	TM9SF1	0.560071286
ILMN_2401155	PUM1	0.560055457
ILMN_1772316	SUN1	0.560025903
ILMN_3265343	LOC100506144	0.559973188
ILMN_1742981	TUBA1A	0.559969364
ILMN_1752877	PHF1	0.559966929
ILMN_1721575	VPS18	0.55995357
ILMN_1713636	S100A6	0.559937131
ILMN_1802553	URGCP	0.559861738
ILMN_1671149	MEG3	0.559814574
ILMN_1653896	ATRIP	0.559805475
ILMN_2404512	PSEN2	0.559741251
ILMN_2408877	MLL3	0.55972039
ILMN_1710514	BCL3	0.559449211
ILMN_2367753	ATP2B4	0.559392904
ILMN_3266411	RPL7	0.559190447
ILMN_1683194	DCN	0.559124828
ILMN_2320336	CLK3	0.55906512
ILMN_1718672	NHP2	0.559058795
ILMN_1705861	AP1M2	0.558953618
ILMN_1759495	XPO5	0.558919148
ILMN_1664863	CTRL	0.558854001
ILMN_1651936	SETD8	0.558790154
ILMN_1736180	FRAT1	0.558754301
ILMN_1781231	SLC25A38	0.558595384
ILMN_1690138	PHF10	0.558555914
ILMN_1787628	NOP56	0.558521602
ILMN_3232921	CCR2	0.558435612
ILMN_1762436	UBB	0.558429894
ILMN_2404548	CRELD1	0.558386677
ILMN_1720344	NIPA2	0.558349935
ILMN_3246391	SNORD52	0.558303592
ILMN_1662768	NHEDC2	0.558000308
ILMN_2065783	EXOC2	0.557890646
ILMN_1794967	EIF4ENIF1	0.557866024
ILMN_1774261	DOK4	0.557861373
ILMN_2127477	G6PC3	0.557832661
ILMN_2362457	ZDHHC16	0.557747235
ILMN_1662328	CNNM3	0.557652012
ILMN_1707685	LOC441242	0.557440489
ILMN_1700168	LARS2	0.557431803
ILMN_1764945	AP3D1	0.557208162
ILMN_1690708	SPTBN1	0.557146174
ILMN_2414848	TBRG4	0.557099047
ILMN_1778352	ABCC6	0.557089138
ILMN_1787026	SEC61G	0.556793589
ILMN_1654099	LRIG2	0.556675919
ILMN_3282829	NA	0.556627646
ILMN_1740441	CYB5R3	0.556587645
ILMN_1673024	RBM15B	0.556566908
ILMN_1659227	CD79A	0.556561974
ILMN_1765116	PAOX	0.556513657
ILMN_1705679	SAFB2	0.556509732
ILMN_1767747	HDAC2	0.556402844
ILMN_1782331	TDG	0.556283615
ILMN_1690826	TNKS1BP1	0.556164428

ILMN_1801996	MASP1	-0.522241656
ILMN_2404033	ZNF205	-0.52225747
ILMN_2089329	SPRY2	-0.522304997
ILMN_3237589	PHAX	-0.522320536
ILMN_1684802	TAF5	-0.522338993
ILMN_1664006	AGL	-0.522390847
ILMN_2346856	GGPS1	-0.522483127
ILMN_3237067	FAM75B	-0.522483559
ILMN_1778253	SRC	-0.522513131
ILMN_2390609	ANK3	-0.522545068
ILMN_1787208	GOLGA8E	-0.522609031
ILMN_1684060	PP1164	-0.522615314
ILMN_1689852	PAQR6	-0.522626889
ILMN_1764034	XCR1	-0.522627036
ILMN_2406586	CC2D2A	-0.522669214
ILMN_3252185	BC037739	-0.522772805
ILMN_3202109	PERP	-0.522792566
ILMN_1781884	KRT12	-0.522896374
ILMN_1678132	BC035390	-0.523109878
ILMN_3251346	NOX4	-0.523167245
ILMN_3251171	DNASE1L1	-0.523215851
ILMN_1659893	DEC1	-0.52329189
ILMN_3241750	CCNI2	-0.523417095
ILMN_1884922	TCRA	-0.523540002
ILMN_3194480	LOC728405	-0.523603483
ILMN_1670918	INSR	-0.523604333
ILMN_1685559	BC035989	-0.523612831
ILMN_1767992	SLC12A6	-0.523671584
ILMN_2290118	MEGF9	-0.523740468
ILMN_1683575	TMLHE	-0.523810946
ILMN_2352036	RTN4	-0.523841806
ILMN_1885629	DB022751	-0.523962967
ILMN_3310441	VTRNA1-2	-0.524185149
ILMN_1776724	LYPD6	-0.524270244
ILMN_3251516	KIAA1609	-0.524320532
ILMN_1834696	BQ187514	-0.524454446
ILMN_2113470	GSTA1	-0.524504804
ILMN_1773505	USP4	-0.524521062
ILMN_1703570	CD359090	-0.524541113
ILMN_3239188	BC040221	-0.524573441
ILMN_1912822	AW237220	-0.524655925
ILMN_1683761	CXorf48	-0.524686789
ILMN_2131828	KIR3DL1	-0.524737011
ILMN_1696513	PCDHGC3	-0.524841712
ILMN_1662147	MANEAL	-0.5249664
ILMN_1696661	FAM186A	-0.525023036
ILMN_1777528	NCBP1	-0.525108106
ILMN_1747968	RBM33	-0.525172618
ILMN_1776216	MMGT1	-0.525295972
ILMN_1681688	LOC645676	-0.525301783
ILMN_1749612	IMMP1L	-0.525372432
ILMN_1835951	abParts	-0.525493836
ILMN_1800179	KCNJ4	-0.525527021
ILMN_1659214	CTXN3	-0.52553159
ILMN_1898723	BC090942	-0.525702578
ILMN_1734198	RNGTT	-0.525741703
ILMN_1865043	BP419082	-0.525776023
ILMN_3286353	TTC34	-0.525778829
ILMN_1680687	NSF	-0.525785308
ILMN_1709300	IL5	-0.525796211
ILMN_3309659	MIR181A2	-0.525819048
ILMN_1695461	PTPRA	-0.525913074
ILMN_3248032	NCCRP1	-0.525925887
ILMN_1701820	ZNF738	-0.526042713
ILMN_1702183	AK125594	-0.526069183
ILMN_1829052	BU753580	-0.526085628
ILMN_1852279	CK299576	-0.526221071
ILMN_1758941	REEP5	-0.526253482
ILMN_1814122	MDC1	-0.52630614
ILMN_1882015	BG951912	-0.526386839
ILMN_2332795	ZNF16	-0.526520983
ILMN_1895897	BF514393	-0.526580628
ILMN_1764087	SYPL1	-0.52662957
ILMN_1674243	TFRC	-0.526634617
ILMN_3188772	NA	-0.526675595
ILMN_1678814	UBL5	-0.526737998
ILMN_1745318	CREM	-0.52676005
ILMN_1777745	FAM133B	-0.526772275
ILMN_1724016	C15orf44	-0.52680822
ILMN_1758034	ETFDH	-0.527142251
ILMN_1711715	LOC100128184	-0.527148892
ILMN_1669820	KRT6C	-0.527149928
ILMN_3238757	SEPP1	-0.527234138
ILMN_1771544	FMO9P	-0.527248666
ILMN_2335072	TAF1C	-0.52732618
ILMN_1754708	TNK2	-0.527352725
ILMN_1804938	TPRA1	-0.527420766
ILMN_1754541	MYCL1	-0.527477176
ILMN_3251737	EEF1A1	-0.527477987
ILMN_2219131	RPS15	-0.527499091
ILMN_1814002	TEAD3	-0.527500091
ILMN_1681943	CDRT15P	-0.527551258
ILMN_3310593	SNORD116-29	-0.52758748
ILMN_1687655	DKFZp434J2031	-0.5276005
ILMN_3247261	RAPGEF2	-0.527602384
ILMN_1836172	CN288601	-0.527736043
ILMN_1795846	PPP2R5C	-0.527747258
ILMN_3236004	ZNF433	-0.527960071

ILMN_2331890	RPL41	0.556046707
ILMN_1808295	SMCR7	0.555991448
ILMN_1656380	LOC652276	0.555969983
ILMN_1712545	S100A3	0.55589111
ILMN_1762801	KLF12	0.555811999
ILMN_1792679	ITGA5	0.555770547
ILMN_2387636	EIF6	0.555701989
ILMN_1666444	RBMS1	0.555584551
ILMN_1764410	C22orf13	0.555493052
ILMN_2336133	SULT1A1	0.555195958
ILMN_1800461	CSNK2B	0.555195088
ILMN_3236270	ACAP2	0.555175418
ILMN_1669447	PYHIN1	0.555153363
ILMN_1672728	KCTD5	0.555123622
ILMN_1794470	ANKFY1	0.555107857
ILMN_2400030	PTPN2	0.555079976
ILMN_1702322	ALS2CL	0.554995319
ILMN_2323048	ERP29	0.554977704
ILMN_1737146	TRAM1	0.554970294
ILMN_2388539	C17orf101	0.554929249
ILMN_1730294	INO80C	0.554913029
ILMN_1740609	CCL15	0.554879786
ILMN_2234710	C12orf60	0.554772822
ILMN_1716679	FAIM	0.554758929
ILMN_3201663	RPL22	0.554281208
ILMN_1671437	MAP3K15	0.554279103
ILMN_1764362	LYAR	0.554162064
ILMN_1788283	COTL1	0.554121116
ILMN_1707804	SLX1B-SULT1A4	0.554033819
ILMN_2297824	UBE2G2	0.553958262
ILMN_1701461	TIMP3	0.553810311
ILMN_1672661	SP110	0.553808071
ILMN_1710495	PAPLN	0.553746291
ILMN_1722774	VPS72	0.553666612
ILMN_2157421	STUB1	0.553625456
ILMN_1749676	KIAA1704	0.553554011
ILMN_2336130	SLX1B-SULT1A4	0.553530842
ILMN_1728676	KIAA0196	0.553484149
ILMN_2364357	RPS6KB2	0.553344434
ILMN_2101034	CAPN12	0.553191989
ILMN_1714489	CIB2	0.553155775
ILMN_2330341	TCEAL4	0.553151803
ILMN_1803744	SELS	0.553145424
ILMN_1674228	abParts	0.55302198
ILMN_1755737	TRABD	0.55295331
ILMN_1707791	TAZ	0.552633163
ILMN_3205603	LOC729737	0.55257537
ILMN_1685403	MMP7	0.552451843
ILMN_2277077	PRKAR1A	0.552272582
ILMN_3239735	DKFZp434K1323	0.552209484
ILMN_1747386	GLIS3	0.552194584
ILMN_2374036	CTSL1	0.552031842
ILMN_2378081	BCL7A	0.551967355
ILMN_2186369	NCOR1	0.551946399
ILMN_1654998	SMEK1	0.551904392
ILMN_1752832	NPIPL2	0.55185267
ILMN_1757537	RGAG4	0.551753602
ILMN_1802100	ADAM28	0.551723901
ILMN_2307025	CPNE1	0.551695706
ILMN_1655225	C19orf25	0.551691567
ILMN_1762103	WDR48	0.551621848
ILMN_3306011	DQ581928	0.551577863
ILMN_1811775	CCDC124	0.551551552
ILMN_1711005	CDC25A	0.551287794
ILMN_1737205	MCM4	0.551224495
ILMN_1663512	COX5B	0.551186942
ILMN_3220265	CP	0.551139761
ILMN_2379130	IRAK1	0.551060052
ILMN_2170930	STRC	0.550967554
ILMN_3232889	CCDC144B	0.550674938
ILMN_2396292	ZNF655	0.550618276
ILMN_1681415	SIGLEC7	0.550614733
ILMN_3225938	RPL21	0.55058312
ILMN_1664051	SEC22C	0.550492308
ILMN_1723729	RSL1D1	0.550471315
ILMN_2377669	CD247	0.550443582
ILMN_1662065	WDR92	0.550304673
ILMN_2280053	CRCP	0.550143519
ILMN_1799837	POTEM	0.550105244
ILMN_2097790	C1orf86	0.550099036
ILMN_1740276	CLDN9	0.550083191
ILMN_2216838	DKFZP586I1420	0.549888811
ILMN_1781819	PAPSS1	0.54982449
ILMN_3253579	HAUS8	0.549703572
ILMN_1783673	LRRC37A2	0.549566492
ILMN_1676528	BTN3A2	0.549419859
ILMN_1738784	PPP2R5A	0.549294709
ILMN_1721495	ADAMTSL2	0.5492616
ILMN_2385866	PHF1	0.549196757
ILMN_1659752	AIDA	0.549051519
ILMN_2086417	NDUFV2	0.54901723
ILMN_2391976	SLC45A4	0.548952631
ILMN_1710413	C11orf95	0.54888568
ILMN_1774071	CD177	0.548731543
ILMN_1719759	TNC	0.54869356
ILMN_1686367	HSPA8	0.548687234
ILMN_3235832	CCL4L2	0.548666473
ILMN_1723884	ARF4	0.548554852

ILMN_1729816	VDAC3	-0.52796326
ILMN_1798433	LNPEP	-0.528038321
ILMN_1684563	SPIN4	-0.5280429
ILMN_1686862	HLX	-0.528049071
ILMN_3251545	CHMP5	-0.528141001
ILMN_1745811	TDRD3	-0.528155948
ILMN_1828225	AW118209	-0.528197218
ILMN_1856480	AK055323	-0.528222156
ILMN_1795672	PRH1-PRR4	-0.528236982
ILMN_1744656	PAGE5	-0.528242293
ILMN_1703244	MAP1LC3B	-0.528255291
ILMN_1797796	AMY1A	-0.528329962
ILMN_1735578	GUCA2B	-0.528380623
ILMN_1735905	LOC283685	-0.528411699
ILMN_1800488	CT47A1	-0.528416635
ILMN_2123119	MFSD8	-0.52852477
ILMN_2164152	ENPEP	-0.528567465
ILMN_1791623	LSP1	-0.528568916
ILMN_2195236	PGRMC2	-0.528590042
ILMN_1704576	AB045832	-0.528624065
ILMN_1766505	COMMD10	-0.528783155
ILMN_1901934	ZNF33B	-0.528832495
ILMN_1760199	CALB1	-0.528853391
ILMN_1661954	NCRNA00114	-0.528896237
ILMN_3238259	SNORA30	-0.528997457
ILMN_1696810	GPR182	-0.529033881
ILMN_1713529	SEMA6A	-0.529127616
ILMN_2246661	FAHD1	-0.529309741
ILMN_1708126	GOLGA6A	-0.529316536
ILMN_1717406	TTC34	-0.529330857
ILMN_1682206	GCC1	-0.529337345
ILMN_3310000	MIR369	-0.529380104
ILMN_2238928	RAD51C	-0.529402032
ILMN_3288525	NA	-0.529429147
ILMN_1703015	ZRANB2	-0.529515346
ILMN_1722446	CCDC60	-0.529567647
ILMN_1749586	ZNF252	-0.529574406
ILMN_1729476	PROL1	-0.529582387
ILMN_1897333	BX098521	-0.529603928
ILMN_1708152	BPIL2	-0.529624989
ILMN_1660103	NA	-0.529669087
ILMN_1671183	AMY2A	-0.529728708
ILMN_1769721	SP6	-0.529733388
ILMN_1908274	AW236020	-0.529803083
ILMN_2146372	KCTD6	-0.529807505
ILMN_1763365	FAM103A1	-0.529816034
ILMN_1893228	BX118370	-0.529819833
ILMN_1753135	ALK	-0.529884301
ILMN_1813517	DISP2	-0.529897247
ILMN_2382998	ZCCHC16	-0.529999699
ILMN_2376142	UBE2W	-0.530011589
ILMN_3246345	UCRP	-0.530259596
ILMN_1678882	KIR3DS1	-0.530279322
ILMN_1692978	PLA2G2D	-0.530373496
ILMN_1775686	FAM182B	-0.53041071
ILMN_2305116	CTH	-0.53045642
ILMN_1755354	YBX2	-0.530496796
ILMN_1671583	MKRN1	-0.530590205
ILMN_1719498	EPHX4	-0.530651558
ILMN_1718832	RAB4A	-0.530678082
ILMN_3248833	RPS26	-0.530719031
ILMN_1765017	COL6A2	-0.530725518
ILMN_1738122	MTL5	-0.530810605
ILMN_1803312	DIMT1L	-0.530844491
ILMN_1730575	GCLC	-0.530858282
ILMN_1680432	KRTAP4-7	-0.530871411
ILMN_1654151	COX6C	-0.530937997
ILMN_1802611	SPEN	-0.531037888
ILMN_1758164	STC1	-0.531077624
ILMN_1809652	UBE2E3	-0.531133045
ILMN_1749032	CREB3L1	-0.531243346
ILMN_1772057	COL28A1	-0.531315665
ILMN_1813296	RDH8	-0.531417797
ILMN_1673111	TSEN34	-0.531421455
ILMN_1863939	AW085986	-0.531458797
ILMN_3199529	FEM1A	-0.531478936
ILMN_1672122	P4HTM	-0.53148948
ILMN_1899147	AA904384	-0.531538745
ILMN_1668179	HNRNPF	-0.531543316
ILMN_1710075	FAM89A	-0.531554754
ILMN_1669034	CCL15	-0.531627357
ILMN_1679177	mars-02	-0.531814449
ILMN_1654583	CHD1	-0.531865007
ILMN_1846567	BX107471	-0.5318707
ILMN_1796749	KIF1C	-0.531903491
ILMN_1819225	LOC100506368	-0.531931905
ILMN_1737426	PCMTD1	-0.531948275
ILMN_3310201	MIR181C	-0.531994805
ILMN_1834324	BU753119	-0.532024037
ILMN_2407799	IL24	-0.53204838
ILMN_1761531	SGPL1	-0.532105772
ILMN_1717765	NUDT11	-0.532174433
ILMN_2305225	NDRG4	-0.532187152
ILMN_3248035	NTN5	-0.532190755
ILMN_1785439	CD79B	-0.532226322
ILMN_1698835	TMEM183B	-0.532407771
ILMN_1690888	CMIP	-0.53243938
ILMN_1781137	RPL36A	-0.532441083

ILMN_1795739	MPZL3	0.548470148
ILMN_1664631	ADAM28	0.548426626
ILMN_1724293	KDELR2	0.548317479
ILMN_1672461	SPPL2B	0.548287273
ILMN_1798123	ELOVL1	0.548175559
ILMN_2287157	BPAG1	0.548142558
ILMN_2390859	NFKB2	0.548091018
ILMN_1787248	SIVA1	0.548052612
ILMN_1750969	FAM120AOS	0.547999299
ILMN_1688848	TMEM44	0.547900205
ILMN_1690982	DDT	0.547870455
ILMN_2307740	CD46	0.547833741
ILMN_1651315	HMG20B	0.547793411
ILMN_2140990	CAMK1	0.547618723
ILMN_2400661	ZNF626	0.547605479
ILMN_1789349	UBQLN4	0.547491933
ILMN_1713732	ABL1	0.54742742
ILMN_2411511	KLK12	0.547210037
ILMN_1770940	CDH1	0.547140166
ILMN_1663884	ARHGEF35	0.547115466
ILMN_3244987	KIAA0895L	0.54704598
ILMN_2398274	PYCARD	0.547045021
ILMN_1664098	FASTK	0.546986088
ILMN_1702911	PKIG	0.546965936
ILMN_3245476	PHRF1	0.546915262
ILMN_1741264	MRPS33	0.546913403
ILMN_1663035	SREBF1	0.546804779
ILMN_1797822	SEL1L3	0.546716008
ILMN_1776080	GTPBP6	0.546659277
ILMN_1804863	FKBP15	0.546610336
ILMN_1691476	MYLK	0.546545297
ILMN_1723944	TARP	0.546542397
ILMN_2394902	ACCN4	0.546539458
ILMN_1727671	SSH1	0.546490813
ILMN_1770548	CLASP2	0.546486607
ILMN_3241692	MICA	0.546402273
ILMN_1755024	IKBKE	0.54639941
ILMN_2377459	CYTH2	0.546364113
ILMN_2106227	KIAA2026	0.546265745
ILMN_1836776	MPRIP	0.54620522
ILMN_2116127	NPEPPS	0.546099902
ILMN_1696591	RB1	0.54597252
ILMN_1657893	TXNRD2	0.5458622
ILMN_2381121	UQCC	0.545781034
ILMN_1724686	CLDN1	0.545684949
ILMN_3197097	TSTD1	0.545522563
ILMN_3245923	AK301885	0.545437893
ILMN_2397028	SERPINB8	0.545383341
ILMN_1759184	C19orf48	0.545090743
ILMN_3186158	NA	0.545077695
ILMN_1719656	MRPL38	0.545049384
ILMN_1657624	NSFL1C	0.544989647
ILMN_1772387	TLR2	0.544908626
ILMN_1687235	HPN	0.544905253
ILMN_3248608	DQ594366	0.544903164
ILMN_1761939	TIPIN	0.544807336
ILMN_2405915	MRPS11	0.544740041
ILMN_1743275	SH3RF3	0.544704479
ILMN_1763828	MTF1	0.5447022
ILMN_1732810	SNX17	0.544513611
ILMN_1729123	PPP2R4	0.544420882
ILMN_1808661	TOMM5	0.544410004
ILMN_1661484	ZBTB45	0.544381248
ILMN_1807825	LY86	0.544366888
ILMN_1738147	NES	0.544326304
ILMN_1780598	PIAS1	0.544312478
ILMN_1748591	ODC1	0.544124687
ILMN_1728521	HDAC7	0.544061861
ILMN_2313079	NLRP1	0.544059475
ILMN_2412761	MAFG	0.543916184
ILMN_2367113	CASP6	0.54388427
ILMN_1795922	ZNF830	0.543815951
ILMN_3238707	TAF1D	0.543800779
ILMN_1751708	ITM2B	0.543733739
ILMN_1730391	MRPS18A	0.543664502
ILMN_2268381	RRBP1	0.54362442
ILMN_1737387	GGT2	0.543579454
ILMN_1810045	NLRP1	0.543425088
ILMN_1774836	PLOD3	0.543403167
ILMN_1666049	NUP214	0.543341464
ILMN_1657631	STAP2	0.543272487
ILMN_1757978	INO80D	0.543261268
ILMN_1799819	MARS	0.54322654
ILMN_2379520	HAGH	0.543178362
ILMN_1730329	MED17	0.543141517
ILMN_1673738	RPL6	0.543140094
ILMN_1769606	PIWIL4	0.543110775
ILMN_1658261	MAPKBP1	0.542943202
ILMN_2347380	ARHGEF11	0.542920845
ILMN_2069267	BAGE	0.54290506
ILMN_1764788	TNFRSF1B	0.542880447
ILMN_1682953	PGAM4	0.542661504
ILMN_1767135	SOS1	0.542622676
ILMN_2409384	SIGLEC7	0.542471169
ILMN_3233753	AK293020	0.542444267
ILMN_2231051	TCP11L2	0.542403923
ILMN_1815759	CTDP1	0.542342455
ILMN_1741003	ANXA5	0.542263676

ILMN_1788650	GPHB5	-0.532510633
ILMN_1658108	BC033582	-0.532512447
ILMN_1786275	PAPOLG	-0.532555341
ILMN_1668490	UNCX	-0.532658306
ILMN_1741483	HPS1	-0.532718182
ILMN_1776213	RGMB	-0.532853568
ILMN_1659885	ACSS3	-0.532870249
ILMN_1826616	BQ029125	-0.53288203
ILMN_1739854	C15orf29	-0.532902866
ILMN_1673553	PTH2	-0.532907273
ILMN_1745038	TAS2R4	-0.533042138
ILMN_1732913	LRP6	-0.533136261
ILMN_1849744	DA584132	-0.533166844
ILMN_1718687	TPPP	-0.533202081
ILMN_1708982	OR10K2	-0.533350103
ILMN_1668035	CRH	-0.533363865
ILMN_2148452	BCAS2	-0.533367115
ILMN_2191634	RPL37	-0.53340909
ILMN_1750885	LOC644100	-0.533445309
ILMN_3308460	MIR135A2	-0.533539983
ILMN_1731051	AF125949	-0.533563411
ILMN_3272227	PLEKHA6	-0.533594818
ILMN_3249598	RGS21	-0.533785511
ILMN_1844128	BC040862	-0.533803626
ILMN_2386100	BUB3	-0.53398527
ILMN_2118621	CDCP2	-0.534042474
ILMN_1885583	AK129941	-0.53414883
ILMN_1808306	SLC26A1	-0.534163948
ILMN_1859614	BF511034	-0.534183789
ILMN_1654001	SMC6	-0.534349892
ILMN_1774091	FECH	-0.534355334
ILMN_1860084	AW237078	-0.534386006
ILMN_1776325	UBE2Q1	-0.534430931
ILMN_1756852	AUP1	-0.534458304
ILMN_3252901	LOC100128105	-0.534500127
ILMN_1716729	AK129567	-0.534565247
ILMN_2094905	COMMD10	-0.534583547
ILMN_3296436	LOC100130992	-0.534584228
ILMN_1683604	TARM1	-0.534621328
ILMN_1743992	DCP1B	-0.534633143
ILMN_1871361	AX747287	-0.534636393
ILMN_1748601	CD8B	-0.534654813
ILMN_1803308	NA	-0.534675391
ILMN_1700202	TMEM135	-0.534680482
ILMN_1679029	ZXDA	-0.534724852
ILMN_1792744	SLC13A2	-0.53481082
ILMN_1703140	NEDD4	-0.534982604
ILMN_1889633	BC071821	-0.535057782
ILMN_1702700	AF257770	-0.535070006
ILMN_1666403	GCSH	-0.53519158
ILMN_1786125	CCNA2	-0.535238391
ILMN_1863114	AI791835	-0.535260363
ILMN_1751102	AMY2A	-0.53526207
ILMN_1662848	TXNDC15	-0.535288042
ILMN_2342554	TAGLN3	-0.535292385
ILMN_1907266	AK057317	-0.535306987
ILMN_3245811	ODF3	-0.535444591
ILMN_1671265	ING2	-0.535467847
ILMN_1799100	PTX4	-0.535533814
ILMN_3237286	LOC100288884	-0.535543737
ILMN_1732555	B4GALT6	-0.535578819
ILMN_3203059	LOC100131176	-0.535702658
ILMN_2153485	NMNAT3	-0.535721477
ILMN_2375156	PHF7	-0.535762823
ILMN_1733396	CDC25A	-0.535849041
ILMN_1741971	KIAA1161	-0.536134296
ILMN_3306997	METTL1	-0.536191817
ILMN_1824554	DA570923	-0.53624704
ILMN_1750130	GSPT1	-0.536321532
ILMN_3309164	MIR1185-1	-0.536324818
ILMN_1667230	C21orf59	-0.536328268
ILMN_1748927	IQCF5	-0.536360427
ILMN_1722209	IL21R	-0.536374828
ILMN_1825179	CD516455	-0.536435296
ILMN_1722884	CR596520	-0.536561716
ILMN_1751465	BNC1	-0.536585185
ILMN_3234892	MARCH11	-0.536586453
ILMN_3275615	LOC645249	-0.536689284
ILMN_1679979	PLK3	-0.536690663
ILMN_1853876	TMEM64	-0.536791335
ILMN_1673535	MYLK4	-0.536834827
ILMN_1751576	TEK	-0.536901853
ILMN_1685978	ATPIF1	-0.536960534
ILMN_1701454	OR2L13	-0.53700668
ILMN_3236289	TIGIT	-0.537093659
ILMN_1889215	BF434110	-0.537106654
ILMN_1761294	OR4K15	-0.537136716
ILMN_1720794	SNORD4B	-0.537175271
ILMN_1765159	ELMOD2	-0.537256775
ILMN_1671678	TECR	-0.537258021
ILMN_1742775	ZNRF1	-0.537259358
ILMN_1764415	ZNF585A	-0.537289014
ILMN_3247792	LOC100169752	-0.537424552
ILMN_1659010	AK124016	-0.53745543
ILMN_1730658	YTHDF2	-0.537491749
ILMN_1772646	PDE4DIP	-0.537579839
ILMN_1879135	BM673749	-0.537674282
ILMN_1783287	S100A14	-0.537708365

ILMN_1750658	HAX1	0.542229238
ILMN_1722855	VEGFB	0.542216226
ILMN_1656840	VPS13D	0.542139297
ILMN_1716907	TMEM214	0.542116006
ILMN_1756501	ST6GAL1	0.542097428
ILMN_2212354	WDR46	0.542096377
ILMN_1701466	PEX16	0.541850809
ILMN_1753340	GGTLC2	0.541827883
ILMN_1724825	PCBP2	0.541822453
ILMN_1813475	HERC2	0.54175676
ILMN_1669635	NUP85	0.541712927
ILMN_2367469	CARS	0.541700379
ILMN_2385178	MIB2	0.541674885
ILMN_1683175	C9orf23	0.541668346
ILMN_2318568	HCFC1R1	0.541611666
ILMN_2120103	AGAP7	0.541525138
ILMN_3271412	SUMO2	0.54152181
ILMN_3247732	ZRSR2	0.541514807
ILMN_1676448	WDFY1	0.541479618
ILMN_2342579	IL7R	0.54145546
ILMN_1795119	MYH3	0.541430329
ILMN_1651236	GGT1	0.541423039
ILMN_3310840	MIR21	0.541388381
ILMN_1682778	MXRA8	0.541303394
ILMN_3213792	PPIA	0.54124109
ILMN_2362847	PPARA	0.541217634
ILMN_1682912	LOC100132287	0.541143475
ILMN_2267787	HPS1	0.5408451
ILMN_2352326	COASY	0.540576871
ILMN_2326578	NRF1	0.540478337
ILMN_1654287	ADCY9	0.54020405
ILMN_1675488	SLC13A3	0.54019794
ILMN_2181125	NAPB	0.540189338
ILMN_1661589	CD151	0.540117332
ILMN_1767717	SLC2A11	0.54005833
ILMN_2349124	OSBPL2	0.539949536
ILMN_2391051	FCGR1B	0.539873093
ILMN_3289508	CR604709	0.539691863
ILMN_1751887	PREP	0.539617719
ILMN_2355738	INCENP	0.539576879
ILMN_1742781	KIRREL	0.539526951
ILMN_2319344	APEX1	0.539395004
ILMN_1805344	DDX5	0.539366843
ILMN_1699112	COPB1	0.539186748
ILMN_1790562	EYA3	0.539129411
ILMN_2211950	SRP14	0.539074989
ILMN_3236373	MSL2	0.538974533
ILMN_1781155	LYN	0.538868168
ILMN_1717461	BC047566	0.538819387
ILMN_1800302	LRTM1	0.538798337
ILMN_3185453	FLJ10661	0.538721271
ILMN_1712400	SERPINB6	0.538713818
ILMN_1711189	EXOSC10	0.538637909
ILMN_1664960	KIAA0284	0.538631958
ILMN_1661266	HLA-DQB1	0.538383765
ILMN_2233334	MYH16	0.538259653
ILMN_1766245	SUPT4H1	0.538167372
ILMN_2340877	MEN1	0.53788514
ILMN_2404688	NUPR1	0.537860896
ILMN_2140895	hCG_1646325	0.537733919
ILMN_2388090	MAPK14	0.53758305
ILMN_1698144	CFH	0.537536593
ILMN_1755657	RASIP1	0.537504532
ILMN_3233135	FAM178A	0.537479012
ILMN_1708304	C11orf16	0.537433404
ILMN_1691662	LOC100101266	0.537321273
ILMN_1770356	POLRMT	0.537297131
ILMN_1722905	MRPS11	0.537256063
ILMN_1773567	LAMA5	0.537218604
ILMN_1689111	CXCL12	0.536992172
ILMN_2077094	C11orf2	0.53681035
ILMN_1729580	NUDT9	0.53669573
ILMN_3207627	NUS1	0.536587496
ILMN_1757644	UBE2H	0.536586495
ILMN_1767722	AFTPH	0.536548965
ILMN_2370685	C6orf1	0.536439852
ILMN_1672004	TOB1	0.536365093
ILMN_1701216	BANP	0.536351685
ILMN_2319292	PAX8	0.536345178
ILMN_1787514	CAPN12	0.536342938
ILMN_1788604	WBP2	0.536266292
ILMN_1787673	PLLP	0.53626291
ILMN_2178226	KRT86	0.536205852
ILMN_2192072	MMP7	0.536179424
ILMN_1655561	ARPC3	0.535981614
ILMN_1786045	SLC24A5	0.535980178
ILMN_1700681	CD99L2	0.535746095
ILMN_1695946	TRNP1	0.535703543
ILMN_2338447	ARID4A	0.535702598
ILMN_2270100	C6orf204	0.535616998
ILMN_1677466	DUSP6	0.535580193
ILMN_3283015	NSA2	0.535525073
ILMN_1693394	BCKDK	0.535516944
ILMN_1760688	SAMD14	0.535506964
ILMN_1726289	C12orf35	0.535473852
ILMN_1671374	C19orf53	0.535459705
ILMN_1752451	CTSH	0.535127895
ILMN_1655720	CNNM4	0.53504077

ILMN_1770641	KLHL3	-0.537790774
ILMN_1697469	SRSF6	-0.537987811
ILMN_1853920	BE220010	-0.538070607
ILMN_3245707	RIMKLB	-0.538092958
ILMN_3251398	KRTAP4-11	-0.538160907
ILMN_1690796	OR4F21	-0.5382357
ILMN_1699011	GTDC1	-0.538238891
ILMN_1800445	NEK10	-0.538268198
ILMN_1796136	RAB37	-0.538332181
ILMN_2367671	LIMK2	-0.538333712
ILMN_1708482	TMEM80	-0.538438576
ILMN_1716658	ZNF543	-0.538471706
ILMN_1799020	MUC12	-0.538582241
ILMN_1728883	AB074415	-0.538704771
ILMN_1773395	BLOC1S1-RDH5	-0.538837959
ILMN_1773289	RFX4	-0.53891441
ILMN_3281719	LOC100131174	-0.539022976
ILMN_2167011	ECHDC1	-0.539186822
ILMN_2350607	C20orf7	-0.539241988
ILMN_1768719	RDH11	-0.539289812
ILMN_1795949	CORO7	-0.539320132
ILMN_2166457	HPGD	-0.539380524
ILMN_2052208	GADD45A	-0.539559446
ILMN_1784678	PBX1	-0.539612578
ILMN_1728933	GOLGA6L1	-0.539626258
ILMN_1710354	KIFC3	-0.539784686
ILMN_1813427	FLJ41327	-0.539830729
ILMN_1666364	COQ10A	-0.540078269
ILMN_1696537	DDIT4L	-0.540110826
ILMN_3250110	LOC283116	-0.540213531
ILMN_3227263	SLC22A23	-0.540285435
ILMN_1720267	TARSL2	-0.540458407
ILMN_1696751	AI636007	-0.540467477
ILMN_1802192	C10orf99	-0.540747559
ILMN_2389810	ATP11C	-0.540871493
ILMN_1662852	IQCK	-0.540915052
ILMN_2312304	INTS6	-0.540935378
ILMN_1721758	ID4	-0.541142078
ILMN_1811880	SLC34A3	-0.541191378
ILMN_2384158	CNGA3	-0.541302467
ILMN_1670725	SYT16	-0.541335223
ILMN_1776577	DSCC1	-0.541352258
ILMN_2198408	MFF	-0.541373972
ILMN_1769280	SPRY1	-0.541473388
ILMN_1709635	SLC38A11	-0.54150317
ILMN_3239568	MYLK4	-0.541586368
ILMN_1651660	TMEM191C	-0.541598727
ILMN_1891927	DCBLD1	-0.541700661
ILMN_2176955	CUL5	-0.54177613
ILMN_1765417	RBBP9	-0.541856616
ILMN_1692705	RECQL	-0.541867697
ILMN_2284222	CDK18	-0.541933755
ILMN_2075292	MAGEA9B	-0.542009029
ILMN_1731137	TXNDC9	-0.542015932
ILMN_1755869	OR51T1	-0.542022023
ILMN_2151304	KERA	-0.542049635
ILMN_3202093	TRIM64B	-0.542156289
ILMN_1753472	SUMF1	-0.542157272
ILMN_1754913	WDR88	-0.542204892
ILMN_1740007	TMPRSS11E	-0.542256616
ILMN_2175764	HSP90Bb	-0.542280619
ILMN_3244755	NA	-0.54230176
ILMN_1723287	SLC6A16	-0.542551788
ILMN_1896082	CA429570	-0.542608698
ILMN_3309374	MIR1301	-0.542753132
ILMN_1694589	PAQR8	-0.54277497
ILMN_1776576	C1QL3	-0.542879033
ILMN_3235803	AQP7P3	-0.542920404
ILMN_1781073	PTPN5	-0.542942627
ILMN_1741134	WDR89	-0.542964113
ILMN_3266304	LOC100129098	-0.543018402
ILMN_1889767	NA	-0.543022592
ILMN_1735908	UTP15	-0.543039066
ILMN_1730562	KRTAP7-1	-0.543042989
ILMN_3240538	C19orf38	-0.543072699
ILMN_2073012	TMEM203	-0.543094312
ILMN_3242325	BSPH1	-0.543118234
ILMN_1715255	MAGI2	-0.543124103
ILMN_1804562	SLC31A1	-0.543147026
ILMN_3235143	LOC731779	-0.543163253
ILMN_1798372	ANX2P2	-0.543301654
ILMN_1806683	LCE4A	-0.543316736
ILMN_1739582	HOXA10	-0.543546003
ILMN_2174045	RNF126P1	-0.543547187
ILMN_1866563	DB341223	-0.543641341
ILMN_3303988	BM719313	-0.543682523
ILMN_1757287	MAPK6	-0.543750109
ILMN_3219380	NANOGNB	-0.543825134
ILMN_1666596	HSPB9	-0.543912953
ILMN_1687865	TMOD3	-0.543934459
ILMN_3247653	LRFN1	-0.543963697
ILMN_2192779	GPR15	-0.544035234
ILMN_3293244	LOC100131744	-0.544053958
ILMN_1724669	RUNX1T1	-0.544085868
ILMN_3251298	THNSL1	-0.544127887
ILMN_3251751	MFSD8	-0.54426949
ILMN_2358718	CHRM2	-0.544407297
ILMN_3249820	RIIAD1	-0.54470007

ILMN_1763267	ZFYVE28	0.534972
ILMN_1770035	NCOA5	0.534948619
ILMN_2329834	CPNE1	0.534760225
ILMN_1807467	RGNEF	0.534731657
ILMN_1784780	CIC	0.534684041
ILMN_1790149	HGF	0.534658546
ILMN_1651657	ADAM33	0.534572917
ILMN_1698386	NEK9	0.534547876
ILMN_1756469	GAMT	0.534525639
ILMN_1755942	O3FAR1	0.534489319
ILMN_2328972	DNMT3B	0.534147315
ILMN_1661647	LOC349114	0.534122605
ILMN_1695362	ZNF32	0.533963436
ILMN_1694801	SLC39A4	0.533954842
ILMN_1660723	RDH13	0.533945494
ILMN_2154853	C14orf50	0.533916924
ILMN_1742808	SF1	0.533858637
ILMN_1773228	DLST	0.533777154
ILMN_1727173	SMARCD1	0.533769824
ILMN_1723156	PTPRA	0.533488614
ILMN_1685002	RCE1	0.533429185
ILMN_1756920	ADAM15	0.533422431
ILMN_1749667	OBSCN	0.5334162
ILMN_2358069	MAD1L1	0.533393302
ILMN_2191759	HNRNPA1	0.533383554
ILMN_1729533	APOA1BP	0.533292157
ILMN_1655137	ZCCHC11	0.533291834
ILMN_1741334	IKZF4	0.533278614
ILMN_1681340	HSPB11	0.533150382
ILMN_1683660	EIF3H	0.533147099
ILMN_3236935	ARHGDIA	0.533129552
ILMN_1765547	IRF2	0.532980201
ILMN_1696110	TUBGCP3	0.532927284
ILMN_1813275	DUSP22	0.532765681
ILMN_1792910	MNT	0.532696659
ILMN_2316173	AP1S1	0.532649346
ILMN_1716276	CCL4	0.532622653
ILMN_1769911	SLC38A1	0.532599739
ILMN_1852224	NA	0.5325967
ILMN_1695763	PDIA5	0.532570875
ILMN_1724064	RPS2P32	0.532517725
ILMN_1812976	DDX54	0.532398099
ILMN_2387175	WDR31	0.532361853
ILMN_2215211	ZNF514	0.532349033
ILMN_1705213	TMBIM1	0.53216113
ILMN_1718299	PDE6G	0.532087042
ILMN_1790918	ZNF236	0.532068655
ILMN_2409793	MAZ	0.531944521
ILMN_1726698	PSME1	0.531890569
ILMN_1657722	RPS3A	0.531790833
ILMN_1677402	C11orf96	0.531759126
ILMN_1802151	OSBPL5	0.531755303
ILMN_1806349	SLC6A8	0.531584392
ILMN_3255971	NA	0.531542184
ILMN_1679984	ZCCHC12	0.531457256
ILMN_1672081	NEURL	0.531386899
ILMN_1655765	MRPS21	0.531357772
ILMN_1711030	OPLAH	0.531335249
ILMN_1809957	AP2S1	0.53132647
ILMN_1688231	TREM1	0.53128971
ILMN_1725597	FXYD4	0.531227263
ILMN_3205924	EIF3F	0.531103051
ILMN_2366334	FERMT3	0.53108053
ILMN_1736700	ALDOA	0.53100245
ILMN_1718420	LCE1D	0.530965835
ILMN_3247154	ZNF799	0.530912208
ILMN_1726107	UBE2V1	0.530903685
ILMN_1748970	PRR15L	0.530875895
ILMN_2378654	B3GALT5	0.530855601
ILMN_1733579	EVI2A	0.530756489
ILMN_3247848	UNQ2487	0.530748531
ILMN_1773018	CUEDC2	0.530615576
ILMN_2068499	POU5F1	0.530322011
ILMN_1783753	TXNDC12	0.530319702
ILMN_2048507	KLF3	0.5302921
ILMN_1741585	SFMBT1	0.530206998
ILMN_2265082	SRSF10	0.530174465
ILMN_1756650	DGKH	0.530077937
ILMN_2227385	SLC16A14	0.530023011
ILMN_2103397	ZNF234	0.529975777
ILMN_3246957	DKFZp666P158	0.529961006
ILMN_2140059	LAMA4	0.529922179
ILMN_1718449	NSUN5P2	0.529762937
ILMN_2238389	LDB3	0.529456192
ILMN_1709814	NMRAL1	0.529343954
ILMN_1668876	HECTD2	0.52931401
ILMN_1814808	BFAR	0.529223526
ILMN_1697499	HLA-DRB1	0.52916615
ILMN_1772929	ATP5J	0.529146169
ILMN_1772466	SH2D3A	0.52914615
ILMN_1691926	HSD17B10	0.529097842
ILMN_1807072	GOSR2	0.529093683
ILMN_1800096	MPST	0.529049515
ILMN_3192001	CCL15	0.529006433
ILMN_2353862	LRRC48	0.528991529
ILMN_1681542	HIST1H4E	0.528930419
ILMN_1678268	VPS8	0.528743947
ILMN_2272270	COL4A3	0.528737399

ILMN_1772603	LOC644172	-0.544731758
ILMN_1690480	FLJ25037	-0.544736567
ILMN_1738751	LOC728175	-0.544795699
ILMN_1700086	DOK1	-0.544823727
ILMN_3224922	BC112987	-0.544965735
ILMN_2162298	DYSFIP1	-0.544984374
ILMN_1782448	IFNA4	-0.545041089
ILMN_1729281	SPHK2	-0.54505441
ILMN_1685441	ASAP3	-0.545074721
ILMN_2377174	SYPL1	-0.54511237
ILMN_1721365	ZNF847P	-0.545141947
ILMN_2297205	ATRN	-0.545179805
ILMN_2275533	DIAPH3	-0.545198024
ILMN_1711514	COCH	-0.545222756
ILMN_2124361	LEAP2	-0.545258022
ILMN_1810327	HNRNPUL2	-0.545343015
ILMN_2069224	PVALB	-0.545372317
ILMN_1729225	SORCS2	-0.545562323
ILMN_1680353	NSF	-0.545661197
ILMN_3308555	SNORD113-7	-0.545674734
ILMN_1870529	AU151944	-0.545703173
ILMN_1730851	PDE6C	-0.545706679
ILMN_1819982	NA	-0.545778064
ILMN_3188180	CR599196	-0.54581573
ILMN_1788538	NCALD	-0.545833937
ILMN_1778301	C6orf132	-0.545902698
ILMN_1657158	RNF168	-0.54591253
ILMN_1722850	VGLL2	-0.545998209
ILMN_2353030	GRAP	-0.546078802
ILMN_1762192	KIF9	-0.546131414
ILMN_1815239	AX746743	-0.546301479
ILMN_1889669	C17orf107	-0.546460005
ILMN_2174215	DIRAS3	-0.546465863
ILMN_1681520	DGAT2	-0.546508748
ILMN_1850374	BG196443	-0.54652512
ILMN_1771964	GSTA4	-0.546540599
ILMN_1765255	CDH26	-0.546558247
ILMN_1797332	NARS2	-0.546668889
ILMN_2150284	RNPC3	-0.54677517
ILMN_1853798	AA398758	-0.54678684
ILMN_1866952	BG698340	-0.546787289
ILMN_1805063	GSG1	-0.546819209
ILMN_1677292	C5orf30	-0.546908178
ILMN_1682399	CLOCK	-0.546965754
ILMN_3279707	AK123890	-0.546992738
ILMN_1725971	TMEM81	-0.547261668
ILMN_1760320	GNB1	-0.547418384
ILMN_1807176	TAF3	-0.547429505
ILMN_1901341	DA953700	-0.547532198
ILMN_2186351	CATSPER2P1	-0.547532754
ILMN_1720531	KDM4B	-0.547533316
ILMN_1793743	DIRC2	-0.547599103
ILMN_2231177	OR7E24	-0.547705317
ILMN_2273053	IL15	-0.5477148
ILMN_1887709	DA654953	-0.547899688
ILMN_3242633	SNORD127	-0.547927595
ILMN_3181565	AK097991	-0.547957166
ILMN_1739734	CLTCL1	-0.54799126
ILMN_2212590	TMEM170A	-0.548008024
ILMN_1738955	ANAPC16	-0.548032026
ILMN_1705346	NBEA	-0.548042101
ILMN_1705689	ADAM12	-0.548047367
ILMN_1880113	BX095318	-0.54814033
ILMN_1749540	RBM20	-0.54817952
ILMN_1801938	NHS	-0.548444477
ILMN_1720844	SSX2IP	-0.548466263
ILMN_1674678	RBMY1A1	-0.548501282
ILMN_1761058	NPHP3-ACAD11	-0.548568247
ILMN_1782084	ALG1L	-0.54866893
ILMN_2244699	MAPK7	-0.548701103
ILMN_1784207	PITHD1	-0.548803897
ILMN_3241262	PABPC4L	-0.548824858
ILMN_1727923	ZNF140	-0.549029457
ILMN_1703678	DNM1P33	-0.549139774
ILMN_2297626	PEG10	-0.549202464
ILMN_1808290	LOC286467	-0.549331181
ILMN_1751911	MESP2	-0.549354906
ILMN_1766247	ZBTB2	-0.549753965
ILMN_1762634	OR10K1	-0.549956159
ILMN_2365595	GMPR2	-0.549995447
ILMN_1765454	KIAA1783	-0.550076951
ILMN_1793549	PTPN4	-0.550085832
ILMN_1908074	AV750156	-0.550249106
ILMN_3281480	LOC201651	-0.550422548
ILMN_1754570	KCTD8	-0.550426216
ILMN_1816870	BE466563	-0.550450553
ILMN_1806703	LOC643037	-0.55059665
ILMN_3270972	ASAP2	-0.550618552
ILMN_1894822	BU570069	-0.550621799
ILMN_1749410	PKP4	-0.550646632
ILMN_3309559	MIR124-2	-0.550693006
ILMN_1668453	TRIM36	-0.55075792
ILMN_1685496	RGS7	-0.550758826
ILMN_1685805	FLJ25758	-0.550884824
ILMN_1852756	BG201089	-0.550893781
ILMN_1770479	LMO7	-0.550930943
ILMN_2100357	SMPDL3A	-0.550957532
ILMN_1658369	TACR3	-0.551029379

ILMN_3223871	ISPD	0.52868958
ILMN_2219512	NPHP4	0.528638501
ILMN_1799082	ZNF609	0.528462937
ILMN_3231390	NHP2	0.528366523
ILMN_1759991	NOL12	0.528246995
ILMN_2278413	VCX3A	0.528150016
ILMN_1741406	HOOK1	0.528133577
ILMN_2075051	PGS1	0.528092492
ILMN_1770733	RIC8A	0.528077418
ILMN_1675055	MUL1	0.528068243
ILMN_2049021	PTTG3	0.528042838
ILMN_1739368	ZNF498	0.5280338
ILMN_2365111	MAP4K1	0.528024589
ILMN_3276019	RPL23AP7	0.527997782
ILMN_1717975	CYHR1	0.527980666
ILMN_2330307	SLC43A3	0.527944683
ILMN_2051867	PTCD2	0.527820145
ILMN_2154950	ZNF423	0.527773762
ILMN_1810875	SYNGR1	0.527645096
ILMN_1743347	AKT2	0.527591771
ILMN_2210739	CDH12	0.527449436
ILMN_1812478	ZNHIT3	0.527428352
ILMN_2371169	ZYX	0.527369176
ILMN_1752953	BCL2L12	0.527287577
ILMN_2409167	ANXA2	0.527276464
ILMN_1686360	ZNF124	0.527187365
ILMN_2124816	ZNF34	0.527077907
ILMN_1698427	TMUB2	0.527056828
ILMN_1781234	CCDC22	0.527053745
ILMN_1737168	THTPA	0.527008793
ILMN_1665449	ROBO4	0.526990326
ILMN_1847308	FAM168A	0.526861887
ILMN_1732705	HCFC1	0.526830369
ILMN_1739400	PAX2	0.526765809
ILMN_1655893	NA	0.526577173
ILMN_1708959	MFN1	0.526570104
ILMN_1684034	STAT5B	0.526522227
ILMN_1734964	P2RX5	0.526503942
ILMN_1735979	BCKDHA	0.526471418
ILMN_1686116	THBS1	0.526316519
ILMN_1782050	CEBPD	0.526311257
ILMN_1707529	TMTC1	0.526228385
ILMN_1695576	MRPL24	0.526139559
ILMN_1655167	ZNF502	0.526044133
ILMN_1701114	GBP1	0.525866439
ILMN_1691916	KIAA1919	0.52586341
ILMN_2405305	ARNTL	0.525810503
ILMN_1672606	OAS1	0.525552098
ILMN_1658290	METTL22	0.525551979
ILMN_1805415	PDE3A	0.525501228
ILMN_1730291	ATP1B1	0.525492118
ILMN_2398039	TCERG1	0.525490282
ILMN_1734950	LOXL1	0.525356521
ILMN_1653438	PHF14	0.52530214
ILMN_1707088	DENND2D	0.525155731
ILMN_1751572	TLE1	0.525146831
ILMN_2249552	GTF2A1	0.525137061
ILMN_1744508	FAM53C	0.524949967
ILMN_3307729	CXXC5	0.524938267
ILMN_1706326	MRPL33	0.52486245
ILMN_1796490	GRINA	0.524766723
ILMN_1779828	EDEM1	0.524540888
ILMN_1813639	NKAIN4	0.524441169
ILMN_1657888	SALL1	0.524321482
ILMN_2373632	IDH3B	0.524260876
ILMN_2070013	SLC25A15	0.524253532
ILMN_2348788	CD44	0.524229178
ILMN_1762861	HLA-F	0.524224708
ILMN_1752333	SLC35E1	0.524200028
ILMN_2143822	ZNF148	0.524185342
ILMN_1656791	RPS6	0.524129558
ILMN_1749789	HIST1H1D	0.52412912
ILMN_2371700	HAUS7	0.524074397
ILMN_1676984	DDIT3	0.523929721
ILMN_1691276	CXXC1	0.523899544
ILMN_1813671	SLC25A1	0.523851164
ILMN_1658677	DTX3	0.523620143
ILMN_1749553	ENTPD2	0.52359935
ILMN_1703650	TNIP1	0.523586044
ILMN_1792176	TADA3	0.523572573
ILMN_2065745	RAMP3	0.52356805
ILMN_2357134	SPHK1	0.523545126
ILMN_1677765	LRP8	0.523538686
ILMN_1784105	SLC38A7	0.52331718
ILMN_1722292	AVL9	0.523256819
ILMN_1750181	TESC	0.523225359
ILMN_1726189	MS4A14	0.523204352
ILMN_1748908	PROSC	0.523202581
ILMN_1673129	PCDH12	0.52317076
ILMN_1694686	HMGXB3	0.523160047
ILMN_2379599	CD163	0.522967416
ILMN_3248906	ZC4H2	0.522772572
ILMN_2234873	NME1	0.522690164
ILMN_1712389	CMTM1	0.522640904
ILMN_1784785	COPS7B	0.522620874
ILMN_2401618	MLX	0.52251382
ILMN_1654016	MYL12B	0.522507458
ILMN_1670609	ATOX1	0.522496934

ILMN_3298993	NA	-0.551078163
ILMN_3242164	SNORD59B	-0.551111173
ILMN_2382121	OCRL	-0.551126133
ILMN_2116516	SCRN3	-0.551212324
ILMN_1664235	BCL2L14	-0.551249217
ILMN_1743638	ABCG1	-0.551322971
ILMN_1706015	FAM43A	-0.55148131
ILMN_3263393	OR7E156P	-0.551522353
ILMN_1719749	PTGES3	-0.551583711
ILMN_1669350	DKFZp434I1020	-0.551597082
ILMN_2327812	IL5RA	-0.551650263
ILMN_1810488	NFYC	-0.551654414
ILMN_1913413	BX105847	-0.551661461
ILMN_1824136	SAMD12	-0.551673805
ILMN_1706410	HUS1B	-0.551689745
ILMN_1765044	CUTC	-0.551772809
ILMN_2405353	SDK1	-0.551824416
ILMN_1791306	C9orf103	-0.55187542
ILMN_1789786	NA	-0.551913971
ILMN_3191655	NA	-0.551962476
ILMN_2169761	CPNE8	-0.551975346
ILMN_1767590	TSPY3	-0.552077969
ILMN_1783313	VOPP1	-0.552226183
ILMN_3267631	LOC100130827	-0.55224386
ILMN_1675321	PSMC3IP	-0.552246644
ILMN_1854469	AK130049	-0.55228309
ILMN_2228809	YWHAG	-0.552441853
ILMN_1833027	AI936635	-0.552450134
ILMN_3247152	RIMBP3	-0.552499407
ILMN_1795976	SFXN2	-0.552536962
ILMN_2287061	HIF3A	-0.552605721
ILMN_1703511	PDZRN3	-0.552705667
ILMN_1779032	SDHC	-0.552722075
ILMN_1905072	CX758427	-0.552727766
ILMN_1705458	OR2W3	-0.552797848
ILMN_1745141	MBL1P1	-0.552825491
ILMN_1706579	SHBG	-0.552836216
ILMN_2279844	LCK	-0.55288553
ILMN_1659601	CCL26	-0.552907747
ILMN_1722502	CCT6A	-0.552928547
ILMN_1716342	CCDC132	-0.553106491
ILMN_1665824	C6orf218	-0.553121024
ILMN_2396697	GRIK2	-0.553153603
ILMN_2075476	GIPC2	-0.553228932
ILMN_1907812	BX119471	-0.553257103
ILMN_2353027	GRAP	-0.553281124
ILMN_1738773	HRC	-0.553362421
ILMN_2083818	EXOC8	-0.553428905
ILMN_3244025	DUX2	-0.553470227
ILMN_1704321	IL2	-0.553493902
ILMN_1832876	AW968764	-0.55356542
ILMN_1780618	TMEM192	-0.553627528
ILMN_1683886	BX484633	-0.553633171
ILMN_1879078	AA844688	-0.553650518
ILMN_1805695	C6orf170	-0.553668243
ILMN_1667101	PRM3	-0.553709556
ILMN_1736218	CHRNB3	-0.553751171
ILMN_1907451	BC038752	-0.553774806
ILMN_1716734	CNOT7	-0.553780994
ILMN_1769934	GRHL1	-0.553802634
ILMN_2113074	UFM1	-0.553879724
ILMN_1790253	TRIM74	-0.55399708
ILMN_1705662	LRRC37B	-0.554126172
ILMN_1713752	SERINC3	-0.554149821
ILMN_1716895	RPA3	-0.554166208
ILMN_3267612	SDHC	-0.554222173
ILMN_1784333	SECISBP2L	-0.554340557
ILMN_1706583	DLAT	-0.554375017
ILMN_1848529	AY235716	-0.554379014
ILMN_1774742	MTTP	-0.554399271
ILMN_1690589	MLC1	-0.554527216
ILMN_1840508	AI084979	-0.554561594
ILMN_1683687	AK024951	-0.55456461
ILMN_1701966	LOC286467	-0.554638599
ILMN_1889404	COL29A1	-0.554642771
ILMN_1908135	AA680058	-0.554783582
ILMN_3237986	MMGT1	-0.554812119
ILMN_1666140	PRY2	-0.554842401
ILMN_1843198	AK4	-0.554878012
ILMN_1809077	RFPL4B	-0.554946708
ILMN_3178252	RPS29	-0.554972166
ILMN_1680072	ZNF32	-0.554973842
ILMN_1849941	AB074280	-0.55501266
ILMN_1683162	COL8A1	-0.55501472
ILMN_1780036	WDR1	-0.555051813
ILMN_1890877	AA789270	-0.555078395
ILMN_1662038	LARGE	-0.555096311
ILMN_1729498	TMEFF1	-0.555125894
ILMN_2048683	FSCB	-0.555359735
ILMN_1674697	TCRA	-0.555409214
ILMN_1751375	ENAM	-0.555486759
ILMN_1800103	NA	-0.555595386
ILMN_1661596	FLVCR1	-0.555746225
ILMN_1843932	NS4ATP2	-0.555856754
ILMN_1675421	ISCA1	-0.555899222
ILMN_1761000	ACER2	-0.555929475
ILMN_2258363	KLC4	-0.556112551
ILMN_1663723	SLC13A4	-0.55619581

ILMN_1719695	NFKBIZ	0.522447017
ILMN_3242091	NCRNA00094	0.522399731
ILMN_1783156	MAP2K3	0.522374494
ILMN_3192156	LOC100130932	0.522354084
ILMN_1750511	NT5C3L	0.522292231
ILMN_1704290	SPTLC2	0.522233392
ILMN_1763688	RNASEK-C17ORF4	0.522178606
ILMN_1778304	ANKHD1	0.522173971
ILMN_2047599	TMEM50B	0.522165784
ILMN_2127098	HSP90AB4P	0.522071778
ILMN_1672176	SMPD2	0.522038044
ILMN_2410864	RAB28	0.522016529
ILMN_2228453	KIAA0562	0.521993089
ILMN_1779492	SMURF1	0.521941258
ILMN_1657421	YWHAE	0.521940665
ILMN_1784661	TMEM2	0.521940543
ILMN_1798874	TMEM85	0.521927533
ILMN_1753547	STAT5A	0.521805762
ILMN_1813911	HEXA	0.521704106
ILMN_1777794	PRKCSH	0.521700002
ILMN_1702534	CD244	0.521697221
ILMN_2399622	AP1G1	0.521653664
ILMN_3199780	CAP1	0.521571789
ILMN_2124757	DKFZp547I014	0.521275019
ILMN_1708098	LIX1L	0.521219837
ILMN_1809488	SPCS2	0.521192027
ILMN_2294442	SNRPN	0.521159539
ILMN_1815086	NINJ1	0.521155242
ILMN_1744929	PTGER1	0.521150724
ILMN_1681239	MYO1F	0.521145106
ILMN_1684887	SAMSN1	0.521063874
ILMN_2229940	PRADC1	0.520938859
ILMN_1709333	OAS2	0.5208269
ILMN_1776077	D26121	0.520721625
ILMN_2104356	COL1A2	0.520673287
ILMN_2396020	DUSP6	0.52063033
ILMN_1694711	MAD2L1BP	0.520506307
ILMN_1766991	ABRA	0.520499192
ILMN_2374164	HERPUD1	0.520382077
ILMN_1797375	KLF12	0.520193813
ILMN_1773751	HRAS	0.520128879
ILMN_1737918	C1QA	0.519820883
ILMN_1702305	NCAM1	0.519814642
ILMN_1794085	PPP6R1	0.519783808
ILMN_1810334	COMMD7	0.519738256
ILMN_3235096	SNORA28	0.51956644
ILMN_1672589	SEMA4B	0.519554333
ILMN_1735910	VMO1	0.519497377
ILMN_1715540	TRAIP	0.519479246
ILMN_2246134	PDE4DIP	0.518877902
ILMN_1807044	UBAC1	0.51886113
ILMN_1669555	FAM48A	0.518808566
ILMN_2071809	MGP	0.518723015
ILMN_2078995	CSRNP3	0.51868854
ILMN_1701413	PIGQ	0.518674161
ILMN_1652065	KCNMB1	0.518563882
ILMN_1653529	TEX10	0.518542176
ILMN_1700822	DPP3	0.518528549
ILMN_1680772	TCRGV	0.51843932
ILMN_2406084	ITGA11	0.51821645
ILMN_1681230	MRPL4	0.518147002
ILMN_1758356	OR2W5	0.517963481
ILMN_1705210	GRASP	0.517799056
ILMN_2276598	AP1G2	0.51777635
ILMN_1775498	FAM100B	0.517734365
ILMN_1772959	PDCD7	0.517590172
ILMN_1803825	CXCL12	0.517572806
ILMN_2331525	PRKD2	0.517489204
ILMN_2405680	CRB3	0.517454367
ILMN_2273796	ANKZF1	0.51741558
ILMN_1742547	NRP1	0.517400003
ILMN_2319913	DGKA	0.517274525
ILMN_1730879	CBY1	0.517211147
ILMN_1776522	SLC50A1	0.517183894
ILMN_1693045	TMED1	0.517018521
ILMN_3300972	SIVA1	0.516987218
ILMN_2347541	NIN	0.516840591
ILMN_1736847	MED8	0.516805208
ILMN_1694980	NAGLU	0.516791892
ILMN_3287157	RPL21	0.516672235
ILMN_1766171	SNF8	0.516647631
ILMN_1762725	EIF3L	0.516564962
ILMN_1674386	PITX1	0.51652334
ILMN_1651336	MLYCD	0.516329111
ILMN_2402168	EXOSC10	0.516239
ILMN_1750518	THOC4	0.516212261
ILMN_1763260	HIF1A	0.516140064
ILMN_1673566	ADAMTS1	0.516111893
ILMN_1679093	ZNF581	0.515722398
ILMN_1695271	RPP25	0.515602243
ILMN_1659047	HIST2H2AA4	0.515538729
ILMN_2180371	C12orf24	0.515470006
ILMN_1743331	ADIG	0.515459529
ILMN_1704195	FUK	0.515425945
ILMN_1795514	C3orf25	0.515382485
ILMN_1657129	SKAP2	0.51525037
ILMN_3300313	P4HTM	0.515209336
ILMN_1702055	ROPN1B	0.51520158

ILMN_1727813	BRP44	-0.556222907
ILMN_1882881	DB297507	-0.556287812
ILMN_1677868	ADRA2B	-0.556371215
ILMN_1707464	MST1	-0.556566912
ILMN_2358783	ASB3	-0.55658734
ILMN_1815570	HOXA6	-0.556688057
ILMN_2110561	IFNA8	-0.556689377
ILMN_1663422	RGL4	-0.556718644
ILMN_1702568	FAM125B	-0.556719324
ILMN_1799765	AK021761	-0.556786941
ILMN_1810400	IDH3B	-0.556788772
ILMN_1713174	TCP11L1	-0.556825414
ILMN_2158548	NACA2	-0.556987297
ILMN_1755423	PTCRA	-0.557042699
ILMN_3277501	LOC283028	-0.557049529
ILMN_1775569	CRISP2	-0.557095236
ILMN_2106902	FOXN3	-0.557109097
ILMN_2099773	SEMA3D	-0.55732925
ILMN_3245006	C12orf68	-0.557379931
ILMN_3292690	NA	-0.55738181
ILMN_2409290	CCPG1	-0.557402047
ILMN_1779673	HDGFL1	-0.557457413
ILMN_1682251	TLR8	-0.557487187
ILMN_1805526	MYL1	-0.557622537
ILMN_1732336	RFC2	-0.557630509
ILMN_3248500	LOC643955	-0.557635822
ILMN_1669846	SLC25A31	-0.55766948
ILMN_1711772	FLJ30679	-0.557719206
ILMN_1903466	AW592968	-0.557725606
ILMN_2381064	TPD52	-0.557773379
ILMN_3247844	C4orf10	-0.557827885
ILMN_1811121	FAM199X	-0.558079674
ILMN_1703973	FLJ00312	-0.558111862
ILMN_1877912	BX102609	-0.558122539
ILMN_1716862	PPM1B	-0.558197485
ILMN_1831111	NA	-0.558374576
ILMN_1775742	RNF128	-0.55852973
ILMN_3239073	AY343892	-0.558575419
ILMN_1769758	FANCA	-0.558646147
ILMN_1751393	ZNF684	-0.558665519
ILMN_1740052	SPEG	-0.558691624
ILMN_1847645	AI200787	-0.558698163
ILMN_3251540	GSTA2	-0.558758187
ILMN_3235584	TRIM66	-0.558909394
ILMN_1899696	BM661908	-0.558961156
ILMN_1747598	PPP1R11	-0.559014784
ILMN_3188132	AK097799	-0.559043209
ILMN_2341645	HNRNPH3	-0.559102296
ILMN_3285206	LOC154860	-0.559155893
ILMN_1754656	TCP10L	-0.559209209
ILMN_1912710	AK294518	-0.559228284
ILMN_1707065	SALL3	-0.559399763
ILMN_2179397	TATDN1	-0.55954104
ILMN_1780758	POLR1A	-0.559674213
ILMN_1782459	OSBPL8	-0.559732853
ILMN_1800898	ARG2	-0.559742405
ILMN_1804661	KALRN	-0.559813374
ILMN_1767535	LOC645676	-0.559905889
ILMN_1659285	PSMG1	-0.560009543
ILMN_1673755	AY927633	-0.560098711
ILMN_1769601	TBCK	-0.56012482
ILMN_2117885	FAM122A	-0.560175901
ILMN_1749081	AUTS2	-0.560176297
ILMN_1859334	BI517762	-0.560180476
ILMN_1814726	SCARB2	-0.56020409
ILMN_2371001	ATP6V1G3	-0.560310718
ILMN_1905413	BU674282	-0.560342623
ILMN_1741204	KLHDC2	-0.560470306
ILMN_1808217	PPP1R1C	-0.560616165
ILMN_2244004	ZMYM3	-0.56075725
ILMN_1725157	NEK6	-0.560798835
ILMN_1915913	BG722090	-0.560851175
ILMN_1724753	NIN	-0.560993095
ILMN_3235888	PABPC1L2A	-0.561084344
ILMN_1782501	XAGE1D	-0.561117747
ILMN_1807339	HRG	-0.561232222
ILMN_1738607	ZNF525	-0.561299776
ILMN_1670542	AK2	-0.56132496
ILMN_1737842	AMAC1L3	-0.561356864
ILMN_3273340	hCG_2014417	-0.561429431
ILMN_1724266	LYPD2	-0.561630927
ILMN_1761804	ALDH9A1	-0.561710471
ILMN_3235196	LOC100129716	-0.561779222
ILMN_1748831	PPP1R13B	-0.561784138
ILMN_3240595	SNORD37	-0.561785442
ILMN_1746552	WDR17	-0.561786594
ILMN_3308195	MIR618	-0.561845155
ILMN_2206224	OR4M2	-0.561858639
ILMN_1753931	CDO1	-0.561900197
ILMN_2172600	ZNF345	-0.561905138
ILMN_1797030	FGF18	-0.561917745
ILMN_1839905	AA704464	-0.561941853
ILMN_1774410	HSPD1	-0.561953934
ILMN_1729060	ARHGEF5	-0.562035803
ILMN_1743829	ATXN2	-0.5620783
ILMN_1680054	LAMB3	-0.562139605
ILMN_2350240	GTF2I	-0.562146786
ILMN_3237846	GAGE12I	-0.562164723

ILMN_1654318	PTPN23	0.515187622
ILMN_1671725	REPIN1	0.515180488
ILMN_1786242	RPL14	0.515117611
ILMN_2206141	BAGE	0.515113462
ILMN_2403896	GGTLC2	0.515007985
ILMN_1798288	MOBKL2C	0.514996638
ILMN_1691341	IL7R	0.514991909
ILMN_3236259	PPIAL4A	0.5148487
ILMN_1683016	PLA2R1	0.514706022
ILMN_1707277	CCDC144B	0.514663813
ILMN_1680208	RPL13A	0.514557584
ILMN_2167617	NACA	0.51452165
ILMN_2412384	CCNE2	0.51449106
ILMN_1766182	GNAZ	0.514469141
ILMN_1746492	IFT27	0.514300817
ILMN_3201480	RPS27A	0.514238567
ILMN_1809813	PGF	0.514146835
ILMN_2043816	ARPC5L	0.514134012
ILMN_1755224	TMEM102	0.513871217
ILMN_1698726	SLC25A27	0.513761258
ILMN_1679640	FXR1	0.5137525
ILMN_1749006	RCSD1	0.513694652
ILMN_2134224	ATP13A1	0.513677438
ILMN_1673960	MAT2B	0.513622122
ILMN_1722218	MBOAT7	0.513619405
ILMN_2373106	DMD	0.513547525
ILMN_1720542	POLR2I	0.513539834
ILMN_2375651	SCNM1	0.513397569
ILMN_1764754	RAMP1	0.513390142
ILMN_2310075	TRMT2A	0.513358553
ILMN_1669409	VSIG4	0.513316669
ILMN_1684217	AURKB	0.513259558
ILMN_1788024	PCID2	0.51317808
ILMN_2196550	C13orf18	0.513066132
ILMN_1681984	GALNT10	0.513048289
ILMN_1656540	RUVBL1	0.51303256
ILMN_1666594	IRF8	0.512999316
ILMN_1671478	CKB	0.512932129
ILMN_2331087	MS4A7	0.512883366
ILMN_1663787	KLK3	0.512871084
ILMN_2342068	ELKS	0.51279549
ILMN_1815169	MCM5	0.512784901
ILMN_1727036	ENAH	0.512607531
ILMN_2364110	GBA	0.512595309
ILMN_1809963	RSAD1	0.512484373
ILMN_1667453	HINFP	0.512470356
ILMN_2135258	RAPGEF3	0.512335503
ILMN_1662949	FAM132A	0.512293164
ILMN_2285802	SEC14L1	0.512216184
ILMN_1800897	NRD1	0.512063144
ILMN_1712184	HIST1H3C	0.511939633
ILMN_1676091	HNRNPA1	0.51187527
ILMN_1766955	VCAM1	0.511860488
ILMN_2122374	FAM49B	0.511851275
ILMN_3278995	RBM39	0.511846671
ILMN_2130409	PEX26	0.511700246
ILMN_1681101	MARCH1	0.511686062
ILMN_1651828	CCT3	0.511652173
ILMN_1707627	TPI1	0.511589391
ILMN_2188451	HIST1H2AH	0.511554701
ILMN_2366463	FN1	0.511537963
ILMN_1791483	PDE4D	0.511465917
ILMN_1665832	ID1	0.511384581
ILMN_1844408	PLXNA2	0.511376894
ILMN_1796085	PPM1B	0.511344389
ILMN_1701455	FBXO6	0.511299633
ILMN_2195462	C1QTNF4	0.511298456
ILMN_2387553	PSMA3	0.511294053
ILMN_1714216	TSC2	0.511160984
ILMN_2343618	SAMD3	0.511113817
ILMN_1774813	GPR173	0.5110492
ILMN_1740903	C7orf49	0.510999889
ILMN_1738424	CDC42	0.510917045
ILMN_1712636	NVL	0.510900659
ILMN_1747824	ZNF85	0.510716995
ILMN_2388070	TMEM44	0.510682026
ILMN_1670272	LRP10	0.510642807
ILMN_2082314	TOM1	0.510474377
ILMN_1713450	MYL6B	0.510430533
ILMN_2403965	TXNDC5	0.510329825
ILMN_1809730	RXRG	0.51032854
ILMN_2352380	TRPM2	0.510267787
ILMN_1775566	ATP1A1	0.510254368
ILMN_3249618	NOP10	0.510162063
ILMN_1726064	PAK1IP1	0.510027814
ILMN_1775073	ZNF273	0.509974732
ILMN_1733930	PREB	0.509962537
ILMN_1716480	ACD	0.50995037
ILMN_2294978	ANG	0.509856649
ILMN_1661733	FOLR1	0.509821831
ILMN_1722537	FLJ40504	0.509698205
ILMN_1784884	LILRB3	0.509625418
ILMN_1690268	HNRNPUL1	0.50961352
ILMN_1669788	NUDT14	0.509565282
ILMN_1668582	CRBN	0.50948951
ILMN_1661599	DDIT4	0.509469343
ILMN_1781565	MESDC1	0.509298115
ILMN_1739241	CHAC1	0.509212881

ILMN_3246556	LOC100133308	-0.562182677
ILMN_1816157	BG911604	-0.56218268
ILMN_2225735	CRBN	-0.562273022
ILMN_1743853	AK128837	-0.562311018
ILMN_1678186	ATP6V0A4	-0.562442534
ILMN_1724040	ANKRD57	-0.562481622
ILMN_3237812	REXO1L1	-0.562506093
ILMN_1686992	TEDDM1	-0.56255987
ILMN_3238358	SCARNA22	-0.56256308
ILMN_1772957	FOXRED2	-0.562570346
ILMN_2413732	ADAMTS13	-0.562584955
ILMN_2299072	LUC7L3	-0.562658483
ILMN_1718671	GJB6	-0.562723291
ILMN_2269193	ZNF331	-0.562762781
ILMN_3269377	FLJ00278	-0.562775518
ILMN_1793514	RPTOR	-0.562794898
ILMN_2048647	NBEA	-0.562863411
ILMN_1873592	NA	-0.562937959
ILMN_3195563	NA	-0.562969611
ILMN_1733654	CXorf40B	-0.563045719
ILMN_3256742	RPS29	-0.563051252
ILMN_1669259	ANKRD49	-0.56307675
ILMN_1762142	CDCA7	-0.563182098
ILMN_3297880	SEC13	-0.563250308
ILMN_1759319	SCAND2	-0.563384142
ILMN_1739154	LSAMP	-0.563443034
ILMN_2122926	VN1R2	-0.563536078
ILMN_1651453	LOC643596	-0.563563737
ILMN_1794011	CHST2	-0.563579377
ILMN_1724544	PPP4R1	-0.563586409
ILMN_1662801	ZMYM3	-0.563758273
ILMN_1704043	PRR21	-0.563783785
ILMN_3285346	LOC643387	-0.563815221
ILMN_1660166	FAM188B2	-0.564306449
ILMN_1790249	F8A1	-0.564381259
ILMN_3239288	ISM1	-0.564446345
ILMN_1842468	BX103636	-0.564455292
ILMN_1670145	DFNA5	-0.564518518
ILMN_2349982	FGF1	-0.564680881
ILMN_2115862	ESPNL	-0.564789836
ILMN_3241182	DKFZp686K04236	-0.564803122
ILMN_3273185	AK311113	-0.564852012
ILMN_2364305	FECH	-0.564905595
ILMN_1758215	ANKRD34C	-0.564980593
ILMN_2213680	ZCCHC10	-0.565019892
ILMN_1727049	SLC29A4	-0.565203721
ILMN_1684168	GSTZ1	-0.565218116
ILMN_1795419	CNPPD1	-0.565473461
ILMN_3259374	ST6GAL2	-0.565703159
ILMN_3200885	TNFRSF14	-0.565836171
ILMN_1702489	TRIM63	-0.565850949
ILMN_1693905	HAT1	-0.56602534
ILMN_1748694	NHSL2	-0.566165118
ILMN_1758189	OR4F17	-0.566175707
ILMN_1876266	GJA3	-0.566248782
ILMN_3248086	C11orf20	-0.566271429
ILMN_1773200	CP110	-0.566273667
ILMN_3287871	LOC100505502	-0.566284456
ILMN_3215268	ABCB10	-0.566424923
ILMN_2194229	TMEM128	-0.566445657
ILMN_1742813	TMEM167A	-0.566566509
ILMN_1877317	AW294700	-0.566619948
ILMN_1692056	HS3ST3A1	-0.566620203
ILMN_2171783	CPEB3	-0.56671523
ILMN_3289997	AATK	-0.566757675
ILMN_1795552	UNC13C	-0.566774398
ILMN_2290896	truncated AKR	-0.566783818
ILMN_2056932	SPEM1	-0.566856123
ILMN_1766115	PLEKHF2	-0.566903782
ILMN_1711650	KCNE1L	-0.566978869
ILMN_1681855	TBC1D25	-0.566984457
ILMN_1770260	NFKBIZ	-0.567037151
ILMN_1690807	MKL2	-0.567047652
ILMN_3294196	LOC400743	-0.567141072
ILMN_1685396	CLIC5	-0.567171906
ILMN_1776260	PPIAL4A	-0.567360874
ILMN_1779633	PSMD6	-0.567384356
ILMN_1813270	ELF5	-0.567469887
ILMN_3207933	ABCE1	-0.567545875
ILMN_1731688	KLB	-0.567547209
ILMN_1770911	NRSN2	-0.567606424
ILMN_1796836	AX747367	-0.567667436
ILMN_1784985	PRRT3	-0.567754825
ILMN_3307767	WNT9A	-0.567765103
ILMN_1675822	PCDHA@	-0.567769767
ILMN_1712298	ANKRD46	-0.567817533
ILMN_1671404	SVIL	-0.567890213
ILMN_1802223	ATP2B2	-0.567951258
ILMN_1728074	PHAX	-0.567954097
ILMN_3177354	LOC100128842	-0.568002674
ILMN_1675542	NUS1	-0.568107602
ILMN_1861916	CR992331	-0.56818175
ILMN_1691395	ZBTB11	-0.56818739
ILMN_1745988	NUDT12	-0.568221175
ILMN_1760174	MCCC1	-0.568288104
ILMN_2131103	PPP1R3B	-0.568413387
ILMN_3243084	AI241366	-0.568437821
ILMN_1804834	C6orf130	-0.568514712

ILMN_1687922	RP9	0.509210825
ILMN_2320906	RTN3	0.509092778
ILMN_2144791	GOLGA6A	0.509077285
ILMN_3236732	LOC100129637	0.509075464
ILMN_1694419	KRTAP8-1	0.508977909
ILMN_1777301	ARL13B	0.508971054
ILMN_1774602	FBLN2	0.508938007
ILMN_1691156	MT1A	0.508842263
ILMN_1653337	USP32	0.508832779
ILMN_1701930	EEF1B2	0.508817094
ILMN_2407954	XCR1	0.508761381
ILMN_2378708	TAF6	0.508712657
ILMN_1681160	CCT6P1	0.508607641
ILMN_3197549	TMEM106A	0.508563308
ILMN_1678143	ARHGDIB	0.508524063
ILMN_1662963	PSD2	0.508388305
ILMN_2335198	NCOA1	0.50837723
ILMN_2106167	RAP1GDS1	0.508333875
ILMN_3276859	LOC401127	0.508316162
ILMN_1664823	TAOK3	0.508254565
ILMN_2370535	GAS2L1	0.50824161
ILMN_2174437	CIDEC	0.50820328
ILMN_1779558	GAS6	0.508109139
ILMN_1799208	CSGALNACT2	0.508108201
ILMN_2154566	RPL10A	0.508022312
ILMN_2412281	PDLIM5	0.508011491
ILMN_2393169	THOC5	0.507974631
ILMN_2378868	SRSF5	0.507970845
ILMN_1701581	LAD1	0.507957793
ILMN_1656837	RBP1	0.507921075
ILMN_1772148	SULT1C4	0.507876533
ILMN_1815039	RRP36	0.50777072
ILMN_2148785	GBP1	0.507715969
ILMN_1652085	MPHOSPH10	0.507569166
ILMN_1679268	PELI1	0.50728964
ILMN_1720430	PPDPF	0.507183442
ILMN_1806023	JUN	0.507105499
ILMN_1760741	NDUFA9	0.507058528
ILMN_2368585	GGT1	0.507022566
ILMN_1684530	SNX18	0.50695708
ILMN_2406313	RBCK1	0.506937607
ILMN_1751598	SESN2	0.506861921
ILMN_3232529	LOC375133	0.506807656
ILMN_3266471	SNRPC	0.506759333
ILMN_1687738	RPL7	0.506613586
ILMN_1794386	IL2RG	0.506517299
ILMN_1724250	GRN	0.506471886
ILMN_1700477	MRPL43	0.506469176
ILMN_1658685	ELFN2	0.50642075
ILMN_1805623	SRCIN1	0.506070414
ILMN_1801927	FIGLA	0.506005141
ILMN_1760628	C9orf86	0.505973952
ILMN_1669878	GUSB	0.505885246
ILMN_1678619	DAK	0.5058648
ILMN_1782560	CD86	0.505847378
ILMN_1769383	GIMAP5	0.505846266
ILMN_2057566	PGP	0.505782838
ILMN_3297141	BC047484	0.505772202
ILMN_1663158	ZNF174	0.505727086
ILMN_1794976	NUDT4	0.505653381
ILMN_1715625	C20orf94	0.505631845
ILMN_1734794	AKAP4	0.505551278
ILMN_2273331	CCDC46	0.505517289
ILMN_1653200	SLC22A17	0.505440908
ILMN_1803838	CNFN	0.505359421
ILMN_2306077	USP33	0.505340647
ILMN_1761181	NOL9	0.505326343
ILMN_1668559	RGS10	0.505228411
ILMN_1699348	MS4A4E	0.505211366
ILMN_2181191	TPI1	0.505174358
ILMN_1738718	CYB561D2	0.505115735
ILMN_2094061	IMPA2	0.505030249
ILMN_2223941	FBLN5	0.504909468
ILMN_3242738	ZFP62	0.504766036
ILMN_1742782	GPANK1	0.504681949
ILMN_1721022	SHC1	0.504568447
ILMN_1759084	INTS8	0.504427434
ILMN_1746252	POLD2	0.504351296
ILMN_1769259	ANO9	0.504240042
ILMN_1721024	SRFBP1	0.504205338
ILMN_1799099	TEKT3	0.50417676
ILMN_1756093	TCEAL4	0.504173606
ILMN_2251961	PCDHGC3	0.504151971
ILMN_1708890	RASSF4	0.504013832
ILMN_1781468	SMAP2	0.503938247
ILMN_1683036	BRWD1-IT2	0.503873495
ILMN_1911059	AV691356	0.503856519
ILMN_1810584	IL1R1	0.503846364
ILMN_1761772	NUP155	0.503788957
ILMN_1812787	HERC2	0.503778804
ILMN_1792489	ARPC2	0.503765551
ILMN_1665761	BCL11B	0.503722886
ILMN_1713686	IL34	0.503677424
ILMN_1746763	ECM2	0.503641187
ILMN_1751571	RAD23A	0.503630739
ILMN_1713249	PHF19	0.50362596
ILMN_2092516	GRP94c	0.503515795
ILMN_1719403	MTX1	0.503440523

ILMN_1651228	RPS28	-0.568515995
ILMN_1778788	AMOTL2	-0.568522012
ILMN_3249566	LOC100130264	-0.568573317
ILMN_1724658	BNIP3	-0.568707883
ILMN_1668353	C22orf45	-0.568754043
ILMN_2076602	ITM2A	-0.568757698
ILMN_1765620	VSTM2A	-0.56879551
ILMN_1807556	VWA3A	-0.568852002
ILMN_2072541	RAB11FIP2	-0.5688717
ILMN_1672947	CAST	-0.568871889
ILMN_1859942	BG201242	-0.568881633
ILMN_1880864	BX102511	-0.56899028
ILMN_1661399	ARL4A	-0.569025019
ILMN_1699082	MAPKAPK5	-0.569041689
ILMN_3245346	EFR3B	-0.56913921
ILMN_1789505	ITPR1	-0.569281204
ILMN_3250322	DPPA3	-0.569337404
ILMN_3241282	TDRD12	-0.56937105
ILMN_1753885	YTHDF1	-0.56945784
ILMN_1715125	HAAO	-0.569514353
ILMN_1682126	LOC100133920	-0.569551598
ILMN_1788625	EXOC5	-0.569671807
ILMN_2276000	CPNE1	-0.56967776
ILMN_1691104	PGAM4	-0.56972259
ILMN_2168747	GSTA2	-0.56977431
ILMN_1770927	KAZN	-0.569867463
ILMN_1756312	SEMA7A	-0.569961164
ILMN_1869087	AK096319	-0.570022137
ILMN_3262909	C6orf176	-0.570056697
ILMN_3239298	OTUD7B	-0.570086742
ILMN_1662617	PPP2R3C	-0.570117165
ILMN_1753773	ANAPC11	-0.570203923
ILMN_1653612	KCTD9	-0.570221895
ILMN_1808220	LCE3E	-0.570231051
ILMN_1705144	ULK1	-0.570332017
ILMN_1716309	EGFL6	-0.570365554
ILMN_3245869	C3orf78	-0.570424614
ILMN_1692433	RECQL5	-0.570435335
ILMN_1770853	CHST5	-0.570625709
ILMN_2182348	SMC3	-0.570683623
ILMN_3249676	BOLA2B	-0.570798213
ILMN_1906579	AI274046	-0.570929393
ILMN_1750722	RPS7	-0.570940252
ILMN_1660636	WWOX	-0.571067846
ILMN_1804051	SNX8	-0.571172012
ILMN_3292042	LOC284688	-0.571213886
ILMN_1665982	AKTIP	-0.571288545
ILMN_2398926	C17orf58	-0.571313927
ILMN_1750508	STMN1	-0.571422366
ILMN_1673322	UNQ9368	-0.571432504
ILMN_1800447	PHKB	-0.571493293
ILMN_1656846	NA	-0.571540266
ILMN_1755222	C9orf82	-0.571580868
ILMN_2276461	MAP2	-0.571592627
ILMN_2400326	DYRK3	-0.571832382
ILMN_1714176	C13orf29	-0.571845428
ILMN_1660698	GTPBP8	-0.571917067
ILMN_1666310	BSX	-0.571980472
ILMN_2289825	ARPP21	-0.572070359
ILMN_1787919	PARVB	-0.572082675
ILMN_1779470	ABCG4	-0.572110928
ILMN_1684321	CYB5B	-0.572237305
ILMN_1685779	FOXR1	-0.572334122
ILMN_2153061	FGF20	-0.572344783
ILMN_1785345	GPR84	-0.57255701
ILMN_2306066	FUS	-0.57272082
ILMN_1677866	FOLH1B	-0.572734415
ILMN_2388177	SPATA7	-0.572829704
ILMN_1765532	RDBP	-0.572868007
ILMN_1689097	NAA20	-0.572886324
ILMN_1814296	TRPM6	-0.572900335
ILMN_3236052	SNORD19B	-0.573051861
ILMN_1801608	KCNN3	-0.573109277
ILMN_1815707	CALML4	-0.573268478
ILMN_2225577	POC5	-0.573417704
ILMN_1802669	PPP3CB	-0.573436814
ILMN_2103761	TLE4	-0.573440651
ILMN_1906948	AA884386	-0.573521229
ILMN_1787705	ATP6V1B2	-0.573628745
ILMN_1768050	SCOC	-0.573652756
ILMN_2407308	HEY1	-0.573664076
ILMN_1790645	HECW1	-0.573751139
ILMN_1670752	KIAA0907	-0.573767314
ILMN_1888252	AI984350	-0.57385942
ILMN_1712836	C6orf27	-0.573898768
ILMN_1745368	TMEM50A	-0.57391777
ILMN_1719599	SYTL4	-0.574031032
ILMN_1658917	SLC1A1	-0.574035296
ILMN_2350970	SOCS5	-0.5740635
ILMN_3305768	GSTA7P	-0.574065199
ILMN_1740010	PCNX	-0.574126328
ILMN_1694630	AK055082	-0.574227201
ILMN_1878795	AW297854	-0.574359263
ILMN_1741970	JUP	-0.574370326
ILMN_1784556	H2AFB2	-0.574371374
ILMN_1708627	SRP9	-0.574467633
ILMN_1727605	FRK	-0.57455547
ILMN_3187357	AKTIP	-0.574611028

ILMN_1690139	KIAA0748	0.503240447
ILMN_1736752	COMTD1	0.503133222
ILMN_3188655	LOC100128239	0.503108163
ILMN_1795826	ATP6V0D1	0.502952492
ILMN_1698395	GNB5	0.502910287
ILMN_1733841	TCF7L1	0.502898328
ILMN_1773741	GOLGA5	0.502767864
ILMN_1695025	CD2	0.50275335
ILMN_1688637	TMEM198	0.502681543
ILMN_1657591	SCN3A	0.502640147
ILMN_2338733	ART5	0.502575532
ILMN_3307796	IKZF5	0.502469435
ILMN_1869442	CR743578	0.502467707
ILMN_1742260	SSFA2	0.502366255
ILMN_1752914	ARHGAP31	0.502250709
ILMN_3246328	PTH1R	0.50221784
ILMN_2128770	CDR2L	0.502086768
ILMN_1779324	GZMA	0.502071448
ILMN_1657373	LEPREL1	0.501885374
ILMN_1813573	SETD1B	0.50187855
ILMN_1667295	VASN	0.501805333
ILMN_1692260	MAFG	0.50177965
ILMN_2317730	ELMO2	0.501684108
ILMN_1699574	NRP1	0.50167323
ILMN_1652472	BRAF	0.501654005
ILMN_1687888	BTN3A1	0.50154188
ILMN_1779373	HIST1H2BF	0.501522357
ILMN_1832656	AW578902	0.501320288
ILMN_1672975	NA	0.501293498
ILMN_1786215	ZNF3	0.501292576
ILMN_1728714	SSSCA1	0.501214556
ILMN_1807609	SPTBN5	0.501082229
ILMN_1713031	PGCP	0.501050116
ILMN_1765923	C17orf101	0.501024521
ILMN_3221790	RPL7L1	0.501013992
ILMN_1711361	ZNF319	0.500940835
ILMN_1757343	PABPC4	0.50089152
ILMN_1725311	GCGR	0.500863863
ILMN_1668465	KIAA1609	0.500773885
ILMN_1758418	TNFSF13B	0.500716068
ILMN_1661886	APEX1	0.50056672
ILMN_1663816	CCDC148	0.50051007
ILMN_2406299	SEMA3B	0.500440212
ILMN_1708787	ZNF627	0.500409489
ILMN_1694323	GLE1	0.500366885
ILMN_2059505	ARPP19	0.50030415
ILMN_1805410	C15orf48	0.500292957
ILMN_1654566	HSPA1L	0.500226651
ILMN_1788250	LDOC1	0.500126154
ILMN_1675984	ARHGEF7	0.500122292
ILMN_3244611	MXRA7	0.499959206
ILMN_1672496	DNAJA1	0.499918471
ILMN_1669310	RHOT2	0.499796796
ILMN_1699520	SLC8A1	0.499701513
ILMN_2388142	CD99L2	0.499566769
ILMN_2390427	FLT4	0.499544211
ILMN_1723004	CD72	0.49952989
ILMN_1781824	FASLG	0.499494548
ILMN_1776021	EIF4H	0.499431478
ILMN_3230286	BCLAF1	0.499425012
ILMN_3244960	NACC1	0.499419937
ILMN_2358560	TIAM2	0.499323276
ILMN_1729980	RNF216	0.499318788
ILMN_1808196	GSTO1	0.49922463
ILMN_1738530	ATAD3A	0.499039779
ILMN_1665696	EFNA4	0.499011758
ILMN_1809751	EIF2C3	0.499011608
ILMN_1662263	MDP1	0.498886432
ILMN_1676924	CD247	0.498844942
ILMN_1722718	BMP2	0.498835671
ILMN_1784178	IFT172	0.498821246
ILMN_1662232	DCTN2	0.498815683
ILMN_2277419	SLC38A10	0.498813353
ILMN_2375319	RASGRP2	0.49879211
ILMN_1665203	SUMO4	0.498755545
ILMN_2318733	BNIP1	0.498681509
ILMN_1678087	MAP3K4	0.498643115
ILMN_1703041	IDUA	0.498572977
ILMN_1658469	P53TG1	0.498550688
ILMN_1703132	LYRM2	0.498517305
ILMN_1785988	CSNK1A1	0.498474825
ILMN_1761309	ADCK5	0.498307104
ILMN_2319766	RUFY2	0.49830362
ILMN_1740197	ZNF234	0.498293974
ILMN_1787360	GUCA1C	0.498240163
ILMN_1763147	NDUFB6	0.498063493
ILMN_1711823	C17orf70	0.498050586
ILMN_2381841	DDX19B	0.498025514
ILMN_1762281	DCTN3	0.498022824
ILMN_1815287	B3GALNT1	0.497960952
ILMN_2320349	BACE1	0.497920736
ILMN_1789001	SLC35B2	0.497574476
ILMN_2316236	HOPX	0.497570437
ILMN_1742544	MEF2C	0.497490332
ILMN_2371055	EFNA1	0.49747535
ILMN_1795383	RPUSD3	0.497455604
ILMN_1697377	ANKRD36B	0.497431751
ILMN_1749177	CCDC48	0.497401721

ILMN_1740855	TCP10L2	-0.574685242
ILMN_1710170	PPAP2C	-0.574695563
ILMN_1722609	PM20D1	-0.574741293
ILMN_2398474	RAP1B	-0.574816701
ILMN_1710128	FAM66B	-0.574872085
ILMN_3200465	LOC100132724	-0.574940853
ILMN_2341626	MARCH8	-0.574995336
ILMN_1836936	BM684558	-0.575061049
ILMN_2194649	TADA1	-0.575144481
ILMN_1740155	COL9A3	-0.575177835
ILMN_1912737	HIPK2	-0.575275402
ILMN_3232128	FLJ37786	-0.575343166
ILMN_1703459	WNT9B	-0.575453489
ILMN_1664592	C1orf95	-0.575511516
ILMN_1775442	SPDYE7P	-0.575542251
ILMN_2326324	LDHC	-0.575693103
ILMN_3193796	LOC100132024	-0.575697308
ILMN_1859393	AI203701	-0.575712126
ILMN_1739275	HTR3C	-0.575716752
ILMN_1766270	OR51M1	-0.575736077
ILMN_1683555	LOC100505679	-0.575740505
ILMN_1812970	RWDD1	-0.575836542
ILMN_2183891	PEX5L	-0.575839481
ILMN_1846543	BF512941	-0.575842211
ILMN_1800951	ATXN1	-0.575875973
ILMN_1658111	MGC34800	-0.575943291
ILMN_2401730	C1GALT1C1	-0.575952777
ILMN_1695355	FOXP2	-0.576172423
ILMN_1727361	LEMD3	-0.576363675
ILMN_3255124	ATL1	-0.57638784
ILMN_1806330	C17orf78	-0.576554627
ILMN_2209163	CHD6	-0.576558534
ILMN_1773337	DKK1	-0.576584985
ILMN_1740450	DLGAP1	-0.576615856
ILMN_1669931	TM9SF3	-0.576666187
ILMN_2211672	DISC1	-0.576674859
ILMN_1887357	AK095715	-0.576676598
ILMN_2276397	RASGRP2	-0.57670015
ILMN_1810942	CYP3A5	-0.576767379
ILMN_1699778	NKIRAS2	-0.576771239
ILMN_1705261	CAPN1	-0.576864816
ILMN_2232712	MYO10	-0.576930623
ILMN_1771800	PRKCA	-0.576942753
ILMN_1775138	PEG3	-0.576981937
ILMN_3245464	NCRMS	-0.577028118
ILMN_3248207	NA	-0.577094681
ILMN_1784176	ABCA5	-0.577236831
ILMN_2117902	PFAAP1	-0.57731466
ILMN_3290715	COL6A4P2	-0.577331196
ILMN_1703330	FEM1C	-0.577495947
ILMN_1667030	HSBP1	-0.577544869
ILMN_2189371	NTNG1	-0.577555251
ILMN_2292646	GAD1	-0.577648872
ILMN_1750800	ACO1	-0.577656143
ILMN_1653092	FAM186A	-0.577704845
ILMN_2127624	UBE2CBP	-0.577779622
ILMN_2061446	NCEH1	-0.577808866
ILMN_1727205	ARHGEF10L	-0.577815437
ILMN_1714567	AHNAK	-0.57796211
ILMN_2394571	FBXW11	-0.578272069
ILMN_2193498	TRMT1L	-0.578451215
ILMN_1754864	SLC25A18	-0.578495179
ILMN_1675721	UBE2R2	-0.578573819
ILMN_1696622	SLC38A6	-0.578580701
ILMN_1778464	TRAPPC10	-0.578609288
ILMN_1653347	BC012881	-0.57866475
ILMN_1756125	H1FNT	-0.578787229
ILMN_3259829	UBXN2B	-0.578844849
ILMN_1685168	RFX4	-0.578867478
ILMN_3236801	NA	-0.578868323
ILMN_3247438	C19orf69	-0.578945018
ILMN_1758907	UGT1A1	-0.579036987
ILMN_1685572	MAGEB6	-0.579043925
ILMN_1678546	PEX11B	-0.579050377
ILMN_1724372	SIRT3	-0.579111646
ILMN_1782110	ZNF295	-0.579486367
ILMN_1868037	CA441597	-0.579533156
ILMN_1779370	ARHGEF9	-0.579593732
ILMN_1706784	H2AFV	-0.579719472
ILMN_1681886	ADAMTS5	-0.579740828
ILMN_2292592	PCSK6	-0.579809584
ILMN_2332440	KCNMB2	-0.579890427
ILMN_2380566	SIAH1	-0.579952848
ILMN_1664776	EFR3A	-0.580063908
ILMN_1874814	AB231761	-0.580128669
ILMN_3245084	AK127966	-0.580237234
ILMN_1800874	MRO	-0.580271586
ILMN_2075829	LMBRD2	-0.580284656
ILMN_1783120	SLMAP	-0.580287365
ILMN_2079488	GNPDA2	-0.580440638
ILMN_1783889	PRKAA1	-0.58047544
ILMN_1844074	BX107348	-0.580570996
ILMN_1686951	TM6SF2	-0.580719141
ILMN_2372124	HNF4A	-0.580828089
ILMN_2323418	KRIT1	-0.580878381
ILMN_1735052	ULK1	-0.580881583
ILMN_1696568	ATP2C1	-0.580889817
ILMN_2279484	C2orf88	-0.580977701

ILMN_2397294	PLCB4	0.497384523
ILMN_1703989	ARFRP1	0.497306492
ILMN_1682775	EDN1	0.497161882
ILMN_1719344	NRBF2	0.497145693
ILMN_1713058	PSTPIP2	0.497000557
ILMN_2244009	LBH	0.49688655
ILMN_1760314	CCDC8	0.496822759
ILMN_1809883	CCDC134	0.496818877
ILMN_1696099	ALDH4A1	0.496744102
ILMN_1724959	SEC31A	0.496634773
ILMN_1712112	RCAN1	0.496583416
ILMN_2302947	CSF1	0.496397735
ILMN_1751559	TMEM204	0.496358751
ILMN_1662658	PUS1	0.496290191
ILMN_1711166	WRAP73	0.496227249
ILMN_1669667	PDLIM2	0.495865798
ILMN_1701243	C10orf2	0.495838357
ILMN_2112638	SVEP1	0.495681127
ILMN_1801105	PRKCD	0.495654909
ILMN_3234116	CCDC72	0.495548099
ILMN_1662681	AMBRA1	0.495392579
ILMN_1703005	EIF3F	0.49520632
ILMN_1684964	ZNF212	0.495190575
ILMN_2372136	P4HTM	0.495162542
ILMN_1701629	AK127589	0.495028628
ILMN_1680579	ATP2B4	0.494956304
ILMN_2191313	FAM21A	0.494936756
ILMN_3284053	LOC100335030	0.494920309
ILMN_2215656	MAGOH	0.494881124
ILMN_3226613	CLEC2D	0.494832251
ILMN_3249748	LDHA	0.494831196
ILMN_1815552	NTAN1	0.494824865
ILMN_1812968	SOX18	0.494824397
ILMN_2137084	LIN9	0.49481219
ILMN_1692247	TRIM7	0.494766765
ILMN_1796923	LOC81691	0.494675391
ILMN_1745385	HNRNPM	0.49465425
ILMN_1713496	ST3GAL5	0.49448718
ILMN_1659659	MAGEH1	0.494431533
ILMN_1788388	NOVA2	0.494394399
ILMN_1695413	ZNF429	0.493936721
ILMN_1667494	SPTB	0.493891273
ILMN_2174884	XPO7	0.493816745
ILMN_1781638	HDDC3	0.493792169
ILMN_1676868	RAB37	0.493736683
ILMN_2068498	POU5F1B	0.493706358
ILMN_1795341	SRSF1	0.49361245
ILMN_1740960	MACROD1	0.493420795
ILMN_3235148	FLJ43681	0.493367993
ILMN_1658821	SAMD1	0.493357878
ILMN_1706149	PDCD2L	0.493346579
ILMN_3241993	RPL32	0.493322434
ILMN_1703284	SPIRE2	0.493322023
ILMN_1743538	MLLT10	0.493161399
ILMN_1802392	RAD51L1	0.493148839
ILMN_2219712	HMGB2	0.492982177
ILMN_1694810	PANX2	0.492960805
ILMN_1755415	SAMD3	0.492955818
ILMN_1692684	SMARCD2	0.492897682
ILMN_1655422	RPL17	0.492663695
ILMN_2364700	ENSA	0.492648874
ILMN_2336037	EPN2	0.492263029
ILMN_1659777	RUFY1	0.492137593
ILMN_1722089	RNF217	0.49209769
ILMN_3240541	EML2	0.492096323
ILMN_1793559	C6orf204	0.492020021
ILMN_1790529	LUM	0.491939043
ILMN_1671263	CACNA1H	0.491766242
ILMN_1736828	CHST10	0.491697467
ILMN_1792710	DAPK3	0.491650777
ILMN_1763422	OR6C65	0.491642353
ILMN_1763386	BID	0.491636209
ILMN_3248324	MGC16384	0.491523545
ILMN_3213531	RPL21	0.491361179
ILMN_1713321	CYP20A1	0.491351087
ILMN_1763447	PLXNB2	0.491313153
ILMN_1745271	EXOSC4	0.491259352
ILMN_1680902	LOC284998	0.491240073
ILMN_1784110	CDK18	0.491208885
ILMN_1781916	AV723037	0.491157459
ILMN_2410421	NBPF1	0.491073657
ILMN_1680644	MIOS	0.491073054
ILMN_1687279	DHPS	0.490911445
ILMN_1872759	BX099468	0.490817151
ILMN_1756541	MXD4	0.490519324
ILMN_1744212	INPP5D	0.490500174
ILMN_1731299	PML	0.490415044
ILMN_1658531	RPL7	0.490366126
ILMN_2402392	COL8A1	0.490360785
ILMN_2126239	SMG5	0.490354889
ILMN_1655694	FBRSL1	0.490057029
ILMN_2211003	GAS2L3	0.490048185
ILMN_3224758	TUBB	0.489981408
ILMN_1685042	TENC1	0.489883813
ILMN_1709975	EDA	0.489815404
ILMN_1763887	SFI1	0.4898106
ILMN_2380605	MTMR3	0.489703551
ILMN_1674874	MFSD10	0.489693258

ILMN_1669410	CHGA	-0.581066882
ILMN_1873492	AU310952	-0.581087784
ILMN_1888492	AA083069	-0.581128033
ILMN_1722135	CNGA3	-0.581247751
ILMN_1766920	EP400NL	-0.581272575
ILMN_1688753	PTDSS1	-0.581334158
ILMN_1803745	SUOX	-0.581423552
ILMN_1794190	CCPG1	-0.581509275
ILMN_2216157	GNA12	-0.581536317
ILMN_2317348	APTX	-0.581570228
ILMN_1715214	PTPN7	-0.581591341
ILMN_1831566	AK129542	-0.581664659
ILMN_1772540	ATMIN	-0.581746656
ILMN_1677043	AKR7A2	-0.581772947
ILMN_1780887	USP21	-0.581941994
ILMN_1739155	OR9A4	-0.58207538
ILMN_1671542	NLGN4X	-0.582127991
ILMN_1685343	NUPL1	-0.582128556
ILMN_1792150	ALOX5	-0.582185411
ILMN_3270493	FAM75A3	-0.582195075
ILMN_1735996	NOX4	-0.582249289
ILMN_3296096	SLC9A7P1	-0.582332519
ILMN_1810738	YIPF5	-0.582474003
ILMN_1734542	OVGP1	-0.582495635
ILMN_1716517	KIAA0467	-0.582550027
ILMN_1676042	NA	-0.582681348
ILMN_2412172	APOBEC3F	-0.582815263
ILMN_1857017	SNX8	-0.582855519
ILMN_1735453	FAM98A	-0.583006311
ILMN_1766896	ZDHHC19	-0.583084403
ILMN_2366490	ZNF706	-0.583093261
ILMN_3236192	NA	-0.583099035
ILMN_1652521	MTMR9	-0.583325039
ILMN_2387799	PDPK1	-0.583360512
ILMN_1873411	DB350505	-0.583499573
ILMN_1655780	METTL10	-0.583559648
ILMN_1852349	AI434856	-0.583602406
ILMN_1786172	GTF2H2	-0.583658313
ILMN_3211047	BASP1P1	-0.583671614
ILMN_1779241	CRYM	-0.58372159
ILMN_1751424	LOC100131910	-0.583909521
ILMN_1715351	AIMP1	-0.584002475
ILMN_2411745	EML1	-0.584002963
ILMN_1749162	DIO2	-0.584033343
ILMN_1742044	GNAI1	-0.584131839
ILMN_1654129	AIDA	-0.584140961
ILMN_1711628	KRTAP1-3	-0.584161315
ILMN_1655509	ASAH1	-0.584172863
ILMN_2184966	ZHX2	-0.584348765
ILMN_1762972	CHD9	-0.584418456
ILMN_1794989	TMEM191B	-0.584474061
ILMN_1729965	CXorf66	-0.58447539
ILMN_1699357	SLC22A5	-0.584522437
ILMN_1654542	FAM172A	-0.584766971
ILMN_2223836	CHORDC1	-0.584972198
ILMN_1702124	LNX2	-0.585050669
ILMN_1781890	KIAA1486	-0.585130905
ILMN_2262362	ABCG1	-0.585150795
ILMN_3243788	TMEM146	-0.585226949
ILMN_1666325	ALG10B	-0.585245166
ILMN_1784818	OPN5	-0.585304882
ILMN_1675346	CCRL2	-0.585395111
ILMN_1709142	PURA	-0.585425954
ILMN_1891277	NA	-0.58545868
ILMN_2262749	SOCS5	-0.585543162
ILMN_3203815	KPNA4	-0.585592432
ILMN_3268232	DKFZp761K2322	-0.585597348
ILMN_2199439	CA2	-0.585640811
ILMN_2174729	ZNF318	-0.585668974
ILMN_2263312	FAM125B	-0.585773002
ILMN_3262439	FLJ46552	-0.585816081
ILMN_1715185	SPRYD5	-0.585950936
ILMN_1762615	FAM175B	-0.585952144
ILMN_3247341	NA	-0.585965915
ILMN_1908463	AA400272	-0.586009584
ILMN_1752559	FBRS	-0.58606086
ILMN_1797301	C1orf183	-0.58621027
ILMN_1824784	BX111027	-0.586328219
ILMN_3261022	NA	-0.586332795
ILMN_2152711	ACVR2A	-0.586467995
ILMN_1801605	BIRC6	-0.586484815
ILMN_1764120	IGF2AS	-0.586500411
ILMN_1718135	PRAMEF11	-0.586628969
ILMN_1786433	BCCIP	-0.586810196
ILMN_2086222	WDR11	-0.586864758
ILMN_1698512	AK096668	-0.586876261
ILMN_2206500	UGT2B4	-0.586946982
ILMN_1787595	B3GALNT1	-0.586961787
ILMN_3225432	LAMTOR3	-0.587002558
ILMN_3299929	CR625008	-0.587079159
ILMN_1810930	C6orf223	-0.587407904
ILMN_1815392	ACTRT1	-0.587531748
ILMN_1701991	SYNJ1	-0.58762013
ILMN_1704070	AK125723	-0.587840633
ILMN_2325998	ICK	-0.587882198
ILMN_1679728	WDR87	-0.587921023
ILMN_1674551	SMAD5	-0.587955068
ILMN_1673867	LIMD2	-0.587969385

ILMN_2319910	DGKA	0.48965396
ILMN_1717490	RPL6	0.489559915
ILMN_2075927	STK40	0.489547738
ILMN_2066124	AFG3L2	0.489399383
ILMN_1700231	IP6K1	0.489326216
ILMN_1741288	ZNF136	0.48929788
ILMN_1709348	ALDH1A1	0.489210941
ILMN_1722073	MLXIPL	0.489186917
ILMN_1793241	SRD5A1	0.489165456
ILMN_2358647	FBXO21	0.489119618
ILMN_3243909	SNORD102	0.488932251
ILMN_1732398	SCGB2A1	0.488917915
ILMN_1788160	KIF5B	0.488494718
ILMN_1729130	C7orf42	0.488467116
ILMN_2365248	HVCN1	0.488455215
ILMN_1765880	C16orf57	0.488362609
ILMN_2377900	MAP1B	0.488362486
ILMN_1676759	DDX27	0.488306383
ILMN_2349393	MDK	0.488186027
ILMN_1727078	C17orf37	0.487884039
ILMN_1715969	SLC25A37	0.487799214
ILMN_1810486	RAB34	0.487773338
ILMN_1753393	OSGEP	0.487730111
ILMN_3180989	DSTYK	0.487585875
ILMN_1735548	HIVEP1	0.487478875
ILMN_1667086	AP3M1	0.487301616
ILMN_1797813	SUZ12	0.487154061
ILMN_1812278	LY9	0.48711102
ILMN_1845201	N35251	0.487095401
ILMN_1757408	ZNF256	0.486992937
ILMN_2060413	CD24	0.486914908
ILMN_2331121	FCGR2B	0.486903071
ILMN_1666372	ATP5H	0.486880836
ILMN_1722025	CPEB4	0.486874006
ILMN_1652237	CBR3	0.486841257
ILMN_1745593	STMN1	0.486820475
ILMN_1807596	UBAP1	0.486787714
ILMN_1723358	SCARA3	0.486715746
ILMN_1797786	MAPK12	0.486691392
ILMN_1677756	UBE2D2	0.486602141
ILMN_1775753	FBXW2	0.486539752
ILMN_3247556	C8orf73	0.486524121
ILMN_1799642	TRIM24	0.486439826
ILMN_3236736	ZFP92	0.486411849
ILMN_1726981	VEGFB	0.486035024
ILMN_1717324	NR_024104	0.486025258
ILMN_1747683	AQP4	0.485859599
ILMN_1729374	ETFB	0.485847974
ILMN_1720442	NCBP2	0.485804813
ILMN_1778213	STK33	0.48579085
ILMN_1906187	LOC283070	0.485789023
ILMN_2327346	SSBP4	0.48575669
ILMN_2072140	BTF3L4	0.485729248
ILMN_3235932	BMS1	0.485669877
ILMN_1845086	AL110204	0.485496259
ILMN_1710078	TMEM181	0.485445646
ILMN_1759350	MED27	0.485378785
ILMN_1700935	HAS2	0.48525124
ILMN_1651415	DERL3	0.485177555
ILMN_1668683	MLL	0.48508848
ILMN_1756542	TBC1D17	0.485069711
ILMN_2399896	SEC31A	0.484750994
ILMN_2045800	C11orf65	0.48471713
ILMN_1674250	NCKAP1L	0.484617595
ILMN_1790533	PHACTR2	0.48449189
ILMN_1795454	CPSF6	0.484478695
ILMN_3236821	HSPB1	0.484336319
ILMN_1776154	COG3	0.48411194
ILMN_1779875	THY1	0.484087824
ILMN_1780291	NFAT5	0.483918901
ILMN_2294784	PRDM1	0.483811541
ILMN_1791144	PMS2L14	0.48373347
ILMN_3243982	SNAP47	0.48370369
ILMN_1728699	SPTY2D1	0.483591535
ILMN_3295235	LOC389493	0.483581402
ILMN_1737164	TM9SF1	0.483485269
ILMN_1669722	LRRC61	0.48333021
ILMN_1779639	IRAK1BP1	0.483264672
ILMN_2371251	MPG	0.483241584
ILMN_1696494	CMTM6	0.483151607
ILMN_1814650	TRAPPC4	0.483108562
ILMN_1681503	MCM2	0.483092373
ILMN_1665871	ANGEL1	0.483055539
ILMN_1791569	PLXNA1	0.482969284
ILMN_3289346	AX747493	0.482968105
ILMN_1801939	CCNB2	0.482962552
ILMN_2374383	TSPAN17	0.4828173
ILMN_1676605	ANKRD42	0.482672258
ILMN_1763326	C5orf25	0.482611271
ILMN_1685810	NFATC3	0.482599264
ILMN_1651610	SPRY1	0.482348217
ILMN_1694966	ASGR2	0.482107371
ILMN_1654851	FAM134A	0.482084688
ILMN_2410826	OAS1	0.48207147
ILMN_1677108	CAPN13	0.482033316
ILMN_2152402	ABHD16A	0.48195782
ILMN_1798808	FOXH1	0.48194349
ILMN_1662302	IL28A	0.481928957

ILMN_1799224	FANCA	-0.588012911
ILMN_1788530	ADPRHL1	-0.588164378
ILMN_1725707	ATG16L1	-0.588199718
ILMN_1687404	HCN1	-0.588209944
ILMN_1773002	C10orf140	-0.588270068
ILMN_1788156	EPM2A	-0.588406785
ILMN_1789676	AOX2	-0.58841275
ILMN_1662914	CTAGE5	-0.588477966
ILMN_1660787	SUCLA2	-0.588533044
ILMN_3223813	FOSL1	-0.588637509
ILMN_1791280	HSPB8	-0.588693904
ILMN_2277201	ATR	-0.588698346
ILMN_2353978	ACOT11	-0.588731591
ILMN_3301824	TSEN15	-0.588753846
ILMN_2057196	LOC100507170	-0.588756485
ILMN_1846537	AW081377	-0.588813021
ILMN_1733669	CDH18	-0.588844486
ILMN_2181064	GBA3	-0.588960793
ILMN_2414302	FDXR	-0.589006994
ILMN_3233229	SNHG7	-0.589143187
ILMN_1698311	ABHD12B	-0.58943117
ILMN_1843648	AW873324	-0.589437869
ILMN_1746457	GTF3C2	-0.589462187
ILMN_2195390	NA	-0.589537788
ILMN_1767103	C11orf63	-0.589587311
ILMN_3185784	LOC100125556	-0.589671653
ILMN_1664102	ISY1	-0.58972931
ILMN_2342762	KCNAB2	-0.589767126
ILMN_2349831	DICER1	-0.589837338
ILMN_2211630	HINT3	-0.589922899
ILMN_2204983	DCTN6	-0.589941584
ILMN_2359710	PTP4A3	-0.589970117
ILMN_1677962	GPHN	-0.589995323
ILMN_1756623	NT5C1B	-0.590033917
ILMN_1788928	ABCB9	-0.590058137
ILMN_2121555	FBXO5	-0.590266239
ILMN_3184599	FLJ37786	-0.590324039
ILMN_1874678	BQ050540	-0.590501685
ILMN_2393456	DDO	-0.590534272
ILMN_1767281	PPBP	-0.590777511
ILMN_2344850	VPS26A	-0.59103292
ILMN_2317493	SEMA6D	-0.591041854
ILMN_1701614	CDC73	-0.591083895
ILMN_1652662	GRB10	-0.591138425
ILMN_1831907	AI935777	-0.591139938
ILMN_1654013	C17orf28	-0.591402486
ILMN_2151056	C10orf32	-0.591428746
ILMN_1899400	DA411236	-0.591434403
ILMN_1808938	PIGF	-0.591474867
ILMN_1770005	PTPN18	-0.591567092
ILMN_2353129	EYA2	-0.591593523
ILMN_3265895	HNRNPR	-0.591633423
ILMN_1838660	BU620514	-0.591681069
ILMN_1732575	SEC14L1	-0.591792705
ILMN_1729019	sept-07	-0.591948442
ILMN_2319000	MATK	-0.592032606
ILMN_1778734	FAN1	-0.592058154
ILMN_1891324	AK126122	-0.592106411
ILMN_1881668	BC021745	-0.592195445
ILMN_1876729	LOC340544	-0.592234266
ILMN_1913641	AK055370	-0.592282692
ILMN_1661799	HIGD1A	-0.592300118
ILMN_1662964	PRMT3	-0.592373018
ILMN_2390526	RARB	-0.592423853
ILMN_1823477	BX117128	-0.592435622
ILMN_1762995	KANK1	-0.592444471
ILMN_1685722	EIF4A2	-0.592463556
ILMN_1805742	DHX37	-0.592519494
ILMN_1702443	DNMBP	-0.59259992
ILMN_1707368	OPRD1	-0.592735903
ILMN_1812503	PHKA1	-0.592763498
ILMN_1713896	FAM188B2	-0.592973459
ILMN_1692041	POT1	-0.592977832
ILMN_1708934	ADM	-0.593002156
ILMN_1699334	AATK	-0.593042199
ILMN_3308015	MIR18B	-0.59314403
ILMN_1798533	ZNF22	-0.593148096
ILMN_3237779	TMEM184C	-0.59315685
ILMN_1804152	KCNH6	-0.593401549
ILMN_1814526	ADD3	-0.593417559
ILMN_1740180	SNX3	-0.593446118
ILMN_2213558	Tmp21-II	-0.59346438
ILMN_1807771	CDK15	-0.593477977
ILMN_2348975	NASP	-0.593481023
ILMN_1689274	NIPA1	-0.593611372
ILMN_2233689	MS4A13	-0.593694833
ILMN_1832072	F09070	-0.593713383
ILMN_1727709	GPBAR1	-0.593834841
ILMN_3309133	MIR429	-0.593957502
ILMN_1806486	ARMC10	-0.593996444
ILMN_1799996	DHH	-0.594017367
ILMN_2167922	TRMT5	-0.594018506
ILMN_1664253	LRRC30	-0.594061059
ILMN_1880983	BC071719	-0.594074532
ILMN_2310253	TARBP2	-0.594109379
ILMN_1805853	PHLDB3	-0.594237784
ILMN_1696510	GPR89A	-0.594264033
ILMN_1831489	TCRA	-0.594284049

ILMN_2129563	LDLRAD3	0.481902388
ILMN_1676099	SPON2	0.481902142
ILMN_2392286	IP6K1	0.481655097
ILMN_2381655	EDA	0.481591922
ILMN_3268914	CLEC2D	0.481540292
ILMN_1694203	ZNF141	0.481528814
ILMN_1872122	LCOR	0.481498748
ILMN_3246953	FTSJD2	0.481485344
ILMN_1653026	PLAC8	0.481479123
ILMN_1678805	POMT2	0.481434704
ILMN_1728761	ERBB2	0.4813953
ILMN_1719975	HOXC4	0.481394934
ILMN_1713380	EIF2B2	0.481364481
ILMN_1658207	MSLNL	0.481315442
ILMN_1787689	RBFOX1	0.481249449
ILMN_1718336	C7orf50	0.481193576
ILMN_1771209	OR2T8	0.481146511
ILMN_2276811	METTL17	0.48094712
ILMN_1815445	IDS	0.480927479
ILMN_1694111	PNKP	0.480923556
ILMN_2044471	NCR3	0.480814864
ILMN_1679917	ARL16	0.480800823
ILMN_1815812	C16orf5	0.480706779
ILMN_1794392	DDX3X	0.480701849
ILMN_1654685	MCTP1	0.48060225
ILMN_2203891	SMAD7	0.48053687
ILMN_1676385	PAK2	0.48043501
ILMN_2117716	SFRS17A	0.480419939
ILMN_3234920	NPIPL3	0.480388729
ILMN_2092160	KIAA1704	0.480301716
ILMN_1720819	SPCS2	0.480022259
ILMN_2328776	MST4	0.480019812
ILMN_3178471	ESPNL	0.479972661
ILMN_1715189	LHX6	0.479917449
ILMN_2261379	SRGAP2	0.479784199
ILMN_2267383	CU687675	0.479714549
ILMN_1768260	GAS6	0.479707023
ILMN_1755290	CEP170	0.479672733
ILMN_2363489	BRE	0.4796454
ILMN_1799232	ATP9A	0.479623987
ILMN_3237448	BEND5	0.479600099
ILMN_1778240	GFOD1	0.479599068
ILMN_1712896	FST	0.479593699
ILMN_1731783	ATP1A1	0.479580672
ILMN_1748221	PADI6	0.479479147
ILMN_1756285	FFAR1	0.479264536
ILMN_2051731	DUSP5P	0.479235722
ILMN_1653788	RNF208	0.479227145
ILMN_1703500	KCNQ2	0.479126517
ILMN_2173835	FTHL3P	0.478856247
ILMN_1724598	RABL2A	0.478834361
ILMN_1755123	GBA	0.478795256
ILMN_2186061	PFKFB3	0.478780719
ILMN_1657406	NUP50	0.478692672
ILMN_1792433	H2BFM	0.478689315
ILMN_1809400	FAM49B	0.478630797
ILMN_1723843	CSNK2A2	0.478575377
ILMN_1762046	ZNF551	0.478456492
ILMN_2112049	DNLZ	0.478407055
ILMN_1658333	ECM1	0.478345883
ILMN_3238369	JHDM1D	0.478079146
ILMN_1700204	ZMYND8	0.477972309
ILMN_1711627	SIAH1	0.477971874
ILMN_1655961	NAG8	0.477942993
ILMN_1783832	GAGE6	0.477939798
ILMN_2411559	PUS1	0.477933775
ILMN_2355971	HSD17B10	0.477858477
ILMN_2363065	RTN3	0.477832464
ILMN_2290618	SEC22C	0.477801527
ILMN_1787691	CITED4	0.477771356
ILMN_2350801	SLC25A29	0.477729782
ILMN_1697629	JMJD7-PLA2G4B	0.477711459
ILMN_1670101	CLEC2D	0.477677585
ILMN_3192316	CLEC2D	0.477626611
ILMN_1663954	TH1L	0.477612686
ILMN_1702775	LRP5	0.477585623
ILMN_1776121	C2orf55	0.47758022
ILMN_1773809	FOXP4	0.477496046
ILMN_1751171	IRF2BP1	0.4774895
ILMN_2196335	C12orf11	0.477472587
ILMN_1740426	RASD1	0.477420498
ILMN_1723684	DARC	0.477385313
ILMN_1664466	KLHL9	0.477356996
ILMN_1806123	MRPL23	0.477335769
ILMN_2338452	SERPINA1	0.477317043
ILMN_1711688	SPDYE3	0.477286344
ILMN_1788019	LAMA2	0.477257898
ILMN_1759595	GNB1L	0.477177985
ILMN_1779813	FAM96B	0.477153543
ILMN_2075818	ZNF598	0.477044232
ILMN_1685678	EEF1B2	0.477017763
ILMN_1671809	DUSP22	0.477009429
ILMN_1703049	AKAP7	0.47698637
ILMN_1653340	RECQL5	0.476873421
ILMN_1723709	C9orf116	0.47669713
ILMN_1797793	BLVRB	0.476617497
ILMN_2081398	KIF3B	0.476582932
ILMN_1729208	NGFRAP1	0.476540375

ILMN_1692219	RAB11FIP1	-0.594302538
ILMN_1661994	ESRRG	-0.594590563
ILMN_1688666	HIST1H2BH	-0.594647942
ILMN_3239679	SNORD115-15	-0.594805109
ILMN_1779674	PTPRG	-0.594918168
ILMN_1706225	N4BP1	-0.594959459
ILMN_3241690	PBMUCL1	-0.594979759
ILMN_3284127	TRIM64B	-0.594992361
ILMN_3207902	GUSBP10	-0.595065886
ILMN_2133996	SHE	-0.59509045
ILMN_1748894	GTPBP3	-0.595227013
ILMN_1799710	CCDC96	-0.595331885
ILMN_2218002	LIPC	-0.595340029
ILMN_3239103	LOC340357	-0.59541972
ILMN_1746952	SLC25A48	-0.595539023
ILMN_1706057	CS	-0.595609612
ILMN_1683067	ELSPBP1	-0.595691076
ILMN_2226628	ZNF184	-0.59571385
ILMN_2115421	SLC25A16	-0.595805095
ILMN_1778876	FAM179B	-0.595854108
ILMN_3233871	ATXN1L	-0.59608404
ILMN_1862302	TMF1	-0.596584225
ILMN_1899387	BC039440	-0.596882805
ILMN_1657968	MAP2K2	-0.596895716
ILMN_1911007	AA448689	-0.597094137
ILMN_3307868	CHI3L1	-0.597202078
ILMN_1702171	LPCAT1	-0.597332606
ILMN_3307836	SLC5A12	-0.59742603
ILMN_2121816	GPR137B	-0.597510422
ILMN_1905548	MBNL3	-0.597533486
ILMN_1693043	BC037970	-0.597593653
ILMN_3296468	AK123450	-0.59760563
ILMN_3184433	LOC100130285	-0.597621607
ILMN_1910294	AA335815	-0.597701354
ILMN_1758474	PRKRA	-0.597721575
ILMN_1664348	PNPLA4	-0.59779371
ILMN_3268535	C15orf34	-0.597813064
ILMN_1746784	SLAIN1	-0.597943313
ILMN_1803211	FBXO2	-0.597952201
ILMN_1776412	KRTAP10-11	-0.59795327
ILMN_1877996	AW195018	-0.598027514
ILMN_1710200	NA	-0.598406758
ILMN_1656798	TIMM17A	-0.598537146
ILMN_2117904	ZNF22	-0.5985483
ILMN_1795704	KIAA0232	-0.598601209
ILMN_2347888	LARP4	-0.598954793
ILMN_1677464	LGR6	-0.599003131
ILMN_1718852	PLCL1	-0.59914607
ILMN_1842039	CA435101	-0.599189882
ILMN_2153916	HSPA2	-0.599224174
ILMN_1772712	UBE2J2	-0.599312391
ILMN_1665632	POU5F1	-0.599541857
ILMN_1666624	RABL2A	-0.59957178
ILMN_3251511	SCAF11	-0.59982377
ILMN_3307025	ZDHHC4	-0.599827347
ILMN_2064237	C1orf88	-0.599841042
ILMN_1755909	C20orf11	-0.59987522
ILMN_1798108	C6orf211	-0.599886585
ILMN_1707775	ZNF257	-0.599968887
ILMN_1787980	SNORD109B	-0.60009845
ILMN_1755834	FEN1	-0.600300503
ILMN_1743427	SCYL3	-0.600349202
ILMN_1727875	MUC6	-0.600430917
ILMN_1672287	MYNN	-0.600607909
ILMN_1760620	TMEM33	-0.600674075
ILMN_1712959	DUSP2	-0.600683812
ILMN_1683859	SLC7A1	-0.600755588
ILMN_2389935	FYTTD1	-0.600780393
ILMN_1727444	C16orf53	-0.600813286
ILMN_1739886	HNF4A	-0.600959687
ILMN_1694043	DNAH14	-0.600990258
ILMN_1754315	MMAA	-0.601084576
ILMN_1712161	BCOR	-0.601249206
ILMN_2377240	AKTIP	-0.601323123
ILMN_1769449	MBLAC2	-0.601356844
ILMN_1718925	C14orf49	-0.601467722
ILMN_1761260	COBLL1	-0.601555441
ILMN_2047354	C1orf97	-0.601574579
ILMN_2088612	XPO4	-0.601621083
ILMN_1696318	LOC100130691	-0.601622136
ILMN_1697550	ACYP2	-0.60175419
ILMN_1746856	RAB21	-0.601828183
ILMN_1893686	W95809	-0.601834274
ILMN_1874302	BE646122	-0.601878248
ILMN_1907861	BM850770	-0.601931262
ILMN_3199172	AI491714	-0.601934544
ILMN_2207562	AP1AR	-0.602037294
ILMN_3309443	MIR365-1	-0.602359615
ILMN_3228470	NACAD	-0.602372132
ILMN_1721138	GRPEL2	-0.602470532
ILMN_3199507	LOC283922	-0.602501233
ILMN_2139061	COPS2	-0.602507418
ILMN_1652357	PDHX	-0.602555056
ILMN_2058347	NT5DC1	-0.602559948
ILMN_1803180	PRDX6	-0.602722017
ILMN_2067813	OR1L4	-0.602738659
ILMN_2145760	YIPF4	-0.603116948
ILMN_1751051	C7orf25	-0.603228511

ILMN_1794666	TSSK1B	0.476446661
ILMN_1680145	SART1	0.476443829
ILMN_1681296	ICAM4	0.476418364
ILMN_1658464	GTF3A	0.47637742
ILMN_2402817	ZBTB16	0.476170656
ILMN_2413041	TEAD4	0.476110254
ILMN_1670028	LPIN2	0.476002341
ILMN_1786326	KCTD15	0.475916765
ILMN_2086105	SPRY4	0.475894501
ILMN_1789243	VPS33B	0.475839188
ILMN_1721046	PTMS	0.475741363
ILMN_1803561	PLA2G4A	0.475652918
ILMN_3242174	SEZ6L2	0.475626322
ILMN_1755405	PGAP2	0.47559919
ILMN_1660412	MGC27345	0.47557798
ILMN_1651719	MBTPS1	0.475576667
ILMN_1662426	AP2S1	0.4754879
ILMN_1736729	OAS2	0.475269334
ILMN_1679003	MLL	0.475269009
ILMN_3300226	LOC731275	0.475265165
ILMN_1730546	GNAO1	0.475202306
ILMN_1693598	RTN4	0.47511278
ILMN_1729832	EIF4H	0.474989502
ILMN_2235283	MAPK1	0.474878262
ILMN_1773082	CYP21A1P	0.474648548
ILMN_1719919	KLHL13	0.474638045
ILMN_1778631	DEFB136	0.474549034
ILMN_2063500	IFT52	0.47448982
ILMN_1781636	VWA1	0.474391788
ILMN_2078334	CNOT10	0.474356606
ILMN_2383913	WDR33	0.474227119
ILMN_1696360	CTSB	0.474226336
ILMN_1696347	CTSC	0.474219817
ILMN_2054510	BCAS3	0.474206862
ILMN_2391512	NAAA	0.474203659
ILMN_1711828	ANKRD10	0.474095753
ILMN_1796710	PHC1	0.474051299
ILMN_1813240	EIF1AX	0.473996482
ILMN_1700248	WDR86	0.473927025
ILMN_3298511	LOC728537	0.473844704
ILMN_1676938	HMGB1	0.473765357
ILMN_2347798	IFI6	0.473758484
ILMN_1672662	SLC20A1	0.473593374
ILMN_1695280	SMG6	0.473583284
ILMN_2364174	CSNK1A1	0.473525113
ILMN_1769207	KCTD7	0.473442011
ILMN_1766668	PCDH7	0.473428218
ILMN_2070375	GPR82	0.473416315
ILMN_1657754	RPAP2	0.473329437
ILMN_1678669	RRM2	0.473265084
ILMN_1777541	KLK6	0.473226936
ILMN_1673023	EP400	0.473220769
ILMN_1738251	DRD3	0.473197075
ILMN_2100000	DHX36	0.473107908
ILMN_2278850	RAB24	0.473043923
ILMN_3239383	CA5BP	0.472949868
ILMN_1733519	HMGB3	0.472913594
ILMN_3218223	MPHOSPH10	0.472871812
ILMN_1778143	GRAP2	0.472758034
ILMN_3200484	RPS4X	0.472728385
ILMN_1655068	TOM1L2	0.472651262
ILMN_1772706	C10orf32-AS3MT	0.47261059
ILMN_1741455	USP30	0.47252127
ILMN_2074432	OR2A42	0.472508058
ILMN_1686245	OR6Q1	0.472407993
ILMN_1786908	KRTAP26-1	0.472403704
ILMN_1698100	ANX2P2	0.472385098
ILMN_1760347	SRGN	0.472361027
ILMN_2359601	CAMK2G	0.472355945
ILMN_1773470	ST5	0.472342718
ILMN_2326953	LAT2	0.47219623
ILMN_1759359	BTF3L4	0.472110796
ILMN_1660663	DYRK1A	0.472090177
ILMN_1678501	ADD3	0.471837333
ILMN_1712432	PSMD2	0.471763835
ILMN_1804339	CAMK1G	0.471749731
ILMN_1804396	IRF2BPL	0.471702152
ILMN_1812940	TRMT1	0.471699321
ILMN_1809477	CARHSP1	0.471666465
ILMN_1812638	ATP5L	0.471641822
ILMN_1733667	DHX35	0.471561968
ILMN_3187497	AK123679	0.471499635
ILMN_1764764	MUM1	0.471390592
ILMN_2318725	EEF1B2	0.471384905
ILMN_3251634	SENP5	0.471344139
ILMN_1744050	ASB7	0.471265822
ILMN_1679299	IGSF1	0.471139825
ILMN_1785644	C20orf29	0.471107483
ILMN_2062620	NMT2	0.471057226
ILMN_1674620	SGCE	0.470996053
ILMN_1707943	C19orf39	0.470965564
ILMN_1738883	RNF135	0.470879291
ILMN_1684690	HDAC11	0.47085967
ILMN_1701789	IFIT3	0.470853221
ILMN_1737394	LMNA	0.470783416
ILMN_1742929	HESX1	0.470502419
ILMN_2300970	ETFB	0.470472229
ILMN_1723017	LOC728643	0.470454756

ILMN_3286751	LOC284648	-0.603384059
ILMN_1809964	OR10J1	-0.603421769
ILMN_1733904	C20orf114	-0.603467928
ILMN_1653622	LOC646329	-0.603516746
ILMN_1759549	SRGAP2	-0.603529404
ILMN_1723333	SCGB2A2	-0.603543197
ILMN_1665501	LOC389634	-0.603612023
ILMN_2283597	FAM134B	-0.603667135
ILMN_1769388	GJB2	-0.60396781
ILMN_1720270	CDR2	-0.604059937
ILMN_3245098	KC6	-0.604083135
ILMN_3274344	NA	-0.604094361
ILMN_1794949	RCAN2	-0.604131718
ILMN_1735762	NPNT	-0.604233822
ILMN_3223246	TBC1D3	-0.604237238
ILMN_1832155	DCUN1D5	-0.604344724
ILMN_1711227	GMDS	-0.604351912
ILMN_2221076	C17orf85	-0.604354545
ILMN_1660793	PAQR4	-0.604385504
ILMN_1670385	TUBAL3	-0.604599497
ILMN_1692477	MGAT5B	-0.604641165
ILMN_1804350	C1orf150	-0.604708563
ILMN_3252862	DKFZp667F0711	-0.6047268
ILMN_1676955	TYK2	-0.604952383
ILMN_1684885	KRTAP4-8	-0.604953301
ILMN_1793655	RGPD6	-0.605172982
ILMN_1888359	BMPR1B	-0.605345225
ILMN_1751839	RUNX3	-0.605366341
ILMN_1781980	CFC1B	-0.605404394
ILMN_1811599	OR52B6	-0.605432231
ILMN_3204346	FAM196B	-0.605560125
ILMN_1798827	SRBD1	-0.605574945
ILMN_1683148	PRICKLE2	-0.605615606
ILMN_1723969	PLCB1	-0.605705571
ILMN_1674458	KLHL7	-0.605739029
ILMN_2050109	GABARAP	-0.605786396
ILMN_3303965	ZC3H11A	-0.605895036
ILMN_1689001	CDK4	-0.605932367
ILMN_1670172	WDR33	-0.606096044
ILMN_3310446	MIR217	-0.606134174
ILMN_3269076	NA	-0.606148305
ILMN_1773073	PHYH	-0.606174475
ILMN_1903021	AX746755	-0.606354576
ILMN_1897741	CR610863	-0.60636129
ILMN_3234456	TSTD1	-0.606428717
ILMN_1806778	UBE2E1	-0.606526246
ILMN_2341815	TFG	-0.606541578
ILMN_3227600	ZNF385B	-0.606665051
ILMN_1706687	KLHL5	-0.606783126
ILMN_2202940	CHPT1	-0.606974967
ILMN_1801443	TSKU	-0.607011833
ILMN_2213247	SPCS2	-0.607097539
ILMN_2133146	NUDT21	-0.60714731
ILMN_3294612	LOC100132831	-0.607155144
ILMN_3251648	AQP7P1	-0.607172791
ILMN_1675018	IQCF4	-0.607229088
ILMN_1678828	KRTAP22-1	-0.607363652
ILMN_1842602	AW594636	-0.607381963
ILMN_1772728	FAM75A5	-0.607416157
ILMN_1834323	LOC151171	-0.607427345
ILMN_1659894	SLC25A26	-0.607584599
ILMN_1727458	HDAC1	-0.607638401
ILMN_1739872	ADAMTS18	-0.607906772
ILMN_2047914	NPY5R	-0.608071152
ILMN_3273383	AY461701	-0.608127369
ILMN_3270542	UQCRFS1	-0.608248654
ILMN_1733341	LOC646851	-0.608539551
ILMN_1788968	C11orf65	-0.60857672
ILMN_1890884	AA854345	-0.608646688
ILMN_1710758	RNF20	-0.608675358
ILMN_1719455	CRIP3	-0.608694921
ILMN_1696512	AHSP	-0.608823965
ILMN_2096985	ALDH6A1	-0.608986505
ILMN_1658684	ATP9B	-0.608991108
ILMN_1706783	TAGLN	-0.609005245
ILMN_1655906	FBXW7	-0.609007119
ILMN_1741219	RRAGB	-0.609053611
ILMN_1758680	AK127725	-0.609096148
ILMN_1758196	LOC619207	-0.609289566
ILMN_2282019	SYN3	-0.609374383
ILMN_1676075	KLHL12	-0.609374953
ILMN_1703475	CLSPN	-0.609467195
ILMN_1742889	WDR77	-0.609616534
ILMN_1782384	SPRYD5	-0.609653447
ILMN_1775814	GHR	-0.609678363
ILMN_2393994	CSPP1	-0.610117052
ILMN_2196097	PPP2CA	-0.610219034
ILMN_3243519	LOC390858	-0.610301244
ILMN_1781481	NA	-0.610418008
ILMN_3244286	TANC2	-0.610514734
ILMN_1838575	BX094949	-0.610679777
ILMN_1717935	SPTA1	-0.610804615
ILMN_1777990	FAM21B	-0.610985258
ILMN_1721680	LOC644075	-0.611084045
ILMN_1718971	TTC7B	-0.611209488
ILMN_3234936	EYS	-0.611212709
ILMN_3185020	AI926420	-0.611391399
ILMN_2131756	LRRC40	-0.611406186

ILMN_2053921	CAPZB	0.470374088
ILMN_3240420	USP18	0.470296977
ILMN_1738821	GOLGA2	0.47017019
ILMN_2401436	PKMYT1	0.47006553
ILMN_1790534	MAP2K3	0.46982654
ILMN_1725071	CCDC12	0.469767069
ILMN_1731180	EXOC2	0.469696705
ILMN_1690035	FLYWCH1	0.46969361
ILMN_1745356	CXCL9	0.469545851
ILMN_1815043	MRPS2	0.469511194
ILMN_1718898	HOXC9	0.469166293
ILMN_1802397	GNA11	0.469155385
ILMN_2077952	GALNTL1	0.469030402
ILMN_1671046	HSPB2	0.468888789
ILMN_1737320	ZDHHC15	0.468869693
ILMN_2191402	HLCS	0.468868484
ILMN_1759232	IRS1	0.468859672
ILMN_1720024	IL11RA	0.468677984
ILMN_1744239	FEM1B	0.468674348
ILMN_1658310	TM4SF19	0.468665886
ILMN_3211199	ZNF316	0.468548618
ILMN_1668979	DNAJB14	0.468471344
ILMN_1729611	REEP4	0.468418144
ILMN_1651949	SCYL3	0.468384948
ILMN_1772537	SCTR	0.468317378
ILMN_1679279	SYPL2	0.468253222
ILMN_1694530	DDA1	0.468249975
ILMN_2094396	LRRC8C	0.468071771
ILMN_1705447	AFG3L1	0.46803022
ILMN_1670378	FAM115A	0.46798101
ILMN_1714335	RDH10	0.467927954
ILMN_2246328	PTPN22	0.46787232
ILMN_1910908	BG205162	0.467862175
ILMN_3282587	H3F3A	0.467765952
ILMN_2334080	TNNT3	0.467743888
ILMN_3271300	NA	0.467654402
ILMN_1658526	RYK	0.467631966
ILMN_1700183	APLNR	0.467629316
ILMN_1655177	PI4KA	0.467589105
ILMN_2413318	C15orf57	0.46758674
ILMN_2392818	RTKN	0.467514782
ILMN_2219466	APOBEC3A	0.467438211
ILMN_1718808	AKAP10	0.467284708
ILMN_1746090	STT3A	0.467264334
ILMN_1743456	ZCCHC14	0.46722998
ILMN_1810531	DRG2	0.467194362
ILMN_1695422	NCL	0.467126139
ILMN_2389218	CDK18	0.467113279
ILMN_1716218	RPS6KA2	0.467021581
ILMN_3241834	RPL7	0.4669969
ILMN_2322446	PABPC1L2B	0.466978422
ILMN_2348093	ATP5J	0.466868302
ILMN_2145050	POM121	0.466793529
ILMN_1677273	TH	0.466772951
ILMN_1758487	CNPY4	0.466700641
ILMN_1683811	TNPO3	0.466651982
ILMN_1768488	TERF2	0.466641103
ILMN_1805522	KLHL31	0.466428599
ILMN_1689029	WDR7	0.466427333
ILMN_1680948	LMOD1	0.466343222
ILMN_1753716	EIF2B1	0.466289803
ILMN_3269405	HNRNPM	0.466165735
ILMN_1809173	LOC349114	0.466119182
ILMN_1755926	DBI	0.466071378
ILMN_3239168	LOC645332	0.466066812
ILMN_1759948	RNF5	0.466034427
ILMN_3240370	C11orf83	0.465980278
ILMN_2253246	DNAJC28	0.465971117
ILMN_2324202	GABRE	0.465854116
ILMN_1656073	MYL12A	0.465826038
ILMN_1683792	LAP3	0.465814484
ILMN_2380588	C6orf108	0.465801926
ILMN_1688625	AIM1	0.465789456
ILMN_1759312	AMPD1	0.465682339
ILMN_2373566	PJA1	0.465461571
ILMN_2078599	ACP5	0.465441991
ILMN_1669523	FOS	0.465385768
ILMN_2273902	ZAN	0.465283734
ILMN_1810915	FAAH	0.465174569
ILMN_1748529	RLN2	0.465173843
ILMN_1694126	KIF24	0.465069355
ILMN_1905564	AW044692	0.464821078
ILMN_1809895	FNBP1L	0.464811494
ILMN_2301722	PDE8B	0.464788472
ILMN_3302177	RPL14	0.464779803
ILMN_1801226	DOCK6	0.464695186
ILMN_2368617	FKRP	0.4646285
ILMN_3243441	EEF1A1	0.464469898
ILMN_1704342	UBE3C	0.464429326
ILMN_1703697	LANCL1	0.464404233
ILMN_1716821	GORASP1	0.464240367
ILMN_3245748	NCOR1	0.464166375
ILMN_1745256	CXXC5	0.46407019
ILMN_1714600	NAV3	0.464056819
ILMN_1760027	WAS	0.464009513
ILMN_1804812	ANAPC1	0.463779116
ILMN_1726928	TCEA3	0.463691086
ILMN_1687301	VCAN	0.46367043

ILMN_1711139	VPS37A	-0.611436682
ILMN_1800786	PARG	-0.611668334
ILMN_1753877	MYADML2	-0.611800266
ILMN_1780576	LPPR1	-0.612015681
ILMN_3241848	MIR3647	-0.61203019
ILMN_1745132	GDF11	-0.61231227
ILMN_1665205	ZNF260	-0.612364425
ILMN_1685493	TMCC3	-0.612403923
ILMN_1842850	AK026659	-0.612439105
ILMN_2052383	R3HDM1	-0.612455755
ILMN_1709925	GPR155	-0.612481682
ILMN_1826521	BX106259	-0.612532348
ILMN_2262462	C19orf29	-0.612580789
ILMN_2406468	NFU1	-0.612637715
ILMN_1706441	UPK3B	-0.612674962
ILMN_3244574	KIAA1430	-0.61274101
ILMN_3311075	MIR1915	-0.612758928
ILMN_1775467	OR2T12	-0.612808277
ILMN_1664921	PPP6C	-0.612921376
ILMN_2293758	AKAP11	-0.613054211
ILMN_1870097	AW561143	-0.613058852
ILMN_1783333	C16orf61	-0.613127137
ILMN_1781791	PRRG1	-0.613147613
ILMN_2391141	UBE3A	-0.613291756
ILMN_2073289	MTSS1	-0.613325118
ILMN_1763730	APPL1	-0.613388222
ILMN_3240451	VWDE	-0.613462933
ILMN_1722496	OR4L1	-0.613647663
ILMN_1811555	C9orf119	-0.613761323
ILMN_1806052	UNC119	-0.614059211
ILMN_1757165	LMBR1L	-0.614130564
ILMN_2084043	TCEAL7	-0.614184997
ILMN_2218780	PDP1	-0.614219948
ILMN_1763975	NCRNA00083	-0.614336441
ILMN_1866389	BQ182905	-0.61433752
ILMN_1683146	FTH1	-0.614584143
ILMN_1731175	XKR6	-0.614920636
ILMN_3230215	TOX2	-0.615030845
ILMN_1798254	ACTR10	-0.615113053
ILMN_1747466	BQ188360	-0.615224168
ILMN_1703006	MTCH1	-0.615449982
ILMN_1717094	ZNF618	-0.615646905
ILMN_1688455	KIAA0564	-0.615653006
ILMN_2166543	DNAJC6	-0.615697584
ILMN_1789387	KCNE3	-0.616031159
ILMN_2376133	KIAA1191	-0.616083074
ILMN_2220187	GFPT1	-0.616135482
ILMN_1680220	GEN1	-0.616358352
ILMN_1806312	C20orf30	-0.616393807
ILMN_1754795	FAT1	-0.61647043
ILMN_2161556	C12orf49	-0.616491946
ILMN_3239881	PRSS36	-0.616515835
ILMN_1853631	BX115738	-0.616522983
ILMN_3249291	F2	-0.616552384
ILMN_1686646	BC031092	-0.616616951
ILMN_3199608	LOC283332	-0.616684047
ILMN_1709728	SLC30A5	-0.616709077
ILMN_1686152	GGA2	-0.616712152
ILMN_3275637	NA	-0.616749354
ILMN_1781669	PTH	-0.616818921
ILMN_1744709	DLG5	-0.61691978
ILMN_1661451	FAM138F	-0.616943117
ILMN_1730432	IGSF8	-0.616952961
ILMN_1740418	CYP27B1	-0.617066574
ILMN_3260017	HNRNPK	-0.617080625
ILMN_1857768	SRCIN1	-0.617084777
ILMN_2230592	MRPL3	-0.617239355
ILMN_1729563	UGDH	-0.617278718
ILMN_1772663	ANKLE2	-0.617428096
ILMN_2129309	SEC23A	-0.617486775
ILMN_1695395	XYLT1	-0.61749004
ILMN_1791466	HMGB1	-0.617584617
ILMN_3261449	LOC100130815	-0.617685758
ILMN_1767474	HAO2	-0.617697931
ILMN_1733851	DACT3	-0.617741536
ILMN_2175317	CYP4X1	-0.61782381
ILMN_2111323	PDCD6IP	-0.617958914
ILMN_1765287	DPY19L3	-0.617980405
ILMN_1761112	PORCN	-0.617981265
ILMN_3308881	MIR196A1	-0.618008129
ILMN_1651254	LPP	-0.618205763
ILMN_1707314	PAX3	-0.618225736
ILMN_2117736	MGAT4C	-0.618297938
ILMN_1794767	OR52N5	-0.618614929
ILMN_1803162	RTEL1	-0.61862746
ILMN_1721922	NAB2	-0.618643193
ILMN_1675687	NA	-0.618645791
ILMN_1694514	ZDHHC11	-0.618683637
ILMN_1764609	PWWP2B	-0.618772007
ILMN_1685228	RHOXF2B	-0.618905384
ILMN_1770172	ZNF669	-0.619049861
ILMN_1693145	BUB3	-0.619079242
ILMN_1721921	BLMH	-0.619217373
ILMN_1703886	SLC16A2	-0.619330504
ILMN_3234735	ERI2	-0.619390768
ILMN_1774476	TCTEX1D4	-0.619423655
ILMN_1665033	NPR3	-0.619449399
ILMN_1786050	RBBP9	-0.619558647

ILMN_1699206	FHDC1	0.463632002
ILMN_1668752	C19orf60	0.463627571
ILMN_1792242	C8orf44	0.463627202
ILMN_1735438	GPM6B	0.463614821
ILMN_1680618	MYC	0.46353379
ILMN_3246005	KDM5B	0.463510041
ILMN_2350634	EFEMP1	0.463480741
ILMN_1768598	LAIR1	0.46345052
ILMN_1692973	BICD1	0.463183758
ILMN_1719123	CRX	0.463175213
ILMN_1709227	CCDC84	0.463124951
ILMN_2395375	GABBR1	0.463076659
ILMN_3245314	FAM176B	0.463073737
ILMN_2347949	G6PD	0.463062136
ILMN_3278170	LOC441455	0.463034854
ILMN_1745826	KATNAL2	0.463024206
ILMN_1903565	GNA11	0.462944974
ILMN_1665107	ITGB1BP1	0.46293471
ILMN_1798172	IPO4	0.462793374
ILMN_1691151	CHKA	0.462676825
ILMN_1687546	HSP90AA1	0.462632466
ILMN_1781897	KLRC3	0.462457513
ILMN_1696657	LRRN2	0.462444852
ILMN_2402079	ZMIZ2	0.462442299
ILMN_1799667	KIF4A	0.462438315
ILMN_2323508	AIF1L	0.462277072
ILMN_2353110	PTGER3	0.462208019
ILMN_1680070	MUC6	0.462192318
ILMN_1789502	GPC4	0.462064654
ILMN_1804306	LYPLA2	0.462046817
ILMN_1775930	PNLIPRP1	0.462026047
ILMN_2341793	CCT7	0.461836907
ILMN_3251379	SLC35E1	0.461827491
ILMN_2414679	SFI1	0.461824856
ILMN_1811616	EEPD1	0.461799949
ILMN_1705107	SDCBP2	0.461743474
ILMN_1701308	COL1A1	0.461741168
ILMN_1652677	FAM89A	0.461708785
ILMN_3241884	SNORA15	0.461676922
ILMN_2348243	STRADA	0.461615534
ILMN_1731178	ARHGAP28	0.461533723
ILMN_2052790	NONO	0.461523551
ILMN_1789358	ZNF628	0.461415454
ILMN_1779190	ALKBH8	0.46137252
ILMN_2242345	LPAL2	0.461203141
ILMN_1661490	PFDN6	0.461060875
ILMN_1799120	WFDC2	0.46102565
ILMN_2315964	PSRC1	0.46089318
ILMN_3236381	MXRA8	0.46089156
ILMN_1790461	C6orf125	0.460885337
ILMN_3241237	RNF208	0.460830256
ILMN_1683328	IP6K2	0.46077479
ILMN_1742923	ZNF584	0.460758874
ILMN_1700428	HLA-DOB	0.46074874
ILMN_1651278	SNIP1	0.460716101
ILMN_2355462	CYFIP1	0.460654931
ILMN_2129015	AFF1	0.460633717
ILMN_1657235	PALM3	0.460628645
ILMN_1855286	C6orf204	0.460493795
ILMN_2089340	FAM127B	0.46042043
ILMN_1804884	C22orf39	0.46038959
ILMN_1662340	ZNF358	0.460325288
ILMN_1684591	ZNF434	0.460303635
ILMN_1673384	GEMIN7	0.460197359
ILMN_1687821	C16orf45	0.460093496
ILMN_1707710	SLC6A13	0.460021814
ILMN_1683692	LRCH1	0.460007563
ILMN_1794132	NDUFS8	0.459938941
ILMN_3238680	C7orf55	0.459751292
ILMN_2292517	EEF1E1-MUTED	0.45970228
ILMN_2227195	CCDC65	0.459489208
ILMN_1766675	CDH6	0.459483544
ILMN_2413622	RFX4	0.459446972
ILMN_1750153	OR52A1	0.459331646
ILMN_1805481	TRMT11	0.459315121
ILMN_3238676	ULBP2	0.459293854
ILMN_1792400	CSN3	0.459293735
ILMN_2201678	FSTL1	0.459255265
ILMN_3247139	C17orf96	0.45910681
ILMN_1742872	UBA2	0.459093753
ILMN_3240301	TOMM40	0.459068361
ILMN_3246560	EMB	0.459017197
ILMN_1745655	PEX16	0.45897321
ILMN_2145914	FAM153A	0.458942741
ILMN_1767219	POLR1C	0.458898028
ILMN_1732375	EEFSEC	0.458815797
ILMN_1723185	ELOF1	0.458774782
ILMN_1724148	ORAI1	0.458665186
ILMN_2339705	MED8	0.45866039
ILMN_2383484	C19orf48	0.458576108
ILMN_1737475	ABHD11	0.458468899
ILMN_1792168	GALE	0.458466091
ILMN_1798204	IL21R	0.458358194
ILMN_1789913	SMPDL3B	0.458293124
ILMN_1790741	RNF126	0.458158688
ILMN_2123871	TMEM18	0.458136739
ILMN_1699172	AFG3L1	0.458037936
ILMN_1761519	EIF4G2	0.457996177

ILMN_1795304	CRLF2	-0.619624165
ILMN_2223720	ATMIN	-0.619650815
ILMN_1693410	BRI3BP	-0.619702079
ILMN_1698673	EFCAB7	-0.619746121
ILMN_2076567	UBE2V2	-0.619780215
ILMN_1815658	C3orf78	-0.619869413
ILMN_1673950	FAM47E-STBD1	-0.619883985
ILMN_1718253	TAS2R3	-0.619889021
ILMN_1803611	SNAPC3	-0.619957826
ILMN_1894900	AA827722	-0.619989022
ILMN_1732374	ATAD5	-0.619999203
ILMN_2385672	ELN	-0.620029955
ILMN_1687751	BAALC	-0.620157648
ILMN_1840316	BC064139	-0.620387556
ILMN_1693519	PRKG1	-0.620392628
ILMN_2197228	DEFB122	-0.620458612
ILMN_1659270	OTP	-0.620532007
ILMN_1714364	PTK2	-0.620658056
ILMN_1709204	CLEC4A	-0.620753683
ILMN_1689156	MMAB	-0.621131872
ILMN_1670547	GRM1	-0.621273653
ILMN_1820505	AA435525	-0.621285357
ILMN_2227817	GSTA3	-0.621417886
ILMN_1736888	SAR1B	-0.621708112
ILMN_1722056	ATP7B	-0.621780799
ILMN_1825529	BX093041	-0.621784653
ILMN_1764826	TFE3	-0.621909651
ILMN_1815442	AK024148	-0.622035968
ILMN_2351241	RNF14	-0.622040756
ILMN_1738849	SLC9A2	-0.622184236
ILMN_2110094	CFC1	-0.62220565
ILMN_1748007	KREMEN1	-0.622299844
ILMN_1844882	BQ024020	-0.622418726
ILMN_2174702	UBE2K	-0.622575192
ILMN_3302625	DA626616	-0.622587454
ILMN_1779279	MAPK1IP1L	-0.622664047
ILMN_1681558	KRTAP1-5	-0.622871621
ILMN_1836218	LOC25845	-0.622918113
ILMN_1752669	ALPI	-0.622952046
ILMN_1782798	MDFI	-0.622971903
ILMN_1804283	GNAS	-0.623119164
ILMN_2120360	LOC729164	-0.623330464
ILMN_2302632	HFE	-0.623333421
ILMN_1687848	C7	-0.623351603
ILMN_1738736	SNX4	-0.623480487
ILMN_3260715	CRTC3	-0.623498877
ILMN_1656111	MYLIP	-0.62361856
ILMN_1750940	SLITRK1	-0.624019657
ILMN_1683231	FAM83F	-0.624030584
ILMN_1700596	ALS2CL	-0.62405157
ILMN_1900929	OR7E12P	-0.624121943
ILMN_3235231	SNORA49	-0.624255086
ILMN_2267131	MAGEA11	-0.624269664
ILMN_1760542	PSMA1	-0.624306264
ILMN_1769556	NPR3	-0.624338246
ILMN_1689473	ATP6V1H	-0.624342012
ILMN_1767690	SENP8	-0.62457379
ILMN_1818620	AW341478	-0.624597146
ILMN_2387680	VNN3	-0.62475693
ILMN_1671621	PCMT1	-0.624781238
ILMN_1780141	TMEM66	-0.624843119
ILMN_1793203	SMCR7L	-0.624879044
ILMN_3238143	D2HGDH	-0.624940808
ILMN_1697922	KIAA1109	-0.625023584
ILMN_1764361	DUSP16	-0.62507808
ILMN_1726359	NECAP1	-0.625079271
ILMN_2228938	ECM2	-0.625247818
ILMN_1666939	MEI1	-0.625281424
ILMN_1805024	ERBB2IP	-0.625367443
ILMN_2111187	ELOVL6	-0.625441968
ILMN_1893466	AW293663	-0.625553739
ILMN_1697118	ARMC6	-0.625569762
ILMN_1655261	ERP27	-0.625592558
ILMN_1794187	FBXL3	-0.62570202
ILMN_1683903	PDPK1	-0.625707287
ILMN_1716591	SPRR1A	-0.625749967
ILMN_1699724	NA	-0.625812502
ILMN_2142117	LYPLAL1	-0.626053103
ILMN_1798870	RBPJL	-0.626182907
ILMN_1658302	PIAS2	-0.626243684
ILMN_1724902	SLC22A18	-0.6263101
ILMN_1847202	AW173314	-0.626313541
ILMN_1672605	C7orf41	-0.626344369
ILMN_1811029	TLK1	-0.626446525
ILMN_3305675	PRAMEF11	-0.626495725
ILMN_1666306	SRRD	-0.626684654
ILMN_1708508	PPM1E	-0.62672036
ILMN_1744517	GNS	-0.626783978
ILMN_1916599	LOC100133985	-0.626852345
ILMN_1762151	PLK5	-0.627074526
ILMN_1893850	BX097731	-0.627181798
ILMN_1911344	AV650212	-0.627231035
ILMN_1702764	MARCH5	-0.627381527
ILMN_1831098	BX092776	-0.627408301
ILMN_1878019	DOK6	-0.627569302
ILMN_1728657	VCX3B	-0.627598747
ILMN_1762021	TRIM48	-0.627676764
ILMN_1687437	CCDC57	-0.627703013

ILMN_2218856	CCL3L3	0.457955512
ILMN_3236156	OSTC	0.457888017
ILMN_1721580	TBX15	0.457856989
ILMN_2054576	EP400NL	0.457828823
ILMN_1745469	SEC24B	0.457799775
ILMN_2249920	FYN	0.457745052
ILMN_1815012	EXOC7	0.457725715
ILMN_1810856	HDAC5	0.457696899
ILMN_1663033	TMEM129	0.45767152
ILMN_1677483	EXOSC1	0.457621799
ILMN_1698213	RBM3	0.457590194
ILMN_1801633	ROMO1	0.457557309
ILMN_2080684	IQCA1	0.457503224
ILMN_2388965	ARHGEF25	0.457444348
ILMN_2386008	MPZL1	0.457301489
ILMN_3307483	MCF2L	0.457157981
ILMN_1740487	CMTM7	0.457134918
ILMN_1763328	GZF1	0.456961327
ILMN_1689560	C14orf181	0.45690569
ILMN_1705594	NAT10	0.456817072
ILMN_2410449	PCDHA@	0.456808366
ILMN_1787127	SLC43A2	0.456752318
ILMN_1782389	LAD1	0.456717106
ILMN_1780756	RBM23	0.456620547
ILMN_1682037	FBXL8	0.456588764
ILMN_2126038	STMN2	0.456539665
ILMN_1706963	ADAR	0.456445649
ILMN_1708064	MAP4	0.456404298
ILMN_2266573	SLC12A6	0.456277708
ILMN_1750145	YWHAG	0.456187584
ILMN_1795865	FGFRL1	0.456137322
ILMN_1706268	PTGDR	0.456111198
ILMN_1710482	APLP2	0.456033576
ILMN_1878016	AA883754	0.456030945
ILMN_1805027	KLC3	0.456008993
ILMN_1691787	RAPH1	0.4558216
ILMN_1804316	TCAP	0.455727356
ILMN_3286235	FAM66A	0.455725456
ILMN_1787370	C2orf56	0.455561775
ILMN_1714433	MARCKSL1	0.455561396
ILMN_1672611	CDH11	0.455531323
ILMN_1773066	CDKN2AIP	0.455444038
ILMN_1714691	HOXD10	0.455431272
ILMN_1698139	TCEA1	0.455411583
ILMN_1803788	LGALS3	0.45515377
ILMN_3234997	MAP7D1	0.455078367
ILMN_3288018	LOC645323	0.455047001
ILMN_1754235	SLC35C2	0.455010218
ILMN_2251225	WDR78	0.45488833
ILMN_1769229	BCL2A1	0.454785583
ILMN_1769027	CDC42SE1	0.454747731
ILMN_2227573	GSTO1	0.45466106
ILMN_2188333	CD69	0.454600475
ILMN_1760305	FBRSL1	0.454474018
ILMN_1738578	FILIP1L	0.454469981
ILMN_1750459	ANKRD36	0.454344701
ILMN_1766408	CBFB	0.454258151
ILMN_3266197	NA	0.454233866
ILMN_1842582	TTC39C	0.454204496
ILMN_3244483	RNF216L	0.454188269
ILMN_2095273	KIAA1407	0.45407177
ILMN_1783846	RAPH1	0.453963601
ILMN_1666222	PHACTR3	0.453896374
ILMN_2276688	DIXDC1	0.453761235
ILMN_1739236	ZNF668	0.453740441
ILMN_1730685	MRPL16	0.453702676
ILMN_2298028	GPR173	0.453701378
ILMN_2372040	MTFP1	0.453697202
ILMN_1662390	ASPHD1	0.453680573
ILMN_1778625	CD44	0.453672298
ILMN_1657683	C1orf198	0.453574021
ILMN_1773154	NFKBIA	0.453569521
ILMN_1701724	GET4	0.45352737
ILMN_1728435	C12orf45	0.453517918
ILMN_3247965	DQ594366	0.453486636
ILMN_1712887	SLC10A3	0.453463088
ILMN_2253759	IL18BP	0.453428118
ILMN_1677002	LOC644083	0.453284703
ILMN_3235556	DQ594366	0.453182785
ILMN_3307926	ADRBK1	0.45317854
ILMN_1761478	AK130343	0.453105179
ILMN_1808939	RPS6	0.453042324
ILMN_1789086	LOC100288069	0.453009053
ILMN_1778587	PPP2R2D	0.452992957
ILMN_1740005	SERPINF2	0.452949688
ILMN_1673329	TXNDC11	0.452907982
ILMN_1777853	MBOAT2	0.452852999
ILMN_1688480	CCND1	0.452528659
ILMN_1745784	ZNF324	0.452343551
ILMN_1797544	HYDIN	0.452304476
ILMN_1829845	NA	0.45217338
ILMN_2110829	RPL23A	0.452121336
ILMN_1711790	DEFB135	0.451942041
ILMN_1695432	TPST2	0.451902668
ILMN_2188264	CYR61	0.451805842
ILMN_1712390	CUTA	0.451702463
ILMN_1830984	BF509118	0.451460918
ILMN_1658955	EIF4E	0.451417652

ILMN_2403946	FEZ2	-0.627797158
ILMN_3307863	TAOK3	-0.627949667
ILMN_1769503	ZNRF2	-0.627963887
ILMN_1763516	SPINK2	-0.627989548
ILMN_1654759	WWC2	-0.628091438
ILMN_1671215	FBXL19	-0.628112417
ILMN_1682781	TEAD2	-0.628125403
ILMN_1659086	DQ895884	-0.628153028
ILMN_1661002	RFWD2	-0.628360139
ILMN_1729515	PIN4	-0.628390005
ILMN_2066904	OR4C12	-0.628623451
ILMN_1715181	ARRDC2	-0.628636416
ILMN_1735822	TTC30A	-0.62863791
ILMN_1764871	PIGP	-0.628816054
ILMN_1843101	AA866199	-0.628970701
ILMN_1713682	FBXO11	-0.629022575
ILMN_3238684	DEFB116	-0.629126282
ILMN_1819991	AI792396	-0.62920237
ILMN_1695290	FERMT2	-0.629204492
ILMN_1892161	DB338885	-0.629323683
ILMN_2352917	LTBP4	-0.629346595
ILMN_1696935	RBM39	-0.62935627
ILMN_1849726	AA526513	-0.629608105
ILMN_1758104	PRPS2	-0.629868632
ILMN_1861414	BF593077	-0.629874058
ILMN_2203768	PRR15	-0.629885318
ILMN_1708721	RABGAP1L	-0.629909316
ILMN_1759910	SERPINA5	-0.629921237
ILMN_2201668	SLC19A2	-0.62993226
ILMN_1798043	LOC339674	-0.629968998
ILMN_1709247	PPP2R1B	-0.630227269
ILMN_1893948	BX109720	-0.630240255
ILMN_1663664	MRPS10	-0.63035728
ILMN_1745904	CCDC6	-0.630421374
ILMN_1808305	RTCD1	-0.630507774
ILMN_1757504	REG3A	-0.630561799
ILMN_3242710	LOC157627	-0.63057538
ILMN_1682034	HEY2	-0.63067584
ILMN_2325008	DHX40	-0.630678218
ILMN_1781356	TSC22D3	-0.630701083
ILMN_1747099	LUC7L2	-0.630839944
ILMN_2238795	NEK3	-0.631005132
ILMN_2190051	CCDC91	-0.631033416
ILMN_2359332	CDKN2C	-0.631093171
ILMN_3177250	WSCD2	-0.631135395
ILMN_1798886	NUDT21	-0.631154191
ILMN_1753856	ZNF407	-0.631174276
ILMN_1669393	GGT1	-0.631391148
ILMN_1670875	PPM1D	-0.63139995
ILMN_1826853	NA	-0.631595895
ILMN_1785406	NAP1L3	-0.631632215
ILMN_1793410	SNTB1	-0.63166814
ILMN_1783448	DYNC1LI2	-0.631752677
ILMN_1677440	ATP6AP2	-0.631856317
ILMN_1756767	EIF5B	-0.63189609
ILMN_1840311	DA494348	-0.631897593
ILMN_3310030	MIR1265	-0.631914288
ILMN_2343097	NCALD	-0.632018372
ILMN_1803110	SF3B3	-0.632078177
ILMN_2360054	DDR1	-0.632235099
ILMN_1775831	LOC100287166	-0.632246955
ILMN_1824490	BG618056	-0.632339471
ILMN_1810324	SGCB	-0.632439536
ILMN_1813352	CDH7	-0.632519004
ILMN_1715437	CASC5	-0.6327943
ILMN_1866337	AK093987	-0.632965656
ILMN_2285480	LBR	-0.633039175
ILMN_1894480	BU855480	-0.633097828
ILMN_1749466	VAT1L	-0.633242721
ILMN_1731521	TSPY3	-0.633431486
ILMN_1804490	LOC100630923	-0.633659548
ILMN_1706553	SMG7	-0.633680726
ILMN_1762360	THSD7A	-0.633773025
ILMN_1702850	FAM90A7	-0.633788412
ILMN_3251467	LRRC58	-0.633817548
ILMN_3240740	EIF3L	-0.633835364
ILMN_2314140	PAX6	-0.633892095
ILMN_1692294	PLEKHA7	-0.633929044
ILMN_1660775	TPM3	-0.633958164
ILMN_2343010	BOLA3	-0.634023846
ILMN_1795300	ARHGEF37	-0.634191134
ILMN_1717180	MTMR6	-0.634227827
ILMN_1688663	IFNA1	-0.634339189
ILMN_1799128	SLC30A9	-0.634465299
ILMN_1754423	C3orf23	-0.634671492
ILMN_1810604	ELMOD1	-0.634693874
ILMN_3309001	MIR1-2	-0.634721119
ILMN_1738818	RAG1	-0.634861433
ILMN_1717852	USH1G	-0.634899267
ILMN_1665423	CNTF	-0.635256533
ILMN_1691466	YES1	-0.635323873
ILMN_1673752	C1orf91	-0.63550161
ILMN_1803279	TMED5	-0.635648275
ILMN_3251269	SRP14	-0.635653234
ILMN_1699216	FLJ45721	-0.635680045
ILMN_2230566	RAB40B	-0.635847552
ILMN_1892573	BC031234	-0.636227278
ILMN_1783709	RRAGA	-0.636256049

ILMN_1693207	KIR2DL4	0.451335769
ILMN_1670640	ZNF664	0.451328026
ILMN_3235718	LOC91316	0.451292638
ILMN_1718552	CXCL13	0.451277745
ILMN_1748358	MBNL1	0.451195365
ILMN_1781721	DDX31	0.451174027
ILMN_2413527	VCL	0.451164379
ILMN_2299221	RABGGTA	0.451139408
ILMN_2192620	DCAF15	0.450983246
ILMN_1700597	CDRT4	0.450980163
ILMN_1769822	PLAA	0.450942904
ILMN_1701213	PIP4K2B	0.450901367
ILMN_1719641	SMOC2	0.450618935
ILMN_3287219	NA	0.450616154
ILMN_1663562	OR2A42	0.45055625
ILMN_1717934	SYT11	0.450473758
ILMN_1794505	SHFM1	0.450420701
ILMN_1757387	UCHL1	0.450410671
ILMN_1786108	TUT1	0.450388486
ILMN_1677603	C1S	0.450361195
ILMN_1806603	MESP1	0.450353106
ILMN_1801934	SERTAD3	0.450348138
ILMN_1707727	ANGPTL4	0.450277072
ILMN_1659888	PPP1R14B	0.450237212
ILMN_1725787	RFX1	0.450228384
ILMN_1730625	AHSG	0.450226388
ILMN_3307882	C2orf50	0.450150111
ILMN_2413084	HSPA8	0.450070148
ILMN_1808114	LYVE1	0.450025942
ILMN_1683179	RRAD	0.450021777
ILMN_2334042	THYN1	0.450003335
ILMN_1761782	RAMP3	0.449990588
ILMN_2398627	TENC1	0.449953541
ILMN_1709860	UNC45A	0.449928803
ILMN_1662492	GDNF	0.449845786
ILMN_1854607	CD673602	0.449798714
ILMN_1721842	RYBP	0.449778316
ILMN_1814661	PHLPP1	0.449720527
ILMN_2355586	ATRN	0.449716285
ILMN_1727790	KHDRBS3	0.44917612
ILMN_1675062	MYL9	0.449035253
ILMN_1698554	AACS	0.449027961
ILMN_1716026	ARMCX6	0.44900416
ILMN_2374340	PLAUR	0.448993584
ILMN_1730709	BSG	0.448969185
ILMN_3181833	AF339771	0.448926066
ILMN_2324421	TXNRD1	0.448893274
ILMN_1654516	TMEM120A	0.448843108
ILMN_1768818	LRRC46	0.448812259
ILMN_2163070	KHDC1	0.448676749
ILMN_1658327	BAZ1A	0.448652182
ILMN_2062714	PTGDR	0.448595318
ILMN_1758084	GLRX3	0.448466704
ILMN_1724437	GCAT	0.448448485
ILMN_2308338	BMF	0.448394207
ILMN_1786843	KCTD13	0.448360188
ILMN_2301083	UBE2C	0.44834603
ILMN_2274420	SPTLC1	0.448278857
ILMN_1755411	RBMS2	0.448275523
ILMN_2138019	HEPACAM	0.448242411
ILMN_1810374	TMEM156	0.448185335
ILMN_1769412	RAPGEF1	0.448157932
ILMN_3204275	SARNP	0.44810152
ILMN_1752299	RAB6A	0.448079171
ILMN_1694400	MSR1	0.447879474
ILMN_1739576	CYB5R2	0.447874687
ILMN_2259815	TBX20	0.447794395
ILMN_1814270	LY6D	0.447707612
ILMN_3247728	RNF115	0.447689733
ILMN_1759460	TAF7	0.447649708
ILMN_2071446	PI15	0.447622973
ILMN_1785520	PPIP5K2	0.447594454
ILMN_1753342	SAT1	0.447551334
ILMN_1752582	RAB5B	0.447479066
ILMN_1698666	CST6	0.447335836
ILMN_1767662	LASS6	0.447276442
ILMN_1726578	ZNF93	0.447253459
ILMN_1656670	HLA-G	0.447239698
ILMN_1656662	RPL24	0.447193867
ILMN_1677178	DEFB121	0.447181985
ILMN_1684278	SNORD38A	0.447136588
ILMN_2408139	CDC42	0.446927372
ILMN_1790962	RINL	0.44691983
ILMN_1710710	DEDD	0.446905608
ILMN_2384561	TJP2	0.446842247
ILMN_1690616	C17orf109	0.446703738
ILMN_2307903	VCAM1	0.446658421
ILMN_1733107	NOC4L	0.446613497
ILMN_1880280	AK025793	0.446593257
ILMN_1739441	GANAB	0.446551282
ILMN_1795991	C22orf28	0.446503569
ILMN_1800512	HMOX1	0.446500699
ILMN_1748625	TCEAL4	0.446465295
ILMN_3191861	NA	0.446371082
ILMN_1728677	CREB5	0.446300877
ILMN_1663042	SDC4	0.44619559
ILMN_1809417	LRFN4	0.446140996
ILMN_1811507	LBH	0.446122219

ILMN_3241607	FLJ00326	-0.636277859
ILMN_1752117	TMEM55A	-0.636371373
ILMN_1656424	SNRPE	-0.636421075
ILMN_1798837	PTPN20A	-0.636496897
ILMN_1863979	CB049834	-0.636636566
ILMN_3218529	ABI2	-0.636684953
ILMN_1670817	GRPEL1	-0.636735065
ILMN_1758811	IMPA1	-0.636977229
ILMN_2161832	VPS37A	-0.637054317
ILMN_1803452	CRCT1	-0.637147389
ILMN_1748323	CXCL14	-0.637398032
ILMN_1651964	ABCC5	-0.63745316
ILMN_2207988	SERPINI1	-0.637568627
ILMN_1704166	LNP1	-0.637570901
ILMN_2080637	ZBTB44	-0.637603061
ILMN_1770080	FAM159B	-0.637620838
ILMN_1763604	CACNB1	-0.637662263
ILMN_1764549	UBE3A	-0.63785974
ILMN_1787627	GPRASP1	-0.637861256
ILMN_2103774	PIP5KL1	-0.637960385
ILMN_1803749	BC010180	-0.637991224
ILMN_1859665	AW629225	-0.638038533
ILMN_1660321	CDH24	-0.638054852
ILMN_1692831	HIST1H1A	-0.638061228
ILMN_2384629	RGS12	-0.638162902
ILMN_3250798	C11orf58	-0.638228436
ILMN_1767070	VN1R5	-0.638241011
ILMN_1699411	GNG4	-0.638263722
ILMN_3225624	TATDN1	-0.638323408
ILMN_1687036	MRPL47	-0.638542343
ILMN_1734660	ICAM4	-0.638645084
ILMN_1671051	CD276	-0.638700831
ILMN_2392674	PRR3	-0.638736166
ILMN_1738938	TIMM8B	-0.638950683
ILMN_1801378	COQ3	-0.639114514
ILMN_1755792	STK38L	-0.639185771
ILMN_2308903	WFDC3	-0.639435112
ILMN_2408815	NAP1L1	-0.639739234
ILMN_1692844	TBC1D19	-0.639872776
ILMN_1896277	BC037739	-0.639895261
ILMN_1727738	RAB33B	-0.639983812
ILMN_1670144	OR10H2	-0.640075907
ILMN_1704247	LAMA3	-0.640419901
ILMN_1856533	CDKL2	-0.640531771
ILMN_1669172	PTPRR	-0.640569578
ILMN_2388363	IMPDH1	-0.640584885
ILMN_1810785	RNF11	-0.640631889
ILMN_1746945	CSMD1	-0.64066436
ILMN_1690329	FAM118B	-0.640698921
ILMN_1692588	HEATR5A	-0.640824744
ILMN_1870747	BX116001	-0.640955856
ILMN_1663538	CLYBL	-0.641020382
ILMN_1699844	SCRN3	-0.641090966
ILMN_1831176	BG208291	-0.64129552
ILMN_1684227	GPR146	-0.641315603
ILMN_1775423	C10orf88	-0.641364867
ILMN_1795701	STARD9	-0.641501915
ILMN_1651617	CTNNB1	-0.641568102
ILMN_1800374	IRX6	-0.641624418
ILMN_1726409	KRT23	-0.641752628
ILMN_1815859	ERCC2	-0.641898385
ILMN_2210713	COMMD8	-0.641903579
ILMN_2364272	MBNL2	-0.641992558
ILMN_1788605	LOC220115	-0.64202456
ILMN_1685176	SGMS2	-0.642301998
ILMN_1808683	OR4A16	-0.642302744
ILMN_1736077	LIAS	-0.642342016
ILMN_2374778	DUT	-0.642448276
ILMN_1711318	PHF8	-0.642500276
ILMN_1805127	C2CD2	-0.642674683
ILMN_1691803	KIR2DS2	-0.642756425
ILMN_2154455	ZNF330	-0.642911365
ILMN_2380754	CCNG1	-0.643196732
ILMN_1681884	LOC100506049	-0.643316242
ILMN_2293594	LAMA3	-0.643400737
ILMN_2132898	SPRN	-0.643459622
ILMN_1727996	BAG4	-0.643605654
ILMN_3238269	NDUFA6	-0.643781724
ILMN_2154654	PTP4A1	-0.643856049
ILMN_1693537	CDY1B	-0.643909553
ILMN_2232036	EFCAB7	-0.644023146
ILMN_2278518	NFATC2IP	-0.644174188
ILMN_1654522	UNC93B1	-0.644616316
ILMN_1776674	SAC3D1	-0.644681378
ILMN_1730441	OR52K2	-0.644818521
ILMN_2136635	ISCA2	-0.645172217
ILMN_1694240	MAP2K1	-0.645185624
ILMN_2057826	PHF3	-0.645270904
ILMN_1671221	GAPVD1	-0.645502181
ILMN_1708432	PLCB1	-0.645611441
ILMN_1907847	DB038222	-0.6457125
ILMN_1680899	TTLL3	-0.645770374
ILMN_1733875	RUNDC1	-0.645793234
ILMN_1675523	PPAP2C	-0.645977244
ILMN_1681882	CETP	-0.64615453
ILMN_2176450	CA3	-0.646190537
ILMN_2413473	GH2	-0.646592681
ILMN_1798712	USP4	-0.646608291

ILMN_1700625	ATP5S	0.446108999
ILMN_1791400	ATE1	0.446090492
ILMN_1854833	CR624291	0.446028781
ILMN_1696027	MPHOSPH8	0.445969936
ILMN_1698996	SLC19A1	0.445889832
ILMN_1735004	C4orf43	0.445766445
ILMN_2389984	RGS9	0.445733381
ILMN_1667081	CCND2	0.445615697
ILMN_1751776	CKAP2L	0.445610909
ILMN_3235647	SIK1	0.445458786
ILMN_2413899	MCM10	0.44545577
ILMN_1811851	PRRX1	0.445440722
ILMN_1705384	PPFIBP1	0.445347389
ILMN_1855278	AW954199	0.445282484
ILMN_1732053	SNRNP70	0.445215904
ILMN_1731223	AMPD3	0.445213557
ILMN_3288218	RPS7	0.445179806
ILMN_1664233	TMC6	0.445178417
ILMN_1787931	PMS2L14	0.445039395
ILMN_1697614	NHP2L1	0.444975732
ILMN_1739812	PRKCG	0.444929715
ILMN_1783161	DAB2IP	0.444697887
ILMN_1773546	MRGPRX3	0.444661828
ILMN_2386179	ZMYND8	0.44452286
ILMN_1767139	NDUFA13	0.444493125
ILMN_1746598	SCNM1	0.444403763
ILMN_1797964	PFAAP1	0.444348651
ILMN_1764082	MBOAT1	0.444300959
ILMN_1677404	RAP2A	0.444164169
ILMN_1689257	MAEA	0.44414427
ILMN_1687510	HRNR	0.44408262
ILMN_1662198	RANGAP1	0.444082329
ILMN_1691731	PARP14	0.443997212
ILMN_1706246	CCT5	0.443985049
ILMN_1804448	MSI2	0.443845107
ILMN_2295518	TRO	0.443734886
ILMN_3242540	CD163L1	0.443725574
ILMN_1652246	NACAD	0.44371602
ILMN_3263469	LOC100240735	0.443704196
ILMN_1690040	TM7SF2	0.443624069
ILMN_1696380	GHRL	0.443623872
ILMN_1696546	FZD1	0.443604317
ILMN_2385278	DCAF4	0.443452588
ILMN_1743646	VASP	0.443437646
ILMN_3245878	SNORA70C	0.443431816
ILMN_1738207	CISH	0.443403942
ILMN_1804451	LEO1	0.443391404
ILMN_3258356	RPSAP52	0.443319566
ILMN_3237574	CHRNA7	0.443294332
ILMN_1678490	RILPL2	0.443272171
ILMN_2094776	CCNL1	0.443235391
ILMN_2328463	IGF2	0.443139288
ILMN_1790162	ZC3H11A	0.443126721
ILMN_3227811	HNRNPA1	0.443056401
ILMN_1714936	CCDC142	0.443022467
ILMN_1655046	NUTF2	0.443013006
ILMN_1743367	FZD4	0.443006374
ILMN_3238196	CYTH4	0.44299515
ILMN_1715680	NEIL2	0.442917073
ILMN_1732343	SIK3	0.442913831
ILMN_1710752	NAPRT1	0.442845509
ILMN_3221156	NBPF14	0.442833948
ILMN_1669227	ADRA1A	0.442744305
ILMN_1687941	RNF4	0.442734846
ILMN_2342835	P2RY14	0.442722114
ILMN_2355776	GIT2	0.442645405
ILMN_2117987	TFDP1	0.44260059
ILMN_1668417	WIPF1	0.442558263
ILMN_2082585	SNAI2	0.442513901
ILMN_1788887	LOC338799	0.442444562
ILMN_1808409	HIF3A	0.442389752
ILMN_2369756	CCDC46	0.442266465
ILMN_3205264	FAM27E3	0.442251606
ILMN_1733998	DHRS9	0.442250772
ILMN_1663631	BANP	0.442173546
ILMN_1692177	TSC22D1	0.44214417
ILMN_3194087	HSPC157	0.442091884
ILMN_1741572	AKAP8	0.442078888
ILMN_1816244	LOC151162	0.442022827
ILMN_1708510	NFATC2IP	0.442016644
ILMN_3238028	RPL17	0.441906938
ILMN_1799243	ANXA13	0.441813246
ILMN_1663015	HIF3A	0.441768804
ILMN_1808356	FAM3A	0.441761938
ILMN_2176755	KIAA0895L	0.441701653
ILMN_3200018	SPDYE2	0.441636666
ILMN_1713156	MSL3	0.441497579
ILMN_1733535	ZNF366	0.441395877
ILMN_1777487	ZNF839	0.441350116
ILMN_1684271	ACBD6	0.441333418
ILMN_1700507	CLASP1	0.441314139
ILMN_1785768	PDE4A	0.441164919
ILMN_1683462	GSS	0.440992053
ILMN_2234709	C12orf60	0.440990876
ILMN_1657237	NUBP2	0.440916318
ILMN_2244108	ITGB1	0.440788214
ILMN_2348808	SNX16	0.44077896
ILMN_1712031	SPDYE2	0.440625588

ILMN_2305416	PCDHGC3	-0.646620775
ILMN_2379835	SUMO1	-0.646757799
ILMN_1807201	FAM104A	-0.646765315
ILMN_1802631	AGA	-0.646824445
ILMN_2119224	KIFAP3	-0.646962251
ILMN_3251567	FBXW2	-0.646963505
ILMN_2196588	C18orf32	-0.647065553
ILMN_1915805	LOC340073	-0.647068276
ILMN_1660754	C13orf25 v_1	-0.647073859
ILMN_1675819	FAM101A	-0.647190681
ILMN_1708004	AY262164	-0.647201297
ILMN_1660260	MCFD2	-0.647290217
ILMN_2205350	NDUFAF4	-0.647357743
ILMN_1706301	RET	-0.647504622
ILMN_1735064	PPP1R3E	-0.647746041
ILMN_1723467	ITGB1	-0.647846304
ILMN_1847133	AW270402	-0.647898601
ILMN_1899105	BC010870	-0.647999766
ILMN_2406656	GATA3	-0.648011243
ILMN_1674366	LHX4	-0.648019667
ILMN_3245281	NPS	-0.648046232
ILMN_1656399	TCEAL8	-0.648121074
ILMN_2148668	RCBTB2	-0.648259107
ILMN_1751760	BCAS2	-0.648322638
ILMN_1682544	CLUL1	-0.648585881
ILMN_1700232	FBXO30	-0.648665629
ILMN_1728168	SLMO2	-0.648732915
ILMN_1702899	PLEKHA7	-0.648904214
ILMN_1744995	RGSL1	-0.649076208
ILMN_3237473	CBWD1	-0.649166451
ILMN_1664071	TNNT2	-0.649224802
ILMN_1845347	BM930795	-0.649226774
ILMN_2395932	UNC45A	-0.649231004
ILMN_1725246	COG8	-0.649296213
ILMN_1735151	EIF5A2	-0.649419081
ILMN_1787180	J05235	-0.649496409
ILMN_1718060	OTUD7A	-0.649608774
ILMN_1795270	MYCL1	-0.649759969
ILMN_2170595	RRM2B	-0.649979497
ILMN_1778242	CALM1	-0.650062635
ILMN_3249546	RASA4	-0.650289762
ILMN_1782439	CNN3	-0.65044538
ILMN_1839962	LOC728724	-0.650549575
ILMN_1911605	FRS2	-0.650643639
ILMN_1894367	LOC401463	-0.650666557
ILMN_1784287	TGFBR3	-0.650683808
ILMN_2324574	RALGAPA1	-0.650929387
ILMN_1874018	LOC729175	-0.650945519
ILMN_3308330	MIR545	-0.65107717
ILMN_1733287	HYDIN	-0.651274595
ILMN_1810200	ARPC2	-0.65131128
ILMN_1772286	OCIAD2	-0.651572483
ILMN_2341548	MYO5B	-0.651612856
ILMN_1716832	ACSF3	-0.65180209
ILMN_1704452	BCL9	-0.651893506
ILMN_1765615	OR1B1	-0.65221666
ILMN_1676523	CCDC91	-0.652433181
ILMN_1655151	ABCA17P	-0.652525454
ILMN_2219556	ISCA1	-0.652635952
ILMN_1751615	COQ10B	-0.652765894
ILMN_1710962	TMEM97	-0.652841414
ILMN_1813343	OR10R3P	-0.652963855
ILMN_1688220	SYNPO2	-0.652969505
ILMN_1721569	C12orf40	-0.653059267
ILMN_1734550	SLC9A3	-0.65334639
ILMN_2225511	BRMS1L	-0.653400854
ILMN_1786034	GPRC5B	-0.653718061
ILMN_1730611	RTN4	-0.654062256
ILMN_1870085	DB279316	-0.654093725
ILMN_1676321	FLJ43826	-0.654233651
ILMN_1697301	ST8SIA3	-0.654267817
ILMN_1672009	DKFZp762B162	-0.654634146
ILMN_2333708	STAG2	-0.654889318
ILMN_1796138	BMX	-0.655009611
ILMN_3266764	LOC728448	-0.655086005
ILMN_3237022	SCGB1C1	-0.65509598
ILMN_1770361	BC040551	-0.655311882
ILMN_1744647	CAND1	-0.655348449
ILMN_1682233	ESCO1	-0.655352854
ILMN_1683212	SLC25A32	-0.655368888
ILMN_1762327	DHRS12	-0.655411763
ILMN_2119555	TMTC3	-0.655413447
ILMN_1667768	EFCAB8	-0.655484059
ILMN_2045911	FBXO28	-0.655484421
ILMN_1815107	MATR3	-0.655485828
ILMN_1720311	SLC25A46	-0.655521299
ILMN_2046683	NAP1L2	-0.655610485
ILMN_1726752	APTX	-0.655709891
ILMN_3206111	HNRNPKP3	-0.655722998
ILMN_1681123	KIAA0562	-0.655788015
ILMN_1684923	ANP32E	-0.655859688
ILMN_1718265	ATG5	-0.655872845
ILMN_1675916	NA	-0.656107519
ILMN_3273620	KLHL33	-0.656165684
ILMN_1711516	ATP6V1A	-0.656269027
ILMN_2244653	CDC14A	-0.656329586
ILMN_1710652	MTMR8	-0.656514715
ILMN_1657697	SAR1A	-0.656594395

ILMN_1654392	KHNYN	0.440224554
ILMN_3255903	NA	0.440175238
ILMN_3262250	LOC100125556	0.440172949
ILMN_3310558	MIR302D	0.440060665
ILMN_1765557	OLFML2B	0.440050877
ILMN_1690224	SPDYE2	0.440030493
ILMN_1719649	TMEM63A	0.440013863
ILMN_2302075	MSI2	0.439971445
ILMN_1807894	SLC7A8	0.439853247
ILMN_3256325	CYB561D1	0.439850332
ILMN_1696532	RBBP5	0.439725027
ILMN_1725696	ATXN3	0.439692427
ILMN_1743663	POMT1	0.439686973
ILMN_1726114	SLC45A3	0.439670757
ILMN_1702979	SHOX2	0.439624582
ILMN_1787212	CDKN1A	0.43945351
ILMN_2362122	AP3M1	0.439383619
ILMN_1678564	DVL1	0.439372327
ILMN_1662256	PARP6	0.439353632
ILMN_2175124	DAZ2	0.43926708
ILMN_3235808	POM121C	0.439235158
ILMN_1716816	TMEM87A	0.439187407
ILMN_2415162	sept-01	0.439119079
ILMN_1779841	PPP2R1B	0.439079752
ILMN_2289775	HAX1	0.439049081
ILMN_1811890	RGS10	0.439036719
ILMN_2329263	NFIC	0.439005169
ILMN_1748245	CTNNA1	0.438970568
ILMN_1809313	CASP8	0.438961739
ILMN_2414533	ARMCX6	0.438939234
ILMN_1706643	COL6A3	0.438923492
ILMN_2279635	EIF4G2	0.438888083
ILMN_1677262	RPL21	0.438859261
ILMN_1747935	GOLGB1	0.438839642
ILMN_1803856	DKFZP586I1420	0.438759813
ILMN_2367233	ZNF654	0.43867078
ILMN_1689431	APCDD1L	0.438669017
ILMN_2103977	OR51F2	0.438666134
ILMN_1652024	CSNK1G3	0.438628524
ILMN_1813796	TMEM169	0.43862191
ILMN_1762769	SPDYE7P	0.438581941
ILMN_1701169	HP1BP3	0.438547286
ILMN_1670611	MFSD6L	0.438430649
ILMN_3192359	NCRNA00204	0.438425612
ILMN_2173611	MT1E	0.438416909
ILMN_1686392	NBAS	0.43833067
ILMN_1674609	CLTB	0.438271746
ILMN_1660277	PDXDC2	0.438222689
ILMN_1771148	CNTN4	0.438148636
ILMN_1809267	CLCC1	0.437955602
ILMN_1699723	RARB	0.437903933
ILMN_1751439	EMX2	0.43773401
ILMN_2319326	ADARB1	0.4377032
ILMN_2331735	AP2B1	0.43769008
ILMN_1791726	TUBB3	0.437652829
ILMN_1722838	MRPL46	0.437649415
ILMN_1692591	RNF183	0.437523098
ILMN_2352090	GPRC5C	0.437354825
ILMN_1709367	FAM186B	0.437208162
ILMN_1742390	FRMD1	0.437196498
ILMN_1798256	UPP1	0.437090569
ILMN_1663415	RPL7	0.436944984
ILMN_2203271	FPR3	0.436939236
ILMN_2359800	MS4A6A	0.436886111
ILMN_1705627	USP2	0.436836354
ILMN_2193233	MZB1	0.436764389
ILMN_1662731	COL4A3	0.436626702
ILMN_2068104	TFPI2	0.436408897
ILMN_3187328	IP6K1	0.436302289
ILMN_2357260	CPSF4	0.43630146
ILMN_3240901	ZBTB7C	0.436243977
ILMN_1734479	ACCN3	0.43622507
ILMN_1654612	ZNF589	0.436178473
ILMN_3301168	UBE2V1	0.436048534
ILMN_1753111	NAMPT	0.435942128
ILMN_2086077	JUNB	0.435924093
ILMN_1801984	VHL	0.435896425
ILMN_1715647	VANGL2	0.435873355
ILMN_2330243	NUDT1	0.435823009
ILMN_3306672	PATL2	0.435702003
ILMN_2364828	OGT	0.435655514
ILMN_1755589	DIP2B	0.435650969
ILMN_1682762	IDH3G	0.435566579
ILMN_2394576	FBXW11	0.435493356
ILMN_1763568	ZDHHC16	0.435187936
ILMN_1659054	CHKB	0.435158046
ILMN_2329171	SPDYE1	0.434964171
ILMN_1764109	C1R	0.434874313
ILMN_3237926	CCNJL	0.43484403
ILMN_1770963	NAALADL1	0.434804246
ILMN_1696375	TTC31	0.434788273
ILMN_2381945	FGA	0.434639725
ILMN_2406439	SPTBN4	0.434615872
ILMN_1756942	SP3	0.43457192
ILMN_1697348	WBSCR22	0.434570014
ILMN_1772722	MRPS33	0.434562378
ILMN_1896406	ZXDA	0.434534957
ILMN_2121437	NCL	0.434428499

ILMN_3247013	EGOT	-0.656690324
ILMN_2133125	LRRC43	-0.656898742
ILMN_1688886	GPC5	-0.656933907
ILMN_1803094	PDGFD	-0.657103774
ILMN_1688276	TRIM29	-0.657259131
ILMN_3242508	C19orf71	-0.657296685
ILMN_1658800	BRPF3	-0.657745527
ILMN_2211546	HAO1	-0.657770836
ILMN_1773306	DDX26B	-0.657816531
ILMN_1765460	AMIGO1	-0.657863429
ILMN_3271942	FLJ45872	-0.658158093
ILMN_1831214	AW592010	-0.658318947
ILMN_1773906	NCOA4	-0.658341063
ILMN_1904052	CCDC122	-0.658945611
ILMN_1752281	DNAJC13	-0.659043673
ILMN_1654430	AK129671	-0.659375117
ILMN_1732514	GALK2	-0.659399827
ILMN_2061979	H3F3C	-0.659462143
ILMN_1657349	EPHA6	-0.65949153
ILMN_1672717	C10orf57	-0.659494963
ILMN_1673042	RBBP6	-0.659610452
ILMN_1695731	TUBG1	-0.659646211
ILMN_1717119	BF510953	-0.659742853
ILMN_1767393	GRIA3	-0.65991863
ILMN_1723043	NAPSB	-0.659944239
ILMN_3251365	PGGT1B	-0.660227302
ILMN_1754912	GLE1	-0.660652634
ILMN_2184708	LIN7C	-0.660715773
ILMN_1805823	BANF2	-0.660773209
ILMN_2351029	MTMR2	-0.661056964
ILMN_1717714	CDKN2A	-0.661132221
ILMN_1694311	NUDT6	-0.661167005
ILMN_2124155	ATP11B	-0.661236999
ILMN_2388112	CR1	-0.661260804
ILMN_1736972	GKAP1	-0.661276899
ILMN_1796074	C18orf56	-0.661278123
ILMN_1651681	BC013377	-0.661317836
ILMN_1803218	GPR139	-0.661439116
ILMN_2069912	MAGEA8	-0.662050181
ILMN_3241127	LOC100128703	-0.662199607
ILMN_1834180	LOC440602	-0.662299129
ILMN_1685399	AK302783	-0.662375447
ILMN_1813434	ATP10A	-0.662378241
ILMN_1801403	DCUN1D4	-0.662408057
ILMN_1708131	BHLHB9	-0.662502593
ILMN_1813256	CRIPT	-0.662671597
ILMN_1814007	NA	-0.66292132
ILMN_1656842	FLJ44054	-0.662960479
ILMN_3236325	LOC441869	-0.66310342
ILMN_3251491	UQCRB	-0.663256139
ILMN_1744795	TBL1X	-0.663557599
ILMN_1743187	C6orf120	-0.663631348
ILMN_3310975	MIR539	-0.664103731
ILMN_3236428	TMEM170B	-0.664139176
ILMN_2229170	MED7	-0.664250009
ILMN_1764850	HPCAL1	-0.664333123
ILMN_2367215	PRCP	-0.664600546
ILMN_1689908	ANKRD13A	-0.664693996
ILMN_1803852	CSAG2	-0.66472857
ILMN_1702384	ZNF706	-0.664909074
ILMN_1756693	ARHGAP17	-0.664915225
ILMN_1801223	WTIP	-0.66500742
ILMN_2230902	CTNNA1	-0.665954135
ILMN_1872769	BX103063	-0.665998374
ILMN_1813100	KIAA1244	-0.666069327
ILMN_1882652	BG697942	-0.666100365
ILMN_1886642	BX096476	-0.666194273
ILMN_1827736	PDK3	-0.6662371
ILMN_1771223	PADI2	-0.666286207
ILMN_1690993	NEUROG2	-0.66638383
ILMN_2094294	NR2E1	-0.666550822
ILMN_1773307	NAP1L5	-0.666597291
ILMN_2168758	RNF11	-0.666693063
ILMN_1855116	AA992213	-0.666917008
ILMN_1713125	SPANXD	-0.666920906
ILMN_3261111	IP6K3	-0.667293454
ILMN_1802628	PPAPDC2	-0.667713938
ILMN_1794074	MXI1	-0.667839131
ILMN_2073010	TMEM203	-0.667926816
ILMN_1807994	PCNP	-0.668057904
ILMN_3276971	DVL1	-0.668083772
ILMN_1656672	MYLK	-0.668168166
ILMN_2179778	PHLDB2	-0.668192085
ILMN_1846630	BE439556	-0.668246515
ILMN_1718657	FLJ43950	-0.668339449
ILMN_3279282	NA	-0.668494523
ILMN_2245087	MFAP2	-0.668526302
ILMN_1680082	Y08699	-0.668591317
ILMN_3268993	ABCB10	-0.668591411
ILMN_1736441	PDXP	-0.668597579
ILMN_1715396	SNORD56B	-0.66889209
ILMN_1739674	PDE4DIP	-0.669017333
ILMN_1744068	ELP3	-0.669056903
ILMN_1751425	ERMP1	-0.669113732
ILMN_1737398	PTPLAD1	-0.669182477
ILMN_1696031	C15orf21	-0.669248532
ILMN_1781803	FIZ1	-0.669491498
ILMN_1696003	GNAI3	-0.669605104

ILMN_2288175	AGPAT3	0.43437662
ILMN_1735148	SEC24B	0.434310923
ILMN_1689189	ZBTB3	0.434266143
ILMN_1666713	LYPLA1	0.434132765
ILMN_1708604	CCZ1	0.434003557
ILMN_1704861	MYO1A	0.433964836
ILMN_2212714	TMEM39B	0.433958269
ILMN_1660786	HIPK1	0.433919818
ILMN_1669703	TNK2	0.4339176
ILMN_1747613	KCNC3	0.433851187
ILMN_1686929	GPATCH3	0.433850179
ILMN_1798706	GPR183	0.433842787
ILMN_1697971	TRIM38	0.433834737
ILMN_1797861	IL6ST	0.43380788
ILMN_1672128	ATF4	0.433726749
ILMN_1772482	SLC6A10P	0.433717862
ILMN_1808590	GUCY1A3	0.433661075
ILMN_2377056	BBS7	0.433642744
ILMN_2110857	GALR3	0.433584581
ILMN_1804279	PTPRC	0.43331679
ILMN_1679133	SERPINB1	0.433283934
ILMN_3300198	MMP24	0.433283012
ILMN_1690894	GRP94c	0.433236971
ILMN_2057573	ESYT2	0.433170109
ILMN_1740762	GRB7	0.433118119
ILMN_1694466	DHRSX	0.433106761
ILMN_2189007	AGAP4	0.433087965
ILMN_2410194	SPRED3	0.433052988
ILMN_2280135	P4HA2	0.432900334
ILMN_2069446	SAFB2	0.432890079
ILMN_1805652	PPP2R2D	0.43286389
ILMN_1720048	CCL2	0.432817283
ILMN_1789418	GSTT2B	0.432783371
ILMN_1696713	POLA2	0.432769396
ILMN_2245305	ABHD12	0.432764267
ILMN_2077905	PTGFRN	0.432719796
ILMN_1910180	AK024680	0.432691709
ILMN_1708328	RAB11FIP3	0.432682035
ILMN_1669928	ARHGEF16	0.432670354
ILMN_1695026	AK311573	0.432619498
ILMN_1712913	UNC5A	0.432607425
ILMN_1798379	NTM	0.43251787
ILMN_1726204	SCRG1	0.432504394
ILMN_1725510	DHCR24	0.432462076
ILMN_1671966	NA	0.432459936
ILMN_1673880	EFEMP1	0.432440115
ILMN_1728914	PRUNE	0.432306326
ILMN_1802411	PELO	0.432272101
ILMN_1729269	LOC285074	0.432240079
ILMN_2194577	PLVAP	0.432230354
ILMN_1850991	AK091342	0.432224299
ILMN_1792689	HIST1H2AC	0.432191951
ILMN_1748488	TXNL4B	0.432163057
ILMN_1746929	DNAH17	0.432148964
ILMN_1758214	RARS2	0.43211007
ILMN_1680037	FAM65A	0.432031443
ILMN_1734653	FNDC1	0.431921249
ILMN_1754507	IRF4	0.431917951
ILMN_1659537	MED8	0.431851124
ILMN_1789614	TPT1	0.431817979
ILMN_1805225	LPCAT3	0.43180842
ILMN_2159384	SBDSP	0.431789272
ILMN_2145501	ANGPTL1	0.431780666
ILMN_1718132	ECHS1	0.431758603
ILMN_1732074	HNRNPA1	0.431754254
ILMN_3302456	RPSA	0.431741028
ILMN_1730765	DUSP22	0.43172798
ILMN_1669394	PIG8	0.431524864
ILMN_3203126	LOC100131662	0.431481047
ILMN_3209883	LOC653075	0.431447536
ILMN_1733684	HIST1H1T	0.431298373
ILMN_1682176	CLEC3B	0.431163222
ILMN_1806165	HSPA7	0.431138143
ILMN_1780108	PTPN22	0.431077434
ILMN_1775879	KRTAP19-2	0.431017751
ILMN_2363361	SFXN4	0.430983861
ILMN_1808152	SLC41A3	0.430941975
ILMN_2290679	TIRAP	0.430870254
ILMN_1807945	ANP32A	0.430866367
ILMN_1772036	STEAP4	0.430865885
ILMN_2365549	BRPF1	0.43084609
ILMN_1793543	C1orf51	0.430792495
ILMN_1782142	S100A13	0.430745606
ILMN_2329114	COLQ	0.430393275
ILMN_1756326	CKS2	0.43021899
ILMN_1702229	CECR6	0.430192023
ILMN_1657760	SYT17	0.430091608
ILMN_2329735	ECM1	0.430051952
ILMN_1706455	TTC5	0.429915951
ILMN_1787260	LCE3B	0.429915094
ILMN_2130838	UTP11L	0.429900476
ILMN_3203801	ZNF532	0.429893996
ILMN_1671288	ODF2	0.429802048
ILMN_1808566	TMEM180	0.429786512
ILMN_1788356	AKIP1	0.429766083
ILMN_1813207	MRPS9	0.4297378
ILMN_1798728	C19orf2	0.429611536
ILMN_1719204	PRPF31	0.429579224

ILMN_1719468	EPM2A	-0.669693474
ILMN_1702009	SV2A	-0.669712116
ILMN_1782994	WBP11P1	-0.669877811
ILMN_1800590	BBS1	-0.669985685
ILMN_1884277	AA913455	-0.670210112
ILMN_2104106	XPR1	-0.670254933
ILMN_1763878	CDADC1	-0.670293059
ILMN_1786444	LPL	-0.670453356
ILMN_1671427	FBXW4	-0.670533115
ILMN_1764522	LMBR1	-0.670590874
ILMN_1717757	CALML4	-0.670669995
ILMN_3238832	RBMY3AP	-0.670755773
ILMN_1777479	CDYL2	-0.670915899
ILMN_1773763	MTA2	-0.67121535
ILMN_2357577	PRKAA1	-0.671347696
ILMN_3235133	MYO5B	-0.671555702
ILMN_1809148	OR6C74	-0.671658129
ILMN_1907218	BM973226	-0.671692532
ILMN_1759023	WFS1	-0.6721559
ILMN_1747678	LOC647946	-0.672168018
ILMN_1712088	CLYBL	-0.672198788
ILMN_1838398	DA268131	-0.672326956
ILMN_1719753	VGLL1	-0.672356367
ILMN_1813664	HIRA	-0.672695595
ILMN_1902209	BU743228	-0.672738224
ILMN_1728965	C11orf57	-0.672749478
ILMN_2195914	GGH	-0.672874327
ILMN_1791030	NEUROG1	-0.672925258
ILMN_1715991	SDPR	-0.672936093
ILMN_1707506	YTHDC1	-0.673502453
ILMN_1656165	USP9X	-0.673636272
ILMN_1665877	RNF149	-0.67402033
ILMN_1651967	TP53I3	-0.674043187
ILMN_1673980	LOC643669	-0.674103256
ILMN_1809139	AHCTF1	-0.674192854
ILMN_1774083	TRIAP1	-0.674243599
ILMN_1730209	OR5A1	-0.674375136
ILMN_1662578	C1GALT1	-0.674650889
ILMN_1821199	CR744726	-0.674828199
ILMN_1695601	AK098200	-0.674925375
ILMN_1652164	UNC93B1	-0.675258658
ILMN_1660730	SLC7A3	-0.675328208
ILMN_1808824	NEBL	-0.675471394
ILMN_2116811	C7orf70	-0.675556088
ILMN_1683905	C19orf21	-0.675565219
ILMN_3248795	ACTR3B	-0.675578244
ILMN_3242011	MOBKL1B	-0.675621047
ILMN_1764861	ISOC1	-0.675754221
ILMN_1774028	MTFR1	-0.675933473
ILMN_3243846	FOLR4	-0.67596258
ILMN_1665717	EIF2S3	-0.67611692
ILMN_1734602	SRRM2	-0.676173188
ILMN_3227060	AMD1	-0.676218398
ILMN_1761721	VPS35	-0.676223536
ILMN_2177732	RANBP6	-0.676302903
ILMN_1811997	RNF115	-0.676755356
ILMN_2180682	MTPN	-0.676906667
ILMN_1696041	CDC42	-0.677189401
ILMN_1691143	RAB18	-0.677541597
ILMN_1797181	LOC93622	-0.677602447
ILMN_1762033	NAPEPLD	-0.677693461
ILMN_3246169	PRR20A	-0.677812687
ILMN_3308808	MIR130A	-0.677911863
ILMN_1815949	SLC24A4	-0.677937647
ILMN_3187535	FAM30A	-0.677995749
ILMN_1793670	LOC100128571	-0.678393362
ILMN_1910645	NA	-0.678461108
ILMN_1744614	PLEKHG6	-0.678624607
ILMN_1757781	NS4ATP2	-0.678858082
ILMN_1739521	NLGN1	-0.67890816
ILMN_1707985	FOXR1	-0.678931664
ILMN_2084182	RPL10	-0.679139925
ILMN_1774955	IMAA	-0.679141014
ILMN_1748427	ZNF239	-0.679275366
ILMN_2093810	POMP	-0.679372522
ILMN_1678217	DMRTC1B	-0.679658188
ILMN_2171102	MSL2	-0.679867377
ILMN_2295698	AFG3L1	-0.679923017
ILMN_1679389	ZFP57	-0.680128135
ILMN_3307659	SFT2D2	-0.680333745
ILMN_1668378	SFT2D2	-0.680356006
ILMN_1702858	ADHFE1	-0.681276007
ILMN_2180582	PNPLA8	-0.681313431
ILMN_2144088	FDFT1	-0.681314013
ILMN_1799614	PNPLA6	-0.681351909
ILMN_1686791	KCNK4	-0.681386108
ILMN_1703346	CHRNE	-0.681504557
ILMN_2064898	CCDC56	-0.68162354
ILMN_1731616	DCLK1	-0.681647579
ILMN_2363668	YIF1B	-0.681662143
ILMN_2046315	GABARAPL2	-0.681692619
ILMN_2350301	PRB1	-0.68171256
ILMN_1782015	FCRLB	-0.681794382
ILMN_3209098	FAM71E2	-0.68180305
ILMN_3237329	FNIP2	-0.682352465
ILMN_3310643	VTRNA2-1	-0.682454978
ILMN_1855996	AI985753	-0.682460077
ILMN_1761113	GNL2	-0.682868313

ILMN_1821887	AK023756	0.429525734
ILMN_1736165	MLL3	0.429500129
ILMN_1791052	FKBPL	0.429442625
ILMN_1695812	KRT72	0.429421822
ILMN_2342271	BCL11A	0.429219435
ILMN_1726181	C19orf52	0.429203773
ILMN_3242105	LYPD1	0.429172726
ILMN_1672554	C17orf81	0.429014162
ILMN_1734783	ZMYND11	0.428890148
ILMN_1780898	PRKCH	0.428829825
ILMN_3249835	NA	0.428775158
ILMN_1784040	NAPSA	0.428759825
ILMN_1695041	GATA6	0.428743879
ILMN_1746846	TTLL4	0.428577042
ILMN_1773126	FANK1	0.428566835
ILMN_3184067	LOC440905	0.428550658
ILMN_3310391	MIR1204	0.428524449
ILMN_1792495	AHNAK	0.428414686
ILMN_2327090	MAEA	0.428333776
ILMN_1763487	CTLA4	0.428094919
ILMN_1696126	STX18	0.428034136
ILMN_2268618	PHF20L1	0.427969647
ILMN_2095820	UTP14A	0.427909425
ILMN_3225406	LOC728640	0.427844639
ILMN_1655766	ANKRD34B	0.427841398
ILMN_2362982	ARID4B	0.427694344
ILMN_2371152	KLC4	0.427606901
ILMN_1702385	LILRA4	0.427575659
ILMN_1726281	ANAPC11	0.427508263
ILMN_2401714	MS4A1	0.427367545
ILMN_1740572	TCN2	0.427329636
ILMN_1698463	ILF3	0.427132896
ILMN_1670796	EXOSC10	0.427062157
ILMN_1728180	LUC7L3	0.42700646
ILMN_1712347	SRSF6	0.426970194
ILMN_1747775	STX2	0.426857701
ILMN_1770697	HNRNPK	0.426762186
ILMN_1712718	BPNT1	0.426702968
ILMN_1694547	IFFO1	0.426684449
ILMN_1815124	CHCHD2	0.426415955
ILMN_3187763	DB453801	0.426352813
ILMN_1789400	FOXD2	0.426199633
ILMN_1705763	SCT	0.426195293
ILMN_1675543	PRM1	0.426177405
ILMN_1751956	MGST3	0.426133021
ILMN_1654586	RASA3	0.426078175
ILMN_1791770	SMARCC2	0.426001812
ILMN_1746673	sept-03	0.425987289
ILMN_2410262	MTMR14	0.425961192
ILMN_1747586	TG	0.425779146
ILMN_1697268	EMILIN2	0.425755903
ILMN_1673108	AP2A1	0.425747463
ILMN_1791057	IFNAR2	0.425688029
ILMN_2059744	IL7	0.425548896
ILMN_3249742	PXMP4	0.425433504
ILMN_1775823	POFUT2	0.425388569
ILMN_1815121	PLAGL1	0.425358318
ILMN_2182198	ICT1	0.425256396
ILMN_1710747	AF208111	0.425249463
ILMN_3236367	IFFO2	0.425235125
ILMN_1790704	SPAG16	0.425226718
ILMN_1802771	C22orf15	0.425170517
ILMN_1658426	WNT10A	0.425104378
ILMN_1691080	OR6B1	0.425077431
ILMN_2231003	MAGEA12	0.425039344
ILMN_1754468	RGS9	0.425001089
ILMN_1810267	DNHD1	0.424976423
ILMN_1657446	NTPCR	0.424972516
ILMN_1766085	REL	0.424957194
ILMN_1786893	RBM5	0.424921957
ILMN_1799198	OTUB2	0.424913869
ILMN_3241005	MFSD6L	0.424866074
ILMN_1715635	ATP6V0E1	0.424826699
ILMN_2078226	MAGEH1	0.424822216
ILMN_1756705	CHTF18	0.424720744
ILMN_1718013	CPSF3L	0.424660905
ILMN_1775761	TSR1	0.42465112
ILMN_2190414	ZNF83	0.424621161
ILMN_1705676	MRAP	0.424594236
ILMN_1811364	CD556913	0.424552496
ILMN_1895548	AK021726	0.424529988
ILMN_1752520	SLFN11	0.424425403
ILMN_2415235	CSNK1E	0.42441913
ILMN_1691409	SEC31A	0.424304105
ILMN_1698608	FOLR1	0.424283405
ILMN_1656129	SLC39A10	0.424184824
ILMN_1702806	PDCL3	0.424162752
ILMN_1750711	MYO19	0.424142668
ILMN_1730630	CXorf56	0.424070845
ILMN_2386040	MYO19	0.424044905
ILMN_1807710	HINT1	0.424022121
ILMN_1703864	SFTA2	0.423915952
ILMN_1815480	NAT8	0.423911383
ILMN_1796430	PSMD3	0.423775637
ILMN_1661755	FAM129B	0.423744518
ILMN_1799600	STARD8	0.423736402
ILMN_1685709	TMEM125	0.423613982
ILMN_2374728	ARPP21	0.423414968

ILMN_1886816	AI334326	-0.683074807
ILMN_1737965	ELOVL4	-0.683241734
ILMN_1655163	STK24	-0.683467453
ILMN_1719163	ZNF557	-0.683828726
ILMN_3289779	NA	-0.683833281
ILMN_2334385	NCKAP5	-0.683897646
ILMN_2315789	PTPRD	-0.684170915
ILMN_2358278	RSRC2	-0.684523651
ILMN_3204686	SLC16A6	-0.684625025
ILMN_1762631	TSC2	-0.68478796
ILMN_2219681	RBP2	-0.684913043
ILMN_2081988	LANCL1	-0.68513847
ILMN_1716019	RHBDL3	-0.685288997
ILMN_1733390	LARP1B	-0.685387817
ILMN_1784737	S1PR4	-0.685399641
ILMN_2253065	H2AFJ	-0.685407672
ILMN_1760110	AK023936	-0.685504259
ILMN_2058512	PSMA2	-0.6856397
ILMN_1709824	PCDHGC3	-0.685767999
ILMN_1893033	DB307015	-0.685769409
ILMN_1738346	EFHA1	-0.685810418
ILMN_1674759	LPCAT4	-0.685885891
ILMN_1678965	FABP1	-0.685917448
ILMN_1660963	CCIN	-0.686023255
ILMN_1762871	RNASE13	-0.68613425
ILMN_1792671	C12orf29	-0.686534743
ILMN_2363744	PACRG	-0.686737085
ILMN_3299330	LOC643719	-0.687218923
ILMN_3244860	LRRC16B	-0.687252203
ILMN_1840036	BM672267	-0.687347276
ILMN_1761820	EDARADD	-0.687689278
ILMN_1717337	MARCH7	-0.687832642
ILMN_1697175	TAF8	-0.687938202
ILMN_1804662	NRG4	-0.688177034
ILMN_1691047	EPS8L2	-0.688263115
ILMN_1775901	PHF5A	-0.688351494
ILMN_1795936	C3orf36	-0.68861231
ILMN_1782954	UBE2K	-0.688658503
ILMN_3250909	AL137495	-0.6886827
ILMN_1668507	DDAH1	-0.688721132
ILMN_2404539	C20orf30	-0.688855398
ILMN_2288915	DNAJB14	-0.688977121
ILMN_1705568	CR596696	-0.689058341
ILMN_3310845	MIR511-2	-0.689228637
ILMN_3211463	HNRNPA1	-0.689412585
ILMN_1837348	AI272125	-0.689463332
ILMN_2283915	MAP2	-0.689542666
ILMN_1684045	CDCA4	-0.689748708
ILMN_2121147	KIAA0825	-0.689769978
ILMN_1665856	CA10	-0.689958743
ILMN_1692517	SORD	-0.689960278
ILMN_1669572	RNF126	-0.690223138
ILMN_3237529	DEFB108B	-0.690338825
ILMN_3305614	ZNF812	-0.690694449
ILMN_1676763	PIP5K1A	-0.690905834
ILMN_2381730	EGFL7	-0.691014879
ILMN_3307648	CS	-0.691055662
ILMN_3274596	EIF4A2	-0.691135013
ILMN_1661264	SHMT2	-0.691294584
ILMN_1878728	DB222718	-0.691703619
ILMN_1847602	HAR1A	-0.691858802
ILMN_1701958	DLG1	-0.691923792
ILMN_1726647	RPS26	-0.691936275
ILMN_2363634	ADHFE1	-0.691938087
ILMN_1688294	LOC100132966	-0.691951368
ILMN_2183610	SERAC1	-0.692209954
ILMN_3271179	NEDD8	-0.692553643
ILMN_1906098	BU182441	-0.692726094
ILMN_3241783	FLJ00326	-0.693059224
ILMN_1690327	CLCN5	-0.693107396
ILMN_1810712	ARHGEF12	-0.69314091
ILMN_1664371	HIATL1	-0.69321508
ILMN_1756723	DPP7	-0.693395567
ILMN_1667263	BMS1P5	-0.693463345
ILMN_1729650	PEX7	-0.69356199
ILMN_2123550	OR8B12	-0.694645482
ILMN_2054607	CYP4V2	-0.694746136
ILMN_1712137	LOC100128219	-0.694787314
ILMN_1763404	SRP9	-0.694951908
ILMN_1849186	ZNF704	-0.694963474
ILMN_2180866	RPS26P11	-0.6950003
ILMN_1766770	SNX31	-0.69500546
ILMN_1767184	BC153822	-0.695052348
ILMN_1768357	RTN1	-0.695132443
ILMN_3235825	UBE2QP2	-0.695166276
ILMN_1737988	PRNP	-0.695201315
ILMN_1807331	BLID	-0.695234969
ILMN_1671777	FGF13	-0.695295341
ILMN_3249756	DNASE1L1	-0.695300808
ILMN_1712231	SH3YL1	-0.695494389
ILMN_1700159	NIPSNAP3A	-0.695559489
ILMN_1722849	UNC13A	-0.695732245
ILMN_2129715	COG3	-0.695740458
ILMN_1677723	ANGPT1	-0.695848284
ILMN_1742324	C1orf9	-0.695878093
ILMN_1819783	FNIP2	-0.695878458
ILMN_1695965	FAM36A	-0.695951049
ILMN_1780861	METRNL	-0.69616279

ILMN_2396813	BABAM1	0.423411978
ILMN_1660900	SNORA7B	0.423389792
ILMN_1806488	CCDC108	0.423313059
ILMN_2382724	METTL23	0.423165518
ILMN_2305544	DBI	0.423094462
ILMN_1744628	FDX1L	0.422997613
ILMN_3237534	UBE2MP1	0.422976331
ILMN_1764891	ZNF384	0.42278448
ILMN_1698159	ANKRD6	0.422777355
ILMN_1671885	MLF2	0.422624127
ILMN_2400854	ICA1L	0.422547761
ILMN_3242035	DA731816	0.422527232
ILMN_1757074	GNG10	0.422499786
ILMN_2066020	TCEB1	0.422458635
ILMN_3239548	STAG3L2	0.422359065
ILMN_1709747	EXOG	0.422240573
ILMN_3245310	CCDC94	0.422122546
ILMN_1743219	CA11	0.422060996
ILMN_2117330	NDUFB2	0.422050481
ILMN_1738742	PLAT	0.42201905
ILMN_3244117	STMN3	0.422005815
ILMN_3294119	BX096650	0.421926664
ILMN_1699085	AJ312026	0.421892408
ILMN_1704529	PPIA	0.421846841
ILMN_3194638	FAM176A	0.421806523
ILMN_1724406	INO80E	0.42170312
ILMN_2175265	TMEM11	0.421694482
ILMN_1671969	UGP2	0.421692191
ILMN_3203765	FLJ00317	0.421605596
ILMN_1732489	SLC10A7	0.421559762
ILMN_2197225	ANKHD1	0.421467871
ILMN_1761275	FUT6	0.421440714
ILMN_1655684	SARS2	0.421433455
ILMN_2091792	ENTPD6	0.421336675
ILMN_1751444	NCAPG	0.421229867
ILMN_1728236	SLFN12	0.421132095
ILMN_2306033	PRAME	0.421086984
ILMN_1654690	CECR5	0.421021833
ILMN_1711904	MXD3	0.420851571
ILMN_1721449	VSX2	0.420800712
ILMN_1656045	CELF6	0.420663241
ILMN_1717234	CAST	0.420631603
ILMN_1703787	ATP4B	0.420536626
ILMN_1755041	PPA2	0.420507586
ILMN_1706571	SLC35D2	0.420485446
ILMN_1905053	BX538256	0.420483815
ILMN_1679727	CLK1	0.420412957
ILMN_1736704	DIXDC1	0.42031107
ILMN_2283195	APOL3	0.420266085
ILMN_3253471	KLHL29	0.420254552
ILMN_1782167	RPL32	0.420249879
ILMN_1728121	OR10R2	0.420230346
ILMN_1703471	ATF6	0.420192718
ILMN_1740345	THYN1	0.42017883
ILMN_1782596	CCL25	0.420090772
ILMN_1700432	ITPKB	0.420063222
ILMN_1740957	NVL	0.420017823
ILMN_2370622	F8	0.420006141
ILMN_1652992	PRDM2	0.419953061
ILMN_1808905	AKAP3	0.419929266
ILMN_1742427	POLR1D	0.419899932
ILMN_1752247	AKAP13	0.41985644
ILMN_3245066	DENND4B	0.419784383
ILMN_1795243	RPS4X	0.419781358
ILMN_1657381	RASSF6	0.419520253
ILMN_1715069	TANK	0.419425942
ILMN_1657423	SPG21	0.419317819
ILMN_3233032	NA	0.419188456
ILMN_2297854	P2RY2	0.41913796
ILMN_1809483	HSD17B14	0.419081671
ILMN_1747451	PLCXD1	0.419016463
ILMN_1652082	ELF4	0.419008364
ILMN_2174341	GPR83	0.418992472
ILMN_1696004	LRRK1	0.418965992
ILMN_1786720	PROM1	0.418784355
ILMN_1733956	IARS	0.418760909
ILMN_1712657	BPTF	0.418747199
ILMN_2112580	FCGR3A	0.418676857
ILMN_1741841	KIAA1429	0.418648285
ILMN_1671686	EPB49	0.418616572
ILMN_1664772	ATP2B4	0.418584947
ILMN_1670087	FBXO43	0.418562678
ILMN_2386849	DSE	0.418545903
ILMN_1799814	SNRNP40	0.418515712
ILMN_1808898	NPR2	0.418511639
ILMN_1699545	PCSK7	0.418463006
ILMN_1666832	RAP1GAP2	0.418454231
ILMN_1792012	OTUB2	0.418421932
ILMN_2363621	RBBP8	0.418385841
ILMN_2048478	SLC22A3	0.418212718
ILMN_3211707	LOC100306975	0.418154831
ILMN_1669352	LY6G6C	0.418073834
ILMN_1756877	C14orf179	0.417963806
ILMN_3225767	ZNF534	0.417862936
ILMN_2178855	GPAM	0.417791298
ILMN_2412139	CABYR	0.417755182
ILMN_1680435	NPEPPS	0.417738751
ILMN_3244969	ZNF48	0.417722157

ILMN_2168166	ASPN	-0.696288811
ILMN_1733807	IGF2BP1	-0.696347478
ILMN_2067607	TMEM106B	-0.696741595
ILMN_1697405	PLCL1	-0.697098418
ILMN_2230016	HIGD1A	-0.697159906
ILMN_1685886	MAPK4	-0.697195147
ILMN_1750636	RPS26	-0.69742275
ILMN_1765072	KRT85	-0.697494679
ILMN_1727798	MOSPD1	-0.697727896
ILMN_2332558	ARL5A	-0.69800835
ILMN_2207504	LEP	-0.69827379
ILMN_1722538	DEFB123	-0.698364608
ILMN_3307954	L3MBTL4	-0.698657465
ILMN_1754655	TTLL5	-0.698683504
ILMN_1758400	GRIN3A	-0.698790966
ILMN_1717982	BZW1	-0.698841052
ILMN_1703174	FREM2	-0.699350823
ILMN_3180456	NA	-0.699364588
ILMN_2156936	SCCPDH	-0.699408528
ILMN_2053679	ACADM	-0.699531257
ILMN_2320850	UBE2D3	-0.699544725
ILMN_1774715	OR5P3	-0.699596792
ILMN_2387395	SCN3A	-0.700189528
ILMN_1891725	BX111675	-0.700670644
ILMN_1785107	NXT2	-0.700732515
ILMN_2183938	LEMD3	-0.700887968
ILMN_1730791	HGC6.4	-0.700951123
ILMN_3308592	MIR98	-0.700953836
ILMN_1741695	COL12A1	-0.701021526
ILMN_1738987	HS2ST1	-0.701322805
ILMN_1654402	IL18BP	-0.701924306
ILMN_2054938	RBAK	-0.701931017
ILMN_1770466	ATP5G3	-0.701969236
ILMN_1735680	TMEM30A	-0.702542973
ILMN_2404085	CLIP1	-0.702615566
ILMN_1739429	HS6ST2	-0.702817509
ILMN_1689070	COQ7	-0.702930688
ILMN_1900347	AI701700	-0.702933506
ILMN_2123525	SYCP3	-0.702961248
ILMN_1685703	ACOX2	-0.702969101
ILMN_2371379	ACLY	-0.703058574
ILMN_1780924	SLC43A1	-0.703527371
ILMN_1800540	CD55	-0.703720728
ILMN_2202637	CRY1	-0.704287809
ILMN_1796337	CXCL17	-0.705039247
ILMN_2096083	PLEKHA5	-0.705869632
ILMN_2401933	ATP2B1	-0.705949306
ILMN_2143148	TM2D1	-0.706436286
ILMN_2136971	FABP3	-0.706659132
ILMN_1812902	CYYR1	-0.706925723
ILMN_1729482	KLK15	-0.707008924
ILMN_1697557	NA	-0.70712293
ILMN_1808769	C1orf97	-0.707236207
ILMN_3182171	FGGY	-0.707624024
ILMN_2388517	MTERFD3	-0.708332665
ILMN_3229083	SNX5	-0.708875483
ILMN_1744534	LYRM5	-0.709181245
ILMN_1839190	BC073918	-0.70943278
ILMN_1656413	ZMPSTE24	-0.709525636
ILMN_1769150	THAP5	-0.709585068
ILMN_1800871	RAB6A	-0.709997633
ILMN_2099798	LYPLA1	-0.710047506
ILMN_1669617	GRB10	-0.710188541
ILMN_1683755	SOX13	-0.710320732
ILMN_3308435	MIR1914	-0.711223463
ILMN_1704943	GPN3	-0.711272573
ILMN_1701624	AK098200	-0.711481605
ILMN_1661363	AF064105	-0.711516126
ILMN_1748650	MRPL45	-0.711555231
ILMN_2400583	USP9X	-0.711599849
ILMN_1776858	DUS4L	-0.711780331
ILMN_1689129	OPRM1	-0.711991224
ILMN_1718001	PHKB	-0.712057135
ILMN_3203672	POM121-like 1	-0.712082089
ILMN_2047856	EFHA2	-0.712182466
ILMN_1746664	WSB2	-0.71284246
ILMN_1756162	EXOSC8	-0.712924187
ILMN_1723521	AJAP1	-0.712929927
ILMN_3245287	SCN5A	-0.71300755
ILMN_2342841	AFTPH	-0.71332124
ILMN_1787830	BASE	-0.71351296
ILMN_3193266	NA	-0.713525307
ILMN_2176768	SEPHS1	-0.713693415
ILMN_2221006	RAD21	-0.71381718
ILMN_2388397	LYPD1	-0.713862517
ILMN_1772925	UBE2NL	-0.713862619
ILMN_3248747	LOC647983	-0.713965608
ILMN_1723874	MRPS6	-0.713976326
ILMN_3244733	AL565221	-0.71407273
ILMN_3239173	WAPAL	-0.714616566
ILMN_3249963	PCBP2	-0.71487689
ILMN_2245180	BUB3	-0.714971426
ILMN_2223922	AGTPBP1	-0.715025908
ILMN_2135232	SRSF3	-0.715149031
ILMN_3201715	NA	-0.715189813
ILMN_2151739	CAT	-0.715310352
ILMN_2184049	COX7B	-0.715497143
ILMN_3235817	LOC100132941	-0.715499999

ILMN_2278636	CUX1	0.417637061
ILMN_1691702	ZNF775	0.417556161
ILMN_2309228	GPS1	0.417525115
ILMN_1723048	GJC2	0.417520591
ILMN_1656900	SULT1A1	0.417497254
ILMN_1675427	NBPF15	0.41748947
ILMN_1698677	C4orf27	0.417369379
ILMN_1747105	CAPS2	0.417215353
ILMN_3279585	N95477	0.41718019
ILMN_1768940	COL15A1	0.417123744
ILMN_2383058	SIRPG	0.417024812
ILMN_1812096	CADM4	0.416995372
ILMN_1786211	HERC1	0.416985532
ILMN_1796682	PARP3	0.416928033
ILMN_1787981	MFAP2	0.416908547
ILMN_2337492	RCE1	0.416883334
ILMN_1697880	PLD2	0.416878358
ILMN_1747303	DDX39A	0.416822843
ILMN_1763344	ADCYAP1	0.416820344
ILMN_1700028	C9orf156	0.416718809
ILMN_1675577	TRMT61A	0.416489355
ILMN_1721008	DUT	0.416402577
ILMN_1679978	WBSCR16	0.416373547
ILMN_2144401	GLB1L2	0.416371024
ILMN_1740234	GSTO2	0.416369981
ILMN_1683243	VPS45	0.416343589
ILMN_1670318	RIOK1	0.416275145
ILMN_1773163	C19orf34	0.416184956
ILMN_1664978	TJP2	0.416161935
ILMN_1769195	KIAA0378	0.416125666
ILMN_1700604	RBM14	0.416111989
ILMN_2295918	PCNXL2	0.416001649
ILMN_2211235	TEX101	0.415898981
ILMN_1704055	NOP16	0.415840099
ILMN_1800267	FAM13A	0.415828766
ILMN_1659936	PPP1R15A	0.41575022
ILMN_3253901	HAVCR1	0.415721819
ILMN_1658626	RREB1	0.415691718
ILMN_1890263	BX101040	0.415678866
ILMN_1665932	ACCN1	0.415649923
ILMN_2169801	TPSAB1	0.415619865
ILMN_3240187	TMEM111	0.415577929
ILMN_1726783	RNASEH1	0.415568526
ILMN_1708580	PDZK1IP1	0.415564411
ILMN_1660816	P2RX2	0.415490739
ILMN_2074801	RGL3	0.41548742
ILMN_3240475	SDHA	0.415483151
ILMN_1681081	AGPAT2	0.415465325
ILMN_1653367	TAF12	0.415317256
ILMN_1727462	ARSE	0.415270016
ILMN_1699091	C14orf118	0.415065575
ILMN_1711462	MNS1	0.415009971
ILMN_1770329	RABGGTA	0.415005538
ILMN_1782567	GUCY1B3	0.415000165
ILMN_1803647	FAM162A	0.414892903
ILMN_1743021	CAMKK2	0.414859874
ILMN_2087692	CYBRD1	0.414716146
ILMN_1675024	BEND5	0.414587304
ILMN_2212909	MELK	0.414509938
ILMN_1671191	UQCRC1	0.414473787
ILMN_1803464	PHTF1	0.41438651
ILMN_1682503	NPFF	0.414370776
ILMN_1691611	HNRNPA1L2	0.414349057
ILMN_1781966	OSBP2	0.414282648
ILMN_3242299	CLDN9	0.414261715
ILMN_1812256	GANC	0.414198465
ILMN_1799278	CD101	0.414178911
ILMN_1684461	CADPS2	0.414157619
ILMN_1731418	SP110	0.41413833
ILMN_1701424	LAMC2	0.414070737
ILMN_1721614	ZNF890P	0.41404992
ILMN_1703108	UBE2L6	0.414006776
ILMN_1678032	NR1H2	0.413988727
ILMN_1685109	POLR3D	0.413937699
ILMN_1797184	PKN3	0.413915391
ILMN_1810992	CAD	0.413908138
ILMN_3307134	FLJ40448	0.413906448
ILMN_1665331	AMT	0.413849001
ILMN_2334112	WHSC1	0.413817385
ILMN_1737005	C19orf61	0.41380485
ILMN_1832720	BM806922	0.413713449
ILMN_1887170	BQ184605	0.413660415
ILMN_1781236	LOC554223	0.413556877
ILMN_3229733	SIGMAR1	0.413454571
ILMN_1752199	LHPP	0.413433605
ILMN_3307782	FBXL18	0.413425782
ILMN_2233513	LRRIQ4	0.413381111
ILMN_1661439	FLOT1	0.413378973
ILMN_2376667	POFUT2	0.4133605
ILMN_1705876	NAP1L1	0.413337337
ILMN_1706645	MB21D1	0.413325048
ILMN_1754894	C1orf162	0.413264001
ILMN_1655614	DSP	0.413176835
ILMN_2137312	KCNJ5	0.413148378
ILMN_1703100	TLK2	0.413111617
ILMN_1792990	ZNF202	0.41308349
ILMN_1711383	STK4	0.413038793
ILMN_1806010	CUL9	0.413030991

ILMN_1698233	RWDD2B	-0.715939319
ILMN_2150019	SUCLA2	-0.716100146
ILMN_1735402	DCAF5	-0.716623769
ILMN_1790052	MIFR-1	-0.71669329
ILMN_1663054	TXNDC11	-0.716873594
ILMN_1764500	C3orf10	-0.716933894
ILMN_1789653	PBLD	-0.717102672
ILMN_3306730	RBM47	-0.717355741
ILMN_1712430	ATP5G1	-0.717686877
ILMN_3289262	PNRC2	-0.718146581
ILMN_1735798	OSBPL3	-0.718211588
ILMN_2252136	YWHAE	-0.71827112
ILMN_2297887	SEZ6L2	-0.718459575
ILMN_3237907	EIF2B3	-0.718526611
ILMN_1830259	AI630821	-0.718869591
ILMN_1769091	PRCP	-0.71931834
ILMN_2202790	MCFD2	-0.719327928
ILMN_1658499	SYT13	-0.719345358
ILMN_1677607	SC5DL	-0.719346796
ILMN_2079285	ATP5L	-0.719667563
ILMN_1805147	ACSM5	-0.71973177
ILMN_1695092	WRB	-0.719763406
ILMN_2128087	NAA30	-0.71989914
ILMN_1794875	AGPAT9	-0.720018536
ILMN_3298348	LINC00273	-0.720026859
ILMN_2085922	WRB	-0.720154286
ILMN_3240736	TRAPPC10	-0.720293747
ILMN_1790105	SUMO1	-0.72056687
ILMN_1709570	IMMP1L	-0.720628009
ILMN_1743040	LOC283104	-0.720924533
ILMN_2141941	TOR1AIP1	-0.721026826
ILMN_1752810	LARP6	-0.721158871
ILMN_1838238	DA717721	-0.721340334
ILMN_2268068	MAPKAP1	-0.721509481
ILMN_1770663	KRT24	-0.722015888
ILMN_3246534	TSG1	-0.722244116
ILMN_1804569	GAS2	-0.722248036
ILMN_1750901	GNAT1	-0.722375595
ILMN_2175114	KCNS3	-0.723065245
ILMN_1802246	KLK10	-0.723076529
ILMN_1763539	IER3IP1	-0.723335782
ILMN_2269092	MANEA	-0.723528656
ILMN_1781560	ST3GAL6	-0.723553457
ILMN_2322747	ARHGAP5	-0.724183569
ILMN_3246214	B3GNT1	-0.724201153
ILMN_2227368	SELT	-0.724294676
ILMN_2204297	DKFZp564F1378	-0.724363137
ILMN_1779374	AMMECR1	-0.724588233
ILMN_3234570	CECR4	-0.724642307
ILMN_1772814	NLK	-0.72475673
ILMN_1753868	POTEC	-0.724911392
ILMN_2147503	ALG13	-0.725390812
ILMN_1658909	OSGEPL1	-0.725705911
ILMN_2372403	ALDH5A1	-0.726012882
ILMN_1869343	D19756	-0.726056184
ILMN_3225886	NOX4	-0.726151816
ILMN_2054053	TMEM67	-0.726502153
ILMN_1836309	MCF2L-AS1	-0.726525807
ILMN_1677384	PTGER3	-0.726730573
ILMN_1773262	ESM1	-0.7268161
ILMN_1700950	DQ896551	-0.726970201
ILMN_1735360	SDAD1	-0.727162961
ILMN_1732808	TOX3	-0.728374817
ILMN_1691789	SMNDC1	-0.729041786
ILMN_1768913	TSN	-0.729268559
ILMN_1769684	LCE1E	-0.729528721
ILMN_1675669	IBTK	-0.729542271
ILMN_3209193	RPS26	-0.729904267
ILMN_2115073	OR1D5	-0.73019223
ILMN_1907620	DB299679	-0.730228041
ILMN_1778890	LRR1	-0.730288954
ILMN_1720617	OR2T6	-0.730794224
ILMN_1660999	WBP11P1	-0.730835024
ILMN_2167709	NIPSNAP3A	-0.731120061
ILMN_2099586	CCDC28B	-0.731159031
ILMN_1659749	DSP	-0.731183689
ILMN_2175465	RSL24D1	-0.73156253
ILMN_1769083	GSTA3	-0.73190857
ILMN_3179275	H03055	-0.731975071
ILMN_1651699	EPS8	-0.73198172
ILMN_1838655	CR614803	-0.732132723
ILMN_1815010	RNF141	-0.732436771
ILMN_2156953	ZFAND6	-0.732452259
ILMN_2155452	HPR	-0.73266241
ILMN_2367191	PSMF1	-0.732779297
ILMN_1882485	CF552129	-0.733140718
ILMN_1728467	GHITM	-0.733368883
ILMN_1691106	CDV3	-0.733460819
ILMN_1740263	P2RX3	-0.733993511
ILMN_3270866	TACC2	-0.734138797
ILMN_2226415	MZT1	-0.734739599
ILMN_1652128	LMBRD1	-0.734908864
ILMN_1729509	C1orf43	-0.735156635
ILMN_2310589	DIABLO	-0.735165084
ILMN_1733453	RHOQ	-0.735645874
ILMN_1812262	DDR1	-0.735704423
ILMN_3237655	SLC29A4	-0.736105651
ILMN_2081162	SH3BGRL	-0.736605401

ILMN_2251279	WDR45	0.413001807
ILMN_1728272	C14orf177	0.412915146
ILMN_1691293	RNF185	0.412863108
ILMN_1702517	CDON	0.412795335
ILMN_1791069	ZNF513	0.412742697
ILMN_2125010	SKAP2	0.412723112
ILMN_1814282	AEN	0.4126517
ILMN_1660577	ATP5G2	0.412629073
ILMN_2180624	TMCO6	0.412512026
ILMN_1764980	SERPINA1	0.412489395
ILMN_2329927	ABCG1	0.41241825
ILMN_2117508	CTHRC1	0.412364501
ILMN_2199947	REV3L	0.412363704
ILMN_1773117	BCOR	0.412338705
ILMN_1771385	GBP4	0.412276077
ILMN_1711899	ANXA2	0.412250418
ILMN_1780444	ARL3	0.412178211
ILMN_1718734	MLLT6	0.412174743
ILMN_1777906	MAP7D1	0.411929414
ILMN_1730529	CAB39L	0.411923344
ILMN_1722798	PLCD3	0.411851052
ILMN_2181445	BCL2L13	0.411815473
ILMN_3188269	NA	0.411782518
ILMN_1808568	PYCR2	0.411749358
ILMN_1763663	AF086132	0.41162147
ILMN_1756439	SCRN1	0.411545364
ILMN_1693341	SNRPN	0.41151404
ILMN_1763634	PEX14	0.41147032
ILMN_3244216	SCARNA3	0.411376797
ILMN_1734827	MKI67	0.411370534
ILMN_2105573	CCL3L3	0.411298028
ILMN_2133100	LPIN2	0.411288098
ILMN_1779095	CEBPE	0.410948747
ILMN_1665964	GAB2	0.410937671
ILMN_1689734	IL1RN	0.410891498
ILMN_1799139	PLOD2	0.410866597
ILMN_1651373	RHD	0.410863994
ILMN_1773247	ZNF410	0.410731455
ILMN_1773125	ENTPD1	0.410553766
ILMN_2366654	NR5A2	0.410508624
ILMN_1665235	CRTAP	0.410503309
ILMN_1797728	HMGCS1	0.41038131
ILMN_1741180	HEXDC	0.410328221
ILMN_2383305	GPATCH4	0.410293886
ILMN_1765520	MTIF2	0.410247201
ILMN_2155228	CRLF3	0.410174422
ILMN_1731048	TLR1	0.410109779
ILMN_1790309	PINX1	0.41010686
ILMN_1909156	SNORD123	0.41009519
ILMN_1745420	PHF19	0.410075817
ILMN_1741755	TRIM29	0.410053217
ILMN_1679483	INTS10	0.410032522
ILMN_1768062	ADK	0.409875245
ILMN_3247018	EIF4A1	0.409817488
ILMN_1682812	C21orf33	0.409784381
ILMN_3246401	AIF1L	0.409733551
ILMN_1733538	RGS10	0.409647598
ILMN_1815292	RPL7	0.409442097
ILMN_1806432	NT5C	0.409432724
ILMN_2363586	SDCBP	0.40937849
ILMN_1724540	ALX1	0.409310257
ILMN_1757262	ZBTB5	0.409248006
ILMN_1752967	DHPS	0.409186474
ILMN_1813503	SEMA6C	0.40914921
ILMN_1685124	TCTN1	0.409092819
ILMN_1747159	OGDH	0.409020755
ILMN_1668526	GVIN1	0.408987173
ILMN_1778168	ELMO2	0.408855353
ILMN_1679798	TLR9	0.408809964
ILMN_3287493	PPIA	0.408760439
ILMN_1671045	RPS3A	0.408725844
ILMN_1810239	BMF	0.408636805
ILMN_2133187	PRSS53	0.408524471
ILMN_1791127	CDH12	0.408487957
ILMN_2217661	SREBF2	0.408428057
ILMN_1806463	CTAGE6P	0.408341038
ILMN_2401964	CDC42EP1	0.408313238
ILMN_2145670	TNC	0.408273732
ILMN_1709471	CNTN4	0.408259626
ILMN_1787265	ZNF503	0.408251986
ILMN_1746924	ZNF610	0.408198692
ILMN_1773388	C13orf18	0.40819197
ILMN_1762080	JMY	0.408164044
ILMN_1796106	MGAT4B	0.408146464
ILMN_2140342	CCDC57	0.408048747
ILMN_3274814	LOC100133286	0.407966211
ILMN_3234124	C17orf101	0.407952894
ILMN_2395389	PSMC4	0.407942487
ILMN_3304203	CLEC2D	0.407924469
ILMN_3308678	MIR412	0.407919659
ILMN_1697631	PRDM16	0.407892923
ILMN_2233454	SPTLC3	0.407771334
ILMN_3235404	SNORA57	0.407766818
ILMN_1728711	RGS3	0.407742429
ILMN_2415144	SP110	0.407670521
ILMN_2214910	EPHB4	0.407625365
ILMN_1653514	RBBP4	0.407581306
ILMN_3248948	LOC100272216	0.407576364

ILMN_2158587	EEF1E1	-0.737038237
ILMN_1698894	OR5AS1	-0.737258149
ILMN_2100815	TMEM9B	-0.737418484
ILMN_1767643	CTRB2	-0.737426734
ILMN_2371825	AGL	-0.737756983
ILMN_2316918	PANK1	-0.73779279
ILMN_1831258	AK094576	-0.73794875
ILMN_1741564	DCTN4	-0.738644266
ILMN_2148497	C20orf107	-0.738989764
ILMN_2323366	SDHC	-0.739007502
ILMN_1664718	CYP51A1	-0.739125888
ILMN_3242494	FLJ41484	-0.739263731
ILMN_3234746	NA	-0.739461375
ILMN_2380181	MAG	-0.739653908
ILMN_1777366	TTC35	-0.739662006
ILMN_1694106	GPD1L	-0.739747042
ILMN_1786343	MUC5B	-0.739747968
ILMN_2377829	NANOS1	-0.739792715
ILMN_1886050	CR622587	-0.73983979
ILMN_2043809	PFKM	-0.740001867
ILMN_1661491	SH3GL2	-0.740011054
ILMN_1802808	FP15737	-0.740097295
ILMN_1662168	SP3	-0.740613424
ILMN_1706005	GALT	-0.740749874
ILMN_1685781	C14orf142	-0.740824685
ILMN_1877039	BU858876	-0.740899534
ILMN_1705049	TMEM67	-0.741356143
ILMN_1788961	PPP2R2A	-0.74160591
ILMN_1722643	DPP6	-0.741630398
ILMN_1803824	ZDHHC9	-0.741845395
ILMN_1712943	CCK	-0.742137193
ILMN_2383077	SLC39A7	-0.742402856
ILMN_3293159	BC015443	-0.742518859
ILMN_1660043	UBXN11	-0.742576659
ILMN_1685854	C5orf53	-0.742602851
ILMN_1724504	SETD3	-0.742768863
ILMN_2358628	ADK	-0.742769936
ILMN_1776601	OR4F5	-0.742954376
ILMN_1820572	BX112399	-0.743135035
ILMN_1655190	LOC100506085	-0.743289202
ILMN_1741431	AOC2	-0.743517723
ILMN_1735445	SLC7A9	-0.743609294
ILMN_3212469	LOC401561	-0.74392639
ILMN_1810120	SYT7	-0.743981994
ILMN_3248857	IL20	-0.74401187
ILMN_3251436	DENND4C	-0.744445317
ILMN_1842533	AL117577	-0.744597251
ILMN_3289679	NA	-0.744804031
ILMN_2406304	PDZRN3	-0.744916125
ILMN_1651708	RESP18	-0.745043962
ILMN_1730229	CGNL1	-0.745793418
ILMN_1763269	TNKS2	-0.745798153
ILMN_1904578	ODZ1	-0.74590827
ILMN_1682495	FOXP1	-0.7463425
ILMN_1765204	ST13	-0.746848289
ILMN_2079803	LSM14A	-0.746988561
ILMN_1753286	MYO19	-0.747224524
ILMN_1777917	DSCR10	-0.747343724
ILMN_2188959	ACOT2	-0.747398355
ILMN_3272424	HNRNPA1	-0.747675338
ILMN_2093980	TMEM38B	-0.748306448
ILMN_1681154	ZBTB39	-0.748459788
ILMN_1685796	CSDE1	-0.748564037
ILMN_1711919	SCYL2	-0.748857512
ILMN_2408001	RFWD2	-0.748954291
ILMN_1681249	KIF21A	-0.748961535
ILMN_1916137	BF513551	-0.749921818
ILMN_2209027	RPS26	-0.750121149
ILMN_2059452	SLC12A2	-0.750134815
ILMN_1764091	R3HDM2	-0.750316321
ILMN_1656869	LGALS9C	-0.750392198
ILMN_2145044	FAM173B	-0.750996216
ILMN_3201658	VDAC1	-0.751802861
ILMN_1769299	MTMR11	-0.752179059
ILMN_1707434	FP15737	-0.752670961
ILMN_1786361	RDM1	-0.752780814
ILMN_3256693	AK130189	-0.752926813
ILMN_1658015	MBNL2	-0.753143956
ILMN_1745991	GNRH1	-0.753259883
ILMN_1686906	TP53INP2	-0.753614105
ILMN_1892506	CACNA1C	-0.753747847
ILMN_2102515	PGAM4	-0.754053821
ILMN_3241354	FLJ00317	-0.754420752
ILMN_2076250	GPBP1L1	-0.75449109
ILMN_1705066	BTBD11	-0.754604535
ILMN_1745079	TRIM2	-0.7546998
ILMN_1684585	ACSL1	-0.754759493
ILMN_3280269	FLJ00317	-0.75552146
ILMN_1911500	DA455367	-0.755934796
ILMN_1750262	BC132949	-0.756531771
ILMN_3293146	C20orf30	-0.756629652
ILMN_1762993	PIGV	-0.756688358
ILMN_1814173	SMARCA4	-0.756958143
ILMN_1739586	FEZ2	-0.757033392
ILMN_1878670	BC068609	-0.757132079
ILMN_1658940	DDX25	-0.757134992
ILMN_1778611	GBAS	-0.757245114
ILMN_1723743	ROM1	-0.757620689

ILMN_1676318	BAZ2A	0.40751374
ILMN_1656886	LIN37	0.407509012
ILMN_1661554	DIAPH1	0.407324486
ILMN_2255256	MARVELD3	0.407248768
ILMN_1708950	ASPHD1	0.407043869
ILMN_1678052	C19orf24	0.407043765
ILMN_3287239	FABP5	0.406970233
ILMN_2280960	DEPDC7	0.406960845
ILMN_3199438	X69637	0.40692756
ILMN_1694070	FAM114A1	0.406892126
ILMN_1760049	SERGEF	0.406877202
ILMN_2299843	ATP5S	0.406832225
ILMN_3307738	FSD2	0.406666175
ILMN_1711208	CELSR2	0.406657608
ILMN_2377109	LCK	0.406640414
ILMN_1797804	BACE1	0.406605837
ILMN_1764239	PMPCA	0.406595626
ILMN_3244348	TAF1D	0.406562126
ILMN_1744914	FUCA2	0.406483114
ILMN_2318638	TGIF1	0.40647714
ILMN_1704500	STAP2	0.406423411
ILMN_1664525	P2RY13	0.406296434
ILMN_1697054	LRRN4CL	0.406263565
ILMN_1688938	KCNRG	0.405997197
ILMN_1673172	IFT52	0.405956181
ILMN_1712684	FAM20C	0.405926667
ILMN_1716195	HIST1H2BG	0.405900585
ILMN_1696243	FLJ23152	0.405884847
ILMN_1775743	BTG1	0.405825512
ILMN_2155025	ZNF681	0.405824456
ILMN_1807359	CLEC11A	0.405732511
ILMN_1682226	CLDN15	0.405704547
ILMN_1760556	BC041843	0.405686255
ILMN_1702866	RP11-206L10.16-(	0.405437389
ILMN_1658504	CHKA	0.405389534
ILMN_3281195	SLC16A6	0.405385052
ILMN_1713751	ADAM19	0.40533434
ILMN_1746138	RLTPR	0.405328433
ILMN_2044027	C20orf191	0.405131856
ILMN_1751289	CHTF8	0.405043161
ILMN_1727577	GLI2	0.405032249
ILMN_3219197	HNRNPA1	0.404992504
ILMN_1852384	ELKS	0.404979368
ILMN_1719661	SEPX1	0.404966497
ILMN_1735502	FAM181B	0.40492402
ILMN_1655093	ING5	0.404892267
ILMN_1789535	DHDDS	0.404771405
ILMN_2067032	TRAF3IP1	0.404603678
ILMN_1733254	WDR93	0.404589808
ILMN_1802690	GULP1	0.404561844
ILMN_1737631	PAQR6	0.404498646
ILMN_1777564	MAD2L1	0.404457821
ILMN_2133316	GIMAP7	0.40444558
ILMN_1673933	NPIP	0.40436311
ILMN_1664367	COQ6	0.404356219
ILMN_1703906	HJURP	0.404313141
ILMN_1658830	C10orf26	0.404293254
ILMN_1710734	GZMK	0.404269335
ILMN_1723018	ZNF117	0.404194553
ILMN_1670304	FAM156A	0.404126289
ILMN_1693060	VEGFA	0.404018015
ILMN_2168276	C15orf56	0.403953186
ILMN_1690907	CCR6	0.403907631
ILMN_2364521	AXL	0.403702165
ILMN_1799280	BDH1	0.403676193
ILMN_1803143	SYVN1	0.403640521
ILMN_1690638	RPS3A	0.403569387
ILMN_1759423	ZKSCAN5	0.403503513
ILMN_1772644	EML3	0.403489393
ILMN_2083333	PMS2L5	0.403427895
ILMN_1766370	PHLDB3	0.403349958
ILMN_1757350	CTNNB1	0.403230567
ILMN_1796508	TOP1MT	0.403108708
ILMN_1676470	TCF7	0.403070695
ILMN_1708833	APCS	0.403064865
ILMN_2287147	UBTF	0.403017829
ILMN_1898124	C7orf40	0.402979932
ILMN_1692200	LOC134466	0.402978106
ILMN_3243069	CDK11A	0.402824749
ILMN_2206722	FER1L4	0.402749127
ILMN_2198516	ARRDC3	0.402699702
ILMN_1675737	PCDHA@	0.402674873
ILMN_1765791	C9orf79	0.402646931
ILMN_1688070	WBSCR20C	0.402587165
ILMN_1669335	RUNX1	0.40252987
ILMN_3242753	NBPF1	0.402498499
ILMN_1684210	NIPAL3	0.402490138
ILMN_3223826	SPNS2	0.402460189
ILMN_1676241	BCOR	0.402435806
ILMN_2394276	DST	0.402391859
ILMN_1702738	KLC3	0.40232499
ILMN_1702691	TNFAIP3	0.402300265
ILMN_2213834	PRO0628	0.402257102
ILMN_1655913	NUCB2	0.402210954
ILMN_2296037	PTPN2	0.402085127
ILMN_1677318	MARCH8	0.402035108
ILMN_2228710	PDCD5	0.401963496
ILMN_1672660	MBP	0.401959967

ILMN_1791925	NFAM1	-0.75780269
ILMN_1728907	SCAMP1	-0.758158603
ILMN_1690689	AK027883	-0.7584085
ILMN_1742837	DISC1	-0.758650352
ILMN_1719985	FEM1A	-0.758895213
ILMN_2380999	RECQL	-0.759172209
ILMN_1809245	PITPNB	-0.759244805
ILMN_1675365	LARP4	-0.759512819
ILMN_3192801	NA	-0.759545447
ILMN_1707475	UBE2E2	-0.75970032
ILMN_2193936	HRG	-0.75990535
ILMN_1773742	DNAJB9	-0.760292452
ILMN_2172221	SUMO1P3	-0.760880029
ILMN_2222992	ETF1	-0.761297443
ILMN_1669484	WDR6	-0.7616216
ILMN_1781039	VPS26A	-0.761975523
ILMN_2406815	LRRC17	-0.762027455
ILMN_1697704	KRTAP12-2	-0.762044289
ILMN_1802649	C11orf58	-0.762565395
ILMN_1790036	C20orf202	-0.762883069
ILMN_1660732	PPP2R2B	-0.762988266
ILMN_1684850	PRKAR2B	-0.763044452
ILMN_2285375	SORD	-0.763123994
ILMN_1749317	C1orf222	-0.76403293
ILMN_2327276	STAU1	-0.764392346
ILMN_2163306	FAM120A	-0.764635795
ILMN_2223010	VBP1	-0.76478172
ILMN_1776493	MTUS1	-0.764939009
ILMN_2221408	ARFGEF1	-0.765175691
ILMN_1764380	GLTP	-0.766416198
ILMN_2369104	TRAPPC6B	-0.766684695
ILMN_2095840	MYST3	-0.766861299
ILMN_1657682	GFM1	-0.768245576
ILMN_1756550	NUP62CL	-0.768390815
ILMN_2230579	RABGEF1	-0.76869827
ILMN_1797925	MOCS2	-0.768725467
ILMN_1710026	RNF133	-0.769056897
ILMN_1812600	NA	-0.769174988
ILMN_1660635	LACTB2	-0.769759989
ILMN_1762330	C1D	-0.76986368
ILMN_2151441	FAM103A1	-0.770111319
ILMN_1769024	FAM41C	-0.770290993
ILMN_1656285	METTL7A	-0.771005151
ILMN_3249343	FIGNL2	-0.771006361
ILMN_1874016	NA	-0.771536905
ILMN_2134888	TUBE1	-0.772265903
ILMN_3308788	SNORD50A	-0.77238174
ILMN_1651604	MARK4	-0.773117892
ILMN_1754441	LRRC10B	-0.773916435
ILMN_3244449	SNORD9	-0.774215193
ILMN_2171384	CXCL5	-0.774607546
ILMN_1674021	LOC401321	-0.775449933
ILMN_3309114	KRTAP20-3	-0.776023619
ILMN_2134039	ACN9	-0.776184095
ILMN_2337941	COPS8	-0.776457742
ILMN_1732216	NARS	-0.776962162
ILMN_1694923	PTPN9	-0.777146774
ILMN_1736054	SUB1	-0.777187172
ILMN_1751061	C16orf54	-0.77787703
ILMN_1756779	CLTC	-0.779508245
ILMN_1678522	RPS26	-0.77975789
ILMN_2227248	SLAMF9	-0.779886362
ILMN_1692511	TMEM106C	-0.781010492
ILMN_1785218	MTCH2	-0.78107202
ILMN_1759973	NDUFA5	-0.781445489
ILMN_2215194	GCLC	-0.782258516
ILMN_1865013	CN291296	-0.782347743
ILMN_1774661	SNRPB	-0.78265398
ILMN_1691959	AKAP2	-0.782849796
ILMN_2313434	TCP1	-0.784643322
ILMN_1765401	RRN3	-0.785794801
ILMN_2359287	ITGA6	-0.786322301
ILMN_2227266	TMEM35	-0.786451422
ILMN_3192034	NA	-0.786556061
ILMN_2198239	HGD	-0.786728237
ILMN_1809259	HRASLS2	-0.786742973
ILMN_1807095	MRPS36	-0.7868877
ILMN_1811592	ARHGAP21	-0.787212462
ILMN_2284810	CIDEA	-0.787684481
ILMN_1778173	AK3	-0.788523596
ILMN_1708660	RWDD4	-0.788641301
ILMN_1905546	AK055145	-0.78881069
ILMN_2188722	GLS	-0.788822778
ILMN_2215370	WWP1	-0.789331029
ILMN_1792305	ZNF318	-0.789468737
ILMN_1735552	KIF1B	-0.789951868
ILMN_2394613	C1D	-0.790057664
ILMN_1766103	KCNK18	-0.790345375
ILMN_1730572	HNRPDL	-0.791048926
ILMN_1788778	sept-11	-0.793622055
ILMN_1805251	MGC26484	-0.793870336
ILMN_2246510	TSC1	-0.794241397
ILMN_1759396	NNT	-0.794401038
ILMN_1776334	UXS1	-0.794470701
ILMN_2413264	SOCS4	-0.794549848
ILMN_3270993	AL582488	-0.794620323
ILMN_2344204	PRR13	-0.795313745
ILMN_3187903	AL582488	-0.797465268

ILMN_1714965	NFKB1	0.40187973
ILMN_1662944	PON1	0.401782278
ILMN_2199409	EFHB	0.401736038
ILMN_1658016	ZNF831	0.401688569
ILMN_2095759	OGFRL1	0.401634144
ILMN_1736013	CIB3	0.401588615
ILMN_1712532	CARD9	0.401558257
ILMN_1766363	FLT3	0.401536338
ILMN_1759787	THBD	0.401464972
ILMN_3238203	LOC349114	0.401422607
ILMN_1748189	RTBDN	0.401405078
ILMN_3251303	NR2C2	0.401381739
ILMN_2245676	BTF3	0.401374927
ILMN_1704785	C10orf125	0.401352794
ILMN_1653771	WDR63	0.401337562
ILMN_2405642	DHDDS	0.401311587
ILMN_1773935	TMEM165	0.401284349
ILMN_1717639	SIK1	0.401187472
ILMN_2217601	ANXA9	0.40114599
ILMN_1657993	ADNP	0.401080204
ILMN_1702177	GLO1	0.401075015
ILMN_1676062	DIP2C	0.401061907
ILMN_1898518	GFRA1	0.400969266
ILMN_1804207	MRPL4	0.400958768
ILMN_1657505	HR	0.40091554
ILMN_1678692	MPRIP	0.400886478
ILMN_1726930	C5orf44	0.400794454
ILMN_1753795	SOCS4	0.400718151
ILMN_3249456	LOC96610	0.400610434
ILMN_1663370	CCDC62	0.400555888
ILMN_1652209	BMS1	0.400523639
ILMN_1806304	ENTPD4	0.400491846
ILMN_1729987	SRC	0.400419869
ILMN_1733937	MMD	0.400334907
ILMN_1704985	CYP27A1	0.400250794
ILMN_1700727	KLF6	0.400229946
ILMN_1692198	GTF2IRD1	0.400224843
ILMN_1705442	CMTM3	0.400176466
ILMN_1805395	LTBP3	0.400020343

ILMN_2093674	GMFB	-0.798299812
ILMN_3249654	UOX	-0.799724084
ILMN_3200717	LOC643387	-0.799926392
ILMN_1700549	ERLIN2	-0.800184161
ILMN_2221066	VDAC2	-0.800260094
ILMN_1720482	CEND1	-0.802347977
ILMN_1652163	DVL2	-0.802656954
ILMN_1652638	LRRC58	-0.803026807
ILMN_1690389	OR10AD1	-0.803450743
ILMN_1701512	KIAA0391	-0.803583084
ILMN_1908989	CB243773	-0.804864023
ILMN_1775171	WIPF2	-0.804895657
ILMN_1696702	NEO1	-0.805038926
ILMN_2284077	SLC38A8	-0.805180114
ILMN_1718932	MTRR	-0.805629801
ILMN_1709815	PURG	-0.806617311
ILMN_3239912	HSFYP1	-0.806774923
ILMN_3244060	LOC285205	-0.808401089
ILMN_1826572	AK094562	-0.80846176
ILMN_1785389	ABHD2	-0.809609092
ILMN_1829989	LOC220930	-0.810246443
ILMN_3238359	NAV3	-0.810515591
ILMN_2355033	KIAA1147	-0.811172337
ILMN_2259949	BCKDHB	-0.811634979
ILMN_1808379	PLCD4	-0.81383001
ILMN_1718619	TRIM11	-0.817204603
ILMN_1793651	UBE2N	-0.81749882
ILMN_1736954	ZBTB7B	-0.817897689
ILMN_1752283	ITCH	-0.818069413
ILMN_1748251	ABHD2	-0.820269873
ILMN_1827211	U43604	-0.821337609
ILMN_1669103	AK021896	-0.821845257
ILMN_1749244	LYRM1	-0.821885934
ILMN_1750861	PML	-0.82221399
ILMN_3232806	HIGD1A	-0.822356102
ILMN_1693882	TAPT1	-0.823485228
ILMN_2375879	VEGFA	-0.823712042
ILMN_1745343	ZMAT2	-0.825210872
ILMN_1662417	LRPPRC	-0.826663351
ILMN_2331266	NUMB	-0.827710556
ILMN_2387952	FAM134B	-0.831814948
ILMN_1718520	RNLS	-0.835208267
ILMN_2050911	SLC22A4	-0.835792253
ILMN_2183885	C7orf11	-0.836062476
ILMN_1651310	MLLT4	-0.840651749
ILMN_3294965	RRAS2	-0.841742351
ILMN_1662675	ADAM2	-0.842496823
ILMN_2183772	CCDC121	-0.842566886
ILMN_1771061	BRD4	-0.845123465
ILMN_2078074	MUT	-0.851181038
ILMN_1720889	SC4MOL	-0.852501908
ILMN_1787657	CDK14	-0.853745374
ILMN_3229770	SKP1	-0.853831512
ILMN_2163796	RABGGTB	-0.856266308
ILMN_1685974	SLC25A33	-0.859615162
ILMN_1807448	FAM8A1	-0.861852083
ILMN_1687974	ITGA6	-0.863208861
ILMN_1757693	C17orf51	-0.863591907
ILMN_1746426	TOMM70A	-0.869757538
ILMN_1803882	VEGFA	-0.870060495
