# Supplementary material for: Intrarenal activation of adaptive immune effectors is associated with tubular damage and impaired renal function in lupus nephritis
Source: Ann Rheum Dis. 2018 Jul 31;77(12):1782–9. doi: 10.1136/annrheumdis-2018-213485 (PMC6241616; doi:10.1136/annrheumdis-2018-213485)
Supplement: Supplementary file 9 [file annrheumdis-2018-213485supp009.pdf]

Supplementary Table 6 : Transcripts and DAVID pathways correlating with the expression of CD19 in lupus kidney biopsies

Positive correlation

Transcripts

Identifier	Gene Symbol	Correlation coefficient with CD19
ILMN_1782704	CD19	1
ILMN_1670796	EXOSC10	0.702812214
ILMN_1707467	CLCN3	0.691181409
ILMN_1741604	CSAG2	0.661518537
ILMN_1801639	IARS	0.655669978
ILMN_1710738	RC3H2	0.639705115
ILMN_1676649	AQP4	0.636286637
ILMN_1737005	C19orf61	0.621971163
ILMN_2067895	CXCL11	0.611598413
ILMN_1692100	ZNF35	0.603316686
ILMN_2343105	LIPT1	0.600465605
ILMN_2324989	IKBIP	0.597809229
ILMN_1792800	LCN6	0.597717921
ILMN_1762233	EFCAB5	0.596276624
ILMN_1693009	FGL2	0.588673504
ILMN_1654504	CLEC7A	0.584455182
ILMN_2323992	CLEC7A	0.582179211
ILMN_1679428	CHIC2	0.580816001
ILMN_1714709	OLFM1	0.580398138
ILMN_1811650	DUS2L	0.579509835
ILMN_1801124	KIAA1826	0.577368078
ILMN_1675583	SPG7	0.574873335
ILMN_1702861	SGCD	0.573858389
ILMN_1725738	DKFZp686E14208	0.571462044
ILMN_1813836	DARS	0.571423103
ILMN_3178043	BEND7	0.57109928
ILMN_1759369	BOLL	0.567126703
ILMN_1715526	ZDHHC21	0.565537039
ILMN_2264788	PPP2R2B	0.564927962
ILMN_3248521	UBXN8	0.562552263
ILMN_1793476	PRKCDBP	0.562535933
ILMN_1669497	OSBPL10	0.562341021
ILMN_1700610	CLEC7A	0.561024471
ILMN_2370692	ANKZF1	0.560713765
ILMN_1782729	CLECL1	0.559580851
ILMN_2063925	CTNNBL1	0.558651714
ILMN_2228155	OR2T5	0.557469081
ILMN_1739770	MED7	0.552820505
ILMN_3249560	DUSP22	0.551687061
ILMN_2048822	NUDCD2	0.5503595
ILMN_1751141	RGS12	0.550235013
ILMN_1761069	UVRAG	0.549976948
ILMN_1737041	HABP2	0.547818182
ILMN_1662843	CD53	0.547084924
ILMN_1705093	ORC5	0.544483991
ILMN_3237878	LILRA3	0.54378515
ILMN_3247679	SNORA52	0.543084213
ILMN_3310005	MIR876	0.542945963
ILMN_2391912	SEC14L1	0.542677611
ILMN_3187210	LOC100129186	0.541839661
ILMN_1716195	HIST1H2BG	0.534993719
ILMN_1771801	SIRPG	0.534895709
ILMN_1662026	BTK	0.534660854
ILMN_1711005	CDC25A	0.53359152
ILMN_1784655	TLCD1	0.532587096
ILMN_2413808	CD53	0.530488128
ILMN_1660654	CDCA2	0.529068327
ILMN_1677997	MAZ	0.526841235
ILMN_1709795	RAC2	0.526317493
ILMN_1708538	LOC642426	0.526196486
ILMN_1720771	STX11	0.524745353
ILMN_1798061	ZFYVE26	0.52311731
ILMN_2148459	B2M	0.522971476
ILMN_1740903	C7orf49	0.522634271
ILMN_1766916	RPAP3	0.521331799
ILMN_2062832	TRHR	0.520863732
ILMN_1750088	VRK2	0.520027153
ILMN_1746683	GOLGA8E	0.519664193
ILMN_1891368	BF526404	0.518246066
ILMN_1769606	PIWIL4	0.517111048
ILMN_1731349	HOXA13	0.516937368
ILMN_2400663	AKAP9	0.516517228
ILMN_1740655	GRIN2D	0.515347665
ILMN_1690706	SNRPB2	0.514297548
ILMN_2208455	DDHD1	0.513973644
ILMN_1805535	VRK3	0.511952326
ILMN_1782810	ARPM1	0.511765168
ILMN_1685682	PCNA	0.511204224
ILMN_2077550	RACGAP1	0.510994819
ILMN_2188451	HIST1H2AH	0.510085216
ILMN_1731742	TNFRSF13C	0.51000425
ILMN_1729832	EIF4H	0.509377268
ILMN_3239965	IDO1	0.507541614
ILMN_1662427	PTP4A3	0.507278362
ILMN_1665049	SPG11	0.505735806
ILMN_1694956	SCN5A	0.505494813
ILMN_1696004	LRRK1	0.504011077
ILMN_3240594	RNU4ATAC	0.503837937
ILMN_1700380	ATP6V0A2	0.503509227
ILMN_1785955	C2orf60	0.503054238
ILMN_1712608	BFSP1	0.502885678

Pathways

**Annotation Cluster 1**	**Enrichment Score: 1.59**		**Count**	**P_Value**	**Benjamini**
	GOTERM_BP_FAT	positive regulation of response to stimulus	15	3.20E-04	4.20E-01
	GOTERM_BP_FAT	immune response-activating cell surface receptor signaling pathway	6	1.10E-03	4.60E-01
	GOTERM_BP_FAT	immune response-regulating cell surface receptor signaling pathway	6	1.50E-03	4.80E-01
	GOTERM_BP_FAT	immune response-activating signal transduction	6	3.90E-03	6.20E-01
	GOTERM_BP_FAT	antigen receptor-mediated signaling pathway	5	4.20E-03	6.00E-01
	GOTERM_BP_FAT	immune response-regulating signal transduction	6	5.30E-03	6.10E-01
	GOTERM_BP_FAT	positive regulation of immune response	9	9.20E-03	6.30E-01
	GOTERM_BP_FAT	immune system development	13	1.10E-02	6.40E-01
	GOTERM_BP_FAT	B cell receptor signaling pathway	3	1.30E-02	6.70E-01
	GOTERM_BP_FAT	hemopoiesis	11	2.20E-02	7.60E-01
	GOTERM_BP_FAT	leukocyte activation	11	2.50E-02	7.70E-01
	GOTERM_BP_FAT	hemopoietic or lymphoid organ development	11	3.80E-02	8.30E-01
	GOTERM_BP_FAT	activation of immune response	6	4.20E-02	8.30E-01
	GOTERM_BP_FAT	lymphocyte activation	9	4.90E-02	8.60E-01
	GOTERM_BP_FAT	positive regulation of immune system process	10	5.20E-02	8.50E-01
	GOTERM_BP_FAT	cell activation	11	6.60E-02	8.60E-01
	GOTERM_BP_FAT	T cell activation	6	1.10E-01	9.10E-01
	GOTERM_BP_FAT	lymphocyte differentiation	5	1.50E-01	9.40E-01
	GOTERM_BP_FAT	B cell activation	4	2.00E-01	9.50E-01
	GOTERM_BP_FAT	leukocyte mediated immunity	4	2.50E-01	9.60E-01
	GOTERM_BP_FAT	leukocyte differentiation	5	2.70E-01	9.70E-01
	GOTERM_BP_FAT	myeloid cell differentiation	4	2.90E-01	9.70E-01
	GOTERM_BP_FAT	T cell differentiation	3	3.80E-01	9.80E-01
**Annotation Cluster 2**	**Enrichment Score: 1.57**		**Count**	**P_Value**	**Benjamini**
	INTERPRO	Serine/threonine protein kinase, active site	17	2.10E-03	7.60E-01
	SP_PIR_KEYWORDS	serine/threonine-protein kinase	17	3.60E-03	2.30E-01
	INTERPRO	Protein kinase, ATP binding site	19	4.80E-03	7.90E-01
	UP_SEQ_FEATURE	binding site:ATP	21	5.40E-03	9.40E-01
	GOTERM_MF_FAT	protein serine/threonine kinase activity	18	6.50E-03	9.60E-01
	INTERPRO	Protein kinase, core	19	7.50E-03	8.10E-01
	GOTERM_MF_FAT	protein kinase activity	22	1.10E-02	9.30E-01
	UP_SEQ_FEATURE	domain:Protein kinase	18	1.20E-02	9.80E-01
	INTERPRO	Serine/threonine protein kinase-related	15	1.40E-02	8.90E-01
	UP_SEQ_FEATURE	nucleotide phosphate-binding region:ATP	30	1.40E-02	9.80E-01
	GOTERM_BP_FAT	phosphate metabolic process	30	2.30E-02	7.50E-01
	GOTERM_BP_FAT	phosphorus metabolic process	30	2.30E-02	7.50E-01
	SP_PIR_KEYWORDS	transferase	39	2.40E-02	5.90E-01
	SP_PIR_KEYWORDS	atp-binding	37	2.90E-02	5.90E-01
	UP_SEQ_FEATURE	active site:Proton acceptor	21	3.60E-02	1.00E+00
	INTERPRO	Serine/threonine protein kinase	11	3.60E-02	9.90E-01
	GOTERM_MF_FAT	purine nucleoside binding	43	4.40E-02	9.90E-01
	GOTERM_MF_FAT	ATP binding	40	4.80E-02	9.80E-01
	GOTERM_MF_FAT	nucleoside binding	43	4.80E-02	9.70E-01
	GOTERM_BP_FAT	protein amino acid phosphorylation	21	5.00E-02	8.50E-01
	SP_PIR_KEYWORDS	kinase	21	5.20E-02	6.90E-01
	SMART	S_TKc	11	5.40E-02	1.00E+00
	GOTERM_MF_FAT	adenyl ribonucleotide binding	40	5.60E-02	9.50E-01
	GOTERM_MF_FAT	adenyl nucleotide binding	41	7.40E-02	9.50E-01
	SP_PIR_KEYWORDS	nucleotide-binding	42	8.50E-02	6.70E-01
	GOTERM_BP_FAT	phosphorylation	23	8.60E-02	8.90E-01
	GOTERM_MF_FAT	purine ribonucleotide binding	46	9.30E-02	9.50E-01
	GOTERM_MF_FAT	ribonucleotide binding	46	9.30E-02	9.50E-01
	GOTERM_MF_FAT	purine nucleotide binding	47	1.20E-01	9.60E-01
	GOTERM_MF_FAT	nucleotide binding	53	1.50E-01	9.70E-01
**Annotation Cluster 3**	**Enrichment Score: 1.49**		**Count**	**P_Value**	**Benjamini**
	GOTERM_CC_FAT	endosomal part	5	2.60E-02	8.90E-01
	GOTERM_CC_FAT	endosome membrane	5	2.60E-02	8.90E-01
	GOTERM_CC_FAT	endosome	12	4.90E-02	7.80E-01
**Annotation Cluster 4**	**Enrichment Score: 1.38**		**Count**	**P_Value**	**Benjamini**
	GOTERM_BP_FAT	DNA metabolic process	23	6.10E-04	4.10E-01
	GOTERM_BP_FAT	nucleotide-excision repair	6	4.90E-03	6.20E-01
	KEGG_PATHWAY	Nucleotide excision repair	5	1.30E-02	5.20E-01
	SP_PIR_KEYWORDS	dna replication	6	2.90E-02	5.70E-01
	KEGG_PATHWAY	DNA replication	4	3.90E-02	6.70E-01
	GOTERM_BP_FAT	response to DNA damage stimulus	14	3.90E-02	8.30E-01
	GOTERM_BP_FAT	nucleotide-excision repair, DNA gap filling	3	4.50E-02	8.50E-01
	GOTERM_BP_FAT	DNA repair	11	6.30E-02	8.70E-01
	GOTERM_BP_FAT	cellular response to stress	18	6.70E-02	8.50E-01
	GOTERM_MF_FAT	damaged DNA binding	4	8.00E-02	9.40E-01
	KEGG_PATHWAY	Base excision repair	3	1.70E-01	8.70E-01
	SP_PIR_KEYWORDS	dna repair	5	5.00E-01	9.60E-01
	SP_PIR_KEYWORDS	DNA damage	5	5.70E-01	9.70E-01
**Annotation Cluster 5**	**Enrichment Score: 1.37**		**Count**	**P_Value**	**Benjamini**
	GOTERM_BP_FAT	cell cycle phase	16	2.10E-02	7.50E-01
	GOTERM_BP_FAT	M phase	13	3.50E-02	8.20E-01
	GOTERM_BP_FAT	meiosis	6	4.80E-02	8.60E-01
	GOTERM_BP_FAT	M phase of meiotic cell cycle	6	4.80E-02	8.60E-01
	GOTERM_BP_FAT	meiotic cell cycle	6	5.20E-02	8.50E-01
	SP_PIR_KEYWORDS	meiosis	4	6.40E-02	6.50E-01
**Annotation Cluster 6**	**Enrichment Score: 1.26**		**Count**	**P_Value**	**Benjamini**
	GOTERM_BP_FAT	male gamete generation	13	2.30E-02	7.60E-01
	GOTERM_BP_FAT	spermatogenesis	13	2.30E-02	7.60E-01
	GOTERM_BP_FAT	multicellular organism reproduction	17	3.80E-02	8.30E-01
	GOTERM_BP_FAT	reproductive process in a multicellular organism	17	3.80E-02	8.30E-01
	GOTERM_BP_FAT	sexual reproduction	16	4.50E-02	8.50E-01
	GOTERM_BP_FAT	gamete generation	14	5.70E-02	8.60E-01
	SP_PIR_KEYWORDS	spermatogenesis	5	1.90E-01	8.20E-01
	GOTERM_BP_FAT	reproductive cellular process	6	2.30E-01	9.60E-01
**Annotation Cluster 7**	**Enrichment Score: 1.25**		**Count**	**P_Value**	**Benjamini**
	GOTERM_CC_FAT	chromosomal part	16	1.00E-02	8.10E-01
	GOTERM_CC_FAT	chromosome	18	1.00E-02	6.80E-01

Negative correlation

Transcripts

Identifier	Gene Symbol	Correlation coefficient with CD19
ILMN_1808163	C11orf24	-0.400014
ILMN_1768856	CHAT	-0.400110384
ILMN_1686562	KIF13B	-0.400166572
ILMN_1772409	FAHD2A	-0.400269582
ILMN_2176693	LIPK	-0.400353994
ILMN_1762952	ARTN	-0.400376425
ILMN_1774427	CALCOCO1	-0.400395592
ILMN_1797642	KIAA1569	-0.400492066
ILMN_1790603	ATPAF1	-0.40061016
ILMN_1851972	BM973882	-0.400735148
ILMN_1832209	AA906426	-0.400781929
ILMN_1791114	CDR1	-0.400864931
ILMN_1878795	AW297854	-0.400885775
ILMN_1866217	DA162273	-0.400924414
ILMN_1718884	MACF1	-0.401120574
ILMN_1721994	HPS4	-0.401274698
ILMN_1849859	BU616091	-0.401380272
ILMN_1812607	MARCH8	-0.401729848
ILMN_1652164	UNC93B1	-0.401850875
ILMN_1691849	LIN7C	-0.401965556
ILMN_2184575	CCNI2	-0.402066709
ILMN_3231538	NA	-0.402078693
ILMN_1847793	CA449405	-0.402290358
ILMN_1848474	AI218484	-0.402409857
ILMN_1685985	EEF1A1	-0.402424309
ILMN_1776685	PCDH15	-0.402614594
ILMN_1768719	RDH11	-0.402672924
ILMN_1787630	BU569799	-0.402705253
ILMN_1703158	SFI1	-0.402716638
ILMN_1754592	EDARADD	-0.402765471
ILMN_3254666	ZNF737	-0.40278136
ILMN_1664907	FAM90A13	-0.40288402
ILMN_2137464	DVL3	-0.402950311
ILMN_1654112	PARD6A	-0.403230385
ILMN_1715814	LMAN1	-0.403478126
ILMN_1758173	TMEM99	-0.403564978
ILMN_1657483	SEC23B	-0.403793332
ILMN_1769406	PIAS2	-0.404015145
ILMN_1662147	MANEAL	-0.404158458
ILMN_1749641	FBXO3	-0.404401919
ILMN_1668814	CENPM	-0.404950021
ILMN_1725157	NEK6	-0.405050117
ILMN_2295330	NEK10	-0.405137379
ILMN_1749821	MED28	-0.405432328
ILMN_1655557	INTS6	-0.405438379
ILMN_1711765	NA	-0.405443898
ILMN_1655498	FAM57B	-0.405897785
ILMN_2338997	PTP4A2	-0.405957284
ILMN_2113126	RNASE3	-0.405967213
ILMN_1681884	LOC100506049	-0.405979469
ILMN_1656338	PTPRS	-0.405985381
ILMN_3309339	MIR100	-0.406011311
ILMN_1763587	TNMD	-0.406065457
ILMN_1716448	OR10A3	-0.406076888
ILMN_1657302	SULT1A1	-0.406077934
ILMN_2164173	DYDC1	-0.406208547
ILMN_1816599	BM468018	-0.406273438
ILMN_1767184	BC153822	-0.406566311
ILMN_1699648	ZNF516	-0.406705672
ILMN_1680348	NBPF3	-0.406971564
ILMN_1702320	JAKMIP2	-0.407272787
ILMN_2397841	SNCB	-0.407372661
ILMN_1748178	KY	-0.408209881
ILMN_1739855	CGB7	-0.408636996
ILMN_3296870	NA	-0.408685001
ILMN_1680094	HEMGN	-0.409079331
ILMN_1775537	TRIM69	-0.409300636
ILMN_1773148	C11orf61	-0.409339975
ILMN_1686244	OR6A2	-0.409467861
ILMN_3190226	LOC401387	-0.409574522
ILMN_3185313	CCDC85C	-0.410302869
ILMN_1907756	BX099104	-0.410327738
ILMN_1693771	ASPH	-0.41038133
ILMN_1668672	LOC401068	-0.4103987
ILMN_1773395	BLOC1S1-RDH5	-0.41071447
ILMN_1725750	BM551183	-0.410717759
ILMN_1813823	LOC149086	-0.410876036
ILMN_1804539	GPR89A	-0.411160688
ILMN_1852040	AW263556	-0.411671611
ILMN_3311165	NCRNA00173	-0.411714002
ILMN_2122953	CISD1	-0.411856848
ILMN_1708508	PPM1E	-0.412092678
ILMN_1709538	RTEL1-TNFRSF6B	-0.412103541
ILMN_2104409	RPL29P2	-0.412143201
ILMN_1773063	OSBPL1A	-0.412160938
ILMN_2204360	TTY1	-0.412453806
ILMN_1815010	RNF141	-0.412644545
ILMN_1754312	KLHDC7A	-0.412879929
ILMN_1749180	ESRP1	-0.412886091
ILMN_1722135	CNGA3	-0.412894306
ILMN_1698218	TRAF1	-0.413046048

Pathways

**Annotation Cluster 1**	**Enrichment Score: 1.43**		**Count**	**P_Value**	**Benjamini**
	KEGG_PATHWAY	Olfactory transduction	17	1.90E-03	1.80E-01
	SP_PIR_KEYWORDS	sensory transduction	21	5.20E-03	5.70E-01
	SP_PIR_KEYWORDS	olfaction	16	6.80E-03	4.20E-01
	GOTERM_BP_FAT	sensory perception of chemical stimulus	18	8.30E-03	1.00E+00
	INTERPRO	Olfactory receptor	16	9.60E-03	1.00E+00
	GOTERM_MF_FAT	olfactory receptor activity	16	1.30E-02	9.40E-01
	GOTERM_BP_FAT	sensory perception of smell	16	1.50E-02	1.00E+00
	PIR_SUPERFAMILY	PIRSF003152:G protein-coupled olfactory receptor, class II	13	2.90E-02	9.90E-01
	SP_PIR_KEYWORDS	receptor	39	3.70E-02	8.20E-01
	GOTERM_BP_FAT	cell surface receptor linked signal transduction	45	5.30E-02	1.00E+00
	INTERPRO	GPCR, rhodopsin-like superfamily	20	5.90E-02	1.00E+00
	SP_PIR_KEYWORDS	g-protein coupled receptor	22	6.00E-02	8.30E-01
	SP_PIR_KEYWORDS	transducer	23	6.60E-02	8.40E-01
	PIR_SUPERFAMILY	PIRSF800006:rhodopsin-like G protein-coupled receptors	19	7.70E-02	1.00E+00
	GOTERM_BP_FAT	sensory perception	22	7.90E-02	1.00E+00
	GOTERM_BP_FAT	neurological system process	30	9.50E-02	1.00E+00
	INTERPRO	7TM GPCR, rhodopsin-like	19	9.60E-02	1.00E+00
	GOTERM_BP_FAT	cognition	23	1.20E-01	1.00E+00
	GOTERM_BP_FAT	G-protein coupled receptor protein signaling pathway	27	1.50E-01	1.00E+00
	SP_PIR_KEYWORDS	cell membrane	46	1.80E-01	9.20E-01
	UP_SEQ_FEATURE	topological domain:Extracellular	53	3.30E-01	1.00E+00
**Annotation Cluster 2**	**Enrichment Score: 1.4**		**Count**	**P_Value**	**Benjamini**
	UP_SEQ_FEATURE	domain:PP2C-like	3	2.20E-02	1.00E+00
	INTERPRO	Protein phosphatase 2C	3	3.60E-02	1.00E+00
	INTERPRO	Protein phosphatase 2C, N-terminal	3	4.00E-02	1.00E+00
	INTERPRO	Protein phosphatase 2C-related	3	4.80E-02	1.00E+00
	SMART	PP2C_SIG	3	5.30E-02	1.00E+00
	SMART	PP2Cc	3	5.30E-02	1.00E+00
**Annotation Cluster 3**	**Enrichment Score: 1.19**		**Count**	**P_Value**	**Benjamini**
	UP_SEQ_FEATURE	repeat:Kelch 5	5	3.10E-02	1.00E+00
	UP_SEQ_FEATURE	repeat:Kelch 4	5	3.70E-02	1.00E+00
	SP_PIR_KEYWORDS	kelch repeat	5	3.80E-02	7.90E-01
	UP_SEQ_FEATURE	repeat:Kelch 1	5	3.90E-02	1.00E+00
	UP_SEQ_FEATURE	repeat:Kelch 2	5	3.90E-02	1.00E+00
	UP_SEQ_FEATURE	repeat:Kelch 3	5	3.90E-02	1.00E+00
	INTERPRO	Kelch-type beta propeller	4	1.20E-01	1.00E+00
	INTERPRO	Kelch repeat type 1	4	1.20E-01	1.00E+00
	SMART	Kelch	4	1.40E-01	1.00E+00
	UP_SEQ_FEATURE	repeat:Kelch 6	3	2.40E-01	1.00E+00
**Annotation Cluster 4**	**Enrichment Score: 1.15**		**Count**	**P_Value**	**Benjamini**
	SP_PIR_KEYWORDS	glycoprotein	101	2.10E-03	4.90E-01
	UP_SEQ_FEATURE	glycosylation site:N-linked (GlcNAc...)	94	8.60E-03	1.00E+00
	SP_PIR_KEYWORDS	membrane	128	4.20E-02	7.90E-01
	SP_PIR_KEYWORDS	transmembrane	102	7.20E-02	8.40E-01
	UP_SEQ_FEATURE	topological domain:Cytoplasmic	72	8.00E-02	1.00E+00
	UP_SEQ_FEATURE	transmembrane region	100	9.90E-02	1.00E+00
	GOTERM_CC_FAT	intrinsic to membrane	113	1.90E-01	1.00E+00
	GOTERM_CC_FAT	integral to membrane	109	2.00E-01	9.90E-01
	UP_SEQ_FEATURE	topological domain:Extracellular	53	3.30E-01	1.00E+00
	GOTERM_CC_FAT	plasma membrane	73	5.30E-01	1.00E+00
**Annotation Cluster 5**	**Enrichment Score: 1.03**		**Count**	**P_Value**	**Benjamini**
	UP_SEQ_FEATURE	domain:SAM	5	5.80E-02	1.00E+00
	INTERPRO	Sterile alpha motif SAM	5	8.30E-02	1.00E+00
	SMART	SAM	5	9.70E-02	1.00E+00
	INTERPRO	Sterile alpha motif-type	4	1.60E-01	1.00E+00
**Annotation Cluster 6**	**Enrichment Score: 1.02**		**Count**	**P_Value**	**Benjamini**
	SP_PIR_KEYWORDS	isopeptide bond	12	2.90E-02	8.50E-01
	SP_PIR_KEYWORDS	ubl conjugation	16	1.10E-01	9.20E-01
	UP_SEQ_FEATURE	cross-link:Glycyl lysine isopeptide (Lys-Gly) (interchain with G-Cter in ubiquitin)	6	2.90E-01	1.00E+00
**Annotation Cluster 7**	**Enrichment Score: 0.96**		**Count**	**P_Value**	**Benjamini**
	GOTERM_BP_FAT	regulation of I-kappaB kinase/NF-kappaB cascade	6	5.00E-02	1.00E+00
	GOTERM_BP_FAT	regulation of protein kinase cascade	9	9.50E-02	1.00E+00
	GOTERM_BP_FAT	positive regulation of signal transduction	10	1.00E-01	1.00E+00
	GOTERM_BP_FAT	positive regulation of I-kappaB kinase/NF-kappaB cascade	5	1.10E-01	1.00E+00
	GOTERM_BP_FAT	positive regulation of cell communication	10	1.60E-01	1.00E+00
	GOTERM_BP_FAT	positive regulation of protein kinase cascade	6	2.00E-01	1.00E+00
**Annotation Cluster 8**	**Enrichment Score: 0.96**		**Count**	**P_Value**	**Benjamini**
	GOTERM_MF_FAT	exodeoxyribonuclease activity, producing 5'-phosphomonoesters	3	8.60E-03	9.80E-01
	GOTERM_MF_FAT	exodeoxyribonuclease activity	3	8.60E-03	9.80E-01
	GOTERM_MF_FAT	deoxyribonuclease activity	4	2.40E-02	9.30E-01
	GOTERM_MF_FAT	exonuclease activity, active with either ribo- or deoxyribonucleic acids and producing 5'-phosphomonoesters	3	5.10E-02	9.90E-01
	GOTERM_MF_FAT	nuclease activity	7	7.00E-02	1.00E+00
	GOTERM_BP_FAT	DNA repair	10	8.50E-02	1.00E+00
	SP_PIR_KEYWORDS	nuclease	5	1.20E-01	9.30E-01
	SP_PIR_KEYWORDS	dna repair	7	1.30E-01	9.10E-01
	GOTERM_BP_FAT	response to DNA damage stimulus	11	1.60E-01	1.00E+00
	SP_PIR_KEYWORDS	DNA damage	7	1.60E-01	9.20E-01
	SP_PIR_KEYWORDS	exonuclease	3	1.80E-01	9.20E-01
	GOTERM_MF_FAT	exonuclease activity	3	2.50E-01	1.00E+00
	GOTERM_MF_FAT	structure-specific DNA binding	5	2.70E-01	1.00E+00
	GOTERM_MF_FAT	endonuclease activity	4	2.80E-01	1.00E+00
	SP_PIR_KEYWORDS	Endonuclease	3	3.20E-01	9.60E-01
	GOTERM_BP_FAT	DNA metabolic process	12	3.40E-01	1.00E+00
	GOTERM_BP_FAT	cellular response to stress	11	6.10E-01	1.00E+00
**Annotation Cluster 9**	**Enrichment Score: 0.95**		**Count**	**P_Value**	**Benjamini**
	INTERPRO	Zinc finger, RING-type	10	8.10E-02	1.00E+00
	UP_SEQ_FEATURE	zinc finger region:RING-type	8	8.50E-02	1.00E+00
	SMART	RING	10	1.00E-01	1.00E+00
	INTERPRO	Zinc finger, C3HC4 RING-type	8	1.10E-01	1.00E+00
	INTERPRO	Zinc finger, RING-type, conserved site	8	2.30E-01	1.00E+00
**Annotation Cluster 10**	**Enrichment Score: 0.87**		**Count**	**P_Value**	**Benjamini**

ILMN_1783583	TMEM17	0.502000137
ILMN_1710734	GZMK	0.501955518
ILMN_1672604	SET	0.501671021
ILMN_3281877	LOC400558	0.501396976
ILMN_1682799	STAMBPL1	0.501158961
ILMN_1798654	MCM6	0.500972075
ILMN_1783643	OR8K1	0.50082013
ILMN_2102960	KIAA1370	0.499361338
ILMN_2184064	ARRDC4	0.498695337
ILMN_1673363	CD97	0.498527144
ILMN_1673529	AGXT2L2	0.497466725
ILMN_1737195	CENPK	0.496879668
ILMN_2385647	ALAS1	0.496093856
ILMN_3238018	NA	0.496023596
ILMN_1743232	MICALCL	0.495981017
ILMN_1712389	CMTM1	0.49550662
ILMN_1701487	TAOK2	0.495063745
ILMN_1723279	SPINT4	0.494698934
ILMN_1701613	RARRES3	0.492433814
ILMN_2313079	NLRP1	0.492296094
ILMN_1663158	ZNF174	0.492293867
ILMN_1664928	ZNF599	0.490901154
ILMN_2050183	MYLK2	0.490388868
ILMN_1910908	BG205162	0.488816635
ILMN_1784584	RINT1	0.488143622
ILMN_1656134	CNOT7	0.487016741
ILMN_1763842	PTRH1	0.483004609
ILMN_1787750	CD200	0.482600527
ILMN_1739576	CYB5R2	0.482184225
ILMN_2313946	CPNE7	0.482093977
ILMN_2237252	LY6H	0.481435604
ILMN_1801766	CCDC109B	0.481401403
ILMN_1790109	THAP8	0.479752867
ILMN_2386891	GLT8D1	0.479472591
ILMN_2096743	TBC1D22A	0.479347347
ILMN_1803423	ARHGEF6	0.478612742
ILMN_1731736	RIN3	0.478419321
ILMN_1763104	TRAF4	0.47789945
ILMN_2205882	NSUN7	0.477750316
ILMN_2408908	MAP4K5	0.47700969
ILMN_3248874	SNORD8	0.476762824
ILMN_1916702	LOC100505576	0.476351765
ILMN_1815610	SYT12	0.47625645
ILMN_1680996	ALOX5	0.475772395
ILMN_1683300	BAX	0.47571239
ILMN_1786423	LOC400927	0.474628235
ILMN_1665943	MAP4K1	0.474001214
ILMN_3235462	NA	0.473936772
ILMN_3226669	TIMM23	0.473748772
ILMN_3309428	SNORD114-17	0.472671246
ILMN_1797046	MTHFSD	0.472482026
ILMN_2067890	CXCL11	0.472041404
ILMN_3244713	SPANXB2	0.470879643
ILMN_1696380	GHRL	0.470696458
ILMN_1793724	C3orf31	0.470529648
ILMN_1780533	RNASE6	0.470271335
ILMN_1704024	TMEM160	0.469994031
ILMN_2317751	REC8	0.469696331
ILMN_1724422	SELL	0.469395018
ILMN_1724533	LY96	0.469392708
ILMN_1737599	TRIM5	0.469329586
ILMN_3268380	NA	0.469267392
ILMN_3226955	RELL1	0.469259132
ILMN_1711786	NFE2	0.468292435
ILMN_3289508	CR604709	0.468049705
ILMN_1695432	TPST2	0.467209589
ILMN_2314121	TSHR	0.466450449
ILMN_1767662	LASS6	0.466292744
ILMN_1676864	DDX31	0.465997147
ILMN_1702821	TTLL7	0.465869965
ILMN_1659523	USP39	0.465748851
ILMN_1677963	TMCC1	0.465728042
ILMN_1711383	STK4	0.465377077
ILMN_3245223	ZSCAN30	0.465328895
ILMN_1670517	SLX1B-SULT1A4	0.465291052
ILMN_1720561	C1orf226	0.465280475
ILMN_1671809	DUSP22	0.464544677
ILMN_1705414	C17orf81	0.464192911
ILMN_2380946	EIF4G2	0.464147192
ILMN_1700428	HLA-DOB	0.463619355
ILMN_1792947	ESCO2	0.463474266
ILMN_1744398	ZSCAN10	0.463334092
ILMN_1698406	ORMDL1	0.463019777
ILMN_2075643	ANKRD29	0.462979061
ILMN_1792396	GOLGA6L1	0.462431071
ILMN_2338733	ART5	0.462174903
ILMN_1675427	NBPF15	0.462042843
ILMN_1696583	ZNF169	0.461816308
ILMN_1781155	LYN	0.46178428
ILMN_1724474	CARD16	0.461370959
ILMN_1741155	ZNF41	0.460770225
ILMN_1740502	CLEC4GP1	0.460481348
ILMN_1662932	LCP1	0.460464332
ILMN_3248759	ZNF626	0.460442404
ILMN_1791403	MTIF2	0.460046808
ILMN_2404112	TRDMT1	0.459577438
ILMN_1754553	MED19	0.459399642
ILMN_1727084	ADAMTSL1	0.459109101
ILMN_1706238	CSE1L	0.458710707
ILMN_1704084	DKFZp686H12134	0.458158655
ILMN_1765547	IRF2	0.457244041

	GOTERM_BP_FAT	protein-DNA complex assembly	7	1.00E-02	6.50E-01
	GOTERM_BP_FAT	nucleosome organization	7	1.10E-02	6.50E-01
	GOTERM_BP_FAT	chromatin assembly or disassembly	8	1.50E-02	6.90E-01
	SP_PIR_KEYWORDS	ubl conjugation	20	2.30E-02	6.10E-01
	GOTERM_BP_FAT	nucleosome assembly	6	2.70E-02	7.90E-01
	GOTERM_BP_FAT	DNA packaging	7	3.10E-02	8.00E-01
	GOTERM_BP_FAT	chromatin assembly	6	3.10E-02	7.90E-01
	GOTERM_CC_FAT	nucleosome	5	3.60E-02	8.70E-01
	PIR_SUPERFAMILY	PIRSF002048:histone H2A	3	3.90E-02	1.00E+00
	INTERPRO	Histone H2A	3	5.40E-02	1.00E+00
	KEGG_PATHWAY	Systemic lupus erythematosus	6	5.70E-02	6.10E-01
	INTERPRO	Histone core	4	6.10E-02	1.00E+00
	SMART	H2A	3	6.20E-02	9.90E-01
	SP_PIR_KEYWORDS	chromosomal protein	7	6.60E-02	6.30E-01
	SP_PIR_KEYWORDS	nucleosome core	4	6.70E-02	6.30E-01
	GOTERM_CC_FAT	protein-DNA complex	5	9.10E-02	8.80E-01
	SP_PIR_KEYWORDS	isopeptide bond	11	9.80E-02	7.00E-01
	UP_SEQ_FEATURE	cross-link:Glycyl lysine isopeptide (Lys-Gly) (interchain with G-Cter in ubiquitin)	8	9.80E-02	1.00E+00
	GOTERM_CC_FAT	chromatin	8	1.00E-01	8.60E-01
	GOTERM_BP_FAT	cellular macromolecular complex subunit organization	12	1.10E-01	9.10E-01
	GOTERM_BP_FAT	chromosome organization	14	1.80E-01	9.40E-01
	INTERPRO	Histone-fold	3	2.90E-01	1.00E+00
	GOTERM_BP_FAT	cellular macromolecular complex assembly	9	3.10E-01	9.70E-01
	GOTERM_BP_FAT	chromatin organization	10	3.50E-01	9.80E-01
	SP_PIR_KEYWORDS	methylation	6	5.10E-01	9.60E-01
**Annotation Cluster 8**	**Enrichment Score: 1.18**		**Count**	**P_Value**	**Benjamini**
	GOTERM_BP_FAT	B cell homeostasis	3	2.00E-02	7.50E-01
	GOTERM_BP_FAT	homeostasis of number of cells	6	5.20E-02	8.50E-01
	GOTERM_BP_FAT	lymphocyte homeostasis	3	1.10E-01	9.00E-01
	GOTERM_BP_FAT	leukocyte homeostasis	3	1.60E-01	9.40E-01
**Annotation Cluster 9**	**Enrichment Score: 1.14**		**Count**	**P_Value**	**Benjamini**
	KEGG_PATHWAY	B cell receptor signaling pathway	7	4.70E-03	4.10E-01
	BIOCARTA	BCR Signaling Pathway	3	1.40E-01	9.80E-01
	BIOCARTA	Fc Epsilon Receptor I Signaling in Mast Cells	3	1.80E-01	9.70E-01
	KEGG_PATHWAY	Fc epsilon RI signaling pathway	4	2.30E-01	9.30E-01
**Annotation Cluster 10**	**Enrichment Score: 1.09**		**Count**	**P_Value**	**Benjamini**
	GOTERM_BP_FAT	regulation of translation	9	6.60E-03	6.50E-01
	GOTERM_BP_FAT	regulation of translational initiation	5	9.10E-03	6.60E-01
	GOTERM_BP_FAT	posttranscriptional regulation of gene expression	9	6.50E-02	8.70E-01
	SP_PIR_KEYWORDS	Initiation factor	4	8.10E-02	6.60E-01
	GOTERM_MF_FAT	translation initiation factor activity	4	1.30E-01	9.60E-01
	SP_PIR_KEYWORDS	translation regulation	4	1.40E-01	7.70E-01
	SP_PIR_KEYWORDS	protein biosynthesis	7	1.60E-01	7.90E-01
	GOTERM_MF_FAT	translation factor activity, nucleic acid binding	4	3.20E-01	9.90E-01
	GOTERM_BP_FAT	translation	8	5.10E-01	9.90E-01
**Annotation Cluster 11**	**Enrichment Score: 1.02**		**Count**	**P_Value**	**Benjamini**
	GOTERM_BP_FAT	cellular component disassembly	4	9.10E-02	8.90E-01
	GOTERM_BP_FAT	cellular macromolecular complex disassembly	3	9.60E-02	8.90E-01
	GOTERM_BP_FAT	macromolecular complex disassembly	3	9.60E-02	8.90E-01
**Annotation Cluster 12**	**Enrichment Score: 1.01**		**Count**	**P_Value**	**Benjamini**
	GOTERM_MF_FAT	DNA-directed DNA polymerase activity	4	2.60E-02	9.60E-01
	GOTERM_MF_FAT	DNA polymerase activity	4	5.20E-02	9.60E-01
	SP_PIR_KEYWORDS	dna-directed dna polymerase	3	6.30E-02	6.60E-01
	GOTERM_MF_FAT	nucleotidyltransferase activity	5	2.50E-01	9.90E-01
	SP_PIR_KEYWORDS	nucleotidyltransferase	3	4.40E-01	9.40E-01
**Annotation Cluster 13**	**Enrichment Score: 1**		**Count**	**P_Value**	**Benjamini**
	GOTERM_BP_FAT	response to carbohydrate stimulus	6	7.10E-03	6.50E-01
	GOTERM_BP_FAT	response to glucose stimulus	3	2.30E-01	9.60E-01
	GOTERM_BP_FAT	response to hexose stimulus	3	2.50E-01	9.60E-01
	GOTERM_BP_FAT	response to monosaccharide stimulus	3	2.50E-01	9.60E-01
**Annotation Cluster 14**	**Enrichment Score: 1**		**Count**	**P_Value**	**Benjamini**
	GOTERM_BP_FAT	regulation of cell cycle	14	1.70E-02	7.30E-01
	GOTERM_BP_FAT	regulation of nuclear division	4	1.00E-01	9.00E-01
	GOTERM_BP_FAT	regulation of mitosis	4	1.00E-01	9.00E-01
	GOTERM_BP_FAT	regulation of organelle organization	8	1.50E-01	9.40E-01
	GOTERM_BP_FAT	regulation of mitotic cell cycle	6	1.90E-01	9.50E-01
	GOTERM_BP_FAT	regulation of cell cycle process	5	2.00E-01	9.50E-01
**Annotation Cluster 15**	**Enrichment Score: 0.98**		**Count**	**P_Value**	**Benjamini**
	GOTERM_BP_FAT	response to carbohydrate stimulus	6	7.10E-03	6.50E-01
	GOTERM_BP_FAT	response to heat	3	3.10E-01	9.70E-01
	GOTERM_BP_FAT	response to temperature stimulus	3	5.00E-01	9.90E-01
**Annotation Cluster 16**	**Enrichment Score: 0.97**		**Count**	**P_Value**	**Benjamini**
	GOTERM_BP_FAT	cell cycle phase	16	2.10E-02	7.50E-01
	KEGG_PATHWAY	Cell cycle	7	4.70E-02	6.60E-01
	GOTERM_BP_FAT	interphase of mitotic cell cycle	6	5.70E-02	8.60E-01
	GOTERM_BP_FAT	interphase	6	6.30E-02	8.70E-01
	GOTERM_BP_FAT	mitotic cell cycle	13	7.20E-02	8.60E-01
	SP_PIR_KEYWORDS	cell cycle control	3	1.20E-01	7.20E-01
	BIOCARTA	Cyclins and Cell Cycle Regulation	3	1.30E-01	9.90E-01
	BIOCARTA	Cell Cycle	3	1.70E-01	9.80E-01
	SP_PIR_KEYWORDS	cell division	8	2.60E-01	8.70E-01
	GOTERM_BP_FAT	G1/S transition of mitotic cell cycle	3	3.10E-01	9.70E-01
	GOTERM_BP_FAT	cell division	8	3.90E-01	9.80E-01
**Annotation Cluster 17**	**Enrichment Score: 0.96**		**Count**	**P_Value**	**Benjamini**
	GOTERM_BP_FAT	somatic diversification of immunoglobulins	3	7.20E-02	8.60E-01
	GOTERM_BP_FAT	somatic diversification of immune receptors	3	1.00E-01	9.00E-01
	GOTERM_BP_FAT	immunoglobulin production	3	1.20E-01	9.20E-01
	GOTERM_BP_FAT	production of molecular mediator of immune response	3	1.30E-01	9.20E-01
	GOTERM_BP_FAT	immune effector process	6	1.30E-01	9.30E-01
**Annotation Cluster 18**	**Enrichment Score: 0.95**		**Count**	**P_Value**	**Benjamini**
	GOTERM_BP_FAT	cell cycle process	23	2.50E-03	5.10E-01
	GOTERM_BP_FAT	cell cycle	27	7.70E-03	6.40E-01
	GOTERM_BP_FAT	cell cycle phase	16	2.10E-02	7.50E-01
	GOTERM_BP_FAT	M phase	13	3.50E-02	8.20E-01
	GOTERM_BP_FAT	mitotic cell cycle	13	7.20E-02	8.60E-01
	SP_PIR_KEYWORDS	cell cycle	13	1.90E-01	8.10E-01
	SP_PIR_KEYWORDS	cell division	8	2.60E-01	8.70E-01
	KEGG_PATHWAY	Progesterone-mediated oocyte maturation	4	2.70E-01	9.30E-01
	SP_PIR_KEYWORDS	mitosis	6	2.90E-01	8.80E-01
	GOTERM_BP_FAT	organelle fission	7	3.20E-01	9.70E-01
	GOTERM_BP_FAT	cell division	8	3.90E-01	9.80E-01

ILMN_2387303	SHPRH	-0.413130314
ILMN_1765299	PRIMA1	-0.41374377
ILMN_1837271	CA449813	-0.413855774
ILMN_1888330	AI809531	-0.414180046
ILMN_1655663	LEPREL4	-0.414199712
ILMN_3234989	LOC100133920	-0.414593865
ILMN_1803870	CDK16	-0.414678203
ILMN_1757142	C12orf37	-0.414684103
ILMN_1717094	ZNF618	-0.414722213
ILMN_2390821	THOC2	-0.41506034
ILMN_1658926	NOTCH3	-0.415156229
ILMN_2150123	OR4B1	-0.415166374
ILMN_1742040	GDPD4	-0.415561259
ILMN_1809457	OR5D16	-0.415812741
ILMN_2058468	BACH2	-0.416030453
ILMN_1678478	CHST5	-0.416236036
ILMN_1687092	KBTBD4	-0.416609598
ILMN_1724021	SLC6A19	-0.416631429
ILMN_1727331	LOC202181	-0.416657466
ILMN_1792014	FAM70B	-0.416958522
ILMN_2129734	LOC441601	-0.417066473
ILMN_2110496	C21orf91	-0.417115206
ILMN_1657864	TMUB2	-0.417345541
ILMN_2165354	DCLK1	-0.417578634
ILMN_1733963	ADRA2C	-0.417762879
ILMN_1699447	TRAF5	-0.418135095
ILMN_1688218	RASGRP4	-0.418330869
ILMN_1811995	FANCM	-0.41900932
ILMN_1732309	TAS2R9	-0.419031976
ILMN_1798037	TMEM215	-0.41937222
ILMN_1719599	SYTL4	-0.420116669
ILMN_3186401	FAM71E2	-0.420329497
ILMN_1729965	CXorf66	-0.42051325
ILMN_1840957	LOC286083	-0.42076178
ILMN_1743711	XPOT	-0.421024637
ILMN_1751276	BDNF	-0.421051879
ILMN_1768820	CYP11A1	-0.421271594
ILMN_1706200	MED15	-0.421360353
ILMN_2331232	TNFRSF6B	-0.421575684
ILMN_1725870	UBE2U	-0.421742285
ILMN_2374115	TFAP2A	-0.421806522
ILMN_2083469	IRS2	-0.421821886
ILMN_1836744	PGP	-0.421877591
ILMN_2327812	IL5RA	-0.422660607
ILMN_2305843	CHAT	-0.42278682
ILMN_2115591	KLHDC10	-0.423323533
ILMN_1785919	TMEM191C	-0.423333725
ILMN_3192034	NA	-0.423348474
ILMN_3235154	SNORD124	-0.423447089
ILMN_1704101	HSF4	-0.423487871
ILMN_3309894	MIR19B2	-0.423753673
ILMN_1709870	HES2	-0.424081744
ILMN_2119692	CSAD	-0.424135025
ILMN_1740305	DLEC1	-0.424172249
ILMN_1793213	SOX30	-0.424290394
ILMN_1717677	ZFHX2	-0.424574404
ILMN_3253310	BC150587	-0.424601281
ILMN_3208833	LOC100131354	-0.424656404
ILMN_1768567	OR10S1	-0.425250983
ILMN_1725912	KLK12	-0.425769984
ILMN_1840311	DA494348	-0.425810047
ILMN_1716957	C1QL1	-0.42583543
ILMN_1823555	AV707313	-0.425904336
ILMN_1734552	LRRC20	-0.426036098
ILMN_1770653	MAL2	-0.42626196
ILMN_1815548	OR52D1	-0.426439368
ILMN_2252701	SLC6A9	-0.42658262
ILMN_1742577	GTPBP4	-0.427036474
ILMN_1837979	BF515945	-0.427092759
ILMN_2318869	UBE2V1	-0.42710593
ILMN_1755033	C7orf51	-0.427110804
ILMN_2170209	RASD2	-0.427277467
ILMN_2176225	IFNW1	-0.427351629
ILMN_3240291	LOC643486	-0.427453858
ILMN_1690753	RBMY1A1	-0.427462365
ILMN_1879031	BX115902	-0.42764937
ILMN_2082273	RGS5	-0.42775618
ILMN_2211534	TMC8	-0.428005102
ILMN_2075892	LRP5	-0.428394976
ILMN_1772455	HDAC3	-0.428493452
ILMN_2334359	GFRA1	-0.428840607
ILMN_1686237	CBX2	-0.42901002
ILMN_1812070	ABCB1	-0.429048983
ILMN_1763419	TOP1	-0.429306765
ILMN_3310281	MIRLET7E	-0.429313087
ILMN_3239465	GALNTL1	-0.429428098
ILMN_1795338	YPEL1	-0.429565174
ILMN_1764022	FXYD1	-0.42997609
ILMN_2131447	LRRC3B	-0.430401332
ILMN_1746502	BC031979	-0.430660343
ILMN_2153280	KIAA0090	-0.430705933
ILMN_1791679	DNER	-0.430857408
ILMN_1662619	TFPI	-0.430944448
ILMN_1709741	BC033670	-0.43130855
ILMN_1775336	TSPYL2	-0.431678235
ILMN_1755423	PTCRA	-0.431869799
ILMN_1831214	AW592010	-0.431878827
ILMN_1730728	OR2B2	-0.432220853
ILMN_1745141	MBL1P1	-0.433113418
ILMN_1853705	AW118645	-0.433205727
ILMN_3287153	C21orf94	-0.433388667

	INTERPRO	PDZ/DHR/GLGF	6	1.30E-01	1.00E+00
	UP_SEQ_FEATURE	domain:PDZ	5	1.40E-01	1.00E+00
	SMART	PDZ	6	1.50E-01	9.90E-01
**Annotation Cluster 11**	**Enrichment Score: 0.8**		**Count**	**P_Value**	**Benjamini**
	GOTERM_BP_FAT	cellular hormone metabolic process	4	9.70E-02	1.00E+00
	GOTERM_BP_FAT	fat-soluble vitamin metabolic process	3	1.30E-01	1.00E+00
	GOTERM_BP_FAT	hormone metabolic process	5	1.40E-01	1.00E+00
	GOTERM_BP_FAT	regulation of hormone levels	6	1.50E-01	1.00E+00
	GOTERM_BP_FAT	vitamin metabolic process	3	4.10E-01	1.00E+00
**Annotation Cluster 12**	**Enrichment Score: 0.77**		**Count**	**P_Value**	**Benjamini**
	GOTERM_CC_FAT	chromosomal part	12	1.20E-01	1.00E+00
	GOTERM_CC_FAT	chromosome	13	1.70E-01	1.00E+00
	GOTERM_CC_FAT	nuclear chromosome	6	2.00E-01	1.00E+00
	GOTERM_CC_FAT	nuclear chromosome part	5	2.00E-01	9.90E-01
**Annotation Cluster 13**	**Enrichment Score: 0.74**		**Count**	**P_Value**	**Benjamini**
	GOTERM_BP_FAT	microtubule-based process	13	2.80E-03	9.90E-01
	GOTERM_BP_FAT	microtubule cytoskeleton organization	8	2.00E-02	1.00E+00
	GOTERM_BP_FAT	microtubule-based movement	6	6.00E-02	1.00E+00
	GOTERM_CC_FAT	microtubule organizing center part	4	9.10E-02	1.00E+00
	GOTERM_CC_FAT	microtubule	9	1.50E-01	1.00E+00
	SP_PIR_KEYWORDS	cytoskeleton	16	1.70E-01	9.20E-01
	SP_PIR_KEYWORDS	microtubule	7	2.30E-01	9.30E-01
	GOTERM_CC_FAT	microtubule cytoskeleton	14	2.50E-01	9.90E-01
	GOTERM_CC_FAT	centrosome	7	2.60E-01	9.90E-01
	GOTERM_BP_FAT	cytoskeleton organization	11	3.00E-01	1.00E+00
	GOTERM_CC_FAT	microtubule organizing center	7	3.50E-01	1.00E+00
	GOTERM_CC_FAT	spindle	4	5.30E-01	1.00E+00
	GOTERM_CC_FAT	cytoskeleton	27	5.60E-01	1.00E+00
	GOTERM_CC_FAT	cytoskeletal part	18	6.50E-01	1.00E+00
	GOTERM_CC_FAT	non-membrane-bounded organelle	47	7.40E-01	1.00E+00
	GOTERM_CC_FAT	intracellular non-membrane-bounded organelle	47	7.40E-01	1.00E+00
**Annotation Cluster 14**	**Enrichment Score: 0.72**		**Count**	**P_Value**	**Benjamini**
	UP_SEQ_FEATURE	domain:EGF-like 10; calcium-binding	3	2.20E-02	1.00E+00
	SP_PIR_KEYWORDS	notch signaling pathway	4	3.60E-02	8.60E-01
	UP_SEQ_FEATURE	domain:EGF-like 3	5	4.60E-02	1.00E+00
	INTERPRO	EGF-like, type 2	3	4.80E-02	1.00E+00
	UP_SEQ_FEATURE	domain:EGF-like 9	3	6.30E-02	1.00E+00
	GOTERM_BP_FAT	Notch signaling pathway	4	7.30E-02	1.00E+00
	UP_SEQ_FEATURE	domain:EGF-like 2	5	7.40E-02	1.00E+00
	INTERPRO	EGF-like calcium-binding, conserved site	4	2.40E-01	1.00E+00
	UP_SEQ_FEATURE	domain:EGF-like 1	4	3.70E-01	1.00E+00
	INTERPRO	EGF	4	3.90E-01	1.00E+00
	SP_PIR_KEYWORDS	egf-like domain	6	3.90E-01	9.70E-01
	INTERPRO	EGF-like region, conserved site	7	4.10E-01	1.00E+00
	INTERPRO	EGF-like	5	4.80E-01	1.00E+00
	INTERPRO	EGF-like calcium-binding	3	5.10E-01	1.00E+00
	INTERPRO	EGF-type aspartate/asparagine hydroxylation conserved site	3	5.10E-01	1.00E+00
	SMART	EGF	5	5.20E-01	1.00E+00
	SMART	EGF_CA	3	5.40E-01	1.00E+00
	INTERPRO	EGF-like, type 3	3	8.60E-01	1.00E+00
**Annotation Cluster 15**	**Enrichment Score: 0.7**		**Count**	**P_Value**	**Benjamini**
	SP_PIR_KEYWORDS	glycoprotein	101	2.10E-03	4.90E-01
	UP_SEQ_FEATURE	glycosylation site:N-linked (GlcNAc...)	94	8.60E-03	1.00E+00
	SP_PIR_KEYWORDS	signal	64	2.50E-01	9.40E-01
	UP_SEQ_FEATURE	signal peptide	64	2.70E-01	1.00E+00
	UP_SEQ_FEATURE	disulfide bond	54	3.80E-01	1.00E+00
	SP_PIR_KEYWORDS	disulfide bond	55	4.10E-01	9.80E-01
	SP_PIR_KEYWORDS	Secreted	28	7.50E-01	9.90E-01
	GOTERM_CC_FAT	extracellular space	12	7.60E-01	1.00E+00
	GOTERM_CC_FAT	extracellular region	32	9.20E-01	1.00E+00
	GOTERM_CC_FAT	extracellular region part	14	9.30E-01	1.00E+00
**Annotation Cluster 16**	**Enrichment Score: 0.67**		**Count**	**P_Value**	**Benjamini**
	GOTERM_BP_FAT	cholesterol metabolic process	6	2.90E-02	1.00E+00
	GOTERM_BP_FAT	sterol metabolic process	6	4.00E-02	1.00E+00
	GOTERM_BP_FAT	sterol transport	3	1.50E-01	1.00E+00
	GOTERM_BP_FAT	cholesterol transport	3	1.50E-01	1.00E+00
	GOTERM_BP_FAT	steroid metabolic process	7	1.80E-01	1.00E+00
	GOTERM_BP_FAT	lipid catabolic process	5	4.00E-01	1.00E+00
	GOTERM_BP_FAT	steroid biosynthetic process	3	4.70E-01	1.00E+00
	GOTERM_BP_FAT	lipid transport	4	5.10E-01	1.00E+00
	GOTERM_BP_FAT	lipid localization	4	5.60E-01	1.00E+00
	GOTERM_BP_FAT	lipid biosynthetic process	5	8.60E-01	1.00E+00
**Annotation Cluster 17**	**Enrichment Score: 0.66**		**Count**	**P_Value**	**Benjamini**
	GOTERM_BP_FAT	sulfur metabolic process	5	1.70E-01	1.00E+00
	GOTERM_BP_FAT	proteoglycan metabolic process	3	1.90E-01	1.00E+00
	GOTERM_BP_FAT	glycoprotein metabolic process	6	3.20E-01	1.00E+00
**Annotation Cluster 18**	**Enrichment Score: 0.63**		**Count**	**P_Value**	**Benjamini**
	GOTERM_BP_FAT	protein stabilization	3	9.50E-02	1.00E+00
	GOTERM_BP_FAT	regulation of protein stability	3	1.80E-01	1.00E+00
	GOTERM_BP_FAT	posttranscriptional regulation of gene expression	4	7.60E-01	1.00E+00
**Annotation Cluster 19**	**Enrichment Score: 0.62**		**Count**	**P_Value**	**Benjamini**
	INTERPRO	Kelch related	4	1.60E-01	1.00E+00
	INTERPRO	BTB/POZ-like	6	2.10E-01	1.00E+00
	INTERPRO	BTB/POZ fold	6	2.20E-01	1.00E+00
	SMART	BTB	6	2.40E-01	1.00E+00
	UP_SEQ_FEATURE	domain:BTB	5	2.50E-01	1.00E+00
	INTERPRO	BTB/POZ	4	3.90E-01	1.00E+00
**Annotation Cluster 20**	**Enrichment Score: 0.62**		**Count**	**P_Value**	**Benjamini**
	GOTERM_BP_FAT	secretion by cell	7	1.90E-01	1.00E+00
	GOTERM_BP_FAT	secretion	9	2.00E-01	1.00E+00
	GOTERM_BP_FAT	exocytosis	4	3.60E-01	1.00E+00
**Annotation Cluster 21**	**Enrichment Score: 0.58**		**Count**	**P_Value**	**Benjamini**
	GOTERM_BP_FAT	sexual reproduction	13	1.50E-01	1.00E+00
	GOTERM_BP_FAT	gamete generation	11	2.00E-01	1.00E+00
	GOTERM_BP_FAT	multicellular organism reproduction	12	3.00E-01	1.00E+00
	GOTERM_BP_FAT	reproductive process in a multicellular organism	12	3.00E-01	1.00E+00
	GOTERM_BP_FAT	spermatogenesis	8	3.50E-01	1.00E+00
	GOTERM_BP_FAT	male gamete generation	8	3.50E-01	1.00E+00
**Annotation Cluster 22**	**Enrichment Score: 0.57**		**Count**	**P_Value**	**Benjamini**
	GOTERM_MF_FAT	carbohydrate binding	10	2.10E-01	1.00E+00
	GOTERM_MF_FAT	glycosaminoglycan binding	5	2.50E-01	1.00E+00

ILMN_1726554	IREB2	0.456797741
ILMN_1740042	TRIM16	0.456786244
ILMN_1773868	ZRSR2	0.45650138
ILMN_1786036	GPATCH2	0.455459435
ILMN_1676594	BX647938	0.455233903
ILMN_1733538	RGS10	0.455080068
ILMN_1770244	CBX1	0.454998997
ILMN_1708604	CCZ1	0.454666487
ILMN_2369018	EVI2A	0.454627163
ILMN_2382500	MS4A14	0.454172365
ILMN_2380850	SDCCAG3	0.453803725
ILMN_1801767	ABHD3	0.45360276
ILMN_1685625	UCP2	0.453319645
ILMN_1708790	CA6	0.452998677
ILMN_1655077	PRDM1	0.45298958
ILMN_3229189	N4BP2L2	0.451626643
ILMN_1735489	F13B	0.451531918
ILMN_1680435	NPEPPS	0.451302929
ILMN_1677765	LRP8	0.451115599
ILMN_3239435	HTT	0.451100733
ILMN_1811814	ZNF90	0.450990914
ILMN_1714818	SSX5	0.450699347
ILMN_1664516	CENPF	0.450560581
ILMN_1730307	MED16	0.44959844
ILMN_3298313	LOC730179	0.449411356
ILMN_2414027	CMTM1	0.449181448
ILMN_1765911	HMX3	0.448601122
ILMN_3225176	BX104084	0.448505497
ILMN_1807491	LAIR2	0.448403281
ILMN_2084353	M6PR	0.448335525
ILMN_1707804	SLX1B-SULT1A4	0.448069164
ILMN_2103107	ADAMDEC1	0.448000782
ILMN_3249677	DEFB105A	0.447895587
ILMN_1664691	DAPP1	0.447794486
ILMN_2109416	NAPSB	0.447713719
ILMN_1813197	SLITRK6	0.447436113
ILMN_1741712	MS4A4A	0.447235542
ILMN_1809488	SPCS2	0.447050788
ILMN_3232926	C1orf167	0.446918955
ILMN_1748642	MORN5	0.446373993
ILMN_2257377	SCML1	0.445673524
ILMN_1795927	PSPC1	0.444860359
ILMN_3232529	LOC375133	0.444592953
ILMN_1659364	RFC5	0.444406075
ILMN_2243697	ZDHHC13	0.444372926
ILMN_1797895	FFAR2	0.444151986
ILMN_2186858	ZNF839	0.443972869
ILMN_2269977	GLUL	0.443885559
ILMN_1721580	TBX15	0.443229666
ILMN_1699113	RP1-177G6.2	0.443014893
ILMN_1680129	NSUN2	0.442992276
ILMN_2195138	SAMD11	0.442651861
ILMN_1744886	OR6T1	0.442574617
ILMN_1733998	DHRS9	0.442400666
ILMN_1652366	NLRP7	0.442150649
ILMN_1698934	CMTM7	0.441865737
ILMN_1678391	SPEF2	0.441372021
ILMN_2120547	ZMYND17	0.441260395
ILMN_1677633	NFATC3	0.441083248
ILMN_1785831	ZDHHC13	0.440974543
ILMN_1801101	ZBTB48	0.440589858
ILMN_3252167	LOC100129484	0.440076823
ILMN_3248631	PTX4	0.439880809
ILMN_1809448	C1orf157	0.439772237
ILMN_1728019	PML	0.439158669
ILMN_3308856	MIR520H	0.439138236
ILMN_1691563	GAGE12I	0.438557019
ILMN_3184843	IP6K3	0.438413551
ILMN_3234756	SNORD6	0.43830063
ILMN_3236988	NA	0.437682392
ILMN_1713688	DHX32	0.437592329
ILMN_1813489	RAF1	0.437267154
ILMN_1812489	CCDC21	0.437228893
ILMN_1677591	CCL4L2	0.437161853
ILMN_1759952	PSMA5	0.437023958
ILMN_1734365	PAX2	0.43587338
ILMN_1669555	FAM48A	0.435625283
ILMN_3249603	NKAIN2	0.435535458
ILMN_1756443	INHA	0.435403864
ILMN_2093343	PLAC8	0.435179228
ILMN_2056687	C17orf56	0.435146742
ILMN_2383484	C19orf48	0.435006611
ILMN_1655827	COPS2	0.43492029
ILMN_1676173	PARVB	0.434420753
ILMN_3247788	ZNF286A	0.43426138
ILMN_1791127	CDH12	0.434210261
ILMN_2144573	CTBS	0.434046955
ILMN_1691441	C1orf126	0.433754972
ILMN_1664946	ZDHHC16	0.433453059
ILMN_1711098	HYMAI	0.433410406
ILMN_1778213	STK33	0.433222665
ILMN_1685792	MAGI1	0.433149528
ILMN_1801700	ZNF461	0.433079819
ILMN_1678922	HERC4	0.432419315
ILMN_1712082	GCNT3	0.432045013
ILMN_1801852	SEC61B	0.431720591
ILMN_1811178	SCAPER	0.431245017
ILMN_1767960	NSUN7	0.431063185
ILMN_1692731	TTYH3	0.431023961

	GOTERM_BP_FAT	mitosis	6	4.60E-01	9.90E-01
	GOTERM_BP_FAT	nuclear division	6	4.60E-01	9.90E-01
	GOTERM_BP_FAT	M phase of mitotic cell cycle	6	4.70E-01	9.90E-01
**Annotation Cluster 19**	**Enrichment Score: 0.93**		**Count**	**P_Value**	**Benjamini**
	UP_SEQ_FEATURE	zinc finger region:DHHC-type	3	9.20E-02	1.00E+00
	INTERPRO	Zinc finger, DHHC-type	3	9.50E-02	1.00E+00
	SP_PIR_KEYWORDS	Acyltransferase	6	1.80E-01	8.10E-01
**Annotation Cluster 20**	**Enrichment Score: 0.92**		**Count**	**P_Value**	**Benjamini**
	KEGG_PATHWAY	B cell receptor signaling pathway	7	4.70E-03	4.10E-01
	KEGG_PATHWAY	VEGF signaling pathway	3	4.70E-01	9.60E-01
	KEGG_PATHWAY	Natural killer cell mediated cytotoxicity	3	7.80E-01	9.90E-01
**Annotation Cluster 21**	**Enrichment Score: 0.91**		**Count**	**P_Value**	**Benjamini**
	GOTERM_CC_FAT	nuclear lumen	39	4.30E-02	8.00E-01
	GOTERM_CC_FAT	nucleoplasm	25	7.00E-02	8.20E-01
	GOTERM_CC_FAT	intracellular organelle lumen	43	1.30E-01	8.70E-01
	GOTERM_CC_FAT	membrane-enclosed lumen	44	1.50E-01	8.90E-01
	GOTERM_CC_FAT	organelle lumen	43	1.60E-01	8.80E-01
	GOTERM_CC_FAT	nucleolus	16	4.10E-01	9.60E-01
**Annotation Cluster 22**	**Enrichment Score: 0.88**		**Count**	**P_Value**	**Benjamini**
	GOTERM_BP_FAT	macromolecular complex subunit organization	23	3.00E-02	8.10E-01
	GOTERM_BP_FAT	macromolecular complex assembly	21	4.90E-02	8.50E-01
	GOTERM_BP_FAT	cellular macromolecular complex subunit organization	12	1.10E-01	9.10E-01
	GOTERM_BP_FAT	cellular macromolecular complex assembly	9	3.10E-01	9.70E-01
	GOTERM_BP_FAT	protein complex biogenesis	13	3.20E-01	9.70E-01
	GOTERM_BP_FAT	protein complex assembly	13	3.20E-01	9.70E-01
**Annotation Cluster 23**	**Enrichment Score: 0.86**		**Count**	**P_Value**	**Benjamini**
	SP_PIR_KEYWORDS	chemotaxis	6	1.30E-02	4.60E-01
	GOTERM_MF_FAT	chemokine receptor binding	4	7.60E-02	9.50E-01
	GOTERM_BP_FAT	taxis	7	1.10E-01	9.00E-01
	GOTERM_BP_FAT	chemotaxis	7	1.10E-01	9.00E-01
	GOTERM_BP_FAT	locomotory behavior	9	1.90E-01	9.50E-01
	GOTERM_MF_FAT	cytokine activity	7	2.00E-01	9.80E-01
	GOTERM_MF_FAT	chemokine activity	3	2.40E-01	9.80E-01
	SP_PIR_KEYWORDS	cytokine	6	2.80E-01	8.80E-01
	GOTERM_BP_FAT	behavior	10	6.10E-01	1.00E+00
**Annotation Cluster 24**	**Enrichment Score: 0.86**		**Count**	**P_Value**	**Benjamini**
	GOTERM_MF_FAT	protein tyrosine phosphatase activity	7	1.90E-02	9.50E-01
	GOTERM_BP_FAT	dephosphorylation	7	9.20E-02	8.90E-01
	INTERPRO	Protein-tyrosine phosphatase, active site	5	1.00E-01	1.00E+00
	GOTERM_MF_FAT	phosphoprotein phosphatase activity	7	1.20E-01	9.60E-01
	UP_SEQ_FEATURE	domain:Tyrosine-protein phosphatase	4	1.20E-01	1.00E+00
	GOTERM_BP_FAT	protein amino acid dephosphorylation	6	1.30E-01	9.30E-01
	INTERPRO	Protein-tyrosine phosphatase	4	2.20E-01	1.00E+00
	UP_SEQ_FEATURE	active site:Phosphocysteine intermediate	4	2.20E-01	1.00E+00
	INTERPRO	Dual-specific/protein-tyrosine phosphatase, conserved region	4	2.30E-01	1.00E+00
	SP_PIR_KEYWORDS	protein phosphatase	5	2.50E-01	8.70E-01
	GOTERM_MF_FAT	phosphatase activity	7	4.00E-01	9.90E-01
**Annotation Cluster 25**	**Enrichment Score: 0.83**		**Count**	**P_Value**	**Benjamini**
	GOTERM_BP_FAT	regulation of cellular protein metabolic process	19	7.80E-03	6.20E-01
	SP_PIR_KEYWORDS	proteinase	3	4.30E-02	6.40E-01
	INTERPRO	Proteasome, subunit alpha/beta	3	5.40E-02	1.00E+00
	SP_PIR_KEYWORDS	threonine protease	3	5.70E-02	6.50E-01
	GOTERM_CC_FAT	proteasome core complex	3	5.90E-02	8.10E-01
	GOTERM_MF_FAT	threonine-type endopeptidase activity	3	6.10E-02	9.40E-01
	GOTERM_MF_FAT	threonine-type peptidase activity	3	6.10E-02	9.40E-01
	GOTERM_BP_FAT	negative regulation of cellular protein metabolic process	8	7.30E-02	8.60E-01
	GOTERM_BP_FAT	regulation of protein modification process	11	7.70E-02	8.70E-01
	GOTERM_BP_FAT	negative regulation of protein metabolic process	8	8.50E-02	8.90E-01
	GOTERM_BP_FAT	negative regulation of protein modification process	6	9.30E-02	8.90E-01
	GOTERM_BP_FAT	negative regulation of ubiquitin-protein ligase activity during mitotic cell cycle	4	1.40E-01	9.30E-01
	GOTERM_BP_FAT	anaphase-promoting complex-dependent proteasomal ubiquitin-dependent protein catabolic process	4	1.40E-01	9.30E-01
	GOTERM_BP_FAT	proteasomal ubiquitin-dependent protein catabolic process	5	1.50E-01	9.40E-01
	GOTERM_BP_FAT	proteasomal protein catabolic process	5	1.50E-01	9.40E-01
	GOTERM_BP_FAT	negative regulation of ubiquitin-protein ligase activity	4	1.50E-01	9.40E-01
	GOTERM_BP_FAT	negative regulation of ligase activity	4	1.50E-01	9.40E-01
	GOTERM_BP_FAT	positive regulation of ubiquitin-protein ligase activity during mitotic cell cycle	4	1.60E-01	9.40E-01
	GOTERM_BP_FAT	positive regulation of ubiquitin-protein ligase activity	4	1.70E-01	9.40E-01
	GOTERM_BP_FAT	regulation of ubiquitin-protein ligase activity during mitotic cell cycle	4	1.70E-01	9.40E-01
	GOTERM_BP_FAT	positive regulation of protein modification process	7	1.80E-01	9.40E-01
	GOTERM_BP_FAT	positive regulation of ligase activity	4	1.80E-01	9.40E-01
	GOTERM_BP_FAT	negative regulation of protein ubiquitination	4	1.90E-01	9.50E-01
	GOTERM_BP_FAT	positive regulation of cellular protein metabolic process	8	1.90E-01	9.50E-01
	GOTERM_BP_FAT	regulation of ubiquitin-protein ligase activity	4	2.10E-01	9.50E-01
	GOTERM_BP_FAT	positive regulation of protein metabolic process	8	2.20E-01	9.50E-01
	GOTERM_BP_FAT	regulation of ligase activity	4	2.20E-01	9.50E-01
	GOTERM_BP_FAT	positive regulation of protein ubiquitination	4	2.40E-01	9.60E-01
	GOTERM_BP_FAT	positive regulation of catalytic activity	14	2.50E-01	9.60E-01
	KEGG_PATHWAY	Proteasome	3	2.60E-01	9.40E-01
	GOTERM_BP_FAT	positive regulation of molecular function	15	3.00E-01	9.70E-01
	SP_PIR_KEYWORDS	proteasome	3	3.00E-01	8.80E-01
	GOTERM_BP_FAT	regulation of protein ubiquitination	4	3.30E-01	9.70E-01
	GOTERM_CC_FAT	proteasome complex	3	3.40E-01	9.50E-01
	GOTERM_BP_FAT	ubiquitin-dependent protein catabolic process	7	3.60E-01	9.80E-01
	GOTERM_BP_FAT	negative regulation of catalytic activity	6	6.60E-01	1.00E+00
	GOTERM_BP_FAT	negative regulation of molecular function	6	8.10E-01	1.00E+00
**Annotation Cluster 26**	**Enrichment Score: 0.8**		**Count**	**P_Value**	**Benjamini**
	SP_PIR_KEYWORDS	inflammatory response	5	6.40E-02	6.40E-01
	GOTERM_BP_FAT	defense response	17	1.80E-01	9.40E-01
	GOTERM_BP_FAT	inflammatory response	10	2.10E-01	9.50E-01
	GOTERM_BP_FAT	response to wounding	14	2.70E-01	9.70E-01
**Annotation Cluster 27**	**Enrichment Score: 0.77**		**Count**	**P_Value**	**Benjamini**
	UP_SEQ_FEATURE	domain:SH2	5	1.20E-01	1.00E+00
	SP_PIR_KEYWORDS	SH2 domain	5	1.70E-01	8.00E-01
	INTERPRO	SH2 motif	5	1.80E-01	1.00E+00
	SMART	SH2	5	2.20E-01	1.00E+00
**Annotation Cluster 28**	**Enrichment Score: 0.75**		**Count**	**P_Value**	**Benjamini**
	SP_PIR_KEYWORDS	zinc	56	3.10E-02	5.60E-01
	SP_PIR_KEYWORDS	zinc-finger	44	5.50E-02	6.50E-01
	GOTERM_MF_FAT	zinc ion binding	58	5.60E-02	9.40E-01

ILMN_1699131	FLJ45340	-0.433542611
ILMN_2264177	UPK3B	-0.433690092
ILMN_1706478	HOXA7	-0.433849467
ILMN_1684931	GPR119	-0.433992015
ILMN_1692255	WDR45	-0.434110545
ILMN_1788384	C9orf5	-0.434142439
ILMN_3310461	MIR196A2	-0.434330371
ILMN_3267852	AX747372	-0.434521747
ILMN_1768489	TTTY20	-0.43497711
ILMN_1705637	ACR	-0.434993417
ILMN_1799031	BG760841	-0.435141666
ILMN_2104141	FGD5	-0.435253283
ILMN_1752582	RAB5B	-0.435271786
ILMN_1748109	PEX1	-0.435444703
ILMN_1753457	PKP3	-0.435779669
ILMN_1752648	HTN3	-0.436144864
ILMN_1751389	LOC100288974	-0.436258279
ILMN_1900275	BQ021435	-0.436301132
ILMN_1893960	BX098094	-0.436962297
ILMN_1691376	JAG1	-0.436965094
ILMN_1682034	HEY2	-0.436974675
ILMN_2342559	SPOCK3	-0.437254853
ILMN_1666494	CRK	-0.437330207
ILMN_3308465	MIR376A1	-0.437429826
ILMN_1668152	C2orf84	-0.438112607
ILMN_1878778	DB339409	-0.438965097
ILMN_3236423	RAB3GAP2	-0.439217578
ILMN_1724254	INSL6	-0.439257033
ILMN_1680892	TREX2	-0.439567746
ILMN_1715113	HARS2	-0.439757721
ILMN_2281186	RGNEF	-0.439876628
ILMN_1712556	ZW10	-0.440049849
ILMN_1742578	MKLN1	-0.440458726
ILMN_3221807	NA	-0.440864721
ILMN_1667831	HOXB6	-0.441012009
ILMN_2276461	MAP2	-0.441062527
ILMN_1729417	GNE	-0.441114768
ILMN_1676307	FIGN	-0.441214626
ILMN_1670263	CNST	-0.441291604
ILMN_1692589	LOC728723	-0.441470802
ILMN_1658679	YJEFN3	-0.441503057
ILMN_1660879	FRMPD2	-0.441977961
ILMN_1776213	RGMB	-0.442031716
ILMN_2343563	ANAPC11	-0.442709314
ILMN_3215627	LOC349114	-0.44344529
ILMN_1680393	SNORD55	-0.443625387
ILMN_1749081	AUTS2	-0.443986948
ILMN_3266133	DKFZp434A062	-0.444076683
ILMN_1781951	SCN8A	-0.444536699
ILMN_2271304	CACNB1	-0.444750513
ILMN_1724349	RGS3	-0.445051394
ILMN_1702824	OR52W1	-0.445166054
ILMN_1759117	XK	-0.44534427
ILMN_1771966	BCCIP	-0.4454526
ILMN_1660356	PPP4R4	-0.445465847
ILMN_2161848	GPR81	-0.445831339
ILMN_1701497	CD5L	-0.445920401
ILMN_1712413	RPL39L	-0.446237439
ILMN_2322499	RORA	-0.4464769
ILMN_1696339	ZIC2	-0.446479245
ILMN_2201596	CYTL1	-0.446543634
ILMN_1738449	STIM2	-0.447025618
ILMN_1688903	THOC3	-0.447238756
ILMN_1750748	KRT16P3	-0.44741153
ILMN_2153029	CGB5	-0.447847882
ILMN_3292963	RPS6KB1	-0.448155369
ILMN_1850240	AW262671	-0.448291643
ILMN_1695807	DYRK3	-0.448555835
ILMN_3243049	SNORD19	-0.448726475
ILMN_1709825	WDR89	-0.448736708
ILMN_2187206	STYK1	-0.448969047
ILMN_1724324	PLD3	-0.449075239
ILMN_1831106	LOC400043	-0.449100757
ILMN_1754220	SF3A2	-0.449571575
ILMN_3308310	RNU86	-0.449863303
ILMN_1773901	STX12	-0.449960308
ILMN_3247159	SCARNA8	-0.450007794
ILMN_1811380	KCNA10	-0.450024446
ILMN_1784834	OR52E6	-0.450120443
ILMN_1651490	CYP26A1	-0.450309287
ILMN_1777251	NEBL	-0.450457858
ILMN_2280816	CEMP1	-0.450927657
ILMN_1653748	CST11	-0.45097075
ILMN_1678928	SLCO3A1	-0.451318893
ILMN_1777980	TAB1	-0.451581486
ILMN_1653347	BC012881	-0.451706365
ILMN_1709768	LOC100128818	-0.451715627
ILMN_1813668	ASB4	-0.451933901
ILMN_1694166	HIST1H2AA	-0.452124279
ILMN_3308886	MIR320B2	-0.452164095
ILMN_1768798	SPAG9	-0.452590658
ILMN_1764166	BCKDHB	-0.452810721
ILMN_1791409	ITGA7	-0.45312739
ILMN_1749253	TUBD1	-0.454276342
ILMN_1709056	FBXW8	-0.45439884
ILMN_1723418	CEL	-0.454403933
ILMN_2043173	TEKT2	-0.454456346
ILMN_1882000	TXLNG	-0.455707905
ILMN_1729835	AANAT	-0.456208355

	GOTERM_MF_FAT	heparin binding	4	2.90E-01	1.00E+00
	GOTERM_MF_FAT	pattern binding	5	3.10E-01	1.00E+00
	GOTERM_MF_FAT	polysaccharide binding	5	3.10E-01	1.00E+00
**Annotation Cluster 23**	**Enrichment Score: 0.57**		**Count**	**P_Value**	**Benjamini**
	SP_PIR_KEYWORDS	antibiotic	4	1.20E-01	9.40E-01
	SP_PIR_KEYWORDS	Antimicrobial	4	1.30E-01	9.10E-01
	GOTERM_BP_FAT	response to bacterium	6	2.90E-01	1.00E+00
	GOTERM_BP_FAT	defense response to bacterium	4	3.50E-01	1.00E+00
	GOTERM_BP_FAT	defense response	9	8.90E-01	1.00E+00
**Annotation Cluster 24**	**Enrichment Score: 0.57**		**Count**	**P_Value**	**Benjamini**
	GOTERM_BP_FAT	inner ear development	4	1.80E-01	1.00E+00
	GOTERM_BP_FAT	ear development	4	2.60E-01	1.00E+00
	GOTERM_BP_FAT	sensory organ development	6	4.20E-01	1.00E+00
**Annotation Cluster 25**	**Enrichment Score: 0.56**		**Count**	**P_Value**	**Benjamini**
	GOTERM_BP_FAT	cellular macromolecule catabolic process	20	8.60E-02	1.00E+00
	GOTERM_BP_FAT	macromolecule catabolic process	21	9.30E-02	1.00E+00
	KEGG_PATHWAY	Ubiquitin mediated proteolysis	6	1.20E-01	9.90E-01
	SP_PIR_KEYWORDS	ubl conjugation pathway	14	1.20E-01	9.20E-01
	GOTERM_BP_FAT	proteolysis involved in cellular protein catabolic process	16	1.50E-01	1.00E+00
	GOTERM_BP_FAT	cellular protein catabolic process	16	1.60E-01	1.00E+00
	GOTERM_BP_FAT	protein catabolic process	16	1.80E-01	1.00E+00
	GOTERM_BP_FAT	modification-dependent macromolecule catabolic process	15	1.80E-01	1.00E+00
	GOTERM_BP_FAT	modification-dependent protein catabolic process	15	1.80E-01	1.00E+00
	GOTERM_MF_FAT	acid-amino acid ligase activity	6	3.10E-01	1.00E+00
	GOTERM_MF_FAT	small conjugating protein ligase activity	5	3.60E-01	1.00E+00
	GOTERM_MF_FAT	ligase activity, forming carbon-nitrogen bonds	6	4.10E-01	1.00E+00
	SP_PIR_KEYWORDS	ligase	7	4.60E-01	9.70E-01
	GOTERM_BP_FAT	protein ubiquitination	3	6.50E-01	1.00E+00
	GOTERM_BP_FAT	proteolysis	19	6.90E-01	1.00E+00
	GOTERM_BP_FAT	protein modification by small protein conjugation	3	7.10E-01	1.00E+00
	GOTERM_MF_FAT	ubiquitin-protein ligase activity	3	7.50E-01	1.00E+00
	GOTERM_BP_FAT	protein modification by small protein conjugation or removal	3	8.00E-01	1.00E+00
**Annotation Cluster 26**	**Enrichment Score: 0.55**		**Count**	**P_Value**	**Benjamini**
	GOTERM_BP_FAT	tissue morphogenesis	7	1.20E-01	1.00E+00
	GOTERM_BP_FAT	morphogenesis of an epithelium	4	2.90E-01	1.00E+00
	GOTERM_BP_FAT	morphogenesis of embryonic epithelium	3	2.90E-01	1.00E+00
	GOTERM_BP_FAT	epithelium development	5	6.10E-01	1.00E+00
**Annotation Cluster 27**	**Enrichment Score: 0.53**		**Count**	**P_Value**	**Benjamini**
	UP_SEQ_FEATURE	repeat:ANK 3	7	1.40E-01	1.00E+00
	UP_SEQ_FEATURE	repeat:ANK 1	7	2.40E-01	1.00E+00
	UP_SEQ_FEATURE	repeat:ANK 2	7	2.40E-01	1.00E+00
	INTERPRO	Ankyrin	7	2.50E-01	1.00E+00
	SP_PIR_KEYWORDS	ank repeat	7	2.60E-01	9.40E-01
	SMART	ANK	7	2.90E-01	1.00E+00
	UP_SEQ_FEATURE	repeat:ANK 5	4	4.20E-01	1.00E+00
	UP_SEQ_FEATURE	repeat:ANK 6	3	4.90E-01	1.00E+00
	UP_SEQ_FEATURE	repeat:ANK 4	4	5.40E-01	1.00E+00
**Annotation Cluster 28**	**Enrichment Score: 0.51**		**Count**	**P_Value**	**Benjamini**
	GOTERM_BP_FAT	microtubule-based movement	6	6.00E-02	1.00E+00
	SP_PIR_KEYWORDS	microtubule	7	2.30E-01	9.30E-01
	GOTERM_MF_FAT	microtubule motor activity	3	4.10E-01	1.00E+00
	SP_PIR_KEYWORDS	motor protein	3	6.90E-01	9.90E-01
	GOTERM_MF_FAT	motor activity	3	7.40E-01	1.00E+00
**Annotation Cluster 29**	**Enrichment Score: 0.5**		**Count**	**P_Value**	**Benjamini**
	GOTERM_BP_FAT	glycosaminoglycan metabolic process	3	2.70E-01	1.00E+00
	GOTERM_BP_FAT	polysaccharide metabolic process	4	3.40E-01	1.00E+00
	GOTERM_BP_FAT	aminoglycan metabolic process	3	3.40E-01	1.00E+00
**Annotation Cluster 30**	**Enrichment Score: 0.46**		**Count**	**P_Value**	**Benjamini**
	GOTERM_MF_FAT	protein kinase binding	5	2.80E-01	1.00E+00
	GOTERM_MF_FAT	enzyme binding	12	3.60E-01	1.00E+00
	GOTERM_MF_FAT	kinase binding	5	4.10E-01	1.00E+00
**Annotation Cluster 31**	**Enrichment Score: 0.45**		**Count**	**P_Value**	**Benjamini**
	GOTERM_MF_FAT	protein serine/threonine phosphatase activity	3	2.00E-01	1.00E+00
	SP_PIR_KEYWORDS	protein phosphatase	5	2.00E-01	9.30E-01
	GOTERM_BP_FAT	protein amino acid dephosphorylation	5	2.40E-01	1.00E+00
	GOTERM_BP_FAT	dephosphorylation	5	3.20E-01	1.00E+00
	GOTERM_MF_FAT	phosphoprotein phosphatase activity	5	3.60E-01	1.00E+00
	INTERPRO	Protein-tyrosine phosphatase	3	4.20E-01	1.00E+00
	INTERPRO	Dual-specific/protein-tyrosine phosphatase, conserved region	3	4.30E-01	1.00E+00
	UP_SEQ_FEATURE	active site:Phosphocysteine intermediate	3	4.40E-01	1.00E+00
	INTERPRO	Protein-tyrosine phosphatase, active site	3	4.70E-01	1.00E+00
	GOTERM_MF_FAT	phosphatase activity	6	4.80E-01	1.00E+00
	GOTERM_MF_FAT	protein tyrosine phosphatase activity	3	5.70E-01	1.00E+00
**Annotation Cluster 32**	**Enrichment Score: 0.43**		**Count**	**P_Value**	**Benjamini**
	GOTERM_BP_FAT	appendage morphogenesis	4	2.80E-01	1.00E+00
	GOTERM_BP_FAT	limb morphogenesis	4	2.80E-01	1.00E+00
	GOTERM_BP_FAT	appendage development	4	3.00E-01	1.00E+00
	GOTERM_BP_FAT	limb development	4	3.00E-01	1.00E+00
	GOTERM_BP_FAT	embryonic appendage morphogenesis	3	4.80E-01	1.00E+00
	GOTERM_BP_FAT	embryonic limb morphogenesis	3	4.80E-01	1.00E+00
	GOTERM_BP_FAT	pattern specification process	6	5.60E-01	1.00E+00
**Annotation Cluster 33**	**Enrichment Score: 0.43**		**Count**	**P_Value**	**Benjamini**
	GOTERM_BP_FAT	negative regulation of neuron apoptosis	4	6.90E-02	1.00E+00
	GOTERM_BP_FAT	regulation of neuron apoptosis	4	2.40E-01	1.00E+00
	GOTERM_BP_FAT	negative regulation of apoptosis	9	3.40E-01	1.00E+00
	GOTERM_BP_FAT	negative regulation of programmed cell death	9	3.50E-01	1.00E+00
	GOTERM_BP_FAT	negative regulation of cell death	9	3.60E-01	1.00E+00
	GOTERM_BP_FAT	regulation of apoptosis	15	6.40E-01	1.00E+00
	GOTERM_BP_FAT	regulation of programmed cell death	15	6.60E-01	1.00E+00
	GOTERM_BP_FAT	regulation of cell death	15	6.60E-01	1.00E+00
	GOTERM_BP_FAT	anti-apoptosis	4	7.40E-01	1.00E+00
**Annotation Cluster 34**	**Enrichment Score: 0.42**		**Count**	**P_Value**	**Benjamini**
	GOTERM_BP_FAT	ER to Golgi vesicle-mediated transport	3	1.80E-01	1.00E+00
	SP_PIR_KEYWORDS	er-golgi transport	3	4.40E-01	9.70E-01
	GOTERM_BP_FAT	Golgi vesicle transport	3	7.00E-01	1.00E+00
**Annotation Cluster 35**	**Enrichment Score: 0.39**		**Count**	**P_Value**	**Benjamini**
	GOTERM_CC_FAT	intrinsic to endoplasmic reticulum membrane	4	1.20E-01	1.00E+00
	SP_PIR_KEYWORDS	endoplasmic reticulum	17	2.10E-01	9.20E-01
	GOTERM_CC_FAT	integral to endoplasmic reticulum membrane	3	2.30E-01	9.90E-01

ILMN_1711462	MNS1	0.431008159
ILMN_3309021	MIR142	0.430991173
ILMN_2400940	L3MBTL1	0.430690373
ILMN_1768293	NUP155	0.430656843
ILMN_1709039	RPL13	0.430527484
ILMN_1689136	LOC644962	0.430472491
ILMN_2190069	DEFB110	0.430215916
ILMN_1774735	DDB1	0.429568009
ILMN_1741404	MSC	0.429338614
ILMN_1696110	TUBGCP3	0.429108896
ILMN_1662362	LGR6	0.429042515
ILMN_1813275	DUSP22	0.428980584
ILMN_1670746	BOLL	0.428900195
ILMN_1804332	GPR137	0.428705046
ILMN_1746435	HIST1H1E	0.428635775
ILMN_1885891	BM671563	0.428438276
ILMN_1693966	ISG20L2	0.428373748
ILMN_3272096	LOC100129520	0.428232646
ILMN_1695311	HLA-DMA	0.428230583
ILMN_1794534	CCDC81	0.428081229
ILMN_1788108	TXNDC5	0.428014073
ILMN_3310401	MIR24-1	0.427240054
ILMN_1684040	THEMIS	0.426428939
ILMN_1766487	LRRC25	0.42642414
ILMN_1710571	PAPD5	0.426395341
ILMN_2370882	ACSL5	0.426284596
ILMN_1676797	C8orf37	0.425625875
ILMN_1682567	CCDC106	0.425603769
ILMN_3239475	LOC100131551	0.425476218
ILMN_1666633	BC010030	0.425459315
ILMN_1862043	BG188151	0.425258138
ILMN_2290628	IL16	0.425137294
ILMN_1697736	EXOSC2	0.424777044
ILMN_1797822	SEL1L3	0.424596312
ILMN_2339796	CDC16	0.424593842
ILMN_1724230	FRG1	0.424403582
ILMN_1686097	TOP2A	0.424343713
ILMN_2219246	ITPRIPL2	0.424112176
ILMN_1749960	FAM78B	0.424025366
ILMN_1802360	KRTAP5-2	0.423763128
ILMN_1729058	SCAMP1	0.423141884
ILMN_1759075	TNFRSF13B	0.42277624
ILMN_1731048	TLR1	0.422616435
ILMN_2087303	XKR8	0.422590908
ILMN_3218223	MPHOSPH10	0.422569347
ILMN_1798977	GPR97	0.422474237
ILMN_1719870	FAM72D	0.422423589
ILMN_2291871	GLIS3	0.422384735
ILMN_2095820	UTP14A	0.421859536
ILMN_1695356	RNF166	0.421808771
ILMN_1760360	LTN1	0.421494761
ILMN_1764320	C8orf58	0.421330844
ILMN_1785756	H2AFX	0.421056118
ILMN_2179283	S100A7L2	0.420808115
ILMN_1694367	SNORD35B	0.420504917
ILMN_1665559	CDK2	0.420458794
ILMN_1792518	STX7	0.4203618
ILMN_1803873	OR5M8	0.419947086
ILMN_2398939	MBP	0.419806914
ILMN_1759991	NOL12	0.419695951
ILMN_2285648	KCNIP4	0.419609131
ILMN_1721629	ZNF654	0.419132155
ILMN_1803941	TBC1D15	0.419043936
ILMN_3296076	AK057422	0.418547832
ILMN_3234948	LOC100130000	0.418535332
ILMN_1660624	LIMK2	0.418327976
ILMN_1728199	POLE	0.417829312
ILMN_1798233	PSMB9	0.417782787
ILMN_2188333	CD69	0.417727537
ILMN_1748283	PIM2	0.417202514
ILMN_1652407	ZMYND8	0.417123696
ILMN_1860540	AA709087	0.416988159
ILMN_1677446	UBE2V1	0.416940628
ILMN_2410262	MTMR14	0.416759803
ILMN_1695443	YSK4	0.416534872
ILMN_1666594	IRF8	0.416454891
ILMN_3238613	SNORA26	0.415975733
ILMN_1804339	CAMK1G	0.41582568
ILMN_1728923	F2RL2	0.415431461
ILMN_1707858	H2AFZ	0.415342164
ILMN_1710735	ZNF451	0.415327771
ILMN_1700918	GAGE8	0.41505743
ILMN_1659801	ATP6V1C1	0.414864623
ILMN_1667803	OR56A1	0.414581723
ILMN_2299862	KCNH1	0.414248429
ILMN_2260082	NAIP	0.414003011
ILMN_2102201	GPR19	0.4138424
ILMN_2170157	VCY	0.413769351
ILMN_3251455	RHOQ	0.413724277
ILMN_1667771	GRB10	0.413639371
ILMN_3237734	TNRC18	0.413572603
ILMN_2099301	SUN2	0.413464971
ILMN_3229033	TDG	0.413359861
ILMN_2408968	UEVLD	0.413130778
ILMN_1694584	CLTCL1	0.412978643
ILMN_1803385	GOLGA8B	0.412805873
ILMN_1704164	ATP13A2	0.412700613
ILMN_2095610	ANXA8	0.412623539
ILMN_1708537	RBPJ	0.412144957
ILMN_1755077	HEBP2	0.411635372

	GOTERM_MF_FAT	transition metal ion binding	64	1.70E-01	9.80E-01
	SP_PIR_KEYWORDS	metal-binding	65	2.20E-01	8.40E-01
	GOTERM_MF_FAT	cation binding	83	6.40E-01	1.00E+00
	GOTERM_MF_FAT	metal ion binding	82	6.60E-01	1.00E+00
	GOTERM_MF_FAT	ion binding	84	6.60E-01	1.00E+00
**Annotation Cluster 29**	**Enrichment Score: 0.74**		**Count**	**P_Value**	**Benjamini**
	KEGG_PATHWAY	Fc gamma R-mediated phagocytosis	6	4.90E-02	6.10E-01
	KEGG_PATHWAY	Fc epsilon RI signaling pathway	4	2.30E-01	9.30E-01
	KEGG_PATHWAY	Chemokine signaling pathway	5	5.60E-01	9.50E-01
**Annotation Cluster 30**	**Enrichment Score: 0.72**		**Count**	**P_Value**	**Benjamini**
	INTERPRO	Sterile alpha motif-type	5	6.80E-02	1.00E+00
	UP_SEQ_FEATURE	domain:SAM	4	2.10E-01	1.00E+00
	INTERPRO	Sterile alpha motif SAM	4	2.80E-01	1.00E+00
	SMART	SAM	4	3.30E-01	1.00E+00
**Annotation Cluster 31**	**Enrichment Score: 0.71**		**Count**	**P_Value**	**Benjamini**
	GOTERM_CC_FAT	condensed chromosome	7	4.30E-02	7.70E-01
	GOTERM_CC_FAT	condensed chromosome, centromeric region	3	3.80E-01	9.50E-01
	GOTERM_CC_FAT	chromosome, centromeric region	4	4.50E-01	9.60E-01
**Annotation Cluster 32**	**Enrichment Score: 0.71**		**Count**	**P_Value**	**Benjamini**
	GOTERM_BP_FAT	cell death	22	5.70E-02	8.60E-01
	GOTERM_BP_FAT	death	22	5.90E-02	8.60E-01
	GOTERM_BP_FAT	apoptosis	17	1.60E-01	9.40E-01
	GOTERM_BP_FAT	positive regulation of apoptosis	13	1.60E-01	9.40E-01
	GOTERM_BP_FAT	positive regulation of programmed cell death	13	1.70E-01	9.40E-01
	GOTERM_BP_FAT	positive regulation of cell death	13	1.70E-01	9.40E-01
	GOTERM_BP_FAT	programmed cell death	17	1.70E-01	9.40E-01
	GOTERM_BP_FAT	regulation of programmed cell death	21	2.10E-01	9.50E-01
	GOTERM_BP_FAT	regulation of cell death	21	2.10E-01	9.50E-01
	GOTERM_BP_FAT	regulation of apoptosis	20	2.70E-01	9.70E-01
	SP_PIR_KEYWORDS	Apoptosis	10	3.30E-01	9.00E-01
	GOTERM_BP_FAT	induction of apoptosis	7	6.30E-01	1.00E+00
	GOTERM_BP_FAT	induction of programmed cell death	7	6.30E-01	1.00E+00
**Annotation Cluster 33**	**Enrichment Score: 0.7**		**Count**	**P_Value**	**Benjamini**
	GOTERM_BP_FAT	membrane organization	13	8.60E-02	8.90E-01
	GOTERM_BP_FAT	membrane invagination	8	1.60E-01	9.40E-01
	GOTERM_BP_FAT	endocytosis	8	1.60E-01	9.40E-01
	GOTERM_BP_FAT	vesicle-mediated transport	15	2.80E-01	9.70E-01
	SP_PIR_KEYWORDS	Endocytosis	3	5.60E-01	9.70E-01
**Annotation Cluster 34**	**Enrichment Score: 0.66**		**Count**	**P_Value**	**Benjamini**
	GOTERM_BP_FAT	germ cell development	5	1.50E-01	9.40E-01
	GOTERM_BP_FAT	reproductive cellular process	6	2.30E-01	9.60E-01
	GOTERM_BP_FAT	spermatid differentiation	3	3.20E-01	9.70E-01
**Annotation Cluster 35**	**Enrichment Score: 0.64**		**Count**	**P_Value**	**Benjamini**
	GOTERM_BP_FAT	response to ionizing radiation	5	3.30E-02	8.00E-01
	GOTERM_BP_FAT	response to radiation	7	2.20E-01	9.50E-01
	GOTERM_BP_FAT	response to light stimulus	5	3.00E-01	9.70E-01
	GOTERM_BP_FAT	response to UV	3	3.30E-01	9.70E-01
	GOTERM_BP_FAT	response to oxidative stress	5	4.20E-01	9.90E-01
	GOTERM_BP_FAT	response to abiotic stimulus	9	4.60E-01	9.90E-01
**Annotation Cluster 36**	**Enrichment Score: 0.63**		**Count**	**P_Value**	**Benjamini**
	GOTERM_BP_FAT	regulation of behavior	4	6.50E-02	8.70E-01
	GOTERM_BP_FAT	regulation of chemotaxis	3	1.30E-01	9.20E-01
	GOTERM_BP_FAT	positive regulation of behavior	3	1.50E-01	9.40E-01
	GOTERM_BP_FAT	positive regulation of response to external stimulus	3	3.70E-01	9.80E-01
	GOTERM_BP_FAT	regulation of locomotion	5	5.50E-01	9.90E-01
	GOTERM_BP_FAT	regulation of response to external stimulus	4	6.30E-01	1.00E+00
**Annotation Cluster 37**	**Enrichment Score: 0.61**		**Count**	**P_Value**	**Benjamini**
	GOTERM_BP_FAT	regulation of cellular response to stress	7	1.80E-02	7.30E-01
	GOTERM_BP_FAT	regulation of stress-activated protein kinase signaling pathway	5	5.10E-02	8.50E-01
	GOTERM_BP_FAT	regulation of protein kinase cascade	10	6.60E-02	8.70E-01
	GOTERM_BP_FAT	positive regulation of stress-activated protein kinase signaling pathway	3	6.60E-02	8.60E-01
	GOTERM_BP_FAT	positive regulation of protein kinase cascade	7	1.20E-01	9.20E-01
	GOTERM_BP_FAT	protein kinase cascade	12	1.30E-01	9.20E-01
	GOTERM_BP_FAT	regulation of JNK cascade	4	1.40E-01	9.30E-01
	GOTERM_BP_FAT	regulation of MAPKKK cascade	5	1.80E-01	9.40E-01
	GOTERM_BP_FAT	positive regulation of signal transduction	9	2.50E-01	9.60E-01
	GOTERM_BP_FAT	positive regulation of catalytic activity	14	2.50E-01	9.60E-01
	GOTERM_BP_FAT	positive regulation of molecular function	15	3.00E-01	9.70E-01
	GOTERM_BP_FAT	MAPKKK cascade	6	3.10E-01	9.70E-01
	GOTERM_BP_FAT	regulation of MAP kinase activity	5	3.20E-01	9.70E-01
	GOTERM_BP_FAT	positive regulation of MAP kinase activity	4	3.40E-01	9.80E-01
	GOTERM_BP_FAT	regulation of phosphorylation	12	3.40E-01	9.80E-01
	GOTERM_BP_FAT	positive regulation of cell communication	9	3.50E-01	9.80E-01
	GOTERM_BP_FAT	regulation of phosphorus metabolic process	12	3.90E-01	9.80E-01
	GOTERM_BP_FAT	regulation of phosphate metabolic process	12	3.90E-01	9.80E-01
	GOTERM_BP_FAT	regulation of protein amino acid phosphorylation	5	4.60E-01	9.90E-01
	GOTERM_BP_FAT	positive regulation of protein kinase activity	6	4.70E-01	9.90E-01
	GOTERM_BP_FAT	activation of MAPK activity	3	5.00E-01	9.90E-01
	GOTERM_BP_FAT	positive regulation of kinase activity	6	5.00E-01	9.90E-01
	GOTERM_BP_FAT	positive regulation of transferase activity	6	5.30E-01	9.90E-01
	GOTERM_BP_FAT	regulation of protein kinase activity	8	5.50E-01	1.00E+00
	GOTERM_BP_FAT	regulation of kinase activity	8	5.80E-01	1.00E+00
	GOTERM_BP_FAT	regulation of transferase activity	8	6.30E-01	1.00E+00
**Annotation Cluster 38**	**Enrichment Score: 0.6**		**Count**	**P_Value**	**Benjamini**
	GOTERM_BP_FAT	cell cycle checkpoint	5	1.10E-01	9.00E-01
	GOTERM_BP_FAT	DNA damage checkpoint	3	2.50E-01	9.60E-01
	GOTERM_BP_FAT	DNA integrity checkpoint	3	2.80E-01	9.70E-01
	GOTERM_BP_FAT	DNA damage response, signal transduction	3	4.80E-01	9.90E-01
**Annotation Cluster 39**	**Enrichment Score: 0.6**		**Count**	**P_Value**	**Benjamini**
	UP_SEQ_FEATURE	repeat:LRR 1	11	9.70E-02	1.00E+00
	UP_SEQ_FEATURE	repeat:LRR 2	11	1.00E-01	1.00E+00
	SP_PIR_KEYWORDS	leucine-rich repeat	11	1.00E-01	7.00E-01
	UP_SEQ_FEATURE	repeat:LRR 6	8	1.20E-01	1.00E+00
	UP_SEQ_FEATURE	repeat:LRR 7	7	1.40E-01	1.00E+00
	UP_SEQ_FEATURE	repeat:LRR 8	6	1.70E-01	1.00E+00
	UP_SEQ_FEATURE	repeat:LRR 5	8	1.70E-01	1.00E+00
	UP_SEQ_FEATURE	repeat:LRR 16	3	2.20E-01	1.00E+00
	UP_SEQ_FEATURE	repeat:LRR 4	8	2.30E-01	1.00E+00
	UP_SEQ_FEATURE	repeat:LRR 3	9	2.30E-01	1.00E+00
	UP_SEQ_FEATURE	repeat:LRR 15	3	2.60E-01	1.00E+00

ILMN_1713533	TKTL2	-0.456411638
ILMN_1722673	PCDHB4	-0.456518686
ILMN_2397846	SNCB	-0.456525276
ILMN_1797728	HMGCS1	-0.45680823
ILMN_1797201	KRTAP10-2	-0.456892062
ILMN_1803033	MGST1	-0.456907348
ILMN_1882015	BG951912	-0.457277256
ILMN_1732426	CRHR1	-0.457723916
ILMN_1665630	HMGN2	-0.457960315
ILMN_2209221	DMRT1	-0.458401794
ILMN_3228746	GDPD5	-0.458663417
ILMN_3247514	C7orf72	-0.458737446
ILMN_1811068	CABYR	-0.458873874
ILMN_3230160	PCLO	-0.459023749
ILMN_1694952	SSX4B	-0.459820308
ILMN_1806510	VPS53	-0.459946356
ILMN_1672074	SHISA8	-0.459984469
ILMN_1711422	PLEKHN1	-0.460330044
ILMN_2045720	OR4K17	-0.460808868
ILMN_1652819	OPA3	-0.461070186
ILMN_1784477	PLEKHG5	-0.461842968
ILMN_1797169	OR4A47	-0.462159392
ILMN_1749341	NCRNA00164	-0.463442659
ILMN_1767015	BCORL1	-0.463836142
ILMN_1661653	DET1	-0.463887314
ILMN_1715669	TP53I11	-0.464112925
ILMN_1766320	IL3	-0.464214463
ILMN_1724669	RUNX1T1	-0.464272871
ILMN_1687544	ZNF567	-0.464950286
ILMN_1691149	LOC100133050	-0.465743359
ILMN_3204668	BM921547	-0.466246826
ILMN_1724427	OR6N2	-0.466336793
ILMN_1667416	TRIM74	-0.46668875
ILMN_1711270	SUGP2	-0.466767484
ILMN_1803850	TSPAN15	-0.466868713
ILMN_1778188	TP73	-0.467235425
ILMN_1669669	KCMF1	-0.468208107
ILMN_1791297	LOC649305	-0.468284027
ILMN_1685854	C5orf53	-0.468398954
ILMN_1660963	CCIN	-0.469449678
ILMN_1728684	PELP1	-0.469451343
ILMN_1696767	SERP2	-0.469531274
ILMN_1714859	TCIRG1	-0.469596699
ILMN_1693483	FGF18	-0.47018915
ILMN_1722070	APOC3	-0.470889218
ILMN_1683096	ASB1	-0.471793824
ILMN_1758735	NLRP12	-0.472896496
ILMN_1705177	DNAH14	-0.472924544
ILMN_2083742	C1orf146	-0.472940877
ILMN_1779401	CHP	-0.474247301
ILMN_1810115	KCTD2	-0.474484429
ILMN_1816781	BF056254	-0.474720322
ILMN_1671055	GSG1L	-0.474869228
ILMN_1743847	ACAP3	-0.475297998
ILMN_3247710	NA	-0.475596695
ILMN_1704497	KRT25	-0.47602419
ILMN_2064090	OR4M1	-0.476516807
ILMN_1652439	KRTAP3-1	-0.476790322
ILMN_1755834	FEN1	-0.477852171
ILMN_3309909	MIR1286	-0.478524198
ILMN_3194067	ZNF44	-0.478668931
ILMN_1678718	AK307134	-0.479131349
ILMN_1883255	AA470148	-0.480474134
ILMN_1747271	ATP1B2	-0.480909483
ILMN_1651554	RGS5	-0.481074924
ILMN_3280792	BC021736	-0.481080106
ILMN_1824767	BM670198	-0.48138506
ILMN_2340908	SCML1	-0.481896176
ILMN_2226324	BRP44L	-0.481907727
ILMN_1665217	C3orf34	-0.482331489
ILMN_1718135	PRAMEF11	-0.482442745
ILMN_3224846	NCRNA00164	-0.483346993
ILMN_1696600	AL832737	-0.484691968
ILMN_2054481	PTCHD3	-0.485272588
ILMN_1912185	GPC6	-0.485390148
ILMN_1734559	SORD	-0.485447218
ILMN_1792076	TRERF1	-0.485566576
ILMN_2294644	RFWD2	-0.485927189
ILMN_1767590	TSPY3	-0.48676253
ILMN_1861128	AW237129	-0.486957076
ILMN_2328029	EXT2	-0.487528628
ILMN_2358272	HAP1	-0.487690053
ILMN_1745151	SHANK2	-0.488607709
ILMN_1718706	ERAL1	-0.48878327
ILMN_1820887	HBCBP	-0.489717692
ILMN_1654609	TIGA1	-0.490351903
ILMN_1674793	LRRC17	-0.490718896
ILMN_1809857	ZRANB3	-0.491465952
ILMN_1661492	ARRDC1	-0.491553042
ILMN_1743594	UBXN7	-0.491698947
ILMN_1761259	EXT2	-0.492854061
ILMN_3244281	SNORD1A	-0.493086508
ILMN_1743673	LOC645332	-0.494322868
ILMN_2261973	DPH3	-0.494897169
ILMN_1684339	LRIT1	-0.49577221
ILMN_1681892	OAZ3	-0.496073039
ILMN_1741719	TEX13A	-0.49629397
ILMN_1805996	SIN3A	-0.496942056
ILMN_1730432	IGSF8	-0.497609655
ILMN_1658809	SEZ6	-0.498571321

	GOTERM_CC_FAT	intrinsic to organelle membrane	5	3.00E-01	1.00E+00
	GOTERM_CC_FAT	endoplasmic reticulum membrane	7	4.10E-01	1.00E+00
	GOTERM_CC_FAT	nuclear envelope-endoplasmic reticulum network	7	4.60E-01	1.00E+00
	GOTERM_CC_FAT	endoplasmic reticulum	20	4.70E-01	1.00E+00
	GOTERM_CC_FAT	endoplasmic reticulum part	8	4.90E-01	1.00E+00
	GOTERM_CC_FAT	integral to organelle membrane	3	6.80E-01	1.00E+00
	GOTERM_CC_FAT	endomembrane system	11	9.40E-01	1.00E+00
	GOTERM_CC_FAT	organelle membrane	15	9.60E-01	1.00E+00
**Annotation Cluster 36**	**Enrichment Score: 0.39**		**Count**	**P_Value**	**Benjamini**
	GOTERM_BP_FAT	embryonic morphogenesis	11	6.20E-02	1.00E+00
	GOTERM_BP_FAT	skeletal system development	9	2.30E-01	1.00E+00
	GOTERM_BP_FAT	embryonic organ morphogenesis	5	2.40E-01	1.00E+00
	GOTERM_BP_FAT	anterior/posterior pattern formation	5	2.60E-01	1.00E+00
	GOTERM_BP_FAT	skeletal system morphogenesis	4	3.50E-01	1.00E+00
	GOTERM_BP_FAT	embryonic organ development	5	4.00E-01	1.00E+00
	INTERPRO	Homeodomain-related	6	4.10E-01	1.00E+00
	GOTERM_BP_FAT	regionalization	5	4.90E-01	1.00E+00
	GOTERM_BP_FAT	pattern specification process	6	5.60E-01	1.00E+00
	INTERPRO	Homeobox, conserved site	5	5.90E-01	1.00E+00
	INTERPRO	Homeobox	5	6.00E-01	1.00E+00
	SP_PIR_KEYWORDS	Homeobox	5	6.30E-01	9.90E-01
	SMART	HOX	5	6.50E-01	1.00E+00
	UP_SEQ_FEATURE	DNA-binding region:Homeobox	4	6.70E-01	1.00E+00
	GOTERM_MF_FAT	sequence-specific DNA binding	11	6.80E-01	1.00E+00
	SP_PIR_KEYWORDS	DNA binding	6	7.30E-01	9.90E-01
**Annotation Cluster 37**	**Enrichment Score: 0.37**		**Count**	**P_Value**	**Benjamini**
	UP_SEQ_FEATURE	repeat:17	3	2.30E-01	1.00E+00
	UP_SEQ_FEATURE	repeat:16	3	2.70E-01	1.00E+00
	UP_SEQ_FEATURE	repeat:1	7	2.70E-01	1.00E+00
	UP_SEQ_FEATURE	repeat:2	7	2.80E-01	1.00E+00
	UP_SEQ_FEATURE	repeat:15	3	3.40E-01	1.00E+00
	UP_SEQ_FEATURE	repeat:3	6	3.40E-01	1.00E+00
	UP_SEQ_FEATURE	repeat:14	3	3.80E-01	1.00E+00
	UP_SEQ_FEATURE	repeat:13	3	4.10E-01	1.00E+00
	UP_SEQ_FEATURE	repeat:4	5	4.10E-01	1.00E+00
	UP_SEQ_FEATURE	repeat:12	3	4.20E-01	1.00E+00
	UP_SEQ_FEATURE	repeat:11	3	4.50E-01	1.00E+00
	UP_SEQ_FEATURE	repeat:10	3	5.10E-01	1.00E+00
	UP_SEQ_FEATURE	repeat:9	3	5.50E-01	1.00E+00
	UP_SEQ_FEATURE	repeat:8	3	6.40E-01	1.00E+00
	UP_SEQ_FEATURE	repeat:7	3	6.80E-01	1.00E+00
	UP_SEQ_FEATURE	repeat:6	3	7.30E-01	1.00E+00
	UP_SEQ_FEATURE	repeat:5	3	7.80E-01	1.00E+00
**Annotation Cluster 38**	**Enrichment Score: 0.37**		**Count**	**P_Value**	**Benjamini**
	GOTERM_BP_FAT	response to cAMP	4	4.30E-02	1.00E+00
	GOTERM_BP_FAT	response to steroid hormone stimulus	6	2.90E-01	1.00E+00
	GOTERM_BP_FAT	response to corticosteroid stimulus	3	4.70E-01	1.00E+00
	GOTERM_BP_FAT	response to endogenous stimulus	9	4.80E-01	1.00E+00
	GOTERM_BP_FAT	response to hormone stimulus	7	6.80E-01	1.00E+00
	GOTERM_BP_FAT	response to metal ion	3	7.00E-01	1.00E+00
	GOTERM_BP_FAT	response to organic substance	11	8.70E-01	1.00E+00
	GOTERM_BP_FAT	response to inorganic substance	3	9.00E-01	1.00E+00
**Annotation Cluster 39**	**Enrichment Score: 0.37**		**Count**	**P_Value**	**Benjamini**
	SP_PIR_KEYWORDS	amino-acid transport	3	1.30E-01	9.00E-01
	GOTERM_BP_FAT	amine transport	4	3.80E-01	1.00E+00
	GOTERM_BP_FAT	amino acid transport	3	5.00E-01	1.00E+00
	GOTERM_BP_FAT	carboxylic acid transport	3	7.60E-01	1.00E+00
	GOTERM_BP_FAT	organic acid transport	3	7.70E-01	1.00E+00
**Annotation Cluster 40**	**Enrichment Score: 0.36**		**Count**	**P_Value**	**Benjamini**
	SP_PIR_KEYWORDS	cilium	3	3.60E-01	9.70E-01
	GOTERM_CC_FAT	cilium	4	4.50E-01	1.00E+00
	SP_PIR_KEYWORDS	cell projection	5	5.10E-01	9.80E-01
**Annotation Cluster 41**	**Enrichment Score: 0.33**		**Count**	**P_Value**	**Benjamini**
	GOTERM_BP_FAT	cell-cell signaling	14	3.30E-01	1.00E+00
	GOTERM_BP_FAT	regulation of neurotransmitter levels	3	3.60E-01	1.00E+00
	GOTERM_BP_FAT	transmission of nerve impulse	7	6.40E-01	1.00E+00
	GOTERM_BP_FAT	synaptic transmission	6	6.60E-01	1.00E+00
**Annotation Cluster 42**	**Enrichment Score: 0.33**		**Count**	**P_Value**	**Benjamini**
	GOTERM_MF_FAT	Ras guanyl-nucleotide exchange factor activity	4	2.20E-01	1.00E+00
	GOTERM_BP_FAT	regulation of G-protein coupled receptor protein signaling pathway	3	2.50E-01	1.00E+00
	UP_SEQ_FEATURE	domain:DH	3	3.40E-01	1.00E+00
	INTERPRO	Dbl homology (DH) domain	3	3.50E-01	1.00E+00
	GOTERM_BP_FAT	regulation of Ras protein signal transduction	6	3.50E-01	1.00E+00
	INTERPRO	Pleckstrin homology	7	3.70E-01	1.00E+00
	SMART	RhoGEF	3	3.70E-01	1.00E+00
	GOTERM_MF_FAT	Rho guanyl-nucleotide exchange factor activity	3	3.90E-01	1.00E+00
	SMART	PH	7	4.20E-01	1.00E+00
	GOTERM_BP_FAT	regulation of small GTPase mediated signal transduction	6	5.10E-01	1.00E+00
	GOTERM_MF_FAT	guanyl-nucleotide exchange factor activity	4	5.30E-01	1.00E+00
	GOTERM_BP_FAT	regulation of Rho protein signal transduction	3	5.50E-01	1.00E+00
	GOTERM_MF_FAT	small GTPase regulator activity	6	5.60E-01	1.00E+00
	GOTERM_MF_FAT	GTPase regulator activity	8	6.10E-01	1.00E+00
	UP_SEQ_FEATURE	domain:PH	5	6.20E-01	1.00E+00
	INTERPRO	Pleckstrin homology-type	6	6.30E-01	1.00E+00
	GOTERM_MF_FAT	nucleoside-triphosphatase regulator activity	8	6.30E-01	1.00E+00
	SP_PIR_KEYWORDS	guanine-nucleotide releasing factor	3	6.40E-01	9.90E-01
	GOTERM_BP_FAT	regulation of hydrolase activity	5	8.80E-01	1.00E+00
	GOTERM_MF_FAT	GTPase activator activity	3	9.10E-01	1.00E+00
**Annotation Cluster 43**	**Enrichment Score: 0.32**		**Count**	**P_Value**	**Benjamini**
	INTERPRO	Glucose/ribitol dehydrogenase	3	2.40E-01	1.00E+00
	INTERPRO	Short-chain dehydrogenase/reductase SDR	3	2.80E-01	1.00E+00
	UP_SEQ_FEATURE	nucleotide phosphate-binding region:NADP	3	3.70E-01	1.00E+00
	INTERPRO	NAD(P)-binding domain	4	5.00E-01	1.00E+00
	SP_PIR_KEYWORDS	oxidoreductase	10	6.80E-01	9.90E-01
	UP_SEQ_FEATURE	binding site:Substrate	5	7.80E-01	1.00E+00
	GOTERM_BP_FAT	oxidation reduction	10	8.50E-01	1.00E+00
**Annotation Cluster 44**	**Enrichment Score: 0.32**		**Count**	**P_Value**	**Benjamini**
	GOTERM_BP_FAT	embryonic organ morphogenesis	5	2.40E-01	1.00E+00
	GOTERM_BP_FAT	embryonic skeletal system morphogenesis	3	2.90E-01	1.00E+00
	GOTERM_BP_FAT	skeletal system morphogenesis	4	3.50E-01	1.00E+00

ILMN_2367070	ACOT9	0.411621491
ILMN_1793959	ADPGK	0.411558185
ILMN_1702585	SET	0.410837391
ILMN_1769520	UBE2L6	0.410491696
ILMN_1660544	ARRDC4	0.410299623
ILMN_1703072	CREB3	0.410127395
ILMN_2328874	GH1	0.40991692
ILMN_2083066	IGLL3P	0.409854039
ILMN_1733318	TEX9	0.409851572
ILMN_1731294	GPR179	0.409843577
ILMN_2353161	MSLN	0.409743974
ILMN_1692276	GGPS1	0.409741762
ILMN_1725338	CLDN23	0.409383367
ILMN_1753716	EIF2B1	0.409269843
ILMN_1692703	CYLC1	0.409159735
ILMN_1697315	GNAT1	0.409157737
ILMN_2124585	GREM1	0.409134098
ILMN_1795662	C16orf82	0.409120346
ILMN_1865559	NA	0.40911403
ILMN_1769245	GLIPR1	0.408664533
ILMN_1745787	POLL	0.408625575
ILMN_1731891	UBXN8	0.408286108
ILMN_1792384	HABP4	0.407685719
ILMN_3179127	C18orf12	0.407681369
ILMN_2198376	PSMA4	0.407664738
ILMN_1785635	BRD3	0.407648554
ILMN_1710989	LOC653061	0.407498806
ILMN_1716276	CCL4	0.407112232
ILMN_1732809	ALG9	0.407003856
ILMN_1781285	DUSP1	0.406902549
ILMN_1656073	MYL12A	0.406726282
ILMN_3307911	AKR1CL1	0.406725627
ILMN_1777637	SLFNL1	0.406696464
ILMN_1664024	APOB	0.406571539
ILMN_1786759	C11orf10	0.40631992
ILMN_2391333	CYP20A1	0.406255936
ILMN_1755383	LRRC1	0.406147992
ILMN_1781886	LOC441179	0.406050934
ILMN_2144574	CTBS	0.405780245
ILMN_3182487	BG722090	0.405548267
ILMN_1672876	MFI2	0.405536726
ILMN_2223887	OTOR	0.405483694
ILMN_1694877	CASP6	0.405220692
ILMN_1751744	ANKLE1	0.405142271
ILMN_1736013	CIB3	0.405040581
ILMN_1797172	ERCC1	0.40492722
ILMN_1825159	BC040587	0.404779777
ILMN_2320513	APBB3	0.404759708
ILMN_1654626	RB1CC1	0.404310246
ILMN_1666173	TCF20	0.404306377
ILMN_1719103	UBE2CBP	0.404270406
ILMN_3247462	GLRX3	0.404262535
ILMN_1873838	IGKC	0.404032974
ILMN_1771870	FAM98C	0.403926193
ILMN_2371169	ZYX	0.403829212
ILMN_1794677	TMC6	0.403565806
ILMN_3221888	NAP1L6	0.403346727
ILMN_1658034	AGAP4	0.403320514
ILMN_1656822	DNM2	0.403249612
ILMN_2337789	MARCH2	0.402882346
ILMN_2258816	SAMD3	0.402753993
ILMN_1809433	XBP1	0.402522842
ILMN_1682894	TMEM191C	0.402225341
ILMN_2246328	PTPN22	0.402101364
ILMN_1798819	DTNB	0.402090183
ILMN_1758731	CYP2J2	0.401988843
ILMN_1713290	GLT8D1	0.401922033
ILMN_1792489	ARPC2	0.401765464
ILMN_1732198	UTS2	0.401759314
ILMN_1699170	C5orf45	0.401731224
ILMN_1661516	POLR2J2	0.401728439
ILMN_1744316	TATDN3	0.401568886
ILMN_1795852	CCNE1	0.401484807
ILMN_3307505	SLC22A24	0.401360557
ILMN_1708427	KPNA3	0.401324064
ILMN_2249720	LAT2	0.401288081
ILMN_2116714	SLC39A1	0.401101157
ILMN_2112049	DNLZ	0.400883692
ILMN_1800530	AGAP1	0.400828558
ILMN_1848913	AF339771	0.400713874
ILMN_2387784	DEAF1	0.400383077
ILMN_1700276	JKAMP	0.40033307
ILMN_1680367	C10orf90	0.400251815
ILMN_1733176	LIMS1	0.400199786

	UP_SEQ_FEATURE	repeat:LRR 9	5	2.70E-01	1.00E+00
	UP_SEQ_FEATURE	repeat:LRR 11	4	3.20E-01	1.00E+00
	UP_SEQ_FEATURE	repeat:LRR 14	3	3.30E-01	1.00E+00
	UP_SEQ_FEATURE	repeat:LRR 10	4	4.00E-01	1.00E+00
	INTERPRO	Leucine-rich repeat	6	4.20E-01	1.00E+00
	UP_SEQ_FEATURE	repeat:LRR 13	3	4.40E-01	1.00E+00
	UP_SEQ_FEATURE	repeat:LRR 12	3	5.40E-01	1.00E+00
	INTERPRO	Leucine-rich repeat, typical subtype	3	7.40E-01	1.00E+00
	SMART	LRR_TYP	3	7.80E-01	1.00E+00
**Annotation Cluster 40**	**Enrichment Score: 0.6**		**Count**	**P_Value**	**Benjamini**
	GOTERM_BP_FAT	macromolecule catabolic process	23	7.10E-02	8.70E-01
	GOTERM_BP_FAT	cellular macromolecule catabolic process	21	9.90E-02	8.90E-01
	GOTERM_BP_FAT	modification-dependent macromolecule catabolic process	15	2.70E-01	9.70E-01
	GOTERM_BP_FAT	modification-dependent protein catabolic process	15	2.70E-01	9.70E-01
	GOTERM_BP_FAT	protein catabolic process	16	2.70E-01	9.70E-01
	GOTERM_BP_FAT	proteolysis	25	3.00E-01	9.70E-01
	GOTERM_BP_FAT	proteolysis involved in cellular protein catabolic process	15	3.30E-01	9.70E-01
	GOTERM_BP_FAT	cellular protein catabolic process	15	3.30E-01	9.70E-01
	GOTERM_BP_FAT	ubiquitin-dependent protein catabolic process	7	3.60E-01	9.80E-01
	SP_PIR_KEYWORDS	ubl conjugation pathway	10	6.70E-01	9.80E-01
**Annotation Cluster 41**	**Enrichment Score: 0.59**		**Count**	**P_Value**	**Benjamini**
	GOTERM_MF_FAT	protein dimerization activity	16	1.30E-01	9.60E-01
	GOTERM_MF_FAT	protein heterodimerization activity	7	2.50E-01	9.80E-01
	GOTERM_MF_FAT	protein homodimerization activity	8	5.20E-01	1.00E+00
**Annotation Cluster 42**	**Enrichment Score: 0.59**		**Count**	**P_Value**	**Benjamini**
	GOTERM_CC_FAT	vacuole	8	2.30E-01	9.40E-01
	GOTERM_CC_FAT	lytic vacuole	7	2.40E-01	9.40E-01
	GOTERM_CC_FAT	lysosome	7	2.40E-01	9.40E-01
	SP_PIR_KEYWORDS	lysosome	5	3.30E-01	9.00E-01
**Annotation Cluster 43**	**Enrichment Score: 0.54**		**Count**	**P_Value**	**Benjamini**
	GOTERM_CC_FAT	nuclear envelope	11	7.70E-03	9.20E-01
	GOTERM_CC_FAT	endomembrane system	24	3.70E-02	8.30E-01
	GOTERM_CC_FAT	organelle envelope	18	1.10E-01	8.70E-01
	GOTERM_CC_FAT	envelope	18	1.10E-01	8.50E-01
	GOTERM_CC_FAT	mitochondrial outer membrane	4	2.60E-01	9.40E-01
	GOTERM_CC_FAT	organelle outer membrane	4	3.40E-01	9.50E-01
	GOTERM_CC_FAT	outer membrane	4	3.60E-01	9.50E-01
	GOTERM_BP_FAT	mitochondrial transport	3	4.10E-01	9.80E-01
	GOTERM_CC_FAT	organelle membrane	24	4.20E-01	9.50E-01
	GOTERM_CC_FAT	mitochondrial membrane	7	8.00E-01	9.90E-01
	GOTERM_CC_FAT	mitochondrion	19	8.20E-01	1.00E+00
	GOTERM_CC_FAT	mitochondrial envelope	7	8.40E-01	1.00E+00
	GOTERM_CC_FAT	organelle inner membrane	5	8.90E-01	1.00E+00
	GOTERM_CC_FAT	mitochondrial inner membrane	4	9.40E-01	1.00E+00
	GOTERM_CC_FAT	mitochondrial part	8	9.50E-01	1.00E+00
**Annotation Cluster 44**	**Enrichment Score: 0.54**		**Count**	**P_Value**	**Benjamini**
	GOTERM_BP_FAT	development of primary male sexual characteristics	4	1.40E-01	9.30E-01
	GOTERM_BP_FAT	reproductive developmental process	9	1.60E-01	9.40E-01
	GOTERM_BP_FAT	male sex differentiation	4	1.80E-01	9.40E-01
	GOTERM_BP_FAT	ovarian follicle development	3	2.00E-01	9.50E-01
	GOTERM_BP_FAT	reproductive structure development	5	2.50E-01	9.60E-01
	GOTERM_BP_FAT	development of primary sexual characteristics	5	2.50E-01	9.60E-01
	GOTERM_BP_FAT	male gonad development	3	2.80E-01	9.70E-01
	GOTERM_BP_FAT	ovulation cycle process	3	3.60E-01	9.80E-01
	GOTERM_BP_FAT	sex differentiation	5	3.60E-01	9.80E-01
	GOTERM_BP_FAT	female gonad development	3	3.70E-01	9.80E-01
	GOTERM_BP_FAT	ovulation cycle	3	3.90E-01	9.80E-01
	GOTERM_BP_FAT	gonad development	4	3.90E-01	9.80E-01
	GOTERM_BP_FAT	development of primary female sexual characteristics	3	4.10E-01	9.80E-01
	GOTERM_BP_FAT	female sex differentiation	3	4.10E-01	9.80E-01
	GOTERM_BP_FAT	rhythmic process	4	4.80E-01	9.90E-01
**Annotation Cluster 45**	**Enrichment Score: 0.51**		**Count**	**P_Value**	**Benjamini**
	GOTERM_BP_FAT	positive regulation of multicellular organism growth	3	9.00E-02	8.90E-01
	GOTERM_BP_FAT	positive regulation of multicellular organismal process	8	2.20E-01	9.50E-01
	GOTERM_BP_FAT	regulation of multicellular organism growth	3	3.50E-01	9.80E-01
	GOTERM_BP_FAT	positive regulation of growth	3	4.80E-01	9.90E-01
	GOTERM_BP_FAT	regulation of growth	6	8.20E-01	1.00E+00
**Annotation Cluster 46**	**Enrichment Score: 0.5**		**Count**	**P_Value**	**Benjamini**
	GOTERM_BP_FAT	intracellular transport	19	1.20E-01	9.10E-01
	GOTERM_BP_FAT	nucleocytoplasmic transport	6	2.10E-01	9.50E-01
	GOTERM_BP_FAT	nuclear transport	6	2.10E-01	9.50E-01
	GOTERM_BP_FAT	protein import into nucleus	4	2.50E-01	9.60E-01
	GOTERM_BP_FAT	nuclear import	4	2.60E-01	9.60E-01
	GOTERM_BP_FAT	protein import	5	2.70E-01	9.70E-01
	GOTERM_BP_FAT	protein transport	19	2.80E-01	9.70E-01
	GOTERM_CC_FAT	pore complex	4	2.90E-01	9.50E-01
	GOTERM_BP_FAT	establishment of protein localization	19	2.90E-01	9.70E-01
	GOTERM_BP_FAT	protein localization in nucleus	4	3.00E-01	9.70E-01
	GOTERM_BP_FAT	cellular protein localization	11	3.20E-01	9.70E-01
	GOTERM_BP_FAT	cellular macromolecule localization	11	3.20E-01	9.70E-01
	GOTERM_BP_FAT	protein localization in organelle	5	3.40E-01	9.80E-01
	GOTERM_BP_FAT	intracellular protein transport	10	3.40E-01	9.80E-01
	GOTERM_BP_FAT	protein localization	20	4.20E-01	9.80E-01
	GOTERM_BP_FAT	protein targeting	6	4.40E-01	9.90E-01
	GOTERM_CC_FAT	nuclear pore	3	4.70E-01	9.60E-01
	GOTERM_MF_FAT	protein transporter activity	3	5.30E-01	1.00E+00
	SP_PIR_KEYWORDS	protein transport	9	7.40E-01	9.90E-01
**Annotation Cluster 47**	**Enrichment Score: 0.5**		**Count**	**P_Value**	**Benjamini**
	GOTERM_BP_FAT	ncRNA metabolic process	9	9.50E-02	8.90E-01
	GOTERM_BP_FAT	tRNA metabolic process	5	2.20E-01	9.50E-01
	GOTERM_BP_FAT	ncRNA processing	6	3.30E-01	9.70E-01
	SP_PIR_KEYWORDS	trna processing	3	3.80E-01	9.20E-01
	GOTERM_BP_FAT	tRNA processing	3	4.60E-01	9.90E-01
	GOTERM_BP_FAT	RNA processing	9	8.70E-01	1.00E+00
**Annotation Cluster 48**	**Enrichment Score: 0.49**		**Count**	**P_Value**	**Benjamini**
	GOTERM_CC_FAT	microtubule organizing center part	4	9.90E-02	8.70E-01
	GOTERM_CC_FAT	microtubule organizing center	7	3.90E-01	9.50E-01
	GOTERM_CC_FAT	centrosome	6	4.60E-01	9.60E-01
	GOTERM_CC_FAT	microtubule cytoskeleton	11	6.50E-01	9.90E-01
**Annotation Cluster 49**	**Enrichment Score: 0.48**		**Count**	**P_Value**	**Benjamini**
	INTERPRO	Immunoglobulin C1-set	4	1.50E-01	1.00E+00
	SMART	IGc1	4	1.80E-01	1.00E+00
	UP_SEQ_FEATURE	domain:Ig-like C1-type	3	1.80E-01	1.00E+00

ILMN_3239440	GAGE12J	-0.498620978
ILMN_1705151	SF3A3	-0.49905569
ILMN_1676997	TREX1	-0.499299762
ILMN_3220532	NA	-0.500834021
ILMN_1709173	HMHB1	-0.501234397
ILMN_1810321	LELP1	-0.50174155
ILMN_1657486	OR2AG2	-0.502128613
ILMN_1865736	LOC729451	-0.502154851
ILMN_1681136	CCDC33	-0.502644953
ILMN_1658710	FAM19A4	-0.503125341
ILMN_1890734	LOC728800	-0.503446465
ILMN_2120210	RCAN2	-0.503736306
ILMN_2342651	OR11H12	-0.503778323
ILMN_1712620	HYAL1	-0.504382704
ILMN_2334350	BTBD3	-0.50526904
ILMN_1812461	WISP2	-0.505650604
ILMN_1703984	TIGD6	-0.507203973
ILMN_1917221	C11orf95	-0.507431219
ILMN_2133090	SDR9C7	-0.508587072
ILMN_1676595	D28514	-0.509240129
ILMN_1706077	DEFB125	-0.510224806
ILMN_1709256	PLEC	-0.510517158
ILMN_1662897	ERCC4	-0.510819735
ILMN_1712530	AKAP1	-0.510964494
ILMN_3238670	SNORA47	-0.514421322
ILMN_1724699	ACAD8	-0.515617146
ILMN_1810834	MSH5	-0.515937514
ILMN_2145423	DET1	-0.51647993
ILMN_3310770	MIR2114	-0.51879106
ILMN_1799717	ZNF398	-0.519114585
ILMN_1698484	RHOV	-0.51954689
ILMN_1720469	BMS1P4	-0.520098002
ILMN_1887177	ODC1	-0.521206241
ILMN_1750625	RSPO4	-0.52124306
ILMN_1878029	AK001020	-0.524101498
ILMN_1656553	LOC653160	-0.524826462
ILMN_2410428	LOC728407	-0.526161999
ILMN_2225151	DLX6	-0.52625173
ILMN_1686637	RBMXL3	-0.529264118
ILMN_1737517	RPL29	-0.529959857
ILMN_3268675	LOC100130880	-0.530107625
ILMN_1761260	COBLL1	-0.530463957
ILMN_1855673	AK027195	-0.531731636
ILMN_1656955	PCDHGC3	-0.53182319
ILMN_1912611	FLJ41130	-0.531824292
ILMN_3189252	PLXNA4	-0.534148487
ILMN_1679782	BLOC1S2	-0.537333163
ILMN_3239748	KRT83	-0.537666675
ILMN_1780041	OR4C3	-0.53784868
ILMN_1909183	CR740230	-0.539205077
ILMN_1892243	AA720941	-0.540135289
ILMN_1744118	ASTN2	-0.54130381
ILMN_1728501	CHRNA9	-0.542777646
ILMN_1738223	C6orf208	-0.544470215
ILMN_1740217	HACE1	-0.546408172
ILMN_1890521	BF109917	-0.549807753
ILMN_2347534	C20orf71	-0.551456754
ILMN_3239775	ODZ4	-0.553806604
ILMN_1806186	P2RX3	-0.555566279
ILMN_1657854	MITF	-0.556979382
ILMN_1767342	EPHA5	-0.559114839
ILMN_1709650	FKBP9L	-0.559601212
ILMN_1795839	SCCPDH	-0.559607785
ILMN_2379560	CDC14B	-0.560037936
ILMN_1779153	KIF18A	-0.560644985
ILMN_1830913	AA484800	-0.561948154
ILMN_2222930	SLC9A11	-0.564267147
ILMN_1692623	LPHN3	-0.568052143
ILMN_1771987	SLC44A2	-0.571042035
ILMN_2070044	PPM1K	-0.573909475
ILMN_1732049	DPM2	-0.57510255
ILMN_1728803	HMP19	-0.585722502
ILMN_1767446	RNF150	-0.58902447
ILMN_3289128	TRMT5	-0.593042989
ILMN_1799086	ZNF643	-0.60043474
ILMN_1708398	SLC25A41	-0.603239438
ILMN_1816501	AI656834	-0.605241699
ILMN_1848374	BU588663	-0.611789209
ILMN_1787595	B3GALNT1	-0.612125818
ILMN_1725899	HOXC10	-0.62792574
ILMN_3233098	KIAA2015	-0.633006248
ILMN_1720484	CRTAP	-0.649169919
ILMN_2343273	FAM70B	-0.651054004
ILMN_1693951	C14orf180	-0.661494443
ILMN_1671326	EIF2C1	-0.681492676

	GOTERM_BP_FAT	embryonic organ development	5	4.00E-01	1.00E+00
	GOTERM_BP_FAT	embryonic skeletal system development	3	4.20E-01	1.00E+00
	GOTERM_BP_FAT	tube development	5	5.90E-01	1.00E+00
	SP_PIR_KEYWORDS	DNA binding	6	7.30E-01	9.90E-01
	GOTERM_BP_FAT	chordate embryonic development	5	8.70E-01	1.00E+00
	GOTERM_BP_FAT	embryonic development ending in birth or egg hatching	5	8.70E-01	1.00E+00
**Annotation Cluster 45**	**Enrichment Score: 0.32**		**Count**	**P_Value**	**Benjamini**
	SP_PIR_KEYWORDS	mrna splicing	7	1.70E-01	9.20E-01
	SP_PIR_KEYWORDS	mrna processing	7	3.20E-01	9.60E-01
	KEGG_PATHWAY	Spliceosome	4	4.30E-01	1.00E+00
	GOTERM_BP_FAT	RNA splicing	7	4.40E-01	1.00E+00
	GOTERM_BP_FAT	mRNA metabolic process	8	5.40E-01	1.00E+00
	GOTERM_BP_FAT	mRNA processing	7	5.50E-01	1.00E+00
	SP_PIR_KEYWORDS	rna-binding	10	6.30E-01	9.90E-01
	GOTERM_MF_FAT	RNA binding	12	7.70E-01	1.00E+00
	GOTERM_BP_FAT	RNA processing	8	8.90E-01	1.00E+00
**Annotation Cluster 46**	**Enrichment Score: 0.32**		**Count**	**P_Value**	**Benjamini**
	GOTERM_CC_FAT	cell fraction	25	2.50E-01	1.00E+00
	GOTERM_CC_FAT	insoluble fraction	16	6.40E-01	1.00E+00
	GOTERM_CC_FAT	membrane fraction	15	6.90E-01	1.00E+00
**Annotation Cluster 47**	**Enrichment Score: 0.32**		**Count**	**P_Value**	**Benjamini**
	GOTERM_BP_FAT	microtubule cytoskeleton organization	8	2.00E-02	1.00E+00
	GOTERM_CC_FAT	spindle microtubule	3	1.10E-01	1.00E+00
	GOTERM_CC_FAT	microtubule	9	1.50E-01	1.00E+00
	GOTERM_CC_FAT	spindle	4	5.30E-01	1.00E+00
	GOTERM_BP_FAT	M phase	7	5.80E-01	1.00E+00
	GOTERM_BP_FAT	cell cycle process	11	6.10E-01	1.00E+00
	GOTERM_BP_FAT	cell division	6	6.50E-01	1.00E+00
	GOTERM_BP_FAT	cell cycle	14	6.90E-01	1.00E+00
	SP_PIR_KEYWORDS	cell division	5	7.00E-01	9.90E-01
	GOTERM_BP_FAT	nuclear division	4	7.80E-01	1.00E+00
	GOTERM_BP_FAT	mitosis	4	7.80E-01	1.00E+00
	GOTERM_BP_FAT	cell cycle phase	7	7.90E-01	1.00E+00
	GOTERM_BP_FAT	M phase of mitotic cell cycle	4	7.90E-01	1.00E+00
	GOTERM_BP_FAT	organelle fission	4	8.00E-01	1.00E+00
	SP_PIR_KEYWORDS	cell cycle	7	8.30E-01	1.00E+00
	SP_PIR_KEYWORDS	mitosis	3	8.40E-01	1.00E+00
	GOTERM_BP_FAT	mitotic cell cycle	5	9.20E-01	1.00E+00
**Annotation Cluster 48**	**Enrichment Score: 0.32**		**Count**	**P_Value**	**Benjamini**
	UP_SEQ_FEATURE	short sequence motif:DEAH box	3	1.70E-01	1.00E+00
	SP_PIR_KEYWORDS	helicase	4	4.30E-01	9.70E-01
	GOTERM_MF_FAT	helicase activity	4	4.70E-01	1.00E+00
	UP_SEQ_FEATURE	domain:Helicase C-terminal	3	5.60E-01	1.00E+00
	INTERPRO	DNA/RNA helicase, C-terminal	3	5.70E-01	1.00E+00
	INTERPRO	DEAD-like helicase, N-terminal	3	5.80E-01	1.00E+00
	UP_SEQ_FEATURE	domain:Helicase ATP-binding	3	5.90E-01	1.00E+00
	SMART	HELICc	3	5.90E-01	1.00E+00
	SMART	DEXDc	3	6.10E-01	1.00E+00
**Annotation Cluster 49**	**Enrichment Score: 0.3**		**Count**	**P_Value**	**Benjamini**
	GOTERM_BP_FAT	RNA export from nucleus	3	1.80E-01	1.00E+00
	GOTERM_BP_FAT	nuclear export	3	3.10E-01	1.00E+00
	GOTERM_BP_FAT	establishment of RNA localization	3	5.40E-01	1.00E+00
	GOTERM_BP_FAT	RNA transport	3	5.40E-01	1.00E+00
	GOTERM_BP_FAT	nucleic acid transport	3	5.40E-01	1.00E+00
	GOTERM_BP_FAT	RNA localization	3	5.60E-01	1.00E+00
	GOTERM_BP_FAT	nucleobase, nucleoside, nucleotide and nucleic acid transport	3	6.30E-01	1.00E+00
	GOTERM_BP_FAT	nucleocytoplasmic transport	3	7.90E-01	1.00E+00
	GOTERM_BP_FAT	nuclear transport	3	8.00E-01	1.00E+00
**Annotation Cluster 50**	**Enrichment Score: 0.3**		**Count**	**P_Value**	**Benjamini**
	INTERPRO	Ion transport	4	3.10E-01	1.00E+00
	GOTERM_CC_FAT	ion channel complex	6	3.50E-01	1.00E+00
	SP_PIR_KEYWORDS	transport	33	3.70E-01	9.70E-01
	GOTERM_MF_FAT	cation channel activity	7	3.90E-01	1.00E+00
	GOTERM_MF_FAT	ion channel activity	9	4.10E-01	1.00E+00
	GOTERM_MF_FAT	ligand-gated channel activity	4	4.10E-01	1.00E+00
	GOTERM_MF_FAT	ligand-gated ion channel activity	4	4.10E-01	1.00E+00
	GOTERM_MF_FAT	substrate specific channel activity	9	4.40E-01	1.00E+00
	GOTERM_BP_FAT	cation transport	12	4.50E-01	1.00E+00
	SP_PIR_KEYWORDS	ion transport	12	4.60E-01	9.70E-01
	GOTERM_CC_FAT	cation channel complex	4	4.60E-01	1.00E+00
	GOTERM_MF_FAT	channel activity	9	4.80E-01	1.00E+00
	GOTERM_MF_FAT	passive transmembrane transporter activity	9	4.80E-01	1.00E+00
	GOTERM_MF_FAT	gated channel activity	7	5.00E-01	1.00E+00
	GOTERM_MF_FAT	voltage-gated cation channel activity	4	5.00E-01	1.00E+00
	SP_PIR_KEYWORDS	ionic channel	7	5.10E-01	9.70E-01
	GOTERM_BP_FAT	monovalent inorganic cation transport	7	5.50E-01	1.00E+00
	GOTERM_MF_FAT	metal ion transmembrane transporter activity	7	5.60E-01	1.00E+00
	GOTERM_BP_FAT	ion transport	15	5.70E-01	1.00E+00
	GOTERM_BP_FAT	potassium ion transport	4	5.80E-01	1.00E+00
	GOTERM_BP_FAT	metal ion transport	9	6.40E-01	1.00E+00
	GOTERM_MF_FAT	voltage-gated ion channel activity	4	6.90E-01	1.00E+00
	GOTERM_MF_FAT	voltage-gated channel activity	4	6.90E-01	1.00E+00
	SP_PIR_KEYWORDS	voltage-gated channel	3	7.50E-01	9.90E-01
	GOTERM_BP_FAT	transmembrane transport	8	9.10E-01	1.00E+00
**Annotation Cluster 51**	**Enrichment Score: 0.28**		**Count**	**P_Value**	**Benjamini**
	GOTERM_BP_FAT	skeletal muscle tissue development	3	3.50E-01	1.00E+00
	GOTERM_BP_FAT	skeletal muscle organ development	3	3.50E-01	1.00E+00
	GOTERM_BP_FAT	striated muscle tissue development	3	6.50E-01	1.00E+00
	GOTERM_BP_FAT	muscle tissue development	3	6.80E-01	1.00E+00
	GOTERM_BP_FAT	muscle organ development	4	7.60E-01	1.00E+00
**Annotation Cluster 52**	**Enrichment Score: 0.27**		**Count**	**P_Value**	**Benjamini**
	GOTERM_BP_FAT	intracellular transport	15	3.40E-01	1.00E+00
	GOTERM_BP_FAT	cellular protein localization	9	5.00E-01	1.00E+00
	GOTERM_BP_FAT	cellular macromolecule localization	9	5.00E-01	1.00E+00
	SP_PIR_KEYWORDS	protein transport	10	5.10E-01	9.80E-01
	GOTERM_BP_FAT	intracellular protein transport	8	5.50E-01	1.00E+00
	GOTERM_BP_FAT	protein transport	15	5.60E-01	1.00E+00
	GOTERM_BP_FAT	protein targeting	5	5.70E-01	1.00E+00
	GOTERM_BP_FAT	establishment of protein localization	15	5.70E-01	1.00E+00
	GOTERM_BP_FAT	protein localization	15	7.70E-01	1.00E+00
**Annotation Cluster 53**	**Enrichment Score: 0.27**		**Count**	**P_Value**	**Benjamini**
	GOTERM_BP_FAT	positive regulation of gene expression	13	4.00E-01	1.00E+00
	GOTERM_BP_FAT	positive regulation of macromolecule biosynthetic process	14	4.50E-01	1.00E+00

	GOTERM_BP_FAT	antigen processing and presentation	4	2.40E-01	9.60E-01
	INTERPRO	Immunoglobulin/major histocompatibility complex, conserved site	4	2.50E-01	1.00E+00
	GOTERM_CC_FAT	MHC protein complex	3	3.10E-01	9.50E-01
	KEGG_PATHWAY	Antigen processing and presentation	3	5.30E-01	9.60E-01
	INTERPRO	Immunoglobulin-like fold	8	9.30E-01	1.00E+00
	INTERPRO	Immunoglobulin-like	7	9.40E-01	1.00E+00
**Annotation Cluster 50**	**Enrichment Score: 0.48**		**Count**	**P_Value**	**Benjamini**
	SP_PIR_KEYWORDS	nuclease	5	1.50E-01	7.80E-01
	GOTERM_MF_FAT	nuclease activity	5	4.00E-01	9.90E-01
	GOTERM_MF_FAT	endonuclease activity	3	6.00E-01	1.00E+00
**Annotation Cluster 51**	**Enrichment Score: 0.47**		**Count**	**P_Value**	**Benjamini**
	SP_PIR_KEYWORDS	rna-binding	16	1.10E-01	7.10E-01
	GOTERM_MF_FAT	RNA binding	17	3.80E-01	9.90E-01
	UP_SEQ_FEATURE	domain:RRM	4	4.10E-01	1.00E+00
	INTERPRO	RNA recognition motif, RNP-1	6	4.10E-01	1.00E+00
	INTERPRO	Nucleotide-binding, alpha-beta plait	6	4.20E-01	1.00E+00
	SMART	RRM	6	4.70E-01	1.00E+00
**Annotation Cluster 52**	**Enrichment Score: 0.47**		**Count**	**P_Value**	**Benjamini**
	GOTERM_BP_FAT	homeostasis of number of cells	6	5.20E-02	8.50E-01
	KEGG_PATHWAY	Intestinal immune network for IgA production	4	8.30E-02	6.60E-01
	GOTERM_BP_FAT	regulation of B cell activation	3	2.80E-01	9.70E-01
	GOTERM_BP_FAT	regulation of lymphocyte activation	5	3.50E-01	9.80E-01
	GOTERM_BP_FAT	regulation of leukocyte activation	5	4.30E-01	9.90E-01
	GOTERM_BP_FAT	positive regulation of T cell activation	3	4.60E-01	9.90E-01
	GOTERM_BP_FAT	regulation of cell activation	5	4.70E-01	9.90E-01
	GOTERM_BP_FAT	positive regulation of lymphocyte activation	3	5.90E-01	1.00E+00
	GOTERM_BP_FAT	positive regulation of leukocyte activation	3	6.30E-01	1.00E+00
	GOTERM_BP_FAT	positive regulation of cell activation	3	6.60E-01	1.00E+00
	GOTERM_BP_FAT	regulation of T cell activation	3	6.90E-01	1.00E+00
**Annotation Cluster 53**	**Enrichment Score: 0.47**		**Count**	**P_Value**	**Benjamini**
	GOTERM_MF_FAT	transcription activator activity	12	2.10E-01	9.80E-01
	GOTERM_MF_FAT	transcription coactivator activity	7	2.70E-01	9.80E-01
	SP_PIR_KEYWORDS	activator	13	3.20E-01	9.00E-01
	GOTERM_MF_FAT	transcription cofactor activity	9	4.50E-01	1.00E+00
	GOTERM_MF_FAT	transcription factor binding	11	5.90E-01	1.00E+00
	GOTERM_BP_FAT	positive regulation of transcription	12	4.80E-01	1.00E+00
	GOTERM_BP_FAT	positive regulation of nucleobase, nucleoside, nucleotide and nucleic acid metabolic process	13	5.00E-01	1.00E+00
	GOTERM_BP_FAT	positive regulation of cellular biosynthetic process	14	5.30E-01	1.00E+00
	GOTERM_BP_FAT	positive regulation of transcription, DNA-dependent	10	5.30E-01	1.00E+00
	GOTERM_BP_FAT	positive regulation of biosynthetic process	14	5.30E-01	1.00E+00
	GOTERM_BP_FAT	positive regulation of nitrogen compound metabolic process	13	5.40E-01	1.00E+00
	GOTERM_BP_FAT	positive regulation of RNA metabolic process	10	5.40E-01	1.00E+00
	GOTERM_BP_FAT	positive regulation of macromolecule metabolic process	16	6.40E-01	1.00E+00
	GOTERM_BP_FAT	positive regulation of transcription from RNA polymerase II promoter	6	8.30E-01	1.00E+00
**Annotation Cluster 54**	**Enrichment Score: 0.26**		**Count**	**P_Value**	**Benjamini**
	GOTERM_BP_FAT	negative regulation of protein modification process	4	3.80E-01	1.00E+00
	GOTERM_BP_FAT	negative regulation of protein ubiquitination	3	4.00E-01	1.00E+00
	GOTERM_BP_FAT	negative regulation of cellular protein metabolic process	5	4.40E-01	1.00E+00
	GOTERM_BP_FAT	negative regulation of protein metabolic process	5	4.60E-01	1.00E+00
	GOTERM_BP_FAT	regulation of protein ubiquitination	3	5.60E-01	1.00E+00
	GOTERM_BP_FAT	negative regulation of molecular function	7	5.90E-01	1.00E+00
	GOTERM_BP_FAT	regulation of protein modification process	6	6.50E-01	1.00E+00
	GOTERM_BP_FAT	negative regulation of catalytic activity	5	7.60E-01	1.00E+00
	GOTERM_BP_FAT	regulation of cellular protein metabolic process	7	8.80E-01	1.00E+00
**Annotation Cluster 55**	**Enrichment Score: 0.26**		**Count**	**P_Value**	**Benjamini**
	GOTERM_BP_FAT	glycoprotein metabolic process	6	3.20E-01	1.00E+00
	GOTERM_BP_FAT	glycoprotein biosynthetic process	4	5.70E-01	1.00E+00
	SP_PIR_KEYWORDS	glycosyltransferase	3	9.00E-01	1.00E+00
**Annotation Cluster 56**	**Enrichment Score: 0.26**		**Count**	**P_Value**	**Benjamini**
	SP_PIR_KEYWORDS	repressor	12	1.50E-01	9.20E-01
	GOTERM_BP_FAT	negative regulation of cellular biosynthetic process	13	3.50E-01	1.00E+00
	GOTERM_BP_FAT	negative regulation of biosynthetic process	13	3.80E-01	1.00E+00
	GOTERM_BP_FAT	negative regulation of macromolecule metabolic process	16	3.90E-01	1.00E+00
	GOTERM_BP_FAT	negative regulation of macromolecule biosynthetic process	12	4.40E-01	1.00E+00
	GOTERM_BP_FAT	negative regulation of transcription from RNA polymerase II promoter	6	5.60E-01	1.00E+00
	GOTERM_BP_FAT	regulation of transcription from RNA polymerase II promoter	14	6.00E-01	1.00E+00
	GOTERM_BP_FAT	negative regulation of nucleobase, nucleoside, nucleotide and nucleic acid metabolic process	10	6.20E-01	1.00E+00
	GOTERM_BP_FAT	negative regulation of nitrogen compound metabolic process	10	6.30E-01	1.00E+00
	GOTERM_MF_FAT	transcription repressor activity	6	6.90E-01	1.00E+00
	GOTERM_BP_FAT	negative regulation of gene expression	9	7.30E-01	1.00E+00
	GOTERM_MF_FAT	transcription corepressor activity	3	7.50E-01	1.00E+00
	GOTERM_BP_FAT	chromatin modification	5	7.50E-01	1.00E+00
	GOTERM_BP_FAT	negative regulation of transcription, DNA-dependent	6	8.00E-01	1.00E+00
	GOTERM_BP_FAT	negative regulation of RNA metabolic process	6	8.10E-01	1.00E+00
	GOTERM_BP_FAT	negative regulation of transcription	7	8.60E-01	1.00E+00
**Annotation Cluster 57**	**Enrichment Score: 0.26**		**Count**	**P_Value**	**Benjamini**
	GOTERM_BP_FAT	negative regulation of signal transduction	6	3.90E-01	1.00E+00
	GOTERM_BP_FAT	negative regulation of cell communication	6	4.90E-01	1.00E+00
	GOTERM_MF_FAT	enzyme activator activity	5	8.60E-01	1.00E+00
**Annotation Cluster 58**	**Enrichment Score: 0.26**		**Count**	**P_Value**	**Benjamini**
	SP_PIR_KEYWORDS	activator	16	4.70E-02	7.80E-01
	SP_PIR_KEYWORDS	nucleus	77	5.40E-01	9.80E-01
	GOTERM_MF_FAT	DNA binding	43	5.60E-01	1.00E+00
	GOTERM_BP_FAT	regulation of RNA metabolic process	33	6.60E-01	1.00E+00
	GOTERM_MF_FAT	transcription regulator activity	27	6.60E-01	1.00E+00
	GOTERM_BP_FAT	regulation of transcription, DNA-dependent	32	6.80E-01	1.00E+00
	SP_PIR_KEYWORDS	dna-binding	32	6.90E-01	9.90E-01
	SP_PIR_KEYWORDS	Transcription	35	7.20E-01	9.90E-01
	SP_PIR_KEYWORDS	transcription regulation	34	7.40E-01	9.90E-01
	GOTERM_BP_FAT	transcription	36	7.90E-01	1.00E+00
	GOTERM_MF_FAT	transcription factor activity	16	7.90E-01	1.00E+00
	GOTERM_BP_FAT	regulation of transcription	44	8.30E-01	1.00E+00
**Annotation Cluster 59**	**Enrichment Score: 0.25**		**Count**	**P_Value**	**Benjamini**
	GOTERM_BP_FAT	chromatin assembly or disassembly	4	4.20E-01	1.00E+00
	GOTERM_BP_FAT	nucleosome assembly	3	4.70E-01	1.00E+00
	GOTERM_BP_FAT	chromatin assembly	3	4.80E-01	1.00E+00
	GOTERM_BP_FAT	protein-DNA complex assembly	3	5.10E-01	1.00E+00
	GOTERM_BP_FAT	nucleosome organization	3	5.20E-01	1.00E+00
	GOTERM_BP_FAT	DNA packaging	3	6.40E-01	1.00E+00
	GOTERM_BP_FAT	cellular macromolecular complex subunit organization	7	6.60E-01	1.00E+00
	GOTERM_BP_FAT	cellular macromolecular complex assembly	6	7.10E-01	1.00E+00
	GOTERM_BP_FAT	chromatin organization	7	7.10E-01	1.00E+00
**Annotation Cluster 60**	**Enrichment Score: 0.25**		**Count**	**P_Value**	**Benjamini**
	GOTERM_CC_FAT	nuclear speck	4	3.10E-01	1.00E+00
	GOTERM_CC_FAT	nuclear body	4	6.30E-01	1.00E+00
	GOTERM_CC_FAT	nucleoplasm part	8	9.10E-01	1.00E+00
**Annotation Cluster 61**	**Enrichment Score: 0.24**		**Count**	**P_Value**	**Benjamini**
	GOTERM_CC_FAT	condensed chromosome	4	4.50E-01	1.00E+00
	GOTERM_BP_FAT	M phase of meiotic cell cycle	3	5.50E-01	1.00E+00
	GOTERM_BP_FAT	meiosis	3	5.50E-01	1.00E+00
	GOTERM_BP_FAT	meiotic cell cycle	3	5.60E-01	1.00E+00
	GOTERM_BP_FAT	M phase	7	5.80E-01	1.00E+00
	GOTERM_BP_FAT	cell cycle phase	7	7.90E-01	1.00E+00
**Annotation Cluster 62**	**Enrichment Score: 0.24**		**Count**	**P_Value**	**Benjamini**
	GOTERM_BP_FAT	carboxylic acid catabolic process	4	3.40E-01	1.00E+00
	GOTERM_BP_FAT	organic acid catabolic process	4	3.40E-01	1.00E+00
	GOTERM_BP_FAT	cellular amino acid catabolic process	3	3.60E-01	1.00E+00
	GOTERM_BP_FAT	amine catabolic process	3	4.30E-01	1.00E+00
	UP_SEQ_FEATURE	transit peptide:Mitochondrion	7	8.50E-01	1.00E+00
	SP_PIR_KEYWORDS	transit peptide	7	8.60E-01	1.00E+00
	GOTERM_CC_FAT	mitochondrial matrix	3	9.30E-01	1.00E+00
	GOTERM_CC_FAT	mitochondrial lumen	3	9.30E-01	1.00E+00
