# Supplementary material for: Intrarenal activation of adaptive immune effectors is associated with tubular damage and impaired renal function in lupus nephritis
Source: Ann Rheum Dis. 2018 Jul 31;77(12):1782–9. doi: 10.1136/annrheumdis-2018-213485 (PMC6241616; doi:10.1136/annrheumdis-2018-213485)
Supplement: Supplementary file 10 [file annrheumdis-2018-213485supp010.pdf]

Supplementary Table 7 : Transcripts and DAVID pathways correlating with expression of CD3 in lupus kidney biopsies

Positive correlation

Transcripts

Identifier	Gene Symbol	Correlation coefficient with CD3
ILMN_2325837	CD3D	1
ILMN_2061043	CD48	0.905183649
ILMN_2261416	CD3D	0.880984568
ILMN_1886655	TCRVB	0.875380574
ILMN_1659800	BCL11A	0.861258356
ILMN_2098126	CCL5	0.853134526
ILMN_1716651	RUNX2	0.839003672
ILMN_3299520	PRKCB	0.838813012
ILMN_1770673	AKNA	0.83418713
ILMN_1665943	MAP4K1	0.830396057
ILMN_1673357	SLA2	0.828638555
ILMN_2208903	CD52	0.824780534
ILMN_2254635	ITGAX	0.811373324
ILMN_1731233	GZMH	0.806929173
ILMN_1769129	CCL19	0.805805201
ILMN_2376204	LTB	0.805671563
ILMN_1806908	PRKCB	0.804641194
ILMN_1794386	IL2RG	0.791218525
ILMN_1786176	CD37	0.791172895
ILMN_1659075	HLA-DOA	0.789961811
ILMN_1750805	ARHGAP30	0.789946281
ILMN_2353732	CD8A	0.788912749
ILMN_1768284	P2RY8	0.785638873
ILMN_2340217	PTPRC	0.785485884
ILMN_3214389	HLA-DQB1	0.785281832
ILMN_1691341	IL7R	0.784804795
ILMN_1763452	EVI2B	0.783038697
ILMN_2376205	LTB	0.780897502
ILMN_1738675	PTPN6	0.780207003
ILMN_2393765	IGLL1	0.78007563
ILMN_2157441	HLA-DRA	0.779288567
ILMN_2196550	C13orf18	0.778303127
ILMN_1804601	DKFZp686O162	0.775626129
ILMN_2400947	CELF2	0.775040816
ILMN_2143314	SPIB	0.773692781
ILMN_1768482	CD8A	0.771129432
ILMN_2309848	FXYD5	0.77024314
ILMN_1704286	FXYD5	0.770242943
ILMN_2375825	CD37	0.769482681
ILMN_1684887	SAMSN1	0.767725215
ILMN_1676924	CD247	0.767126598
ILMN_1695025	CD2	0.766718855
ILMN_1700428	HLA-DOB	0.765918713
ILMN_1697554	SASH3	0.764417308
ILMN_2208413	ARHGAP15	0.763687084
ILMN_1754507	IRF4	0.763024234
ILMN_1739794	CD3E	0.758439012
ILMN_1811049	POU2AF1	0.755417112
ILMN_1682993	NKG7	0.755323021
ILMN_1710734	GZMK	0.753460918
ILMN_1653652	PTPRC	0.752709707
ILMN_1803686	ADA	0.752455696
ILMN_1677505	CCL21	0.752131602
ILMN_1762594	NOD2	0.751679484
ILMN_1773388	C13orf18	0.749302367
ILMN_1779257	CD40	0.748078332
ILMN_2114568	GBP5	0.747336346
ILMN_1781626	C1S	0.746396111
ILMN_2083066	IGLL3P	0.745838169
ILMN_1802106	APOBEC3G	0.7452804
ILMN_1724422	SELL	0.74420065
ILMN_2246328	PTPN22	0.743352305
ILMN_2175912	ITGB2	0.743016506
ILMN_1688959	CD27	0.742592537
ILMN_1739508	abParts	0.742273943
ILMN_1685625	UCP2	0.741491632
ILMN_1662799	GPSM3	0.741472537
ILMN_2412192	CFH	0.741262487
ILMN_1779324	GZMA	0.74074612
ILMN_2093343	PLAC8	0.737857748
ILMN_1713561	C20orf103	0.737266474
ILMN_3243238	TPSAB1	0.737163562
ILMN_1761733	HLA-DMB	0.736745071
ILMN_1745356	CXCL9	0.735582717
ILMN_1652631	GLIPR2	0.734206859
ILMN_1695851	PARVG	0.733408816
ILMN_1760509	EOMES	0.732844369
ILMN_2120965	NPAT	0.727578708
ILMN_2168217	GPR183	0.727406789
ILMN_1797822	SEL1L3	0.727006476
ILMN_2345353	LST1	0.726210713
ILMN_1772359	LAPTM5	0.726004222
ILMN_1680274	abParts	0.725307949
ILMN_1733579	EVI2A	0.725160501
ILMN_1763487	CTLA4	0.725124087
ILMN_1717261	HLA-DRB1	0.724374021

Pathways

**Annotation Cluster 1**	**Enrichment Score: 11.22**		**Count**	**P_Value**	**Benjamini**
	SP_PIR_KEYWORDS	ribosome	42	5.40E-21	1.20E-18
	GOTERM_BP_FAT	translational elongation	49	2.00E-18	8.60E-15
	GOTERM_CC_FAT	cytosolic ribosome	42	3.50E-17	2.30E-14
	SP_PIR_KEYWORDS	protein biosynthesis	64	1.10E-16	1.00E-14
	KEGG_PATHWAY	Ribosome	44	6.90E-15	1.10E-12
	GOTERM_CC_FAT	cytosolic part	55	8.50E-14	1.80E-11
	GOTERM_CC_FAT	ribosomal subunit	48	8.70E-13	1.40E-10
	GOTERM_MF_FAT	structural molecule activity	132	1.10E-10	1.30E-07
	GOTERM_MF_FAT	structural constituent of ribosome	52	1.30E-10	7.90E-08
	SP_PIR_KEYWORDS	ribonucleoprotein	69	2.00E-10	1.30E-08
	GOTERM_CC_FAT	cytosolic small ribosomal subunit	22	8.30E-10	8.90E-08
	GOTERM_BP_FAT	translation	81	1.10E-09	3.90E-07
	GOTERM_CC_FAT	ribonucleoprotein complex	111	2.10E-09	1.70E-07
	SP_PIR_KEYWORDS	ribosomal protein	51	2.10E-09	1.10E-07
	GOTERM_CC_FAT	cytosolic large ribosomal subunit	20	1.50E-08	1.10E-06
	GOTERM_CC_FAT	ribosome	57	2.20E-08	1.40E-06
	GOTERM_CC_FAT	large ribosomal subunit	26	1.30E-07	7.10E-06
	GOTERM_CC_FAT	small ribosomal subunit	22	1.10E-05	3.60E-04
**Annotation Cluster 2**	**Enrichment Score: 9.44**		**Count**	**P_Value**	**Benjamini**
	GOTERM_BP_FAT	cell activation	84	1.90E-14	2.70E-11
	GOTERM_BP_FAT	leukocyte activation	74	6.90E-14	7.40E-11
	GOTERM_BP_FAT	lymphocyte activation	62	3.70E-12	3.20E-09
	GOTERM_BP_FAT	T cell activation	43	4.90E-10	3.40E-07
	GOTERM_BP_FAT	leukocyte differentiation	44	5.30E-10	2.80E-07
	GOTERM_BP_FAT	hemopoiesis	63	2.80E-09	7.40E-07
	GOTERM_BP_FAT	hemopoietic or lymphoid organ development	67	3.90E-09	9.80E-07
	GOTERM_BP_FAT	immune system development	68	2.10E-08	3.40E-06
	GOTERM_BP_FAT	lymphocyte differentiation	35	2.90E-08	4.30E-06
	GOTERM_BP_FAT	T cell differentiation	23	4.90E-06	3.40E-04
**Annotation Cluster 3**	**Enrichment Score: 8.81**		**Count**	**P_Value**	**Benjamini**
	GOTERM_BP_FAT	defense response	129	5.90E-10	2.50E-07
	GOTERM_BP_FAT	inflammatory response	80	1.00E-09	4.00E-07
	GOTERM_BP_FAT	response to wounding	112	6.00E-09	1.30E-06
**Annotation Cluster 4**	**Enrichment Score: 8.14**		**Count**	**P_Value**	**Benjamini**
	GOTERM_BP_FAT	programmed cell death	127	1.50E-09	4.80E-07
	GOTERM_BP_FAT	apoptosis	125	2.20E-09	6.10E-07
	SP_PIR_KEYWORDS	Apoptosis	82	4.30E-09	2.00E-07
	GOTERM_BP_FAT	regulation of apoptosis	154	9.00E-09	1.90E-06
	GOTERM_BP_FAT	cell death	141	9.10E-09	1.80E-06
	GOTERM_BP_FAT	death	141	1.40E-08	2.60E-06
	GOTERM_BP_FAT	regulation of programmed cell death	154	1.80E-08	3.10E-06
	GOTERM_BP_FAT	regulation of cell death	154	2.30E-08	3.70E-06
**Annotation Cluster 5**	**Enrichment Score: 7.39**		**Count**	**P_Value**	**Benjamini**
	GOTERM_CC_FAT	intracellular non-membrane-bounded organelle	416	1.90E-10	2.50E-08
	GOTERM_CC_FAT	non-membrane-bounded organelle	416	1.90E-10	2.50E-08
	GOTERM_CC_FAT	cytoskeleton	205	1.90E-03	3.50E-02
**Annotation Cluster 6**	**Enrichment Score: 6.51**		**Count**	**P_Value**	**Benjamini**
	GOTERM_BP_FAT	cell adhesion	133	1.80E-07	2.10E-05
	GOTERM_BP_FAT	biological adhesion	133	1.90E-07	2.20E-05
	SP_PIR_KEYWORDS	cell adhesion	81	8.80E-07	2.90E-05
**Annotation Cluster 7**	**Enrichment Score: 6.44**		**Count**	**P_Value**	**Benjamini**
	SP_PIR_KEYWORDS	ubl conjugation	118	1.30E-10	9.60E-09
	SP_PIR_KEYWORDS	isopeptide bond	62	1.30E-05	2.60E-04
	UP_SEQ_FEATURE	cross-link:Glycyl lysine isopeptide (Lys-Gly) (interchain with G-Cter in ubiquitin)	43	2.90E-05	1.50E-02
**Annotation Cluster 8**	**Enrichment Score: 6.16**		**Count**	**P_Value**	**Benjamini**
	GOTERM_CC_FAT	nuclear lumen	252	1.70E-09	1.60E-07
	GOTERM_CC_FAT	organelle lumen	291	3.60E-07	1.70E-05
	GOTERM_CC_FAT	membrane-enclosed lumen	293	1.00E-06	4.50E-05
	GOTERM_CC_FAT	nucleoplasm	156	1.30E-06	5.30E-05
	GOTERM_CC_FAT	intracellular organelle lumen	279	3.60E-06	1.30E-04
	GOTERM_CC_FAT	nucleolus	122	3.70E-05	1.20E-03
**Annotation Cluster 9**	**Enrichment Score: 5.63**		**Count**	**P_Value**	**Benjamini**
	GOTERM_BP_FAT	regulation of protein kinase cascade	64	1.00E-08	2.00E-06
	GOTERM_BP_FAT	positive regulation of protein kinase cascade	44	1.30E-06	1.20E-04
	GOTERM_BP_FAT	positive regulation of cell communication	70	4.40E-06	3.00E-04
	GOTERM_BP_FAT	positive regulation of I-kappaB kinase/NF-kappaB cascade	29	9.10E-06	5.60E-04
	GOTERM_BP_FAT	positive regulation of signal transduction	63	1.20E-05	7.10E-04
	GOTERM_BP_FAT	regulation of I-kappaB kinase/NF-kappaB cascade	30	2.40E-05	1.20E-03
**Annotation Cluster 10**	**Enrichment Score: 5.35**		**Count**	**P_Value**	**Benjamini**
	GOTERM_BP_FAT	regulation of apoptosis	154	9.00E-09	1.90E-06
	GOTERM_BP_FAT	regulation of programmed cell death	154	1.80E-08	3.10E-06
	GOTERM_BP_FAT	regulation of cell death	154	2.30E-08	3.70E-06
	GOTERM_BP_FAT	positive regulation of apoptosis	89	6.70E-07	7.10E-05
	GOTERM_BP_FAT	positive regulation of programmed cell death	89	9.10E-07	9.20E-05
	GOTERM_BP_FAT	positive regulation of cell death	89	1.10E-06	1.10E-04
	GOTERM_BP_FAT	negative regulation of apoptosis	70	5.60E-05	2.30E-03
	GOTERM_BP_FAT	anti-apoptosis	46	7.10E-05	2.80E-03
	GOTERM_BP_FAT	induction of apoptosis	64	8.40E-05	3.20E-03
	GOTERM_BP_FAT	negative regulation of programmed cell death	70	8.70E-05	3.30E-03
	GOTERM_BP_FAT	induction of programmed cell death	64	9.20E-05	3.40E-03
	GOTERM_BP_FAT	negative regulation of cell death	70	9.60E-05	3.40E-03
	GOTERM_BP_FAT	induction of apoptosis by extracellular signals	28	3.80E-04	1.00E-02
**Annotation Cluster 11**	**Enrichment Score: 5.3**		**Count**	**P_Value**	**Benjamini**
	GOTERM_BP_FAT	negative regulation of macromolecule metabolic process	148	5.10E-10	3.10E-07
	GOTERM_BP_FAT	negative regulation of gene expression	98	3.20E-06	2.60E-04
	GOTERM_BP_FAT	negative regulation of nucleobase, nucleoside, nucleotide and nucleic acid metabolic process	99	3.70E-06	2.70E-04

Negative correlation

Transcripts

Identifier	Gene Symbol	Correlation coefficient with CD3
ILMN_1726667	LOC728758	-0.40000032
ILMN_1767032	DDI1	-0.400066325
ILMN_3307863	TAOK3	-0.400088253
ILMN_1840934	AJ420430	-0.400098378
ILMN_3235584	TRIM66	-0.400113232
ILMN_2145760	YIPF4	-0.400116748
ILMN_2146648	PRB3	-0.400169733
ILMN_1659285	PSMG1	-0.400176912
ILMN_1907812	BX119471	-0.400196296
ILMN_3248032	NCCRP1	-0.40021094
ILMN_1660730	SLC7A3	-0.400261553
ILMN_2295698	AFG3L1	-0.400443702
ILMN_1752283	ITCH	-0.400444333
ILMN_1767178	LOC146880	-0.400491102
ILMN_1709142	PURA	-0.400693024
ILMN_1813350	HSD11B2	-0.400701446
ILMN_2278518	NFATC2IP	-0.400724215
ILMN_1670918	INSR	-0.40077098
ILMN_1652164	UNC93B1	-0.400807543
ILMN_1879135	BM673749	-0.401053769
ILMN_1727272	CCT8L2	-0.401068379
ILMN_1675523	PPAP2C	-0.401219234
ILMN_1673537	WFDC3	-0.40142833
ILMN_1756311	UFSP2	-0.401502034
ILMN_2415949	MRRF	-0.40159747
ILMN_2246188	SLC45A2	-0.401677326
ILMN_1776325	UBE2Q1	-0.401841236
ILMN_2395506	SOX6	-0.401852901
ILMN_1742775	ZNRF1	-0.401857034
ILMN_3238793	C9orf129	-0.40193216
ILMN_1772873	CPLX2	-0.401938593
ILMN_3226405	PRAMEF11	-0.402033476
ILMN_1907861	BM850770	-0.402129106
ILMN_1808956	C15orf27	-0.402167288
ILMN_1743441	GSTTP2	-0.402186142
ILMN_1706065	FAM182A	-0.402215847
ILMN_1700042	TLN2	-0.402283739
ILMN_1849418	AW661821	-0.40233352
ILMN_3294277	CYP4F24P	-0.402349711
ILMN_2208373	TMEM164	-0.402532748
ILMN_1733035	MZT2A	-0.40257141
ILMN_1658152	MAP3K10	-0.402590451
ILMN_1721339	ZNF276	-0.402627478
ILMN_1812461	WISP2	-0.40264155
ILMN_2134888	TUBE1	-0.402689098
ILMN_1726273	ATP6V1G3	-0.402792831
ILMN_1788267	PPP1R14D	-0.402876165
ILMN_2206224	OR4M2	-0.402891662
ILMN_1712943	CCK	-0.40290978
ILMN_1779597	CIDEC	-0.402971794
ILMN_1804907	OR1F2	-0.403000541
ILMN_1851487	AI051233	-0.403002651
ILMN_1704176	PSKH2	-0.403037338
ILMN_1690689	AK027883	-0.403054195
ILMN_3185784	LOC100125556	-0.403106213
ILMN_3250143	REPS2	-0.403140696
ILMN_1652188	EPG5	-0.403154388
ILMN_1850372	AI798827	-0.403338422
ILMN_3243578	LOC348120	-0.403358976
ILMN_1759879	LOC220115	-0.403403636
ILMN_1806710	ESPN	-0.403423836
ILMN_3182712	NA	-0.40345686
ILMN_1724211	GPRC5C	-0.403517298
ILMN_1727798	MOSPD1	-0.403533644
ILMN_1659224	FMN1	-0.403546244
ILMN_2154654	PTP4A1	-0.403661681
ILMN_3311045	MIR216A	-0.403738395
ILMN_3247792	LOC100169752	-0.403766568
ILMN_3291253	SBF1P1	-0.403821065
ILMN_3239946	LOC729375	-0.403829371
ILMN_1764090	AK4	-0.404040144
ILMN_3251751	MFSD8	-0.404194901
ILMN_1874673	BM681652	-0.404243205
ILMN_1727606	C12orf59	-0.404301321
ILMN_1771538	PSCA	-0.404331358
ILMN_1841564	CD517297	-0.404352664
ILMN_1719199	TULP3	-0.40446544
ILMN_1735798	OSBPL3	-0.404517057
ILMN_1749612	IMMP1L	-0.404533366
ILMN_1810532	CAV3	-0.404603346
ILMN_2104141	FGD5	-0.404645123
ILMN_3269324	AK094963	-0.404848263
ILMN_3241218	ANKIB1	-0.404869986
ILMN_3284127	TRIM64B	-0.404885171
ILMN_3282032	USP17L2	-0.404928903
ILMN_1706225	N4BP1	-0.404993058

Pathways

**Annotation Cluster 1**	**Enrichment Score: 8.3**		**Count**	**P_Value**	**Benjamini**
	SP_PIR_KEYWORDS	oxidoreductase	98	1.10E-11	3.90E-09
	GOTERM_BP_FAT	oxidation reduction	105	3.70E-10	1.40E-06
	GOTERM_MF_FAT	electron carrier activity	40	3.10E-05	5.90E-03
**Annotation Cluster 2**	**Enrichment Score: 7.1**		**Count**	**P_Value**	**Benjamini**
	SP_PIR_KEYWORDS	mitochondrion	143	3.20E-16	2.30E-13
	GOTERM_CC_FAT	mitochondrion	176	1.60E-14	8.80E-12
	SP_PIR_KEYWORDS	transit peptide	86	3.60E-11	8.40E-09
	UP_SEQ_FEATURE	transit peptide:Mitochondrion	84	1.00E-10	4.10E-07
	GOTERM_CC_FAT	mitochondrial part	98	1.00E-08	2.90E-06
	GOTERM_CC_FAT	mitochondrial envelope	73	1.10E-07	2.00E-05
	GOTERM_CC_FAT	organelle inner membrane	59	8.10E-07	1.10E-04
	GOTERM_CC_FAT	mitochondrial inner membrane	56	8.40E-07	9.40E-05
	GOTERM_CC_FAT	mitochondrial membrane	66	2.00E-06	1.90E-04
	SP_PIR_KEYWORDS	mitochondrion inner membrane	35	3.60E-05	4.20E-03
	GOTERM_CC_FAT	organelle envelope	86	9.60E-05	6.70E-03
	GOTERM_CC_FAT	envelope	86	1.10E-04	6.70E-03
	GOTERM_CC_FAT	mitochondrial membrane part	23	2.10E-03	8.70E-02
	GOTERM_CC_FAT	organelle membrane	121	2.00E-02	4.00E-01
**Annotation Cluster 3**	**Enrichment Score: 4.83**		**Count**	**P_Value**	**Benjamini**
	GOTERM_MF_FAT	carboxylic acid binding	35	1.20E-07	5.10E-05
	GOTERM_MF_FAT	monocarboxylic acid binding	18	2.80E-06	9.00E-04
	GOTERM_MF_FAT	fatty acid binding	13	7.80E-05	1.10E-02
	GOTERM_MF_FAT	acyl-CoA binding	7	1.90E-03	1.70E-01
**Annotation Cluster 4**	**Enrichment Score: 4.54**		**Count**	**P_Value**	**Benjamini**
	GOTERM_CC_FAT	mitochondrial part	98	1.00E-08	2.90E-06
	GOTERM_CC_FAT	mitochondrial lumen	36	1.50E-03	6.90E-02
	GOTERM_CC_FAT	mitochondrial matrix	36	1.50E-03	6.90E-02
**Annotation Cluster 5**	**Enrichment Score: 4.34**		**Count**	**P_Value**	**Benjamini**
	GOTERM_BP_FAT	coenzyme metabolic process	34	1.00E-06	9.50E-04
	GOTERM_BP_FAT	cofactor metabolic process	39	2.30E-06	1.70E-03
	GOTERM_BP_FAT	cofactor biosynthetic process	20	7.10E-04	8.00E-02
	GOTERM_BP_FAT	coenzyme biosynthetic process	15	2.40E-03	1.70E-01
**Annotation Cluster 6**	**Enrichment Score: 4.25**		**Count**	**P_Value**	**Benjamini**
	GOTERM_BP_FAT	carboxylic acid catabolic process	30	6.30E-08	7.70E-05
	GOTERM_BP_FAT	organic acid catabolic process	30	6.30E-08	7.70E-05
	GOTERM_BP_FAT	fatty acid metabolic process	37	2.10E-05	1.10E-02
	GOTERM_BP_FAT	amine catabolic process	20	3.40E-05	1.50E-02
	GOTERM_BP_FAT	fatty acid catabolic process	13	3.50E-05	1.30E-02
	GOTERM_BP_FAT	lipid oxidation	13	8.60E-05	2.10E-02
	GOTERM_BP_FAT	fatty acid oxidation	13	8.60E-05	2.10E-02
	GOTERM_BP_FAT	cellular amino acid catabolic process	17	2.20E-04	3.80E-02
	GOTERM_BP_FAT	cellular lipid catabolic process	18	2.70E-04	4.30E-02
	GOTERM_BP_FAT	lipid catabolic process	29	1.20E-03	1.00E-01
	GOTERM_BP_FAT	lipid modification	15	2.40E-03	1.70E-01
	GOTERM_BP_FAT	fatty acid beta-oxidation	8	9.20E-03	3.30E-01
**Annotation Cluster 7**	**Enrichment Score: 3.94**		**Count**	**P_Value**	**Benjamini**
	KEGG_PATHWAY	Propanoate metabolism	14	5.40E-06	1.00E-03
	KEGG_PATHWAY	Valine, leucine and isoleucine degradation	15	6.10E-05	5.80E-03
	KEGG_PATHWAY	beta-Alanine metabolism	8	4.50E-03	1.10E-01
**Annotation Cluster 8**	**Enrichment Score: 3.46**		**Count**	**P_Value**	**Benjamini**
	GOTERM_BP_FAT	glucose metabolic process	30	6.10E-05	1.70E-02
	GOTERM_BP_FAT	monosaccharide metabolic process	38	1.10E-04	2.30E-02
	GOTERM_BP_FAT	hexose metabolic process	33	3.20E-04	4.80E-02
	GOTERM_BP_FAT	cellular carbohydrate biosynthetic process	14	6.60E-03	2.70E-01
**Annotation Cluster 9**	**Enrichment Score: 3.1**		**Count**	**P_Value**	**Benjamini**
	GOTERM_BP_FAT	monovalent inorganic cation transport	53	9.00E-06	5.40E-03
	GOTERM_BP_FAT	ion transport	101	3.90E-05	1.30E-02
	SP_PIR_KEYWORDS	ion transport	78	5.20E-05	5.20E-03
	GOTERM_BP_FAT	cation transport	77	5.80E-05	1.80E-02
	SP_PIR_KEYWORDS	transport	186	8.70E-05	6.70E-03
	SP_PIR_KEYWORDS	Sodium	21	1.80E-03	8.60E-02
	SP_PIR_KEYWORDS	Sodium transport	21	1.80E-03	8.60E-02
	SP_PIR_KEYWORDS	Symport	20	2.50E-03	1.10E-01
	GOTERM_MF_FAT	sodium ion binding	21	3.70E-03	2.80E-01
	GOTERM_BP_FAT	transmembrane transport	70	3.80E-03	2.20E-01
	GOTERM_MF_FAT	symporter activity	23	5.30E-03	3.00E-01
	GOTERM_BP_FAT	metal ion transport	58	6.60E-03	2.80E-01
	GOTERM_BP_FAT	sodium ion transport	21	9.80E-03	3.40E-01
	GOTERM_MF_FAT	alkali metal ion binding	32	1.30E-02	4.70E-01
**Annotation Cluster 10**	**Enrichment Score: 2.8**		**Count**	**P_Value**	**Benjamini**
	GOTERM_CC_FAT	microbody	21	9.60E-04	5.20E-02
	GOTERM_CC_FAT	peroxisome	21	9.60E-04	5.20E-02
	UP_SEQ_FEATURE	short sequence motif:Microbody targeting signal	11	1.90E-03	7.90E-01
	SP_PIR_KEYWORDS	peroxisome	17	3.60E-03	1.30E-01
**Annotation Cluster 11**	**Enrichment Score: 2.55**		**Count**	**P_Value**	**Benjamini**
	PIR_SUPERFAMILY	PIRSF000092:short-chain dehydrogenase	7	7.10E-04	3.90E-01
	INTERPRO	NAD(P)-binding domain	26	1.30E-03	9.20E-01
	INTERPRO	Short-chain dehydrogenase/reductase SDR	13	4.50E-03	8.40E-01
	INTERPRO	Glucose/ribitol dehydrogenase	11	1.50E-02	8.90E-01
**Annotation Cluster 12**	**Enrichment Score: 2.46**		**Count**	**P_Value**	**Benjamini**
	SMART	ALBUMIN	4	1.60E-03	3.90E-01
	UP_SEQ_FEATURE	domain:Albumin 2	4	2.30E-03	7.90E-01
	UP_SEQ_FEATURE	domain:Albumin 3	4	2.30E-03	7.90E-01
	UP_SEQ_FEATURE	domain:Albumin 1	4	2.30E-03	7.90E-01
	INTERPRO	Serum albumin, N-terminal	4	2.50E-03	9.20E-01

ILMN_1659913	ISG20	0.720788802
ILMN_1686623	CSF1R	0.719386116
ILMN_1689655	HLA-DRA	0.719306076
ILMN_3240316	TMSB4X	0.718480782
ILMN_1747593	ISLR	0.718048697
ILMN_1752526	RNF144B	0.71789487
ILMN_1775542	FAIM3	0.717776151
ILMN_2163819	KIF21B	0.717569435
ILMN_1654319	HAPLN3	0.714474123
ILMN_2320888	CXCR4	0.714409677
ILMN_1790529	LUM	0.713300466
ILMN_1768391	ARL4C	0.711079313
ILMN_2399264	sept-06	0.710317335
ILMN_2214232	AIM2	0.710313019
ILMN_1794473	PHF17	0.710199357
ILMN_2377669	CD247	0.709789075
ILMN_1695311	HLA-DMA	0.709740523
ILMN_2388701	ST3GAL5	0.708362039
ILMN_1713751	ADAM19	0.708012808
ILMN_2088124	TMEM154	0.706145943
ILMN_1655935	ADCY7	0.706143133
ILMN_1671565	RNASET2	0.705882766
ILMN_3232921	CCR2	0.705590779
ILMN_1693233	KIAA0513	0.702123676
ILMN_2258816	SAMD3	0.700768439
ILMN_1815734	FCHSD2	0.699476934
ILMN_2396991	HCST	0.698467271
ILMN_1793672	SIX5	0.698307293
ILMN_1717197	CD3G	0.697920802
ILMN_1808395	ACAP1	0.697819799
ILMN_2109489	GZMB	0.697080181
ILMN_1727284	CD4	0.696722503
ILMN_1758315	SLC9A9	0.696684321
ILMN_1791211	DOK2	0.695659933
ILMN_1738116	TMEM119	0.693779927
ILMN_2125747	CORO1A	0.693492562
ILMN_1716563	PRKCB	0.692591185
ILMN_1670256	PDK1	0.692228005
ILMN_2313926	CDC42SE2	0.691505896
ILMN_1713749	CORO1A	0.69140047
ILMN_3240375	abParts	0.689921115
ILMN_1699931	HCST	0.688834736
ILMN_2188333	CD69	0.688698809
ILMN_2366330	FERMT3	0.688531109
ILMN_1667081	CCND2	0.687282488
ILMN_1752755	VWF	0.685711671
ILMN_1752592	HLA-DRB4	0.685537809
ILMN_2058251	VIM	0.685507019
ILMN_1652199	abParts	0.685216126
ILMN_2329165	MYO1C	0.684392479
ILMN_1696591	RB1	0.68437244
ILMN_1685122	COL9A2	0.684001701
ILMN_1801504	RUNX1	0.683528319
ILMN_1737015	RPL39	0.683453724
ILMN_1673108	AP2A1	0.683438812
ILMN_1726392	NIN	0.683376712
ILMN_1733538	RGS10	0.683216712
ILMN_1807825	LY86	0.682094979
ILMN_1672611	CDH11	0.681989008
ILMN_1798006	ANKRD35	0.681797187
ILMN_1682312	CYBB	0.681573994
ILMN_1664099	GLIS3	0.681475792
ILMN_1794677	TMC6	0.680457575
ILMN_1683271	TMSB4X	0.67790693
ILMN_1711566	TIMP1	0.677063212
ILMN_1666594	IRF8	0.676614114
ILMN_1751034	ITPRIPL2	0.676380723
ILMN_1658327	BAZ1A	0.676036771
ILMN_2280548	FYB	0.675307672
ILMN_1724407	TACC3	0.674051763
ILMN_1781155	LYN	0.673841371
ILMN_1664464	PTGDS	0.673478597
ILMN_1665219	LTBP4	0.673190304

	GOTERM_BP_FAT	negative regulation of nitrogen compound metabolic process	100	3.80E-06	2.80E-04
	GOTERM_BP_FAT	negative regulation of macromolecule biosynthetic process	104	4.40E-06	3.10E-04
	GOTERM_BP_FAT	negative regulation of RNA metabolic process	75	5.40E-06	3.60E-04
	SP_PIR_KEYWORDS	repressor	79	1.00E-05	2.30E-04
	GOTERM_BP_FAT	negative regulation of transcription, DNA-dependent	73	1.10E-05	6.30E-04
	GOTERM_MF_FAT	transcription repressor activity	65	1.40E-05	1.90E-03
	GOTERM_BP_FAT	negative regulation of cellular biosynthetic process	104	1.40E-05	7.70E-04
	GOTERM_BP_FAT	negative regulation of transcription from RNA polymerase II promoter	58	1.50E-05	8.10E-04
	GOTERM_BP_FAT	negative regulation of transcription	88	1.90E-05	9.90E-04
	GOTERM_BP_FAT	negative regulation of biosynthetic process	105	2.10E-05	1.10E-03
	GOTERM_BP_FAT	regulation of transcription from RNA polymerase II promoter	124	1.00E-04	3.60E-03
**Annotation Cluster 12**	**Enrichment Score: 5.28**		**Count**	**P_Value**	**Benjamini**
	GOTERM_BP_FAT	positive regulation of immune system process	65	5.50E-10	2.60E-07
	GOTERM_BP_FAT	positive regulation of response to stimulus	63	2.80E-09	7.40E-07
	GOTERM_BP_FAT	positive regulation of immune response	43	5.20E-08	7.40E-06
	GOTERM_BP_FAT	immune response-regulating signal transduction	22	1.20E-06	1.10E-04
	GOTERM_BP_FAT	immune response-regulating cell surface receptor signaling pathway	18	3.70E-06	2.80E-04
	GOTERM_BP_FAT	activation of immune response	28	1.40E-05	7.90E-04
	GOTERM_BP_FAT	immune response-activating signal transduction	19	2.50E-05	1.20E-03
	GOTERM_BP_FAT	antigen receptor-mediated signaling pathway	14	7.20E-05	2.80E-03
	GOTERM_BP_FAT	immune response-activating cell surface receptor signaling pathway	15	1.30E-04	4.20E-03
	GOTERM_BP_FAT	T cell receptor signaling pathway	9	4.50E-03	7.70E-02
	GOTERM_BP_FAT	B cell receptor signaling pathway	5	1.70E-02	2.10E-01
**Annotation Cluster 13**	**Enrichment Score: 4.67**		**Count**	**P_Value**	**Benjamini**
	KEGG_PATHWAY	Allograft rejection	24	1.40E-11	1.10E-09
	KEGG_PATHWAY	Graft-versus-host disease	24	1.40E-10	8.00E-09
	KEGG_PATHWAY	Viral myocarditis	33	4.50E-10	1.90E-08
	SP_PIR_KEYWORDS	heterodimer	36	5.00E-10	2.70E-08
	UP_SEQ_FEATURE	domain:Ig-like C1-type	21	1.10E-09	1.90E-06
	KEGG_PATHWAY	Type I diabetes mellitus	23	7.80E-09	2.60E-07
	KEGG_PATHWAY	Cell adhesion molecules (CAMs)	44	8.40E-08	2.00E-06
	KEGG_PATHWAY	Autoimmune thyroid disease	24	1.30E-07	2.80E-06
	KEGG_PATHWAY	Systemic lupus erythematosus	36	1.40E-07	2.70E-06
	GOTERM_BP_FAT	antigen processing and presentation of peptide antigen	16	1.60E-07	2.00E-05
	GOTERM_BP_FAT	antigen processing and presentation	29	2.80E-07	3.20E-05
	UP_SEQ_FEATURE	region of interest:Connecting peptide	17	1.00E-06	7.30E-04
	KEGG_PATHWAY	Intestinal immune network for IgA production	22	1.30E-06	2.00E-05
	INTERPRO	Immunoglobulin C1-set	23	5.20E-06	3.90E-03
	KEGG_PATHWAY	Antigen processing and presentation	29	7.40E-06	9.50E-05
	SP_PIR_KEYWORDS	mhc ii	14	1.10E-05	2.30E-04
	INTERPRO	Immunoglobulin/major histocompatibility complex, conserved site	26	1.30E-05	7.30E-03
	GOTERM_BP_FAT	antigen processing and presentation of peptide or polysaccharide antigen via MHC class II	15	1.40E-05	7.60E-04
	KEGG_PATHWAY	Asthma	15	1.50E-05	1.70E-04
	UP_SEQ_FEATURE	region of interest:Alpha-1	11	1.80E-05	1.00E-02
	UP_SEQ_FEATURE	region of interest:Alpha-2	11	1.80E-05	1.00E-02
	GOTERM_MF_FAT	MHC class II receptor activity	11	1.90E-05	2.40E-03
	SMART	IGc1	23	3.70E-05	1.40E-02
	GOTERM_BP_FAT	antigen processing and presentation of peptide antigen via MHC class I	10	5.90E-05	2.50E-03
	INTERPRO	MHC class II, alpha/beta chain, N-terminal	11	7.90E-05	2.50E-02
	GOTERM_CC_FAT	MHC protein complex	19	9.90E-05	2.60E-03
	PIR_SUPERFAMILY	PIRSF001991:class II histocompatibility antigen	12	1.70E-04	1.50E-01
	SP_PIR_KEYWORDS	mhc i	7	2.60E-04	3.50E-03
	GOTERM_CC_FAT	MHC class II protein complex	12	3.80E-04	8.80E-03
	UP_SEQ_FEATURE	region of interest:Beta-1	7	9.40E-04	2.60E-01
	UP_SEQ_FEATURE	region of interest:Beta-2	7	9.40E-04	2.60E-01
	UP_SEQ_FEATURE	region of interest:Alpha-3	6	1.40E-03	3.20E-01
	PIR_SUPERFAMILY	PIRSF001990:class I histocompatibility antigen	6	2.70E-03	7.20E-01
	INTERPRO	MHC class II, beta chain, N-terminal	7	1.00E-02	5.30E-01
	GOTERM_MF_FAT	MHC class I receptor activity	7	1.10E-02	2.40E-01
	INTERPRO	MHC class II, alpha chain, N-terminal	5	4.30E-02	8.50E-01
	SP_PIR_KEYWORDS	transplantation antigen	3	6.30E-02	3.00E-01
	INTERPRO	MHC class I, alpha chain, C-terminal	3	7.00E-02	9.30E-01
	GOTERM_CC_FAT	MHC class I protein complex	7	1.20E-01	5.50E-01
	INTERPRO	MHC class I, alpha chain, alpha1 and alpha2	6	2.00E-01	9.90E-01
	INTERPRO	MHC class I-like antigen recognition	6	2.20E-01	9.90E-01
	BBID	70.Signal_peptides_(MHC)_class_I_molecules	3	2.40E-01	1.00E+00
**Annotation Cluster 14**	**Enrichment Score: 4.64**		**Count**	**P_Value**	**Benjamini**
	GOTERM_MF_FAT	SH3 domain binding	31	4.10E-07	1.00E-04
	SP_PIR_KEYWORDS	sh3-binding	21	4.70E-06	1.20E-04
	UP_SEQ_FEATURE	short sequence motif:SH3-binding	14	6.20E-03	6.50E-01
**Annotation Cluster 15**	**Enrichment Score: 4.45**		**Count**	**P_Value**	**Benjamini**
	GOTERM_BP_FAT	protein amino acid phosphorylation	132	1.60E-08	2.90E-06
	SP_PIR_KEYWORDS	kinase	123	6.90E-08	2.70E-06

ILMN_3237062	STBD1	-0.405087255
ILMN_1857897	CR608907	-0.405090306
ILMN_1700202	TMEM135	-0.405106665
ILMN_1817251	DB336584	-0.405128709
ILMN_1777439	TCL6	-0.405142126
ILMN_1677868	ADRA2B	-0.405147894
ILMN_1658710	FAM19A4	-0.405156475
ILMN_2208435	CLDN16	-0.405202911
ILMN_1657870	ABL2	-0.405251762
ILMN_1652163	DVL2	-0.405274403
ILMN_1652638	LRRC58	-0.405436479
ILMN_1734775	TISP43	-0.405500331
ILMN_1860645	CD706264	-0.405538145
ILMN_1782938	SLC16A10	-0.4055592
ILMN_1732216	NARS	-0.405595084
ILMN_1750941	DLX4	-0.405618139
ILMN_2108025	KRTAP3-2	-0.405661409
ILMN_1667101	PRM3	-0.405674041
ILMN_1776395	ARMC4	-0.405781379
ILMN_3247653	LRFN1	-0.405859513
ILMN_1739521	NLGN1	-0.405903329
ILMN_1755741	DACH1	-0.405910028
ILMN_2372124	HNF4A	-0.405923157
ILMN_1764959	FAM154A	-0.40602828
ILMN_1742444	X63359	-0.40602929
ILMN_1672057	AQP7	-0.406060866
ILMN_1788538	NCALD	-0.406074349
ILMN_1734568	PC	-0.406120075
ILMN_1711139	VPS37A	-0.406137631
ILMN_1669350	DKFZp434I1020	-0.40621545
ILMN_3239298	OTUD7B	-0.406284973
ILMN_1683067	ELSPBP1	-0.406347674
ILMN_1743638	ABCG1	-0.40636277
ILMN_1688730	DNAJC18	-0.406478246
ILMN_2336585	CTAG2	-0.406504795
ILMN_1700232	FBXO30	-0.406526487
ILMN_1878670	BC068609	-0.406606167
ILMN_1893686	W95809	-0.4066317
ILMN_1721549	PLA2G1B	-0.406685298
ILMN_1705372	THEG	-0.40672733
ILMN_1665970	KIAA0786	-0.406728341
ILMN_1752393	ATOH7	-0.406735465
ILMN_1696568	ATP2C1	-0.406758609
ILMN_1739586	FEZ2	-0.406765731
ILMN_2073012	TMEM203	-0.406866021
ILMN_1773307	NAP1L5	-0.406873309
ILMN_1726756	FANCB	-0.406913222
ILMN_1814007	NA	-0.406938518
ILMN_3245351	UNC119B	-0.406985493
ILMN_1747466	BQ188360	-0.407091095
ILMN_2184966	ZHX2	-0.407112947
ILMN_3251061	LOC338667	-0.407153019
ILMN_1790988	OR5K1	-0.407193115
ILMN_1757220	PLAC1L	-0.407217336
ILMN_2138249	PRSS45	-0.407268872
ILMN_1656915	PSD3	-0.407334396
ILMN_1673111	TSEN34	-0.407338039
ILMN_1745849	ENTPD5	-0.407416568
ILMN_1849186	ZNF704	-0.407435734
ILMN_1806754	GLDC	-0.407533469
ILMN_1877620	DB226000	-0.407570085
ILMN_2322996	EYA2	-0.407581968
ILMN_1769752	FER1L5	-0.407597131
ILMN_1770484	FBXW7	-0.407609577
ILMN_2063584	CLIC4	-0.407612711
ILMN_3246534	TSG1	-0.407620588
ILMN_1695395	XYLT1	-0.407647827
ILMN_1796969	POU4F3	-0.407647976
ILMN_2377829	NANOS1	-0.407713427
ILMN_1794807	CACHD1	-0.407879838
ILMN_2067607	TMEM106B	-0.407918017
ILMN_1664350	GBA3	-0.407999197
ILMN_1749996	KCNN2	-0.408028834

	INTERPRO	Serum albumin	4	5.80E-03	7.70E-01
	INTERPRO	Alpha-fetoprotein	3	2.20E-02	9.50E-01
**Annotation Cluster 13**	**Enrichment Score: 2.44**		**Count**	**P_Value**	**Benjamini**
	SMART	PAN_AP	5	1.70E-03	2.30E-01
	INTERPRO	Apple-like	5	3.10E-03	8.70E-01
	INTERPRO	PAN-1 domain	5	3.10E-03	8.70E-01
	UP_SEQ_FEATURE	domain:PAN	4	1.00E-02	9.70E-01
**Annotation Cluster 14**	**Enrichment Score: 2.41**		**Count**	**P_Value**	**Benjamini**
	GOTERM_BP_FAT	generation of precursor metabolites and energy	59	3.60E-08	6.60E-05
	GOTERM_MF_FAT	monovalent inorganic cation transmembrane transporter activity	25	1.30E-05	3.30E-03
	GOTERM_MF_FAT	inorganic cation transmembrane transporter activity	31	2.60E-05	5.70E-03
	GOTERM_CC_FAT	proton-transporting two-sector ATPase complex	15	3.00E-05	2.40E-03
	GOTERM_BP_FAT	nitrogen compound biosynthetic process	52	3.50E-05	1.40E-02
	GOTERM_MF_FAT	hydrogen ion transmembrane transporter activity	22	3.60E-05	5.90E-03
	GOTERM_BP_FAT	hydrogen transport	17	8.20E-05	2.10E-02
	GOTERM_BP_FAT	ATP synthesis coupled proton transport	13	1.10E-04	2.50E-02
	GOTERM_BP_FAT	energy coupled proton transport, down electrochemical gradient	13	1.10E-04	2.50E-02
	SP_PIR_KEYWORDS	Hydrogen ion transport	14	1.30E-04	9.20E-03
	GOTERM_BP_FAT	proton transport	16	2.00E-04	3.80E-02
	GOTERM_BP_FAT	ATP biosynthetic process	20	2.30E-04	3.90E-02
	GOTERM_BP_FAT	purine nucleoside triphosphate biosynthetic process	21	3.40E-04	4.90E-02
	KEGG_PATHWAY	Oxidative phosphorylation	27	4.30E-04	2.70E-02
	GOTERM_BP_FAT	nucleoside triphosphate biosynthetic process	21	5.10E-04	6.00E-02
	GOTERM_BP_FAT	nucleobase, nucleoside, nucleotide and nucleic acid biosynthetic process	32	7.50E-04	8.20E-02
	GOTERM_BP_FAT	nucleobase, nucleoside and nucleotide biosynthetic process	32	7.50E-04	8.20E-02
	GOTERM_BP_FAT	purine ribonucleoside triphosphate biosynthetic process	20	8.10E-04	8.60E-02
	GOTERM_BP_FAT	oxidative phosphorylation	20	8.10E-04	8.60E-02
	GOTERM_BP_FAT	nucleotide biosynthetic process	31	8.50E-04	8.70E-02
	GOTERM_BP_FAT	ion transmembrane transport	13	8.80E-04	8.80E-02
	GOTERM_BP_FAT	ribonucleoside triphosphate biosynthetic process	20	9.20E-04	8.90E-02
	GOTERM_CC_FAT	proton-transporting V-type ATPase complex	8	1.40E-03	7.00E-02
	GOTERM_BP_FAT	ATP metabolic process	20	1.90E-03	1.40E-01
	GOTERM_BP_FAT	purine nucleotide biosynthetic process	25	2.50E-03	1.70E-01
	GOTERM_BP_FAT	ribonucleotide biosynthetic process	22	2.60E-03	1.70E-01
	GOTERM_BP_FAT	purine ribonucleotide biosynthetic process	21	3.00E-03	1.80E-01
	GOTERM_MF_FAT	proton-transporting ATPase activity, rotational mechanism	8	3.10E-03	2.60E-01
	GOTERM_MF_FAT	cation-transporting ATPase activity	10	3.80E-03	2.70E-01
	GOTERM_CC_FAT	proton-transporting two-sector ATPase complex, catalytic domain	7	4.10E-03	1.40E-01
	GOTERM_BP_FAT	purine nucleoside triphosphate metabolic process	21	4.80E-03	2.30E-01
	GOTERM_BP_FAT	nucleoside triphosphate metabolic process	22	5.10E-03	2.40E-01
	INTERPRO	ATPase, F1/V1/A1 complex, alpha/beta subunit, nucleotide-binding domain	4	5.80E-03	7.70E-01
	INTERPRO	ATPase, F1/V1/A1 complex, alpha/beta subunit, N-terminal	4	5.80E-03	7.70E-01
	INTERPRO	ATPase, F1/V1/A1 complex, alpha/beta subunit, C-terminal	4	5.80E-03	7.70E-01
	GOTERM_BP_FAT	purine ribonucleoside triphosphate metabolic process	20	6.60E-03	2.70E-01
	GOTERM_BP_FAT	ribonucleoside triphosphate metabolic process	20	7.20E-03	2.80E-01
	GOTERM_CC_FAT	vacuolar proton-transporting V-type ATPase complex	5	9.40E-03	2.80E-01
	GOTERM_BP_FAT	purine nucleotide metabolic process	27	1.20E-02	3.90E-01
	GOTERM_MF_FAT	ATPase activity, coupled to transmembrane movement of ions	14	1.40E-02	4.80E-01
	GOTERM_BP_FAT	purine ribonucleotide metabolic process	21	1.80E-02	4.80E-01
	GOTERM_BP_FAT	ribonucleotide metabolic process	22	1.80E-02	4.80E-01
	GOTERM_CC_FAT	mitochondrial proton-transporting ATP synthase complex	6	2.50E-02	4.00E-01
	GOTERM_CC_FAT	proton-transporting ATP synthase complex	6	3.80E-02	4.90E-01
	GOTERM_BP_FAT	ATP hydrolysis coupled proton transport	4	3.80E-02	6.40E-01
	GOTERM_BP_FAT	energy coupled proton transport, against electrochemical gradient	4	3.80E-02	6.40E-01
	GOTERM_MF_FAT	hydrolase activity, acting on acid anhydrides, catalyzing transmembrane movement of substances	17	3.90E-02	7.00E-01
	GOTERM_CC_FAT	proton-transporting V-type ATPase, V1 domain	4	4.20E-02	5.20E-01
	PIR_SUPERFAMILY	PIRSF001274:F-type ATP synthase, alpha/beta subunits/V-type ATPase, subunit B	3	4.80E-02	1.00E+00
	GOTERM_MF_FAT	hydrogen ion transporting ATP synthase activity, rotational mechanism	5	4.90E-02	7.30E-01
	KEGG_PATHWAY	Vibrio cholerae infection	11	5.00E-02	3.60E-01
	GOTERM_CC_FAT	proton-transporting two-sector ATPase complex, proton-transporting domain	6	6.30E-02	6.30E-01
	GOTERM_MF_FAT	ATPase activity, coupled to transmembrane movement of substances	16	6.30E-02	7.50E-01
	GOTERM_MF_FAT	ATPase activity, coupled to movement of substances	16	6.70E-02	7.70E-01
	GOTERM_MF_FAT	primary active transmembrane transporter activity	17	8.00E-02	7.80E-01
	GOTERM_MF_FAT	P-P-bond-hydrolysis-driven transmembrane transporter activity	17	8.00E-02	7.80E-01
	INTERPRO	ATPase, F1/V1/A1 complex, alpha/beta subunit, nucleotide-binding domain, active site	4	8.10E-02	1.00E+00
	KEGG_PATHWAY	Epithelial cell signaling in Helicobacter pylori infection	11	1.40E-01	6.40E-01
	GOTERM_BP_FAT	regulation of pH	6	1.80E-01	9.30E-01
	GOTERM_BP_FAT	monovalent inorganic cation homeostasis	7	2.00E-01	9.40E-01
	GOTERM_MF_FAT	ATPase activity	34	3.10E-01	9.80E-01
	GOTERM_CC_FAT	vacuolar part	8	3.70E-01	9.60E-01
	GOTERM_CC_FAT	vacuolar membrane	7	4.10E-01	9.70E-01
	GOTERM_MF_FAT	ATPase activity, coupled	26	4.80E-01	9.90E-01
**Annotation Cluster 15**	**Enrichment Score: 2.24**		**Count**	**P_Value**	**Benjamini**

ILMN_1718936	LST1	0.672248888
ILMN_1777998	ARHGAP25	0.672026444
ILMN_1782057	ATP8B2	0.67191762
ILMN_2222688	TMSB4X	0.671654519
ILMN_1772218	HLA-DPA1	0.670692724
ILMN_2379644	CD74	0.670235327
ILMN_2109416	NAPSB	0.67015396
ILMN_1756937	ST8SIA4	0.668912627
ILMN_3228688	HLA-DRB1	0.666064054
ILMN_1653498	IGSF6	0.666018108
ILMN_1691290	CELSR3	0.665881098
ILMN_2066066	HLA-DRB6	0.665431097
ILMN_1676528	BTN3A2	0.665034585
ILMN_2113333	LGALS9B	0.664937858
ILMN_1685403	MMP7	0.66473021
ILMN_1709795	RAC2	0.66408783
ILMN_2249920	FYN	0.662601355
ILMN_1710434	TBC1D10C	0.662466513
ILMN_1653026	PLAC8	0.661502048
ILMN_1717334	VAV1	0.661386087
ILMN_1814194	TCF4	0.661347794
ILMN_3249400	abParts	0.660118602
ILMN_1713496	ST3GAL5	0.659938916
ILMN_1723684	DARC	0.659105387
ILMN_1797362	LIMK1	0.659089684
ILMN_2167416	MR1	0.658802524
ILMN_2201678	FSTL1	0.658754384
ILMN_1695640	PTPN22	0.658512971
ILMN_1660368	TRRAP	0.658380229
ILMN_1690139	KIAA0748	0.658030327
ILMN_1716983	LILRA2	0.657877054
ILMN_1718552	CXCL13	0.657876289
ILMN_1768016	TNFRSF17	0.657768889
ILMN_1761788	MOXD1	0.657081246
ILMN_2388155	CASP3	0.656504153
ILMN_1778723	AMICA1	0.656465201
ILMN_1812392	TMSB10	0.656166009
ILMN_2331525	PRKD2	0.655512294
ILMN_3232447	AI880175	0.655153418
ILMN_1707070	PCOLCE	0.654474893
ILMN_1734205	RASSF1	0.654311811
ILMN_1796762	CCDC102A	0.653972062
ILMN_1678143	ARHGDIB	0.653491914
ILMN_2342271	BCL11A	0.653451229
ILMN_1666078	HLA-H	0.652991422
ILMN_1675979	RBMS3	0.652287815
ILMN_1690907	CCR6	0.65223837
ILMN_1733998	DHRS9	0.652071693
ILMN_2395711	ARRB2	0.651877828
ILMN_2347145	DCN	0.65133399
ILMN_1676256	TPSAB1	0.651275576
ILMN_1702787	SEMA4A	0.650728045
ILMN_1763907	CENPW	0.650074396
ILMN_1773352	CCL5	0.649943939
ILMN_2176063	FCGR1C	0.649461219
ILMN_1770085	BTG2	0.649217271
ILMN_1713058	PSTPIP2	0.648615032
ILMN_2369018	EVI2A	0.648472613
ILMN_1814985	PDLIM7	0.646894802
ILMN_1761464	CD74	0.646714479
ILMN_1765258	HLA-E	0.646113068
ILMN_2192072	MMP7	0.646035365
ILMN_2193233	MZB1	0.644949722
ILMN_2079655	KLRB1	0.644921487
ILMN_2348788	CD44	0.644575991
ILMN_2160476	CCL22	0.644222303
ILMN_1700147	VPREB3	0.644060661
ILMN_1759312	AMPD1	0.643717364
ILMN_1665761	BCL11B	0.642540306
ILMN_3219558	TUBA1C	0.642395865
ILMN_2117508	CTHRC1	0.642089036
ILMN_3234841	RPL23AP7	0.640238317
ILMN_1800638	NAPOR-1	0.640073624
ILMN_1719759	TNC	0.638694332
ILMN_1716547	NAGK	0.638512775
ILMN_1687888	BTN3A1	0.638139215
ILMN_1691693	FCRH3	0.63761599
ILMN_2074258	BARD1	0.63760165
ILMN_2087692	CYBRD1	0.636093207
ILMN_1727402	HCLS1	0.635139063
ILMN_2074432	OR2A42	0.635033426
ILMN_2316236	HOPX	0.634952281
ILMN_1653220	PITPNM1	0.633781969
ILMN_1798379	NTM	0.633384125
ILMN_1685387	PIGR	0.632940527
ILMN_1864900	MIAT	0.632288927
ILMN_2059549	SYK	0.632054354
ILMN_1719905	TLR10	0.632037054
ILMN_1785272	COL1A2	0.631449596

	UP_SEQ_FEATURE	domain:Protein kinase	90	2.50E-07	2.50E-04
	INTERPRO	Protein kinase, core	94	3.30E-07	7.60E-04
	UP_SEQ_FEATURE	binding site:ATP	100	3.50E-07	3.00E-04
	INTERPRO	Protein kinase, ATP binding site	90	5.70E-07	6.40E-04
	SP_PIR_KEYWORDS	phosphotransferase	46	2.70E-06	7.80E-05
	GOTERM_MF_FAT	protein kinase activity	111	3.60E-06	6.30E-04
	UP_SEQ_FEATURE	nucleotide phosphate-binding region:ATP	152	5.30E-06	3.30E-03
	SP_PIR_KEYWORDS	serine/threonine-protein kinase	71	1.30E-05	2.60E-04
	SP_PIR_KEYWORDS	atp-binding	196	1.30E-05	2.50E-04
	SP_PIR_KEYWORDS	nucleotide-binding	241	1.30E-05	2.50E-04
	GOTERM_MF_FAT	protein tyrosine kinase activity	40	2.30E-05	2.50E-03
	GOTERM_BP_FAT	phosphorylation	137	3.40E-05	1.60E-03
	INTERPRO	Serine/threonine protein kinase, active site	68	4.10E-05	1.50E-02
	GOTERM_BP_FAT	phosphorus metabolic process	161	4.40E-05	1.90E-03
	GOTERM_BP_FAT	phosphate metabolic process	161	4.40E-05	1.90E-03
	UP_SEQ_FEATURE	active site:Proton acceptor	107	4.70E-05	2.20E-02
	GOTERM_MF_FAT	protein serine/threonine kinase activity	79	9.70E-05	7.90E-03
	INTERPRO	Serine/threonine protein kinase-related	67	1.10E-04	3.20E-02
	GOTERM_MF_FAT	purine ribonucleotide binding	266	3.40E-04	2.20E-02
	GOTERM_MF_FAT	ribonucleotide binding	266	3.40E-04	2.20E-02
	GOTERM_MF_FAT	ATP binding	219	3.50E-04	2.10E-02
	GOTERM_MF_FAT	nucleotide binding	318	3.90E-04	2.20E-02
	SP_PIR_KEYWORDS	transferase	193	6.00E-04	7.20E-03
	GOTERM_MF_FAT	adenyl ribonucleotide binding	219	7.40E-04	3.50E-02
	INTERPRO	Serine/threonine protein kinase	49	7.80E-04	1.00E-01
	GOTERM_MF_FAT	purine nucleotide binding	272	1.00E-03	4.80E-02
	GOTERM_MF_FAT	purine nucleoside binding	229	1.80E-03	7.20E-02
	GOTERM_MF_FAT	nucleoside binding	230	2.10E-03	7.50E-02
	GOTERM_MF_FAT	adenyl nucleotide binding	225	2.30E-03	7.50E-02
	SMART	S_TKc	49	9.00E-03	4.00E-01
**Annotation Cluster 16**	**Enrichment Score: 4.45**		**Count**	**P_Value**	**Benjamini**
	GOTERM_BP_FAT	transcription from RNA polymerase II promoter	52	2.60E-05	1.30E-03
	GOTERM_BP_FAT	transcription, DNA-dependent	61	3.40E-05	1.60E-03
	GOTERM_BP_FAT	RNA biosynthetic process	61	5.20E-05	2.30E-03
**Annotation Cluster 17**	**Enrichment Score: 4.29**		**Count**	**P_Value**	**Benjamini**
	GOTERM_BP_FAT	antigen processing and presentation of peptide antigen	16	1.60E-07	2.00E-05
	GOTERM_BP_FAT	antigen processing and presentation of peptide or polysaccharide antigen via MHC class II	15	1.40E-05	7.60E-04
	GOTERM_BP_FAT	antigen processing and presentation of exogenous antigen	9	7.60E-05	3.00E-03
	GOTERM_BP_FAT	antigen processing and presentation of exogenous peptide antigen	8	8.70E-05	3.30E-03
	GOTERM_BP_FAT	antigen processing and presentation of peptide antigen via MHC class II	6	1.10E-03	2.60E-02
	GOTERM_BP_FAT	antigen processing and presentation of exogenous peptide antigen via MHC class II	6	1.10E-03	2.60E-02
**Annotation Cluster 18**	**Enrichment Score: 4.27**		**Count**	**P_Value**	**Benjamini**
	SP_PIR_KEYWORDS	extracellular matrix	57	4.60E-08	1.90E-06
	GOTERM_CC_FAT	extracellular matrix	68	7.70E-05	2.20E-03
	GOTERM_CC_FAT	proteinaceous extracellular matrix	64	8.40E-05	2.30E-03
	GOTERM_CC_FAT	extracellular region part	153	4.10E-04	9.10E-03
	GOTERM_CC_FAT	extracellular matrix part	26	3.70E-03	6.10E-02
**Annotation Cluster 19**	**Enrichment Score: 4.27**		**Count**	**P_Value**	**Benjamini**
	GOTERM_BP_FAT	leukocyte mediated immunity	29	6.40E-07	7.00E-05
	GOTERM_BP_FAT	immune effector process	37	3.30E-06	2.60E-04
	GOTERM_BP_FAT	lymphocyte mediated immunity	23	1.90E-05	1.00E-03
	GOTERM_BP_FAT	B cell mediated immunity	18	2.60E-04	7.50E-03
	GOTERM_BP_FAT	adaptive immune response	22	2.80E-04	7.90E-03
	GOTERM_BP_FAT	adaptive immune response based on somatic recombination of immune receptors built from immunoglobulin superfamily domains	22	2.80E-04	7.90E-03
	GOTERM_BP_FAT	immunoglobulin mediated immune response	16	1.60E-03	3.60E-02
**Annotation Cluster 20**	**Enrichment Score: 4.22**		**Count**	**P_Value**	**Benjamini**
	GOTERM_BP_FAT	regulation of protein kinase cascade	64	1.00E-08	2.00E-06
	GOTERM_BP_FAT	regulation of cellular response to stress	34	1.00E-07	1.40E-05
	GOTERM_BP_FAT	regulation of stress-activated protein kinase signaling pathway	25	1.00E-06	1.00E-04
	GOTERM_BP_FAT	regulation of JNK cascade	23	4.90E-06	3.40E-04
	GOTERM_BP_FAT	regulation of phosphorus metabolic process	93	1.10E-05	6.40E-04
	GOTERM_BP_FAT	regulation of phosphate metabolic process	93	1.10E-05	6.40E-04
	GOTERM_BP_FAT	regulation of phosphorylation	90	1.10E-05	6.60E-04
	GOTERM_BP_FAT	regulation of MAPKKK cascade	31	1.30E-05	7.10E-04
	GOTERM_BP_FAT	positive regulation of protein kinase activity	50	3.00E-05	1.40E-03
	GOTERM_BP_FAT	positive regulation of kinase activity	51	3.70E-05	1.70E-03
	GOTERM_BP_FAT	positive regulation of molecular function	105	5.20E-05	2.30E-03
	GOTERM_BP_FAT	positive regulation of transferase activity	52	5.20E-05	2.20E-03
	GOTERM_BP_FAT	regulation of MAP kinase activity	35	7.00E-05	2.90E-03
	GOTERM_BP_FAT	stress-activated protein kinase signaling pathway	20	1.30E-04	4.30E-03
	GOTERM_BP_FAT	regulation of protein kinase activity	67	1.40E-04	4.70E-03
	GOTERM_BP_FAT	JNK cascade	19	1.60E-04	5.30E-03
	GOTERM_BP_FAT	positive regulation of MAP kinase activity	27	1.90E-04	5.90E-03
	GOTERM_BP_FAT	MAPKKK cascade	41	2.00E-04	6.10E-03
	GOTERM_BP_FAT	regulation of kinase activity	68	2.30E-04	6.90E-03
	GOTERM_BP_FAT	positive regulation of catalytic activity	92	2.60E-04	7.50E-03
	GOTERM_BP_FAT	activation of MAPK activity	23	2.60E-04	7.50E-03
	GOTERM_BP_FAT	regulation of transferase activity	69	4.40E-04	1.20E-02
	GOTERM_BP_FAT	positive regulation of JUN kinase activity	12	1.00E-03	2.40E-02
	GOTERM_BP_FAT	activation of JUN kinase activity	10	2.70E-03	5.20E-02
	GOTERM_BP_FAT	regulation of JUN kinase activity	12	3.70E-03	6.60E-02
	GOTERM_BP_FAT	activation of protein kinase activity	19	1.60E-01	7.50E-01
**Annotation Cluster 21**	**Enrichment Score: 3.88**		**Count**	**P_Value**	**Benjamini**
	GOTERM_MF_FAT	MHC protein binding	13	9.60E-06	1.50E-03
	GOTERM_MF_FAT	MHC class I protein binding	10	2.60E-05	2.60E-03
	GOTERM_CC_FAT	MHC class I peptide loading complex	7	2.00E-04	4.90E-03
	GOTERM_CC_FAT	TAP complex	5	5.80E-03	8.40E-02
**Annotation Cluster 22**	**Enrichment Score: 3.83**		**Count**	**P_Value**	**Benjamini**
	SP_PIR_KEYWORDS	hydroxylation	23	4.60E-06	1.20E-04

ILMN_1774955	IMAA	-0.408066886
ILMN_1809931	NDRG1	-0.408085479
ILMN_1666140	PRY2	-0.408171803
ILMN_1828083	DB338706	-0.408246315
ILMN_3308748	MIR346	-0.408259317
ILMN_1688504	OR1L8	-0.40834295
ILMN_1714643	MGAM	-0.40835672
ILMN_1868037	CA441597	-0.408414703
ILMN_1658909	OSGEPL1	-0.40842325
ILMN_1712161	BCOR	-0.408430008
ILMN_2219681	RBP2	-0.408475596
ILMN_1716822	KRTAP9-2	-0.408499237
ILMN_1743187	C6orf120	-0.408592716
ILMN_1696162	MME	-0.408664717
ILMN_2320850	UBE2D3	-0.40875338
ILMN_1782439	CNN3	-0.408756645
ILMN_1750624	RXRG	-0.408768828
ILMN_2200210	GJA10	-0.408775674
ILMN_1651848	ITSN1	-0.408787859
ILMN_2381730	EGFL7	-0.408792059
ILMN_1739886	HNF4A	-0.40884571
ILMN_1846567	BX107471	-0.408896432
ILMN_1654013	C17orf28	-0.4088974
ILMN_1764120	IGF2AS	-0.408918369
ILMN_1813775	GAK	-0.408950301
ILMN_1836827	AI809874	-0.408958924
ILMN_1861915	CA392115	-0.408962177
ILMN_3286751	LOC284648	-0.408996705
ILMN_1802649	C11orf58	-0.409033992
ILMN_2062754	SRA1	-0.409059362
ILMN_1655537	ING1	-0.409077239
ILMN_1699216	FLJ45721	-0.409104592
ILMN_2195665	PANK3	-0.409113445
ILMN_3250032	XPR1	-0.409191586
ILMN_3310775	MIR1323	-0.409214172
ILMN_1714884	GHRHR	-0.409228733
ILMN_1784176	ABCA5	-0.409318636
ILMN_1786125	CCNA2	-0.40941327
ILMN_2293067	PM20D1	-0.409425626
ILMN_1779370	ARHGEF9	-0.409453126
ILMN_1755869	OR51T1	-0.409467678
ILMN_1904931	KCNQ5	-0.40950761
ILMN_1743635	ALG14	-0.409575205
ILMN_3245464	NCRMS	-0.409667941
ILMN_1764826	TFE3	-0.409700564
ILMN_1703826	UNC5D	-0.409720559
ILMN_3310725	MIR323B	-0.409746154
ILMN_1908989	CB243773	-0.409754221
ILMN_2321301	GRIN1	-0.409763089
ILMN_1726752	APTX	-0.409776527
ILMN_1758196	LOC619207	-0.409777666
ILMN_3286312	NEK2	-0.409809723
ILMN_1760088	CCKBR	-0.40994123
ILMN_1691106	CDV3	-0.410200762
ILMN_1740920	ACADSB	-0.410384879
ILMN_1653006	CSN1S1	-0.410399232
ILMN_1803676	ENOSF1	-0.410436355
ILMN_1793578	ZFP37	-0.410478588
ILMN_3200717	LOC643387	-0.41049239
ILMN_1687291	CDNF	-0.410499483
ILMN_1675873	SLC35F3	-0.41072001
ILMN_2396702	GRIK2	-0.410763892
ILMN_2404539	C20orf30	-0.410861945
ILMN_1821517	X64709	-0.410868466
ILMN_1723874	MRPS6	-0.411063935
ILMN_2111187	ELOVL6	-0.411121341
ILMN_1876371	AI650261	-0.411136408
ILMN_1811555	C9orf119	-0.411140367
ILMN_3306691	LOC729467	-0.411221641
ILMN_1907700	AA551223	-0.41125527
ILMN_1740185	TPMT	-0.411263483
ILMN_1656869	LGALS9C	-0.411346951
ILMN_1754708	TNK2	-0.411390222
ILMN_1682399	CLOCK	-0.411557524
ILMN_2122669	TOPORS	-0.411704836
ILMN_2156953	ZFAND6	-0.411824148
ILMN_2162819	UGT2B11	-0.411885624
ILMN_1810488	NFYC	-0.41190177
ILMN_1800898	ARG2	-0.412011618
ILMN_1717094	ZNF618	-0.41201267
ILMN_3236192	NA	-0.412168305
ILMN_1718718	MKKS	-0.412325615
ILMN_1746457	GTF3C2	-0.412343401
ILMN_3249598	RGS21	-0.412358441
ILMN_3239408	REXO1L1	-0.412402962
ILMN_3298163	CR594811	-0.412502208
ILMN_2234605	AGRN	-0.412512302
ILMN_1698745	CLRN1	-0.412580136
ILMN_2290089	UBN1	-0.4126015

	GOTERM_BP_FAT	protein tetramerization	12	3.90E-04	5.20E-02
	GOTERM_BP_FAT	protein homotetramerization	9	1.30E-03	1.10E-01
	GOTERM_BP_FAT	protein homooligomerization	15	3.80E-02	6.40E-01
	GOTERM_BP_FAT	protein oligomerization	23	5.40E-02	7.10E-01
**Annotation Cluster 16**	**Enrichment Score: 2.1**		**Count**	**P_Value**	**Benjamini**
	GOTERM_BP_FAT	alcohol biosynthetic process	11	4.20E-03	2.30E-01
	GOTERM_BP_FAT	hexose biosynthetic process	9	4.50E-03	2.30E-01
	GOTERM_BP_FAT	gluconeogenesis	8	4.70E-03	2.30E-01
	GOTERM_BP_FAT	cellular carbohydrate biosynthetic process	14	6.60E-03	2.70E-01
	GOTERM_BP_FAT	pyruvate metabolic process	10	9.70E-03	3.40E-01
	GOTERM_BP_FAT	monosaccharide biosynthetic process	9	1.40E-02	4.10E-01
	GOTERM_BP_FAT	carbohydrate biosynthetic process	17	2.50E-02	5.40E-01
**Annotation Cluster 17**	**Enrichment Score: 2.09**		**Count**	**P_Value**	**Benjamini**
	UP_SEQ_FEATURE	binding site:Substrate; via amide nitrogen	9	4.30E-04	4.40E-01
	SP_PIR_KEYWORDS	Flavoprotein	21	6.40E-04	3.60E-02
	GOTERM_MF_FAT	FAD binding	15	4.40E-03	2.90E-01
	UP_SEQ_FEATURE	binding site:Substrate; via carbonyl oxygen	8	5.10E-03	9.20E-01
	INTERPRO	Acyl-CoA dehydrogenase/oxidase, N-terminal	6	5.20E-03	7.80E-01
	INTERPRO	Acyl-CoA oxidase/dehydrogenase, type1/2, C-terminal	6	7.30E-03	8.00E-01
	INTERPRO	Acyl-CoA oxidase/dehydrogenase, central region	6	7.30E-03	8.00E-01
	SP_PIR_KEYWORDS	FAD	19	7.60E-03	2.20E-01
	GOTERM_MF_FAT	acyl-CoA dehydrogenase activity	6	8.10E-03	3.80E-01
	INTERPRO	Acyl-CoA dehydrogenase, N-terminal	5	1.20E-02	8.80E-01
	UP_SEQ_FEATURE	nucleotide phosphate-binding region:FAD	12	1.30E-02	9.80E-01
	PIR_SUPERFAMILY	PIRSF000182:acyl-CoA dehydrogenase	4	1.40E-02	9.60E-01
	INTERPRO	Acyl-CoA oxidase/dehydrogenase, type 1	5	2.20E-02	9.30E-01
	UP_SEQ_FEATURE	nucleotide phosphate-binding region:FAD; shared with dimeric partner	3	3.90E-02	1.00E+00
	UP_SEQ_FEATURE	binding site:FAD; shared with dimeric partner	3	3.90E-02	1.00E+00
	INTERPRO	Acyl-CoA dehydrogenase, conserved site	4	5.00E-02	9.90E-01
**Annotation Cluster 18**	**Enrichment Score: 2.05**		**Count**	**P_Value**	**Benjamini**
	GOTERM_BP_FAT	alditol metabolic process	9	9.80E-04	9.00E-02
	GOTERM_BP_FAT	glycerol metabolic process	8	2.80E-03	1.80E-01
	GOTERM_BP_FAT	polyol metabolic process	9	2.10E-02	5.20E-01
	GOTERM_BP_FAT	alditol catabolic process	3	2.20E-02	5.20E-01
	GOTERM_BP_FAT	polyol catabolic process	3	4.10E-02	6.40E-01

ILMN_2062714	PTGDR	0.630949881
ILMN_1773059	GPR124	0.630604415
ILMN_1663080	LFNG	0.630140795
ILMN_1803036	TARBP1	0.629876238
ILMN_1653828	CHFR	0.62976732
ILMN_2101885	TUBB	0.629549873
ILMN_1764788	TNFRSF1B	0.62953518
ILMN_1712419	DCDC2	0.628327105
ILMN_1803988	MCL1	0.628242295
ILMN_1662843	CD53	0.62805919
ILMN_2226955	VOPP1	0.627357053
ILMN_2220283	HNRNPA1	0.627342749
ILMN_1681679	TSPO	0.627134432
ILMN_1719986	PIK3IP1	0.626360579
ILMN_2373062	RHBDF2	0.626286979
ILMN_1687301	VCAN	0.625654632
ILMN_1796316	MMP9	0.625351613
ILMN_1672417	PTPRCAP	0.625195448
ILMN_2384544	ADAM15	0.62435307
ILMN_1807042	MARCKS	0.624161335
ILMN_1764362	LYAR	0.624044588
ILMN_1652825	IL10RA	0.62357022
ILMN_1673708	HDAC9	0.623570075
ILMN_1815895	HLA-DRB1	0.623526187
ILMN_2098947	AK095700	0.62323007
ILMN_1674228	abParts	0.622984005
ILMN_1784661	TMEM2	0.622003556
ILMN_1767556	C10orf10	0.621536433
ILMN_1792323	HDC	0.621524668
ILMN_1780368	GPR18	0.621486037
ILMN_1805027	KLC3	0.621319246
ILMN_1705442	CMTM3	0.621090964
ILMN_1774733	SOCS1	0.621085046
ILMN_2309180	SMARCD3	0.621082937
ILMN_1683980	PLEKHM2	0.620958514
ILMN_3229033	TDG	0.620946069
ILMN_2330307	SLC43A3	0.620584718
ILMN_3200140	LOC645638	0.620553634
ILMN_1706268	PTGDR	0.619671617
ILMN_1750321	TRANK1	0.619300429
ILMN_1715760	HUAT	0.619230785
ILMN_1679880	THOC6	0.618651764
ILMN_1778401	HLA-B	0.618578585
ILMN_1696004	LRRK1	0.618463232
ILMN_2328776	MST4	0.618160073
ILMN_1722872	MYH9	0.617879296
ILMN_1669447	PYHIN1	0.617666299
ILMN_1676361	ARHGAP22	0.61742161
ILMN_2413779	SEZ6L2	0.615604254
ILMN_1688780	S100A4	0.615565657
ILMN_1693552	CD300A	0.61547995
ILMN_1727761	GMEB1	0.615245742
ILMN_2122374	FAM49B	0.61497118
ILMN_1733937	MMD	0.614608612
ILMN_2400030	PTPN2	0.614584008
ILMN_1702229	CECR6	0.614340915
ILMN_2391458	ACSL4	0.613769072
ILMN_1708167	CDCP1	0.613151894
ILMN_1792951	ZHX2	0.613149012
ILMN_1769705	H3F3C	0.61313978
ILMN_1742450	TAPBP	0.613127452
ILMN_1722622	CD163	0.613040778
ILMN_2360710	TPM1	0.612664875
ILMN_1701643	GDPD5	0.612269833
ILMN_1736538	C9orf68	0.61165406
ILMN_1683026	PSMB10	0.611652785
ILMN_1711383	STK4	0.611352388
ILMN_1765446	EMP3	0.611310839
ILMN_1684040	THEMIS	0.610118066
ILMN_1740298	SIGLEC5	0.609968392
ILMN_1730773	RPL10	0.609924309
ILMN_1699440	ZBTB47	0.609841785
ILMN_2368318	FGR	0.609671673
ILMN_2231051	TCP11L2	0.609604496
ILMN_1662932	LCP1	0.609601461
ILMN_2088990	CXorf21	0.609529471
ILMN_3194911	ATF6B	0.609526465
ILMN_1690085	STK11IP	0.608686385
ILMN_2143685	CLDN7	0.608599961
ILMN_1686401	PDCD2	0.608402761
ILMN_1774077	GBP2	0.608164155
ILMN_1659888	PPP1R14B	0.608089957
ILMN_2408851	ARHGAP30	0.607830215
ILMN_2352303	RASSF2	0.607805009
ILMN_1778977	TYROBP	0.607657684
ILMN_2151277	LPGAT1	0.607159992
ILMN_3235514	GPR183	0.606852749
ILMN_2227195	CCDC65	0.606729942
ILMN_3289346	AX747493	0.606345057
ILMN_1736567	CD74	0.605612199
ILMN_1737146	TRAM1	0.605360914
ILMN_1737935	MACF1	0.605311406
ILMN_1790562	EYA3	0.605093823
ILMN_1713829	PTGES	0.604939851
ILMN_2046730	S100A10	0.604766717
ILMN_1704380	INCA1	0.604094335

	SP_PIR_KEYWORDS	triple helix	14	1.10E-05	2.30E-04
	SP_PIR_KEYWORDS	hydroxylysine	14	1.10E-05	2.30E-04
	SP_PIR_KEYWORDS	hydroxyproline	15	2.00E-05	3.70E-04
	UP_SEQ_FEATURE	glycosylation site:O-linked (Gal...)	7	1.20E-04	4.10E-02
	GOTERM_CC_FAT	collagen	14	1.50E-04	3.60E-03
	SP_PIR_KEYWORDS	collagen	24	2.10E-04	3.00E-03
	SP_PIR_KEYWORDS	trimer	11	2.60E-04	3.60E-03
	INTERPRO	Collagen triple helix repeat	22	5.20E-04	8.70E-02
	UP_SEQ_FEATURE	region of interest:Triple-helical region	9	2.40E-03	4.40E-01
	GOTERM_MF_FAT	extracellular matrix structural constituent	21	2.40E-03	7.70E-02
	GOTERM_CC_FAT	extracellular matrix part	26	3.70E-03	6.10E-02
**Annotation Cluster 23**	**Enrichment Score: 3.83**		**Count**	**P_Value**	**Benjamini**
	GOTERM_BP_FAT	lymphocyte differentiation	35	2.90E-08	4.30E-06
	GOTERM_BP_FAT	B cell activation	19	4.10E-03	7.20E-02
	GOTERM_BP_FAT	B cell differentiation	12	2.80E-02	2.90E-01
**Annotation Cluster 24**	**Enrichment Score: 3.77**		**Count**	**P_Value**	**Benjamini**
	GOTERM_MF_FAT	transcription factor binding	103	1.00E-07	3.20E-05
	GOTERM_MF_FAT	transcription cofactor activity	66	5.20E-04	2.80E-02
	GOTERM_MF_FAT	transcription activator activity	70	2.00E-03	7.40E-02
	GOTERM_MF_FAT	transcription coactivator activity	39	7.90E-03	1.90E-01
**Annotation Cluster 25**	**Enrichment Score: 3.59**		**Count**	**P_Value**	**Benjamini**
	GOTERM_BP_FAT	positive regulation of immune system process	65	5.50E-10	2.60E-07
	GOTERM_BP_FAT	regulation of leukocyte activation	45	4.20E-07	4.70E-05
	GOTERM_BP_FAT	regulation of lymphocyte activation	41	8.00E-07	8.30E-05
	GOTERM_BP_FAT	regulation of cell activation	45	2.00E-06	1.80E-04
	GOTERM_BP_FAT	positive regulation of leukocyte activation	32	2.30E-06	2.00E-04
	GOTERM_BP_FAT	regulation of T cell activation	34	2.60E-06	2.20E-04
	GOTERM_BP_FAT	positive regulation of cell activation	32	6.50E-06	4.10E-04
	GOTERM_BP_FAT	positive regulation of lymphocyte activation	29	9.10E-06	5.60E-04
	GOTERM_BP_FAT	positive regulation of T cell activation	24	2.40E-05	1.20E-03
	GOTERM_BP_FAT	regulation of lymphocyte differentiation	21	3.80E-05	1.70E-03
	GOTERM_BP_FAT	regulation of leukocyte proliferation	25	4.70E-05	2.10E-03
	GOTERM_BP_FAT	regulation of mononuclear cell proliferation	25	4.70E-05	2.10E-03
	GOTERM_BP_FAT	regulation of lymphocyte proliferation	24	1.10E-04	3.90E-03
	GOTERM_BP_FAT	positive regulation of lymphocyte differentiation	14	1.50E-04	4.80E-03
	GOTERM_BP_FAT	regulation of alpha-beta T cell activation	14	2.00E-04	6.30E-03
	GOTERM_BP_FAT	regulation of B cell activation	17	2.60E-04	7.60E-03
	GOTERM_BP_FAT	regulation of T cell differentiation	17	2.60E-04	7.60E-03
	GOTERM_BP_FAT	positive regulation of leukocyte proliferation	18	2.60E-04	7.50E-03
	GOTERM_BP_FAT	positive regulation of mononuclear cell proliferation	18	2.60E-04	7.50E-03
	GOTERM_BP_FAT	regulation of B cell proliferation	12	2.70E-04	7.60E-03
	GOTERM_BP_FAT	positive regulation of lymphocyte proliferation	17	6.70E-04	1.70E-02
	GOTERM_BP_FAT	positive regulation of T cell differentiation	12	1.00E-03	2.40E-02
	GOTERM_BP_FAT	positive regulation of B cell activation	12	1.30E-03	3.10E-02
	GOTERM_BP_FAT	positive regulation of alpha-beta T cell activation	10	2.70E-03	5.20E-02
	GOTERM_BP_FAT	regulation of T cell proliferation	16	6.90E-03	1.10E-01
	GOTERM_BP_FAT	positive regulation of B cell proliferation	8	7.50E-03	1.10E-01
	GOTERM_BP_FAT	positive regulation of T cell proliferation	11	1.70E-02	2.10E-01
	GOTERM_BP_FAT	regulation of alpha-beta T cell differentiation	8	2.70E-02	2.90E-01
	GOTERM_BP_FAT	regulation of CD4-positive, alpha beta T cell differentiation	6	2.90E-02	3.00E-01
	GOTERM_BP_FAT	regulation of B cell differentiation	6	3.80E-02	3.50E-01
	GOTERM_BP_FAT	positive regulation of B cell differentiation	4	4.40E-02	3.90E-01
	GOTERM_BP_FAT	positive regulation of alpha-beta T cell differentiation	6	8.90E-02	5.80E-01
	GOTERM_BP_FAT	positive regulation of CD4-positive, alpha beta T cell differentiation	4	1.40E-01	7.30E-01
	GOTERM_BP_FAT	regulation of interleukin-4 production	3	3.00E-01	9.20E-01
**Annotation Cluster 26**	**Enrichment Score: 3.52**		**Count**	**P_Value**	**Benjamini**
	SP_PIR_KEYWORDS	actin-binding	53	3.30E-06	9.20E-05
	GOTERM_MF_FAT	actin binding	63	1.30E-04	9.20E-03
	GOTERM_CC_FAT	actin cytoskeleton	50	2.80E-03	4.90E-02
	GOTERM_MF_FAT	cytoskeletal protein binding	80	6.70E-03	1.70E-01
**Annotation Cluster 27**	**Enrichment Score: 3.43**		**Count**	**P_Value**	**Benjamini**
	UP_SEQ_FEATURE	domain:PH	48	5.30E-05	2.20E-02
	INTERPRO	Pleckstrin homology	54	1.90E-04	3.90E-02
	INTERPRO	Pleckstrin homology-type	56	5.70E-04	8.30E-02
	SMART	PH	54	3.20E-03	2.20E-01
**Annotation Cluster 28**	**Enrichment Score: 3.42**		**Count**	**P_Value**	**Benjamini**
	GOTERM_BP_FAT	regulation of cell adhesion	41	8.50E-08	1.20E-05
	GOTERM_BP_FAT	positive regulation of cell adhesion	17	1.90E-03	3.90E-02
	GOTERM_BP_FAT	regulation of cell-substrate adhesion	14	2.80E-03	5.30E-02
	GOTERM_BP_FAT	positive regulation of cell-substrate adhesion	8	4.80E-02	4.10E-01
**Annotation Cluster 29**	**Enrichment Score: 3.37**		**Count**	**P_Value**	**Benjamini**
	SP_PIR_KEYWORDS	sh3 domain	44	4.60E-05	7.50E-04
	UP_SEQ_FEATURE	domain:SH3	38	1.00E-04	4.00E-02
	INTERPRO	Src homology-3 domain	42	8.50E-04	1.10E-01
	SMART	SH3	42	8.00E-03	4.10E-01
**Annotation Cluster 30**	**Enrichment Score: 3.28**		**Count**	**P_Value**	**Benjamini**
	GOTERM_BP_FAT	vasculature development	52	1.70E-04	5.60E-03
	GOTERM_BP_FAT	blood vessel development	50	3.40E-04	9.30E-03
	GOTERM_BP_FAT	angiogenesis	34	4.20E-04	1.10E-02
	GOTERM_BP_FAT	blood vessel morphogenesis	41	3.20E-03	5.80E-02
**Annotation Cluster 31**	**Enrichment Score: 3.18**		**Count**	**P_Value**	**Benjamini**
	GOTERM_BP_FAT	myeloid cell differentiation	26	9.90E-05	3.50E-03
	GOTERM_BP_FAT	homeostasis of number of cells	25	8.30E-04	2.10E-02
	GOTERM_BP_FAT	erythrocyte differentiation	14	1.40E-03	3.20E-02
	GOTERM_BP_FAT	erythrocyte homeostasis	15	1.70E-03	3.70E-02
**Annotation Cluster 32**	**Enrichment Score: 3.09**		**Count**	**P_Value**	**Benjamini**
	GOTERM_BP_FAT	cell motion	94	2.50E-06	2.10E-04
	GOTERM_BP_FAT	cell migration	50	4.70E-03	7.90E-02
	GOTERM_BP_FAT	cell motility	54	6.00E-03	9.70E-02
	GOTERM_BP_FAT	localization of cell	54	6.00E-03	9.70E-02
**Annotation Cluster 33**	**Enrichment Score: 3.08**		**Count**	**P_Value**	**Benjamini**
	GOTERM_MF_FAT	identical protein binding	111	4.30E-05	4.00E-03
	GOTERM_MF_FAT	protein dimerization activity	90	1.10E-03	4.60E-02
	GOTERM_MF_FAT	protein homodimerization activity	55	1.30E-02	2.60E-01
**Annotation Cluster 34**	**Enrichment Score: 3.07**		**Count**	**P_Value**	**Benjamini**
	GOTERM_BP_FAT	response to molecule of bacterial origin	25	7.10E-05	2.90E-03

ILMN_1654430	AK129671	-0.412605565
ILMN_3184433	LOC100130285	-0.412650539
ILMN_1793832	GRB14	-0.412696831
ILMN_1681882	CETP	-0.412715762
ILMN_1816458	DB298233	-0.412747859
ILMN_1775138	PEG3	-0.412824696
ILMN_1800923	OR4M1	-0.412857678
ILMN_1740728	SMTNL2	-0.412909528
ILMN_1843648	AW873324	-0.413007328
ILMN_1752281	DNAJC13	-0.41302852
ILMN_1810233	UGT2B11	-0.413038655
ILMN_3309374	MIR1301	-0.413065195
ILMN_3237645	LRTOMT	-0.41313146
ILMN_1796337	CXCL17	-0.413215692
ILMN_2151281	GABARAPL1	-0.413219986
ILMN_1666510	SNURF-SNRPN	-0.413239596
ILMN_2231177	OR7E24	-0.41332793
ILMN_1756779	CLTC	-0.413379014
ILMN_3262909	C6orf176	-0.413382783
ILMN_1916599	LOC100133985	-0.413388093
ILMN_2284157	CLDN18	-0.413403205
ILMN_1903082	AI769787	-0.413431488
ILMN_1808769	C1orf97	-0.413446668
ILMN_1730575	GCLC	-0.413471068
ILMN_3225978	NA	-0.413478373
ILMN_1880983	BC071719	-0.413514628
ILMN_1676383	ENPP6	-0.413587126
ILMN_1795673	C9orf173	-0.413685852
ILMN_1790810	HCN4	-0.413705885
ILMN_1802628	PPAPDC2	-0.413715285
ILMN_2413264	SOCS4	-0.413735775
ILMN_1673234	PKDREJ	-0.413858526
ILMN_1753745	HDDC2	-0.413894248
ILMN_1769416	TTN	-0.413924612
ILMN_2406304	PDZRN3	-0.413983308
ILMN_2393968	SYNJ1	-0.413995013
ILMN_2046315	GABARAPL2	-0.414003193
ILMN_1695601	AK098200	-0.414080787
ILMN_1847612	AA554377	-0.414087167
ILMN_1659524	NDUFAF4	-0.414344572
ILMN_3270493	FAM75A3	-0.41445667
ILMN_1690796	OR4F21	-0.414544236
ILMN_3310030	MIR1265	-0.41468888
ILMN_1813741	KCNJ16	-0.414760556
ILMN_1719857	GRIPAP1	-0.41477515
ILMN_1725556	TMEM229A	-0.414776159
ILMN_1674678	RBMY1A1	-0.41486818
ILMN_1669367	LOC100130827	-0.414944988
ILMN_1723846	METTL21B	-0.414963649
ILMN_1664001	ARSF	-0.41517798
ILMN_3237226	MBD3L2	-0.415237183
ILMN_1751615	COQ10B	-0.415264295
ILMN_3238159	MAOA	-0.415279276
ILMN_2360988	SEMA6B	-0.415324484
ILMN_1661417	MAGED2	-0.415345173
ILMN_1880885	AL042883	-0.415354593
ILMN_1730401	LOC442421	-0.415372318
ILMN_2178994	TUBB8	-0.4153855
ILMN_1677805	DNAH14	-0.415420478
ILMN_1776412	KRTAP10-11	-0.415431221
ILMN_1764175	GPD1	-0.415493143
ILMN_1783835	ZNF845	-0.415497474
ILMN_3307777	RETNLB	-0.415508894
ILMN_2153495	WNT7B	-0.415526638
ILMN_1747854	GOLGA8E	-0.415553681
ILMN_1783705	GRXCR2	-0.415578478
ILMN_2142353	GRTP1	-0.415616858
ILMN_1691466	YES1	-0.415623012
ILMN_1747696	TPM3	-0.415637933
ILMN_1809844	MAP2K6	-0.415701214
ILMN_2056975	HPRT1	-0.415968793
ILMN_3250909	AL137495	-0.416082649
ILMN_1732468	HSPA4L	-0.416155751
ILMN_2110094	CFC1	-0.416202827
ILMN_1725098	RTN4RL2	-0.416237184
ILMN_1755120	MAN1A2	-0.416249794
ILMN_3299929	CR625008	-0.416269994
ILMN_1652333	FN3KRP	-0.416397568
ILMN_1747207	EYA1	-0.416439267
ILMN_1662438	SOD1	-0.416442406
ILMN_1682495	FOXP1	-0.416638683
ILMN_1798535	SMR3A	-0.416678713
ILMN_1739582	HOXA10	-0.41668763
ILMN_1729868	SPRYD4	-0.416704212
ILMN_1862900	BQ447249	-0.416720569
ILMN_1672742	ARHGAP23	-0.416730482
ILMN_1732127	RBKS	-0.416752208
ILMN_2374191	FNTA	-0.416753198
ILMN_1705568	CR596696	-0.416759786
ILMN_3251171	DNASE1L1	-0.416969036
ILMN_2122926	VN1R2	-0.416990785
ILMN_1769091	PRCP	-0.417067754
ILMN_1753287	DLL3	-0.417101695
ILMN_1662914	CTAGE5	-0.417154243
ILMN_1672545	GIPC2	-0.417169392
ILMN_1663620	GLYCAM1	-0.417174223

ILMN_1790692	GNLY	0.603689842
ILMN_1654586	RASA3	0.603376818
ILMN_1748767	H3F3A	0.603326677
ILMN_2082035	GALNT1	0.603322079
ILMN_1752520	SLFN11	0.603231831
ILMN_2056032	CD99	0.603123297
ILMN_3209399	KRT18	0.603098497
ILMN_1701558	MAP1A	0.603086915
ILMN_2347234	PRMT1	0.602918858
ILMN_1768595	DLG4	0.602669042
ILMN_1809400	FAM49B	0.602608444
ILMN_1652198	CCM2	0.602568861
ILMN_2415162	sept-01	0.601925714
ILMN_1750678	TIMD4	0.601850364
ILMN_1697864	CXorf38	0.601731587
ILMN_1803560	LAT2	0.601483034
ILMN_1796216	VASH1	0.601372713
ILMN_1730777	KRT19	0.601322531
ILMN_1689968	PLEKHO2	0.601230939
ILMN_2140059	LAMA4	0.601035403
ILMN_1791447	CXCL12	0.600986897
ILMN_2348905	CTLA4	0.600403943
ILMN_1766275	PIK3CD	0.600270687
ILMN_1785005	NCF4	0.599983751
ILMN_1783909	COL6A2	0.599978988
ILMN_2383964	CDCA4	0.599760922
ILMN_1711894	MYB	0.599614447
ILMN_1746565	CD6	0.59927011
ILMN_2319913	DGKA	0.599105464
ILMN_1681798	sept-06	0.598843451
ILMN_1691717	RHBDF2	0.598843015
ILMN_1757877	HCFC1R1	0.598804869
ILMN_1683597	RPL23A	0.598585478
ILMN_1768197	ROD1	0.598554081
ILMN_1736178	AEBP1	0.598076355
ILMN_1777342	PREX1	0.598002135
ILMN_1787345	FKBP11	0.597893093
ILMN_1742001	CD160	0.597871141
ILMN_3294339	LOC100131289	0.597150836
ILMN_1736180	FRAT1	0.59667339
ILMN_1782538	VIM	0.596233172
ILMN_2326953	LAT2	0.596065383
ILMN_1682799	STAMBPL1	0.596032895
ILMN_3241756	FAM136A	0.595989307
ILMN_1676448	WDFY1	0.595422899
ILMN_2370535	GAS2L1	0.59503625
ILMN_1788108	TXNDC5	0.594644508
ILMN_1789599	C1orf151	0.594455471
ILMN_2340027	TSPAN4	0.59381957
ILMN_2365465	XBP1	0.593775767
ILMN_3253579	HAUS8	0.592503306
ILMN_2179837	BANF1	0.59143982
ILMN_1676128	DNMT3A	0.591163242
ILMN_1709747	EXOG	0.591080607
ILMN_1758418	TNFSF13B	0.590937067
ILMN_3236904	LOC648740	0.590877737
ILMN_1692398	CNTNAP1	0.590772255
ILMN_2370414	CHD3	0.590717352
ILMN_1715508	NNMT	0.590544477
ILMN_1707491	FAM30A	0.590449224
ILMN_1803392	P2RX5-TAX1BP:	0.590227142
ILMN_1724686	CLDN1	0.590032246
ILMN_1797875	ALOX5AP	0.589490815
ILMN_2407434	DCLK2	0.588966034
ILMN_2278636	CUX1	0.588620616
ILMN_1748488	TXNL4B	0.58851918
ILMN_3289508	CR604709	0.587948602
ILMN_1799011	CEP250	0.587896074
ILMN_1751368	HNRNPD	0.587671895
ILMN_1811851	PRRX1	0.587388686
ILMN_1708858	CSNK1E	0.587219559
ILMN_1709486	SRPX	0.587180055
ILMN_1761801	ZNF761	0.586840497
ILMN_2119774	CYP2R1	0.586478776
ILMN_1672589	SEMA4B	0.586456275
ILMN_1795495	ERCC5	0.586305673
ILMN_1777190	CFD	0.586088619
ILMN_2310968	RUFY1	0.585874794
ILMN_1670101	CLEC2D	0.585592686
ILMN_3240321	AEN	0.585588791
ILMN_3235832	CCL4L2	0.58547926
ILMN_2412214	HUAT	0.58523934
ILMN_1703692	TUBB	0.585076042
ILMN_3235647	SIK1	0.585035279
ILMN_1742025	OLFM1	0.584576844
ILMN_2384591	HN1	0.583722392
ILMN_1712687	PAK2	0.583487566
ILMN_2413808	CD53	0.583416615
ILMN_2141482	SERPINF1	0.583322678
ILMN_1789639	FMOD	0.583148575
ILMN_1798874	TMEM85	0.58261848
ILMN_1756445	PMF1	0.582299734
ILMN_3176090	RCC2	0.58228734
ILMN_1765876	ARHGAP24	0.582135806

	GOTERM_BP_FAT	response to lipopolysaccharide	21	7.70E-04	1.90E-02
	GOTERM_BP_FAT	response to bacterium	36	1.10E-02	1.50E-01
**Annotation Cluster 35**	**Enrichment Score: 3.07**		**Count**	**P_Value**	**Benjamini**
	GOTERM_BP_FAT	regulation of cytokine production	44	1.20E-05	7.10E-04
	GOTERM_BP_FAT	positive regulation of cytokine production	21	5.60E-03	9.10E-02
	GOTERM_BP_FAT	positive regulation of multicellular organismal process	44	8.80E-03	1.30E-01
**Annotation Cluster 36**	**Enrichment Score: 3.06**		**Count**	**P_Value**	**Benjamini**
	SP_PIR_KEYWORDS	tyrosine-protein kinase	29	2.10E-05	3.70E-04
	GOTERM_MF_FAT	protein tyrosine kinase activity	40	2.30E-05	2.50E-03
	INTERPRO	Tyrosine protein kinase	29	1.80E-04	3.90E-02
	SP_PIR_KEYWORDS	tyrosine-specific protein kinase	15	4.90E-04	6.30E-03
	GOTERM_MF_FAT	non-membrane spanning protein tyrosine kinase activity	14	6.80E-04	3.40E-02
	SMART	TyrKc	29	1.30E-03	2.20E-01
	GOTERM_BP_FAT	peptidyl-tyrosine modification	15	1.40E-03	3.20E-02
	SP_PIR_KEYWORDS	autophosphorylation	14	2.00E-03	1.90E-02
	GOTERM_BP_FAT	peptidyl-tyrosine phosphorylation	14	2.80E-03	5.30E-02
	INTERPRO	Tyrosine protein kinase, active site	21	7.80E-03	4.70E-01
	GOTERM_MF_FAT	transmembrane receptor protein tyrosine kinase activity	13	9.80E-02	7.40E-01
**Annotation Cluster 37**	**Enrichment Score: 2.95**		**Count**	**P_Value**	**Benjamini**
	SP_PIR_KEYWORDS	signal	450	4.20E-08	1.80E-06
	UP_SEQ_FEATURE	signal peptide	450	6.80E-08	8.60E-05
	GOTERM_CC_FAT	extracellular region part	153	4.10E-04	9.10E-03
	UP_SEQ_FEATURE	disulfide bond	361	1.40E-03	3.20E-01
	SP_PIR_KEYWORDS	disulfide bond	371	1.60E-03	1.60E-02
	GOTERM_CC_FAT	extracellular space	104	1.50E-02	1.70E-01
	SP_PIR_KEYWORDS	Secreted	213	2.20E-02	1.50E-01
	SP_PIR_KEYWORDS	glycoprotein	509	4.90E-02	2.60E-01
	GOTERM_CC_FAT	extracellular region	256	2.70E-01	7.70E-01
	UP_SEQ_FEATURE	glycosylation site:N-linked (GlcNAc...)	468	3.10E-01	1.00E+00
**Annotation Cluster 38**	**Enrichment Score: 2.86**		**Count**	**P_Value**	**Benjamini**
	GOTERM_BP_FAT	lymphocyte proliferation	15	3.10E-04	8.60E-03
	GOTERM_BP_FAT	mononuclear cell proliferation	15	5.30E-04	1.40E-02
	GOTERM_BP_FAT	leukocyte proliferation	15	5.30E-04	1.40E-02
	GOTERM_BP_FAT	T cell proliferation	10	3.60E-03	6.50E-02
	GOTERM_BP_FAT	B cell proliferation	6	1.50E-02	1.90E-01
**Annotation Cluster 39**	**Enrichment Score: 2.84**		**Count**	**P_Value**	**Benjamini**
	GOTERM_BP_FAT	regulation of myeloid cell differentiation	23	1.90E-05	1.00E-03
	GOTERM_BP_FAT	positive regulation of myeloid cell differentiation	14	7.20E-05	2.80E-03
	GOTERM_BP_FAT	regulation of myeloid leukocyte differentiation	15	2.30E-04	6.90E-03
	GOTERM_BP_FAT	positive regulation of myeloid leukocyte differentiation	9	7.00E-04	1.80E-02
	GOTERM_BP_FAT	regulation of osteoclast differentiation	9	3.30E-03	6.00E-02
	GOTERM_BP_FAT	positive regulation of osteoclast differentiation	4	2.80E-02	2.90E-01
	GOTERM_BP_FAT	positive regulation of macrophage differentiation	4	2.80E-02	2.90E-01
	GOTERM_BP_FAT	regulation of macrophage differentiation	5	3.70E-02	3.50E-01
**Annotation Cluster 40**	**Enrichment Score: 2.8**		**Count**	**P_Value**	**Benjamini**
	GOTERM_BP_FAT	actin cytoskeleton organization	48	1.70E-04	5.60E-03
	GOTERM_BP_FAT	actin filament-based process	49	4.30E-04	1.10E-02
	GOTERM_BP_FAT	actin filament organization	18	5.40E-03	8.90E-02
	GOTERM_BP_FAT	cytoskeleton organization	70	1.50E-02	1.90E-01
**Annotation Cluster 41**	**Enrichment Score: 2.78**		**Count**	**P_Value**	**Benjamini**
	GOTERM_BP_FAT	macromolecular complex assembly	113	2.50E-04	7.40E-03
	GOTERM_BP_FAT	macromolecular complex subunit organization	117	6.30E-04	1.60E-02
	GOTERM_BP_FAT	cellular macromolecular complex subunit organization	63	2.80E-03	5.40E-02
	GOTERM_BP_FAT	protein complex assembly	84	3.10E-03	5.70E-02
	GOTERM_BP_FAT	protein complex biogenesis	84	3.10E-03	5.70E-02
	GOTERM_BP_FAT	cellular macromolecular complex assembly	56	5.00E-03	8.40E-02
**Annotation Cluster 42**	**Enrichment Score: 2.77**		**Count**	**P_Value**	**Benjamini**
	GOTERM_BP_FAT	regulation of cellular protein metabolic process	95	1.20E-06	1.10E-04
	GOTERM_BP_FAT	regulation of protein modification process	64	6.10E-06	3.90E-04
	GOTERM_BP_FAT	regulation of protein amino acid phosphorylation	40	1.00E-04	3.60E-03
	GOTERM_BP_FAT	regulation of peptidyl-tyrosine phosphorylation	19	1.10E-03	2.50E-02
	GOTERM_BP_FAT	positive regulation of protein metabolic process	47	1.70E-03	3.70E-02
	GOTERM_BP_FAT	positive regulation of protein modification process	38	2.10E-03	4.30E-02
	GOTERM_BP_FAT	positive regulation of cellular protein metabolic process	45	2.20E-03	4.50E-02
	GOTERM_BP_FAT	positive regulation of peptidyl-tyrosine phosphorylation	12	2.40E-02	2.70E-01
	GOTERM_BP_FAT	positive regulation of phosphorus metabolic process	20	3.40E-02	3.30E-01
	GOTERM_BP_FAT	positive regulation of phosphate metabolic process	20	3.40E-02	3.30E-01
	GOTERM_BP_FAT	positive regulation of phosphorylation	19	4.70E-02	4.00E-01
	GOTERM_BP_FAT	positive regulation of protein amino acid phosphorylation	17	7.40E-02	5.30E-01
**Annotation Cluster 43**	**Enrichment Score: 2.68**		**Count**	**P_Value**	**Benjamini**
	GOTERM_BP_FAT	negative regulation of response to stimulus	30	5.70E-06	3.70E-04
	GOTERM_BP_FAT	regulation of inflammatory response	21	6.40E-04	1.60E-02
	GOTERM_BP_FAT	negative regulation of defense response	11	9.40E-03	1.40E-01
	GOTERM_BP_FAT	negative regulation of inflammatory response	9	3.00E-02	3.00E-01
	GOTERM_BP_FAT	negative regulation of response to external stimulus	12	3.70E-02	3.50E-01
**Annotation Cluster 44**	**Enrichment Score: 2.66**		**Count**	**P_Value**	**Benjamini**
	GOTERM_BP_FAT	chordate embryonic development	60	1.90E-03	3.90E-02
	GOTERM_BP_FAT	embryonic development ending in birth or egg hatching	60	2.30E-03	4.60E-02
	GOTERM_BP_FAT	in utero embryonic development	36	2.50E-03	4.90E-02
**Annotation Cluster 45**	**Enrichment Score: 2.61**		**Count**	**P_Value**	**Benjamini**
	SP_PIR_KEYWORDS	basement membrane	13	7.20E-04	8.40E-03
	GOTERM_CC_FAT	extracellular matrix part	26	3.70E-03	6.10E-02
	GOTERM_CC_FAT	basement membrane	19	5.50E-03	8.20E-02
**Annotation Cluster 46**	**Enrichment Score: 2.61**		**Count**	**P_Value**	**Benjamini**
	GOTERM_BP_FAT	viral reproductive process	19	3.20E-04	9.00E-03
	GOTERM_BP_FAT	viral infectious cycle	14	1.40E-03	3.20E-02
	GOTERM_BP_FAT	viral reproduction	19	1.90E-03	3.90E-02
	GOTERM_BP_FAT	initiation of viral infection	7	4.40E-02	3.90E-01
**Annotation Cluster 47**	**Enrichment Score: 2.57**		**Count**	**P_Value**	**Benjamini**
	GOTERM_BP_FAT	regulation of lymphocyte activation	41	8.00E-07	8.30E-05
	GOTERM_BP_FAT	negative regulation of immune system process	27	3.60E-06	2.80E-04
	GOTERM_BP_FAT	regulation of mononuclear cell proliferation	25	4.70E-05	2.10E-03
	GOTERM_BP_FAT	regulation of leukocyte proliferation	25	4.70E-05	2.10E-03
	GOTERM_BP_FAT	regulation of lymphocyte proliferation	24	1.10E-04	3.90E-03

ILMN_1665435	GPER	-0.41717648
ILMN_1719753	VGLL1	-0.417193327
ILMN_1750092	SEPSECS	-0.417318057
ILMN_1889669	C17orf107	-0.417368407
ILMN_1728965	C11orf57	-0.417516536
ILMN_1872769	BX103063	-0.417517017
ILMN_3230215	TOX2	-0.417517707
ILMN_1666403	GCSH	-0.417535439
ILMN_2136737	OR5M10	-0.417537723
ILMN_1668531	BC029817	-0.417592356
ILMN_1756393	VWA5B2	-0.417629432
ILMN_2149292	TMEM40	-0.417642781
ILMN_1785095	ATP6V0E2	-0.417649878
ILMN_3273383	AY461701	-0.4177177
ILMN_1775569	CRISP2	-0.417746142
ILMN_2075221	C17orf47	-0.41779733
ILMN_1788530	ADPRHL1	-0.417812312
ILMN_1666364	COQ10A	-0.417819305
ILMN_1750018	SEL1L2	-0.417836011
ILMN_1910784	AI809077	-0.41801807
ILMN_1756693	ARHGAP17	-0.418053307
ILMN_1658416	MRPS18C	-0.418109576
ILMN_1710219	CALCR	-0.418120921
ILMN_2158548	NACA2	-0.41822759
ILMN_2406656	GATA3	-0.41833134
ILMN_1764043	TTL	-0.418349306
ILMN_3226181	NUDT7	-0.418363416
ILMN_1804248	FDPS	-0.418368569
ILMN_1757281	GPRIN1	-0.418415079
ILMN_1779412	OR2AT4	-0.41841926
ILMN_1784678	PBX1	-0.418431011
ILMN_1766920	EP400NL	-0.418538419
ILMN_1824784	BX111027	-0.418561031
ILMN_1680081	B3GNT6	-0.418626007
ILMN_1769280	SPRY1	-0.418670787
ILMN_1684549	RBM39	-0.418780068
ILMN_1870923	AI125404	-0.418850674
ILMN_1754241	C16orf73	-0.418896555
ILMN_1803279	TMED5	-0.418899959
ILMN_1702310	LGR5	-0.418921971
ILMN_2290776	AGBL5	-0.418939526
ILMN_2073289	MTSS1	-0.419021742
ILMN_1742250	CCNH	-0.419063665
ILMN_3204346	FAM196B	-0.419084348
ILMN_3206565	XAGE5	-0.419233545
ILMN_1905072	CX758427	-0.41932768
ILMN_1773886	MED21	-0.419373684
ILMN_1652379	SUCLG2	-0.419375033
ILMN_3256693	AK130189	-0.419409486
ILMN_1757867	MTM	-0.419499016
ILMN_2195236	PGRMC2	-0.419566013
ILMN_1692762	NA	-0.419608944
ILMN_1698171	INCENP	-0.419650816
ILMN_1765017	COL6A2	-0.419661235
ILMN_3253310	BC150587	-0.419757456
ILMN_2093810	POMP	-0.419811917
ILMN_1670959	CEACAM5	-0.419821814
ILMN_1761511	APOH	-0.419855543
ILMN_2319952	VDR	-0.41986632
ILMN_1910732	AI829589	-0.41993127
ILMN_2397230	USP16	-0.420133526
ILMN_1813530	AGT	-0.4202188
ILMN_1784630	KBTBD11	-0.420259506
ILMN_1731670	LPA	-0.420401887
ILMN_2227248	SLAMF9	-0.420471703
ILMN_1752810	LARP6	-0.420513821
ILMN_2415583	HS6ST2	-0.420538164
ILMN_1798166	POM121C	-0.42055161
ILMN_1763419	TOP1	-0.420570219
ILMN_1773395	BLOC1S1-RDH5	-0.420667133
ILMN_1813517	DISP2	-0.420698638
ILMN_1834696	BQ187514	-0.420778244
ILMN_1750262	BC132949	-0.420805456
ILMN_2195060	OR8K5	-0.420889101
ILMN_3187273	PDZRN3	-0.42089265
ILMN_1676545	AK093382	-0.420922085
ILMN_1774387	ZHX3	-0.420965929
ILMN_1801045	SPIN3	-0.421072578
ILMN_1793651	UBE2N	-0.421121413
ILMN_1778734	FAN1	-0.421127836
ILMN_3239232	C11orf86	-0.421274671
ILMN_1846763	AI393318	-0.421275522
ILMN_3272441	PTPRQ	-0.421335239
ILMN_1879041	BM673750	-0.421349401
ILMN_2133146	NUDT21	-0.421401033
ILMN_1684227	GPR146	-0.421442163
ILMN_1789505	ITPR1	-0.421455275
ILMN_3244650	FLJ41941	-0.421707176
ILMN_3284078	KRTAP16-1	-0.421830054
ILMN_1706615	SLC22A7	-0.421864526
ILMN_1886642	BX096476	-0.42190229
ILMN_3242125	VSTM2A	-0.421968091
ILMN_2227368	SELT	-0.42211969
ILMN_3232949	FAM71E2	-0.42216904

ILMN_2408987	SMARCD3	0.582012338
ILMN_2181968	CBL	0.581676762
ILMN_2049536	TRPV2	0.581354955
ILMN_3245923	AK301885	0.581079914
ILMN_1796409	C1QB	0.580684682
ILMN_1780397	TRAF3IP3	0.580663775
ILMN_1811507	LBH	0.580488991
ILMN_1703538	AIF1	0.58038134
ILMN_1718769	ITSN1	0.580361282
ILMN_2376108	PSMB9	0.580040355
ILMN_1811313	SLIT3	0.579817647
ILMN_1768181	TOR3A	0.579242482
ILMN_1744212	INPP5D	0.578964428
ILMN_1737170	FLII	0.578891312
ILMN_3248324	MGC16384	0.578882471
ILMN_1801766	CCDC109B	0.578626489
ILMN_1742872	UBA2	0.578590497
ILMN_1667813	RPL7	0.57855642
ILMN_3311070	MIR33B	0.578038666
ILMN_2402392	COL8A1	0.57797605
ILMN_1813314	HIST1H2BK	0.577956811
ILMN_1699711	DEDD	0.577916889
ILMN_1655876	TMEM159	0.577869237
ILMN_1789839	GTF3C1	0.577848579
ILMN_1679482	TUBA3C	0.577745347
ILMN_1744239	FEM1B	0.577659125
ILMN_2361427	PMS2P3	0.577624327
ILMN_1651958	MGP	0.577584136
ILMN_2080342	LGALS9C	0.577393023
ILMN_1723042	CLDN3	0.577331445
ILMN_1796712	S100A10	0.577000053
ILMN_1800091	RARRES1	0.576948689
ILMN_1675848	MYL12A	0.576938145
ILMN_2325763	VCAM1	0.576839706
ILMN_1730670	FSTL3	0.576514432
ILMN_1683082	RPUSD1	0.576101515
ILMN_1710937	IFI16	0.575044095
ILMN_1668876	HECTD2	0.57449214
ILMN_2307861	COL6A3	0.574443407
ILMN_1819608	ARRB1	0.574418072
ILMN_1682929	SYTL2	0.57403203
ILMN_1681101	MARCH1	0.57384308
ILMN_1756204	RPS6KA4	0.573830573
ILMN_1713901	KDELR3	0.573829026
ILMN_1811702	GRN	0.573654683
ILMN_1693338	CYP1B1	0.573647424
ILMN_1780806	ANKRD36B	0.573644256
ILMN_3302905	RPL23A	0.573315862
ILMN_1717793	C19orf33	0.573096317
ILMN_1712950	PFN1	0.572880495
ILMN_2349138	CDC42SE1	0.572511379
ILMN_3242485	TLK2	0.572449425
ILMN_1735594	CDC42SE2	0.571971164
ILMN_1800317	WNT5A	0.571920647
ILMN_1815023	PIM1	0.571882145
ILMN_1754179	AP1G2	0.57104597
ILMN_2042651	EVI2B	0.571000196
ILMN_2402930	H3F3A	0.570954992
ILMN_2348808	SNX16	0.570929403
ILMN_1730622	EVL	0.570906005
ILMN_1695356	RNF166	0.570833737
ILMN_1671295	MST151	0.570817484
ILMN_1798819	DTNB	0.57077645
ILMN_1782977	UBA52	0.570731865
ILMN_2090105	TAGLN2	0.570351273
ILMN_2154115	PSD4	0.570019016
ILMN_1803977	GALNT1	0.569910276
ILMN_3236021	ANKRD36	0.569755972
ILMN_1787509	PRIC285	0.569500766
ILMN_1687442	TNIK	0.569478593
ILMN_3215206	SMN1	0.569421985
ILMN_1659227	CD79A	0.569344616
ILMN_1712305	CYBRD1	0.569302846
ILMN_1790819	MEMO1	0.569113584
ILMN_3226613	CLEC2D	0.568930384
ILMN_2232177	ACTN1	0.568653487
ILMN_3248562	SNORD49A	0.568646959
ILMN_2067890	CXCL11	0.568627161
ILMN_1809433	XBP1	0.568567724
ILMN_2243912	RAB37	0.568563566
ILMN_1678827	SPDYE7P	0.568524789
ILMN_1754121	CSK	0.568372449
ILMN_1711792	GPBP1	0.568242369
ILMN_2405009	C1orf151	0.568205721
ILMN_1747622	CD33	0.568194159
ILMN_2386008	MPZL1	0.568182182
ILMN_1734740	GPR65	0.568151875
ILMN_1702738	KLC3	0.567854449
ILMN_1728435	C12orf45	0.567710946
ILMN_1725612	NUP50	0.567502654
ILMN_1745807	TMEM62	0.567296705
ILMN_1792473	AIF1	0.567223698
ILMN_1681239	MYO1F	0.567095375

	GOTERM_BP_FAT	negative regulation of leukocyte activation	16	2.90E-03	5.50E-02
	GOTERM_BP_FAT	negative regulation of lymphocyte activation	15	4.60E-03	7.80E-02
	GOTERM_BP_FAT	negative regulation of cell activation	16	5.80E-03	9.40E-02
	GOTERM_BP_FAT	regulation of T cell proliferation	16	6.90E-03	1.10E-01
	GOTERM_BP_FAT	negative regulation of B cell activation	7	1.70E-02	2.10E-01
	GOTERM_BP_FAT	negative regulation of T cell activation	11	2.80E-02	2.90E-01
	GOTERM_BP_FAT	negative regulation of mononuclear cell proliferation	9	3.60E-02	3.40E-01
	GOTERM_BP_FAT	negative regulation of leukocyte proliferation	9	3.60E-02	3.40E-01
	GOTERM_BP_FAT	negative regulation of lymphocyte proliferation	9	3.60E-02	3.40E-01
	GOTERM_BP_FAT	negative regulation of B cell proliferation	4	6.40E-02	4.90E-01
	GOTERM_BP_FAT	negative regulation of T cell proliferation	7	1.00E-01	6.30E-01
	GOTERM_BP_FAT	regulation of activated T cell proliferation	3	4.40E-01	9.70E-01
**Annotation Cluster 48**	**Enrichment Score: 2.53**		**Count**	**P_Value**	**Benjamini**
	GOTERM_BP_FAT	regulation of cell morphogenesis	33	8.70E-05	3.30E-03
	GOTERM_BP_FAT	positive regulation of cell projection organization	16	3.30E-04	9.00E-03
	GOTERM_BP_FAT	regulation of cell morphogenesis involved in differentiation	21	7.70E-04	1.90E-02
	GOTERM_BP_FAT	regulation of cell development	42	9.90E-04	2.40E-02
	GOTERM_BP_FAT	positive regulation of axonogenesis	10	2.00E-03	4.10E-02
	GOTERM_BP_FAT	regulation of cell projection organization	22	2.10E-03	4.40E-02
	GOTERM_BP_FAT	positive regulation of cellular component organization	37	2.20E-03	4.40E-02
	GOTERM_BP_FAT	regulation of axonogenesis	16	2.90E-03	5.50E-02
	GOTERM_BP_FAT	positive regulation of cell development	18	3.40E-03	6.10E-02
	GOTERM_BP_FAT	regulation of developmental growth	14	3.40E-03	6.10E-02
	GOTERM_BP_FAT	regulation of neuron projection development	18	4.00E-03	7.00E-02
	GOTERM_BP_FAT	positive regulation of neurogenesis	16	4.20E-03	7.20E-02
	GOTERM_BP_FAT	regulation of nervous system development	36	1.00E-02	1.50E-01
	GOTERM_BP_FAT	regulation of neuron differentiation	26	1.90E-02	2.20E-01
	GOTERM_BP_FAT	regulation of axon extension	7	2.90E-02	3.00E-01
	GOTERM_BP_FAT	regulation of neurogenesis	29	4.90E-02	4.10E-01
**Annotation Cluster 49**	**Enrichment Score: 2.41**		**Count**	**P_Value**	**Benjamini**
	SP_PIR_KEYWORDS	chromatin regulator	43	1.40E-04	2.10E-03
	GOTERM_BP_FAT	chromatin organization	71	2.50E-04	7.40E-03
	GOTERM_CC_FAT	chromatin	42	5.90E-04	1.30E-02
	GOTERM_BP_FAT	chromatin modification	52	1.40E-03	3.20E-02
	GOTERM_BP_FAT	chromosome organization	83	1.50E-03	3.30E-02
	GOTERM_CC_FAT	nuclear chromatin	16	2.00E-03	3.60E-02
	GOTERM_CC_FAT	nuclear chromosome part	26	6.50E-03	9.10E-02
	GOTERM_CC_FAT	chromosomal part	62	2.10E-02	2.00E-01
	GOTERM_CC_FAT	nuclear chromosome	30	2.30E-02	2.10E-01
	GOTERM_CC_FAT	chromosome	72	2.30E-02	2.10E-01
	GOTERM_BP_FAT	covalent chromatin modification	24	3.20E-02	3.20E-01
	GOTERM_BP_FAT	histone modification	22	6.70E-02	5.00E-01
**Annotation Cluster 50**	**Enrichment Score: 2.39**		**Count**	**P_Value**	**Benjamini**
	SP_PIR_KEYWORDS	SH2 domain	25	9.20E-04	1.00E-02
	UP_SEQ_FEATURE	domain:SH2	21	3.90E-03	5.30E-01
	INTERPRO	SH2 motif	24	4.30E-03	3.70E-01
	SMART	SH2	24	1.80E-02	5.50E-01
**Annotation Cluster 51**	**Enrichment Score: 2.38**		**Count**	**P_Value**	**Benjamini**
	BIOCARTA	CTL mediated immune response against target cells	11	1.30E-04	3.60E-02
	BIOCARTA	Lck and Fyn tyrosine kinases in initiation of TCR Activation	9	2.70E-04	3.70E-02
	BIOCARTA	HIV Induced T Cell Apoptosis	8	3.60E-04	3.30E-02
	BIOCARTA	T Helper Cell Surface Molecules	9	6.80E-04	4.50E-02
	BIOCARTA	T Cytotoxic Cell Surface Molecules	9	6.80E-04	4.50E-02
	INTERPRO	Phosphorylated immunoreceptor signaling ITAM	6	8.90E-04	1.10E-01
	UP_SEQ_FEATURE	domain:ITAM	6	1.40E-03	3.20E-01
	SMART	ITAM	6	1.60E-03	1.90E-01
	GOTERM_CC_FAT	T cell receptor complex	7	1.60E-03	3.10E-02
	BIOCARTA	The Co-Stimulatory Signal During T-cell Activation	11	1.90E-03	6.20E-02
	BIOCARTA	Activation of Csk by cAMP-dependent Protein Kinase Inhibits Signaling through the T Cell Receptor	10	3.00E-03	7.90E-02
	GOTERM_CC_FAT	alpha-beta T cell receptor complex	4	6.70E-03	9.20E-02
	BIOCARTA	T Cell Receptor and CD3 Complex	4	2.90E-02	3.40E-01
	OMIM_DISEASE	Severe combined immunodeficiency, T cell-negative, B-cell/natural killer-cell positive	3	3.50E-02	9.90E-01
	PIR_SUPERFAMILY	PIRSF001993:T-cell surface glycoprotein CD3 delta chain	3	4.50E-02	1.00E+00
	BIOCARTA	IL 17 Signaling Pathway	7	6.80E-02	4.90E-01
	BIOCARTA	IL12 and Stat4 Dependent Signaling Pathway in Th1 Development	7	2.10E-01	7.80E-01
	BIOCARTA	Role of Tob in T-cell activation	5	3.20E-01	8.70E-01
**Annotation Cluster 52**	**Enrichment Score: 2.36**		**Count**	**P_Value**	**Benjamini**
	KEGG_PATHWAY	Pathogenic Escherichia coli infection	27	1.50E-08	4.10E-07
	UP_SEQ_FEATURE	site:Involved in polymerization	5	6.90E-04	2.10E-01
	KEGG_PATHWAY	Gap junction	25	1.50E-03	9.70E-03
	INTERPRO	Tubulin/FtsZ, 2-layer sandwich domain	9	2.40E-03	2.50E-01
	INTERPRO	Tubulin, conserved site	9	3.30E-03	3.10E-01
	PIR_SUPERFAMILY	PIRSF002306:tubulin	9	4.20E-03	7.30E-01
	INTERPRO	Tubulin/FtsZ, GTPase domain	9	4.50E-03	3.60E-01
	INTERPRO	Tubulin	9	4.50E-03	3.60E-01
	GOTERM_BP_FAT	protein polymerization	14	5.00E-03	8.40E-02
	INTERPRO	Alpha tubulin	5	1.40E-02	5.90E-01
	GOTERM_BP_FAT	cellular protein complex assembly	27	9.10E-02	5.90E-01
	INTERPRO	Beta tubulin	4	1.30E-01	9.80E-01
	GOTERM_BP_FAT	microtubule-based movement	17	3.10E-01	9.20E-01
	INTERPRO	Beta tubulin, autoregulation binding site	4	3.50E-01	1.00E+00
**Annotation Cluster 53**	**Enrichment Score: 2.35**		**Count**	**P_Value**	**Benjamini**
	GOTERM_BP_FAT	regulation of cell motion	43	1.30E-04	4.50E-03
	GOTERM_BP_FAT	regulation of locomotion	39	1.80E-03	3.90E-02
	GOTERM_BP_FAT	regulation of cell migration	35	2.30E-03	4.60E-02
	GOTERM_BP_FAT	positive regulation of cell motion	22	7.10E-03	1.10E-01
	GOTERM_BP_FAT	positive regulation of cell migration	18	4.10E-02	3.70E-01
	GOTERM_BP_FAT	positive regulation of locomotion	19	5.10E-02	4.20E-01
**Annotation Cluster 54**	**Enrichment Score: 2.34**		**Count**	**P_Value**	**Benjamini**
	SP_PIR_KEYWORDS	rna-binding	96	2.70E-06	8.10E-05
	UP_SEQ_FEATURE	domain:RRM 2	22	3.20E-03	5.00E-01
	UP_SEQ_FEATURE	domain:RRM 1	22	3.20E-03	5.00E-01
	INTERPRO	RNA recognition motif, RNP-1	39	4.30E-03	3.60E-01

ILMN_3300358	ZNF84	-0.422285533
ILMN_1834180	LOC440602	-0.422334579
ILMN_1800873	SEZ6L2	-0.422495904
ILMN_1762330	C1D	-0.422498696
ILMN_1773906	NCOA4	-0.422502695
ILMN_2216637	STK32B	-0.422559387
ILMN_1708627	SRP9	-0.42256786
ILMN_1797209	TSGA14	-0.422583969
ILMN_2323418	KRIT1	-0.422599341
ILMN_1770336	AGRP	-0.42267809
ILMN_1784287	TGFBR3	-0.422755965
ILMN_1654759	WWC2	-0.422779311
ILMN_1662801	ZMYM3	-0.422782814
ILMN_1671542	NLGN4X	-0.422906898
ILMN_1725969	RPP14	-0.423034722
ILMN_1803611	SNAPC3	-0.423044429
ILMN_3284020	LOC100132319	-0.423058029
ILMN_1739734	CLTCL1	-0.423068014
ILMN_3242633	SNORD127	-0.423134839
ILMN_1876924	WNK1	-0.423240219
ILMN_1823207	CR618071	-0.423280106
ILMN_1772446	C12orf34	-0.423383445
ILMN_1705116	SLC22A23	-0.423397565
ILMN_1799628	NBR1	-0.423398946
ILMN_1762629	ANO9	-0.423463037
ILMN_1786658	BOLA3	-0.423466058
ILMN_1714461	RNF14	-0.42348096
ILMN_1810554	PDPK1	-0.423578863
ILMN_1717406	TTC34	-0.423620949
ILMN_1769155	CYP2C8	-0.423644686
ILMN_2061116	MATN1	-0.423661167
ILMN_1776542	SLC35C2	-0.42370559
ILMN_1674703	UBA5	-0.423760487
ILMN_1842533	AL117577	-0.423788834
ILMN_1724783	D2HGDH	-0.423806521
ILMN_1781031	CTBP2	-0.423856291
ILMN_2176768	SEPHS1	-0.423898114
ILMN_3251298	THNSL1	-0.423922292
ILMN_1784709	GNPDA1	-0.423975124
ILMN_2081988	LANCL1	-0.424008223
ILMN_1662168	SP3	-0.424161688
ILMN_1882560	NA	-0.424335798
ILMN_1804217	RFX7	-0.424441971
ILMN_1790105	SUMO1	-0.424530931
ILMN_1719471	MSH3	-0.424640368
ILMN_1674759	LPCAT4	-0.424755317
ILMN_1742901	RSPH10B2	-0.424758805
ILMN_1866244	TCRA	-0.424808584
ILMN_1666718	NA	-0.424842592
ILMN_1860662	BM264234	-0.424867835
ILMN_1763695	C9orf24	-0.424914916
ILMN_3241354	FLJ00317	-0.424920827
ILMN_2354269	FAM164C	-0.424923388
ILMN_1666881	AK125965	-0.424938226
ILMN_1735905	LOC283685	-0.424954476
ILMN_2223922	AGTPBP1	-0.42495749
ILMN_3239103	LOC340357	-0.424979405
ILMN_1758811	IMPA1	-0.425017515
ILMN_3248961	MAPK1IP1L	-0.425101829
ILMN_1690693	CTPS2	-0.425158785
ILMN_3201658	VDAC1	-0.425179603
ILMN_1708432	PLCB1	-0.425186553
ILMN_1801240	OR5D14	-0.425220314
ILMN_1806052	UNC119	-0.425233643
ILMN_2102515	PGAM4	-0.425277772
ILMN_1865680	BM677017	-0.42528074
ILMN_1697166	C9orf78	-0.425352025
ILMN_1879031	BX115902	-0.425379396
ILMN_1780041	OR4C3	-0.425388382
ILMN_1715396	SNORD56B	-0.425432086
ILMN_2376553	SLC23A1	-0.425455281
ILMN_1718253	TAS2R3	-0.425642502
ILMN_1821279	BM678326	-0.425761247
ILMN_1763011	C7orf10	-0.425833742
ILMN_1847133	AW270402	-0.426097743
ILMN_2096083	PLEKHA5	-0.426356143
ILMN_1668285	APBA1	-0.426433214
ILMN_1845347	BM930795	-0.42643452
ILMN_1765103	CST8	-0.426506898
ILMN_1755792	STK38L	-0.42657714
ILMN_2115350	TNXB	-0.426619191
ILMN_1770203	TUBGCP6	-0.426633001
ILMN_1806407	SEMA6D	-0.426710511
ILMN_1666310	BSX	-0.426743575
ILMN_1656399	TCEAL8	-0.426768837
ILMN_1734352	TPH2	-0.426797854
ILMN_1860308	NA	-0.426814959
ILMN_1662158	RPL26L1	-0.42683373
ILMN_1701468	HGFAC	-0.426844103
ILMN_1675756	KCNJ15	-0.426876464
ILMN_1690993	NEUROG2	-0.426888432
ILMN_1767393	GRIA3	-0.426918485
ILMN_1705587	GPR97	-0.427007449

ILMN_1739103	MPZL1	0.567073062
ILMN_1739496	PRRX1	0.566988682
ILMN_1785902	C1QC	0.566423932
ILMN_1684306	S100A4	0.566364041
ILMN_1758895	CTSK	0.566225952
ILMN_2400935	TAGLN	0.565933422
ILMN_1782403	PRR11	0.565643498
ILMN_1656998	CHMP4B	0.565633187
ILMN_1703433	PLSCR3	0.565592831
ILMN_3307796	IKZF5	0.565447846
ILMN_1719449	DCLK2	0.565426547
ILMN_1790637	C11orf80	0.565344718
ILMN_2371685	UBE2E1	0.565285802
ILMN_1669362	IGFBP6	0.565148947
ILMN_2313079	NLRP1	0.564835846
ILMN_1698144	CFH	0.564747164
ILMN_2103362	ARHGAP27	0.564712021
ILMN_2161577	CXCL6	0.564709362
ILMN_1652512	C2CD2	0.564640359
ILMN_2383419	GMEB1	0.564210631
ILMN_1680453	ITM2C	0.564180717
ILMN_1677104	TRAF5	0.564123125
ILMN_1800261	TUBA1B	0.563732432
ILMN_1749372	GGT5	0.563558012
ILMN_2184184	ANXA1	0.563465251
ILMN_3240793	OR2A4	0.563448369
ILMN_1658821	SAMD1	0.56332409
ILMN_1726809	BHLHE41	0.563117144
ILMN_1772991	MIR671	0.563015281
ILMN_1761463	EFHD2	0.562964442
ILMN_1805519	DQ893812	0.56265291
ILMN_1762899	EGR1	0.562533075
ILMN_1741404	MSC	0.562517704
ILMN_1788689	PHIP	0.562079612
ILMN_1781824	FASLG	0.561748179
ILMN_1700541	FBLN1	0.561618666
ILMN_2206722	FER1L4	0.561580205
ILMN_2159453	STXBP2	0.561259059
ILMN_1678605	CDC123	0.561180066
ILMN_1791067	TESK1	0.561090609
ILMN_1760347	SRGN	0.560746241
ILMN_1810559	RHOQ	0.560449631
ILMN_2255133	BCL11A	0.560445274
ILMN_1777296	ACTB	0.560083048
ILMN_1778360	PYGB	0.560076557
ILMN_1715332	TTC21A	0.560059309
ILMN_2392043	SPI1	0.560026049
ILMN_2085862	SLC15A3	0.559642175
ILMN_1773964	H1FX	0.559634508
ILMN_1745242	PLSCR1	0.559399187
ILMN_1687533	SEMA4D	0.558835195
ILMN_1691662	LOC100101266	0.558677748
ILMN_1698307	DBNL	0.558653506
ILMN_1778064	FICD	0.558502593
ILMN_2076640	KHDRBS1	0.558396359
ILMN_1673682	GATAD2A	0.558386315
ILMN_1760563	PRRC2A	0.55831406
ILMN_1775762	GNAI2	0.55803172
ILMN_2398274	PYCARD	0.557936114
ILMN_1746408	MIDN	0.557904658
ILMN_1770247	HOXA10-HOXA!	0.557814311
ILMN_3266186	HDAC7	0.557527385
ILMN_2114422	NOD1	0.557283755
ILMN_2379326	MAP3K7	0.557021827
ILMN_1662768	NHEDC2	0.556802758
ILMN_1785061	EPHB2	0.556615308
ILMN_1751161	COL7A1	0.556580643
ILMN_1809751	EIF2C3	0.55647979
ILMN_1706643	COL6A3	0.556342419
ILMN_1772177	ASCC3	0.556308469
ILMN_1801043	GSN	0.556269459
ILMN_2104356	COL1A2	0.5560443
ILMN_1737344	DDX41	0.556015692
ILMN_2335604	SKAP1	0.556009613
ILMN_1906187	LOC283070	0.555788016
ILMN_1737683	FGG	0.555710428
ILMN_1735467	NPIPL2	0.555517486
ILMN_1689725	RPLP1	0.55534293
ILMN_3247064	SNRNP40	0.555169482
ILMN_1748283	PIM2	0.554839431
ILMN_1701596	RPS20	0.554803793
ILMN_2330243	NUDT1	0.554605791
ILMN_1724059	GAS2L1	0.554260819
ILMN_1798706	GPR183	0.554191974
ILMN_1706539	KDM3B	0.554169852
ILMN_1786720	PROM1	0.554003049
ILMN_1677484	SNAPC4	0.553837739
ILMN_2402806	TRPC4AP	0.553715824
ILMN_1723944	TARP	0.553487836
ILMN_1674519	ZP2	0.553413507
ILMN_3203801	ZNF532	0.553406184
ILMN_3239965	IDO1	0.553206399

	INTERPRO	Nucleotide-binding, alpha-beta plait	39	5.10E-03	3.70E-01
	SMART	RRM	39	2.80E-02	6.10E-01
	UP_SEQ_FEATURE	domain:RRM 3	10	9.70E-02	1.00E+00
	UP_SEQ_FEATURE	domain:RRM	19	1.10E-01	1.00E+00
**Annotation Cluster 55**	**Enrichment Score: 2.3**		**Count**	**P_Value**	**Benjamini**
	KEGG_PATHWAY	B cell receptor signaling pathway	26	2.90E-05	3.00E-04
	KEGG_PATHWAY	Fc epsilon RI signaling pathway	23	1.20E-03	8.70E-03
	KEGG_PATHWAY	VEGF signaling pathway	16	1.20E-01	3.30E-01
	KEGG_PATHWAY	Non-small cell lung cancer	12	1.50E-01	3.90E-01
**Annotation Cluster 56**	**Enrichment Score: 2.28**		**Count**	**P_Value**	**Benjamini**
	UP_SEQ_FEATURE	short sequence motif:Prevents secretion from ER	15	4.40E-03	5.50E-01
	INTERPRO	Endoplasmic reticulum, targeting sequence	13	4.50E-03	3.50E-01
	GOTERM_CC_FAT	endoplasmic reticulum lumen	19	7.30E-03	9.80E-02
**Annotation Cluster 57**	**Enrichment Score: 2.28**		**Count**	**P_Value**	**Benjamini**
	GOTERM_BP_FAT	T cell selection	10	1.80E-04	5.70E-03
	GOTERM_BP_FAT	negative T cell selection	6	2.30E-03	4.60E-02
	GOTERM_BP_FAT	thymic T cell selection	7	2.70E-03	5.20E-02
	GOTERM_BP_FAT	negative thymic T cell selection	5	1.00E-02	1.50E-01
	GOTERM_BP_FAT	T cell differentiation in the thymus	9	1.30E-02	1.80E-01
	GOTERM_BP_FAT	positive T cell selection	5	1.70E-02	2.10E-01
	GOTERM_BP_FAT	positive thymic T cell selection	4	4.40E-02	3.90E-01
**Annotation Cluster 58**	**Enrichment Score: 2.27**		**Count**	**P_Value**	**Benjamini**
	GOTERM_CC_FAT	T cell receptor complex	7	1.60E-03	3.10E-02
	GOTERM_BP_FAT	regulation of calcium-mediated signaling	8	7.50E-03	1.10E-01
	GOTERM_BP_FAT	positive regulation of calcium-mediated signaling	7	1.30E-02	1.70E-01
**Annotation Cluster 59**	**Enrichment Score: 2.26**		**Count**	**P_Value**	**Benjamini**
	GOTERM_BP_FAT	regulation of inflammatory response	21	6.40E-04	1.60E-02
	GOTERM_BP_FAT	regulation of response to external stimulus	33	3.00E-03	5.70E-02
	GOTERM_BP_FAT	positive regulation of inflammatory response	10	7.90E-03	1.20E-01
	GOTERM_BP_FAT	positive regulation of response to external stimulus	16	9.30E-03	1.40E-01
	GOTERM_BP_FAT	negative regulation of response to external stimulus	12	3.70E-02	3.50E-01
**Annotation Cluster 60**	**Enrichment Score: 2.22**		**Count**	**P_Value**	**Benjamini**
	GOTERM_BP_FAT	regulation of transcription from RNA polymerase II promoter	124	1.00E-04	3.60E-03
	GOTERM_BP_FAT	positive regulation of macromolecule metabolic process	142	1.20E-04	4.10E-03
	GOTERM_MF_FAT	transcription activator activity	70	2.00E-03	7.40E-02
	GOTERM_BP_FAT	positive regulation of cellular biosynthetic process	110	2.40E-03	4.70E-02
	GOTERM_BP_FAT	positive regulation of biosynthetic process	111	2.60E-03	5.20E-02
	GOTERM_BP_FAT	positive regulation of macromolecule biosynthetic process	104	4.10E-03	7.20E-02
	GOTERM_BP_FAT	positive regulation of nitrogen compound metabolic process	99	1.30E-02	1.80E-01
	GOTERM_BP_FAT	positive regulation of gene expression	90	1.50E-02	1.90E-01
	GOTERM_BP_FAT	positive regulation of nucleobase, nucleoside, nucleotide and nucleic acid metabolic process	95	1.90E-02	2.30E-01
	GOTERM_BP_FAT	positive regulation of transcription	86	2.60E-02	2.80E-01
	GOTERM_BP_FAT	positive regulation of transcription, DNA-dependent	73	3.60E-02	3.40E-01
	GOTERM_BP_FAT	positive regulation of RNA metabolic process	73	4.20E-02	3.80E-01
	GOTERM_BP_FAT	positive regulation of transcription from RNA polymerase II promoter	54	1.40E-01	7.10E-01
**Annotation Cluster 61**	**Enrichment Score: 2.07**		**Count**	**P_Value**	**Benjamini**
	SP_PIR_KEYWORDS	nucleus	588	4.50E-10	2.70E-08
	GOTERM_BP_FAT	regulation of transcription from RNA polymerase II promoter	124	1.00E-04	3.60E-03
	GOTERM_MF_FAT	transcription regulator activity	217	2.20E-03	7.40E-02
	SP_PIR_KEYWORDS	Transcription	266	4.50E-03	3.90E-02
	SP_PIR_KEYWORDS	transcription regulation	259	6.40E-03	5.30E-02
	GOTERM_MF_FAT	transcription factor activity	135	4.30E-02	5.30E-01
	GOTERM_BP_FAT	transcription	273	1.60E-01	7.70E-01
	SP_PIR_KEYWORDS	dna-binding	219	1.90E-01	5.80E-01
	GOTERM_BP_FAT	regulation of transcription	329	3.00E-01	9.10E-01
	GOTERM_BP_FAT	regulation of RNA metabolic process	227	4.20E-01	9.70E-01
	GOTERM_MF_FAT	DNA binding	281	5.00E-01	9.90E-01
	GOTERM_BP_FAT	regulation of transcription, DNA-dependent	216	5.90E-01	9.90E-01
**Annotation Cluster 62**	**Enrichment Score: 2.06**		**Count**	**P_Value**	**Benjamini**
	GOTERM_BP_FAT	taxis	39	3.90E-05	1.70E-03
	GOTERM_BP_FAT	chemotaxis	39	3.90E-05	1.70E-03
	SP_PIR_KEYWORDS	inflammatory response	21	2.00E-04	2.80E-03
	INTERPRO	Small chemokine, interleukin-8-like	14	5.40E-04	8.40E-02
	GOTERM_MF_FAT	chemokine activity	15	6.80E-04	3.50E-02
	GOTERM_MF_FAT	chemokine receptor binding	15	1.40E-03	5.70E-02
	SMART	SCY	14	1.80E-03	1.60E-01
	SP_PIR_KEYWORDS	chemotaxis	18	1.80E-03	1.80E-02
	KEGG_PATHWAY	Cytokine-cytokine receptor interaction	52	1.50E-02	6.80E-02
	GOTERM_BP_FAT	locomotory behavior	47	1.60E-02	2.00E-01
	PIR_SUPERFAMILY	PIRSF002522:CXC chemokine	6	2.50E-02	9.90E-01
	INTERPRO	Small chemokine, C-X-C, conserved site	6	4.00E-02	8.40E-01
	INTERPRO	Small chemokine, C-X-C	5	5.60E-02	9.00E-01
	BBID	109.Chemokine_families	13	6.10E-02	1.00E+00
	SP_PIR_KEYWORDS	cytokine	27	9.80E-02	4.00E-01
	INTERPRO	Small chemokine, C-C group, conserved site	6	1.60E-01	9.90E-01
	PIR_SUPERFAMILY	PIRSF001950:small inducible chemokine, C/CC types	6	1.60E-01	1.00E+00
	GOTERM_MF_FAT	cytokine activity	29	1.80E-01	8.70E-01
	GOTERM_BP_FAT	behavior	65	2.00E-01	8.20E-01
	INTERPRO	Small chemokine, C-X-C/Interleukin 8	4	2.20E-01	9.90E-01

ILMN_1744539	GOLGA6L6	-0.427124594
ILMN_3299074	NOX4	-0.427196511
ILMN_1744056	TAAR1	-0.427200336
ILMN_1663054	TXNDC11	-0.427251149
ILMN_2342841	AFTPH	-0.427293872
ILMN_1732140	ABCC11	-0.427369785
ILMN_1713550	LGR6	-0.427382452
ILMN_1783333	C16orf61	-0.427497362
ILMN_1803452	CRCT1	-0.427555658
ILMN_1794989	TMEM191B	-0.427606874
ILMN_1773850	FXC1	-0.427680506
ILMN_1683755	SOX13	-0.427755615
ILMN_1695418	IQGAP3	-0.427775092
ILMN_1732189	SYCE1	-0.427866552
ILMN_1771898	NBPF3	-0.427871066
ILMN_1684106	NR1I3	-0.42788188
ILMN_1778611	GBAS	-0.428005127
ILMN_1783546	CARKD	-0.428044385
ILMN_1775716	GOLGA8G	-0.428109839
ILMN_1808494	ITIH2	-0.42812918
ILMN_1744614	PLEKHG6	-0.428199197
ILMN_2044645	CGB1	-0.428243203
ILMN_1800590	BBS1	-0.428294225
ILMN_1819158	U03241	-0.428377976
ILMN_1785069	KIAA1845	-0.428401812
ILMN_3226841	AK302451	-0.428544204
ILMN_1657478	MAGEL2	-0.428587789
ILMN_1784302	LIPJ	-0.428598261
ILMN_1752793	SAP18	-0.428631753
ILMN_1691884	STC2	-0.428656605
ILMN_2363744	PACRG	-0.428713503
ILMN_1710075	FAM89A	-0.428759811
ILMN_1741970	JUP	-0.428896141
ILMN_3274344	NA	-0.428903196
ILMN_1658806	SLC5A10	-0.428918158
ILMN_1736101	GLIS1	-0.429133688
ILMN_3301551	LPA	-0.429303777
ILMN_3193266	NA	-0.429343629
ILMN_1836172	CN288601	-0.429378193
ILMN_1746952	SLC25A48	-0.42940315
ILMN_1701881	ZNF365	-0.429405299
ILMN_1811345	GLYAT	-0.429439288
ILMN_2171102	MSL2	-0.429444173
ILMN_1792423	LOC644961	-0.42946463
ILMN_1704943	GPN3	-0.429574677
ILMN_3273340	hCG_2014417	-0.429775756
ILMN_2413473	GH2	-0.429859627
ILMN_2387952	FAM134B	-0.42989879
ILMN_1776826	ARHGAP42	-0.4299703
ILMN_3249291	F2	-0.430079256
ILMN_3248035	NTN5	-0.430144778
ILMN_2191634	RPL37	-0.430296327
ILMN_2372698	RGN	-0.430315299
ILMN_3277282	LOC115110	-0.430386399
ILMN_1702700	AF257770	-0.430393088
ILMN_1804509	DPYSL5	-0.430454543
ILMN_1846922	BC042566	-0.430566404
ILMN_3289997	AATK	-0.430588982
ILMN_1797181	LOC93622	-0.430609188
ILMN_1675819	FAM101A	-0.430709189
ILMN_3251467	LRRC58	-0.430856072
ILMN_1729453	TSPAN9	-0.430859832
ILMN_3242059	C6orf221	-0.430860923
ILMN_3309059	MIR93	-0.430882004
ILMN_1807244	STGC3	-0.430997736
ILMN_1766643	HOPX	-0.431033918
ILMN_1748694	NHSL2	-0.431047539
ILMN_1661173	TRIP4	-0.431057639
ILMN_1658123	DAZ2	-0.431068658
ILMN_1729650	PEX7	-0.43110017
ILMN_1875967	BG222050	-0.431129926
ILMN_1669393	GGT1	-0.431134004
ILMN_1877317	AW294700	-0.431142663
ILMN_1756767	EIF5B	-0.431156047
ILMN_3203534	LOC100132474	-0.431207172
ILMN_2401884	IL25	-0.43122188
ILMN_1656170	JAKMIP1	-0.431223211
ILMN_1885583	AK129941	-0.431226915
ILMN_1806633	LOC348926	-0.431368407
ILMN_1667030	HSBP1	-0.431374866
ILMN_1691047	EPS8L2	-0.431379754
ILMN_1802942	AJ292264	-0.431439344
ILMN_1802923	APOC4-APOC2	-0.431531315
ILMN_1827966	AK023961	-0.431624848
ILMN_1677880	MYADML2	-0.431697013
ILMN_2262362	ABCG1	-0.431721461
ILMN_2346856	GGPS1	-0.431748105
ILMN_1763409	LRRC8D	-0.431790485
ILMN_1764871	PIGP	-0.431816064
ILMN_1709635	SLC38A11	-0.431833101
ILMN_1657010	FAM90A12	-0.432183438
ILMN_1772312	BARX1	-0.432227998

ILMN_3232889	CCDC144B	0.55292122
ILMN_1710923	SLAMF7	0.552702793
ILMN_1665964	GAB2	0.552536823
ILMN_1680403	SSR4	0.551702875
ILMN_2267383	CU687675	0.551410183
ILMN_1656082	H3F3A	0.55134115
ILMN_1664691	DAPP1	0.550969145
ILMN_3235148	FLJ43681	0.550909586
ILMN_1734878	CD79A	0.550883029
ILMN_1752899	BCL11A	0.550696952
ILMN_1678842	THBS2	0.55068604
ILMN_1805999	MYO1D	0.550593852
ILMN_1744604	CYBA	0.550384242
ILMN_3246391	SNORD52	0.550299649
ILMN_2367753	ATP2B4	0.549909557
ILMN_1745533	FAM117A	0.54958491
ILMN_1700935	HAS2	0.549571013
ILMN_3246560	EMB	0.549285006
ILMN_1727043	GLT25D1	0.548728586
ILMN_2366041	ITM2C	0.548725912
ILMN_1738681	NUP62	0.548626944
ILMN_1764754	RAMP1	0.548623472
ILMN_1720124	RCC2	0.548578614
ILMN_1701424	LAMC2	0.548480763
ILMN_1702585	SET	0.548444111
ILMN_1734653	FNDC1	0.548360055
ILMN_1652223	WDR91	0.5483342
ILMN_2124757	DKFZp547I014	0.548217269
ILMN_1802523	HS3ST5	0.548055696
ILMN_2402341	MAPK3	0.54803558
ILMN_3229210	FLJ45340	0.548027104
ILMN_2145501	ANGPTL1	0.547941147
ILMN_1772148	SULT1C4	0.54793533
ILMN_1661266	HLA-DQB1	0.547896835
ILMN_1716080	CBL	0.547829669
ILMN_2071809	MGP	0.547754686
ILMN_1651278	SNIP1	0.547535533
ILMN_1749011	NECAP2	0.547404754
ILMN_1776464	PARP4	0.54721293
ILMN_1779185	SPECC1L	0.547141821
ILMN_1781468	SMAP2	0.547100114
ILMN_1658411	CHD4	0.546917272
ILMN_2367707	PKN1	0.546643632
ILMN_2408400	NSUN5	0.546475799
ILMN_1744113	SCNM1	0.546437795
ILMN_1786197	NR2F1	0.546252144
ILMN_1805693	GMIP	0.546201792
ILMN_2323491	NUP62	0.546185502
ILMN_1702592	WDR73	0.545864042
ILMN_1910908	BG205162	0.54585304
ILMN_1691487	TRAF2	0.545723381
ILMN_1669727	WAC	0.545671025
ILMN_2375319	RASGRP2	0.545668792
ILMN_2340131	MAPK10	0.545477793
ILMN_1698666	CST6	0.54546783
ILMN_1688381	BPTF	0.545358876
ILMN_1662166	PTK7	0.545296129
ILMN_1711994	TCIRG1	0.54517658
ILMN_1769911	SLC38A1	0.545103305
ILMN_1712389	CMTM1	0.545019123
ILMN_1803429	CD44	0.544977202
ILMN_1815652	O1/O11 and JK:	0.544935566
ILMN_1654031	TPTE2	0.544926855
ILMN_1699160	ITK	0.544809408
ILMN_1795442	LAMA4	0.544654643
ILMN_1666819	PHLDB1	0.544619949
ILMN_1903914	AK129699	0.544588846
ILMN_1689004	TNFRSF12A	0.544524564
ILMN_1766408	CBFB	0.544134546
ILMN_1786024	POLR3H	0.54395202
ILMN_1665985	SPDYE7P	0.543883902
ILMN_1676241	BCOR	0.543717463
ILMN_1679268	PELI1	0.543698603
ILMN_2350266	FBXO46	0.543693315
ILMN_2155719	NBPF1	0.543593059
ILMN_2114720	SLPI	0.5435842
ILMN_1672661	SP110	0.543398714
ILMN_1802100	ADAM28	0.543392053
ILMN_2380237	C1QTNF1	0.543376559
ILMN_1678290	HMG20A	0.54329152
ILMN_2319910	DGKA	0.543127195
ILMN_1799062	NFKB2	0.543124885
ILMN_1753805	PRKD2	0.543106627
ILMN_1765021	TOP3B	0.542844661
ILMN_1677765	LRP8	0.542422512
ILMN_1769702	GPAA1	0.542256818
ILMN_1767349	ABCB4	0.542255234
ILMN_2403555	DHX30	0.541823749
ILMN_1683781	ADAMTS15	0.54176659
ILMN_3236381	MXRA8	0.541723628
ILMN_2358914	SLC35C2	0.541684841
ILMN_1769031	MTOR	0.541447789
ILMN_1782741	CD300LB	0.541439873
ILMN_1719237	SPDYE8P	0.541089119
ILMN_1790859	PLAC9	0.541046739
ILMN_1758823	SMARCB1	0.540769103
ILMN_1706505	COL5A1	0.540648831
ILMN_2112638	SVEP1	0.54027013

ILMN_3236052	SNORD19B	-0.432305533
ILMN_1724040	ANKRD57	-0.432323418
ILMN_1724266	LYPD2	-0.432366109
ILMN_1665821	DHFR	-0.432505727
ILMN_1713964	BTBD3	-0.432601182
ILMN_3309524	MIR122	-0.432684615
ILMN_1766966	ACSM5	-0.43271135
ILMN_1669572	RNF126	-0.432755224
ILMN_1681249	KIF21A	-0.4328047
ILMN_1689070	COQ7	-0.432972141
ILMN_2189371	NTNG1	-0.433017117
ILMN_2385410	GPX5	-0.433064277
ILMN_1754929	OR6N1	-0.433155779
ILMN_1911171	DB337954	-0.433319493
ILMN_1695731	TUBG1	-0.433353558
ILMN_1825067	AW665831	-0.433422119
ILMN_2155452	HPR	-0.433490506
ILMN_1888264	DB302602	-0.433503941
ILMN_1718971	TTC7B	-0.433622644
ILMN_1712523	MAP6	-0.433789726
ILMN_1655509	ASAH1	-0.433795221
ILMN_1705458	OR2W3	-0.433802511
ILMN_1740576	BC169297	-0.433868348
ILMN_1673716	KIF6	-0.433914223
ILMN_3249654	UOX	-0.433969337
ILMN_1670725	SYT16	-0.433978467
ILMN_1795839	SCCPDH	-0.434002431
ILMN_1682988	RNF34	-0.434042364
ILMN_2075962	DSCAML1	-0.434052981
ILMN_3235171	CCDC64B	-0.434093153
ILMN_1800179	KCNJ4	-0.434094402
ILMN_2306565	MTX2	-0.434099124
ILMN_2054938	RBAK	-0.434221343
ILMN_1776577	DSCC1	-0.43431878
ILMN_1656940	ABLIM3	-0.434432128
ILMN_1895763	BC030212	-0.434497221
ILMN_2067408	CLRN3	-0.434514999
ILMN_1684885	KRTAP4-8	-0.434639599
ILMN_1748366	SLC5A9	-0.434650696
ILMN_3237956	ZC3H12C	-0.434693863
ILMN_1766028	TBC1D26	-0.434792267
ILMN_1908074	AV750156	-0.434819831
ILMN_1665887	WDR61	-0.434890729
ILMN_1675018	IQCF4	-0.434959631
ILMN_1669034	CCL15	-0.434991748
ILMN_1670255	MAGEA9B	-0.435002146
ILMN_1680353	NSF	-0.435053281
ILMN_2393461	DDO	-0.435145971
ILMN_1765401	RRN3	-0.435297486
ILMN_1671427	FBXW4	-0.435330758
ILMN_1663646	DMXL1	-0.435339313
ILMN_2293594	LAMA3	-0.435355689
ILMN_2076567	UBE2V2	-0.435397841
ILMN_3252185	BC037739	-0.435398496
ILMN_1668932	CCT8L2	-0.435475039
ILMN_3191988	NA	-0.435476991
ILMN_1675346	CCRL2	-0.435518175
ILMN_3243129	SNHG7	-0.435518254
ILMN_2219556	ISCA1	-0.435650919
ILMN_1668490	UNCX	-0.435683278
ILMN_1847602	HAR1A	-0.435803635
ILMN_1802223	ATP2B2	-0.435811298
ILMN_1808938	PIGF	-0.436006095
ILMN_1742586	TMPRSS11F	-0.436052742
ILMN_1752668	DAAM2	-0.436105858
ILMN_2392674	PRR3	-0.436142688
ILMN_1708611	RDX	-0.436376696
ILMN_1812073	ATP6V1B1	-0.436449442
ILMN_2269092	MANEA	-0.436517619
ILMN_1729816	VDAC3	-0.436535347
ILMN_1689098	ATP1A3	-0.436619687
ILMN_1712137	LOC100128219	-0.436697467
ILMN_1741483	HPS1	-0.4367126
ILMN_1753286	MYO19	-0.437102377
ILMN_1699562	EFCAB1	-0.437133167
ILMN_1727091	ACMSD	-0.437135898
ILMN_3309489	MIR563	-0.437139838
ILMN_1681651	FOLH1	-0.437158108
ILMN_1696751	AI636007	-0.4371919
ILMN_1779897	NPR1	-0.437212103
ILMN_1738987	HS2ST1	-0.437240421
ILMN_1762327	DHRS12	-0.437257816
ILMN_3238259	SNORA30	-0.437264859
ILMN_1827050	BC031235	-0.437295522
ILMN_1726409	KRT23	-0.437300792
ILMN_1792182	RDH12	-0.437378101
ILMN_1796583	CLRN2	-0.437395407
ILMN_1686083	MAGEA11	-0.437550747
ILMN_1702739	TMEM146	-0.43760553
ILMN_1701454	OR2L13	-0.43769345
ILMN_3240108	SNORD90	-0.437823343
ILMN_1707426	CPLX4	-0.437919518
ILMN_3288525	NA	-0.437932808
ILMN_3237022	SCGB1C1	-0.437951591
ILMN_1675671	ICOSLG	-0.437958388
ILMN_1770361	BC040551	-0.438135509
ILMN_1669846	SLC25A31	-0.438136162
ILMN_1706301	RET	-0.438162644

ILMN_3270833	NA	0.539841587
ILMN_1789186	OBFC1	0.53969901
ILMN_1814998	POTEKP	0.539678705
ILMN_2110908	MYC	0.539609467
ILMN_1804117	FAM89B	0.539544192
ILMN_2178226	KRT86	0.539541131
ILMN_1703305	TWF2	0.539526755
ILMN_1786843	KCTD13	0.539383377
ILMN_1704294	CDH3	0.539228445
ILMN_1698803	ZAK	0.539077767
ILMN_1809484	TMOD3	0.53900706
ILMN_3289171	RPS27	0.53856999
ILMN_1749006	RCSD1	0.538425879
ILMN_1705750	TGM2	0.538392247
ILMN_2353202	PTK7	0.538364395
ILMN_1694778	KRT18	0.538254575
ILMN_2355738	INCENP	0.538148445
ILMN_1682332	GYPC	0.537897769
ILMN_1656732	LOC440300	0.537732381
ILMN_1758324	SLCO5A1	0.537693217
ILMN_1795025	NFX1	0.537691725
ILMN_1755173	PLEKHA4	0.53761862
ILMN_1691892	TAGLN2	0.537611163
ILMN_2203950	HLA-A	0.537421579
ILMN_2146566	CLASRP	0.537314354
ILMN_1711272	SERPING1	0.537180051
ILMN_1760089	RPS2	0.537150013
ILMN_2282641	TBXAS1	0.537032197
ILMN_1778144	SLC48A1	0.536886462
ILMN_1786697	TRIM9	0.536865233
ILMN_3309995	MIR383	0.536789307
ILMN_2342579	IL7R	0.536487734
ILMN_2403852	CYTH1	0.536431858
ILMN_2406716	CCDC41	0.536306974
ILMN_1775692	EIF4G3	0.53628433
ILMN_1741371	TMEM8A	0.536266053
ILMN_3183573	NA	0.536198637
ILMN_2349071	GPR64	0.536091202
ILMN_3251361	NBPF1	0.536053446
ILMN_2347693	NCOA3	0.536025658
ILMN_1803810	RRBP1	0.535994997
ILMN_1659688	LGALS3BP	0.535808574
ILMN_1764770	OXNAD1	0.535799278
ILMN_1793241	SRD5A1	0.535737137
ILMN_2367469	CARS	0.535713395
ILMN_2353862	LRRC48	0.535610478
ILMN_1798679	ANO9	0.535524838
ILMN_1741585	SFMBT1	0.535242418
ILMN_1689111	CXCL12	0.535187637
ILMN_1782488	RNASEH2B	0.535006608
ILMN_2169801	TPSAB1	0.534989045
ILMN_1790533	PHACTR2	0.534988834
ILMN_2371053	EFNA1	0.534823103
ILMN_2225135	GCNT1	0.534711059
ILMN_3209220	NA	0.534490962
ILMN_1792012	OTUB2	0.534427731
ILMN_1716276	CCL4	0.534356305
ILMN_1740633	PRF1	0.534334227
ILMN_2105441	IGJ	0.534199307
ILMN_1702691	TNFAIP3	0.534136986
ILMN_1726289	C12orf35	0.534024145
ILMN_1702018	MLL3	0.534004102
ILMN_1759700	NLGN3	0.533918795
ILMN_1730842	PTPRC	0.533838566
ILMN_1674250	NCKAP1L	0.533623878
ILMN_2390299	PSMB8	0.533598609
ILMN_1808707	FSCN1	0.533359104
ILMN_1662026	BTK	0.533337906
ILMN_3307930	RAN	0.533326757
ILMN_2382657	ARHGAP9	0.53319793
ILMN_2205050	PRKX	0.532711768
ILMN_2257015	AGER	0.532659845
ILMN_2327346	SSBP4	0.532605223
ILMN_2352190	CLIP2	0.532601197
ILMN_1778625	CD44	0.532596113
ILMN_3237966	KIAA1004	0.532593702
ILMN_1693762	LRRC48	0.532585856
ILMN_1680772	TCRGV	0.532563872
ILMN_1774836	PLOD3	0.532518204
ILMN_1810037	RUSC2	0.532393327
ILMN_2367384	EPHB2	0.532381994
ILMN_1719972	PLXNA3	0.5322351
ILMN_1771599	PLOD2	0.532222667
ILMN_1710216	AVEN	0.531921417
ILMN_2136133	PABPC1	0.531919204
ILMN_1682778	MXRA8	0.53177551
ILMN_1778668	TAGLN	0.531699296
ILMN_1754279	FBXW7	0.531558561
ILMN_1762046	ZNF551	0.531549438
ILMN_1659103	PSMA8	0.531461181
ILMN_2328433	NOP2	0.531219462
ILMN_1733221	SEMA4D	0.531160484
ILMN_2375992	SPINT1	0.530748077
ILMN_1653871	NAMPT	0.530615214
ILMN_1655137	ZCCHC11	0.53061132
ILMN_3251733	C19orf43	0.530611079
ILMN_1721241	CYTH2	0.530565094
ILMN_1668039	GYPC	0.530287297

ILMN_2375386	RNPS1	-0.438282898
ILMN_1866389	BQ182905	-0.438296536
ILMN_1754570	KCTD8	-0.438412482
ILMN_1888359	BMPR1B	-0.438502315
ILMN_2395932	UNC45A	-0.438519487
ILMN_1910307	BM987531	-0.438541697
ILMN_1900604	BX108309	-0.438569901
ILMN_3179396	MUC7	-0.438626072
ILMN_2098743	ACOT13	-0.438635743
ILMN_3287646	EFCAB8	-0.438658344
ILMN_1730611	RTN4	-0.438756614
ILMN_1664371	HIATL1	-0.438785502
ILMN_1903159	PPM1A	-0.438995313
ILMN_3251648	AQP7P1	-0.439041213
ILMN_1736888	SAR1B	-0.43905864
ILMN_1651752	CXorf21	-0.439076647
ILMN_1771841	FOSL1	-0.439124369
ILMN_1801608	KCNN3	-0.439216439
ILMN_2170595	RRM2B	-0.439300346
ILMN_1772728	FAM75A5	-0.439329654
ILMN_3205404	DQ059386	-0.439355251
ILMN_2206420	UGT1A1	-0.439368527
ILMN_2406634	DNMT3L	-0.439381504
ILMN_3236866	ULK4	-0.439445893
ILMN_1798853	CTSL1P2	-0.439475566
ILMN_1707342	LRIG1	-0.439516518
ILMN_1722850	VGLL2	-0.439633621
ILMN_1746552	WDR17	-0.439669298
ILMN_1671547	HMGA2	-0.439674587
ILMN_1695965	FAM36A	-0.439727135
ILMN_1808979	CLEC4D	-0.439844365
ILMN_1687265	NXF5	-0.439854203
ILMN_1772814	NLK	-0.439874279
ILMN_1668736	AX748125	-0.439903351
ILMN_1727605	FRK	-0.439977517
ILMN_1736969	SLITRK3	-0.439984436
ILMN_1716517	KIAA0467	-0.439985538
ILMN_1749403	TSPAN33	-0.439991803
ILMN_1654171	LOC1720	-0.439996783
ILMN_1755354	YBX2	-0.440050082
ILMN_1661458	ARF1	-0.440063555
ILMN_1683302	SMC4	-0.44015941
ILMN_1915480	BX107598	-0.440227206
ILMN_1852179	BX118817	-0.440236396
ILMN_1692831	HIST1H1A	-0.440251411
ILMN_1707368	OPRD1	-0.440267259
ILMN_1746819	C5	-0.440288879
ILMN_1736453	RCVRN	-0.440331465
ILMN_2147503	ALG13	-0.44035349
ILMN_2152028	AK027082	-0.440366812
ILMN_2407308	HEY1	-0.440652676
ILMN_2258977	XAGE1A	-0.440693012
ILMN_1776685	PCDH15	-0.440703926
ILMN_3237520	C6orf127	-0.440759862
ILMN_3272101	LOC100130429	-0.440843584
ILMN_1771800	PRKCA	-0.440849673
ILMN_1846499	AI376590	-0.440869235
ILMN_1662453	PRDM6	-0.440934371
ILMN_1726603	ATP5I	-0.440940781
ILMN_3235231	SNORA49	-0.441020894
ILMN_3182898	LL0XNC01-19D8.1	-0.441042855
ILMN_2407346	LDHD	-0.441096311
ILMN_1812678	AGMO	-0.441118254
ILMN_1750062	PPARGC1A	-0.441154263
ILMN_1672591	SCN9A	-0.441281912
ILMN_1756852	AUP1	-0.441346639
ILMN_3201163	NA	-0.441360934
ILMN_1809245	PITPNB	-0.441445006
ILMN_3280269	FLJ00317	-0.441637463
ILMN_1810785	RNF11	-0.441666978
ILMN_2231911	AUH	-0.44179334
ILMN_2245087	MFAP2	-0.441808023
ILMN_1741971	KIAA1161	-0.441890595
ILMN_1767070	VN1R5	-0.441898736
ILMN_1734762	OPA3	-0.442022966
ILMN_1709815	PURG	-0.442052542
ILMN_2358278	RSRC2	-0.442073627
ILMN_1840316	BC064139	-0.44213748
ILMN_1680251	SORCS1	-0.44217703
ILMN_1670172	WDR33	-0.442178968
ILMN_1767274	FBLL1	-0.44219475
ILMN_1769684	LCE1E	-0.442247469
ILMN_1666363	FOXN1	-0.442275576
ILMN_2205162	ZNF699	-0.442277901
ILMN_1763558	RGN	-0.442301057
ILMN_1786273	C1orf122	-0.442301786
ILMN_1810684	TINAG	-0.442311751
ILMN_2376263	SMARCA1	-0.442318148
ILMN_1780551	ADRA1A	-0.442396256
ILMN_1866337	AK093987	-0.442566597
ILMN_1726437	EFCAB3	-0.442583547
ILMN_1748251	ABHD2	-0.44279715
ILMN_1748543	STRC	-0.442820405
ILMN_1738246	OR10A7	-0.442825442
ILMN_2211780	SLC25A4	-0.442884928
ILMN_1798485	ATP6V1E1	-0.443147261
ILMN_1732410	SLC16A9	-0.443226705
ILMN_2189314	HDGFL1	-0.443342522

ILMN_1753064	TTC13	0.530199899
ILMN_1708077	DAXX	0.530180592
ILMN_2355953	LILRB4	0.530129682
ILMN_2328972	DNMT3B	0.529988945
ILMN_1738424	CDC42	0.529974729
ILMN_1663866	TGFBI	0.529822779
ILMN_2312906	MIA3	0.529781779
ILMN_2349129	DPP3	0.529641755
ILMN_1715024	LSS	0.529616126
ILMN_2313158	MBNL1	0.529442899
ILMN_1766955	VCAM1	0.529413749
ILMN_1701441	LPAR1	0.529321409
ILMN_1733511	GOLGA3	0.529273901
ILMN_1781916	AV723037	0.528964564
ILMN_2108709	ANKRD11	0.528895006
ILMN_2351466	NTM	0.528732544
ILMN_1714499	TLK2	0.528386891
ILMN_1795454	CPSF6	0.528382182
ILMN_1724533	LY96	0.528332172
ILMN_2352380	TRPM2	0.52813751
ILMN_2049021	PTTG3	0.527670943
ILMN_2169439	ITGAV	0.527618679
ILMN_1804444	PDIA2	0.52736693
ILMN_1696839	RPS27	0.527276796
ILMN_3303101	AK027616	0.527025418
ILMN_2396444	CD14	0.52699294
ILMN_1701308	COL1A1	0.526989929
ILMN_3264073	RPS27	0.5269222
ILMN_3225505	RPS8	0.526736277
ILMN_1806956	ATAD5	0.52669634
ILMN_1670134	FADS1	0.52667533
ILMN_3288161	RPS27	0.526481928
ILMN_1695354	BMF	0.526434494
ILMN_1698067	NBPF1	0.526426542
ILMN_1860540	AA709087	0.526397893
ILMN_1684647	ILKAP	0.526387807
ILMN_1735014	KLF6	0.52634619
ILMN_1761155	PABPC1	0.52613115
ILMN_1740385	CEP164	0.526024435
ILMN_1772113	U2AF1	0.52594375
ILMN_1737964	HIATL1	0.525853067
ILMN_1672547	MYO9B	0.525791659
ILMN_1743646	VASP	0.525591436
ILMN_2070072	RPS7	0.525204784
ILMN_1680102	C11orf45	0.52495396
ILMN_1683415	CAMK2D	0.524934928
ILMN_1766222	LARP4B	0.524819385
ILMN_3247802	PRRC2B	0.524571078
ILMN_1800425	SLC9A1	0.524483562
ILMN_1784364	STARD5	0.524348515
ILMN_1685005	TNFRSF1A	0.52423743
ILMN_2400352	LRDD	0.524223833
ILMN_1741003	ANXA5	0.524103929
ILMN_1756071	MFGE8	0.524002305
ILMN_1756360	RPL35A	0.523218248
ILMN_1732967	KIAA1949	0.523135629
ILMN_1777344	RPS13	0.523025119
ILMN_1663577	S100PBP	0.523014136
ILMN_1686116	THBS1	0.522963866
ILMN_1723978	LGALS1	0.522886886
ILMN_1724293	KDELR2	0.52274885
ILMN_1814799	S100A13	0.522540721
ILMN_2380850	SDCCAG3	0.522439665
ILMN_1654099	LRIG2	0.522323776
ILMN_1806017	PSME1	0.522186205
ILMN_1674640	CXCR6	0.521975711
ILMN_1780663	KLHL30	0.521910788
ILMN_1681591	PTPN1	0.521761328
ILMN_1700628	DDX24	0.521760279
ILMN_2354334	ATXN2L	0.521663211
ILMN_1789418	GSTT2B	0.521561723
ILMN_1659158	SAMD3	0.521391493
ILMN_1842582	TTC39C	0.521213291
ILMN_1719695	NFKBIZ	0.521158695
ILMN_1763842	PTRH1	0.521018699
ILMN_1658677	DTX3	0.520952299
ILMN_1670841	CPNE1	0.520728643
ILMN_2381037	LIMS1	0.520686218
ILMN_1801934	SERTAD3	0.520666965
ILMN_1767894	POLB	0.520512762
ILMN_2219712	HMGB2	0.520494794
ILMN_2170814	LAMP3	0.520478717
ILMN_3246005	KDM5B	0.520373166
ILMN_1651788	MAP3K11	0.520184928
ILMN_1693598	RTN4	0.519994896
ILMN_1809928	COL6A2	0.519791821
ILMN_1773276	WHAMML2	0.519716383
ILMN_1675162	AK097193	0.519621796
ILMN_1745994	GAS7	0.519429444
ILMN_2338348	UBASH3A	0.519366627
ILMN_1708375	IRF1	0.519317006
ILMN_2106331	H3F3A	0.519306227
ILMN_1805466	SOX9	0.519264633
ILMN_1761797	CSTB	0.519196864
ILMN_1693218	ART3	0.519157669
ILMN_1790918	ZNF236	0.519085678
ILMN_1808501	SH3KBP1	0.518804449
ILMN_2379734	CTBP1	0.518786728

ILMN_1907218	BM973226	-0.443394293
ILMN_1906678	BX117604	-0.443541169
ILMN_1900929	OR7E12P	-0.443564548
ILMN_3297082	LOC729080	-0.443703364
ILMN_1814526	ADD3	-0.44375417
ILMN_1657679	VAV3	-0.443811158
ILMN_1763187	PAK4	-0.443855987
ILMN_3266764	LOC728448	-0.443869614
ILMN_1737402	HEATR2	-0.443930474
ILMN_1764861	ISOC1	-0.443935489
ILMN_1715742	SLC22A1	-0.443973213
ILMN_1797201	KRTAP10-2	-0.443980551
ILMN_1786433	BCCIP	-0.443990109
ILMN_2371147	BCL7C	-0.444015661
ILMN_1701058	TATDN1	-0.44401947
ILMN_3310548	MIR889	-0.444077896
ILMN_1695355	FOXP2	-0.444178607
ILMN_3251283	HDAC2	-0.444518742
ILMN_3239912	HSFYP1	-0.444529869
ILMN_1690979	SLC17A3	-0.444533323
ILMN_2176450	CA3	-0.444571305
ILMN_1779032	SDHC	-0.444613106
ILMN_2171783	CPEB3	-0.444653532
ILMN_1657855	ACTL6B	-0.444688295
ILMN_2238795	NEK3	-0.444720442
ILMN_2094294	NR2E1	-0.444729092
ILMN_2054053	TMEM67	-0.444767356
ILMN_1775441	ARHGAP24	-0.444776493
ILMN_1896277	BC037739	-0.444934824
ILMN_2279645	BCL2L14	-0.444961639
ILMN_2093674	GMFB	-0.444974128
ILMN_2137208	GOLT1A	-0.445004196
ILMN_1680580	LOC148709	-0.445437287
ILMN_3311075	MIR1915	-0.445444389
ILMN_1728746	GAB2	-0.445483396
ILMN_1725352	RUVBL1	-0.445526174
ILMN_2340643	INSC	-0.445593881
ILMN_1704206	NPSR1	-0.445618686
ILMN_1852756	BG201089	-0.445712894
ILMN_1802246	KLK10	-0.445737972
ILMN_1678546	PEX11B	-0.445915264
ILMN_1656638	BHMT	-0.445972055
ILMN_3237085	NTF4	-0.446100033
ILMN_1663118	RASGEF1C	-0.446122005
ILMN_1703459	WNT9B	-0.446171697
ILMN_1874302	BE646122	-0.446319413
ILMN_1796075	VPS41	-0.44634519
ILMN_1756623	NT5C1B	-0.446348625
ILMN_1904301	MECP2	-0.446457844
ILMN_2336595	ACSS2	-0.446554572
ILMN_1689473	ATP6V1H	-0.446591268
ILMN_1765232	RNLS	-0.446756943
ILMN_3237779	TMEM184C	-0.446843877
ILMN_1787657	CDK14	-0.446966445
ILMN_1769104	ZCCHC10	-0.446980846
ILMN_3244060	LOC285205	-0.447026723
ILMN_1786388	RNF113A	-0.447083526
ILMN_1720531	KDM4B	-0.447351029
ILMN_1680673	NT5DC1	-0.447372204
ILMN_1751897	PM20D1	-0.447452213
ILMN_1718135	PRAMEF11	-0.447618763
ILMN_1805823	BANF2	-0.44773542
ILMN_1768913	TSN	-0.447804237
ILMN_1658017	SPN	-0.447928196
ILMN_2357062	IL1RAP	-0.447959276
ILMN_2209180	RPRD1A	-0.448000042
ILMN_1788625	EXOC5	-0.448196038
ILMN_1685978	ATPIF1	-0.448318509
ILMN_1710652	MTMR8	-0.448383171
ILMN_1784578	LRRC37A3	-0.448410958
ILMN_1914284	AI191966	-0.448468775
ILMN_1751814	DDX11L2	-0.448568101
ILMN_1836936	BM684558	-0.448568777
ILMN_1782705	GAGE12B	-0.448590728
ILMN_1728785	GPR116	-0.448706382
ILMN_1751941	TMEM106A	-0.448708562
ILMN_1795778	P4HA2	-0.448948229
ILMN_1652357	PDHX	-0.449005874
ILMN_1733781	FSD1	-0.449106425
ILMN_2204297	DKFZp564F1378	-0.449361865
ILMN_1692056	HS3ST3A1	-0.449364863
ILMN_2371001	ATP6V1G3	-0.44936908
ILMN_1771019	MTMR4	-0.449408516
ILMN_1764850	HPCAL1	-0.449493079
ILMN_1681994	AJ005282	-0.449537453
ILMN_3248857	IL20	-0.449555191
ILMN_1694491	CCNG1	-0.449565379
ILMN_1680727	GLRX2	-0.449586196
ILMN_1727205	ARHGEF10L	-0.449590924
ILMN_1727178	C11orf92	-0.449617112
ILMN_1800786	PARG	-0.449662649
ILMN_1789702	GBE1	-0.44987261
ILMN_1736546	SLC16A14	-0.449917903
ILMN_3182102	MUC12	-0.449970799
ILMN_1661500	B4GALT4	-0.450119101
ILMN_1697175	TAF8	-0.450158576
ILMN_3239173	WAPAL	-0.450258968
ILMN_1745343	ZMAT2	-0.450268456

ILMN_1738578	FILIP1L	0.518760155
ILMN_2111739	MAN2C1	0.51875767
ILMN_1660817	DDB2	0.518740783
ILMN_1769229	BCL2A1	0.518710685
ILMN_1737406	KLF6	0.518698119
ILMN_1769671	RYK	0.518616143
ILMN_1674034	H2AFY	0.518317831
ILMN_1740015	CD14	0.518274429
ILMN_2410924	PLOD2	0.518175313
ILMN_1676721	NF2	0.518168116
ILMN_1679185	LEF1	0.518157518
ILMN_1786789	FAM102B	0.517792932
ILMN_1784141	JAKMIP1	0.517703273
ILMN_1712435	NA	0.517621871
ILMN_1808011	FAM46B	0.51757909
ILMN_1757343	PABPC4	0.517577227
ILMN_1784717	RPS19	0.517476592
ILMN_3284584	RPL10	0.517395185
ILMN_3304898	TUBB	0.516868944
ILMN_1724341	ALG13	0.516724765
ILMN_1655961	NAG8	0.516696065
ILMN_1677997	MAZ	0.516605608
ILMN_2062468	IGFBP7	0.51650137
ILMN_2152402	ABHD16A	0.516337465
ILMN_2349124	OSBPL2	0.516277028
ILMN_1765860	DOCK11	0.516208369
ILMN_1704385	H3F3A	0.516127825
ILMN_1681269	DYRK4	0.516104288
ILMN_1687358	TMPRSS4	0.516026528
ILMN_1657983	TERF2IP	0.515927854
ILMN_1657836	PLEKHG2	0.515819988
ILMN_1651767	MKL1	0.515742035
ILMN_2409167	ANXA2	0.515672881
ILMN_1664541	POLR2J4	0.515233206
ILMN_1769606	PIWIL4	0.515200905
ILMN_1728009	TMEM171	0.51504154
ILMN_1660900	SNORA7B	0.514979315
ILMN_1789913	SMPDL3B	0.514881157
ILMN_1656920	CRIP1	0.514712757
ILMN_3247732	ZRSR2	0.514650955
ILMN_1652366	NLRP7	0.514551676
ILMN_1724984	EIF2AK3	0.514412048
ILMN_1703263	SP140	0.514383781
ILMN_1676213	SRPX2	0.514343479
ILMN_2165753	HLA-A	0.514256
ILMN_1754660	ZCCHC24	0.514255181
ILMN_1658586	GPR161	0.514174339
ILMN_2392940	CEP250	0.514155199
ILMN_1765146	IFNAR2	0.514054974
ILMN_1708959	MFN1	0.514028073
ILMN_3247163	TET1	0.513849711
ILMN_2366634	PKM2	0.513848767
ILMN_2311089	BRCA1	0.513823714
ILMN_1666269	CTSZ	0.513740302
ILMN_1806782	ZNF235	0.513675341
ILMN_1751330	RBCK1	0.513668822
ILMN_1652008	C15orf23	0.51363966
ILMN_1704079	RBM38	0.513555688
ILMN_1745573	TTC13	0.513515524
ILMN_1763688	RNASEK-C17OR	0.513392444
ILMN_1758250	TRAFD1	0.513384721
ILMN_1704362	ZNF174	0.513068504
ILMN_1752340	ARF5	0.512944554
ILMN_1700081	FST	0.512897711
ILMN_1794333	POU2F1	0.512534655
ILMN_1695856	BOD1L	0.512429562
ILMN_2209115	MAK	0.512162163
ILMN_2319414	BTF3	0.511954208
ILMN_1728106	TNF	0.511868545
ILMN_2374362	FAM108B1	0.511728399
ILMN_3307693	WFDC2	0.511713223
ILMN_3238018	NA	0.511644855
ILMN_1655577	TIAM1	0.511622751
ILMN_1673804	ZNF426	0.511611349
ILMN_2413622	RFX4	0.51139969
ILMN_1737074	RPS2	0.511350395
ILMN_2337241	RPS15A	0.5112651
ILMN_1731418	SP110	0.511264518
ILMN_1681603	UBE2A	0.511111085
ILMN_1795918	AGAP3	0.510834826
ILMN_1732885	SLX4	0.510816739
ILMN_1699085	AJ312026	0.510779541
ILMN_3244117	STMN3	0.510587928
ILMN_1680145	SART1	0.510568902
ILMN_2115005	FGD2	0.510544691
ILMN_1654246	SIRT6	0.510460056
ILMN_2098616	C5orf39	0.510381148
ILMN_1731991	SCYL1	0.510317348
ILMN_1742427	POLR1D	0.510115687
ILMN_1747023	PTGIR	0.510035336
ILMN_2325168	ARRB1	0.509927371
ILMN_1713676	FLJ40330	0.509899889
ILMN_2099528	BTLA	0.509893222
ILMN_1726114	SLC45A3	0.509790687
ILMN_2137084	LIN9	0.509715322
ILMN_1811682	CLIP2	0.509381497
ILMN_2410783	GAA	0.509319512
ILMN_1749662	GPX1	0.509202341

ILMN_1762714	VGLL2	-0.450275786
ILMN_2390416	BAG6	-0.450278324
ILMN_1706077	DEFB125	-0.450307585
ILMN_1782501	XAGE1D	-0.450464982
ILMN_3238832	RBMY3AP	-0.450476814
ILMN_3309914	MIR660	-0.450490541
ILMN_1781073	PTPN5	-0.450615652
ILMN_2133038	UGT3A1	-0.450644867
ILMN_1761820	EDARADD	-0.450722396
ILMN_1810441	TRIM3	-0.450781409
ILMN_1743427	SCYL3	-0.450834314
ILMN_3185020	AI926420	-0.45087834
ILMN_2414786	DIO1	-0.450907165
ILMN_1724699	ACAD8	-0.450986868
ILMN_2302983	TRPM3	-0.45102007
ILMN_2223720	ATMIN	-0.451045932
ILMN_1665647	CD180	-0.451055426
ILMN_1660337	PPT2-EGFL8	-0.451069003
ILMN_1790645	HECW1	-0.45114006
ILMN_1756878	SLC39A9	-0.451146114
ILMN_1676625	SS18L1	-0.451175582
ILMN_1730441	OR52K2	-0.451191644
ILMN_1755281	FBXO15	-0.45121228
ILMN_1726421	METTL9	-0.451216921
ILMN_1666967	BRP44L	-0.451232642
ILMN_1709796	AGXT	-0.451285244
ILMN_1659947	FGA	-0.451342525
ILMN_1783889	PRKAA1	-0.451586698
ILMN_1763730	APPL1	-0.451595484
ILMN_1747678	LOC647946	-0.451756252
ILMN_1709937	KCNN4	-0.45178978
ILMN_2141118	C15orf59	-0.45179845
ILMN_1683390	FLCN	-0.451839447
ILMN_2310814	MAPT	-0.451841069
ILMN_1661363	AF064105	-0.451870558
ILMN_1781563	DPPA3	-0.451924636
ILMN_1678186	ATP6V0A4	-0.451965291
ILMN_1884357	AI734261	-0.452061859
ILMN_1794074	MXI1	-0.452095339
ILMN_1773333	TMCO2	-0.452414524
ILMN_1693674	TRDN	-0.452448607
ILMN_1861916	CR992331	-0.452498733
ILMN_1823732	NA	-0.452509173
ILMN_1699648	ZNF516	-0.45255533
ILMN_1828225	AW118209	-0.452576923
ILMN_1697301	ST8SIA3	-0.452643399
ILMN_2388397	LYPD1	-0.452648363
ILMN_1769083	GSTA3	-0.452771898
ILMN_1876912	U79300	-0.452816624
ILMN_1775831	LOC100287166	-0.452856732
ILMN_2205350	NDUFAF4	-0.452858684
ILMN_2365595	GMPR2	-0.4528833
ILMN_1655296	UTRN	-0.453001686
ILMN_3292114	LOC100131510	-0.453006416
ILMN_2153061	FGF20	-0.453054674
ILMN_1803033	MGST1	-0.453056147
ILMN_1774806	SLC9A7	-0.453073082
ILMN_1873592	NA	-0.453100197
ILMN_1709256	PLEC	-0.453152239
ILMN_1751061	C16orf54	-0.453192658
ILMN_1793655	RGPD6	-0.453198947
ILMN_1657302	SULT1A1	-0.45324094
ILMN_1652722	C14orf2	-0.453365526
ILMN_1825421	BC033844	-0.453429456
ILMN_1659885	ACSS3	-0.453457175
ILMN_1761721	VPS35	-0.453534029
ILMN_1891885	AI219815	-0.453611785
ILMN_1808238	RBPMS2	-0.453669718
ILMN_1758189	OR4F17	-0.45377389
ILMN_1694923	PTPN9	-0.453790955
ILMN_1758164	STC1	-0.453852468
ILMN_1662331	PDSS2	-0.454090612
ILMN_3237035	SNORA54	-0.45415526
ILMN_1856662	BM680619	-0.454157761
ILMN_1696702	NEO1	-0.454204043
ILMN_1719581	MYOM3	-0.454217726
ILMN_1732808	TOX3	-0.454246536
ILMN_1692588	HEATR5A	-0.454362455
ILMN_1683146	FTH1	-0.454420105
ILMN_1668270	ZDHHC18	-0.454478684
ILMN_1791925	NFAM1	-0.454497971
ILMN_2115862	ESPNL	-0.45469075
ILMN_1685260	DNM1L	-0.454767603
ILMN_2101410	ZNF20	-0.454807607
ILMN_1749466	VAT1L	-0.454924149
ILMN_1681374	ALDOA	-0.455033285
ILMN_1691572	TST	-0.455044592
ILMN_2085659	OTOGL	-0.455131814
ILMN_1698673	EFCAB7	-0.45521512
ILMN_1902060	CD000292	-0.455219022
ILMN_1730572	HNRPDL	-0.455245339
ILMN_2246510	TSC1	-0.455268854
ILMN_1833027	AI936635	-0.455286475
ILMN_1696692	BAZ1B	-0.455565922
ILMN_1733298	C1orf158	-0.45559248
ILMN_1833415	BC021736	-0.455717338
ILMN_1660261	TDRD6	-0.455822431
ILMN_1692535	DPP4	-0.455824107

ILMN_1802456	DCTD	0.509131786
ILMN_1787844	C19orf10	0.509128331
ILMN_1677906	PTMA	0.509004327
ILMN_1808202	C19orf22	0.508999086
ILMN_1715715	CEBPA	0.508837293
ILMN_1742929	HESX1	0.508824051
ILMN_1718303	PVRL2	0.50877734
ILMN_1713384	TSHZ2	0.508727764
ILMN_2229019	SEBOX	0.508649377
ILMN_2339266	LAMA2	0.508494999
ILMN_2318568	HCFC1R1	0.508338613
ILMN_1669286	YWHAZ	0.508259404
ILMN_1768940	COL15A1	0.508141221
ILMN_1653601	TLK2	0.50813116
ILMN_1799134	KLRD1	0.50810044
ILMN_3248975	PPP4C	0.507814645
ILMN_1685810	NFATC3	0.507631583
ILMN_1794987	C17orf100	0.507598878
ILMN_1668748	MED15	0.507582006
ILMN_1735548	HIVEP1	0.507572491
ILMN_2145670	TNC	0.507480053
ILMN_1747195	PSMB8	0.507441401
ILMN_1717737	FKBP7	0.507392499
ILMN_2398711	SIRT2	0.507386135
ILMN_1705522	SPAG1	0.507218642
ILMN_1722781	EGR3	0.507193294
ILMN_2133360	RPS2	0.507070177
ILMN_1829845	NA	0.50703923
ILMN_1666049	NUP214	0.506981642
ILMN_1744487	C1QTNF5	0.506955012
ILMN_1699206	FHDC1	0.50694868
ILMN_1743130	PTGFRN	0.506847987
ILMN_2246882	SP140	0.506827386
ILMN_1715169	HLA-DRB1	0.506819474
ILMN_2206098	ATG10	0.506759035
ILMN_1768505	IL13RA1	0.506755885
ILMN_1691843	RNPS1	0.506684344
ILMN_2148819	TUBA1A	0.506667743
ILMN_2287296	SYF2	0.506447845
ILMN_1737205	MCM4	0.506270707
ILMN_3282436	EIF3F	0.506255021
ILMN_1677466	DUSP6	0.506237651
ILMN_1761159	ESYT1	0.506101833
ILMN_1764980	SERPINA1	0.506053923
ILMN_3249667	HLA-DQA1	0.505830231
ILMN_1660832	RPL18A	0.505686184
ILMN_1668417	WIPF1	0.50558921
ILMN_1745420	PHF19	0.505565869
ILMN_1798224	KDM5C	0.505260784
ILMN_1731714	CREB5	0.505214628
ILMN_1676868	RAB37	0.505029132
ILMN_3243909	SNORD102	0.504939992
ILMN_3275345	RPS27	0.504934823
ILMN_3248247	LOC349114	0.504832849
ILMN_1679319	PSIP1	0.504615319
ILMN_1692539	SH3BP1	0.504592664
ILMN_2123743	FCER1G	0.504572155
ILMN_3251100	MEF2A	0.504472912
ILMN_1754894	C1orf162	0.504445438
ILMN_1801118	SNRNP25	0.504395879
ILMN_1714489	CIB2	0.504336777
ILMN_1764201	MAP2	0.504245777
ILMN_1786612	PSME2	0.504243476
ILMN_2167805	LUM	0.504212169
ILMN_1751572	TLE1	0.504062806
ILMN_3307887	PRR14L	0.503915548
ILMN_1692168	UBE2Z	0.503888953
ILMN_3238375	CENPT	0.503814022
ILMN_2349393	MDK	0.503805277
ILMN_1747775	STX2	0.503647646
ILMN_1673352	IFITM2	0.503529777
ILMN_1733947	FKBP8	0.503429627
ILMN_1689160	DPEP2	0.503404871
ILMN_2278539	WDR16	0.503375051
ILMN_3252771	RPL18A	0.503368818
ILMN_2206746	BGN	0.503337334
ILMN_1709953	DONSON	0.503309055
ILMN_1679809	GSTP1	0.503215153
ILMN_1803476	KCTD20	0.50306082
ILMN_1708101	LMNB2	0.502689934
ILMN_1755733	RPLP2	0.502522172
ILMN_1750178	FAM21C	0.50243158
ILMN_2229922	C12orf35	0.502429549
ILMN_1797594	NFAT5	0.50221768
ILMN_1653708	CORO1B	0.502125834
ILMN_1810228	TTF2	0.502096816
ILMN_2111932	SERINC2	0.501939306
ILMN_3233179	HS6ST1	0.501730054
ILMN_1743347	AKT2	0.501490072
ILMN_1797988	KLRD1	0.501364206
ILMN_1703949	KPNB1	0.501283333
ILMN_1779639	IRAK1BP1	0.501247463
ILMN_2144791	GOLGA6A	0.501122041
ILMN_1701877	AXL	0.5010946
ILMN_1678841	UBD	0.501055834
ILMN_1812559	SLC7A6	0.501019635
ILMN_2390427	FLT4	0.500866883
ILMN_1698213	RBM3	0.500859767

ILMN_1910645	NA	-0.45610193
ILMN_1772712	UBE2J2	-0.456122674
ILMN_1889215	BF434110	-0.45619257
ILMN_2406815	LRRC17	-0.456199837
ILMN_1738818	RAG1	-0.456251867
ILMN_2145044	FAM173B	-0.456324445
ILMN_1810400	IDH3B	-0.456331048
ILMN_1672287	MYNN	-0.456381305
ILMN_1764415	ZNF585A	-0.456381993
ILMN_1793203	SMCR7L	-0.456634087
ILMN_1669747	UNC13B	-0.456720613
ILMN_1762506	LOC100506422	-0.456812887
ILMN_1787595	B3GALNT1	-0.456881398
ILMN_1669410	CHGA	-0.457021844
ILMN_1723418	CEL	-0.457055681
ILMN_1761112	PORCN	-0.457174998
ILMN_1735064	PPP1R3E	-0.457232952
ILMN_1891927	DCBLD1	-0.457244118
ILMN_2279844	LCK	-0.457266783
ILMN_1723834	KNCN	-0.457341855
ILMN_1683162	COL8A1	-0.457720581
ILMN_1863858	AI745199	-0.457903412
ILMN_1867214	LOC400654	-0.457937357
ILMN_3241848	MIR3647	-0.45796134
ILMN_2371825	AGL	-0.458113699
ILMN_2156267	EIF2AK1	-0.458129896
ILMN_1785919	TMEM191C	-0.458353223
ILMN_1838655	CR614803	-0.458430967
ILMN_2272519	FGR	-0.458486532
ILMN_1881408	AL120241	-0.458509247
ILMN_3248193	AI796012	-0.458569306
ILMN_1753472	SUMF1	-0.458592795
ILMN_1807633	HRSP12	-0.458777564
ILMN_1774708	ORMDL2	-0.458861726
ILMN_3289779	NA	-0.458938543
ILMN_1728074	PHAX	-0.459031257
ILMN_1729402	CHRM2	-0.459037826
ILMN_1692511	TMEM106C	-0.459056832
ILMN_2412849	SLC5A10	-0.459067856
ILMN_1777556	SOAT2	-0.459073562
ILMN_3292042	LOC284688	-0.459104961
ILMN_1766115	PLEKHF2	-0.459236048
ILMN_1904791	AK023763	-0.459242528
ILMN_1787705	ATP6V1B2	-0.459272998
ILMN_3246902	ABCC6P1	-0.459417962
ILMN_3241987	DGCR9	-0.459444892
ILMN_1789387	KCNE3	-0.459470537
ILMN_1677919	GMPR2	-0.459575838
ILMN_1678968	GEM	-0.459664064
ILMN_1784333	SECISBP2L	-0.459669435
ILMN_3309114	KRTAP20-3	-0.459813652
ILMN_1735822	TTC30A	-0.459830155
ILMN_3246634	SDHA	-0.459917245
ILMN_1653761	MYCN	-0.459918351
ILMN_1756125	H1FNT	-0.460039553
ILMN_2311020	DNAJC12	-0.460061622
ILMN_1731157	MYOZ1	-0.460066713
ILMN_1781859	UGT2B28	-0.460070664
ILMN_1775034	MEIG1	-0.460092104
ILMN_1736162	GC	-0.460177403
ILMN_2322458	OPA1	-0.460227093
ILMN_1707649	MPDZ	-0.460300028
ILMN_1773174	PDPK1	-0.460313276
ILMN_1799710	CCDC96	-0.460329346
ILMN_3308592	MIR98	-0.460441304
ILMN_1673867	LIMD2	-0.460614452
ILMN_1680754	A4GNT	-0.460730483
ILMN_1810738	YIPF5	-0.460741971
ILMN_2094905	COMMD10	-0.460767473
ILMN_1789410	ZSCAN21	-0.460815507
ILMN_3248747	LOC647983	-0.460815902
ILMN_1826447	BX107250	-0.460921282
ILMN_1746652	UNC93A	-0.461103159
ILMN_1892161	DB338885	-0.461143829
ILMN_1813664	HIRA	-0.461172351
ILMN_1717295	C6orf122	-0.461203638
ILMN_3187903	AL582488	-0.461300264
ILMN_1782204	AFP	-0.461371801
ILMN_1755352	TUB	-0.461399675
ILMN_1781381	AK123658	-0.461466029
ILMN_1719032	LSM3	-0.461512404
ILMN_1694514	ZDHHC11	-0.461519713
ILMN_1877039	BU858876	-0.461581381
ILMN_3247013	EGOT	-0.461854066
ILMN_1692651	PHB	-0.461859181
ILMN_2381697	P4HA2	-0.461916276
ILMN_2097943	C1orf87	-0.461957805
ILMN_1809117	MAD2L1BP	-0.461977685
ILMN_2178116	MMP20	-0.462015161
ILMN_2171384	CXCL5	-0.462047193
ILMN_1701565	UPK3B	-0.462133779
ILMN_1751576	TEK	-0.46218456
ILMN_1776283	LGALS12	-0.46221185
ILMN_1798712	USP4	-0.462281091
ILMN_1657455	CEACAM4	-0.462525625
ILMN_1766678	NKX2-6	-0.462538832
ILMN_3234746	NA	-0.462544963
ILMN_1770080	FAM159B	-0.462570066

ILMN_1779399	SNRPA	0.500802318
ILMN_1749448	GLMN	0.5008
ILMN_1710885	RPSA	0.500792543
ILMN_1698100	ANX2P2	0.500651966
ILMN_1755462	UGCGL1	0.500643916
ILMN_1714433	MARCKSL1	0.5004155
ILMN_1796336	MAP1S	0.50036164
ILMN_2110271	OLFM2	0.500226517
ILMN_1807277	IFI30	0.500173286
ILMN_2129563	LDLRAD3	0.500045186
ILMN_1711790	DEFB135	0.499986343
ILMN_1775327	PKM2	0.499758276
ILMN_2347949	G6PD	0.499623346
ILMN_1701655	SLC24A6	0.49962306
ILMN_1762260	C3	0.499430819
ILMN_1667925	PDCL3	0.49941449
ILMN_1709549	PLEKHM1	0.499390185
ILMN_3242330	FAM65C	0.499383395
ILMN_1805737	PFKP	0.499321945
ILMN_1758523	ABCA3	0.499218375
ILMN_1656310	IDO1	0.499149402
ILMN_2384496	ST6GAL1	0.499119137
ILMN_1695829	TTYH2	0.499028723
ILMN_1725992	RAPGEF6	0.4990114
ILMN_2349658	TSPO	0.498984191
ILMN_1799815	XYLT2	0.498834189
ILMN_1724480	AXIN2	0.498728079
ILMN_1760027	WAS	0.498483529
ILMN_1651826	BASP1	0.498416917
ILMN_1786823	ICAM2	0.498320995
ILMN_1795089	RASAL3	0.498290124
ILMN_1712431	FAM113B	0.498251163
ILMN_2381834	TRIM69	0.498174843
ILMN_1690826	TNKS1BP1	0.498173242
ILMN_1805750	IFITM3	0.498123785
ILMN_3241884	SNORA15	0.498086022
ILMN_1696704	TLE4	0.498075863
ILMN_2396020	DUSP6	0.498052385
ILMN_2380605	MTMR3	0.498033688
ILMN_1714216	TSC2	0.497853945
ILMN_1695414	ASF1B	0.497837517
ILMN_1722798	PLCD3	0.497765121
ILMN_1723706	GRAMD4	0.49773219
ILMN_1665583	TUBB	0.497718795
ILMN_1755234	SSH3	0.497692691
ILMN_3208973	LAMR1P15	0.497440617
ILMN_3238803	RASAL3	0.49733205
ILMN_1688573	CREB5	0.497330082
ILMN_1672650	PKM2	0.497208153
ILMN_2160819	RPS27	0.497083978
ILMN_2075847	ZC3H10	0.49677906
ILMN_1716382	C12orf75	0.496711769
ILMN_1663068	C1orf228	0.49660777
ILMN_1747556	CDK9	0.496395963
ILMN_1808405	HLA-DQA1	0.496314436
ILMN_3230608	NBPF14	0.496001807
ILMN_1754871	TSC1	0.495961226
ILMN_1670302	HK3	0.495922778
ILMN_2368530	IL32	0.495831726
ILMN_1670878	YTHDC1	0.495572725
ILMN_1695590	ADRB2	0.495514983
ILMN_3281563	RPL12	0.495377705
ILMN_2300396	COMMD5	0.495363107
ILMN_1731048	TLR1	0.495256822
ILMN_1676091	HNRNPA1	0.495221126
ILMN_1669842	CHAF1A	0.495111968
ILMN_1657810	PPM1M	0.495018764
ILMN_1788149	NEK11	0.494904841
ILMN_3310840	MIR21	0.494839702
ILMN_2206141	BAGE	0.494676322
ILMN_1690105	STAT1	0.494645785
ILMN_1651429	SELM	0.49449607
ILMN_1798204	IL21R	0.49440278
ILMN_1773885	DMAP1	0.494242806
ILMN_1791127	CDH12	0.49408368
ILMN_1811984	KRT86	0.494024915
ILMN_1715647	VANGL2	0.493962373
ILMN_1690610	RALY	0.49380656
ILMN_3206132	RPS8	0.493795952
ILMN_1750271	MOBP	0.493648844
ILMN_1697377	ANKRD36B	0.493628314
ILMN_1784467	NUP210	0.493486687
ILMN_1681543	RHBDD1	0.493432693
ILMN_1654685	MCTP1	0.493369158
ILMN_1654392	KHNYN	0.493214934
ILMN_1796247	CRTAM	0.493178274
ILMN_1803825	CXCL12	0.493169884
ILMN_2163187	FLJ35776	0.493134733
ILMN_1755850	ZNF350	0.493129021
ILMN_1673363	CD97	0.493094559
ILMN_1806320	PPFIBP1	0.493081766
ILMN_1656904	SLC1A4	0.493062129
ILMN_1708025	RBPMS	0.492933116
ILMN_2389876	TGFB1I1	0.492805038
ILMN_1725427	B2M	0.492624699
ILMN_1772888	AX746718	0.492619987
ILMN_2382431	ANKRD36	0.492608395
ILMN_2130180	RPL13P5	0.492453171

ILMN_1745152	UQCC	-0.462632529
ILMN_2119945	NDUFB3	-0.462646285
ILMN_1755251	ITIH1	-0.462687274
ILMN_2167808	TM4SF5	-0.462769431
ILMN_1795419	CNPPD1	-0.462791817
ILMN_1769299	MTMR11	-0.462850426
ILMN_1754315	MMAA	-0.462851519
ILMN_2402629	METTL9	-0.462853523
ILMN_3229083	SNX5	-0.462939543
ILMN_1749540	RBM20	-0.46296462
ILMN_1682544	CLUL1	-0.463150364
ILMN_3177250	WSCD2	-0.463217692
ILMN_2370910	RAD51L3	-0.463288394
ILMN_1659270	OTP	-0.463325444
ILMN_1677607	SC5DL	-0.463469783
ILMN_1735719	UNG	-0.463505571
ILMN_3270866	TACC2	-0.463604444
ILMN_1804350	C1orf150	-0.463621419
ILMN_1710522	RUNX1T1	-0.46373247
ILMN_1800253	ZNF711	-0.464001054
ILMN_1784286	NDUFA1	-0.464014704
ILMN_1722643	DPP6	-0.464026305
ILMN_1871488	BQ027500	-0.464047278
ILMN_1699164	FAM108A1	-0.464225448
ILMN_3247723	NOP16	-0.464372955
ILMN_1786361	RDM1	-0.464431726
ILMN_2179018	NDUFAB1	-0.464452205
ILMN_1698587	PLG	-0.464586084
ILMN_2080637	ZBTB44	-0.4646131
ILMN_2337941	COPS8	-0.46465209
ILMN_1761260	COBLL1	-0.46465647
ILMN_1719546	LOC202181	-0.464713821
ILMN_1736054	SUB1	-0.464844628
ILMN_2211790	ADAMTS7	-0.464870383
ILMN_2224034	CETN3	-0.464931593
ILMN_1903021	AX746755	-0.464933947
ILMN_2052331	MYOC	-0.465128098
ILMN_1681032	C16orf54	-0.465198352
ILMN_2197381	PCK1	-0.465278579
ILMN_1714197	ACSS2	-0.46549125
ILMN_1652291	EVX2	-0.465650286
ILMN_2117736	MGAT4C	-0.465718663
ILMN_2215194	GCLC	-0.465779716
ILMN_2125618	SMEK3P	-0.465789886
ILMN_1782579	IMMT	-0.465790573
ILMN_1874172	BX097339	-0.465988979
ILMN_3182120	NA	-0.466011643
ILMN_1715324	HSD17B8	-0.466161786
ILMN_1684339	LRIT1	-0.466168577
ILMN_1758673	SLC44A1	-0.466174208
ILMN_1809148	OR6C74	-0.46640513
ILMN_1692938	PSAT1	-0.466441772
ILMN_1696031	C15orf21	-0.466469582
ILMN_1676288	ACBD4	-0.466482457
ILMN_1703091	C2orf27B	-0.466590892
ILMN_1663128	LOC643406	-0.46663598
ILMN_1715437	CASC5	-0.466698894
ILMN_2119555	TMTC3	-0.466783933
ILMN_3237655	SLC29A4	-0.466892564
ILMN_3238823	CXorf49	-0.466979771
ILMN_2253805	GFRA1	-0.46699992
ILMN_1796749	KIF1C	-0.467027571
ILMN_3187862	DNAH14	-0.467110484
ILMN_3273620	KLHL33	-0.467113418
ILMN_2350301	PRB1	-0.467141418
ILMN_2180380	KRTAP5-6	-0.46728225
ILMN_1878191	AA177072	-0.467318138
ILMN_3292122	NA	-0.467486966
ILMN_1714739	AK096522	-0.467517971
ILMN_2344956	ACP1	-0.467646043
ILMN_1698894	OR5AS1	-0.467663285
ILMN_2414302	FDXR	-0.467669718
ILMN_1758941	REEP5	-0.467677508
ILMN_1750689	MPP5	-0.467782776
ILMN_1797878	OR7G3	-0.467858823
ILMN_1676086	KRT82	-0.467893747
ILMN_1848440	BX493900	-0.467926902
ILMN_1815599	FOXE3	-0.467946897
ILMN_1798886	NUDT21	-0.468037318
ILMN_1836309	MCF2L-AS1	-0.468221339
ILMN_3270993	AL582488	-0.468226382
ILMN_1847202	AW173314	-0.468275473
ILMN_1764034	XCR1	-0.468298573
ILMN_1753249	DDX10	-0.468478543
ILMN_1667670	SLC25A15	-0.46852534
ILMN_2058512	PSMA2	-0.468528567
ILMN_1762207	SGSM1	-0.468557067
ILMN_1804490	LOC100630923	-0.468572027
ILMN_1772882	OR2T1	-0.468597208
ILMN_3269076	NA	-0.468697623
ILMN_1666624	RABL2A	-0.4689534
ILMN_2167709	NIPSNAP3A	-0.469103728
ILMN_1754055	APOA5	-0.46914733
ILMN_1683737	SNX32	-0.469292956
ILMN_1793537	MUC15	-0.469468373
ILMN_1803073	DNAJC12	-0.469511403
ILMN_1660787	SUCLA2	-0.469522167
ILMN_2131828	KIR3DL1	-0.469583464

ILMN_1658206	OSCP1	0.492431169
ILMN_2068104	TFPI2	0.492384832
ILMN_1733581	C16orf35	0.492072043
ILMN_2278729	EIF3B	0.492037584
ILMN_2338447	ARID4A	0.491954335
ILMN_1693650	FES	0.49181402
ILMN_1693986	WHAMML2	0.491752407
ILMN_2363586	SDCBP	0.49173784
ILMN_1750088	VRK2	0.491640857
ILMN_2356578	TH	0.491371873
ILMN_1679929	KLF13	0.491253007
ILMN_1753716	EIF2B1	0.491217209
ILMN_1667551	SMA4	0.491001738
ILMN_2113049	QPCT	0.490989386
ILMN_2059689	TMEM54	0.490749543
ILMN_2089073	ATP9A	0.490697833
ILMN_1694203	ZNF141	0.49066539
ILMN_2112673	POTEKP	0.490634365
ILMN_1738347	RNPEP	0.49056004
ILMN_1670383	PCDH7	0.490551108
ILMN_1666444	RBMS1	0.490401754
ILMN_1739847	EIF3D	0.490340905
ILMN_3200362	NBPF1	0.490265002
ILMN_2359800	MS4A6A	0.490259581
ILMN_1898518	GFRA1	0.490161623
ILMN_2361807	OS9	0.490131931
ILMN_1684836	AKAP12	0.490069997
ILMN_1787109	CLK2	0.490031476
ILMN_1705876	NAP1L1	0.489988946
ILMN_2333107	AES	0.489912392
ILMN_2324202	GABRE	0.489727064
ILMN_1666222	PHACTR3	0.489640078
ILMN_1659762	BTF3	0.489124501
ILMN_1772036	STEAP4	0.488940059
ILMN_1754842	DLGAP4	0.488782341
ILMN_1898124	C7orf40	0.488742778
ILMN_2370772	EIF4G1	0.488666468
ILMN_2391891	MAP2K5	0.488648588
ILMN_1696749	LMNA	0.488487034
ILMN_3247018	EIF4A1	0.488240838
ILMN_1663799	RPL32	0.488173185
ILMN_1664177	ATXN7L2	0.48817078
ILMN_1655766	ANKRD34B	0.488128536
ILMN_2345908	DDX11	0.488091505
ILMN_2388090	MAPK14	0.487997242
ILMN_1782543	EEF1D	0.487893334
ILMN_2092756	TMEM109	0.487843306
ILMN_3205924	EIF3F	0.487713197
ILMN_2219351	CENPO	0.487514536
ILMN_1711899	ANXA2	0.487218913
ILMN_1675857	COL4A6	0.487136195
ILMN_1697742	JF432672	0.486956817
ILMN_1815445	IDS	0.486891202
ILMN_2317751	REC8	0.486855074
ILMN_1855286	C6orf204	0.48672629
ILMN_3181411	ATL1	0.486647294
ILMN_3204210	RPL10	0.486480882
ILMN_1687546	HSP90AA1	0.486392337
ILMN_2064606	TBC1D2B	0.486354685
ILMN_1715947	HNRNPA1	0.486339916
ILMN_1814971	TCF25	0.48620247
ILMN_1675709	ARFGAP1	0.486110135
ILMN_1792489	ARPC2	0.486051782
ILMN_1733799	FAM195B	0.486004186
ILMN_3234828	TMEM229B	0.485929642
ILMN_1710524	PARD3	0.485925348
ILMN_1808777	EHD2	0.485812889
ILMN_1748812	TMEM130	0.485666537
ILMN_3221790	RPL7L1	0.48563651
ILMN_1790782	MED16	0.485454101
ILMN_1730940	KLHDC3	0.485368731
ILMN_2184640	NOLC1	0.485301222
ILMN_1660554	VWA1	0.485296262
ILMN_1670926	CHST15	0.485292237
ILMN_1723486	HK2	0.485224916
ILMN_1760922	GIT2	0.485200091
ILMN_1728984	PA2G4	0.48510665
ILMN_2286514	GTPBP8	0.485083078
ILMN_2139943	RPS3A	0.484952685
ILMN_3267760	RPL10A	0.484899777
ILMN_1684983	OTUB2	0.484738814
ILMN_1703622	PPIB	0.484419851
ILMN_1676099	SPON2	0.484362427
ILMN_1699015	H3F3A	0.484233016
ILMN_1746604	IL6ST	0.484127925
ILMN_3238001	LOC349114	0.484064396
ILMN_2088172	POLR2B	0.483964985
ILMN_2398847	ARHGAP17	0.483899355
ILMN_1718558	PARP12	0.483813946
ILMN_1771593	RRM1	0.483716918
ILMN_1739618	ZNF408	0.483694837
ILMN_1745963	FOLR2	0.483684606
ILMN_1664706	HIST2H3D	0.483556889
ILMN_2141444	RPL18A	0.483539395
ILMN_1763386	BID	0.483336977
ILMN_1674386	PITX1	0.483209242
ILMN_1689371	GIMAP2	0.482977587
ILMN_2230683	CDCA7L	0.482896994

ILMN_1719163	ZNF557	-0.469745889
ILMN_1770005	PTPN18	-0.469813959
ILMN_1654689	ZNF197	-0.469814789
ILMN_2347097	KLK15	-0.469915103
ILMN_2388669	GRIA3	-0.469957587
ILMN_3235817	LOC100132941	-0.469959777
ILMN_1765460	AMIGO1	-0.470206894
ILMN_1916311	DB069547	-0.470213757
ILMN_3306028	BM932296	-0.470350667
ILMN_2372398	ALDH5A1	-0.470359076
ILMN_1655626	KIAA1931	-0.470389733
ILMN_1691959	AKAP2	-0.470447712
ILMN_1796074	C18orf56	-0.470452022
ILMN_3244025	DUX2	-0.470690968
ILMN_2215370	WWP1	-0.470730825
ILMN_3305339	UBA5	-0.470799455
ILMN_1739813	HYAL1	-0.470816403
ILMN_2163306	FAM120A	-0.470834054
ILMN_1794844	THRSP	-0.470841943
ILMN_1779501	MC5R	-0.47084618
ILMN_1840311	DA494348	-0.470855677
ILMN_2314140	PAX6	-0.470956121
ILMN_1683133	KLF15	-0.47102753
ILMN_1727466	KCNMB4	-0.47105307
ILMN_3249439	ZNF878	-0.471099889
ILMN_1671619	HMX2	-0.471104661
ILMN_1753856	ZNF407	-0.471231463
ILMN_3180456	NA	-0.471231526
ILMN_1782070	NPL	-0.471299675
ILMN_1767649	PCDHGC3	-0.471299724
ILMN_1744647	CAND1	-0.471304907
ILMN_2123525	SYCP3	-0.47143686
ILMN_1726144	CDC27	-0.471522961
ILMN_1900457	NA	-0.471595509
ILMN_1788156	EPM2A	-0.471620005
ILMN_1795300	ARHGEF37	-0.471865665
ILMN_2230016	HIGD1A	-0.471919625
ILMN_3249546	RASA4	-0.471949103
ILMN_2100357	SMPDL3A	-0.472110672
ILMN_1739275	HTR3C	-0.472208005
ILMN_1709570	IMMP1L	-0.472288038
ILMN_1685079	TELO2	-0.472298581
ILMN_3250322	DPPA3	-0.472369947
ILMN_3242164	SNORD59B	-0.47239334
ILMN_2210713	COMMD8	-0.47239676
ILMN_1803774	STK16	-0.47240134
ILMN_2156699	ACOT6	-0.472489087
ILMN_1711650	KCNE1L	-0.472530513
ILMN_2124471	SLC36A1	-0.472643621
ILMN_1680925	SLC9A3R1	-0.472649398
ILMN_2296843	GCDH	-0.472675867
ILMN_1712751	HADHA	-0.472749042
ILMN_1671482	GALM	-0.472866255
ILMN_2100815	TMEM9B	-0.473093147
ILMN_1782881	DLEU7	-0.473114697
ILMN_3200203	LOC100131180	-0.473206426
ILMN_1768867	AP3B1	-0.473216348
ILMN_1813938	CHCHD4	-0.473283547
ILMN_1750901	GNAT1	-0.473343079
ILMN_1696537	DDIT4L	-0.473447746
ILMN_1672443	QDPR	-0.473608872
ILMN_1849744	DA584132	-0.473721398
ILMN_1736218	CHRNB3	-0.473819383
ILMN_1734897	SLC4A4	-0.473841751
ILMN_1660963	CCIN	-0.474004041
ILMN_1658083	ABT1	-0.474040114
ILMN_3251278	DPPA3	-0.474046263
ILMN_1704477	COX5A	-0.474132301
ILMN_1812503	PHKA1	-0.47422592
ILMN_1806040	TYMS	-0.47424293
ILMN_1706005	GALT	-0.474279075
ILMN_3240451	VWDE	-0.474322237
ILMN_2268068	MAPKAP1	-0.474373916
ILMN_1745851	LOC389676	-0.474526324
ILMN_1859393	AI203701	-0.474631805
ILMN_1775467	OR2T12	-0.474794437
ILMN_1809695	CAMK2G	-0.474817302
ILMN_3308976	MIR135B	-0.474903616
ILMN_1690783	TREML1	-0.474967789
ILMN_1665856	CA10	-0.475005065
ILMN_1678170	MME	-0.475022872
ILMN_1785731	MAPK8IP2	-0.475056564
ILMN_1895518	AW468861	-0.47507391
ILMN_1663220	MRPL22	-0.475133187
ILMN_1687589	CPT1A	-0.475200341
ILMN_1741843	RD3	-0.475235434
ILMN_1682034	HEY2	-0.475239162
ILMN_1806787	CSDC2	-0.475298893
ILMN_1740263	P2RX3	-0.475326862
ILMN_1731948	PCK1	-0.475439981
ILMN_1770260	NFKBIZ	-0.47544968
ILMN_2317739	ITSN2	-0.475511129
ILMN_2400583	USP9X	-0.475697757
ILMN_1699320	LOC339524	-0.475849926
ILMN_1706901	HOXD13	-0.475933025
ILMN_1775742	RNF128	-0.475975354
ILMN_1762582	ARNT	-0.476038327
ILMN_3271630	FGGY	-0.47609754

ILMN_1798181	IRF7	0.482883978
ILMN_1758100	GALR3	0.482734247
ILMN_2399328	COBRA1	0.48267825
ILMN_1745148	ZNFX1	0.482664352
ILMN_1783771	UBE2Z	0.482628862
ILMN_1742167	TUBA1C	0.482601782
ILMN_1719835	OSCP1	0.482467305
ILMN_1744109	OR2A42	0.482420344
ILMN_2081269	OR2A9P	0.482216933
ILMN_1808634	DRAM2	0.482211079
ILMN_1663390	CDC20	0.482209597
ILMN_2057573	ESYT2	0.482076289
ILMN_1763260	HIF1A	0.482050535
ILMN_1666257	XCL1	0.48197394
ILMN_1685202	KRBA2	0.481951013
ILMN_1737157	GRAMD1A	0.48188054
ILMN_1702044	CNTROB	0.481766901
ILMN_3185223	NA	0.481727021
ILMN_3230572	NCRNA00152	0.481508806
ILMN_1687501	MOXD1	0.481339123
ILMN_2041101	ANX2P2	0.481272798
ILMN_1855278	AW954199	0.481263936
ILMN_1738523	MYD88	0.481210536
ILMN_1779234	CXCL6	0.481203102
ILMN_1720287	E4F1	0.481191621
ILMN_3250321	PTP4A3	0.481181193
ILMN_1661888	MEF2A	0.481131349
ILMN_1716488	PACS1	0.48101636
ILMN_1728677	CREB5	0.480994618
ILMN_2127605	LRP3	0.480942874
ILMN_1793950	POTEM	0.480843437
ILMN_1705737	IMPDH2	0.480830044
ILMN_1695341	KCNK10	0.480803968
ILMN_1793990	ID2	0.480587867
ILMN_2059505	ARPP19	0.480479573
ILMN_3307926	ADRBK1	0.480454474
ILMN_2053178	ACTG1	0.480437449
ILMN_1689953	CD81	0.480345489
ILMN_1722820	KDELR3	0.480313251
ILMN_1737314	BCL6	0.480285393
ILMN_3180989	DSTYK	0.48027329
ILMN_1667295	VASN	0.480182817
ILMN_1785831	ZDHHC13	0.480154459
ILMN_1807972	MICAL1	0.480054752
ILMN_2401978	STAT3	0.480032138
ILMN_1753862	SRP54	0.479991104
ILMN_3244666	FLJ00326	0.479938802
ILMN_2397294	PLCB4	0.479459389
ILMN_2387496	COAS3	0.479425844
ILMN_1773126	FANK1	0.47939042
ILMN_3246360	RPS18	0.479382421
ILMN_1770206	GEMIN4	0.479069377
ILMN_1658399	KLRG1	0.479056045
ILMN_1723895	GTF3C5	0.479027686
ILMN_3192001	CCL15	0.478991336
ILMN_2301624	MACF1	0.478981637
ILMN_1679039	CASKIN1	0.478953822
ILMN_1674036	BIRC7	0.478915805
ILMN_1737918	C1QA	0.478730542
ILMN_2169152	SRGN	0.478598646
ILMN_1872759	BX099468	0.478513883
ILMN_1662397	FDPSL2A	0.478467504
ILMN_3252556	CARD16	0.478457455
ILMN_2402272	TCEAL8	0.478244957
ILMN_1755758	RIF1	0.47821724
ILMN_1720083	EHD4	0.478167319
ILMN_1703866	SUPT5H	0.478106941
ILMN_2407482	ITPA	0.478085581
ILMN_2292178	CLEC12A	0.477959658
ILMN_2199409	EFHB	0.477925873
ILMN_1776678	GIMAP7	0.477667448
ILMN_3241979	TMEM179B	0.477664085
ILMN_2060413	CD24	0.477635065
ILMN_1714158	PON2	0.477572737
ILMN_3245236	FBRS	0.47737823
ILMN_1658182	MEX3C	0.477301928
ILMN_1775111	SND1	0.477288295
ILMN_1773389	PLTP	0.477275193
ILMN_1661337	SRM	0.477221112
ILMN_3247679	SNORA52	0.477215449
ILMN_1715639	FLJ00310	0.477045295
ILMN_1753608	TMEM131	0.477040388
ILMN_1728236	SLFN12	0.476983805
ILMN_2141452	RPL18A	0.47681638
ILMN_2312719	EXOSC9	0.476736639
ILMN_3246962	SPECC1L	0.476688213
ILMN_1811574	MAPK8IP3	0.476622907
ILMN_1767256	MBTPS1	0.476620386
ILMN_1691535	CUL4B	0.476564104
ILMN_2377733	CASP8	0.476382638
ILMN_1784320	ELMO1	0.476323106
ILMN_1806705	ASB6	0.476217045
ILMN_1781151	ARMC8	0.476086072
ILMN_1721977	NAA10	0.476033805
ILMN_2280568	ANKRD36	0.475894393
ILMN_1804476	GMPPA	0.475774645
ILMN_1761772	NUP155	0.475757137
ILMN_1652407	ZMYND8	0.475741927

ILMN_1778890	LRR1	-0.476143591
ILMN_1735453	FAM98A	-0.476182077
ILMN_1664118	CADM2	-0.476239069
ILMN_1696450	KLK1	-0.476329071
ILMN_1798187	MYST2	-0.476558638
ILMN_2248632	PCDH10	-0.47666392
ILMN_1705266	RELA	-0.476994016
ILMN_1716729	AK129567	-0.476998472
ILMN_2385672	ELN	-0.477058648
ILMN_1739872	ADAMTS18	-0.477103074
ILMN_1708508	PPM1E	-0.477352438
ILMN_3305273	PSAT1	-0.477380166
ILMN_1746618	PAQR7	-0.477530732
ILMN_1718932	MTRR	-0.477565956
ILMN_1808677	BC141517	-0.47771127
ILMN_1659010	AK124016	-0.47783717
ILMN_1782412	IRX2	-0.477858541
ILMN_1891277	NA	-0.477869221
ILMN_1683687	AK024951	-0.477876888
ILMN_1757081	SYN2	-0.477893042
ILMN_1803852	CSAG2	-0.477938264
ILMN_1699852	CES4A	-0.477943227
ILMN_1791030	NEUROG1	-0.477998554
ILMN_1672445	BC040831	-0.478001571
ILMN_1774901	GDPD3	-0.478040603
ILMN_1911500	DA455367	-0.478275537
ILMN_3244402	RIMBP3	-0.478442297
ILMN_3242011	MOBKL1B	-0.478573949
ILMN_1884220	AW513776	-0.478684565
ILMN_1785252	SLC26A6	-0.478697423
ILMN_2115073	OR1D5	-0.47882952
ILMN_1664102	ISY1	-0.47896282
ILMN_1795936	C3orf36	-0.47899691
ILMN_1791951	KRTAP13-2	-0.479049143
ILMN_2404085	CLIP1	-0.479070379
ILMN_1665824	C6orf218	-0.479120585
ILMN_2399463	VAV3	-0.479227385
ILMN_3244860	LRRC16B	-0.479264591
ILMN_2133996	SHE	-0.479363904
ILMN_2230566	RAB40B	-0.479397257
ILMN_1778617	TAF9	-0.479546418
ILMN_1795467	LOC399715	-0.479604734
ILMN_1755843	SLC26A8	-0.479648412
ILMN_1718657	FLJ43950	-0.479734113
ILMN_1722726	ARHGAP32	-0.479819092
ILMN_1697704	KRTAP12-2	-0.479839512
ILMN_1788729	TCF23	-0.479971888
ILMN_1685917	EMCN	-0.479982456
ILMN_1897741	CR610863	-0.480046609
ILMN_3240147	CXorf51	-0.480102671
ILMN_2193498	TRMT1L	-0.480294894
ILMN_1734660	ICAM4	-0.480390249
ILMN_2123550	OR8B12	-0.480647463
ILMN_1805127	C2CD2	-0.480743694
ILMN_1679979	PLK3	-0.480867472
ILMN_1651681	BC013377	-0.48096803
ILMN_1712999	MGAM	-0.481004616
ILMN_1785345	GPR84	-0.481087298
ILMN_1770927	KAZN	-0.481148785
ILMN_1673605	PRSS57	-0.481289027
ILMN_3235143	LOC731779	-0.48132559
ILMN_3191541	PTPRQ	-0.481500919
ILMN_1718060	OTUD7A	-0.481642657
ILMN_3252862	DKFZp667F0711	-0.481716443
ILMN_1856998	BC053535	-0.481758379
ILMN_2349982	FGF1	-0.481894847
ILMN_1861128	AW237129	-0.481917802
ILMN_1801443	TSKU	-0.48197255
ILMN_1767643	CTRB2	-0.482121464
ILMN_1723414	HACL1	-0.482204169
ILMN_2393341	LIAS	-0.482229813
ILMN_3306482	GCSH	-0.482374629
ILMN_1795304	CRLF2	-0.482400361
ILMN_1817715	CK001807	-0.482495321
ILMN_3293146	C20orf30	-0.482566137
ILMN_1701386	STRADB	-0.482573638
ILMN_2225511	BRMS1L	-0.482608079
ILMN_1821199	CR744726	-0.482629491
ILMN_1685396	CLIC5	-0.48275062
ILMN_2393456	DDO	-0.482759669
ILMN_1673752	C1orf91	-0.482941319
ILMN_1745688	HPD	-0.483020718
ILMN_3293159	BC015443	-0.48306216
ILMN_1745043	LDHC	-0.483174088
ILMN_1783443	ALOX15	-0.483209556
ILMN_1694780	GCHFR	-0.483225923
ILMN_1803094	PDGFD	-0.483289944
ILMN_2066151	TEK	-0.483338071
ILMN_1658499	SYT13	-0.483382217
ILMN_1724162	ARX	-0.483418842
ILMN_1681855	TBC1D25	-0.483439637
ILMN_1689001	CDK4	-0.483458466
ILMN_2079285	ATP5L	-0.483501275
ILMN_1694078	AY358310	-0.483513776
ILMN_1740233	UGT1A1	-0.483568453
ILMN_1913413	BX105847	-0.483629896
ILMN_1809304	PRAMEF12	-0.483674198
ILMN_1843101	AA866199	-0.4837002

ILMN_1720048	CCL2	0.47569866
ILMN_1652525	FAM125B	0.475673227
ILMN_1652412	PHKB	0.475580457
ILMN_1749789	HIST1H1D	0.475330558
ILMN_2415011	DCTD	0.475309398
ILMN_1658955	EIF4E	0.475309133
ILMN_1655572	C6orf59	0.475289642
ILMN_3203186	AL543610	0.475249303
ILMN_1683204	GMEB2	0.474844701
ILMN_1721967	OR2A7	0.474693143
ILMN_2149566	VPS25	0.474690159
ILMN_1873838	IGKC	0.474645264
ILMN_2383435	PSMD10	0.474522463
ILMN_1789733	CLIP3	0.474472305
ILMN_1684663	ZDHHC13	0.474451655
ILMN_1780987	RFXANK	0.474450388
ILMN_3238555	FKSG29	0.474365393
ILMN_1654560	TLR1	0.474308115
ILMN_2333440	TM9SF1	0.474181545
ILMN_1657435	MT1M	0.474087628
ILMN_1690268	HNRNPUL1	0.474017095
ILMN_1664823	TAOK3	0.473980023
ILMN_1794707	ATHL1	0.473922333
ILMN_3237826	CNTNAP3B	0.473907243
ILMN_1793371	KIAA0430	0.473876984
ILMN_1699348	MS4A4E	0.473762017
ILMN_1657095	STARD3	0.473745325
ILMN_1772189	ABCD1	0.473731207
ILMN_1775999	ATAT1	0.473720204
ILMN_1805161	LZTR1	0.473709257
ILMN_1769245	GLIPR1	0.473698778
ILMN_1659936	PPP1R15A	0.473490307
ILMN_1801939	CCNB2	0.473395166
ILMN_1759003	SNX12	0.473345944
ILMN_1805812	TOR1A	0.473296555
ILMN_1727642	MOGS	0.47329253
ILMN_1796085	PPM1B	0.473210471
ILMN_1728802	SDCCAG8	0.473200571
ILMN_1779010	MAP3K3	0.473112094
ILMN_1794740	CD151	0.473072831
ILMN_1692473	PRMT1	0.47304883
ILMN_1737394	LMNA	0.473033224
ILMN_1720476	PHF2	0.473003079
ILMN_1700028	C9orf156	0.472899317
ILMN_1815130	MICALL1	0.472796437
ILMN_3239397	NA	0.472767779
ILMN_2103107	ADAMDEC1	0.472759097
ILMN_1659843	DNAJC3	0.472683521
ILMN_1769201	ELF3	0.472616649
ILMN_1776490	C17orf53	0.472524573
ILMN_3199798	RPL10	0.47251705
ILMN_1698139	TCEA1	0.472514223
ILMN_1736814	CLNS1A	0.472481425
ILMN_1686981	SULF2	0.472459163
ILMN_1693009	FGL2	0.472404388
ILMN_1761138	C9orf142	0.472404039
ILMN_1755235	XPO6	0.47233406
ILMN_1815519	EPN2	0.47221135
ILMN_1653292	PFKFB4	0.472132939
ILMN_2147078	PIWIL4	0.471950849
ILMN_1808860	STX5	0.471910167
ILMN_1666050	TMUB1	0.471732573
ILMN_1774779	EMB	0.471629371
ILMN_1697559	G6PD	0.471511836
ILMN_1795236	SIGLEC9	0.471499151
ILMN_3211187	TUBB2A	0.471456099
ILMN_1811117	DKFZp667P092	0.471405326
ILMN_1659905	FKBP10	0.471280524
ILMN_2376289	DBNL	0.471276698
ILMN_1724066	PLCB2	0.471130711
ILMN_1682928	CPVL	0.471076607
ILMN_1814789	UBAP2L	0.471064613
ILMN_2155228	CRLF3	0.471058137
ILMN_1667966	FAM129A	0.470975231
ILMN_2382906	PLCG1	0.470743885
ILMN_2414325	TNFAIP8	0.470670993
ILMN_3274666	CR601848	0.4706621
ILMN_1811921	CSRP1	0.470652294
ILMN_1805448	EPB41L2	0.470640447
ILMN_1774602	FBLN2	0.470596384
ILMN_2322446	PABPC1L2B	0.470584651
ILMN_2379599	CD163	0.470498642
ILMN_1802376	FAM48A	0.470219254
ILMN_1661917	RPL10	0.470204378
ILMN_1782729	CLECL1	0.470130354
ILMN_1862018	ATXN7L3	0.470108933
ILMN_1668185	ZNF282	0.470026401
ILMN_1698653	ODF2	0.469932959
ILMN_1717934	SYT11	0.469927444
ILMN_2372974	SIRPA	0.469922456
ILMN_2415776	WWOX	0.469915973
ILMN_1772486	ELF2	0.469829868
ILMN_3304012	NA	0.469812549
ILMN_1790460	ZNF574	0.469744684
ILMN_1778255	FARSA	0.469439492
ILMN_3310436	MIR567	0.469398817
ILMN_3229324	SGK1	0.469355562
ILMN_2215640	TUBA3D	0.469275408

ILMN_1749641	FBXO3	-0.48370793
ILMN_1806434	PAQR5	-0.483756088
ILMN_1806473	BEX5	-0.483826227
ILMN_2244631	STEAP1	-0.483909151
ILMN_1814790	SHANK2	-0.483942768
ILMN_1711289	GYS1	-0.484240752
ILMN_1651636	CYP2A13	-0.484350478
ILMN_1767503	GABRB3	-0.484415091
ILMN_2380999	RECQL	-0.484533999
ILMN_2384629	RGS12	-0.484690158
ILMN_1769547	DIO1	-0.484873245
ILMN_1699384	PPP2R5D	-0.484944671
ILMN_1781060	SYN2	-0.485047939
ILMN_3209156	LOC100131372	-0.485090498
ILMN_1855996	AI985753	-0.485126739
ILMN_1705049	TMEM67	-0.48524443
ILMN_1706690	KHK	-0.485374178
ILMN_1728107	GNG7	-0.48608627
ILMN_1708382	C3orf75	-0.486255566
ILMN_1900347	AI701700	-0.486439212
ILMN_1732269	GP6	-0.486552973
ILMN_1877939	BX109006	-0.486695141
ILMN_1788778	sept-11	-0.486757637
ILMN_2338038	AK4	-0.486793132
ILMN_2094587	USP8	-0.486834593
ILMN_1708721	RABGAP1L	-0.486957896
ILMN_2183687	LIME1	-0.487017792
ILMN_2103003	OR4N3P	-0.487116783
ILMN_1688663	IFNA1	-0.487198793
ILMN_3292690	NA	-0.487245737
ILMN_2388517	MTERFD3	-0.487249164
ILMN_1907451	BC038752	-0.487257821
ILMN_3237589	PHAX	-0.487358527
ILMN_1761131	ECI2	-0.487374134
ILMN_1801223	WTIP	-0.487385895
ILMN_2327276	STAU1	-0.487547336
ILMN_1715991	SDPR	-0.487560836
ILMN_1792671	C12orf29	-0.487714467
ILMN_2246661	FAHD1	-0.487786692
ILMN_1727309	FAM82A2	-0.487817035
ILMN_3192034	NA	-0.48783753
ILMN_3234570	CECR4	-0.487842607
ILMN_1687711	ZNF576	-0.487846247
ILMN_1766851	TMEM126B	-0.487860967
ILMN_1790603	ATPAF1	-0.487946851
ILMN_1729095	PDZD2	-0.487958154
ILMN_1870747	BX116001	-0.487998165
ILMN_2151056	C10orf32	-0.488297265
ILMN_2128489	UQCRB	-0.48844072
ILMN_1693882	TAPT1	-0.488620355
ILMN_1794951	SLC39A5	-0.488645171
ILMN_1685636	KCNN2	-0.488675995
ILMN_1733419	CCNE1	-0.488757466
ILMN_1815203	HMGCS2	-0.488901136
ILMN_1785650	OR11L1	-0.488902741
ILMN_1751040	RAB23	-0.488999744
ILMN_2229170	MED7	-0.489038428
ILMN_2203147	TMPRSS12	-0.489078475
ILMN_3225432	LAMTOR3	-0.489488112
ILMN_1717357	AIFM1	-0.489503455
ILMN_1711516	ATP6V1A	-0.489546503
ILMN_1661799	HIGD1A	-0.489738091
ILMN_1772677	CNOT4	-0.489932327
ILMN_1792931	LYSMD1	-0.489965956
ILMN_2207988	SERPINI1	-0.490085426
ILMN_1660635	LACTB2	-0.490135051
ILMN_2207720	ITM2B	-0.49015411
ILMN_1723287	SLC6A16	-0.490278746
ILMN_1740265	ACOT7	-0.4902972
ILMN_1724504	SETD3	-0.490368341
ILMN_1798254	ACTR10	-0.490465505
ILMN_1681670	SLC25A4	-0.490549749
ILMN_1671791	PCK2	-0.490731145
ILMN_1759549	SRGAP2	-0.490847514
ILMN_1869343	D19756	-0.490914989
ILMN_2166865	ENY2	-0.491075085
ILMN_3232128	FLJ37786	-0.491257499
ILMN_1718853	UQCRC2	-0.491322489
ILMN_1757807	AGXT2L1	-0.491382787
ILMN_1749405	KIAA1191	-0.491406757
ILMN_1702960	LOC644100	-0.491571559
ILMN_1660793	PAQR4	-0.491646326
ILMN_1763539	IER3IP1	-0.491725172
ILMN_1912130	AK097103	-0.491859958
ILMN_1905829	BC063871	-0.491877672
ILMN_1776674	SAC3D1	-0.491953697
ILMN_1675916	NA	-0.492006535
ILMN_1720998	CA12	-0.492209124
ILMN_3234456	TSTD1	-0.492304589
ILMN_1750861	PML	-0.492368621
ILMN_1748819	MRPL22	-0.49245462
ILMN_1860963	BM715829	-0.492473154
ILMN_2188722	GLS	-0.492562587
ILMN_2104106	XPR1	-0.49257819
ILMN_1916462	DB157938	-0.492589678
ILMN_1764607	UGT1A1	-0.49266701
ILMN_1670606	GABRG1	-0.492813811
ILMN_2136635	ISCA2	-0.492859516

ILMN_1655625	GPATCH1	0.469144675
ILMN_2303166	MBNL3	0.469120518
ILMN_1727051	DDX19A	0.469081682
ILMN_2064655	CXorf40A	0.469049289
ILMN_1712605	ZNF829	0.469043086
ILMN_1782621	RPS12	0.469016198
ILMN_1686725	DICER1	0.468915003
ILMN_1665335	DIABLO	0.468901134
ILMN_3242753	NBPF1	0.468897823
ILMN_2116714	SLC39A1	0.468834857
ILMN_3272374	NA	0.468637205
ILMN_1797005	PGLS	0.468603855
ILMN_3237946	PXDN	0.468548423
ILMN_1704941	PPCDC	0.468499957
ILMN_1675992	DST	0.468445452
ILMN_1654357	ZNF24	0.468424404
ILMN_1748093	PAFAH1B3	0.468231375
ILMN_1770454	AGRN	0.468219425
ILMN_2070052	NPIPL3	0.468136559
ILMN_1800354	CST3	0.468093502
ILMN_1810584	IL1R1	0.468057087
ILMN_3246625	KIAA1045	0.467820614
ILMN_1653797	C6orf62	0.467806575
ILMN_1699887	ST14	0.467800717
ILMN_2160005	NUMA1	0.467797672
ILMN_1802096	ABTB1	0.467675146
ILMN_1765557	OLFML2B	0.467464027
ILMN_2368068	TCF20	0.467246755
ILMN_2148459	B2M	0.467174478
ILMN_1683179	RRAD	0.467171183
ILMN_1679693	TMEM87B	0.466961295
ILMN_3305397	RPL7	0.466740928
ILMN_2321578	P2RY10	0.466713155
ILMN_3304435	LOC728640	0.46660385
ILMN_2173004	RAB8B	0.466551746
ILMN_1673638	RPS3A	0.466487099
ILMN_1798081	PTPRF	0.466429549
ILMN_2176755	KIAA0895L	0.466364225
ILMN_1789775	WDR74	0.466263455
ILMN_1691476	MYLK	0.466182657
ILMN_1787360	GUCA1C	0.466167796
ILMN_1758337	ZNF213	0.465941854
ILMN_1709333	OAS2	0.465861948
ILMN_1686697	FBXO38	0.465846547
ILMN_1704753	EPAS1	0.465821259
ILMN_1729269	LOC285074	0.46580848
ILMN_2316806	RWDD1	0.465781296
ILMN_2306189	MAGED1	0.465693892
ILMN_1756146	WDR45	0.465635123
ILMN_1712887	SLC10A3	0.465608892
ILMN_2400759	CPVL	0.465500641
ILMN_1716687	TPM1	0.465481182
ILMN_2371280	CSF3R	0.46544771
ILMN_3290380	XRCC6	0.465432484
ILMN_2218277	RPS2	0.465425542
ILMN_1700031	PRAME	0.465411366
ILMN_2046856	RPS15	0.465330027
ILMN_1742230	BAZ1A	0.465236618
ILMN_2139970	ALDH1A3	0.465236372
ILMN_1793474	INSIG1	0.465195484
ILMN_2316104	IQCB1	0.465150108
ILMN_1686388	TPM3	0.465077097
ILMN_1716056	LMF2	0.465076951
ILMN_1804789	KIAA1967	0.46505663
ILMN_2408039	EEF1D	0.464950098
ILMN_1813836	DARS	0.46487948
ILMN_1708041	PLEKHF1	0.464836165
ILMN_1675156	CDC42	0.464794422
ILMN_1745686	MFHAS1	0.464770528
ILMN_2192620	DCAF15	0.464723076
ILMN_1807372	ADORA2A	0.464708124
ILMN_1781897	KLRC3	0.464495649
ILMN_2050112	RPSA	0.46446664
ILMN_2260313	CRYBA2	0.464422106
ILMN_1652918	RHOF	0.464363996
ILMN_2147133	NBPF1	0.464317858
ILMN_2200562	LOC595101	0.464314215
ILMN_1714600	NAV3	0.464293779
ILMN_1680424	CTSG	0.464280754
ILMN_1670305	SERPING1	0.464259891
ILMN_1678329	TNRC18	0.464175058
ILMN_1681542	HIST1H4E	0.464173644
ILMN_1770768	SLAMF1	0.464141744
ILMN_1762861	HLA-F	0.463969368
ILMN_1697417	CEP170	0.463776879
ILMN_1806415	TTLL1	0.463759662
ILMN_1675038	PRMT2	0.463745057
ILMN_1713369	RPL13A	0.463686279
ILMN_1695261	RPL10	0.463595221
ILMN_1791726	TUBB3	0.463581398
ILMN_1763091	C14orf43	0.463563476
ILMN_2374340	PLAUR	0.463560037
ILMN_2109536	GOLGA8F	0.463439078
ILMN_1815287	B3GALNT1	0.463412432
ILMN_1785424	ABLIM1	0.463346601
ILMN_2151817	PFN1	0.463196782
ILMN_1683450	CDCA5	0.46315182
ILMN_1742238	SET	0.463107946

ILMN_1676055	AK055272	-0.492974498
ILMN_1782045	FKBP4	-0.492985678
ILMN_3190834	ELMOD3	-0.493178106
ILMN_1737025	PLCL2	-0.49323254
ILMN_3283869	FRG1B	-0.493275132
ILMN_1888558	AI378936	-0.493447734
ILMN_1874814	AB231761	-0.493635358
ILMN_1838137	BF514549	-0.493808722
ILMN_1763269	TNKS2	-0.493819221
ILMN_1887357	AK095715	-0.493829679
ILMN_1813270	ELF5	-0.493968336
ILMN_3238269	NDUFA6	-0.493981829
ILMN_1769449	MBLAC2	-0.494002745
ILMN_1688864	CABP4	-0.494077863
ILMN_1688218	RASGRP4	-0.494396411
ILMN_1735360	SDAD1	-0.494425252
ILMN_3307772	PACSIN2	-0.494876967
ILMN_1676660	DNAH6	-0.494924682
ILMN_3275685	LOC727721	-0.494945733
ILMN_1756550	NUP62CL	-0.495221428
ILMN_1691175	ADH6	-0.495294814
ILMN_1664993	SLX1B-SULT1A4	-0.495383092
ILMN_1811363	NOVA1	-0.495405995
ILMN_1754600	FNBP1L	-0.495567838
ILMN_1697405	PLCL1	-0.496010931
ILMN_2297887	SEZ6L2	-0.496039104
ILMN_1664216	NKIRAS1	-0.496117903
ILMN_1765607	SLC6A2	-0.496143283
ILMN_1729476	PROL1	-0.496148391
ILMN_3309724	MIR509-2	-0.496168751
ILMN_1876729	LOC340544	-0.496320973
ILMN_1694983	DDX20	-0.496419095
ILMN_1815938	TRPM3	-0.496595893
ILMN_1785302	REG1P	-0.496607767
ILMN_1759488	DUSP10	-0.496638981
ILMN_1888492	AA083069	-0.496733356
ILMN_1660732	PPP2R2B	-0.496785533
ILMN_2286800	DNM1L	-0.496809182
ILMN_1751258	NDUFA4	-0.496940865
ILMN_1813055	LOC401093	-0.497180227
ILMN_2224333	C3orf65	-0.497271084
ILMN_3244070	URG7	-0.497285365
ILMN_2223010	VBP1	-0.497369206
ILMN_1784269	AASDH	-0.49754422
ILMN_1744534	LYRM5	-0.497584626
ILMN_1826521	BX106259	-0.498141706
ILMN_1740528	ASB15	-0.498155645
ILMN_1815319	CMTM4	-0.498404435
ILMN_1796165	GLRX5	-0.498413744
ILMN_1715125	HAAO	-0.498442129
ILMN_1813400	CBR4	-0.498774656
ILMN_1807101	F11	-0.499111097
ILMN_1742968	NMNAT2	-0.499114137
ILMN_3229770	SKP1	-0.499172579
ILMN_1669083	TXNRD2	-0.499195538
ILMN_1674648	LOC338588	-0.499318444
ILMN_3310845	MIR511-2	-0.499357279
ILMN_3235196	LOC100129716	-0.49938515
ILMN_1912737	HIPK2	-0.499485158
ILMN_1784818	OPN5	-0.499630574
ILMN_1677292	C5orf30	-0.499931875
ILMN_2372403	ALDH5A1	-0.500078572
ILMN_1839190	BC073918	-0.50028427
ILMN_2195721	KRTAP21-1	-0.500316763
ILMN_2308903	WFDC3	-0.500615848
ILMN_1804662	NRG4	-0.500705838
ILMN_1679782	BLOC1S2	-0.500749121
ILMN_1775171	WIPF2	-0.500810154
ILMN_1729509	C1orf43	-0.500852604
ILMN_2202790	MCFD2	-0.500896404
ILMN_1698733	CNIH2	-0.500941487
ILMN_1871361	AX747287	-0.500994903
ILMN_1683886	BX484633	-0.50109808
ILMN_1732860	FLJ26850	-0.501168237
ILMN_1897796	BQ773461	-0.501478572
ILMN_2389064	C15orf48	-0.501568281
ILMN_3235833	NA	-0.501604922
ILMN_1756723	DPP7	-0.501613329
ILMN_1732096	TRIM77P	-0.501769092
ILMN_1865013	CN291296	-0.501857258
ILMN_1685696	C3orf55	-0.501935686
ILMN_1660043	UBXN11	-0.502028561
ILMN_1889633	BC071821	-0.502047034
ILMN_1697557	NA	-0.502224799
ILMN_1771268	BICD2	-0.502231328
ILMN_1809352	PRL	-0.502331052
ILMN_1750885	LOC644100	-0.502394996
ILMN_1766159	MIER2	-0.502417724
ILMN_1793367	LOC729966	-0.502501192
ILMN_1762531	FGF9	-0.502608578
ILMN_2332440	KCNMB2	-0.50261219
ILMN_1771223	PADI2	-0.502783108
ILMN_2282019	SYN3	-0.502919807
ILMN_1765072	KRT85	-0.502984036
ILMN_3234936	EYS	-0.503320019
ILMN_1744517	GNS	-0.503333434
ILMN_1701507	EHHADH	-0.503481158
ILMN_3239679	SNORD115-15	-0.503525132

ILMN_1740487	CMTM7	0.463103295
ILMN_3310431	MIR874	0.463060114
ILMN_2365307	CD276	0.463036636
ILMN_1795514	C3orf25	0.463019193
ILMN_2347541	NIN	0.462988546
ILMN_1717639	SIK1	0.462973541
ILMN_1676792	RPS11	0.462909236
ILMN_1652185	IL4R	0.46284459
ILMN_2300695	IKZF3	0.462700802
ILMN_1711853	MED24	0.462521232
ILMN_2070375	GPR82	0.462444562
ILMN_3291098	RPL29	0.462409968
ILMN_2041222	FLJ40504	0.462320346
ILMN_1732672	LPAAT-delta	0.46230701
ILMN_1685088	RPL36	0.462248198
ILMN_3239181	ITPRIP	0.462130732
ILMN_1805922	EBPL	0.461995803
ILMN_1812327	RNF19A	0.461991854
ILMN_2190414	ZNF83	0.461907396
ILMN_1805623	SRCIN1	0.461773721
ILMN_1669117	PARP8	0.46176588
ILMN_3278995	RBM39	0.461727074
ILMN_1701613	RARRES3	0.461719307
ILMN_1763884	CORO7	0.461707778
ILMN_1703412	LATS2	0.461635433
ILMN_1732328	RPL22	0.461631877
ILMN_2252309	DPP7	0.461591241
ILMN_1675130	NFIC	0.461476352
ILMN_3241462	RPS18	0.461450247
ILMN_3225761	RPS14	0.461444636
ILMN_3244065	C9orf69	0.461430772
ILMN_1815656	SERINC3	0.461318565
ILMN_1717234	CAST	0.461316582
ILMN_2056087	IGF1	0.461277177
ILMN_3220792	RPL13AP5	0.461258531
ILMN_2343775	RPL38	0.461232084
ILMN_2334210	ITGB4	0.461227232
ILMN_1742808	SF1	0.461151904
ILMN_2315979	LBH	0.461107524
ILMN_1761912	MGAT1	0.461097801
ILMN_2350183	ST5	0.461043011
ILMN_1703593	BAIAP2L1	0.460997445
ILMN_3195401	GABPB1	0.460898485
ILMN_2386530	RPLP1	0.460894509
ILMN_1652082	ELF4	0.460888151
ILMN_1765649	IRF3	0.460879014
ILMN_3212833	RPL7A	0.460828895
ILMN_1757350	CTNNB1	0.460624541
ILMN_1652768	RPS2	0.460478045
ILMN_2355776	GIT2	0.460460238
ILMN_1651950	TPST1	0.460353918
ILMN_1698605	TMEM43	0.46033139
ILMN_1680831	BAZ1B	0.460323466
ILMN_1716876	MED27	0.460314161
ILMN_2188264	CYR61	0.460304717
ILMN_1765833	SLC9A3R2	0.460304566
ILMN_3310326	MIR221	0.460304111
ILMN_2361163	SSBP3	0.460284082
ILMN_1709880	RPLP0	0.460207624
ILMN_2186369	NCOR1	0.460093464
ILMN_1782788	CSDA	0.460078993
ILMN_1660498	RPS27	0.459902519
ILMN_1772605	FRS3	0.459900721
ILMN_1774207	ANGPT2	0.459766366
ILMN_2110167	POLR1E	0.459654191
ILMN_1811195	ZNF211	0.459643581
ILMN_3243700	RPS8	0.459598727
ILMN_2351795	C18orf1	0.459590453
ILMN_2349061	IRF7	0.45958832
ILMN_1691942	CCNI	0.459564313
ILMN_1709101	AKAP13	0.45951683
ILMN_1713249	PHF19	0.459476756
ILMN_2412139	CABYR	0.4591591
ILMN_1764721	RPL8	0.459091628
ILMN_3247893	F8A1	0.459086352
ILMN_1698766	PYCARD	0.459055203
ILMN_1898605	BX119059	0.459011136
ILMN_1714945	ITPKC	0.459003403
ILMN_1697567	TPM3	0.458994636
ILMN_2359945	CES1	0.458979959
ILMN_1804316	TCAP	0.458896126
ILMN_2078334	CNOT10	0.458843159
ILMN_2054233	SENP6	0.458767315
ILMN_3299558	SFRS18	0.458730031
ILMN_2411963	RBM39	0.458597766
ILMN_1680996	ALOX5	0.458593537
ILMN_2211065	TMEM91	0.4585901
ILMN_2286870	CSNK1D	0.458534388
ILMN_1659058	PPP1R10	0.458496397
ILMN_1758983	OTOP1	0.458474609
ILMN_1756705	CHTF18	0.458452406
ILMN_2403994	RALY	0.458416988
ILMN_2407619	CDC25C	0.458327386
ILMN_1790741	RNF126	0.458294463
ILMN_1771057	XAB2	0.45823484
ILMN_1669215	SRPK2	0.458136454
ILMN_1710078	TMEM181	0.458102199
ILMN_1815169	MCM5	0.458022442

ILMN_1761946	PROM2	-0.503555696
ILMN_1715214	PTPN7	-0.503560166
ILMN_3237817	REXO1L1	-0.503632609
ILMN_1687913	ELFN1	-0.503662309
ILMN_1789096	OSTalpha	-0.503669878
ILMN_1692674	NKD1	-0.503718238
ILMN_2128967	C11orf1	-0.503737188
ILMN_1911344	AV650212	-0.504206738
ILMN_3190167	AK098015	-0.504251274
ILMN_3297880	SEC13	-0.504493703
ILMN_1738268	IP6K3	-0.504508001
ILMN_1852279	CK299576	-0.504514836
ILMN_1819939	DR980387	-0.504591517
ILMN_1786936	BM919382	-0.504621457
ILMN_1745887	FBXO21	-0.504646385
ILMN_1655190	LOC100506085	-0.504720484
ILMN_2353129	EYA2	-0.504787763
ILMN_1733390	LARP1B	-0.504800625
ILMN_1736441	PDXP	-0.504809173
ILMN_1724658	BNIP3	-0.504861638
ILMN_1703886	SLC16A2	-0.504887878
ILMN_1687576	EIF4E1B	-0.505213691
ILMN_2136971	FABP3	-0.505288227
ILMN_1853920	BE220010	-0.505497234
ILMN_2073010	TMEM203	-0.505581376
ILMN_2315789	PTPRD	-0.505668113
ILMN_1853824	MGAT3	-0.505867266
ILMN_2404407	ABAT	-0.505972293
ILMN_1764494	ATP5A1	-0.506207932
ILMN_2383229	A1CF	-0.506440289
ILMN_1699357	SLC22A5	-0.50659589
ILMN_2373073	LIG4	-0.506675355
ILMN_3310176	MIR593	-0.506752614
ILMN_1677866	FOLH1B	-0.506754882
ILMN_1760620	TMEM33	-0.50683517
ILMN_1737988	PRNP	-0.507165205
ILMN_1838702	BG188175	-0.507191302
ILMN_1713139	DLGAP2	-0.507370587
ILMN_1720838	DECR1	-0.507691584
ILMN_1675687	NA	-0.507910038
ILMN_1779997	MEIS3	-0.508118875
ILMN_3252969	LOC100128300	-0.508145796
ILMN_1692844	TBC1D19	-0.508185308
ILMN_1808306	SLC26A1	-0.508258079
ILMN_1747962	UTS2R	-0.508337173
ILMN_1736972	GKAP1	-0.508355847
ILMN_1755423	PTCRA	-0.50839223
ILMN_1733135	TFAP2A	-0.508586926
ILMN_1811933	SHMT1	-0.508693259
ILMN_1655348	GPR1	-0.508706522
ILMN_2380561	PKLR	-0.508832718
ILMN_1776112	SLC10A1	-0.508930173
ILMN_1907620	DB299679	-0.509248462
ILMN_3245006	C12orf68	-0.509580572
ILMN_1675445	KIAA2018	-0.510026763
ILMN_1673535	MYLK4	-0.510061044
ILMN_1730351	TEX19	-0.510294664
ILMN_1754913	WDR88	-0.510335484
ILMN_2359287	ITGA6	-0.510506363
ILMN_1910805	BE326664	-0.510634568
ILMN_1671337	SLC2A5	-0.511135467
ILMN_3212469	LOC401561	-0.511203851
ILMN_1750879	TSPY3	-0.511230545
ILMN_1707741	TBC1D26	-0.511296813
ILMN_1752294	PCDH9	-0.511345644
ILMN_1704022	TMEM207	-0.511435344
ILMN_1687824	SEPHS2	-0.511508303
ILMN_1814173	SMARCA4	-0.511740028
ILMN_1853705	AW118645	-0.511781706
ILMN_1702489	TRIM63	-0.512012595
ILMN_1786050	RBBP9	-0.512156365
ILMN_1807095	MRPS36	-0.512169671
ILMN_2331266	NUMB	-0.51227903
ILMN_2133125	LRRC43	-0.512488119
ILMN_1704247	LAMA3	-0.512530561
ILMN_3251491	UQCRB	-0.51260398
ILMN_1814296	TRPM6	-0.512728476
ILMN_2144088	FDFT1	-0.512739479
ILMN_1772057	COL28A1	-0.512864912
ILMN_3207902	GUSBP10	-0.512905482
ILMN_1715463	SOSTDC1	-0.513154285
ILMN_2183610	SERAC1	-0.513248865
ILMN_1752214	BC141517	-0.513370201
ILMN_1705637	ACR	-0.513385788
ILMN_1800488	CT47A1	-0.513720192
ILMN_1778173	AK3	-0.513854365
ILMN_1762033	NAPEPLD	-0.514106867
ILMN_1749317	C1orf222	-0.514114082
ILMN_1785439	CD79B	-0.514138304
ILMN_1794349	XYLB	-0.51425359
ILMN_1687974	ITGA6	-0.514301779
ILMN_1804152	KCNH6	-0.514395182
ILMN_3209098	FAM71E2	-0.514427173
ILMN_1736954	ZBTB7B	-0.514450282
ILMN_1679647	CHIA	-0.514500895
ILMN_1669363	TGM4	-0.514722009
ILMN_1782274	NYX	-0.51475449
ILMN_1880103	BU734093	-0.514811679

ILMN_1747386	GLIS3	0.458015336
ILMN_1747499	EMID1	0.45799925
ILMN_1730660	LGALS3	0.457966858
ILMN_2141453	RPL18A	0.4579375
ILMN_3235358	AB007962	0.457851179
ILMN_1756439	SCRN1	0.457820095
ILMN_1755811	FBXO4	0.457794517
ILMN_2166506	XRCC6	0.457676898
ILMN_2082244	FOXK1	0.457567612
ILMN_1811765	ADAMTS2	0.457472245
ILMN_1815500	ITPR3	0.457408683
ILMN_1750459	ANKRD36	0.457356405
ILMN_2124769	YBX1	0.457305924
ILMN_2190214	CHIC1	0.457200764
ILMN_1740587	RPS11	0.457043372
ILMN_1740466	FAM46A	0.457032748
ILMN_1774823	RPL34	0.457027974
ILMN_1683576	MAGED2	0.457021028
ILMN_1761844	ZCCHC17	0.45686556
ILMN_2390472	TTC14	0.456593733
ILMN_1801767	ABHD3	0.456589212
ILMN_1669703	TNK2	0.456551279
ILMN_1810844	RARRES2	0.456525248
ILMN_1752249	FAM38A	0.45641201
ILMN_1708881	RAB20	0.45638184
ILMN_1762835	HELZ	0.45637095
ILMN_1807467	RGNEF	0.456352172
ILMN_1790689	CRISPLD2	0.456320521
ILMN_1782167	RPL32	0.456037408
ILMN_1808114	LYVE1	0.455884003
ILMN_3222402	FLJ45340	0.455596647
ILMN_1752802	CLPTM1L	0.455592646
ILMN_3304203	CLEC2D	0.455569776
ILMN_1750401	C17orf62	0.455566774
ILMN_1754865	PABPC4	0.455544053
ILMN_1760683	SRSF9	0.455512329
ILMN_1710495	PAPLN	0.455396058
ILMN_1716552	ENAH	0.455332832
ILMN_2403458	SMARCB1	0.455260414
ILMN_1717101	MINK1	0.455231216
ILMN_3281599	RPL3	0.455229163
ILMN_3237368	MAFG	0.455169143
ILMN_2386179	ZMYND8	0.455167454
ILMN_2209748	DERL1	0.455148183
ILMN_1778561	WEE1	0.455142241
ILMN_3238633	SDHAF2	0.45514007
ILMN_3225784	LAMR1P15	0.455105808
ILMN_2400940	L3MBTL1	0.455092074
ILMN_1709471	CNTN4	0.455042631
ILMN_1761939	TIPIN	0.455010296
ILMN_2293131	ARHGEF1	0.454964334
ILMN_1742330	PLXNB1	0.454712793
ILMN_1723358	SCARA3	0.454660442
ILMN_3202432	RPSA	0.454545945
ILMN_1722898	SFRP2	0.454527613
ILMN_2072140	BTF3L4	0.454515068
ILMN_3287219	NA	0.454394549
ILMN_2325194	PHF6	0.454342039
ILMN_2352574	ZNF274	0.454333695
ILMN_1659095	RPL7A	0.454243873
ILMN_2390919	FBLN2	0.453962243
ILMN_2370208	CMTM3	0.453902998
ILMN_1804530	ARPC1B	0.453900765
ILMN_1807493	ACVRL1	0.453889881
ILMN_3233388	RELL1	0.453888401
ILMN_2169261	TNNI2	0.453826047
ILMN_1792679	ITGA5	0.453730692
ILMN_1805158	TAF1A	0.45364869
ILMN_2354391	RAB4B-EGLN2	0.453546915
ILMN_3309849	MIR140	0.453366351
ILMN_2410986	STAT3	0.453245808
ILMN_2202948	BUB1	0.453223566
ILMN_1721035	MS4A6A	0.453172568
ILMN_1664631	ADAM28	0.453147463
ILMN_1713934	LITAF	0.453113233
ILMN_1754199	BCL7B	0.453082335
ILMN_1714965	NFKB1	0.453071023
ILMN_1664063	FAM129C	0.453066622
ILMN_2288582	PCDHA@	0.452997744
ILMN_1713182	C3	0.452927213
ILMN_3242105	LYPD1	0.452759217
ILMN_3217172	RPS2	0.452752332
ILMN_1681503	MCM2	0.452715003
ILMN_1734608	ZNF77	0.452703777
ILMN_1704196	DSG2	0.452690639
ILMN_1676984	DDIT3	0.452662387
ILMN_1740523	KTN1	0.452153171
ILMN_1754195	RPL31	0.45209446
ILMN_1714352	DMWD	0.451937413
ILMN_1680435	NPEPPS	0.4518869
ILMN_2329834	CPNE1	0.451856577
ILMN_1727862	HPS4	0.451679467
ILMN_2130218	PAK3	0.451596701
ILMN_1716062	MYL4	0.451554519
ILMN_1674402	TMEM71	0.45145907
ILMN_1695872	CDC42	0.451363878
ILMN_1775734	SH2D3C	0.451324834
ILMN_1721088	MS4A15	0.451315449

ILMN_1877421	BX092644	-0.514868083
ILMN_1680082	Y08699	-0.514946911
ILMN_3243846	FOLR4	-0.515076024
ILMN_1803454	PCGF3	-0.515393286
ILMN_2183891	PEX5L	-0.515493834
ILMN_1792432	CSPG4P5	-0.515546233
ILMN_1653358	FUT3	-0.51559486
ILMN_2148497	C20orf107	-0.515687731
ILMN_1856533	CDKL2	-0.515754955
ILMN_1688455	KIAA0564	-0.515760304
ILMN_1838238	DA717721	-0.515901685
ILMN_1780861	METRNL	-0.515964956
ILMN_1804569	GAS2	-0.516041405
ILMN_1809139	AHCTF1	-0.516047655
ILMN_2386444	ANGPTL4	-0.516108487
ILMN_1739235	OMG	-0.516202447
ILMN_1804090	SLC25A10	-0.516229726
ILMN_1778951	C6orf203	-0.516335007
ILMN_1904400	AW136452	-0.51649313
ILMN_1662413	HGSNAT	-0.516611445
ILMN_2323801	MOCS1	-0.516629579
ILMN_1700810	HSP90AA1	-0.516630997
ILMN_2158587	EEF1E1	-0.516785613
ILMN_3245287	SCN5A	-0.516893107
ILMN_3246169	PRR20A	-0.516980538
ILMN_1679209	HSPA9	-0.517134807
ILMN_3307025	ZDHHC4	-0.517198247
ILMN_1697353	SIRPB2	-0.517212459
ILMN_3241149	TMEM236	-0.517326934
ILMN_1752952	MMP28	-0.517357633
ILMN_2149494	NPL	-0.517381667
ILMN_1693932	LOC285733	-0.517443074
ILMN_3244449	SNORD9	-0.517474095
ILMN_2085922	WRB	-0.517617115
ILMN_2146372	KCTD6	-0.517665062
ILMN_1739155	OR9A4	-0.517794518
ILMN_3259884	SLC6A19	-0.517852299
ILMN_1685805	FLJ25758	-0.517939203
ILMN_1749032	CREB3L1	-0.518131877
ILMN_1687315	RXRA	-0.518304332
ILMN_3184048	FAM115C	-0.518494964
ILMN_3245084	AK127966	-0.518640285
ILMN_2195914	GGH	-0.518653758
ILMN_1668463	SON	-0.518783942
ILMN_1685781	C14orf142	-0.518848938
ILMN_2341626	MARCH8	-0.51890008
ILMN_2381397	HSPD1	-0.519105994
ILMN_1889767	NA	-0.519133595
ILMN_1752557	NEU2	-0.519343522
ILMN_2211189	IKBKAP	-0.519346455
ILMN_1797154	AZGP1	-0.519401517
ILMN_1731326	PRR23C	-0.519563632
ILMN_2369104	TRAPPC6B	-0.519696486
ILMN_2120360	LOC729164	-0.519777819
ILMN_1778253	SRC	-0.520138353
ILMN_1765417	RBBP9	-0.520252826
ILMN_2215355	AK126539	-0.520298379
ILMN_2177732	RANBP6	-0.520347294
ILMN_1678217	DMRTC1B	-0.520372543
ILMN_1874018	LOC729175	-0.520486507
ILMN_1803824	ZDHHC9	-0.521031001
ILMN_1686951	TM6SF2	-0.521118573
ILMN_2205767	CTXN3	-0.52114872
ILMN_1740949	LRRC19	-0.521160758
ILMN_2380754	CCNG1	-0.521574504
ILMN_2175020	RPL32P3	-0.521678436
ILMN_1663723	SLC13A4	-0.521752141
ILMN_2181064	GBA3	-0.521898466
ILMN_1786343	MUC5B	-0.521918333
ILMN_1681892	OAZ3	-0.521989156
ILMN_1797310	ATP6V1D	-0.522311281
ILMN_1905488	CR738423	-0.522326041
ILMN_1768877	CCDC7	-0.522390203
ILMN_2355463	CYFIP1	-0.522525587
ILMN_1813434	ATP10A	-0.522658766
ILMN_1765438	C6orf184	-0.522887536
ILMN_1764500	C3orf10	-0.522959513
ILMN_1697820	HINT2	-0.523044941
ILMN_1677384	PTGER3	-0.523183544
ILMN_1795270	MYCL1	-0.523308175
ILMN_1766103	KCNK18	-0.523316736
ILMN_2383455	SUOX	-0.523417062
ILMN_1671215	FBXL19	-0.52366558
ILMN_1689200	DHDH	-0.523803741
ILMN_1730209	OR5A1	-0.523856155
ILMN_1714446	PLG	-0.523944958
ILMN_1757165	LMBR1L	-0.524102052
ILMN_1717935	SPTA1	-0.524396463
ILMN_1696699	FAM184A	-0.52440729
ILMN_1767857	TCP10L2	-0.524678266
ILMN_2228180	MSRA	-0.524773489
ILMN_1899387	BC039440	-0.52485151
ILMN_1882652	BG697942	-0.524859177
ILMN_1697118	ARMC6	-0.524922616
ILMN_3244200	FLJ00326	-0.525393238
ILMN_1769433	IQGAP2	-0.525487026
ILMN_2136926	PPY2	-0.525546117
ILMN_1743357	XPNPEP2	-0.52561451

ILMN_3234997	MAP7D1	0.451262206
ILMN_2348243	STRADA	0.451205842
ILMN_1705594	NAT10	0.451196855
ILMN_1717324	NR_024104	0.451153386
ILMN_3241491	NA	0.45108691
ILMN_1844408	PLXNA2	0.451060115
ILMN_1788886	TOX	0.451018036
ILMN_1782331	TDG	0.451008464
ILMN_2041780	SNTG2	0.450976087
ILMN_3238560	IFI27L2	0.450857376
ILMN_1797813	SUZ12	0.450856446
ILMN_1652797	FAM174B	0.450827583
ILMN_3302356	RPS14	0.45068858
ILMN_1780825	RRAS	0.450549552
ILMN_1723115	CLEC4F	0.450517603
ILMN_1760143	ADRM1	0.450449874
ILMN_2054145	PAK1IP1	0.450434438
ILMN_1666633	BC010030	0.450353744
ILMN_2353697	HINFP	0.450332812
ILMN_1783606	MLL5	0.450215896
ILMN_1785202	STAT4	0.450208557
ILMN_2375651	SCNM1	0.450185331
ILMN_1814213	PQLC3	0.450116862
ILMN_3243664	NPIPL3	0.450100439
ILMN_2272074	TROVE2	0.450095038
ILMN_2318811	RANBP3	0.45007834
ILMN_2397024	SPOP	0.4499268
ILMN_1686664	MT2A	0.449912477
ILMN_1653469	RPL12	0.449821287
ILMN_3268914	CLEC2D	0.449719824
ILMN_1706645	MB21D1	0.449715235
ILMN_1702708	PPP2R3A	0.449700627
ILMN_1753353	SLBP	0.449485162
ILMN_1668535	JOSD1	0.449402047
ILMN_1728224	OGFR	0.449345766
ILMN_1688938	KCNRG	0.449288469
ILMN_1757627	ZMYND19	0.449276631
ILMN_1676765	RPL18A	0.44926599
ILMN_2066060	HLA-DRB6	0.449254111
ILMN_1726064	PAK1IP1	0.449243504
ILMN_3239060	KRBA1	0.449215514
ILMN_1673023	EP400	0.449195924
ILMN_2041577	GPR172A	0.4491461
ILMN_1695404	LY6E	0.44907354
ILMN_1769779	PTP4A3	0.449049784
ILMN_1777444	STX5	0.449042902
ILMN_1681415	SIGLEC7	0.449004907
ILMN_3253901	HAVCR1	0.448970223
ILMN_1661647	LOC349114	0.448844574
ILMN_3226467	AK023375	0.448828688
ILMN_1691611	HNRNPA1L2	0.448776653
ILMN_1679727	CLK1	0.44866923
ILMN_1711909	EDEM2	0.448644553
ILMN_2377496	ERCC1	0.448495252
ILMN_1797566	SNAP47	0.448490352
ILMN_1792681	CCDC86	0.448458781
ILMN_2116366	RPL12	0.448323302
ILMN_1666635	RPS14	0.448322896
ILMN_1666169	NBR2	0.44831965
ILMN_1726222	FLOT2	0.448298233
ILMN_2174341	GPR83	0.448103924
ILMN_1753468	CD63	0.448076982
ILMN_1689932	WDR85	0.44807457
ILMN_1770742	TMEM55B	0.447957298
ILMN_2244108	ITGB1	0.447946666
ILMN_3265343	LOC100506144	0.447843321
ILMN_3250389	GPAA1	0.447828016
ILMN_1688279	PVRIG	0.447814035
ILMN_1703565	GLTSCR2	0.447796417
ILMN_1779190	ALKBH8	0.447747574
ILMN_1755658	ABI3	0.44757417
ILMN_1654324	HEYL	0.447545097
ILMN_1762316	CPSF3L	0.447500149
ILMN_1778111	FLJ45340	0.447414897
ILMN_1678235	KIAA1267	0.447408591
ILMN_2393254	CAPNS1	0.447369863
ILMN_1667476	LTBR	0.447368787
ILMN_1728049	S100A16	0.447354028
ILMN_1783806	DTNBP1	0.447298964
ILMN_2357809	G3BP1	0.447275208
ILMN_1769329	GOLGA6	0.447130448
ILMN_1727271	WARS	0.447090957
ILMN_1803846	EIF1	0.446886529
ILMN_2077905	PTGFRN	0.446878305
ILMN_2112301	DRAP1	0.446867542
ILMN_1756542	TBC1D17	0.44667715
ILMN_1781810	NFE2L3	0.44663234
ILMN_1659771	RPL13AP3	0.446629337
ILMN_2062524	RBBP4	0.446593908
ILMN_1730797	RUNX1	0.446559507
ILMN_2351298	WIPF1	0.446350575
ILMN_3198464	BC053580	0.446204934
ILMN_1735180	NCSTN	0.446189674
ILMN_1754839	DHX15	0.446140419
ILMN_1732725	PPP6R3	0.446139203
ILMN_1663132	ADCK2	0.446134057
ILMN_1681160	CCT6P1	0.446086097
ILMN_1752953	BCL2L12	0.446061554

ILMN_1770692	WDR12	-0.525692812
ILMN_1764207	RPRD1A	-0.525750873
ILMN_2145396	AKR7A3	-0.525902866
ILMN_3245038	AQP7	-0.526290941
ILMN_1778242	CALM1	-0.526334897
ILMN_1684919	ADAMTSL1	-0.526380749
ILMN_2226628	ZNF184	-0.526471014
ILMN_2167011	ECHDC1	-0.526494888
ILMN_1855386	CR622974	-0.526498186
ILMN_1741204	KLHDC2	-0.52649952
ILMN_1870085	DB279316	-0.526766571
ILMN_1695290	FERMT2	-0.527068055
ILMN_3249756	DNASE1L1	-0.527390238
ILMN_1677723	ANGPT1	-0.527429853
ILMN_1893948	BX109720	-0.527493019
ILMN_1735192	COLEC11	-0.52760065
ILMN_3239073	AY343892	-0.527653051
ILMN_2163796	RABGGTB	-0.527666892
ILMN_1764609	PWWP2B	-0.527707985
ILMN_1738849	SLC9A2	-0.527817289
ILMN_1763202	LOC441179	-0.527902656
ILMN_1771732	C10orf32-AS3MT	-0.527944663
ILMN_1791306	C9orf103	-0.528003081
ILMN_1819515	AF009300	-0.52818435
ILMN_2379931	PIGO	-0.528240346
ILMN_1796136	RAB37	-0.528429348
ILMN_1837271	CA449813	-0.528434192
ILMN_2289576	VWA1	-0.528447683
ILMN_1668507	DDAH1	-0.528519104
ILMN_1735552	KIF1B	-0.528843509
ILMN_1753026	LPPR1	-0.528963333
ILMN_1738346	EFHA1	-0.529123938
ILMN_1786172	GTF2H2	-0.52914079
ILMN_2326324	LDHC	-0.529191283
ILMN_1681154	ZBTB39	-0.52925974
ILMN_1720235	ADSSL1	-0.529377624
ILMN_1697550	ACYP2	-0.529467745
ILMN_1800935	ARV1	-0.52948337
ILMN_1656492	NA	-0.529721452
ILMN_3274671	LOC283481	-0.529906515
ILMN_3238359	NAV3	-0.53031015
ILMN_2184556	SLC4A4	-0.530418995
ILMN_1745988	NUDT12	-0.530561329
ILMN_1776263	TMEM174	-0.530709472
ILMN_1656672	MYLK	-0.530930463
ILMN_1775175	RPL3L	-0.531010155
ILMN_2282352	PHYH	-0.531011426
ILMN_3241066	FAM90A7	-0.531094196
ILMN_1692477	MGAT5B	-0.531174179
ILMN_1762134	ECHDC1	-0.531384733
ILMN_2347888	LARP4	-0.531421623
ILMN_3187758	SELK	-0.531481525
ILMN_1698731	SEC31B	-0.531772106
ILMN_1716591	SPRR1A	-0.531791322
ILMN_1806330	C17orf78	-0.531865063
ILMN_1808584	FAM36A	-0.531935391
ILMN_1712836	C6orf27	-0.532007312
ILMN_1775814	GHR	-0.532190939
ILMN_1831176	BG208291	-0.532228289
ILMN_1780924	SLC43A1	-0.532524345
ILMN_1667018	ACE2	-0.532784987
ILMN_2406586	CC2D2A	-0.532971626
ILMN_1756162	EXOSC8	-0.532995794
ILMN_1859905	BM932227	-0.533101596
ILMN_3254046	BI599339	-0.533133917
ILMN_2343010	BOLA3	-0.533559245
ILMN_1748650	MRPL45	-0.533597335
ILMN_3238299	NHEDC1	-0.533661583
ILMN_2183885	C7orf11	-0.533824808
ILMN_3307252	STMN1	-0.534020662
ILMN_1681221	C9orf100	-0.534027857
ILMN_1763461	ALDH7A1	-0.53438585
ILMN_1712405	LOC100506085	-0.53449266
ILMN_1904378	BX104799	-0.534579135
ILMN_1657349	EPHA6	-0.534852751
ILMN_2364305	FECH	-0.534944557
ILMN_1669781	PRLR	-0.535165674
ILMN_1755047	LRRC2	-0.535259457
ILMN_1820572	BX112399	-0.535497646
ILMN_1904578	ODZ1	-0.535609623
ILMN_1793072	tMDC II	-0.535885453
ILMN_1658917	SLC1A1	-0.535931802
ILMN_1705066	BTBD11	-0.535935482
ILMN_1762631	TSC2	-0.535969948
ILMN_3298348	LINC00273	-0.535978642
ILMN_1814770	CPNE4	-0.536217397
ILMN_1658800	BRPF3	-0.536451813
ILMN_1770911	NRSN2	-0.536619549
ILMN_3232806	HIGD1A	-0.536715038
ILMN_1672693	IGSF11	-0.536856645
ILMN_1665775	MOSC2	-0.537325568
ILMN_1728975	SCO1	-0.537326825
ILMN_2067682	LOC401431	-0.537384697
ILMN_1705225	PRTG	-0.537502154
ILMN_1819225	LOC100506368	-0.537886225
ILMN_2064898	CCDC56	-0.538002776
ILMN_3261449	LOC100130815	-0.53848543
ILMN_3192801	NA	-0.538583716

ILMN_1762801	KLF12	0.445941449
ILMN_1808500	CEP68	0.445938563
ILMN_2337923	TPD52L1	0.445789782
ILMN_1807234	ZNF700	0.445720791
ILMN_2358652	NXF1	0.44564709
ILMN_1779828	EDEM1	0.44563996
ILMN_1790891	CKAP4	0.445543014
ILMN_2407589	CTDP1	0.445528548
ILMN_3276019	RPL23AP7	0.445517116
ILMN_1800451	MED16	0.445503687
ILMN_1753534	RPS18	0.445483461
ILMN_1706434	CSDAP1	0.445445888
ILMN_2396287	RFX2	0.445416343
ILMN_3302919	MYOF	0.445406739
ILMN_1724581	EDC3	0.445361033
ILMN_2331087	MS4A7	0.445317122
ILMN_2188533	RPL12	0.445311071
ILMN_3211199	ZNF316	0.445307619
ILMN_1737586	EIF4H	0.445294865
ILMN_1796229	FGFR1	0.445259056
ILMN_1657750	CNNM1	0.445193596
ILMN_1732705	HCFC1	0.445102441
ILMN_1716973	POLM	0.445082064
ILMN_3247325	PP13759	0.444981661
ILMN_3235096	SNORA28	0.444960147
ILMN_2294784	PRDM1	0.444955218
ILMN_1690464	TMEM20	0.444814407
ILMN_1749345	STX5	0.444801406
ILMN_2388070	TMEM44	0.444799547
ILMN_2056551	RBMX2	0.444737082
ILMN_1723709	C9orf116	0.444634122
ILMN_1740395	RAVER1	0.444612397
ILMN_2371911	MUC1	0.444565986
ILMN_1667432	HYAL3	0.444524804
ILMN_1684258	RPS28	0.444474075
ILMN_2211003	GAS2L3	0.444417969
ILMN_1663313	AMY1A	0.444409425
ILMN_1713086	RPL27A	0.444336027
ILMN_2304020	GRXCR2	0.444290607
ILMN_1775743	BTG1	0.444269769
ILMN_3201663	RPL22	0.444258191
ILMN_1801383	SMG1	0.444220046
ILMN_1872122	LCOR	0.444133794
ILMN_1694398	EIF3F	0.444128372
ILMN_1804279	PTPRC	0.444121772
ILMN_1773079	COL3A1	0.443958528
ILMN_1780315	PUSL1	0.443928897
ILMN_1736597	TKT	0.443811163
ILMN_2342835	P2RY14	0.443785743
ILMN_1802251	PTTG1IP	0.443758713
ILMN_2326737	PPIE	0.443728586
ILMN_3247797	abParts	0.443688699
ILMN_3246766	NPIPL3	0.443631858
ILMN_1770963	NAALADL1	0.443538817
ILMN_1747460	TMEM184B	0.44353101
ILMN_1706992	ADAMTSL1	0.443477637
ILMN_2203588	MYL5	0.443248189
ILMN_1669772	LRP1	0.443216306
ILMN_1815745	SOX4	0.443069787
ILMN_1708743	NT5DC2	0.443060534
ILMN_1665107	ITGB1BP1	0.443051536
ILMN_1744822	BECN1	0.442981595
ILMN_2319344	APEX1	0.442963805
ILMN_1781752	CLEC16A	0.442787825
ILMN_1672081	NEURL	0.442760296
ILMN_2354673	PAK4	0.442524944
ILMN_2401155	PUM1	0.44252448
ILMN_2410909	AKT1	0.442497425
ILMN_1698159	ANKRD6	0.442387216
ILMN_1724598	RABL2A	0.442386477
ILMN_1760714	RPS3	0.442263578
ILMN_1728845	SMARCD1	0.442226584
ILMN_1661589	CD151	0.442179675
ILMN_1740319	IFI27L2	0.442164014
ILMN_1729408	C7orf28B	0.442143418
ILMN_1709809	NHP2L1	0.442111759
ILMN_1738237	HS1BP3	0.441985862
ILMN_2401714	MS4A1	0.441976782
ILMN_2287157	BPAG1	0.441941983
ILMN_1682775	EDN1	0.441840112
ILMN_1681998	AP2B1	0.441819304
ILMN_2352042	MRPL4	0.441781632
ILMN_1806023	JUN	0.441666005
ILMN_1757439	RBM14	0.441652846
ILMN_1779109	AGPAT1	0.441628701
ILMN_1757827	VOPP1	0.441589766
ILMN_2312709	LCMT1	0.44152176
ILMN_1670638	PITPNC1	0.441519568
ILMN_2403247	CMTM7	0.441510553
ILMN_1652166	GUCY1B2	0.441470903
ILMN_2095759	OGFRL1	0.441469741
ILMN_1694571	SPDYE7P	0.441456687
ILMN_3178471	ESPNL	0.44133197
ILMN_2186061	PFKFB3	0.44129708
ILMN_1745992	FAM203A	0.441290727
ILMN_1724990	C3orf75	0.44122011
ILMN_2356786	ADD1	0.440987661
ILMN_2411076	MATR3	0.440767037

ILMN_1687508	ALDH7A1	-0.53861205
ILMN_2216815	MAP7	-0.538736807
ILMN_1769721	SP6	-0.538766055
ILMN_3234735	ERI2	-0.5387683
ILMN_1779673	HDGFL1	-0.538773018
ILMN_1886117	AI187771	-0.538847947
ILMN_1775442	SPDYE7P	-0.538962737
ILMN_3246345	UCRP	-0.539329545
ILMN_1785284	ALDH6A1	-0.539367889
ILMN_1762605	SERPINC1	-0.539476815
ILMN_3273185	AK311113	-0.539499476
ILMN_1844882	BQ024020	-0.539578779
ILMN_1777366	TTC35	-0.539754379
ILMN_2218104	PAH	-0.539769584
ILMN_3241783	FLJ00326	-0.540080258
ILMN_2183216	TDP2	-0.540341644
ILMN_1787591	XPA	-0.540371524
ILMN_1656285	METTL7A	-0.540388451
ILMN_1916137	BF513551	-0.540600349
ILMN_1719083	OR56A3	-0.540737267
ILMN_3179275	H03055	-0.541055978
ILMN_1797925	MOCS2	-0.541074915
ILMN_1777797	AFM	-0.541093545
ILMN_1797281	RGPD6	-0.541182387
ILMN_2219767	MYCN	-0.541364186
ILMN_1736533	RND2	-0.541373319
ILMN_1725157	NEK6	-0.541804926
ILMN_1711245	SCG3	-0.541853722
ILMN_1781242	SOST	-0.541907144
ILMN_1740076	SLC16A12	-0.542099163
ILMN_2154101	UPRT	-0.542168355
ILMN_1746664	WSB2	-0.542190398
ILMN_1769150	THAP5	-0.54228159
ILMN_1676248	SEMA4G	-0.542820602
ILMN_3236437	PCAN1	-0.543033726
ILMN_2296510	SLC5A12	-0.543113336
ILMN_2173740	ASB8	-0.543180105
ILMN_3237529	DEFB108B	-0.543328327
ILMN_1892506	CACNA1C	-0.543445122
ILMN_3245331	DHRS4L1	-0.543603027
ILMN_1746945	CSMD1	-0.543670605
ILMN_1817816	BC032017	-0.543678273
ILMN_1801378	COQ3	-0.543689498
ILMN_2108493	RHOF	-0.543707812
ILMN_1708660	RWDD4	-0.543818504
ILMN_1721011	LCN1	-0.544125823
ILMN_1786444	LPL	-0.544561707
ILMN_1770853	CHST5	-0.545087916
ILMN_1799128	SLC30A9	-0.545157432
ILMN_1802027	MGST2	-0.545369097
ILMN_1701293	COX7A2	-0.545480406
ILMN_1663640	MAOA	-0.545548392
ILMN_1803218	GPR139	-0.545553832
ILMN_1702568	FAM125B	-0.545748858
ILMN_1813374	C19orf28	-0.54576594
ILMN_1803211	FBXO2	-0.545908623
ILMN_1762993	PIGV	-0.54593742
ILMN_1745991	GNRH1	-0.546138577
ILMN_1893033	DB307015	-0.54640171
ILMN_3264466	FAM54B	-0.546688574
ILMN_1701497	CD5L	-0.546701974
ILMN_2352633	ARHGAP24	-0.546898917
ILMN_1681123	KIAA0562	-0.546937047
ILMN_3243788	TMEM146	-0.546982262
ILMN_1840036	BM672267	-0.547017824
ILMN_1718079	HOXB1	-0.547144388
ILMN_2099798	LYPLA1	-0.547279687
ILMN_3296096	SLC9A7P1	-0.547687232
ILMN_1696318	LOC100130691	-0.54782038
ILMN_1682763	ALB	-0.54811069
ILMN_3238143	D2HGDH	-0.548932305
ILMN_1781980	CFC1B	-0.548937221
ILMN_1662081	KRTAP3-2	-0.548970507
ILMN_2115421	SLC25A16	-0.549106052
ILMN_1685974	SLC25A33	-0.549215452
ILMN_1746788	TRIM10	-0.549229945
ILMN_2339779	ATP6V1E1	-0.549360649
ILMN_1902209	BU743228	-0.549379216
ILMN_2226324	BRP44L	-0.549446296
ILMN_2194467	SGCB	-0.549648683
ILMN_1754423	C3orf23	-0.550346849
ILMN_2352917	LTBP4	-0.550669887
ILMN_3302625	DA626616	-0.550799273
ILMN_1774028	MTFR1	-0.550816676
ILMN_2143220	ANGPTL3	-0.550923395
ILMN_2375879	VEGFA	-0.550954072
ILMN_1757504	REG3A	-0.550957418
ILMN_1683903	PDPK1	-0.550991105
ILMN_2228780	SLC5A11	-0.550993879
ILMN_1659214	CTXN3	-0.551090942
ILMN_2134039	ACN9	-0.551346829
ILMN_1728803	HMP19	-0.55157368
ILMN_2373515	HSP90AA1	-0.551724529
ILMN_3276971	DVL1	-0.551893951
ILMN_1652128	LMBRD1	-0.552027522
ILMN_1749081	AUTS2	-0.552080282
ILMN_1677962	GPHN	-0.552189823
ILMN_1727813	BRP44	-0.552442047

ILMN_2198893	MAP2K2	0.440753859
ILMN_2307656	AGTRAP	0.440742558
ILMN_2319994	RPL3	0.440723986
ILMN_1742981	TUBA1A	0.440704045
ILMN_1910180	AK024680	0.440686781
ILMN_2394027	CLK3	0.440384975
ILMN_3272768	HSPC157	0.440357494
ILMN_1788019	LAMA2	0.440354805
ILMN_2044471	NCR3	0.440233969
ILMN_2296950	APOBEC3F	0.440147559
ILMN_1712636	NVL	0.440027379
ILMN_1715131	CCR7	0.440022458
ILMN_1758798	NPIPL2	0.43997324
ILMN_1741259	RPS2	0.439900787
ILMN_3251404	NUCKS1	0.439817826
ILMN_1742923	ZNF584	0.439725921
ILMN_1703041	IDUA	0.439644113
ILMN_1773247	ZNF410	0.439619329
ILMN_2337492	RCE1	0.439561788
ILMN_1678831	GCC2	0.439539153
ILMN_1806937	BABAM1	0.43946141
ILMN_1661695	IRAK3	0.439436439
ILMN_1690561	GZMM	0.439360197
ILMN_1705301	TEAD4	0.439327991
ILMN_1731287	ARFGAP3	0.439237345
ILMN_1697024	STYX	0.439073528
ILMN_1666976	PLD3	0.43906753
ILMN_1756982	CLIC1	0.439011307
ILMN_1727809	STK35	0.438945902
ILMN_1763436	SETX	0.438910946
ILMN_3188110	C19orf60	0.438860125
ILMN_1709851	TCEA1	0.438809449
ILMN_1710738	RC3H2	0.438778357
ILMN_1661309	XKR4	0.438724162
ILMN_1651262	HNRNPAB	0.438638534
ILMN_1783226	SSR2	0.438614591
ILMN_1684271	ACBD6	0.438564354
ILMN_2143795	NCRNA00152	0.438539324
ILMN_1687805	RPS12	0.438536206
ILMN_3278548	RPL7	0.438477083
ILMN_2170813	LAMP3	0.438456337
ILMN_1789909	TBC1D9B	0.438401891
ILMN_1746257	DAZAP1	0.438396615
ILMN_1749070	HLA-DPB1	0.438265341
ILMN_2378654	B3GALT5	0.438170164
ILMN_1675092	VLDLR	0.438115105
ILMN_1744665	EP300	0.438108108
ILMN_1666111	YTHDC1	0.437960882
ILMN_1750373	KAL1	0.437924096
ILMN_1656720	PTPN11	0.437829648
ILMN_1660663	DYRK1A	0.437659546
ILMN_1700727	KLF6	0.437583206
ILMN_1706935	CCDC136	0.437568793
ILMN_2255579	RAB37	0.437568017
ILMN_1676385	PAK2	0.437545748
ILMN_2338785	RPS14	0.437541057
ILMN_2343618	SAMD3	0.437514516
ILMN_1719641	SMOC2	0.437357824
ILMN_3241051	RPL32	0.437285546
ILMN_2403965	TXNDC5	0.437242243
ILMN_2377150	WDHD1	0.437233028
ILMN_1756920	ADAM15	0.437167031
ILMN_1688848	TMEM44	0.437129062
ILMN_1781276	TMCO4	0.437123259
ILMN_1715203	DNAH7	0.437032911
ILMN_1782560	CD86	0.437001422
ILMN_2296776	PTPRA	0.436996461
ILMN_3245403	RPL17	0.436750022
ILMN_1803739	RPGRIP1	0.436679509
ILMN_1658261	MAPKBP1	0.43666893
ILMN_1671291	EIF3I	0.436649361
ILMN_2361862	VLDLR	0.43663067
ILMN_3200830	CCT6P3	0.436594794
ILMN_1669523	FOS	0.436533863
ILMN_1702636	TUBB6	0.436472465
ILMN_1815682	C3orf37	0.436433388
ILMN_1739199	WASH2P	0.436418022
ILMN_3247223	TPBG	0.436404394
ILMN_1721022	SHC1	0.436390444
ILMN_1686097	TOP2A	0.43638848
ILMN_1792528	RPS25	0.436333476
ILMN_1790909	NFE2L2	0.436308912
ILMN_2219134	RPS15	0.436300076
ILMN_1661306	RPL29	0.43617973
ILMN_1755415	SAMD3	0.436173661
ILMN_1788250	LDOC1	0.436149373
ILMN_1847308	FAM168A	0.436139833
ILMN_1769191	GNAS	0.435990046
ILMN_1749362	CD28	0.435928943
ILMN_1727041	EWSR1	0.435853016
ILMN_1786328	DCAF12	0.435692854
ILMN_3241446	ODF3B	0.43563952
ILMN_1749447	RPS9	0.435522063
ILMN_1815261	PDIA4	0.43525957
ILMN_1813704	KIAA1199	0.435052352
ILMN_3244323	hCG_20425	0.435036848
ILMN_1731184	MELK	0.435034043
ILMN_1778321	SLC2A6	0.434978267

ILMN_3307954	L3MBTL4	-0.552558557
ILMN_1779633	PSMD6	-0.552629026
ILMN_2199676	CEP152	-0.552682518
ILMN_1859665	AW629225	-0.552695515
ILMN_2207562	AP1AR	-0.552754274
ILMN_3283960	BC046381	-0.552797991
ILMN_1908274	AW236020	-0.553535285
ILMN_1805147	ACSM5	-0.553621205
ILMN_2267131	MAGEA11	-0.553793353
ILMN_1681884	LOC100506049	-0.553849886
ILMN_1699011	GTDC1	-0.553961748
ILMN_1789653	PBLD	-0.554030314
ILMN_1726198	CTSL3	-0.554138601
ILMN_3228470	NACAD	-0.554285186
ILMN_1915734	DA722162	-0.554321924
ILMN_2161832	VPS37A	-0.554375353
ILMN_1677197	RARA	-0.554568556
ILMN_1758034	ETFDH	-0.554598268
ILMN_1695193	ANGPTL3	-0.554634768
ILMN_2283196	ZSWIM3	-0.554713922
ILMN_1664071	TNNT2	-0.554717531
ILMN_1744295	CDKN2A	-0.555026031
ILMN_1733333	CALML3	-0.5552724
ILMN_1690327	CLCN5	-0.555640746
ILMN_1901774	BF513180	-0.555713615
ILMN_1686906	TP53INP2	-0.555893959
ILMN_1745817	NELL1	-0.555947508
ILMN_2172174	PNP	-0.555980855
ILMN_2313434	TCP1	-0.556179767
ILMN_1741695	COL12A1	-0.556231988
ILMN_1712298	ANKRD46	-0.556550242
ILMN_2379560	CDC14B	-0.556701972
ILMN_1687437	CCDC57	-0.557071035
ILMN_1905546	AK055145	-0.557188201
ILMN_2141259	SPAG5	-0.557205137
ILMN_1831907	AI935777	-0.557244497
ILMN_1882485	CF552129	-0.557291735
ILMN_1787830	BASE	-0.557408852
ILMN_1763749	GUCA2A	-0.557459136
ILMN_1855966	DB338170	-0.558009544
ILMN_1666966	IGF2	-0.558516768
ILMN_1752646	NA	-0.558561984
ILMN_2292646	GAD1	-0.558698733
ILMN_1831258	AK094576	-0.558752574
ILMN_1710124	CMTM8	-0.558761704
ILMN_1741431	AOC2	-0.559091333
ILMN_2355033	KIAA1147	-0.559104308
ILMN_2131811	CLPS	-0.559104551
ILMN_1656257	DKFZp434O0320	-0.559390343
ILMN_2183772	CCDC121	-0.559431534
ILMN_1774091	FECH	-0.559675342
ILMN_1701512	KIAA0391	-0.55984236
ILMN_2233552	POLD3	-0.55999496
ILMN_3241607	FLJ00326	-0.560188167
ILMN_2103774	PIP5KL1	-0.560198512
ILMN_1671051	CD276	-0.560204024
ILMN_1713219	RGS12	-0.560950865
ILMN_1804150	HIBADH	-0.560951746
ILMN_1699684	PBLD	-0.561005416
ILMN_1770172	ZNF669	-0.561078346
ILMN_1813256	CRIPT	-0.561351322
ILMN_1785821	TDP2	-0.561539162
ILMN_1848529	AY235716	-0.561978959
ILMN_2059294	RTCD1	-0.562131782
ILMN_1717757	CALML4	-0.562189019
ILMN_1693270	SUSD2	-0.562442027
ILMN_1791588	S71824	-0.562714579
ILMN_1681601	SUCNR1	-0.5627991
ILMN_1654402	IL18BP	-0.5628914
ILMN_1722849	UNC13A	-0.562923593
ILMN_1698832	AW410744	-0.562940772
ILMN_1882015	BG951912	-0.563053232
ILMN_2166543	DNAJC6	-0.563201663
ILMN_1819991	AI792396	-0.563275393
ILMN_1695461	PTPRA	-0.563411263
ILMN_3261022	NA	-0.563632812
ILMN_1781104	MAP2K7	-0.564402786
ILMN_2199439	CA2	-0.564821895
ILMN_1761804	ALDH9A1	-0.564886886
ILMN_1677043	AKR7A2	-0.565099509
ILMN_1735996	NOX4	-0.565673777
ILMN_3249963	PCBP2	-0.565719212
ILMN_2259949	BCKDHB	-0.565763103
ILMN_1729965	CXorf66	-0.5658798
ILMN_3251497	GSTA1	-0.566046487
ILMN_1669103	AK021896	-0.56607682
ILMN_3294965	RRAS2	-0.566160978
ILMN_2069224	PVALB	-0.566234664
ILMN_1712786	AHCYL2	-0.566534571
ILMN_1699334	AATK	-0.566711869
ILMN_1738058	CPNE6	-0.567531379
ILMN_1860789	BM974067	-0.567688597
ILMN_1795704	KIAA0232	-0.567775564
ILMN_1712231	SH3YL1	-0.567977311
ILMN_1672354	AX721218	-0.568260913
ILMN_1807448	FAM8A1	-0.5682898
ILMN_3310281	MIRLET7E	-0.568349261
ILMN_1777031	PKLR	-0.56864997

ILMN_1728262	SAA2	0.434893422
ILMN_1722900	EIF4A1	0.434796794
ILMN_1790962	RINL	0.434769567
ILMN_1689002	DTX1	0.434707369
ILMN_1811775	CCDC124	0.434595908
ILMN_1665515	NCRNA00152	0.434564013
ILMN_1750100	TUBB8	0.434471956
ILMN_1734895	SFT2D1	0.434410172
ILMN_2328835	IP6K2	0.434393725
ILMN_1811593	NIPAL1	0.434362344
ILMN_1711208	CELSR2	0.434253209
ILMN_1657153	ACTR3	0.43424429
ILMN_1696380	GHRL	0.434132412
ILMN_1652464	TUBA3E	0.434126098
ILMN_1704315	RPL27A	0.434064438
ILMN_2302947	CSF1	0.434053591
ILMN_2247594	RPLP1	0.433988142
ILMN_1734964	P2RX5	0.433911022
ILMN_2209260	KRTAP1-3	0.433887065
ILMN_1741148	ALDOA	0.433878961
ILMN_1763640	NCKAP5L	0.433826092
ILMN_1707810	RPS5	0.433754912
ILMN_1806757	MYBBP1A	0.433658048
ILMN_1717207	MMP25	0.433652197
ILMN_1651209	SLC35E2	0.433640606
ILMN_1658920	MCTP1	0.433602381
ILMN_1653529	TEX10	0.433552605
ILMN_3220952	ANKRD36	0.433515574
ILMN_2349696	CNIH2	0.4334979
ILMN_1797451	SRGAP2P2	0.43349389
ILMN_1736500	GNB2L1	0.433345035
ILMN_1812526	TGFB2	0.433263171
ILMN_3221499	ZNF735	0.433245046
ILMN_1737036	GGT7	0.433229757
ILMN_1708093	ARHGEF5	0.433163176
ILMN_2350801	SLC25A29	0.433157881
ILMN_1742260	SSFA2	0.433147972
ILMN_1738027	BRCA1	0.433147621
ILMN_1654065	ATOH8	0.433135866
ILMN_1803813	ASTE1	0.433126479
ILMN_1685830	SLC7A2	0.433057722
ILMN_1776327	BM008292	0.433001133
ILMN_1814917	TLE2	0.432928277
ILMN_1661599	DDIT4	0.432905003
ILMN_2299045	BMP2K	0.432829225
ILMN_1809818	PRCC	0.432804045
ILMN_1763603	FAM177A1	0.43279007
ILMN_1662256	PARP6	0.432770912
ILMN_1697308	AFF3	0.432657448
ILMN_1660376	RPL13A	0.432654395
ILMN_1665390	SPDYE7P	0.432510749
ILMN_1651316	CD69	0.432460208
ILMN_2140389	TMEM185A	0.432445374
ILMN_3259751	C14orf56	0.432438185
ILMN_2319077	FAS	0.432417806
ILMN_2209515	SNORD56	0.432402465
ILMN_1688749	RPS2	0.432397069
ILMN_1759359	BTF3L4	0.432376921
ILMN_2206716	JTB	0.432310808
ILMN_3193623	RPL17	0.432289812
ILMN_1775522	MAGED1	0.432257815
ILMN_1753890	IFT20	0.432243174
ILMN_1793559	C6orf204	0.432166325
ILMN_2391912	SEC14L1	0.432065133
ILMN_1693430	NME1	0.432006868
ILMN_3238203	LOC349114	0.431987317
ILMN_1780940	PPP2R5D	0.431891593
ILMN_3249419	FLJ14186	0.431726913
ILMN_1815039	RRP36	0.431512793
ILMN_1696911	FTH1	0.431465211
ILMN_2135798	NR2C2AP	0.431339862
ILMN_1722774	VPS72	0.431312924
ILMN_1763000	ADAP2	0.431275572
ILMN_1690646	SATB1	0.431250526
ILMN_1750563	CERCAM	0.431188455
ILMN_2377025	TCOF1	0.431125379
ILMN_1710710	DEDD	0.431119455
ILMN_1798256	UPP1	0.430915581
ILMN_1813840	KDM1A	0.430866815
ILMN_1659744	PRMT1	0.430847423
ILMN_1715926	RPL32	0.430804403
ILMN_1771957	MAN1B1	0.430756659
ILMN_1719611	CCT6P3	0.430706024
ILMN_1690125	PDLIM7	0.430654628
ILMN_1685433	COL8A1	0.43048902
ILMN_1680367	C10orf90	0.430381101
ILMN_1761442	WDR24	0.430309104
ILMN_1790149	HGF	0.430300026
ILMN_1697499	HLA-DRB1	0.430263778
ILMN_2408576	FAM129B	0.430195674
ILMN_3238481	SNORD60	0.430179272
ILMN_2328224	MADD	0.430028095
ILMN_1766914	MFAP4	0.429990139
ILMN_3279306	RPL17	0.42991776
ILMN_1770822	RPS6KA3	0.429913416
ILMN_1652237	CBR3	0.429843139
ILMN_1656868	NPIPL3	0.429805073
ILMN_1733863	FAM100A	0.429707997

ILMN_1809259	HRASLS2	-0.568766501
ILMN_1670817	GRPEL1	-0.568775132
ILMN_1731941	APOM	-0.568800948
ILMN_1651310	MLLT4	-0.56884737
ILMN_3239568	MYLK4	-0.568932433
ILMN_2177234	DEFB104B	-0.56913169
ILMN_1659086	DQ895884	-0.569460565
ILMN_1808262	OR8K3	-0.569851249
ILMN_1716803	RALYL	-0.569899854
ILMN_1795976	SFXN2	-0.570251332
ILMN_1719498	EPHX4	-0.570304644
ILMN_1770245	EPB41L5	-0.570417562
ILMN_1701831	GSTA1	-0.570452279
ILMN_2402463	SHMT1	-0.570963318
ILMN_3243519	LOC390858	-0.571006993
ILMN_3249343	FIGNL2	-0.57104764
ILMN_1759973	NDUFA5	-0.571117263
ILMN_1707548	RAD18	-0.571605885
ILMN_1781560	ST3GAL6	-0.571634856
ILMN_2382998	ZCCHC16	-0.572075759
ILMN_1723743	ROM1	-0.572179886
ILMN_1759396	NNT	-0.572264827
ILMN_1695092	WRB	-0.572960761
ILMN_1803882	VEGFA	-0.573094531
ILMN_1776334	UXS1	-0.57309988
ILMN_2390609	ANK3	-0.573137143
ILMN_1694359	UPF0639	-0.573316975
ILMN_1805404	GRIN1	-0.573400877
ILMN_1792744	SLC13A2	-0.573500513
ILMN_1753502	IGSF11	-0.573604665
ILMN_1709768	LOC100128818	-0.573626926
ILMN_1770466	ATP5G3	-0.573763136
ILMN_2232036	EFCAB7	-0.574103941
ILMN_2331232	TNFRSF6B	-0.574125462
ILMN_1737588	C21orf33	-0.574661627
ILMN_2397841	SNCB	-0.574741402
ILMN_1850574	BG943031	-0.574812741
ILMN_1839750	NA	-0.574888313
ILMN_2070792	ACSM5	-0.574928929
ILMN_1781472	CDC42BPA	-0.57511068
ILMN_1808305	RTCD1	-0.575144348
ILMN_1808487	PLA2G12B	-0.575228473
ILMN_1672605	C7orf41	-0.575345032
ILMN_3201715	NA	-0.575849174
ILMN_1689129	OPRM1	-0.575924713
ILMN_1675365	LARP4	-0.576044483
ILMN_1721680	LOC644075	-0.57638841
ILMN_1830259	AI630821	-0.576395461
ILMN_1776493	MTUS1	-0.576456836
ILMN_1707592	RGN	-0.576506217
ILMN_1696675	CES2	-0.576677274
ILMN_2174215	DIRAS3	-0.576794541
ILMN_1771084	ACSM3	-0.577076248
ILMN_1661491	SH3GL2	-0.577090851
ILMN_3182171	FGGY	-0.577268026
ILMN_1758750	EARS2	-0.577340997
ILMN_1735445	SLC7A9	-0.577619449
ILMN_2117526	DMGDH	-0.577800436
ILMN_1813296	RDH8	-0.577811541
ILMN_2351241	RNF14	-0.578028958
ILMN_1780625	HR	-0.578099283
ILMN_1804834	C6orf130	-0.578296049
ILMN_1903568	CR625988	-0.578319639
ILMN_1907705	BM717102	-0.578401221
ILMN_1693645	DPY19L2P4	-0.578434091
ILMN_1707464	MST1	-0.578654819
ILMN_1829989	LOC220930	-0.579187555
ILMN_1773835	ZNF585B	-0.57933754
ILMN_1788131	CYP26A1	-0.57939404
ILMN_1849726	AA526513	-0.579403193
ILMN_1853631	BX115738	-0.579591127
ILMN_1706579	SHBG	-0.579690441
ILMN_2358628	ADK	-0.579819384
ILMN_1738938	TIMM8B	-0.579898352
ILMN_1767590	TSPY3	-0.579981857
ILMN_1669123	AK125437	-0.580029339
ILMN_2188959	ACOT2	-0.580177873
ILMN_3261111	IP6K3	-0.580525736
ILMN_1773742	DNAJB9	-0.580584257
ILMN_1717853	DMGDH	-0.580704733
ILMN_1861652	BQ575680	-0.580888802
ILMN_1712088	CLYBL	-0.581184242
ILMN_3251346	NOX4	-0.58142813
ILMN_2284077	SLC38A8	-0.581757471
ILMN_1658299	CCR2	-0.582129448
ILMN_1694106	GPD1L	-0.582285339
ILMN_1787751	AB529228	-0.582431117
ILMN_2121816	GPR137B	-0.582622069
ILMN_1667263	BMS1P5	-0.583316955
ILMN_1774742	MTTP	-0.583443334
ILMN_1748927	IQCF5	-0.583566409
ILMN_2284810	CIDEA	-0.583598534
ILMN_1776724	LYPD6	-0.583734681
ILMN_2380181	MAG	-0.583803339
ILMN_1811278	RNF186	-0.583910224
ILMN_1887508	AI206543	-0.584135783
ILMN_2045729	WDR12	-0.584383024
ILMN_1676042	NA	-0.584492043

ILMN_2273331	CCDC46	0.429696614
ILMN_1801130	STOML1	0.429648568
ILMN_2339627	COPE	0.429587659
ILMN_2067656	CCND2	0.429578228
ILMN_2337655	WARS	0.429530509
ILMN_1774828	VEZT	0.42924749
ILMN_1760374	CD8A	0.429227115
ILMN_1801105	PRKCD	0.429225326
ILMN_3235176	C2orf89	0.429203682
ILMN_2324672	USF2	0.429170824
ILMN_1723177	ANAPC5	0.429143285
ILMN_1768973	HIST2H2AC	0.42913053
ILMN_3195253	RPL7	0.429062095
ILMN_3241870	FRMD8	0.42904272
ILMN_1756424	SNX21	0.428941526
ILMN_1715804	PITPNA	0.428830837
ILMN_2059744	IL7	0.428793083
ILMN_2400512	PRKCSH	0.428773642
ILMN_1712545	S100A3	0.428752501
ILMN_2364529	EZH2	0.428741256
ILMN_1791057	IFNAR2	0.428602056
ILMN_2115633	CD320	0.428578666
ILMN_2272270	COL4A3	0.428533128
ILMN_1787718	SLC27A1	0.428469876
ILMN_3238196	CYTH4	0.428466959
ILMN_1694426	ROR2	0.428379288
ILMN_1718633	LRP5L	0.428343106
ILMN_1782829	GLTSCR1	0.428233018
ILMN_1755883	RPS27A	0.428222766
ILMN_2163070	KHDC1	0.428193025
ILMN_2394250	PLEKHA1	0.428149307
ILMN_3227912	RPL15	0.42810414
ILMN_1815057	PDGFRB	0.428007457
ILMN_1675956	LYST	0.427926132
ILMN_1684571	RSPH1	0.427918178
ILMN_1669584	ILF3	0.427754339
ILMN_1723156	PTPRA	0.427700133
ILMN_2241953	PILRA	0.427675837
ILMN_1717524	LIPT1	0.427606855
ILMN_2331501	UBC	0.427565674
ILMN_1815874	NANS	0.427552724
ILMN_3236169	MACC1	0.427508463
ILMN_1746720	TTC39C	0.427478166
ILMN_2133638	CTDNEP1	0.42746431
ILMN_1781281	B9D1	0.427305036
ILMN_1709044	TGIF2	0.427297779
ILMN_1660810	TTC17	0.427296245
ILMN_1730294	INO80C	0.427255201
ILMN_2363621	RBBP8	0.427253636
ILMN_1793384	JAK1	0.427183667
ILMN_1808590	GUCY1A3	0.427173588
ILMN_2109708	TYMP	0.427128714
ILMN_2297824	UBE2G2	0.427064118
ILMN_1745223	CDC42EP4	0.426993841
ILMN_3205289	GOLGA8G	0.426933979
ILMN_1656016	DHX9	0.426903262
ILMN_1802205	RHOB	0.426860419
ILMN_1653165	AAMP	0.426765664
ILMN_1757978	INO80D	0.426744926
ILMN_1709860	UNC45A	0.426663437
ILMN_1684051	WASF2	0.426656957
ILMN_1768488	TERF2	0.426604826
ILMN_1656129	SLC39A10	0.426597891
ILMN_2321064	BAX	0.426591967
ILMN_2378048	HNRNPK	0.426568604
ILMN_1808657	FMO3	0.426548244
ILMN_3207060	RPL4	0.426490435
ILMN_1713394	RPS16	0.426405039
ILMN_1795933	CHMP7	0.426389068
ILMN_1659752	AIDA	0.426373826
ILMN_3247578	FAT1	0.426359163
ILMN_1765801	GAA	0.426358414
ILMN_1758214	RARS2	0.426337458
ILMN_1703288	C19orf6	0.426320061
ILMN_1779795	BMS1P4	0.426238596
ILMN_1810922	PCNT	0.426238467
ILMN_2411723	RPSA	0.426190643
ILMN_1811364	CD556913	0.426076709
ILMN_1663925	OR2L5	0.426016664
ILMN_2399503	UBN1	0.426015018
ILMN_1690035	FLYWCH1	0.426002684
ILMN_1737005	C19orf61	0.4258819
ILMN_1713636	S100A6	0.425766244
ILMN_1655710	RPS25	0.425764229
ILMN_1651507	PYROXD1	0.425763831
ILMN_1764613	OR52E8	0.425721556
ILMN_3231621	LOC349114	0.42558267
ILMN_2414007	NME1	0.425531862
ILMN_1778319	DMKN	0.425478152
ILMN_1746138	RLTPR	0.425469782
ILMN_3243573	abParts	0.425468171
ILMN_1683824	ANO7	0.425332957
ILMN_1727574	ZNF827	0.425274747
ILMN_1663618	STAT3	0.425197188
ILMN_2380163	PTPRF	0.425147105
ILMN_1667453	HINFP	0.425084784
ILMN_1708151	LAGE3	0.425050772
ILMN_2101278	RGS18	0.424934702

ILMN_1685796	CSDE1	-0.584552858
ILMN_3240173	MTSS1L	-0.584630817
ILMN_1672800	RASA4	-0.58477931
ILMN_1776601	OR4F5	-0.585185162
ILMN_1703475	CLSPN	-0.586658479
ILMN_2150019	SUCLA2	-0.58705234
ILMN_1892573	BC031234	-0.587416371
ILMN_1754491	IYD	-0.58747539
ILMN_1651604	MARK4	-0.587530805
ILMN_1662417	LRPPRC	-0.587595287
ILMN_1657682	GFM1	-0.587909839
ILMN_1727438	KCND3	-0.588124252
ILMN_1770663	KRT24	-0.588468622
ILMN_1686152	GGA2	-0.588508308
ILMN_1654151	COX6C	-0.5888205
ILMN_1774757	ZNF496	-0.588936263
ILMN_2053679	ACADM	-0.588989762
ILMN_1688220	SYNPO2	-0.589304154
ILMN_1662091	FLJ43860	-0.589372997
ILMN_1720889	SC4MOL	-0.590261238
ILMN_3247438	C19orf69	-0.590553673
ILMN_1767474	HAO2	-0.590913188
ILMN_1739429	HS6ST2	-0.591133663
ILMN_3200885	TNFRSF14	-0.591159023
ILMN_1718520	RNLS	-0.591213071
ILMN_1801403	DCUN1D4	-0.591388141
ILMN_1652037	UPP2	-0.591463807
ILMN_1702858	ADHFE1	-0.591888712
ILMN_1826853	NA	-0.592409393
ILMN_2323366	SDHC	-0.592495126
ILMN_3241953	A2LD1	-0.592562539
ILMN_1664592	C1orf95	-0.592741202
ILMN_1749244	LYRM1	-0.59279522
ILMN_1685854	C5orf53	-0.592829006
ILMN_1656145	GOT1	-0.592892974
ILMN_1661994	ESRRG	-0.593104016
ILMN_1720617	OR2T6	-0.593177961
ILMN_1651967	TP53I3	-0.593472646
ILMN_2096985	ALDH6A1	-0.595140367
ILMN_1803673	LOC113230	-0.595260122
ILMN_1748323	CXCL14	-0.59558514
ILMN_1751943	MME	-0.595606499
ILMN_3305768	GSTA7P	-0.596797544
ILMN_1915913	BG722090	-0.596970535
ILMN_1800659	PGM1	-0.596979454
ILMN_1778357	DNMT3L	-0.597271234
ILMN_1700549	ERLIN2	-0.597481448
ILMN_1799293	MINA53	-0.597514518
ILMN_1686573	DEFB1	-0.597906817
ILMN_1769601	TBCK	-0.597970057
ILMN_1750800	ACO1	-0.598288703
ILMN_1874770	DA691010	-0.598586086
ILMN_1661399	ARL4A	-0.59888559
ILMN_1811880	SLC34A3	-0.599313836
ILMN_2207504	LEP	-0.599498868
ILMN_1674021	LOC401321	-0.599513951
ILMN_1783645	DKFZp666P158	-0.599842435
ILMN_1746426	TOMM70A	-0.599914099
ILMN_1794890	SLC38A4	-0.600327923
ILMN_3290715	COL6A4P2	-0.600615208
ILMN_1766896	ZDHHC19	-0.600771378
ILMN_1666939	MEI1	-0.60082162
ILMN_1718001	PHKB	-0.60084729
ILMN_2151739	CAT	-0.60151176
ILMN_1674098	SORBS2	-0.602113565
ILMN_1728883	AB074415	-0.602141567
ILMN_3269377	FLJ00278	-0.602186411
ILMN_2363634	ADHFE1	-0.602664293
ILMN_1832155	DCUN1D5	-0.602887631
ILMN_1800874	MRO	-0.603093202
ILMN_2184049	COX7B	-0.603248473
ILMN_1739925	SLC28A1	-0.603834799
ILMN_1742040	GDPD4	-0.603982854
ILMN_1690621	GPR98	-0.604487069
ILMN_1757693	C17orf51	-0.605284391
ILMN_3187741	IGLON5	-0.605466215
ILMN_1843198	AK4	-0.606145197
ILMN_2058347	NTSDC1	-0.606245016
ILMN_1685886	MAPK4	-0.607198422
ILMN_3225886	NOX4	-0.607521093
ILMN_2344204	PRR13	-0.607564895
ILMN_1770725	AIF1L	-0.608129893
ILMN_2341548	MYO5B	-0.608930064
ILMN_1794875	AGPAT9	-0.609030517
ILMN_1891311	AA744242	-0.609122061
ILMN_2113470	GSTA1	-0.609382488
ILMN_1771061	BRD4	-0.610112796
ILMN_1815570	HOXA6	-0.610290947
ILMN_1670385	TUBAL3	-0.611274609
ILMN_2276461	MAP2	-0.611430482
ILMN_1668012	SLC25A13	-0.611442065
ILMN_1805251	MGC26484	-0.611747185
ILMN_1810604	ELMOD1	-0.612119237
ILMN_1761058	NPHP3-ACAD11	-0.612238313
ILMN_1760110	AK023936	-0.612625894
ILMN_1733807	IGF2BP1	-0.612764778
ILMN_1763878	CDADC1	-0.613896032
ILMN_1669617	GRB10	-0.614259593

ILMN_2397199	NDEL1	0.424904033
ILMN_1659610	TJP3	0.424892577
ILMN_2053546	RPL19	0.424857176
ILMN_1787567	TSC22D1	0.424844354
ILMN_2328952	CLCC1	0.424760419
ILMN_1750181	TESC	0.424720123
ILMN_1726783	RNASEH1	0.424708853
ILMN_1777487	ZNF839	0.424695299
ILMN_1764723	SH3PXD2B	0.424689495
ILMN_2331121	FCGR2B	0.42466149
ILMN_1708936	EXOSC3	0.424658838
ILMN_1657554	TSPYL2	0.424637287
ILMN_3217815	RPS2	0.424588448
ILMN_2350634	EFEMP1	0.424541232
ILMN_1716608	NGF	0.424487788
ILMN_1890263	BX101040	0.424456008
ILMN_1759787	THBD	0.424229009
ILMN_2148785	GBP1	0.424083849
ILMN_1653861	SCMH1	0.424076146
ILMN_1701832	RPL19	0.424027863
ILMN_1723017	LOC728643	0.423922063
ILMN_2409055	FAM13C	0.423894969
ILMN_1652313	RNF215	0.423867072
ILMN_3242466	NA	0.423819712
ILMN_1760314	CCDC8	0.423793373
ILMN_1673024	RBM15B	0.423757017
ILMN_1658398	FAM198A	0.423676241
ILMN_1660786	HIPK1	0.423675704
ILMN_1684183	RAD9A	0.423660267
ILMN_1677589	hCG_1818547	0.423642917
ILMN_1686626	DDX39B	0.423605392
ILMN_1724250	GRN	0.423604333
ILMN_1715189	LHX6	0.423603861
ILMN_1669281	CLN3	0.423475194
ILMN_1656882	abParts	0.423433397
ILMN_1751726	RPLP0	0.423365384
ILMN_1748105	DDB1	0.423341188
ILMN_1701629	AK127589	0.423300499
ILMN_1651378	AUP1	0.423286479
ILMN_1666208	MIS18BP1	0.42325709
ILMN_2397028	SERPINB8	0.423189851
ILMN_1722537	FLJ40504	0.423143589
ILMN_1737599	TRIM5	0.423002514
ILMN_1793017	DGKQ	0.422992
ILMN_1775073	ZNF273	0.422987712
ILMN_1814402	NFIC	0.42288822
ILMN_1779147	ENC1	0.422868928
ILMN_1663562	OR2A42	0.422799553
ILMN_2234873	NME1	0.422791379
ILMN_2400661	ZNF626	0.422748439
ILMN_1759757	IGSF22	0.422721248
ILMN_1779071	FEZ1	0.422708606
ILMN_1746276	EPC1	0.42264625
ILMN_1795119	MYH3	0.422559709
ILMN_1651504	FAM193A	0.422543939
ILMN_2399174	TRAK1	0.422375231
ILMN_2112417	PGAM1	0.422248673
ILMN_2109994	MIA-RAB4B	0.422229887
ILMN_1653514	RBBP4	0.422208446
ILMN_1712944	AES	0.42211953
ILMN_1774844	MAPKAPK2	0.421996988
ILMN_1699354	EPHA2	0.421980098
ILMN_1753584	KRT8	0.4219652
ILMN_1693604	GRM2	0.421932555
ILMN_1675626	PRPF38A	0.421919567
ILMN_1655167	ZNF502	0.421913878
ILMN_1784847	CREBZF	0.4219098
ILMN_2396571	GPR108	0.421898045
ILMN_1663113	TTLL12	0.421821408
ILMN_1658283	RPL17	0.421811457
ILMN_1748797	GRB2	0.421796731
ILMN_1794442	ZNF469	0.421740778
ILMN_1778226	EXTL3	0.421740471
ILMN_1781769	CCRL1	0.421689567
ILMN_1692223	LCN2	0.421677716
ILMN_1911059	AV691356	0.421654664
ILMN_1766675	CDH6	0.421641599
ILMN_1674780	SF3B1	0.421565408
ILMN_2058782	IFI27	0.421507941
ILMN_1680579	ATP2B4	0.421503116
ILMN_1669550	MAD2L2	0.421501
ILMN_1724145	CBX4	0.421495136
ILMN_1793959	ADPGK	0.421445031
ILMN_1789007	APOC1	0.421378915
ILMN_2103841	AIP	0.421373411
ILMN_1708604	CCZ1	0.421272574
ILMN_1810467	PPP2R1A	0.421264781
ILMN_1699856	RALGDS	0.421239729
ILMN_1785646	PMP22	0.421239224
ILMN_2389013	ADRM1	0.421236356
ILMN_1765082	RBM10	0.421224803
ILMN_3246292	RPL23A	0.421211143
ILMN_1732060	ARHGAP1	0.421145743
ILMN_1716397	LAYN	0.421039469
ILMN_1721842	RYBP	0.421022343
ILMN_2215211	ZNF514	0.420992436
ILMN_1769757	C14orf104	0.420971567
ILMN_1766184	LGALS9C	0.420705901

ILMN_1748352	CTSL2	-0.615118619
ILMN_1782937	GSTA1	-0.616042056
ILMN_1663538	CLYBL	-0.616277661
ILMN_2078074	MUT	-0.61719272
ILMN_2124361	LEAP2	-0.617212148
ILMN_2322747	ARHGAP5	-0.617346894
ILMN_2211546	HAO1	-0.617356384
ILMN_1788895	SRD5A2	-0.618498355
ILMN_3245250	CTSL1P8	-0.618681461
ILMN_1763196	WDR72	-0.618841599
ILMN_1837348	AI272125	-0.619584978
ILMN_1753729	KNG1	-0.619723113
ILMN_1769556	NPR3	-0.620165173
ILMN_1818856	BE549596	-0.62117714
ILMN_1730229	CGNL1	-0.621180739
ILMN_1701127	CC2D1A	-0.621193388
ILMN_1729281	SPHK2	-0.622073624
ILMN_1806310	A1CF	-0.623898909
ILMN_1714384	PCCA	-0.624288495
ILMN_1707775	ZNF257	-0.624374926
ILMN_1658940	DDX25	-0.624959634
ILMN_1703174	FREM2	-0.625868265
ILMN_1767277	PLGLA	-0.626729555
ILMN_1827736	PDK3	-0.626833401
ILMN_1815010	RNF141	-0.627416016
ILMN_1779241	CRYM	-0.627549742
ILMN_1678720	SULT1C2P1	-0.627784801
ILMN_1824490	BG618056	-0.627794004
ILMN_1759910	SERPINA5	-0.629102664
ILMN_1831098	BX092776	-0.629520577
ILMN_1713846	PPM1H	-0.629628149
ILMN_1801938	NHS	-0.629825905
ILMN_1720482	CEND1	-0.629950483
ILMN_1689156	MMAB	-0.630108638
ILMN_1681437	DCXR	-0.630178199
ILMN_1788122	GSTA5	-0.631440444
ILMN_2166457	HPGD	-0.63149439
ILMN_1780758	POLR1A	-0.631708232
ILMN_1673503	CACNB4	-0.632149985
ILMN_1703678	DNM1P33	-0.632470368
ILMN_1810200	ARPC2	-0.633362127
ILMN_1686405	KDR	-0.633720646
ILMN_1731181	TEX2	-0.634143846
ILMN_3305614	ZNF812	-0.634374453
ILMN_2196588	C18orf32	-0.634551418
ILMN_1754501	C2orf74	-0.634764572
ILMN_3270542	UQCRFS1	-0.634847033
ILMN_1807339	HRG	-0.635585876
ILMN_1887505	BX648650	-0.636672263
ILMN_1734707	CHST13	-0.636957482
ILMN_2057220	HRSP12	-0.637332227
ILMN_1803745	SUOX	-0.638489655
ILMN_2054607	CYP4V2	-0.638561371
ILMN_1655613	GSTA2	-0.639125143
ILMN_3239925	LOC25845	-0.63960895
ILMN_3227600	ZNF385B	-0.639838529
ILMN_1895897	BF514393	-0.640576254
ILMN_1662675	ADAM2	-0.642135861
ILMN_1737152	DAO	-0.642324644
ILMN_1897333	BX098521	-0.642553747
ILMN_1785218	MTCH2	-0.643883244
ILMN_1831214	AW592010	-0.644402469
ILMN_1670542	AK2	-0.644510759
ILMN_1749162	DIO2	-0.644917834
ILMN_2343097	NCALD	-0.645108495
ILMN_1741662	TMC5	-0.646602162
ILMN_1704166	LNP1	-0.6472601
ILMN_1805024	ERBB2IP	-0.647276469
ILMN_1673980	LOC643669	-0.647385101
ILMN_1706441	UPK3B	-0.649422153
ILMN_1688886	GPC5	-0.649635694
ILMN_1763092	MCCD1	-0.650242118
ILMN_3251540	GSTA2	-0.651710351
ILMN_3307836	SLC5A12	-0.651739172
ILMN_1808379	PLCD4	-0.653267363
ILMN_1783497	PANK1	-0.65332673
ILMN_1685441	ASAP3	-0.653794881
ILMN_2316918	PANK1	-0.654167045
ILMN_1729482	KLK15	-0.654440701
ILMN_1738736	SNX4	-0.655078367
ILMN_2043809	PFKM	-0.655791172
ILMN_1808457	TBX18	-0.656103841
ILMN_3246214	B3GNT1	-0.656269212
ILMN_1718619	TRIM11	-0.656550139
ILMN_1782015	FCRLB	-0.657444507
ILMN_3204686	SLC16A6	-0.65908455
ILMN_1665033	NPR3	-0.65921345
ILMN_1812600	NA	-0.659288916
ILMN_2168747	GSTA2	-0.659552028
ILMN_1670547	GRM1	-0.660564003
ILMN_1836218	LOC25845	-0.662409452
ILMN_1827211	U43604	-0.662631303
ILMN_2285375	SORD	-0.662790469
ILMN_1703346	CHRNE	-0.664596815
ILMN_1891725	BX111675	-0.66514725
ILMN_1695991	COLQ	-0.665724101
ILMN_1687392	NRK	-0.669383172
ILMN_1740418	CYP27B1	-0.672281957

ILMN_1798808	FOXH1	0.420698951
ILMN_2364376	ILK	0.420687364
ILMN_2406501	SOD2	0.420634617
ILMN_1751793	PCNXL2	0.420516025
ILMN_2316278	MAGED4B	0.420489464
ILMN_3242016	POTEE	0.420463941
ILMN_2329171	SPDYE1	0.420385725
ILMN_1699598	AP2M1	0.420370408
ILMN_1809477	CARHSP1	0.420297319
ILMN_1678805	POMT2	0.420263232
ILMN_2342066	METRNL	0.420155165
ILMN_1769195	KIAA0378	0.419957221
ILMN_1707612	FIGF	0.419939856
ILMN_1760890	SEPN1	0.419910671
ILMN_1691930	CBX6	0.419850985
ILMN_2413064	ST6GALNAC4	0.419687419
ILMN_2402798	AP2M1	0.419623905
ILMN_1800302	LRTM1	0.419490477
ILMN_1669032	PPIC	0.419425301
ILMN_1699574	NRP1	0.419403028
ILMN_2367020	SEC61G	0.419331582
ILMN_1698386	NEK9	0.419311756
ILMN_1744347	DQ895289	0.419242596
ILMN_1916702	LOC100505576	0.419222887
ILMN_3241657	TMEM204	0.419215253
ILMN_1806733	COL18A1	0.419142458
ILMN_1740505	SGMS1	0.419129981
ILMN_1656521	RRP7A	0.419128472
ILMN_1651719	MBTPS1	0.419031669
ILMN_1789502	GPC4	0.419028205
ILMN_1683441	NCAPD3	0.418966595
ILMN_1698404	AK055561	0.418947946
ILMN_1720819	SPCS2	0.418902701
ILMN_1748755	KCND2	0.418845083
ILMN_1811063	RPL29	0.418839348
ILMN_1803220	PODNL1	0.418830551
ILMN_1780898	PRKCH	0.418827339
ILMN_2117623	PLXNC1	0.418780247
ILMN_2348503	PPIE	0.418737054
ILMN_1706706	WDR68	0.418730061
ILMN_1759915	ARPC1A	0.418698
ILMN_2188451	HIST1H2AH	0.41855239
ILMN_1741406	HOOK1	0.418381793
ILMN_1846771	BU521176	0.418359696
ILMN_1686261	TOM1L2	0.418336096
ILMN_1684897	SPDYE1	0.41815769
ILMN_1787815	TRIB3	0.418114203
ILMN_2402168	EXOSC10	0.418107404
ILMN_2115434	RAB32	0.41808058
ILMN_1674874	MFSD10	0.418014934
ILMN_3192791	HNRNPM	0.418012196
ILMN_3236373	MSL2	0.417993974
ILMN_1703487	LMO4	0.41791432
ILMN_2291954	SLA	0.417890391
ILMN_2163723	KRT7	0.41776627
ILMN_1815083	WHSC2	0.417637599
ILMN_3287687	TUBB2A	0.417624834
ILMN_1819494	NA	0.417611222
ILMN_1792710	DAPK3	0.417582402
ILMN_1736256	CALR	0.417461004
ILMN_1800897	NRD1	0.417330641
ILMN_2364521	AXL	0.417330213
ILMN_1762787	RNF26	0.417244756
ILMN_1802646	EPHB6	0.417240547
ILMN_2322972	ZFYVE27	0.417199223
ILMN_1898771	TCRA	0.41710118
ILMN_3235254	SPDYE8P	0.417092331
ILMN_2191759	HNRNPA1	0.417074925
ILMN_3282245	NA	0.417016807
ILMN_2411897	KLF10	0.417010648
ILMN_1682428	HENMT1	0.416961959
ILMN_1718044	ELAC2	0.416862887
ILMN_2302654	LRP8	0.416862642
ILMN_1655068	TOM1L2	0.416748006
ILMN_1705763	SCT	0.41669961
ILMN_2110829	RPL23A	0.416529212
ILMN_2396875	IGFBP3	0.416456144
ILMN_3287068	LAMR1P15	0.416433029
ILMN_1790951	C19orf50	0.416433006
ILMN_1706959	TIMM22	0.416356571
ILMN_2338116	TRAF5	0.416322532
ILMN_1770281	ERC1	0.416243995
ILMN_1773497	UCMA	0.416036571
ILMN_2210581	B3GAT3	0.416009382
ILMN_1779512	AP4M1	0.415889712
ILMN_1754580	FRMPD1	0.415877264
ILMN_2154950	ZNF423	0.415862463
ILMN_3233787	SRRM1	0.415784482
ILMN_1738684	NRXN2	0.415682932
ILMN_3234831	RPL17	0.415663191
ILMN_2185866	PCDHB17	0.415578577
ILMN_1656011	RGS1	0.415497116
ILMN_1799819	MARS	0.415462305
ILMN_1775708	SLC2A3	0.415451314
ILMN_2143261	CXorf40B	0.415402806
ILMN_1766185	AXIN1	0.415401135
ILMN_2385178	MIB2	0.415377485
ILMN_3233753	AK293020	0.415368026

ILMN_1763975	NCRNA00083	-0.673116154
ILMN_1690733	EGF	-0.67440008
ILMN_1753877	MYADML2	-0.675902758
ILMN_1692517	SORD	-0.685948045
ILMN_1815949	SLC24A4	-0.686388229
ILMN_1709247	PPP2R1B	-0.687951674
ILMN_1881598	AW451118	-0.688893178
ILMN_1878728	DB222718	-0.690400521
ILMN_1736077	LIAS	-0.691197766
ILMN_2198239	HGD	-0.691320158
ILMN_1773262	ESM1	-0.693815511
ILMN_2352609	OGG1	-0.693999252
ILMN_1826572	AK094562	-0.695140014
ILMN_1790036	C20orf202	-0.697173494
ILMN_1712430	ATP5G1	-0.700285025
ILMN_2212878	ESM1	-0.702506931
ILMN_1685703	ACOX2	-0.707345597
ILMN_2193936	HRG	-0.711993441
ILMN_1719468	EPM2A	-0.713587976
ILMN_1678965	FABP1	-0.71591851
ILMN_1684585	ACSL1	-0.716298112
ILMN_2376133	KIAA1191	-0.716380563
ILMN_2227817	GSTA3	-0.71678987
ILMN_1773073	PHYH	-0.716860112
ILMN_1810324	SGCB	-0.721013784
ILMN_3235803	AQP7P3	-0.72766752
ILMN_1728467	GHITM	-0.727930358
ILMN_1800049	MAPT	-0.736598213
ILMN_2050911	SLC22A4	-0.736910448
ILMN_1682062	RYR1	-0.739442417

ILMN_2316540	MRPL11	0.415345423
ILMN_2139740	ZNF503	0.415323444
ILMN_1653438	PHF14	0.415317609
ILMN_1701875	ZYX	0.415310054
ILMN_2319996	RPL3	0.415259722
ILMN_1745469	SEC24B	0.415253462
ILMN_1761068	DKFZp434K132	0.415241285
ILMN_2372922	PPP2R4	0.41521036
ILMN_1799120	WFDC2	0.415124526
ILMN_1754746	CCNB3	0.415116689
ILMN_1678087	MAP3K4	0.415068125
ILMN_2118773	ASAH2B	0.415008141
ILMN_1656501	DUSP5	0.414985758
ILMN_1753342	SAT1	0.414979153
ILMN_1694325	NFIX	0.414970801
ILMN_1710544	PCDH7	0.4149537
ILMN_1691201	FAM21C	0.414919691
ILMN_2350357	ZNF254	0.414846167
ILMN_3248487	RPL7A	0.41475777
ILMN_1731546	RPL26	0.414696557
ILMN_1793378	KCTD16	0.414554353
ILMN_1774389	LRRC36	0.414538408
ILMN_1769473	SETD2	0.414511344
ILMN_1664012	CANT1	0.414499981
ILMN_2121437	NCL	0.414447826
ILMN_2396269	CDH24	0.414400529
ILMN_2086105	SPRY4	0.414361563
ILMN_1759883	SRP9	0.414332034
ILMN_2388507	AKT1	0.414301161
ILMN_1742521	GRB2	0.414287477
ILMN_1669607	PHKG2	0.414266088
ILMN_1696434	LAMA1	0.414215177
ILMN_1801101	ZBTB48	0.414174162
ILMN_1796755	ITGB5	0.414086349
ILMN_3242293	SPDYE7P	0.414066772
ILMN_2413780	SEZ6L2	0.414007236
ILMN_1807492	PSMC1	0.413926101
ILMN_1705783	NXF1	0.413882068
ILMN_2382500	MS4A14	0.413861363
ILMN_2057566	PGP	0.413861357
ILMN_1701195	PLA2G7	0.413844395
ILMN_1790472	SLC25A28	0.413832575
ILMN_1669424	YBX1	0.413829372
ILMN_3243112	RNF182	0.413799628
ILMN_1722292	AVL9	0.413769992
ILMN_3235326	LOC388796	0.413757497
ILMN_1777164	DIP2A	0.413691749
ILMN_1799099	TEKT3	0.413641894
ILMN_2200636	KIAA1267	0.413619997
ILMN_1757604	TPM2	0.413367853
ILMN_3247452	OST4	0.413367623
ILMN_2069128	EPB41L2	0.413309125
ILMN_1696494	CMTM6	0.413271563
ILMN_1726434	UNC45A	0.413255256
ILMN_1662963	PSD2	0.413240678
ILMN_2367010	GPR126	0.413228458
ILMN_1705991	SMA4	0.413177901
ILMN_1750507	RPL9	0.413170652
ILMN_3250147	ZNF626	0.413098319
ILMN_1654289	ELK1	0.413084135
ILMN_1652161	PNKD	0.412891514
ILMN_3301749	SPNS2	0.412842842
ILMN_1731907	LOC349114	0.412714787
ILMN_1757631	DBNDD1	0.412671784
ILMN_3310558	MIR302D	0.412571785
ILMN_1703326	GPR44	0.412488714
ILMN_1714622	TNRC6A	0.412387366
ILMN_1678669	RRM2	0.412377867
ILMN_2211122	TRAPPC2	0.412281427
ILMN_1802706	IDH3G	0.412277646
ILMN_1663681	SNORD54	0.41224746
ILMN_2226304	ANKRD50	0.412245973
ILMN_1757134	MPV17L2	0.412167977
ILMN_3237734	TNRC18	0.412148375
ILMN_1707631	MED10	0.412122191
ILMN_2038772	RPS9	0.412072053
ILMN_1735148	SEC24B	0.412059987
ILMN_1759341	MAN2B1	0.412020445
ILMN_1666522	ZDHHC14	0.411954974
ILMN_2129161	LRRC32	0.411927301
ILMN_1706273	MOB2	0.411911863
ILMN_1830984	BF509118	0.411886485
ILMN_1674236	HSPB1	0.411843784
ILMN_2332368	VPS13B	0.411843169
ILMN_3249624	PKD1P1	0.411812212
ILMN_2318643	TGIF1	0.411806036
ILMN_1694475	GTPBP2	0.411794443
ILMN_1799642	TRIM24	0.411697892
ILMN_1744912	CTTN	0.411642308
ILMN_1685676	TTC9C	0.411540501
ILMN_1808435	OGT	0.411534892
ILMN_1745075	RPLP0	0.411503174
ILMN_1751871	STK11	0.411351123
ILMN_1687867	KRT8	0.411336505
ILMN_2055330	KIF26B	0.41130123
ILMN_1727194	CALU	0.411277094
ILMN_1721762	IL18RAP	0.411255921
ILMN_1693438	FBXL12	0.411221277

ILMN_1662174	ORMDL3	0.411188442
ILMN_1731609	CHMP6	0.411147077
ILMN_1812353	CSPP1	0.411132188
ILMN_1802627	PSMG3	0.410926077
ILMN_3249110	CSRNP2	0.410921199
ILMN_1764794	PSMB2	0.410889953
ILMN_1724907	NUDT3	0.410883013
ILMN_1755589	DIP2B	0.410721407
ILMN_1789642	DNAJC5	0.410680809
ILMN_1664910	RPSA	0.410643154
ILMN_3209883	LOC653075	0.410586377
ILMN_1797964	PFAAP1	0.410489515
ILMN_1716300	CDH7	0.410483828
ILMN_2093720	THG1L	0.410468345
ILMN_1656316	ZMYM3	0.410271862
ILMN_3188984	ZNFX1-AS1	0.410213424
ILMN_1724540	ALX1	0.410139184
ILMN_2051867	PTCD2	0.410135002
ILMN_2092850	HPSE	0.410033399
ILMN_1682717	IER3	0.410032938
ILMN_1716089	KANK2	0.409980956
ILMN_1770787	DDAH2	0.409945563
ILMN_1668721	CCND3	0.409890697
ILMN_1717990	CALD1	0.409884539
ILMN_2386849	DSE	0.409850625
ILMN_1725417	NELL2	0.409847752
ILMN_1718960	SERPINB8	0.409828357
ILMN_2098446	PMAIP1	0.409768518
ILMN_1804415	SMAGP	0.409746615
ILMN_2110252	NPM3	0.409717791
ILMN_2089167	RHOD	0.40955041
ILMN_1738326	EIF4E2	0.409488942
ILMN_1703370	ZDHHC12	0.409486505
ILMN_1758939	RIPK2	0.409350404
ILMN_1703803	ADAMTS3	0.409302079
ILMN_3214532	RPL21	0.409176968
ILMN_1822775	DB336446	0.409147852
ILMN_1713491	VAMP2	0.409124865
ILMN_1680626	PDIA6	0.409102317
ILMN_1740160	PLCG1	0.40900996
ILMN_1772644	EML3	0.408997467
ILMN_1708416	ARL6IP1	0.408944764
ILMN_1777906	MAP7D1	0.408937038
ILMN_3218223	MPHOSPH10	0.408877248
ILMN_1700477	MRPL43	0.408876708
ILMN_1677402	C11orf96	0.40885333
ILMN_1671142	GPR68	0.408771073
ILMN_1791759	CXCL10	0.408677901
ILMN_1795228	ZFAND5	0.408652086
ILMN_1783712	C16orf88	0.408588556
ILMN_2412024	JMJD6	0.408527496
ILMN_1763207	BATF3	0.408519996
ILMN_1758963	NADK	0.408457254
ILMN_3247506	FCGR1C	0.40845678
ILMN_1763326	C5orf25	0.408340905
ILMN_1746516	RPS25	0.408334136
ILMN_1687538	ETS1	0.408278332
ILMN_1767747	HDAC2	0.408237327
ILMN_1651610	SPRY1	0.408222638
ILMN_1691165	CLSPN	0.408217541
ILMN_2320964	ADAR	0.408158886
ILMN_1765923	C17orf101	0.408144772
ILMN_3236825	RAPGEF5	0.408126524
ILMN_2128750	PTTG1IP	0.408064748
ILMN_2136455	C3orf64	0.408048425
ILMN_1733157	THSD1	0.407925136
ILMN_1770892	YY1	0.407903778
ILMN_1768470	EIF4G1	0.407882427
ILMN_1712026	NLRP3	0.407749917
ILMN_2069267	BAGE	0.407714137
ILMN_2399036	SEPN1	0.40768172
ILMN_1809488	SPCS2	0.407660792
ILMN_2203891	SMAD7	0.4076416
ILMN_2213136	LEF1	0.407625403
ILMN_2261600	FCGR1B	0.407594246
ILMN_1689146	GABRP	0.407519805
ILMN_1773470	ST5	0.407437136
ILMN_1797684	PDCD2	0.40741978
ILMN_1708029	TMEM8B	0.407416221
ILMN_1723477	RUNX1T1	0.4073579
ILMN_1765409	STAM	0.407331668
ILMN_1776105	PSPH	0.407258303
ILMN_1763347	PIK3CB	0.407165148
ILMN_3233748	NA	0.407135937
ILMN_2364828	OGT	0.407106022
ILMN_1799198	OTUB2	0.406981091
ILMN_2211922	AK057583	0.406899266
ILMN_1763524	LZTS2	0.406896904
ILMN_1733023	CCT6P1	0.406888095
ILMN_1682960	DTNBP1	0.406884948
ILMN_1715809	OGFRL1	0.406877773
ILMN_2350574	MYADM	0.406799755
ILMN_2244087	DNAJC28	0.406778079
ILMN_3274814	LOC100133286	0.406766764
ILMN_1773620	SMARCC2	0.406739161
ILMN_1825027	AK124463	0.406701413
ILMN_1781999	ABCF2	0.406599267
ILMN_1746171	H2AFY	0.406586338

ILMN_1767448	LHFP	0.406574465
ILMN_1698179	TAGLN3	0.406574047
ILMN_2395389	PSMC4	0.406507737
ILMN_1673757	CASP8	0.406458862
ILMN_1802089	SYMPK	0.406242619
ILMN_2296037	PTPN2	0.406176224
ILMN_2103591	MORC2	0.406088712
ILMN_1723004	CD72	0.406010162
ILMN_3302177	RPL14	0.405934475
ILMN_2376403	TSC22D3	0.405884985
ILMN_2298958	MRRF	0.405876843
ILMN_1672662	SLC20A1	0.405864645
ILMN_1741422	FUT8	0.405862056
ILMN_1803997	SDCCAG3	0.40584687
ILMN_1747589	HIST2H2AB	0.405839161
ILMN_1657591	SCN3A	0.405688667
ILMN_1654072	CX3CL1	0.40563885
ILMN_1657771	CRTC2	0.405549282
ILMN_1675124	DDX17	0.405499802
ILMN_3305055	TP63	0.405448798
ILMN_1783303	HSP90AB4P	0.405344615
ILMN_2364131	TTPAL	0.405335744
ILMN_1683692	LRCH1	0.405298274
ILMN_2395375	GABBR1	0.405263892
ILMN_1779034	NADSYN1	0.405249166
ILMN_1745385	HNRNPM	0.405216565
ILMN_1766593	PHRF1	0.405129581
ILMN_1683598	ACSL4	0.40507251
ILMN_1772959	PDCD7	0.404938384
ILMN_2404049	RBM38	0.404846124
ILMN_1712755	LRRC41	0.404624074
ILMN_3203126	LOC100131662	0.404609942
ILMN_1715636	EIF3B	0.404530213
ILMN_1659054	CHKB	0.404494928
ILMN_2374449	SPP1	0.404457264
ILMN_3243291	HNRNPA1	0.404438085
ILMN_1734742	ARHGDIA	0.404389914
ILMN_1664610	RPS17	0.404355237
ILMN_1708728	H2AFJ	0.404314257
ILMN_1789283	PPP2R5C	0.404298505
ILMN_1751444	NCAPG	0.404246717
ILMN_1670378	FAM115A	0.404146453
ILMN_1798543	STK17B	0.404092572
ILMN_1805410	C15orf48	0.404025984
ILMN_2133675	SGSH	0.40399417
ILMN_2297864	MTMR14	0.403989515
ILMN_1785161	CHCHD6	0.403986296
ILMN_2145280	DEF6	0.4039455
ILMN_2316386	GPBAR1	0.403898621
ILMN_1664828	APOBEC3H	0.403819571
ILMN_1343291	EEF1A1	0.403803862
ILMN_1686082	C14orf79	0.40373627
ILMN_1810531	DRG2	0.403721239
ILMN_2327244	CALCA	0.403681481
ILMN_1724532	C11orf49	0.403674215
ILMN_1743217	SNORD32A	0.403643742
ILMN_1674811	OASL	0.403598694
ILMN_1754303	RPL30	0.403591802
ILMN_1791144	PMS2L14	0.403523494
ILMN_2358457	ATF4	0.403474728
ILMN_3251536	FAM127C	0.403458741
ILMN_1724994	COL4A2	0.403373861
ILMN_2242463	CTSC	0.403366721
ILMN_1772387	TLR2	0.403325042
ILMN_3192156	LOC100130932	0.403192604
ILMN_1804863	FKBP15	0.403099948
ILMN_2251279	WDR45	0.40306563
ILMN_1794392	DDX3X	0.403050847
ILMN_1668465	KIAA1609	0.403042801
ILMN_1652929	POGZ	0.403036708
ILMN_1743199	EGR2	0.402939005
ILMN_2231608	CTAGE15P	0.402926558
ILMN_1651850	RPS16	0.402920932
ILMN_1692973	BICD1	0.402885937
ILMN_1744508	FAM53C	0.402883781
ILMN_1699545	PCSK7	0.402851248
ILMN_3263329	GAR1	0.402845042
ILMN_1800512	HMOX1	0.402787547
ILMN_1768260	GAS6	0.402666935
ILMN_1689456	ZBTB20	0.402666654
ILMN_1791483	PDE4D	0.402652414
ILMN_1717477	PSD3	0.402648169
ILMN_1715500	SS18L1	0.402618138
ILMN_1716704	NLRC5	0.402611189
ILMN_3238931	DEFB130	0.402542894
ILMN_1683194	DCN	0.402403818
ILMN_2415179	CLSTN1	0.402334922
ILMN_1767665	GPX8	0.402294945
ILMN_1764082	MBOAT1	0.40221169
ILMN_2121207	FAM125A	0.402174669
ILMN_1781099	ISY1	0.402108913
ILMN_1704500	STAP2	0.402034859
ILMN_1726589	CD248	0.401958747
ILMN_1666910	GAB1	0.401764262
ILMN_3229467	EBPL	0.401759333
ILMN_1685680	SPATA1	0.401698996
ILMN_1803788	LGALS3	0.401609945
ILMN_2366334	FERMT3	0.401603952

ILMN_1745583	EXOC1	0.401546342
ILMN_1658472	APH1A	0.401429831
ILMN_3243972	SNORA70B	0.401392847
ILMN_1671010	ZNF267	0.401383992
ILMN_1775004	KCNQ3	0.401376642
ILMN_1756992	MUC1	0.401361793
ILMN_1754145	CAPRIN1	0.401300493
ILMN_1680437	EEF1G	0.401277935
ILMN_2205999	OSMR	0.401239187
ILMN_2106167	RAP1GDS1	0.401085211
ILMN_1712357	HNRNPK	0.401044617
ILMN_1651642	GPC2	0.401004703
ILMN_3289352	RPL6	0.400913527
ILMN_1731648	FOXJ2	0.400880227
ILMN_2140895	hCG_1646325	0.400849638
ILMN_3187324	CNRIP1	0.400846679
ILMN_2336902	TRIM6	0.40084519
ILMN_2284941	ABCF2	0.40080859
ILMN_1723729	RSL1D1	0.400775187
ILMN_1757847	C11orf68	0.400694991
ILMN_1690546	PPP3CC	0.40028074
ILMN_1704290	SPTLC2	0.400275193
ILMN_1710756	ENO1	0.400251804
ILMN_1664028	CENPB	0.400174047
ILMN_1774287	CFB	0.400082623
ILMN_1727249	FAM21C	0.400079383
ILMN_1773868	ZRSR2	0.400058231
