# Supplementary material for: Intrarenal activation of adaptive immune effectors is associated with tubular damage and impaired renal function in lupus nephritis
Source: Ann Rheum Dis. 2018 Jul 31;77(12):1782–9. doi: 10.1136/annrheumdis-2018-213485 (PMC6241616; doi:10.1136/annrheumdis-2018-213485)
Supplement: Supplementary file 11 [file annrheumdis-2018-213485supp011.pdf]

Supplementary Table 8 : Transcripts and DAVID pathways correlating with quantitative evaluation of the SDC1 stain in lupus kidney biopsies

Positive correlation

Transcripts

Identifier	Gene Symbol	Correlation coefficient with SDC1
ILMN_3309544	MIR877	0.657242365
ILMN_1760199	CALB1	0.648070582
ILMN_1661416	LOC400958	0.644564297
ILMN_3237520	C6orf127	0.643753183
ILMN_3254147	NA	0.62940516
ILMN_1676822	C2orf40	0.613500711
ILMN_1724021	SLC6A19	0.610864834
ILMN_1693771	ASPH	0.610054077
ILMN_2069224	PVALB	0.608871774
ILMN_1749081	AUTS2	0.605854938
ILMN_1766377	PCDHB6	0.599821141
ILMN_1813350	HSD11B2	0.59942549
ILMN_2232036	EFCAB7	0.598356763
ILMN_1747697	CHML	0.597798225
ILMN_1702114	NOMO3	0.596449862
ILMN_1794597	ZNF167	0.590083387
ILMN_1845757	LOC100506136	0.590019194
ILMN_1727178	C11orf92	0.589992731
ILMN_1846630	BE439556	0.585631049
ILMN_2199439	CA2	0.583298098
ILMN_1775537	TRIM69	0.582725957
ILMN_1721392	PTPRQ	0.581516666
ILMN_3243111	LOC339240	0.57437053
ILMN_2351272	DZIP1	0.57225211
ILMN_1792075	CD22	0.572176906
ILMN_1850240	AW262671	0.571878013
ILMN_1684629	C20orf26	0.570755896
ILMN_2094631	TRIM60	0.568571793
ILMN_1706579	SHBG	0.567732437
ILMN_1777182	ACAN	0.563640508
ILMN_1726273	ATP6V1G3	0.562990495
ILMN_3241322	SLC34A3	0.562124345
ILMN_1808272	KCNJ10	0.559645009
ILMN_1910294	AA335815	0.55908461
ILMN_1785285	ABHD13	0.557856945
ILMN_1651501	ABCD4	0.557567054
ILMN_1915734	DA722162	0.555139175
ILMN_1735474	R3HCC1	0.554782714
ILMN_1839994	DB338252	0.554455409
ILMN_1712577	FAM174A	0.553252779
ILMN_1659631	LOC440905	0.553042012
ILMN_1720998	CA12	0.552419691
ILMN_1666512	SKIV2L	0.552274834
ILMN_1792317	SLCO1C1	0.552162331
ILMN_1668242	PHEX	0.552005252
ILMN_1672049	LOC387895	0.550515789
ILMN_1695969	TMPRSS15	0.548355577
ILMN_1724062	LIN54	0.547560842
ILMN_1746502	BC031979	0.546645494
ILMN_2371001	ATP6V1G3	0.546464043
ILMN_1876924	WNK1	0.545321691
ILMN_3308195	MIR618	0.54529389
ILMN_2276461	MAP2	0.544809465
ILMN_2133125	LRRC43	0.544711109
ILMN_1651403	TCRBV22S1A2N1T	0.544440276
ILMN_1789162	MAB21L2	0.543145142
ILMN_3249533	BDNF-AS1	0.542725292
ILMN_1715713	NFASC	0.542491978
ILMN_1801443	TSKU	0.54244717
ILMN_2341132	TNNT2	0.541910581
ILMN_2226324	BRP44L	0.540679506
ILMN_1760065	OR2T11	0.540188765
ILMN_1743040	LOC283104	0.538846465
ILMN_3240349	OCM2	0.536913684
ILMN_1651609	SPRED3	0.535954525
ILMN_1659352	KCNK13	0.535793069
ILMN_1886792	BF475239	0.535543624
ILMN_1880251	AI088492	0.531945129
ILMN_1807339	HRG	0.531944971
ILMN_3306997	METTL1	0.528915781
ILMN_2209180	RPRD1A	0.528814232
ILMN_1682857	NDUFAF2	0.528554966
ILMN_3242612	BI833415	0.528073619
ILMN_1812031	PALM	0.527797953
ILMN_2229379	KIT	0.526060377
ILMN_1659378	TFAP2D	0.524737621
ILMN_3200560	ARMCX4	0.523815791

Pathways

			Count	P_Value	Benjamini
**Annotation Cluster 1**	**Enrichment Score: 1.91**		**Count**	**P_Value**	**Benjamini**
	UP_SEQ_FEATURE	domain:Kazal-like	6	3.20E-03	1.00E+00
	INTERPRO	Proteinase inhibitor I1, Kazal	6	6.60E-03	7.50E-01
	SMART	KAZAL	6	6.90E-03	4.60E-01
	INTERPRO	Protease inhibitor, Kazal-type	3	1.50E-01	9.90E-01
**Annotation Cluster 2**	**Enrichment Score: 1.66**		**Count**	**P_Value**	**Benjamini**
	INTERPRO	EF-HAND 2	15	1.50E-03	7.20E-01
	INTERPRO	EF-Hand type	15	3.60E-03	7.80E-01
	UP_SEQ_FEATURE	domain:EF-hand 1	12	6.80E-03	9.90E-01
	UP_SEQ_FEATURE	domain:EF-hand 3	8	9.90E-03	9.90E-01
	INTERPRO	EF-HAND 1	13	1.20E-02	8.60E-01
	INTERPRO	Calcium-binding EF-hand	10	1.20E-02	8.30E-01
	SMART	EFh	10	1.30E-02	5.50E-01
	UP_SEQ_FEATURE	calcium-binding region:1	9	1.50E-02	1.00E+00
	SP_PIR_KEYWORDS	EF hand	6	1.70E-02	8.50E-01
	UP_SEQ_FEATURE	domain:EF-hand 2	11	1.80E-02	1.00E+00
	UP_SEQ_FEATURE	calcium-binding region:2	8	2.70E-02	1.00E+00
	GOTERM_MF_FAT	calcium ion binding	35	4.10E-02	9.40E-01
	SP_PIR_KEYWORDS	calcium	29	5.10E-02	7.00E-01
	UP_SEQ_FEATURE	domain:EF-hand 4	5	6.20E-02	1.00E+00
	UP_SEQ_FEATURE	calcium-binding region:3	4	6.20E-02	1.00E+00
	INTERPRO	EF hand	7	1.20E-01	9.80E-01
	SP_PIR_KEYWORDS	calcium binding	3	7.40E-01	9.90E-01
**Annotation Cluster 3**	**Enrichment Score: 1.41**		**Count**	**P_Value**	**Benjamini**
	GOTERM_MF_FAT	inorganic cation transmembrane transporter activity	13	8.60E-04	4.10E-01
	GOTERM_CC_FAT	proton-transporting two-sector ATPase complex	7	1.30E-03	3.70E-01
	GOTERM_MF_FAT	hydrogen ion transmembrane transporter activity	9	3.10E-03	6.10E-01
	GOTERM_BP_FAT	proton transport	7	4.60E-03	9.40E-01
	GOTERM_BP_FAT	hydrogen transport	7	5.40E-03	9.20E-01
	SP_PIR_KEYWORDS	Hydrogen ion transport	6	6.90E-03	7.80E-01
	GOTERM_MF_FAT	monovalent inorganic cation transmembrane transporter activity	9	7.40E-03	7.80E-01
	GOTERM_BP_FAT	purine ribonucleotide biosynthetic process	9	1.00E-02	9.40E-01
	GOTERM_CC_FAT	mitochondrial proton-transporting ATP synthase complex	4	1.40E-02	9.10E-01
	GOTERM_BP_FAT	ribonucleotide biosynthetic process	9	1.50E-02	9.10E-01
	GOTERM_BP_FAT	ATP metabolic process	8	1.80E-02	9.30E-01
	GOTERM_CC_FAT	proton-transporting ATP synthase complex	4	1.90E-02	8.00E-01
	GOTERM_BP_FAT	energy coupled proton transport, down electrochemical gradient	5	1.90E-02	9.20E-01
	GOTERM_BP_FAT	ATP synthesis coupled proton transport	5	1.90E-02	9.20E-01
	GOTERM_CC_FAT	mitochondrial membrane part	9	2.20E-02	7.10E-01
	SP_PIR_KEYWORDS	mitochondrion inner membrane	11	2.50E-02	7.00E-01
	GOTERM_BP_FAT	purine ribonucleotide metabolic process	9	2.60E-02	9.30E-01
	GOTERM_BP_FAT	ATP biosynthetic process	7	2.70E-02	9.30E-01
	GOTERM_BP_FAT	purine ribonucleoside triphosphate metabolic process	8	3.10E-02	9.40E-01
	GOTERM_BP_FAT	generation of precursor metabolites and energy	15	3.20E-02	9.30E-01
	GOTERM_BP_FAT	ribonucleoside triphosphate metabolic process	8	3.20E-02	9.30E-01
	KEGG_PATHWAY	Oxidative phosphorylation	9	3.40E-02	8.90E-01
	GOTERM_BP_FAT	monovalent inorganic cation transport	15	3.50E-02	9.30E-01
	GOTERM_BP_FAT	ribonucleotide metabolic process	9	3.60E-02	9.20E-01
	GOTERM_BP_FAT	ion transmembrane transport	5	3.60E-02	9.10E-01
	GOTERM_BP_FAT	purine nucleotide biosynthetic process	9	3.70E-02	9.10E-01
	GOTERM_BP_FAT	purine nucleoside triphosphate metabolic process	8	3.80E-02	9.00E-01
	GOTERM_BP_FAT	oxidative phosphorylation	7	4.00E-02	9.00E-01
	GOTERM_BP_FAT	purine ribonucleoside triphosphate biosynthetic process	7	4.00E-02	9.00E-01
	SP_PIR_KEYWORDS	atp synthesis	3	4.10E-02	7.30E-01
	GOTERM_BP_FAT	nitrogen compound biosynthetic process	15	4.10E-02	9.00E-01
	GOTERM_BP_FAT	purine nucleoside triphosphate biosynthetic process	7	4.20E-02	8.90E-01
	GOTERM_BP_FAT	ribonucleoside triphosphate biosynthetic process	7	4.20E-02	8.90E-01
	GOTERM_BP_FAT	nucleoside triphosphate biosynthetic process	7	4.70E-02	9.00E-01
	GOTERM_BP_FAT	nucleoside triphosphate metabolic process	8	5.20E-02	9.10E-01
	GOTERM_MF_FAT	cation-transporting ATPase activity	4	7.40E-02	9.60E-01
	GOTERM_CC_FAT	mitochondrial inner membrane	14	7.90E-02	9.00E-01
	GOTERM_MF_FAT	hydrolase activity, acting on acid anhydrides, catalyzing transmembrane movement of substances	7	8.20E-02	9.60E-01
	GOTERM_BP_FAT	purine nucleotide metabolic process	9	1.10E-01	9.70E-01
	GOTERM_BP_FAT	nucleotide biosynthetic process	9	1.10E-01	9.70E-01
	GOTERM_MF_FAT	P-P-bond-hydrolysis-driven transmembrane transporter activity	7	1.20E-01	9.60E-01
	GOTERM_MF_FAT	primary active transmembrane transporter activity	7	1.20E-01	9.60E-01
	GOTERM_CC_FAT	organelle inner membrane	14	1.20E-01	8.90E-01
	GOTERM_BP_FAT	nucleobase, nucleoside, nucleotide and nucleic acid biosynthetic process	9	1.20E-01	9.80E-01
	GOTERM_BP_FAT	nucleobase, nucleoside and nucleotide biosynthetic process	9	1.20E-01	9.80E-01
	GOTERM_CC_FAT	proton-transporting two-sector ATPase complex, proton-transporting domain	3	1.40E-01	9.10E-01
	GOTERM_MF_FAT	ATPase activity, coupled to transmembrane movement of ions	5	1.40E-01	9.60E-01
	GOTERM_MF_FAT	ATPase activity, coupled to transmembrane movement of substances	6	1.80E-01	9.80E-01
	GOTERM_MF_FAT	ATPase activity, coupled to movement of substances	6	1.80E-01	9.70E-01
	GOTERM_MF_FAT	ATPase activity	13	2.00E-01	9.80E-01
	GOTERM_BP_FAT	sensory perception of sound	5	2.40E-01	9.90E-01
	GOTERM_BP_FAT	sensory perception of mechanical stimulus	5	2.70E-01	9.90E-01
	GOTERM_MF_FAT	ATPase activity, coupled to transmembrane movement of ions, phosphorylative mechanism	3	4.20E-01	1.00E+00
	GOTERM_MF_FAT	ATPase activity, coupled	9	4.60E-01	1.00E+00

Negative correlation

Transcripts

Identifier	Gene Symbol	Correlation coefficient with SDC1
ILMN_1693009	FGL2	-0.400117445
ILMN_1739222	ETV5	-0.40012701
ILMN_2309848	FXYD5	-0.400160778
ILMN_1797875	ALOX5AP	-0.400421549
ILMN_1815895	HLA-DRB1	-0.400434105
ILMN_1713829	PTGES	-0.400477832
ILMN_1747943	ZNF670	-0.400517268
ILMN_1728236	SLFN12	-0.400528006
ILMN_3243333	GAGE8	-0.400608873
ILMN_1809417	LRFN4	-0.400614983
ILMN_2179083	LOXL4	-0.401130036
ILMN_3286131	AL359580	-0.401163232
ILMN_3248505	EMID2	-0.401266389
ILMN_2408139	CDC42	-0.401300008
ILMN_1714709	OLFM1	-0.401354158
ILMN_2174341	GPR83	-0.401434792
ILMN_2160750	TTPA	-0.401450186
ILMN_1670676	TBRG1	-0.40172899
ILMN_2381037	LIMS1	-0.401750965
ILMN_1758250	TRAFD1	-0.401968247
ILMN_1710734	GZMK	-0.402063601
ILMN_1680102	C11orf45	-0.402352723
ILMN_1671898	LMX1A	-0.402355085
ILMN_2277077	PRKAR1A	-0.402696129
ILMN_2172269	TMEM183B	-0.402722715
ILMN_2065254	ELAVL4	-0.402947206
ILMN_1683215	ZBED3	-0.40299364
ILMN_1668417	WIPF1	-0.403038978
ILMN_1749424	C21orf122	-0.403050896
ILMN_1734021	CLSTN3	-0.403245085
ILMN_2408877	MLL3	-0.403529226
ILMN_1661236	GLYCAM1	-0.403560897
ILMN_3287855	TRIM16L	-0.403760453
ILMN_1732060	ARHGAP1	-0.403814171
ILMN_1747775	STX2	-0.403839758
ILMN_3241838	FLJ00326	-0.404105684
ILMN_2077952	GALNTL1	-0.404269043
ILMN_3235617	WASH2P	-0.40429643
ILMN_1721116	USP10	-0.404360771
ILMN_1857069	KIAA1147	-0.404476404
ILMN_1752280	FAM131B	-0.404502323
ILMN_1702684	MTRR	-0.404713536
ILMN_1674519	ZP2	-0.40476552
ILMN_2308582	CYB5R3	-0.404949778
ILMN_1813114	IDH3GL	-0.405055265
ILMN_1679195	C20orf24	-0.40516112
ILMN_1801349	TAS2R14	-0.405196864
ILMN_1687335	FLNA	-0.405294402
ILMN_1768470	EIF4G1	-0.405570124
ILMN_1780302	DYNC1H1	-0.405673101
ILMN_2109994	MIA-RAB4B	-0.405765655
ILMN_1685625	UCP2	-0.405801711
ILMN_1725666	GTF2H3	-0.406073522
ILMN_1813131	KRT8	-0.406222246
ILMN_1787844	C19orf10	-0.406283696
ILMN_1708167	CDCP1	-0.406363427
ILMN_2327346	SSBP4	-0.406563586
ILMN_1811049	POU2AF1	-0.406564013
ILMN_1805535	VRK3	-0.406806443
ILMN_1786065	hNp95	-0.406968942
ILMN_2371119	EPHA6	-0.407024468
ILMN_1719759	TNC	-0.407319633
ILMN_1760305	FBRSL1	-0.407330938
ILMN_1760029	HOXC12	-0.407392567
ILMN_2203271	FPR3	-0.40772581
ILMN_2261600	FCGR1B	-0.407972381
ILMN_2066756	NCAPG2	-0.408008085
ILMN_3204619	GOLGA6L5	-0.408034507
ILMN_1689786	ASMTL	-0.408192912
ILMN_1776709	LOC653075	-0.408231226
ILMN_2370296	ENAH	-0.408359162
ILMN_3226059	MYCNOS	-0.408488464
ILMN_1656715	THSD7A	-0.408589978
ILMN_1783998	STARD5	-0.408667901
ILMN_1761733	HLA-DMB	-0.408733744
ILMN_3250899	SLX1B-SULT1A4	-0.408764673
ILMN_3228688	HLA-DRB1	-0.408879533

Pathways

			Count	P_Value	Benjamini
**Annotation Cluster 1**	**Enrichment Score: 5.83**		**Count**	**P_Value**	**Benjamini**
	SP_PIR_KEYWORDS	signal	126	9.60E-08	3.80E-05
	UP_SEQ_FEATURE	signal peptide	126	1.20E-07	2.00E-04
	SP_PIR_KEYWORDS	disulfide bond	112	1.20E-06	1.70E-04
	UP_SEQ_FEATURE	disulfide bond	109	1.50E-06	1.20E-03
	SP_PIR_KEYWORDS	Secreted	74	1.50E-06	1.50E-04
	SP_PIR_KEYWORDS	glycoprotein	151	1.90E-06	1.50E-04
	GOTERM_CC_FAT	extracellular region	85	1.70E-05	5.80E-03
	UP_SEQ_FEATURE	glycosylation site:N-linked (GlcNAc...)	141	2.30E-05	7.30E-03
**Annotation Cluster 2**	**Enrichment Score: 3.32**		**Count**	**P_Value**	**Benjamini**
	SP_PIR_KEYWORDS	cell division	20	3.20E-05	1.60E-03
	SP_PIR_KEYWORDS	cell cycle	28	3.20E-05	1.40E-03
	SP_PIR_KEYWORDS	mitosis	16	5.00E-05	2.00E-03
	GOTERM_BP_FAT	mitotic cell cycle	24	1.50E-04	5.80E-02
	GOTERM_BP_FAT	cell cycle	37	8.10E-04	1.70E-01
	GOTERM_BP_FAT	nuclear division	16	8.20E-04	1.50E-01
	GOTERM_BP_FAT	mitosis	16	8.20E-04	1.50E-01
	GOTERM_BP_FAT	cell division	19	9.50E-04	1.50E-01
	GOTERM_BP_FAT	M phase of mitotic cell cycle	16	9.90E-04	1.40E-01
	GOTERM_BP_FAT	organelle fission	16	1.20E-03	1.60E-01
	GOTERM_BP_FAT	cell cycle phase	23	1.70E-03	2.00E-01
	GOTERM_BP_FAT	cell cycle process	28	2.40E-03	2.50E-01
	GOTERM_BP_FAT	M phase	19	3.20E-03	2.70E-01
**Annotation Cluster 3**	**Enrichment Score: 3.14**		**Count**	**P_Value**	**Benjamini**
	SP_PIR_KEYWORDS	extracellular matrix	20	9.00E-06	5.20E-04
	GOTERM_CC_FAT	collagen	7	3.10E-04	3.50E-02
	GOTERM_CC_FAT	extracellular matrix part	12	3.20E-04	2.70E-02
	GOTERM_CC_FAT	extracellular matrix	22	4.50E-04	3.10E-02
	GOTERM_CC_FAT	proteinaceous extracellular matrix	20	1.10E-03	5.40E-02
	GOTERM_MF_FAT	extracellular matrix structural constituent	9	2.00E-03	2.00E-01
	GOTERM_CC_FAT	basement membrane	8	5.00E-03	1.80E-01
	SP_PIR_KEYWORDS	basement membrane	5	1.50E-02	1.60E-01
**Annotation Cluster 4**	**Enrichment Score: 3.02**		**Count**	**P_Value**	**Benjamini**
	SP_PIR_KEYWORDS	immune response	22	1.80E-07	3.60E-05
	UP_SEQ_FEATURE	domain:Ig-like C1-type	9	5.10E-06	2.10E-03
	KEGG_PATHWAY	Viral myocarditis	12	6.50E-06	8.20E-04
	GOTERM_BP_FAT	antigen processing and presentation	12	1.20E-05	1.20E-02
	KEGG_PATHWAY	Systemic lupus erythematosus	13	3.20E-05	2.00E-03
	UP_SEQ_FEATURE	region of interest:Connecting peptide	8	3.50E-05	9.50E-03
	INTERPRO	Immunoglobulin/major histocompatibility complex, conserved site	11	7.00E-05	2.70E-02
	KEGG_PATHWAY	Allograft rejection	8	7.50E-05	3.10E-03
	GOTERM_BP_FAT	antigen processing and presentation of peptide antigen	7	7.80E-05	5.10E-02
	KEGG_PATHWAY	Autoimmune thyroid disease	9	1.10E-04	3.60E-03
	KEGG_PATHWAY	Graft-versus-host disease	8	1.30E-04	3.20E-03
	GOTERM_CC_FAT	MHC protein complex	9	1.40E-04	2.30E-02
	KEGG_PATHWAY	Antigen processing and presentation	11	1.60E-04	3.30E-03
	GOTERM_BP_FAT	antigen processing and presentation of peptide or polysaccharide antigen via MHC class II	7	2.10E-04	6.60E-02
	KEGG_PATHWAY	Type I diabetes mellitus	8	2.10E-04	3.70E-03
	INTERPRO	Immunoglobulin C1-set	9	3.20E-04	6.00E-02
	BIOCARTA	Antigen Processing and Presentation	5	5.40E-04	6.70E-02
	GOTERM_MF_FAT	MHC class I protein binding	5	7.00E-04	9.10E-02
	SP_PIR_KEYWORDS	mhc ii	6	8.90E-04	1.60E-02
	SP_PIR_KEYWORDS	heterodimer	10	1.00E-03	1.70E-02
	SMART	IGc1	9	1.20E-03	2.30E-01
	UP_SEQ_FEATURE	region of interest:Alpha-1	5	1.30E-03	2.10E-01
	UP_SEQ_FEATURE	region of interest:Alpha-2	5	1.30E-03	2.10E-01
	KEGG_PATHWAY	Asthma	6	1.50E-03	1.90E-02
	KEGG_PATHWAY	Cell adhesion molecules (CAMs)	12	1.80E-03	2.10E-02
	INTERPRO	MHC class II, alpha/beta chain, N-terminal	5	2.10E-03	2.80E-01
	KEGG_PATHWAY	Intestinal immune network for IgA production	7	3.10E-03	3.20E-02
	PIR_SUPERFAMILY	PIRSF001991:class II histocompatibility antigen	5	5.90E-03	7.50E-01
	GOTERM_CC_FAT	MHC class II protein complex	5	7.30E-03	2.20E-01
	GOTERM_MF_FAT	MHC class II receptor activity	4	1.30E-02	6.50E-01
	GOTERM_CC_FAT	TAP complex	3	1.40E-02	3.60E-01
	GOTERM_CC_FAT	MHC class I peptide loading complex	3	2.30E-02	4.90E-01
	SP_PIR_KEYWORDS	mhc i	3	2.40E-02	2.20E-01
	INTERPRO	MHC class II, alpha chain, N-terminal	3	3.60E-02	9.30E-01
	GOTERM_CC_FAT	MHC class I protein complex	4	3.90E-02	6.30E-01
	BIOCARTA	The Co-Stimulatory Signal During T-cell Activation	4	4.50E-02	9.50E-01
	GOTERM_BP_FAT	antigen processing and presentation of peptide antigen via MHC class I	3	7.40E-02	8.80E-01
	INTERPRO	MHC class I, alpha chain, alpha1 and alpha2	3	1.50E-01	9.80E-01
	INTERPRO	MHC class I-like antigen recognition	3	1.60E-01	9.80E-01
	BIOCARTA	Th1/Th2 Differentiation	3	2.10E-01	9.50E-01
**Annotation Cluster 5**	**Enrichment Score: 2.61**		**Count**	**P_Value**	**Benjamini**
	GOTERM_MF_FAT	MHC protein binding	7	4.00E-05	1.10E-02
	GOTERM_BP_FAT	antigen processing and presentation of peptide antigen	7	7.80E-05	5.10E-02
	GOTERM_BP_FAT	antigen processing and presentation of exogenous peptide antigen	5	1.40E-04	6.80E-02
	GOTERM_BP_FAT	antigen processing and presentation of peptide or polysaccharide antigen via MHC class II	7	2.10E-04	6.60E-02

ILMN_2240009	ADSSL1	0.52210583
ILMN_2227368	SELT	0.52173568
ILMN_1688138	ZNF713	0.521301671
ILMN_1853193	BX090577	0.520942222
ILMN_1712836	C6orf27	0.520733231
ILMN_1750880	LOC196394	0.520050659
ILMN_1831098	BX092776	0.519939549
ILMN_1725157	NEK6	0.519594586
ILMN_1795304	CRLF2	0.519356063
ILMN_1766736	BPI	0.519001413
ILMN_1696189	KCNAB1	0.51842538
ILMN_1744676	RNF112	0.518000226
ILMN_2173909	ZNF14	0.517416659
ILMN_1812503	PHKA1	0.51734871
ILMN_2357015	GRK6	0.516521115
ILMN_1874814	AB231761	0.516435353
ILMN_1706200	MED15	0.515772641
ILMN_1685441	ASAP3	0.515125115
ILMN_1888335	CB305835	0.515102206
ILMN_1790603	ATPAF1	0.514053492
ILMN_1777845	NOTCH2	0.51405217
ILMN_3243312	ZC3H12D	0.513972814
ILMN_3305763	LOC728460	0.513482705
ILMN_1760466	AP4S1	0.512227511
ILMN_2339825	YME1L1	0.511893709
ILMN_1689237	BVES	0.511362151
ILMN_2125395	GPR128	0.510486345
ILMN_2120695	TSPAN7	0.509944111
ILMN_3273898	NA	0.509136913
ILMN_1718142	TRIM42	0.509021514
ILMN_1780996	ETAA1	0.508959589
ILMN_1710722	ZNF474	0.507937804
ILMN_1776493	MTUS1	0.507556965
ILMN_2184575	CCNI2	0.507492779
ILMN_1716639	PRHOXNB	0.505370651
ILMN_2406634	DNMT3L	0.504159519
ILMN_1812678	AGMO	0.504111789
ILMN_2268068	MAPKAP1	0.503239001
ILMN_1729650	PEX7	0.503159622
ILMN_1759579	ZNF91	0.502035141
ILMN_2245292	TGIF1	0.501750297
ILMN_2071186	CORT	0.501726346
ILMN_1714121	PRMT8	0.501652683
ILMN_1732808	TOX3	0.500746745
ILMN_2166457	HPGD	0.500591921
ILMN_3249444	POM121-like 1	0.500434706
ILMN_1739429	HS6ST2	0.500390756
ILMN_3297880	SEC13	0.500305747
ILMN_1779778	PRR19	0.499753671
ILMN_3248932	KCNV2	0.499439456
ILMN_1729251	MYH4	0.497711323
ILMN_2043306	EPB41L5	0.497514107
ILMN_3247055	POM121-like 1	0.497354603
ILMN_3233838	LOC399940	0.49731636
ILMN_1675425	OR4D1	0.497017646
ILMN_1685496	RGS7	0.496898627
ILMN_3261111	IP6K3	0.496330047
ILMN_1814464	RAMP2	0.495968428
ILMN_1809291	TSPAN7	0.495083036
ILMN_1768719	RDH11	0.494976195
ILMN_1662935	C1QTNF7	0.494922178
ILMN_2369953	ARL4A	0.494825354
ILMN_2380754	CCNG1	0.494418375
ILMN_1742534	COL4A5	0.494339582
ILMN_1729060	ARHGEF5	0.493824817
ILMN_1791409	ITGA7	0.493507984
ILMN_1838581	AI217658	0.492998396
ILMN_1802942	AJ292264	0.491908571
ILMN_1676973	CABP5	0.491850015
ILMN_1761511	APOH	0.491620301
ILMN_1813964	SLC6A18	0.491459307
ILMN_3247844	C4orf10	0.491280432
ILMN_1774757	ZNF496	0.490860215
ILMN_1703679	FCGR3A	0.490420219
ILMN_1689241	RAD17	0.489931472
ILMN_1694734	RUNX1T1	0.488775952
ILMN_2218104	PAH	0.488767145
ILMN_1802223	ATP2B2	0.488401735
ILMN_1771488	OR12D2	0.488237961
ILMN_3310548	MIR889	0.487701946
ILMN_1742272	REN	0.487599373
ILMN_1715255	MAGI2	0.485905355
ILMN_1748352	CTSL2	0.485487842
ILMN_1669846	SLC25A31	0.485360874
ILMN_1760047	VAX2	0.485351811
ILMN_1676005	KPNA1	0.485296039
ILMN_1688633	CLCN2	0.484897848
ILMN_1877421	BX092644	0.484765506
ILMN_1653386	SULT6B1	0.484704
ILMN_1715113	HARS2	0.4844395
ILMN_1762993	PIGV	0.484244706
ILMN_1762531	FGF9	0.48419459
ILMN_1660698	GTPBP8	0.483650204

**Annotation Cluster 4**	**Enrichment Score: 1.3**		**Count**	**P_Value**	**Benjamini**
	GOTERM_MF_FAT	solute:solute antiporter activity	6	1.50E-02	9.00E-01
	INTERPRO	Sulphate anion transporter, conserved site	3	2.60E-02	9.20E-01
	UP_SEQ_FEATURE	domain:STAS	3	3.00E-02	1.00E+00
	INTERPRO	Sulphate transporter/antisigma-factor antagonist STAS	3	3.20E-02	9.30E-01
	INTERPRO	Sulphate transporter	3	3.20E-02	9.30E-01
	GOTERM_BP_FAT	inorganic anion transport	7	3.20E-02	9.20E-01
	GOTERM_MF_FAT	antiporter activity	6	3.70E-02	9.80E-01
	GOTERM_MF_FAT	sulfate transmembrane transporter activity	3	4.10E-02	9.60E-01
	GOTERM_BP_FAT	sulfate transport	3	5.50E-02	9.10E-01
	GOTERM_BP_FAT	anion transport	8	7.50E-02	9.30E-01
	GOTERM_MF_FAT	anion:anion antiporter activity	3	1.00E-01	9.70E-01
	GOTERM_MF_FAT	anion transmembrane transporter activity	7	2.10E-01	9.80E-01
	GOTERM_MF_FAT	inorganic anion transmembrane transporter activity	3	2.30E-01	9.80E-01
**Annotation Cluster 5**	**Enrichment Score: 1.23**		**Count**	**P_Value**	**Benjamini**
	GOTERM_CC_FAT	proton-transporting two-sector ATPase complex	7	1.30E-03	3.70E-01
	GOTERM_BP_FAT	proton transport	7	4.60E-03	9.40E-01
	GOTERM_BP_FAT	hydrogen transport	7	5.40E-03	9.20E-01
	SP_PIR_KEYWORDS	Hydrogen ion transport	6	6.90E-03	7.80E-01
	GOTERM_CC_FAT	vacuolar proton-transporting V-type ATPase complex	3	2.90E-02	7.60E-01
	KEGG_PATHWAY	Oxidative phosphorylation	9	3.40E-02	8.90E-01
	GOTERM_MF_FAT	hydrolase activity, acting on acid anhydrides, catalyzing transmembrane movement of substances	7	8.20E-02	9.60E-01
	GOTERM_CC_FAT	proton-transporting V-type ATPase complex	3	1.00E-01	9.00E-01
	GOTERM_CC_FAT	vacuolar membrane	3	4.60E-01	9.60E-01
	KEGG_PATHWAY	Vibrio cholerae infection	3	4.90E-01	9.90E-01
	GOTERM_CC_FAT	vacuolar part	3	5.30E-01	9.80E-01
	KEGG_PATHWAY	Epithelial cell signaling in Helicobacter pylori infection	3	5.90E-01	9.90E-01
	GOTERM_CC_FAT	vacuole	6	8.20E-01	1.00E+00
**Annotation Cluster 6**	**Enrichment Score: 1.17**		**Count**	**P_Value**	**Benjamini**
	GOTERM_BP_FAT	behavioral defense response	3	5.50E-02	9.10E-01
	GOTERM_BP_FAT	behavioral fear response	3	5.50E-02	9.10E-01
	GOTERM_BP_FAT	multicellular organismal response to stress	4	7.80E-02	9.30E-01
	GOTERM_BP_FAT	fear response	3	8.40E-02	9.40E-01
**Annotation Cluster 7**	**Enrichment Score: 1.08**		**Count**	**P_Value**	**Benjamini**
	INTERPRO	FERM, C-terminal PH-like domain	4	2.60E-02	9.40E-01
	INTERPRO	Band 4.1 subgroup	4	2.60E-02	9.40E-01
	INTERPRO	FERM, N-terminal	4	4.20E-02	9.60E-01
	INTERPRO	FERM/acyl-CoA-binding protein, 3-helical bundle	4	1.00E-01	9.70E-01
	UP_SEQ_FEATURE	domain:FERM	4	1.20E-01	1.00E+00
	INTERPRO	FERM central domain	4	1.20E-01	9.90E-01
	INTERPRO	FERM domain	4	1.20E-01	9.90E-01
	INTERPRO	FERM conserved site	4	1.40E-01	9.90E-01
	INTERPRO	Band 4.1 domain	4	1.40E-01	9.90E-01
	SMART	B41	4	1.40E-01	9.60E-01
**Annotation Cluster 8**	**Enrichment Score: 1.08**		**Count**	**P_Value**	**Benjamini**
	GOTERM_BP_FAT	regulation of transforming growth factor beta receptor signaling pathway	5	1.70E-02	9.30E-01
	GOTERM_BP_FAT	negative regulation of transforming growth factor beta receptor signaling pathway	3	6.90E-02	9.30E-01
	GOTERM_BP_FAT	negative regulation of cell communication	10	1.90E-01	9.90E-01
	GOTERM_BP_FAT	negative regulation of signal transduction	9	2.10E-01	9.90E-01
**Annotation Cluster 9**	**Enrichment Score: 1.07**		**Count**	**P_Value**	**Benjamini**
	GOTERM_CC_FAT	extracellular matrix part	9	1.50E-02	8.20E-01
	GOTERM_CC_FAT	basement membrane	7	2.00E-02	7.50E-01
	SP_PIR_KEYWORDS	hydroxylation	6	3.30E-02	7.30E-01
	SP_PIR_KEYWORDS	extracellular matrix	12	4.30E-02	6.90E-01
	GOTERM_CC_FAT	proteinaceous extracellular matrix	15	5.80E-02	9.00E-01
	GOTERM_BP_FAT	extracellular structure organization	9	5.90E-02	9.10E-01
	GOTERM_MF_FAT	extracellular matrix structural constituent	6	8.50E-02	9.60E-01
	SP_PIR_KEYWORDS	collagen	6	9.20E-02	7.40E-01
	GOTERM_CC_FAT	extracellular matrix	15	9.30E-02	9.10E-01
	UP_SEQ_FEATURE	region of interest:Triple-helical region	3	1.10E-01	1.00E+00
	INTERPRO	Collagen triple helix repeat	5	1.70E-01	9.90E-01
	GOTERM_CC_FAT	collagen	3	2.50E-01	9.50E-01
	SP_PIR_KEYWORDS	basement membrane	3	2.60E-01	8.80E-01
	GOTERM_BP_FAT	extracellular matrix organization	3	7.50E-01	1.00E+00
**Annotation Cluster 10**	**Enrichment Score: 1.03**		**Count**	**P_Value**	**Benjamini**
	GOTERM_BP_FAT	dicarboxylic acid transport	3	5.50E-02	9.10E-01
	GOTERM_MF_FAT	dicarboxylic acid transmembrane transporter activity	3	6.90E-02	9.60E-01
	GOTERM_MF_FAT	anion transmembrane transporter activity	7	2.10E-01	9.80E-01
**Annotation Cluster 11**	**Enrichment Score: 1.01**		**Count**	**P_Value**	**Benjamini**
	UP_SEQ_FEATURE	domain:Laminin G-like 1	5	5.80E-03	1.00E+00
	UP_SEQ_FEATURE	domain:Laminin G-like 2	5	5.80E-03	1.00E+00
	INTERPRO	Laminin G	6	6.00E-03	8.10E-01
	SMART	LamG	6	6.30E-03	6.80E-01
	INTERPRO	Laminin G, subdomain 2	5	2.30E-02	9.40E-01
	INTERPRO	Concanavalin A-like lectin/glucanase, subgroup	5	1.40E-01	9.90E-01
	SP_PIR_KEYWORDS	egf-like domain	9	2.30E-01	8.80E-01
	INTERPRO	EGF	6	2.40E-01	1.00E+00
	INTERPRO	EGF-like region, conserved site	11	2.40E-01	9.90E-01
	UP_SEQ_FEATURE	domain:EGF-like	4	3.00E-01	1.00E+00
	INTERPRO	EGF-like	7	4.30E-01	1.00E+00
	SMART	EGF	7	4.40E-01	9.90E-01
	INTERPRO	EGF-type aspartate/asparagine hydroxylation conserved site	4	4.60E-01	1.00E+00
	INTERPRO	EGF-like, type 3	6	5.70E-01	1.00E+00
	UP_SEQ_FEATURE	domain:EGF-like 1	3	8.10E-01	1.00E+00
**Annotation Cluster 12**	**Enrichment Score: 1**		**Count**	**P_Value**	**Benjamini**
	GOTERM_BP_FAT	carboxylic acid transport	10	1.40E-02	9.40E-01
	GOTERM_BP_FAT	organic acid transport	10	1.40E-02	9.30E-01
	GOTERM_BP_FAT	amino acid transport	7	2.80E-02	9.30E-01
	GOTERM_BP_FAT	amine transport	8	3.20E-02	9.30E-01
	GOTERM_MF_FAT	solute:sodium symporter activity	5	5.20E-02	9.50E-01
	PIR_SUPERFAMILY	PIRSF002444:gamma-aminobutyric acid transporter	3	8.30E-02	1.00E+00
	INTERPRO	Sodium	3	9.40E-02	9.70E-01
	GOTERM_MF_FAT	neurotransmitter:sodium symporter activity	3	1.00E-01	9.70E-01

ILMN_1673566	ADAMTS1	-0.408974653
ILMN_3257482	LOC100128130	-0.409003089
ILMN_1724533	LY96	-0.40917178
ILMN_2056479	GLRX3	-0.409293599
ILMN_1654319	HAPLN3	-0.409299232
ILMN_1748018	GORASP2	-0.409438114
ILMN_1774238	R3HDML	-0.409441704
ILMN_1786707	C19orf63	-0.409721645
ILMN_1653828	CHFR	-0.409790619
ILMN_1721732	GSDMC	-0.410017689
ILMN_1747016	CEP55	-0.41009031
ILMN_1799120	WFDC2	-0.410161895
ILMN_2338323	CDC25B	-0.41025237
ILMN_3307134	FLJ40448	-0.410427248
ILMN_1651354	SPP1	-0.410613824
ILMN_1782704	CD19	-0.411072918
ILMN_2140059	LAMA4	-0.411258032
ILMN_1673108	AP2A1	-0.411338253
ILMN_1749829	DLGAP5	-0.411579084
ILMN_1694367	SNORD35B	-0.411982364
ILMN_1767662	LASS6	-0.412240339
ILMN_1735573	DPH1	-0.412347115
ILMN_1691080	OR6B1	-0.412449334
ILMN_1669842	CHAF1A	-0.412560358
ILMN_1754894	C1orf162	-0.412877309
ILMN_1705991	SMA4	-0.41291435
ILMN_1719103	UBE2CBP	-0.412941567
ILMN_1731231	ERN1	-0.413149036
ILMN_1752150	LOC100129408	-0.413157875
ILMN_2203950	HLA-A	-0.413234465
ILMN_1758487	CNPY4	-0.413423985
ILMN_1705522	SPAG1	-0.413506693
ILMN_1801864	NTAN1	-0.413656285
ILMN_1767377	SMA4	-0.413926909
ILMN_1665457	CLEC4C	-0.4142894
ILMN_2285141	USP48	-0.414428249
ILMN_1790891	CKAP4	-0.414505047
ILMN_2345353	LST1	-0.414524556
ILMN_1665985	SPDYE7P	-0.414739232
ILMN_1734773	PRSS1	-0.414839227
ILMN_2148913	TMEM45A	-0.414937811
ILMN_2395711	ARRB2	-0.414953191
ILMN_1683441	NCAPD3	-0.415203138
ILMN_1731907	LOC349114	-0.415465219
ILMN_1754969	LMCD1	-0.415467053
ILMN_1798006	ANKRD35	-0.415643444
ILMN_1699854	GRIN3B	-0.41569116
ILMN_2380850	SDCCAG3	-0.415857814
ILMN_2180171	SLC18A3	-0.416060468
ILMN_1739170	TM4SF18	-0.416585603
ILMN_1848913	AF339771	-0.416624256
ILMN_1724994	COL4A2	-0.416646449
ILMN_1677589	hCG_1818547	-0.416829592
ILMN_1711899	ANXA2	-0.417077867
ILMN_1777487	ZNF839	-0.417176145
ILMN_1736863	TMEM140	-0.417199709
ILMN_1662932	LCP1	-0.41727338
ILMN_3248562	SNORD49A	-0.417712537
ILMN_3211199	ZNF316	-0.417787591
ILMN_1664691	DAPP1	-0.417892802
ILMN_1768293	NUP155	-0.417913879
ILMN_1756091	C14orf184	-0.418137147
ILMN_1792404	TM4SF4	-0.418298984
ILMN_2228035	HS3ST1	-0.418311987
ILMN_1719517	CTTNBP2NL	-0.418473853
ILMN_1792344	LOC653075	-0.418677219
ILMN_1706154	ICK	-0.419001971
ILMN_1790518	PHF16	-0.419111786
ILMN_2205405	HLA-DPB1	-0.419299909
ILMN_1659511	NA	-0.419317509
ILMN_1660669	LOC653075	-0.419399147
ILMN_1766675	CDH6	-0.419428079
ILMN_3235027	TAF1D	-0.419435921
ILMN_1669376	DRAM1	-0.419810111
ILMN_1752953	BCL2L12	-0.419832393
ILMN_1764329	C21orf59	-0.419953322
ILMN_1669502	E2F3	-0.420068903
ILMN_1769550	SLFN5	-0.420078831
ILMN_1687867	KRT8	-0.420191175
ILMN_1796229	FGFR1	-0.420239031
ILMN_3226669	TIMM23	-0.420340481
ILMN_2177413	TBL3	-0.42057366
ILMN_2363591	SDCBP	-0.420609789
ILMN_1777658	SCARF1	-0.420862169
ILMN_1788955	PDLIM1	-0.421013923
ILMN_1703864	SFTA2	-0.42122854
ILMN_3246470	OR7E91P	-0.421510211
ILMN_1706708	EPPK1	-0.421630765
ILMN_1787179	MEGF11	-0.421838445
ILMN_1681542	HIST1H4E	-0.421855961
ILMN_1714730	UBE2C	-0.422048348
ILMN_3304898	TUBB	-0.422235717
ILMN_1721022	SHC1	-0.422535302

	GOTERM_BP_FAT	antigen processing and presentation of exogenous antigen	5	4.00E-04	1.10E-01
	GOTERM_BP_FAT	antigen processing and presentation of exogenous peptide antigen via MHC class II	4	9.50E-04	1.60E-01
	GOTERM_BP_FAT	antigen processing and presentation of peptide antigen via MHC class II	4	9.50E-04	1.60E-01
	GOTERM_BP_FAT	lymphocyte mediated immunity	8	2.50E-03	2.50E-01
	GOTERM_BP_FAT	immune effector process	11	3.10E-03	2.80E-01
	GOTERM_BP_FAT	leukocyte mediated immunity	8	7.90E-03	4.60E-01
	GOTERM_BP_FAT	immunoglobulin mediated immune response	6	1.40E-02	5.90E-01
	GOTERM_BP_FAT	positive regulation of immune response	10	1.50E-02	6.00E-01
	GOTERM_BP_FAT	B cell mediated immunity	6	1.60E-02	6.10E-01
	GOTERM_BP_FAT	adaptive immune response based on somatic recombination of immune receptors built from immunoglobulin superfamily domains	7	1.70E-02	6.10E-01
	GOTERM_BP_FAT	adaptive immune response	7	1.70E-02	6.10E-01
	GOTERM_BP_FAT	positive regulation of immune system process	13	2.60E-02	7.50E-01
	GOTERM_BP_FAT	positive regulation of response to stimulus	11	1.00E-01	9.00E-01
**Annotation Cluster 6**	**Enrichment Score: 2.18**		**Count**	**P_Value**	**Benjamini**
	SP_PIR_KEYWORDS	triple helix	8	8.30E-06	5.60E-04
	SP_PIR_KEYWORDS	hydroxylysine	7	9.50E-05	3.20E-03
	SP_PIR_KEYWORDS	collagen	11	1.20E-04	3.30E-03
	SP_PIR_KEYWORDS	hydroxyproline	7	2.60E-04	7.00E-03
	GOTERM_CC_FAT	collagen	7	3.10E-04	3.50E-02
	GOTERM_CC_FAT	extracellular matrix part	12	3.20E-04	2.70E-02
	SP_PIR_KEYWORDS	trimer	6	3.80E-04	9.50E-03
	KEGG_PATHWAY	ECM-receptor interaction	10	8.20E-04	1.10E-02
	UP_SEQ_FEATURE	glycosylation site:O-linked (Gal...)	4	1.10E-03	2.10E-01
	SP_PIR_KEYWORDS	hydroxylation	8	1.80E-03	2.80E-02
	GOTERM_MF_FAT	extracellular matrix structural constituent	9	2.00E-03	2.00E-01
	GOTERM_MF_FAT	platelet-derived growth factor binding	4	2.60E-03	2.10E-01
	GOTERM_CC_FAT	fibrillar collagen	4	3.60E-03	1.40E-01
	INTERPRO	Collagen triple helix repeat	8	5.50E-03	4.60E-01
	SP_PIR_KEYWORDS	skin	3	5.80E-03	7.20E-02
	SP_PIR_KEYWORDS	Pyrrolidone carboxylic acid	7	6.10E-03	7.40E-02
	GOTERM_BP_FAT	collagen fibril organization	5	6.90E-03	4.40E-01
	PIR_SUPERFAMILY	PIRSF002255:collagen alpha 1(I) chain	3	7.70E-03	5.90E-01
	UP_SEQ_FEATURE	propeptide:N-terminal propeptide	3	8.60E-03	6.90E-01
	UP_SEQ_FEATURE	propeptide:C-terminal propeptide	3	1.60E-02	7.90E-01
	UP_SEQ_FEATURE	region of interest:Triple-helical region	4	1.80E-02	8.30E-01
	SP_PIR_KEYWORDS	Ehlers-Danlos syndrome	3	2.90E-02	2.50E-01
	UP_SEQ_FEATURE	domain:Fibrillar collagen NC1	3	2.90E-02	9.20E-01
	INTERPRO	Fibrillar collagen, C-terminal	3	3.00E-02	9.10E-01
	SMART	COLFI	3	4.50E-02	9.60E-01
	GOTERM_BP_FAT	extracellular matrix organization	7	5.90E-02	8.70E-01
	GOTERM_BP_FAT	extracellular structure organization	9	7.00E-02	8.80E-01
	SP_PIR_KEYWORDS	pyroglutamic acid	4	1.00E-01	5.00E-01
	GOTERM_MF_FAT	growth factor binding	6	1.50E-01	9.40E-01
	GOTERM_BP_FAT	skin development	3	1.80E-01	9.40E-01
	GOTERM_MF_FAT	structural molecule activity	20	3.30E-01	9.60E-01
	GOTERM_BP_FAT	ectoderm development	7	4.40E-01	9.90E-01
	GOTERM_BP_FAT	epidermis development	5	7.30E-01	1.00E+00
**Annotation Cluster 7**	**Enrichment Score: 2.14**		**Count**	**P_Value**	**Benjamini**
	GOTERM_MF_FAT	IgG binding	5	3.20E-05	1.70E-02
	INTERPRO	Immunoglobulin-like fold	32	3.40E-05	2.60E-02
	INTERPRO	Immunoglobulin-like	29	8.50E-05	2.20E-02
	SP_PIR_KEYWORDS	immunoglobulin receptor	5	1.00E-04	3.20E-03
	SP_PIR_KEYWORDS	igg-binding protein	4	4.80E-04	1.10E-02
	GOTERM_MF_FAT	immunoglobulin binding	5	5.40E-04	9.30E-02
	SP_PIR_KEYWORDS	Immunoglobulin domain	24	1.50E-03	2.40E-02
	INTERPRO	Immunoglobulin V-set	12	5.40E-02	9.70E-01
	UP_SEQ_FEATURE	domain:Ig-like C2-type 1	9	1.00E-01	9.90E-01
	UP_SEQ_FEATURE	domain:Ig-like C2-type 2	9	1.00E-01	9.90E-01
	INTERPRO	Immunoglobulin subtype	13	1.40E-01	9.80E-01
	GOTERM_MF_FAT	protein complex binding	8	2.70E-01	9.60E-01
	SMART	IG	13	3.40E-01	9.80E-01
	INTERPRO	Immunoglobulin	7	4.10E-01	1.00E+00
	UP_SEQ_FEATURE	domain:Ig-like C2-type 3	3	8.10E-01	1.00E+00
**Annotation Cluster 8**	**Enrichment Score: 1.96**		**Count**	**P_Value**	**Benjamini**
	KEGG_PATHWAY	Focal adhesion	17	2.90E-04	4.50E-03
	SP_PIR_KEYWORDS	trimer	6	3.80E-04	9.50E-03
	KEGG_PATHWAY	ECM-receptor interaction	10	8.20E-04	1.10E-02
	UP_SEQ_FEATURE	domain:VWFC	4	9.30E-03	6.90E-01
	INTERPRO	von Willebrand factor, type C	4	8.10E-02	9.80E-01
	SMART	VWC	4	1.30E-01	1.00E+00
	GOTERM_MF_FAT	growth factor binding	6	1.50E-01	9.40E-01
	UP_SEQ_FEATURE	short sequence motif:Cell attachment site	5	1.70E-01	1.00E+00
**Annotation Cluster 9**	**Enrichment Score: 1.86**		**Count**	**P_Value**	**Benjamini**
	GOTERM_BP_FAT	cell separation during cytokinesis	3	4.10E-03	3.20E-01
	GOTERM_BP_FAT	cytokinetic process	3	6.70E-03	4.40E-01
	GOTERM_BP_FAT	cytokinesis	4	9.50E-02	9.00E-01
**Annotation Cluster 10**	**Enrichment Score: 1.66**		**Count**	**P_Value**	**Benjamini**
	GOTERM_BP_FAT	maintenance of location	7	7.00E-03	4.30E-01
	GOTERM_BP_FAT	maintenance of protein location	6	8.70E-03	4.80E-01
	GOTERM_BP_FAT	maintenance of location in cell	5	3.80E-02	8.10E-01
	GOTERM_BP_FAT	maintenance of protein location in cell	4	9.50E-02	9.00E-01
**Annotation Cluster 11**	**Enrichment Score: 1.55**		**Count**	**P_Value**	**Benjamini**
	SP_PIR_KEYWORDS	glycoprotein	151	1.90E-06	1.50E-04
	UP_SEQ_FEATURE	glycosylation site:N-linked (GlcNAc...)	141	2.30E-05	7.30E-03
	UP_SEQ_FEATURE	topological domain:Cytoplasmic	102	2.10E-02	8.60E-01
	GOTERM_CC_FAT	plasma membrane	117	5.40E-02	6.80E-01
	UP_SEQ_FEATURE	topological domain:Extracellular	81	5.40E-02	9.70E-01
	UP_SEQ_FEATURE	transmembrane region	130	2.30E-01	1.00E+00
	SP_PIR_KEYWORDS	transmembrane	131	2.30E-01	6.60E-01
	SP_PIR_KEYWORDS	membrane	162	2.60E-01	6.90E-01
	SP_PIR_KEYWORDS	cell membrane	57	4.30E-01	8.30E-01
	GOTERM_CC_FAT	integral to membrane	140	6.90E-01	9.80E-01
	GOTERM_CC_FAT	intrinsic to membrane	141	8.30E-01	9.90E-01

ILMN_2049909	HTN3	0.483597501
ILMN_1715214	PTPN7	0.483123777
ILMN_3257004	AK124173	0.48287198
ILMN_2182656	APBA1	0.482700949
ILMN_1663569	FTCD	0.482681888
ILMN_1711174	SPRR1B	0.482271937
ILMN_1811478	MFI2	0.482224044
ILMN_2381064	TPD52	0.481961611
ILMN_1763695	C9orf24	0.481474788
ILMN_2374076	C14orf102	0.481373038
ILMN_1740155	COL9A3	0.481244548
ILMN_1708508	PPM1E	0.480735785
ILMN_1763516	SPINK2	0.480454123
ILMN_1680246	MAT2B	0.480359758
ILMN_1788061	sept-12	0.480237139
ILMN_1812432	TIRAP	0.480048887
ILMN_1694106	GPD1L	0.479702079
ILMN_1691339	CLEC1A	0.479644146
ILMN_1740418	CYP27B1	0.479625809
ILMN_2188091	FGF6	0.478807468
ILMN_1779370	ARHGEF9	0.478640384
ILMN_3243485	NA	0.478601382
ILMN_1823229	BX094072	0.47834329
ILMN_1675192	IFNA21	0.478298999
ILMN_1796455	RYR3	0.478159042
ILMN_1759910	SERPINA5	0.477855604
ILMN_1800898	ARG2	0.477789433
ILMN_1708482	TMEM80	0.477426366
ILMN_3190833	CCRL2	0.476593704
ILMN_1768101	HOXB6	0.476452783
ILMN_1732197	MN1	0.475739659
ILMN_2163306	FAM120A	0.475655213
ILMN_3203059	LOC100131176	0.475357289
ILMN_1683390	FLCN	0.475264237
ILMN_1722156	RWDD2A	0.475010252
ILMN_1814296	TRPM6	0.474998097
ILMN_3271555	LOC100130458	0.474727564
ILMN_1789323	LINC00477	0.474249876
ILMN_2363744	PACRG	0.474171513
ILMN_1798546	HDAC6	0.473870828
ILMN_1781242	SOST	0.473449127
ILMN_3234989	LOC100133920	0.473419099
ILMN_1784176	ABCA5	0.472364967
ILMN_3294277	CYP4F24P	0.472297417
ILMN_2311456	COL11A2	0.472243836
ILMN_1806294	RPS6KA3	0.472238708
ILMN_1723846	METTL21B	0.471799847
ILMN_2206282	GEN1	0.471717331
ILMN_2205162	ZNF699	0.471632314
ILMN_1724410	USP46	0.471482053
ILMN_2120210	RCAN2	0.471174638
ILMN_1826771	CD520639	0.470306935
ILMN_1677586	KRT83	0.470279963
ILMN_1813296	RDH8	0.470110609
ILMN_1659976	CLP1	0.469997317
ILMN_1653878	CACNB2	0.469988908
ILMN_1802896	ZBTB8B	0.469840777
ILMN_2290118	MEGF9	0.469714572
ILMN_1728803	HMP19	0.469029449
ILMN_1739631	FRMPD2P1	0.468753862
ILMN_1794844	THRSP	0.468726423
ILMN_1669474	PCDHA@	0.468459878
ILMN_1724873	OR10T2	0.468450576
ILMN_1741431	AOC2	0.468428678
ILMN_1885147	AW292656	0.46818801
ILMN_1671731	AVPI1	0.46785055
ILMN_3187300	NA	0.467790871
ILMN_1711422	PLEKHN1	0.467729701
ILMN_2343917	ABCD4	0.467714154
ILMN_1664595	IFNA13	0.467364904
ILMN_3226181	NUDT7	0.467247462
ILMN_1710000	PEX11G	0.466838917
ILMN_1756669	POGK	0.466568831
ILMN_2091978	FREM2	0.466303909
ILMN_1708779	GNLY	0.466303893
ILMN_1792972	ZNF439	0.466195478
ILMN_2353030	GRAP	0.466062031
ILMN_2195665	PANK3	0.465827814
ILMN_1821997	AI924184	0.465787624
ILMN_2105223	FCRL5	0.465674974
ILMN_1831214	AW592010	0.465490245
ILMN_1781906	RBM17	0.464799869
ILMN_3304072	PRG4	0.464703631
ILMN_2058512	PSMA2	0.4646667
ILMN_1726815	HIST1H3G	0.464623604
ILMN_1886026	AI221824	0.464448872
ILMN_2148193	MPPED2	0.464431138
ILMN_1749546	LOC653545	0.464264717
ILMN_1778980	AX747641	0.464203439
ILMN_1738268	IP6K3	0.464106824
ILMN_1703222	FRS2	0.46407076
ILMN_2297997	LIPT1	0.463960059
ILMN_2397846	SNCB	0.463225815
ILMN_1752631	CGGBP1	0.463216498
ILMN_1706213	LOC342346	0.463166795
ILMN_1728746	GAB2	0.463037484

			Count	P_Value	Benjamini
	GOTERM_MF_FAT	neurotransmitter transporter activity	3	1.40E-01	9.70E-01
	GOTERM_MF_FAT	amine transmembrane transporter activity	5	1.40E-01	9.60E-01
	SP_PIR_KEYWORDS	Symport	6	1.50E-01	8.20E-01
	GOTERM_MF_FAT	amino acid transmembrane transporter activity	4	2.20E-01	9.80E-01
	SP_PIR_KEYWORDS	amino-acid transport	3	2.30E-01	8.80E-01
	GOTERM_MF_FAT	solute:cation symporter activity	5	2.60E-01	9.80E-01
	GOTERM_MF_FAT	L-amino acid transmembrane transporter activity	3	2.80E-01	9.80E-01
	GOTERM_MF_FAT	symporter activity	6	3.20E-01	9.90E-01
	GOTERM_BP_FAT	neurotransmitter transport	4	3.60E-01	9.90E-01
**Annotation Cluster 13**	**Enrichment Score: 1**		**Count**	**P_Value**	**Benjamini**
	UP_SEQ_FEATURE	nucleotide phosphate-binding region:NADP	6	3.60E-02	1.00E+00
	INTERPRO	NAD(P)-binding domain	9	4.20E-02	9.50E-01
	SP_PIR_KEYWORDS	nadp	9	4.40E-02	6.80E-01
	INTERPRO	Glucose/ribitol dehydrogenase	5	4.90E-02	9.60E-01
	PIR_SUPERFAMILY	PIRSF000092:short-chain dehydrogenase	3	5.10E-02	1.00E+00
	INTERPRO	Short-chain dehydrogenase/reductase SDR	5	6.70E-02	9.70E-01
	UP_SEQ_FEATURE	binding site:Substrate	13	7.60E-02	1.00E+00
	UP_SEQ_FEATURE	binding site:NADP	3	1.60E-01	1.00E+00
	GOTERM_BP_FAT	regulation of hormone levels	6	3.50E-01	9.90E-01
	GOTERM_BP_FAT	cellular hormone metabolic process	3	4.50E-01	1.00E+00
	GOTERM_BP_FAT	hormone metabolic process	4	5.10E-01	1.00E+00
**Annotation Cluster 14**	**Enrichment Score: 0.97**		**Count**	**P_Value**	**Benjamini**
	SP_PIR_KEYWORDS	glycoprotein	125	5.40E-02	7.00E-01
	SP_PIR_KEYWORDS	Secreted	54	5.60E-02	6.90E-01
	UP_SEQ_FEATURE	glycosylation site:N-linked (GlcNAc...)	118	9.10E-02	1.00E+00
	SP_PIR_KEYWORDS	signal	94	9.40E-02	7.40E-01
	UP_SEQ_FEATURE	signal peptide	94	1.10E-01	1.00E+00
	UP_SEQ_FEATURE	disulfide bond	81	1.50E-01	1.00E+00
	SP_PIR_KEYWORDS	disulfide bond	82	1.80E-01	8.60E-01
	GOTERM_CC_FAT	extracellular region	61	2.50E-01	9.40E-01
**Annotation Cluster 15**	**Enrichment Score: 0.96**		**Count**	**P_Value**	**Benjamini**
	GOTERM_BP_FAT	skeletal system development	15	3.60E-02	9.20E-01
	GOTERM_BP_FAT	ossification	6	1.70E-01	9.90E-01
	GOTERM_BP_FAT	bone development	6	2.10E-01	9.90E-01
**Annotation Cluster 16**	**Enrichment Score: 0.93**		**Count**	**P_Value**	**Benjamini**
	GOTERM_BP_FAT	regulation of pH	4	5.60E-02	9.00E-01
	GOTERM_BP_FAT	monovalent inorganic cation homeostasis	4	1.10E-01	9.60E-01
	GOTERM_CC_FAT	apical plasma membrane	7	1.60E-01	9.00E-01
	GOTERM_BP_FAT	cation homeostasis	11	2.00E-01	9.90E-01
**Annotation Cluster 17**	**Enrichment Score: 0.92**		**Count**	**P_Value**	**Benjamini**
	SP_PIR_KEYWORDS	peroxisome	6	7.70E-02	7.20E-01
	GOTERM_CC_FAT	microbody	6	1.50E-01	9.00E-01
	GOTERM_CC_FAT	peroxisome	6	1.50E-01	9.00E-01
**Annotation Cluster 18**	**Enrichment Score: 0.9**		**Count**	**P_Value**	**Benjamini**
	SP_PIR_KEYWORDS	heparin-binding	6	2.40E-02	7.70E-01
	GOTERM_MF_FAT	heparin binding	7	6.20E-02	9.60E-01
	GOTERM_MF_FAT	glycosaminoglycan binding	8	8.80E-02	9.50E-01
	GOTERM_MF_FAT	carbohydrate binding	15	1.10E-01	9.60E-01
	GOTERM_MF_FAT	pattern binding	8	1.30E-01	9.70E-01
	GOTERM_MF_FAT	polysaccharide binding	8	1.30E-01	9.70E-01
	GOTERM_BP_FAT	regulation of blood coagulation	3	2.40E-01	9.90E-01
	GOTERM_BP_FAT	regulation of coagulation	3	2.80E-01	9.90E-01
	GOTERM_BP_FAT	regulation of response to external stimulus	5	5.90E-01	1.00E+00
**Annotation Cluster 19**	**Enrichment Score: 0.88**		**Count**	**P_Value**	**Benjamini**
	UP_SEQ_FEATURE	metal ion-binding site:Manganese 2	4	3.00E-02	1.00E+00
	UP_SEQ_FEATURE	metal ion-binding site:Manganese 1	4	3.00E-02	1.00E+00
	GOTERM_MF_FAT	protein serine/threonine phosphatase activity	5	3.30E-02	9.80E-01
	SP_PIR_KEYWORDS	manganese	9	3.90E-02	7.60E-01
	UP_SEQ_FEATURE	metal ion-binding site:Manganese 1; via carbonyl oxygen	3	4.20E-02	1.00E+00
	GOTERM_BP_FAT	dephosphorylation	9	4.50E-02	8.90E-01
	INTERPRO	Protein phosphatase 2C, manganese/magnesium aspartate binding site	3	5.70E-02	9.50E-01
	SP_PIR_KEYWORDS	magnesium	18	5.70E-02	6.80E-01
	INTERPRO	Protein phosphatase 2C	3	7.10E-02	9.70E-01
	INTERPRO	Protein phosphatase 2C, N-terminal	3	7.80E-02	9.70E-01
	INTERPRO	Protein phosphatase 2C-related	3	9.40E-02	9.70E-01
	SMART	PP2C_SIG	3	9.60E-02	9.50E-01
	SMART	PP2Cc	3	9.60E-02	9.50E-01
	SP_PIR_KEYWORDS	protein phosphatase	7	1.10E-01	7.70E-01
	GOTERM_MF_FAT	magnesium ion binding	18	1.10E-01	9.60E-01
	GOTERM_MF_FAT	manganese ion binding	8	1.30E-01	9.70E-01
	GOTERM_BP_FAT	protein amino acid dephosphorylation	7	1.30E-01	9.70E-01
	GOTERM_MF_FAT	phosphatase activity	11	1.40E-01	9.70E-01
	GOTERM_MF_FAT	phosphoprotein phosphatase activity	8	1.60E-01	9.80E-01
	INTERPRO	Protein-tyrosine phosphatase, receptor/non-receptor type	3	2.80E-01	1.00E+00
	SMART	PTPc	3	2.80E-01	9.90E-01
	GOTERM_CC_FAT	protein serine/threonine phosphatase complex	3	3.00E-01	9.60E-01
	UP_SEQ_FEATURE	domain:Tyrosine-protein phosphatase	3	4.70E-01	1.00E+00
	UP_SEQ_FEATURE	active site:Phosphocysteine intermediate	3	6.30E-01	1.00E+00
	INTERPRO	Protein-tyrosine phosphatase	3	6.30E-01	1.00E+00
	INTERPRO	Dual-specific/protein-tyrosine phosphatase, conserved region	3	6.30E-01	1.00E+00
	INTERPRO	Protein-tyrosine phosphatase, active site	3	6.80E-01	1.00E+00
	GOTERM_MF_FAT	protein tyrosine phosphatase activity	3	7.80E-01	1.00E+00
**Annotation Cluster 20**	**Enrichment Score: 0.85**		**Count**	**P_Value**	**Benjamini**
	GOTERM_BP_FAT	bicarbonate transport	3	1.30E-02	9.50E-01
	GOTERM_BP_FAT	inorganic anion transport	7	3.20E-02	9.20E-01
	GOTERM_BP_FAT	chloride transport	5	7.10E-02	9.30E-01
	GOTERM_BP_FAT	anion transport	8	7.50E-02	9.30E-01
	GOTERM_BP_FAT	organic anion transport	4	1.00E-01	9.60E-01
	GOTERM_MF_FAT	anion transmembrane transporter activity	7	2.10E-01	9.80E-01
	GOTERM_MF_FAT	anion binding	5	2.40E-01	9.80E-01
	SP_PIR_KEYWORDS	chloride	4	2.90E-01	9.00E-01
	GOTERM_MF_FAT	chloride ion binding	4	3.50E-01	9.90E-01
	GOTERM_MF_FAT	chloride channel activity	3	5.90E-01	1.00E+00
	GOTERM_MF_FAT	anion channel activity	3	6.30E-01	1.00E+00
**Annotation Cluster 21**	**Enrichment Score: 0.85**		**Count**	**P_Value**	**Benjamini**
	GOTERM_CC_FAT	mitochondrial membrane part	9	2.20E-02	7.10E-01

ILMN_1676689	PPT2	-0.422750039
ILMN_1675162	AK097193	-0.422852546
ILMN_1690291	CC2D1B	-0.423276444
ILMN_1792947	ESCO2	-0.423418073
ILMN_1748283	PIM2	-0.42349798
ILMN_1797102	ST6GAL2	-0.423616969
ILMN_1769779	PTP4A3	-0.423746265
ILMN_1728478	CXCL16	-0.423755713
ILMN_1684887	SAMSN1	-0.424374195
ILMN_2364521	AXL	-0.424757106
ILMN_1736806	PAG1	-0.424864596
ILMN_1809199	FER1L4	-0.425014766
ILMN_1772218	HLA-DPA1	-0.425068848
ILMN_3248213	DKFZp434I138	-0.425071327
ILMN_1789991	MARCH4	-0.425445839
ILMN_1701402	IKBIP	-0.425585322
ILMN_1786429	LPAR6	-0.425642878
ILMN_1661516	POLR2J2	-0.425886816
ILMN_1672608	PPAP2B	-0.426004347
ILMN_1657991	LOC391722	-0.426227969
ILMN_1828216	CB987747	-0.426380272
ILMN_1757347	KIAA0930	-0.42679339
ILMN_2113490	NTN4	-0.426795891
ILMN_1796949	TPX2	-0.427001524
ILMN_2144654	SPDYE2	-0.427015169
ILMN_1699022	ENDOD1	-0.427050519
ILMN_1800317	WNT5A	-0.427270995
ILMN_1785852	OBFC2A	-0.42730499
ILMN_2353732	CD8A	-0.427520637
ILMN_1815552	NTAN1	-0.427608429
ILMN_1775183	CBX8	-0.427683901
ILMN_2369018	EVI2A	-0.427763324
ILMN_1713274	OTOS	-0.427820039
ILMN_1683456	CCL7	-0.42789135
ILMN_1669876	CUX2	-0.427943065
ILMN_3220934	NCRNA00152	-0.427987027
ILMN_2163819	KIF21B	-0.428175715
ILMN_2113049	QPCT	-0.428374512
ILMN_3255699	LOC100128185	-0.428479617
ILMN_1793959	ADPGK	-0.428638192
ILMN_1671452	MRPL44	-0.428689108
ILMN_2222688	TMSB4X	-0.428764912
ILMN_1715203	DNAH7	-0.428782399
ILMN_1785635	BRD3	-0.429066348
ILMN_1806692	HEXB	-0.42914
ILMN_3270833	NA	-0.429284233
ILMN_2287147	UBTF	-0.429717374
ILMN_1672042	DOLPP1	-0.429732041
ILMN_1777541	KLK6	-0.429754457
ILMN_1790529	LUM	-0.430114886
ILMN_1771299	C2orf81	-0.430400424
ILMN_2112402	PHF5A	-0.430572734
ILMN_1672908	TWIST1	-0.431032186
ILMN_2199614	LRCH1	-0.431101558
ILMN_1791052	FKBPL	-0.431337499
ILMN_1808946	POLR2J4	-0.431369486
ILMN_1706305	COX16	-0.431395498
ILMN_1674285	LOC401233	-0.431569825
ILMN_1653028	COL4A1	-0.431623913
ILMN_3235176	C2orf89	-0.431642403
ILMN_2212909	MELK	-0.431860343
ILMN_1771921	HSCB	-0.431915914
ILMN_1734306	OR52E4	-0.432073122
ILMN_2157441	HLA-DRA	-0.432129938
ILMN_1782142	S100A13	-0.432388792
ILMN_1768940	COL15A1	-0.432731645
ILMN_2130158	OR2L5	-0.432754739
ILMN_1707292	C17orf67	-0.432757072
ILMN_1742947	SEC14L4	-0.43352279
ILMN_2049021	PTTG3	-0.433614878
ILMN_1686116	THBS1	-0.433854581
ILMN_1659029	FAM116B	-0.433917866
ILMN_1716952	FBXO10	-0.433962206
ILMN_1753716	EIF2B1	-0.433975398
ILMN_1846771	BU521176	-0.434352285
ILMN_2103388	ZBTB49	-0.434540126
ILMN_1812091	FAM20A	-0.434608507
ILMN_1789364	ZNF789	-0.434708232
ILMN_2112580	FCGR3A	-0.434846282
ILMN_2360710	TPM1	-0.435024253
ILMN_1711566	TIMP1	-0.435043161
ILMN_1747197	SLC41A2	-0.435220319
ILMN_2242345	LPAL2	-0.435335761
ILMN_1678842	THBS2	-0.43538813
ILMN_1700935	HAS2	-0.435449187
ILMN_1674228	abParts	-0.43623239
ILMN_2393765	IGLL1	-0.436340736
ILMN_2181968	CBL	-0.436358913
ILMN_3239373	HNRNPCL1	-0.436400946
ILMN_1739876	RAB3GAP1	-0.436586695
ILMN_1790782	MED16	-0.436602583
ILMN_1812494	GDA	-0.436699575
ILMN_1750321	TRANK1	-0.436709416
ILMN_1676907	U12978	-0.436738507
ILMN_1779228	CDH2	-0.436790747
ILMN_1653026	PLAC8	-0.436802084

			Count	P_Value	Benjamini
**Annotation Cluster 12**	**Enrichment Score: 1.5**		**Count**	**P_Value**	**Benjamini**
	GOTERM_BP_FAT	innate immune response	10	1.10E-02	5.50E-01
	GOTERM_BP_FAT	response to wounding	24	1.50E-02	5.90E-01
	GOTERM_BP_FAT	defense response	24	6.30E-02	8.80E-01
	GOTERM_BP_FAT	inflammatory response	14	9.50E-02	9.00E-01
**Annotation Cluster 13**	**Enrichment Score: 1.44**		**Count**	**P_Value**	**Benjamini**
	INTERPRO	Actinin-type, actin-binding, conserved site	5	2.50E-03	2.80E-01
	UP_SEQ_FEATURE	domain:CH 1	5	4.10E-03	4.60E-01
	UP_SEQ_FEATURE	domain:CH 2	5	4.10E-03	4.60E-01
	INTERPRO	Calponin-like actin-binding	7	8.60E-03	5.80E-01
	UP_SEQ_FEATURE	domain:Actin-binding	4	1.30E-02	7.40E-01
	SP_PIR_KEYWORDS	actin binding	5	1.70E-02	1.70E-01
	SMART	CH	7	2.20E-02	9.00E-01
	UP_SEQ_FEATURE	repeat:Spectrin 9	3	4.00E-02	9.40E-01
	UP_SEQ_FEATURE	repeat:Spectrin 7	3	4.00E-02	9.40E-01
	UP_SEQ_FEATURE	repeat:Spectrin 8	3	4.00E-02	9.40E-01
	UP_SEQ_FEATURE	repeat:Spectrin 6	3	4.00E-02	9.40E-01
	UP_SEQ_FEATURE	repeat:Spectrin 5	3	4.60E-02	9.60E-01
	SP_PIR_KEYWORDS	actin-binding	11	8.60E-02	4.50E-01
	UP_SEQ_FEATURE	repeat:Spectrin 4	3	9.40E-02	9.90E-01
	INTERPRO	Spectrin repeat	3	9.80E-02	9.70E-01
	UP_SEQ_FEATURE	repeat:Spectrin 3	3	1.00E-01	9.90E-01
	UP_SEQ_FEATURE	repeat:Spectrin 2	3	1.30E-01	9.90E-01
	UP_SEQ_FEATURE	repeat:Spectrin 1	3	1.30E-01	9.90E-01
	INTERPRO	Spectrin/alpha-actinin	3	1.90E-01	9.90E-01
	SMART	SPEC	3	2.50E-01	9.70E-01
**Annotation Cluster 14**	**Enrichment Score: 1.41**		**Count**	**P_Value**	**Benjamini**
	GOTERM_BP_FAT	phagocytosis, engulfment	4	2.00E-03	2.20E-01
	GOTERM_BP_FAT	phagocytosis	4	1.40E-01	9.30E-01
	GOTERM_BP_FAT	positive regulation of cytokine production	5	2.20E-01	9.50E-01
**Annotation Cluster 15**	**Enrichment Score: 1.29**		**Count**	**P_Value**	**Benjamini**
	GOTERM_BP_FAT	collagen metabolic process	4	3.70E-02	8.20E-01
	GOTERM_BP_FAT	multicellular organismal macromolecule metabolic process	4	4.80E-02	8.60E-01
	GOTERM_BP_FAT	multicellular organismal metabolic process	4	7.50E-02	8.80E-01
**Annotation Cluster 16**	**Enrichment Score: 1.26**		**Count**	**P_Value**	**Benjamini**
	GOTERM_BP_FAT	protein complex biogenesis	22	2.90E-02	7.60E-01
	GOTERM_BP_FAT	protein complex assembly	22	2.90E-02	7.60E-01
	GOTERM_BP_FAT	macromolecular complex assembly	25	8.00E-02	8.80E-01
	GOTERM_BP_FAT	macromolecular complex subunit organization	25	1.30E-01	9.30E-01
**Annotation Cluster 17**	**Enrichment Score: 1.2**		**Count**	**P_Value**	**Benjamini**
	GOTERM_BP_FAT	positive regulation of immune system process	13	2.60E-02	7.50E-01
	GOTERM_CC_FAT	external side of plasma membrane	10	4.10E-02	6.20E-01
	GOTERM_CC_FAT	cell surface	13	2.30E-01	8.90E-01
**Annotation Cluster 18**	**Enrichment Score: 1.17**		**Count**	**P_Value**	**Benjamini**
	SP_PIR_KEYWORDS	heparin-binding	6	2.20E-02	2.10E-01
	GOTERM_MF_FAT	polysaccharide binding	10	2.20E-02	6.70E-01
	GOTERM_MF_FAT	pattern binding	10	2.20E-02	6.70E-01
	GOTERM_MF_FAT	glycosaminoglycan binding	9	3.40E-02	7.60E-01
	GOTERM_MF_FAT	carbohydrate binding	16	5.30E-02	8.40E-01
	UP_SEQ_FEATURE	domain:TSP type-1 3	4	5.90E-02	9.70E-01
	UP_SEQ_FEATURE	region of interest:Heparin-binding	3	8.70E-02	9.90E-01
	UP_SEQ_FEATURE	domain:TSP type-1 2	4	9.50E-02	9.90E-01
	UP_SEQ_FEATURE	domain:TSP type-1 1	4	9.50E-02	9.90E-01
	GOTERM_MF_FAT	heparin binding	6	1.40E-01	9.40E-01
	INTERPRO	Thrombospondin, type 1 repeat	4	2.30E-01	9.90E-01
	SMART	TSP1	4	3.30E-01	9.80E-01
**Annotation Cluster 19**	**Enrichment Score: 1.17**		**Count**	**P_Value**	**Benjamini**
	UP_SEQ_FEATURE	domain:TLC	3	5.80E-02	9.70E-01
	INTERPRO	TRAM, LAG1 and CLN8 homology	3	6.10E-02	9.70E-01
	SMART	TLC	3	8.80E-02	9.90E-01
**Annotation Cluster 20**	**Enrichment Score: 1.15**		**Count**	**P_Value**	**Benjamini**
	GOTERM_CC_FAT	endosomal part	5	6.80E-02	6.90E-01
	GOTERM_CC_FAT	endosome membrane	5	6.80E-02	6.90E-01
	GOTERM_CC_FAT	early endosome	6	8.00E-02	7.30E-01
**Annotation Cluster 21**	**Enrichment Score: 1.14**		**Count**	**P_Value**	**Benjamini**
	GOTERM_BP_FAT	cell motion	20	5.00E-02	8.40E-01
	GOTERM_BP_FAT	localization of cell	14	6.80E-02	8.80E-01
	GOTERM_BP_FAT	cell motility	14	6.80E-02	8.80E-01
	GOTERM_BP_FAT	cell migration	12	1.20E-01	9.20E-01
**Annotation Cluster 22**	**Enrichment Score: 1.13**		**Count**	**P_Value**	**Benjamini**
	GOTERM_BP_FAT	endocytosis	12	3.40E-02	8.00E-01
	GOTERM_BP_FAT	membrane invagination	12	3.40E-02	8.00E-01
	GOTERM_BP_FAT	membrane organization	17	4.90E-02	8.50E-01
	GOTERM_BP_FAT	vesicle-mediated transport	20	2.00E-01	9.40E-01
	SP_PIR_KEYWORDS	Endocytosis	5	2.10E-01	6.60E-01
**Annotation Cluster 23**	**Enrichment Score: 1.1**		**Count**	**P_Value**	**Benjamini**
	INTERPRO	Zinc finger, PHD-finger	7	2.10E-02	8.50E-01
	INTERPRO	Zinc finger, PHD-type	6	7.90E-02	9.80E-01
	INTERPRO	Zinc finger, PHD-type, conserved site	6	8.80E-02	9.70E-01
	UP_SEQ_FEATURE	zinc finger region:PHD-type	4	1.40E-01	9.90E-01
	SMART	PHD	6	1.50E-01	9.80E-01
**Annotation Cluster 24**	**Enrichment Score: 1.08**		**Count**	**P_Value**	**Benjamini**
	GOTERM_BP_FAT	cytoskeleton organization	21	1.30E-02	5.70E-01
	GOTERM_MF_FAT	cytoskeletal protein binding	23	1.60E-02	6.30E-01
	GOTERM_MF_FAT	actin binding	15	5.40E-02	8.30E-01
	GOTERM_BP_FAT	actin cytoskeleton organization	11	8.20E-02	8.80E-01
	SP_PIR_KEYWORDS	actin-binding	11	8.60E-02	4.50E-01
	GOTERM_BP_FAT	actin filament-based process	11	1.10E-01	9.00E-01
	GOTERM_BP_FAT	actin filament organization	4	3.00E-01	9.70E-01
	GOTERM_CC_FAT	actin cytoskeleton	6	8.60E-01	1.00E+00
**Annotation Cluster 25**	**Enrichment Score: 1.03**		**Count**	**P_Value**	**Benjamini**
	GOTERM_BP_FAT	transcription initiation	7	2.30E-02	7.20E-01
	GOTERM_BP_FAT	transcription initiation from RNA polymerase II promoter	6	3.50E-02	8.00E-01
	GOTERM_BP_FAT	transcription from RNA polymerase II promoter	12	4.90E-02	8.50E-01
	GOTERM_BP_FAT	transcription, DNA-dependent	14	5.00E-02	8.40E-01
	GOTERM_BP_FAT	RNA biosynthetic process	14	5.40E-02	8.50E-01

ILMN_1811022	HMX1	0.462095085
ILMN_1778687	RHCG	0.461497302
ILMN_1663234	KATNAL1	0.461378624
ILMN_1672605	C7orf41	0.461024999
ILMN_1771538	PSCA	0.460745747
ILMN_1798547	FAM71D	0.460729111
ILMN_1804662	NRG4	0.46044759
ILMN_1672591	SCN9A	0.460215358
ILMN_1810956	NA	0.460003281
ILMN_3237025	MBD3L2	0.45938999
ILMN_2114812	ONECUT1	0.459289696
ILMN_1662117	TCP11	0.458658873
ILMN_1668498	CWF19L2	0.458476369
ILMN_3308078	MIR31	0.458378767
ILMN_1761260	COBLL1	0.45820738
ILMN_1759453	UQCRB	0.45818621
ILMN_1653347	BC012881	0.458174699
ILMN_1727606	C12orf59	0.458130454
ILMN_2050911	SLC22A4	0.457992418
ILMN_1663598	LINC00266-1	0.457747221
ILMN_2178509	OR52A5	0.457415595
ILMN_1776788	C5orf41	0.457415099
ILMN_1826447	BX107250	0.457236701
ILMN_1915805	LOC340073	0.457226627
ILMN_1853824	MGAT3	0.457084375
ILMN_1720406	HOXB9	0.457054146
ILMN_1756125	H1FNT	0.456922142
ILMN_3242710	LOC157627	0.456502902
ILMN_1768430	AK128037	0.456291996
ILMN_1745043	LDHC	0.456168426
ILMN_1678186	ATP6V0A4	0.456137709
ILMN_1693471	KLHL35	0.455895201
ILMN_1776382	NA	0.455548234
ILMN_1730432	IGSF8	0.455433754
ILMN_3234812	RFPL4A	0.455372421
ILMN_1652017	PPEF1	0.455299277
ILMN_1777708	LRRC16B	0.455196041
ILMN_1696675	CES2	0.454981465
ILMN_3289262	PNRC2	0.454103044
ILMN_2225537	PTGR1	0.45399979
ILMN_1732271	BTBD9	0.453974081
ILMN_1753729	KNG1	0.453902226
ILMN_1757769	PLCZ1	0.453546094
ILMN_1729402	CHRM2	0.453177517
ILMN_1827283	BX104024	0.453048403
ILMN_1663580	DPP6	0.451867564
ILMN_1664581	MICALL2	0.451724672
ILMN_1723735	AB020696	0.451128817
ILMN_1843616	AA620741	0.450770403
ILMN_1697373	LINC00207	0.450678322
ILMN_1662989	PRB2	0.450628303
ILMN_3309148	MIR556	0.450432616
ILMN_1690276	ZNF354C	0.450418155
ILMN_3307178	SPRNP1	0.4503888
ILMN_2135756	HSF2BP	0.450211159
ILMN_3238299	NHEDC1	0.450082928
ILMN_1777905	NA	0.450040289
ILMN_1888546	AI039233	0.449913566
ILMN_1750062	PPARGC1A	0.449578377
ILMN_1759297	PATZ1	0.44957295
ILMN_1674017	C11orf53	0.449365067
ILMN_1736981	BRD4	0.449107422
ILMN_1746819	C5	0.448490609
ILMN_2224230	TTTY21	0.448257663
ILMN_1810423	RPP40	0.44810477
ILMN_1739659	ZDHHC6	0.447889038
ILMN_1738276	TMEM185A	0.447589995
ILMN_1703511	PDZRN3	0.447441981
ILMN_1751630	TDRD10	0.447301705
ILMN_3280496	RPL26	0.447040861
ILMN_1670535	NDRG2	0.446856671
ILMN_2345016	PTGES2	0.446802186
ILMN_1810942	CYP3A5	0.446570554
ILMN_1833027	AI936635	0.446215379
ILMN_1778683	RTN4RL1	0.445286974
ILMN_1749253	TUBD1	0.445262155
ILMN_3285762	SLC4A1	0.445201434
ILMN_1761551	FAT4	0.445087127
ILMN_1808122	C1QTNF4	0.444791718
ILMN_1657870	ABL2	0.444759765
ILMN_1665630	HMGN2	0.444753539
ILMN_1795251	SPARCL1	0.444318341
ILMN_2230566	RAB40B	0.443617626
ILMN_2347298	TSPYL2	0.443604453
ILMN_1739274	PDHB	0.443348918
ILMN_1704629	SLC1A7	0.4430208
ILMN_1816781	BF056254	0.442956138
ILMN_1778161	DNAJC25	0.442876209
ILMN_2115421	SLC25A16	0.442864058
ILMN_2380181	MAG	0.442592495
ILMN_1675429	SMURF2	0.442549218
ILMN_1678448	UNC13C	0.442522839
ILMN_2393994	CSPP1	0.442182392

	SP_PIR_KEYWORDS	mitochondrion inner membrane	11	2.50E-02	7.00E-01
	SP_PIR_KEYWORDS	mitochondrion	31	3.10E-02	7.40E-01
	KEGG_PATHWAY	Oxidative phosphorylation	9	3.40E-02	8.90E-01
	GOTERM_CC_FAT	mitochondrial membrane	17	7.60E-02	9.10E-01
	GOTERM_CC_FAT	mitochondrial inner membrane	14	7.90E-02	9.00E-01
	GOTERM_CC_FAT	mitochondrial envelope	17	1.10E-01	9.00E-01
	GOTERM_CC_FAT	organelle inner membrane	14	1.20E-01	8.90E-01
	GOTERM_CC_FAT	mitochondrial part	22	1.40E-01	9.10E-01
	GOTERM_CC_FAT	mitochondrion	36	1.80E-01	9.10E-01
	UP_SEQ_FEATURE	transit peptide:Mitochondrion	15	2.90E-01	1.00E+00
	SP_PIR_KEYWORDS	transit peptide	15	3.20E-01	9.10E-01
	GOTERM_CC_FAT	organelle envelope	20	3.40E-01	9.60E-01
	GOTERM_CC_FAT	envelope	20	3.50E-01	9.60E-01
	GOTERM_CC_FAT	organelle membrane	31	5.30E-01	9.80E-01
	GOTERM_CC_FAT	mitochondrial lumen	6	7.50E-01	9.90E-01
	GOTERM_CC_FAT	mitochondrial matrix	6	7.50E-01	9.90E-01
**Annotation Cluster 22**	**Enrichment Score: 0.85**		**Count**	**P_Value**	**Benjamini**
	GOTERM_BP_FAT	inner ear morphogenesis	5	5.50E-02	9.20E-01
	GOTERM_BP_FAT	embryonic organ morphogenesis	8	5.50E-02	9.10E-01
	GOTERM_BP_FAT	ear morphogenesis	5	8.90E-02	9.50E-01
	GOTERM_BP_FAT	sensory organ development	10	1.40E-01	9.80E-01
	GOTERM_BP_FAT	inner ear development	5	1.40E-01	9.80E-01
	GOTERM_BP_FAT	embryonic organ development	8	1.50E-01	9.80E-01
	GOTERM_BP_FAT	embryonic morphogenesis	12	1.70E-01	9.90E-01
	GOTERM_BP_FAT	axis specification	3	1.80E-01	9.90E-01
	GOTERM_BP_FAT	ear development	5	2.30E-01	9.90E-01
	GOTERM_BP_FAT	regionalization	6	5.70E-01	1.00E+00
**Annotation Cluster 23**	**Enrichment Score: 0.84**		**Count**	**P_Value**	**Benjamini**
	SP_PIR_KEYWORDS	calmodulin-binding	7	8.30E-02	7.40E-01
	GOTERM_MF_FAT	calmodulin binding	8	8.80E-02	9.50E-01
	UP_SEQ_FEATURE	region of interest:Calmodulin-binding	3	4.10E-01	1.00E+00
**Annotation Cluster 24**	**Enrichment Score: 0.84**		**Count**	**P_Value**	**Benjamini**
	GOTERM_CC_FAT	brush border	5	3.70E-02	8.00E-01
	GOTERM_CC_FAT	apical part of cell	9	1.20E-01	9.00E-01
	GOTERM_CC_FAT	apical plasma membrane	7	1.60E-01	9.00E-01
	GOTERM_CC_FAT	brush border membrane	3	1.70E-01	9.00E-01
	GOTERM_CC_FAT	cell projection membrane	3	5.30E-01	9.80E-01
**Annotation Cluster 25**	**Enrichment Score: 0.84**		**Count**	**P_Value**	**Benjamini**
	GOTERM_BP_FAT	negative regulation of DNA metabolic process	4	7.80E-02	9.30E-01
	GOTERM_BP_FAT	negative regulation of DNA replication	3	1.60E-01	9.80E-01
	GOTERM_BP_FAT	regulation of DNA metabolic process	6	1.70E-01	9.90E-01
	GOTERM_BP_FAT	regulation of DNA replication	4	2.10E-01	9.90E-01
**Annotation Cluster 26**	**Enrichment Score: 0.83**		**Count**	**P_Value**	**Benjamini**
	INTERPRO	G-protein, gamma-like subunit	3	7.80E-02	9.70E-01
	SMART	GGL	3	8.00E-02	9.80E-01
	GOTERM_CC_FAT	heterotrimeric G-protein complex	3	1.90E-01	9.10E-01
	GOTERM_CC_FAT	extrinsic to plasma membrane	3	4.00E-01	9.60E-01
**Annotation Cluster 27**	**Enrichment Score: 0.81**		**Count**	**P_Value**	**Benjamini**
	GOTERM_BP_FAT	negative regulation of BMP signaling pathway	3	1.00E-01	9.60E-01
	GOTERM_BP_FAT	regulation of BMP signaling pathway	3	1.80E-01	9.90E-01
	GOTERM_BP_FAT	negative regulation of signal transduction	9	2.10E-01	9.90E-01
**Annotation Cluster 28**	**Enrichment Score: 0.79**		**Count**	**P_Value**	**Benjamini**
	INTERPRO	WW/Rsp5/WWP	4	1.60E-01	9.90E-01
	SMART	WW	4	1.60E-01	9.60E-01
	UP_SEQ_FEATURE	domain:WW 1	3	1.70E-01	1.00E+00
	UP_SEQ_FEATURE	domain:WW 2	3	1.70E-01	1.00E+00
**Annotation Cluster 29**	**Enrichment Score: 0.78**		**Count**	**P_Value**	**Benjamini**
	UP_SEQ_FEATURE	domain:PDZ 1	4	8.00E-02	1.00E+00
	UP_SEQ_FEATURE	domain:PDZ 2	4	8.00E-02	1.00E+00
	INTERPRO	PDZ/DHR/GLGF	6	3.50E-01	1.00E+00
	SMART	PDZ	6	3.60E-01	9.80E-01
**Annotation Cluster 30**	**Enrichment Score: 0.74**		**Count**	**P_Value**	**Benjamini**
	SP_PIR_KEYWORDS	glycoprotein	125	5.40E-02	7.00E-01
	SP_PIR_KEYWORDS	membrane	175	5.80E-02	6.70E-01
	UP_SEQ_FEATURE	glycosylation site:N-linked (GlcNAc...)	118	9.10E-02	1.00E+00
	UP_SEQ_FEATURE	topological domain:Cytoplasmic	97	1.20E-01	1.00E+00
	UP_SEQ_FEATURE	topological domain:Extracellular	76	2.30E-01	1.00E+00
	UP_SEQ_FEATURE	transmembrane region	132	2.60E-01	1.00E+00
	SP_PIR_KEYWORDS	transmembrane	132	2.80E-01	8.90E-01
	GOTERM_CC_FAT	integral to membrane	144	6.10E-01	9.80E-01
	GOTERM_CC_FAT	intrinsic to membrane	149	6.20E-01	9.80E-01
**Annotation Cluster 31**	**Enrichment Score: 0.7**		**Count**	**P_Value**	**Benjamini**
	SP_PIR_KEYWORDS	muscle protein	6	1.70E-02	8.50E-01
	GOTERM_BP_FAT	muscle contraction	9	4.40E-02	8.90E-01
	GOTERM_BP_FAT	muscle system process	9	6.80E-02	9.30E-01
	GOTERM_MF_FAT	structural constituent of muscle	4	1.10E-01	9.70E-01
	GOTERM_BP_FAT	striated muscle contraction	4	1.10E-01	9.70E-01
	GOTERM_CC_FAT	contractile fiber	6	2.40E-01	9.40E-01
	GOTERM_CC_FAT	sarcomere	5	2.80E-01	9.60E-01
	GOTERM_CC_FAT	actin cytoskeleton	10	3.20E-01	9.60E-01
	GOTERM_CC_FAT	myofibril	5	3.60E-01	9.60E-01
	GOTERM_CC_FAT	contractile fiber part	5	3.70E-01	9.60E-01
	GOTERM_MF_FAT	actin binding	11	3.90E-01	1.00E+00
	GOTERM_MF_FAT	cytoskeletal protein binding	16	4.00E-01	1.00E+00
	SP_PIR_KEYWORDS	actin-binding	7	5.90E-01	9.70E-01
	GOTERM_BP_FAT	actin filament-based process	5	8.70E-01	1.00E+00
**Annotation Cluster 32**	**Enrichment Score: 0.69**		**Count**	**P_Value**	**Benjamini**
	SP_PIR_KEYWORDS	mitochondrion inner membrane	11	2.50E-02	7.00E-01
	KEGG_PATHWAY	Oxidative phosphorylation	9	3.40E-02	8.90E-01
	SP_PIR_KEYWORDS	oxidative phosphorylation	3	2.20E-01	8.80E-01
	KEGG_PATHWAY	Parkinson's disease	5	5.10E-01	9.90E-01
	KEGG_PATHWAY	Alzheimer's disease	6	5.10E-01	9.90E-01
	SP_PIR_KEYWORDS	respiratory chain	3	5.40E-01	9.70E-01

ILMN_2144573	CTBS	-0.436911641
ILMN_1722923	SRGAP3	-0.437067173
ILMN_1710482	APLP2	-0.43713701
ILMN_2101278	RGS18	-0.437180044
ILMN_2196337	C12orf11	-0.437331657
ILMN_2176063	FCGR1C	-0.437527663
ILMN_1713726	NFYA	-0.437584919
ILMN_3269395	GGT3P	-0.437719701
ILMN_3247064	SNRNP40	-0.438026191
ILMN_2053345	KCNT2	-0.438147927
ILMN_1790162	ZC3H11A	-0.43830335
ILMN_1730825	SGOL1	-0.438513186
ILMN_1677404	RAP2A	-0.438628968
ILMN_1763452	EVI2B	-0.438903937
ILMN_2102201	GPR19	-0.439076739
ILMN_1747593	ISLR	-0.439123757
ILMN_1797975	CXCR3	-0.439422238
ILMN_1732705	HCFC1	-0.439536144
ILMN_1691364	STAT1	-0.439675889
ILMN_1731745	NINJ2	-0.44012633
ILMN_1671565	RNASET2	-0.440154229
ILMN_2260313	CRYBA2	-0.44040346
ILMN_1810185	CHADL	-0.440503808
ILMN_3272374	NA	-0.440568527
ILMN_1757336	LRCH3	-0.440887308
ILMN_1754121	CSK	-0.44107005
ILMN_2398274	PYCARD	-0.441282847
ILMN_1745537	GNAT2	-0.441330596
ILMN_3236752	COAS3	-0.442053411
ILMN_2308950	AKAP12	-0.442414522
ILMN_1776354	KRTAP1-3	-0.442624768
ILMN_1717163	F13A1	-0.442743769
ILMN_2080684	IQCA1	-0.442791004
ILMN_1711517	OR6C6	-0.442881779
ILMN_1726842	TYW3	-0.442920092
ILMN_1762210	HTR3B	-0.442962137
ILMN_1765954	HSD17B3	-0.443291068
ILMN_3310015	MIR631	-0.443571896
ILMN_3242679	C2orf90	-0.443736361
ILMN_3199780	CAP1	-0.443768172
ILMN_1690921	STAT2	-0.444000062
ILMN_1769702	GPAA1	-0.444254816
ILMN_1730799	PHF12	-0.444499447
ILMN_1765109	TNFRSF25	-0.444665991
ILMN_3236404	SNORD7	-0.444674545
ILMN_1763487	CTLA4	-0.4451135
ILMN_1661888	MEF2A	-0.44517223
ILMN_3237946	PXDN	-0.445277665
ILMN_1689655	HLA-DRA	-0.445342326
ILMN_1736700	ALDOA	-0.445365476
ILMN_2336186	LCMT1	-0.445481891
ILMN_1754507	IRF4	-0.445733954
ILMN_3239606	BC037297	-0.445753765
ILMN_3261175	CCDC85C	-0.445880735
ILMN_3246465	SNHG12	-0.445904824
ILMN_1662427	PTP4A3	-0.4459451
ILMN_1694571	SPDYE7P	-0.446726716
ILMN_1805673	ALKBH4	-0.4468412
ILMN_1763640	NCKAP5L	-0.447244345
ILMN_2337880	NRG2	-0.447481413
ILMN_2066066	HLA-DRB6	-0.447599173
ILMN_1721629	ZNF654	-0.447685866
ILMN_2391419	SYNE2	-0.4480415
ILMN_1684897	SPDYE1	-0.448528464
ILMN_1670033	HIST1H4I	-0.448623819
ILMN_1798975	EGFR	-0.44894061
ILMN_2321578	P2RY10	-0.449123894
ILMN_2379644	CD74	-0.449129973
ILMN_1703593	BAIAP2L1	-0.449255977
ILMN_2387452	MBD2	-0.449922339
ILMN_1779989	GLI1	-0.450124948
ILMN_1719963	LOC29034	-0.450325871
ILMN_1839051	AK021888	-0.450326085
ILMN_1695443	YSK4	-0.4506986
ILMN_2255877	OSGIN1	-0.451726441
ILMN_1670718	FAM69C	-0.451934775
ILMN_2043060	PLCH1	-0.452078932
ILMN_2295987	NBPF1	-0.452200755
ILMN_1684663	ZDHHC13	-0.452328193
ILMN_3240989	FLJ00326	-0.452535113
ILMN_1714602	CD86	-0.453682073
ILMN_3248122	MED1	-0.453729228
ILMN_1663916	ARHGAP9	-0.453881877
ILMN_3177285	HNRNPR	-0.454162676
ILMN_1770922	TMEM45A	-0.454704738
ILMN_2344455	G3BP1	-0.454884534
ILMN_2343677	SOX5	-0.455128109
ILMN_1809477	CARHSP1	-0.455253242
ILMN_1825249	NA	-0.455276184
ILMN_1781758	ROPN1B	-0.455571115
ILMN_2255811	TRAF5	-0.455603331
ILMN_3239548	STAG3L2	-0.455755753
ILMN_1701308	COL1A1	-0.455970385

	GOTERM_BP_FAT	RNA elongation from RNA polymerase II promoter	3	3.70E-01	9.80E-01
	GOTERM_CC_FAT	DNA-directed RNA polymerase II, holoenzyme	4	3.70E-01	9.30E-01
	GOTERM_BP_FAT	RNA elongation	3	4.00E-01	9.90E-01
**Annotation Cluster 26**	**Enrichment Score: 1.03**		**Count**	**P_Value**	**Benjamini**
	GOTERM_BP_FAT	skeletal system development	17	1.20E-02	5.40E-01
	GOTERM_BP_FAT	bone development	7	1.10E-01	9.00E-01
	GOTERM_BP_FAT	ossification	6	1.90E-01	9.40E-01
	GOTERM_BP_FAT	osteoblast differentiation	3	3.10E-01	9.70E-01
**Annotation Cluster 27**	**Enrichment Score: 1**		**Count**	**P_Value**	**Benjamini**
	BIOCARTA	IFN alpha signaling pathway	3	5.40E-02	9.10E-01
	GOTERM_BP_FAT	response to virus	7	7.10E-02	8.70E-01
	GOTERM_BP_FAT	JAK-STAT cascade	4	8.50E-02	8.80E-01
	KEGG_PATHWAY	Jak-STAT signaling pathway	7	3.20E-01	8.00E-01
**Annotation Cluster 28**	**Enrichment Score: 0.98**		**Count**	**P_Value**	**Benjamini**
	UP_SEQ_FEATURE	domain:SH3	9	7.40E-02	9.80E-01
	SP_PIR_KEYWORDS	sh3 domain	10	7.60E-02	4.40E-01
	INTERPRO	Src homology-3 domain	10	9.40E-02	9.70E-01
	SMART	SH3	10	2.30E-01	9.70E-01
**Annotation Cluster 29**	**Enrichment Score: 0.98**		**Count**	**P_Value**	**Benjamini**
	UP_SEQ_FEATURE	domain:FCH	3	8.70E-02	9.90E-01
	INTERPRO	Fps/Fes/Fer/CIP4 homology	3	9.80E-02	9.70E-01
	SMART	FCH	3	1.40E-01	9.90E-01
**Annotation Cluster 30**	**Enrichment Score: 0.97**		**Count**	**P_Value**	**Benjamini**
	SP_PIR_KEYWORDS	tyrosine-specific protein kinase	5	3.00E-02	2.50E-01
	GOTERM_MF_FAT	transmembrane receptor protein tyrosine kinase activity	6	3.30E-02	7.80E-01
	INTERPRO	Tyrosine protein kinase, active site	7	3.70E-02	9.20E-01
	SP_PIR_KEYWORDS	tyrosine-protein kinase	7	5.70E-02	3.70E-01
	GOTERM_BP_FAT	transmembrane receptor protein tyrosine kinase signaling pathway	11	7.70E-02	8.80E-01
	INTERPRO	Tyrosine protein kinase	7	8.00E-02	9.80E-01
	SP_PIR_KEYWORDS	autophosphorylation	4	1.20E-01	5.40E-01
	SP_PIR_KEYWORDS	phosphotransferase	9	1.20E-01	5.40E-01
	GOTERM_BP_FAT	enzyme linked receptor protein signaling pathway	14	1.30E-01	9.20E-01
	GOTERM_MF_FAT	protein tyrosine kinase activity	8	1.60E-01	9.40E-01
	SMART	TyrKc	7	1.60E-01	9.70E-01
	INTERPRO	Protein kinase, ATP binding site	16	1.80E-01	9.90E-01
	UP_SEQ_FEATURE	domain:Protein kinase	16	1.90E-01	1.00E+00
	SP_PIR_KEYWORDS	ATP	9	2.30E-01	6.70E-01
	INTERPRO	Protein kinase, core	16	2.30E-01	9.90E-01
	UP_SEQ_FEATURE	binding site:ATP	17	2.70E-01	1.00E+00
**Annotation Cluster 31**	**Enrichment Score: 0.92**		**Count**	**P_Value**	**Benjamini**
	SP_PIR_KEYWORDS	SH2 domain	7	5.70E-02	3.70E-01
	UP_SEQ_FEATURE	domain:SH2	6	9.00E-02	9.90E-01
	INTERPRO	SH2 motif	6	1.50E-01	9.80E-01
	SMART	SH2	6	2.70E-01	9.70E-01
**Annotation Cluster 32**	**Enrichment Score: 0.92**		**Count**	**P_Value**	**Benjamini**
	INTERPRO	Laminin G, thrombospondin-type, N-terminal	3	1.10E-01	9.70E-01
	UP_SEQ_FEATURE	domain:TSP N-terminal	3	1.10E-01	9.90E-01
	SMART	TSPN	3	1.50E-01	9.90E-01
**Annotation Cluster 33**	**Enrichment Score: 0.91**		**Count**	**P_Value**	**Benjamini**
	GOTERM_BP_FAT	hemopoietic or lymphoid organ development	12	8.90E-02	8.90E-01
	GOTERM_BP_FAT	myeloid cell differentiation	6	1.00E-01	9.00E-01
	GOTERM_BP_FAT	immune system development	12	1.20E-01	9.20E-01
	GOTERM_BP_FAT	leukocyte differentiation	7	1.40E-01	9.30E-01
	GOTERM_BP_FAT	hemopoiesis	10	1.80E-01	9.40E-01
**Annotation Cluster 34**	**Enrichment Score: 0.9**		**Count**	**P_Value**	**Benjamini**
	GOTERM_BP_FAT	protein modification by small protein conjugation	10	8.70E-03	4.70E-01
	GOTERM_BP_FAT	protein modification by small protein conjugation or removal	10	2.70E-02	7.60E-01
	GOTERM_BP_FAT	proteolysis	38	5.00E-02	8.40E-01
	SP_PIR_KEYWORDS	ubl conjugation pathway	19	8.10E-02	4.40E-01
	GOTERM_BP_FAT	macromolecule catabolic process	27	1.40E-01	9.20E-01
	GOTERM_BP_FAT	modification-dependent protein catabolic process	20	1.90E-01	9.40E-01
	GOTERM_BP_FAT	modification-dependent macromolecule catabolic process	20	1.90E-01	9.40E-01
	SP_PIR_KEYWORDS	ligase	11	2.20E-01	6.70E-01
	GOTERM_BP_FAT	cellular macromolecule catabolic process	24	2.20E-01	9.50E-01
	GOTERM_BP_FAT	proteolysis involved in cellular protein catabolic process	20	2.50E-01	9.60E-01
	GOTERM_BP_FAT	cellular protein catabolic process	20	2.50E-01	9.60E-01
	GOTERM_BP_FAT	protein catabolic process	20	3.00E-01	9.70E-01
	GOTERM_BP_FAT	ubiquitin-dependent protein catabolic process	8	4.70E-01	9.90E-01
**Annotation Cluster 35**	**Enrichment Score: 0.88**		**Count**	**P_Value**	**Benjamini**
	SP_PIR_KEYWORDS	lysosome	8	7.60E-02	4.50E-01
	GOTERM_CC_FAT	lysosome	10	1.20E-01	7.60E-01
	GOTERM_CC_FAT	lytic vacuole	10	1.20E-01	7.60E-01
	GOTERM_CC_FAT	vacuole	11	1.40E-01	8.00E-01
	SP_PIR_KEYWORDS	endosome	8	2.60E-01	6.90E-01
**Annotation Cluster 36**	**Enrichment Score: 0.86**		**Count**	**P_Value**	**Benjamini**
	SP_PIR_KEYWORDS	zymogen	13	5.20E-03	7.00E-02
	UP_SEQ_FEATURE	propeptide:Activation peptide	7	9.40E-03	6.60E-01
	SP_PIR_KEYWORDS	serine proteinase	5	4.40E-02	3.20E-01
	SP_PIR_KEYWORDS	Protease	18	9.50E-02	4.80E-01
	SP_PIR_KEYWORDS	Serine protease	7	1.20E-01	5.40E-01
	UP_SEQ_FEATURE	domain:Peptidase S1	6	1.20E-01	9.90E-01
	INTERPRO	Peptidase S1A, chymotrypsin	6	1.30E-01	9.70E-01
	INTERPRO	Peptidase S1/S6, chymotrypsin/Hap, active site	6	1.30E-01	9.70E-01
	INTERPRO	Peptidase S1 and S6, chymotrypsin/Hap	6	1.70E-01	9.80E-01
	GOTERM_MF_FAT	peptidase activity	20	1.90E-01	9.60E-01
	GOTERM_MF_FAT	serine-type peptidase activity	8	2.00E-01	9.60E-01
	GOTERM_MF_FAT	serine hydrolase activity	8	2.10E-01	9.50E-01
	UP_SEQ_FEATURE	active site:Charge relay system	8	2.10E-01	1.00E+00
	GOTERM_MF_FAT	serine-type endopeptidase activity	7	2.30E-01	9.60E-01
	SP_PIR_KEYWORDS	hydrolase	44	2.30E-01	6.70E-01
	PIR_SUPERFAMILY	PIRSF001135:trypsin	3	2.70E-01	1.00E+00
	SMART	Tryp_SPc	6	3.00E-01	9.80E-01
	GOTERM_MF_FAT	peptidase activity, acting on L-amino acid peptides	18	3.00E-01	9.60E-01

ILMN_1658499	SYT13	0.441593161
ILMN_1805903	DOK2	0.441424544
ILMN_1799369	ANKMY1	0.441239927
ILMN_2041648	TMPRSS7	0.440963818
ILMN_1758102	GLB1	0.440944857
ILMN_1751081	EHMT2	0.440844526
ILMN_1684885	KRTAP4-8	0.440816579
ILMN_1803774	STK16	0.440701486
ILMN_1851107	AI681560	0.44062437
ILMN_2216815	MAP7	0.440318056
ILMN_1715376	LRRIQ3	0.440221245
ILMN_1759448	CPZ	0.440144125
ILMN_1681454	RP1	0.439371358
ILMN_1703684	NAP1L2	0.4392403
ILMN_2109343	SGTB	0.439174624
ILMN_1790202	C1orf35	0.438999813
ILMN_1669747	UNC13B	0.438697032
ILMN_1677043	AKR7A2	0.437996331
ILMN_1654151	COX6C	0.437959279
ILMN_3241944	Nbla00301	0.437957082
ILMN_1813746	CORO2A	0.437868289
ILMN_1705397	PDK2	0.437701435
ILMN_1782938	SLC16A10	0.437492729
ILMN_1801391	SKP2	0.437108142
ILMN_2241317	FOXK2	0.436782294
ILMN_1733560	SPG21	0.43638791
ILMN_2413833	TOX3	0.436382044
ILMN_1899387	BC039440	0.43634999
ILMN_1727466	KCNMB4	0.436315253
ILMN_1673322	UNQ9368	0.436305924
ILMN_1762207	SGSM1	0.436175367
ILMN_1683858	EFCAB6	0.436131279
ILMN_3261022	NA	0.436024685
ILMN_1824029	AW290884	0.435984414
ILMN_1873411	DB350505	0.435970899
ILMN_2153280	KIAA0090	0.435959158
ILMN_3245983	NEURL1B	0.435955767
ILMN_1654823	MGC70863	0.435934149
ILMN_1877996	AW195018	0.435860981
ILMN_1908274	AW236020	0.435630963
ILMN_1759542	PTGER3	0.435581444
ILMN_1764029	FLJ40448	0.435149981
ILMN_3249203	LOC728855	0.434856836
ILMN_1702489	TRIM63	0.434497524
ILMN_2278246	PDE11A	0.434439851
ILMN_1696582	BTBD11	0.434194032
ILMN_3228269	CR625008	0.43419009
ILMN_1745988	NUDT12	0.434066406
ILMN_2354478	CYFIP2	0.434011512
ILMN_1803676	ENOSF1	0.434002492
ILMN_2213759	DBX2	0.433953316
ILMN_1705172	SLC26A7	0.433886849
ILMN_1911873	CD252206	0.433874027
ILMN_1658957	DDX11L2	0.433765433
ILMN_1685854	C5orf53	0.433703083
ILMN_1679646	FTCD	0.43369003
ILMN_1772506	ATP5I	0.433658717
ILMN_2193936	HRG	0.433628036
ILMN_3296870	NA	0.433274617
ILMN_1683096	ASB1	0.433127731
ILMN_1760087	SLC26A3	0.433029213
ILMN_2110050	ZNF460	0.433028864
ILMN_1903159	PPM1A	0.432898836
ILMN_1683046	OR6Y1	0.432834289
ILMN_1812312	NDUFS4	0.432642041
ILMN_1829052	BU753580	0.432232929
ILMN_3241087	INPP5J	0.432066535
ILMN_1758272	MYPN	0.431785919
ILMN_3250412	CNGB1	0.431611078
ILMN_1753627	DOLK	0.431414856
ILMN_1685396	CLIC5	0.431157419
ILMN_1686948	CASC4	0.430615857
ILMN_1883780	AK311113	0.430490426
ILMN_1809662	MZT1	0.430372953
ILMN_1704247	LAMA3	0.430201402
ILMN_1763083	MYRIP	0.43011137
ILMN_1769394	PLCD1	0.430016239
ILMN_1696318	LOC100130691	0.429989414
ILMN_1701991	SYNJ1	0.429911672
ILMN_3305768	GSTA7P	0.429762901
ILMN_1787576	CLCNKA	0.429002198
ILMN_1731314	OR10H3	0.428923475
ILMN_1786034	GPRC5B	0.42877914
ILMN_1726603	ATP5I	0.428726129
ILMN_1840957	LOC286083	0.428359313
ILMN_2193817	CLEC4G	0.428190933
ILMN_2087608	C6orf221	0.428072317
ILMN_2396697	GRIK2	0.427956227
ILMN_1754678	ZNF100	0.42781589
ILMN_2052871	TMEM116	0.427795462
ILMN_1662897	ERCC4	0.427786551
ILMN_1753648	SPRYD5	0.427681584
ILMN_1828111	BE466189	0.427015722
ILMN_3248223	SCARNA12	0.426832178
ILMN_1732806	FNDC7	0.426766748
ILMN_1831907	AI935777	0.42663742
ILMN_1728107	GNG7	0.426545897
ILMN_1788638	FAM75A3	0.426437036
ILMN_1657527	CLEC12B	0.426008027
ILMN_1694973	DEFB127	0.425971914
ILMN_1746888	PCOLCE2	0.425761025

	KEGG_PATHWAY	Huntington's disease	6	6.00E-01	9.90E-01
**Annotation Cluster 33**	**Enrichment Score: 0.67**		**Count**	**P_Value**	**Benjamini**
	INTERPRO	Helix-turn-helix motif, lambda-like repressor	4	5.40E-02	9.60E-01
	INTERPRO	Homeobox, conserved site	11	8.20E-02	9.70E-01
	INTERPRO	Homeobox	11	8.80E-02	9.70E-01
	SP_PIR_KEYWORDS	Homeobox	11	8.80E-02	7.40E-01
	SMART	HOX	11	9.20E-02	9.70E-01
	INTERPRO	Homeodomain-related	11	9.40E-02	9.80E-01
	UP_SEQ_FEATURE	DNA-binding region:Homeobox	8	2.10E-01	1.00E+00
	GOTERM_BP_FAT	pattern specification process	8	5.40E-01	1.00E+00
	GOTERM_BP_FAT	regionalization	6	5.70E-01	1.00E+00
	GOTERM_MF_FAT	sequence-specific DNA binding	16	6.90E-01	1.00E+00
	GOTERM_BP_FAT	anterior/posterior pattern formation	4	7.00E-01	1.00E+00
	GOTERM_MF_FAT	transcription factor activity	22	8.90E-01	1.00E+00
**Annotation Cluster 34**	**Enrichment Score: 0.67**		**Count**	**P_Value**	**Benjamini**
	GOTERM_BP_FAT	cellular amino acid biosynthetic process	4	1.40E-01	9.80E-01
	GOTERM_BP_FAT	organic acid biosynthetic process	7	2.10E-01	9.90E-01
	GOTERM_BP_FAT	carboxylic acid biosynthetic process	7	2.10E-01	9.90E-01
	GOTERM_BP_FAT	amine biosynthetic process	4	3.40E-01	9.90E-01
**Annotation Cluster 35**	**Enrichment Score: 0.66**		**Count**	**P_Value**	**Benjamini**
	GOTERM_BP_FAT	calcium ion transport	8	7.30E-02	9.30E-01
	GOTERM_BP_FAT	di-, tri-valent inorganic cation transport	9	8.40E-02	9.40E-01
	GOTERM_BP_FAT	metal ion transport	17	1.40E-01	9.80E-01
	SP_PIR_KEYWORDS	calcium transport	5	1.70E-01	8.50E-01
	SP_PIR_KEYWORDS	calcium channel	3	4.60E-01	9.50E-01
	GOTERM_MF_FAT	metal ion transmembrane transporter activity	10	5.20E-01	1.00E+00
	GOTERM_MF_FAT	calcium channel activity	3	6.40E-01	1.00E+00
**Annotation Cluster 36**	**Enrichment Score: 0.66**		**Count**	**P_Value**	**Benjamini**
	GOTERM_CC_FAT	nerve terminal	4	8.00E-02	8.90E-01
	GOTERM_CC_FAT	terminal button	3	1.50E-01	9.10E-01
	GOTERM_CC_FAT	axon part	4	1.80E-01	9.10E-01
	GOTERM_CC_FAT	perikaryon	3	2.00E-01	9.10E-01
	GOTERM_CC_FAT	axon	7	2.70E-01	9.50E-01
	GOTERM_CC_FAT	dendrite	7	2.90E-01	9.50E-01
	GOTERM_CC_FAT	cell soma	7	3.10E-01	9.60E-01
	GOTERM_CC_FAT	cell projection part	8	4.60E-01	9.60E-01
**Annotation Cluster 37**	**Enrichment Score: 0.66**		**Count**	**P_Value**	**Benjamini**
	INTERPRO	NAD(P)-binding domain	9	4.20E-02	9.50E-01
	UP_SEQ_FEATURE	nucleotide phosphate-binding region:NAD	5	1.30E-01	1.00E+00
	GOTERM_MF_FAT	NAD or NADH binding	4	1.40E-01	9.70E-01
	SP_PIR_KEYWORDS	nad	6	5.20E-01	9.60E-01
	GOTERM_MF_FAT	cofactor binding	8	5.20E-01	1.00E+00
	GOTERM_MF_FAT	coenzyme binding	6	5.50E-01	1.00E+00
**Annotation Cluster 38**	**Enrichment Score: 0.63**		**Count**	**P_Value**	**Benjamini**
	GOTERM_BP_FAT	ion transport	35	1.30E-03	9.20E-01
	GOTERM_BP_FAT	cation transport	25	8.30E-03	9.20E-01
	SP_PIR_KEYWORDS	ion transport	25	1.20E-02	8.20E-01
	GOTERM_BP_FAT	transmembrane transport	24	2.10E-02	9.20E-01
	GOTERM_BP_FAT	metal ion transport	17	1.40E-01	9.80E-01
	SP_PIR_KEYWORDS	voltage-gated channel	7	1.80E-01	8.60E-01
	SP_PIR_KEYWORDS	ionic channel	12	1.80E-01	8.50E-01
	SP_PIR_KEYWORDS	transport	49	1.90E-01	8.50E-01
	GOTERM_MF_FAT	ion channel activity	13	3.60E-01	9.90E-01
	GOTERM_MF_FAT	substrate specific channel activity	13	4.00E-01	1.00E+00
	GOTERM_MF_FAT	voltage-gated ion channel activity	7	4.40E-01	1.00E+00
	GOTERM_MF_FAT	voltage-gated channel activity	7	4.40E-01	1.00E+00
	GOTERM_MF_FAT	channel activity	13	4.50E-01	1.00E+00
	GOTERM_MF_FAT	passive transmembrane transporter activity	13	4.50E-01	1.00E+00
	GOTERM_MF_FAT	gated channel activity	10	4.70E-01	1.00E+00
	GOTERM_MF_FAT	metal ion transmembrane transporter activity	10	5.20E-01	1.00E+00
	GOTERM_MF_FAT	alkali metal ion binding	7	5.90E-01	1.00E+00
	GOTERM_MF_FAT	cation channel activity	8	6.20E-01	1.00E+00
	GOTERM_CC_FAT	ion channel complex	6	6.70E-01	9.80E-01
	GOTERM_MF_FAT	potassium channel activity	4	7.10E-01	1.00E+00
	GOTERM_MF_FAT	voltage-gated cation channel activity	4	7.70E-01	1.00E+00
	GOTERM_BP_FAT	potassium ion transport	4	7.80E-01	1.00E+00
	SP_PIR_KEYWORDS	potassium transport	3	7.90E-01	9.90E-01
	SP_PIR_KEYWORDS	potassium	3	8.30E-01	1.00E+00
	GOTERM_MF_FAT	potassium ion binding	3	8.70E-01	1.00E+00
**Annotation Cluster 39**	**Enrichment Score: 0.62**		**Count**	**P_Value**	**Benjamini**
	INTERPRO	Complement C1q protein	3	2.00E-01	9.90E-01
	SMART	C1Q	3	2.10E-01	9.80E-01
	INTERPRO	Tumour necrosis factor-like	3	3.30E-01	1.00E+00
**Annotation Cluster 40**	**Enrichment Score: 0.61**		**Count**	**P_Value**	**Benjamini**
	SP_PIR_KEYWORDS	laminin egf-like domain	3	1.70E-01	8.60E-01
	INTERPRO	EGF-like, laminin	3	2.90E-01	1.00E+00
	SMART	EGF_Lam	3	2.90E-01	9.80E-01
**Annotation Cluster 41**	**Enrichment Score: 0.61**		**Count**	**P_Value**	**Benjamini**
	GOTERM_BP_FAT	catecholamine metabolic process	3	2.20E-01	9.90E-01
	GOTERM_BP_FAT	diol metabolic process	3	2.20E-01	9.90E-01
	GOTERM_BP_FAT	catechol metabolic process	3	2.20E-01	9.90E-01
	GOTERM_BP_FAT	phenol metabolic process	3	2.30E-01	9.90E-01
	GOTERM_BP_FAT	biogenic amine metabolic process	5	2.40E-01	9.90E-01
	GOTERM_BP_FAT	cellular amino acid derivative metabolic process	6	4.20E-01	1.00E+00
**Annotation Cluster 42**	**Enrichment Score: 0.6**		**Count**	**P_Value**	**Benjamini**
	GOTERM_CC_FAT	plasma membrane part	71	9.60E-02	9.00E-01
	GOTERM_CC_FAT	intrinsic to plasma membrane	36	4.00E-01	9.60E-01
	GOTERM_CC_FAT	integral to plasma membrane	35	4.10E-01	9.60E-01
**Annotation Cluster 43**	**Enrichment Score: 0.58**		**Count**	**P_Value**	**Benjamini**
	GOTERM_MF_FAT	enzyme inhibitor activity	11	2.00E-01	9.80E-01
	GOTERM_MF_FAT	endopeptidase inhibitor activity	7	2.00E-01	9.80E-01
	GOTERM_MF_FAT	peptidase inhibitor activity	7	2.40E-01	9.80E-01
	GOTERM_MF_FAT	serine-type endopeptidase inhibitor activity	5	2.40E-01	9.80E-01
	SP_PIR_KEYWORDS	protease inhibitor	5	2.80E-01	8.90E-01
	UP_SEQ_FEATURE	site:Reactive bond	3	3.20E-01	1.00E+00
	KEGG_PATHWAY	Complement and coagulation cascades	4	3.20E-01	1.00E+00
	SP_PIR_KEYWORDS	Serine protease inhibitor	4	3.30E-01	9.10E-01
**Annotation Cluster 44**	**Enrichment Score: 0.58**		**Count**	**P_Value**	**Benjamini**
	INTERPRO	Bromodomain, conserved site	3	2.40E-01	9.90E-01
	SP_PIR_KEYWORDS	bromodomain	3	2.60E-01	8.80E-01
	INTERPRO	Bromodomain	3	2.80E-01	1.00E+00

ILMN_2078995	CSRNP3	-0.45609699
ILMN_1812392	TMSB10	-0.456257337
ILMN_1883937	CR936671	-0.456703896
ILMN_1706203	SOBP	-0.456818587
ILMN_1724480	AXIN2	-0.456923436
ILMN_1702198	HMGN1	-0.457004613
ILMN_1682054	SRI	-0.457053564
ILMN_2319913	DGKA	-0.457298732
ILMN_1772964	CCL8	-0.457723629
ILMN_2143155	KIF11	-0.457774057
ILMN_1697268	EMILIN2	-0.458176406
ILMN_3258356	RPSAP52	-0.458239112
ILMN_1784655	TLCD1	-0.458281729
ILMN_1708369	EPS15L1	-0.458408114
ILMN_1715169	HLA-DRB1	-0.458628491
ILMN_1662843	CD53	-0.458653285
ILMN_3244348	TAF1D	-0.458800884
ILMN_1724598	RABL2A	-0.458868127
ILMN_2226304	ANKRD50	-0.459055592
ILMN_2337789	MARCH2	-0.459182086
ILMN_2364174	CSNK1A1	-0.459226166
ILMN_1761464	CD74	-0.459385781
ILMN_1737041	HABP2	-0.460021875
ILMN_1710937	IFI16	-0.460998065
ILMN_1800091	RARRES1	-0.461021387
ILMN_3242591	NA	-0.461051557
ILMN_2238640	RFX3	-0.461174142
ILMN_2297864	MTMR14	-0.461194221
ILMN_1761788	MOXD1	-0.461553353
ILMN_1736836	BRF1	-0.461718356
ILMN_1815412	TMEM218	-0.461763883
ILMN_1663390	CDC20	-0.46186967
ILMN_2388701	ST3GAL5	-0.462425408
ILMN_1769517	PRKDC	-0.462463463
ILMN_1682799	STAMBPL1	-0.462774488
ILMN_1782581	DUSP7	-0.46291532
ILMN_2083066	IGLL3P	-0.462915511
ILMN_1752526	RNF144B	-0.462925213
ILMN_1737918	C1QA	-0.463044489
ILMN_2316236	HOPX	-0.463097874
ILMN_1759883	SRP9	-0.463496025
ILMN_1702618	ASB4	-0.463498067
ILMN_3256325	CYB561D1	-0.463742414
ILMN_1695311	HLA-DMA	-0.464246623
ILMN_3251599	LOC388152	-0.46471855
ILMN_2381286	DMD	-0.464834583
ILMN_2359014	TBCE	-0.464905909
ILMN_1801257	CENPA	-0.465000726
ILMN_1723156	PTPRA	-0.465355933
ILMN_1716687	TPM1	-0.465411579
ILMN_1776888	RHOXF1	-0.465480782
ILMN_1711361	ZNF319	-0.466236124
ILMN_1783954	FOXN3	-0.466283341
ILMN_1726879	PRR15	-0.466380995
ILMN_1708537	RBPJ	-0.466507454
ILMN_1803968	SSBP3	-0.466736021
ILMN_1722537	FLJ40504	-0.467007505
ILMN_2206098	ATG10	-0.467139812
ILMN_1754102	TGIF1	-0.467399142
ILMN_2347999	IFNAR2	-0.467831881
ILMN_1677997	MAZ	-0.468117069
ILMN_2413808	CD53	-0.468175008
ILMN_1707760	OR4C15	-0.469134446
ILMN_2367233	ZNF654	-0.46946544
ILMN_1709024	DKFZp434I1020	-0.469761866
ILMN_1667081	CCND2	-0.46980691
ILMN_1784105	SLC38A7	-0.47016971
ILMN_1793922	AK021896	-0.470207814
ILMN_1678728	FOXA2	-0.470274376
ILMN_1712184	HIST1H3C	-0.470448152
ILMN_3225143	AK311627	-0.471440264
ILMN_1699574	NRP1	-0.471670828
ILMN_1691714	ACSL4	-0.471780805
ILMN_1773079	COL3A1	-0.472641702
ILMN_1908488	IAH1	-0.473159568
ILMN_1680274	abParts	-0.473824902
ILMN_3274814	LOC100133286	-0.473836073
ILMN_1766275	PIK3CD	-0.474016198
ILMN_1687558	SYNE1	-0.474302276
ILMN_1651346	TICAM2	-0.474480748
ILMN_1788108	TXNDC5	-0.474542966
ILMN_1786789	FAM102B	-0.476121946
ILMN_1692260	MAFG	-0.476463806
ILMN_1785272	COL1A2	-0.477066807
ILMN_1716265	PGM2L1	-0.477191123
ILMN_2148459	B2M	-0.478295388
ILMN_1754249	TARP	-0.479386166
ILMN_1773389	PLTP	-0.479483924
ILMN_1734740	GPR65	-0.479722378
ILMN_1680435	NPEPPS	-0.48020652
ILMN_3200242	BC049386	-0.480531596
ILMN_1689342	NUBP1	-0.480553938
ILMN_3244433	FLJ00326	-0.480606306
ILMN_2170515	METTL11A	-0.480707279
ILMN_1796082	DHX57	-0.481059964
ILMN_1761069	UVRAG	-0.482496131
ILMN_1742032	CCDC144B	-0.483369312
ILMN_2137312	KCNJ5	-0.483594844
ILMN_1728714	SSSCA1	-0.484169568
ILMN_1729611	REEP4	-0.48427152
ILMN_1708954	GAR1	-0.484966189

	SP_PIR_KEYWORDS	cleavage on pair of basic residues	8	5.10E-01	8.80E-01
	GOTERM_MF_FAT	endopeptidase activity	11	5.40E-01	9.90E-01
**Annotation Cluster 37**	**Enrichment Score: 0.85**		**Count**	**P_Value**	**Benjamini**
	GOTERM_BP_FAT	protein modification by small protein conjugation	10	8.70E-03	4.70E-01
	GOTERM_BP_FAT	protein modification by small protein conjugation or removal	10	2.70E-02	7.60E-01
	GOTERM_BP_FAT	protein ubiquitination	8	4.00E-02	8.20E-01
	GOTERM_MF_FAT	ubiquitin-protein ligase activity	7	2.00E-01	9.60E-01
	GOTERM_MF_FAT	small conjugating protein ligase activity	7	2.80E-01	9.60E-01
	GOTERM_MF_FAT	acid-amino acid ligase activity	8	2.90E-01	9.60E-01
	GOTERM_MF_FAT	ligase activity, forming carbon-nitrogen bonds	8	4.20E-01	9.70E-01
	GOTERM_CC_FAT	ubiquitin ligase complex	4	4.40E-01	9.30E-01
	KEGG_PATHWAY	Ubiquitin mediated proteolysis	4	7.80E-01	9.60E-01
**Annotation Cluster 38**	**Enrichment Score: 0.81**		**Count**	**P_Value**	**Benjamini**
	GOTERM_MF_FAT	MHC protein binding	7	4.00E-05	1.10E-02
	GOTERM_BP_FAT	regulation of T cell differentiation	5	4.60E-02	8.50E-01
	GOTERM_BP_FAT	T cell activation	8	5.10E-02	8.40E-01
	GOTERM_BP_FAT	regulation of lymphocyte differentiation	5	8.70E-02	8.90E-01
	GOTERM_BP_FAT	leukocyte differentiation	7	1.40E-01	9.30E-01
	GOTERM_BP_FAT	cell activation	12	1.50E-01	9.30E-01
	GOTERM_BP_FAT	regulation of leukocyte activation	8	1.50E-01	9.30E-01
	GOTERM_BP_FAT	T cell differentiation in the thymus	3	1.60E-01	9.30E-01
	GOTERM_BP_FAT	lymphocyte activation	9	1.60E-01	9.30E-01
	GOTERM_BP_FAT	hemopoiesis	10	1.80E-01	9.40E-01
	GOTERM_BP_FAT	regulation of cell activation	8	1.90E-01	9.40E-01
	GOTERM_BP_FAT	leukocyte activation	10	2.00E-01	9.40E-01
	GOTERM_BP_FAT	regulation of T cell activation	6	2.00E-01	9.40E-01
	GOTERM_BP_FAT	regulation of lymphocyte activation	7	2.00E-01	9.40E-01
	GOTERM_BP_FAT	positive regulation of T cell differentiation	3	2.10E-01	9.50E-01
	GOTERM_BP_FAT	positive regulation of lymphocyte differentiation	3	2.40E-01	9.60E-01
	GOTERM_BP_FAT	T cell differentiation	4	2.50E-01	9.60E-01
	GOTERM_BP_FAT	positive regulation of developmental process	9	4.70E-01	9.90E-01
	GOTERM_BP_FAT	lymphocyte differentiation	4	5.20E-01	9.90E-01
	GOTERM_BP_FAT	positive regulation of leukocyte activation	4	5.40E-01	9.90E-01
	GOTERM_BP_FAT	positive regulation of cell activation	4	5.70E-01	9.90E-01
	GOTERM_BP_FAT	positive regulation of cell differentiation	7	5.70E-01	9.90E-01
	GOTERM_BP_FAT	positive regulation of T cell activation	3	6.00E-01	1.00E+00
	GOTERM_BP_FAT	positive regulation of lymphocyte activation	3	7.30E-01	1.00E+00

ILMN_1680420	NUDT13	0.42569911
ILMN_2401883	IL25	0.4256847
ILMN_3308753	SNORD114-1	0.425533706
ILMN_1661788	WNT5B	0.425031878
ILMN_2182647	PINX1	0.424800304
ILMN_1769082	TXNDC5	0.424728728
ILMN_3237665	COX7A2L	0.42467603
ILMN_2298301	BNC1	0.424609751
ILMN_1711318	PHF8	0.424551545
ILMN_1793435	KIAA1107	0.424337458
ILMN_1693274	USHBP1	0.424336115
ILMN_1827211	U43604	0.42408094
ILMN_2131123	AMY2A	0.424077168
ILMN_1733333	CALML3	0.423894869
ILMN_1681437	DCXR	0.42381519
ILMN_1676563	HTRA1	0.423765639
ILMN_2410428	LOC728407	0.423732925
ILMN_1847602	HAR1A	0.423689387
ILMN_1728798	TEKT4P2	0.42364087
ILMN_1753877	MYADML2	0.423535332
ILMN_2358278	RSRC2	0.423224566
ILMN_1752935	TMEM30B	0.42321611
ILMN_3235350	TLX1NB	0.423032934
ILMN_1652181	KIF2B	0.422911015
ILMN_1662161	TBC1D13	0.422790791
ILMN_3292963	RPS6KB1	0.422434032
ILMN_1713873	THSD7B	0.422172384
ILMN_1895935	CB242622	0.422123986
ILMN_1684255	MYL4	0.422114125
ILMN_1758189	OR4F17	0.421934121
ILMN_1686259	PRODH2	0.421887774
ILMN_1901382	GRIN2A	0.421878257
ILMN_1697585	ACTR6	0.421792229
ILMN_1683755	SOX13	0.421747443
ILMN_1658800	BRPF3	0.421732732
ILMN_2323979	WARS2	0.421483524
ILMN_2070349	POLR3G	0.421084854
ILMN_3243606	SAMD15	0.421054105
ILMN_1686906	TP53INP2	0.420975536
ILMN_1764494	ATP5A1	0.420793797
ILMN_1766178	C22orf43	0.420742775
ILMN_2153495	WNT7B	0.420560874
ILMN_2343563	ANAPC11	0.420373087
ILMN_1759396	NNT	0.420266479
ILMN_2289301	TCERG1	0.420233168
ILMN_1685978	ATPIF1	0.420093092
ILMN_1685974	SLC25A33	0.419985699
ILMN_2195821	C5orf41	0.419843848
ILMN_1849068	BF978818	0.419482982
ILMN_1688205	KLK10	0.419176004
ILMN_3298560	LOC729291	0.419170092
ILMN_1739573	TNRC6A	0.419162804
ILMN_1717485	AL832378	0.418872103
ILMN_2155998	PSMD6	0.418840384
ILMN_1915996	AI435238	0.418810846
ILMN_1802146	ZNF74	0.418632357
ILMN_2127379	ST6GALNAC3	0.41861049
ILMN_1898311	BI481473	0.418068205
ILMN_3233871	ATXN1L	0.418047722
ILMN_1779597	CIDEC	0.418037479
ILMN_1674050	COL8A2	0.417961672
ILMN_2394557	WNK3	0.417956076
ILMN_1681884	LOC100506049	0.41780551
ILMN_1746013	SPOCK1	0.417766834
ILMN_2079285	ATP5L	0.417760383
ILMN_1679127	CCAR1	0.417676205
ILMN_2282081	QRICH1	0.417674852
ILMN_3293173	DQ059386	0.417648322
ILMN_1700763	BC153822	0.4175651
ILMN_1670438	PRAMEF7	0.417185112
ILMN_1746375	CSNK2A1	0.417107366
ILMN_1660305	PDE12	0.416750244
ILMN_3251477	RG9MTD3	0.416747539
ILMN_1797965	PDP2	0.416519504
ILMN_1825443	BX089525	0.41644475
ILMN_1797808	PRH2	0.416415099
ILMN_1664071	TNNT2	0.416052978
ILMN_1663945	PTPN20A	0.41600311
ILMN_3237226	MBD3L2	0.415840736
ILMN_1686011	KIAA0979	0.415795373
ILMN_1785252	SLC26A6	0.41565584
ILMN_2071316	FGF23	0.415304698
ILMN_1769416	TTN	0.415118028
ILMN_1776577	DSCC1	0.415040072
ILMN_2054607	CYP4V2	0.414842143
ILMN_1812073	ATP6V1B1	0.414710177
ILMN_3239050	SLC22A20	0.414595313
ILMN_2062754	SRA1	0.4145672
ILMN_1809695	CAMK2G	0.414544205
ILMN_2401933	ATP2B1	0.414506281
ILMN_1805271	ABCA11P	0.414444017
ILMN_1653646	UBL7	0.414116894
ILMN_1652736	RPS6KA3	0.413975001
ILMN_1770641	KLHL3	0.413852521
ILMN_1669114	WNK4	0.41383385
ILMN_1762802	CNIH2	0.413714076
ILMN_1780222	SLC6A7	0.413528375
ILMN_2152028	AK027082	0.413357368
ILMN_1805024	ERBB2IP	0.413350256

	SMART	BROMO	3	2.80E-01	9.90E-01
**Annotation Cluster 45**	**Enrichment Score: 0.56**		**Count**	**P_Value**	**Benjamini**
	INTERPRO	Pleckstrin homology-type	14	5.40E-02	9.60E-01
	INTERPRO	Pleckstrin homology	9	4.40E-01	1.00E+00
	SMART	PH	9	4.50E-01	9.80E-01
	UP_SEQ_FEATURE	domain:PH	7	5.60E-01	1.00E+00
**Annotation Cluster 46**	**Enrichment Score: 0.56**		**Count**	**P_Value**	**Benjamini**
	GOTERM_BP_FAT	regulation of system process	12	1.70E-01	9.90E-01
	GOTERM_BP_FAT	regulation of neurological system process	7	2.00E-01	9.90E-01
	GOTERM_BP_FAT	regulation of synaptic plasticity	4	2.30E-01	9.90E-01
	GOTERM_BP_FAT	regulation of synaptic transmission	6	2.70E-01	9.90E-01
	GOTERM_BP_FAT	regulation of transmission of nerve impulse	6	3.30E-01	9.90E-01
	GOTERM_BP_FAT	behavior	12	6.60E-01	1.00E+00
**Annotation Cluster 47**	**Enrichment Score: 0.53**		**Count**	**P_Value**	**Benjamini**
	GOTERM_BP_FAT	biological adhesion	22	2.60E-01	9.90E-01
	GOTERM_BP_FAT	cell adhesion	22	2.70E-01	9.90E-01
	GOTERM_BP_FAT	cell-cell adhesion	10	2.80E-01	9.90E-01
	SP_PIR_KEYWORDS	cell adhesion	13	3.80E-01	9.30E-01
**Annotation Cluster 48**	**Enrichment Score: 0.52**		**Count**	**P_Value**	**Benjamini**
	UP_SEQ_FEATURE	domain:CUB 1	3	2.30E-01	1.00E+00
	UP_SEQ_FEATURE	domain:CUB 2	3	2.30E-01	1.00E+00
	INTERPRO	CUB	3	3.80E-01	1.00E+00
	SMART	CUB	3	3.90E-01	9.80E-01
**Annotation Cluster 49**	**Enrichment Score: 0.52**		**Count**	**P_Value**	**Benjamini**
	GOTERM_BP_FAT	cellular amino acid catabolic process	4	2.50E-01	9.90E-01
	GOTERM_BP_FAT	organic acid catabolic process	5	3.20E-01	9.90E-01
	GOTERM_BP_FAT	carboxylic acid catabolic process	5	3.20E-01	9.90E-01
	GOTERM_BP_FAT	amine catabolic process	4	3.20E-01	9.90E-01
**Annotation Cluster 50**	**Enrichment Score: 0.5**		**Count**	**P_Value**	**Benjamini**
	INTERPRO	SPla/RYanodine receptor SPRY	5	1.70E-01	9.90E-01
	INTERPRO	B302 (SPRY)-like	5	1.90E-01	9.90E-01
	UP_SEQ_FEATURE	domain:B30.2/SPRY	4	3.40E-01	1.00E+00
	INTERPRO	SPla/RYanodine receptor subgroup	3	4.20E-01	1.00E+00
	SMART	SPRY	3	4.30E-01	9.90E-01
	INTERPRO	Butyrophylin-like	3	4.80E-01	1.00E+00
**Annotation Cluster 51**	**Enrichment Score: 0.5**		**Count**	**P_Value**	**Benjamini**
	GOTERM_MF_FAT	electron carrier activity	11	8.00E-02	9.70E-01
	SP_PIR_KEYWORDS	Monooxygenase	5	1.30E-01	8.00E-01
	INTERPRO	Cytochrome P450, C-terminal region	4	1.80E-01	9.90E-01
	INTERPRO	Cytochrome P450	4	2.10E-01	9.90E-01
	INTERPRO	Cytochrome P450, conserved site	4	2.20E-01	9.90E-01
	COG_ONTOLOGY	Secondary metabolites biosynthesis, transport, and catabolism	4	2.40E-01	9.90E-01
	INTERPRO	Cytochrome P450, E-class, group I	3	3.70E-01	1.00E+00
	SP_PIR_KEYWORDS	metalloprotein	5	3.80E-01	9.30E-01
	SP_PIR_KEYWORDS	iron	9	4.40E-01	9.50E-01
	UP_SEQ_FEATURE	metal ion-binding site:Iron (heme axial ligand)	4	4.50E-01	1.00E+00
	GOTERM_MF_FAT	iron ion binding	10	4.60E-01	1.00E+00
	SP_PIR_KEYWORDS	chromoprotein	3	4.60E-01	9.60E-01
	SP_PIR_KEYWORDS	heme	4	5.90E-01	9.70E-01
	GOTERM_MF_FAT	heme binding	4	6.40E-01	1.00E+00
	GOTERM_MF_FAT	tetrapyrrole binding	4	6.90E-01	1.00E+00
**Annotation Cluster 52**	**Enrichment Score: 0.49**		**Count**	**P_Value**	**Benjamini**
	SP_PIR_KEYWORDS	plasma	6	8.50E-02	7.40E-01
	GOTERM_BP_FAT	protein maturation by peptide bond cleavage	4	3.80E-01	9.90E-01
	GOTERM_BP_FAT	protein processing	4	5.50E-01	1.00E+00
	GOTERM_BP_FAT	protein maturation	4	6.10E-01	1.00E+00
**Annotation Cluster 53**	**Enrichment Score: 0.49**		**Count**	**P_Value**	**Benjamini**
	GOTERM_BP_FAT	glucose metabolic process	7	2.00E-01	9.90E-01
	SP_PIR_KEYWORDS	carbohydrate metabolism	4	3.00E-01	9.00E-01
	GOTERM_BP_FAT	hexose metabolic process	7	3.70E-01	1.00E+00
	GOTERM_BP_FAT	monosaccharide metabolic process	7	5.00E-01	1.00E+00
**Annotation Cluster 54**	**Enrichment Score: 0.48**		**Count**	**P_Value**	**Benjamini**
	INTERPRO	C2 membrane targeting protein	6	1.40E-01	9.80E-01
	INTERPRO	C2 calcium-dependent membrane targeting	6	2.80E-01	1.00E+00
	SMART	C2	6	2.90E-01	9.90E-01
	UP_SEQ_FEATURE	domain:C2 1	3	4.20E-01	1.00E+00
	UP_SEQ_FEATURE	domain:C2 2	3	4.20E-01	1.00E+00
	UP_SEQ_FEATURE	domain:C2	3	6.10E-01	1.00E+00
**Annotation Cluster 55**	**Enrichment Score: 0.47**		**Count**	**P_Value**	**Benjamini**
	GOTERM_BP_FAT	fibroblast growth factor receptor signaling pathway	4	3.70E-02	9.00E-01
	INTERPRO	Interleukin 1/heparin-binding growth factor	3	1.30E-01	9.80E-01
	SMART	FGF	3	1.30E-01	9.60E-01
	GOTERM_BP_FAT	transmembrane receptor protein tyrosine kinase signaling pathway	9	2.20E-01	9.90E-01
	KEGG_PATHWAY	Melanoma	3	6.10E-01	9.90E-01
	SP_PIR_KEYWORDS	growth factor	4	6.40E-01	9.80E-01
	KEGG_PATHWAY	Pathways in cancer	9	7.40E-01	1.00E+00
	KEGG_PATHWAY	Regulation of actin cytoskeleton	6	7.50E-01	1.00E+00
	KEGG_PATHWAY	MAPK signaling pathway	7	7.90E-01	1.00E+00
	GOTERM_MF_FAT	growth factor activity	4	8.20E-01	1.00E+00
**Annotation Cluster 56**	**Enrichment Score: 0.46**		**Count**	**P_Value**	**Benjamini**
	UP_SEQ_FEATURE	domain:GPS	3	2.30E-01	1.00E+00
	INTERPRO	GPS	3	2.60E-01	1.00E+00
	SMART	GPS	3	2.60E-01	9.90E-01
	PIR_SUPERFAMILY	PIRSF800007:secretin receptor-like G protein-coupled receptors	3	3.40E-01	1.00E+00
	INTERPRO	GPCR, family 2, secretin-like	3	3.40E-01	1.00E+00
	INTERPRO	GPCR, family 2, secretin-like, conserved site	3	3.60E-01	1.00E+00
	INTERPRO	GPCR, family 2-like	3	4.60E-01	1.00E+00
	GOTERM_BP_FAT	neuropeptide signaling pathway	3	6.90E-01	1.00E+00
**Annotation Cluster 57**	**Enrichment Score: 0.46**		**Count**	**P_Value**	**Benjamini**
	GOTERM_BP_FAT	phosphate metabolic process	39	6.20E-03	9.00E-01
	GOTERM_BP_FAT	phosphorus metabolic process	39	6.20E-03	9.00E-01
	GOTERM_BP_FAT	phosphorylation	28	8.60E-02	9.40E-01
	UP_SEQ_FEATURE	active site:Proton acceptor	23	1.10E-01	1.00E+00
	SP_PIR_KEYWORDS	kinase	22	2.10E-01	8.80E-01
	GOTERM_BP_FAT	protein amino acid phosphorylation	21	2.60E-01	9.90E-01
	SP_PIR_KEYWORDS	atp-binding	38	2.80E-01	8.90E-01
	SP_PIR_KEYWORDS	serine/threonine-protein kinase	12	3.70E-01	9.20E-01
	UP_SEQ_FEATURE	nucleotide phosphate-binding region:ATP	27	3.80E-01	1.00E+00
	INTERPRO	Serine/threonine protein kinase, active site	11	4.40E-01	1.00E+00

ILMN_2155228	CRLF3	-0.485279697
ILMN_1685387	PIGR	-0.485709826
ILMN_3236270	ACAP2	-0.485944237
ILMN_1651767	MKL1	-0.486103257
ILMN_1695356	RNF166	-0.487614045
ILMN_2088172	POLR2B	-0.487664609
ILMN_1665049	SPG11	-0.488494716
ILMN_1795025	NFX1	-0.489215304
ILMN_2367113	CASP6	-0.489460115
ILMN_1811303	NR5A2	-0.489755554
ILMN_1689899	MTERFD2	-0.490132275
ILMN_2104356	COL1A2	-0.490777805
ILMN_1669635	NUP85	-0.490794452
ILMN_1697764	HYDIN	-0.490856845
ILMN_2363621	RBBP8	-0.491491897
ILMN_1742230	BAZ1A	-0.491520425
ILMN_2289849	FCGR2A	-0.491603956
ILMN_3310710	MIR518E	-0.492289256
ILMN_1797822	SEL1L3	-0.492290952
ILMN_3209399	KRT18	-0.49252061
ILMN_1713686	IL34	-0.492543677
ILMN_1701613	RARRES3	-0.493003148
ILMN_1695357	CCDC99	-0.493008062
ILMN_1685403	MMP7	-0.49323272
ILMN_3310456	MIR888	-0.493533174
ILMN_1677827	TLR7	-0.49353519
ILMN_3309929	MIR668	-0.493815158
ILMN_1737146	TRAM1	-0.494320399
ILMN_2361807	OS9	-0.495470541
ILMN_1808501	SH3KBP1	-0.49569148
ILMN_2414027	CMTM1	-0.495728776
ILMN_2183424	LOC374491	-0.495802109
ILMN_1654504	CLEC7A	-0.495903096
ILMN_1678052	C19orf24	-0.49598602
ILMN_2400759	CPVL	-0.497195211
ILMN_1861376	AL080095	-0.49723252
ILMN_1709795	RAC2	-0.497469242
ILMN_1751020	PACSIN1	-0.49829481
ILMN_1733538	RGS10	-0.499701636
ILMN_1711005	CDC25A	-0.500128368
ILMN_2338116	TRAF5	-0.501000695
ILMN_1652787	PIK3AP1	-0.501120007
ILMN_1739454	USP34	-0.501294611
ILMN_1686097	TOP2A	-0.502058525
ILMN_1762861	HLA-F	-0.502523124
ILMN_1805466	SOX9	-0.503084958
ILMN_2326273	CHI3L2	-0.504740548
ILMN_1801307	TNFSF10	-0.50500173
ILMN_1765557	OLFML2B	-0.505009325
ILMN_1737110	BF034544	-0.505176608
ILMN_2101885	TUBB	-0.505339724
ILMN_1676256	TPSAB1	-0.505555778
ILMN_1771599	PLOD2	-0.506310472
ILMN_2388539	C17orf101	-0.506377457
ILMN_1808590	GUCY1A3	-0.506751014
ILMN_1808874	AK127262	-0.506820041
ILMN_1652407	ZMYND8	-0.506903075
ILMN_1752247	AKAP13	-0.507519726
ILMN_1652366	NLRP7	-0.507797003
ILMN_3310191	MIR411	-0.508443368
ILMN_3289508	CR604709	-0.509239984
ILMN_1694041	PDCL	-0.510464544
ILMN_3187324	CNRIP1	-0.510632892
ILMN_3246713	SNORA22	-0.510654137
ILMN_2350514	TBX1	-0.510729956
ILMN_2120103	AGAP7	-0.511371321
ILMN_1687501	MOXD1	-0.511803253
ILMN_1687301	VCAN	-0.512409939
ILMN_2192072	MMP7	-0.512764845
ILMN_3309021	MIR142	-0.513030792
ILMN_1745558	CXADR	-0.513856425
ILMN_1801504	RUNX1	-0.514143702
ILMN_1690224	SPDYE2	-0.514572873
ILMN_1800739	SPINT2	-0.515556592
ILMN_1691662	LOC100101266	-0.515775764
ILMN_1777040	GOLGA9P	-0.515835428
ILMN_1662896	WDR11	-0.516846426
ILMN_1721129	NEK4	-0.518560964
ILMN_2123743	FCER1G	-0.52068187
ILMN_3308133	MIR1296	-0.520832393
ILMN_1666633	BC010030	-0.523413831
ILMN_1682312	CYBB	-0.523839317
ILMN_1774287	CFB	-0.524498536
ILMN_2241412	FGFR4	-0.524676954
ILMN_1789361	PKD2L1	-0.525002478
ILMN_3309649	MIR1226	-0.526174767
ILMN_1716080	CBL	-0.526305287
ILMN_2365881	ATG16L1	-0.526618681
ILMN_1661196	CSF2RA	-0.526968759
ILMN_1666932	FCGR2A	-0.527225104
ILMN_1792587	VPS4B	-0.528489135
ILMN_3248232	SNORA2A	-0.529334422
ILMN_1781236	LOC554223	-0.529357422
ILMN_1744455	NOTUM	-0.529672397
ILMN_1698952	TNFRSF11A	-0.530129601
ILMN_1796738	SPAST	-0.530789207
ILMN_2283388	C20orf24	-0.533142285
ILMN_2352448	NSUN5P1	-0.533627585
ILMN_1717706	PLK2	-0.535281417

ILMN_1778734	FAN1	0.413132918
ILMN_2347534	C20orf71	0.412977872
ILMN_1718783	EXTL1	0.412814725
ILMN_1789500	KIAA1875	0.412650369
ILMN_1701331	UBE2M	0.41251765
ILMN_1664502	CRMP1	0.412457833
ILMN_1713682	FBXO11	0.412368493
ILMN_3234570	CECR4	0.412280635
ILMN_1798779	LSP1	0.412224684
ILMN_1693411	ZDHHC11	0.412106946
ILMN_1916313	BX111153	0.41210336
ILMN_1662497	KRTAP3-2	0.412050205
ILMN_1747084	AKD1	0.41197527
ILMN_2388112	CR1	0.411858505
ILMN_2150049	C6orf138	0.411821988
ILMN_1780861	METRNL	0.411821247
ILMN_2389064	C15orf48	0.411758097
ILMN_3243320	SBDSP	0.411537201
ILMN_1747580	NF2	0.411436364
ILMN_2176693	LIPK	0.411260679
ILMN_3198338	LOC100128496	0.411188392
ILMN_1770690	CDKL3	0.411170417
ILMN_1701967	SHC1	0.411160427
ILMN_1666096	ACSL3	0.411157523
ILMN_1782579	IMMT	0.411080162
ILMN_1781398	NRXN1	0.410992617
ILMN_2360988	SEMA6B	0.410916518
ILMN_1788384	C9orf5	0.410897808
ILMN_1797281	RGPD6	0.410618914
ILMN_1808078	GPR128	0.410281369
ILMN_1700382	HOXA1	0.410112518
ILMN_3249073	FAM25C	0.409990702
ILMN_2364072	CLCNKA	0.40975989
ILMN_3275056	NA	0.40968643
ILMN_1808163	C11orf24	0.409645732
ILMN_2045351	SPINK9	0.409602736
ILMN_3241647	OPN1MW	0.409310311
ILMN_1704598	HYAL4	0.409138562
ILMN_1766261	SLC2A12	0.409039877
ILMN_1692623	LPHN3	0.408602093
ILMN_3178295	LOC100129407	0.408478504
ILMN_1674560	GBA2	0.408401176
ILMN_1749944	DLGAP4	0.408155044
ILMN_1773063	OSBPL1A	0.407940344
ILMN_3242384	DEFB108B	0.407932398
ILMN_2406304	PDZRN3	0.407901128
ILMN_1721081	SP4	0.407757076
ILMN_1741960	DGKG	0.407655793
ILMN_1792419	FRMD7	0.407639178
ILMN_1861652	BQ575680	0.407415665
ILMN_1859665	AW629225	0.407121402
ILMN_2111237	MN1	0.406997478
ILMN_1751051	C7orf25	0.40698491
ILMN_1696480	NAV1	0.406925582
ILMN_3289057	NA	0.406906852
ILMN_1782862	TDGF3	0.406897563
ILMN_1651693	GSG1	0.406713735
ILMN_1806473	BEX5	0.406704764
ILMN_1664014	STOX1	0.406646338
ILMN_1856498	AW087252	0.406552338
ILMN_3277608	BC007817	0.406330651
ILMN_1766565	KRTAP5-9	0.406085222
ILMN_1663605	RNF123	0.406050408
ILMN_1698673	EFCAB7	0.406040389
ILMN_2227473	PIBF1	0.406014699
ILMN_3185313	CCDC85C	0.405996053
ILMN_1770725	AIF1L	0.405689204
ILMN_1669851	STAG3L4	0.405641235
ILMN_1738849	SLC9A2	0.405554617
ILMN_1760698	WHAMML2	0.40551548
ILMN_1801891	HDX	0.405514475
ILMN_1682316	TRIM33	0.405445568
ILMN_1696946	NUS1	0.405328263
ILMN_1660658	MUC12	0.405271826
ILMN_1768624	CETN1	0.405212194
ILMN_2067682	LOC401431	0.405175671
ILMN_1685043	CYP3A7	0.405123734
ILMN_1660674	LOC619207	0.40496766
ILMN_1739155	OR9A4	0.404803388
ILMN_1691432	PRDM4	0.404794663
ILMN_1667609	SNORD35A	0.404724942
ILMN_2201386	ADAM18	0.404621139
ILMN_2152865	PHOSPHO2	0.404544558
ILMN_1775861	DDI2	0.404122262
ILMN_2159322	SLK	0.404112396
ILMN_3242481	AK025118	0.404071949
ILMN_2394552	WNK3	0.403902136
ILMN_2197577	SPRR2D	0.40382084
ILMN_1777797	AFM	0.403706098
ILMN_2343264	PRDM16	0.403629066
ILMN_1739486	BX647938	0.403297236
ILMN_2072568	CLDN8	0.403051187
ILMN_1755741	DACH1	0.402648662
ILMN_1738866	DEXI	0.402642724
ILMN_1757693	C17orf51	0.402498092
ILMN_1712943	CCK	0.402157125
ILMN_1757651	BC041879	0.401718018
ILMN_1656554	LOC727924	0.401649076
ILMN_1740265	ACOT7	0.401646887
ILMN_1671839	TAF1C	0.401643202
ILMN_1796243	SLC13A3	0.401563359
ILMN_2394975	OTUD4	0.401483567

	INTERPRO	Serine/threonine protein kinase-related	11	4.60E-01	1.00E+00
	SP_PIR_KEYWORDS	nucleotide-binding	44	4.80E-01	9.60E-01
	GOTERM_MF_FAT	protein serine/threonine kinase activity	13	5.00E-01	1.00E+00
	UP_SEQ_FEATURE	binding site:ATP	15	5.10E-01	1.00E+00
	INTERPRO	Serine/threonine protein kinase	8	5.20E-01	1.00E+00
	INTERPRO	Protein kinase, ATP binding site	13	5.20E-01	1.00E+00
	SMART	S_TKc	8	5.30E-01	9.90E-01
	INTERPRO	Protein kinase, core	13	5.80E-01	1.00E+00
	GOTERM_MF_FAT	ATP binding	40	6.00E-01	1.00E+00
	GOTERM_MF_FAT	adenyl ribonucleotide binding	40	6.40E-01	1.00E+00
	UP_SEQ_FEATURE	domain:Protein kinase	12	6.40E-01	1.00E+00
	SP_PIR_KEYWORDS	transferase	34	6.70E-01	9.80E-01
	GOTERM_MF_FAT	protein kinase activity	16	6.80E-01	1.00E+00
	GOTERM_MF_FAT	purine nucleoside binding	42	6.90E-01	1.00E+00
	GOTERM_MF_FAT	nucleotide binding	59	6.90E-01	1.00E+00
	GOTERM_MF_FAT	purine ribonucleotide binding	48	7.00E-01	1.00E+00
	GOTERM_MF_FAT	ribonucleotide binding	48	7.00E-01	1.00E+00
	GOTERM_MF_FAT	nucleoside binding	42	7.00E-01	1.00E+00
	GOTERM_MF_FAT	adenyl nucleotide binding	41	7.10E-01	1.00E+00
	GOTERM_MF_FAT	purine nucleotide binding	49	7.60E-01	1.00E+00
**Annotation Cluster 58**	**Enrichment Score: 0.45**		**Count**	**P_Value**	**Benjamini**
	PIR_SUPERFAMILY	PIRSF002458:ADP,ATP carrier protein	3	2.10E-01	1.00E+00
	UP_SEQ_FEATURE	repeat:Solcar 3	3	3.70E-01	1.00E+00
	INTERPRO	Mitochondrial substrate carrier	3	3.90E-01	1.00E+00
	UP_SEQ_FEATURE	repeat:Solcar 2	3	4.00E-01	1.00E+00
	UP_SEQ_FEATURE	repeat:Solcar 1	3	4.00E-01	1.00E+00
	INTERPRO	Mitochondrial substrate/solute carrier	3	4.30E-01	1.00E+00
**Annotation Cluster 59**	**Enrichment Score: 0.44**		**Count**	**P_Value**	**Benjamini**
	UP_SEQ_FEATURE	domain:SET	3	3.50E-01	1.00E+00
	INTERPRO	SET	3	3.70E-01	1.00E+00
	SMART	SET	3	3.70E-01	9.80E-01
**Annotation Cluster 60**	**Enrichment Score: 0.44**		**Count**	**P_Value**	**Benjamini**
	GOTERM_MF_FAT	histone methyltransferase activity	3	2.90E-01	9.80E-01
	GOTERM_MF_FAT	protein methyltransferase activity	3	4.00E-01	1.00E+00
	GOTERM_MF_FAT	N-methyltransferase activity	3	4.20E-01	1.00E+00
**Annotation Cluster 61**	**Enrichment Score: 0.43**		**Count**	**P_Value**	**Benjamini**
	GOTERM_BP_FAT	icosanoid metabolic process	3	3.40E-01	9.90E-01
	GOTERM_BP_FAT	unsaturated fatty acid metabolic process	3	3.80E-01	1.00E+00
	GOTERM_BP_FAT	fatty acid metabolic process	7	4.00E-01	1.00E+00
**Annotation Cluster 62**	**Enrichment Score: 0.42**		**Count**	**P_Value**	**Benjamini**
	SP_PIR_KEYWORDS	antibiotic	4	2.40E-01	8.70E-01
	SP_PIR_KEYWORDS	Antimicrobial	4	2.60E-01	8.80E-01
	GOTERM_BP_FAT	defense response to bacterium	4	5.50E-01	1.00E+00
	GOTERM_BP_FAT	response to bacterium	6	5.60E-01	1.00E+00
**Annotation Cluster 63**	**Enrichment Score: 0.42**		**Count**	**P_Value**	**Benjamini**
	GOTERM_BP_FAT	response to nutrient levels	8	2.40E-01	9.90E-01
	GOTERM_BP_FAT	response to starvation	3	2.90E-01	9.90E-01
	GOTERM_BP_FAT	response to extracellular stimulus	8	3.40E-01	9.90E-01
	GOTERM_BP_FAT	response to nutrient	3	8.80E-01	1.00E+00
**Annotation Cluster 64**	**Enrichment Score: 0.42**		**Count**	**P_Value**	**Benjamini**
	GOTERM_BP_FAT	mitotic sister chromatid segregation	4	6.40E-02	9.30E-01
	GOTERM_BP_FAT	sister chromatid segregation	4	6.90E-02	9.30E-01
	GOTERM_BP_FAT	organelle fission	9	2.40E-01	9.90E-01
	GOTERM_BP_FAT	nuclear division	8	3.40E-01	9.90E-01
	GOTERM_BP_FAT	mitosis	8	3.40E-01	9.90E-01
	GOTERM_BP_FAT	chromosome segregation	4	3.40E-01	9.90E-01
	GOTERM_BP_FAT	mitotic cell cycle	12	3.50E-01	9.90E-01
	GOTERM_BP_FAT	M phase of mitotic cell cycle	8	3.60E-01	9.90E-01
	SP_PIR_KEYWORDS	cell cycle	13	5.10E-01	9.60E-01
	GOTERM_BP_FAT	cell cycle	21	5.20E-01	1.00E+00
	SP_PIR_KEYWORDS	mitosis	5	6.80E-01	9.80E-01
	GOTERM_BP_FAT	cell cycle process	14	7.00E-01	1.00E+00
	GOTERM_BP_FAT	cell cycle phase	10	7.40E-01	1.00E+00
	GOTERM_BP_FAT	M phase	8	7.40E-01	1.00E+00
	GOTERM_BP_FAT	cell division	7	7.70E-01	1.00E+00
	SP_PIR_KEYWORDS	cell division	6	8.00E-01	9.90E-01
**Annotation Cluster 65**	**Enrichment Score: 0.41**		**Count**	**P_Value**	**Benjamini**
	SP_PIR_KEYWORDS	Lectin	7	2.10E-01	8.80E-01
	UP_SEQ_FEATURE	domain:C-type lectin	4	3.20E-01	1.00E+00
	INTERPRO	C-type lectin	4	3.60E-01	1.00E+00
	INTERPRO	C-type lectin, conserved site	4	3.60E-01	1.00E+00
	SMART	CLECT	4	3.70E-01	9.80E-01
	GOTERM_MF_FAT	sugar binding	7	4.40E-01	1.00E+00
	INTERPRO	C-type lectin-like	4	4.50E-01	1.00E+00
	UP_SEQ_FEATURE	domain:Ig-like V-type	3	8.30E-01	1.00E+00
**Annotation Cluster 66**	**Enrichment Score: 0.41**		**Count**	**P_Value**	**Benjamini**
	GOTERM_BP_FAT	cell-cell adhesion	10	2.80E-01	9.90E-01
	UP_SEQ_FEATURE	domain:Cadherin 6	4	2.80E-01	1.00E+00
	INTERPRO	Cadherin	5	3.10E-01	1.00E+00
	SMART	CA	5	3.20E-01	9.80E-01
	UP_SEQ_FEATURE	domain:Cadherin 5	4	4.30E-01	1.00E+00
	GOTERM_BP_FAT	homophilic cell adhesion	5	4.30E-01	1.00E+00
	UP_SEQ_FEATURE	domain:Cadherin 3	4	4.80E-01	1.00E+00
	UP_SEQ_FEATURE	domain:Cadherin 4	4	4.80E-01	1.00E+00
	UP_SEQ_FEATURE	domain:Cadherin 1	4	5.00E-01	1.00E+00
	UP_SEQ_FEATURE	domain:Cadherin 2	4	5.00E-01	1.00E+00
**Annotation Cluster 67**	**Enrichment Score: 0.4**		**Count**	**P_Value**	**Benjamini**
	GOTERM_BP_FAT	ectoderm development	8	2.50E-01	9.90E-01
	GOTERM_BP_FAT	epithelial cell differentiation	6	2.80E-01	9.90E-01
	GOTERM_BP_FAT	keratinocyte differentiation	3	5.10E-01	1.00E+00
	GOTERM_BP_FAT	epidermis development	6	5.10E-01	1.00E+00
	GOTERM_BP_FAT	epidermal cell differentiation	3	5.50E-01	1.00E+00
**Annotation Cluster 68**	**Enrichment Score: 0.39**		**Count**	**P_Value**	**Benjamini**
	UP_SEQ_FEATURE	domain:Ig-like C2-type 4	6	4.40E-02	1.00E+00
	UP_SEQ_FEATURE	domain:Ig-like C2-type 5	4	1.60E-01	1.00E+00
	UP_SEQ_FEATURE	domain:Ig-like C2-type 3	6	2.00E-01	1.00E+00
	UP_SEQ_FEATURE	domain:Ig-like C2-type 6	3	2.40E-01	1.00E+00
	UP_SEQ_FEATURE	domain:Ig-like C2-type 1	7	3.50E-01	1.00E+00
	UP_SEQ_FEATURE	domain:Ig-like C2-type 2	7	3.50E-01	1.00E+00
	INTERPRO	Immunoglobulin	7	4.40E-01	1.00E+00
	INTERPRO	Immunoglobulin subtype 2	7	4.50E-01	1.00E+00
	SMART	IGc2	7	4.50E-01	9.80E-01

ILMN_1784294	CPA4	-0.536360956
ILMN_1798172	IPO4	-0.538146121
ILMN_3243801	LOC100131199	-0.538893785
ILMN_1803977	GALNT1	-0.539899657
ILMN_2384591	HN1	-0.541073408
ILMN_1669592	MARCH2	-0.541431434
ILMN_1742547	NRP1	-0.541618661
ILMN_1791838	LYG1	-0.541795257
ILMN_2335704	NCF4	-0.542832534
ILMN_1712389	CMTM1	-0.545150482
ILMN_1796409	C1QB	-0.545893534
ILMN_1707467	CLCN3	-0.546687737
ILMN_3251634	SENP5	-0.547992471
ILMN_1662026	BTK	-0.549067566
ILMN_1658830	C10orf26	-0.549730681
ILMN_3242738	ZFP62	-0.549828251
ILMN_1852151	BX110351	-0.552652418
ILMN_2208413	ARHGAP15	-0.556485771
ILMN_1806415	TTLL1	-0.556664641
ILMN_1759084	INTS8	-0.557510582
ILMN_1680927	IGM1	-0.55976012
ILMN_3285055	CDC27	-0.559963265
ILMN_1715133	KIAA0850	-0.560574716
ILMN_3243839	TPSAB1	-0.565515627
ILMN_1693452	GAL3ST4	-0.565870028
ILMN_1666594	IRF8	-0.568246395
ILMN_1657129	SKAP2	-0.572059504
ILMN_2385662	LRIF1	-0.573330524
ILMN_3253901	HAVCR1	-0.574535298
ILMN_1679917	ARL16	-0.575414418
ILMN_1713058	PSTPIP2	-0.575672486
ILMN_3309284	MIR137	-0.576632678
ILMN_3180113	C12orf33	-0.579379967
ILMN_1756308	NAE1	-0.58285913
ILMN_1748438	POLR2G	-0.584076631
ILMN_2212714	TMEM39B	-0.594218147
ILMN_1721868	KPNA2	-0.594762898
ILMN_1769789	C6orf142	-0.597108352
ILMN_3244216	SCARNA3	-0.598117001
ILMN_3240321	AEN	-0.59922375
ILMN_1729832	EIF4H	-0.602356108
ILMN_1652237	CBR3	-0.611449024
ILMN_2110857	GALR3	-0.617655486
ILMN_1754272	GINS3	-0.617691768
ILMN_3248324	MGC16384	-0.617709434
ILMN_1762071	C17orf80	-0.618710119
ILMN_1747622	CD33	-0.623280157
ILMN_3209883	LOC653075	-0.625002725
ILMN_2301083	UBE2C	-0.628416512
ILMN_1659463	APAF1	-0.633243567
ILMN_1775357	RUNDC2A	-0.634672824
ILMN_1656073	MYL12A	-0.649843086
ILMN_2115434	RAB32	-0.658989634
ILMN_2077550	RACGAP1	-0.665170782
ILMN_1901304	AL137309	-0.673578385
ILMN_1780533	RNASE6	-0.696658419
ILMN_1660817	DDB2	-0.731471486

ILMN_1808290	LOC286467	0.401277488
ILMN_2387712	AK5	0.40116742
ILMN_2055396	TTTY17	0.401148479
ILMN_1752952	MMP28	0.401009814
ILMN_1856031	BC040892	0.400943199
ILMN_3282387	LOC100131373	0.400909017
ILMN_2225151	DLX6	0.400906363
ILMN_1739357	OPRM1	0.400869347
ILMN_1843648	AW873324	0.400725729
ILMN_3251278	DPPA3	0.40066857
ILMN_1659209	LOC728012	0.400541369
ILMN_1718932	MTRR	0.400435898
ILMN_1694359	UPF0639	0.400405737
ILMN_3202373	MAP1LC3B2	0.400270563
ILMN_3310216	MIR1911	0.400214107
ILMN_1849399	LOC100630918	0.400171982
ILMN_2087060	TOMM7	0.400157156
ILMN_3253310	BC150587	0.4000732

	INTERPRO	Immunoglobulin subtype	10	4.90E-01	1.00E+00
	SMART	IG	10	5.00E-01	9.90E-01
	KEGG_PATHWAY	Cell adhesion molecules (CAMs)	5	5.30E-01	9.90E-01
	INTERPRO	Immunoglobulin I-set	4	7.00E-01	1.00E+00
	SP_PIR_KEYWORDS	Immunoglobulin domain	10	8.40E-01	1.00E+00
	INTERPRO	Immunoglobulin-like	10	9.10E-01	1.00E+00
	INTERPRO	Immunoglobulin-like fold	10	9.50E-01	1.00E+00
	INTERPRO	Immunoglobulin V-set	4	9.60E-01	1.00E+00
**Annotation Cluster 69**	**Enrichment Score: 0.39**		**Count**	**P_Value**	**Benjamini**
	GOTERM_BP_FAT	camera-type eye development	5	2.90E-01	9.90E-01
	GOTERM_BP_FAT	eye development	5	4.40E-01	1.00E+00
	GOTERM_BP_FAT	eye morphogenesis	3	5.30E-01	1.00E+00
**Annotation Cluster 70**	**Enrichment Score: 0.39**		**Count**	**P_Value**	**Benjamini**
	GOTERM_MF_FAT	ion binding	124	2.00E-01	9.80E-01
	GOTERM_MF_FAT	metal ion binding	121	2.10E-01	9.80E-01
	SP_PIR_KEYWORDS	metal-binding	82	2.30E-01	8.70E-01
	GOTERM_MF_FAT	cation binding	121	2.50E-01	9.80E-01
	SP_PIR_KEYWORDS	zinc-finger	42	6.60E-01	9.80E-01
	SP_PIR_KEYWORDS	zinc	52	7.40E-01	9.90E-01
	GOTERM_MF_FAT	transition metal ion binding	72	7.50E-01	1.00E+00
	GOTERM_MF_FAT	zinc ion binding	55	9.10E-01	1.00E+00
**Annotation Cluster 71**	**Enrichment Score: 0.38**		**Count**	**P_Value**	**Benjamini**
	GOTERM_BP_FAT	carbohydrate catabolic process	5	3.10E-01	9.90E-01
	GOTERM_BP_FAT	cellular carbohydrate catabolic process	4	3.70E-01	9.90E-01
	GOTERM_BP_FAT	alcohol catabolic process	3	6.20E-01	1.00E+00
**Annotation Cluster 72**	**Enrichment Score: 0.38**		**Count**	**P_Value**	**Benjamini**
	GOTERM_BP_FAT	visual perception	8	3.20E-01	9.90E-01
	GOTERM_BP_FAT	sensory perception of light stimulus	8	3.20E-01	9.90E-01
	SP_PIR_KEYWORDS	vision	4	6.80E-01	9.80E-01
**Annotation Cluster 73**	**Enrichment Score: 0.38**		**Count**	**P_Value**	**Benjamini**
	UP_SEQ_FEATURE	zinc finger region:RING-type	8	2.80E-01	1.00E+00
	INTERPRO	Zinc finger, B-box	4	2.90E-01	1.00E+00
	SMART	BBOX	4	2.90E-01	9.80E-01
	INTERPRO	Zinc finger, RING-type	9	5.10E-01	1.00E+00
	SMART	RING	9	5.20E-01	9.90E-01
	INTERPRO	Zinc finger, C3HC4 RING-type	7	5.50E-01	1.00E+00
	INTERPRO	Zinc finger, RING-type, conserved site	8	5.90E-01	1.00E+00
**Annotation Cluster 74**	**Enrichment Score: 0.38**		**Count**	**P_Value**	**Benjamini**
	GOTERM_BP_FAT	synaptic transmission	10	3.60E-01	9.90E-01
	GOTERM_BP_FAT	cell-cell signaling	18	3.70E-01	9.90E-01
	GOTERM_BP_FAT	transmission of nerve impulse	10	5.40E-01	1.00E+00
**Annotation Cluster 75**	**Enrichment Score: 0.38**		**Count**	**P_Value**	**Benjamini**
	UP_SEQ_FEATURE	repeat:1	10	1.60E-01	1.00E+00
	UP_SEQ_FEATURE	repeat:2	10	1.70E-01	1.00E+00
	UP_SEQ_FEATURE	repeat:3	9	1.80E-01	1.00E+00
	UP_SEQ_FEATURE	repeat:4	6	4.90E-01	1.00E+00
	UP_SEQ_FEATURE	repeat:6	4	7.10E-01	1.00E+00
	UP_SEQ_FEATURE	repeat:5	4	7.60E-01	1.00E+00
	UP_SEQ_FEATURE	repeat:8	3	8.10E-01	1.00E+00
	UP_SEQ_FEATURE	repeat:7	3	8.40E-01	1.00E+00
**Annotation Cluster 76**	**Enrichment Score: 0.38**		**Count**	**P_Value**	**Benjamini**
	UP_SEQ_FEATURE	repeat:ANK 3	7	3.70E-01	1.00E+00
	UP_SEQ_FEATURE	repeat:ANK 1	8	3.70E-01	1.00E+00
	UP_SEQ_FEATURE	repeat:ANK 2	8	3.80E-01	1.00E+00
	SP_PIR_KEYWORDS	ank repeat	8	4.10E-01	9.40E-01
	UP_SEQ_FEATURE	repeat:ANK 6	4	4.10E-01	1.00E+00
	UP_SEQ_FEATURE	repeat:ANK 5	5	4.20E-01	1.00E+00
	INTERPRO	Ankyrin	8	4.30E-01	1.00E+00
	SMART	ANK	8	4.40E-01	9.80E-01
	UP_SEQ_FEATURE	repeat:ANK 4	5	5.70E-01	1.00E+00
**Annotation Cluster 77**	**Enrichment Score: 0.37**		**Count**	**P_Value**	**Benjamini**
	SP_PIR_KEYWORDS	sushi	3	4.10E-01	9.40E-01
	INTERPRO	Sushi/SCR/CCP	3	4.30E-01	1.00E+00
	SMART	CCP	3	4.40E-01	9.80E-01
	INTERPRO	Complement control module	3	4.40E-01	1.00E+00
**Annotation Cluster 78**	**Enrichment Score: 0.36**		**Count**	**P_Value**	**Benjamini**
	GOTERM_BP_FAT	aerobic respiration	3	2.30E-01	9.90E-01
	GOTERM_BP_FAT	cellular respiration	5	2.40E-01	9.90E-01
	GOTERM_BP_FAT	energy derivation by oxidation of organic compounds	6	3.10E-01	9.90E-01
	GOTERM_BP_FAT	respiratory electron transport chain	3	4.90E-01	1.00E+00
	GOTERM_CC_FAT	mitochondrial respiratory chain	3	5.30E-01	9.80E-01
	KEGG_PATHWAY	Huntington's disease	6	6.00E-01	9.90E-01
	GOTERM_CC_FAT	respiratory chain	3	6.10E-01	9.80E-01
	GOTERM_BP_FAT	electron transport chain	3	7.90E-01	1.00E+00
